# Accurate Simulation and Detection of Coevolution Signals in Multiple Sequence Alignments

**DOI:** 10.1371/journal.pone.0047108

**Published:** 2012-10-16

**Authors:** Sharon H. Ackerman, Elisabeth R. Tillier, Domenico L. Gatti

**Affiliations:** 1 Department of Biochemistry and Molecular Biology, Wayne State University School of Medicine, Detroit, Michigan, United States of America; 2 Cardiovascular Research Institute, Wayne State University School of Medicine, Detroit, Michigan, United States of America; 3 Department of Medical Biophysics, University of Toronto, Campbell Family Institute for Cancer Research, Ontario Cancer Institute, University Health Network, Toronto, Ontario, Canada; University of Rome, Italy

## Abstract

**Background:**

While the conserved positions of a multiple sequence alignment (MSA) are clearly of interest, non-conserved positions can also be important because, for example, destabilizing effects at one position can be compensated by stabilizing effects at another position. Different methods have been developed to recognize the evolutionary relationship between amino acid sites, and to disentangle functional/structural dependencies from historical/phylogenetic ones.

**Methodology/Principal Findings:**

We have used two complementary approaches to test the efficacy of these methods. In the first approach, we have used a new program, MSAvolve, for the *in silico* evolution of MSAs, which records a detailed history of all covarying positions, and builds a global coevolution matrix as the accumulated sum of individual matrices for the positions forced to co-vary, the recombinant coevolution, and the stochastic coevolution. We have simulated over 1600 MSAs for 8 protein families, which reflect sequences of different sizes and proteins with widely different functions. The calculated coevolution matrices were compared with the coevolution matrices obtained for the same evolved MSAs with different coevolution detection methods. In a second approach we have evaluated the capacity of the different methods to predict close contacts in the representative X-ray structures of an additional 150 protein families using only experimental MSAs.

**Conclusions/Significance:**

Methods based on the identification of global correlations between pairs were found to be generally superior to methods based only on local correlations in their capacity to identify coevolving residues using either simulated or experimental MSAs. However, the significant variability in the performance of different methods with different proteins suggests that the simulation of MSAs that replicate the statistical properties of the experimental MSA can be a valuable tool to identify the coevolution detection method that is most effective in each case.

## Introduction

During the past decade many efforts have been devoted to uncover the evolutionary dynamic of organisms through the examination of multiple sequence alignments (MSAs). The MSA of the members of a protein family provides a 2-dimensional view of a protein history, in which the 3^rd^ and 4^th^ dimensions, structure and time, are flattened. In a MSA some positions are highly conserved, while others vary. The conserved positions are clearly important, but the non-conserved positions are also important because the net stabilization of the folded state of proteins relative to the unfolded state is usually so small, that all positions may at some point contribute significantly to protein stability. For example, the destabilizing effects of a given amino acid at one position can be compensated by the stabilizing effect of a certain amino acid at another position. The existence of physical and functional interactions between sites in protein sequences leads to non-independence of their evolution: in other words, two (or more) positions in a protein sequence could be coevolving, and for any mutation to become fixed at such sites, compensatory mutations are needed at the related sites.

However, a high background of different interacting factors often hides the coevolutionary relationships between amino acid sites. A simple model to explain the correlation *C*_ij_ between two sites *i* and *j* in a sequence alignment was proposed by Atchley et al. [1], [2]:
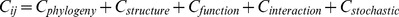



In this model *C_phylogeny_* is the correlation originating from phylogenetic relationships between homologous sequences that belong to the same branch of an evolutionary tree. For example, a mutation in an ancestral protein, which is clearly a single evolutionary event, appears in the MSA as an independent event that occurred in each of the proteins that descended from that ancestor. *C_structure_* and *C_function_* represent the correlation originating from structural and functional constraints. Ultimately, the preservation of structure is also related to the preservation of function because small changes in atom distances in the active site (produced by overall changes of structure) can have effects on the binding and activation energies as dramatic as those produced by very localized mutations in the active sites [3]. As a consequence, these sources of correlation, which are the most important signal that coevolution analyses try to extract from the MSA, are typically not independent from one another. Furthermore, as mutations are fixed elsewhere in the sequence throughout the evolutionary process, the functional and structural constraints on the correlation between sites may even change. *C_interaction_* describes both the interaction between the aforementioned sources of correlation, and the correlation originating from atomic interactions in homo-oligomeric proteins. Finally, *C_stochastic_* represents the correlation originating from casual co-variation and/or from uneven or incomplete sequence sampling. Low-quality and poorly populated MSAs are likely to produce a high degree of false coevolution signals as a result of the significant effect of stochasticity [4].

A wide variety of algorithms have been developed to detect coevolving positions from a MSA (reviewed in [5]–[7]). Some of these methods use χ^2^-tests [8], [9], some are perturbative [10]–[12], others employ amino acid substitution matrices [13], and many work within the frame of information theory [14]–[24].

In practice, most approaches to correlated mutation analysis do not discriminate between structural and functional correlations, and the sensitivity of most methods to detect these two forms of coevolution (the important signal) is ultimately dependent on the quality of the MSA, and is compromised in various degrees by the ability of these methods to filter out the stochastic and phylogenetic noise. In all cases, it appears that the inclusion of three-dimensional information may significantly enhance the methods' power to detect a clean coevolution signal [25], [26].

Another problem shared by most methods that use co-evolution analysis to predict interactions among residues is that many structurally distant pairs appear to be strongly correlated. One source of this correlation is the phylogenetic relationship between sequences in a multiple alignment. Another source is the propagation of statistical dependencies along chains of co-evolving contacts [11], [27], which tends to confound direct with indirect interactions. Disentanglement of these two types of interactions was attempted with the MIp [18], Zres [20] and Zpx [26] corrections of MI statistics, with the application of Bayesian network modeling in the logR method [22], with Direct Coupling Analysis (DCA) [24], [28], a maximum entropy method, and most recently with the use of sparse inverse covariance estimation in the PSICOV method [23]. PSICOV and DCA capacity to separate direct from indirect interactions was exploited to predict correctly the three-dimensional structures of both soluble and membrane proteins [29]–[31].

In the absence of an analytical model for covariation, many studies have relied on the simulations of MSAs as a tool to test a method capacity to filter out the background coevolution signal [4], [9], [15]–[17], [32]. Simulated MSAs can reproduce some of the evolutionary parameters of the real MSA (e.g., the level of conservation and the amino acid distribution at each site, the distribution of pair-wise similarities between individual sequences) and thus, given a certain amino acid substitution model, can easily yield the true coevolution signal. Earlier simulations of MSAs developed for the purpose of studying coevolution have not included recombination processes (e.g., horizontal transfers), which by their own nature can produce large coevolving blocks inside a protein. In this study we have developed a new program, MSAvolve (implemented as a Matlab Toolbox and distributed under BSD licensing), for the *in silico* evolution of MSAs, which allows for both substitutions at individual sites, and recombination in and between branches of an evolutionary tree. The program records a detailed history of all covarying positions, and automatically builds a global coevolution matrix as the accumulated sum of individual matrices for the structure/function coevolution (the positions that we force to co-vary), the recombinant coevolution, and the stochastic (random) coevolution. The degree of phylogenetic coevolution can be derived independently from the previous three matrices, which however, by themselves describe completely the coevolution history of the sequences in the MSA.

In this study we simulated the MSAs for 8 protein families, which reflect sequences of different sizes and proteins with widely different functions. The calculated coevolution matrices (the true coevolution in the evolved MSAs) were compared with the coevolution matrices obtained for the same evolved MSAs with a variety of methods to detect covariation, including three new ones (used here for the first time) based on a binary representation of the alignment. In a second approach we have evaluated the capacity of the different methods to predict close contacts in the representative X-ray structures of the 8 aforementioned families, represented by MSAs with less than 500 sequences, and an additional 150 protein families, represented by MSAs with over 1000 sequences.

## Results

### Development of the differential binary methods

The differential binary methods were developed empirically using the feature of MSAvolve that allows a direct comparison between the true coevolution signal buried in a simulated MSA and the coevolution signal identified *a posteriori* by a coevolution detection method. Rationale for these methods initially stems from the consideration that the Mutual Information (MI) between two positions (*i* and *j*) in a MSA contains two kinds of information: (a) the first kind is just the information that something changes: for example when the amino acid (aa) at position *i* changes from sequence 1 (s1) to sequence 2 (s2), also the aa at position *j* changes from s1 to s2. (b) the second kind is the information about what aa changes at *i* from s1 to s2, compared to what aa changes at *j* from s1 to s2. Many non-MI methods use external information, such as substitution models and the properties of amino acids, that puts extra weight on the second kind of information. In contrast, we sought to find methods that put more emphasis on the simple occurrence of substitutions, disregarding the type of substitution involved: in principle, it should be possible to factorize the MI matrix in such a way that only information of type (a) is included.

**Figure 1:**
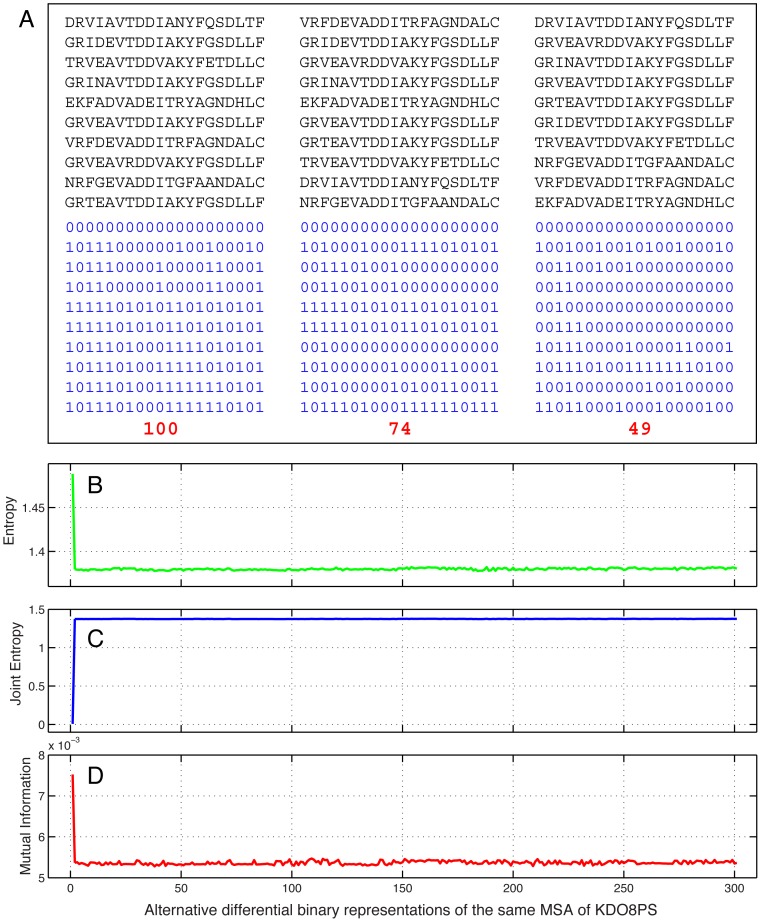
The differential binary (db) method. **A.** Resorting of the MSA and differential binary translations. The upper half of the panel shows the same 10 sequences in three different orders. The lower half of the panel shows the corresponding differential binary translations, with the values of the sums highlighted in red. The order of sequences on the left produces the highest sum ( = 100) after differential binary translation. The order in the middle produces an intermediate value ( = 74) of the sum, while the order on the right produces the smallest possible sum ( = 49) of any resorting of the sequences. **B.** Entropy changes in the binary translations of a simulated MSA of KDO8P synthase. The first point in the plot is the mean entropy <H(*i+j*)> = <H(*i*)+H(*j*)> of the unsorted binary MSA. The remaining points are the values of the same quantity in each of the 300 possible binary MSAs obtained by first resorting the original MSA, using in each case a different reference sequence (which is assigned a value of 0 in all the positions). **C.** Same as B but reporting on the mean joint entropy <H(*i,j*)> of all possible pairs. **D.** Mean mutual information <MI(*i*;*j*)> = <H(*i*)+H(*j*)−H(*i,j*)> for all possible pairs.

A numeric translation of the MSA that retains only the information on the occurrences of changes between sequences, but loses the information of what type of changes occur between them, was obtained by assigning a value of zero to every position of the first row of the MSA, and then in each consecutive row a 0 at the positions in which the symbol was the same as in the previous row and a 1 when the symbol changed from the previous row (**Figure 1A**). In this binary translation of the MSA, every 0 in a row represents a ‘no change’ with respect to the row immediately above, and every 1 represents a ‘change’: for this reason, we refer to this particular numeric formulation of the MSA as a ‘differential binary translation’.

As might be predicted, changing the order of the sequences in the original alignment results in different binary alignments (**Figure 1A**). Initial testing with simulated MSAs generated with MSAvolve indicated that a MI matrix calculated from the differential binary translation of the MSA had the best performance in the assignment of coevolving residues when the ordering of the sequences was calculated from the binary MSA with the smallest sum of 1's, that is when the number of changes from sequence to sequence was minimized. It is important to notice that the minimum obtained in the sum of the binary matrix depends on the choice of the 1st sequence in the MSA. The algorithm essentially orders the sequences phylogenetically, such that similar sequences are grouped together. An example of this optimal binary translation is shown in **Figure 1A**. In the upper half of the panel the same 10 sequences are shown in three different orders. The lowest half of the panel shows the corresponding differential binary translations. The order on the left produces the largest binary sum ( = 100). The order in the middle produces an intermediate value ( = 74) of the sum, while the order on the right produces the smallest possible value of the sum ( = 49) for any resorting of the sequences.

As shown in the following sections of the manuscript, the binary methods are among the most effective methods at detecting coevolving positions from just sequence data. To understand the origin of this performance, we have explored the effect of different ordering of the MSA by measuring the entropy, joint entropy, and MI of all possible pairs of positions in the corresponding binary alignments. For example, these are shown in panels B through D of **Figure 1** for a simulated MSA of KDO8PS containing 300 sequences of 280 aa's each. The first point in panel B represents the mean value of the sum of the entropies, H = H(*i*)+H(*j*), for all possible columns *i* and *j*, in the unsorted MSA. The remaining points in the plot show the mean value of the same sum for the 300 possible binary MSAs obtained by resorting the original MSA using in each case a different sequence as the top sequence (which is assigned a value of 0 everywhere), with the rest of the ordering still minimizing 1's. Panel C is similar to panel B except that in this case the mean values of the joint entropy H(*i,j*) are shown, which increase after resorting. Since the mutual information between columns *i,j* of the MSA is defined as MI(*i*;*j*) = H(*i*)+H(*j*)−H(*i,j*), resorting of the MSA leads to a reduction in the mean value of MI (**Figure 1D**). We found that after the MSA is translated into differential binary format, the initial resorting of sequences aimed at minimizing the number of changes (1's) always leads to a decrease in the mean MI and entropy and to an increase in the mean joint entropy of the columns of the binary MSA.

After the differential binary representation of the original MSA is obtained, a MI matrix is calculated from the binary alignment, and is further processed to obtain a ZPX2 matrix, defined here as the square of the ZPX matrix described by Gloor *et al.* [21]. The final ZPX2 matrix is thus named ‘differential binary ZPX2’ or ‘dbZPX2’. Our testing with simulated MSAs has shown that on average the best results in term of prediction of the coevolving positions and speed of the algorithm, are obtained when the 1^st^ and 2^nd^ sequence of the resorted MSA are the two most similar sequences.

In a refined version of the algorithm, also a covariance matrix (COV) is calculated from the ‘differential’ binary MSA: the MI matrix, and the covariance matrix are scaled by linear regression and merged. Finally, the ZPX2 matrix is calculated from the merged MI/COV matrix.

There are special advantages in calculating the MI matrix from a binary MSA of 0's and 1's. For example, calculation of a dbZPX2 matrix from a MSA of 300 sequences of 280 aa's takes less than 2s.

As previously noted, the basic formulation of the binary method discards the information about the type of aa that changes between two sequences at a given position in the MSA. We have used two avenues to reintroduce this information in the method. One avenue relies on introducing a ‘global’ binary representation of the MSA [12], in which each sequence of length *n* (possibly containing gaps) is treated as a vector of 0's and 1's of size 21×*n*: in this representation each column of the original MSA is expanded in 21 columns of the binary MSA. For example, if the original MSA consists of 10 rows and 10 columns, the equivalent global binary MSA consists of 10 rows and 210 columns. In this case, first, a MI matrix is calculated from the simple ‘differential’ binary representation of the resorted MSA producing a matrix of dimensions (10×10). Then, the same resorted MSA is converted to a ‘global differential’ binary matrix. A covariance matrix of dimensions (210×210) is calculated from the ‘global differential’ binary matrix: in this matrix the covariance between columns *i* and *j* of the original MSA is contained in the submatrix of indices [*i*×21:*i*×21+20] and [*j*×21:*j*×21+20]. The Frobenius norm of this submatrix is then used as the value at the *ij* index of a ‘collapsed’ covariance matrix of dimensions (10×10), which reflects directly the original MSA of dimensions (10×10). In the next step, the MI matrix from the ‘differential’ binary MSA, and the covariance matrix from the ‘global differential’ binary MSA are scaled by linear regression and merged as already described for the dbZPX2method. Finally, the ZPX2 matrix is calculated from the merged MI/COV matrix. Because of its derivation from a ‘global binary’ representation of the MSA, the resulting coevolution matrix is named differential global binary ZPX2 (dgbZPX2).

**Figure 2:**
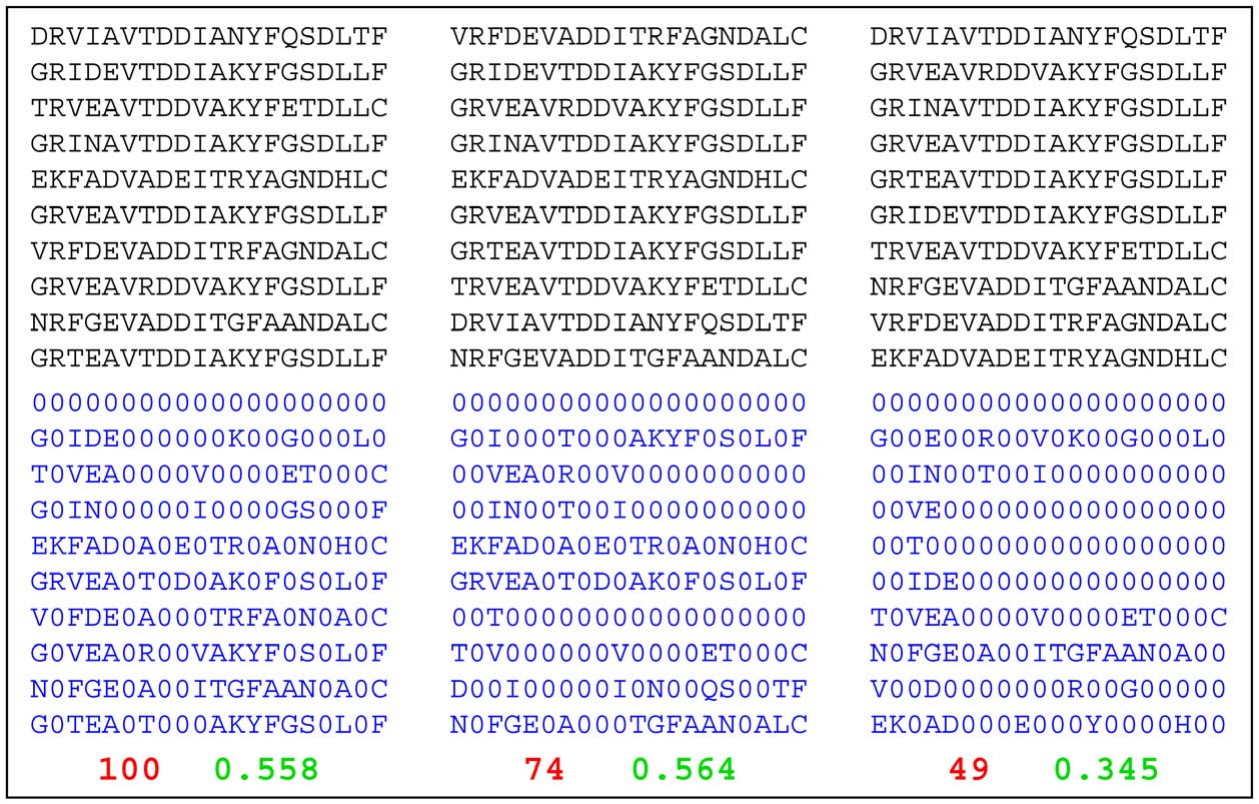
The normal/binary (nb) method. The upper half of the panel shows the same 10 sequences in three different orders. The lower half of the panel shows the corresponding normal/binary translations. The number of non-zero elements and the mean MI of the normal/binary alignments are highlighted in red and green, respectively. The <MI> reaches its lowest values only when the number of non-zero elements is fully minimized.

A second avenue to reintroduce information on the type of aa changes between consecutive sequences in the MSA is based on a combination between the ‘normal’ MSA and the ‘binary’ MSA. In practice all the positions of the resorted ‘normal’ MSA that correspond to a 0 in the resorted ‘binary’ MSA are assigned a value of 0. In this way a ‘no change’ at a position of the MSA between two consecutive sequences is still represented by a 0, while a ‘change’ is represented by the standard range of 21 symbols, including gaps (**Figure 2**). In this approach, the information of repeated changes to the same pair of amino acids in the same positions can be detected by MI. Finally a ZPX2 coevolution matrix is calculated from the MI matrix: because of its derivation from a combination of a ‘normal’ and a ‘binary’ MSA, this type of coevolution matrix is named ‘nbZPX2’.

A flowchart of the db, dgb, and nbZPX2 algorithms is provided as **Text S1**. Matlab functions (NMSA_to_dbZPX2, NMSA_to_dgbZPX2, NMSA_to_nbZPX2) with detailed comments for each step in the three algorithms are provided as part of the MSAvolve Toolbox (Toolbox S1).

### Covariation in simulated MSAs

Typically, some *a posteriori* criteria are employed to ascertain whether the coevolving positions identified from the analysis of a MSA are correct. Among these, the most common one is whether the coevolving positions are close in space in the three-dimensional structure of the protein under study. However, some methods of coevolution detection [10]–[12] often lead to the identification of residues that are distant from each other in the protein structure, with the implication that these residues are involved in a long distance functional connection between sites. Since different strategies of coevolution detection lead to different hypotheses on the role of coevolving residues in protein structure and function, there is really no way of knowing which strategy is correct.

In a first set of studies, we have tested several coevolution detection methods for their capacity to detect residues that were forced to coevolve in the simulated MSAs of 8 protein families (KDO8P synthase, (KDO8PS [33]), the F_1_ chaperones Atp11p and Atp12p [34], the catalytic subunit ArsA of the arsenic transporter [35], the arsenate reductase ArsC [36], *p*-hydroxybenzoate hydroxylase (PHBH [37]), phthalate dioxygenase reductase (PDR [38]), and (*S*)-mandelate dehydrogenase (sMDH [39])), with which we are particularly familiar because of our previous work on the X-ray structures of representative members of these families. For each family, 100 different MSAs were simulated with MSAvolve (see Methods). Details of the simulations (e.g., number of sequences, number of covarions, recombination sites) are given in Table 1.

**Table 1:** Simulated MSAs and reference experimental MSAs and X-ray structures for the set of 8 protein families analyzed in this study.

Protein	KDO8PS	ArsA	ArsC	PHBH	PDR	MDH	Atp11p	Atp12p
PDB	2QKF	1IHU	1JZW	1DOB	2PIA	1HUV	2P4F	2R31
UniProt	Q70JW1	P08690	P08692	P20586	P33164	n.a.	Q6FJS2	A1B060
# sequences	348	202	294	183	271	391	178	230
# positions	280	583	141	394	321	353	205	236
# covarions	28	87	21	59	48	53	31	24
Covarions relative entropy	Low or medium	Medium	Medium	Low, medium	Low, medium, high	Low, medium	Low	Low
# recomb. zones	9	18	9	13	15	13	10	10
crossover points	[1] 20 61 89 167 193 216 235 259 [280]	[1] 19 45 86 114 148 206 228 280 302 337 361 388 423 453 503 519 567 [583]	[1] 9 12 34 65 93 107 127 137 [141]	[1] 12 45 69 102 159 184 210 237 269 293 343 385 [394].	[1] 13 43 57 81 103 123 145 171 199 223 245 277 291 309 [321]	[1] 22 48 76 106 126 153 213 228 252 286 305 335 [353].	[1] 10 29 43 64 91 107 124 137 162 184 [205]	[1] 12 37 57 70 103 122 142 178 211 [236]
Figure #	S1	S2	S3	S4	S5	S6	S7	S8

Since MSAvolve builds coevolution matrices of each simulated MSA by counting the mutations that segregate at specific times during the evolution of a protein, this function can be equated to that of an external observer who witnesses the historical process of evolution. With respect to this point there are two observers in MSAvolve: one is a ‘low power’ observer, who counts all the co-segregating mutations regardless of whether they originate from chance, recombination, or functional/structural demands (the latter are the mutations under constraint of coevolution). There is also a ‘high power’ observer, who counts selectively the true co-segregating mutations of functional/structural significance.

**Figure 3:**
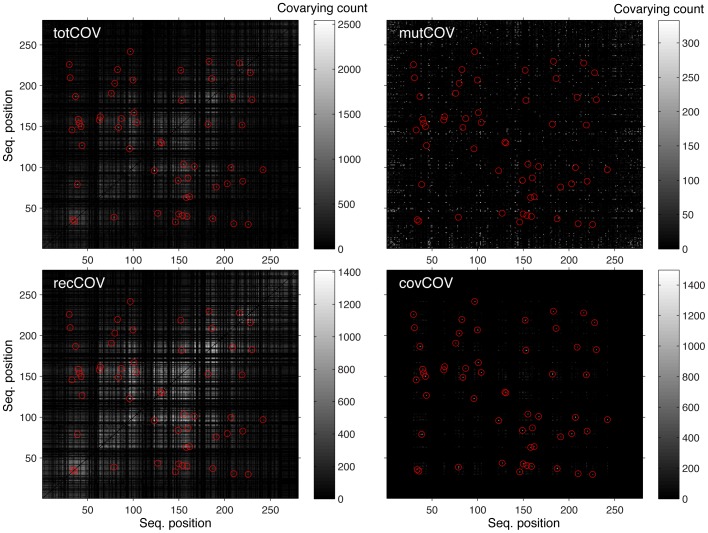
Coevolution matrices derived from a simulated MSA. ***totCOV***: total count of all coevolution events. Although this matrix is built independently during the simulated evolution of the MSA from a single ancestor, it can also be obtained as the sum of the mutCOV, covCOV, and recCOV matrices (see below). Residue pairs under coevolution constraint (true covarions) are indicated by red circles. ***mutCOV***: count of coevolution events due to random point mutations at positions that are not set to coevolve. ***recCOV***: count of coevolution events due to recombination. This count includes residues pairs that are true covarions. ***covCOV***: count of true covarions. There are counts also at pairs of positions that were not set to be covarying because when two or more covarion pairs mutate and segregate also the cross-counts between pairs are added.

An example of the information gathered by the observers (as reported in the construction of coevolution matrices) is shown in **Figure 3** for one simulated MSAs of KDO8P synthase. The panel labeled ‘totCOV’ represents the total count of all the coevolution events. The residue pairs that are under constraint of coevolution are indicated by red circles. The panel labeled ‘mutCOV’ represents the count of the coevolution events due to random point mutations at positions that are not set to coevolve. The panel labeled ‘recCOV’ represents the count of coevolution events due to recombination; since recombination affects simultaneously large blocks of sequence, its effects on the coevolution count are much larger that those of point mutations.

The matrix labeled ‘covCOV’ is the count of the true covarions. In this matrix there are counts also at pairs of positions that were not set to covary. This is due to the fact that when by chance two or more covarion pairs mutate and segregate during an arbitrary amount of time, also the cross-count between pairs are added. For example if pairs 12–34 and 70–93 both mutate, then the count increases by 1 not only for those two pairs but also for the pairs 12–70, 12–93, 34–70, and 34–93. Since are not always the same pairs that mutate at the same time, the background from cross-pairs counts is not as high as the count from the true covarying pairs.

While the individual matrices are built independently during the simulated evolution of the MSA from a single ancestor, in the end they are all consistent with each other: for example, a matrix identical to the totCOV matrix is obtained independently as the sum of the mutCOV, covCOV, and recCOV matrices.

### Detection of covariation in simulated MSAs

Calculated coevolution matrices like that shown in **Figure 3** describe in full the true coevolutionary history of a simulated MSA, and can be compared with those derived for the same MSA by some of the leading methods to detect coevolution. In this study we have tested several established algorithms based on mutual information (MI), including Z-scored cross-product positional MI (ZPX, and its square, the form used here, which we refer to as ZPX2) [21], logR [22], Direct Coupling Analysis (DCA) [24], [29], and the three binary methods (db, dgb, and nbZPX2) described above.

We have also tested several non-MI algorithms including Observed Minus Expected Squared Covariance (OMES) [8], [9], McLachlan Based Substitution Correlation (McBASC) [13], [40], Explicit Likelihood of Subset Co-variation (ELSC) [10], Statistical Coupling Analysis (SCA) [11], [12], [41]. Sparse Inverse Covariance (PSICOV) [23] could not be tested because all the simulated MSAs were smaller than 500 sequences, and the original PSICOV program (downloaded from the authors' website) uses the GLASSO sparse inverse function [42], which has problems of convergence with less than 500 sequences. We modified the original code in order to override the default minimum requirement of 500 sequences, but in that case PSICOV failed to converge to a solution with most of our MSAs.

A measure of how well different methods identify covarying pairs in a set of 100 simulated MSAs of a protein family can be obtained by using the counts of the true coevolution events for each pair provided by the covCOV matrices as the values for the corresponding pairs identified by the different methods. In this analysis the *ij* pairs of the covCOV matrix and the ‘method’ matrix are first sorted in descending order based on their value. Then, the values of the *ij* pairs of the covCOV matrix are assigned to the corresponding *ij* pairs of the ‘method’ matrix. For example, if pair 20∶40 was counted 500 times in the covCOV matrix the corresponding pair 20∶40 of the ‘method’ matrix is assigned a value of 500 regardless of its original value. In this way a plot of the cumulative sum of the values of the sorted ‘method’ matrix visualizes how much the coevolution recognition provided by the method approaches the ‘ideal’ coevolution recognition provided by the cumulative sum of the values of the sorted covCOV matrix.

**Figure 4:**
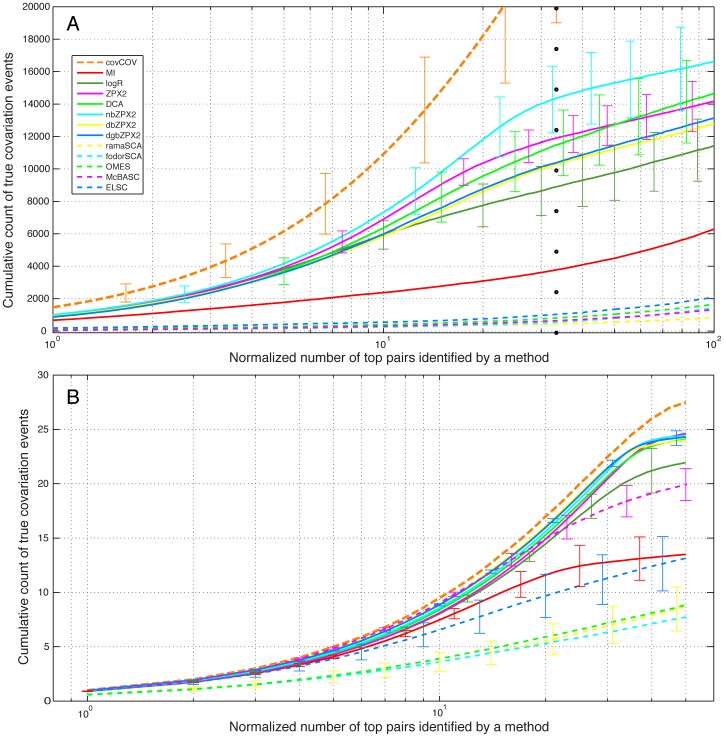
Averaged results for 8 protein families with simulated MSAs under 500 sequences. Cumulative count of covariation events corresponding to the top scoring pairs in the coevolution matrices generated by different methods. **A.** MSAs simulated with MSAvolve: a dotted vertical line marks the total number of true covarying pairs controlled by the program (as shown in Table 1). **B.** MSAs simulated with SIMPROT: in these simulations a total number of 50 covarying pairs was used regardless of the sequence length. Since each curve of the two panels is not the average of independent replicas of the same experiment, traditional standard deviation (*std*) has no meaning in this case. The error bars for selected points *i* represent a weighted *std* (wσ_i_) calculated as follows:
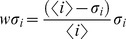
.

Results of this analysis were first averaged over all 100 simulated MSAs of each protein family (see **Figure S1, S2, S3, S4, S5, S6, S7, S8**). Then, the results obtained with all 8 protein families were further merged (**Figure 4A**). Based on this synthesis, nbZPX2 was on average the most effective method to identify covarying positions in these protein families, although both DCA and ZPX2 were within the margins of error of nbZPX2.

One might wonder if the results obtained with different coevolution detection methods are somehow biased by the particular program (in this case MSAvolve) used to simulate the MSAs. For this reason, we also simulated 100 MSAs for each of the 8 families using an earlier program, SIMPROT, developed by one of us [32], which is based on a simulation strategy completely different from that of MSAvolve. In these simulations, there is no recombination between fragments and no cross-covariations between pairs, and thus the background of stochastic covariation is much lower than in the simulations carried out with MSAvolve. As expected, the coevolution detection performance of all the methods (summarized in **Figure 4B**) was closer to the internal ideal record generated by the program (orange line). With the exception of logR, which scored more poorly with weak covarions (tail of the cumulative covarions count in **Figure 4B**), the newest methods (ZPX2, db/dgb/nbZPX2, and DCA) all scored very similarly to each other. Among the remaining methods, ELSC approached the performance of simple MI, and McBASC exceeded it, still showing lower performance than logR in the detection of weakly covarying pairs. OMES and SCA were again much less effective than even simple MI in the entire range of covariation strength tested.

### Prediction of close contacts in X-ray crystal structures

A second approach we have used to evaluate the performance of different coevolution detection methods is based on the assumption that a large proportion of all coevolving residues in a protein are residues in close proximity of each other. ZPX, logR, DCA, and PSICOV [21]–[24], [28], [29] were developed with the specific goal of separating direct from indirect coupling between residues, and thus are expected to be more selective in the detection of coevolving positions that are close in space, but distant in sequence, in the structure of representative members of a protein family. In order to verify this claim, the experimental MSAs of the 8 protein families that served as reference in the MSA simulations, and a larger set of MSAs of 150 protein families (downloaded from http://bioinf.cs.ucl.ac.uk/downloads/PSICOV/suppdata) were examined with the same coevolution detection methods used in the analysis of the simulated MSAs. We kept the two sets separate, because in the first case (8 protein families) all the experimental MSAs contained less than 500 sequences, while in the second case (150 protein families) all the experimental MSAs contained more than 1000 sequences. The performance of PSICOV (which has a lower limit of 500 sequences) could be tested only with the larger set.

**Figure 5:**
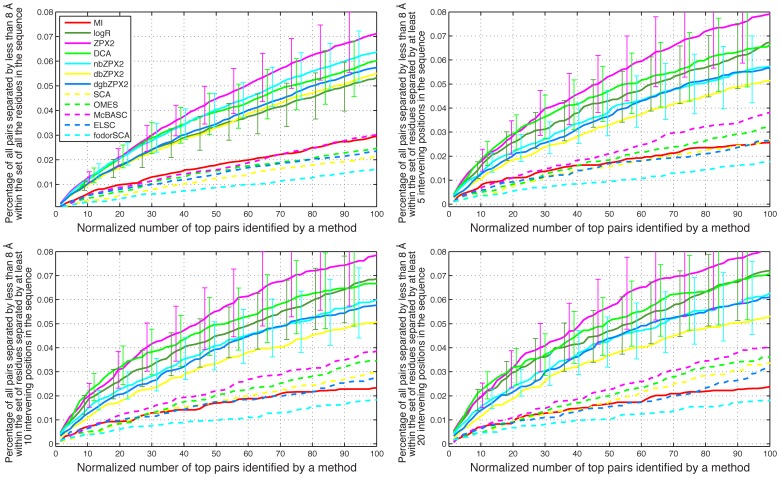
Averaged results for 8 protein families with experimental MSAs under 500 sequences. Each panel shows the percentage in the top coevolving pairs identified by each method, among the residue pairs separated by less than 8 Å in the X-ray structure of each protein. The abscissa scale is normalized in such a way that 100 corresponds to a number of pairs equal to the number of residues in the sequence. In the **top left** panel all protein pairs are considered, including those represented by consecutive residues in the sequence. In the **top right** panel only pairs whose residues are separated by at least 5 intervening positions in sequence space are considered. In the **bottom left** and **bottom right** panels only pairs whose residues are separated by at least 10 and 20 intervening positions in sequence space are considered.

**Figure 6:**
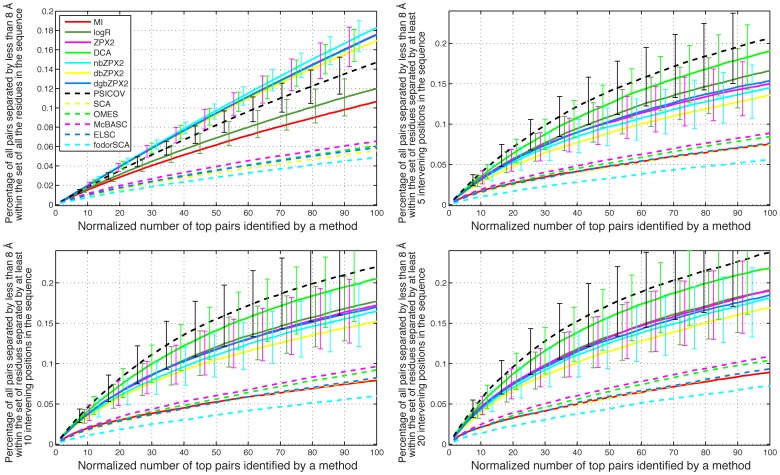
Averaged results for 150 protein families with experimental MSAs larger than 1000 sequences. The meaning of each panel is the same as in Figure 5.

To quantify the detection of close contacts, we measured what percentage of all residue pairs separated by less than 8 Å in the X-ray structure was represented in the top coevolving pairs identified by each method (**Figure S1, S2, S3, S4, S5, S6, S7, S8** **, panels D–E**; see also the **Rightmost panels** for examples of contact maps predicted by some of the methods). A number of pairs equal to the number of residues in each sequence was considered. This result was further filtered in order to include either all the pairs or only pairs whose component residues are separated by at least 5, 10, and 20 positions in sequence space. The merged result of this analysis for the small set of 8 experimental MSAs and the larger set of 150 MSAs are shown in **Figures 5** and **6**, respectively.

The averaged results obtained for both sets of families show that the newest methods (ZPX2, db/dgb/nb_ZPX2, logR, DCA, PSICOV), are all within each other's margins of error, and are generally superior to the older methods (MI, OMES, McBASC, ELSC, SCA) (**Figures 5** **,** **6**). The number of sequences in the MSA is clearly an important factor in determining the accuracy of the predictions. For example, no more than 8% of all the pairs separated by less than 8 Å are found in the set of 8 families with MSAs smaller than 500 sequences (**Figure 5**), while at least 20% are found in the set of 150 families with MSAs larger than 1000 sequences (**Figure 6**).

## Discussion

The current study was initiated to provide an updated testing of the most commonly used methods to detect coevolution from only sequence data, using both simulated and experimental MSAs. In the case of simulated MSAs, we used two different programs, MSAvolve and SIMPROT, to generate more than 1600 different MSAs for 8 families of proteins of different sizes, folds and functions. The ‘true’ coevolution matrices recorded by the programs were compared with the coevolution matrices obtained for the same evolved MSAs with a variety of MI and non-MI methods. The same methods were also tested with the experimental MSAs of the same 8 families and of additional 150 families, for their ability to identify coevolving pairs that matched pairs of residues within 8 Å of each other in the representative X-ray structures.

**Figure 7:**
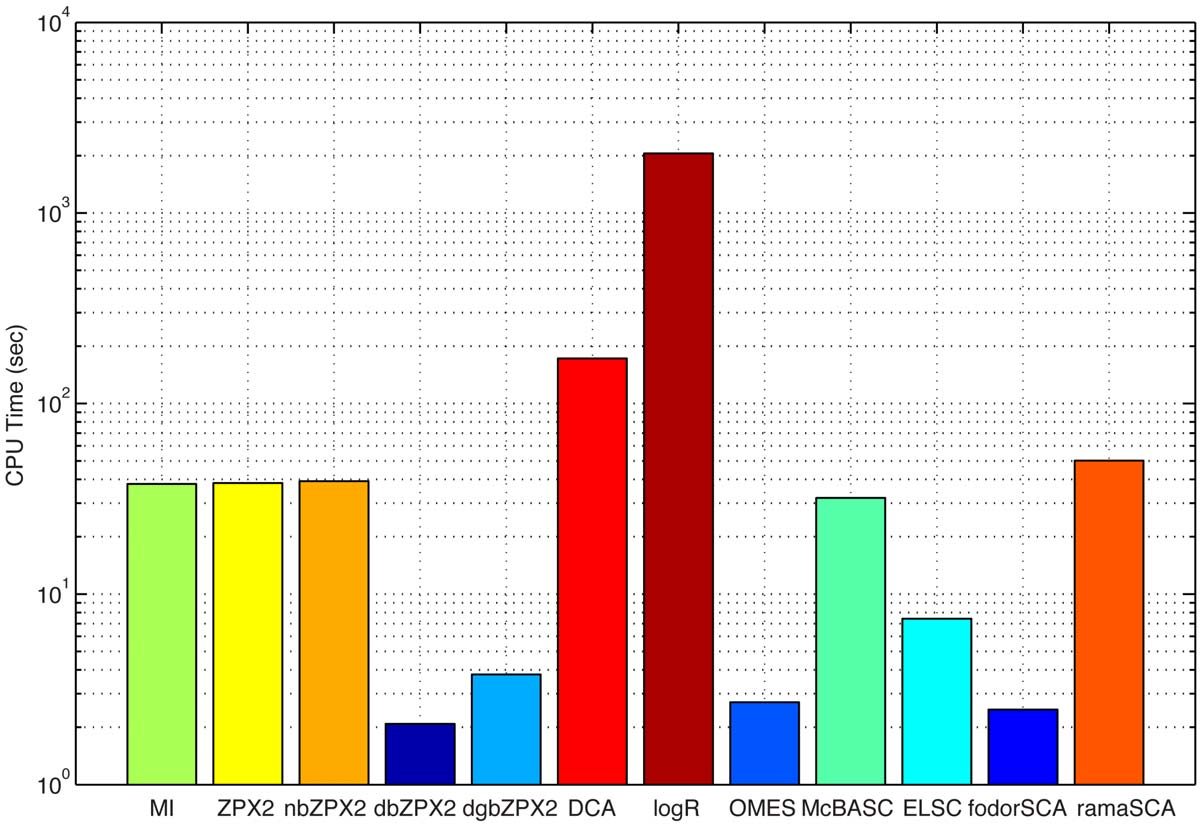
Execution (CPU) time of different coevolution detection methods for the experimental MSA of PHBH (183 seq.×394 aa.).

The averaged results obtained for both sets of families show that the most advanced methods (ZPX2, db/dgb/nbZPX2, logR, DCA, PSICOV), which take into account the global correlations between pairs, are generally superior to methods based only on local correlations (MI, OMES, McBASC, ELSC, SCA) in their capacity to identify coevolving residues using either simulated (**Figure 4**) or experimental MSAs (**Figures 5** **,** **6**). However, there are significant differences in execution time between the best methods, which become particularly pronounced with longer sequences. For example, logR performed very well with experimental MSAs, but it was also the slowest of all the methods tested (**Figure 7**).

Most coevolution detection methods will work best with MSAs of at least 1000 sequences in order to obtain the level of accuracy required for structure prediction (as shown by a direct comparison of **Figure 6** with **Figure 5**). However, earlier work by Martin [16] has shown that useful coevolution signals can be extracted from as few as 130 sequences. In the case of our set of 8 protein families (with MSAs ranging in size between 178 and 348 sequences), the best coevolution detection methods performed reasonably well despite the small number of sequences, and contact maps predicted from coevolving pairs revealed the salient features of the contact maps derived from the representative X-ray structure of each family (**Figure S1, S2, S3, S4, S5, S6, S7, S8, Rightmost panels**). We also found a good correlation between the performance of each method in simulated (**Figure S1. S2, S3, S4, S5, S6, S7, S8, panels A–C**) and experimental (**Figure S1, S2, S3, S4, S5, S6, S7, S8, panels D–E**) MSAs, suggesting that testing different coevolution methods with simulated MSAs of the protein of interest, can help identify which method is the most appropriate for the specific sequence length and the size and statistical features of the MSA. Simulated MSAs may in fact represent a more sensitive tool for testing coevolution detection methods because the level of background noise that obscures the true covariation signal can be increased as needed in order to discriminate between strong and weak methods (see for example the difference in methods performance between **Figure 4A and 4B**).

We also point out that the goal of structure prediction is not the only justification for studying coevolution in proteins. Clearly, there are many situations (e.g. residues that affect stability in opposite directions) in which the true covarions are not in close contact. There are also situations in which consecutive residues in the sequence are part of the active site, and coevolve. Thus, deciding which coevolution detection methods to use solely on the base of their capacity to predict from experimental MSAs the existence of close contacts between distant residues in sequence space introduces a bias toward structure prediction that may lead to the use of sub-optimal methods for other types of biochemical studies.

Whether one considers simulated or experimental MSA there is significant variability in the performance of different methods with individual MSAs (as evidenced by the large error bars in **Figures 4** **,** **5** **,** **6**; see also **Figure S1, S2, S3, S4, S5, S6, S7, S8**). This variability suggests that there is not a single best method for all proteins, and that short trial runs with simulated MSAs that reproduce faithfully the statistical properties of the experimental MSA can be very useful in deciding which method is likely to provide the best identification of covarions with a specific protein. Our new program, MSAvolve, offers a wide range of customization of the simulations, and thus it is ideally suited for this task.

This study introduces three new algorithms, dbZPX2, dgbZPX2, and nbZPX2, which comprise a new class of MI methods termed ‘differential binary’. These methods aim to capture only substitution events and translate the MSA into a matrix of 0's (no change from the amino acid at the same position in the previous sequence) and 1's (where a substitution occurred). Furthermore, the order of the original sequences in the MSA is changed in order to minimize the number of changes (the number of 1's) between juxtaposed sequences, and, as a consequence, in the entire MSA. This operation generally results in MSAs that are ordered phylogenetically, and leads to a decrease in the mean mutual information of the columns of the corresponding binary MSA. Thus, in contrast to methods like DCA [24], [28], which are grounded in the principle of entropy maximization, the binary methods work by *minimizing mutual information*. The overall decrease in the average MI of the alignment allows for an easier identification of concerted amino acid changes in sequences throughout the alignment by reducing the entropy associated noise. This is reminiscent of the method of Gloor *et al.* [17], who divided MI by the joint entropy to reduce the effect of entropy on MI. Strategies of MI minimization are commonly used also in other fields of science. For example, in signal processing some of the algorithms for ‘independent component analysis’ (ICA) rely on finding a representation of the signal sources by either maximizing the entropy of the signal probability distribution or by minimizing the output mutual information [43], [44].

Altogether the binary methods provide a fast numerical solution to the problem of identifying covarying residues from sequence data alone. For example dbZPX2 and dgbZPX2 are about 50-25 times faster than DCA (**Figure 7**). Since these methods are only slightly less sensitive than the other best performers (**Figures 4** **,** **5** **,** **6**), they offer a good compromise between speed and performance, and thus may be particularly useful for the analysis of very large MSAs of very large proteins.

## Methods

### Simulation of MSAs with MSAvolve

MSAvolve v.1.0a was implemented as a Matlab Toolbox under BSD licensing: it is provided as a zip file (**Toolbox S1**) as part of the Supporting Information. Details of the simulations carried out in this study are provided as **Text S2**. Details of the additional features and of the internal workings of MSAvolve are provided as **Text S3** and **Figures S9, S10, S11**.

### Experimental MSAs

MSAs for the set of 8 protein families (**MSA S1, S2, S3, S4, S5, S6, S7, S8**) were calculated independently with T-Coffee [45], Muscle [46], and Mafft [47] and then merged together with T-Coffee. A list of the MSAs and PDBs contained in the set of 150 protein families used in this study is provided in **Text S4**. This set was originally described in [23], and can be downloaded from http://bioinf.cs.ucl.ac.uk/downloads/PSICOV/suppdata.

### Coevolution detection methods

The MI algorithms, including db, dgb, and nbZPX2, were implemented inside MSAvolve and are thus available as part of the Toolbox. logR analysis was carried out with the original code kindly provided to us by Lukas Burger and Erik van Nimwegen. DCA analysis was carried out with the original Matlab code kindly provided to us by Andrea Pagnani. OMES, McBASC, and ELSC analyses were carried out using the Java code made available by Anthony Fodor (http://www.afodor.net/). This code also provides an implementation of the SCA algorithm (called here ‘fodorSCA’). SCA analysis in its most recent implementation with noise filtering [12] (called here ‘ramaSCA’) was carried out using the Matlab Toolbox SCA v4.5 kindly made available to us by Rama Ranganathan. Source code for PSICOV was downloaded from http://bioinf.cs.ucl.ac.uk/downloads/PSICOV/suppdata, and compiled according to the authors' recommendations.

### Synthesis of results from all the protein families

Merging of the results obtained with different protein families was achieved by calculating for each trace a ‘shape preserving model’ based on a piecewise cubic Hermite interpolation (PCHIP, [48]) between points. Then, the X scale representing the number of pairs identified by each method was normalized such that the entire range would be represented for all traces by an equal number of points. Finally, for each normalized value of the X axis the corresponding Y values were determined from the fitted models of each curve. This process can be equated to stretching or compressing the different plots in such a way that they all have the same size. The meaning of every point of each plot remains unchanged in this procedure: for example, the dotted vertical line in the averaged plot of **Figure 4A** represents the last of the ‘true’ coevolving pairs, despite the fact that in each individual simulation that vertical line corresponds to a different number on the X axis. The final result is that all the simulations are placed on the same scale and can now be averaged.

## Supporting Information

Figure S1 Performance of MI and non-MI methods with a set of 100 simulated MSAs with covarions in positions of mid-level relative entropy, and with the experimental MSA of KDO8PS. **A.** Distributions among 100 MSAs of the percentage of true covarions in the top 28 zscores of each matrix of different MI methods. Only the fits to the actual distributions are shown. The dashed grey and orange lines represent respectively the counts of covariation events made by the hidden observers inside MSAvolve. The ‘low power’ observer (grey line) sees only the totCOV matrix; the ‘high power’ observer sees the covCOV matrix. **B.** Same as panel A, but for non-MI methods. **C.** Cumulative count of covariation events corresponding to the top scoring pairs in the coevolution matrices generated by different methods. A dotted vertical line marks the 28^th^ highest scoring pair. The inset shows the relative entropy of the experimental MSA with the position of the covarions superimposed. **D.** Coevolution analysis of the experimental MSA of the KDO8PS family. Percentage in the top coevolving pairs identified by each method of all residue pairs separated by less than 8 Å in the X-ray structure of *Neisseria meningitidis* KDO8PS (PDB 2QKF). The abscissa scale is normalized in such a way that 100 corresponds to a number of pairs equal to the number of residues in the sequence. **E.** Same as D, but including in the analysis only pairs whose residues are separated by at least 20 intervening positions in sequence space. **Rightmost panels.** Four examples of contact map predictions using ZPX2, nbZPX2, DCA, and logR with the experimental MSA. Gray regions represent the native map of the representative X-ray structure with a cutoff of 8 Å on the distance between the centroids of different residues. Predictions by the four methods are shown as spots colored as the traces in panels A-E, with the size of each spot proportional to the coevolution score.
(TIF)

Figure S2 Performance of MI and non-MI methods with a set of 100 simulated MSAs, and with the experimental MSA of ArsA. All panels as in **Figure S1**. The top 87 zscores of each matrix of different methods were considered in A and B, and correspond to the vertical dotted line in C. Reference X-ray structure: *Escherichia coli* ArsA (PDB 1IHU).
(TIF)

Figure S3 Performance of MI and non-MI methods with a set of 100 simulated MSAs, and with the experimental MSA of ArsC. All panels as in **Figure S1**. The top 21 zscores of each matrix of different methods were considered in A and B, and correspond to the vertical dotted line in C. Reference X-ray structure: *Escherichia coli* ArsC (PDB 1JZW).
(TIF)

Figure S4 Performance of MI and non-MI methods with a set of 100 simulated MSAs, and with the experimental MSA of PHBH. All panels as in **Figure S1**. The top 59 zscores of each matrix of different methods were considered in A and B, and correspond to the vertical dotted line in C. Reference X-ray structure: *Pseudomonas aeruginosa* PHBH (PDB 1DOB).
(TIF)

Figure S5 Performance of MI and non-MI methods with a set of 100 simulated MSAs, and with the experimental MSA of PDR. All panels as in **Figure S1**. The top 48 zscores of each matrix of different methods were considered in A and B, and correspond to the vertical dotted line in C. Reference X-ray structure: *Pseudomonas (burkholderia) cepacia* PDR (PDB 2PIA).
(TIF)

Figure S6 Performance of MI and non-MI methods with a set of 100 simulated MSAs, and with the experimental MSA of MDH. All panels as in **Figure S1**. The top 53 zscores of each matrix of different methods were considered in A and B, and correspond to the vertical dotted line in C. Reference X-ray structure: *Pseudomonas putida* MDH-GOX chimera (PDB 1HUV).
(TIF)

Figure S7 Performance of MI and non-MI methods with a set of 100 simulated MSAs, and with the experimental MSA of Atp11p. All panels as in **Figure S1**. The top 31 zscores of each matrix of different methods were considered in A and B, and correspond to the vertical dotted line in C. Reference X-ray structure: *Candida glabrata* Atp11p (UniProt Q6FJS2, PDB 2P4F).
(TIF)

Figure S8 Performance of MI and non-MI methods with a set of 100 simulated MSAs, and with the experimental MSA of Atp12p. All panels as in **Figure S1**. The top 24 zscores of each matrix of different methods were considered in A and B, correspond to the vertical dotted line in C. Reference X-ray structure: *Paracoccus denitrificans* ATP12p (UniProt A1B060, PDB 2R31).
(TIF)

Figure S9 Covariance, branch and entropy distributions in the experimental and in simulated MSAs generated with MSAvolve. **A.** Distributions of the overall similarity score (OSS, see **Text S3**) values among 100 simulated MSAs of the Atp12p family (blue histogram), correlation between the relative entropy profiles of the experimental and simulated MSAs (yellow histogram), mean correlation between the HMM emissions calculated from the experimental and from the simulated MSAs (orange histogram), correlation between the ancestor and the consensus sequence derived from the final MSA (grey histogram). **B.** Cluster size in the experimental MSA (blue histogram), branch size requested to MSAvolve for this round of simulation (green histogram), branch size found *a posteriori* by cluster analysis of a simulated MSA selected at random from the set of 100. **C.** Covariance analysis of the experimental MSA (REF, upper row) and of the simulated MSA (#10, lower row). Upon eigen decomposition, the columns in the eigenvector matrix represent the principal components (PCs) of the covariance matrix, and the coefficents in each vector represent the contributions of the various sequences to the direction of that principal component in the *n*-sequence space. A scatter plot of the first three PCs reveals 5 clusters of sequences in the experimental as well as the simulated MSA. The last inset of the upper row shows the relative entropy at each position of the experimental MSA, and a group of positions (green circles) that are set to coevolve in the simulated MSAs with a group of corresponding positions (red circles). In the last inset of the lower row, the blue trace represents the relative entropy of the experimental MSA, while the red trace shows the relative entropy of the simulated MSA.
(TIF)

Figure S10 Statistical features of simulated MSAs generated with MSAvolve. Rows represent different MSA's including the experimental MSA of the KDO8PS family (top row labeled REF), and 4 simulated ones (labeled with their number in the set), randomly selected from a set of 100. Each simulated MSA was derived from a different ancestor randomly assigned from the emission probabilities at each position of the HMM model of the experimental MSA. In each row, the first two panels from left to right represent a histogram and a heat map of the covariance matrix of each MSA in binary format. The third panel is a spectral analysis of the covariance matrix. A scatter plot of the first two PCs reveals two clusters of sequences in the experimental as well as the simulated MSAs of the KDO8PS family. The fourth panel shows a UPGMA phylogenetic distance tree of the MSAs derived with the Jukes-Cantor method [49], and drawn with the Equal-Daylight algorithm [50]. The fifth panel of the first row shows the relative entropy at each position of the experimental MSA, and a group of positions (green circles) that are set to coevolve in the simulated MSAs with a group of corresponding positions (red circles). The fifth panel in the lower rows shows the relative entropy of the simulated MSAs (red trace) superimposed to that of the experimental MSA.
(TIF)

Figure S11 MSAvolve flowchart. **LEVEL 1**: the simulation starts with 3 identical copies of the ancestor (only the first 9 residues of the ancestor are shown). Each copy is subjected to cycles of mutations (dashed orange arrows) and recombination (green crosses). The height of the crosses reflects which sequences undergo recombination. **LEVEL 2**: the tree is expanded by adding two copies of each of the 3 sequences of level 1 to the MSA matrix, which now contains 9 rows and 3 different sequences derived from a single ancestral protein. **LEVEL 3**: 4 copies of each of the 9 sequences of level 2 are added to the MSA matrix, which now contains 45 rows and 9 different sequences (3 for each of the original 3 branches of the tree) derived from a single ancestral protein. Only the 3^rd^ branch of the tree is shown for level 3. Thin blue arrows highlight steps that can be repeated as desired in both level 2 and 3.
(TIF)

MSA S1 MSA of KDO8PS.
(DOCX)

MSA S2 MSA of ArsA.
(DOCX)

MSA S3 MSA of ArsC.
(DOCX)

MSA S4 MSA of PHBH.
(DOCX)

MSA S5 MSA of PDR.
(DOCX)

MSA S6 MSA of MDH.
(DOCX)

MSA S7 MSA of ATP11.
(DOCX)

MSA S8 MSA of ATP12.
(DOCX)

Text S1 Flowchart/pseudocode of the db, dgb, and nbZPX2 algorithms.
(PDF)

Text S2 Descriptions and simulation conditions for the 8 protein families (KDO8PS, ArsA, ArsC, PHBH, PDR, MDH, ATP11, ATP12) with experimental MSAs under 500 sequences.
(DOCX)

Text S3 *In silico* evolution of a protein family and simulation of multiple sequence alignments with MSAvolve.
(PDF)

Text S4 List of MSAs and corresponding PDB files for the set of 150 protein families analyzed in this study.
(DOCX)

Toolbox S1 MSAVOLVE_v1.0a Toolbox for Matlab.
(ZIP)
