# Supplementary figures and images for: Accurate Simulation and Detection of Coevolution Signals in Multiple Sequence Alignments
Source: PLoS One. 2012 Oct 16;7(10):e47108. doi: 10.1371/journal.pone.0047108 (PMC3473043; doi:10.1371/journal.pone.0047108)

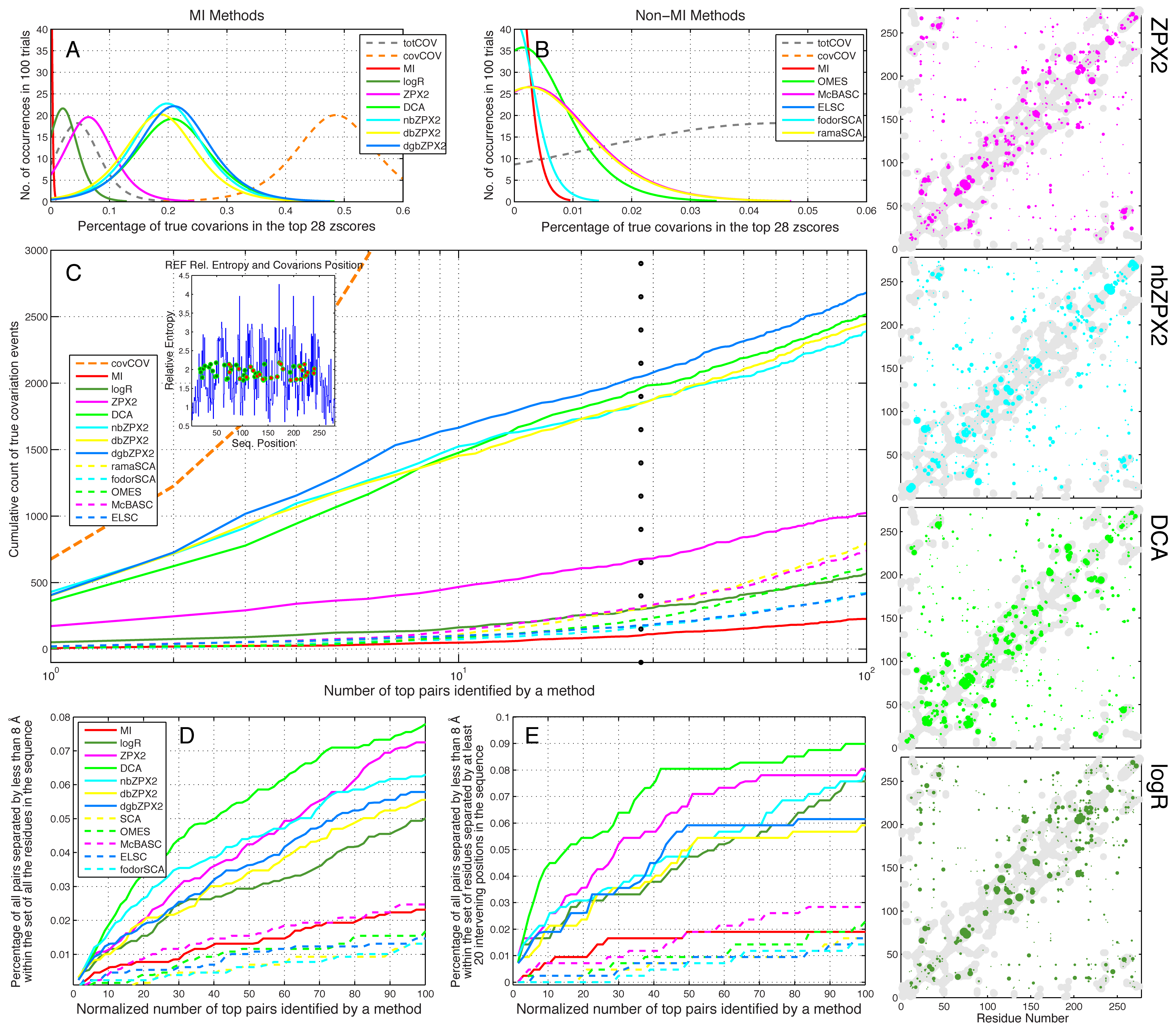

Supplement: Figure S1 — Performance of MI and non-MI methods with a set of 100 simulated MSAs with covarions in positions of mid-level relative entropy, and with the experimental MSA of KDO8PS. A. Distributions among 100 MSAs of the percentage of true covarions in the top 28 zscores of each matrix of different MI methods. Only the fits to the actual distributions are shown. The dashed grey and orange lines represent respectively the counts of covariation events made by the hidden observers inside MSAvolve. The ‘low power’ observer (grey line) sees only the totCOV matrix; the ‘high power’ observer sees the covCOV matrix. B. Same as panel A, but for non-MI methods. C. Cumulative count of covariation events corresponding to the top scoring pairs in the coevolution matrices generated by different methods. A dotted vertical line marks the 28th highest scoring pair. The inset shows the relative entropy of the experimental MSA with the position of the covarions superimposed. D. Coevolution analysis of the experimental MSA of the KDO8PS family. Percentage in the top coevolving pairs identified by each method of all residue pairs separated by less than 8 Å in the X-ray structure of Neisseria meningitidis KDO8PS (PDB 2QKF). The abscissa scale is normalized in such a way that 100 corresponds to a number of pairs equal to the number of residues in the sequence. E. Same as D, but including in the analysis only pairs whose residues are separated by at least 20 intervening positions in sequence space. Rightmost panels. Four examples of contact map predictions using ZPX2, nbZPX2, DCA, and logR with the experimental MSA. Gray regions represent the native map of the representative X-ray structure with a cutoff of 8 Å on the distance between the centroids of different residues. Predictions by the four methods are shown as spots colored as the traces in panels A-E, with the size of each spot proportional to the coevolution score. (TIF) [file pone.0047108.s001.tif]

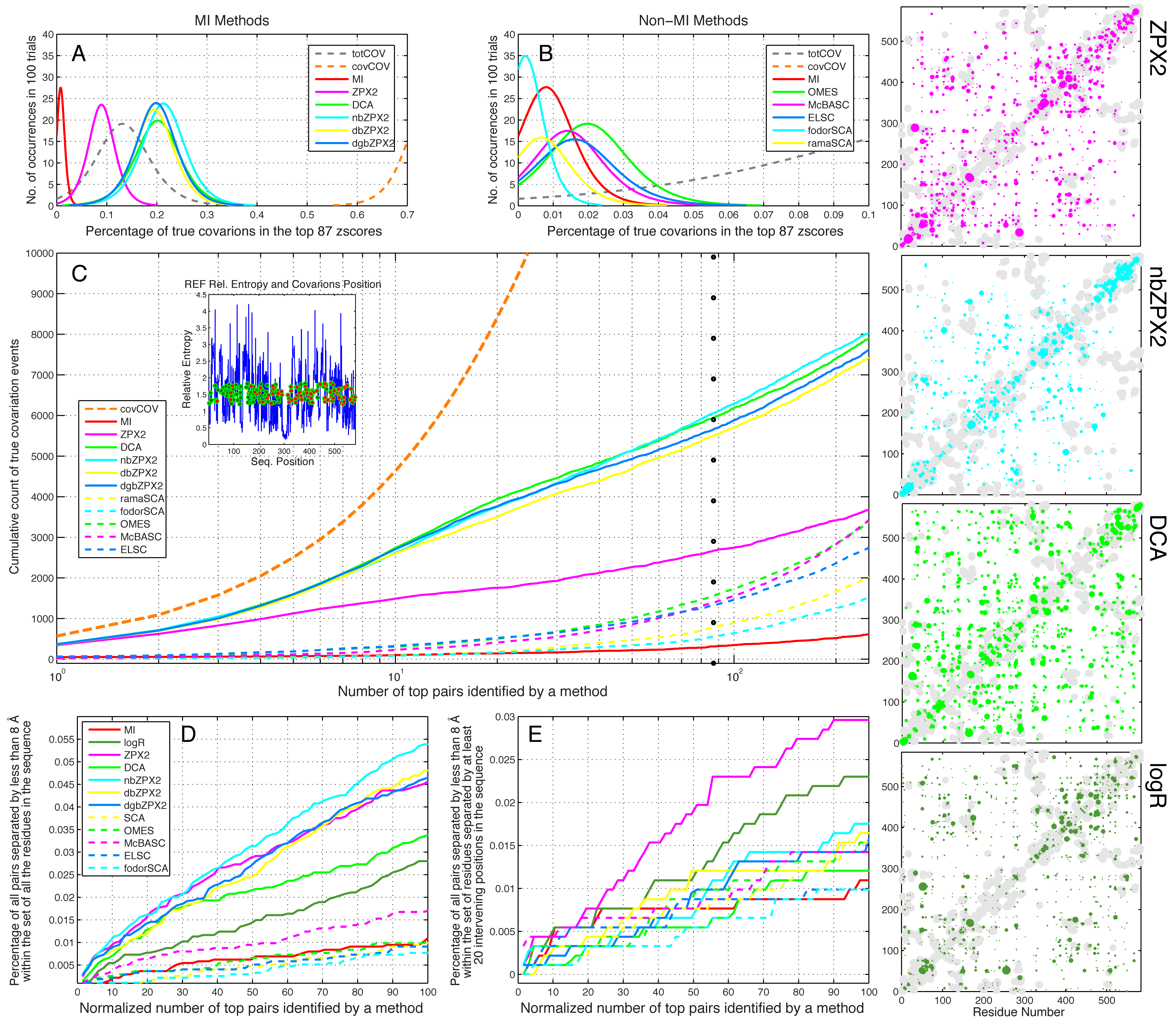

Supplement: Figure S2 — Performance of MI and non-MI methods with a set of 100 simulated MSAs, and with the experimental MSA of ArsA. All panels as in Figure S1. The top 87 zscores of each matrix of different methods were considered in A and B, and correspond to the vertical dotted line in C. Reference X-ray structure: Escherichia coli ArsA (PDB 1IHU). (TIF) [file pone.0047108.s002.tif]

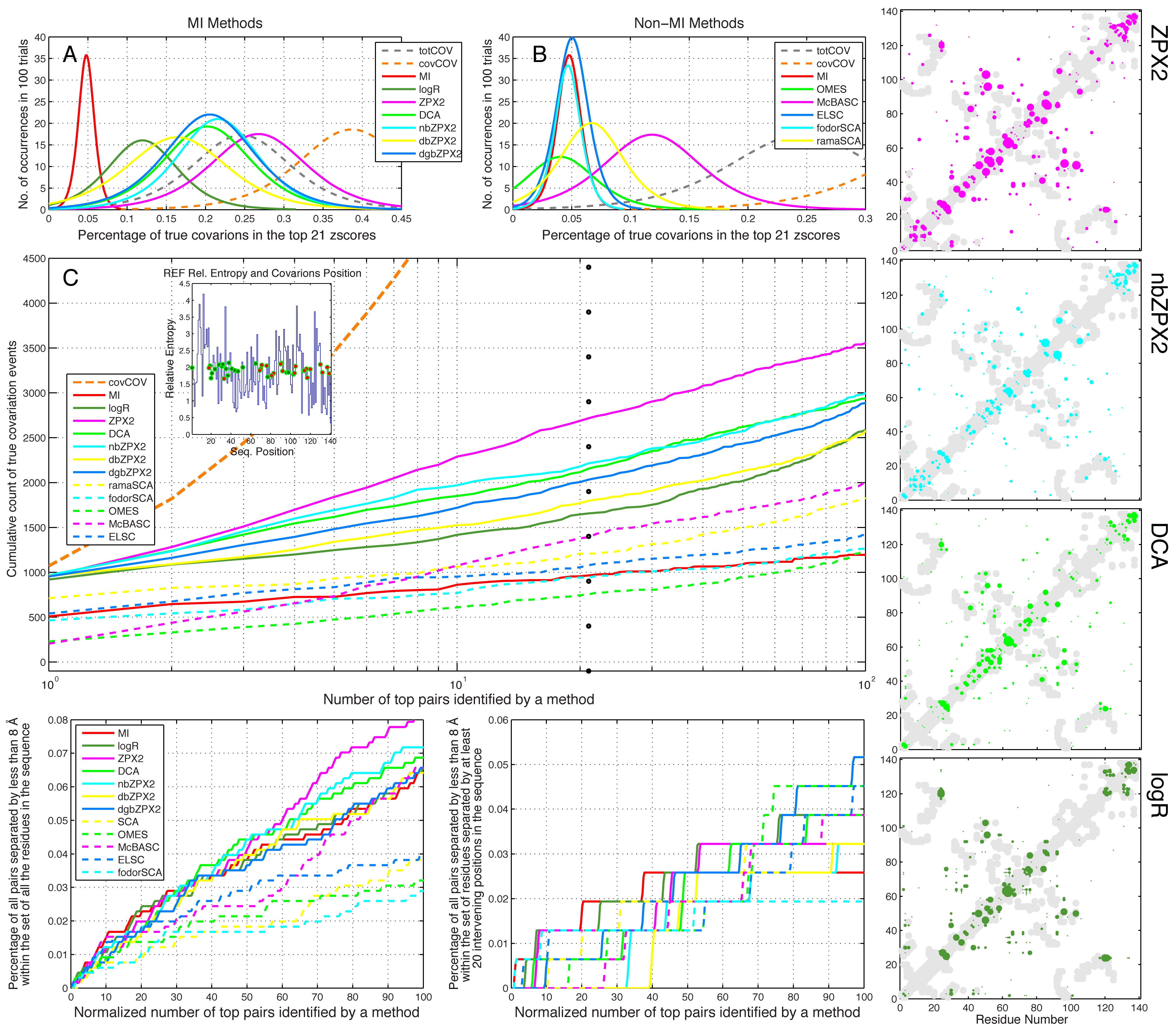

Supplement: Figure S3 — Performance of MI and non-MI methods with a set of 100 simulated MSAs, and with the experimental MSA of ArsC. All panels as in Figure S1. The top 21 zscores of each matrix of different methods were considered in A and B, and correspond to the vertical dotted line in C. Reference X-ray structure: Escherichia coli ArsC (PDB 1JZW). (TIF) [file pone.0047108.s003.tif]

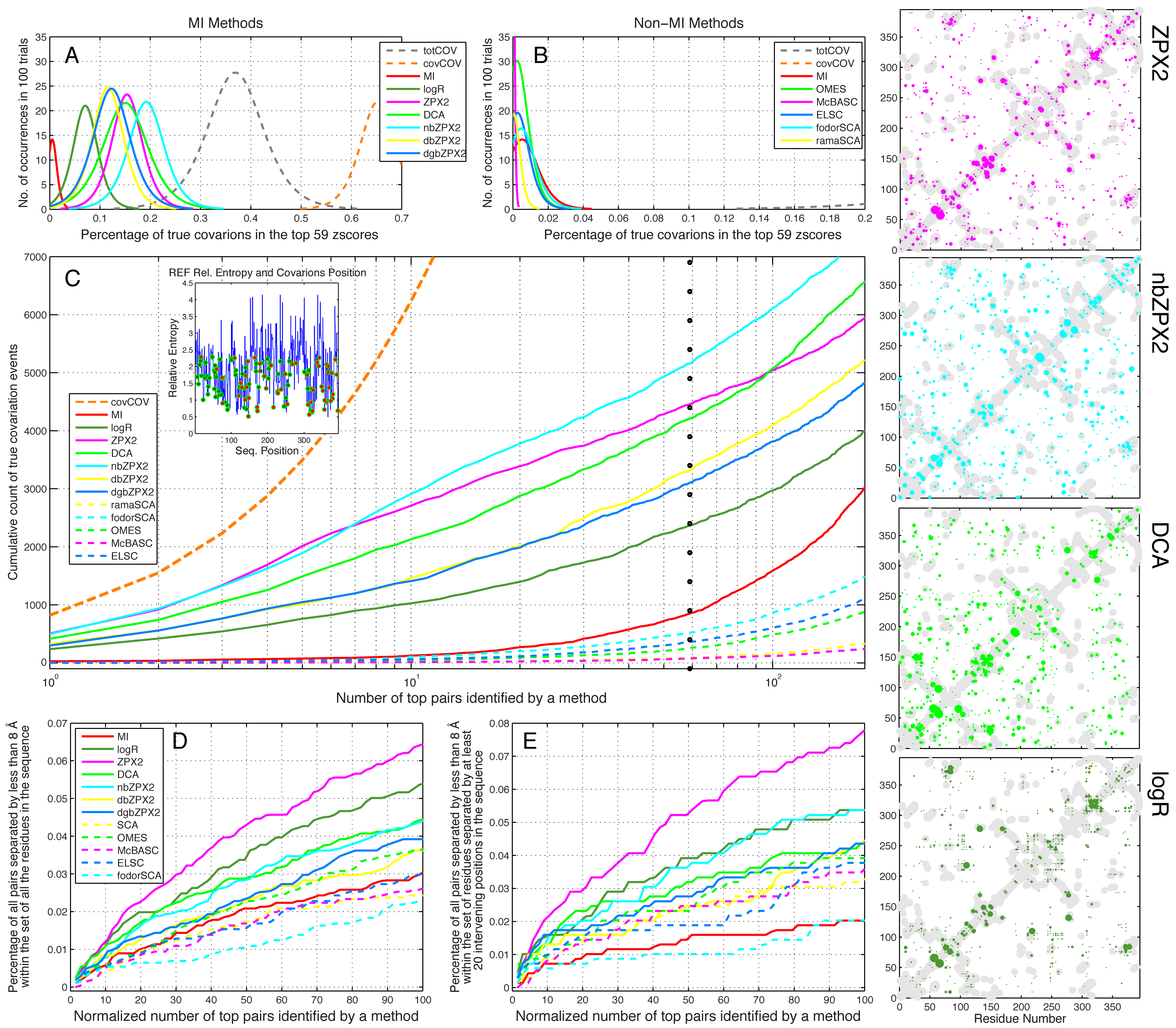

Supplement: Figure S4 — Performance of MI and non-MI methods with a set of 100 simulated MSAs, and with the experimental MSA of PHBH. All panels as in Figure S1. The top 59 zscores of each matrix of different methods were considered in A and B, and correspond to the vertical dotted line in C. Reference X-ray structure: Pseudomonas aeruginosa PHBH (PDB 1DOB). (TIF) [file pone.0047108.s004.tif]

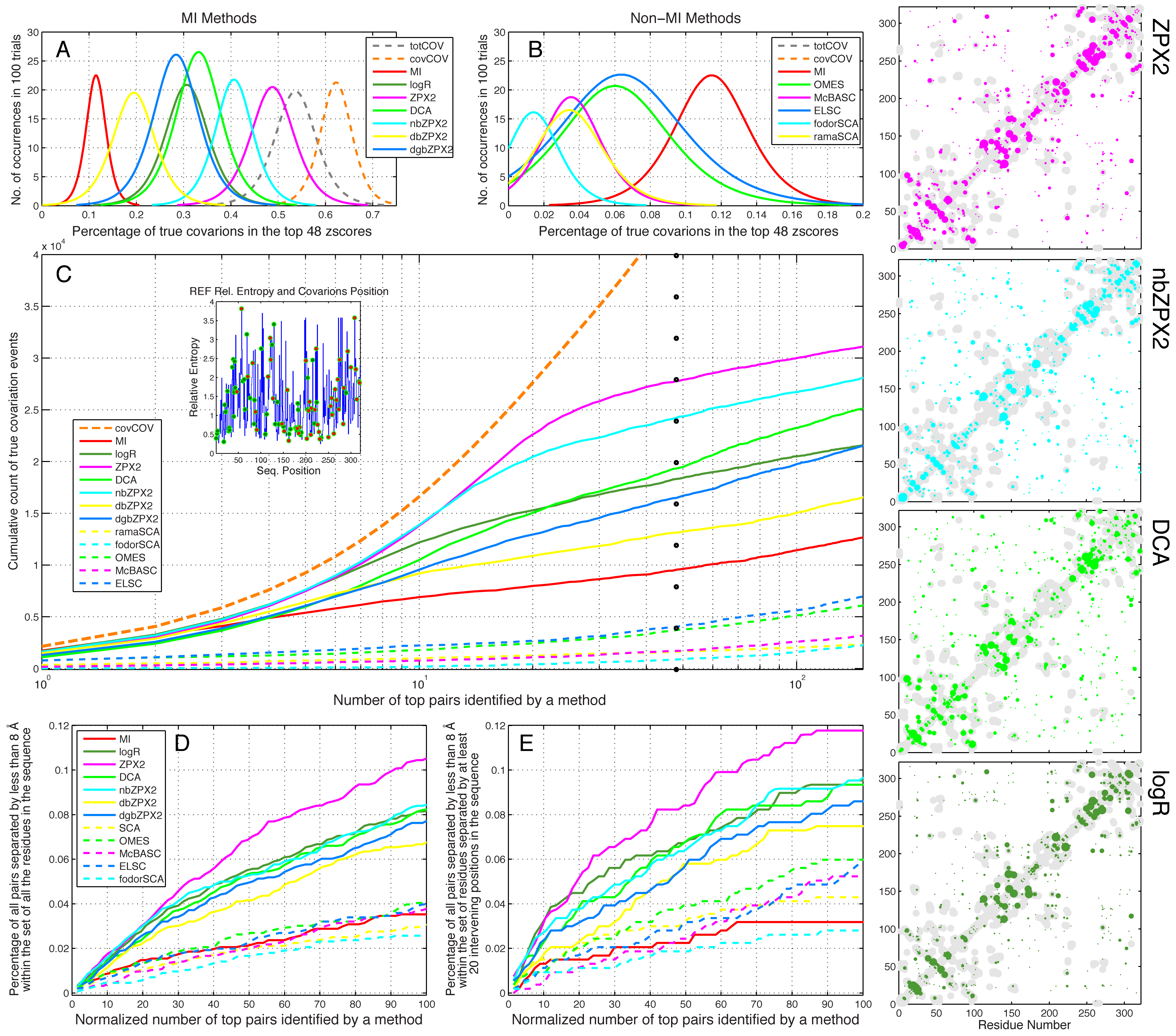

Supplement: Figure S5 — Performance of MI and non-MI methods with a set of 100 simulated MSAs, and with the experimental MSA of PDR. All panels as in Figure S1. The top 48 zscores of each matrix of different methods were considered in A and B, and correspond to the vertical dotted line in C. Reference X-ray structure: Pseudomonas (burkholderia) cepacia PDR (PDB 2PIA). (TIF) [file pone.0047108.s005.tif]

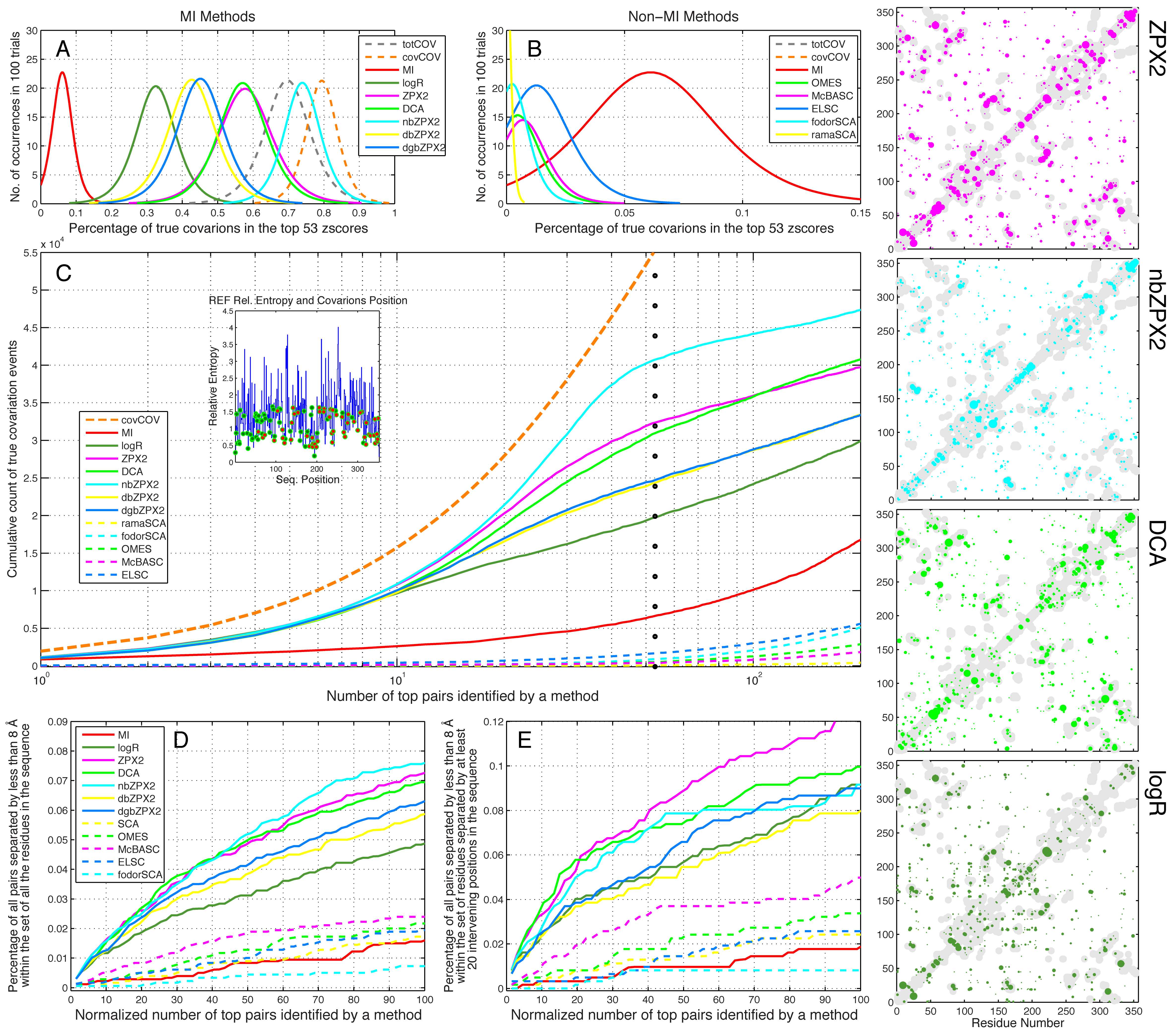

Supplement: Figure S6 — Performance of MI and non-MI methods with a set of 100 simulated MSAs, and with the experimental MSA of MDH. All panels as in Figure S1. The top 53 zscores of each matrix of different methods were considered in A and B, and correspond to the vertical dotted line in C. Reference X-ray structure: Pseudomonas putida MDH-GOX chimera (PDB 1HUV). (TIF) [file pone.0047108.s006.tif]

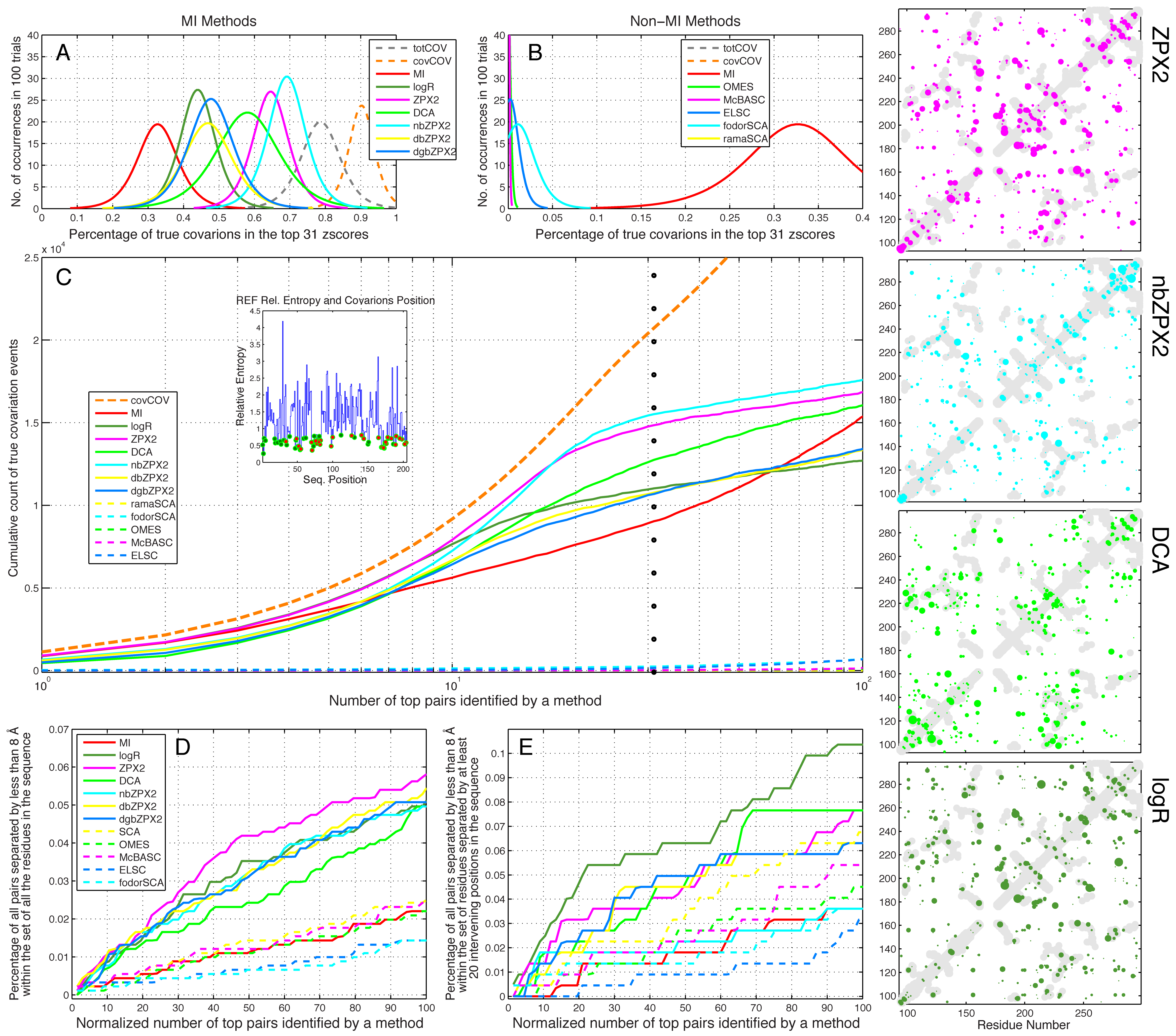

Supplement: Figure S7 — Performance of MI and non-MI methods with a set of 100 simulated MSAs, and with the experimental MSA of Atp11p. All panels as in Figure S1. The top 31 zscores of each matrix of different methods were considered in A and B, and correspond to the vertical dotted line in C. Reference X-ray structure: Candida glabrata Atp11p (UniProt Q6FJS2, PDB 2P4F). (TIF) [file pone.0047108.s007.tif]

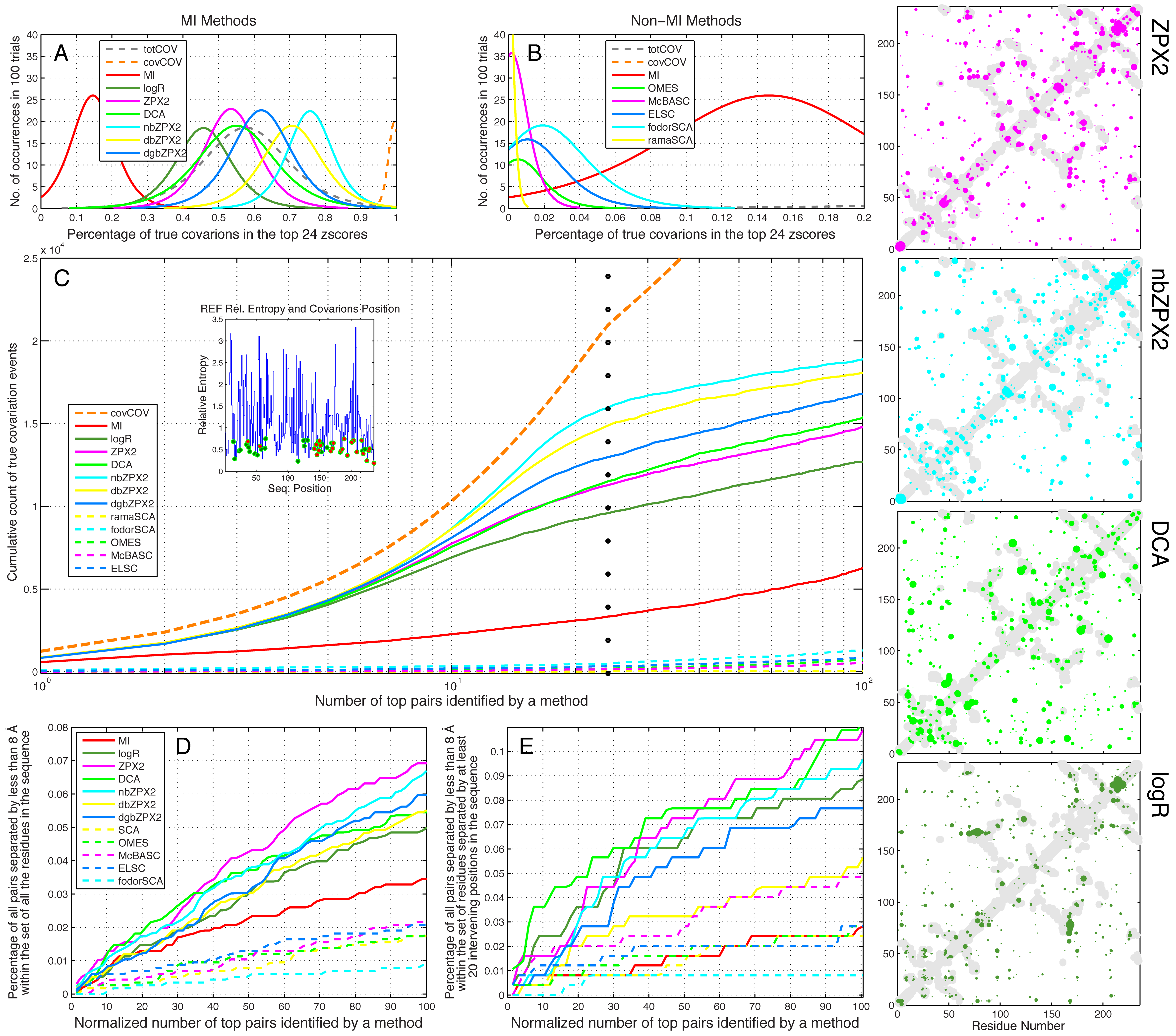

Supplement: Figure S8 — Performance of MI and non-MI methods with a set of 100 simulated MSAs, and with the experimental MSA of Atp12p. All panels as in Figure S1. The top 24 zscores of each matrix of different methods were considered in A and B, correspond to the vertical dotted line in C. Reference X-ray structure: Paracoccus denitrificans ATP12p (UniProt A1B060, PDB 2R31). (TIF) [file pone.0047108.s008.tif]

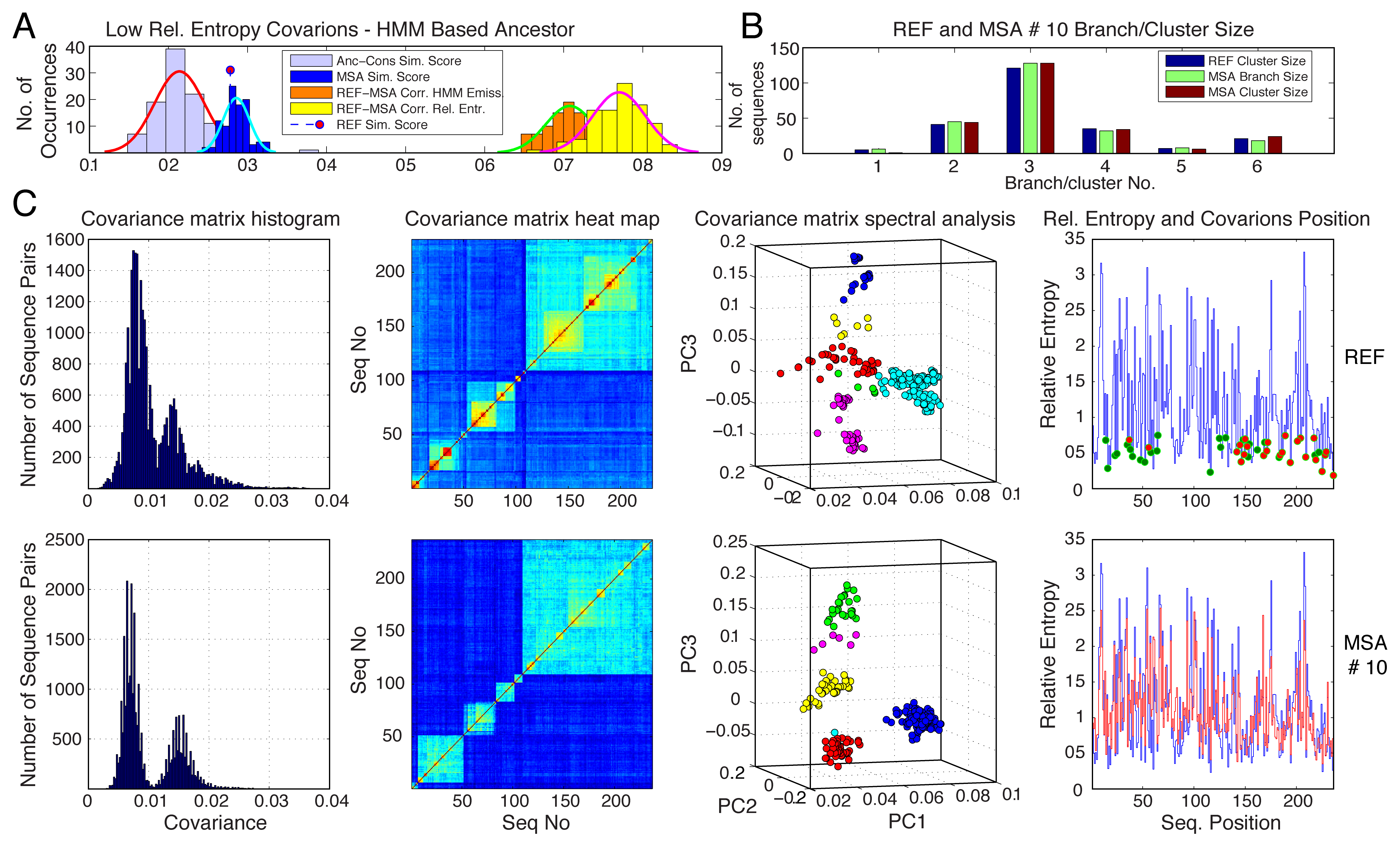

Supplement: Figure S9 — Covariance, branch and entropy distributions in the experimental and in simulated MSAs generated with MSAvolve. A. Distributions of the overall similarity score (OSS, see Text S3) values among 100 simulated MSAs of the Atp12p family (blue histogram), correlation between the relative entropy profiles of the experimental and simulated MSAs (yellow histogram), mean correlation between the HMM emissions calculated from the experimental and from the simulated MSAs (orange histogram), correlation between the ancestor and the consensus sequence derived from the final MSA (grey histogram). B. Cluster size in the experimental MSA (blue histogram), branch size requested to MSAvolve for this round of simulation (green histogram), branch size found a posteriori by cluster analysis of a simulated MSA selected at random from the set of 100. C. Covariance analysis of the experimental MSA (REF, upper row) and of the simulated MSA (#10, lower row). Upon eigen decomposition, the columns in the eigenvector matrix represent the principal components (PCs) of the covariance matrix, and the coefficents in each vector represent the contributions of the various sequences to the direction of that principal component in the n-sequence space. A scatter plot of the first three PCs reveals 5 clusters of sequences in the experimental as well as the simulated MSA. The last inset of the upper row shows the relative entropy at each position of the experimental MSA, and a group of positions (green circles) that are set to coevolve in the simulated MSAs with a group of corresponding positions (red circles). In the last inset of the lower row, the blue trace represents the relative entropy of the experimental MSA, while the red trace shows the relative entropy of the simulated MSA. (TIF) [file pone.0047108.s009.tif]

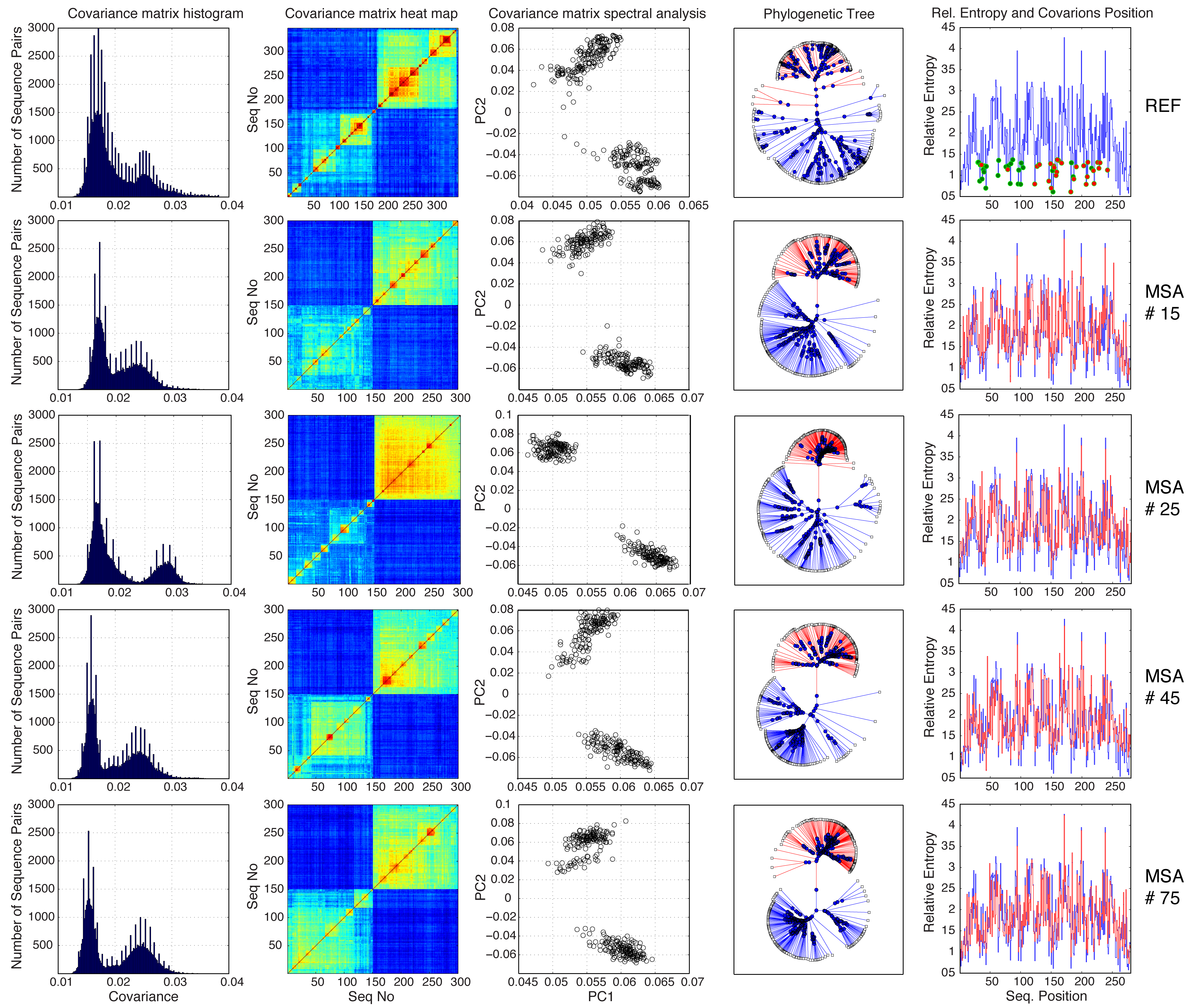

Supplement: Figure S10 — Statistical features of simulated MSAs generated with MSAvolve. Rows represent different MSA's including the experimental MSA of the KDO8PS family (top row labeled REF), and 4 simulated ones (labeled with their number in the set), randomly selected from a set of 100. Each simulated MSA was derived from a different ancestor randomly assigned from the emission probabilities at each position of the HMM model of the experimental MSA. In each row, the first two panels from left to right represent a histogram and a heat map of the covariance matrix of each MSA in binary format. The third panel is a spectral analysis of the covariance matrix. A scatter plot of the first two PCs reveals two clusters of sequences in the experimental as well as the simulated MSAs of the KDO8PS family. The fourth panel shows a UPGMA phylogenetic distance tree of the MSAs derived with the Jukes-Cantor method [49], and drawn with the Equal-Daylight algorithm [50]. The fifth panel of the first row shows the relative entropy at each position of the experimental MSA, and a group of positions (green circles) that are set to coevolve in the simulated MSAs with a group of corresponding positions (red circles). The fifth panel in the lower rows shows the relative entropy of the simulated MSAs (red trace) superimposed to that of the experimental MSA. (TIF) [file pone.0047108.s010.tif]

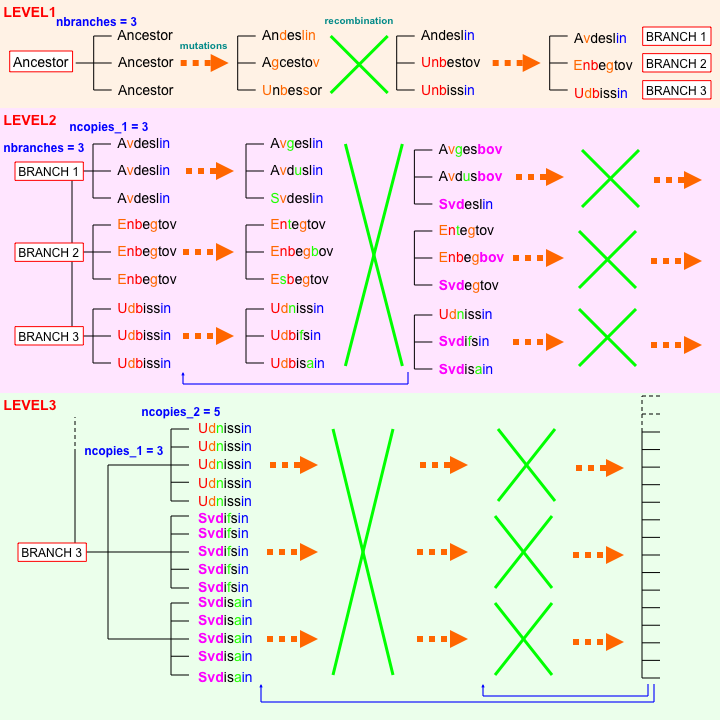

Supplement: Figure S11 — MSAvolve flowchart. LEVEL 1: the simulation starts with 3 identical copies of the ancestor (only the first 9 residues of the ancestor are shown). Each copy is subjected to cycles of mutations (dashed orange arrows) and recombination (green crosses). The height of the crosses reflects which sequences undergo recombination. LEVEL 2: the tree is expanded by adding two copies of each of the 3 sequences of level 1 to the MSA matrix, which now contains 9 rows and 3 different sequences derived from a single ancestral protein. LEVEL 3: 4 copies of each of the 9 sequences of level 2 are added to the MSA matrix, which now contains 45 rows and 9 different sequences (3 for each of the original 3 branches of the tree) derived from a single ancestral protein. Only the 3rd branch of the tree is shown for level 3. Thin blue arrows highlight steps that can be repeated as desired in both level 2 and 3. (TIF) [file pone.0047108.s011.tif]
