# Supplementary material for: Accurate Simulation and Detection of Coevolution Signals in Multiple Sequence Alignments
Source: PLoS One. 2012 Oct 16;7(10):e47108. doi: 10.1371/journal.pone.0047108 (PMC3473043; doi:10.1371/journal.pone.0047108)
Supplement: MSA S1 — MSA of KDO8PS. (DOCX) [file pone.0047108.s012.docx]

>UniRef100_P61656

KRDFLNGTKIGGDEPFFLISGPCVMENRDLLDRVCAEMIEVCGELKIPYIFKSSFDKANRSSVNSYRGPG

LAEGIKNLEYIKNKYNVPVLTDIHETSQISPLKDVIDVYQIPAFLCRQTDLISQSAQTGKWVNVKKGQFL

APADTRHIAVKMNESGNNKLLVTERGTSFGYGNLIFDGRAIPIIHGF--DIPLVFDATHSAQLPGAAGNS

TGGQREFIPSILRSAVSFGIEGIFMEVHPDPPNALSDATTQYPLSQIKSLLKEMIGLDRYIKKEILISRS

>Q050X1_LEPBL

---------IGGDEPFFLIAGPCVMENRDLLDRVCAEMIEICDELKISYVFKSSFDKANRSSVNSYRGPG

LTEGIQNLEYIKNKYNVPVLTDIHETHQIAPLKDAIDIYQIPAFLCRQTDLIAESAKTGRWVNVKKGQFL

APSDTRHIAVKMKESGNDKLLVTERGTSFGYGNLIFDGRAIPIIHGF--DIPLIFDATHSAQLPGAAENS

TGGQREFIPSILRSAVSLGIEGVFMEVHPDPQKALSDATTQYPLSRIKDLLKEMIGLDRYVKKE------

>Q8D2L1_WIGBR/13-279

------NIKISNSLPMVVFGGLNVLEKYDITMKVCEHFVKITNKLNIPFVFKASFDKANRSSINSYRGPG

LKNGIKIFSEIKKKFNVKIMTDIHDSTQADHVSEVVDIIQIPAFLARQTDLIASAANTGKVLNIKKPQFL

SPDQIIYIIEKIKSLGNKKIIICERGTMFGYNNLIVDILGLDVMKKISGGYPIILDVTHSLQNRKFLSPV

SSGNNDQVNSLSRAGISVGIAGIFIESHTNPEQAKCDGPCAIPLKKLELTLNQAKTIDDLIKS-------

>Q492V6_BLOPB/10-276

------DIKVSNNLPFVLFGGMNVLESRDITMKVCEHYAKITHKLNIPHIFKASFDKANRSSIDSYRGPG

LEEGLRLFQELQEIFGVKLMTDVHEIYQVKTVSEVVDVLQIPAFLARQTDLIASIAQTGMPINIKKPQYM

SPTQIIHIVKKCRSFSNKNIILCERGTVFGYDNLVVDMLGFNVMNHVSKGCPIIVDVTHALQKRNPLSPI

SGGRNRQIYDMARASTAVGIAGLFLEAHPNPHRAKCDGSSALPLNQLENFLTQIKSIDELVKS-------

>Q7VR72_BLOFL/10-277

------NIKISNKLPFVLFGGMNVLEKYDTIMQVCEYYVSITQKLNIPYIFKASFDKANRTSIHSYRGPG

LESGLRLFQILKKKFGVKLMTDVHEINQAQIISEVVDVIQLPAFLARQTDLIKAIAQTGSVVNIKKPQYM

SPEQVQYIVNKFHFFKNNKIILCERGTMFGYNDLIVDILGLHKMQHVSKGCPIIVDVTHALQTRQPYSAV

EGKNRLQIFDLAKASIAVGIAGLFIEAHPDPDNAKCDGPIALPLYQLPCFLNKIKLLDTLVKS-------

>A3S6J5_PROMA/4-246

TSVKVAKIEIGNDRPLVLIGGVNVIESRDMAMRAAEVFVREATRLGLPLVFKASFDKANRSSIDSFRGPG

LDEGLKIFEEIKAAFEVPVISDVHEIHQAKPAADVLDILQIPAFLARQTDLVRAIAETGKPINVKKPQFL

SPQQVPNIVDKIKSCGNDQVMICERGTSFGYDNLVVDILGFGVMKKENDDIPIVFDVTHSLQTRAPGSKA

SGGRREQIYELARAGVAAGIAALFLEAHEDPD--------------------------------------

>A6DQH2_9BACT/5-266

-----------NDKPFTLFGGLNVLEDLDSTLFACEKYVKVTNKLSIPYVFKASFDKANRSSVHSYRGVG

LDKGMRIFEEIKKEFGVKIITDVHEIYQVEQVAEVVDILQLPAFLARQTDLVVAISKAKKPINIKKPQFM

SPSQVRYIVNKCKEAGNDNVLLCERGTCFGYDNLVVDMLGFSQMKIATNNAPIIFDVTHSLQQRDPLSDA

SGGRRAQVLELAKAGISTRIAGLFLESHPDPDKAKCDGPSALPLSLLEAFLQQIKDLDDLIKS-------

>A0GD65_9BURK/13-279

------AVTVSNRLPFVLFGGINVLEDLDSTLYACRHYVETTAKLNIPFVFKASFDKANRSSIHSYRGVG

IDEGLSIFEAVKREFGVPVITDVHEVEQAARVAEVVDVLQVPAFLARQTDLVVAIAKTGRVINIKKPQFM

SPTQVAHIVGKCREVGNDNVILCERGSSFGYDNLVVDMLGFRQMIEASDGAPAIFDVTHSLQYRDPGGAA

SGGRRRQVVELARAGLAVGLSGLFLEAHPDPDHARCDGPSALPLAVLEPFLQQMKDLDDLIKS-------

>Q2SYU2_BURTA

---------AGNDRPFVLFGGINVLESLDFTLEVCSEYAAVTRKLGIPFVFKASFDKANRSSIHSYRGVG

LDEGLKILAEVKARFGVPVITDVHEVEQAAPVAEVADVLQVPAFLARQTDLVVAIAKAAKPVNVKKPQFM

SPTQVKHVVSKCLEAGNDRVMLCERGSSFGYDNLVVDMLGFRQMAEATGGCPVIFDVTHSLQCRDPLGEA

SGGRRRQVLDLARAGVAVGIAGLFVEAHPDPERARCDGPSALPLPLLEGFLSQMKAIDDLVKRM------

>A4MEK2_BURPS/2-274

NVAISPGVTAGNSLPFVLFGGINVLESLDFTLDVCGEYAAVTRKLGIPFVFKASFDKANRSSIHSYRGVG

LDEGLKIFAEVKARFGVPVITDVHEVEQAAPVAEIADVLQVPAFLARQTDLVVAIAKAGKPVNVKKPQFM

SPTQLKHVVSKCGEVGNDRVMLCERGSSFGYDNLVVDMLGFRQMAETTSGCPVIFDVTHSLQCRDPLGDA

SGGRRRQVLDLARAGIAVGIAGLFLEAHPDPDRARCDGPSALPLHQLEGFLSQMKAIDDLVKR-------

>A3ND03_BURP6

---------AGNSLPFVLFGGINVLESLDFTLDVCGEYVAVTRKLGIPFVFKASFDKANRSSIHSYRGVG

LDEGLKIFAEVKARFGVPVITDVHEAEQAAPVAEIADVLQVPAFLARQTDLVVAIAKAGKPVNVKKPQFM

SPTQLKHVVSKCGEVGNDRVMLCERGSSFGYDNLVVDMLGFRQMAETTGGCPVIFDVTHSLQWRDPLGDA

SGGRRRQVLDLARAGIAVGIAGLFLEAHPDPDRARCDGPSALPLHQLEGFLSQMKAIDDLVKRM------

>Q3JP68_BURP1/2-274

NVAISPGVTAGNSLPFVLFGGINVLESLDFTLDVCGEYVAVTRKLGIPFVFKASFDKANRSSIHSYRGVG

LDEGLKIFAEVKARFGVPVITDVHEAEQAAPVAEIADVLQVPAFLARQTDLVVAIAKAGKPVNVKKPQFM

SPTQLKHVVSKCGEVGNDRVMLCERGSSFGYDNLVVDMLGFRQMAETTGGCPVIFDVTHSLQCRDPLGDA

SGGRRRQVLDLARAGIAVGIAGLFLEAHPDPDRARCDGPSALPLHQLEGLLSQMKAIDDLVKR-------

>A3NYR7_BURP0/2-274

NVAISPGVTAGNSLPFVLFGGINVLESLDFTLDVCGEYVAVTRKLGIPFVFKASFDKANRSSIHSYRGVG

LDEGLKIFAEVKARFGVPVITDVHEVEQAAPVAEIADVLQVPAFLARQTDLVVAIAKAGKPVNVKKPQFM

SPTQLKHVVSKCGEVGNDRVMLCERGSSFGYDNLVVDMLGFRQMAETTGGCPVIFDVTHSLQCRDPLGDA

SGGRRRQVLDLARAGIAVGIAGLFLEAHPDPDRARCDGPSALPLHQLEGFLSQMKAIDDLVKR-------

>A8EHV0_BURPS/2-274

NVAISPGVTAGNSLPFVLFGGINVLESLDFTLDVCGEYVAVTRKLGIPFVFKASFDKANRSSIHSYRGVG

LDEGLKIFAEVKARFGVPVITDVHEAEQAAPVAEIADVLQVPAFLARQTDLVVAIAKAGKPVNVKKPQFM

SPTQLKHVVSKCGEVGNDRVMLCERGSSFGYDNLVVDMLGFRQMAETTGGCPVIFDVTHSLQCRDPLGDA

SGGRRRQVLDLARAGIAVGIAGLFLEAHPDPDRARCDGPSALPLHQLEGFLSQMKAIDDLVKR-------

>Q39M80_BURS3/37-309

NVSITPDVTVGNSLPFVLFGGLNVLESVEFTLYVCSRYVEVTRRLGIPLVFKASFDKANRSSIHSYRGVG

FDEGLRIFEEIKSRFGVPVITDVHEVWQAEPASHVADVLQVPAFLARQTDLVVAIARTGNAVNIKKPQFM

SPTQIEHVVSKCREAGNDKVILCERGASFGYDNLVVDMLGFRQMRDATGDCPVIFDVTHSLQMRDPHGAA

SGGRRRQVLDLARAGMAVGLAGLFLEAHPEPDQARCDGPSALPLAQLDAFLQQVKAVDDLVKR-------

>A0TEM3_9BURK/2-274

NVTITPDVTVGNDLPFALFGGLNVLESLDFTLDVCNTFVEVTRRLGIPLVFKASFDKANRSSIHSYRGVG

FDEGLRIFDEVKRRFGMPVITDVHEVWQAEPVSKVVDVLQVPAFLARQTDLVVAIAQTGNAVNIKKPQFM

SPTQIEHVVSKCREAGNDKVILCERGSSFGYDNLVVDMLGFGQMRDATGDCPVIFDVTHSLQMRDPHGAA

SGGRRRQVLDLARAGMAVGLAGLFLEAHPDPDRARCDGPSALPLALLDPFLQQVKAVDDLVKQ-------

>Q0B297_BURCM/2-274

NVTIAPDVTVGNDSPFVLFGGLNVLEGLDFTLDVCNTFVEVTRRLGIPLVFKASFDKANRSSIHSYRGVG

FDEGLRIFDEVKRRFGVPVITDVHEVWQAEPVSQVVDVLQVPAFLARQTDLVVAIAQTGNAVNIKKPQFM

SPTQIEHVVSKCREAGNDKVILCERGSSFGYDNLVVDMLGFRQMRDATGDCPVIFDVTHSLQMRDPHGAA

SGGRRRQVLDLARAGMAVGLAGLFLEAHPDPDRARCDGPSALPLALLDPFLQQVKAVDDLVKR-------

>Q1QZX8_CHRSD/9-276

---EFAGLRAGNSLPLALFGGMNVLESREMALEVAEAYVEVTGRLGMPYVFKSSFDKANRSSIHSYRGPG

LEKGLEWLAEIKERFNVPVITDVHEPWQAKPVAEVADVIQLPAFLARQTDLVVAMAETGAAINIKKPQFI

APHEMRHIIRKCEEVGNSRLILCERGSSFGYNNLVVDMLGFGEMKQT--GYPVFFDVTHALQRPGGRADS

ADGRRAQVAELARAGVAVGLAGVFLEAHPDPDNAKCDGPCALPLNRLEGFLSQLKAIDDLVKG-------

>Q6Y840_MORCA/9-283

SNITVSGIEIGNDKPFVLFGGMNVLESRELAFEIAEQYIDICQRLNIGYVFKASFDKANRSSLHSFRGPS

LENGLTWLNDIKETYNVPIITDVHEPYQAAPVAKVADILQLPAFLSRQTDLVSAIARTGKIINVKKAQFL

APHEMRHIINKCLEAGNDQVILCERGSAFGYNNLVVDMLGFDTMKSM--NVPVFFDVTHALQEPGARGDS

AGGRRHQITTLARAGMATGLAGLFLEAHPDPDKAKCDGPSALRLNQLEPFLIQIKQLDDLVKG-------

>UniRef100_Q6FAT8

EIVRLGDIQMANHLPFVLFGGMNVLESKDLAFEIAETYVDICKRLEIPYVFKASFDKANRSSLHSFRGPG

LEKGIEWLGEIKKHFNVPIITDVHEPYQAAPVAEVADIIQLPAFLSRQTDLVEAMAKTQAIINIKKAQFL

SPREMNHILNKCLEAGNDKLILCERGTSFGYNNLVVDMLGFDTMKEM--NVPVFFDVTHALQTPGGRADS

AGGRRAQITTLARAGMATGLAGLFLEAHPDPEKAKCDGPCALRLSQLEPFLAQLKELDALVKGFKKLDTH

>UniRef100_B0VQI5

EVVRLGDIQMANHLPFVLFGGMNVLESKDLAFEIAETYIDICKRLDIPYVFKASFDKANRSSLHSFRGPG

LEKGIEWLGDIKKHFNVPIITDVHEPYQAAPVAEVADIIQLPAFLSRQTDLVEAMAKTQAIINIKKAQFL

APHEMRHILHKCLEVGNDKLILCERGSAFGYNNLVVDMLGFDIMKEM--NVPVFFDVTHALQTPGGRSDS

AGGRRAQITTLARAGMATGLAGLFLESHPDPDKAKCDGPSALRLSQLEPFLAQLKELDTLVKGFKKLDTH

>UniRef100_B7H226

EVVRLGDIQMANHLPFVLFGGMNVLESKDLAFEIAETYIDICKRLDIPYVFKASFDKANRSSLHSFRGPG

LEKGIEWLGDIKKHFNVPIITDVHEPYQAAPVAEVADIIQLPAFLSRQTDLVEAMAKTQAIINIKKAQFL

APHEMRHILHKCLEAGNDKLILCERGSAFGYNNLVVDMLGFDIMKEM--NVPVFFDVTHALQTPGGRSDS

AGGRRAQITTLARAGMATGLAGLFLESHPDPDKAKCDGPSALRLSQLEPFLAQLKELDTLVKGFKKLDTH

>A5WG17_PSYWF

---------IGNDQPFVLFGGMNVLESKELAFEIAEGYIDICNRLGIGYVFKASFDKANRSSLNSYRGPG

LEKGVDWLSQIKEKYNVPIITDVHEPYQAEPVAEVADVIQLPAFLSRQTDLVQAMAKTGAIINIKKAQFL

APHEMRHIINKCLEAGNDKIILCERGTAFGYNNLVVDMLGFDTMKQM--DVPVFFDVTHSLQQPGARSDS

AGGRREQITTLARAGMATGLAGLFLEAHPEPEVAKCDGPCALRMSQLEAFLSQLKQIDDLVKGF------

>Q1Q9K2_PSYCK

---------IGNDQPFVLFGGMNVLESKEMAFEIAEQYVDICQRLGIGYVFKASFDKANRSSLTSYRGPG

LEKGIDWLGQIKEKFQVPIITDVHEPYQAAPVAEVADIIQLPAFLSRQTDLVHAMAKTDAIINIKKAQFL

APHEMRHIVNKCLEGGNDKLILCERGSAFGYNNLVVDMLGFDTMKQM--EIPVFFDVTHSLQQPGGRSDS

AGGRREQITTLARAGMATGIAGLFLEAHPNPEVAKCDGPCALRMSQLEPFLRQLKQLDDLVKGF------

>Q4FR70_PSYAR

---------IGNDQPFVLFGGMNVLESKEMAFEIAEQYVDICQRLGIGYVFKASFDKANRSSLTSYRGPG

LEKGIDWLGQIKEKFQVPIITDVHEPYQAAPVAEVADIIQLPAFLSRQTDLVHAMAKTDAIINIKKAQFL

APHEMRHIVNKCLEGGNDKLILCERGSAFGYNNLVVDMLGFDTMKQM--EIPVFFDVTHSLQQPGARSDS

AGGRREQITTLARAGMATGLAGLFLEAHPNPETAKCDGPCALRMSQLEPFLRQLKQLDDLVKGF------

>UniRef100_A1SV93

KIIKVGNIDVANDRPFVLFGGMNVLESRDMAMRVVETYVEATTALNIPYVFKASFDKANRSSIHSYRGPG

LEEGLRIFEEIKNTFNVPIITDVHEPSQAAPVAEVVDVIQLPAFLSRQTDLVKAMAETNAVINVKKAQFL

APHEMKHIITKFNEAGNDNIILCERGSCFGYNNLVVDMLGFSTMKET--GYPVIFDVTHSLQKPGARSDS

ADGRRSQVVQLGLAGMSQGIAGLFLESHPDPANAKCDGPCALPLHQLKAFLSQMKQMDELAKSFDIIDTE

>Q1ZGE9_9GAMM/11-275

------NIDVANDLPFVLFGGMNVLESRDLAMRMVEKYVEVTSALNIPYVFKASFDKANRSSINSYRGPG

LEEGLRIFEEIKKTFNVPIITDVHEPHQAAAVAEVVDVIQLPAFLSRQTDLVQAMASTNAVINVKKAQFL

APHEMAHIITKFKEAGNDKIILCERGSCFGYNNLVVDMLGFSTMKET--GYPVIFDVTHSLQKPGARSDS

ADGRRSQVVQLGLAGMSQGIAGLFLESHPDPSNAKCDGPCALALDKLKPFLTQMKQMDELAKS-------

>A3WLY1_9GAMM/8-271

------NITFDNQRPFVLFGGLNVLESQDLALRTAEHFMKVTEALGIPYVFKASFDKANRSSIHSFRGPG

LEEGLKIFEAVKQAFNVPIITDVHEPYQASPVAEVADIIQLPAFLARQTDLVMAMAKTGAVVNVKKPQFL

APHEMKHIIKKFNEAGNERILLCERGSCFGYNNLIVDMLGMDDMKSM---APVVFDATHALQRPGGRLDS

ADGRRAQAAELARSGMALGLAGLFIEAHPNPDEAKCDGPCALPLAKLEPYLQQMKAVDDLVKS-------

>Q5QUZ7_IDILO

---------IANEKPFVLFGGMNVLESRDLALRIAEHYVEVTSRLGIPYVFKASFDKANRSSIHSFRGPG

LEEGLRIFEEVKQQFNVPLITDVHEPHQAAPVAEVVDVLQLPAFLARQTDLVVALAETGSMVNIKKPQFL

APQEMKHIIKKFGEAGNEKLMLCERGSSFGYNNLIVDMLGMDEMKPM---APVIFDATHALQRPGGRADS

ADGRRAQAAQLARSGMSLGIAGLFIEAHPNPDEAKCDGPCALPLHALEPYLQQMKAIDDLVKSF------

>KDSA_COLP3

---------VANEQPFVLFGGMNVLESRDLAMKIAEHYVEVTQKLGIPYVFKASFDKANRSSVNSYRGPG

LDEGLKIFEEIKSTFNVPIITDVHESYQAQPVSEVVDVIQLPAFLARQTDLVVAMAKTGAVINVKKPQFL

AAHEMKHIITKFGEAGNENIILCERGSCYGYNNLVVDMLAMDEMKNY---APVIFDATHALQKPGGRSDS

ADGRRAQAAQLARSGMAIGIAGLFIEAHPDPSAAKCDGPCALPLDKLEPYLAQMKALDDLVKGF------

>A4B6G2_ALTMA/12-275

------NVEVANHLPFVLFGGMNVLESRDLAMRIAEHYVEVTQKLNIPYVFKASFDKANRSSVTSYRGPG

MEEGLRIFEEIKSTFNVPLITDVHEPHQAAPVADVVDVIQLPAFLARQTDLVVAMAKTGAVINVKKPQFL

APHEMRHIIGKLGEAGNDNVILCERGSCFGYNNLVVDMLGMDAMKEY---APVIFDATHALQMPGGRATS

ADGRRAQAAQLARSGMALGLAGLFIEAHPNPDEALCDGPCALPLAKLEPYLQQMKALDELVKG-------

>Q1N0A1_9GAMM/5-272

--VSVASLDVANNIPFTLFGGMNVLESRDLAMSICEEYVKVTEKLGIPYVFKASFDKANRSSINSYRGPG

MDEGLKIFQEIKDTFGVPIITDVHEPYQAAPVAEVVDVIQLPAFLARQTDLVVAMAKTGAAINVKKPQFL

APHEMRHIIKKFEEAGNQRVMLCERGSSFGYNNLVVDMLGMDEMKTM---APVIFDATHALQRPGGRSDS

ADGRRAQAFELARSGMALGIAGLFLEAHPNPDEAKCDGPCALPLDKLEPYLEQMKAVDDLVKS-------

>UniRef100_A1S8Q7

KTIQLGDIAIANDKPFVLFGGMNVLESRDLAMSIAEKYLEVTQKLGIPYVFKASFDKANRSSINSYRGPG

MEEGLKIFQEIKDTFNVPLITDVHEPHQCAPVAEVVDIIQLPAFLARQTDLVIAMARTGAIINVKKPQFL

APHEMRHIVKKFNEAGNDEIILCERGSCFGYNNLVVDMLGMDEMKQS--GYPVIFDATHALQRPGGREDS

AGGRRAQATELARSGMALGLAGLFIEAHPDPDNAKCDGPCALPLHQLEAYLSQMKAVDDLVKSFPALDTS

>KDSA_SHEFN/11-275

------NVQVANDKPFVLFGGMNVLESRDLAMSIAEHYVEVTQKLGIPYVFKASFDKANRSSVNSYRGPG

MEEGLRIFEEIKSTFNVPMITDVHEIHQCAPVAEVVDIIQLPAFLARQTDLVVAMAKTGAVINVKKPQFL

APHEMRHIITKFNEAGNDNIILCERGSSFGYNNLVVDMLGMDEMKQT--GYPVIFDATHALQRPGGRADS

AGGRRAQATELARSGMALGLAGLFIEAHPDPDNAKCDGPCALPLHLLEKYLTQMKAVDELVKS-------

>KDSA_SHEDO/5-278

LATEAGAIEIANDKPFVLFGGMNVLESRDLAMSIAEHYVEVTQKLGIPYVFKASFDKANRSSVNSYRGPG

MEEGLKIFEEIKRTFNVPLITDVHEVHQCAPVAEVVDIIQLPAFLARQTDLVVAMAKTGAIINVKKPQFL

APHEMRHIVTKFNEAGNDKIILCERGSSFGYNNLVVDMLGMDDMKKT--GYPVIFDATHALQRPGGRADS

AGGRRAQATELARSGMALGIAGLFIEAHPDPDNAKCDGPCALPLHQLENYLKQMKAVDDLVKS-------

>UniRef100_A3QH22

KTIKLGSIEIANDKPFVLFGGMNVLESRDLAMQIAETYAEVTQKLGIPYVFKASFDKANRSSVNSYRGPG

MEEGLKIFQEIKDTFNLPLITDVHEPYQCQPVAEVVDIIQLPAFLARQTDLVVAMAKTGAIINVKKPQFL

APHEMRHIITKFNEAGNDEIILCERGSCFGYNNLVVDMLGMDEMKQS--GYPVIFDATHALQRPGGRADS

AGGRRAQATELARSGMALGLAGLFIEAHPDPDNAKCDGPCALPLHQLENYLTQMKAIDDLVKSFAPIDTS

>UniRef100_A4Y3K6

KIINLGSIEIANDKPFVLFGGMNVLESRDLAMSIAETYAEVTQKLGIPYVFKASFDKANRSSVNSYRGPG

MEEGLKIFEEIKKTFNLPLITDVHETYQCAPVAEVVDIIQLPAFLARQTDLVVAMAKTGAIINVKKPQFL

APHEMRHIITKFNEAGNDEIILCERGSCFGYNNLVVDMLGMDEMKQS--GYPVIFDATHALQRPGGRSDS

AGGRRAQATELARSGMALGLAGLFIEAHPDPDNAKCDGPCALPLHQLENYLKQMKAIDDLVKSFEPIDTS

>UniRef100_A0KT94

KIINLGSIEIANDKPFVLFGGMNVLESRDLAMSIAETYAEVTQKLGIPYVFKASFDKANRSSVNSYRGPG

MEEGLKIFEEIKKTFNLPLITDVHETYQCAPVAEVVDIIQLPAFLARQTDLVVAMAKTGAIINVKKPQFL

APHEMRHIITKFNEAGNDEIILCERGSCFGYNNLVVDMLGMDEMKQS--GYPVIFDATHALQRPGGRADS

AGGRRAQATELARSGMALGLAGLFIEAHPDPDNAKCDGPCALPLHQLENYLKQMKAIDDLVKSFDPIDTS

>UniRef100_Q8EAR9

KIIKLGSIEIANDKPFVLFGGMNVLESRDLAMSIAETYAEVTQKLGIPYVFKASFDKANRSSVNSYRGPG

MEEGLKIFEEIKKTFNLPLITDVHETYQCAPVAEVVDIIQLPAFLARQTDLVVAMAKTGAIINVKKPQFL

APHEMRHIITKFNEAGNDEIILCERGSCFGYNNLVVDMLGMDEMKQS--GYPVIFDATHALQRPGGRADS

AGGRRAQATELARSGMALGLAGLFIEAHPDPDNAKCDGPCALPLHQLENYLKQMKAIDDLVKSFEPIDTS

>KDSA_SHESM/10-274

------SIEIANDKPFVLFGGMNVLESRDLAMSIAETYAEVTQKLGIPYVFKASFDKANRSSVNSYRGPG

MEEGLKIFEEIKKTFNLPLITDVHEPYQCAPVAEVVDIIQLPAFLARQTDLVVAMAKTGAIINVKKPQFL

APHEMRHIITKFNEAGNDEIILCERGSCFGYNNLVVDMLGMDEMKQS--GYPVIFDATHALQRPGGRADS

AGGRRAQATELARSGMALGLAGLFIEAHPDPDNAKCDGPCALPLHQLENYLKQMKAIDDLVKS-------

>UniRef100_A3D0G8

KIINLGSIEIANDKPFVLFGGMNVLESRDLAMSIAETYAEVTQKLGIPYVFKASFDKANRSSINSYRGPG

MEEGLKIFEEIKKTFNLPLITDVHEVHQCAPVAEVVDIIQLPAFLARQTDLVVAMAKTGAIINVKKPQFL

APHEMRHIITKFNEAGNDEIILCERGSSFGYNNLVVDMLGMDEMKQS--GYPVIFDATHALQRPGGRADS

AGGRRAQATELARSGMALGLAGLFIEAHPDPDNAKCDGPCALPLHQLENYLKQMKAIDDLVKSFEPIDTS

>UniRef100_A6WSE4

KIINLGSIEIANDKPFVLFGGMNVLESRDLAMSIAETYAEVTQKLGIPYVFKASFDKANRSSINSYRGPG

MEEGLKIFEEIKKTFNLPLITDVHEVHQCAPVAEVVDIIQLPAFLARQTDLVVAMAKTGTIINVKKPQFL

APHEMRHIITKFNEAGNDEIILCERGSSFGYNNLVVDMLGMDEMKQS--GYPVIFDATHALQRPGGRADS

AGGRRAQAAELARSGMALGLAGLFIEAHPDPDNAKCDGPCALPLHQLENYLKQMKAIDDLVKSFDPIDTS

>UniRef100_A8FYY1

KIIGLGSIEIANDKPFVLFGGMNVLESRDLAMKIAETYAEVTQKLGIPYVFKASFDKANRSSVNSYRGPG

MEEGLKIFEEIKSTFNLPLITDVHEVHQCAPVAEVVDIIQLPAFLARQTDLVVAMAKTDAIINVKKPQFL

APHEMRHIITKFNEAGNDKIILCERGSSFGYNNLVVDMLGMDEMKQS--GYPVIFDATHALQRPGGRSDS

AGGRRAQATELARSGMALGLAGLFIEAHPDPDNAKCDGPCALPLHQLEAYLTQMKAVDDLVKSFDAIDTS

>A0JA01_9GAMM/10-274

------SIEIANDKPFVLFGGMNVLESRDLAMKIAETYAETTQKLGIPYVFKASFDKANRSSVNSYRGPG

MEEGLKIFEEIKSTFNLPLITDVHEIHQCAPVAEVVDIIQLPAFLARQTDLVVAMAKTGAIINVKKPQFL

APHEMRHIITKFNEAGNDEIILCERGSSFGYNNLVVDMLGMDEMKQT--GYPVIFDATHALQRPGGRADS

AGGRRAQATELARSGMALGLAGLFIEAHPDPDNAKCDGPCALPLHQLEAYLTQMKAVDDLVKS-------

>UniRef100_B8CKA0

KTIDLGSIEIANDKPFVLFGGMNVLESRDLAMQIAETYAEVTQKLGIPYVFKASFDKANRSSVNSYRGPG

MEEGLKIFEEIKSTFNLPLITDVHEVHQCAPVAEVVDIIQLPAFLARQTDLVVAMAKTGAIINVKKPQFL

APHEMRHIISKFNEAGNDEIILCERGSSFGYNNLVVDMLGMDEMKQS--GYPVIFDATHALQKPGGRSDS

AGGRRAQATELARSGMALGLAGLFIEAHPDPDNAKCDGPCALPLHQLENYLTQMKAVDDLVKSFAPIDTS

>UniRef100_B0TR22

KIISLGSIEIANDKPFVLFGGMNVLESRDLAMQIAETYAEVTQKLGIPYVFKASFDKANRSSVNSYRGPG

MEEGLKIFEEIKSTFNLPLITDVHEVQQCAPVAEVVDVIQLPAFLARQTDLVVAMAKTGAIINVKKPQFL

APHEMRHIITKFNEAGNDEIMLCERGSSFGYNNLVVDMLGMDEMKQT--GYPVIFDATHALQRPGGRSDS

AGGRRAQATELARSGMALGLAGLFIEAHPDPDNAKCDGPCALPLHQLENYLTQMKAVDDLVKSFEPIDTS

>A8H7A0_9GAMM/3-274

KIISLGSIEIANDKPFVLFGGMNVLESRDLAMQIAETYAEVTQKLGIPYVFKASFDKANRSSVNSYRGPG

MEEGLKIFEEIKSTFNLPLITDVHEVHQCAPVAEVVDVIQLPAFLARQTDLVVAMAKTGAIINVKKPQFL

APHEMRHIITKFNEAGNDEIMLCERGSSFGYNNLVVDMLGMDEMKQT--GYPVIFDATHALQRPGGRSDS

AGGRRAQATELARSGMALGLAGLFIEAHPDPDNAKCDGPCALPLHQLENYLTQMKAIDDLVKS-------

>Q15TR5_PSEA6

---------VANDKPFVLFGGMNVLESRDMAMRIAEHYKEITSKLGIPYVFKASFDKANRSSVNSYRGPG

MDEGLEIFAEIKATFDVPLITDVHEPAQAAPVAEVVDVIQLPAFLARQTDLVVAMAKTNAIINVKKPQFL

APHEMRHIITKFMEAGNDKIILCERGSSFGYNNLVVDMLGMDEMKQY---APVIFDATHALQKPGGRTDS

ADGRRAQAAQLARSGMALGLAGLFIEAHPNPNEAKCDGPCALSLDKLEPYLQQMLALDDLVKGF------

>A4CFL3_9GAMM/10-273

------SIEVANNKPFVLFGGMNVLESRDLAMRIAEYYVEVTTKLGIPYVFKASFDKANRSSINSYRGPG

LDEGLKIFQEIKQTFNVPLITDVHEPHQAAPVAEVVDVIQLPAFLARQTDLVVAMAKTGAVINVKKPQFL

APHEMRHIIQKFHEAGNDKVILCERGSSFGYNNLVVDMLGMDDMKQQ---APVIFDATHALQRPGGRTDS

ADGRRAQAAELARSGMALGLAGLFIEAHPNPNEAKCDGPCALQLDKLAGYLTQMKAVDDLIKS-------

>UniRef100_Q3IK90

QIIKINDIELANNKPFVLFGGINVLESRDLAMRVAEHYVEVTTKLNIPYVFKASFDKANRSSINSYRGPG

LDEGLKIFEEIKKTFNIPLITDVHEPHQAAPVAEVVDVIQLPAFLARQTDLVVAMAKTGAIINVKKPQFL

APHEMRHIITKFNEAGNNNVALCERGSSFGYNNLVVDMLGMDDMKVM---APVIFDATHALQRPGGRADS

ADGRRAQAAELARSGMALGIAGLFIEAHPNPNEAKCDGPCALALSKLEGYLTQMKAVDDLIKSFAPLDTS

>A0Y6B5_9GAMM/10-273

------DIELANNKPFVLFGGMNVLESRDLAMRIAEHYVEVTTKLNIPYVFKASFDKANRSSINSYRGPG

MEEGLKIFEEIKNTFNIPLITDVHEPHQAAPVAEVVDVIQLPAFLARQTDLVVAMAKTGAIINVKKPQFL

APHEMRHIITKFNEAGNNNIALCERGSSFGYNNLVVDMLGMDDMKAM---APVIFDATHALQRPGGRADS

ADGRRAQAAELARSGMALGIAGLFIEAHPNPNEAKCDGPCALALSKLEGYLSQMKAVDDLVKS-------

>A3YC98_9GAMM/10-281

IIEINQDVKVANHLPFVLFSGVNVLESRDLAMSVAEEHVKVCQALNIPYVFKGSFDKANRSSINSFRGPG

MEEGLRILEDVKASFNVPVITDVHEVAQCAPVAEVADVIQLPAFLSRQTDLVVAMAQTDAVINIKKAQFL

APHEMQHILTKCEEAGNDRLMLCERGTMMGYNNLVVDMLGFGLMKKF--DYPVMFDVTHALQKPGGRAES

ADGRRAQVAELARSGMALGLSSLFLETHPNPNQAKCDGPCALPLDKLKPFLHQLKQIDELVKG-------

>A6VUC6_9GAMM/16-280

------KIEVANHLPFVLFSGVNVLESRDLAMRVAEEHVKVTEALGIPYVFKGSFDKANRSSINSFRGPG

MEEGLKILQEIKDTFDVPVITDVHEAYQCAPVAEVADVIQLPAFLSRQTDLVIAMAKTGAVINIKKAQFL

APHEMKHILTKCEEAGNNNLMLCERGTMMGYNNLVVDMLGFGEMKKF--GYPVMFDVTHSLQRPGGREDS

ADGRRAQVTELARAGMSLGLSSLFLETHPDPDSAKCDGPCALPLGKLAPFLKQMKQLDELVKT-------

>Q2BP40_9GAMM/4-274

KTVQVAGIEIANDKPMVLFGGMNVLESRDLALKIAEYYVEVTQKLGIPYVFKASFDKANRSSLTSFRGPG

LDEGLKILQEIKDTFKVPLITDVHEPDQAAPAAEVCDIIQLPAFLSRQTDLVQAMAQTDAVINIKKAQFL

APQEMKHILHKCEEAGNSDLILCERGSCYGYNNLIVDMLGFSIMKEF--GYPVMFDATHALQMPGGRTDS

AGGRRALAAQLSRAGLSQGIAGLFLEAHPDPEQAKCDGPCALPLHTLEAYLSQMKAVDDLVKS-------

>A4BE16_9GAMM/10-274

------NIECSNDKPFTLFGGINVLESRDLAMSAAEQYVRITEELDIPYVFKASFDKANRSSINSFRGPG

LEEGLKILEEVKNTFNVPVITDVHEPYQAQPAAEVADIIQLPAFLSRQTDLVMAMAKTGATINIKKAQFL

APHEMAHILTKFQEAGNDQLILCERGTSFGYNNLVVDMLGFGIMKET--GFPVIFDVTHALQRPGGRLDS

ADGRRAQVTDLARAGMSQKIAGLFLESHPDPSQAKCDGPSALPLDKLKAFLEQMKAVDTVVKE-------

>Q0VQD7_ALCBS

---------FANDKPMALFGGMNVLESRDLAMQVAEAYAEATSKLNMPWVFKASFDKANRSSLSSYRGPG

MEEGLKIFEEVKKTFNCPLITDVHEPGQAGPVAEVVDIIQLPAFLARQTDLVVAMAKTGAIINIKKPQFL

APHEMSHILHKCEDAGNDQLILCERGSSFGYNNLVVDMLGFGIMKQF--NYPVFFDVTHALQKPGGRADS

ADGRRAQVAELGRAGISQGIAGLFLEAHPDPDNAKCDGPCALRLDRLEPFLAQMQALDNTVKAL------

>A3JGV7_9ALTE/6-274

--VIVADIEIANHKPFVLFAGMNVLESRELAFEVAETYVEVCRKLGIPFVFKASFDKANRSSVNSFRGPG

LDKGLQILADIKARFGVPIISDVHEAAQAAPAAEVCDIIQLPAFLSRQTDLVVAMAKTGAVINIKKAQFL

APQEMRHIIAKFEEAGNDQIILCERGSSFGYNNLVVDMLGFGIMKSM--NVPVMFDVTHSLQMPGGRADS

AGGRRAQVTELALAGMSQGLAGLFLEAHPNPDEAMCDGPCSLRLSQLEPFLERVKAVDDLVKS-------

>A6F3D0_9ALTE/10-274

------GIEVANHKPFVLFGGMNVLESPEMAMEVAEAYVEATRKLGIPYVFKASFDKANRSSVHSFRGPG

LEKGLQILADIKSKFGVPIISDVHEPEQAAPAAEVCDIIQLPAFLSRQTDLVVAMAETGAVINIKKAQFL

APQEMKHIIAKCGEAGNDKVILCERGTSFGYNNLVVDMLGFGIMKSL--DVPVMFDVTHALQMPGGRADS

AGGRRAQVTDLALAGMSQGLAGLFLEAHPDPDQAKCDGPCALRLSQLEPFLARVKAVDDLVKS-------

>UniRef100_A1TZ47

SNLNVSGIEIANDRPFVLFGGMNVLESRELAFEVAEKYVDVCTRLGIPYVFKASFDKANRSSVNSFRGPG

LEKGLQILADIKSKFGVPIISDVHEPGQAAPAAEVCDIIQLPAFLSRQTDLVVAMAKTGAVINIKKAQFL

APQEMKHIITKCEEAGNDQIILCERGTSFGYNNLVVDMLGFGIMKQM--NVPVFFDVTHALQMPGGRADS

AGGRRAQVTDLALAGMSQGLAGLFLEAHPDPDKARCDGPCALRLSQLEPFLERVKAVDDLVKSFKPIDTA

>UniRef100_Q2SKX1

RTVKVGGIDIANDRPFVLFGGMNVLESRDLALQVAESYVKVTQKLGIPYVFKASFDKANRSSINSYRGPG

LDEGLRIFEEIKRTFNVPVITDIHEPHQAQPVAEVADIIQLPAFLSRQTDLVAAMARTGCPVNIKKAQFL

APQEMKHILSKCEEAGNDQLILCERGSSFGYNNLVVDMLGFGIMKQF--GYPVFFDVTHSLQMPGGLSSS

AGGRREQVVQLARSGMAVGLAGLFLEAHPDPDVAKCDGPCALRLSQLEPFLAQMKALDDMVKQFPNIDTA

>Q4IXV1_AZOVI/10-274

------DIEIANDRPMALFGGMNVLESRDLALRVCEEYVRVTGKLGIPYVFKASFDKANRSSITSFRGPG

LEEGMRIFEEVKKAFGVPVLTDVHEPWQAAPVAEVCDILQLPAFLSRQTDLVVAMAKTSAVINIKKAQFL

APQEMQHILRKCEEAGNDQLILCERGTSFGYNNLVVDMLGFGIMKQF--EYPVFFDVTHALQMPGGRADS

AGGRRAQVTDLAKAGISQGLAGLFLEAHPDPDNAKCDGPCALRLDKLEPFLAQLAQLDALVKK-------

>UniRef100_A6V1F2

KIVRVGDIQIGNDLPFVLFGGMNVLESRDLAMQVCEEYVRVTEKLGIPYVFKASFDKANRSSIHSFRGPG

LEEGMKIFEEIKKTFKVPVITDVHEPHQAQPVAEVCDIIQLPAFLSRQTDLVVAMARTNAVINIKKAQFL

APQEMKHILTKCEEAGNDRLILCERGSSFGYNNLVVDMLGFGIMKQF--EYPVFFDVTHALQMPGGRADS

AGGRRAQVTDLAKAGLSQKLAGLFLEAHPDPEHAKCDGPCALRLNKLEAFLSQLKQLDELIKSFPAIETA

>UniRef100_Q02RA8

KIVRVGDIQIGNDLPFVLFGGMNVLESRDLAMQVCEEYVRVTEKLGIPYVFKASFDKANRSSIHSFRGPG

LEEGMKIFEEIKKTFKVPVITDVHEPFQAQPVAEVCDIIQLPAFLSRQTDLVVAMARTNAVINIKKAQFL

APQEMKHILTKCEEAGNDRLILCERGSSFGYNNLVVDMLGFGIMKQF--EYPVFFDVTHALQMPGGRADS

AGGRRAQVTDLAKAGLSQKLAGLFLEAHPDPEHAKCDGPCALRLNKLEAFLSQLKQLDELIKSFPAIETA

>UniRef100_Q48F78

KTIRVGSIEIANDKPMVLFGGMNVLESRDMAMQVCEEYVRVTEKLGIPYVFKASFDKANRSSVNSYRGPG

LEEGMRIFEEIKRTFNVPLITDVHEPHQAAVVAEVCDIIQLPAFLSRQTDLVVAMAKTGAVINIKKAQFL

APQEMKHILTKCEEAGNDQLILCERGSSFGYNNLVVDMLGFGVMKQF--EYPILFDVTHALQMPGGRSDS

AGGRRAQVLDLAKAGISQNLAGLFLEAHPDPDNAKCDGPCALRLDKLEPFLAQLKSLDELVKSFPIVETA

>UniRef100_Q886M4

KIIRTGSIEIANDKPMVVFGGMNVLESRDMAMQVCEEYVRVTEKLGIPYVFKASFDKANRSSVNSYRGPG

LEEGMRIFEEIKRTFNVPLITDVHEPHQAAVVAEVCDIIQLPAFLSRQTDLVVAMAKTGAIINIKKAQFL

APQEMKHILTKCEEAGNDQLILCERGSSFGYNNLVVDMLGFGIMKQF--EYPVLFDVTHALQMPGGRSDS

AGGRRAQVLDLAKAGISQNLAGLFLEAHPDPDNAKCDGPCALRLDKLEPFLAQLKSLDELVKSFPIVETA

>UniRef100_Q4ZWQ9

KIIRVGSIEVANDKPMVLFGGMNVLESRDMAMQVCEEYVKVTEKLGIPYVFKASFDKANRSSVNSYRGPG

LEEGMRIFEEIKRTFNVPLITDVHEPHQAAVVAEVCDIIQLPAFLSRQTDLVVAMAKTGAIINIKKAQFL

APQEMKHILTKCEEAGNDQLILCERGSSFGYNNLVVDMLGFGIMKQF--EYPILFDVTHALQMPGGRSDS

AGGRRAQVLDLAKAGISQNLAGLFLEAHPDPDNAKCDGPCALRLDKLEPFLAQLKSLDELVKSFPIVETA

>UniRef100_Q4KHF7

KIIRVGNIEIANDKPMVLFGGMNVLESRDMAMQVCEEYVRVTEKLGIPYVFKASFDKANRSSVTSYRGPG

LEEGMRIFEDVKQAFGVPIITDVHEPAQAAVVAEVCDIIQLPAFLSRQTDLVVAMAKTGAVINIKKAQFL

APQEMKHILSKCEEAGNDQLILCERGSSFGYNNLVVDMLGFGIMKQF--EYPVFFDVTHALQMPGGRADS

AGGRRAQVTDLAKAGMSQSLAGLFLEAHPDPDNAKCDGPCALRLDKLEPFLAQLKALDELVKSFPTVETA

>KDSA_PSEPF

---------IANDKPMVLFGGMNVLESRDMAMQVCEEYVKVTEKLGIPYVFKASFDKANRSSVTSYRGPG

LEEGMRIFQDIKQAFGVPIITDVHEPDQAAVVAEVCDIIQLPAFLSRQTDLVVAMAKTNAVINIKKAQFL

APQEMKHILNKCVEAGNDQLILCERGSSFGYNNLVVDMLGFGIMKQF--EYPVFFDVTHSLQMPGGRSDS

AGGRRAQVTDLAKAGMSQSLAGLFLEAHPDPDNAKCDGPCALRLDKLEPFLAQLKALDELVKSF------

>UniRef100_Q1I645

KIIRVGNIEIANDKPFVLFGGMNVLESRDLALKVCEEYVRVTEKLGIPYVFKASFDKANRSSVNSYRGPG

MEEGLKIFEEIKRTFNVPVITDVHEPYQCEPVAQVCDIIQLPAFLSRQTDLVVAMAKTGAVINIKKAQFL

APHEMKHILAKCVEAGNDQLILCERGSSFGYNNLVVDMLGFGIMKQF--EYPVFFDVTHSLQTPGGRADS

AGGRRAQVTDLAKAGMSQGLAGLFLEAHPDPDNAKCDGPCALRLDKLEPFLAQLKQLDDLVKSFPTVETA

>A5W830_PSEP1

---------IANDKPFVLFGGMNVLESRDLAMKVCEEYVRVTEKLGIPYVFKASFDKANRSSVTSYRGPG

MEEGLKIFEEIKRTFNVPVITDVHEPYQAEPVAKVCDIIQLPAFLSRQTDLVVAMAKTGVVINIKKAQFL

APQEMKHILAKCEEAGNDQLILCERGSSFGYNNLVVDMLGFGIMKQF--EYPVFFDVTHALQMPGGRADS

AGGRRAQVTDLAKAGMSQGLAGLFLEAHPDPDNAKCDGPCALRLDKLEPFLVQLKQLDDLVKSF------

>B0KSB8_PSEPG

---------IANDKPFVLFGGMNVLESRDLAMKVCEEYVRVTEKLGIPYVFKASFDKANRSSVTSYRGPG

MEEGLKIFEEIKRTFNVPVITDVHEPYQAEPVAKVCDIIQLPAFLSRQTDLVVAMAKTGAVINIKKAQFL

APQEMKHILAKCEEAGNDQLILCERGSSFGYNNLVVDMLGFGIMKQF--EYPVFFDVTHALQMPGGRSDS

AGGRRAQVTDLAKAGMSQGLAGLFLEAHPDPDNAKCDGPCALRLDKLEPFLAQLKQLDDLVKSF------

>UniRef100_A4VJU0

KTIRVGNIEIANDKPFVLFGGINVLESRDLAMQACEEYVRVTEKLGIPYVFKASFDKANRSSITSFRGPG

LEEGMKIFEKVKKTFGVPVITDVHEPWQAQPVADVCDIIQLPAFLSRQTDLVVAMAKTGAVINIKKAQFL

APQEMKHILKKCEEAGNDQLILCERGSSFGYNNLVVDMLGFGIMKQF--EYPVFFDVTHALQMPGGRADS

AGGRRAQVTDLAKAGLSQGLAGLFLEAHPDPDNAKCDGPCALRLNKLEAFLTQLKQLDDLVKNFPPIETA

>UniRef100_A4XWS2

KIIKVRDIEIANDKPFVLFGGINVLESRDLAMQACEEYVRVTEKLGIPYVFKASFDKANRSSITSFRGPG

LEEGMKIFEEVKKTFGVPVITDVHEPHQAAAVAEVCDIIQLPAFLSRQTDLVVAMAKTGAVINIKKAQFL

APQEMKHILTKCEEAGNDQLILCERGSSFGYNNLVVDMLGFGIMKQF--EYPVFFDVTHALQMPGGRADS

AGGRRAQVTDLAKAGMSQGLAGLFLEAHPDPENAKCDGPCALRLNKLEPFLSQLKQLDDLVKSFPPIETA

>A0Z6I8_9GAMM/3-278

VIVSAGDIQFANHLPFVLIGGINVLESEQFAVDVAGHYQEVCRRLSIPLVFKASYDKANRSSVASYRGPG

LDLGLRQLGAVKSALGVAILTDVHAPEEAAAAAEVCDIIQLPAFLARQTDLIQAMANTGAVINIKKPQFL

SPEQMVHIVDKFRECGNGQLLLCERGSNFGYDNLVVDMLGFGVMQRVTGGLPLIFDVTHALQRRDPGQSA

SGGRRQQVLSLARSGMAIGLAGLFLEAHPEPDKALCDGPSALPLELLEPFLEQIKAVDTMAKA-------

>A2CCS7_PROM3/20-293

PSVSIDGIRVANDQPFVLIGGVNVLESLDFAINVAGHYVEVCQRLDIPFVFKASYDKANRSSIHSYRGPG

ITTGLNILRAVKETYKIPVLTDVHSPEEASEAAAICDVIQLPAFLARQTDLVRAMAETNAVINIKKPQFL

SPEQMKNITEKFRECGNTKLLLCERGSNFGYDNLVVDMLGFGVMKHICNDLPLIFDVTHALQCRDAGGAA

SGGRRAQVLELARAGMAVGLAGLFLEAHPNPDEAFCDGPSALPLAKLEPFLTQIKAIDDLVKS-------

>A4AAB7_9GAMM/9-275

------ELRIANDAPFVLLGGVNVLESREFALEVAQEYVSVCAELAVPLVFKASYDKANRSSIHSFRGPG

LSAGLEILAEVKSRFQVPVITDVHTPEEAAPAAEVCDIIQLPAFLARQTDLVRAMAETGAAINIKKPQFL

SPSQMANVVEKFRECGNERLMLCERGSNYGYDNLVVDMLGFGVMRQATAGVPQIFDVTHALQCRDPMGAA

SGGRRSQVTELARAGMAVGLAGLFLESHPDPDKALCDGPSALPLEKLKPFLEQVQAVDKLVKA-------

>KDSA_SYNPX

---------FANDRPFALLGGVNVLEDLDFALRCAGHYKQVCERLGIPLVFKASYDKANRSSIHSFRGPG

LEAGLQILQAVKDTLGIPVITDVHSPEEAKAAAKVADIIQLPAFLARQTDLVRAMAETGAVINIKKPQFL

SPEQIRNIVDKFRECGNEQLLICERGTNFGYDNLVVDMLGFGVMKRTCDDLPLIFDVTHALQCRDPGGAA

SGGRRSQVVDLAKAGMAVGLAGLFLEAHPDPNQARCDGPSALPLDQLEPFLTQVKAIDDLVKSQ------

>Q3AN81_SYNSC

---------FANDRPFALLGGVNVLEDLDFALRCSKHYKDVCERLNIPLVFKASYDKANRSSIHSFRGPG

LKHGLEILQAVKDTHGIPVITDVHSPEEAAAAAKVADIIQLPAFLARQTNLVRAMAETGAVINIKKPQFL

SPEQMRNIVDKFRECGNEQLLLCERGTNFGYDNLVVDMLGFGVMKRTCDDLPLIFDVTHALQCRDPGGAA

SGGRRTQVVDLARSGMAVGLAGLFLEAHPDPAKARCDGPSALPLDQLEPFLTQVKAIDDLVKGM------

>Q3ANB7_SYNSC

---------FANDRPFALLGGVNVLEDLDFALRCAGHYKNVCGRLNIPLVFKASYDKANRSSIHSFRGPG

LNEGLQILQAVKDTHGIPVITDVHSPEEAAAAAKVADIIQLPAFLARQTDLVRAMAETGAMINVKKPQFL

SPEQMRNIVDKFRECGNENLLLCERGTNFGYDNLVVDMLGFGVMKRTCDDLPLIFDVTHALQCRDPGGAA

SGGRRSQVVDLARSGMAVGLAGLFLEAHPDPARARCDGPSALPLDQLEPFLTQVKAIDDLVKAM------

>Q063B9_9SYNE/2-275

RQVQLGNITFANDQPFVLLGGVNVLEDLDFALNCAGHYKTVCERLGIPLVFKASYDKANRSSIHSFRGPG

LEKGLAILQAVKDTHGIPVITDVHTPEEAVAAAAVADIIQLPAFLARQTDLVTAMANTGSVINIKKPQFL

SPEQMANIVEKFKECGNDQLLLCERGSNFGYDNLVVDMLGFGVMKKTCQDIPLIFDVTHALQCRDPGGAA

SGGRRSQVVDLARAGIAVGLAGLFLEAHPDPDKARCDGPSALPLDLLEPFLSQLKALDQLVKT-------

>Q3B0E7_SYNS9/2-275

RQVQLGNITFANDRPFVLLGGVNVLEDLDFALHCASHYKTVCERLGIPLVFKASYDKANRSSIHSFRGPG

LEQGLSILEAVKNTHGIPVITDVHTPEGAVAAAAVADIIQLPAFLARQTDLVTAMANTGAVINIKKPQFL

SPEQMANIVEKFKECGNEQLLLCERGSNFGYDNLVVDMLGFGVMKQTCQDIPLIFDVTHALQCRDPGCAA

SGGRRSQVVDLARSGMAIGLAGLFLEAHPNPAQARCDGPSALPLNLLEPFLTQVKAIDDLVKG-------

>UniRef100_Q7V4M4

RSIALGTIRFANDAPFVLIGGINVLESREFALEVAGHYKTVCTKLGIPLVFKASFDKANRSSIHSYRGPG

LREGLEILQVVKDTHGIPVITDVHSPEEATPAAEVCDIIQLPAFLARQTDLIEAMAKTGAVINIKKPQFL

SPSQMSNVVEKFRECGNENLLICERGSNFGYDNLVVDMLAFGVMKHCCNDLPLIFDVTHALQCRDPSGAA

SGGRRSQVVDLARSGMAVGLAGLFLESHPDPDKARCDGPSALPLALLEPFLQQVKAIDEVVKALPTLSVS

>Q05UB6_9SYNE/3-276

RCVALGDIRFANDAPFVLIGGVNVLESRDFAVEVAGHYKAVCQRLGIPLVFKASYDKANRSSIHSYRGPG

LKDGLQMLQAVKDTHGIPVITDVHSPEEAAPAAAVCDIIQLPAFLARQTDLVEAMASTGAVINIKKPQFL

SPSQMANVVEKFRECGNEQLLICERGSNFGYDNLVVDMLGFGVMKRSCHDLPLIFDVTHALQCRDPGGAA

SGGRRSQVVDLARAGMAVGLAGLFLESHPDPNTARCDGPSALPLDQLEAFLSQVKAIDKVVKA-------

>A3Z4D3_9SYNE/3-276

QQVALGTLSFANDAPFVLIGGVNVLESRDFALEVAGHYKAVCERLDIPLVFKASFDKANRSSIHSYRGPG

LNEGLAMLQAVKARHGIPVITDVHTPEQAAPAAAVCDIIQLPAFLARQTDLVEAMARTGAVINIKKPQFL

SPSQMANVVEKFRECGNERLLICERGSNFGYDNLVVDMLGFGVMKRCCNDLPLIFDVTHALQCRDPGGAA

SGGRRSQVLELARAGMAVGLAGLFLESHPDPDQARCDGPSALPLEQLEPFLSQLKAVDHLVKN-------

>UniRef100_A5GI92

RQVSLGSITFANDAPFVLIGGVNVLESQQFALDAAAVYADVCRRLSIPLVFKASFDKANRSSIHSFRGPG

LSDGLAMLQAVKDTHGIPVITDVHTPEQAAPAAEVCDIIQLPAFLARQTDLVQAMAGTGAVINIKKPQFL

SPSQMANVVEKFRECGNEQLLICERGSNFGYDNLVVDMLGFGVMKRCCDDLPLIFDVTHALQCRDPGGAA

SGGRRSQVLDLARAGMAVGLAGLFLEAHPDPSQARCDGPSALPLHQLEPFLSQLKAVDDLVKSLPALTIQ

>A4CT88_SYNPV/3-276

RQVSLGSITFANDAPFVLIGGVNVLESQQFALDAAAKYADVCRRLAIPLVFKASFDKANRSSVHSFRGPG

LEEGLAMLQAVKDTHGMPVITDVHTPEQAAPAAEVCDIIQLPAFLARQTDLVEAMAQTGAVINIKKPQFL

SPSQMANVVEKFRECGNERLLICERGSNFGYDNLVVDMLGFGVMKRCCDDLPLIFDVTHALQCRDPGGAA

SGGRRSQVLDLARAGMAIGLAGLFLESHPDPSQARCDGPSALPLEQLEPFLSQLKAVDDLVKS-------

>A6F6J4_9GAMM/4-277

IVKIGNDIVCANNLPFVLFGGINVLEDELTTLRAVEAYKTVTDKLGIPFIFKASFDKANRSSIHSFRGVG

LEKGLHIFQRVKAEFNVPIITDVHTEAQCQPVADVVDVIQLPAFLARQTDLVQAMAATGAVINIKKPQFM

SPHQVGNIVDKFAECGNEQVIICERGHCHGYDNLIVDPLAFGVMKELTQGTPIILDSTHATQQRDAGSAA

SGGRRKQVPELSYAGLATGIAGLFIEAHENPAQARCDGPSALPFAKLEAFLAQAKAIDDVVKS-------

>A6DJU3_9BACT/9-276

------NISLANDKPFVLFGGLNVLENEADTLKVCEKYLSVCEELSIPYVFKASFDKANRSSVHSFRGVG

LEKGMEIFKALKREFNVPIITDVHEVEQCQPVADVVDVIQLPAFLARQTDLVVAMAKTNAVINIKKPQFL

SPGQMKNIVEKFAEAGNEKTILCERGSCMGYDNLVVDMLGFRTMKQSTGGAPVIFDVTHSLQCRDPLGAA

SGGRREQVLELARSGMATRLAGLFLESHPNPEEAKCDGPSALPLDLLKPFLQQVKAIDDLVKS-------

>Q1LB84_RALME

---------VPNDASLVLFGGINVLESRDLAMQACAAYVEITTRLGIPYVFKASFDKANRSSIHSYRGPG

LEEGLRILQDVKAAFDVPVLTDIHEPWQAAPVSEVADVLQLPAFLARQTDLVVSLARTGRPINIKKPQFM

SPTQIQHIVEKFREAGNENLLLCDRGTCFGYDNLVVDMLGFSVMRRATGDLPIVFDVTHSLQQRDPGNAA

SGGRRTQVVELARAGVAVGIAGLFLEAHPEPDRALCDGPSALPLDQLEPFLTQVKMLDDVVKSL------

>KDSA_BORA1

---------CANNLPFVLFGGINVLESEDLALRSCAEYVRVTDKLGIPYVFKASFDKANRSSIHSYRGPG

LDEGMRIFEKVKQEFGVPIITDVHEPWQAAIVAQTADILQLPAFLARQTDLVIALAKTGKVINIKKPQFL

SPSQMAHIAEKFREAGNDQLILCDRGSCFGYDNLVVDMLGFDVMTKSTGGLPVIFDVTHALQQRDPGGAA

SGGRRQQSAQLARSAMALGLAGLFLEAHPDPAKALCDGPSALPLAQLEPYLAQIKAIDDLVKSF------

>KDSA_CHRVO

---------VGNEAPFTLFGGINVLESRDLALRAAEEYVRVTQKLGIPYVFKGSFDKANRSSIHSYRGPG

LEEGMKILQAVKDTFGVPVITDVHEPWQAAPVAEVADVVQLPAFLARQTDLVEALAKTGRAVNIKKPQFM

SPAQIQNVVEKFVEAGNEQLILCDRGTCLGYDNLVVDMLGFGVMKKVSGNRPVIFDVTHSLQQRESGAAA

SGGRRAQVAELARAGMAVGLAGLFLEAHPNPAEAKCDGPSALPLAQLEPFLAQVKAIDDLVKSF------

>A5W7C2_PSEP1

---------CSNDAPFVLFGGINVLESEELALTACAEYVRVTEKLGIPYVFKASFDKANRSSIHSYRGPG

MEEGLRIFEKVKAEFGVPIITDVHEISQAAPVAEVVDVLQLPAFLARQTDLVVALAKTGKPVNIKKPQFL

SPSQMRNIVDKFQEAGNDQLILCDRGSCMGYDNLVVDMLGFGVMKRTCNNLPIIFDVTHALQNRDPSGAA

SGGRREQVVDLARAGMGVGLAGLFLEAHPNPIQAKCDGPSALPLDKLEPFLAQIKALDDLVKSF------

>KDSA2_PSEPK

---------CSNSAPFVLFGGINVLESEDLALTACAEYVRVTQKLGIPYVFKASFDKANRSSIHSYRGPG

MEEGLRIFEKVKAEFGVPIITDVHEIYQTAPVAEVVDVLQLPAFLARQTDLVVALAKTGKPVNIKKPQFL

SPSQMQNIVHKFKEAGNDQLILCDRGTCMGYDNLIVDMLGFGVMKRTCQDLPIIFDVTHALQNRDPSGAA

SGGRREQVVELARAGMGVGLAGLFLEAHPNPDQAKCDGPSALPLDKLEPFLAQIKALDDLVKSF------

>A1FL61_PSEPU/1-273

MIKITQNIECSNAAPFVLFGGINVLESEDLALTACAEYVRVTEKLGIPYVFKASFDKANRSSIHSYRGPG

MEEGLRIFEKVKAEFGVPIITDVHEIYQCAPVAEVVDVLQLPAFLARQTDLVVALAKTGKPVNIKKPQFL

SPSQMQNIVQKFKEAGNDQLILCDRGTCMGYDNLVVDMLGFGVMKRTCDDLPIIFDVTHALQNRDPSGAA

SGGRREQVVDLARAGMGVGLAGLFLEAHPNPDQAKCDGPSALPLDKLEPFLAQIKALDDLVKS-------

>UniRef100_A6SVC5

MININGEIKVDNAAPFVLFGGINVLESRDLAMRSCEEYVRVTGKLGIPYVFKASFDKANRSSIHSYRGPG

LEEGLRIFEDVKKTFGVPLITDVHEPYQAKIAAEVIDVLQLPAFLARQTDLVVALAQTGRVINIKKPQFL

SPPQMLNIVEKFREAGNDKLILCDRGTCFGYDNLVVDMLGFGVMKKVTGNLPIIFDVTHALQQRDSLGTA

SGGRREQVADLARAGMGVGLAGLFLEAHPDPKVAKCDGPSALPLDKLEPFLAQLKQLDDLVKSFAPLDIE

>A4G2D3_HERAR/6-272

------DIKVDNALPFVLFGGINVLESRDLAMKSCEEYVRVTQKLGIPYVFKASFDKANRSSIHSYRGPG

LEEGMRIFEEVKKTFNVPVITDVHEVHQAEIVAEVADVLQLPAFLARQTDLVVALAKTGSVINIKKPQFL

SPSQMMNIVEKFKEAGNEQLILCDRGTCFGYDNLVVDMLGFGVMKKVCNNLPIIFDVTHALQQRDPGGAA

SGGRREQVADLARAGMSVGLAGLFLEAHPDPKSAKCDGPSALPLDKLEPFLAQLKQLDDLVKS-------

>Q21LC3_SACD2

---------VANNKPFTLFGGMNVLESKDLALRTAEAYVNVTQKLGIPYVFKASFDKANRSSINSYRGPG

MEEGLRIFQAVKDEFGVPIITDVHECDQAAPVAEVVDVIQLPAFLARQTDLVKAMAATNAVINVKKPQFL

SPGQMKNIVEKFAESGNEKVILCERGACMGYDNLVVDMLGFRTMKEVSGGLPVIFDVTHSLQCRDPLGAA

SGGRRHQTAELGRAGIAVGIAGLFLEAHPNPAEAKCDGPSALPLEQLEAFLTQMKTVDDVIKGL------

>A0KMZ4_AERHH

---------VANDKPFVLFGGMNVLESRDMAMAVCEKYVEVTSKLGIPFVFKASWDKANRSSIHSYRGPG

LEEGMKIFQELKATFGMPVISDLHEPHQAKPVAEVVDVIQLPAFLARQTDLVEAMARTGNVINIKKPQFL

SPSQMKNITDKFEECGNDRLILCERGANFGYDNLVVDMLGLGVMKKSTGGYPVIFDVTHSLQCRDPMGAA

SGGRREQVTELARAGMAVGLAGLFIESHPDPKIARCDGPSALPLEKLEGFLKQMKAIDDLIKGF------

>A4SK67_AERS4

---------VANDKPFVLFGGMNVLESRDMAMAVCEKYVEVTSKLGIPFVFKASWDKANRSSIHSYRGPG

LEEGMKIFQELKATFGMPVISDLHEVHQAKPVAEVVDVIQLPAFLARQTDLVEAMARTGNVINIKKPQFL

SPSQMKNITDKFEECGNDQLILCERGANFGYDNLVVDMLGLGVMKKSTGGYPVIFDVTHSLQCRDPLGAA

SGGRRDQVTELARAGMAVGLAGLFIESHPDPKVARCDGPSALPLDKLEGFLKQMKAIDDLIKGF------

>KDSA_BDEBA

---------VANDKPFVLFAGLNVLESRDLAMQVCEHFVKVTDKLKIPYVFKSSFDKANRSSIHSYRGPG

MEEGLKIFAELKKTFGVKVITDVHEIHQAKPVAEVVDVIQLPAFLARQTDLVEAMARTGAVINVKKPQFL

SPGQMGNIVDKFAECGNDKIILCDRGTNFGYDNLVVDTLGFNIMKKVSKGSPVILDATHALQCRDPFGAA

SGGRRGQVAELSRAGLAVGLAGLFIESHPNPDKALCDGPSALPLSKVEPFLQQMKALDDLVKSF------

>UniRef100_Q6LNB8

KTVRVGDIDVANDKPFVLFGGMNVLESRDLAMKICEHYVEVTNKLGIPFVFKASFDKANRSSVHSYRGPG

MEEGLKIFQELKDTFGVKIITDIHEIYQAQPVADVVDVIQLPAFLARQTDLVEAMAKTGAVINVKKPQYM

SPGQVGNIVEKFAECDNENVILCERGALHGYDNLVVDMLGFDVMKKASKGSPIIFDVTHALQCRDPLGAA

SGGRREQTVDLARSGIATGIAGLFMEAHPAPDQARCDGPSAFPLDKLEPFLNQIKQLDDLIKGFEAIEIN

>Q1Z2A5_PHOPR/11-277

------DIDVANDKPFVLFGGMNVLESRDLAMKICEHYVEVTNKLGIPFVFKASFDKANRSSVHSYRGPG

MEEGLKIFQELKDTFGVKIITDIHEIYQAQPVADVVDVIQLPAFLARQTDLVEAMAKTGAVINVKKPQYM

SPGQVGNIVEKFAECDNENVILCERGALHGYDNLVVDMLGFDVMKKASKGSPIIFDVTHALQCRDPLGAA

SGGRREQTVDLARSGIATGIAGLFMEAHPTPDQARCDGPSAFPLDKLEPFLNQIKQLDDLIKG-------

>Q1ZJD2_9VIBR/11-277

------DIDVANDKPFVLFGGMNVLESRDLAMQMCEHYVKVTEKLGIPYVFKASFDKANRSSVHSYRGPG

MEEGLKIFQELKDTFGVKIITDIHEKEQAQPVADVVDVIQLPAFLARQTDLVEAMAKTGAVINVKKPQFM

SPGQVGNIVEKFAECDNHNVILCERGVLHGYDNLVVDMLGFDVMKKASKGSPIIFDVTHALQCRDPLGAA

SGGRREQTVDLARSGIATGIAGLFMEAHPTPDLALCDGPSAFPLDKLEPFLAQIKQLDDLIKS-------

>Q2C6E5_9GAMM/11-277

------DIDVANDKPFVLFGGMNVLESRDLAMQMCEHYVKVTEKLGIPFVFKASFDKANRSSVHSYRGPG

MEEGLKIFQELKDTFGVKIITDIHEKDQAQPVADVVDVIQLPAFLARQTDLVEAMAKTGAVINVKKPQFM

SPGQVGNIVEKFAECDNHNVILCERGVLHGYDNLVVDMLGFDVMKKASQGSPIIFDVTHALQCRDPQGAA

SGGRREQTVDLARSGIATGIAGLFMEAHPTPDLARCDGPSAFPLDKLEPFLAQIKQLDDLIKS-------

>A7K3F5_9VIBR/10-276

------HIEVANDKPFTLFGGMNVLESRDLAMQICEYYVKVTEKLGIPYVFKASFDKANRSSVHSYRGPG

LDEGMKIFQELKDTFGVKIITDVHTEAQAQPVAEVVDVIQLPAFLARQTDLVEAMAKTGAVINVKKPQFM

SPGQVGNIVEKFGECGNDRIILCERGACHGYDNLVVDMLGFGVMKKASQGSPIIFDVTHSLQMRDPSGAA

SGGRREQTVELALSGMATGIAGLFMEAHPEPNKAKCDGPSAFPLAKLEQFLSQVKQVDDLVKS-------

>UniRef100_Q5E6S9

KTVKIGNIDVANDKPFTLFAGMNVLESRDLAMQICEKYVEVTDRLGIPYVFKASFDKANRSSVHSYRGPG

MEEGLKIFQELKDTFGVKIITDIHTEAQAQPVADVVDVIQLPAFLARQTDLVEAMAKTGAVINVKKPQFM

SPDQVGNIIDKFSECGNENIILCERGSCMGYDNLVVDMLGFGVMKKASNGSPIIFDVTHSLQNRDPSGKA

SGGRRSQTVELAKAGLATGIAGLFIEAHPNPDKALCDGPSALPLDQLEPFLKQMKSLDDLIKGFEHIDIK

>UniRef100_B6EHH9

KTVKIGNIDVANDKPFTLFAGMNVLESRDLAMRMCETYVEITDRLGIPYVFKASFDKANRSSVHSYRGPG

MEEGLKIFQELKDTFGVKIITDIHTEAQAQPVADVVDVIQLPAFLARQTDLVETMARTGAVINVKKPQFM

SPDQVGNIVDKFAECGNENIILCERGSCMGYDNLVVDMLGFGVMKKVSNGSPIIFDVTHSLQNRDPSGKA

SGGRRSQTVELAKAGLATGIAGLFIEAHPTPDKALCDGPSALPLDQLEPFLKQMKALDDLIKGFEHIDIK

>A5L8D7_9GAMM/10-276

------DMPIANDKPFTLFAGMNVLESRDLAMQICEHYVKVTEKLGIPYVFKASFDKANRSSVHSYRGPG

MEEGLKIFQELKDTFGVKIITDVHTEAQAQPVADVVDVIQLPAFLARQTDLVEAMAKTGAVINVKKPQFM

SPNQVGNIVDKFAECGNDKIILCERGSCMGYDNLVVDMLGFGVMKKSSNGSPIIFDVTHSLQMRDPSGAA

SGGRREQTVELAKAGLATGIAGLFIEAHPNPDQARCDGPSALPLDKLEPFLKQMKALDDLIKG-------

>A3V064_VIBSP/10-276

------DMPIANDKPFTLFAGMNVLESRDLAMQICEHYVKVTEKLGIPYVFKASFDKANRSSVHSYRGPG

MEEGLKIFQELKDTFGVKIITDIHTEAQAQPVADVVDVIQLPAFLARQTDLVEAMAKTGAVVNVKKPQFM

SPDQVGNIVDKFAECGNDNIILCERGSCMGYDNLVVDMLGFGVMKKSSNGSPIIFDVTHALQMRDPSGAA

SGGRREQTVELAKAGLATGIAGLFLEAHPNPDQARCDGPSALPLDKLEPFLKQMKALDDLIKG-------

>A3Y4B6_9VIBR/10-276

------NMPIANDKPFTLFAGMNVLESRDLAMQICEHYVKVTEKLGIPYVFKASFDKANRSSVHSYRGPG

MEEGLKIFQELKDTFGVKIITDIHTEAQAQPVADVVDVIQLPAFLARQTDLVEAMAKTGAVINVKKPQFM

SPDQVGNIVDKFAECGNDNIILCERGSCMGYDNLVVDMLGFGVMKKSSNGSPIIFDVTHALQMRDPSGAA

SGGRREQTVELAKAGIATGIAGLFLEAHPNPDQARCDGPSALPLDKLEPFLKQMKALDDLIKG-------

>A6D987_9VIBR/10-276

------DIPVANDKPFTLFAGMNVLESRDLAMQICEHYVKVTEKLGIPYVFKASFDKANRSSVHSYRGPG

LEEGMKIFQELKDTFGVKIITDVHTEEQAQPVADVVDVIQLPAFLARQTDLVEAMAKTNAVINVKKPQFM

SPGQVGNIVDKFAECGNDKIILCERGSCHGYDNLVVDMLGFGVMKKASNGSPIIFDVTHSLQMRDPSGAA

SGGRREQTVELAKAGLATGIAGLFIEAHPNPDKALCDGPSALPLDKLEPFLAQMKALDDLIKG-------

>UniRef100_A5F692

KIVHVGDIPVANDKPFTLFAGMNVLESRDLAMQICEHYVKVTDKLGIPYVFKASFDKANRSSVHSYRGPG

LEEGMKIFQELKETFGVKIITDVHTEAQAQPVADVVDVIQLPAFLARQTDLVEAMAKTGAVINVKKPQFM

SPGQVGNIVEKFAECGNDKVILCERGSCHGYDNLVVDMLGFGVMKQASNGSPIIFDVTHSLQMRDPSGAA

SGGRREQTVELAKAGLATGIAGLFIEAHPNPDKARCDGPSALPLDKLEPFLAQMKALDDLIKSFAHIDIR

>KDSA_VIBVY

---------VANDKPFTLFAGMNVLESRDLAMQICEHYVKVTDKLGIPYVFKASFDKANRSSVHSYRGPG

LEEGMKIFQELKDTFGVKIITDVHTEAQAQPVADVVDVIQLPAFLARQTDLVEAMAKTGAVINVKKPQFM

SPGQVGNIVEKFAECGNDKIILCERGSCHGYDNLVVDMLGFGVMKKASKGSPIIFDVTHSLQMRDPSGAA

SGGRREQTVELAKAGLATGIAGLFIEAHPNPDQARCDGPSALPLDKLEPFLAQMKALDDLVKSF------

>UniRef100_A7MY69

KIVNIGDIQVANDKPFTLFAGMNVLESRDLAMQICEHYVKVTDKLGIPYVFKASFDKANRSSVHSYRGPG

LEEGMKIFQELKDTFGVKIITDVHTEAQAQPVADVVDVIQLPAFLARQTDLVEAMAKTGAVINVKKPQFM

SPGQVGNIVEKFAECGNENIILCERGSCMGYDNLVVDMLGFGVMKNASKGSPIIFDVTHSLQMRDPSGAA

SGGRREQTVELAKAGLATGIAGLFIEAHPNPDQARCDGPSALPLDKLEPFLAQMKSLDDLIKSFADIDIK

>KDSA_VIBPA

---------VANDKPFTLFAGMNVLESRDLAMQICEHYVKVTDKLGIPYVFKASFDKANRSSVHSYRGPG

LEEGMKIFQELKDTFGVKIITDVHTEAQAQPVADVVDVIQLPAFLARQTDLVEAMAKTGAVINVKKPQFM

SPGQVGNIVEKFAECGNDKIILCERGSCHGYDNLVVDMLGFGVMKNASKGSPIIFDVTHSLQMRDPSGAA

SGGRREQTVELAKAGLATGIAGLFIEAHPNPDQARCDGPSALPLDKLEPFLAQMKSLDDLIKSF------

>Q1V3A5_VIBAL/10-276

------DIQVANDKPFTLFAGMNVLESRDLAMQICEHYVKVTEKLGIPYVFKASFDKANRSSVHSYRGPG

LEEGMKILQELKDTFGVKIITDVHTEAQAQPVADVVDVIQLPAFLARQTDLVEAMAKTGAVINVKKPQFM

SPGQVGNIVEKFAECGNDKIILCERGSCHGYDNLVVDMLGFGVMKNASKGSPIIFDVTHSLQMRDPSGAA

SGGRREQTVELAKAGLATGIAGLFIEAHPNPDQARCDGPSALPLDKLEPFLAQMKSLDDLIKS-------

>A7K6G7_9VIBR/25-291

------DIQVANDKPFTLFAGMNVLESRDLAMQICEHYVKVTEKLGIPYVFKASFDKANRSSVHSYRGPG

LEEGMKIFQELKDTFGVKIITDVHTEAQAQPVADVVDVIQLPAFLARQTDLVEAMAKTGAVINVKKPQFM

SPGQVGNIVEKFAECGNDKIILCERGSCHGYDNLVVDMLGFGVMKNASKGSPIIFDVTHSLQMRDPSGAA

SGGRREQTVELAKAGLATGIAGLFIEAHPNPDQARCDGPSALPLDKLEPFLAQMKSLDDLIKS-------

>UniRef100_Q7VMV6

KSVTVGQLEIANDRPFTLFGGMNVLESRDMAMRVCEKYVAVTNKLNVPYIFKASFDKANRSSIHSYRGPG

MEEGLKIFQELKQTFGVNIITDVHEIYQCQPVAEVVDIIQLPAFLARQTDLVEAMARTGAVINVKKPQFL

SPGQMGNIVEKIVECGNQQIILCDRGTNFGYDNLVVDMLSFNIMKKVSDGCPVIFDVTHALQCRDPFGSA

SGGRRDQVTELARAGLATGLAGLFLEAHPDPNTAKCDGPSALPLDKLEAFVAQMKAIDNLVKSFAELDTA

>KDSA_PASMU/10-276

------EIEISNDKPFVLFGGMNVLESRDMAMQVCEAYVKVTEKLGVPYVFKASFDKANRSSIHSYRGPG

MEQGLKIFQELKDTFGVKLITDVHEIYQCQPVAEVVDIIQLPAFLARQTDLVAAMAKTGAIINVKKPQFL

SPGQMGNIVEKIEECGNDNVILCDRGTNFGYDNLVVDMLGFSVMKKVSKGCPVIFDVTHALQCRDPFGAA

SGGRRGQVTELARAGMAVGLAGLFLEAHPNPSQAKCDGPSALPLSALEGFVHQMKAIDDLVKH-------

>KDSA_HAES1/3-276

QVIQLGNIEIGNNKPFVLFGGMNVLESRDMAMQVCEKYVEVTQKLGVPYIFKASFDKANRSSIHSYRGPG

MEEGLKIFQELKETFGVKVITDVHEIYQCRPVAEVVDIIQLPAFLARQTDLVEAMARTGAVINVKKPQFL

SPGQIGNIVEKIAECGNNKVILCDRGTNFGYDNLVVDMLGFNIMKKVSQGCPVIFDVTHSLQCRDPFGAA

SGGRRDQVTELARSGMAIGLAGLFLESHPNPNQAKCDGPSALPLSKLEPFIAQMKAIDDLVKS-------

>UniRef100_B0UUE2

QVIQLGNIEIGNNKPFVLFGGMNVLESRDMAMQVCEKYVEVTQKLGVPYIFKASFDKANRSSIHSYRGPG

MEEGLKIFQELKETFGVKVITDVHEIYQCRPVAEVVDIIQLPAFLARQTDLVEAMARTGAVINVKKPQFL

SPGQIGNIVEKIAECGNNKVILCDRGTNFGYDNLVVDMLGFNIMKKVSQGCPVIFDVTHSLQCRDPFGAA

SGGRRDQVTELARSGMAIGLAGLFLESHPNPNQAKCDGPSALPLSKLEPFVAQMKAIDDLVKSFEEIDTS

>UniRef100_Q65TB4

KIVRIGDINVANDNPFVLFGGMNVLESRDMAMQVCEKYVEVTNKLGVPYVFKASFDKANRSSIHSYRGPG

MEEGLKIFQELKQTFGVKIITDVHEIYQCKPVAEVADVIQLPAFLARQTDLVEAMARTGAVINVKKPQFL

SPGQMGNIVEKIEECGNDKVILCDRGSNFGYDNLVVDMLGFGVMKKVSKGAPVIFDVTHSLQCRDPFGAA

SGGRRDQVTELARAGLAVGIAGLFLEAHPDPNNAKCDGPSALPLSVLEGFVSQMKALDDLVKSFPQLDTS

>UniRef100_A6VP95

KTVKIGNIDVANHKPFVLFGGMNVLESRDMAMQVCEKYVEVTDKLGVPYVFKASFDKANRSSIHSYRGPG

MEEGLKIFQELKKTFGVKVIADVHEIYQCNPVAEVVDVIQLPAFLARQTDLVEAMARTGAVINVKKPQFL

SPGQMGNIVEKIEECGNDKVILCDRGTNFGYDNLVVDMLGFGVMKKASKGCPVIFDVTHSLQCRDPFGAA

SGGRREQVTELARAGMAVGIAGLFLEAHPDPNNAKCDGPSALPLSALEGFVVQMKAIDELVKSFPELDTS

>A4N9U5_HAEIN/10-276

------NIDVANDKPFVLFGGMNVLESRDMAMQVCEAYVKVTEKLGVPYVFKASFDKANRSSIHSYRGPG

MEEGLKIFQELKDTFGVKIITDVHEIYQCQPVADVVDVIQLPAFLARQTDLVEAMARTGAVINVKKPQFL

SPGQMGNIVDKFEECGNDKIILCDRGSNFGYDNLIVDMLGFSVMKKASKGSPVIFDVTHSLQCRDPFGAA

SSGRRAQVTELARSGLAVGIAGLFLEAHPNPNQAKCDGPSALPLSALEGFVSQMKAIDDLVKS-------

>A4NFI7_HAEIN/3-276

KIVKIGNIDIANDKPFVLFGGMNVLESRDMAMQVCESYVKVTEKLGVPYVFKASFDKANRSSIHSYRGPG

MEEGLKIFQELKDTFGVKIITDVHEIYQCQPVADVVDVIQLPAFLARQTDLVEAMAKTGAVINVKKPQFL

SPGQMGNIMEKIEECGNDKIILCDRGTNFGYDNLIVDMLGFSVMKKASKGSPVIFDVTHSLQCRDPFGAA

SSGRRAQVTELARSGLAVGIAGLFLEAHPNPNQAKCDGPSALPLSALEGFVFQMRAIDDLVKS-------

>A4P0G1_HAEIN/2-257

-----------------LFGGMNVLESRDMAMQVCEAYVKVTEKLGVPYVFKASFDKANRSSIHSYRGPG

MEEGLKIFQELKDTFGVKIITDVHEIYQCQPVADVVDVIQLPAFLARQTDLVEAMARTGAVINVKKPQFL

SPGQMGNIVEKIEECGNDKIILCDRGTNFGYDNLIVDMLGFSVMKKASKGSPVIFDVTHSLQCRDPFGAA

SSGRRAQVTELARSGLAVGIAGLFLEAHPNPNQAKCDGPSALPLSALEGFVFQMKAIDDLVKS-------

>A4N0U1_HAEIN/10-276

------NIDVANDKPFVLFGGMNVLESRDMAMQVCEAYVKVIEKLGVPYVFKASFDKANRSSIHSYRGPG

MEEGLKIFQELKETFGVKIITDVHEIYQCQPVADVVDIIQLPAFLARQTDLVEAMAKTGAVINVKKPQFL

SPGQMGNIVEKIEECGNDKIILCDRGTNFGYDNLIVDMLGFSVMKKASKGSPVIFDVTHSLQCRDPFGAA

SGGRRAQVTELARSGLAVGIAGLFLEAHPNPNQAKCDGPSALPLSALEGFVSQMKAIDDLVKS-------

>UniRef100_A5UCE8

KIVKIGNIDVANDKPFVLFGGMNVLESRDMAMQVCESYVKVTEKLGVPYVFKASFDKANRSSIHSYRGPG

MEEGLKIFQELKDTFGVKIITDVHEIYQCQPVADVVDIIQLPAFLARQTDLVEAMAKTGAVINVKKPQFL

SPSQMGNIVEKIEECGNDKIILCDRGTNFGYDNLIVDMLGFSVMKKASKGSPVIFDVTHSLQCRDPFGAA

SSGRRAQVTELARSGLAVGIAGLFLEAHPNPNQAKCDGPSALPLSALEGFVFQMKAIDDLVKSFPELDTS

>A4NMT8_HAEIN/10-276

------NIDVANDKPFVLFGGMNVLESRDMAMQVCESYVKVTEKLGVPYVFKASFDKANRSSIHSYRGPG

MEEGLKIFQELKDTFGVKIITDVHEIYQCQPVADVVDIIQLPAFLARQTDLVEAMAKTGAVINVKKPQFL

SPSQMGNIVEKIEECGNDKIILCDRGTNFGYDNLIVDMLGFSVMKKASKGSPVIFDVTHSLQCRDPFGAA

SSGRRAQVTELARSGLAVGIAGLFLEAHPNPNQAKCDGPSALPLSALEGFVFQMRAIDDLVKS-------

>A5UEF6_HAEIG/10-276

------NIDVANDKPFVLFGGMNVLESRDMAMQVCEAYVKVTEKLGVPYVFKASFDKANRSSIHSYRGPG

MEEGLKIFQELKDTFGVKIITDVHEIYQCQPVADVVDIIQLPAFLARQTDLVEAMAKTGAVINVKKPQFL

SPSQMGNIVEKIEECGNDKIILCDRGTNFGYDNLIVDMLGFSVMKKASKGNPVIFDVTHSLQCRDPFGAA

SSGRRAQVTELARSGLAVGIAGLFLEAHPNPNQAKCDGPSALPLSALEGFVSQMKAIDDLVKS-------

>UniRef100_Q4QKR7

KIVKIGNIDVANDKPFVLFGGMNVLESRDMAMQVCEAYVKVTEKLGVPYVFKASFDKANRSSIHSYRGPG

MEEGLKIFQELKDTFGVKIITDVHEIYQCQPVADVVDIIQLPAFLARQTDLVEAMAKTGAVINVKKPQFL

SPGQMGNIVEKIEECGNDKIILCDRGTNFGYDNLIVDMLGFSVMKKASKGSPVIFDVTHSLQCRDPFGAA

SSGRRAQVTELARSGLAVGIAGLFLEAHPNPNQAKCDGPSALPLSALEGFVSQMKAIDDLVKSFPELDTS

>KDSA_HAEIN

---------VANDKPFVLFGGMNVLESRDMAMQVCEAYVKVTEKLGVPYVFKASFDKANRSSIHSYRGPG

MEEGLKIFQELKDTFGVKIITDVHEIYQCQPVADVVDIIQLPAFLARQTDLVEAMAKTGAVINVKKPQFL

SPSQMGNIVEKIEECGNDKIILCDRGTNFGYDNLIVDMLGFSVMKKASKGSPVIFDVTHSLQCRDPFGAA

SSGRRAQVTELARSGLAVGIAGLFLEAHPNPNQAKCDGPSALPLSALEGFVSQMKAIDDLVKSF------

>KDSA_PASHA/10-276

------NLEIANDKPFTLFGGMNVLESRDMAMAVCEKYVEVTNKLGVPYVFKASFDKANRSSIHSYRGPG

MEEGLKIFQELKDTFGVKIITDVHEIYQCKPVAEVVDIIQLPAFLARQTDLVEAMARTGAVINVKKPQFL

SPGQMGNIVEKIEECGNDQVILCDRGTNFGYDNLVVDMLGFSVMKQVSKGCPVIFDVTHSLQCRDPFGAA

SSGRRAQVTELARAGLAVGIAGLFLEAHPDPNNAKCDGPSALPLSTLEAFVGQMKAIDDLVKS-------

>A7JUS6_PASHA/10-276

------NIEVANHKPFTLFGGMNVLESRDMALRVCEQYVEVTQKLNVPYVFKASFDKANRSSIHSYRGPG

MEEGLKIFQELKQTFGVNIITDVHEIYQCKPVAEVVDVIQLPAFLARQTDLVEAMARTGAVINVKKPQFL

SPGQMGNIVEKIAECGNDQVILCDRGTNFGYDNLVVDMLGFGIMKKVSKGCPVIFDVTHSLQCRDPFGAA

SGGRRDQVTELARSGMAIGLAGLFLEAHPDPNSAKCDGPSALPLSKLEAFVSQMKAIDELVKS-------

>A3N2M6_ACTP2

---------VANDKPFTLFGGMNVLESRDMAMRVCEQYVEVTNKLGVPYVFKASFDKANRSSIHSYRGPG

MEEGLKIFQELKDTFGVSIITDVHEIYQCKPVAEVVDIIQLPAFLARQTDLVEAMARTGVVINVKKPQFL

SPGQMGNIVEKIAECGNENVILCDRGTNFGYDNLVVDMLGFNIMKKVSKGCPVIFDVTHSLQCRDPFGAA

SGGRRDQVTELARSGMAIGLAGLFLEAHPDPNSAKCDGPSALPLSKLEAFVSQMKAIDDLVKSF------

>A3N3X8_ACTP2/10-276

------NIEVANDKPFTLFGGMNVLESRDMAMRVCEQYVEVTNKLGVPYVFKASFDKANRSSIHSYRGPG

MEEGLKIFQELKDTFGVNIITDVHEIYQCKPVAEVVDIIQLPAFLARQTDLVEAMARTGAVINVKKPQFL

SPGQMGNIVEKIAECGNENVILCDRGTNFGYDNLVVDMLGFNIMKKVSKGCPVIFDVTHSLQCRDPFGAA

SGGRRDQVTELARSGMAIGLAGLFLEAHPDPNNAKCDGPSALPLSKLEAFVSQMKAIDDLVKS-------

>UniRef100_B0BU69

KIVKVGNIEVANDKPFTLFGGMNVLESRDMAMRVCEQYVEVTNKLGVPYVFKASFDKANRSSIHSYRGPG

MEEGLKIFQELKDTFGVNIITDVHEIYQCKPVAEVVDIIQLPAFLARQTDLVEAMARTGAVINVKKPQFL

SPGQMGNIVEKIAECGNENVILCDRGTNFGYDNLVVDMLGFNIMKKVSKGCPVIFDVTHSLQCRDPFGAA

SGGRRDQVTELARSGMAIGLAGLFLEAHPDPNSAKCDGPSALPLSKLEAFVSQMKAIDDLVKSFEEIDTS

>UniRef100_Q2NRS7

LVVNIGDIQAANDLPFVLFGGMNVLESRDLAMRICEHYVTVTEKLGIPYVFKASFDKANRSSIHSYRGPG

LDEGMKIFSELKAQFGVKVITDVHEAGQAQSVADVVDVIQLPAFLARQTDLVEAMARTGAVINVKKPQFI

SPGQVGNIVDKFREAGNHQVILCDRGSNFGYDNLVVDMLGFNVMKQVSGGCPVIFDVTHALQTRDPFGAA

SGGRRAQVSELARAGMAVGIAGLFIEAHPDPANAKCDGPSALPIAKLEPFLRQMKAIDELVKSFPELDTG

>UniRef100_B4EVS0

KVVSIGDIKVANNLPFVLFGGMNVLESRDLAMRICEHYVTVTQKLDIPYVFKASFDKANRSSIHSYRGPG

LDEGMKIFQELKETFGVKIITDVHEPSQAQPVSEVVDVIQLPAFLARQTDLVEAMAKTGAVINVKKPQFV

SPGQMGNIVEKFKEGGNDQVILCDRGSNFGYDNLVVDMLGFHVMMQASEGAPVIFDVTHSLQCRDPFGAA

SGGRRGQVAELARAGMAVGLAGLFLEAHPDPDHARCDGPSALPLAKLEPFLAQIKAIDSLVKSFPELDTS

>UniRef100_Q6D547

KVVKIGDIPVANDLPFVLFGGMNVLESRDLAMRICEHYVTVTQKLGIPYVFKASFDKANRSSIHSYRGPG

LEEGMKIFQELKQTFGVKIITDVHDSHQAQPVADVVDVIQLPAFLARQTDLVEAMAKTGAVINVKKPQFV

SPGQMGNIVDKFIEGGNDQIILCDRGSNFGYDNLVVDMLGFNVMKHASNGSPVIFDVTHALQCRDPFGAA

SGGRRGQVTELARAGMAVGLAGLFIEAHPDPANAKCDGPSALPLDKLEPFLQQIKAIDDLVKSFPELDTS

>A7MKA7_ENTS8/10-276

------DINVANDLPFVLFGGMNVLESRDLAMRICEHYVTVTQKLGIPYVFKASFDKANRSSIHSYRGPG

LEEGMKIFQELKQTFGVKIITDVHTAEQAQPVADVVDVIQLPAFLARQTDLVEAMAKTGAVINVKKPQFI

SPGQIGNIVDKFKEGGNEQVILCDRGTNFGYDNLVVDMLGFGVMKKVSGNAPVIFDVTHALQCRDPFGAA

SSGRRAQVTELARAGMATGLAGLFIEAHPDPENAKCDGPSALPLAKLEAFLTQIKAIDDLVKS-------

>UniRef100_B2VEI7

KVVSIGDIKVANDLPFVLFGGMNVLESRDLAMRICEHYVTVTQKLGIPYVFKASFDKANRSSIHSYRGPG

LEEGMKIFQEIKQAFGVKIITDVHEASQAQTVADVVDVIQLPAFLARQTDLVEAMAKTGAVINVKKPQFV

SPGQMGNIVDKFAEGGNENVILCDRGANFGYDNLVVDMLGFNVMKQVSNNSPVIFDVTHALQCRDPMGAA

SSGRRGQVSELARAGMAVGIAGLFIEAHPDPANAKCDGPSALPLDKLEPFLLQMKAIDDLVKSFPELDTN

>UniRef100_A4WBC2

KVVSIGDINVANDLPFVLFGGMNVLESRDLAMRICEHYVTVTQKLGIPYVFKASFDKANRSSINSYRGPG

LEEGMKIFQELKQTFGVKVITDVHEASQAQPVAEVVDVIQLPAFLARQTDLVEAMAKTGAVINVKKPQFV

SPGQMGNIVDKFIEGGNDQIILCDRGANFGYDNLVVDMLGFSVMKKVSNNSPVIFDVTHALQCRDPFGAA

SSGRRGQVTELARAGMATGIAGLFIEAHPDPANAKCDGPSALPLDKLEPFLKQIKAIDDLVKSFDELDTS

>A6TAN5_KLEPN/10-276

------DINVANDLPFVLFGGMNVLESRDLAMRICEHYVTVTQKLGIPYVFKASFDKANRSSIHSYRGPG

LEEGMKIFQELKQTFGVKIITDVHEASQAQPVADVVDVIQLPAFLARQTDLVEAMAKTGAVINVKKPQFV

SPGQMGNIVDKFIEGGNDKVILCDRGANFGYDNLVVDMLGFGVMKKASNNSPVIFDVTHALQCRDPFGAA

SGGRRAQVSELARAGMAVGIAGLFIEAHPDPDHAKCDGPSALPLDKLEPFLKQMKAIDDLVKS-------

>A9MPA5_SALAR

---------VANDLPFVLFGGMNVLESRDLAMRICEHYVTVTQKLGIPYVFKASFDKANRSSIHSYRGPG

LEEGMKIFQELKQTFGVKVITDVHEASQAQPVADVVDVIQLPAFLARQTDLVEAMAKTGAVINVKKPQFV

SPGQMGNIVDKFHEGGNDKVILCDRGANFGYDNLVVDMLGFGVMKKVSGNCPVIFDVTHALQCRDPFGAA

SGGRRGQVTELARAGMAVGLAGLFLESHPDPANAKCDGPSALPLAKLEQFLTQIKAIDDLVKSF------

>KDSA_SALCH

---------VANDLPFVLFGGMNVLESRDLAMRICEHYVTVTQKLGIPYVFKASFDKANRSSIHSYRGPG

LEEGMKIFQELKQTFGVKVITDVHEASQAQPVADVVDVIQLPAFLARQTDLVEAMAKTGAVINVKKPQFV

SPGQMGNIVDKFHEGGNDKVILCDRGANFGYDNLVVDMLGFSVMKKVSGNSPVIFDVTHALQCRDPFGAA

SGGRRGQVTELARAGMAVGLAGLFLESHPDPANAKCDGPSALPLAKLEQFLTQIKAIDDLVKSF------

>KDSA_SALPA

---------VANDLPFVLFGGMNVLESRDLAMRICEHYVTVTQKLGIPYVFKASFDKANRSSIHSYRGPG

LEEGMKIFQELKQTFGVKVITDVHEASQVQPVADVVDVIQLPAFLARQTDLVEAMAKTGAVINVKKPQFV

SPGQMGNIVDKFHEGGNDKVILCDRGANFGYDNLVVDMLGFSVMKKVSGNSPVIFDVTHALQCRDPFGAA

SGGRRGQVTELARAGMAVGLAGLFLESHPDPANAKCDGPSALPLAKLEQFLTQIKAIDDLVKSF------

>KDSA_CITK8

---------VANDLPFVLFGGMNVLESRDLAMRICEHYVTVTQKLGIPYVFKASFDKANRSSIHSYRGPG

LEEGMKIFQELKQTFGVKVITDVHEASQAQPVADVVDVIQLPAFLARQTDLVEAMAKTGAVINVKKPQFV

SPGQMGNIVDKFHEGGNDKVILCDRGANFGYDNLVVDMLGFSVMKKVSGNSPVIFDVTHALQCRDPFGAA

SGGRRAQVTELARAGMAVGLAGLFIEAHPDPEHAKCDGPSALPLAKLEPFLKQIKAIDDLVKSF------

>UniRef100_B7LSH5

KVVSIGDINVANDLPFVLFGGMNVLESRDLAMRICEHYVTVTQKLGIPYVFKASFDKANRSSIHSYRGPG

LEEGMKIFQELKQTFGVKIITDVHEPSQAQPVADVVDVIQLPAFLARQTDLVEAMAKTGAVINVKKPQFV

SPGQMGNIVDKFKEGGNDKVILCDRGANFGYDNLVVDMLGFSVMKKVSGNSPVIFDVTHALQCRDPFGAA

SGGRRAQVTELARAGMAVGLAGLFIEAHPDPEHAKCDGPSALPLAKLEPFLKQMKAIDELVKGFEELDTS

>KDSA_ECOL5/10-276

------DINVANDLPFVLFGGMNVLESRDLAMRICEHYLTVTQKLGIPYVFKASFDKANRSSIHSYRGPG

LEEGMKIFQELKQTFGVKIITDVHEPSQAQPVADVVDVIQLPAFLARQTDLVEAMAKTGAVINVKKPQFV

SPGQMGNIVDKFKEGGNEKVILCDRGANFGYDNLVVDMLGFSIMKKVSGNSPVIFDVTHALQCRDPFGAP

SGGRRAQVAELARAGMAVGLAGLFIEAHPDPEHAKCDGPSALPLAKLEPFLKQMKAIDDLVKG-------

>UniRef100_Q8FHZ8

KVVSIGDINVANDLPFVLFGGMNVLESRDLAMRICEHYVTVTQKLGIPYVFKASFDKANRSSIHSYRGPG

LEEGMKIFQELKQTFGVKIITDVHEPSQAQPVADVVDVIQLPAFLARQTDLVEAMAKTGAVINVKKPQFV

SPGQMGNIVDKFKEGGNEKVILCDRGANFGYDNLVVDMLGFSIMKKVSGNSPVIFDVTHALQCRDPFGAA

SGGRRAQVTELARAGMAVGLAGLFIEAHPDPEHAKCDGPSALPLAKLEPFLKQMKAIDDLVKGFEELDTS

>KDSA_ECO57/10-276

------DINVANDLPFVLFGGMNVLESRDLAMRICEHYVTVTQKLGIPYVFKASFDKANRSSIHSYRGPG

LEEGMKIFQELKQTFGVKIITDVHEPSQAQPVADVVDVIQLPAFLARQTDLVEAMAKTGAVINVKKPQFV

SPGQMGNIVDKFKEGGNEKVILCDRGANFGYDNLVVDMLGFSIMKKVSGNSPVIFDVTHALQCRDPFGAA

SGGRRAQVAELARAGMAVGLAGLFIEAHPDPEHAKCDGPSALPLAKLEPFLRQMKAIDDLVKG-------

>A2UH75_ECOLX/10-276

------DINVANDLPFVLFGGMNVLESRDLAMRICEHYVTVTQKLGIPYVFKASFDKANRSSIHSYRGPG

LEEGMKIFQELKQTFGVKIITDVHEPSQAQPVADVVDVIQLPAFLARQTDLVEAMAKTGAVINVKKPQFV

SPGQMGNIVDKFKEGGNEKVILCDRGANFGYDNLVVDMLGFSIMKKVSGNSPVIFDVTHALQCRDPFGAA

SGGRRAQVAELARAGMAVGLAGLFIEAHPDPEHAKCDGPSALPLAKLEPFLKQMKAIDDLVKG-------

>KDSA_PHOLL/10-276

------DIKVANDLPFVLFGGMNVLESRDLAMSICEHYVTVTQKLGIPYVFKASFDKANRSSIHSYRGPG

LEEGMKIFQELKQTFGVKIITDVHESAQAQPVAEVVDVIQLPAFLARQTDLVEAMARTGAVINVKKPQFI

SPGQMGNIVDKFKEGGNDQVILCDRGSNFGYDNLVVDMLGFNVMAQATGGHPVIFDVTHSLQCRDPFGAA

SGGRRAQVAELARAGMAVGIAGLFLEAHPDPANAMCDGPSALPLAKLEPFLSQMKAIDDVVKS-------

>A8GDA7_9ENTR/10-276

------DINVANDLPFVLFGGMNVLESRDLAMRICEHYVTVTQKLGIPYVFKASFDKANRSSIHSYRGPG

LEEGMKIFEEIKKTFGVKTITDVHEASQAQPVSEVVDVIQLPAFLARQTDLVEAMAKTGAVINVKKPQFV

SPGQMGNIVDKFKEGGNDQVILCDRGSNFGYDNLVVDMLGINVMKNATGGHPVIFDVTHALQTRDPFGAA

SGGRRAQVAELARAGMAVGLAGLFIEAHPEPNSAKCDGPSALPLDKLEPFLVQMKAIDDLVKS-------

>UniRef100_A1JRS8

KVVSIGDIKVANDLPFVLFGGMNVLESRDLAMRICEHYVTVTQKLGIPYVFKASFDKANRSSIHSYRGPG

LEEGMKIFQELKQQFGVKVITDVHEISQCQPVAEVVDVIQLPAFLARQTDLVEAMARTGAVINVKKPQFV

SPGQMGNIVEKFKEAGNDQVILCDRGSNFGYDNLVVDMLGINVMVKATGGHPVIFDVTHALQCRDPFGSA

SGGRRAQVAELARAGMAVGLAGLFIEAHPEPNSAKCDGPSALPLDKLEPFLMQMKAIDDLVKSFPELDTS

>A6BQZ2_YERPE/10-276

------DINVANDLPFVLFGGMNVLESRDLAMRICEHYVTVTQKLGIPYVFKASFDKANRSSIHSYRGPG

LEEGMKIFQELKQQFGVKVITDVHEASQAQPVSEVVDVIQLPAFLARQTDLVEAMARTGAVINVKKPQFV

SPGQMGNIVEKFKEAGNDQVILCDRGSNFGYDNLVVDMLGINVMVQATGGHPVIFDVTHALQCRDPFGAA

SGGRRAQVAELARAGMAVGLAGLFIEAHPEPNSAKCDGPSALPLDKLEPFLVQMKAIDDLVKS-------

>A0YEN9_9GAMM/10-276

------SVSISNDLPFVLFGGMNVLESRDMAMTMAEQYVEITQRLGIPYVFKASFDKANRSSIHSFRGPG

LEEGLKIFEEIKQTFNVPVITDIHEIEQAQAVADVCDVIQLPAFLARQTDLVAAMAATGNVINIKKPQFL

SPSQMKNIVEKFAECGNDKVMLCERGSNFGYDNLVVDMLGFRTMKEVSGGAPIIFDVTHALQCRDPMGAA

SGGRRHQVAELGRAGMSQKLAGLFLEAHPDPDNAKCDGPSALPMAVLEDFLKEMKAVDDLMKS-------

>Q1YSS8_9GAMM/10-276

------DIDVSNDAPMVLFGGMNVLESRDMAMQIAEAYVRATEKLGIPYVFKASFDKANRSSITSYRGPG

LDEGLKIFQEIKQTFNIPLITDVHEIEQAKPVAEVCDVIQLPAFLARQTDLVQAMAETGAVINVKKPQFL

SPGQMGNIVDKFRECGNDQIMLCERGTCMGYDNLVVDMLGFRTMKDVSGGLPLIFDVTHALQCRDPLGAA

SGGRRHQVAELGRAGIAVGIAGLFLEAHPDPDQARCDGPSALPLDKLEPFLAQMKAFDDLIKS-------

>Q70JW1_NEIME/10-276

------NIKISNSLPFTLFGGINVLEDLDSTLFACEKYVEVTEKLKIPYVFKASFDKANRSSIHSYRGVG

LEEGMNIFSAVKDRFGVPVITDVHEPYQCNAVAEVVDILQLPAFLARQTDLVFAMAQTGRVINIKKPQFL

SPEQMKNIVEKFKEAGNDQLILCERGANFGYDNLVVDMLGFGVMKKTCDDLPVIFDVTHALQKRDSGSSA

SGGRREQVLDLAMAGMATGLAGLFLEAHPNPDKARCDGPSALPLDKLEDFLLRVKAVDETVKS-------

>KDSA_NEIMA

---------LGNNLPFVLFGGINVLESLDSTLQTCAHYVEVTRKLGIPYIFKASFDKANRSSIHSYRGVG

LEEGLKIFEKVKAEFGIPVITDVHEPHQCQPVAEVCDVIQLPAFLARQTDLVAAMAETGNVINIKKPQFL

SPSQMKNIVEKFHEADNGKLILCERGSSFGYDNLVVDILGFGVMKQTCGNLPVIFDVTHSLQTRDAGSAA

SGGRRAQALDLALAGMATRLAGLFLESHPDPKLAKCDGPSALPLHLLEDFLIRIKALDDLIKSQ------

>UniRef100_Q9JZ55

MDIKINDITLGNNSPFVLFGGINVLESLDSTLQTCAHYVEVTRKLGIPYIFKASFDKANRSSIHSYRGVG

LEEGLKIFEKVKAEFGIPVITDVHEPHQCQPVAEVCDVIQLPAFLARQTDLVVAMAKTGNVVNIKKPQFL

SPSQMKNIVEKFHEAGNGKLILCERGSSFGYDNLVVDMLGFGVMKQTCGNLPVIFDVTHSLQTRDAGSAA

SGGRRAQALDLALAGMATRLAGLFLESHPDPKLAKCDGPSALPLHLLEDFLIRIKALDDLIKSQPILTIE

>UniRef100_A9LZL2

MDIKINDFTLGNNSPFVLFGGINVLESLDSTLQTCAHYVEVTRKLGIPYIFKASFDKANRSSIHSYRGVG

LEEGLKIFEKVKAEFGIPVITDVHEPHQCQPVAEVCDVIQLPAFLARQTDLVVAMAKTGNVVNIKKPQFL

SPSQMKNIVEKFHEAGNGKLILCERGSSFGYDNLVVDMLGFGVMKQTCGNLPVIFDVTHSLQTRDAGSAA

SGGRRAQALDLALAGMATRLAGLFLESHPDPKLAKCDGPSALPLHLLEDFLIRIKALDDLIKSQPILTIE

>UniRef100_A1KUB4

MDIKINGITVGNDAPFVLFGGINVLEDLDSTLQTCAHYVEVTRKLGIPYIFKASFDKANRSSIHSYRGVG

LEEGLKIFEKVKAEFGIPVITDVHEPHQCQPVAEVCDVIQLPAFLARQTDLVAAMAETGNVINIKKPQFL

SPSQMKNIVEKFREAGNGKLILCERGSSFGYDNLVVDMLGFGVMKQTCGNLPVIFDVTHSLQTRDAGSAA

SGGRRAQALDLALAGMATRLAGLFLESHPDPKLAKCDGPSALPLHLLEDFLIRIKALDDLIKSQPILTIE

>UniRef100_Q5F8Z0

MDIKINDITLGNNSPFVLFGGINVLEDLDSTLQTCAHYVEVTRKLGIPYIFKASFDKANRSSIHSYRGVG

LEEGLKIFEKVKAEFGIPVITDVHEPHQCQPVAEVCDVIQLPAFLARQTDLVAAMAETGNVINIKKPQFL

SPSQMKNIVEKFREAGNGKLILCERGSSFGYDNLVVDMLGFGVMKQTCGNLPVIFDVTHSLQTRDAGSAA

SGGRRAQALDLALAGMATRLAGLFLESHPDPKLAKCDGPSALPLHLLENFLIRIKALDDLIKSQPILTIE

>A4RVU8_9CHLO/18-280

-------------QPFFLMAGPNVIQSEEHIFAMCRELKRVTDELDVPFIFKSSFDKANRTSITSFRGPG

IDDGLRILQAVKTTFDVPIVTDIHTEEQAEPVSAVADVMQIPAFLCRQTDLLLAAGRTGRVINIKKGQFC

APSVMRNSAKKVFSTGNENVLLCERGSTFGYSDLVVDMRNIPLMRDA--GCPIVADVTHSLQQPAGLGAV

SGGSRELIPTIARSCVAAGVDGLFMEVHNDPNSSPVDGPTQWPLRHFPELLAELKAIALATKA-------

>Q9ARD1_SOLLC/10-277

--------QLKAAEPFFLLAGPNVIESEDHILYMAKHLKNITSKLGLKFVFKSSFDKANRTSSKSFRGPG

LAEGLKILEKVKTIYDIPIVTDVHESIQCEAVGRVADIIQIPAFLCRQTDLLVAAAKTGKVINIKKGQFC

ASSVMVNSAEKVRLAGNQNVMVCERGTMFGYNDLIVDPRNFEWMREA--NCPVVADITHSLQQPAGKGGV

SGGLRELIPCIARTAVAVGGDGLFMEVHNDPLSAPVDGPTQWPLRHLEELLEELVALGRVSKG-------

>A7QMI8_VITVI/16-277

--------------PFFLLAGPNVIESEEHIMYMAKAIKSLTSKFGLKLVFKSSFDKANRTSSKSFRGPG

MIEGLKILEKVKVAYDIPIVTDVHEIEQCEPVGRVADIIQIPAFLCRQTDLLVAAAKTGKIINIKKGQFC

APSVMVNSAEKIRLAGNENVMVCERGTMFGYNDLIVDPRNLEWMREA--NCPIVADVTHSLQQPAGRGGV

SGGLRELIPCIARTAVAVGVDGIFMEVHNDPLNAPVDGPTQWPLRHLEELLEELVAIARVSKG-------

>Q0IPE5_ORYSJ/11-278

--------QLKAAQPFFLLAGPNVIESEEHVLKMAKHIKGITTKLGLPLVFKSSFDKANRTSSKSFRGPG

LEEGLKILEKVKATYDIPVVTDVHESHQCEAVGRVADIIQIPAFLCRQTDLLVAAAKTGKIINIKKGQFC

APSVMANSAEKIRLAGNQNVMVCERGTMFGYNDLIVDPRNFEWLREA--NCPVVADVTHALQQPAGKGGV

SGGLRELIPCIARTSVAVGVDGIFMEVHDDPLNAPCDGPTQWPLRNLEELLEELIAIARVTKG-------

>A2Q1V7_MEDTR/10-277

--------QLKAAHPFFLLAGPNVIESEEHIMKMAKHIKAISSKFGIPLVFKSSFDKANRTSSKSFRGPG

MVEGLKILEKVKVAYDIPIVTDVHEASQCEAVGRVADIIQIPAFLCRQTDLLVAAAQTGKIINIKKGQFC

APSVMANSAEKVRLAGNPNVMVCERGTMFGYNDLIVDPRNLEWLREA--NCPVVADITHSLQQPAGKGGV

SGGLRELIPCIARTSVAVGVDGIFMEVHDDPLSAPVDGPTQWPLRHLEELLEELIAISGKQRE-------

>UniRef100_Q9AV97

AATSLLYNQLKAAEPFFLLAGPNVIESEEHILRMAKHIKDISTKVGLPLVFKSSFDKANRTSSKSFRGPG

MAEGLKILEKVKVAYDLPIVTDVHESSQCEAVGKVADIIQIPAFLCRQTDLLVAAAQTGKIINIKKGQFC

APSVMENSAEKIRLAGNPNVMVCERGTMFGYNDLIVDPRNFEWMREA--NCPVVADITHSLQQPAGKGGV

SGGLRELIPCIARTAVAVGVDGIFMEVHDDPLSAPVDGPTQWPLRHLEELLEELIAIARVTKGKQRLQID

>Q9SA33_ARATH/25-286

--------------PFFLLAGPNVIESEEHVLRMAKSIKDISTKLGLPLVFKSSFDKANRTSSKSFRGPG

MAEGLKILEKVKVAFDLPIVTDVHESSQCEAVGKVADIIQIPAFLCRQTDLLVAAAQSGKIINIKKGQFC

GHSVMRNSAEKVRLAGNPNVMVCERGTMFGYNDLIVDPRNLEWMREA--DCPVVADITHSLQQPAGKGGV

SGGLRELIPCIARTAVAVGVDGIFMEVHDDPLNAPVDGPTQWPLRHLEELLEELIAIASVTKG-------

>Q0C410_HYPNA/3-283

LTHTPSGWTIGGKGPLAVMAGLNVLEDEGLALEVGTELKRICVSLGLPYVFKASFDKANRSSITSYRGPG

LETGLKILSSVKAKLGVPVVTDLHEPAQAEPVAAVADIIQLPAFLVRQTDLVIATAREKALIQAKKAQFM

APWDAKNILTKIAEVAETGVIMCERGVSFGYNNLVVDMLAIPEMKKL--GCPVTIDATHAVQLPGADGAS

TGGRRDGVAIIAKSAIAAGADGVFLEFHTDPDKALCDGPSCLALSGAEALLSTLKSIHSAANG-------

>A7HXZ8_9RHIZ/3-280

LNLSKWNIAIGDGQPLFCIAGLNVLEDRDLAFETARHLKAVMEKLELPFVFKASFDKANRSSIKSYRGPG

LEKGLAILAEVKEKFDLPIATDIHDETQAEAVAEVADLVQIPAFLCRQTDLVVAAAKAGGLLHVKKGQFL

APWDCRNIVSKIKEAAGDLTILCERGSSFGYNNLVVDMLAFAEMKKL--GVPVTIDATHAVQLPGADGAS

TGGRRDGVPIIAKAAVAAGADGVFLEFHPEPDKALCDPLSCLPLAGAEELFASLKAIRAA----------

>Q0M589_9CAUL/1-283

VHLKRWNISMGAGQPLAVIAGLNVLENLDLALEVGRELKAITAELGLPFVFKASFDKANRSSITSYRGPG

LKDGLAMLAEVKAKLNVPIATDIHEIDQAQPVAAVADIVQIPAFLCRQTDLIVAASAAGGWLHVKKAQFL

APWDCKNIISKAREAADLQIILCERGASFGYNNLVVDMLGIPQMQSL--GVPVTIDATHAVQLPGADGGS

TGGRREGVPIIAKAAVAAGADGVFLEFHPEPDKALCDAPSCLALGDAAGLLKTLKALHGVVRA-------

>KDSA_PARUW

---------IGPKEPLVIMSGPCVIESETHCLKAAETLKNMFEKYNVSLIFKSSYDKANRSAYDSFRGPG

LEEGLRILERIQKEFGLAVVTDVHSPQEATTAGSVCEIIQIPAFLCRQTDLILAAAQTGAIVSIKKGQFL

APWDMENVIRKMESGGNSNIILVDRGTTFGYNNLISDMRGIPIMQEL--GYPVCFDATHSVQKPGGLGSK

SGGDREFIPILAKAALAAGANCLFIESHPNPSEAKSDAASVMDFKDLDQLLPQFKELYELIQKQ------

>KDSA_RHOBA

---------CGPGEPLLVIAGPCVLQSRELALQIGEELARINQRSDVQVIFKASFDKANRTSLAAQRGPG

IEQGLGLLEAVRANTGLPVTTDIHLPEQAAAVGEVCDLLQIPAFLARQTDLLVAAASTGRPVNVKKGQFM

SPGDMRYVVDKLRGSGDGGVMACERGTFFGYGRLVNDMQAIPIMRSL--GVPVVFDATHSVQQPGGLGGA

TGGNREMVEPLARAAVAIGCDALFFETHPDPQTSPSDGPNMIPLDRFAGTLDRLLRLREAVDGL------

>UniRef100_Q8RE91

NKVKVGNIVFGGKKRFVLIAGPCVMESQELMDEVAGGIKEICDRLGIEYIFKASFDKANRSSIYSYRGPG

LEEGMKMLTKIKEKFNVPVITDVHEAWQCKEVAKVADILQIPAFLCRQTDLLIAAAETGKAVNIKKGQFL

APWDMKNIVVKMEESRNKNIMLCERGSTFGYNNMVVDMRSLLEMRKF--NYPVIFDVTHSVQKPGGLGTA

TSGDREYVYPLLRAGLAIGVDAIFAEVHPNPAEAKSDGPNMLYLKDLEEILKIAIEIDKIVKGV------

>A6Q4R3_NITSB

---------------MILIAGPCVIESQEQLEIIASDLKRYHKSDEIDFYFKASFDKANRTSLDSYRGPG

LEKGLEMLAKIKEDFGYKILTDVHESYQVEAVAEVADVIQIPAFLCRQTDLLVAAATTERIVNIKKGQFM

HPEDMKYSVLKVLKTRDAGVWLTERGTSFGYRNLVVDMRSLVIMREF---APVIFDATHAVQMPGGAGGK

SSGNKEFVAPLSRAVAAVGVDGFFFETHYDPSCALSDGPNMVTPSELEAIVDDIFKIERALHV-------

>KDSA_HELAH

---------KTKTPKLVLIAGPCVIESLDNLRGIAIKLQPLANNERLDFYFKASFDKANRTSLESYRGPG

LEKGLEMLQAIKDEFGYKILTDVHESYQVSIAVKVADILQIPAFLCRQTDLIVEVSQTNAIINIKKGQFM

SPKDMQYSVLKALKTRTNGVWLCERGSSFGYGNLVVDMRSLKIMREF---APVIFDATHSVQMPGGANGK

SSGESSFAPILARAAAAVGVDGLFAETHIDPKNALSDGANMLKPNELESLVTDMLKIQNLF---------

>UniRef100_B6JPA2

----MKTSNTKTPKP-VLIAGPCVIESLENLRSIAIKLQPLANNERLDFYFKASFDKANRTSLESYRGPG

LEKGLEMLQTIKDEFGYKILTDVHESYQASVAAKVADILQIPAFLCRQTDLIVAVSQTDAIVNIKKGQFM

NPKDMQYSVLKALKTRANGVWLCERGSSFGYGNLVVDMRSLTIMREF---APVIFDATHSVQMPGGANGK

SSGDSSFAPILARAAAAVGIDGLFAETHVDPKNALSDGANMLKPDELEHLVTDMLKIQNLF---------

>KDSA_HELPJ

--------NTKTPKP-VLIAGPCVIESLENLRSIAIKLQPLANNERLDFYFKASFDKANRTSLESYRGPG

LEKGLEMLQTIKDEFGYKILTDVHESYQASVAAKVADILQIPAFLCRQTDLIVEVSQTNAIVNIKKGQFM

NPKDMQYSVLKALKTRTNGVWLCERGSSFGYGNLVVDMRSLKIMREF---APVIFDATHSVQMPGGANGK

SSGDSSFPPILPRAAAAVGIDGLFAETHIDPKNALSDGANMLKPDELEHLVTDMLKIQNLF---------

>UniRef100_B2UW07

-----MKTSKTKTPKSVLIAGPCVIESLENLRSIAIKLQPLANNERLDFYFKASFDKANRTSLESYRGPG

LGKGLEMLQTIKDEFGYKILTDVHESYQASAAAKVADILQIPAFLCRQTDLIVEVSQTNAIINIKKGQFM

NPKDMQYSVLKALKTRTNGVWLCERGSSFGYGNLVVDMRSLKIMREF---APVIFDATHSVQMPGGANGK

SSGDSSFAPILARAAAAVGIDGLFAETHVDPKNALSDGANMLKPDELEHLVADMLKIQNLF---------

>UniRef100_P56060

-----MKTSKTKTPKSVLIAGPCVIESLENLRSIATKLQPLANNERLDFYFKASFDKANRTSLESYRGPG

LEKGLEMLQTIKEEFGYKILTDVHESYQASVAAKVADILQIPAFLCRQTDLIVEVSQTNAIVNIKKGQFM

NPKDMQYSVLKALKTRTNGVWLCERGSSFGYGNLVVDMRSLKIMREF---APVIFDATHSVQMPGGANGK

SSGDSSFAPILARAAAAVGIDGLFAETHVDPKNALSDGANMLKPDELEQLVTDMLKIQNLF---------

>KDSA_HELPH

---------KTKPPKSVLIAGPCVIESLENLRSIAIKLQPLANNERLDFYFKASFDKANRTSLESYRGPG

LEKGLEMLQIIKEEFGYKILTDVHESYQASVAAKVADILQIPAFLCRQTDLIVEVSQTNAIINIKKGQFM

NPKDMQYSVLKALKTRTNGVWLCERGSSFGYGNLVVDMRSLTIMREF---APVIFDATHSVQMPGGANGK

SSGDSSFAPILARAAAAVGIDGLFAETHVDPKNALSDGANMLKPDELERLVTDMLKIQNLF---------

>UniRef100_Q7VK53

---------------MILMSGPCVIESYEALSAVAQALKPLSEKAHIDFYFKASFDKANRTSLESYRGPG

LEKGLELLSEIKKQFGYKIITDIHESYQAKHIAKVADVIQIPAFLCRQTDLIVEVAKTERIVNIKKGQFM

NPSDMQHSVLKAIKTRTYGIWLTERGSTFGYGNLVVDMRSLVIMRSF---APVIFDATHSVQMPGAAGGK

SGGDSSFVPYLARAAAAVGVDGFFMETHLNPKEALSDGPNMVQTNALIPLIEQLYDIENLTQHKNGK---

>KDSA_WOLSU

---------------MILIAGPCVIESQEMLRKIASELQPLSEDARIDFYFKASFDKANRTSLESYRGPG

LEKGLEMLGRIKEEFGYKLLTDIHETQQVAKAAQVVDILQIPAFLCRQTDLIVEAAKSQSIVNIKKGQFM

NPADMSHSVLKAIKTRSLGIWLTERGSSFGYGNLVVDMRSLMIMRQF---APVIFDATHAVQMPGAAGGK

SGGDSRFVAPLSRAAAAVGVDGFFTETHVSPKEALSDGPNMITPQALKVLVHQLLALSEI----------

>KDSA_CAMJD

------------MKKMILIAGPCVIESKDLIFKVAKQLKNFNENPNIEFYFKSSFDKANRTSINSFRGPG

LEEGLKILQSVKDEFGMKILTDIHESNQANPVSEVADVLQIPAFLCRQTDLLVAAAKTKAKVNIKKGQFL

NPSDIKYSVKKVLQTRGNGVFVAERGASFGYGNLVVDMRSLVIMREF---APVIFDATHSVQMPGAAGGS

SGGKSEFVEPLARAAAAVGIDGFFFETHINPCEALCDGPNMLDLTRLKNCVNTLLEIQNII---------

>KDSA_CAMJJ

------------MKKMILIAGPCVIESKDLIFKVAEQLKNFNENPNIEFYFKSSFDKANRTSINSFRGPG

LEEGLKILQSVKDEFGMKILTDIHESNQANPVSEVADVLQIPAFLCRQTDLLVAAAKTKAKVNIKKGQFL

NPSDIKYSVKKALQTRGNGVFVAERGSSFGYGNLVVDMRSLVIMREF---APVIFDATHSVQMPGAAGGS

SGGKSEFVEPLARAAAAVGIDGFFFETHINPCEALCDGPNMLNLTRLKNCVNTLLEIQNIIKEN------

>KDSA_CAMJ8

------------MKKMILIAGPCVIESKDLIFKVAEQLKNFNENPNIEFYFKSSFDKANRTSINSFRGPG

LEEGLKILQSVKDEFGMKILTDIHESNQAAAVSEVADVLQIPAFLCRQTDLLVAAAKTKAKVNIKKGQFL

NPSDIKYSVKKVLQTRGNGVFVAERGASFGYGNLVVDMRSLVIMREF---APVIFDATHSVQMPGAAGGS

SGGKSEFVEPLARAAAAVGIDGFFFETHINPCEALCDGPNMLNLTRLKNCVNTLLEIQNIIKEN------

>KDSA_CAMJE

------------MKKMILIAGPCVIESKDLIFKVAEQLKNFNENPNIEFYFKSSFDKANRTSINSFRGPG

LEEGLKILQSVKDEFGMKILTDIHESNQANPVSEVADVLQIPAFLCRQTDLLVAAAKTKAKINIKKGQFL

NPSDIKYSVKKVLQTRGNGVFVAERGASFGYGNLVVDMRSLVIMREF---APVIFDATHSVQMPGAAGGS

SGGKSEFVEPLARAAAAVGIDGFFFETHINPCEALCDGPNMLNLTRLKNCVNTLLEIQNIIKEN------

>A7HZI5_CAMHC

---------------MIIIAGPCVIESKELVFAVAEKLREIANNKEIEFYFKSSFDKANRTSISSFRGPG

LKKGCEILNEVKEKFGYKILTDIHESHQAAPVSEVADVLQIPAFLCRQTDLLIAAAKTNAVVNIKKGQFL

APDQMKFSVKKILETRGNGVWLTERGSTFGYGNLVVDMRSLMIMREF---APVIFDATHSVQMPGGLNGK

SGGDSRFVPILARAAAATGCDGFFYETHINPCEALCDGPNMLNLDELYKTINETIKIKDVLKYE------

>A0RQK0_CAMFF

----------LGNIIMVLIAGPCVIESRDLVFKVAEKLSKFNEMGWIDFYFKSSFDKANRTSISSFRGPG

LEEGCKILSEVKKEFGFKILTDIHESYQATPVSQVADALQIPAFLCRQTDLLVAAAKTKAMVNIKKGQFL

SPDAMKYSVKKVLETRGNGVYLCERGSTFGYGNLVVDMRSLVIMKEF---APVIFDATHSVQMPSANGAT

SGGDSRFVPYLARAAAAVGVDGFFYETHINPCDALCDGANMLTLEALEKNINDIKNIKEVL---------

>A7GWZ3_CAMC5

---------------MILIAGPCVIESEKLVFDVAKRLVKFNEDARIDFYFKSSFDKANRTSISSFRGPG

LEKGCEILAKVKKEFGFKILTDIHESYQAAPMGEVADVLQIPAFLCRQTDLLVAAARTKAVVNIKKGQFL

AASAMKHSVKKVLETRGNGVWLTERGSTFGYGNLVVDMRNLVLMREF---APVIFDATHSVQMPSALGEK

SGGDAKFVPYLARAAAAVGVDGFFYETHINPCEALCDGPNMLNLDELDANINEIFRIQEALNFA------

>A7ZBS3_CAMC1

---------------MILIAGPCVIESKELVFEVAKRLVKFNENPKLDFYFKSSFDKANRTSISSFRGPG

LEKGCEILAEVKKEFGFKILTDIHESYQAQPVGEVADVLQIPAFLCRQTDLLVAAAKTKAVVNIKKGQFL

AASAMKHSVKKVLETRGNGVWLTERGSTFGYGNLVVDMRNLVMMREF---APVIFDATHSVQMPSALGEK

SGGDARFVPYLARAAASVGVDGFFYETHINPCEALCDGPNMLNLDELEKVVNDTLKIEEILNF-------

>Q30TJ9_SULDN

---------------MKLLAGPCVIESEENIFKIAKSLETYQNDKTIDFYFKSSFDKANRTSLDSFRGLG

IDEGLRILQKVKDDFGYKVVTDVHESCQVAQVAEVVDMLQIPAFLCRQTDLLVACAKTNREVNIKKGQFI

NPPDMRYSVAKVLKTRSHGVFLCERGSSFGYGNIIVDMRSLVIMREF---APTIFDATHSVQAPGALGGK

TGGDRTMVPYLSSAAAAVGVDGFFFETHFDPSIALSDGPNMIKLDELETIINKIKKIQEI----------

>A6QBK8_SULNB

---------------MILIAGPCVLESRENVMRIAESLGKYDDDCTKDFYFKASFDKANRTSLDSFRGPG

LEEGLKMLQEVKEQFGYKILTDVHDYTQPAAVAEVADVLQIPAFLSRQTDLLVAAAKTSAVVNIKKGQFL

APAAMEHSVAKVLKTRNYNVWLTERGSSFGYGNLVVDMRGLVTMRQF---APVIFDATHSVQMPASGGAS

SGGDSSFVPYLSRAAAAVGVDGFFFETHFDPSIALSDGPNMIELQKLDGLIEQIDAIRSIVE--------

>Q2IH82_ANADE

---------VGDGQPLLLIAGPCVMEDEAHGLRHARRVKELAAQHGVPVVFKASFDKANRSSGKSYRGPG

LEAGLAAFQAVKRETGLPCLTDVHETWQAEPAGRVVDVLQVPAFLCRQTDLVIACARHGRAVNVKKGQFL

APREMRHAIAKCREGGNENVFLTERGATFGYGNLVVDMRALVQMREL--GVPVCMDATHSVQMPGSGGDT

TAGDRQFVAPLARAAAAVGIDALFMEIHEDPAVAKSDGPNSLDFPTADRVLREVLAVRRALGQP------

>KDSA_CHLMU

---------MFPNNKMLLIAGPCVIED-NSVFETARRLKEIVAPYSVHWIFKSSYDKANRSSLQNYRGPG

LKLGLQTLAKIKETFDVEILTDVHSPDEAREAAKVCDIIQVPAFLCRQTDLLVTAGETQAIVNIKKGQFL

SPWEMQGPIDKVLSTGNNKIILTERGCSFGYNNLVSDMRSIEVLRRF--GFPVIFDGTHSIQLPGALQSQ

SGGQTEFIPVLTRSAIAAGVHGLFIETHPNPASALSDAASMLSLKDLERLLPSWVQLFTYIQEM------

>UniRef100_B0B8N1

---------MFPENKMLLIAGPCVIED-NSVFETARRLKEIVAPYSVHWIFKSSYDKANRSSVHNYRGPG

LRLGLQTLAKIKEELDVEILTDVHSPDEAREAAKVCDIIQVPAFLCRQTDLLVTAGETQAIVNIKKGQFL

SPWEMQGPIDKVLSTGNNKIILTERGCSFGYNNLVSDMRSIEVLRRF--GFPVVFDGTHSVQLPGALHSQ

SGGQTEFIPVLTRSAIAAGVQGLFIETHPNPSSALSDAASMLSLKDLERLLPAWVQLFTYIQEMDAVSV-

>KDSA_CHLTA

---------MFPENKMLLIAGPCVIED-NSVFETARRLKEIVAPYSVHWIFKSSYDKANRSSVHNYRGPG

LKLGLQTLAKIKEELDVEILTDVHSPDEAREAAKVCDIIQVPAFLCRQTDLLVTAGETQAIVNIKKGQFL

SPWEMQGPIDKVLSTGNNKIILTERGCSFGYNNLVSDMRSIEVLRRF--GFPVVFDGTHSVQLPGALHSQ

SGGQTEFIPVLTRSAIAAGVQGLFIETHPNPSSALSDAASMLSLKDLERLLPAWVQLFTYIQEM------

>UniRef100_Q9Z7I4

----------MFNNKMILIAGPCVIEGEDITLEIAGKLQSILAPYDIQWFFKSSYDKANRSSLNSFRGPG

LTEGLRILAKVKETFGVGILTDVHTPQDAYAAAEVCNILQVPAFLCRQTDLLVATAETGAIVNLKKGQFL

SPWDMEGPINKVLSTGNNKILLTERGCSFGYNNLVSDMRSIPVLSRS--GFPVIFDATHSVQLPGALSTE

SGGLTEFVPTLSRAALAAGAHGLFIETHTNPKIAKSDAASMLSLEEFAALLPTWDQLFTCVSSFDMVSA-

>UniRef100_Q5L789

----------MFSDKMILIAGPCVIEEEETTLEIAAKIQEIVAPYDIHWIFKSSYDKANRSSIHSYRGPG

LKEGLRILSKVKQTFGVEILTDVHSPEEARAAAEVCDILQIPAFLCRQTDLLVAAAETQAVINIKKGQFL

SPWDMQGPVDKVLSTGNSKIILTERGCSFGYNNLVSDMRAIAVLSKM--GFPVVFDGTHSVQLPGGLKTH

SGGQTEFIPTLTRAALAAGAHGLFIETHTNPAIAKSDAASMLSLKAFEVLLPVWNQLYQCVRSFEMASV-

>KDSA_CHLPS

-----------FSDKMILIAGPCVIEEEETTLEIASKIQEIVAPYDIHWIFKSSYDKANRSSINSYRGPG

LQEGLRILSKVKQTFGVEILTDVHSPEEARAAAEVCDILQIPAFLCRQTDLLVAAAETHAVINIKKGQFL

SPWDMQGPVDKVLSTGNSKIILTERGCSFGYNNLVSDMRSIAVLSKM--GFPVVFDGTHSVQLPGGLKTH

SGGQTEFIPTLTRAALAAGAHGLFIETHMNPAIAKSDAASMLSLKTFEALLPIWNQLYQCVRSF------

>UniRef100_Q252N1

----------MFSDKMILIAGPCVIEGEEITLEIASKIHELVSPYDIHWIFKSSYDKANRSSINSYRGPG

LSEGLRILSKVKETLGVEILTDVHSPEEARAAAEVCDILQIPAFLCRQTDLLVAAAETNAIINIKKGQFL

SPWDMQGPVDKVLSTGNNKIILTERGCSFGYNNLVSDMRSIPVLSSM--GFPVVFDGTHSVQLPGGLKTQ

SGGQTEFIPTLTRAALAAGAHGLFIETHSHPAIAKSDAASMLPLKTFEALLPLWDQLYTCVRSFEMASV-

>UniRef100_Q824W7

----------MFSDKMILIAGPCVIEEEETTLEIASRIQELVTPYDIHWIFKSSYDKANRSSINSYRGPG

LNEGLRILSKVKDTLGVEILTDVHSPEEARAAAKVCDILQIPAFLCRQTDLLVAAAETNAVINIKKGQFL

SPWDMQGPVDKVLSTGNNKIILTERGCSFGYNNLVSDMRSIPVLSGM--GFPVVFDGTHSVQLPGGLKTH

SGGQTEFIPTLTRAALAAGAHGLFIETHANPAIAKSDAASMLSLKTFEALLPLWDQLYTCVRSFEMASV-

>UniRef100_Q1IKD0

EPFKVKDVEIGGPK-LFLIAGPCVIESEAHAMKMAEAISGVCKAMKIPYIFKASYDKANRTSLSSFRGPG

LHEGLRILAKVADEVNVPVLTDVHDTEQATAAGDIVDVLQIPAFLCRQTDLLVAAAKTGKVVNVKKGQFV

APNDMQYAVTKVRESGNQRVCLTERGASFGYNNLVVDMRALPIMRQF---APVIFDATHSVQLPSAGGHA

SGGQPEFIPVLSRAAVAAGVDGVFMEVHDDPPHAKSDGANALDLKLLRGVLTSLLRIREAVTPPAAS---

>KDSA_AQUAE

------------MEKFLVIAGPCAIESEELLLKVGEEIKRLSEKFEVEFVFKSSFDKANRSSIHSFRGHG

LEYGVKALRKVKEEFGLKITTDIHESWQAEPVAEVADIIQIPAFLCRQTDLLLAAAKTGRAVNVKKGQFL

APWDTKNVVEKLKFGGAKEIYLTERGTTFGYNNLVVDFRSLPIMKQW---AKVIYDATHSVQLPGGLGDK

SGGMREFIFPLIRAAVAVGCDGVFMETHPEPEKALSDASTQLPLSQLEGIIEAILEIREVASKYYETIPV

>Q2GAF0_NOVAD

---------MKRRRRPFLISGPCVIEGLDLCLGMAEALARLADKLDIAIVFKASFDKANRTSAASFRGPG

LERGLRILESVKRESGLPVLTDVHEAGHCAEVAEVVDFLQTPAFLARQTDLIEAAAATGRPVNLKKAQFM

APADMLAVVDKARMASQGSLAVCERGTAFGYGNLVVDMRSLDELRAT--GCPVIFDATHSVQLPGALGSC

SGGQRRHVPTLARAAVAVGVDGLFLECHPDPDRALSDGPNSLALADVGPLMRVLVEIDAARRAE------

>UniRef100_A0L7X4

RTITVDTVRFGNHLPMTLIAGPCVIEGLEFALRTAEALKQICEGVGVELVYKSSFDKANRTSEGSFRGPG

MEAGLRILERVRREVGVPVITDVHEADQCVAVAEVVDMLQTPAFLCRQTDFIQAAARPGKPVNIKKGQFL

APQDMARVAAKAAATGNENILLCERGFSFGYQNLVVDMRGLSIMAQS--GYPVIFDATHSVQQPGALGGA

SGGDRRFVSDLARAAVAVGVAGVFMEVHPDPDHAPCDGPNMLPMAHLSPLLDQLKALDRIRKEAMALESG

>Q2A271_FRATH

---------VGNGKPFFLMSGPCVIESEQMAMDTAGYLAEVTKDLGINFVYKSSFDKANRSSINSFRGLG

VDKGLEILAKVKKTYNVPVVTDVHEDTPFAEVAEVVDVLQIPAFLCRQTNFILEVCKQGKPVNIKKGQFL

APWDMQHVVTKAKSTGNEQIMVCERGVSFGYNNLVSDMRSLEIMKAT--GCPVVFDATHSVQLPGGQGSS

SGGQREFVPVLSKAAMAVGIDGLFMETHPNPDEAKSDGPNSFPMYKIKEFLSLLKELDHLVKSQ------

>A0Q5J1_FRATN

---------VGNGKPFFLMSGPCVIESEQMAMDTAGYLAEVTRDLGINFVYKSSFDKANRSSINSFRGLG

VDKGLEILAKVKKTYNVPVVTDVHEDTPFAEVAEVVDVLQTPAFLCRQTNFILEVCKQGKPVNIKKGQFL

APWDMQHVVTKAKSTGNEQIMVCERGVSFGYNNLVSDMRSLEIMKAT--GCPVVFDATHSVQLPGGQGSS

SGGQREFVPVLSKAAMAVGIDGLFMETHPNPDEAKSDGPNSFPMYKIKEFLSLLKELDHLVKSQ------

>Q5NGX5_FRATT

---------VGNGKPFFLMSGPCVIESEQMAMDTAGYLAEVTKDLGINFVYKSSFDKANRSSINSFRGLG

VDKGLEILAKVKKTYNVPVVTDVHEDTPFAEVAEVVDVLQTPAFLCRQTNFILEVCKQGKPVNIKKGQFL

APWDMQHVVTKAKSTGNEQIMVCERGVSFGYNNLVSDMRSLEIMKAT--GCPVVFDATHSVQLPGGQGSS

SGGQREFVPVLSKAAMAVGIDGLFMETHPNPDEAKSDGPNSFPMYKIKEFLSLLKELDHLVKSQ------

>A5EW23_DICNV

---------VDNDRPFFLIAGPCVIESEYLALHTAEVLKTITDELKIPFIYKSSFDKANRSSGASFRGLG

LAEGLRILAKVKETFAVPILTDVHEYTPLDEVASVVDVLQTPAFLCRQTDFITRVCAQGKPVNIKKGQFL

APWDMKHVVAKAKATGNEQIMLCERGASFGYNNLVADMRSLAVMKTF--SAPVVFDATHSVQLPGGNGAS

SGGQREFVPVLARAAIAVGVAGIFMETHPEPEKALSDGANSIPLAQMRHLLQQLRALDTLVKTN------

>A9N9V0_COXBR

---------IGLNNPLFLIAGPCVIESEALVMDVAGELKSITQQLDMPFIFKASFDKANRSSHLSYRGPG

IEKGLTILEKVKKTLEVPIITDVHEDTPLQEVAAVVDVLQTPAFLCRQSNFIRSVAACGKPVNIKKGQFL

SPWEMKQVVAKAWATGNKKIMVCERGYSFGYNNLISDMRALAILRET--ACPVIFDATHSVQLPGGHGTN

SGGQREFVPVLARAATAAGIAGIFMETHPDPDRALSDGPNSWPLAKMQPLLETLKELDKIVKNA------

>KDSA_COXBU

---------IGLNNPLFLIAGPCVIESEALVMDVAGELKSITQQLDMPFIFKASFDKANRSSHLSYRGPG

IEKGLTILEKVKKTLEVPIITDVHEDTPLQEVAAVVDVLQTPAFLCRQSNFIRSVAACGKPVNIKKGQFL

APWEMKQVVAKAWATGNKKIMVCERGYSFGYNNLISDMRALAILRET--ACPVIFDATHSVQLPGGHGTN

SGGQREFVPVLARAATAAGIAGIFMETHPDPDRALSDGPNSWPLAKMQPLLETLKELDKVVKNA------

>UniRef100_A9KDH6

MLMQIADFEIGLNNPLFLIAGPCVIESEALVMDVAGELKSITQQLDMPFIFKASFDKANRSSHLSYRGPG

IEKGLTILEKVKKTLEVPIITDVHEDTPLQEVAAVVDVLQTPAFLCRQSNFIRSVAACGKPVNIKKGQFL

APWEMKQVVAKAWATGNKKIMVCERGYSFGYNNLISDMRALAILRET--ACPVIFDATHSVQLPGGHGTS

SGGQRKFVPVLARAATAAGIAGIFMETHPDPDRALSDGPNSWPLAKMQPLLETLKELDKVVKNAGFLEQS

>UniRef100_B6J4X2

MLMQIADFEIGLNNPLFLIAGPCVIESEALVMDVAGELKSITQQLDMPFIFKASFDKANRSSHLSYRGPG

IEKGLTILEKVKKTLEVPIITDVHEDTPLQEVAAVVDVLQTPAFLCRQSNFIRSVAACGKPVNIKKGQFL

APWEMKQVVAKAWATGNKKIMVCERGYSFGYNNLISDMRALAILRET--ACPVIFDATHSVQLPGGHGTS

SGGQREFVPVLARAATAAGIAGIFMETHPDPDRALSDGPNSWPLAKMQPLLETLKELDKVVKNAGFLEQS

>UniRef100_B7J6R3

--MRLCGFEAGLQHPFFLMAGPCAIESESLALRTAEDLRDICARLGIPFIYKSSYDKANRSSGQSFRGPG

MDEGLRILEKVRREVGVPVVTDVHEKEDVSAVAEVVDVLQTPAFLCRQTDFIQAVAAAGKPVNIKKGQFL

APWDMLHVASKAKATGNEQIMVCERGASFGYNNLVSDMRSLAVMRQT--GCPVVFDATHSVQLPGGQGDR

SGGQREFIPVLARAAVAAGVSGLFMETHPNPADALSDGPNAWPLGRMEDLLRILQHIDHVVKNQDFPENY

>UniRef100_Q9PDU0

--MKLCGFEVGLNQPLFLIAGPCVIESLQLQLDTAGVLKEITSKLGLNFIFKSSFDKANRTSGSSFRGPG

LEEGLKVLEAVKTQIGVPVLTDVHEYTPIDEVATVVDVLQTPAFLVRQTDFIRNVCAVGKPVNIKKGQFL

SPWDMKPVVEKAKSTGNSQILVCERGASFGYNNLVSDMRSLAVMRET--GCPVVFDATHSVQLPGAQGGR

SGGQREFVPVLARAAVAVGISGLFAETHPDPPNALSDGPNAWPLHTMAMLLETLVELDAVTKKRGFLEQD

>KDSA_XYLFT

---------VGLNQPLFLIAGPCVIESLQLQLDTAGVLKEITSKLGLNFIFKSSFDKANRTSGSSFRGPG

LEEGLKVLEAVKTQIGVPVLTDVHEYTPIDEVATVVDVLQTPAFLVRQTDFIRNVCAVGKPVNIKKGQFL

SPWDMKPVVEKAKSTGNSQILVCERGASFGYNNLVSDMRSLAVMRET--GCPVVFDATHSVQLPGAQGGR

SGGQREFVPVLARAAVAVGISGLFAETHPDPPNALSDGPNAWPLRTMAMLLETLVELDAVTKKR------

>UniRef100_B2FK85

--MKLCGFEVGLDQPLFLIAGPCVIESMQLQLDTAGKLKEVTDKLGVNFIFKSSFDKANRTSGTAFRGPG

MEEGLKVLAEVRKQIGVPVLTDVHEYTPMDEVASVVDVLQTPAFLVRQTDFIRKVCSAGKPVNIKKGQFL

APWDMKPVVEKAKATGNEQIMVCERGASFGYNNLVSDMRSLAVMRDT--GCPVVFDATHSVQLPGGQGTS

SGGQREHVPVLARAAVAVGISGLFAETHPDPSKALSDGPNAWPLDQMEALLETLMELDAVTKKHGFSRFA

>UniRef100_B4SR83

--MKLCGFEVGLDQPLFLIAGPCVIESMQLQLDTAGKLKEVTDRLGVNFIFKSSFDKANRTSGTAFRGPG

MEEGLKVLAEVKKQIGVPVLTDVHEYTPMDEVASVVDVLQTPAFLVRQTDFIRKVCSAGKPVNIKKGQFL

APWDMKPVVEKAKATGNEQIMVCERGASFGYNNLVSDMRSLAVMRDT--GCPVVFDATHSVQLPGGQGTS

SGGQREHVPVLARAAVAVGISGLFAETHPDPSKALSDGPNAWPLDQMEALLETLMELDAVTKKHGFSRFA

>UniRef100_Q4UTP0

--MKLCDFEVGLDQPLFLIAGPCVIESMQLQLDVAGKLKEITGKLGINFIFKSSFDKANRTSGTSFRGPG

LEEGLKVLDAVKKQIGVPVLTDVHEYTPMNEVAAVVDVLQTPAFLVRQTDFIKNVCAAGKPVNIKKGQFL

APWDMKPVVDKAKSTGNEQIMVCERGASFGYNNLVSDMRSLSVMRDT--GCPVVFDATHSVQLPGGQGSS

SGGQREFVPVLARAAVAVGISGLFAETHPDPSKALSDGPNAWPLDRMEELLETLMELDAVTKKHGFARFA

>KDSA_XANAC

---------VGLDQPLFLIAGPCVIESMQLQLDVAGKLKEITGKLGVNFIFKSSFDKANRTSGTSFRGPG

LEEGLKVLDAVKKQIGVPVLTDVHEYTPMNEVAAVVDVLQTPAFLVRQTDFIKNVCAAGKPVNIKKGQFL

APWDMKPVVDKAKSTGNEQIMVCERGASFGYNNLVSDMRSLSVMRDT--GCPVVFDATHSVQLPGGQGSS

SGGQREFVPVLARAAVAVGISGLFAETHPDPSKALSDGPNAWPLDRMEELLETLMELDTVTKKH------

>KDSA_XANC5

---------VGLDQPLFLIAGPCVIESMQLQLDVAGRLKEITGKLGVNFIFKSSFDKANRTSGTSFRGPG

LEEGLKVLDAVKKQIGVPVLTDVHEYTPMNEVAAVVDVLQTPAFLVRQTDFIKNVCAAGKPVNIKKGQFL

APWDMKPVVDKAKSTGNEQIMVCERGASFGYNNLVSDMRSLSVMRDT--GCPVVFDATHSVQLPGGQGSS

SGGQREFVPVLARAAVAVGISGLFAETHPDPSKALSDGPNAWPLDRMEELLETLMELDAVTKKH------

>Q5GYK1_XANOR

---------VGLDQPLFLIAGPCVIESMQLQLDVAGKLKEITGKLGVNFIFKSSFDKANRTSGTSFRGPG

LEEGLKVLDAVKRQIGVPVLTDVHEYTPMNEVAAVVDVLQTPAFLVRQTDFIKNVCAAGKPVNIKKGQFL

APWDMKPVVDKAKSTGNAQIMVCERGASFGYNNLVSDMRSLSVMRDT--GCPVVFDATHSVQLPGGQGSS

SGGQREFVPVLARAAVAVGISGLFAETHPDPSKALSDGPNAWPLDRMEELLETLMELDAVTKKH------

>A1AX91_RUTMC

---------VSIDQPFFLIAGLCVIESEALVMETATYLAKVTNNLGVNFIYKSSYDKANRTSVNSFRGLG

LEQGLRILQKVKDEVGVPVLTDVHEDTPLDEVASVVDMMQTPAFLVRQTNFIQNVCKQGLPVNIKKGQFQ

APWDMNNVVEKAHATGNQQITVCDRGTSFGYNTLISDMRGLVSMRNT--NCPVVFDATHSVQQPGGQGTT

SGGQREMVPVLARAAVAVGISGIFMETHPNPTNAKSDGPNSIPINKMDELLKILQELDNVTKKN------

>A5CW13_VESOH

---------VGINHPFFLIAGPCVIESEALALETATYLKRVTEDLGINFIYKSSYDKANRTSTNSFRGLG

VKEGLRILQKVKDEVGVPVLTDVHEDTPLSEVASVVDMMQTPAFLVRQTNFIQNVCKQGIPVNIKKGQFQ

APWDIGNVVNKAYETGNRLITICERGTSFGYNTLISDMRGLSTMRST--GCPIVFDATHSVQQPGGNGLT

SGGQREMVPVVARAAIGVGVSGFFMETHPNPNEAFSDGPNMVPIDKIDELLKTLQELDAVTKKH------

>UniRef100_Q5WXA7

--MRLCGFEAGLDKPLFLIAGPCVIESEELALETAGYLKEMCSQLNIPFIYKSSFDKANRSSISSYRGPG

FEKGLSILEKVKSQIGVPVLTDVHEDTPLFEVSSVVDVLQTPAFLCRQTNFIQKVAAMNKPVNIKKGQFL

APWEMKHVIAKAKAQGNEQIMACERGVSFGYNNLVSDMRSLVIMRET--GCPVVYDATHSVQLPGGNNGV

SGGQREFIPALARAAVAVGISGLFMETHPDPDNALSDGPNSWPLDKMKQLLESLKAADEVYKKYSTDF--

>UniRef100_Q5X5Y5

--MRLCGFEAGLDKPLFLIAGPCVIESEELALETAGYLKEMCSQLNIPFIYKSSFDKANRSSISSYRGPG

FEKGLSILEKVKSQIGVPVLTDVHEDTPLFEVSSVVDVLQTPAFLCRQTNFIQKVAAMNKPVNIKKGQFL

APWEMKHVIAKAKAQGNEQIMACERGVSFGYNNLVSDMRSLVIMRET--GCPVVYDATHSVQLPGGNNGV

SGGQREFIPALARAAVAVGISGLFMETHPDPDKALSDGPNSWPLDKMKQLLESLKAADEVYKKYSTDF--

>UniRef100_Q604M5

RPFELCGFPVGLEHPLFLIAGPCVIETEQLALDTAGALKEITDGLGIPFIYKSSFDKANRSSHASYRGPG

MEEGLRILAEVKRQIGVPVLTDVHEDTPLQEVASVVDVLQTPAFLCRQTNFIQNVANTGKPVNLKKGQFL

APWDMKHVAAKALATGNRHIMVCERGVSFGYNNLVSDMRSLSIMRET--GCPVVYDATHSVQLPGGQGTA

SGGQREFVPALARAAVAVGISGLFMETHPDPDRALSDGPNSWPLDRMKALLELLSTLDRTVKASPLL---

>Q31G67_THICR

---------VGIDQPLFLIAGPCVIESEQLAIETAGQLKELTDALGIPFIYKSSYDKANRSSTKSFRGLG

VEEGLRILQKVKDEIGVPVLTDVHEDTPLDEVASVVDVMQTPAFLVRQTNFIQNVCRQGLPVNIKKGQFQ

APWDMDQVVAKAREVGNDQIMVCDRGTSFGYNTLISDMRGLASMRQT--GCPVVFDATHSVQQPGGQGTT

SGGQREMVPVLARAAIAAGISGVFMETHPDPENALSDGPNMWPISRLKPLLETMKELDEVVKRH------

>UniRef100_Q3JCT2

--MELCGFEVGLERALFLIAGPCVIESEQLALDTAGRLKEMTERLGIPFIYKSSFDKANRTAVESFRGLG

MEAGLGILEKVKATLGVPVLTDVHEDTPLQEVAAVVDVLQTPAFLCRQTNFIQNVARQGRPVNIKKGQFL

APWDMKHVVEKARAVGNQQIMVCERGVSFGYNTLVSDMRSLAVMRDT--GCPVVFDATHSVQQPGGKGGC

SGGQREWVPVLARAAVAAGVAGVFMETHPDPERALSDGPNAWPLGAMEKLLETLSTIDKTVKTDGFLEAE

>KDSA_THIDA

---------AGLDQPLFLISGPCVIESEQLAMDTAGQLKEMCAGLGIPFIYKSSFDKANRSSTKSFRGLG

LEEGLRILADVKAKIGVPVLTDVHEDTPLDEVAAVVDVLQTPAFLCRQTNFIQNVARAGRPVNIKKGQFL

APWDMQNVVDKAREVGNDQIMVCERGVSFGYNTLVSDMRGLAIMRGT--GCPVVFDATHSVQQPGGQGAT

SGGQREFVPVLARAAVASGVAGVFAETHPDPACALSDGPNAWPLGMMRELLETLKEIDALVKQR------

>UniRef100_Q0A7K3

--MDLCGFRVGLDRPLFLIAGPCVVESEQLALDTAGALAEMTGELGIPFIYKSSFDKANRSSHESYRGPG

LEAGLRVLEQVRRQIGVPVITDVHEDTPLVEVASVVDVLQTPAFLCRQTNFIQNVARQGRPVNIKKGQFL

APWDMRHVVAKAREAGNQQIMVCERGVSFGYNNLVSDMRALAVMRET--GAPVVFDATHSVQLPGGQGSA

SGGQREFVPVLARAAVAAGVAGLFMETHPDPDQALSDGPNAWPLERMRELLETLMELDQVVKGRGFTEQG

>A1WWZ3_HALHL

---------AGIEQPLFLIAGPCVVESEALALESALELRDLCAELGVPLIYKSSFDKANRSSAQSYRGPG

MEAGLRVLERVRREVGVPVVTDVHEDTPLDEVASVVDVLQTPAFLCRQTNFIQRVAACGLPVNLKKGQFL

APWDMTHVVDKARATGNEHILVCERGVSFGYNNLISDMRALAVMRQT--GAPVVFDATHSVQLPGGQGHA

SGGQREFVPVLARAAVAAGVSGLFMETHPDPDAALSDGPNAWPLDRMRELLATLIALDRVVKEQ------

>UniRef100_Q1H010

--MQLCGFEVGIDQPLFLIAGPCVIESQEMAIETAGQLKEITTALGINFIYKSSYDKANRSSNKTFRGFG

MDEGLKILDEVRRQVGVPILTDVHTEAQVPHVAAVVDVLQTPAFLCRQTDFITACAQSGKPVNIKKGQFL

APGDMKQVVQKAKEANGDTIMVCERGASFGYNTLISDMRGLAIMRET--NCPVVFDATHSVQQPGGQGDK

SGGQREFVPVLARAAVASGVAGVFMETHPDPAQALSDGPNAWPLSKMKALLEVLVDLDRVVKKAGFIEHT

>UniRef100_A1W4R2

--MQLCGFDVGLDQRFFLIAGTCSIEGLEMSLDVAGQLKEACAPLGIPLIYKGSFDKANRSSGTSKRGVG

LDAGLKILDEVRRQLQLPILTDVHDTSHVAEVASVVDVLQTPAFLCRQTDFIRAVAQSGKPVNIKKGQFL

APWDMKNVIDKARAAARDRFLACERGVSFGYNNLVADMTSLAEMRNS--GAPVVFDVTHSVQKPGGLGAV

SGGARDMVPVLARAGVAVGVAGLFMETHPKPAEAWSDGPNAVPLKHMRALLETLVALDDVTKKNGFLENN

>UniRef100_B1Y4K5

--MKLCGFDVGIDQPFFLIAGTCSIEGLQMSLDVAGLLKEACAGLGIPLIYKGSFDKANRSSGTTNRGLG

LEAGLKILDEVRRQTGLPVLTDVHDESQVAEVASVVDVLQTPAFLCRQTDFIRAVAMSGKPVNIKKGQFL

APWDMKNVIDKARAAARDRFLACERGVSFGYNNLVADMTSLAEMRNT--GAPVVFDVTHSVQKPGGLGGS

SGGAREMVPVLARAGVAVGVAGLFMETHPDPAKAFSDGPNAVPLKHMKTLLEQLVALDRVVKTRPLLEND

>KDSA_DECAR

---------AGLDQPFFLIAGPCTAESEQLCLDVAGHMKEVCARLGINYIFKASYDKANRSSGKSVRGLG

LEEGLRIFSEVQRQIGVPVLTDVHTIEDIPAVAAVVDVLQTPAFLCRQTDFIHAVATCGKPVNIKKGQFL

APGDMKNVVDKAREVNNDNIMVCERGASFGYNNLVSDMRSLAIMRET--GCPVVFDATHSVQLPGGQGTT

SGGQREFVPVLARAAIAVGISGLFMETHPCPEKAWSDGPNSWPLDRMESLLATLVALDKAVKTA------

>UniRef100_A9IIP6

--MKACGFDIGLEHPFFLIAGPCVIESRELAFDTAGRLKEITSRLGIPFIYKSSFDKANRSSGKSFRGLG

IDEGLKILADVRDQVGVPVLTDVHETEQVEPVAAVVDMLQTPAFLCRQTDFIRACAASLKPVNIKKGQFL

APHDMVQVAQKARDAAISNILVCERGASFGYNNLVSDMRSLAIMRET--GCPVVFDATHSVQLPGGQGTS

SGGQREFVPVLARAAVAVGVSGLFMETHPNPACALSDGPNAVPLDLMPALLESLVELDRVTKRNGFVENQ

>UniRef100_Q7W5N7

-MMKACGFDIGLDHPFFLIAGPCVIESRELAFETAGRLKEITGKLGVPFIYKSSFDKANRSSGKSFRGPG

MDEGLKILADVRAQLDVPVLTDVHDIDQVAPVAAVVDMLQTPAFLCRQTDFIRACAATLKPVNIKKGQFL

APHDMLQVARKARDAALNNILVCERGASFGYNNLVSDMRSLAIMRET--DCPVVFDATHSVQLPGGQGAS

SGGQREFVPVLARAAVAVGVAGLFMETHPNPACAMSDGPNAVPLDRMAELLESLVALDRVTKRSGFLENQ

>UniRef100_Q7WD73

-MMKACGFDIGLDHPFFLIAGPCVIESRELAFETAGRLKEITGKLGVSFIYKSSFDKANRSSGKSFRGPG

MDEGLKILADVRAQLDVPVLTDVHDIDQVAPVAAVVDMLQTPAFLCRQTDFIRACAATLKPVNIKKGQFL

APHDMLQVARKARDAALNNILVCERGASFGYNNLVSDMRSLAIMRET--DCPVVFDATHSVQLPGGQGAS

SGGQREFVPVLARAAVAVGVAGLFMETHPNPACAMSDGPNAVPLDRMAELLESLVALDRVTKRSGFLENQ

>UniRef100_A1K7F7

--MNLCGFEVGLDKPVFLIAGPCVIESREMAFETAGALKDICAEVGIPFIYKSSYDKANRSSGKSYRGMG

MEKGLDILADVKKQLGVPVLTDVHSIEEIPAVAAVVDVLQTPAFLCRQTDFIHAVASCGRPVNIKKGQFL

APGDMKNVVDKAREANGDNIMVCERGASFGYNNLVSDMRSLAIMRET--GCPVVFDATHSVQLPGGQGTA

SGGQREFVPVLARAAVAVGVAGLFMETHPNPAQALSDGPNAWPLPRMKALLSTLKAIDELVKSRGLLETE

>UniRef100_Q5NZ70

--MKLCGFEAGLDKPFFLIAGPCVIESRDMAFETAGALKEICVELGIPFIYKSSYDKANRSSGKSYRGMG

MEKGLEILADVKKQLGVPVLTDVHAIDEIPAVAAAVDVLQTPAFLCRQTDFIHAVAASGRPVNIKKGQFL

APGDMKNVVDKAREANGDTIMVCERGASFGYNNLVSDMRSLAIMRET--GCPVVFDATHSVQLPGGQGTA

SGGQREFVPVLARAAVAVGIAGLFMESHPDPAKALSDGPNAWPLPKMKALLATLKEIDALVKAHGFMEMA

>A4SXE8_POLSQ

---------VGLDHRFFLIAGPCVIESEQSAIDIAGELKEITSALKIPFIYKSSFDKANRSSGTSFRGLG

MEKGLEILAKVKKEVGVPVLTDVHDIGEIAAVAKVVDVLQTPAFLCRQTDFIRACAQSGKPVNFKKGQFL

SPHEMLNVIEKARGAAADQFMVCERGASFGYNNLVSDMRSLAILRES--KAPVVFDATHSVQLPGGQGNS

SGGQREFVPVLARAAVAVGISGLFMETHPDPAKALSDGPNAVPLNRMKELLQSLVDIDSVVKAN------

>Q2SXC6_BURTA

---------VGLDKPFFLIAGTCVVESEQMTIDTAGRLKEICAKLGVPFIYKSSYDKANRSSGKSFRGLG

MDEGLRILAEVKRQLNVPVLTDVHEIDEIAPVAAVVDVLQTPAFLCRQTDFIRACAQSGKPVNIKKGQFL

APHDMKNVIDKARDAARDRFMACERGVSFGYNNLVSDMRSLAIMRET--GAPVVFDATHSVQLPGGQGTS

SGGQREFVPVLARAAVATGVAGLFMETHPNPAEAKSDGPNAVPLGRMAALLETLVTLDQAVKRV------

>UniRef100_A3ML78

--MNLAGFEVGLDKPFFLIAGTCVVESEQMTIDTAGRLKEICATLGVPFIYKSSYDKANRSSGKSFRGLG

MDEGLRILAEVKRQLNVPVLTDVHEIDEIAPVAAVVDVLQTPAFLCRQTDFIRACAQSGKPVNIKKGQFL

APHDMKNVIDKARDAARDRFMACERGVSFGYNNLVSDMRSLAIMRET--GAPVVFDATHSVQLPGGQGTS

SGGQREFVPVLARAALATGVAGLFMETHPNPAEAKSDGPNAVPLGRMAALLETLVTLDRAVKRVPFLEND

>A3NB83_BURP6

---------VGLDKPFFLIAGTCVVESEQMTIDTAGRLQEICATLGVPFIYKSSYDKANRSSGKSFRGLG

MDEGLRILAEVKRQLNVPVLTDVHEIDEIAPVAAVVDVLQTPAFLCRQTDFIRACAQSGKPVNIKKGQFL

APHDMKNVIDKARDAARDRFMACERGVSFGYNNLVSDMRSLAIMRET--GAPVVFDATHSVQLPGGQGTS

SGGQREFVPVLARAALATGVAGLFMETHPNPAEAKSDGPNAVPLGRMAALLETLVTLDRAVKRV------

>UniRef100_B2JIX1

--MKLGDFEIGLDKPFFLIAGTCVVESEQMTIDTAGKLKEICAKLKIPFIYKSSYDKANRSSGKSFRGLG

MDEGLRILSEVKRQLGLHVLTDVHSEHEIEPVASVVDVLQTPAFLCRQTDFIHACARSGKPVNIKKGQFL

APHDMKNVIDKARDAARDRFMACERGVSFGYNNLVSDMRSLAIMRET--GAPVVFDATHSVQLPGGQGTS

SGGQREFVPVLARAAVATGIAGLFMETHPNPAEAKSDGPNAVPLHRMADLLETLMTLDQAVKRTPFLEND

>KDSA_BURXL

---------IGLDRPFFLIAGTCVVESEQMTIDTAGRLKEICAKLNIPFIYKSSYDKANRSSGKSFRGLG

MDEGLRILSEVKRQLGLPVLTDVHAEHEIEQVASVVDVLQTPAFLCRQTDFIHACARSGKPVNIKKGQFL

APHDMKNVIDKARDAARDRFMACERGVSFGYNNLVSDMRSLAIMRET--NAPVVFDATHSVQLPGGQGTS

SGGQREFVPVLARAAVAVGVAGLFMETHPNPAQAKSDGPNAVPLHRMADLLETLVTLDRAVKRA------

>Q0BDS2_BURCM

---------VGLDQPFFLIAGTCVVESEQMTIDTAGRLKEICEKLNVPFIYKSSYDKANRSSGKSFRGLG

MDEGLRILGEVKRQLGLPVLTDVHSIDEIEQVASVVDVLQTPAFLCRQTDFIHACARSGKPVNIKKGQFL

APHDMKNVIDKAREAARDRFMACERGVSFGYNNLVSDMRSLAIMRET--SAPVVFDATHSVQLPGGQGTS

SGGQREFVPVLARAAVATGVAGLFMETHPNPAEAKSDGPNAVPLNRMGALLETLVTLDQAVKRH------

>KDSA_BURVG

---------VGLDQPFFLIAGTCVVESEQMTIDTAGRLKEICAKLNVPFIYKSSYDKANRSSGKSFRGLG

MDEGLRILGEVKRQLGLPVLTDVHSIDEIEQVASVVDVLQTPAFLCRQTDFIHACARSGKPVNIKKGQFL

APHDMKNVIDKARDAARDRFMACERGVSFGYNNLVSDMRSLAIMRET--NAPVVFDATHSVQLPGGQGTS

SGGQREFVPVLARAAVATGVAGLFMETHPNPAEAKSDGPNAVPLHRMGALLETLVTLDQAVKRN------

>KDSA_BURCA

---------VGLDQPFFLIAGTCVVESEQMTIDTAGRLKEICEKLNVPFIYKSSYDKANRSSGKSFRGLG

MDEGLRILGEVKRQLGLPVLTDVHSIDEIEQVASVVDVLQTPAFLCRQTDFIHACARSGKPVNIKKGQFL

APHDMKNVIDKARDAARDRFMACERGVSFGYNNLVSDMRSLAIMRET--NAPVVFDATHSVQLPGGQGTS

SGGQREFVPVLARAAVATGVAGLFMETHPNPAEAKSDGPNAVPLNRMGALLETLVTLDQAVKRN------

>UniRef100_B1JUY7

--MKLCDFEVGLDQPFFLIAGTCVVESEQMTIDTAGRLKEICEKLNVPFIYKSSYDKANRSSGKSFRGLG

MDEGLRILGEVKRQLGLPVLTDVHSIDEIEQVASVVDVLQTPAFLCRQTDFIHACARSGKPVNIKKGQFL

APHDMKNVIDKARDAARDRFMACERGVSFGYNNLVSDMRSLAIMRET--NAPVVFDATHSVQLPGGQGTS

SGGQREFVPVLARAAVATGVAGLFMETHPNPAEAKSDGPNAVPLNRMGALLETLVTLDQAVKRNPFLEND

>UniRef100_B4EDA2

--MKLCDFEVGLDQPFFLIAGTCVVESEQMTIDTAGRLKEICEKLNVPFIYKSSYDKANRSSGKSFRGLG

MDEGLRILSEVKRQLGLPVLTDVHSIDEIEQVASVVDVLQTPAFLCRQTDFIHACARSGKPVNIKKGQFL

APHDMKNVIDKARDAARDRFMACERGVSFGYNNLVSDMRSLAIMRET--NAPVVFDATHSVQLPGGQGTS

SGGQREFVPVLARAAVATGVAGLFMETHPNPAEAKSDGPNAVPLNRMGALLETLVTLDQAVKRNPFLEND

>UniRef100_A9AGW1

--MKLCDFEVGLDKPFFLIAGTCVVESEQMTIDTAGRLKEICAKLNIPFIYKSSYDKANRSSGKSFRGLG

MDEGLRILSEVKRQLGLPVLTDVHSIDEIEQVASVVDVLQTPAFLCRQTDFIHACARSGKPVNIKKGQFL

APHDMKNVIDKARDAARDRFMACERGVSFGYNNLVSDMRSLAIMRET--NAPVVFDATHSVQLPGGQGTS

SGGQREFVPVLARAAVATGVAGLFMETHPNPAEAKSDGPNAVPLNRMSALLETLVTLDQAVKRGPFLEND

>Q39EV8_BURS3

---------VGLDKPFFLIAGTCVVESEQMTIDTAGRLKEICAKLNIPFIYKSSYDKANRSSGKSFRGLG

MDEGLRILSEVKRQLGLPVLTDVHSIDEIEQVASVVDVLQTPAFLCRQTDFIHACARSGKPVNIKKGQFL

APHDMKNVIDKAREAARDRFMACERGVSFGYNNLVSDMRSLAIMRET--SAPVVFDATHSVQLPGGQGTS

SGGQREFVPVLARAAVATGVAGLFMETHPNPAEAKSDGPNAVPLNRMGALLETLVTLDQAVKRN------

>UniRef100_Q473G6

--MKLCGFEVGLDKPFFLIAGPCVIESEQMALDTAGELKAITGELGIPFIYKSSFDKANRSSGKSFRGLG

MEKGLEILATVKREIGVPVLTDIHEIDEIKPVAAVVDVLQTPAFLCRQTDFIRACAQSGKPVNIKKGQFL

APHDMKNVIDKARDAARDVFMACERGVSFGYNNLVSDMRSLAIMRET--GAPVVFDATHSVQLPGGQGTS

SGGQREFVPVLSRAAVATGIAGLFMETHPDPNKAMSDGPNAVPLSRMKELLAVLRDLDALTKRAGFLEDN

>Q1LPI7_RALME

---------VGLDKPFFLIAGPCVIESEQMALDTAGELKAITGELGIPFIYKSSFDKANRSSGKSFRGLG

MEKGLEILATVKRQIGVPVLTDIHEIDEIKPVAAVVDVLQTPAFLCRQTDFIRACAQSGKPVNIKKGQFL

APHDMKNVIDKARDAARDVFMACERGVSFGYNNLVSDMRSLAIMRET--GAPVVFDATHSVQLPGGQGTS

SGGQREFVPVLSRAAVATGVAGLFMETHPDPSKAMSDGPNAVPLSRMKELLTVLRDLDGMVKRA------

>UniRef100_B3R497

--MKLCGFEVGLDKPFFLIAGPCVIESEQMALDTAGELKAITTELGIPFIYKSSFDKANRSSGKSFRGLG

MEKGLEILAHVKREIGVPVLTDIHEIDEIKPVAAVVDVLQTPAFLCRQTDFIRACAQSGKPVNIKKGQFL

APHDMKNVIDKARDAARDVFMACERGVSFGYNNLVSDMRSLAIMRET--GAPVVFDATHSVQLPGGQGTS

SGGQREFVPVLSRAAVATGVAGLFMETHPDPSKAMSDGPNAVPLSRMKELLTVLKELDTLVKRAGFLEDN

>UniRef100_Q0KCE4

--MKLCGFEAGLDKPFFLIAGPCVIESEQMALDTAGELKAITAELGIPFIYKSSFDKANRSSGKSFRGLG

MEKGLEILATVKREIGVPVLTDIHEIDEIKPVAAVVDVLQTPAFLCRQTDFIRACAQSGKPVNIKKGQFL

APHDMKNVIDKARDAARDVFMACERGVSFGYNNLVSDMRSLAIMRET--GAPVVFDATHSVQLPGGQGTS

SGGQREFVPVLSRAAVATGVAGLFMETHPDPSKAMSDGPNAVPLSRMKELLAVLKELDTLVKRAGFLEDN

>UniRef100_B2U9C1

--MKLCDFEVGLDKPFFLIAGTCVIESEQMAIDTAGTLKEITGALGIPFIYKSSFDKANRSSDASFRGLG

MEEGLRILSEVRRQVGVPVLTDIHEIDEIKPVAEVVDVLQTPAFLCRQTDFIRACAQSGKPVNIKKGQFL

APHDMKNVIDKARHAARDNFMACERGASFGYNNLVSDMRSLAIMRET--GAPVVFDATHSVQLPGGQGTS

SGGQREFVPVLARAAIAVGVAGVFMETHPDPACAKSDGPNAVPLRRMKDLLSVLKELDALTKRSGFLENQ

>KDSA_RALSO

---------IGLDRPFFLIAGTCVIESEQMAIDTAGTLKEITGALGIPFIYKSSFDKANRSSDASFRGLG

MEEGLRILAEVRRQVGVPVLTDIHEIDEIKPVASVVDVLQTPAFLCRQTDFIRACAQSGKPVNIKKGQFL

APHDMKNVIDKARHAARDSFMACERGASFGYNNLVSDMRSLAIMRET--GAPVVFDATHSVQLPGGQGTS

SGGQREFVPVLARAAVATGVAGVFMETHPDPACAKSDGPNAVPLRRMKDLLSVLKELDTLTKRN------

>UniRef100_A1WL88

--MKLCGFDVGLEQRLFLIAGPCVIESAQLQMDVAGQLREITARLGMPFIFKSSFDKANRSSGSSYRGPG

RDKGLEILARVRRELALPVLTDVHTEDDIAAAAQVVDVLQTPAFLCRQTDFISAAARCGKPVNIKKGQFL

APHEMHNVIDKARAAARDRFMACERGASFGYNNLVSDMRSLAIMRAS--GAPVVFDATHSVQLPGSQGSR

SGGQREMVPVLARAAVAAGVAGIFMETHPDPDRALSDGPNAVPLKHMQALLETLVALDGVTKRHGFLENR

>UniRef100_A2SJR1

--MKLCGFDVGLDRPFFLISGPCVVESEQLQMDVAGRLKEITAALGIPFIFKSSYDKANRSSGTSFRGPG

MEKGLEILAKVRRDLQVPVLTDVHSEAEIPVVAQHVDVLQTPAFLCRQTDFIRAVAQSGKPVNIKKGQFL

APGDMKNVIQKARDAARDVFMACERGASFGYNNLVSDMRSLAIMRET--GSPVVFDATHSVQLPGGQGTS

SGGQREFVPVLSRAAIAVGVAGVFMETHPDPAKALSDGPNAVPLKHMKALLEQLVSIDRVIKAQPLLEND

>UniRef100_A9BN40

--MKLCGFDVGLDRRFFLIAGPCVVESEQLQMDVAGHLKEITAALGIHFIFKSSYDKANRSSGASFRGPG

MDKGLEILAKVKKDLQVPILTDVHTESEIPAVSQVVDVLQTPAFLCRQTDFIRAVAQSGRPVNIKKGQFL

APHDMKNVIDKARAAAKDSFMACERGASFGYNNLVSDMRSLSIMRET--GAPVVFDATHSVQLPGGNGTS

SGGMREMVPVLSRAAVAVGVAGLFMETHPNPPCALSDGPNAVPLKHMKALLETLVALDDVTKKNGFLEND

>UniRef100_A1TLS9

--MKLCGFDVGLDRPFFLIAGPCVIESEQLQMDVAGRLKETTAALGIPFIFKSSFDKANRSSGTSFRGPG

REKGLEILAKIRRELNVPVLTDVHTEDDITEAAKVVDVLQTPAFLCRQTDFIRAVAQSGKPVNIKKGQFL

APHDMKNVIDKARAAARDSFMACERGASFGYNNLVSDMRGLAIMRET--GAPVVFDATHSVQLPGGQGTS

SGGQREMVPVLARAAVAVGVAGLFMETHPDPCNALSDGPNAVPLKHMKALLETLVALDSVTKRNGFLEND

>UniRef100_Q21V38

--MKLCGFEVGLNQPLFLIAGPCVIESEQLQMDTAGTLKEITASLGIAFIFKSSFDKANRSSGSTFRGPG

IERGLEILAKVKRELGVPILTDIHSEDQIAQVASVVDVLQTPAFLCRQTDFIRAVAQSGLPVNIKKGQFL

APGDMKNVIDKARAAARDNFMACERGVSFGYNNLVSDMRSLAIMRDT--GAPVVFDATHSVQLPGGQGTS

SGGQREMVPVLARAAVAVGVAGLFMETHPDPSKALSDGPNAVPLKHMRALLETLVALDAVTKKNGFLENS

>UniRef100_A1VLH2

--MKLCGFDIGLDQPFFLIAGPCVVESEQLQMDTAGTLKEITASLGIPFIFKSSYDKANRSSGTSFRGPG

MEKGLQILAKVKRELGLPILTDVHSEAEITAVAAVVDVLQTPAFLCRQTDFIHAVAQSGRPVNIKKGQFL

APGDMKNVIDKARAAARDRFMACERGASFGYNNLVSDMRSLAIMRET--RAPVVFDATHSVQLPGGMGTS

SGGQREMVPVLARAAVAVGIAGLFMETHPDPANAMSDGPNAVPLKHMKALLETLLELDRVTKKNGYLENS

>UniRef100_Q128E6

--MKLCGFDIGLNQPFFLIAGPCVVESEQLQMDTAGTLKEITSSLGIPFIFKSSYDKANRSSGTSFRGPG

MVKGLEILAKVKRELNLPLLTDVHSEADIATVASVVDVLQTPAFLCRQTDFIHAVAQSGKPVNIKKGQFL

APGDMKNVIDKARAAARDNFMACERGASFGYNNLVSDMRSLAIMRET--NAPVVFDATHSVQLPGGQGTS

SGGQREMVPVLARAAVAVGVAGLFMETHPDPAKALSDGPNAVPLKHMKALLETLLELDRVTKKNGYLENS

>Q5NME8_ZYMMO

---------VGLGHKLFLIAGPCVIETEDLTLRTAERLKKIAEKLGILLIYKSSFDKANRSSSQSFRGPG

IDEGLRILEKVQRDVGLPVLTDVHTASQVQTVAEVVDVLQTPAFLVRQTDLIEAAAASGRPVNIKKGQFL

APGDMANVVAKARVAAAPSIMVAERGACFGYNNLVSDMRGLVIMRET--NCPLVFDATHSVQLPGGLGNK

SSGERQFVSVLARAAVSVGIAGLFLETHPDPANALSDGPNTVPLDHLEPLLTNLVDLDSFVKER------

>A5VC20_SPHWW

---------VGLDEPLFVIAGPCVIESEALVLSVAERLRAIADRLGLFAIFKSSFDKANRSSGASFRGPG

LDDGLRILEKVRAETGLPVLTDVHDVDQVRAAAQVVDVLQTPAFLARQTDLIEAVGASGRVANIKKAQFM

APHDMAHVVAKAQGAARTPVLVTERGTSFGYNNLVTDLRGLAIMRDT--GCPVVFDATHSVQLPGGLGAS

SGGQREYVPVLARGAVAVGIAGLFMETHPDPASAMSDGPNAWPLDRLEPLLAELQALDAHGIEA------

>UniRef100_A8GUR8

KIIKLKDLKIGNDLPFTLIAGPCQIEGLDHALFMAEELVKLTTRLNIPFIYKSSFDKANRTSVNGARGLG

IDKGLEVLAEVKRAFNCPIVTDVHSENQCAEVAKIVDMLQIPAFLCRQTDLLQAAAATGKIVNVKKGQFL

APWDMKNVHKKLESFGAKDILLTERGTCFGYNNLVSDMRGLAIMAEL--NTPVIFDATHSVQQPGGLGGS

TGGERKYVELLAKAAVAVGVAGLYMEVHQDPDNAPSDGPCMIRLDNLERVLTKLKKYDEITKEK------

>UniRef100_Q9ZE84

KVVKLNNIKIGNDLQFVLIAGPCQIEGKDHALFMAEKLMKLTSKLSIPFIYKSSFDKANRTSINGIRGLG

IEKGLEILSKVKSEFDCPIITDVHSESQCIETAKVVDILQIPAFLCRQTDLLKAAAKTGKIVKVKKGQFL

APWDMKNVQKKLEVFGAKDILFTERGSCFGYNNLVSDMRSLAIMSEL--NVPVVFDATHSVQQPGGRGGS

SGGERKYVELLAKAAISVGIAGIYMEVHQDPDNAPSDGPCMIKLDNLESILIKLKKYDKITKEIV-----

>KDSA_RICTY

---------IGNDLQFVLIAGPCQIEDEDHALFMAEKLIQLTSKLSIPFIYKSSFDKANRTSINGIRGLG

IEKGLEILSKVKSEFNCPIITDVHSESQCTDTAKVVDILQIPAFLCRQTDLLKAAAKTGKIVKVKKGQFL

APWDMKNVQTKLEAFGAKDILFTERGSCFGYNNLISDMRSLAIMSEL--NVPVVFDATHSVQQPGGRGGS

SGGERKYVELLAKAAISVGIAGIYMEVHQDPDNAPSDGPCMIKLDHLESILIKLKKYDKITKEE------

>UniRef100_A8EXC8

KVVKLHNIKIGNDLPFILIAGPCQIEGQDHALFMAEKLVKLTSKLDIPFIYKSSFDKANRTSINGVRGLG

IEKGLEVLSKVKAEFDCPIVTDVHSESQCTETARVVDILQIPAFLCRQTDLLQAAAKTGKIVKVKKGQFL

APWDMKNVQTKLKAFGAENILFTERGTCFGYNNLVSDMRSLAIMAKL--NVPVIFDATHSVQQPGGLGDS

TGGERKYVELLAKAATTVGIAGMYMEVHQDPDNAPSDGPCMIKLDNLESILIKLKKYDKITKEK------

>UniRef100_A8GM05

KIVKLNNIKIGNDLPFVLIAGPCQIEGKDHALFMAEKLVKLTSKLDIAFIYKSSFDKANRTSVNGIRGVG

IEKGLEILSKVKSEFDCPIVTDIHSESQCTETAEVADILQIPAFLCRQTDLLQAAAKTGKIVKVKKGQFL

APWDMKNVQTKLQAFGVKDILFTERGACFGYNNLVSDMRSLAIMAEL--NVPIVFDATHSVQQPGGLGGS

TGGDRKYVELLAKAAIAVGIAGMYMEVHQDPDNAPSDGPCMMKLDNLESILIKLKKYDKITKEK------

>UniRef100_Q4UN94

KVVKLNNIKIGNDLPFVLIAGPCQIEGKDHALFMAEKLVKLTSKLEIPFIYKSSFDKANRTSVNGIRGLG

IEKGLEILSKVKSEFDCPIVTDVHSESQCTETAEIADILQIPAFLCRQTDLLQAAAKTGKIVKVKKGQFL

APWDMKNVQTKLEAFGVKDILFTERGACFGYNNLVSDMRSLAIIAEL--NVPVVFDATHSVQQPGGLGGS

TGGERKYVELLAKAATAVGIAGMYMEVHQDPDNAPSDGPCMIKLDNLESILIKLKKYDKITKEK------

>A8F0G9_RICM5

---------IGNDLPFVLITGPCQIEGKDHALFMAEKLVKLTSKLEIPFIYKSSFDKANRTSVHGIRGLG

IEKGLEILSKVKSEFDCPIVTDVHSESQCTETAEVADILQIPAFLCRQTDLLQAAAKTGKIVKVKKGQFL

APWDMKNVQTKLEAFGVKDILFTERGACFGYNNLVSDMRSLAIMAEL--NVPVVFDATHSVQQPGGLGSS

TGGERKYVELLAKAATSVGIAGMYMEVHQDPDNAPSDGPCMMKLDNLESILIKLKKYDKITKEK------

>B0BW09_RICRI

---------IGNDLPFVLITGPCQIEGKDHALFMAEKLVKLTSKLEIPFIYKSSFDKANRTSVHGIRGVG

IEKGLEILSKVKSEFDCPIVTDVHSESQCTATAEVADILQIPAFLCRQTDLLQAAAKTGKIVKVKKGQFL

APWDMKNVQTKLETFGVKDILFTERGACFGYNNLVSDMRSLAIMAEL--NVPVVFDATHSVQQPGGLGGS

TGGERKYVELLAKAATSVGIAGMYMEVHQDPDNAPSDGPCMMKLDNLESILIKLKKYDKITKEK------

>UniRef100_Q92JH7

KVVKLNNIKIGNDLPFVLITGPCQIEGKDHALFMAEKLVKLTSKLEIPFIYKSSFDKANRTSVHGIRGVG

IEKGLEILSKVKSEFDCPIVTDVHSESQCTETAEVADILQIPAFLCRQTDLLQAAAKTGKIVKVKKGQFL

APWDMKNVQTKLEAFGVKDILFTERGACFGYNNLVSDMRSLAIMAEL--NVPVVFDATHSVQQPGGLGGS

TGGERKYVELLAKAATSVGIAGIYMEVHQDPDNAPSDGPCMMKLDNLESILIKLKKYDKITKEK------

>UniRef100_B0BZZ8

RTQVTDTLAIGEGCPLTLLSGPCVIESEDFTLKMASGIARICEKLGISYVFKSSFDKANRTSISSFRGHP

MEEGLRILQRVKDEVGVPVVTDIHESHQAAPVAEVADILQIPAFLCRQTDLLMAAAATGRTINVKKGQFL

APWDMKNVVSKLEAGGAKKILLTERGTSFGYNALVVDFRSLPQMRSF--GYPVVFDATHSVQMPGGQGSS

SGGQREYAPYLARAAAAIGVDALFMEVHENPDQAPSDGPNMIPLHQLEDVLRPILEVHKIMNSTPVLA--

>UniRef100_Q31KV0

RAKVGDRFSIGDGCPLTLFGGPCVIEGEDFSLKMAESIAKVCDRLGVQFVFKSSFDKANRTSIGSFRGYG

LEEGLRILERVKTELGLPVLTDIHESQQAATVAEVVDILQIPAFLCRQTDLLLAAAATGRTVNVKKAQFL

APWDMKNVVNKLRQGGAENLLLTERGSCFGYNALVVDFRSLPLMREL--GCPVVFDATHSVQIPGGAGDR

SSGQREFVPVLARAAAAVGIDALFMEIHENPDQALSDGPNMIRLADLEATLRQILRVREAVAEPIGASA-

>KDSA_SYNP6

---------IGDGCPLTLFGGPCVIEGEDFSLKMAESIAKVCDRLGVQFVFKSSFDKANRTSIGSFRGYG

LEEGLRILERVKTELGLPVLTDIHESQQAATVAEVVDILQIPAFLCRQTDLLLAAAATGRTVNVKKAQFL

APWDMKNVVNKLRQGGAENLLLTERGSCFGYNALVVDFRSLPLMREL--GCPVVFDATHSVQIPGGAGDR

SSGQREFVPVLARAAAAVGIDALFMEIHENPDQALSDGPNMIRLADLEATLRHILRVREAVAEP------

>A5GQM9_SYNR3

-----------MSIPFTLIAGPCVLESPELVFDMAAALKAMSERLGIRLLFKTSFDKANRSSGGSFRGPG

MEQGLEVLAQLKQELGVQLLTDIHESHQAAVVAQVVDVLQIPAFLCRQTDLLLAAAAAASVVNVKKGQFL

APWDMAQVVSKLREAG-EQLWLTERGTTFGYNTLVVDMRSLPQLRQQ--GCPVIFDATHSVQQPGGRGSS

TGGQREFVAPLARAAMAVGVDGLFIETHPDPDQALSDGPNMVPLHRLEPLLEQLLAIRAAAAAQ------

>A3PS35_RHOS1

---------IGGGHPIALITGPCQLESLDHARMMAERIAEACAPTGTKFIFKASYDKANRSSLSTARGLG

MEKGLEILGRIREEFGVPVLTDVHEPGHCATAAEVCDVLQIPAFLCRQTDLLLAAGETGRAVNVKKGQFL

APWDMKNVADKVASTGNRRILLCERGTSFGYNTLVTDFRGLPTMAAT--GWPVVFDATHSVQQPGGLGGS

SGGQREFAPVLARAACAVGVSALFIETHEDPDRAPSDGPNMIPVDRMGRLIADLCAFDALAKSL------

>A4X0C6_RHOS5

---------LGGAHPIALITGPCQLESLEHARMMAGRISEACAPTGTKFIFKASYDKANRSSLGTARGLG

MEKGLEILGAIRDEFGVPVLTDVHEPWQCAKAAEVCDVLQIPAFLCRQTDLLLAAGETGKAVNIKKGQFL

APWDMGNVAAKVASTGNRKILLCERGTSFGYNTLVTDFRGLPTMAAT--GWPVVFDATHSVQQPGGLGGS

SGGQREFAPVLARAACAVGVSALFIETHEDPDRAPSDGPNMIPVDRMGRLIAELRAFDALAKSL------

>Q28L37_JANSC

---------VSNTAPLLVIAGPCQLESLDHARMIAGHMSQVCAQVGAQFVFKGSYDKANRTSLDGKRGLG

MTAGLEILATIRDELGCPVLTDVHTPEQCDPVAGAVDILQIPAFLCRQTDLLLAAGKTGAAINVKKGQFL

APWDMANVAQKVASTGNERLMLTERGVSFGYNALVADMRSLPEMAKT--GYPVIMDATHAVAQPGGLGGS

SGGQREFAPVLARSAVALGIGGVFLETHEDPDTAPSDGPNMIRLDDMEGVLGTLMALDKVAKAN------

>A8LPK6_DINSH

---------VGNDQPLTLIAGPCQLESFEQGLEIGAALKEACDRSGAQFIFKASYDKANRTSAGGVRGMG

MAAGLDALGRLRETLGVPVLTDVHAAEHCAPAAEAVDILQIPAFLCRQTDLLIAAGETGAVVNVKKGQFL

APWDMPHVIGKIESTGNTRIMLTERGASFGYNTLVADMRSLPTMMKT--GYPVVMDATHSVQQPGGLGGA

SGGQREFAPVMARAAVSLGIAAVFIETHPAPDTAPSDGPNMIPLAQMQDLIGDLMAFDALAKTN------

>A1B1E4_PARDP

---------VGNDQPLALIAGPCQLETLDHALMIAETVAESCAKAGAGFVFKASYDKANRTSLKGRRGIG

IDEGLRMLDEVRRRIGCPVLTDVHDIEQARLAGAVVDVIQIPAFLSRQTDLLLAAGETPAAVNIKKGQFL

APWDMPNVAEKVASTGNERILLTERGVSFGYNTLVADMRSLPIMART--GWPVIMDATHSVQQPGGQGGS

SGGQREFAPVMARAAVALGVAGVFIETHQDPDNAPSDGPNMIHLDRMPALIASLMRFDALAKSD------

>Q1GIY0_SILST

---------IGNDCPLTVIAGPCQLESADHAQMIAGKLKEACDAAGAQFIFKASYDKANRTSLSGKRGLG

IDKGLEVLAGVRDTFGVPVLTDVHTEAQCIVAGDVVDVLQIPAFLCRQTDMLLAAGNTGKAVNIKKGQFL

APWDMDNVVAKVESTGNDKILLTERGTSFGYNALVADMRGLPRMAQT--GYPVVMDATHSVQQPGGQGGS

TGGQREFAPVMARAAVSLGIASVFIETHEDPDNAPSDGPNMIYLKDMPQLIETLMGFDALAKAN------

>Q5LUV5_SILPO

---------VGNDLPLTIIAGPCQLESADHAQMIAGTLKEACDAAGAQYVFKASYDKANRTSLGGKRGMG

IDAGLKVLDDIRRTMGVPVLTDVHSEAQCAIAAEAVDVLQIPAFLCRQTDMLLAAGNTGAVINVKKGQFL

APWEMGNIVDKIASTGNENILLTERGVSFGYNTLVADMRALPIMAQT--GYPVVMDATHSVQQPGGRGGS

SGGQREFAPVMARAAVAVGTAAVFMETHQDPDNAPCDGPNMIYLDEMPGLIDTLMRFDALAKAN------

>Q169Q7_ROSDO

---------IGNDRPLTVIAGPCQLESADHAQMIAGRMKEACDKAGAQYVFKASYDKANRTSLSGIRGPG

MDEGLKILQSVGQAIDVPVLTDVHNEGQCAPAAQAVDILQIPAFLCRQTDMLLAAGQTGAAVNVKKGQFL

APWEMSNITTKIESTGNTRILLTERGTSFGYNTLVADMRALPQMAQT--GYPVVMDATHSVQQPGGKGGS

SGGQREFAPVMARAAVAIGVAAVFIETHENPDASPSDGPNMIYLDQMPDLIETLMQFDALAKAR------

>Q4FM38_PELUB

---------ISNDNKICIIAGPCQLETEQHAMDMAGKIQEITKKLGLGFIYKTSFDKANRTSLKGQRGAG

LEASLPVFDKIKKELNVPILTDIHNAEQCSLLKDHVDILQIPAFLCRQTDLLIAAAKTNKIINVKKGQFL

APWDMVNVTKKISDSGNNNILVTERGASFGYNTLVSDMRSLPIMAKN--GYPVIFDATHSVQQPGGLGET

SGGQREFVEYLARAAVAVGVAGVFIETHQDPDNAPSDGPNMVPLDKLEKLLSQLFEIDNLIKK-------

>Q0BTX5_GRABC

---------FGNDLPFVLIAGPCQIESRAHAEGVAAELHAICARLDIPLVFKASYDKANRTSVGGARGVG

MERGLDILAGIRERFDIPVLTDIHDIGQCKPAAEAVDILQIPAFLCRQTDLLLAAGETGRTINVKKGQFL

APWDMRNVAAKIASTGNHNLLLTERGASFGYNTLVTDFRALPIMAQT--GYPVVFDATHSVQQPGGQGTA

SGGDRTFAPILARAALAVGVASVFIECHPDPDRAPSDGPNMIPLDQMEETLRRLKQFDVLGKAG------

>A5FXW8_ACICJ

---------VGNDLPFTLIAGPCQIESAEHAGEVAGALAAMTRELGIGLIFKSSYDKANRTSLTAGRGVG

MAQGLQILADIRAKFGIPVLTDVHDAAQCAPAAEAVDVLQIPAFLCRQTDLLLAAGHTGRAINVKKGQFL

APWDMRNVAAKIASTGNERILLTERGASFGYNTLVSDLRALPIMAQT--GYPVVFDATHSVQQPGGQGTA

SGGQREFAPILARAALAVGCAAVFIECHPDPDHAPSDGPNMIRLAEMPALLARLQQFDRLAKSA------

>A9HJ79_GLUDA

---------VGNDRPFVLIAGPCQIESEAHAMETADALHGIARETGVGLIYKSSFDKANRTSLGAARGVG

MAAGLEILARVRERFGVPVLTDVHAADQCAPTAAAVDVLQIPAFLCRQTDLLLAAGETGAAINVKKGQFL

APWDMTNVAAKIASTGNERIMLCERGTSFGYNTLVNDMRGLPIMAAT--GYPVVYDATHSVQQPGGLGGS

SGGQREFAPILARGALAIGVAAVFIETHQDPDSAPSDGPNMIPIRDMKALVQRLAQYDRLTKAG------

>Q5FNN3_GLUOX

---------VGQDQPFTLIAGPCQIESREHATEMADAISSLCRELGIGLIYKSSFDKANRSSVNAARGVG

MDAGLDILASIRERFGVPVLTDVHTAEQCAPAAQAVDVLQIPAFLCRQTDLLLAAGKTGRAINIKKGQFL

APWDMANVAAKIASTGNERIMLCDRGTSFGYNTLVSDMRGLPIMAQT--GYPVVFDATHSVQQPGGLGAS

TGGQREFVEPLARAALSIGVAAVFIETHQDPDHAPSDGPTMVPLSEMSGLLTRLKAFDALAKSF------

>Q2W699_MAGMM

---------LGNDLPLVLIAGPCQMESRDHAMECAEALKIMAAEAGIALIYKSSFDKANRTSLAGQRGVG

LDKALPIFAEIKAKLGLPVLTDVHTEEQCVIAAKVADVLQIPAFLCRQTDLLVAAGRTGAVINVKKGQFL

APWDMANVAAKIESTGNSRVLLTERGASFGYNTLVTDMRGLPIMART--GWPVIMDATHAVQAPGGQGNS

SGGDRRFAPVLARAAVAIGVAGVFVECHPDPDHAPSDGPNMIAMKDMPALVEVLMKLDATAKSF------

>Q2RT59_RHORT

---------FGNDLPMVLIAGPCQMESREHALETASILVALCADLGVPFIYKTSFDKANRTSLSGQRGVG

MEKALPIFAEIRETLGCPVLTDVHDAGQCAPVAEAVDVLQIPAFLCRQTDLLLAAAATGRVINVKKGQFL

APWDMKNVANKVMSAGNERVLLTERGASFGYNTLVTDMRSLPIMRDT--GCPIIIDATHSVQQPGGQGGS

SGGQREFAPVIARAALAVGVAGVFLETHADPDHAPSDGPNMIALDAMPALIAVLRDLDAFAKKY------

>A7HXX8_PARL1

---------IANDKPFVLIAGPCQLETRQHAFDMAGAVKEITDRLGIGFIYKTSFDKANRTSRNATRGLG

LELALPIFEDLKKELGLPVLTDVHERDQCAEVASVVDVLQIPAFLCRQTDLLEAAAQTGKVVNVKKGQFL

APWDMKNVVNKVVDAGNPNVLATERGVSFGYNTLVNDMRALPIMKEQC-RCPVVIDATHSVQQPGGQGTA

SGGDRRMVPHIATAAVSLGVAAVFIETHQDPDNAPSDGPNMVKLDELEALLARLRKFDELAKVA------

>A8IBG0_AZOC5

---------IGNSLPLALIAGPCQMESRDHALETAAALKEITDRLGIGLIYKTSFDKANRTSVNAARGMG

LEKALPVFAEIGERFGLPTLTDVHEIHQCAPVGEVVDVLQIPAFLCRQTDLLVAAARTGRVVNVKKGQFL

APWDMVNVVAKLTQSGNGRVLLTERGASFGYNTLVSDMRGLPVMAQT--GAPVVFDATHSVQQPGGKGAS

SGGQREFVPVLARAAVAVGVAAVFIETHPDPDHAPSDGPNMVPLKDLESLVALLQEIDRVAKAH------

>UniRef100_B1LZU2

PVVQVGDARFANHLPLTLIAGPCQLEGRQHALEIAAALKEMAAGLGVGLVFKTSFDKANRTSGSAARGIG

LDGALPVFAEIRETLGLPVLTDVHAAEQCARAAEAVDVLQIPAFLCRQTDLLLAAAATGRAVNIKKGQFL

APWDMKHVAAKVTEAGNPNVIVTERGASFGYNTLVSDMRSLPIMAQVTQGAPVVFDATHSVQQPGGQGAS

SGGQREFVAVLARAAVAVGVAGVFIETHPDPDRAPSDGPNMVALRDMPALLEELLAFDRLAKRRTA----

>UniRef100_Q89KV0

PVVTIGRVKFGNDLPISIIAGPCQLESRQHALEVASALKEIAARLNIGLVYKTSFDKANRTSASAARGLG

LAQSLPIFAEIRSSLGLPVLTDVHEATQCAEVAQAVDILQIPAFLCRQTDLLLAAAATGKVVNVKKGQFL

APWDMANVVTKITSANNPNVLVTERGASFGYNTLVSDMRALPILARTT-GAPVIFDATHSVQQPGGKGTS

SGGEREFVPVLARAAVAVGVAGVFIETHPDPDSAPSDGPNMVPLREFEGLIRRLMAFDALAKNPR-----

>KDSA_BRASB

---------FGNDLPLAVIAGPCQLESRGHALEVASALKEIATRLNIGLVYKTSFDKANRTSGSAARGLG

LAQSLPIFAEIRSSLGLPVLTDIHDASQCAEVAQAVDVLQIPAFLCRQTDLLLAAAATGKVVNVKKGQFL

APWDMANVVAKITGAGNPNVLATERGVSFGYNTLISDMRSLPIMARTT-GAPVIFDATHSVQQPGGQGTS

SGGQREFVPVLARAAVAVGVAGVFIETHPDPDHAPSDGPNMVPLAEFEGLLRRLMAFDKLAKAP------

>KDSA_BRASO

---------FGNQLPLAVIAGPCQLESRGHALEVASALKEIATRLNISLVYKTSFDKANRTSGSAARGLG

LAQSLPIFAEIRSSLGLPVLTDVHDASQCAEVAQAVDILQIPAFLCRQTDLLLAAAATGKVVNVKKGQFL

APWDMANVVAKITGAGNPNVLATERGVSFGYNTLISDMRSLPIMAKTT-GAPVIFDATHSVQQPGGQGTS

SGGQREFVPVLARAAVAVGVAGVFIETHPDPDHAPSDGPNMVPLAQFEGLLRTLMAFDALAKGQ------

>UniRef100_Q1QMJ3

PVVSAGSVTFGQDRPLSIIAGPCQMESRAHALEVAGALKEIAARLNIGLVFKTSFDKANRTSASGARGLG

LKQALPVFAEIGSSLGLPVLTDVHEAAQCTEVAQAVDVLQIPAFLCRQTDLLLAAAATGKVVNVKKGQFL

APWDMANVVAKITGGGNPNVLVTERGASFGYNTLVSDMRSLPILARTT-GAPVIFDATHSVQQPGGNGTS

SGGEREFVPVLARAAVAVGVAGVFIETHPDPDHAPSDGPNMVPLREFEALVRRLMAFDALAKAADPALPK

>UniRef100_Q3SRK0

PVVSVGSVTFGQDRPLSIIAGPCQMESRAHALEVAGALKDIAARLNVGLVFKTSFDKANRTSASGARGIG

LKQALPVFADIRSSLGLPVLTDVHEAAQCAEVAQVVDVLQIPAFLCRQTDLLLAAAATGKVVNVKKGQFL

APWDMGNVVAKITGGGNRNILVTERGASFGYNTLVSDMRALPILARTT-GAPVIFDATHSVQQPGGKGAS

TGGEREFVPVLARAAVAVGVAGVFIETHPDPDHAPSDGPNMVPLREFEALVRRLMAFDALAKAADPACPE

>Q07NE5_RHOP5

---------FGNALPLSIIAGPCQLESRAHALEVAGALKEISTRLGIGLVFKTSFDKANRTSASAARGVG

LNDALPVFAEIRETLGLPVLTDVHEIEQCARAAEAVDVLQIPAFLCRQTDLLLAAAATGKVVNVKKGQFL

APWDMANVVAKLVGAGNPNVLVTERGASFGYNTLVSDMRALPIMARTT-GAPVIFDATHSVQQPGGKGTS

SGGEREFVPVLARAAVAVGVAGVFIETHPDPDHAPSDGPNMVPLREFEALVRRLMQFDALAKAT------

>Q215A8_RHOPB

---------FGNALPLAVIAGPCQLESRAHALETASALKEIAQKLGIGLVYKTSFDKANRTSATSQRGLG

LEQALPIFAEIRESFGLPVLTDVHESEQCARAAEAVDILQIPAFLCRQTDLLLAAAATGKVVNVKKGQFL

APWDMANVVQKLTSAGNANVLVTERGVSFGYNTLVSDMRALPILARTT-GAPVIFDATHSVQQPGGKGTS

SGGEREFVPVLARAAVAVGVAGVFIETHPDPDHAPSDGPNMVPLREFEALMQRLMQFDALAKAR------

>UniRef100_B3Q6L6

TSVVAGNVKFGNALPLSVIAGPCQLESRAHALEVASALKEIATRLGIGLVYKTSFDKANRTSAASARGLG

LDAALPIFAEIRDHLGLPVLTDVHENEQCARAAEAVDILQIPAFLCRQTDLLLAAAATGRIVNVKKGQFL

APWDMGNVVSKITHAGNAKVLVTERGVSFGYNTLVSDMRALPIMAKTT-GAPVIFDATHSVQQPGGKGTS

SGGEREYVPVLARAAVAVGVAGVFIETHPDPDHAPSDGPNMVPLREFEALIKTLMEFDALAKKRSTVGAV

>UniRef100_P61657

TSVVAGNVKFGNALPLSVIAGPCQLESRAHALEVASALKEIATRLGIGLVYKTSFDKANRTSAASARGLG

LDAALPIFAEIRDHLGLPVLTDVHENEQCARAAEAVDILQIPAFLCRQTDLLLAAAATGRIVNVKKGQFL

APWDMGNVVSKITHAGNSKVLVTERGVSFGYNTLVSDMRALPIMAKTT-GAPVIFDATHSVQQPGGKGTS

SGGEREYVPVLARAAVAVGVAGVFIETHPDPDHAPSDGPNMVPLREFEALIKTLMEFDALAKKRSTVGAV

>UniRef100_Q136E1

PVVAAGDVKFGNSLPLSVIAGPCQLESRAHALEVASALKEIATRLGIGLVYKTSFDKANRTSAASARGIG

LDAALAIFAEIRDSVGLPVLTDVHEAEQCARAAEAVDILQIPAFLCRQTDLLLAAAATGRIVNVKKGQFL

APWDMGNVVAKITTAGNQKVLVTERGASFGYNTLVSDMRALPIMARTT-GAPVIFDATHSVQQPGGKGTS

SGGEREFVPVLARAAVAVGVAGVFIETHPDPDHAPSDGPNMVPLRDFEALLRTLMEFDALAKKRPGAGTI

>KDSA_RHOP2

---------FGNALPLSVIAGPCQLESRAHALEVAAALQEIGKRLGIGIVYKTSFDKANRTSAASARGIG

LDGALPIFAEIRDSIGLPVLTDVHEAGQCARAAEAVDILQIPAFLCRQTDLLLAAAATGRIVNVKKGQFL

APWDMGNVVAKITSAGNPKVLVTERGVSFGYNTLVSDMRALPIMAKTT-GAPVIFDATHSVQQPGGKGTS

SGGEREFVPVLARAAVAVGVAGVFIETHPDPDHAPSDGPNMVPLRDFESLLRTLMEFDALAKRR------

>KDSA_CAUCR

---------IGNGEKLSIIAGPCQMESRQHALETAHALKEMADRLGVGLIYKTSYDKANRTSANAQRGIG

LKDSLAIFQEIREVTGLPTLTDVHETSHCSIVAEAVDVIQIPAFLCRQTDLLVAAASTGRAINIKKGQFL

APWDMKNVIAKVTGAGNPNVMACERGASFGYNTLVSDMRALPIMKEI--GCPVVFDATHSVQQPGGQGTS

SGGQREFVPTLARAAVAVGVACVFIETHPDPDNAPSDGPNMVPVKEFEALVANLLRYDALTKAA------

>Q0APK5_MARMM

---------IGNDRKLSIIAGPCQLESRQHGLDVSARLREIADRLDVGLIYKTSFDKANRTSASAQRGLG

LEASLPVFEEIRETSGLPVLTDVHSEEQARIAATAVDVLQIPAFLCRQTDLLIAAAKTGKPINVKKGQFL

APWDMKNVIEKIAAAGNPDIMACERGASFGYNTLVSDMRSLPIMAQF--GAPVVFDATHSVQQPGGQGTS

SGGQREFVPTLARAAVAVGVAAVFIETHPDPDNAPSDGPNMVPLGEFEALVKRLLEFDALAKGY------

>UniRef100_A1US93

VSVKVGNVVFSNKAPLSLIVGPCQIESRDHAFEMAGRIKAIADQAGIGFVYKSSYDKANRTSLSAARGIG

LEKAIAIFADLKKEFGFPILTDVHTEEQCRAVSSVVDVLQIPAFLCRQTDLLVAAAKTGCVINIKKGQFL

APWDMENVLKKVTQSGNPDVMLCERGTSFGYNRLVSDMRSLPIMSSF--GAPVIFDATHSVQEPGGKGDS

SGGQRQFVEILARAAVAVGVAGIFLETHQDPDNAPSDGANMIEIDNLQRLIEILMNFDRLTKNELNIHA-

>UniRef100_A9IS46

ARVKVGNIIFSNEMPLSLIAGPCQMESRDHAFEMAGRIKTMTDQLGIGFVYKSSYDKANRTSLSAARGIG

LEKAMAIFSDLKKEFDCPILTDVHTEEQCTIVSSTVDILQIPAFLCRQTDLLVAAAKTGCVVNIKKGQFL

APWDMENVLKKVVHSGNPNVMLCERGTSFGYNRLISDMRSLPILRSF--GAPVIFDATHSVQEPGGQGAS

SGGQRQFVEVLARAAVSVGVAGIFLETHQDPDHAPSDGPNMIKIDDLQRLIETLMEFDDLSKKLN-----

>UniRef100_Q6G026

TIVKVGNIVFSNETPFSLIVGPCQMESRDHAFEMAGRIKTITDQIGIGFVYKSSYDKANRTSLGAARGIG

LEKAMAIFSDIKKEFGCPVLTDVHTEEQCAAVGSVVDVLQIPAFLCRQTDLLVAAAKTGRVINIKKGQFL

APWDMENVLKKVTQSGNPHVMLCERGTSFGYNRLISDMRSLPILRSF--GAPVIFDATHSVQEPGGQGDS

SGGQRQFVEVLARAAVSVGVAGIFLETHQDPDNAPSDGPNMVKIDHLQRLLETLMEFDYLSKKVN-----

>KDSA_BARHE

---------FSNEAPFSLIAGPCQIESRDHAFEMAGRIKTITDQVGIGFVYKSSYDKANRTSLSAVRGVG

LENAMAIFSDLKKEFGCPILTDVHTEEQCTVVASTVDILQIPAFLCRQTDLLVAAAKTGRVINIKKGQFL

APWDMKNVLRKVTQSGNRNVMLCERGTSFGYNRLISDMRSLPILRSF--GAPVIFDATHSVQEPGGQGDS

SGGQRQFVEILARAAVSVGVAGIFLETHQDPDNAPSDGPNMIKIDHLQRLLETLMEFDYLSKKT------

>UniRef100_A6X0L7

SSVKIGNVTFSNSAPFALIAGPCQMETREHAFDMAGRLKEMTDKLGIDLVYKSSFDKANRTSLKAERGIG

LEKAMEVFADLKKEFGFPVLTDIHTEEQCAAVAPVVDVLQIPAFLCRQTDLLIAAAKTGRVVNVKKGQFL

APWDMKNVLSKITESGNPNVLATERGVSFGYNTLVSDMRSLPIMAGL--GSPVVFDATHSVQQPGGHGGS

SGGQREFVETLARAAVAVGVAGLFIETHQDPDNAPSDGPNMVPVDKMPALLEKLMAFDRIAKGL------

>KDSA_BRUA2

---------FSNSAPLALIAGPCQMETRDHAFEMAGRLKEMTDKLGIGLVYKSSFDKANRTSLKAARGIG

LEKALEVFSDLKKEYGFPVLTDIHTEEQCAAVAPVVDVLQIPAFLCRQTDLLIAAARTGRVVNVKKGQFL

APWDMKNVLAKITESGNPNVLATERGVSFGYNTLVSDMRALPIMAGL--GAPVIFDATHSVQQPGGQGGS

TGGQREFVETLARAAVAVGVAGFFIETHEDPDNAPSDGPNMVPIDKMPALLEKLMAFDRIAKAL------

>B0CGT3_BRUSI

---------FSNSAPLALIAGPCQMETRDHAFEMAGHLKEMTDKLGIGLVYKSSFDKANRTSLKAARGIG

LEKALEVFSDLKKEYGFPVLTDIHTEEQCAAVAPVVDVLQIPAFLCRQTDLLIAAARTGRVVNVKKGQFL

APWDMKNVLAKITESGNPNVLATERGVSFGYNTLVSDMRALPIMAGL--GAPVIFDATHSVQQPGGQGGS

TGGQREFVETLARAAVAVGVAGLFIETHEDPDNAPSDGPNMVPIDKMPALLEKLMAFDRIAKAL------

>KDSA_BRUME

---------FSNSAPLALIAGPCQMETRDHAFEMAGRLKEMTDKLGIGLVYKSSFDKANRTSLKAARGIG

LEKALEVFSDLKKEYGFPVLTDIHTEEQCAAVAPVVDVLQIPAFLCRQTDLLIAAARTGRVVNVKKGQFL

APWDMKNVLAKITESGNPNVLATERGVSFGYNTLVSDMRALPIMAGL--GAPVIFDATHSVQQPGGQGGS

TGGQREFVETLARAAVAVGVAGLFIETHEDPDNAPSDGPNMVPIDKMPALLEKLMAFDRIAKAL------

>Q1MH37_RHIL3

---------FSNAGRLSLIAGPCQMESRDHAFMVAGTLKELCGKLGIGLVYKSSFDKANRTSLSAERGIG

IEKGMEVFADLKKEFGFPVLTDVHTAEQCEEVAKVVDVLQIPAFLCRQTDLLIAAAKTGRVVNVKKGQFL

APWDMKNVLKKLNASGNPNVLLCERGASFGYNTLVSDMRSLPIMAAM--GAPVVFDATHSVAQPGGQGES

SGGQREFVETLARAAVAAGIAGVFVETHQDPDNAPSDGPNMVYLKDMPRLLEKLLAFDAVAKA-------

>Q2K8X0_RHIEC

---------FSNAGRLSLIAGPCQMESRDHAFMVAGTLKELCAKLGVGLVYKSSFDKANRTSLSAERGIG

LEKGMEVFADLKKEFGFPVLTDVHTAEQCAEVAEVIDVLQIPAFLCRQTDLLIAAARTGRVVNVKKGQFL

APWDMKNVLKKLNASGNPNVLLCERGASFGYNTLVSDMRSLPIMTAM--GAPVIFDATHSVAQPGGQGES

SGGQREFVETLARAAVATGIAGVFLETHQDPDNAPSDGPNMVYLKDMPRLLEKLLAFDAVAKA-------

>KDSA_AGRT5

---------FCNTERFSLIAGPCQMESRDHAFMIAGVLKELCDSLGIGLVYKSSFDKANRTSLSGKRGIG

LDSAMEIFADLKKEFGFPVLSDIHTEEQCAIVSEVVDVLQIPAFLSRQTDLLVAAAKTGRVINVKKGQFL

APWDMKNVLAKLNESGNPNVLLCERGASFGYNTLVSDMRSLPIMASL--GAPVIFDATHSVQQPGGQGGS

TGGQREFVETLARAAVAVGVAGLFIETHEDPDNAPSDGPNMVHLKDMPKLLEKLLAFDAITKA-------

>A6U8E4_SINMW

---------FSQKERLTLIAGPCQMESREHAFTIAGKLVELCRSLGIGLVYKSSFDKANRTSLSGKRGIG

LDNAMEVFADLKREFGFPVLTDIHTEEQCALVAETVDILQIPAFLSRQTDLLVAAAKTGRAINVKKGQFL

APWDMKNVLAKFTQSGNPNVLLCERGASFGYNTLVSDMRSLPIMAAL--GAPVVFDATHSVQQPGGQGGS

SGGQREFVETLARAAVAVGVAGVFVETHEDPDNAPSDGPNMVPLKDMPRLLEKLLAFDAIAKG-------

>KDSA_RHIME

---------FSQKERLTLIAGPCQMESREHAFMIAGELVELCRSLGLGLVYKSSFDKANRTSLSGKRGIG

LDKAMEVFADLKREFGFPVLTDIHTEEQCAAVAETVDILQIPAFLSRQTDLLVAAAKTGRTINVKKGQFL

APWDMKNVLAKFTESGNPNVLLCERGASFGYNTLVSDMRSLPIMAAL--GAPVVFDATHSVQQPGGQGGS

TGGQREFVETLARAAVAVGVAGVFVETHEDPDNAPSDGPNMVPLKDMPRLLEKLLAFDAVAKA-------

>KDSA_RHILO

---------FDNNAALALIAGPCQFESRQHAFDMAGALKELTARLGIGLVYKTSYDKANRTSLSATRGAG

MDAALPVFDELRKEFSLPVLTDVHTEEQCAIVAPHVDVLQIPAFLSRQTDMLVAAAKTGKVINVKKGQFL

APWDMKNVVAKITGSGNPNVLTTERGASFGYNTLVSDMRALPVMAEI--GAPVIFDATHSVQQPGGQGGS

SGGERRFVETLARAAVAVGVAGVFIETHQDPDNSSSDGPNMLPLKDMPALLERLMAFDRIAKGR------

>UniRef100_Q11HU7

PRVEIGQAVFANDAPLALIAGPCQLESREHAFEMAGRLKEMAEKLGLGFVYKTSFDKANRTSLLGKRGAG

LDAALPIFADLRRELGVPVLTDVHTEGQCEILADAVDVLQIPAFLCRQTDLLVAAAKTGKVVNVKKGQFL

APWDMKNVVAKITASGNPNVLTTERGVSFGYNTLVTDMRALPIMAEI--GAPVIFDATHSVQQPGGQGSS

SGGDRRFVETLARAAVAVGVAGVFIETHQDPDNAPSDGPNMVQLDKMPALLERLMAFDRIAKSK------

>A0LK05_SYNFM

---------VGTNR-FFVIGGPCVIENRDMTLRIAEFLRNACARLHIPCVFKSSYDKANRTSIHSYRGPG

IEEGLAILSEVRREIGIPVLTDVHGVSEVAPAKEVVDILQVPAFLARQTDLVVAVGLTGKPVNLKKAQFL

APRDMSLVIEKVRGTGNDKILVTERGTQFGYNNLVVDMRSIPILSES--GCPVVFDATHSVQLPGGQGTR

SGGERRYVAALACAAVAAGAHGVFLEMHEDPDRALCDGPNSLPLEQVSPLLEKLLDIHRIVRDT------

>Q310T3_DESDG

--------KTLCDGPFIIA-GPCVLESYELALETARIVREAADRHAVTAIFKSSFDKANRTSASSFRGPG

MTTGLEWLARIREETGLPVITDIHEPAQAAPVAAVADILQIPAFLCRQTALLQAAAATGSVVNVKKGQFV

APWDMGPVVEKLHAAGNRHVMLTERGASFGYNNLVVDFRSFSIMRSL--NVPVIFDATHSVQLPGGQGSC

SGGDRRHVPALARAAVAAGANGVFLECHPDPDKALCDGPNSWPAGRLSDLVGDLKSIWSIDYAC------

>KDSA_DESVH

---------ARLAARRFILAGPCALEDFDVAMETAHAVREAAEAAGLFAVFKSSWDKANRTSITSFRGPG

LVRGMEWLARIREESGLPVVTDIHLPEQAAPVAEVADIIQIPAFLCRQTDLLVAAAATGRVVNVKKGQFV

APWDMRPAVEKLRAAGNERILLTERGASFGYNNLVVDYRSIPTMQGF--GVPVVFDATHSVQLPGGLGGS

SGGERRHVPVLARAAVAAGVDGVFLECHPDPDKALCDGPNSWPLDRLPALLKELSALWSLEHVC------

>KDSA_DESVV

---------ARLAARRFILAGPCALEDFDVAMETAHAVREAAEAAGLFAVFKSSWDKANRTSITSFRGPG

LVRGMEWLARIREESGLPVVTDIHLPEQAAPVAEVADIIQIPAFLCRQTDLLVAAAATGRVVNVKKGQFV

APWDMRPAVEKLRAAGNERILLTERGTSFGYNNLVVDYRSIPTMQGF--GVPVVFDATHSVQLPGGLGGS

SGGERRHVPVLARAAVAAGVDGVFLECHPDPDKALCDGPNSWPLDRLPALLKELSALWSLEHVC------

>UniRef100_Q6AQ79

AQPNGSDILVGSGQPLLLIGGPCSLESEQLGREVAETVSKICKKLGISYVFKASFDKANRTSISSYRGPG

LKKGLASLARIREDLQVPVISDIHDISQVGPAAEVLDIIQIPAFLCRQTDLLVAAAKSGKPVNVKKGQFV

SPWDMENAIGKLRDAGSDKVMLVERGACFGYNNLVVDMRSLPVMRSF--NCPVIYDATHSVQLPGGAGGS

SSGQREFIEPLSKAAIAAGIDGLFMEIHPDPDKALCDGPNSIALDQVEELLTKLVKVRAAVEE-------

>UniRef100_A8ZU09

DTIVLNDISIGGGAPLVVIAGPCVIEDYDTTLQAAEFLKTVTDRLGIPFIFKASYDKANRSSVHSFRGPG

PDLGLDILRRVKERLHVPVLSDVHTESQIGPAAQVLDIIQTPAFLCRQTDFITAVAATGKIINIKKGQFL

APWDITQVVEKARTAGNENILITERGAMFGYNNLVVDFRAIPIMQQT--GKPVIFDATHSVQLPGGQGTC

SGGQREFVPCLARAAVAAGADGIFLEVHRNPDKALCDGPNSLPLEQVEPLLTALVAIRQAATEQAGHA--

>UniRef100_B8F9X0

TFSIGNNMNVGLGAPLLLIAGPCVIENEEKTLEIAERIKGIVRDMDVNFVFKASFDKANRTSIDSFRGPG

LEQGLAILGKVKSRLGLPVISDVHSPDQVGPASEVLDILQIPAFLCRQTDLLTAAGNSGKPVNVKKGQFV

GPWDMKHVTGKVLSTGNERIMLTERGSSFGYNNLVVDFRNFSIMRDL--GFPVVFDATHSVQMPGGLGSC

SGGDRSYVPLLARAAAAAGVDGVFFEVHTDPDKALCDGPNSLTMEMLESILPQLLAIRKAAS--------

>Q2LSM7_SYNAS

---------VGGGSPLVLIAGPCVIEDEERTRDIALYLKKLTDEMDIPFIFKASYDKANRTSGKAYRGPG

LERGIEILKAIKGDLGIPILSDVHSREEIEKAAEILDVVQIPAFLCRQTDLVREVARLARAVNIKKGQFL

APWDVRNIIEKVLSEGNERILVTERGTSFGYNNLVVDFRSLPLLRRE--GYPVIFDATHSVQLPGGQGTA

SGGQREMVPCLMRAAAAVGIDGLFLEVHPDPEKALCDGPNSLYLDALPELLSTVKSIDRLVKNR------

>UniRef100_Q3A372

DTLKVADVTFGGNHPVALIAGPCVMENEAHTLAIARQLLEVKNELGVGVVFKASFDKANRTSVSAYRGPG

LESGLRILDKVRQQTGLPIVSDIHDVSQVEAAAEVLDILQIPAFLCRQTDLLLAAGRSGKVVNIKKGQFL

APWDMANAVAKVASTGNDRILLTERGTSFGYNNLVVDMRSLAVMREL--GCPVVFDATHAVQLPGGAGTS

SGGQRQFVAALSRAAVAVGVDGLFWEVHPDPDRALCDGANSLPLDQVKKTLKEMMAIDAIVKGNTES---

>UniRef100_B3E467

KELSIGSVKMGGGRLLVLIAGPCVIESEEATLRHAERLMTICNGLGMPLIFKSSYDKANRTSINAFRGPG

MDEGLRILSKVKESLGIPVLSDIHSIEQVAPAAQVLDVLQIPAFLCRQTDLVVAAAKTGKVVNVKKGQFL

APWDMRNVVGKVAASGNENIILTERGASFGYNNLVVDMRSFPVMRSY--GYPVVFDATHSVQLPGGQGES

SGGQREFVETLSRAAVATGIDGIFMEVHEDPSCALCDGPNSIPLAELPALLKRLQALDAVVR--------

>UniRef100_A1APG0

REIVIGGVKIGAKRPLALVAGPCVIESELATMRQAERLMTICNALSLPLIFKASYDKANRTSIGAYRGPG

MREGLRILRKVKESLGLAVLSDVHSIEQVAPAAEVLDVLQIPAFLCRQTDLLIAAAATGRVINVKKGQFL

APWDMKNVAAKIASSGNENIILTERGASFGYNNLVVDMRSFPVMRAS--GYPVIFDATHSVQLPGGQGES

SGGQREFVEFLSRAAVAAGVDGIFMEVHEEPEKALCDGPNSIALNDLPALLATLKAIDAVVK--------

>UniRef100_B5E8N4

REITVGGVKIGGGRPLALVAGPCVIENETATLRCAERLMSICNGVGISLIFKASYDKANRTSVTAFRGPG

MKEGLRILAKVKEALGVPVLSDIHSIEQVEPAAEVLDVLQIPAFLCRQTDLLVAAGNTGKVINVKKGQFL

APWDMKNVVGKISSCDNDNIILTERGASFGYNNLVVDMRSFPIMRST--GYPVIFDATHSVQLPGGEGTS

SGGQREYVEFLSRAAVAAGVDGIFMEVHEEPEQALCDGPNSVRLDDMPALLKKLKAIDAIVNQ-------

>A5G5T2_GEOUR

---------IGGDRPLVLIAGPCVIENEVATMRCAERLMTICNGVSIPLIFKASYDKANRTSVNSFRGPG

MKDGLKILKKVKEALGVPVLSDIHSIEQVEPAAEVLDVVQIPAFLCRQTDLVVAAARSGKVVNIKKGQFL

APWDMENVAGKAVSTGNDNIILTERGVSFGYNNLVSDMRSFPILRKI--GYPVVFDATHSVQLPGGLGGS

SGGQREFVEYLGRAAVATGIDGIFMEVHDDPEKALCDGPNSVKLEDLPALLKKLKAIDAIVK--------

>UniRef100_Q39W61

REIAIGNVKMGGSRPLVLIAGPCVIENETATLRCAERLMTIVNGLSIPLIFKASYDKANRTSVTAFRGPG

LKEGLRILAKVKESLGLPVISDIHSIEQVQPAAEVLDILQIPAFLCRQTDLLVEAARTGCVVNVKKGQFL

APWDMENVVGKIVASGNERIILTERGASFGYNNLVSDMRSLPIMRRF--GFPVVFDATHSVQLPGGQGGS

SGGQREFVEYLSRAAVATGIDGVFLEVHEEPDKALCDGPNSVPLDDLPVLLKKLKAIDAIVK--------

>KDSA_GEOSL

---------IGGDRPLVLIAGPCVIENEAATLRCAERLMTICNGVSVSLVFKASYDKANRTSVTSFRGPG

MQEGLRILQKVKDSLGIPVISDIHSIEQVKPAAEVLDIIQVPAFLCRQTDLVVEVGRTNRVVNVKKGQFM

APWDMENVVGKILSTGNERIILTERGVTFGYNNLVSDMRSLPIMRRI--GFPVVFDATHSVQLPGGQGGS

SGGQREFVEYLSRAAVATGIDGIFMEVHEDPEKALCDGPNSVKLDDLPALLKKLKAIDAIVK--------
