# Supplementary material for: Accurate Simulation and Detection of Coevolution Signals in Multiple Sequence Alignments
Source: PLoS One. 2012 Oct 16;7(10):e47108. doi: 10.1371/journal.pone.0047108 (PMC3473043; doi:10.1371/journal.pone.0047108)
Supplement: MSA S2 — MSA of ArsA. (DOCX) [file pone.0047108.s013.docx]

>F0YAE3|F0YAE3_A

GDLPRLAKKFVFFTGKGGQGKTSVACATALALADGGKKTLLVSTDPASNLGQALGLADGAGVSAAVGAPS

SVCGNLWAANAAAAYRKKALRALMARADDDELDRAEEGLAGQCTVEIAAFDAFAGVLARP--DAAYDVVV

FDTAPTGHTLRLLGLAWEWTAFFGKSPTGASCLGPVEALAAQAETFAAARAALADGARTALLLVARPESS

ALAEAARTAGELAALDVAPAGLVLNGVLETPG------------------DDAPENLQIVAS--LALAAF

DVVGLGALRALVGPPP--PAAAPVAAAA---THSLAAVVDELARGPPTVVLLVGKGGVGKTTLAAAVGVG

LAARGLRTHVTTTDPAAHVGATLGSSIPNLTVDAVDAAAATRAYVERAVAAKGP-LSARDEALLREELDS

PCTEELAVFDAFAEKIVMD--GFVVVDTAPTGHTLLLLDQTGAYQRDVDKHESGGAAAATPLELLR--AR

ATILVVALPEPTPVAEALALEADLRRAGLPVAGFVVNQLVPATD--HAVLGRRAAAQAGRVAAVAAAAAT

TVVAVPWRGGDLVGADALRALCP

>B8YJN2|B8YJN2_9

FTREHPTTPFLFFTGKGGVGKTSTASSLSIALAKEGRRVLLISTDPASNLQDIFGQDLKNEPTKINGTEN

LYALNLDPEQAAASYKEQMVGPYRGKLPEVVIQNMEEQLSGACTVEIAAFNEFAMLLTNDAYTEQYDTIV

FDTAPTGHTLRLLQLPSAWSTFLDENTTGTSCLGPLKGLEPQREVYKQAVAQLTNEKQTTLMLVTRPEAN

PLKEAARASEELFEIGLRNQQLIINGFLQSA--SDDPIEQAFYERQVIALKEMPEPLKAFDHYYLPFVPY

SLSSIERMEAWMMSEL--QTKENQTS--LN-PPDLEKMIEDYVARKPKIIFAMGKGGVGKTTVASYIALR

LAEEGIRVHLTTTDPAAHISWTFEE-QEAVTVSRIDPKVEIFNYEAEVLAKASETMNEDGLAFVKEDLAS

PCTEEIAVFKAFANLVETHQDEVIIIDTAPTGHTLLLLDATESYHREISRSGEVAVSNLLP--RLRDQHY

TSVAIITLPEATPVYEASRLQEDLERAGLHVDWWVVNQSFAGLTLTSPTLMQKQQSEVKWIEEVKQLSKN

RFVTIPWVKEAPVGKDGLHALKG

>B1HYK9|B1HYK9_L

FTRSHPDTPFLFFTGKGGVGKTSVACSLSIAIAKERKKVLLISTDPASNLQDIFGQTLSNSPTKIEGIDN

LFAINLDPEQAAQHYKEQMVGPYRGKLPDVVLQNMEEQLSGACTVEIAAFNEFATLLTDTSVIGNFDTVV

FDTAPTGHTLRLLQLPSAWSTFLDDNTTGTSCLGPLKGLEQQREVYKEAVERLKNANQTTLMLVTRPEEN

PLKEAARASQELFEIGIQNQTLLINGYMSNVY-STDDIEEAFIARQADALARIPDELNRFEHFYLPFVPY

SLSSIERLQAWMTQEV--VYEDENEE--TK-IPEIEEMIADYLERKPKLIFTMGKGGVGKTTVASYIALR

LAEEGTHVHLTTTDPAAHLNWTFDN-VKNLTISRIDPKAEVANYEAEVLAKASETMNEEGLAFVKEDLAS

PCTEEIAVFRAFANVVENHQDEVIIIDTAPTGHTLLLLDATEAYHRG---SGDPAVSNLLP--RLRDASY

TSVAIVTLPEATPVYEASRLQEDLHRAGLSVDWRVVNQTFSSIYTTSPTLIQKQQAETKWLKEVRSISNN

QFVAIPWVKTPPVGTQGLHELKG

>D7WNF0|D7WNF0_9

FTRSHPDTPFLFFTGKGGVGKTSVACSLSIAIAKEGKKVLLISTDPASNLQDIFGQTLSNSPTKIAGIDN

LFAINLDPEQAAQHYKEQMVGPYRGKLPEVVLQNMEEQLSGACTVEIAAFNEFASLLTDTSVIGNFDTVV

FDTAPTGHTLRLLQLPSAWSTFLDDNTTGTSCLGPLKGLEPQREVYKEAVERLKNANQTTLLLVTRPEEN

PLKEAARASHELFEIGIQNQTLLINGYMSNAN-STDDIEEAFIARQADAIARIPKELNQFEHFYLPFVPY

SLSSIESMQAWMTQEV--IHEDENEE--TK-IPEIEEMIADYLERKPKIIFTMGKGGVGKTTVASYIALR

LAEEGMHVHLTTTDPAAHLNWTFDK-IKNVTVSRIDPKVEVSNYEAEVLAKASETMNEEGLAFVKEDLAS

PCTEEIAVFRAFANVVENHQDEVIIIDTAPTGHTLLLLDATEAYHREISRSGDPAVSNLLP--RLRDASY

TSVAIVTLPEATPVYEASRLQEDLQRAGLSVDWWVVNQTFSSIYTTSPTLIQKQQAETKWLKEVKSISNN

QFVAIPWVKTPPVGTQGLHELKG

>C7UHP2|C7UHP2_E

YQPDQALTKYLFFTGKGGVGKTTTACGTATYLADSGKKVMLVSTDPASNLQDVFQTELTNKGKEIPEVPG

LTVANFDPVTAADDYKESVVGPFRGKLPDSVLANMEEQLSGSCTVEIAAFNEFSGFLTDPEAEKKYDYII

FDTAPTGHTLRMLQLPSAWSNFMDENTTGASCLGQLSGLGDKKEIYEHAVTTLADGEKTTLMLVTRPQKS

PLLEADRASKELQEIGIKNQVLLVNGVLE--E-ATDKVSQLIYAGQQEALAQMPDSLKAFPEYSIPLRSY

NVTGVENLRQLLKDQG--EFLTEVAQ--RT-FPRLKDIVNELDQSGKKVIFTMGKGGVGKTTIAAAIATG

LADKGKKVHLATTDPAAHLQFVI---SDQIKVSHIDEDKELADYTEEVLSKARETMSPDDVAYVEEDLRS

PCTQEIAVFRAFAEIVDDADCDVVVIDTAPTGHTLLLLDSTQSYHKEVERTGEESVKRLLP--RLQDGKE

TEVVMVTLPETTPVYESMRLQEDLDRAGIAHTWWVVNNSMLTSGTTNPMLLARAQNENTWIDKVAELSNN

HYGVVEWHAEEISGAALHNILN-

>C7WC06|C7WC06_E

YQPDQPLTKYLFFTGKGGVGKTTTACGTATYLADSGKKVMLVSTDPASNLQDVFQTELTNKGKEIPEVPG

LTVANFDPVTAADDYKESVVGPFRGKLPDSALANMEEQLSGSCTVEIAAFNEFSGFLTDPEAEKKYDYII

FDTAPTGHTLRMLQLPSAWSNFMDENTTGASCLGQLSGLGDKKEIYEHAVATLADGAKTTLMLVTRPQKA

PLLEADRASKELQEIGIENQVLLVNGVLE--E-ATDKVSQLIYDGQQEALTQMPESLKAFPEYSIPLRSY

NVTGVENLRQLLKNQG--EFLTEVTP--RA-FPRLKDIVNELHQSGKKVIFTMGKGGVGKTTIAAAIATG

LADKGKKVHLATTDPAAHLQFVI---SDQIKVSHIDEDKELADYTEEVLSKARETMSPDDVAYVEEDLRS

PCTQEIAVFRAFAEIVDDADCDVVVIDTAPTGHTLLLLDSTQSYHKEVERTGEESVKRLLP--RLQDGKE

TEVVMVTLPETTPVYESMRLQEDLDRAGIAHTWWIVNNSMLTSGTTNPMLIARAQNENTWIDKVAELSNN

HYGVVEWHAEEISGEALHNILN-

>C7WQN3|C7WQN3_E

YQPEEHLTKYLFFTGKGGVGKTSTASATATYLADKGKQVMLVSTDPASNLQDVFELELSNKGTKIPNVEG

LVVANFDPVEAANDYKESIVGPYRGKLPDSVLENMEEQLSGSCTVEIASFNEFANFLTDKEASTNFDHII

FDTAPTGHTLRMLQLPSAWNNYLDENETATAPLGQLSGAVDKKEMYDLAVKTLIDGQKTTLMLVTRPQKT

SLLEADRASKELEEMGIKNQALIINGVLE--E-ATDKVSEEFYAIQQEALKQMPASLAKYPEYFVPLRPY

NLTGIENIRKLLNHQE--ELLRGIKP--KE-FPHLQVIVDNLYQTDKKVIFTMGKGGVGKTTIAAAIAMA

LADKGKKVHLATTDPAAHLQFVI---TDKISVSHIDEEKELADYQSEVLTKARETMSEEDVAYVEEDLRS

PCTQEIAVFRKFAEIVEGADSDVVVIDTAPTGHTLLLLDSTQSYHKEIERSGDSLSRSYCL--SYKMGMK

QK--------------------------------------------------------------------

-----------------------

>E6IBX8|E6IBX8_E

YQPEEHLTKYLFFTGKGGVGKTSTASATATYLADKGKQVMLVSTDPASNLQDVFELELSNKGTKIPNVEG

LVVANFDPVEAANDYKESIVGPYRGKLPDSVLENMEEQLSGSCTVEIASFNEFANFLTDKEASTNFDHII

FDTAPTGHTLRMLQLPSAWNNYLDENETATAPLGQLSGAVDKKEMYDLAVKTLIDGQKTTLMLVTRPQKT

SLLEADRASKELEEMGIKNQALIINGVLE--E-ATDKVSEEFYAIQQEALKQMPASLAKYPEYFVPLRPY

NLTGIENIRKLLNHQE--ELLRGIKP--KE-FPHLQVIVDNLYQTDKKVIFTMGKGGVGKTTIAAAIAMA

LADKGKKVHLATTDPAAHLQFVI---TDKISVSHIDEEKELADYQSEVLTKARETMSEEDVAYVEEDLRS

PCTQEIAVFRKFAEIVEGADSDVVVIDTAPTGHTLLLLDSTQSYHKEIERSGDESVQKLLP--KLQNGDE

TEVVMVTLPEATPVYESIRLKDDLERAGIARTWWVVNNSMLSSGTTNPMLLARAQNEEVWIEKVAELSNN

HYAVIEWKTEDISGDALRDIL--

>F9V9Y2|F9V9Y2_L

-----KLTKYLFFTGKGGVGKTTSACATAVNLADSGKKVILVSTDPASNLQDVFQTELTNKPKVI-----

LKVANFDPVTAANDYKESIVGPYRG-LPDSAVENMEEQLSGSCTVEIAAFNEFAGFLTD-----SYDYVV

FDTAPTGHTLRMLALPSAWSNYLDENDTG-SCL--LSGLGDKKDSYEQAVKTLSDVTLTTLMLVTRPQKA

AIQEASRASKELSDLGIHNQKLIINGLLD-----DDEISEIIYNQQRNDLDNLP-Q-EDYEQLYIPLRPY

NVTGLDKIRVLLSK---------------------EIALTNSNSYPKKIIFTMGKGGVGKTAIKIAQAL-

--KESGKKHLATTDPADHLNFYL-GDTSGLSLSHIDEEKELREYKEEVLAKAREMSGDD-FDYVKEDLES

PCTQEIAVFRAFAEIV-EKADEIVVIDTAPTGHTLLLLESTQSY----ARTGEEAIQKLLP--RLQNLDE

TEVLMVTLPETTPVYESMRLADDLDRANISHTWWLVNQSMVATHTTNAVLKARASNEIEWIEKVAKLSDN

KEKWQPDFEK-------------

>A2RKM7|A2RKM7_L

YDPEL--TKYLFFTGKGGVGKTTSACATAVSLADSGYKVILVSTDPASNLQDVFQTELTNKPKEI-----

LKVANFDPVSAANDHKESIVGPYRG-LPDSAIENMEEQLSGSCTVEIASFNEFAGFLE-------FDYVI

FDTAPTGHTLRMLALPSAWSNYLDENDTG-SCL--LSGLGDKKESYERAVKTLANGELTTLMLVTRPQKA

SIEEAKRASGELAELGINNQKLIINGLLT-----DDEVSKIIAKQQEDDLEHLP-N-FKYEQFFIPLRPY

NVTGLDKIRILLDV---------------------ELPEIDSEMYPKKIIFTMGKGGVGKTTVAIKVAQA

L-QKQGKKHLATTDPADHLNFYL-GDTSGLSLSHIDEEKELREYKEEVLSKAREMSGDD-FDYVKEDLES

PCTQEIAVFRAFAEIV-EKADEIVVIDTAPTGHTLLLLESTQSY----ARTGEKSIQKLLP--RLQNSDE

TEVLMVTLPEATPVYESMRLADDLDRANIAYTWWLVNQSMSATHTSNEVLKARASNEVEWINKVAKLSNQ

HAKWQADFEK-------------

>G2KBK6|G2KBK6_L

-----IQTKYLFFTGKGGVGKTTTACATATSLAQDNKKVMLVSTDPASNLQDVFQTTLTNKPTPI-----

LQVANFDPITAAAEYKESIVGPYRG-LPDSALANMEEQLSGSCTVEIAAFNEFANFLTD-----EFDYVI

FDTAPTGHTLRMLQLPSAWNNYLDENTTG-SCL--LSGLGDKKDMYEKAVETLTDAEQTTLILVTRPQKA

PLIEAERASEELRKLGIQNQKLVVNGLLE-----DDEISQLIYQEQTHDLENMP-E-PDFDTFYIPLRPY

NVTGIDKLQILLSQ---------------------ALEEQEKEVFPKKIIFTMGKGGVGKTAIKIAKKL-

--AQEGKKHLATTDPADHLNMFI-SDDLPISISHIDEEKELADYKEEVLSKARAMNDDD-VAYVEEDLRS

PCTQEIAVFRAFAEIV-DKSDEIVVIDTAPTGHTLLLLDSTQSY----ARSGEVSIQKLLP--RLQNSDD

TEVLMVTLPETTPVYESMRLDEDLDRAKISHTWWLVNQSMYAADTQNDVLKARSFNELEWIEKVAELSNG

KEEWQPDFSPVGV----------

>F4FXL8|F4FXL8_L

YSPQENLTKYLFFTGKGGVGKTTVASATAISLADAGHRVMIVSTDPASNLQDVFKVSLTNQPKSIPNISG

LFAANFDPVIAANEYREQVIQPYRGVLPKEAIQNMAEQLSGSCTVEIAAFNEFANFLTSSKINQQFDYII

FDTAPTGHTLRMLQLPSAWSNYLDKNDRGASCLGQLAGLNDKKAMYQKAVETLGNPQATTLFLVTRPQKG

ALLEAQRASHDLAALNIKNQQLIINGILN--Q-PTDAVSQTIFKQQQADLQNMPVTLDQLPKLAIPLRAY

NVLGLANLRLLLKQQP--QITEEVTA--SH-FPDLDVVVKNLVQSNKKIIFTMGKGGVGKTTVAVQIAQK

IAAQHKTVHLATTDPADHLKYFK---SPLIKVSHIDEKKSLKEYQNEVLTTAKKTMKSNDVDYVAEDLRS

PCTQEIAVFRAFAELVAQNDSDVVVVDTAPTGHTLLLLNSTQSYAQEVAHTGSQAVVNLLP--RLQDPKQ

TEIVMVTLPETTPVYESMRLDDDLKRASLAHTWWVVNQSMLATQTTDPCLLARVQSEVKWIDQVKELSNN

HFAVMQWQPNYEKTLLTV-----

>D0DTD4|D0DTD4_L

YSPQENLTKYLFFTGKGGVGKTTVASATAISLVDAGHRVMIVSTDPASNLQDVFKVSLTNQPKPIPNISG

LFAANFDPVIAANEYREQVIQPYRGVLPKEAIQNMAEQLSGSCTVEIAAFNEFANFLTSSKINQQFDYII

FDTAPTGHTLRMLQLPSTWSNYLDKNDRGASCLGQLAGLNDKKAMYQKAVETLGNPQATTLFLVTRPQKG

ALLEAQRASHELAALNIKNQQLIINGILN--Q-PTDAVSQTIFKQQQADLQNMPVTLDQLPKLAIPLRAY

NVLGLANLRLLLKQQP--QITEEVTA--SH-FPDLDVVVKNLVQSNKKIIFTMGKGGVGKTTVAVQIAQK

IAAQHKTVHLATTDPADHLKYFK---SPLIKVSHIDEKKSLKEYQNEVLTTAKKTMKSNDVDYVAEDLRS

PCTQEIAVFRAFAELVAQNDSDVVVVDTAPTGHTLLLLNSTQSYAQEVAHTGSQAVVNLLP--RLQDPKQ

TEIVMVTLPEATPVYESMRLDDDLKRASLAHTWWVVNQSMLATQTTDPCLLARVQSEVKWIDQVKELSNN

HFAVMQ-----------------

>F6IYS9|F6IYS9_L

YQPQNNLTHYLFFTGKGGVGKTTTASATAINLADAGNQVMLVSTDPASNLQDVFNTALTNKPQAINGVSG

LFAANFDPVTAAGEYRESVVGPYRGVLPDAAVKNMEEQLSGSCTVEIAAFNEFANFLTDPAVDQRFDYII

FDTAPTGHALRMLQLPSAWNNYLDENDRGASCLGQLAGMGDKKAIYAKAVATLSNGDLTTLMLVTRPQKA

SLLEAARAAQELAAIGMTNQQLIINGTLK--T-PTDHASQAIFAQQQADLQQMPAVLKKFAQYEVPLRAY

NVTGLNKLHLVLQTQP--ALATYPLT--NN-YPNLDTIVADLIKTDKKIIFTMGKGGVGKTTVAVQITQK

LVAQHKTVHLATTDPADHLDFFK---DPAVTISHIDEQQVLKDYQAEVLATARQTMKAADVDYVAEDLRS

PCTQEIAVFRAFANIVAQNDSDVVVIDTAPTGHTLLLLDSTQSYAQEVKRTGDQAIIDLLP--RLQDPQQ

TEIVMVTLPETTPVYESMRLNEDLNRAQIAHTWWLVNQSMLATQTTHPCLQARAQNEVEWIEKVKDVSAN

HFAVEQWQPDFEQTLLTI-----

>C7HWH9|C7HWH9_9

FSPEKKLGKYIFFTGKGGVGKTSTACATAVNLCEKGSKVLLVSTDPASNLQDIFVEKLDNEITKIEEVEG

LFVANLDPIKAADEYKKSIVSPYIGKLPDSVINKMEEELSGSCTVEIAAFNEFAKYITDKETEDKYDFII

FDTAPTGHTLRMLQLPQAWDNFIEDNTQGVSCLGQLSGLESQKGMYKKAVETLSDKYKTSLILVTRPEDG

PLKEADRASKELKDIGIKNQILLVNGLLT--I-YDDKISKVYYEKQKESLEKMPKNLKKLQNYLIPLRGY

NITGISNLRALFDDTY--TEKTFKEI--KI-DKKLKDIIDDLYKNNKKVIFTMGKGGVGKTTVAAAIALG

LSKKGKKVHLTTTDPADHLKYTI---NENLSVSHIDEKLELEKYRKEVLQKAKETMSNDDLSYIEEDLRS

PCTQEIAVFRAFADIVEKSEEEIVVIDTAPTGHTLLLLESTQSYNQEIMRSGDESTKKLLP--RLKNEKE

TEVIIVTLAEPTPVYEALRLEEDLKRAGIFSKWWLINSSLYASNTTNKILKSKANEEVKWINYLNDHTKE

NLAIIGWNPKKLSGDVLEDLIK-

>B6W9R1|B6W9R1_9

FSPDKKLGKYIFFTGKGGVGKTSTACATAVNLCDKGSKVLLVSTDPASNLQDIFVEKLNNKITKIEEVEG

LFVANLDPIKAADEYKKSVVSPYIGKLPDSVINKMEEELSGSCTVEIAAFNEFAKYITDKETKEKYDYIV

FDTAPTGHTLRMLQLPQAWDNFIEENTQGTSCLGQLSGLESQKGMYKKAVETLSDNDKTSLILVTRPEDG

PLKEADRASKELKDIGIKNQILLVNGLLT--I-YDDQISKAYYEKQKESLEKMPENLKKLQKYLIALRGY

NITGISNLRALFDDSY--TENNLDEI--KI-DKKLKDIIDDLYKNDKKVIFTMGKGGVGKTTVAAAIALG

LSKKGKKVHLTTTDPADHLKYTI---NENLSISHIDEKLELEKYRKEVLQKAKETMSDDDLSYIEEDLRS

PCTQEIAVFRAFADLVEKSEEEIVVIDTAPTGHTLLLLESTQSYNQEIMRSGDEATKKLLP--RLKNEKE

TEVIIVTLAEPTPVYEALRLEEDLKRAGIFNKWWLINSSLYASNTSNKILKSKANEEVKWINYLDEHTNK

NLALIAWNPKKLSGEILEDIID-

>E3GHY6|E3GHY6_E

FNPENGLTKYLFYTGKGGVGKTSTACATAVSLADKGKKVFLISTDPASNLQDVFETELDNKGVSIKQVPN

LVVANLNPEEAAAEYRESVVGPYRGKMPESIIANMEEQLSGSCTVEIASFNEFAHFITDDKINNQYDYII

FDTAPTGHTLRMLQLPSAWDSFIDESTHGASCLGQLSGLGDKKEMYAHAVKTLSDKDMTTLILVSRPEKA

PLDEAARASKELSDIGVNNQELIINGVLERVN-NSDPVSESFFEKQQKAMAGIPESLKAMPVFVLPLRSY

NVSGIDNIRRMLIDDL--SELDDNNN--FE-FPGLTTLINDLHKSKKKVIFTMGKGGVGKTTIAAAIALG

LAKFGEKVHLTTTDPAGNISAQA---NRNVEVSNIDEAEELQKYKDEVISKAIAGVSKEDLDYIKEDLRS

PCTQEIAVFRAFADIVEKAEEKVVVIDTAPTGHTLLLLNSTESYHKEMERSAEESVMKLLP--RLKNKNE

TEVVIVALPEATPYFEAYRLEEDLNRADIDNKWWVINASLLAADTNDPFLKARAANEEKWINRIAEETKG

NYVVIPWQK--------------

>F8X0N1|F8X0N1_9

YNPSVDLTKYIFFTGKGGVGKTSIACATAVNLADSGKKVLLISTDPASNLQDVFNMDLDNKGKQIKEVPN

LTVVNLDPEQAAAEYRESVIAPFRGKLPESVITNMEEQLSGSCTIEIAAFNEFSDFITDERKRKDFDFII

FDTAPTGHTLRMLQLPSAWDTFIAENTTGASCLGQLSGLEDRKEVYKNAVNTLSDSKQTTLILVGRPDES

PLKEVERSSKELLDLNIISHNLIINGVLENYD-DNDTVSKQIYQRQQNALNSRSEVLLKLKTYFVPLRSY

NMTGIDNIRNMLND-M--QIETKAEIDTKG-FQTIDNIIDDLYNSGKRVIFTMGKGGVGKTTIAGNIARG

LAQKGVKVHLTTTDPANHLSFIE---VEGITVSHIDERAVLAAYQKNVLEKARETMGDADLSYIEEDLRS

PCTQEIAVFNSFAEIVAKADNEVVVIDTAPTGHTLLLLDSTQSYHKEVERTGNPAVKNLLP--RLRNEKE

TEVVIVTLPETTPVFEAQRLQQDLGRAGIKNKWWLVNSSLLLTPTISPFLKAKAQSEIQWIEKVNEISNG

NFAVIEWKDKV------------

>B3C8P0|B3C8P0_9

FNLSDDFTKYLFFTGKGGVGKTSIACATAVGLADKGKKILLISTDPASNLQDVFNQSLNGHSSEITEIPG

LTVVNLDPEQAAAEYRERVIAPFRGKLPESVIQNMEEQLSGSCTVEIAAFNEFSDFITDAQKQLEFDHII

FDTAPTGHTLRMLQLPSAWSTFISESTHGASCLGQLSGLEERKEIYKKAVETLSDTGTTRLMLVSRPEIA

PLKEAARSSHELQLLGIKNQILIINGVLKQLD-KDDSVSSQLHERQQKALQSMPAELSGYPLYHVPLRSY

NLSSIADIRRMLYDSL--FNEVNKPV--AA-PKDVDEMVDDLYTSGKRVIFTMGKGGVGKTTLATEIALK

LTRLGAKVHLTTTDPANHLNYEL---RAGITVSRIDEAEVLEKYKNEVRSKAAEAVTAEDMEYIEEDLRS

PCTQEIAVFKAFAEIVDKAETEIVVIDTTPTGHTLLLLDATQSYHKEVERTGEGAVANLLP--RLRNSKE

TEVVIVTLPEATPVFEAERLQMDLQRAGINNKWWVVNACLSLTDTQNLFLKAKAQNELAWIKKVEQLSQG

NAALIEWRNV-------------

>A6L7X3|A6L7X3_B

FNLSDELTKYLFFTGKGGVGKTSIACATAVGLADNGKKILLISTAPASNLQDVFNQTLNGHGTDIQEVPG

LTVVNLDPEQAAAEYRESVIAPFRGKLPDSVIQNMEEQLSGSCTVEIAAFNQFSDFITDADKAKEYDHII

FDTAPTGHTLRMLQLPSAWSTFISESTHGASCLGQLSGLEERKGIYKQAVETLSDANATRLVLVSRPEIA

PLKEAARSSHELQLLGINNQLLVINGLLLQLD-EADSVSKQIYDRQQTALKQTPAELLDYPSYSVPLRSY

NLSNIANIRRMLYDNL--TDNADQRI--TD-AKGIDELVNDLYQSGKRVVFTMGKGGVGKTTLATEIALK

LTKLGAKVHLTTTDPANHLNYNL---QAGITVSRIDEAEVLEAYKNEVRSKAAETMTAEDMEYIEEDLRS

PCTQEIAVFRAFAEIVDKAENEIVVIDTAPTGHTLLLLDATESYHKEVQRTGDASVRKLLP--RLRNQQE

TEVVIVTLPEATPVFEAERLQKDLQRAGINNKWWVVNACLSLTDTENSFLRAKAQNELTWIKKVEELSKG

NAALIAWKNN-------------

>F4KML3|F4KML3_P

FNLSDDLTKYLFFTGKCGVGKTSIACATAVGLADNGKKILLISTDPASNLQDVFNQTLNGHGTDIQEVPG

LMVVNLDPEEAAAEYRESVIAPFRGQLPESVIQNMEEQLSGSCTVEIAAFNQFSDFITDADKAKEYDHII

FDTAPTGHTLRMLQLPSAWSTFISESTHGASCLGQLSGLEERKGIYKQAVETLSDANATRLVLVSRPEIA

PLKEAARSSHELQLLGIKNQLLVINGLLLQLD-ETDNVSKQIYDRQQNALKQTPAELLEYPSYYVPLRSY

NLSNIANIRQMLYDDL--TNDANQRI--TD-AKGMDELVNDLYQSGKRVVFTMGKGGVGKTTLATEIALK

LTKLGAKVHLTTTDPANHLNYNL---QAGITVSRIDEAEVLEAYKNEVRSKAAETMTAEDMEYIEEDLRS

PCTQEIAVFRAFAEIVDKAEKEVVVIDTAPTGHTLLLLDATESYHKEVQRTGDASVRKLLP--RLRNPQE

TEIVIVTLPEATPVFEAERLQMDLQRAGINNKWWIVNACLSLTDTENSFLRAKAQNELVWIKKVEELSKG

NAALIPWKNN-------------

>F3XNK3|F3XNK3_9

FNLSDDLTKYLFFTGKGGVGKTSIACATAVGLADNGKKILLISTDPASNLQDVFNQTLNVHGTDIQEVPG

LTVVNLDPEQAAAEYKEGVIAPFRGQLPESVIQNMEEQLSGSCTVEIAAFNQFSDFITDADKAKEYDHII

FDTAPTGHRLRMLQLPSAWSTFISESTHGASCLGQLSGLEERKGIYKQAVETLSDANATRLVLVSRPEIA

PLKEAARSSHELQLLGIRNQLLVINGLLLQLD-EADNVSKQIYDRQQNALKQIPAELLEYPSYYVPLRSY

NLSNIANIRRMLYDNL--TNDANQRI--TD-AKGMDELVNDLYQSGKRVVFTMGKGGVEKNTLATEIALK

LTKLGAKVHLTTTDPANHLNYNL---QAGITVSRIDEAEVLEAYKNEVRSKAAETMTAEDMEYIEEDLRS

PCTQEIAVFRAFAEIVDKAEKEVVVIETAPTGHTLLLLDATESYHKEVQRTGDASIRKLLP--RLRNP--

----------------------------------------------------------------------

-----------------------

>D9RXF4|D9RXF4_P

FNLSDDLTKYLFFTGKGGVGKTSIACATAVGLADNGKKILLISTDPASNLQDVFNQTLNGHGTDIQEVPG

LTVVNLNPEQAAAEYREGVIAPFRGQLPESVIQNMEEQLSGSCTVEIAAFNQFSDFITDADKAKKYDHII

FDTAPTGHTLRMLQLPSAWSTFISESTHGASCLGQLSGLEERKGIYKQAVETLSNANATRLVLVSRPEIA

PLKEAARSSHELQLLGIKNQLLVINGLLLQLD-EADNVSKQIYDRQQNALKQTPVELLEYPSYYVPLRSY

TLSNIANIRRMLYDNL--TNDANQRI--TD-AKGMDELVNDLYQSGKRVVFTMGKGGVGKTTLATEIALK

LTKLGAKVHLTTTDPANHLNYNL---QAGITVSRIDEAEVLEAYKNEVRSKAAETMTAEDMEYIEEDLRS

PCTQEIAVFRAFAEIVDKAENEVVVIDTAPTGHTLLLLDATESYHKEVQRTGDASIRKLLP--RLRNQQE

TEVVIVTLPEATPVFEAERLQMDLQRAGINNKWWVVNACLSLTDTENSFLRAKAQNELVWIKKVEELSKG

NAALIAWKNN-------------

>C6IGB1|C6IGB1_9

FNLSDALTKYLFFTGKGGVGKTSIACATAVGLADMGKKILLISTDPASNLQDVFDQSLNGHGTAISEVPG

LTVVNLDPEQAAAEYRESVIAPFRGKLPESVIQNMEEQLSGSCTVEIAAFNEFSDFITDAVKAKEYDHII

FDTAPIGHTLRMLQLPSAWSTFISESAHGASCLGQLSGLEERKGIYKQAVETLSDTSATRLVLVSRPEIA

PLKEAARSSHELQLLGIKNQLLVINGILQQLN-EADDVSRQLHNRQQKALQGMPAELSEYPMYSVPLRSY

NLSDIANIRRMLYDSL--TDDICQPI--SD-AKSIDDLVNDLYTSGKRVVFTMGKGGVGKTTLATEIALK

LIKLGAKVHLTTTDPANHLNYDI---KSGITVSHIDEAEVLENYKNEVRSKAAETMTAEDMEYIEEDLRS

PCTQEIAVFKAFAEIVDKADNEIVVIDTAPTGHTLLLLDATQSYHREVERTGAGAVANLLP--RLRNPKE

TEVVIVTLPEATPVFEAERLQMDLQRAGINNKWWAVNACLSMTNTENTFLQAKAQNEVNWIEKVEQLSKG

NAALIGWKNI-------------

>Q8A9L3|Q8A9L3_B

FNLSDELTKYLFFTGKGGVGKTSIACATAVGLADKGKKILLISTDPASNLQDVFDQSLNGHGTAISEVPG

LTVVNLDPEQAAAEYRESVIAPFRGKLPESVIQNMEEQLSGSCTVEIAAFNEFSDFITDADKAKEYDHII

FDTAPTGHTLRMLQLPSAWSTFISESTHGASCLGQLSGLEERKGIYKQAVETLSNTSATRLVLVSRPEIS

PLKEAARSSSELQLLGIKNQLLVINGILQQLN-EADDVSRQLHNRQQKALQGMPAELSEYPMYSVPLRSY

NLSDIANIRRMLYDSL--ADDICQPV--SG-AKSIDDLVNDLYTSGKRVVFTMGKGGVGKTTLATEIALK

LTKLGAKVHLTTTDPANHLNYDL---KSGITVSHIDEAEVLENYKNEVRSKAAETMTAEDMEYIEEDLRS

PCTQEIAVFKAFAEIVDKADNEIVVIDTAPTGHTLLLLDATQSYHKEVERTGAGAVANLLP--RLRNPKE

TEVVIVTLPEATPVFEAERLQMDLQRAGINNKWWVVNACLSMTNTENSFLQAKAQNEVNWIKKVEQLSKG

NAALIGWKNI-------------

>D6D3B8|D6D3B8_9

FNLSDELTKYLFFTGKGGVGKTSIACATAVGLADKGEKILLISTDPASNLQDVFNQTLNGHGTAISEVPG

LTVVNLDPEQAAAEYRESVIAPFRGQLPESVIQNMEEQLSGSCTVEIAAFNEFSDFITDADKAKEYDHII

FDTAPTGHTLRMLQLPSAWSTFISESTHGASCLGQLSGLEERKGIYKQAVDTLSDTSATRLVLVSRPEIA

PLKEAARSSHELQLLGIKNQLLVINGVLQQLD-EADNVSQQLYNRQQKALQSMPIALSEYPMYSVPLRSY

NLSNIANIRRMLYDSI--TNEIRQPI--TD-SKSIDELVNDLYTSGKRVVFTMGKGGVGKTTLATEIALK

LTKLGAKVHLTTTDPANHLNYDF---KSGITVSHIDEAEVLEKYKNEVRSKAAETMTAEDMEYIEEDLRS

PCTQEIAVFKAFAEIVDKAENEIVVIDTAPTGHTLLLLDATQSYHKEVERTGEGAVANLLP--RLRNPQE

TEVVIVTLPEATPVFEAERLQMDLQRAGINNKWWVVNACLSLTNTANSFLQAKAQSELTWIKKVEELSKG

NTALIEWKNL-------------

>B7ACT3|B7ACT3_9

FNLSDELTKYLFFTGKGGVGKTSIACATAVGLADKGEKILLISTDPASNLQDVFNQTLNGHGTAISEVPE

LTVVNLDPEQAAAEYRESVIAPFRGQLPESVIQNMEEQLSGSCTVEIAAFNEFSDFITDADKAKEYDHII

FDTAPTGHTLRMLQLPSAWSTFISESTHGASCLGQLSGLEERKGIYKQAVETLSDTNATRLVLVSRPEIA

PLKEAARSSHELQLLGIKNQLLVINGILQQLD-EADNVSQQLHDRQQKALQSMPVAISEYPMYSVPLRSY

NLSNVANIRRMLYDSL--TDNSCQPI--TD-AKSIDELVNDLYTSGKRVVFTMGKGGVGKTTLATEIALR

LTKLGAKVHLTTTDPANHLNYDF---KSGITVSHIDEAEVLEKYKNEVRSKAAETMTAEDMEYIEEDLRS

PCTQEIAVFKAFAEIVDKAENEIVVIDTAPTGHTLLLLDATQSYHKEVERTGEGAVANLLP--RLRNPKE

TEVVIVTLPEATPVFEAERLQKDLQRAGINNKWWVVNACLSLTNTKNSFLQAKAQSELTWINKVEELSKG

NTALIEWKNL-------------

>G2HDP9|G2HDP9_9

----------------------------------------------------------------------

-------------------MAPYRGKLPDAVIASMEEQLSGSCTVEIAAFDQFSNFITDQGLSEAYDHII

FDTAPTGHTLRMLQLPSAWSNFISESTHGASCLGQLAGLEGKQGMYRHAVATLADGGTTTLVLVSRPETA

PLLEADRSSAELRELGIENQMLVVNAVLD--Q-ATDDVSSRILERQQAALRAMPARLKDIRAYGIPLRSY

NIIGLAGIRAFLNELV--ETRRSVPQ--SP-AHTLGNLVDALHETGKKVIFTMGKGGVGKTMVAAAIAMG

LAQRGAKVHLTSTDPANNLQNVM---TPGVTVSRIDEQEELGRYRDEVLGAARQVMSGNDLAYIEEDLRS

PCTQEIAVFRAFAEIVEKAGEEVVVIDTAPTGHTLLLLDATQSYHKEVQRTGVPSVEKLLP--RLRDARQ

TEVVIVTLPEATPVFEAERLREDLLRAGIRNTWWVVNQCLSAVETTSDILRARANEETNWINRVSAICSG

NIVTVPWKHAPAVDELLGDS---

>B8DIN3|B8DIN3_D

FDLGSPLTKYLFFTGKGGVGKTSVACAVAVTLADQGKRILLISTDPASNLQDVFDTELDGHGVPIQGVDG

LVVANLDPEEAAREYRESVVAPYRGKLPDSAIASMEEQLSGSCTLEIAAFDQFTTFLTNDAINREYDHII

FDTAPTGHTLRMLQLPSAWSSFISESTHGASCLGQLAGLEGKQQMYRHAVGALGDAAATTLVLVSRPDAA

PLKEADRSSAELRELGIRNQILIINAVLE--N-PTDAVSGKLHAKQQAALRDMPQQLAEIRTYTIPLVPY

TILGLDALRAFLSTRP--EQPAAEHP--ER-TRTLCDLVADLHATGKKVIFTMGKGGVGKTTIAASIALG

LARKGAKVHLTSTDPASDLQTIL---PANITLSRIDEKEELARYRKEVLDTAMQVMSKDDVAYIEEDLRS

PCTQEIAVFRAFAEIVEKAGEEIVIIDTAPTGHTLLLLDATQSHHKEVQRTGIPSVRNLLP--RLRDPQQ

TEVVIVTLPEATPVFEAERLQADLRRAGIHTQWWVVNQCFSLLETDSPILRHRAAAEAGWINKVNALSAG

HTATIPWMHDTPVTRLLDTL---

>G2HAM9|G2HAM9_9

FDLGSPLTKYLFFTGKGGVGKTSVACAVAVTLADQGKRILLISTDPASNLQDVFNTELDGRGVPIHGVDG

LVVANLDPEEAAREYRESVVAPYRGKLPDSAIASMEEQLSGSCTLEIAAFDQFTTFLTDDAINREYDHII

FDTAPTGHTLRMLQLPSAWSSFISESTHGASCLGQLAGLEGKQAMYRHAVDALGDAAATTLVLVSRPDAA

PLEEAGRSSAELRDLGIKNQILIINAVLE--N-PTDAVSGKLHAKQQAALRNIPQQLAGIRTYAIPLVPY

NILGLDGVRAFLTKHP--AQPATEHP--EQ-TRTLGDLVADLHATGKKVIFTMGKGGVGKTTIAAVIALG

LARKGAKVHLTSTDPASDLQTIM---SPNITLSRIDEQEELHRYRKEVLDTAMQVMSKDDIAYIEEDLRS

PCTQEIAVFRAFAEIVDKADEEVVIIDTAPTGHTLLLLDATQSHHKEVQRTGIPSVRNLLP--RLRDPRQ

TEVVIVTLPEATPVFEAERLQADLRRAGIHTQWWVANQCFSRLETDSSILRHRATAEAGWINKVNALSAG

HTATIPWVHDEPVNRLLDSL---

>Q893D3|Q893D3_C

FNADKDLTKYLFYTGKGGVGKTSIACATAVSLADSGKKVLLISTDPASNLQDVFHTELSNKETKIKETPN

LSVVNLNPEEAAREYRDSMINPYKGKLPEAVLKNMEEQLSGSCTVEIAAFNEFSNYLTDKNIENEYEFII

FDTAPTGHTLRMLQLPSAWTNFISESTHGASCLGQLSGLEDRKEMYKEAVQTLANGKLTTLILVARPESS

TLLEAERASDELSQLGINNRMLIINGVMQTVN-KDDEVSLEFYNKQQSTLKNIPNAIKDIKTYAVPLRSY

NVSGIDSIRMMLKDSE--VITTELDN--ID-FPSINDLINDLYKSKKKVIFTMGKGGVGKTTVASTIALA

LSQKGVKVHLTTTDPSNHIKYIM---HKNITISEINEQEELKKYQNEVISKASETMSGEDLEYIKEDLRS

PCTQEIAVFRAFAEIVEKADDEVVVIDTAPTGHTLLLLDSTQSYHKEVERTGDKSVKKLLP--RLRDENE

TDVVIVTLPEATPVYEAERLNTDLKRAGIHSKWWVINSSLLLTGTKSTLLQSKAQSEIKWINEVNKYSNG

NYTVVKWLPKEVNDTTLLEIIKL

>D2RLF5|D2RLF5_A

YDPFAIRTQYVFFTGKGGVGKTSTACATAVALADAGQKVLLVSTDPASNLQDIFSLELKEKPTPIPGVPG

LEAANLDPVKAAAAYRESVVGPYRGILPEAALRNMEEQLSGSCTIEIAAFNAFTDFLTDPETATAYDKII

FDTAPTGHTLRMLQLPSAWSQFISTSTHGASCLGQLSGLESRKALYRQAVETLADPGRTTLLLVARPDRP

PLLEADRASRELAKLGIGNQQLIINGLLE--K-TDDPVTKELFAKQQMALAEMSQGLKILPSWQIPFRSY

NVTGLDHVRALLKEVA--PVAGREQQ--AP-VYTLQEVAEDLERNHRKVIFTMGKGGVGKTTVAASLALR

LAQKGHQVLLTTTDPAGHLQEIL---TDRLQLCQIDEQAELRRYQKEVLDQARKGLKEGDLAYIEEDLRS

PCTQEIAVFRAFAEIVARSGDRIVIIDTAPTGHTLLLLESTENYDREIRRTGAPAVQQLLP--RLKG-PE

TEVVIVTLPEATPVYEALRLEADLKRTRLYSRWWIINRCFSQTAAQSPLLKAKADREIPWINQVAAHASG

CAAVIPWKQAEIKGKVLETL---

>G0VLQ0|G0VLQ0_M

YDPFRVQTKYLFLTGKGGVGKTSVACATAVALADAGKKVLLISTDPASNLQDVFSMELTNKATAIDAVPN

LAVANLDPVQAAAEYRESVIGPYRGLLPEAALRNMEEQLSGSCTVEIAAFNEFTNFLTDETVARDYDHIL

FDTAPTGHTLRMLQLPSAWSQFIQKSKHGASCLGQLSGLESRKELYKQAVQTLADGAQTTMLLVARPDSL

PLKEAARASKELAALGIANQQLIINGLLP--S-YDDDATKTLYEKQQAALQAMPGAISDLPRFQIPLRTY

NITGLDHVRALLKEVK--EAATELTL--EW-VQPLQAVIDDLESSHRKVIFTMGKGGVGKTTVAAAIALG

LARRGHRVHLTTTDPAAHLQYIV---TDNLTLSHIDEGEELRKYQDEVLNQAKAGLGPSDLAYIEEDLRS

PCTQEIAVFHAFADIVEAADDQIVVIDTAPTGHTLLLLESTESYDKEIRRTGSPSVQHLLP--RLKG-KE

TEVVIVTLPEATPVYEALRLEADLKRTKLRSKWWVINKSFYQAKTTSPLLKAKASHEIPWINKVDEHTKG

HTALIAWRPDEVRGDVLGTL---

>F6DK49|F6DK49_D

FDISKKLTKYLFFTGKGGVGKTSTACAVAVSLADSGKNVLLISTDPASNLQDVFNIALNGKGVPVEGAPG

LVVANLNPEEAAREYRESVIAPYRGKLPGSVIANMEEQLSGSCTVEIAAFDQFSNFITDRATEDQYDNII

FDTAPTGHTLRMLQLPSAWSHFISESTHGASCLGQLAGLEDKKDMYKDAVANLADKDKTTLILVSRPEKT

PLLEADRSSLELRDLGINNQLLILNGVLA--E-ASDDVSQGIYDKQQEALENMPANLKQLKAFTLPLRSY

NILGLDKIRAFFKDHY--LSSNTIKN--LD-LKPMDGLINDIYASGKKVIFAMGKGGVGKTTIAAAIALA

LAKKGVKVHLTSTDPADHLQYVI---AENITLSKIDEKQELLNYQTEVLSKARETMSEDDIAYVEEDLRS

PCTQEIAVFRAFAEIVDKAENEVVVIDTAPTGHTLLLLDSTQSYHREVQRSGELSVQKLLP--RLRDERQ

TEVVIVTLPEATPVFEALRLREDLSRAGINNKWWVVNQCLSMTDTCNAMLAARSGAEKQWLEKVKQISKG

YFATIPWLVDASVQSISQKYLTG

>Q0AYF1|Q0AYF1_S

FDIDKNLTQYLFFTGKGGVGKTSAACAVAVNLADSGKKVLLISTDPASNLQDVFNTELNGKGVQIEGVPG

LVVANLDPEEAAREYRESVISPYRGKLPDSVINNMEEQLSGSCTIEIAAFDQFSHFITDNTSENEFDYII

FDTAPTGHTLRMLQLPSAWSNFIAESTHGASCLGQLAGLQDKKDMYKNAVINLADKAKTTLILVSRPEET

PLLEAERSSRELSNLGINNQLLIINGVLG--G-ASDDLSQKILSKQQHALKNLPPGLRGFKTYTIPLRSY

NILGLDKIRAFLNDDY--ISADASKL--TD-LKPINVLVEDVYAAGKKVIFTMGKGGVGKTSVAATIAVA

LSKKGVKVHLTSTDPADHLSYVF---AENITVSHIDEKKELKDYQNEVLAKARETMSEDDVAYIEEDLRS

PCTQEIAVFRAFAEIVDKAENEVVIIDTAPTGHTLLLLDSTLSYHREVQRTGEISVQRLLP--RLRDEKQ

TEVVIVTLPEATPVFEAIRLREDLSRAGINNKWWVVNQSLSVADTSNPMLVARAEAERTWIEKVQQISDD

NFVVIPWLQDPSIKSIG-NFKD-

>F7Z2M5|F7Z2M5_B

FSLDGNLTKYMFFTGKGGVGKTSTACAVAVNLADNGKSVLLISTDPASNLQDVFNTELDGKGVPIDGVPG

LVVANLDPEEAAREYRESVIAPYRGKLPDSVIVNMEEQLSGSCTVEIAAFDQFSNFITDKSTENKYDYII

FDTAPTGHTLRMLQLPSAWSNFISESTHGASCLGQLAGLQDKKDMYKNAVENLADKDKTTLILVSRPEET

PLIEAERSSHELSELGINNQVLIINGILS--E-ATDDVSIKMLDKQQKALENMPQGLKKFKIFTIPLRSY

NVVGIDNIRTFLYDEY--TKNSISKS--LN-LRHLDVLIEDIYRAGKKVIFTMGKGGVGKTTIAATIAVA

LARKGVKVHLTSTDPADHLKYVV---TENIKLSKIDEKKELLRYQNEVLSKARETMSEDDIAYVEEDLRS

PCTQEIAVFRAFAEIVDKAENEVVIIDTAPTGHTLLLLDSTQSYHREVQRTGEVSVQRLLP--RLRDEKQ

TEVIIVTLPEATPVFEAQRLSDDLNRAGINNKWWVVNQCLSLTNTKNSMLIARADAEKQWLEKVKEISSD

NFVGIPWFQDASIESIV-NCSGG

>C7RFZ2|C7RFZ2_A

FDIKEDLTKYLFFTGKGGVGKTSTACASAISLADEGNEVLLISTDPASNLQDVFETELDNKGVRIEGVDG

LTVANLDPIEAANEYKESVVGPYRGKLPKSVIENMEEQLSGSCTVEIAAFNEFSKFITDADLKDKYDYII

FDTAPTGHTLRMLQLPSAWTSFISESTHGASCLGQLSGLEDEKETYKYAVDTLADGKLTSLVLVARPEET

PLLEANRASYELAELGINNQILIINGLLS--G-HDDEVSEAFYKKQKESLDKMPEGIKDLKTFFIPLRGY

NLNSIENLRSLLVDKE--NTSDVLNI--GE-IPRLKDIIDDLYKNKKKVIFTMGKGGVGKTTMASAIAKG

LTAKGEKVHLTTTDPANHLTGMI---DDLLTISHIDEEEELKKYQEEVLENARKTMSDEDLEYIKEDLRS

PCTQEIAVFRAFADVVDRADDEIVVIDTAPTGHTLLLLDSTESYNKEIEKNGNESAKKLLP--RLKNSDE

TEVLIVTLAEPTPFYESQRLEEDLKRAGIYSKWWIINSSIYKTGSRNKTLQAKANSEIEWIKKIDDRTDG

NFTIIPWSSDEIKGSSLDKLIK-

>F0GXI8|F0GXI8_9

FDIKEDLTKYLFFTGKGGVGKTSTACASAISLADMGNEVLLISTDPASNLQDVFETELDNKGVRIEGVAG

LTVANLDPIEAANEYKESVVGPYRGKLPASVIENMEEQLSGSCTVEIAAFNEFSKFITDADLKDKYDYII

FDTAPTGHTLRMLQLPSAWTSFISESTHGASCLGQLSGLEDEKETYKFAVDTLADGKLTSLVLVARPEET

PLVEANRASYELRELGINNQILIINGLLS--A-HDDEVSEAFYKKQKESLDKMPEAIKDLKTFFIPLRGY

NLNSIENLRSLLTDKD--YTSDQLNI--DE-TPSLKYIVDDLYKNEKKVIFTMGKGGVGKTTMASAIAKG

LTAKGEKVHLTTTDPANHLTGMI---DDLLTISHIDEEEELRKYQEEVLENARKTMSEDDLEYIKEDLRS

PCTQEIAVFRAFADIVDRADDEIVVIDTAPTGHTLLLLDSTESYNTEIEKNGNESAKKLLP--RLKNSDE

TEVLIVTLAEPTPFYESQRLEEDLKRAGIYSKWWIINSSIAKTGSRNKTLRAKANSELEWINKVDNRTDG

NFTIIPWSSGEIKGSNLDKLIK-

>F5UA48|F5UA48_S

FNIEDKLTKYLFFTGKGGVGKTSTACATAVSLADEGKKVLLISTDPASNLQDVFETELDGNAKPIKGVDN

LEVINLDPLEAAHNYKESVVGPFRGKLPDSVIENMEEQLSGSCTVEIAAFNEFSNFITNSKLNIDYDHII

FDTAPTGHTLRMLQLPSAWTDFISESTHGASCLGQLSGLEVKKETYKKAVENLSDENLTTLVLVTRPDKT

PLNEVARASKELSEIGIKNQILVINGVLE--N-HDDDLSESIFNKQKLALENMPDMLTEFDTYTIALRSY

NITGIDSIRNLLKDQI--NEQKVVK---GK-LFNLDDVVNDLVRNNRKVIFTMGKGGVGKTTIASSIALK

LSKLGKKVHLATTDPADHIKYMI---SSGISMSHIDEKEELKKYQDEVLENARETMSEDDVAYIEEDLRS

PCTQEIAVFRAFAELVDKADDEIVVIDTAPTGHTLLLLDSTQSYHQEVERTGQESVKKLLP--RLRG-EE

TEVLILSLAEATPFYEAYRLEEDLKRASIHTNWWLVNSSLYKANPTNKMLSAKANEEVKWINKILDHTSG

KLAVIEWTKEDLYGDKLYDI---

>F2BY05|F2BY05_9

FNIEDKLTKYLFFTGKGGVGKTSTACATAVNLADEGKKVLLISTDPVSNLQDVFETELDGKAKPIKGVDN

LEVINLDPLEAAHNYKESVVGPFRGKLPDSVIENMEEQLSGSCTVEIAAFNEFSNFITNSKLNTDYDHII

FDTAPTGHTLRMLQLPSAWTDFISESTHGASCLGQLSGLEAKKEIYKKAVENLSDESLTTLVLVTRPDKT

PLNEVARASKELSEIGVKNQILVINGLLE--K-YDDDLSESIYKKQQSALENMPEMLTEFDTYTIALRSY

NITGIDSIRNLLKDQI--NEQKVVK---GK-LFNLDDVVNDLVRNNRKVIFTMGKGGVGKTTIASSVALK

LSKLGKKVHLATTDPADHIKYMI---SSGISMSHIDEKEELKKYQDEVLENARKTMSEDDVAYIEEDLRS

PCTQEIAVFRAFAELVEKADDEIVVIDTAPTGHTLLLLDSTQSYHQEVERTGQESVKKLLP--RLRG-EE

TEVLILSLAEATPFYEAYRLEEDLKRASIHTNWWLVNSSLYKANPSNKMLSAKANEEVKWINKILDHNSG

KLAVVEWTKEDLHGDKLYDI---

>A4NIR5|A4NIR5_H

FNLANKLTKYLFFTGKGGVGKTSTACATAVALADEGKKVLLISTDPASNLQDVFETELDGKAKPIKGVDN

LEVINLDPLEAAHNYKESVVGPFRGKLPDSVIENMEEQLSGSCTVEIAAFNEFSNFITNSKLNTDYNHII

FDTAPTGHTLRMLQLPSAWSDFISESTHGASCLGQLSGLETKKETYKKAVENLSDKSLTTLVLVTRPDKT

PLNEVARASKELSEIGIKNQILVINCVLE--N-YDDDLSESIFNKQKLALENMPEMLTEFDTYTIALRSY

NITGIDSIRNLLKDQI--NEQKVVK---GK-LFNLDDVVSDLIKNNRKVIFTMGKGGVGKTTIASSVALK

LSKLGKKVHLATTDPADHIKFMI---SSGISMSHIDEKEELKKYQDEVLGNARKTMSEDDVAYIEEDLRS

PCTQEIAVFRAFAELVEKPDDEIVVIDTAPTGHTLLLLDSTLSYHQEVQRTGQESVKNLLP--RLRG-EE

TEVLIVSLAEATPFYEAYRLEEDLTRASIHTNWWIVNSSLYKANPSNKMLSAKANEEVKWINKILDHTSG

KLAVIEWTKEYLCGDRLYQI---

>C2L0D8|C2L0D8_9

FNLADKLSKYLFFTGKGGVGKTSTACATAVALADEGKKVLLISTDPASNLQDVFETELDGKAKPIKGVDN

LEVINLDPLEAAHNYKESVVAPFRGKLPDSVIESMEEQLSGSCTVEIAAFNEFSNFITNSKLNTDYSHII

FDTAPTGHTLRMLQLPSAWTDFISKSTHGASCLGQLSGLEAKKEIYKKAVENLLDKSLTTLVLVTRPDKT

PLNEVARASKELSEIGIKNQILVINGILE--K-YDDDLSESIFNKQKLALENMPDILTEFDTYTIALRSY

NITGIDSIRNLLKDQI--NEQKVVE---GK-LFNLDDVVSDLVKNNRKVIFTMGKGGVGKTTIASSVALK

LSKLGKKVHLATTDPADHIKFMI---SSGISMSHIDEKEELKKYQDEVLGNARKTMSEDDVAYIEEDLRS

PCTQEIAVFRAFAELVEKADDEIVVIDTAPTGHTLLLLDSTLSYHQEVQRTGQESVKNLLP--RLRG-EE

TEVLIVSLAEATPFYEASRLEEDLTRASIHTNWWIVNSSLYKVNPGNKMLSAKANEEVKWINKILDHTSG

KLAVIEWTKEDLCGDKLYQI---

>C9KLP2|C9KLP2_9

FALSKHLTQYLFFTGKGGVGKTSTACAVATSLADSGKRVLLVSTDPASNLQDVFAMELHSKPTPITEVRG

LSAANLDPIEAAAEYRESVIAPYRGVLPDAAIANMEEQLSGSCTIEIATFNAFANFITDEAIQKSYDHII

FDTAPTGHTLRILQLPSAWSNFINESTHGASCLGQLSGLEEKKAVYKTAVDTLADGGKTMLLLVTRPETA

PLQEANRASCELRELGIQNQLLIVNGVLM--Q-HDDALSSELYRKQQAALADMPAELKKLTQYAVPLRAY

PITGIANIRAMLTDCF--EQQQQIDR--TK-LHRLNDLVNTLDAENKKVIFTMGKGGVGKTTIAAAIALG

LAKRGKKVHLTTTDPAAHLKFVL---QDGITMSHIDEAAELKKYQEEVLSKARQGLSGEDIAYIEEDLRS

PCTQEIAVFRAFAEVVEKADDEIIVIDTAPTGHTLLLLESTENYDREIRRTGEESVKHLLP--RLKG-KE

TEVIIVTLPEATPVYEAMRLEDDLKRAGLSANWWVINQSFALTGSSNRTLAVKANNEAEWINKVDQHTHG

KTVLLPWHPEKVKGDKLLSL---

>A9KJA6|A9KJA6_C

FNPNSHLTKYLFYTGKGGVGKTSTACSTAVSLADQGKKVLLISTDPASNLQDVFETQLNSKGIAIPTVPG

LVVANLDPLKAAAEYRESVVAPYRDLLPEEVITNMEEQLSGSCTVEIAAFNEFSNFITDKEAQKEYDHII

FDTAPTGHTLRMLQLPSAWSGFISENTHGASCLGQLSGLEERKDVYKLAVSTLSNSELTTLILVTRPEES

PLKEATRASEELKELGIRNQILVVNGVLE--T-VDDSVSRSLYNKQKNALENMPEELKDIKTYMIPLRAY

NVMGIDNIRSFLSDSY--TMNSTIVS--NE-LSKLQDVVDDLYLSGKKVIFTMGKGGVGKTTVAAAIALG

LAEKGCKVHLSTTDPAAHLKYVI---QENISMSHIDEHAVLKKYQETVLSKARANLSEDDIAYIEEDLRS

PCTQEIAVFREFASIVERADNEIVVIDTAPTGHTLLLLDSTQSYHKEVQRTGDESVKKLLP--RLRNEKE

TAVMIVTLAETTPVFEALRLEEDLKRAGIQSKWWIINSSLYATQTTNPVLRAKASNEIEWINKVYEHATG

KVGVISWSPEEIKGDKLKDIIK-

>F0SUY3|F0SUY3_S

FDPGSKLTKYLFYTGKGGVGKTSVACATAVSLADSGKKVLLISTDPASNLQDVFSMELTNKGVPISDVPN

LVVANLDPIQAAAEYRESVIAPYRGKLPASVITNMEEQLSGSCTIEIAAFNEFSNFITDGKVQQEYDHII

FDTAPTGHTLRMLQLPSAWSNFISESTHGASCLGQLSGLESKKAIYKQAVETLADGSLTTLLLVTRSETA

PFKEAERASGELSALGVDNQMLVVNGILM--E-HNDDLSSSLYEKQQSALAAMPESLRALPIHSVPLRAY

NVTGLENIRALLNDYF--TAPVQLNA--TH-IPALNDVIDELAMEGKRVIFTMGKGGVGKTTVAAAVALG

LAKRGKKVHLTTTDPAAHLKFVL---TGGVSMSHIDEAEELKKYQSEVLFKARAGMGDEDIAYVEEDLRS

PCTQEIAVFRAFAEIVEKADDQVVVIDTAPTGHTLLLLESTQSYNHEIQRTGEESVAQLLP--RLKS-EE

TEVLIVTLPEATPVYEAHRLEDDLKRAGIAAKWWVVNQSLYGTNTTNPMLVSKAAGEVEWLNLIDEHAGG

KFALIAWSAEEIKGDSLLAL---

>F6BJ59|F6BJ59_T

FNLQNNLTKYMFFTGKGGVGKTSVACATAVALADSGKNVFLISTDPASNLQDVFNTKLDNKGVSIQEVPN

LVVANLDPVQAATEYRESVISPYRGILPDDVLKNMEEQLSGSCTIEIAAFNEFSKFITDKEIQTRYDYII

FDTAPTGHTIRMLQLPSAWSNFISENTHGTSCLGQLSGLESQKEVYKKAVKTLTDGKLTMLILVSRPETV

PLAEAERASNELGNIGVENQILIINGVMT--S-YDDKISESLFLKQQKILSKMPEGLKSIVTYIVPLRGY

NIIGIDNVRAMLNDNL--TLSKEIEV--ES-IPGLKDVVEDLYTNNKKAIFTMGKGGVGKTTISAAIALG

LSQKGRKVHLTTTDPAAHLKYVI---TDGISISYIDEQAELKKYQEEVLSKARETMSEEDLAYVEEDLRS

PCTQEIAVFRAFAEIVDKAENEIVVIDTAPTGHTLLLLESAQSYNHEIERSGEESAKKLLP--RLHNADE

TEVIIVTLPEATPVYEAMRLEEDLRRAGINNKWWIINSSLYKTGTSNKILLAKASNEIEWINKVAAYTNG

HFAVIAWSPDEIKGEKLKALLA-

>C1I718|C1I718_9

FDVQDNITKYLFFTGKGGVGKTSTACATAVSLADEGKKIMLISTDPASNLQDVFDTELNNKGVQIKDVPN

LVVANFDPEQAATEYKESVIAPYRGKLPETVLNNMEEQLSGSCTVEIAAFNEFSSFITDEKAANEFDHII

FDTAPTGHTLRMLQLPSAWSNFISQSTHGASCLGQLSGLESKKEIYKNAVSNLADGTKTTLILVSRPEKS

PLKEAERASNELKEIGVLNQILIVNGILE--N-HDDNLSAALYNKQQNALKDIPLGLKSIKTYKIPLRPY

NITGLKNMRALLKNNI--EIITDINT--SN-IHKLNSIIEDLFKSDKKVIFTMGKGGVGKTTIAAAIALG

LDKKGKKVHLTTTDPAAHLKFVV---SYGITLSSIDEKKELEKYKEEVLSKARETMSDADIEYVEEDLRS

PCTQEIAVFRAFAEIVDKSENEVVVIDTAPTGHTLLLLDSTQSYHREIQRSGDESVKKLLP--KLRNEKE

TEVIIVTLAETTPVFEAMRLQKDLNRAGINSKWWIINSSLYAINTSNEILKAKSSNEIKWINKVDEISSG

NFSVIEWKAEEVKGEKLLEIIK-

>C6PZ27|C6PZ27_9

FSLENELTKYIFFTGKGGVGKTSAACATAVALADEGKKIMLISTDPASNLQDVFNTELNNKGIAIKEVPN

LVVANFDPEQAAREYRESVISPYKGKLPDAVIKNMEEQLSGSCTVEIAAFNEFSNFITDKKVEKGFEHII

FDTAPTGHTLRMLQLPSAWSNFISENTHGASCLGQLAGLESKKEVYKNAVKTLADGEKTTLILVSRPQRT

PLKEAERASKELQDIGVNNQILLINGVLK--I-HDDRLSSSIYKKQEEALKNIPKHLRKLNTYKIPLRPY

NVTGLENVRAFLKDNI--KYSDELNN--EK-MLKLKTVIDDLYSTNKKVIFTMGKGGVGKTTIAAAIALG

LAERDKKVHLTTTDPASHLKFVL---SYGITLSHIDEKKELEKYKEEVLSKARETMGEEDLAYVEEDLRS

PCTQEIAVFRAFAEIVDRSENEIVVIDTAPTGHTLLLLDSTQSYNKEIERSGDESVKKLLP--KLRNADE

TEVIIVTLAEATPVYEAMRLEADLERAGINSKWWVINSSLFATDTTNDILRAKASNEIAWINKVSEISDG

NFAVIEWKEKEIKGDRLLDLIK-

>D8GTI1|D8GTI1_C

FNLNDHLTKYIFFTGKGGVGKTSAASAVAVSLADQGKKIMLVSTDPASNLQDVFNTDLNNKGTVIKKVPN

LVVANFNPEDAAKEYRESVISPYRGKLPDTVIKNMEEQLSGSCTVEIAAFNEFSNFITDEKIQKEFDHII

FDTAPTGHTLRMLQLPSAWSNFISENTHGASCLGQLAGLESKKEVYKNAVKTLANGKKTTLILVSRPQNT

PLKEAERASKELRDIGINNQTLIINGVLK--N-HDDGLSNAIYEKQQSALKYISEYLKKLETYAIPLRPY

NVTGLENVRAFLKDNI--EYSNELND--KK-ISKLKDVIDDLYNRDKKVIFAMGKGGVGKTTVAAAIALG

IARKGKKVHLTTTDPAAHLKFVL---SFGINLSHVDEKKELEKYKEEVLSKARETMGEEDIAYVEEDLRS

PCTQEIAVFRAFAEIVEKSEEEIVVIDTAPTGHTLLLLDSTQNYNREIKRSGDKSVKKLLP--KLRNADE

TEVIIVTLAEATPVYEAVRLEKDLKRAGISSKWWIINSSLFATNTTNDILKVKANSEISWINKVSEISKG

NFAVIEWKPEEVKGDKLLDLIK-

>B2UWF7|B2UWF7_C

FDLEKNLTKYLFFTGKGGVGKTSTACAVAVTLADLGKKIMLVSTDPASNLQDVFDTELNNKGVKINGIPN

LMVANFSPEDAAAEYRESVISPYRGKLPEAVLNNMEEQLSGSCTVEIAAFNEFSAMITDKNVSNEYDHII

FDTAPTGHTLRMLQLPSAWSNFINESTHGASCLGQLAGLEDKKDMYKKAVYTLADKEKTTLILVSRAEIT

PLKEAERASKELEDIGVKNQILIINGVLQ--Q-CDDYLSKSIYDKQQNSLKDIPEGLKNIKTFEIPLRPY

NITGLENVRSLLKNYT--VVDNELNI--TE-IPKLNSIIEDLYKNDKKVIFTMGKGGVGKTTIAAAIALG

LSKKGKKVHLTTTDPAGHLKFVL---SYGISLSNIDEKEELEKYKEEVLKKARENMSDEDIAYVEEDLRS

PCTQEIAVFRAFAEIVERSENEVVVIDTAPTGHTLLLLESTESYNREIVRSGDESVIKLLP--KLKNHKD

TEVIIVTLAETTPIYEAMRLQEDLNRAEIYSNWWVINSSLYATNTSNEILKAKASNEIRWINKVDKISNG

NFAVIKWKPEEIKDDRLKELLD-

>C5USP7|C5USP7_C

FDLEKNLTKYLFFTGKGGVGKTSTACAVAVTLADQGKKVMLVSTDPASNLQDVFDTKLNNKGILIKGVPN

LVVANFSPEEAANEYKESVIAPYRGKLPEVILNNMEEQLSGSCTVEIAAFNEFSSMITNKNVSAQYDYII

FDTAPTGHTLRMLQLPSAWSNFISESTHGASCLGQLAGLEDKKEMYKKAVETLTDKEKTTLILVSRPERT

PLKEAERAAKELQDIGVKNQLLIINGVLQ--E-CDDYLSKAIYNKQQNALKNISGILKNMECFEIPLRSY

NITGLKNVRALLKNYT--KFDNELKV--ME-IPSLNKIIEDLYVNNKKVIFTMGKGGVGKTTIAAAIALG

LSKKGKKVHLTTTDPAGHLNFIL---SQGITLSNIDEKQELDKYKEEVLGKARETMSEEDIAYVEEDLRS

PCTQEIAVFRAFAEIVERSENEVVVIDTAPTGHTLLLLDSTESYNREIQRSGDNSVKKLLP--KLKNHKE

TEVVIVTLAEATPVYEARRLQEDLNRAKIYSNWWAINSSLYATNTTNSILKAKASNEVKWINEVNNISNS

NFCAIEWRADEIKGDKLIELLK-

>A8MGW2|A8MGW2_A

FDPAENLTKYLFFTGKGGVGKTSTACAIAVALADKGKKIMLISTDPASNLQDVFNTELNNKGVPIKEVPN

LVVANFEPEKAAAEYKESVIAPYRGKLPEAVLTNMEEQLSGSCTVEIAAFNEFSGFITDEKASKEYDHII

FDTAPTGHTLRMLQLPSAWTNFISENTHGASCLGQLSGLEDKKEIYKHAVENLADGGKTTLILVSRPEES

ALKEAERASIELQDIGVNNQLLVVNGVLK--V-HDDELSTSIYIKQKSALDNMPEGIKSIQAFEIPLRPY

NVTGIENVRSFFNDNI--KHSTELNV--DK-IPNLNDVIEDLYKSDKKVIFTMGKGGVGKTTIAATVALE

LAKKGKKVHLTTTDPAAHLKFVL---GYGITISNIDEKKELEKYKEEVLSKARETMSGDDLAYIEEDLRS

PCTQEIAVFRAFAEIVERSENEVVVIDTAPTGHTLLLLDSTQSYHKEIQRSGDESVKNLLP--KLRDEKH

TEVLIITLPEATPVYEAIRLKEDLERADIFVKWWVINSSFYATNTTNDILKVKAGNEVGWINKVNEVSKG

NFAVIKWTHEEVKGENLSKLLK-

>A6LV97|A6LV97_C

FDIEKNLTKYLFLTGKGGVGKTSTACAVAVNLADQGKKIMLVSTDPASNLQDVFNTELNNKGVQIKEVPN

LTVANFEPEAAAAEYRESVIAPYRGKLPQAVIDNMEEQLSGSCTVEIAAFNEFSAMITDEKVFNEYDHII

FDTAPTGHTLRMLQLPSAWSDFINESTHGASCLGQLAGLEEKKEMYKSAVDTLADGEKTTLILVSRPETS

PLKEAERASGELQEIGVDNQILIINGVLQ--S-HDDELSNAIYEKQQKALSNMPPKFKDIETFEIPLRPY

NITGLENVRAFLKDYI--KISEELNA--VA-MPKLKDVIEDLYNSSKKVIFTMGKGGVGKTTIAASIALG

LAKKGKKVHLTTTDPAAHLKFVL---SYGISLSNIDEKEELEKYRQEVIGKARENMTDEDIEYIEEDLRS

PCTQEIAVFRAFAEIVERSENEVVVIDTAPTGHTLLLLDSTESYNKEISRSGDESVIKLLP--RLRNESE

TEVVIVTLAETTPVYEAMRLQKDLDRAQIHSKWWIINSSLYATDTTNEILKVKASNEIQWINKVDEISSG

NFAVIEWKAEDVRGNNLNNLIK-

>C1FUI9|C1FUI9_C

FNVEDNLTKYLFFTGKGGVGKTSTACAIAVALADSGKKIMLVSTDPASNLQDVFNTKLNNKGVYIKEVPN

LVVANFEPEEAAAEYRESVIAPYRGKLPEVVLKNMEEQLSGSCTVEIAAFNEFSTFITDEKVEKEYDHII

FDTAPTGHTLRMLQLPSAWSNFINESTHGASCLGQLSGLESKREVYKNAVNTLADKDKTTLILVSRPEVS

PLKEAERASKELQDIGVNNQVLVINGVLE--E-HEDYLSNAIYTKQQKALEDIPESLKTVETFQIPLRPY

NVTGLENVRAFLRNNI--KYNDELNT--TN-IPKLNEVIEDLYNTDKKVIFTMGKGGVGKTTIAAAIAVG

LAKKGKKVHLTTTDPADHLKFVL---DYGITLSHIDEKEELEKYKEEVLSKARKTMSEDDIAYVEEDLRS

PCTQEIAVFRAFAEIVERSENEVVVIDTAPTGHTLLLLDSTQSYNKEIQRSGDESVKKLLP--KLRNEEN

TEVIIVTLAETTPVYEAMRLQEDLNRAGIHSKWWVINSSFYVADTTNSILKVKANNEVQWINKVNEISKG

NFAVIEWIPKEVKGEILNDLIN-

>A5HZU8|A5HZU8_C

FNVEDNLTKYLFFTGKGGVGKTSTACAVAVTLADKGKKIMLVSTDPASNLQDVFNTKLNNKGVTIKEVPN

LVVANFEPEEAAAEYRESVIAPYRGKLPEAVLKNMEEQLSGSCTVEIAAFNEFSTFITDEKVEKEYDHII

FDTAPTGHTLRMLQLPSAWSNFINESTHGASCLGQLSGLESKKEVYKNAVNTLADKDKTTLILVSRPEVS

PLKEAERASKELQDIGVNNQVLVINGVLE--E-HEDYLSNAIYTKQQKALEDIPESLKIVETFQIPLRPY

NVTGLENLRAFLKNNI--KYNDELNT--TN-IPKLNKVIEDLYNTDKKVIFTMGKGGVGKTTIAAAIAVG

LAKKGKKVHLTTTDPADHLKFVL---DYGITLSHIDEKKELEKYKEEVLIKARKTMSEDDIAYVEEDLRS

PCTQEIAVFRAFAEIVERSENEVVVIDTAPTGHTLLLLDCTQSYNKEIQRSGDKSVKKLLP--KLRNEEH

TEVIIVTLAETTPVYEAMRLQEDLNRAGIHSKWWVINSSFYVADTTNSILKVKANNEIQWINKVNEISKG

NFAVIEWMPEEVKGEMLNRLIK-

>A7GBE7|A7GBE7_C

FNVEDNLTKYLFFTGKGGVGKTSTACAVAVTLADKGKKIMLVSTDPASNLQDVFNTELNNKGVTIKEVPN

LVVANFEPEEAVEEYRESVIAPYRGKLPEVVLRNMEEQLSGSCTVEIAAFNEFSTFITDEKAEKEYNHII

FDTAPTGHTLRMLQLPSAWSNFINESTHGASCLGQLSGLESKKEVYKNAVNTLADKDKTTLILVSRPEVS

PLKEAERASKELQDIGVNNQVLVINGVLE--E-HEDYLSNAIYTKQQKALEDIPESLKTVETFQIPLRPY

NVTGLENVRAFLRNNI--EYNDELNT--TN-MPKLNKVIEDLYNTDKKLIFTMGKGGVGKTTIAAAIAVG

LAKKGKKVHLTTTDPADHLKFVL---DYGITLSHIDEKEELEKYKEEVLSKARKTMSEDDIAYVEEDLRS

PCTQEIAVFRAFAEIVERSENEVVVIDTAPTGHTLLLLDSTQSYNKEIQRSGDESVKKLLP--KLRNEEH

TEVIIVTLAETTPVYEAMRLQEDLNRAGIHSKWWVINSSFYVADTTNSILKVKANNEIQWINKVNEISKG

NFAVIEWMPEEVKGEMLNRLIK-

>B1Q6M2|B1Q6M2_C

FNVKDNLTKYLFFTGKGGVGKTSTACAIAVALADEGKKIMLVSTDPASNLQDVFNTELNNKGVHIKEVPN

LVVANFEPEEAAAEYKESVISPYRGKLPEVVLKNMEEQLSGSCTVEIAAFNEFSTFITDEKVEKEYDHII

FDTAPTGHTLRMLQLPSAWSNFINESTHGASCLGQLSGLESKKEVYKNAVNTLADKDKTTLILVSRPEVS

PLKEAERASKELQDIGVNNQVLVINGVLE--E-HEDYLSNAIYTKQQKALEDIPQSLKTVETFQIPLRPY

NVTGLENVRAFLKNNI--KYNDELNT--NN-IPKLNNVIEDLYNTDKKVIFTMGKGGVGKTTIAAAIAVG

LAKKGKKVHLTTTDPAAHLKFVL---DYGITLSHIDEKEELEKYKEEVLSKARKTMSEDDIAYVEEDLRS

PCTQEIAVFRAFAEIVERSENEVVVIDTAPTGHTLLLLDSTQSYHKEIQRSGDESVKKLLP--KLRNEDH

TEVIIVTLAETTPVYEAIRLQEDLNRAGIHSKWWVINSSFYTANTTNSILKVKGNNEVQWINKVNEISKG

NFAAIGWIPEEVKGEILNHLIN-

>E8ZM91|E8ZM91_C

FNVKDNLTKYLFFTGKGGVGKTSTACAVAVTLADKGKKIMLISTDPASNLQDVFNTELNNKGVHIKEVPN

LVVANFEPEEAAAEYRESVISPYRGKLPEVVLKNMEEQLSGSCTVEIAAFNEFSTFITDEKVEKEYDHII

FDTAPTGHTLRMLQLPSAWSNFINESTHGASCLGQLSGLESKKEVYKNAVNTLADKDKTTLILVSRPEVS

PLKEAERASKELQDIGVNNQVLVINGVLE--E-HEDYLSNAIYTKQQKALEDIPQSLKTVETFQIPLRPY

NVTGLENVRAFLKNNI--KYNDELNT--TN-VPKLNKVIEDLYNTDKKVIFTMGKGGVGKTTIAAAIAVG

LAKKGKKVHLTTTDPADHLKFVL---SYGITLSHIDEKKELEKYKEEVLSKARKTMSEDDIAYVEEDLRS

PCTQEIAVFRAFAEIVERSENEVVVIDTAPTGHTLLLLDSTQSYNKEIQRSGDESVKKLLP--KLRNEEN

TEVIISTLAETTPVYEALRLQKDLNRAGIHSKWWVINSSFYATNTTNSILKVKANNEVHWINKVNEISKG

NFAVIGWMPEEVKGEILNHLIN-

>Q24NK5|Q24NK5_D

FTPEKKLTKYLFYTGKGGVGKTSTACATAVNLADNGKKVLLISTDPASNLQDVFKTDLNNKGVAIQEVPN

LVVANLDPVQAAAEYRESMIAPYRDKLPEVVIKNMEEQLSGSCTVEIAAFNEFSQFITDETMQEEYDHII

FDTAPTGHTLRMLQLPSAWSQFISESTHGASCLGQLSGLESKKEMYKKAVETLAEGDLTTLILVSRPEET

PLKEAVRASKELADLGVNNQVLILNGVLA--S-YDDAISESLYLKQQKALEEMPKGLQNLLTYTVPLRAY

NVTGIENVRALLTDNL--IIHKEIQE--ET-IPQLKDVIDDLHNTHKKVIFTMGKGGVGKTTIAAAVALG

LSERGKKVHLTTTDPAAHLRFVI---NSGITLSHIDEQAELKKYQEEVLSKARETMSEEDIAYIEEDLRS

PCTQEIAVFRAFAQIVEKAEDQVVVIDTAPTGHTLLLLDSTQSYHQEIKRSGDESVKKLLP--RLRNSEE

TEVIIVTLAEATPVYEAMRLEGDLKRAGIATKWWVINSSLYRTGTTNQLLAAKASHEIEWINKVDEHSKG

NFAVIPWSAEDIKGDKLLEL---

>Q24PV1|Q24PV1_D

FHPDRQLTQYLFFTGKGGVGKTSTACATAVNLADNGKKVLLVSTDPASNLQDVFNRELSNKGVPMPEVPN

LVVANLDPIQAAAEYRESVIAPYRGKLPEAVLKNMEEQLSGSCTVEIAAFNEFSHFITDKEMESQYDYII

FDTAPTGHTLRMLQLPSAWSNFISENTHGASCLGQLSGLESKKEIYAQAVRTLADGRETTLVLVSRPEDT

PLKEAARASQELAELGVSKQLLVINGVIS--S-YDDSISEGLYEKQQKALQDMPRELQSLTAYTIPLRAY

NITGIGNVRALLTDDY--AFSDELSV--QH-LPQLKDVINDLDTSNRKVIFTMGKGGVGKTTIAAAIAMG

IAARGKKVHLTTTDPAAHLKFVI---TDSITMSHIDEEAELKKYQEEVLSKARETMSEEDLAYIEEDLRS

PCTQEIAVFRAFAQIVEKAEDQVVVIDTAPTGHTLLLLDSTQSYHKEIQRSGDESVKKLLP--RLRSAEE

TEVIIVTLAEATPVYEALRLEADLKRADIATNWWVINSSLYTTNTTNQLLAAKASKEIEWINKVAEHSHG

KLAVIPWRAEDIKSENLLEL---

>B8G132|B8G132_D

FHPDKQLTQYLFFTGKGGVGKTSTACATAVNLADNGKKVLLVSTDPASNLQDVFNRELSNKGVPIPEVPN

LMVANLDPIQAAADYRESVIAPYRGKLPEAVLKNMEEQLSGSCTVEIAAFNEFSHFITDQEMESQYDYII

FDTAPTGHTLRMLQLPSAWSNFISENTHGASCLGQLSGLESQKEIYAQAVRTLADGYKTTLVLVSRPEDT

PLKEAARASQELAELGVSNQLLVINGVIS--S-YDDSISAGLYGKQQKALQDMPGQLQGLTAYTIPLRAY

NITGIDNVRALLTDNY--ALSDELSV--QH-LPQLKDVINDLDVSNRKVIFTMGKGGVGKTTIAAAIAMG

ISARGKKVHLTTTDPAAHLKFVI---TDGITMSHIDEDAELKKYQEEVLSKARETMSEEDLAYIEEDLRS

PCTQEIAVFRAFAQIVEKAEDQVVVIDTAPTGHTLLLLDSTQSYHKEIQRSGDESVKKLLP--RLRSAEE

TEVIIVTLAEATPVYEALRLEADLKRADIATKWWVINSSLYMTDTTNQLLAAKASNEIEWINKVAEHSHG

KLAVIPWRAEDIKSENLLEL---

>D9R5Q8|D9R5Q8_C

FDLNKQLTKYLFFTGKGGVGKTSTACASAVTLADLGKKVLLVSTDPASNLQDVFRMELTGKGTPIRDVPG

LMVANLDPLKAAADYRESVMAPYRGKLPESVLKNMEEQLSGSCTVEIAAFNEFSNFITDEKAKEEYDHII

FDTAPTGHTLRMLQLPSAWSNFISESTHGASCLGQLSGLESRKEIYKKAVSTLASGDMTTLLLVSRPETA

PLKEAVRAAKELSDIGVSNQIMIVNGVLN--S-YDDPISESLYQKQQKALVQMPEELKRLKQYYVPLRAY

NITGLENVRNLLVDNF--NLVHALKA--QD-IPTLNHVVDDLYESGKKVIFTMGKGGVGKTTIAAAIAMG

LSAKGVNVHLTTTDPAAHLKFVM---PSGITMSHINEKEELLRYQQEVLAKARETLSEDDVAYVEEDLRS

PCTQEIAVFRAFAEIVENAEDQVVVIDTAPTGHTLLLLDSTQSYHKEVKRTGDESVKKLLP--RLRNAEE

TEVIIVTLAETTPVFEAMRLEEDLERAEITVKWWVINSSLYATETTNRLLKAKASHEIQWINKVDDISKG

NFAVIQWSADEIKGEKLLSI---

>D9RAP4|D9RAP4_C

FDLQHNLTKYLFYTGKGGVGKTSIACATAVTLADSGKKVLLVSTDPASNLQDVFNTDLDGKGVSIAEVPG

LVVANLDPIQAAVEYRESVVAPYRGKLPDAVILNMEEQLSGSCTVEIAAFNEFTNFITDQAAAKEYDHII

FDTAPTGHTLRMLQLPSAWSNFISESTHGASCLGQLSGLEERKEMYKEAVDTLADSKLTTLVLVSRPESS

PLKEAERASKELADLGVHNQLLAVNGVLP--A-YDDNISESLYNKQQKALAGMPAGLKDIAAYFVPLRAY

NITGIENVRNLLVDSL--FVSGNLNA--AD-IPTLRKVIDDLDTSGKKVIFTMGKGGVGKTTIAASIALG

LSSRGKKVHLTTTDPAAHLKFVM---TSGITMSHIDEKEELRRYQEEVLSKARETMSADDVAYVEEDLRS

PCTQEIAVFRAFAEIVERAENEIVVIDTAPTGHTLLLLDSTQSYHREVKRTGDEAVKKLLP--RLRNTDE

TEVIIVTLAETTPVFEAMRLEEDLKRAEIATKWWVINSSLYATETSNQLLKAKASHEIEWINKVGEISHD

NFAVIRWNAEEIKDDKLLAL---

>D6XYX5|D6XYX5_B

-TPQDTDTQYLFFTGKGGVGKTSAACATAVSLADQGKKVLIVSTDPASNLQDVFGTTLANEPAPVPGVDN

LFAANLDPEEAAAAYRNKMIDPYRETLPQAALDSMEEQLSGACTVEIAAFDEFSSLLANEEATADFDHIL

FDTAPTGHTLRLLQLPNAWSDFLEGNENGASCLGPLAGLADKKALYQKTVEALANGERTKLILVARPDES

TLVEAGKAAKELGEIGILNQLLVINGVFER-T-SADPTAIQLEKKQQEALQTIPAYFDDKPVFTLPLVPY

NLTGFQALRDLFDDAT--QLSESPTAEPID-LPSVSDMIDELAARNKGVFMTMGKGGVGKTTVAAAVAAG

LADRGHKVLLTTTDPAAHVDLVIDQMEGTLAVSRIDPKQEVENYKAQVLNNVSSELTEDELAYIKEDLES

PCTEEIAVFRAFAETVDQAKDAFVVIDTAPTGHTLLLLDAAQSYHKEVERTGDESVKQLLP--RLRNPEE

TFISLVTLPEATPAYEAGRLQDDLRRAQIEPAWWIINQSYHETGTTDPILAGRAFAERKWIKEVKESYSQ

KACIIPWQADQVKGLEKLKALTK

>D3FW68|D3FW68_B

FNPVKELTPYLFFTGKGGVGKTSTACATAVALADQGKKVLLVSTDPASNLQDVFEQEIGYHETKIDELPN

VTAINLDPEEAAAAYRNKMIGPFRDKLPAPVIDQMEEQLSGACTVEIAAFDEFATILTNPERTNEYDHIL

FDTAPTGHTLRLLQLPTAWSGFLDTSTHGASCLGPLSGLAEKKALYEHTVKALSDAAKTTLILVARPEPS

TLKEAARASEELKEIGLVNQWLLINGKMQSYV-NDDLTSTAFYKRQEQALQSMPAKLQETKQYELPYVPY

QLTGIESLRSLF-ELP--KCNARVPRADVQ-ADSLSVLINDLAKKEQGVILTMGKGGVGKTTVAAAVAAG

LANKGLKVKLTTTDPAAHVSDLFKE-DQLITISRIDPKAEVEAYKQEVLSASSEHLDEDGLAYLEEDLNS

PCTEEIAVFRAFADVVEEADDAFVVIDTAPTGHTLLLLDAAHSYHKEMERSGEESVKKLLP--RLRNPKE

TDVLVVTLPEATPVFEASRLQDDLIRADITPSWWVINQSFAGTNTIDPILSERAQSEFKWINEVITTHAK

HTVVIPWQSETIEGRKQLTSL--

>A6CIH3|A6CIH3_9

FQPQTAGAKFIFFTGKGGVGKTSTACATALKLAEEGEKVLLVSTDPASNLQDVLEVDLTNTPMPVPGASN

LSACNLDPEEAARTYREKVIGPFRGKLPESVVSTMEEQLSGACTVEIAAFDEFTNLLSDRSVIESYDHIL

FDTAPTGHTLRLLQLPTAWSGFLEESTHGASCLGPLSGLAEKKQAYSDTMKALADSEQTTLYLVARPDES

SLQEASRASKELREIGIGNQHLIINGLMQTHV-EEDKISASLYHRQQTALQHMPEELKRVSSFSLPFVSY

SLTGLSNLRNLLNQII--SDEEASEKQTIK-LPGLNEMVEDFSSNGTRVIFTMGKGGVGKTTMASAIAVG

LVEKGHKVHLTTTDPAAHLEFMFGELNPNLSISRIDPKKEVEDYKTEVLSNVSEDLDEEALAYIEEDLNS

PCTEEIAVFRAFAEVVDKAKEEIVVIDTAPSGHTLLLLDAAQSYHKELARSGEASVKELLP--RLRNPEE

TSVVIVTLAEATPVLEASRLQDDLKRADINPKWWLINQSLYATETSDPVLKGRAIAETVWIQKVSNELSD

RCAIVPLMSDDRTGYDELRKYTE

>C2TMP5|C2TMP5_B

YNPQTEFTPFLFFTGKGGVGKTSTACATAVTLADKGQRVLLVSTDPASNLQDVFNMELTNHPVEIPSVNN

LFVANLDPETAASEYKERVVGPFRGKLPEAVINQMEEQLSGACTVEIAAFDEFSSLLTNKELTKQYDYII

FDTAPTGHTLRLLQLPTAWSGFLEESTHGASCLGPLAGLGAKKKLYEETVTALSSGKQTTLVLVTRPDVS

PLQEAARASKELGDIGVQNQMLLINGMMQNHV-KEDEVSKAFYERQTKALEQMPNELKQIPTYAVPLAPF

NITGIENIRKLF-ENA--TSNVDLEAKDIK-TTPLKELIENIQQTGQRVIFTMGKGGVGKTTVASTIAVG

LVEKGHKVHLTTTDPAAHLEHVMESLHGSLSVSRIDPKVEVENYREEILNQSSELLDEEGLAYLEEDLRS

PCTEEIAIFRAFANIVEKASNEIVVIDTAPTGHTLLLLDAAHSYHKEIERSGEVSVQQLLP--RLRNPQE

TGVVIVTLAEATPVFEASRLQEDLKRASITPTWWVINQSLYATNTKDPILHGRAMSEIEWMREVEKKSNG

QYVVIPWKAQDIVGYENLKHLTV

>C2WG14|C2WG14_B

YNPNTAFTPFLFFTGKGGVGKTSTACATAITLADMGKQVLLISTDPASNLQDVFEIELTNKPKEIPSVPN

LQVANLDPETAAHEYKERVVGPYRGKLPDTVIATMEEQLSGACTVEIAAFDEFSTLLTNKELTSKFDHII

FDTAPTGHTLRLLQLPTAWSGFLEESTHGASCLGPLAGLGDKKELYSQTVQALSNPKQTILLLVTRPDNS

PLQEAERAAKELKEIGVTNQYLLVNGMLRDFM-QNDAVSKALFTRQLHALENMAEELKSLPTYEIPLVPF

NVTGIENMRKLV-PME--NILISEEKQGIS-MPSLQALITNLSETGKKVIFTMGKGGVGKTTVASAIAVG

LAEKGHHVHLTTTDPAAHIDYVME--QGNITISRIDPKVEVENYRKEVIEQAKETVDEEGLAYLEEDLRS

PCTEEIAVFRALADIVEKANDEIVVIDTAPTGHTLLLLDAAQTYHKEIARSGERSVKNLLP--RLRNPEE

TSVVIVTLAEATPVHEASRLQGDLKRADITPKWWVINQSFYATHTSDPVLRGRAQSEVQWIQAVQKESQN

NCVIIPWQSEDIIGYEKLKAL-V

>C2TVW7|C2TVW7_B

YNPNTAFTPFLFFTGKGGVGKTSTACATAITLADMGKQVLLISTDPASNLQDVFEIELTNKPKEIPSVPN

LQVANLDPETAANEYKERVVGPYRGKLPDAVIATMEEQLSGACTVEMAAFDEFSTLLTNKELTSKFDHII

FDTAPTGHTLRLLQLPTAWSGFLEESTHGASCLGPLAGLGDKKELYSQTVQALSNPNQTMLLLVTRPDSS

PLQEAERAAHELKEIGVSNQFLLVNGILKDYV-QNDDVSNALFKRQSSALENMAEELKNLPTYEIPLVPF

NVTGIENMRKLV-PME--NLSIAEEANAVS-IPSLQTLITNFSESEKRVIFTMGKGGVGKTTVASAIAVG

LAEKGHHVHLTTTDPAAHIDYVME--QGNITISRIDPKVEVENYRKEVIEQAKDTVDEEGLAYLEEDLRS

PCTEEIAVFRALADIVERANDEIVVIDTAPTGHTLLLLDAAQTYHKEIARSGEQSVKNLLP--RLRNPEE

TSVVIVTLAEATPVHEASRLQGDLKRADINPKWWVINQSFYATHTSDSVLRGRAQSEIQWIQAVQKESQN

NCVIIPWQSDDIVGYEKLKAL-V

>Q5Q1Q5|Q5Q1Q5_9

FNPSTTFTPFLFFTGKGGVGKTSTACATAITLADMGKQVLLISTDPASNLQDVFEIELTNKPKEIPSVPN

LQVANLDPETAAHEYKERVVGPYRGKLPDAVIATMEEQLSGACTVEMAAFDEFSTLLTNKELTSKFDHII

FDTAPTGHTLRLLQLPTAWSGFLEESTHGASCLGPLAGLGDKKELYSQTVQALSNPSQTMLLLVTRPDSS

PLQEAERAAHELKEIGVSNQFLLVNGILKDYM-QNDNVSNALFKRQSRALENMAEELKNLPTYEIPLVPF

NVTGIENMRKLV-PME--NLSISEEANTVS-IPSLQTLITNLSESGKRVIFTMGKGGVGKTTVASAIAVG

LAEKGHHVHLTTTDPAAHIDYVME--QGNITISRIDPKVEVENYRKEVIEQAKDTVDEEGLAYLEEDLRS

PCTEEIAVFRALADIVERANDEIVVIDTAPPGHTLLLLDAAQTYHKEIARSGEQSVKNLLP--RLRNPEE

TSVVIVTLAEATPVHEASRLQEDLKRADITPKWWVINQSFYATHTTDLVLRGRAQSEIQWIQAVQKESQN

SCVIIPWQSEDIVGYEKLKDL-V

>C2MJP9|C2MJP9_B

YNPNTAFTPFLFFTGKGGVGKTSTACATAITLADMGKQVLLISTDPASNLQDVFEIELTNKPKEIPSVPN

LQVANLDPETAAHEYKERVVSPYRGKLPDAVIATMEEQLSGACTVEMAAFDEFSTLLTNKELTAKFDHII

FDTAPTGHTLRLLQLPTAWSGFLEESTHGASCLGPLAGLGDKKELYSQTVQALSNPNQTMLLLVTRPDSS

PLQEAERAAHELKEIGVSNQFLLVNGILKDYM-QSDNVSNALFKRQSRALENMAKELKDLPTYEIPLVPF

NVTGIENMRKLV-PME--NLSISEETNTVS-IPSLQTLITNLSESGKRVIFTMGKGGVGKTTVASAIAVG

LAEKGHHVHLTTTDPAAHIDYVME--QGNITISRIDPKVEVENYRKEVIEQAKDTVDEEGLAYLEEDLRS

PCTEEIAVFRALADIVERANDEIVVIDTAPTGHTLLLLDAAQTYHKEIARSGEQSVKNLLP--RLRNPEE

TSVVIVTLAEATPVHEASRLQEDLKRADINPKWWVINQSFYATHTSDFVLRGRAQSEIQWIQEVQKESQN

NCVIIPWQSEDIVGYEKLKEL-V

>Q9RA89|Q9RA89_9

YNPNTAFTPFLFFTGKGGVGKTSTACATAITLADMGKQVLLISTDPASNLQDVFEIELTNKPKEIPSVPN

LQVANLDPETAAYEYKERVVGPYRGKLPDAVIATMEEQLSGACTVEMAAFDEFSTLLTNKELTSKFDHII

FDTAPTGHTLRLLQLPTAWSGFLEESTHGASCLGPLAGLGDKKELYSQTVQALSNPNQTMLLLVTRPDSS

PLQEAGRAAKELKEIGVNNQYLLINGVLTNYV-QNDAISKALFTRQVRALENMSEELKGLPAYELPLVPF

NVTGIENMRKLV-PIE--SLSILEIQEEIA-IPPLQNLIADLSETGKRVIFTMGKGGVGKTTVASAIAVG

LAEKGHRVHLTTTDPAAHIDYVME--QGNITISRIDPKVEVENYRKEVIEQAKDTVDEEGLAYLEEDLRS

PCTEEIAVFRALADIVEIANDEIVVIDTAPTGHTLLLLDAAQTYHKEIARSGEQSVKNLLP--RLRNPEE

TSVVIVTLAEATPVHEASRLQGDLKRAEIHPKWWVINQSFYATHTIDPVLKGRSQSEVPWIQEVQKESQH

NCVIIPWQSEDVIGYEKLKELTV

>C2QKX2|C2QKX2_B

FNPSTTFTPFLFFTGKGGVGKTSTACATAITLADMGKRVLLISTDPASNLQDVFEIELTNKPKVIPNVPN

LHVANLDPETAAHEYKERVVGPYRGKLPDTVIATMEEQLSGACTVEIAAFDEFSTLLTNKELTSKFDHII

FDTAPTGHTLRLLQLPTAWSGFLEESTHGASCLGPLAGLGDKKELYSQTVEALSNPKQTSLMLVTRPDSS

PLQEAERAAKELKEIGVSNQYLLVNGVLTNYV-QNDSVSKALFTRQVRALENMTEELKDLPAYEIPLVPF

NVTGIENMRKLV-PIE--NLSISEEQHNVS-IPPLQNLITDLSKTGKRVIFTMGKGGVGKTTVASAIAVG

LAEKGHHVHLTTTDPAAHIDYVME--QGNITISRIDPKLEVENYRKEVIEQAKDTVDEEGLAYLEEDLRS

PCTEEIAVFRALADIVEKANDEIVVIDTAPTGHTLLLLDAAQTYHKEIARSGEQSVKNLLP--RLRNPEE

TSVVIVTLAEATPVHEASRLQEDLKRAEIYPKWWVINQSFYATHTIDPVLKGRSQSEVQWIQEVQKESKH

NSVIIPWQSEDVIGYEKLKELTV

>C4LKI7|C4LKI7_C

MSLPELSTKFAFFTGKGGVGKTTVACSLATRAASEGKRVLLVSTDPASNIGQVFGREIGELTDLVPGATS

FDAVEIDPEAEAARYRESILGPVRGLLPPEVLATTEETLSGSCTVEVASFNRFVDYLTNEDITSRYDHII

FDTAPTGHTLRLLSLPGDWSSFIDKGAGDASCLGPMSGLEKNRQTYHEAVAALADPAQTSLVLVARAQIS

TLQEANRSTGELLEMGIKPTLLVINGILPANA-ATDQLSESIYAREQALLDGLASAINSIPTVRLELKAN

PVMGVDGLSHLGEDGNESNAGPAPGFTQSTSHVDLSNLVDELAEGKPKLVLCMGKGGVGKTTVAQMLALE

LAKRGKPVHLSTTDPANHLDGSLEDSISGLTVSSIDPDAVTASYREEVLASKGASLDADGYAQLEEDLRS

PCTEEVAVFNAFSEIVATAENQWVVLDTAPTGHTLLLLDATGSYHRELMRQ--GRDDSATTLQHLQNTEV

TRPILVTLPEATPMLEAQSLAADLSRAGIEPWAWVINQSLPATETSSPFLLRRANAQQEVINSVVNQAGV

EVVQLPVIPD-------------

>C4L6C4|C4L6C4_E

FDFMTRPTRYVFLTGKGGVGKTSTASSLALTLADRGKRVLLVSTDPASNLQDVFEMELDETPRLVPET-K

LAIANFDPEEAAKQYMERMVGPYRGVLPDVAIQSMEEQLSGACTIEIAAFDQFTDLLTSVSARETYDHVI

FDTAPTGHTLRLLTLPSAWDEFLETNTTGASCLGPLAGLADKQMQYKKAVETLTDGKETTLLLVARPETS

TLIEAERAATELRAIGIEQMQLIVNGLVQQDE-RADRYTKRLIERQQQALRELPEGITDLEAFKLPYAYE

NLIGVSALRRFVNETV-------AVANACYSYGSLDRLVDEIEADGPSVIMTMGKGGVGKTTLASLIAVA

LHERGHSVHLTTTDPAAHVAFTITNIADTFTVDAIDPKVEIERYRQDVFKEAG-DLNEEQRMMLEEDLRS

PCTEEIAVFRAFANQVAKAKDCFVVIDTAPTGHTLLLLDATESYHRELERSGVEAVRDLLP--KLRDPKQ

THVIITTLPEATPVYEAERLVADLNRAKILPFAWVVNQSFTPLDVTSKLLTAKVANEVPWLERVNSY--R

RPTMLAWQEHLPVGYSALSQLLK

>C5T841|C5T841_A

LALLDHPTKYLFFTGKGGVGKTSVSTAVSIALADSGKKVLLVSTDAASNLDEMLGITLSNHPVAVPGVPG

LQVLNIDPEAAAVDYRARVIAQMGPQASEQDIATVREQLSGACTTEIATFDEFSSLLSRA--GNTYDHVI

FDTAPTGHTLRLLSLPKAWSGFLDGNDRGASCLGPHSGLKMQEQLFNEALAALNDATLTTVVLVARPEKG

ALDEAARTCEELRALGLTHQRLVVNAVFKSSN-AQDPIGTAIEALGHKALDEMPAALRQLATDRIPLKAV

DSVGLPALRALLS-SPAPAGSVAPPSPDPA-HHRLAELVAELAQGERGLIMVMGKGGVGKTTIAAAIALG

LVRHGKSVHLSTTDPAAHLAVTLNADIPGLSVGRIDPKVETQKYVDKIVASRAATLTEDEKALLIEDLRS

PCTEEVAVFHAFSHVVAQARSAFVVLDTAPTGHSLLLMDATGAYHRQMLREEG-AARIVTPLMRLQDADY

TRIVLVTLPEATPVSQAAALQDDLRRAHIEPFAWVVNKSLLATGTADPLLQARLDSERRQMARVAAHTSH

TITVVPWTAEPPIGPDALARLG-

>A1W8U8|A1W8U8_A

FSLLDAPTKYLFFTGKGGVGKTSVSTAVSIALADAGKKVLLVSTDAASNLDEMLGIALRNHPVPVPGVPG

LMVLNIDPEAASQNYRARVIEQMGTQATAEEVDLVREQLSGACTTEIATFDEFSQLLSEG--GRDFDHVV

FDTAPTGHTLRLLSLPKAWSGFLKGNDRGASCLGPHSGLKMQEQLFNRALASLNDASLTTIVLVARPEAG

ALNEAARSSDELRELGLSHQRLVINAVFKAQD-PRDAVALAIEALGQEAMREMPQALKRLAADVIPMRPV

DSVGLQALRALLS-SPTEP-AAALEAPDFA-HQKLSALVAELARGERGLIMVMGKGGVGKTTIAAAIALG

LVKLGKSVHLSTTDPAAHLAVTLDGEIPGLTVGRIDPKAETQKYVDKVVAAKSAGMTPAEKDLLMEDLRS

PCTEEVAVFHAFSHVVSQARSAFVVLDTAPTGHSLLLMDATGAYHRQMVREEG-SGRITTPLMRLQDPDY

TKIILVTLPEATPVSQAAALKEDLQRARIGTFAWVVNKSLLASDTRDPLLQARLSSERRQMERVQALAQG

RFYLIPWAATPPVGAKALGDLLE

>C9YA76|C9YA76_9

LQLLQHPTRHLFFTGKGGVGKTSLSTACAIALADSGKQVLLVSTDAASNLDEMLGITLANTPVHVPGVPD

LSVLNIDPDNAAQSYRARVLAQMDPTSTEAERSQVQEQLSGACTTEIAAFDEFANLLSGD--AAGYDHVV

FDTAPTGHTLRLLSLPKAWTGFLADNDRGASCLGPHSGLKMQEARFAQALQTLSDAAQTTVVLVTRPDAG

AIAEAARTSLELQDLELHNQRLAINGVFHASA-PGDAVATAFEALGAQALAAMPAHLQALPQDQIALRAF

DTVGLSALRALLSKEPVAVQPAAAHAGIHP-THTLSALTDALATQGRGLIMVMGKGGVGKTTIAAAIAAG

LVQRGHSVHLSTTDPADHLQVTVDGKLPGLKVDRIDPRAETEKYIAKVMAAKSPGLDAEGIALLREDLQS

PCTEEVAVFHAFSRIVSEARSAFVVLDTAPTGHSLLLMDATGAYHRQMLRE-GNPGRLVTPLMRLQDPNH

TKIVLVTLPEITPVSQAAALQDDLRRAKIEPYAWVINKSMSAAGTRDPLLAARLAGEEKQMARIAHGLSQ

HSYVVPWMAERPVGLAALGTLTT

>A1VTP9|A1VTP9_P

PGFLLAPTRFVFFTGKGGVGKTSLSTATAIALADAGRRVLLVSTDAASNLDEMLGMPMSNQPAQVPGVPR

LRMLNIDPDAAAEAYRLRVLEQLGLDASDDERKTVREQLSGACTTEIAAFDEFAALLAGEGAGSGYDHVI

FDTAPTGHTLRLLSLPKAWSGFLAGNDRGASCLGPHSGLKMQEARFNAALAALSDAKLTTVVLVTRPDPR

PMQEAARTAEELRTLGLSNQRLVINGVFHASR-PDDPTARALEALGLQAIAQMPDALAGLPRDEVPLRAF

DTVGLSALRALLG-GAIPSAPVALAAEL-P-AEPLSRLADELAAMGHGLIMVMGKGGVGKTTIASALAVG

LVQRGHSVHLTTTDPAAHVAETLNGSLPNLKVGRIDPRAETEAYIAKIMATRGKALDEQGRALLLEDLQS

PCTEEVAVFHAFSRVVNEARSAFVVLDTAPTGHSLLLMDATGAYHRQMLQQESNAMHLITPLMRLQDASM

THVILVTLPEVTPVSQAAALQDDLRRAKIEPWAWVINKSIAATGTNDPLLKARLAGEHRQAARIAGGLAQ

RTFVLPWLPESPVGVRALEALAA

>Q1MAP0|Q1MAP0_R

QQMLTNPTRYLFFTGKGGVGKTSLSCASAITLCDGGKQVLLVSTDPASNLDEMLATPLRAIPTLVPSVPG

LYAMNINPEAAAEDYRERVLAQMAASASESERNTVREQLSGACTTEIAAFDAFVGLLADD--MSTFDHII

FDTAPTGHTLRLLSLPKAWTGFLEGNDRGASCLGPHSGLKMQEARFREALACLGDEKRTRIVLVARPDSS

ALREASRTSGELAALGLKNQLLAINGRFRATK-RDDPIAVGIERSQDAALSTMPVELSRLPSDEFPMLPF

DMVGLDALRALFA-QSQATIQEPVAAAQTT-LPSLAKLIDEIAGGRKGLVMVMGKGGVGKTTVAAALAIG

LVERGHAVHLTTTDPAAHLSFIVEGSMPGLTVDRIDPAAETKRYIEKVMSSRGRDLDEEGKALLREDLES

PCTEEVAVFHAFSRIVAEARDAFVVIDTAPTGHTLLLLDATGAYHRQMTRQET-PGRIVTPLMRLQDPEY

TRVILVSLPETTPVSEAAMLQEDLRRAKIEPYGWVVNRTMAASGTHDPLLQSRLAGERVQIDRITRKLAK

RAYILPFQAVPPVGIDALKRLSG

>E8RI92|E8RI92_D

KNFLEQPSRYLFFTGKGGVGKTALACASAIRLAEMGKRILLVSTDPASNLDEMLGIQLSLRATAVPGVDR

LSALNIDPELAATHYRERVIAPYRAIWTESQIAELQEQLAGACTVEIAAFDEFAELLAGSENEVPYDHVL

FDTAPTGHTLRLLQLPSAWTSFLQSTTRGASCLGPHSGLKMEEARFSAAFSALSDPERTIIVLVTRPDRA

ALREIARSSAELTELGLLNQQLVINAVFAAAD-PTDRTAAALERRGRVALIEMPDRLQSLIQMRIPLRPF

NMVGLPALRALLDTKAAETHSSVVQQTD---YPRLSGLIDEIAQSGKGLVMVMGKGGVGKTTIAAAIAVE

LASRGAKVHLSTTDPAAHLATTLEGSVENLSVSRIDPVLETKAYVDQVMSTRGARLNKDERALLAEDLRS

PCYEEIAVFTAFSRLIMQARSTFVILDTAPTGHTLLLLDTTGAYHRQVVGSDTESVGIVTPLMKLRDPKH

TKILLVTLAETTPVSEAARLQADLNRAGVEPYAWVINSSLTASGTRDPCLAQRVTAEIMQIDAVRTHHAR

RFAIVPWMPEEPVGPVRLLSLAR

>D7GG35|D7GG35_P

THLLEEMPRFLFLTGKGGVGKTSVACASAVALASAGRKVLLVSTDPASNVAQVFGQEIGNHITAISALPG

LDALEIDPQAAAEEYRARALAPVRDFLSAKDLASATEQLSGSCTTEIAAFNEFTDLLTAHGPGAGYDHVV

FDTAPTGHTVRLLKLPGEWSQFLSDGLGDPSCLGPMSGLEKTRDSYAEALGALADPGRTRLALVARAQES

SLHEASRTFDELLEAGIAATHLVINGLLPGAH-SDDALARSISHQESEAIGSAPARLRALVTDTLELRHG

DMVGVDALRTLLSTAQTESDVDVPRRPSVA-GPQLSELIAELSQRDHGLVMVMGKGGVGKTTLASALAMG

LADRRKDVLLTTTDPAAHLDWTIAGQAP-FDVTSIDPEVATRQYRDHVMATKGASLDEQGRANLAEDLRS

PCTEEVAVFQSFAQAVEQSDHRFVVMDTAPTGHTLLLMDATGSYHREVARN--PELASTTPLTRLRDPGH

TAVIIATLPETTPVLEASGLQDDLERAGIRPWAWVINRSLSATDTEDPFLQRRIEAETAPIAAARSN-CP

RTAQVAYLASPPVGLDALRAVAR

>E6SD05|E6SD05_I

MPYLANPPRFVFFTGKGGVGKTSLACATAVALTEGGASALLVSTDPASNVGQVFGITIGNVVTPVPGLPG

LDAIEIDPQQAAHAYRARIIDPVRQLLPAAEVAAMTEQLSGACTTEIASFNEFAALLADDARTARYDHVL

FDTAPTGHTIRLLQLPGEWTSYLADGKGDVSCLGPVAGLDRLRTDYAGALATLTDPARTRLVLVSRPQPS

ALREAARTLDELRDLGMHDAHLVINGTMPAG-LDDDPLAAAVTRREKAALGALPQRLASLPRTTIPLLPF

NTVGVPALRALLDSFEPPHAHPAAAVPDG--FAGLRTLVDQVEEDGPALVLCMGKGGVGKTTVAAAVALE

LARRGHEVLLTTTDPAAHLTETLAADVAHLTVSRIDPGRAITEYRARVLATKGASLDEAGRAVLAEDLKS

PCTDEVAVFQQFSQVVFRSRKQFVVLDTAPTGHTLLLLDATGSYHREIVRQMSPGAQYTTPLMRLQDPKT

TKVVLVTLPETTPVLEAEDLQADLARAGISPFAWVVNNSLTAAEPTSPFLRARAAAEAPALARVAE-LTS

RVAVVPLLANEPVGTAALSELTQ

>G4CWA3|G4CWA3_9

VQFLDDAPRHIFFTGKGGVGKTSVACATAVELTRRGKRVLLVSTDPASNVGHVFGQTIGNRMTPIDAVAR

LDALEIDPEQAVEAYRNRIIDPVRAILPASEIATITEQLSGSCTVEIASFNEFTNLLVDPSRTAGYDHVI

FDTAPTGHTIRLLQLPGDWTAYLDAGKGDASCLGPMSGLDKSKNTYRAAVEALTDSNVTRLVLVARAQPS

SLRETNRTLAELAEIGIQASHLVINGLLPHAD-DADPLRHAIEEREHAALEAMPAGLAALRRDDIPLKAT

TMIGVDALSRMFTDEAHR-SPDDVDLPE---QPGLDELVDDLASQDHGLVMTMGKGGVGKTTIAAAIATE

LARRGKKVLLTTSDPATHLAATLDGEMAGLTVDSIDPERATQAYRERVMATRGSSLDEEGRAALAEDLRS

PCTEEIAVFQEFSHAVNTARHQFVIMDTAPTGHTLLLMDATGSYHRDVLRHMDQRLHTTTPLMCLQDPEH

TKIIIVTLPDTTPVLEATSLVTDLARADIHPWAWVVNNSLAAAHPTSALLGRRARDEVTQIENVTAQ-AT

RWAVVPALANEPIGQAHLAALAS

>E4HMZ7|E4HMZ7_P

TQFLDNPPRHFFFTGKGGVGKTSIACATAVHLAHQGKRVLLVSTDPASNIAQVFGATIGNKITPIPQVPG

LDALEIDPDEAAEAYRNKILEPVRAVLPIKEIEAITEQLSGSCTTEIASFNEFTDLLADTQATAGYDHVI

FDTAPTGHTIRLLQLPGSWTSYLDNGKGDASCLGPMSGLEKNRATYRAAVEALTDPTSTRLVLVARAQQS

TLREVARTLDELAELDIHATNLVVNGVLPAVA-ATDDLSRAIHDREQAALAAMPAALAALPRDTVSLKSV

NMVGLEALATLFHEDAPQDAQDTQGIGQ---QPHLAGLVDQLAEADHGLVMTMGKGGVGKTTVAAAIAIA

LVQRGKKVLLTTTDPAAHLSTTLGNDVDGLEVSAIDPEKAIQEYRDHVMASKGAKLDDAGRAALAEDLMS

PCTEEIAVFQQFSRAVNKARDQFVIMDTAPTGHTLLLMDATGSYHRDITRHLDQRGHVTTPLMRLQDPDH

TKIIVVTLPETTPVTEAQALASDLERANIHPWAWVVNNSLAAAHPTSPLLATRARSEQQQLDAVAAT-TN

RMAVIPTQTREPVGTQQLTALSN

>A0JRE0|A0JRE0_A

VKFLQNAPRFLFFTGKGGVGKTSVACATALTLARAGRKVLLVSTDPASNVGQVFGVTIGNTVTAIQDVPG

LSALEIDPEQAVEAYRERIIAPVRGLLPETELAGIAESLSGSCTTEIASFDEFTNLLADDSSYREYDHIV

FDTAPTGHTIRLLQLPGSWTDFLAAGKGDPSCLGPLSGLEKHKQVYAKAVQALTDPAKTRLVLVSRAQTS

SLGEIERTYLELNQIGIGSGYVVVNGVLPDAAGE-EALAQALRAREAAAMEAIPDAVAGLPRDVLDLKPG

NMVGIPALESLFATSGAPTDDAALVP-EIE-DAPLAALVNEVELDGHGLVMCMGKGGVGKTTVAAAIAVA

LAKRGHAVHLTTTDPAAHLTETLHGSIPGLKVSRIDPEAAIQEYRIHVMETKGRNLDDDGRAALAEDLMS

PCTDEVAVFRQFSRVVQESRRHFVVIDTAPTGHTLLLLDATGSYHREIARQVGDTMGFVTPLMRLQDPAQ

TKVVLVTLAETTPVLEAEELKSDLERAGIHPWAWVINNSIAAAHPQTPFLRARAASEIEQITKVHT-LTD

RVALIPLLPEEPIGEEKLSALTV

>G4HTW8|G4HTW8_M

PRFLTDPPRFVFFTGKGGVGKTSIACASAVALARSGKAVLLVSTDPASNVGQVFGVPIGNTITDIPAVPG

LSALEIDPEQAASAYRERIVGPVRGVLPESAIASITEQLSGSCTTEVASFDEFTALLTDDGAVSRFDHVL

FDTAPTGHTIRLLQLPGNWTEFLEAGKGDASCLGPLAGLDKHKTTYAAAVAALANPDRTRLVLVTRPSRS

ALREIDRTRTELAALGMHRQYVVVNAVMPQQQNDSDPLADAIIRREQAALSGMPDALRVLPQDRIDLKAT

NMVGVEALQSLFATAGVDAGSEPAGIAPVA-DAPLVRLIDDIERGGTGLVMCMGKGGVGKTTVAAAIAVA

LANRGHDVHLTTTDPAAHLAETLQGGLAHLQVSRIDPVEANCAYRERVMATKGANLDEAGRATLAEDLRS

PCTEEVAVFQAFSKVIHESRSKFVVLDTAPTGHTLLLLDATGSYHREIARQMGDSGHYTTPLMRLQDPEL

TKVLLVTLAETTPVLEAAELQHDLERAGITPWAWVINNSVAAAAPTDPLLRQRAAAEVDQIDTVRTKLAT

RYAVIPLLTFEPVGSPALESLAA

>A1TFB6|A1TFB6_M

LRFLDAPPRFLFFTGKGGVGKTSIACAAAIHLARNGKRVLLVSTDPASNVGQVFGLRVGNAITTVPAVEG

LSALEIDPEQAAEAYRERIVAPVRGLLPDREVQSITEQLSGSCTTEIASFNEFTELLTDDALTGQFDHVL

FDTAPTGHTIRLLQLPGSWTDFLNEGKGDASCLGPLSGLDKQRAIYAEAVEALADPLRTRLVLVARAQRS

TLAEITRTHRELAAIGLTRQYVVINGVLASPAGNGDPLAAAIHTREQQAIAALPDELRGLPLDQVELKAT

NIVGVDALDSLFTDARIPGDDG-TGL-PVV-DAPLSALIDELAEGDHGLIMCMGKGGVGKTTIAAAIAVA

LADRGHPVHLTTTDPAGHLSGTLHGTLPNLHVSSIDPVEATRAYRDHVLATKGAALDEQGRSMLEEDLRS

PCTEEVAVFQAFSRVISESRNKFVVVDTAPTGHTLLLLDATGSYHREVARQLGNR-HFTTPLMRLQDPEL

TKVIVVTLAETTPVLEAAGLQSELERAQIQPWAWVVNNSLAAAHPTSPLLRQRAVAELPQIDKVRTDYAD

RVAVIPLLACEPVGIPALEALAG

>A4T7G2|A4T7G2_M

MKFLEAPPRFLFFTGKGGVGKTSIACAAAIHLAGSGKRVLLVSTDPASNVGQVLGLTIGNTITRVPAVDG

LSALEIDPDQAAEAYRERIIGPVRGLLPAPELQSITEQLSGSCTTEIASFNEFTELLTDDGPVKQFDHVL

FDTAPTGHTIRLLQLPGSWTDFLDAGKGDASCLGPLSGLGKQRALYAGAVDALADPQRTRMVLVARAQRS

ALTEIARTHGELAAIGLTHQHVVINGVLPACAGSGDPLAAAIYARERDAIATMPDELRALPLDQVDLKQV

NIVGLEALTTLFEDAVTPTDRE-VDF-AVA-DAPLAKLVDELATSEHGLIMCMGKGGVGKTTIAAAIAVA

LADRGHPVHLTTTDPAGHLTDMLHGTMENLHVSRIDPAEATRAYRDHVLATKGAALDDQGRVMLEEDLRS

PCTEEVAVFQAFSRVIAESRRKFVVVDTAPTGHTLLLLDATGSYHREVARQLGDR-HFTTPLMRLQDPDL

TKVIIVTLAETTPVLEAAGLESDLERAQIHPWAWVVNNSTSAAHPTAPLLRHRAVAELPEIDKVRTRHAD

RLAVVPLLAAEPVGIRALEALTG

>C1AS19|C1AS19_R

MKFLLAPPRFLFFTGKGGVGKTSIACATAITLARAGKRVLLVSTDPASNVGQVFGLTIGNTVTEVLTVPG

LSALEIDPEQAADAYRERIIGPVRGLLPEKELASITEQLSGSCTTEIASFNEFTALLTD-DTLADFDHVL

FDTAPTGHTIRLLQLPGSWTDFLNEGKGDASCLGPMSGLDKQRSIYEGAVAALADPQRTRLVLVARAQRS

PLAEIARTHTELADIGLTRQHVVINGVLPTPTGG-DELAAAIHQREQAALADLPTELAGLPLDLVELKAT

NMVGISALEGLFTNPDTPPDSA-PAP-AVP-EAPLSALVDELADDGHGLIMCMGKGGVGKTTLAAAIAVA

LAQRGHQVHLTTTDPAAHLTDTLDGNLAGLTVSRIDPAEATDAYRQRVLATKGAALDEQGRATLAEDLAS

PCTEEVAVFQAFSRIIHESRRKFVVVDTAPTGHTLLLLDATGSYHREIARQMGDNSNFTTPLMRLQNPQE

TKVLLVTLAETTPRLEAEGLQTDLQRAGIAPWAWVVNNSLAAAAPTSPLLRQRAATELEEIGAVTAAHP-

RFSVVPKLATDPVGVDALTAMVG

>C3JSD1|C3JSD1_R

MKFLNDAPRFLFFTGKGGVGKTSIACASAITLARAGKKVLLVSTDPASNVGQVFGVSIGNTITDIPAAPG

LSALEINPEQAAAAYRERIIGPVRGLLPEKEIAAIAEQLSGSCTTEIASFNEFTGLLSCGDITADFDHVL

FDTAPTGHTIRLLQLPGSWTEFLDDGRGDASCLGPLSGLEKQRAIYADAVAALADPQRTRLVLVSRAQRS

TLAEITRTHRELADIGLTHQHVVINGVLPAPGDDTDPLATAIYRREQAAIAGLPDELAQLPTDQVPLKAT

NIVGIDALESLFTDLSPVAASADPAL-QLP-SAPLSSLIDELDTDDHGLIMCMGKGGVGKTTIAAAIAVA

LAERGHQVHLTTTDPAAHLTETLNGELDNLQVSRIDPAEATEQYRTRVLTTKGKNLDERGRANLAEDLRS

PCTEEVAVFQAFSRVIHESSRKFVVVDTAPTGHTLLLLDATGSYHREIARQMGENTNFTTPLMRLQDPNA

TKVLLVTLAETTPVLEAAGLQADLQRAGIHPWAWVVNNSLAAAEPTSTLLQQRAAGEITEIDSITNKYSQ

RTAIVPMLAEEPVGTDALAALSK

>Q6XN05|Q6XN05_R

MKFLNDAPRFLFFTGKGGVGKTSIACASAITLARAGKKVLLVSTDPASNVGQVFGVSIGNTITDIPAAPG

LSALEIDPEQAAAAYRERIIGPVRGLLPEKEIAAIAEQLSGSCTTEIASFNEFTGLLSGGDITADFDHVL

FDTAPTGHTIRLLQLPGSWTEFLDDGKGDASCLGPLSGLEKQRAIYADAVAALADPQRTRLVLVSRAQRS

TLAEITRTHRELADIGLTHQHVVINGVLPDPGDDTDPLATAIYRREQAAIAALPDGLAQLPTDQVPLKAT

NIVGIDALESLFTDLGPVAASADPSL-QLP-SSPLASLIDELDTDDHGLIMCMGKGGVGKTTIAAAIAVA

LAERGHQVHLTTTDPAAHLTETLNGELDNLQVSRIDPTDATEQYRTRVLTTKGKNLDERGRANLAEDLRS

PCTEEVAVFQAFSRVIHESSRKFVVVDTAPTGHTLLLLDATGSYHREIARQMGENTNFTTPLMRLQDPNA

TKVLLVTLAETTPVLEAAGLQADLQRAGIEPWAWVVNNSLAAAEPTSPLLQQRAAGEITEIGTITSQYSQ

RTAIVPMLAEEPIGEAALAALSK

>A7MV37|A7MV37_V

INYLTDVPRYLFFTGKGGVGKTSHACASALALAESGKRVLIVSTDPASNVGQVFDTKIGETITVINHVEN

LFGMEIDPQAAAQKYRERIVGPIRGKLPELVVKSIEEQLSGACTTEVAAFDEFTGLLTDEYLSVDFDHIV

FDTAPTGHTIRLLQLPGAWNEFLEHGQGDASCLGPLAGLEKQRIKYANAIDVLSNENKTRLILVARAQSA

TIKEAAKTHLELSDIGLKGQHLVLNGLFPAEQSDDDDLAHAIIEREQKVIQELPESLAKLPLAQVPLLSS

NVLGLDSLRALIDQRK--ATNTIDTTTINVQYDSLSVMIEKLAEQKHGLVMLMGKGGVGKTTMAAAIAAK

LAEKGFDVHLTTSDPAAHLQYTLNSDETKFTVSKIDPASETARYRDTILNEKGASLDEDGRKLLEEDLRS

PCTEEIAVFQAFSEVIKQADKKFVIMDTAPTGHTLLLLDATGAYHKEMTRQ---NQNVETPLVMLQNPNL

TKVIITTLAEPTPVQEAYDLQQDLQRANINPWGWIVNNSLVQDGIQAELLKERAASQQPLIDKVIQQYST

HTAVVAMQKKEPVGVSALNALSD

>B5EVR6|B5EVR6_V

MNYLENVPPYLFFTGKGGVGKTSHACASALALAESGKKVLIVSTDPASNVGQVFDTEIGEYITAINHVEN

LFGMEIDPQAAAQNYRERIVGPIRGKLPDLIVKNIEEQLSGACTTEVAAFDEFTSLLTDESLQSDFDHIV

FDTAPTGHTIRLLQLPGAWNEFLEHGQGDASCLGPLAGLEKQRVKYANAIAVLSDESKTRLILVARAQSA

TIKEAAKTHIELSDLGLKGQHLVLNGLFPVAESSGDELALAIIEREQKIIEDLPRSLAKLPLTQVPLLSS

NVVGLDSLRVLLEQPI--VTRSVNSTAINVKYDSLSVMIDELAKQKHGLIMLMGKGGVGKTTMAAAIASK

LAEQGLDVHLTTSDPAAHLQFTLNSEDTKFTVSKIDPAAETTRYREAILKEKGASLDEDGRKLLEEDLRS

PCTEEIAVFQAFSEVIKQADKKFVIMDTAPTGHTLLLLDATGAYHKEMTRQ---NQNVATPLVMLQNSDV

TKVIITTLAEPTPVQEAYALQQDLQRANINPWGWIINNSLVQSDINAPLLKERAASQQPLIDKVINEYSS

RTAVVALQKKEPVGVLALNALSD

>B5RJY3|B5RJY3_K

MPFLKDIPPFIFFTGKGGVGKTSLACATAVWLADQGKRTLLVSTDPASNVGQVFSQTIGHRITDISTVEN

LAAMEVDPMAAAQAYRDRVLDPVRGLMPADVVSSIEEQLSGSCTTEIAAFDEFTGLLTNHELREKYDHIV

FDTAPTGHTIRMLELPGAWSGYLEANPDAAAHLGPLVGLEKQQHQYSDAVKALSDAALTRLVLVARAQAS

TLKEVSHTHDELSAIGLQHQHLAINGVLPPFAGENDPLAQSILAREEKALQAMPDNLANLPRSQLYLKPF

NLVGLEALRELFTSKAAPLPATTLNVLD---LPKLSSLVDELSQTGNGLVMTMGKGGVGKTTIAASVAVS

LAKRGHKVHLTTSDPAAHLSYTLDGSLPNLQVSRIDPKVETERYRRFVLENQGKGLDAEGLAVLEEDLRS

PCTEEIAVFQAFSRIIKEADDHFVIMDTAPTGHTLLLLDATGAYHREMVRQMGTHDNLLTPMMLLQDPEK

TKVIIVTLAETTPVLEAANLQQDLRRAEIEPWAWVINSSLAAAKPSSPFLVTRASRELPLINDVTEQFAE

RIALTPLQNEEPVGAALLAKMAG

>A9N3V2|A9N3V2_S

MPFLKDIPPFIFFTGKGGVGKTSLACATAVWLADQGKRTLLVSTDPASNVGQVFNQTIGHRITDISTVQN

LAALEVDPMAAAQAYRDRVLDPVRELMPADVVSSIEEQLSGSCTTEIAAFDEFTGLLTNHELREKFDHIV

FDTAPTGHTIRMLELPGAWSGYLEANPDAAANLGPLVGLEKQQHQYSDAVKALSDAALTRLVLVARAQAS

TLKEVSHTHDELSAIGLQHQHLAINGVLPPIAGENDPLAQSILAREEKALQAMPDNLANLPRSQLSLKPF

NLVGLEALRELFTSKAPLLSNTTPNGLD---LPKLSSLVDELSLTGKGLVMTMGKGGVGKTTVAASVAVL

LAKRGHKVHLTTSDPAAHLSYTLDGSLPNLQVSRIDPKVETERYRRFVLENQGKGLDAEGLAVLEEDLRS

PCTEEIAVFQAFSRIIKEADDHFVIMDTAPTGHTLLLLDATGAYHREMVRQMGTHDHVMTPMMQLQDPEK

TKVIIVTLAETTPVLEAANLQQDLRRAGIEPWAWVVNNSLAASEPSSPFLKTRASRELPLISDVEEQYAK

RIALTALQSEEPVGIDLLEEMAK

>Q8L248|Q8L248_S

MPFLKNIPPFIFFTGKGGVGKTSLACATAVWLADQGKRTLLVSTDPASNVGQVFSQTIGHRITDISTVEN

LAAMEVDPMAAAQAYRDRVLDPVRELMPADVVSSIEEQLSGSCTTEIAAFDEFTGLLTNHELREKYDHIV

FDTAPTGHTIRMLELPGAWSGYLEANPDAAANLGPLVGLEKQQHQYSDAVKALSDAALTRLVLVARAQAS

TLKEVSHTHDELSAIGLQHQHIAINGVLPPFAGEDDPLAQSILAREEKALQAMPDNLANLPRSQLYLKPF

NLVGLEALRELFTSKAAPLPATTLNTLD---LPKLSSLVDELSQTGKGLVMTMGKGGVGKTTVAASVAVS

LAKRGHKVHLTTSDPAAHLSYTLDGSLPNLQVSRIDPKVETERYRRFVLENQGKGLDAEGLAVLEEDLRS

PCTEEIAVFQAFSRIIKEADDHFVIMDTAPTGHTLLLLDATGAYHREMVRQMGTHDHVMTPMMQLQDPEK

TKVIIVTLAETTPVLEAANLQQDLRRAGIEPWAWVVNNSLAAAEPSSPFLRTRANRELPLISDVEEQHAE

RIALTALQSEEPVGIDLLEEMAK

>C1M6W1|C1M6W1_9

MPFLKDIPPFIFFTGKGGVGKTSLACATAVWLADQGKRTLLVSTDPASNVGQVFSQTIGHRITDISTVEN

LAAMEVDPMAAAQAYRDRVLDPVRGLMPTEVVSSIEEQLSGSCTTEIAAFDEFTGLLTNHELREKYDHIV

FDTAPTGHTIRMLELPGAWSGYLEANPDAAANLGPLVGLEKQQHQYSDAVKALSDAALTRLVLVARAQTS

TLKEVSHTHDELYAIGLQHQHLAINGVLPPFAGENDPLAQSILAREEKALQAMPENLANLPRSQLYLKPF

NLVGLEALRQLFTNKASLLPATTLNTLD---LPKLSSLVDELSQTGKGLVMTMGKGGVGKTTVAASVAVS

LAKRGHKVHLTTSDPAAHLSYTLDGSLPNLQVSRIDPKVETERYRRFVLENQGKGLDAEGLAVLEEDLRS

PCTEEIAVFQAFSRIIKEADDHFVIMDTAPTGHTLLLLDATGAYHREMVRQMGTHDHVMTPMMQLQDPEK

TKVIIVTLAETTPVLEAANLQQDLRRAGIEPWAWVVNNSLAAAEPSSPFLKTRASRELPLISDVGEQYAK

RIALTALQSQEPVGINLLEEMAK

>D7YAZ9|D7YAZ9_E

MPFLKDIPPFIFFTGKGGVGKTSLACATAVWLADQGKRTLLVSTDPASNVGQVFSQTIGHRITDISTVEN

LAAMEVDPMAAAQAYRDRVLDPVRGLMPADVVSSIEEQLSGSCTTEIAAFDEFTGLLTNHELREKYDHIV

FDTAPTGHTIRMLELPGAWSGYLEANPDAAANLGPLVGLEKQQHQYSDAVKALSDAALTRLVLVARAQAS

TLKEVSHTHDELSAIGLQHQHLAINGVLPPFAGENDPLAQSILAREEKALQAMPENLANLPRSQLYLKPF

NLVGLEALRQLFTNKASLLPATTLNTLD---LPKLASLVDELSLTGKGLVMAMGKGGVGKTTVAASIAVS

LAKRGHKVHLTTSDPAAHLSYTLDGSLPNLQVSRIDPKVETERYRRFVLENQGKGLDAEGLAVLEEDLRS

PCTEEIAVFQAFSRIIKEADDHFVIMDTAPTGHTLLLLDATGAYHREMVRQMGTHDHVMTPMMQLQDPEK

TKVIIVTLAETTPVLEAANLQQDLRRAGIEPWAWVVNNSLAAAEPSSPFLKTRANRELPLISDVEEQYAK

RIALTALQSEEPVGIDLLKEMAK

>Q1ZFE1|Q1ZFE1_9

QKFLTDPPPFLFFTGKGGVGKTSIACSSALFLAEQGHNVLLVSTDPASNIAQVFNTKIGEHITNIESVKG

LSALEIDPQAAAKAYREKILSPVRGKLPDAVLNSIEEQLSGACTTEIAAFDEFTALLTDPALRQTYQYII

FDTAPTGHTIRLLQLPGAWNEFLEQGLGDASCLGPLAGLDKQRKTYAQAVDALSDPEQTRLILVARAQAS

TLDEVARTHIELSHIGLKDQFLVINGLYPQQDIKEDALANAIYEQEQQALLMMPATLKNLHLDKLTLLSN

SLVSLDALRKLIASVDKNKKETEQSAPIIINHHSLVCLIDELDKLDHGLIMLMGKGGVGKTTIAAALACE

LAKRGHNVHLTTSDPAANLSATLHGTLENLEVTRIDPEIETTRYRQKIMLSKGKHLDSAGKALLEEDLRS

PCTEEIAVFQAFSQVISEANKRFVIMDTAPTGHTLLLLDATGAYHKEVARKMEKQISFSTPMMRLQDSTL

TKVIIVTLAEPTPIQEALSLQADLQRAGIEPWAWVVNNTLSLHTLKAPLMLQRVAQQQSYINAVKQQHSQ

KYAIVPLQAIEPVGVNALQKLCA

>Q1Z8S2|Q1Z8S2_P

IGFLHTPPPFLFFTGKGGVGKTSLACASALALAEQGNRVLLVSTDPASNVGQVFDTQIGNQITPIQHVEN

LSAIEIDPQAAAQAYREKIVGPVRGKLPEAVVNSIEEQLSGACTTEIAAFDEFTALLTDKTLQTTYQYIV

FDTAPTGHTIRLLQLPGAWNEFLEHGQGDASCLGPLAGLDKQREKYSQAVDALSDKTQTRLILVARAQAS

TLQEVARTHVELGDIGLKEQYLAINGLYPEAAIDNDALALAVYQREQHALVAMPEGLQRLPQDRLPLLSS

NIVGLDSLRALVSSVE---SVSETSDVTTIQYPHLDTLVDELAEAGHGLVMLMGKGGVGKTTIAAALATE

LALRGHDVHLTTSDPAAHLSETLNGSLANLEVSRIDPEVETQRYRERVMATKGKNLDEAGKALLEEDLRS

PCTEEIAVFQAFSRVIRDANKRFVVMDTAPTGHTLLLLDATGAYHKEVSRQMGKGMHFTTPMMQLQDPIL

TKVVITTLAEPTPVQEAVSLQSDLQRAGISPWAWVVNHALVQAPLTAPLMQERAAQQLPYIEAVQAQHSQ

RLAVVPLQHAEPIGTKALHALCC

>Q6LRX3|Q6LRX3_P

IGFLHTPPPFLFFTGKGGVGKTSLACASALALAEQGNKVLLVSTDPASNVGQVFDTQIGNQITPIQHVEK

LSAIEIDPQAAAQAYREKIVGPVRGKLPEAVVNSIEEQLSGACTTEIAAFDEFTALLTDKTLQTTYQYIV

FDTAPTGHTIRLLQLPGAWNEFLEHGQGDASCLGPLAGLDKQREKYAQAVDALSDKAQTRLILVARAQAS

TLQEVARTHVELGDIGLKEQYLAINGLYPEAAIDNDALALAVYQREQHALVAMPESLQQLPQDRLPLLSS

NIVGLDSLRALVSSVE---SVSEASNVTTIQYPHLDTLVDELAETGHGLVMLMGKGGVGKTTIAAALATE

LALRGNDVHLTTSDPAAHLSETLNGSLANLEVSRIDPEVETQRYRDRVMATKGKNLDEAGKALLEEDLRS

PCTEEIAVFQAFSRVIRDANKRFVVMDTAPTGHTLLLLDATGAYHKEVSRQMGKGMQFTTPMMQLQDPAL

TKVVITTLAEPTPVQEAVSLQADLQRAGISPWAWVVNHALIQAPLTAPLMQERAAQQLPYIEAVQVQHSQ

RLAVVPLQHAEPVGTEALHALCC

>G4T2A2|G4T2A2_M

MNFLQQLPKFLFFTGKGGVGKTSLSCATAVHLADAGKKVLLVSTDPASNVGQVFGTVIGHRIAPISEVGN

LEALEIDPVQAAALYRERIVGPIRGKLPESAVNSIEEQLSGACTTEIAAFDEFTALLTDAELIKRYDHIV

FDTAPTGHTIRLLQLPGAWGNFIESNPEGASCLGPLAGLEKQQQRYQQAVAALSDPSRTRLVLVARAQSS

TLQEVARTSGELADTGLSNQYLVINGLMPESE-TSDPLAEAIFQRESRALKSLLEPLRELKTDYTRLLPC

NLVGLNALRSLLNDQQTAATDGNYQLPD---LPSLQAMIDEMAQPGHGLIMLMGKGGVGKTTLSAAIAIA

LAERGYRVLLTTTDPAAHLTETLAGEIDNLTVSRIDPQAETERYRQHILETKGKKLDTQGRALLEEDLRS

PCTEEIAVFQAFSGAIREASAHFVVMDTAPTGHTLLLLDATGSYHREIARHMEKSLHFKTPMMRLQDPEQ

TKVLIATLAETTPVLEAANLQEDLRRAGIEPWAWIVNSSLVAARPTSALLRQRANEEQQQLALIRQDHAE

RLAIVPVQMREPVGVERLRALLS

>C9P417|C9P417_V

MKFLNDLPPYLFFTGKGGVGKTSISCATALHLVEQGKRVLLVSTDPASNVAQVFNQVIGNQITAITQIEH

LSAIEIDPQQAAEQYRQRIVGPLQGLLPSDALRSIEEQLSGACTTEIAAFDEFTDLLTNPELAQRFDHIV

FDTAPTGHTIRLLQLPGAWSSFIEANPEGASCLGPMAGLEKQREQYAHAVSMLADAKQTRLILVARAQRS

ALEEVARTYQELYSLGIKQQCLVVNGRMPESAARQDPLAASIRQREQAALQALPEILQGLSTDTIDLKAD

NMVGIKALASLLQESILEMPTQISQIKL--ESPTLANMIDDIAEDKHGLIMLMGKGGVGKTTLAASIAVG

LANKGFDVHLTTSDPAAHLQATLQGERPNLQVSRIDPREETERYRQHVLATKGKNLSAAEKALLEEDLRS

PCTEEIATFQAFSRAIRDSAQRFVVMDTAPTGHTLLLLDATGAYHKEIAKKMGDNQRFTTPLMQLQDPKR

TKVLIATLAETTPVLEAKHLQDDLVRAGIHPWGWIVNNALSVATIQSPLLQQRAQQECQQIKQVKD-YAQ

RIALVPMLVNEPVGIEALSQMAH

>B2Q3R5|B2Q3R5_P

MNFLDSTPNYLFFTGKGGVGKTSISCATAIKLAEEGKKVLLVSTDPASNVGQVFSQSIGNNIQPITLVPN

LFAIEIDPQAAAEEYRNKIINPIKESLPEAVIQSITEQLSGACTTEIAAFDEFTGLLTNTEITRQFDHII

FDTAPTGHTIRLLQLPSAWSDFISDNPDGATCLGPMSGLEKQREQYSMAVQALSDNSLTRLVLVARPQSA

ALREVARTYSELSSLGIKNQQLIINGVFPETATENDKLSHALYSREQAALKSLPEALRSLPIDILYLQNI

NMVGVDALKQLLSDMPQFTITPTKQPIS---LPSLSELVGEISQQQHGLIMLMGKGGVGKTTIAASIAVK

LAEKGLDVHLTTSDPAAHVESTLDGVLPNLQVSRIDPIAETERYRNYVLETKGKDLDAEGRALLEEDLRS

PCTEEIAVFQAFSRIIRDAGKRFVVMDTAPTGHTLLLLDATGAYHKEIAKKMGKKGHFLTPMMQLQDPER

TKVIITTLAETTPVLEAENLQNDLIRADIHPWAWVINNSLSITETTSPLLLSRAEQEVVQIEKVASTLAK

RVAIVPLLETEPVGISALSQLAE

>C7BK46|C7BK46_P

MKFLANIPDYLFFTGKGGVGKTSISCATAIKLAEEGKKVLLVSTDPASNVGQVFSQIIGNSIKQIPLVPN

LHAIEIDPQAAAEEYRNKIINPIKDSLPEAVIQSITEQLSGACTTEIAAFDEFTELLTNKEITDQFDHII

FDTAPTGHTIRLLQLPSAWRDFISDNPDGTSCLGPMSGLEKQREQYSMAVDALSDKQLTRLMLVARPQSA

ALREVARTYKELSDLGLKNQQLIINGVFPLSASENDKLSHALYRREQAAIQNMPEELRSLPKDMLYLQIL

NMVGIDALKQLLSKVQDLAIKAVKPKTF---LPTISTLVKEIALQKHGLIMLMGKGGVGKTTIAASIAVK

LAEQGLDVHLTTSDPAAHIENTLNGSLPNLQVSRIDPIAEIERYRNYVLETKGKDLNEEGRAILEEDLRS

PCTEEIAVFQAFSRIIREASKRFVVMDTAPTGHTLLLLDATGAYHKEIAKKWEKKGHFLTPMMQLQDPER

TKVIITTLPETTPVLEAENLQNDLMRADIHPWAWVINNSLSVTETTSPLLLSRAEQEVPQIEKVGSTLTK

RVAIVPLLEDEPVGVSALSKLAD

>C6HZR6|C6HZR6_9

MKFLENPPRFLFFTGKGGVGKTSTACAAAISLAESGKSVLLVSTDPASNIGQVFGLTIGHHVVAISSVPH

LSALEIDPEEAARVYRERLVGPVRGVLPDGVVREIEESLSGACTTEIAAFDEFTGLLTGSVLTAGFDHVL

FDTAPTGHTIRLLRLPGAWSGYLESGKGEASCLGPLAGLEKQRIQYSQAVEALADPLQTRLVLVSRPQRS

AIAEVARTHEELAGIGLTRQFLVINGILPETEGTADLLAASICQREQSAIRELPSVLARLPIDRISLKPF

NLVGLPALKQLLVEAPLVQ----KAPSPAVDAPTLDGLVDEMAREGKGMIMLMGKGGVGKTTLAAAIAVA

LARRGHSVHLTTSDPAAHLAETLEGSLDHLTVSRIDPLAETERYRQEVLRSKGGALDAEGLALLEEDLRS

PCTEEIAVFQALSTVIQQSKEKFVVLDTAPTGHTLLLLDATGAYHREVTRQAGGGQEVLTPLMQLQDPTQ

TKVMIVTLAETTPVLEAANLQDDLRRAGIVPWGWIVNQSVAATDTDSPLLRQRAAGELREIEAVATRHAT

RYAVVGLMREEPVGVERLAELVR

>C6I052|C6I052_9

MKFLENPPRILFFTGKGGVGKTSAACAAAISLAESGKSVLLVSTDPASNIGQVFDLTIGHHVVAIPSVPH

LSALEIDPEEAARLYRERLVGPVRGVLPDGVVKEIEESLSGGCTTEIAAFDEFTGLLTGSVLTAGFDHVL

FDTAPTGHTIRLLRLPGAWSGYLESGKGEASCLGPLAGLEKQRIQYSQAVEALADPLQTRLVLVSRPQRS

AIAEVARTHEELAGIGLTRQFLVINGILPETEGTADLLAASICQREQSAIRELPSVLARLPIDRISLKPF

NLVGLPALKQLLVEAPLVQ----KAPSPAVDAPTLDGLVDEMAREGKGMIMLMGKGGVGKTTLAAAIAVA

LARRGHSVHLTTSDPAAHLAETLEGSLDHLTVSRIDPLAETERYRQEVLRSKGGALDAEGLALLEEDLRS

PCTEEIAVFQALSKVIQQSTERFVVLDTAPTGHTLLLLDATGAYHREVTRQAGGGQEVLTPLMQLQDPTQ

TKVMIVTLAETTPVLEAANLQDDLRRAGIVPWGWIVNQSVAATDTDSPLLRQRAAGELREIEAVATRHAT

RYAVVGLMREEPVGIERLAGLVR

>B5NDZ5|B5NDZ5_S

MLMLRQVPPFLFFTGKGGVGKTSLACATAIHLTASGKRVLLVSTDPASNVAQVFEQTIGHQITPIAAVNG

LSALEVDPSAAAAKYRERIVGPVRGILPDDIVAGIEEQLSGACTTEIAAFDEFTALLTNQQLRDEYDHIV

FDTAPTGHTLRMLQLPGAWSGYLDNSQHGASCLGPLAGLEKQRSQYRAAVDALANAELTRMVLVARAQTA

TLKEVSRTYDELAAIGLTQQYLVINSLLPEQETAHDKLAQALYQREQQALQHLPDNLRTLPCDRLPLKPF

NMVGLSALRGLLDDSATGAPMETISPVD---LPSLSSLIDGFASQGYGLIMLMGKGGVGKTTLAAGIAVE

LARRGHPVHLSTSDPAAHLTNTLDGSFDGLTVSRIDPQAETERYRQQVMAEQGKNLDEQGRAVLEEDLRS

PCTEEIAVFQAFSRIIQEAGKQFVVMDTAPTGHTLLLLDATGAYHREIARLDGHGQPVLTPMMRLQDSEQ

TKVLIATLAETTPVLEAAHLQDDLRRAGIEPWGWVINNSLINTPTTSPLLRQRAERERPQIDAVCTHHAR

RCALVPLQAEEPVGVERLLQLST

>B5EWW9|B5EWW9_S

MLMLRQVPPFLFFTGKGGVGKTSLACATAIHLTASGKRVLLVSTDPASNVAQVFEQTIGHQITSVAAVNR

LSALEVDPSAAAAAYRERIVGPVRGILPDDIMAGIEEQLSGACTTEIAAFDEFTALLTNQQLRDEYDHIV

FDTAPTGHTLRMLQLPGAWSGYLDNSQHGASCLGPLVGLEKQRSQYRAAVDALANAELTRMVLVARAQTA

TLKEVSRTYDELAAIGLTQQYLVINGLLPEQETVRDKLAQALYQREQQALQHLPDNLRALPCDRLPLKPF

NMVGLAALRGLLDDSSTGSPAEVISPVD---LPSLSSLIDGFASQGHGLIMLMGKGGVGKTTLAAAIAVE

LARRGYPVHLSTSDPAAHLTDTLDGSFDGLSVSRIDPQAETERYRQQVMAEQGKNLDEQGRAVLEEDLRS

PCTEEIAVFQAFSRIIQEAGKQFVVMDTAPTGHTLLLLDATGAYHREIARLAGHGQPVLTPMMRLQDSEQ

TKVLIATLAETTPVLEAAHLQDDLRRAGIEPWGWVINNSLINTPTTSPLLRQRAERERSQIDAVCTHHAR

RCALVPLQAEEPVGVERLLQLST

>B5MS06|B5MS06_S

MLMLRQVPPFLFFTGKGGVGKTSLACATAIHLTASGKRVLLVSTDPASNVAQVFEQTIGHQITPIAAVNG

LAALEVDPSAAAAAYRERIVGPVRGILPDDIVAGIEEQLSGACTTEIAAFDEFTALLTNQQLRDEYDHIV

FDTAPTGHTLRMLQLPGAWSGYLDNSQHGASCLGPLAGLEKQRSQYRAAVDALANAELTRMVLVARAQTA

TLKEVSRTYDELAAIGLTQQYLVINGLLPDQEAAHDKLAQALYQREQQALQHLPDNLRALPCDRLPLKPF

NMVGLVALRGLLDDSATGAPVESIPPVD---LPSLSSLIDGFASQGHGLIMLMGKGGVGKTTLAAAIAVE

LARRGYPVHLSTSDPAAHLTDTLDGSFDGLTVSRIDPLAETERYRQQVMAEQGKNLDEQGRAVLEEDLRS

PCTEEIAVFQAFSRIIQEAGKQFVVMDTAPTGHTLLLLDATGAYHREIARLAGHGQPVLTPMMRLQDSEQ

TKVLIATLAETTPVLEAAHLQDDLRRAGIEPWGWVINNSLINTPTTSPLLRQRAERERSQIDAVCTHHAR

RCALVPLQAEEPVGVERLLQLST

>C4SZ55|C4SZ55_Y

DEILTTTPRFSLFTGKGGVGKTSISCASAITLADAGKRVLLVSTDPASNVGQVFNQTIGNTITAIAVVPG

LFALEIDPQVAAQLYRARIVDPVRGRLPEAVVRSIEEQLSGACTTEIAAFDEFTSLLTDASVLDDFDHVI

FDTAPTGHTIRLLQLPGAWSSFIDSNPEGSSCLGPLAGLEKQRDRYTHALQALSDAERTRLILVARAQKS

ALSEVARTHYELAAVGLRNQYLVINGILPEAEAQVDTLARALYLREQQALSELPEVLTGLPRDDLMLQSV

NMVGTDALRQLLLNETQHIS---DNPMAEVLLPTLSSLVEEIAADQHGLVMLMGKGGVGKTTLAASIAVK

LAEKGLDVHLTTSDPAAHIENTLNGQLNNLTVSRIDPLAETERYRRHVLSTKGKSLDAQGKALLEEDLRS

PCTEEIAVFQAFSRVIREAGKRFVVMDTAPTGHTLLLLDATGAYHREVEKRMGDNGHYLTPMMQLQDEKR

TKVLLVTLPETTPVLEAAILQEDLRRAGIEPWAWLINNSLAAAATSSPLLQLRAAHEVEQIKKVRDILSA

RLALIPLQEEEPIGIARLSHLAG

>D5CDS0|D5CDS0_E

MNYLNTIPKFLFFTGKGGVGKTSLSCATAIRLADEGKRILLVSTDPASNVGQVFGQQIGNTLTVISDVPG

LTALEIDPQAAAQQYRARIVDPVKGILPEDVVRSIEEQLSGACTTEIAAFDEFTGLLTDDALLNDFDHVI

FDTAPTGHTIRLLQLPGAWSSFIETNPDGASCLGPLAGLEKQRERYSHALSVLADGEKTRLILVARAQQS

TLAEVARTHDELLHVGLKHQYLVINGIMPAGEASEDSLASALYQREQRILSHMPQVLASLPTDRLSLQQE

NLVGVTALRRLLTENKPCLPAAKSEPLS---VPSLEMLIDDIARGGHGLVMLMGKGGVGKTTLAAAVAVA

LADKGFDVHLTTSDPAAHLESTLNGQLPHLQVSRIDPHAETARYREHVLATKGKELDPQGRALLEEDLRS

PCTEEIAVFQAFSRVIREAGKRFVVMDTAPTGHTLLLLDATGAYHREVVKRMGEKGHYLTPMMQLQDPER

TKIMLVTLPETTPVLEAAGLQEDLRRAGIEPWAWLINNSLVATETTSPLLLTRAAHEAAQINKVRTELAS

RMALIPLQQEEPTGIEQLKRLTR

>Q79JC2|Q79JC2_S

MKFIKNPPAFLFFTGKGGVGKTSLSCATAINLADKGKRVLLVSTDPASNVGQVFGQTIGNQLTPIDSVAG

LTALEIDPQAAAAQYRARIVDPVKGILPPDVVRSIEEQLSGACTTEIAXFDEFTGLLTDDSLQQDFDHII

FDTAPTGHTIRLLQLPGAWSSFIEANPEGASCLGPLAGLEKQAERYAQALIALADPDKTRLILVARPQQS

TLIEVERTHQELRQVGLKNQYLVINGVLPQNAALDDPLANALYRREQAVLANLSPILAALPHETLPLQSM

NMVGVAPLRQLLLPAQPNLLNINEQSHQGSHVPTLDNLRAEIAKQDHGLIMLMGKGGVGKTTLAAAIAVR

LAELGLDVHLTTSDPAAHLEHTLHGQLANLQVSRIDPVEVTTRYREQVLATKGKELDAQGKALLEEDLRS

PCTEEIAVFQAFSRIIREAGKRFVVMDTAPTGHTLLLLDATGAYHREVAKRMGETTHYSTPMMQLQDKER

XKVLLVTLPETTPVLEAANLQEDLRRAGIEPWAWLINNSLAVARTTSPLLKVRARHEVAQIDKVQRGLAS

RLALIPLQEEEPIGIERLSQLAK

>A1RM70|A1RM70_S

MKFIQNPPAFLFFTGKGGVGKTSLSCATAINLADKGKRVLLVSTDPASNVGQVFGQTIGNQLTPIDSVAG

LTALEIDPQAAAEQYRNRIVDPVKGLLPPDVVRSIEEQLSGACTIEIAAFDEFTGLLTDESLQQDFDHII

FDTAPTGHTIRLLQLPGAWSSFIETNPEGASCLGPLAGLEKQAERYAQALTALADPDKTRLILVARPQQS

TLIEVERTHQELRQVGLKNQYLVINGVLPQSATLKDALANALYQREQTVLANLSPILAALPHETLPLQSV

NMVGVAPLRQLFMPMQDLLPAIDTLPIDEQQVPSLESLIDEIAQQDHGLIMLMGKGGVGKTTLAAAIAVR

LAELGLDVHLTTSDPAAHLEDTLHGQLANLQVSRIDPVDVTTRYREQVLATKGKDLDAQGKALLEEDLRS

PCTEEIAVFQAFSRIIREAGKRFVVMDTAPTGHTLLLLDATGAYHREVAKRMGETAHYSTPMMQLQDKLR

TKVLLVTLPETTPVLEATNLQEDLRRAGIEPWAWLINNSLAVARTTSPLLKVRARHELAQIEKVQQGLAS

RLALIPLQQEEPIGITRLSQLAQ

>D6IVS0|D6IVS0_E

MKFLQNIPPYLFFTGKGGVGKTSISCATAIHLAEQGKRVLLVSTDPASNVGQVFNQTIGNAITPVTAVPD

LSALEIDPQQAAEQYRARIVDPIKGLLPEEVVNSIREQLSGACTTEIAAFDEFTGLLTDASLLARFDHII

FDTAPTGHTIRLLQLPGAWSSFIESNPDGASCLGPMAGLEKQREQYADAVEALSDPERTRLVLVARLQKS

TLQEVARTHEELAAIGLKNQYLVINGVLPESGQQNDPLAMAIVQREQEALVNLPAGLSTIPADTLFLQPV

NMVGVPALKGLFNTRFEPAPLSGQVRQQFSENMSFSELVNDIARSEHGLIMLMGKGGVGKTTMAAAIAVR

LADMGFDVHLTTSDPAAHINTTLNGTLNNLQVSRINPQEETERYRQHVLETKGKDLDEAGKRLLEEDLRS

PCTEEIAVFQAFSRVIREAGKRFVVMDTAPTGHTLLLLDATGAYHREIAKKMGEKGHFTTPMMQLQDPQR

TKVLLVTLPETTPVLEAANLQADLKRAGIHPWGWIINNCLSIAETRSSLLCQRATQERPQIEAVMARHAK

RVALVPLFAQEPTGIEKLRKLAS

>A1JPZ3|A1JPZ3_Y

MKFLQNIPPYLFFTGKGGVGKTSISCATAIRLAEQGKRVLLVSTDPASNVGQVFSQTIGNTIQPIASVPG

LSALEIDPQAAAQQYRARIVDPIKGVLPDDIVSSINEQLSGACTTEIAAFDEFTGLLTDASLLTRFDHII

FDTAPTGHTIRLLQLPGAWSSFIDSNPEGASCLGPMAGLEKQREQYAHAVEALSDPKRTRLVLVARLQKS

TLQEVARTHLELAAIGLKNQYLVINGVLPKTEAANDTLAATIWDREQEALANLPSELSGLPTDTLFLQPV

NMVGVSALSGLLSTQPVAVSSSEEYIQQRPDIPPLSALVDDIARNEHGLIMLMGKGGVGKTTMAAAIAVR

LAEMGFDVHLTTSDPAAHLSTTLNGSLNNLQVSRIDPHEETERYRQHVLETKGKELDEAGKRLLEEDLRS

PCTEEIAVFQAFSRVIREAGKRFVVMDTAPTGHTLLLLDATGAYHREIAKKMGDKGHFTTPMMQLQDPER

TKVLLVTLPETTPVLEAANLQADLERAGIHPWGWIINNSLSIADTRSPLLRLRAQQERPQIESVKLQHAS

RVALVPLLASEPTGIDKLRQLAG

>B5LUS5|B5LUS5_E

--------------RRRGVGKTFISCATAIRLAEQGKRVLLVSTDPASNVGQVFSQTIGNTIQAIASVPG

LSALEIDPQAAAQQYRARIVDPIKGVLPDDVVSSINEQLSGACTTEIAAFDEFTGLLTDASLLTRFDHII

FDTAPTGHTIRLLQLPGAWSSFIDSNPEGASCLGPMAGLEKQREQYAYAVEALSDPKRTRLVLVARLQKS

TLQEVARTHLELAAIGLKNQYLVINGVLPKSEAANDTLAAAIWEREQEALANLPADLAGLPTDTLFLQPV

NMVGVSALSRLLSTQPVASPSSDEYLHQRPDNPSLSALVDDIARNEHGLIMLMGKGGVGKTTMAAAIAVR

LADMGFDVHLTTSDPAAHLSMTLSGSLNNLQVSRIDPHEETERYRQHVLETKAIELDEAGKRLLEEDLRS

PCTEEIAVFQAFSRVIREAGKRFVVMDTAPTGHTLLLLDATGAYHREIAKKMGEKGHFTAPMMLLQDPER

TKVLLVTLPETTHVLEAANLQAALERAGIHPW--------------------------------------

-------------------ELDY

>P08690|ARSA1_EC

MQFLQNIPPYLFFTGKGGVGKTSISCATAIRLAEQGKRVLLVSTDPASNVGQVFSQTIGITIQAIASVPG

LSALEIDPQAAAQQYRARIVDPIKGVLPDDVVSSINEQLSGACTTEIAAFDEFTGLLTDASLLTRFDHII

FDTAPTGHTIRLLQLPGAWSSFIDSNPEGASCLGPMAGLEKQREQYAYAVEALSDPKRTRLVLVARLQKS

TLQEVARTHLELAAIGLKNQYLVINGVLPKTEAANDTLAAAIWEREQEALANLPADLAGLPTDTLFLQPV

NMVGVSALSRLLSTQPVASPSSDEYLQQRPDIPSLSALVDDIARNEHGLIMLMGKGGVGKTTMAAAIAVR

LADMGFDVHLTTSDPAAHLSMTLNGSLNNLQVSRIDPHEETERYRQHVLETKGKELDEAGKRLLEEDLRS

PCTEEIAVFQAFSRVIREAGKRFVVMDTAPTGHTLLLLDATGAYHREIAKKMGEKGHFTTPMMLLQDPER

TKVLLVTLPETTPVLEAANLQADLERAGIHPWGWIINNSLSIADTRSPLLRMRAQQELPQIESVKRQHAS

RVALVPVLASEPTGIDKLKQLAG

>E3G6Q1|E3G6Q1_E

MKFLQNIPPYLFFTGKGGVGKTSISCATAIRLAEQGKRVLLVSTDPASNVGQVFGQTIGNTIVSVAAVPG

LCALEIDPQAAAQQYRERIVNPVKGLLPDDVVSSINEQLSGACTTEIAAFDEFTGVLTDADLLTRFESII

FDTAPTGHTIRLLQLPGAWSRFIDSNPDGASCLGPMAGLEKQREQYAQAVAALSDPERTRLVLVARLQKS

TLLEVARTHEELAAIGLKNQYLVINGVLPETETETDALAVAIWQREQEALAHLPAGLSALPTDTLFLQPV

NMVGVSALKGLLTPGSAAAMLPETTTPDKTDNLSLSELVDDIARSEHGLIMLMGKGGVGKTTMAAAIAVR

LADMGFDVHLTTSDPAAHLSTTLNGSLNHLQVSKINPHDETERYRQHVLATKGRDLDEAGKRLLEEDLRS

PCTEEIAVFQAFSRVIREAGKRFVVMDTAPTGHTLLLLDATGAYHREIARKMGDNGHFTTPMMQLQDPER

TKVLLVTHAETTPVLEAANLQADLERAGIHPWGWIINNSFAIADTRSPLLCQRARQELPQIEAVKRQHAD

RIALVPVLASEPAGIEKLRALTV

>C1MF02|C1MF02_9

MHFLQNIPPYLFFTGKGGVGKTSISCATAIRLAEQGKRVLLVSTDPASNVGQVFDQTIGKTIQPVTAVSG

LSALEIDPQDAAQQYRARIVDPIKGLLPDDVVNSISEQLSGACTTEIAAFDEFTGLLTDASLLTRFDHII

FDTAPTGHTIRLLQLPGAWSSFIESNPDGASCLGPMAGLEKQREQYAHAVEALSDPERTRLVLVARLQKS

TLQEVARTHGELSAIGLKNQYLVINGVLPASETERDALAAAIWQREQEALANLPARLSDLPTDNLYLQPL

NMVGVSALKGLLNEHAEITSLPERSTQNKPENMSLSVLVDDIARSEHGLIMLMGKGGVGKTTMAAAIAVS

LADKGFDVHLTTSDPAAHLSTTLNGSLKNLQVGRINPHDETERYRQHVLETKGRDLDEAGKRLLEEDLRS

PCTEEIAVFQAFSRVIREAGKRFVVMDTAPTGHTLLLLDATGAYHREIARKMEDKGHFTTPMMQLQDPER

TKVLLVTLPETTPVLEAANLQSDLERAGIHPWGWIINNSLSIAQTQSPLLCQRARQELPQIEVVKNQHAS

CIALVPVMAAEPTGIEKLRELAV

>B5LUS6|B5LUS6_E

--------------------KTSISCATAIRLAELGKRVLLVSTDPASNVGQVFDQTIGNTIQPVTAVSG

LSALEIDPQDAAQQYRARIVDPIIGLLPDDVVNSISEQLSGACTTEIAAFDEFTGLLTDASLLTRFDHII

FDTAPTGHTIRLLQLPGAWSSFIESNPDGASCLGPMAGLEKQREQYAHAVEALSDPERTRLVLVARLQKS

TLQEVARTHDELSAIGLKNQYLVINGVLPASEEKRDALAAAIWQREQEALANLPAGLSDLPTDNLYLQPL

KMVGVSALKGLLNEHAEITSLPEQSPQNKPENMSLSVLVDDIARSEHGLIILMGKGGVGKTTMAAAIAVS

LADKGFNVHLTTSDPAAHLSTTLNGSLKNLQVSRVNPLDETERYRQHVLETKGRDLDEAGKRLLEEDLRS

PCTEEIAVFQAFSRVIREAGKRFVVMDTAPTGHTLLLLDATGAYHREIARKMGDKGHFTTPMMQVQDQER

TKVLLVTLPETTPVLEAANLQSDLERAGIHPWGWIINISLWIAQTQSPLLCQRAIHERPQIEVVKNQHAS

RIALVPVMAAEPTGIET------

>P52145|ARSA2_EC

MKFLENIPSYLFFTGKGGVGKTSISCATAIRLAELGKRVLLVSTDPASNVGQVFDQTIGNTIQPVTAVSG

LSALEIDPQDAAQQYRARIVDPIIGLLPDDVVNSISEQLSGACTTEIAAFDEFTGLLTDASLLTRFDHII

FDTAPTGHTIRLLQLPGAWSSFIESNPDGASCLGPMAGLEKQREQYAHAVEALSDPERTRLVLVARLQKS

TLQEVARTHDELSAIGLKNQYLVINGVLPASEEKRDALAAAIWQREQEALANLPAGLSDLPTDNLYLQPL

NMVGVSALKGLLNEHAEITSLPEQSPQNKPENMSLSVLVDDIARSEHGLIMLMGKGGVGKTTMAAAIAVS

LADKGFNVHLTTSDPAAHLSTTLNGSLKNLQVSRINPHDETERYRQHVLETKGRDLDEAGKRLLEEDLRS

PCTEEIAVFQAFSRVIREAGKRFVVMDTAPTGHTLLLLDATGAYHREIARKMGDKGHFTTPMMQLQDQER

TKVLLVTLPETTPVLEAANLQSDLERAGIHPWGWIINNSLWIAQTQSPLLCQRALQERPQIEVVKNQHAS

RIALVPVMAAEPTGIEKLRELVV

>A9N3W0|A9N3W0_S

MEMLKQVPVFLFFTGKGGVGKTSISCATAIRLVEQGKRVLLVSTDPASNVGQVFNQTIGNTILPVTAVPG

LSALEIDPQAAAQQYRARIVDPIKNLLPDDVVSSISEQLSGACTTEIAAFDEFTGLLTDASLLTRFDHII

FDTAPTGHTIRLLQLPGAWSSFIESNPDGASCLGPMAGLEKQREQYAHAVEALSDPERTRLVLVARLQKS

TLQEVARTHDELSAIGLKNQYLVINGVLPKSETENDVLAAAIWQREQEALANLPAGLSELPRDTLFLQPV

NMVGVAALKGLLREHSEPAPLHEQSTQYKPENMSLSGLVDDIARSEHGLIMLMGKGGVGKTTLAAAIAVR

LADMGFDVHLTTSDPAAHLSTTLNGSLKNLQVSRINPHDETERYRQHVLETKGRDLDEAGRRLLEEDLRS

PCTEEIAVFQAFSRVIREAGKRFVVMDTAPTGHTLLLLDATGAYHREIAKKMGNKGHFTTPMMQLQDPDR

TKVLLVTLPETTPVLEAANLQADLERAGIHPWGWIINNSLSIADTHSPLLCQRAQQEQPQIEVVKHQYAD

RIALVPVLASEPTGIEKLRELVG

>Q9KJI3|Q9KJI3_K

MKFLQNIPPYLFFTGKGGVGKTSISCATAIRLAEQGKRVLLVSTDPASNVGQVFDQAIGNTIRPVTAVHG

LSALEIDPQNAAQQYRARIVDPIKGLLPDDVVNSISEQLSGACTTEIAAFDEFTGLLTDGFLLTRFDHII

FDTAPTGHTIRLLQLPGAWSSFIESKPDGASCLGPMAGLEKQREQYAHAVEALSDPERTRLVLVARLQKS

TLQEVARTHEELSAIGLKNQYLVINGVLPKAEAEHDGLAAAIWQREQEALANLPSGLSELPTDTLLLQPV

NMGGVSALKGLLDTRSETLPLPVNEHPCTRENLSLSGLVDDIARSEHGLIMLMGKGVVGKTTMGAGIAVR

VAEMGFDVFLTTFDPGAHLSTTLNGSLKNLQVSRINPHDETERYRQHVLETKGRDLDEAGKRLLEEDLRS

PCTEEIAVFQAFSRVIREAGKRFVVMDTAPTGHTLLVLDATGAYHREIAKKMGSKGHFTTPMMQLQDPDR

TKVLLVTLPETTPVLEAANLQADLERAGIHPWGWIINNSLSIADTRSPLLCQRARQEQPQIEAVKNQYAN

RIALVPVLTSEPAGIEKLRELMS

>B5RJX9|B5RJX9_K

MKFLQNIPPYLFFTGKGGVGKTSISCATAIRLAEQGKRVLLVSTDPASNVGQVFDQAIGNTIRPVTAVHG

LSALEIDPQDAAQQYRARIVDPIKGLLPDDVVNSISEQLSGACTTEIAAFDEFTGLLTDASLLTRFDHII

FDTAPTGHTIRLLQLPGAWSSFIESNPDGASCLGPMAGLEKQREQYAHAVEALSDPERTRLVLVARLQKS

TLQEVARTHEELSAIGLKNQYLVINGVLPKAEAEHDALAAAIWQREQEALANLPSGLSELPTDTLLLQPV

NMVGVSALKGLLDIRSETLPLPVTNILYTPENLSLSGLVDDIARSEHGLIMLMGKGGVGKTTMAAAIAVR

LADMGFDVHLTTSDPAAHLSTTLNGSLKNLQVSRINPHDETERYRQHVLETKGRDLDEAGKRLLEEDLRS

PCTEEIAVFQAFSRVIREAGKRFVVMDTAPTGHTLLLLDATGAYHREIAKKMGSKGHFTTPMMQLQDPDR

TKVLLVTLPETTPVLEAANLQADLERAGIHPWGWIINNSLSIADTRSPLLCQRARQEQPQIETVKNQYAN

RIALVPVLTSEPAGIEKLREFMS

>C1M6V8|C1M6V8_9

MKFLQNIPPYLFFTGKGGVGKTSISCATAIRLAEQGKRVLLVSTDPASNVGQVFDQAIGNTIHPVAAVPG

LSALEIDPQEAAKQYRSRIVDPIKGLLPDDVVNSISEQLSGACTTEIAAFDEFTGLLTDASLLTRFDHII

FDTAPTGHTIRLLQLPGAWSSFIESNPDGASCLGPMAGLEKQREQYAHAVEALSDPERTRLVLVARLQKS

TLQEVARTHEELAAIGLKNQYLVINGVLPKAETEYDALAAAIWQREQEALANLPAGLSELPTDTLLLQPV

NMVGVSALKGLLDTRSEALPLPVTNILYTPENLSLSGLVDDIARSEHGLIMLMGKGGVGKTTMAAAIAVR

LADMGFDVHLTTSDPAAHLSTTLNGSLKNLQVSRINPHDETERYRQHVLETKGRDLDEAGKRLLEEDLRS

PCTEEIAVFQAFSRVIREAGKRFVVMDTAPTGHTLLLLDATGAYHREIAKKMGSKGHFTTPMMQLQDPER

TKVLLVTLPETTPVLEAANLQADLERAGIHPWGWIINNSLSIADTRSPLLCQRARQERPQIEAVKNQHAE

RIALVPVLASEPAGIKKLRELMS

>C9Y5U8|C9Y5U8_C

MKFLQNIPPYLFFTGKGGVGKTSISCATAIRLAEQGKRVLLVSTDPASNVGQVFDQAIGNTIRPVTAVPA

ISALEIDPQEAARQYRARIVDPIKGLLPDDVVNSISEQLSGACTTEIAAFDEFTGLLTDASLLTRFDNII

FDTAPTGHTIRLLQLPGAWSSFIESNPDGASCLGPMAGLEKQREQYAHAVEALSDPERTRLVLVARLQNS

TLQEVARTHEELAEIGLKNQYLVINGVLPEAEAEHDALAAAIWQREQEALANLPAGLSELPTDTLLLQPV

NMVGVSALKGLLDTRSEALPLPVTNILYTPENLSLSGLVDDIARSEHGLIMLMGKGGVGKTTMAAAIAVR

LADMGFDVHLTTSDPAAHLSTTLNGSLKNLQVSRINSHDETERYRQHVLETKGRDLDEAGKRLLEEDLRS

PCTEEIAVFQAFSRVIREAGKRFVVMDTAPTGHTLLLLDATGAYHREIAKKNGE----------------

----------------------------------------------------------------------

-----------------------

>B5LUS4|B5LUS4_E

---------YFHFLRD------------AIHLAEQGKRVLLVSTDPASNVGQVFDLAIGNTIRPVTAVPG

LSALEIDPQEAARQYRATIVDPIKGLLPDDVVNSISEQLSGACTTEIAAFDEFTGLLTDASLLTRFDHII

FDTAPTGHTIRLLQLPGAWSSFIESNPDGASCLGPMAGLEKQREQYAHAVEALSDPERTRLVLVARLQNS

TLQEVARTHEELAEIGLKNQYLVINGVLPEAEAEHDALAAAIWQREQEALANLPAGLSELPTDTLLLQPV

NMVGVSALKGLLATRSEALPLPVTNILYTPENLSLSGLVDDIARSEHGQIMLMGKGGVGKTTMTAAIAVR

LADMGFDVHLTTSDPAAHLSTTLNGSLKNLQVSRINPHDETERYRQHVLETKGRDLDEAGKRLLEEDLRS

PCTEEIAVFQAFSRVIREAGKRFVVMDTAPTGHTLLLLDATGAYHREIAKKMGSKGHFTTPMMQLQDPDR

TKVLLVTLPETTPVLEAANLQADLERAGIHPWSWIIINSLSIADTRSPLLCQRAQQELPQIEAVKNQHAD

-----------------------

>F3Q2G9|F3Q2G9_9

MKLLQNIPPYLFFTGKGGVGKTSISCATAIHLAEQGKRVLLVSTDPASNVGQVFDLAIGNTIRPVTAVPG

LSALEIDPQEAARQYRARIVDPIKGLLPDDVVNSISEQLSGACTTEIAAFDEFTGLLTDASLLTRFDHII

FDTAPTGHTIRLLQLPGAWSSFIESNPDGASCLGPMAGLEKQREQYAHAVEALSDPERTRLVLVARLQNS

TLQEVARTHEELAEIGLKNQYLVINGVLPEAEAEHDALAAAIWQREQEALANLPAGLSELPTDTLLLQPV

NMVGVSALKGLLATRSEALPLPVTNILYTPENLSLSGLVDDIARSEHGLIMLMGKGGVGKTTMAAAIAVR

LADMGFDVHLTTSDPAAHLSTTLNGSLKNLQVSRINPHDETERYRQHVLETKGRDLDEAGKRLLEEDLRS

PCTEEIAVFQAFSRVIREAGKRFVVMDTAPTGHTLLLLDATGAYHREIAKKMGSKGHFTTPMMQLQDPDR

TKVLLVTLPETTPVLEAANLQADLERAGIHPWGWIINNSLSIADTRSPLLCQRAQQELPQIEAVKNQHAD

RIALVPVLASEPAGIEKLRELMS

>A1XP70|A1XP70_9

MKFLSDAPKFIFFTGKGGVGKTSLSCATAIHLAEQGKQVLLVSTDPASNVGQVFGRTIGNKITAIDTVRG

LSALEIDPQAAAQQYRDRVIEPVRAALPADVIKGMEEQLSGACTTEIAAFDEFTALLTDSELVEQYDHIV

FDTAPSGHTIRLLQLPGAWSGFIDKNPDGASCLGPLTGLDKQRQRYAEAVQALSDPARTRLVLVARAQKS

TLDEVARTHAELAEIGLSKQHLVINGVLPAVEAEHDMLAAAVYRREQAAIAEIPAALRQLPLDQLPLKAF

NMVGVEALRRLFNAADDAGAVAPEIRATPANLPSLAVLVDELAANGHGLIMMMGKGGVGKTTLAAAVAVG

LAKRGLPVHLTTSDPAAHLSDTLAGSLDNLEVSRIDPRAETERYRQHVLATKGQDLDAEGRAMLEEDLRS

PCTEEIAVFQAFSRIIREADKKFVV--TAPTGHTLLLLDATGAYHREVARHMEGKAHYTTPMMQLQDPAR

TKVMVVTLAETTPVLEAARLQDDLRRAGIEPWAWVINNSLAAGETEAPLLKQRAGHELAQIEAVRTEHAK

RVALVPVQTEEPVGVERLLALAE

>G0JS75|G0JS75_9

MLFLQNPPTFLFFTGKGGVGKTSLSCATAIHLVRLGKKILLVSTDPASNVGQVFSQAIGNKITAISAVPG

LFALEIDPQQAAQAYRERIVGPVRGALPDAVVKGIEEQLSGACTTEIAAFDEFTALLTDATLTVDYDHII

FDTAPTGHTIRLLQLSGAWSGFIEKNPEGASCLGPLAGLEKQRQRYAEAVKALSDPARTRLILVARAQKT

TLDEVARTHQELAAIGLTRQNLVINGVLPAEEAVHDALAAAIRQREQAAIASMPTALRDLPVDQLPLKAF

NMVGVAALCHLFSDQDESAPAASAAPTERIELPQLSTLVEAIAGTGHGLIMMMGKGGVGKTTLAAAVAVA

LAERGLPVHLTTSDPAAHLSDTLAGSLGNLEVSRIDPELETERYRQQVLDTKGQDLDAEGCAMLEEDLRS

PCTEEIAVFQAFSRVIREAGKQFVVMDTAPTGHTLLLLDATGAYHREVTRHVDPQVHYTTPMMQLQDPER

TQVLIVTLPEPTPVLEAAQLQEDLRRAGIEPWAWVINNSLAAAPTASPLLKQRAALEVAQIEAVRTRYAK

RVALVPMQTEEPVGIDHLRALLN

>Q3T560|Q3T560_9

MLFLQNPPAFLFFTGKGGVGKTSLSCATAIHLAGQGKKILLVSTDPASNVGQVFSQEIGNKITTISTVAG

LSALEIDPQQAAQQYRERIVGPVRGALPDDVVKGIEEQLSGACTTEIAAFDEFTALLTDATLIADYDHII

FDTAPTGHTIRLLQLPGAWSGFIEKNPEGASCLGPLAGLEKQRQRYAEAVKALSDPERTRLILVARAQKT

TLDEVARTHEELAAIGLSRQNLVINGVLPESEAVHDALAAAIHRREQEAIANMPAVLRDLPLDQLPLKAF

NLVGVEALSSLFSDRDEAAPVVAGAPAKPVDLPPLSTLVDEIAETGHGLIMMMGKGGVGKTTLAAAVAVA

LAERGLPVHLTTSDPAAHLTDTLAGSLDNLEVSRIDPQAETERYRQHVLVTKGKDLDAEGRAMLEEDLRS

PCTEEIAVFQAFSRVIREAGKKFVVMDTAPTGHTLLLLDATGAYHREVDRHAESNVRYTTPMMQLQDPAR

AKVMIVTLAETTPVLEAANLQEDLRRAGIEPWAWLINNSLSAAPTASPLLKRRAAFELTQIEAVRTRYTK

RVALVPMQAEEPIGIEPLLALVE

>Q0YQ10|Q0YQ10_9

MKFLEHPPRFLLFTGKGGVGKTSIACATAITLANSGLRVLLVSTDPASNVGQVFGITIGNRVTPVTAVTN

LYALEIDPQAAAAEYRERIIGPVRGLLPESVLHTIEEQLSGACTTEIAAFDEFTSLLTDPALSVDYDHIV

FDTAPTGHTIRMLQLPGAWSGFLEAAKGDASCLGPLAGLAKQRTQYKEAVNALADHLRTRMVLVSRPQQA

ALSEVARTCEELSAIGISQHYLVLNGILPEGEAAKDELARAIVMREQSALSAMPESLKALPTDQIALKPF

NLVGLDALSALFNEPQAQLPALSKEPAT-FTAPPLSRLVDEIAADGHGLIMFMGKGGVGKTTLAAALAVE

LAHRGLPVHLSTSDPAAHLIETLSGRQENLTVSRIDPEAETERYRQEVVDSKGKGLDSEGLALLEEDLRS

PCTEEIAVFKAFSRIIGDADRKFVVMDTAPTGHTLLLLDATGAYHREFTRQSGSEEQKITPMMQLQNRKQ

TKVLIVTLAETTPVLEAANLQEDLRRASIEPWAWIINNSVAAAVTHSTLLRERAENELVEIKSVAHHYAA

RYAVVPLLKEEPVGEQRLRALTC

>D9SJY5|D9SJY5_G

MKMLKNLPRFLFFTGKGGVGKTSLACATAVRLADGGRRVLLVSTDPASNVGQVFGVTIGNTITPISVVPN

LSALEIDPQAAAQAYRDRIVGPVRGVLPEAVVRGIEEQLSGACTTEIAAFDEFTALLTDTVLTDGFDHII

FDTAPTGHTIRLLQLPGAWTGFLDAGKGDASCLGPLAGLEKQRQRYSDAVAVLSDSRRTRLILVARAQRS

TLQEAARTCEELAHIGFADPYLVVNGCMPESE-TTDPVALAIFRRERNALDTMPEALRNLTTDRRSLLPF

NLVGVEALRALLSDSPVSPAALMHRDVE---LPNLAGLIDEFEKNGHGLIMLMGKGGVGKTTLAAAIAVE

LAARGHTVNLTTSDPAAHLADTLHGEMANLSVSRIDPRLETERYRQNVLASKGKELDAPGRALLEEDLRS

PCTEEIAVFQAFSRAIREANHAFVVMDTAPTGHTLLLLDATGAYHREVARNMKENSHFTTPMMQLQNQEH

TRILIVTLAETTPVLEAASLQQDLRRAGIEPWAWIVNQSLAAAHPASPLLALRAEHEWAQLEAVQQQHSK

RIAVVPVQAEEPVGIAALRALVK

>F4D7A3|F4D7A3_A

YHFLTNPPPFLFFTGKGGVGKTSLACASAVALCDIGKRVLLVSTDPASNVGQVFATEIGNRVTAIATVPG

LSALEIDPPAAAKAYRERIVGPVRGKLPESVVRSIEEQLSGACTTEIAAFDEFTALLTDHTLLAEYDHIV

FDTAPTGHTIRLLQLPGAWSEFLEQGKGDASCLGPLAGLEKQRSQYGEAVAALADAASTRLVLVARAQQS

TLREVARTHEELAAIGLWNQQLVINGLFPEGALAGDPLAMAIWQREQQSLAQMPQGIKGLAQDHIPLLAT

NLVGLDSLRQLLQPQDAGQVSL---ATSAHLQPELSSLVDELAADGHGLIMLMGKGGVGKTTLAAAVAVE

LASRGLPVHLTTSDPAAHLNETLAGSLPHLEVSRIDPHAETERYRAQVLATKGAALDEQGRALLEEDLRS

PCTEEIAVFQAFSRIIREAGKRFVVMDTAPTGHTLLLLDATGAYHREIARQMGKGLHFTTPMMQLQDPKQ

TKVLIVTLPEPTPVQEAANLQSDLRRAGIEPWGWLINHSLQQAAVSSPLLQLRAERQQPHIEAVEQQHAS

RFAVVPLLAEEPVGPDALHRLCQ

>A0KJ10|A0KJ10_A

YSFA---PPFLFFTGKGGVGKTSLACASAVALCDSGKRVLLVSTDPASNVGQVFDQ-IGNSITTI-----

LSALEIDPQAAAQAYRERIVGPVRG-LPESVIRSIEEQLSGACTTEIAAFDEFTALLG------EYDHIV

FDTAPTGHTIRLLQLPGAWSEFLAQGK-G-SCL--LAGLEKQRSQYAEAVAALSDAGKTRLILVARAQAS

TLREVARTHDELAAIGLANQQLVINGIFP--QEL-DPLATAVWQREQQAMADMP-A-LRLPQDSVPLLAT

NLVGVDSLRQLLGS----TVSGV------------EIECTIAESQQHGLIMLMGKGGVGKTTLAAAVAVE

LASRGLPVHLTTSDPAAHLGETLAGALPSLEVSRIDPHAETERYRAQVLATKGALDAQG-RALLEEDLRS

PCTEEIAVFQAFSRIIR-EAGRFVVMDTAPTGHTLLLLDATGAY-----IAQMKGLHFTTPMMQLQDPKQ

TKVMIITLPEPTPVQEATNLQADLRRAGIEPWAWVVNHMLAPTSVTSPLLAQRAYRQQPHIDAVKQEHAK

RVPLQAVELV---KALQ------

>A4SIL5|A4SIL5_A

YSFA---PPFLFFTGKGGVGKTSLACASAVALCDSGKRVLLVSTDPASNVGQVFDQ-IGNKITAI-----

LSALEIDPQAAAQAYRERIVGPVRG-LPDSVVRSIEEQLSGACTTEIAAFDEFTALLG------EYDHIV

FDTAPTGHTIRLLQLPGAWSEFLEQG-KG-SCL--LAGLEKQRSQYAEAVAALSDAGKTRLVLVARAQAS

TLREVARTHDELAAIGLANQQLVINGIFP--QEL-DPLASAVWQREQQAMAQMP-GLLRLPQDRVPLLAT

NLVGVDSLRQLLGAVNPAESQDQPGL---------KQLVDELAT--HGLIMLMGKGGVGKTTLAAAVAVE

LASRGLPVHLTTSDPAAHLGETLAGALPSLEVSRIDPHAETERYRAQVLATKG-------RALLEEDLRS

PCTEEIAVFQAFSRIIR-EAGRFVVMDTAPTGHTLLLLDATGAY-----IAQMKGMHFTTPMMQLQDPKQ

TKVMIVTLPEPTPVQETANLQADLRRAGIEPWAWVVNHMLAPASVTSPLLAQRAYRQQPHIDAVKGEHAR

RMPLCRYAVMPLCTCIRASLLPR

>Q5P147|Q5P147_A

MRFLDQPPRYLFFTGKGGVGKTSLACATAIHLAATGRSVLLVSTDPASNVAQVFEQEIGNRITPLTAVPG

LSALEIDPQAAAQAYRERIVGPVRDVLPEAVVRGIEEQLSGACTTEIAAFDEFTGLLTDAALTAGFDHVV

FDTAPTGHTIRLLQLPGAWSGFLQNNTDGASCLGPLAGLDKQREQYGAAVSALADPARTRLVLVARAQAS

TLREVARTHVELAAIGLARQFLVINGVLPAAEAADDALAAAVLRREAAALDELPAGLRDLPTDRVPLMPF

NLVGLPALRALLGSTEAAQASAPAAAPDDRPLPDLASLVDELATDRHGLVMLMGKGGVGKTTLAAAIAVE

LAQRGFPVHLTTSDPAAHLADTLDGSLANLTVSRIDPQAETERYRRDVLDTRGASLDAQGRALLEEELRS

PCTEEIAVFQAFSRVIGEAGRKFVIMDTAPTGHTLLLLDAAGAYHREVARHMDGVAQFTTPMMQLQDPAQ

TKVLVVTLAETTPVLEAASLQADLRRAGIEPWAWIINNSLAATTTAAPMLRRRAGQEWAQIEAVREHHAK

RLALVAMQADEPTGIGRLRALAG

>Q21S89|Q21S89_R

MKFLDQAPRFLFFTGKGGVGKTSIACASALELTRLNKRVLLVSTDPASNVGQVFGIRIGNQITNVAEVPN

LSALEIDPQAAAQAYRDRIVGPVRGVLPDAVVKGIEEQLSGACTTEIAAFDEFTALLTDGALTQNFDHII

FDTAPTGHTIRMLQLPGAWSGFLEDGKGDASCLGPLAGLEKQRTQYKAAVDALADPLKTRLILVARAQAA

TLNEAARTHGELAGIGLSKQYLVINGVFPESETVHDALAQAIFDREQAVLANLPDALRDLPTDQIGLKAF

NLVGLAPLRQLLAAAEPAVPTAADVPPGAPPAPSLASLVEAIAREGHGLVMLMGKGGVGKTTLAAAVAVE

LATRSLPVHLTTSDPAAHLMETLDGTLEHLTVSRIDPHEVTEHYRAQVLASKGAKLDAAGRAVLEEDLRS

PCTEEIAVFQAFSRVIREAGKKFVVMDTAPTGHTLLLLDATGAYHRDVARQMGSGRHFTTPMMQLQDPRQ

TKVLIVTLAETTPVLEAANLQTDLRRAGIEPWAWVINNSVAAAAVTSPLLQARAHNELREIAAVATHHAS

RYALVPLLKDEPIGMQRLQLLSK

>B7SKG0|B7SKG0_9

MKFLKTPPRFLFFTGKGGVGKTSIACAAAVQLAGDGKRVLLVSTDPASNVGQVFGLDIGNKVTEIRGVPR

LAAFEIDPQQAAQAYRDRIVGPVRGVLPEDIVKGIEEQLSGACTTEIAAFDEFTALLTDDSLIAEFDHII

FDTAPTGHTIRLLQLPGAWSGFLENSKGDATCLGPLAGLEKHQAQYKVAVEALSDPLRTRLVLVARAQNA

ALREVARTHEELSEIGLKQQYLVVNGVLPASAADGDELASAIYRREQQVLANFPEVLHALPLDQLSLKPF

NLVGLDTLGRLFADMNSDSGGITGGEPD-LKFPELSILVDNIAADGHGLVMLMGKGGVGKTTMAAAVAVD

LARRGLPVHLTTSDPAAHLAETLEGALDNLTVSRIDPQAETERYRQHVLKTKGAHLDPEGLALLEEDLRS

PCTEEIAVFQAFSRVIREAGKKFVVMDTAPTGHTLLLLDATGAYHREIVRQMGSKVSYVTPMMQLQDPKQ

TKVLLVTLAETTPVLEAANLQADLRRADIEPWAWIVNNSVAAARPVSAFLRKRAENERREIDAVAKEHAT

RYAVMPLLKDEPVGLNRLQALFR

>F5KK87|F5KK87_P

MQFLQSPPRFLFFTGKGGVGKTSIACAAAVQLAGENKRVLLVSTDPASNVGQVFSTEIGNKVTEIREVAR

LAALEIDPQQAAQAYRDKIVGPVKGVLPEDVVKGIEEQLSGACTTEIAAFDEFTSLLTDDALIAEFDHII

FDTAPTGHTIRLLQLPGAWSGFLESGKGDASCLGPLAGLEKHRAQYKAAVAALADPVRTRLILVARAQNA

ALREVARTHEELADIGLKQQYLVVNGVLPAAAAEGDELAAAIYQREQQVLAKIPEVLRSLPLDQVALKPF

NLVGLDALKHLFLDVAIGPVTETETNTE-LHLPKLSPLVDEIAAEGHGLIMLMGKGGVGKTTMAAAVAVD

LARRGLPVHLTTSDPAAHLAETLEGVMENLTVSRIDPQAETERYRQHVLKTKGASLDAEGRALLEEDLRS

PCTEEIAVFQAFSRVIREAGKKFVVMDTAPTGHTLLLLDATGAYHREIVRQMGTKVGYTTPMMQLQDPKQ

TKVLLVTLAETTPVLEAANLQDDLRRAGIEPWAWVINNSVAAAAPRSALLLRRAENERREINAVATKHSS

RYAVVPLLKDEPVGIDRLQSLVG

>B9Z4A6|B9Z4A6_9

MLFLDHLPPFLFFTGKGGVGKTSLACATAIRLADQGKRVLLVSTDPASNVGQVFGVTIGNVVTPIDTVPG

LNALEIDPQAAAQAYRDRIVDPVRGVLPETVVKGIEEQLSGACTTEIAAFDEFTALLTDTALQAGYDHIV

FDTAPTGHTIRLLQLPGAWSGFLEEGKGDASCLGPLAGLEKQRSQYQGAVEALSDPRRTRLILVARAQRS

TLREVARTHEELAAIGLTQHYLVVNGILPQSETSGDALAAAVWQREQVALAELPPVLRDLPTDHVSLMPF

NLVGLPALRRMLSSPEDCAGDVIEAIVL-PDAPDLATLVDGLAGDGHGLIMLMGKGGVGKTTLAAAVAVE

LARRGYPVHLTTSDPAAHLAETLEGSLANLSVSRIDPHTETERYRQQVLATKGKGLDEQGRAMLEEDLRS

PCTEEIAVFQAFSRVIREAGRKFVVMDTAPTGHTLLLLDATGAYHREVARQMGSGIHFTTPMMQLQDPAQ

TKVLIVTLAETTPVLEAANLQSDLRRAGIEPWAWIINNSLALANPTSPLLRRRAANELAQIDAVATRHAQ

RWAVVPLQAEEPVGVERLQGLTR

>G2J4E9|G2J4E9_9

----------MFFTGKGGVGKTSLACATAIRLADQGKRVLLVSTDPASNVGQVFGVTIGNVVTPIDAVPG

LNALEIDPQAAAQAYRDRIVGPVRGVLPETVVKGIEEQLSGACTTEIAAFDEFTALLTDTALQAGYDHIV

FDTAPTGHTIRLLQLPGAWSGFLEEGKGDASCLGPLAGLEKQRSQYQGAVAALSDARRTRLILVARAQRS

TLREVARTHEELAAIGLTQHYLVVNGILPQSETSGDALAAAVWQREQAALAELPPVLRDLPTDHVPLMPF

NLVGLPALRRMLSSSEDCAGDAVEAIVL-PDAPDLATLVDGLAGDGHGLIMLMGKGGVGKTTLAAAVAVE

LARRGYPVHLTTSDPAAHLAETLEGSLANLSVSRIDPHTETERYRQQVLATKGKGLDEQGRAMLEEDLRS

PCTEEIAVFQAFSRVIREAGRKFVVMDTAPTGHTLLLLDATGAYHREVARQMGSGIHFTTPMMQLQDPAQ

TKVLIVTLAETTPVLEAANLQADLRRAGIEPWAWIINNSLALANPTSPLLRRRAANELAQIDAVATRHTQ

RWAVVPLQAEEPVGVERLQGLTR

>G0A185|G0A185_M

MKFLDQAPRFLFFTGKGGVGKTSVACATAIRLAESGQRVLLVSTDPASNVGQVFGVSIGNQITPIPAVPG

LAALEIDPQAAAQAYRDRIVGPVRGLLPDTVIQGIEEQLSGACTTEIAAFDEFTALLTDPALTGDFEHIV

FDTAPTGHTIRLLQLPGAWSDFLDTGKGDASCLGPLAGLEKQRTQYKSAVDALSDGARTRLILVARAQHS

TLTEVARTHTELAAIGLRNQYLLINALLPAGETTEDALAQAIYMREQTALRQIPATLQELPRDDVFLKPF

NMVGLAALRQLLLDTPV-QPTAVAAVAD-LDTPKLAELVDTIAKDGHGLIMLMGKGGVGKTTLAAAVAVQ

LAQRGLPVHLTTSDPAAHLNETLNGALEQLTVSRIDPVVETEHYRRHVLETKGAKLDAQGRALLEEDLRS

PCTEEIAVFQAFSRIIREAGQKFVVMDTAPTGHTLLLLDATGAYHREVARQMDSGVHYLTPMMQLQDPKQ

TKVLLVTLAETTPVLEAANLQADLRRAGIEPWAWIINNSVAATPVQAPLLRQRAANELREIDTVARSHAQ

RYAVVPLLQEEPIGVKRLSQLAG

>D5QV13|D5QV13_M

MKFLERPPRFLFFTGKGGVGKTSIACATAIALAEAGRRVLLVSTDPASNVAQVFGTIIGNRMTDIPGAPG

LSALEIDPQAAAQAYRDRIVGPVRGLLPDAVVKGIEEQLSGACTTEIAAFDEFTALLVDSALTATYDHIV

FDTAPTGHTIRLLQLPGAWSGFLDSGKGDASCLGPLAGLDKQRAQYGRAVEALADAARTRLVLVARAQRS

TLTEVARTHKELAAIGIAQQYLIINGLLPEEEAKQDRLAAAIYEREQAALRALPAELAALPCDRVALRPF

NLVGLDALRQLLVATPSPANASEDEPIA-LDASSLGDLVDAIAADGHGLVMLMGKGGVGKTTLAAAVAVE

LARRGLPVHLTTSDPAAHLAETLHGSLEHLTVSRIDPHAETERYRREILAAKGAELDAAGRALLEEDLRS

PCTEEIAVFQAFSRIIREAGESFVVMDTAPTGHTLLLLDATGAYHREVARQLDKGAHYTTPMMQLQNPKQ

TKVLLATLAETTPVLEAAGLQADLRRAGIEPWAWIINNSVAAARPQSPLLRQRARNEIREIAAVAATHAR

RFAIVPLLKEEPVGVSRLLELAG

>E8L372|E8L372_9

MKFLEQPPRFLFFTGKGGVGKTSIACATAIQLAEAGRRVLLVSTDPASNVGQVFGVGIGDKITAIDAVPN

LFALEIDPQAAAQAYRDRIVGPVRGKLPDAVVKGIEEQLSGACTTEIAAFDEFTALLTDTAITSGYDHII

FDTAPTGHTIRLLQLPVAWSGFLEAGKGDASCLGPLAGLEKQRAQYSAAVDALADGQRTRLILVARAQNS

ALREAARTHEELAAIGLNQQFLVVNGLLPKEEAALDALAAALYAREQSALGATPDALRGLPCDYVPLKPF

NLVGLDALRQLLVETPPHLDAAVGEAVE-LHAPSLSELVDGVAADGHGLVMLMGKGGVGKTTLAAAVAVE

LAHRGLPVHLTTSDPAAHLAETLHGSLENLTVSRIDPHAETERYRQEVLRTKGAKLDAQGRALLEEDLRS

PCTEEIAVFQAFSRIIREAGEKFVVMDTAPTGHTLLLLDATGAYHREVARMLDKGAHYTTPMMQLQDERR

TKVLLVTLAETTPVLEAANLQADLRRAGIEPWAWVVNNSVAAARPHSPLLRQRAKNELREVEKVATTHAR

RYAVVPLLREEPVGVSRLLELAG

>E8L9V8|E8L9V8_9

MKFLEGAPRFLFFTGKGGVGKTSIACATAIQLSEAGRRVLLVSTDPASNVGQVFGVSIGNNITPISDVPR

LFALEIDPEAAAQAYRDRIVDPVRGKLPAPVVKSIEEQLSGACTTEIASFDEFTALLTDAEITTAYDHIV

FDTAPTGHTIRLLQLPVAWSGFLEAGKGDASCLGPLAGLEKQRAQYNAAVQALADGQRTRLVLVARPQNS

ALREAARTHGELAAIGLTHQFLIVNGLLPEEEAALDPLAKAIYGREQAALAAMPDELRALPRDDLPLKPF

SLVGLDALRQLLVETWSQPDAASGEPVD-LRAPSLSDLVDDIAADGHGLVMLMGKGGVGKTTLAAAVAVE

LARRGLPVHLTTSDPAAHLTETLHGSMEHLAVSRIDPHAETERYRQEVLRTKGGSLDSQGRALLEEDLRS

PCTEEIAVFQAFSRIIREAGEKFVVMDTAPTGHTLLLLDATGAYHREVARMLDKGAHYTTPMMQLQDEKR

TKVLLVTLAETTPVLEAVNLQADLRRAGIEPWAWVINNSVAAARPRSPLLRKRAQNELGEVKKVATVHAR

RYAAVPLLEEEPVGVERLLKLAA

>F8GDV2|F8GDV2_N

PKFLNQPPRFLFFTGKGGVGKTSLACATAITLADAGQRVLLVSTDPASNVGQVFGITIGNQVTAITAVPR

LAALEIDPQAAAQAYRDRIVGPVRGVLPDTVVKGIEEQLSGACTTEIAAFDEFTALLTDSALTADYDHII

FDTAPTGHTIRLLQLPGAWSDFLAEGKGDASCLGPLAGLEKQRTQYKAAVDALADSKRSRLILVARAQQA

TLREVARTHEELTVIGLTKQFLVINGLLPQAETSRDPLAAAIFQREQDALAAMPEVLNHLPCDRIALKSF

NLVGLTALRKLLIDAPP-DEGGKEFIHTAIPFPSLSSLIDEIAEDGCGLIMLMGKGGVGKTTLAAAIAVN

LAHRGLPVHLTTSDPAAHLSETLSGAMENLLVDRIDPHVETERYRQHVLETKGAHLDAKGRALLEEDLRS

PCTEEIAVFQAFSRIIREAGKKFVVMDTAPTGHTLLLLDATGAYHREVIKQMDSIVHYATPMMQLQNPKQ

TKILLVTLAETTPVLEAASLQADLRRAGIEPWAWIINNSVAATTVHSPLLCQRASNELAEIETVSSIHAQ

RYAVVPLLKEEPVGVIRLLELAG

>F9ZKC5|F9ZKC5_9

RKFLDQPPHFLFFTGKGGVGKTSLACATAITLADTGRQVLLVSTDPASNVGQVFGMTIGNQITTINAVPG

LAALEIDPQAAAQIYRDRIVNPVRGVLPDTVVKGIEEQLSGACTTEIAAFDEFTALLVDSALTADYDHII

FDTAPTGHTIRLLQLPGAWSDFLQEGKGDASCLGPLAGLEKQRAQYKAAVDALADPQRTRLVLVARAQQT

TLREVARTQEELTAIGLTNQFLVINGLLPPSEVTQDDLAAAIFEREQKTLAAIPEILKPLPRDHIALKPF

NLVGLPALRQLLMDELP-AVSGNNALHQAMPFPGLSRLIDDIAKDGSGLIMLMGKGGVGKTTLAAAIAVN

LAHRGLPVHLTTSDPAAHLNETISGTMDNLQVDRIDPHVETERYRQRILQTKGAHLDAKGKALLEEDLRS

PCTEEIAVFQAFSRIIREAGKKFVVMDTAPTGHTLLLLDATGAYHREISKQMDSIAHYTTPMMQLQKPKQ

TKMLLVTLAETTPVLEAASLQAELRRAGIEPWAWIINNSIAATTVQSPLLCQRASNELKDIEAVSTIHAQ

RYAVVPLLKEEQVGVQKLLELSR

>D6CMG7|D6CMG7_T

MKFLELPPRFFFFTGKGGVGKTSLSCATAMHLAGQGKQVLLVSTDPASNVGQVFGQAIGNTITRIHGVPG

LSALEIDPQQAAQQYRERIVGPVRGTLPDDIVNGIEEQLSGACTTEIAAFDEFTALLTDSVLTADYDHII

FDTAPTGHTIRLLQLPGAWSDFLEAGKGDISCLGPLAGLDKQRAQYKAAVQALADARRTRLVLVARAQQA

TLREVARTHAELAGVGLSQQYLVINGVLPPEEAAHDPLAAAICAHERAALDAIPEVLKALPRDRVALKPF

NLVGLDALRQLLVTTASQAPSTHAVPVA-LDAPSLSELVDDIAEDGRGLVMLMGKGGVGKTTLAAAIAVE

LAHRGLPVHLTTSDPAAHLTETLSGALDNLTVSRIDPHIETERYRQHVLATKGAQLDAQGRALLDEDLRS

PCTEEIAVFQAFSHIIREAGKKFVVMDTAPTGHTLLLLDATGAYHREIARQMATGLHYITPMMQLQDPRQ

TKVLIVTLAETTPVLEAANLQADLRRAGIEPWAWIINNSIAAATPNSPLLRQRACHELREIDAVATRHAR

RYAVVPLLKEEPVGVDRLLELTA

>Q47CR4|Q47CR4_D

MKFLEQPPRYLFFTGKGGVGKTSIACATAIQLAEAGQRVLLVSTDPASNVGQVFGVDIGNRIVGIEAVPG

LFALEIDPQAAAQAYRDRIVGPVRGVLPEAVVKGIEEQLSGACTTEIAAFDEFTALLTDSALVAGYDHII

FDTAPTGHTIRLLQLPGAWTGFLEEGKGDASCLGPLAGLEKQRTQYKSAVEALADPVRTRLILVARAQAA

TLREVSRTHEELAGIGLKQQYLVINGVLPESAVGSDLLAKAIYNREQAALLEIPAVLKALPIDQVGLKPF

NLVGLDALRKLLVASVFDAASDIPAATVLPEVPSLAVLVDGIAADGHGLVMLMGKGGVGKTTLAAAVAVE

LASRGLPVHLTTSDPAAHLSETLAGSLEHLTVSRIDPQAETERYRQHVLESKGATLDAQGRALLEEDLRS

PCTEEIAVFQAFSRVIREAGKKFVVMDTAPTGHTLLLLDATGAYHREIARQMSKDMHYTTPMMQLQDAKQ

TKVLIATLAETTPVLEAANLQSDLRRAGIEPWAWVINNSVAAVQSSSPLLRQRAVNELAQIDLVAHTHAV

RYAVVPLIQDEPVGVERLRALAN

>G3J256|G3J256_9

CKFLDHPPRFLFFTGKGGVGKTSIACATAIQLADSGYQVLLVSTDPASNVGQVFGITIGNHITPIKTVPG

LAALEIDPQAAAQAYRDRIVGPVRGVLPETVVKGIEEQLSGACTTEIAAFDEFTALLTDSTLTAGYDHII

FDTAPTGHTIRLLQLPGAWSGFLEEGKGDASCLGPLAGLEKQRSQYKSAVEALADPNRSRLILVARAQQA

TLQEVARTHEELNAIGLSQQYLVVNGILPEAEAMQDSLATAICQREQRALSEIPEALQALPRDQIELKPF

NLVGLDALRQLLTVATP-VITQFDATTQAIQVPNLSKLVDDIAADGHGLVMLMGKGGVGKTTLAAALAVQ

LAQRGLPVHLTTSDPAAHLSETLNGSLDNLTVSRIDPHAETERYRQQVLKTKGAQLDAQGRALLEEDLRS

PCTEEIAVFQAFSGIIREAGKKFVVMDTAPTGHTLLLLDATGAYHREVSRQM-SSMHYLTPMMRLQDPKQ

TKILLVTLAETTPVLEAANLQADLRRAGIEPWAWVINNSVAAVAVHSPLLRQRAANELREIDAVANRYAQ

RYAVVPLLQQEPVGVSRLLELTD

>Q2LMN5|Q2LMN5_9

MIFLQLPPRFLFFTGKGGVGKTSIACATAIQLAEAGKRVLLVSTDPASNVGQVFGVDIGNRVTPIPAVPR

LSALEIDPEAAASAYRERLVGPVRGVLPDDVVKGIEESLSGACTTEIAAFDEFTALLTNVALTADYEHII

FDTAPTGHTIRLLQLPGAWSGFLEAGKGDASCLGPLAGLEKQRTQYKAAVEALADPLQTRLVLVARAQQA

TLREVARTHEELAAIGLKQQHLVINGILPHIEAATDPLAAAIHEREQTALKNIPATLTALPCDHVELKPF

NLVGLDALRQLLTDLPPQAPVAVDSPIE-LDEPGVADLIDGIAADGHGLVMLMGKGGVGKTTLAAAIAVE

LAHRGLPVHLTTSDPAAHLTDTLDSSLDNLTVSRIDPHAETERYRQHVLETKGAQLDAEGRALLEEDLRS

PCTEEIAVFQAFSRIIREAGKKFVVMDTAPTGHTLLLLDATGAYHREVTRQMGTGMHFTTPMMQLQDPKQ

TKVLIVTLAETTPVLEAANLQADLRRAGIEPWAWIINTSVAAASAKSPLLRQRAANELREISAVANQHAD

RYAVVPLLKEEPIGADRLRALIH

>B9MAZ1|B9MAZ1_A

MKFLQLPSRFLFFTGKGGVGKTSIACATAIQLAEAGKRVLLVSTDPASNVGQVFGVDIGNRVTPIPAVPR

LSALEIDPEAAASAYRERLVGPVRGVLPDDVVKGIEESLSGACTTEIAAFDEFTALLTNTALTADYEHII

FDTAPTGHTIRLLQLPGAWSGFLEAGKGDASCLGPLAGLEKQRNQYKAAVEALADPLHTRLVLVARAQQA

TLREVARTHEELAAIGLKQQHLVINGILPHVEAATDPLAAAIHEREQTALKNIPTTLTALPRDHVELKPF

NLVGLEALRQLLTDLPPQAPAAVDSPIE-LDEPGMADLIDGIAADGHGLVMLMGKGGVGKTTLAAAIAVE

LAHRGLPVHLTTSDPAAHLTDTLEASLDNLTVSRIDPHAETERYRQHVLETQGAQLDAEGRALLEEDLRS

PCTEEIAVFQAFSRIIREAGKKFVVMDTAPTGHTLLLLDATGAYHREVSRQMGTGMHFTTPMMQLQDPKQ

TKVLVVTLAETTPVLEAANLQADLRRAGIEPWAWIINTSVAAASAKSPLLRQRAANELREISAVANQHAD

RYAVVPLLKEEPIGTERLRALIH

>Q1H178|Q1H178_M

MKFLQLPPRFLFFTGKGGVGKTSIACATAIQLAEAGKRVLLVSTDPASNVGQVFGVDIGNRVTPIPAVPR

LSALEIDPEAAASAYRERLVGPVRGVLPDDVVKGIEESLSGACTTEIAAFDEFTALLTNAALTADYEHII

FDTAPTGHTIRLLQLPGAWSGFLEAGKGDASCLGPLAGLEKQRTQYKAAVEALADPLQTRLVLVARAQQA

ALREVARTHEELAAIGLKQQHLVINGILPHVEAATDPLAAAIHEREQTALKNIPATLTALPRDHVELKPF

NLVGLEALRQLLTDLPPQAPAAVDSPIE-LDEPSVAELIDGIAADGHGLIMLMGKGGVGKTTLAAAIAVE

LAHRGLPVHLTTSDPAAHLTDTLDSSLDNLTVSRIDPHAETERYRQHVLETKGAQLDAEGRALLEEDLRS

PCTEEIAVFQAFSRIIREAGKKFVVMDTAPTGHTLLLLDATGAYHREVSRQMGTGVHFTTPMMQLQDPKQ

TKVLVVTLAETTPVLEAANLQADLRRAGIEPWAWIINTSVAAASAKSPLLRQRAANELREISAVANQHAD

RYAVVPLLKEEPIGTERLRALIH

>A4VG43|A4VG43_P

MHFLNQPPRYLFFTGKGGVGKTSIACATAVQLASEGKRVLLVSTDPASNVGQVFGESIGNHITAIPAVPN

LWALEIDPQAAAQAYRDRIVGPVRGVLPETVVNGIEEQLSGACTTEIAAFDEFTALLIDSTLTADYEHII

FDTAPTGHTIRLLQLPGAWSGFLEEGKGDASCLGPLAGLEKQRTQYKAAVDALADPQRTRLVLVARAQRA

TLREVARTHEELAAIGLSQQYLVVNGVLPAREAAHDELAAAIHQREQTALTAMPDVLKALPRDQIDLKPF

NLVGLDALRHLLVATTSGATSSIELPAQ-LDAPSLSSLVDEIAVDGHGLVMLMGKGGVGKTTLAAAVAVE

LAHRGLPVHLTTSDPAAHLSETLEGSLSSLTVSRIDPHEETERYRKHVLDTKGAQLDAEGRALLEEDLRS

PCTEEIAVFQAFSRIIREAGKKFVVMDTAPTGHTLLLLDATGAYHREVTRQMGTGMHYTTPMMQLQDPKQ

TKVLIATLAETTPVLEAANLQSDLRRAGIEPWAWIVNNSVAATRPDSLLLRQRAQNELREIDSVATHHAK

RFAVVPLLKHEPVGVDRLRDLAN

>B3G200|B3G200_P

MHFLTQPPRFLFFTGKGGVGKTSIACATAVQLATQGKRVLLVSTDPASNVGQVFGERIGNHITTIAAVPN

LWALEIDPQAAAQAYRDRIVGPVRGVLPDAVVDGIEESLSGACTTEIAAFDEFTALLTDAALTQDYAHII

FDTAPTGHTIRLLQLPGAWSGFLEAGKGDASCLGPLAGLEKQRSQYKAAVEALADPLRTRLVLVARAQRP

TLREAARTHEELAAIGLSQQHLVINGVFPASEAETDTLAAAIYEKEQATLAAIPSVLQALPRDQIALKPF

NLVGLDALRHLLVDTDATGADAIELPDQ-INAPDLSALVDEVAADGHGLVMVMGKGGVGKTTLAAAIAVE

LAGRGLPVHLTTSDPAAHLADTLEGSLPNLALSRIDPQEATARYRQHVMDTKGVQLDAEGRALLEEDLRS

PCTEEIAVFQAFSRAIREGGRKFVVMDTAPTGHTLLLLDATGAYHRDIARQMGTGVHFTTPMMQLQDPKQ

TKVLIVTLAETTPVLEAANLQDDLRRAGIEPWAWIVNNSVAAAHPHSPLLRQRARNELREIDAVATQHAR

RHAVVPLLTEEPVGVERLRALAN

>C6BDL6|C6BDL6_R

MHFLTQPPRFLFFTGKGGVGKTSIACATAVQLAAQDKRVLLVSTDPASNVGQVFDERIGNRITAIAAVPS

LWALEIDPQTAAQAYRDRIVGPVRGVLPDAVVDGIEESLSGACTTEIAAFDEFTALLTDTALTQDYEHVI

FDTAPTGHTIRLLQLPGAWSGFLEAGKGDASCLGPLAGLEKQRSQYKAAVEALADPLRTRLVLVARAQRP

TLREAARTHEELAAIGLSQQHLVINGVFPAGGAENDTLAAAIYEREQATLAAIPQALQALPRDQIALKPF

NLVGLDALRHLLVDTDATSVDAIELPDQ-INASDLSVLVDEIAADGHGLVMVMGKGGVGKTTLAAAIAVE

LAGRGLPVHLTTSDPAAHLADTLEGSLPNLMLSRIDPQEATARYRQHVMDTKGAQLDAEGRALLEEDLRS

PCTEEIAVFQAFSRAIREGSRKFVVMDTAPTGHTLLLLDATGAYHRDIARQMGTGVHFTTPMMQLQNPKQ

TKVLIVTLAETTPVLEAANLQTDLRRAGIEPWAWVVNNSVAAAHPHSPLLRLRARNELREIDAVAMRHAQ

RYAVVPLLTEEPVGVERLQALAR

>B5JE64|B5JE64_9

PDFLAKATRFLFFTGKGGVGKTTLACSTALALAERGSSVLLVSTDPASNLDEVLGTQLATTPTPIEKAPG

LHALNIDPEAAAQQYRERIVDPIRGILPDATVANIEEQLSGACTTEIASFNEFSRLLGDSDSVSAYQYVI

LDTAPTGHTLRLLALPAAWSDFIADNKTGSTCLGPLAGLADQRVVYEAALSVLRDPNQTTLTLVARADTP

SLQEADRARAELAELGVLNQVLILNALFRATD-KTDPLASSMQARAEEALERSPTGLEKLPTYQSPFRIN

GLSGIEKLRNLY------SNNTEPQDTPKSLYPELDTIVDQLSANGRGVIMTMGKGGVGKTSLAKQIAIE

LAARGHKTLLTTTDPANHVTDLSPELDSLLSVSAIDPKEVTRQHVETVLATSGQDLDSSARELLEEELRS

PCTEEIAVFTAFAHEVAKGEEQFVVLDTAPTGHTLLLLDATEAYHREVLKNSAEAVQQLLP--RLRDPEF

TRTLIVALPEATPVHEAAQLQSDLRRAEIEPYAWVVNQCINTSDTQDPQLLAKAAFEETYIQEVATQHAK

RWAINYWTHIPSTASVK------

>D6Z0Q7|D6Z0Q7_D

MLLIDQAPRYLFFTGKGGVGKTSISCMVATALARRGKKVLLISTDPASNLDEVLETELSGAPTPVVGCPG

LLAMNIDPEEAAAAYRERMVAPYRGVLPDEAVQKIEEQLSGACTVEIAAFNEFSQVIGQPTTVADYHHVV

LDTAPTGHTLRLLSLPAAWNDFVLENKSGSSCLGPLAGLKKQRLIYEGAVASLTDPALTLLVLVSRPELF

ALEEAARAATELAGQGMKNQHLFINGLFQAA--SDDPVAKALEAKAEQALANLPPILARLPRSSMAFRPH

GLVGMGAIDRALATPDQAQGEIRATDLQPISTPW-PEIMAQLAAPGKGLIMTMGKGGVGKTATAAAIAVE

LARRGHRVCLSTTDPAAHVAAMLPEAPTNLTVSRIDPKAETRAYTAQVLADREAELSPEDLELLREELRS

PCIEEIAVFQAFAREVASAREQFLVLDTAPTGHTLLLLDATESYHREVAKNTGEAVKELLP--RLRDPQY

TKLLLVTLPEATPVHEAASLQEDLRRAGIEPWGWAINQCFSLSGTRDPQLAARGVMELGFIKEVLQLCSG

PVAASPWLATELKGDEPLRRLLS

>Q1NPV7|Q1NPV7_9

NNLLAKAPRYLFFTGKGGVGKTTISCITAAALAQQGKKVLLISTDPASNLDEVLETRLSGVPAPIEGIPG

LLAMNIDPEEAAATYKERMVGPYRGVLPDETVKSIEEQLSGACTVEIAAFNEFSQVIGHPETVAEYDHIV

LDTAPTGHTLRLLSLPAAWNDFVLENKGGSSCLGPLAGLKEQRLIYEGAVASLTNPELTLLVLVTRPEPF

TLEEAQRAALELDELGMKNQHLVINGRFQAT--SADPVARALESKGEEALRALPPTLAGLPRSELNFRPH

GLVGRQAIATALEEAEPVSGEVEAQSLEQTATPW-PELLAQLAAPGKGLIMTMGKGGVGKTATAVAIAVE

LADRGHQVRLSTTDPAAHVAQMLPDPPARLTVSRIDPKAETQAYVAGVLAAREKELSADDLELLKEELRS

PCIEEIAVFQAFAREVADAKDQFLVLDTAPTGHTLLLLDATESYHKEVEKNAGEAVKELLP--RLRDPQY

TRILLVTLPEATPVHEAKRLQEDLQRAAINPYGWAINQCFALSGTKDPLLAARGLKELDYIAEVLGVSPG

PVAASPWLATEIKGSEQLRQLLV

>A5GA11|A5GA11_G

QDFLRSQPKNLFFTGKGGVGKTTTSAATAIALADCGKLVLLVSTDPASNLDEVLGLTLSAEPSQVPDVPG

LFALNVDPEKAAAAYREKLVGPYRGILPHSALRSMDEQLSGACTVEIAAFNEFATLMGNPAAVEGFDHII

FDTAPTGHTLRLLSLPAAWSDFIDTNVRGTSCLGPLAGLKVQHLLYANTRKVLGDAKQTLVVMVSRPDEA

PLKEAARASAELQELGVANQRLILNGVFRMTD-SSDPVAVTLREINMSALAAMPDALALLPRTEIPLQGA

ELVGIPALRSFF-SIEPKAASCPDLTDP---PPPLTDLIDKFANQPGGVIMTMGKGGVGKTTLAVTIATE

LARQGKQVHLTTTDPAAHVALTIGEAPSGMRVSRVDPEVETEAYRHEVLTTVGASLDDEARALLEEDLRS

PCTEEIAVFRAFAKVVADGTDSFVVIDTAPTGHTLLLLDAAETYHREVSRSNEEAVRQLLP--RLRDPEF

TKIFLVTLPEATPVHEALSLQSDLRRAGIEAAGWIINQSLAPLPLTEPVLAQRRAREHRYIEEVVRQGIS

TY-LIPWQAQPTATLRSNKLRT-

>D5SNA9|D5SNA9_P

APLVEQPPRHLFFTGQGGVGKTSIASAVAIALAESGRRVLIVSTDPASNLDEVFETQLGNRPTPVHNIPG

LWGLNLDPHQAAAEYREKMVGPYRGVLPESAIASIEEQFSGACTVEIAAFDQFAQLLGDERTTKDFDHVI

FDAAPTGHTLRLLALPSSWASYLDANTTGTTCIGPLAGLAAQQKLYHATAHALCDGELTTLVLVTRPESS

AIREAARTSHELQALGVEQQWLVINGLFEATS-T-DPLAMAMEQRMQAALAALPDELKKLPSITVPFQPG

GLIGKAALQHLG-HSVETTNSQAAQGTPTSEPPWKQELTSELAQREHGVIMTMGKGGVGKTTIAAAIAIR

LAQLGRNVLLTTTDPAGHIVGLVTGELPLLSVERIDAHAETERYREEVRQTAGAQLDDAGRALLEEDLRS

PCTEEIAVFRAFARTVARGKQQIVVLDTAPTGHTILLLDAAMAFHQEAQRLNQEEVAQLLP--VLRDHQQ

TRILLITLPEATPVHEARLLQADLQRAGMEPMGWVVNQSLSLLDMYDPGLVTRKSFERKYIVEVAQAHPS

MMTIVPWQATIPTGAAGLLSMTE

>B2T6S3|B2T6S3_B

MTLPLVTTRHLFFTGKGGVGKTSLACATALQLAEKGKTVLLVSTDPASNLDEVLETQLSGQPTPVAQVPN

LHALNIDPELAAAAYRERTVSPYRGVLPDAAIRSMEEQFSGGCTVEIAAFDAFADLLGGSATANAYDHII

FDTAPTGHTLRLLTLPSAWSNFLSTNTTGNSCLGPLAGLEQNRQLYAAAVAELTNHDRTTVVLVTRPEAS

AFREAERTRIELADLGVRNLVLAVNGVFKAVS-SDDAIARAMEEQQAVSIKTMPPGLAALPRSETGFIPR

GLVGLTALKAYL-HPEQ-IAAPRVRLSV---PGGLLPLVDDLEKAGHGLVMTMGKGGVGKTTVAAAIALD

LAQRGHAVLLSTTDPAAHVAWTLQESLPGLTVSRIDAEQEVNRYRDEVLAKAGAHLDAQGKAMLEEDLRS

PCTEEIAVFRAFARTVDEARDSFVILDTAPTGHTILLMDSAEAYHREVTRTGDEAVRQLLP--RLRDPDF

TRVLIVTLAEATPVHEAERLQADLRRAQIEPYAWVIDQSLLASGTHDPALAERGRYEVPFIERVMKQDAK

RAVLLPWQAHAPVGLHGLEELAR

>D5NRC7|D5NRC7_9

MTLPVANTRHLFFTGKGGVGKTSLACATALKLAEDGKTVLLVSTDPASNLDEVLETPLSGQPTPVSQVPY

LHALNIDPERAASAYRERMVAPYRGVLPDAAIRSMEEQFSGGCTVEIAAFDAFANLLAGNVITKAYDHVI

FDTAPTGHTLRLLTLPSAWGSFLSTNTTGNSCLGPLAGLEPNKQLYAAAVAELTDRDHTTVVLVARPDTS

AFREAERTRIELADLGVRNLVLAVNGVFKATS-PDDVIAHAMEAQQAAAIDAMPRGLAALERSQTGFIPM

GLVGLRALKAYL-HPEQ-IGASQASLSV---PGGLLPLIDDLEKARHGLIMTMGKGGVGKTTVASAIALE

LAQRGHNVLLSTTDPAAHVAWTLQESLPGLSVAHIDAALEVDRYRDEVLARAGAYLDAQAKAMLEEDLRS

PCTEEIAVFRAFARTVDEARDSFVILDTAPTGHTILLMDSAEAYHREVMRTGDEAVRQLLP--RLRDPEF

TRVLIVTLAEATPVHEAERLQADLRRAQIEPYAWVIDQSLLASGTHDPALAERGRYEAPFIERVIKQDAK

RSVLLPWQATPPVGLHGLEELSR

>G2T710|G2T710_R

MALPETPTRFIFFTGKGGVGKTSLSCASGLALAEAGKTVLIVSTDPASNLDEVLGTPLGDQPTAIAGAPG

LYGLNIDPEAAAFAYRERMVAPYRGLLPAAAIASMEEQFSGACTVEIAAFDAFAKLLGDDAATAAFDHVI

FDTAPTGHTLRLLTLPSAWGDFIASATGGASCLGPLAGLETQKALYGATVAHLADPKMTTVVLVSRAEGA

ALREAERTRGELADLGVTNQRLALNGVFTAPR-GEDAIADAMTLRGLEALADMPAALAALPRSQTPFLPL

GTVGLTALRQIA--ADT-AAEATGPAPAAAPPGGLEDLIDEIAAAGHGVVMTMGKGGVGKTTIAAALAIA

LARRGHRVTLSTTDPAAHVAQAVDGTVPGLTIARIDPKVEIAGYRAEVLEKAGKNLDAAGRAMLEEDLRS

PCTEEIAVFQAFARTVDGGKDHFVVLDTAPTGHTILLLDAAEAYHREVLRTADEAVRALLP--RLRDPGF

TKAIIVTLAEATPVHEAERLQRDLARAGITPFAWVITQSLLASGTTDRLLRQRGAYEAPFIARVATELAK

RTALIPWAA--------------

>Q2W688|Q2W688_M

MSLPQVETRILFFTGKGGVGKTSLSCATGLALAEAGRRVLIVSTDPASNLDEVLGATLSQVPTAIPGAPG

LFALNIDPEAAARDYRERMVGPYRGILPAAAIASMEEQFSGACTVEIAAFDEFAKLLGDPAATAEFDHVI

FDTAPTGHTLRLLTLPSAWTEFIASSTGGASCLGPLAGLEKQKALYAATVAQLADPKATTLVLVSRPERS

ALREAERTRGELAELGVSNLRLALNGVFTAAS-PGDAIADAMTERGRDALATMPAGLASLPRSDTPFLPR

GTVGLDALRAMG----Q-AGAAHGFEPMPR-PGGLEALVAEIAASGHGVVMTMGKGGVGKTSVAAAIATA

LARLGHKVTLSTTDPAAHVQDAVEGKVAGLTVTRIDPEREVADYRDEVLAKAGGTLDMAGRAMLEEDLRS

PCTEEIAVFRAFSRTVDEGRDRFVILDTAPTGHTILLLDAAEAYHREVLRTAEEAVRSLLP--RLRDRDF

TKTIIVTLAEATPVHEAERLQDDLARAGITPFAWVINQSLLASGTRDPLLTQRGTYEVPFIERVAANPSS

RTALIPWTA--------------

>A1K818|A1K818_A

MTLPRASTRYLFFTGKGGVGKTSLSCATGLALAEAGRRVLIVSTDPASNLDEVLGTELGQTPTAIAGAPG

LYALNIDPEAAAAAYRERMVGPYRGILPAAAIQSMEEQFSGACTVEIAAFDEFSKLLGEPAATAGFDHVI

FDTAPTGHTLRLLTLPSAWSEFISSSTGGASCLGPLAGLQQQKALYAATVERLADPQATTVILVSRAETA

ALREAERTRGELAELGIRNQVLAINGLFADTD-TDDAIAAAMSQRGAKALAAMPAALRELPATTIPFLPA

GTVGLDALRVMA-QPER-MAAPTEPAALPQ---GLGAMVDDIARSGHGVVMTMGKGGVGKTTAAAAIALA

LAQRGHKVTLSTTDPAAHLSTTLAEAVPGLSLARIDPAREVADYSAEVMARAGQGLDAAARAMLEEDLRS

PCTEEIAVFRAFARTVDQGKDGFVVLDTAPTGHTILLLDAAEAYHREVSRTGEEAVRQLLP--RLRDPAF

THVLIVTLPEATPVHEAERLQADLARAGITPYGWVVNQSLLASGTHHPLLAQRAHHELPFIRRVSQDLAA

RFALLPWLAEAPVGVEGLRQVL-

>A4JMY5|A4JMY5_B

MSLPDTQTRYLFFTGKGGVGKTSLSCATGLAMADAGKKVLIVSTDPASNLDEVLGVGLSQSPTAVPGAPD

LFALNIDPEAAAQAYRERMVGPYRGVLPAAAIRNMEEQFSGACTVEIAAFDEFSKLLGDPAATADFDHVI

FDTAPTGHTLRLLTLPSAWNEFISSSTGGASCLGPLAGLEKQKALYAATVERLSSAVDTTVVLVSRPEVA

ALREANRTRHELAELGIRNQVLAINGLFATER-HDDAIATAMAERARQALADMPRELSVLPQTRIPFLPR

GTVGLDALRDMA-HPER-VRMPTR---MPD-PPGLGGLVDALSATGHGVIMTMGKGGVGKTTVAAAIAVA

LAGRGHDVILSTTDPASHVAATVDGVVPRLSVTRIDPAREVQQYTEEVLAKAGSHLDAGGRAMLEEDLRS

PCTEEIAVFRAFARTVDQGKSGFVVLDTAPTGHTILLLDAAEAYHREVMRTGDESVRQLLP--RLRDPDY

SRILIVTLPEATPVHEAERLSADLARAGITPYAWVINQSLLASGTTDPMLCQRGTYEVPFVRRVADNLAQ

RTALIPWLAEAPVGPVGLEHVIR

>A9AS12|A9AS12_B

MSLPDTQTRYLFFTGKGGVGKTSLSCATGLAMADAGKKVLIVSTDPASNLDEVLGVGLSQLPTAVPGAPG

LFALNIDPEAAAHAYRERMVGPYRGILPTAAIRNMEEQFSGACTVEIAAFDEFSKLLGDPAVTADFDHVI

FDTAPTGHTLRLLTLPSAWNEFISSSTGGASCLGPLAGLEKQKALYAATVERLSSATETTVVLVSRPEVA

ALREANRTRHELAELGVRNQMLAINGLFATDR-HDDAIATAMAERAQQALADMPRELAGLPQTRIPFLPR

GTVGLDALRDMA-QPER-VRMPSR---MPE-PPGLGGLVDELSIVGHGVIMTMGKGGVGKTTVAAAIAVA

LAQRGHDVILSTTDPAAHVAATIDGVVPGLTVTRIDPAHEVRQYTEEVLAKAGTALDAGGRAMLEEDLRS

PCTEEIAVFRAFARAVDQGKSGFVVLDTAPTGHTILLLDAAEAYHREVMRTGDESVRQLLP--RLRDPDY

TRILIVTLPEATPVHEAERLRADLARAGIEPYAWVINQSLLASGTTDPLLCQRGTYEVPFVRRVADDLAP

RTALIPWLAEAPVGEAGLEQVIT

>F0SJU4|F0SJU4_P

DAWLSNPTRYYFFTGKGGVGKTTLSTAFAVQLADAGQRVLIVSTDPASNLDAVLGCSLSSVPTAVPEVAN

LSAANLDPEQAAAAYRERLIGPYRNVLPESAVTAMEEQFSGSCTIEIAAFNEFAQLLGDRSATADFDVVL

FDTAPTGHTLRLLSLPSAWSGFLDDNTTGTSCLGPLAGLQQQRELYEQTLQTLKDPLLTSVVLVTRPEEA

ALREAARSSHELKELGVENQQLIINGTFTATD-ANDPYAVAWQAQSEAALADMPAALNDLPQRRVPLASR

NLMGPALLRSIR-WNGE-PAELPSFSTPAS-PQSLSTLVDRLAEPGHGVIMTMGKGGVGKTTLAAAIAVR

LAEAGYPVELSTTDPAAHLADALADSLSNLKITRIDPKAEVAAYTAEVLATSGKNLDAAGRALLEEDLRS

PCTEEIAVFRAFAKAVASAEERYVVLDTAPTGHTILLLDSALSYHRDVKRLQQASVESLLK--RLRDSEY

TRLLIATLPETTPIQEARQLQADLQRADIQPFAWIVNQSLTPLSLTDPFLKLRQQAEAERLTEVAEASGG

IFSLVPWQIAAPVGVEKLLQLSE

>Q0A537|Q0A537_A

MQFLENATRNLFFTGKGGVGKTSLACATALALAERGKRVLLVSTDPASNIDEVLETDLTGTPRPVNGVDN

LHALNIDPEKAAEEYRERVVGPYRGQLPDAIVRSMEEQLSGACTVEIAAFDAFAGLLGDPRAAEGYDHLV

FDTAPTGHTLRLLSLPSAWSGYIETNTSGTSCLGPLEGLSAQKDVYAGAVEALAEADRTTLVLVSRPEGA

ALDEAARTSEELRDLGVKNQHLVVNGVFRATD-ADDPVARALEARGQRALEAMPAGLAELPRSERPLRAH

APMGLDGLRILL-GEQA-PDIPEAEDAPEG-----EQLIDSLERDGRGAVMTLGKGGVGKTTLAARIAVA

LASRGHSVHLTTTDPAAHVAAAVGELPTGLTVGRVDPKAETERYREHVMATAGADMDEEGRKLLEEDLRS

PCTEEIAVFQAFARTVARAEDEIVVLDTAPTGHTILLLDAAQAYHRELGRQQEPEVEQLLP--RLRDPHY

THMLICTLPEATPVHEAAALQADLRRAEIEPAAWIVNQSLTPLAVTDPVLRARQAQEARWLREIVSEHHS

RLIIEPWSEDYS-----------

>D2R960|D2R960_P

MKFLLHPTRNLFFTGKGGVGKTSVACAAAVRLADAGKRVLLVSTDPASNLDEVLGVALENRPTAIPAVPT

LYAMNLDPEKSAAAYRERMIGPYRGLLPDAAVKSMEEQFSGSCTLEIAAFDEFSRLLGDPAATSQFDHVI

FDTAPTGHTLRLLTLPSAWAGFMEENTTGTSCLGPLAGLQAQQKLYQETVKALGDPQVTTLVLVARAEVS

ALREAARTSGELAELGVENQHLVVNGVFEALD-TSDPYAVALEQRGAVALAATPESLRALPTTIVPLSPS

GIVGVESLRLLG--TPP-SYSPETLDSSDR-LPLLGELIDDLVAPGHGVILAMGKGGVGKTTVAAAVAVA

IAERGYEVHLSTTDPAAHIAAALNQQLANLTVSRIDPAAETAKYSAEVLSSAGANLDQQGRALLEEDLRS

PCTEEIAVFRAFADAVAAGTNKFVVLDTAPTGHTVLLLDSALAYHREVTRQSAEAVENLLP--RLRDPGF

TRVLIVTLAEATPVHEAAALQRDLRRAEIEPFAWVINQVLSPLPLTDPLMKQRQQHEQKYLREVKEVQAS

RFAIIPWQIEPPIGIQQLRKLTH

>F2AKA1|F2AKA1_R

MHYLESPTRNLFFTGKGGVGKTSMACATAVRLADRGRRVLLVSTDPASNLDEVLGVTLSNKPTSVPAVDN

LSAMNIDPEQAAAEYRERMVGPYRDVLPEAAVQSMEEQFSGSCTVEIAAFDEFARLLGDENATADFDHIV

FDTAPTGHTLRLLTLPSAWSGFMDDNTSGTSCLGPLAGLQKQQAIYHSTVESLGDSSRTTLVLVTRPEAS

TLREADRTSDELRELGVENQALVINGTFEAQD-KSDPIANAMEQRCKEALMAMPSGLAQLQRTVVPLAPH

GLIGIDALRLLG-RTEP-NGEMAEIAGEER-LEALSALIDQLASQQHGVIMTMGKGGVGKTTIAAAVAVA

LAERGLKVHLSTTDPAAHVSATLAEELSGLTISRIDPAEVTEAYRQEVLRTAGDGLDEQGRALLEEDLRS

PCTEEIAVFRAFADAVAEGEDGFVVLDTAPTGHTILLLDSALAYHREVSRQNEESVRELLP--RLRDSDF

TRVLVVTLPESTPVHEAQRLQQDLRRAEIEPFAWVVNQSLSPLVVKDRILVGRQQNEEPYLREVFTQHAK

QAVIIPWQLDPPIGIEGLRELTA

>Q2JLU4|Q2JLU4_S

RVNHSHPQRLLLFSGKGGVGKTTLTCALARQLAD-PRRLLLMSTDPAHSLGDVLQISV-AQPLPD-----

LQVRALQAEILLQSFRQELRGSWFG--REDLLPIWDLAW--PGVDELMAILEVNRLLAG-----EVDTVI

LDTAPTGHTLRLLELPDNLLAVFAKH-RE------PDEADAFLAQLQELEGRLTNPESTSAWLVMIPEQL

SVAETRRFCQQLQNRRVPIGGLLVNQVLL--RNNSSPLYSARQQEQGRVLKALQ-E-EGYSIWVCPQLQ-

EPVGLAALDELVQL--------------RPPIPEQKGIPSLPDFQGIRLVLVGGKGGVGKTTVAGALAWN

LAKRHPDKLLLVSIDPAHSLGDLIPLLPNLLGQEIDAAAVLEQFRQDYLEEVAALAGEGTVQYDPQAWRQ

LLQMPPPGLDEVMALLSTSGQDLVVLDTAPTGHLLRFLQMPQALYRDVVWARMLLAQVRQLRQQLQDPQF

VTFIPVFNPEQAVLAETERLLAELDLGIPHPYAVLNRVWLEDSTPFGEALRRRHQTLLAQLPQLFSQQAI

LFLHPPSLENIGS----------

>Q2JQN8|Q2JQN8_S

RLSPC--QRLLLFSGKGGVGKTTLTCALARHLAQ-PRRLLLLSTDPAHSLGDVLGIPVADVPQPL-----

LQVRALQAEVLLQDFKQELRGSWLA--KEDLLPLWDLDW--PGVDELMAILEVNRLLAG-----EVDTVI

LDTAPTGHTLRLLELPDNLLAVFATFQHR-EVV--PDEADAFLARLQELEGRLTDPESTAAWLVLIPEPL

SVAETRRFCQQLQSRRVPIGGLLVNQVLL---GAASPLYLARQQEQRRWLKALQ-E-EGYPIWVCPQLE-

EPIGPAALDKLVQL-----------------LPEVSPPRVALPDAQIRLLLVGGKGGVGKTTVAGALAWN

LAQRHPDRLLLVSIDPAHFGVKLNLLGQEIEAAAVLERFRQEYLEEVAAILAGETAGVE-VQYDPQAWRQ

LLQMAPPGLDEVMALLTASGQNLVVVDTAPTGHLLRFLQMPQALYRDVVWARLLLAQVRQLRQRLRDPQF

ASFIPVFNPEQAVLAETERLLAELDLGIPHPYAVLNRVWPEDSTPFGAALRRRHQAILAQLPQRFPQQAI

VFLHPPSLEAIGA----------

>B5W7R2|B5W7R2_S

VEDLS--LAL--FSGKGGVGKTTLACGLALHWASQDDRILLLSTDPAHSLGDVLQQ-VENTPHHV-----

LSIRALDAHILLKDFKERYGDTLQS-V--DLTPVWDLEW--PGVDELMGLLEIQRLFD------EIDRVV

VDMAPSGHALNLLGLMDNLLESLEKHRGG--------FLDEMRDKLASGRARLQDADHTSCFCVAIPEPM

SLFETRRFLESLQTLKIHLGGVWINHIVT------EAVDGDRYREQQPLMAEFI-K-IDQPIFLVPLDT-

EPLGVTALNSLFAT-----------------DDQLPKLPDFLSL--RRLVIVGGKGGVGKTTVAAAIGWA

MAERYSARVRMVSIDPAHSLGDALNITDNLSGQEIDADQVLDRFRSDYLWELAEMMGGDSLAYGPEAWRR

IVSQSLPGLDEMLSLVEESHEDLIVLDTAPTGHLLRFLEIPTAMYQNVLFMRLLRQRVVKAQKRLADPEY

TEFIGVVQNREAILAEAERLVESVREQSIAQNYIVHNRAELGKAISGDRFGELAIVPLPNLPRCVEPIFR

IAELIF-----------------

>D4ZWD5|D4ZWD5_S

-MVHK--LSLALFSGKGGVGKTTLACGLALHWASQDDRILLLSTDPAHSLGDVLQQ-VDNTPNHV-----

LSIRALDA-HLLKDFKERYGDTLQS-VGEDLTPVWDLEW--PGVDELMGLLEIQRLFD------EIDRVV

VDMAPSGHALNLLGL-MDFLDNLFQEKGG------DEFLDEMRDKLASGRARLQDADHTSCFCVAIPEPM

SLFETRRFLESLQTLKIHLGGIWINHIVA------ETVDGDRYREQQPLMAEFI-K-IDQPIFLVPLDT-

EPLGVTALNSLFAT---------KPLGNDDL----TQTENQPVSTFRRLVIVGGKGGVGKTTVAAAIGWA

MAERYSARVRMVSIDPAHFGCQLLNITDNLSGQEIDADQVLDRFRSDYLWELAEMMGGDSLAYGPEAWRR

IVSQSLPGLDEMLSLVEESHEDLIVLDTAPTGHLLRFLEIPTAMYQNVLFMRLLRQRVVKAQKRLADPEY

TEFIGVVQNRSAILAEAERLVESVRQSIAQNYIVHNRAELGKAISGDRFGELAIVPLPNLPRCVEPIFRI

QELIF------------------

>B2J651|B2J651_N

-------DNLHLFSGKGGVGKTTISCTFACRWAQKNEQILLISTDPAHSLGDVLQS-VDDIPRPI-----

LLVRALDAKRLLQDFKEELRGSFVE--GEDLSPVWDLNW--PGLDELMGLLEIQRLLNE-----QVDRVV

VDMAPSGHTLNLFGLMDTFLHSLELFQEK-HRI--PDRADDFLQTLKELSQLLQDPTHTACLLVAIAEPM

SWLESKRFLEALQTMQIPCGGLFVNQVLA------SATDPDRYQEQQPLIGQYT-A-LEKPMFIVPQQDE

EPLGILALSHLIDI---------------------LSFIDLLPVPERRLLLIGGKGGVGKTTVAAAIGWA

MAQQHPDRIRMVSIDPAHSLGDAYQITANLRGQEVDGDRILDQFRADYLWELAQ-MMSGEMAYAPVAWRK

IVDQALPGIDEMLSLLTEQQEDLIILDTAPTGHLLRFLEMPTALYQNVLFMRLLRQRVVKAQKVLKDPQQ

TEFIGVTLNQASVLAEQQRLFKSMQEIGVSQNYVVLNRFTSTATINCDFPGLTMVRLPMLPRSVQPLERI

QGCLF------------------

>Q112M5|Q112M5_T

-------LNLVMFSGKGGVGKTTNSCAFAGHWAKKNEKVLLISTDPAHSLGDVLQS-VTDTPRPL-----

LLVRALDVNLLLEEFKKEVRGSFVE--GGDLTPVWDLDW--PGLDELMALLEIQRLCN------EVDRVV

VDMAPSGHTLNLFKLMDTFLNSLELFQEK-HKY--PNEVDEVLQNLKELAALLQNSSNTACLVVALPEPM

SFRETQRFLSSLEEIKIPYAGIVVNQIVV------DKDGNDRYHEQQKLVNDFI-K-LDKPVFLVPEKA-

EPLGVTALEKLTNI--------------------NKVPPGFTDFKGKRLLIVGGKGGVGKTTIAAAISWE

MAKRYPERVRAVSIDPAHSLGDASIISSNLKGQEIEANKVLEKFREDYLWELAEMMSGEKMAFAPKGWRQ

IVEQALPGIDEILSFITEEKQDLIVLDTAPTGHLLRFLEMPTAIYQDIIFMRLLRQRVVKAQKILKDPKK

TEFIGVIRPQKGVIAEAERLYKSLAMHIPQNYLVLNCFTSNSVIPTDQFPGVQFVCMPMLPRSIEPIEQI

KTYIF------------------

>Q3MH41|Q3MH41_A

-----MMLHLVMFSGKGGVGKTTISCSFARYWARKQEKILLISTDPAHSLGDVLQSEI--ALTDL-----

LSVQALDAQKLLLEFKAEIRGSLAGG---DLAPVWDLNW--PGLNELMGLLEIQRLLAE-----EADRVI

IDMAPSGHTLNLLRLKDVILNSLELFQEK-HRV--ADEVDSFLVEMKQLAELLQDEKFTGCLVVGISEPM

CFYETERFLNSLETLDVPYAGLFINHILL------NSELEDRYAEQQNLLNKYL-NLHNQPVFIVPQQRV

EPLGASQIQKIASE---------------------QVLPGFHDFEECKLIIIGGKGGVGKTTVAAAIAWA

SAQQHPDKIQVISIDPAHSLGDAISLSSNLCGQEIDANRVLEQFRRDYLWELADMISGEGVAYVPEAWRQ

IMSQALPGIDEMLSLITDSNQDLIILDTAPTGHLLRFLEMPSALYQDVLFMRLLRQQVVQAQKKLKNSQH

TQFIGVIQSEVAIVSEHIRLTESLKNMGVNQRYIVQNRYSPEVEIDHSLFPEQTMIRLPGLPRSVEAIDR

VASLLFEVEELTA----------

>Q8YUT7|Q8YUT7_N

YDS----LHLVMFSGKGGVGKTTISCSFARYWA-RPQKILLISTDPAHSLGDVLQSEV-KDI-AL-----

LSVQALDAQKLLLEFKAEIRGSLAD--GGDLAPVWDLNW--PGLNELMGLLEIQRLLAD-----EADRVV

IDMAPSGHTLNLLRL-KDFLDVIFQEKH--RVI--ADEVDNFLVEMKQLAELLQDEKFTGCLVVGISEPM

CFSETERFLNSLETLDVPYAGLFINHILL------NSELEDRYAEQQNLLTKYL-N-LNQPVFIVPQRV-

EPLGV-ALDSLASI---------------------QVLPSFHDFEGCKLIIIGGKGGVGKTTVAAGIAWA

SAQQHPDKIQVISIDPAHSLGDAISLTSNLSGQEIDANRVLEQFRRDYLWELADMISGEGVAYVPEAWRQ

IMSQALPGIDEMLSLITDSNQDLIILDTAPTGHLLRFLEMPSALYQDVLFIRLLRQQVVQAQKKLKNFQH

TQFVGVIQSEVAIISEHIRLTESLKNMGVSQRYIVQNRYSPEVEIDHSLFPEQTMIRLPGLPRSVEAIDR

VASLLFEVEELTA----------

>A0ZJM9|A0ZJM9_N

---------M--FSGKGGVGKTTISCCFARYWATKEEKILLLSTDPAHSLGDILLSKVTDDGSQV-----

LSVRALDAEKLLVEFRAELRGSLAD--GEDLAPVWELNW--PGLNELMGLLEIQRLLSE-----TVDRIV

LDMAPSGHTLNLLQLEDVILNSFELFQQK-HRV--PDEVDDFLEHMKQLAELLQDETFTGCLVVTIAEPM

CLSETERFLDNLKTLNIHYTGILINRIIT-----DSDINTDRYAEQQNLTDKFL-K-INQPVFIVPQQAK

EPLGPLALDDLARI---------------------RILPSFSDFEGCQLIIVGGKGGVGKTTVAGALGWG

LANRHPQQIRIISIDPAHSLGDASSLATNLTGQEIDADEILDQFRTDYLWELADMISGEGIAYLPGAWRQ

IMSQALPGIDEILSLITDSNQDLIILDTAPTGHLLQFLAMPSALYQNVVLIRLLRQQVVQAQKKLKNPKH

TQFVGVIQAEDAIISEHIRLTASLKDMGIQQRYVVQNRYSQEIGIDPGLFPEQTIIRLPNLPRSVEPIAR

IANLLFDFD--------------

>D4TEP4|D4TEP4_9

----------ML-SGKGGVGKTTLSCCLARYWARKEEKILLLSTDPAHSLGDVLLTEVTNEPQSA-----

LSIQALDAQNLLLEFKAEIRGSLAD--GEDLAPVWDLDW--PGLNELMGLLEIQRLLSE-----NVDRIV

VDMAPSGHTVSLLKLKDVILHSLELFQKK-YQV--PDAVDDFLIDFKQLSELLQDSQFTGFLIVGIAEPM

CLAETERFLEQLKTLEVPFGGILINRILT------DPNMDDRYAEQQNYVQKFL-NLSGQPVFIIPQQPA

APLGSQALDKLAGI---------------------RIPPSFTDFQGCKLIIVGGKGGVGKTTVSAAMGWA

FASHYPQKISVISIDPAHSLGDAQPITPNLCGQEIDADKILDQFRTDYLWELADMISGEGIAYLPEAWRQ

IMSQALPGIDEMLSLITETNQDLIILDTAPTGHLLQFLSMPSALYQDVLFIRLLRQQVVKAQKKLKDPRH

TQFVGVIQAESAITSEHVRLTASLKNMGIEQRYVVQNRYTQAVEVDHNLFPEQTIIHLPLLPRSVEPIER

IANLLFAFEK-------------

>D8G9S6|D8G9S6_9

-----NQLAM--FSGKGGVGKTTLSCGFARRWA-KPHNILLISTDPAHSLGDVLQMEVEDAPKAI-----

LSVRALDSQKILQEFKAELRGSFVG----DLTPVWDLSW--PGLDELMGFLEIQKLLTE-----AADRVV

VDMAPSGHTVNLFGLKDVMLASLELFQQK-HRV--TDEADRFLTDMKELAELLEDTDFSACNVVAIAEPM

SLLETQRFLDSLHKLEIPCGGLFVNHIIT-----NTDTNADRYSEQQQLLQKFL-Q-LNHHIFTVPQQNS

EPLGAEALDHLIAI---------------------KIPPSFSDFEERQLILVGGKGGVGKTTVAAAMGWA

MANRYPDKIRIISIDPAHSLGDAQQLSDNLSAQEIDAEIVLDQFREDYLWELAE-MISGEMAYSPEAWRQ

IVAQALPGIDEMLSLVADSKQDLIILDTAPTGHLLRFLEMPTALYQNVLLMRLLRQQVVQSQKKLKDPSH

TEFIGVFQAQAAIVAEQVRLAESLKTMGVQQRYAVHNCYQPGVDIDGDLLPEQTIIRLPMLPRSVAPIAR

IANLLF-----------------

>F5UNY9|F5UNY9_9

-----DLLHLAMFSGKGGVGKTTIACGFARRWARLNQQILLISTDPAHSLGDVLQTKVQHNAFPL-----

LSVRALDAKELLLEFKAEVRGSFVE--GEDLTPVWDLDW--PGLDEVMGLVEIQRLLT------EVDRVV

VDMAPSGHTLNLLGIKNIVLNSLELFQEK-HRV--ADEVDDFLVKMKELAELLQNKDISACLVVAIAEPM

SLLETERFLESLQVLEIQSGGLFINRILT------DANTDDRYSEQQQLLKKFV-E-LKQPVFIVPQQAS

EPLGISQIQKIDTA---------------------KVLPSFCDFEGRQLILVGGKGGVGKTTVAAAIGWA

LASRYPDKIRLISIDPAHSLGDATQITTNLSGQEINADIVLEQFRNDYLWELAE-MMSGEIAYTPEAWRQ

IVAQALPGIDEMLSLIAERKQDLIILDTAPTGHLLRFLEMPSALYQNVLFMRLLRQQVVQAQKKLKDPNH

TEFIGVIQAQAAIIAEQVRLNESLQNMGVPQRYIVHNRYSQDSSLDAGLFPDQTIIHLPILPRSVEALDR

IANLLF-----------------

>A8Y9S9|A8Y9S9_M

MTNVNSLRHLVMFSGKGGVGKTTLSCGFARRWAKLEEQILLISTDPAHSLGDVLQTEVSDEALPV-----

LKVRALDAEKLLLEFKEELRGSFVGE---DLTPVWDLDW--PGLDEIMGLLEIQRLLL------EVDRIV

VDMAPSGHTLNLLEIKEIILNSLELFQEK-HRV--ADDVDDFLVKTKELTELLQNRDFTLCLIVAIAEPM

SLLETERLLNSLHHLNIPCGNLFINRILT------DPNQNDRYSEQQQLLDKFL-KI-PGQIFTLPQQAK

EPLGGEALDQIMSI---------------------KILPSFSDFDKRQLIIIGGKGGVGKTTVAAAIGWA

LANRHPEQIRIISIDPAHSLGDATQLTDNLSGQEVDANIVLEKFRDDYLWELAE-MISGELAYTPEAWRQ

IVSQSLPGIDEMLSLVKDQKEDLIILDTAPTGHLLRFLEMPTALYQNVLLMRLLRQQVMSAQKKLKDLQH

TEFIGVLQSQDAIVAEQLRLTASLKKMGVYQRYVVQNRYHANEEIDQDLFPDQTLIRLPSLPRSVEPLAR

VADLLF-----------------

>B0JPL9|B0JPL9_M

---------M--FSGKGGVGKTTLSCGFARRWAKLEEQILLISTDPAHSLGDVLQTEVSDQASPV-----

LKVRALDAEKLLLEFKEELRGSFVE--GEDLTPVWDLDW--PGLDEIMGLLEIQRLLI------DVDRIV

VDMAPSGHTLNLLGIKEIILNSLELFQEK------FSDVDDFLVKTQELTEILQDRDFTLCLIVAIAEPM

SLLETERLLNSLHHLNIPCGSLFINRILT------DPNQNDRYSEQQQLLDKFL-K-IQETIFTLPQQSK

EPLG-EALDQIMSI---------------------KILPSFSDFDKRQLIIIGGKGGVGKTTVAAAIGWA

LANRHPDQIRIISIDPAHSLGDATQLTDNLSGQEVDANIILEKFRDDYLWELAE-MISGELAYTPEAWRQ

IVAQSLPGIDEMLSLVTDQKQDLIILDTAPTGHLLRFLEMPTALYQNVLLMRLLRQQVMSAQKKLKDPQH

TEFIGVLQAQDAIVAEQLRLTASLKKMGVYQRYVVQNRYHANEEIDWDLFPDQTLIRLPSLPRSVEPLAR

VADLLF-----------------

>Q08Z69|Q08Z69_S

-------RVLHFFGGKGGVGKTTLAASYALMLSEDAKKVLLVSLDATRSLSDLVKKKLPAKPTKL-----

LYAAELEPLKFAAKYIPALAGKGTH-SEEDLGKIFAQAV--PGLEELVGLFHLQTLLED-----EFDRIV

VDASPTSHTLRLFDLPRKFLGIVKTG-AE------VVFLEETGARAERLLALLKDGTRSAFHLVALAEPV

PEAQTRMLFAQLRERGIPVTEILVNQVEA------CPACHGRRGLQAPHVRKFQ-A-LNVPVHLVAKREL

APRGLDGLKEFSKW---------------------FSAAEGPPARATRLIFFVGQGGVGKSSCAAAAAVT

LTEKEGPVLISTDPAHSLSRLTDGLYARELDVAGWFNALRKRLKEKAEKAFEGAKSGND-VPPDLAALRN

LLECAPPGIDELAALSCVQERKRIVVDPAPMVTAMRVVELADTAYRAKGADVALKHVKRFE-EALASPNE

SRFVVVTRGEDLAASRTERLVEYLKKKLQVERVLVNRVGPKSTCPKCENRRKLELNAAKAIEKKIGLPVT

MLGRHPAGLRELK----------

>F8CHQ0|F8CHQ0_M

-------RVLHFFGGKGGVGKTTLAAAYALRLSEEAKRVLLVSLDPVRSLSDLVKKKLPAKATRL-----

VYGLEVEPMKFLASYLPALAAKGTH-SEDDLGKLYQQAV--PGLEELVALFHVVDLLEG-----SFDRVV

VDAAPTSHTLRLFDLPRKFLGLVKAG-AD------AAFLEQVGQKAEKLLALLKDPARTAFHLVALAEPV

PEAQTRMLFTQLRERGLPVTEIVVNQIED------CPACQGRRGLQAPHVRKFQ-A-LTVPVHLLGRREV

APRGLEGLALFAKW---------------------FAAAEGPPARATRLIFFVGQGGVGKSSCAAAAAVT

LTEKEGPVLISTDPAHSLSRLTDGLYARELDIAGWFNALRKRLKEKAEKAFEGARSGSE-VPADLLYLRN

LLECAPPGIDELAALSCVQERKRIVVDSSPVVTSVRVVELAETAHRAKGADLAIKHVKRFE-DALASPSE

ARFVVVTRGEELAAARTERLVEYLKKGLPVERVLVNRVGPKSTCEKCENRRKLELNAAKAIEKKLGLPVT

MLGRHPAGLRELK----------

>Q1D553|Q1D553_M

-------RVLHFFGGKGGVGKTTLAAAYALRLSEDAKRVLLVSLDPVRSLSDLVKKKLPAKATKL-----

VYGLEVEPMKFLASYLPALAAKGTH-SEDDMGKLYQQAV--PGLEELVALFHVVDLLEG-----EFDRIV

VDAAPTSHTLRLFDLPRKFLGLVKAG-GD------AAFLEQVGQKAEKLLALLKDPARTAFHLVALAEPV

PEAQTRMLFTQLRERGLPVTEIVVNQIED------CPACQGRRGLQAPHVRKFQ-A-LTVPVHLLGRREV

APRGLDGLALFAKW---------------------FAAAEGPPARATRLIFFVGQGGVGKSSCAAAAAVT

LTEKEGPVLISTDPAHSLSRLTDGLYARELDIAGWFNALRKRVKEKAEKAFEGARSGSE-VPADLLYLRN

LLECAPPGIDELAALSCVQERKRIVVDSAPVVTSVRVVEMAETAHRAKGADIAIKHVKRFE-DALASPSE

ARFVVVTRGEELAAARTERLVEYLKKKLPVERVLVNRVGPKSTCDKCESRRKLEFNAAKAIEKKLGLPVT

MLGRHPAGLRELK----------

>E3K4G7|E3K4G7_P

-------ILWKILMSKGGKGGVAS----------CRESVLLVSMDPAHNLADTFCQKFSKHAMRVS---E

LYGIEIELQGDQDG---------------GMMPDPVFTI--PGMDEAMRFAEIMKRVQS-----KYSVII

FDTAPTSHALRFLSFPENAIEKLSLLSGHGPIMQ-FGKLNGMRALIAEVNGQLKDPRFFIYIILAGPDDI

QLTEELRGVKSITNFKMLIAPVLAQQTIVNAQ--ERELAQARWSIRQRYWKALGGIFGAFKKLHLSVVAR

AIRGPNGLMILQKIWEMQSVKFAKGRDSRTTNRTQAENSSTYDRSLFK------SGDVGPNPTKNQLKEW

GKIRLSAFSMAVFFLYGVAGWWSQKDVWSIVQLAHVKNKWIYAPDHLPTQHADEPWYTTDETNMACRDMT

QIDWEFAPAFWAKHVTELNIAFALEDILNQVCAQNPLKSLNMHGKVSPDDARKEKIKASISSGWKSTFKS

HHQSTSDHQQSTSTHKDSRKGGAEVLTRGV----------------------------------------

-----------------------

>B7FSN9|B7FSN9_P

-----DISRSVFVGGKGGVGKTTVSSALAVSLASEKDKVLIVSTDPAHSLGDALDEDLAMTDSLTG---G

LDACEVDASAALEDFRENIAAFDIDRADADLLESFGNPP--PGLDELVALSNVLD-SESA----GYDVVI

VDTAPTGHTLRLLALPDGLLGKLIKIRLQSGLAS-VNRLEQFRRKMSNLRERLQDSQSTRFVVVTVPTKL

GVAESKRLAAELNYQGVSITDIVVNQCVGSEA--LQQYYDRRKDGQKKWIAKLEEGSSPIGITRVPFFDV

ELVGVPALGYFTELSFSSEPRVVGGKGGVGTSSALSMASKGHKVISTD------PAHSIGDAIEIDLSGG

KLVDVPLIIPTTDGSLSVLEIDPVVDQLIGGDDNPSDAGLRNTLRDLQEVFDTLAGTDEVLVKKGGFDRI

VLDTAPTGHTLRMLSTPGFLALIDRLLIIAEKVNSNTAIKMLIGSSARSNAATSTLLSFQLQMYDLENLF

ADAAQTEFLIVTVPTELAVRESMRLNDLTFESPDMPIKCRNIVANQVLGDDGNDAKTFLDHVGQTQAISV

KDAVSSYPAPPLI----------

>F2NHZ5|F2NHZ5_D

LTFPD--LRLIFFGGKGGVGKTTCAAATALHHAS-PQSVLLVSTDPAHSLVDSLG-SF-HLP-DN-----

LTAKEFNAQQALKSFQAQHRDKFVSFDEEDIRQVLELSL--PGLDELMALLEIAGWVET-----SYTQII

VDTAPTGHTLRLLTIPRNWLKALDALMH--RFM--YQEMDRFLASLTKVKQLLHHTRLCRFVPVMLAEEI

VISETLKLLGELRRRQIPVVEIIVNRLYP------CPRCAAGYQRQRQLLAELA-SLSSWG---LPLFPD

EIRG-ANLATLWLL---------------------TPSGPATIQSPPTFLIFAGKGGVGKSTVASATALR

LAREFPERCLLFSTDPAHLKLPVGLTAIEIDAPGEFAAFKKRYRQDLERFFQSTKNID--VPFDRQVLER

LLDLSPPGLDEIMALVIDQGSDLFILDAAPTGHLLRLLELPELIYQLAFLSEMISRKVKLLRKMWQDPVK

TALYTVSILTEMAFQETSDLLAACNQGLWTPVLFLNQATPASDCPLCAALNRREALIRAKYQESFAKLHQ

TRQTTPRGLDLLD----------

>Q1INY9|Q1INY9_K

-------PSFTFVIGKGGVGKTTVAASLALHTAH-PRKTLLLSTDPAHSLADVLETKLGDTPKKL-----

LYARELDASAAVEEFLAAQREGILSLTRDEIAPLLDSAL--PGMAEVAALLAIHDLLES-----DYDEVI

VDTAPMGHTLRLFELPERFLH-LLEVSRD-AVLGGVARWQEMVRKVASLDH-----EHARLLLVTSSEKF

SLNEAIRAREQLQRAPVPIAEIVLNRAVT--ASG-CKRCTTAAKAARRFLKEFK-R---VPLREDP---G

SPIGVDALTAFGKV---------------------ALRLKQSKPEKTPLTLTLGKGGVGKTSAAMAFHAR

AKNAKEAVICSIDPAPSLDDVFQVLDDAKLFAAEIDAVGEYQRWAEEMRARVEDTSTEVRERDLFLAILD

VVPPGVDELFATFRILDLVERGRVQIDMAPTGHALEVLRTPARLHRTLPAAEIVSQRVRELSTTLSDSKR

SQVWVVMLAEPLPDRETRRLLCDLQ-----ELKAPVAGVFVNRVLMDETHCPRCSRAQAWQRQTLAKMKD

GVFVVPEMPEEIA----------

>A5G5D4|A5G5D4_G

---------M--FGGKGGCGKTTSAAAASIYLARLGKKVVLISLDPAHSLGDCFES-VGGDITRV-----

LWLLEMDARKLFQDFRKKYEGVMKK-AERGTYEDVEGFF--PGLDEVMAVIEVVRLLKS-----EFDLIV

LDTAPTGHTLRLLALPKKWIAVFDLMQH--RLL--RDATDEFLKTMTDLDRLLKDGMMTEFVPVTIPEPA

AIEETGRLLASLKEYRIAVRSLIVNRVVE-----DCPFCSSRRKGIEGYLAEIG-E-RDCNLVFAPLIHH

EINGFENLCKFAEL-----------------PVKIAAFRNAIAERKLEFLLFGGKGGVGKTTMAASTALY

MARENPERILILSTDPAHFDRTISSAGGHLFALEMDASRMLNVFQKEYCADIEAFSPFVAKEVMLGLIEL

SPPGLDEIMGLKKMLEL-RGADLFVIDTAPTGHALRFLETPEIVYKEIVAAGILLRDVKNVKKALTDSLQ

TEFVAVTIPESLAILETERLLSGIR------RLGIPSRHIIVNMVTPPAGCRCCKGEQEKYLRQVTAKWG

NVAVVPLFLSPVK----------

>Q011W9|Q011W9_O

IDVMIGRRKYYMVGGKGGVGKTSLSSSLAVKFAASGHKTLVVSTDPAHSLSDSLAQNVGGQPIEVNDTDG

LYALEIDPESAKAEFTQQKTDGARDFMSSVGLGGFADSILGPGLDEAIAIAKVLQFTKD-EKFSKFTRIV

FDTAPTGHTLRLLSLPDASIGKLTSATDAKGIFGVVEKLEKLKAQVKEVRSLFRNKETTEFIIVTIPTVL

GVSESGRLLQSLRDEDVPCTRLIVNQVLKAAQ-AAVNFCSIKEKDQSRALQMCD-GLRSLNRTEAPLFDM

EIRGVPALKFFASHSTESRRGQTPGTPPRHRHEDSPKFSFEIESRRATRAPFPSRRDLGLTASFLTPCDF

TTASEKARIAIAAEEEFNIEKLAAKEYERKETTVDTAKKIEASTSRNAMRLRVLAREEAMRRLGEVSGDA

RRYKDLLRALIVQGAKKLGDKVIVRCRESDAAVVRESTVAAAAELVGVSESRLAPACSGGVEVANSTGQI

VCDNTLDARLRIAYEQNTPLIREKMRRLATILST------------------------------------

-----------------------

>B0RA87|B0RA87_H

AKEPNEDTEFVFFSGKGGVGKSTVSCATATWLADNDYDTLLVTTDPAPNLSDIFNQDIGHEVTAIDDVPN

LSAIEIDPDVAAEEYRQETIEPMRALLGDEEIQTVEEQLNSPCVEEIAAFDNFVDFMDSP----EYDVVV

FDTAPTGHTIRLMELPSDWNAELEK--GGSTCIGPAASMDDKKADYERAIDTLSDESRTSFAFVGKPESS

SIDEIERSASDLAELGISSQLLVVNGYLPESV-CEDPFFEGKRADEQAVIDRVESTFDQQALATYPLQPG

EIAGLELLSDVGGLYDTVVDAATATNEDTVFFTDADAVAEELVPEETRYLFFTGKGGVGKSTIASTTAVS

LAEAGYETLVVTTDPAAHLADIFSVGQANLDAARIDQERALEEYRTQVLDHVREYDEKDDVANVEEELES

PCAEEMAALEKFVSYFEEDGYDIVVFDTAPTGHTLRLLELPSDWKGFMDTKAANGGKYDEVIETMQDPSR

SSFAFVMYPEFTPMMEAYRAAMDLQQVGIETSVVVANYLLPEDYGDNAFFENRRAQQAEYLEEISERFDV

PMMLAPLRQEEPVGLDDLREFGA

>B9LWW4|B9LWW4_H

-RDPSDDTEFVFFSGKGGVGKSTVSCATATWLADNDYETLLVTTDPAPNLSDIFDQVIGHEVTEIEGIEN

LSAIEIDPDTAAEEYRQETIEPMRQLLGDDEIETVEEQLNSPCVEEIAAFDNFVDFMDSP----EYDVVV

FDTAPTGHTIRLMELPSDWNAELEK--GGSTCIGPAASMEDKKVQYERAIDTLQDTEQTTFAFVGKPEDS

SIDEVERSAGDLAELGIESQLLILNGYLPESV-CEDPFFEGKREDEQAVIERAREEFDADATGTYPLQPG

EITGLDLLSDVAGLYDTVVGSATIETDQSVVLADPASVADRVTPDETRYLFFTGKGGVGKSTIAAASATK

LAEAGYETLVVTTDPAAHLEDIFSVSQANLDAARIDQEKALEEYRTQVLDHVTEYEDKEDIANVEEELES

PCAEEMAALEKFVSYFQQDGYDVVVFDTAPTGHTLRLLELPSDWKGFMDTKAAKGDQYDEVIETMQDPER

SSFAFVMYPEYTPMMEAYRAAEDLNQVGIETAFVVANYLLPEEYGDNAFFANRRAQQEKYLGEIKDRFET

PLMCAPLRRDEPIGLEELSAFGD

>C7P537|C7P537_H

VRAPTKETEFVFFSGKGGVGKSTVSCATATWLADNDYETLLVTTDPAPNLSDIFGQEIGHDVTAIDDIEN

LSAIEIDPDTAAEEYRQETIEPMQQLLDDEQLETVEEQLNSPCVEEIAAFDNFVDFMDCP----EYDVVV

FDTAPTGHTIRLMELPSDWNAELEK--GGSTCIGPAASMEERKQDYERAIDTLQDGERTSFAFVGKPEDS

SIDEIERSARDLGELGIESQLLIINGYLPEPV-CEDPFFQGKRADEQAVIERARTEFDADAMATYPLQPG

EIAGLDLLADVGGLYDTVVGTATVDAETAVFMADSEAVADQLQPDETRYLFFTGKGGVGKSTIASTAATK

LAEAGHETLVVTTDPAAHLEDIFSVGQANLDAARIDQEKALEEYRTQVLDHVTEYEDKEDIANVEEELES

PCAEEMAALEKFVSYFDEDGYDVVVFDTAPTGHTLRLLELPSDWKGFMDTKAAKGDQYDEVIETMKDPER

STFAFVMYPEYTPMMEAYRAAADLKQVGIETSLVVTNYLLPEEYGDNAFFENRRAQQAEYLGKINDRFDV

PMMLAPLRQDEPIGLDELRAFGE

>Q18H24|Q18H24_H

TRAPTEETEFVFFSGKGGVGKSTVSCATATWLANNNYETLLVTTDPAPNLSDIFGQNIGHEVTAINDIEN

LSAIEIDPDTAAEEYRQETIEPMRQLLDDEQLETVEEQLNSPCVEEIAAFDNFVDFMDSP----EYDIVV

FDTAPTGHTIRLMELPSDWNAELEK--GGSTCIGPAASMEERKHEYERAIDTLQNNNRTSFGFVGKPEDS

SIDEIKRSASDLGDLGIESQLLIINGYLPESV-CEDPFFEGKYEDEQAVIDRAQTEFDADAMATYPLQPG

EIAGLDLLADVGGLYDSVVGTATAEAETVGFMVDAEAVADQLRPDETQYLFFTGKGGVGKSTIASTTATK

LAEAGYETLVVTTDPAAHLQDIFSVSQANLDAARIDQQKALEEYRTQVLEHVTEYENKEDIANVEEELES

PCAEEMAALEKFVSYFDEDGYDVVIFDTAPTGHTLRLLELPSDWKGFMDTKAAKGDQYDKVIDIMKDPKR

SSFAFVMYPEYTPMMEAYRAAADLEQVGIETSLVVANYLLPEEYGDNAFFKNRRAQQAKYLTEISDRFDV

PMMLAPLRQEEPVGLDELRAFGE
