# Supplementary material for: Accurate Simulation and Detection of Coevolution Signals in Multiple Sequence Alignments
Source: PLoS One. 2012 Oct 16;7(10):e47108. doi: 10.1371/journal.pone.0047108 (PMC3473043; doi:10.1371/journal.pone.0047108)
Supplement: MSA S3 — MSA of ArsC. (DOCX) [file pone.0047108.s014.docx]

>E0TFQ0|E0TFQ0_PARBH

MAMIVIYHNSRCGTSRVTLSLIKSTGADPVIVDYLKAGWTEGHLLGLFAAAGLTPREALRAKEAKAKELG

LHEEGVPAADILAAMVAHPVLVNRPFVCTKKGVRLCRPSETVLTLLDGF-KGPVTRENGEMLIDGDGRPV

-

>D5VK30|D5VK30_CAUST

DFPVVIYHNPSCGTSRNVLAMIRAAGYEPTVIEYLQTGWTHDQLRDLASAAGLTFRQLMRTRGAPAEELG

LTADDVSEAEILDAMVAHPILVNRPLVVTQRGVKLCRPSEVVFDLLD--LPSSFTKEDGEVVDLS-----

-

>D5VK91|D5VK91_CAUST

DFPVVIYHNPNCGTSRNVLAMIRAAGYEPKVIEYLRTGWTHDQLQDLAKEAGLSFHQLMRTRGTPAEELG

LTAEGVSEAKILEAMIAHPILVNRPLVVTPRGVKLCRPSEVVFDLLDR-HPDSFTKEDGEVVDLS-----

-

>B0T3M8|B0T3M8_CAUSK

DFPIVVFHNPSCGTSRNVLAMIQATGAEPTVVEYLKAGWTREQLTELLAEAGATPRDWLRDKGAPAAELG

LLAPEASDAAILDAMAAHPILVNRPIVVTPKGVALCRPSERVFDLLE--APESFTKEDGEVVSSR-----

-

>B8H5W8|B8H5W8_CAUCN

AFPITIFHNPACGTSRNTVAMVQAAGYAPQVVEYLKTGWTREQLQDLAAKSGGSLRALMREKGTPAETLG

LLADEVSDERLLDAMVEHPILVNRPIVVTPKGVKLCRPSEQVLDLLDR-KPEQFTKEDGEVVTP------

-

>D5VM96|D5VM96_CAUST

DFPITIFHNPACGTSRNVVAMVRAAGYEPEIVEYLKAGWTRDQLKDLAAKSGLSVRQLLREKGTPAEALG

LLADGVAEDRILDAMIEQPILVNRPIVVSPKGANLCRPSELVLDLLDQ-RPASFTKEDGEVVKL------

-

>A8HZ79|A8HZ79_AZOC5

DFPVTIYHNPACGTSRNTLAMIRAAGYAPTVVEYLKTGWTQDQLKGLISAMGGTARDILREKGTPAAELG

LLVPEVSEADLLAAMVAHPILVNRPIVVTPLGTRLCRPSERVLDLLE--KPDSFTKEDGEVVALK-----

-

>B4R9T5|B4R9T5_PHEZH

EFPITIFHNPACGTSRNTVAMVRAAGYEPEVVEYLQAGWTKPQLQELLAAMGATPRDILRGKGTPAAELG

LLEPHVGDDAILDAMVAHPILVNRPIVVTPKGVKLSRPSEAVLDLLD--KPDSFTKEDGEVVRLA-----

-

>Q28QJ3|Q28QJ3_JANSC

-MSIVIHHNPGCGTSRNALSILRASGVEPVVVEYLETGWTKSQLQGLFAAAGLTAREALRVSNSPAAELG

MLEEGVTEDALLTAMIAHPVLVNRPIICTAKGVRLCRPSARVLDLLDEP-TGAVYADDGEMILNEKGAPV

-

>E8RW09|E8RW09_ASTEC

-MQPVIYHNPDCGTSRNVLAVIQAAGYEPEIIEYLKVGWNADELRNLLAYAGLTPRQALRETKSPAKELG

LLDPAVTDDVIFEQMLVHPVLVNRPIVITDKGTKLCRPSEVVLDLLDTP-KGPFLKEDGTEMINSAGKRG

L

>E8RMF5|E8RMF5_ASTEC

-MTVTIYHNPDCGTSRNVLAVIEAAGYSPQIVSYLSVGWKREQLLTLFAEAGLTPRQALRESKSPATELG

LLDPGVSDEALIEAMLTHPILVNRPFVVTPKGTRLCRPSEVVLDLLEHP-AGPFFKEDGALVIDANGHRA

-

>E8RM43|E8RM43_ASTEC

-MTVTIYHNPDCGTSRNVLAVIEAAGYQPDVVEYLQTGWHSDHLSKLLADAGLSPRQALRETKSPARDLG

LLEPGVSDDVIFEAMLTTPILVNRPIVVTPKGTRLCRPSEFVLDLLENP-AGPFQKEDGELMIDAEGQR-

-

>F4QMH2|F4QMH2_9CAUL

-MSVTIYHNPDCGTSRNVLAVIEAAGYAPVVVEYLQAGWEREPLVKLLADAGLTPRQALRETKSPAKELG

LLEPGVSDEQILAAMMSTPVLVNRPFVVTPKGTRLCRPSEIVLDLLERP-AGPFYKEDGALMIDVGGQR-

-

>C9D116|C9D116_9RHOB

-MDIVIHHNPDCSNSRNVLRLIRDAGYEPVVVEYLKTGWTRGQLQALFAAAELTPYSAMRKERSPAKELG

LLEDNVTADQILDQMLLHPSLVNRPIVACKNGVALCRPPERVLDLLESP-SGPWSKEDGTLLIDADGNR-

-

>B0U0E1|B0U0E1_FRAP2

--MIVIYHNQECGTSRNVLEIIRAAGYDPIVIEYLKEGWTKSQLLGLFAAADLTPKEALRKTKSPAKELG

LLDDNISDDVIINAMINNPILVNRPIVCSPKGVKLCRPSEEVLSILDKP-SGPFYKEDGQLLIDIKGQRL

-

>A9D2D1|A9D2D1_9RHIZ

-MSIVIHHNPGCGTSRNVLEIIRGFAGEPVVVEYLETGWTRPQLLGLFAAAGLTPREALRVSKSPAEELG

LTDPSVSDEVILDAMIEHPVLVNRPIVCTPKGVALCRPSEAVFALLDVS-GASYTKEDGEVVTAP-----

-

>F5ZD77|F5ZD77_ALTSS

--MIVIHHNPACGTSRNVLKIIEDAGYSPIVVDYLNEGWTRSQLLGLFAAANLTPRQALRTTKSPAEELG

LLDESVSDDALLDAMLVHPVLVNRPIVCTEKGVGLCRPSEKVLDLLPSP-SGPYFKEDGEQILDELGKR-

-

>F6CYC9|F6CYC9_MARPP

--MVLIHHNPDCGTSRNVLRIIKDAGYTPLVIDYLKEGWTKAQLLALFAAADLTPRSALRETKSPAKELG

LLNKSVSDDIILEAMLEHPVLVNRPIVCSPKGVKLCRPSEAVLDLLEQP-EGPYEKEDGELILDAKGNRS

H

>A3SNR8|A3SNR8_9RHOB

-MNIVIHHNPACGTSRNVLQIIRDAGYEPTVIEYLDTGWTRPQLQALFAAAHLTPREALRVSKSPAEELG

LTDPSVSDEAILDAMLEHPVLVNRPLVASPKGVRLCRPSETVLDLLELP-AGPYAKEDGELIIDAEGNRV

-

>F4ANM3|F4ANM3_GLAS4

--MVVIHHNPACGTSRNVLSIIQDAGYQPEVIEYLEEGWTRPQLLALFAAANLSPRRALRTTKSPAKELG

LLDEDVSDEAILDAMLTHPILVNRPIVCTEKGVKLCRPSEQVLDLLAQP-PGPYKKEDGELIIDDKGERV

G

>Q2C1L0|Q2C1L0_9GAMM

--MVVIHHNPECGTSRNVLQIIQDAGYEPVVIEYIQEGWTKPQLQALFAAANLTPRTALRTSKSPAKELG

LLDESVSDEVILEAMLEHPVLVNRPIVCTAKGVKLCRPSEEVLDVLENP-KGPLIKEDGEVIIDSNGNRL

-

>B6IXM5|B6IXM5_RHOCS

-MAVVIHHNPACGTSRNVLTMLRAAGYEPVVIEYLKTGWTEPQLRGLFAAAGLTAREALRSRGTPAEQLG

LLDPTVSEEELIAAMVRHPVLVERPIVCTPKGVRLCRPSETVLDLLDRP-PGPLAKEDGELVIDGAGRRR

-

>A9EEE2|A9EEE2_9RHOB

-MSVVIHHNPDCGTSRNVLAIIKASGEMPMVIEYLETGWTRPQLLGLFAAAGLTPRTALRTTKSPAEELG

LLDPAVDEETLNAAMLEHPVLVNRPIVCAPKGVRLCRPSETVLDLLDRP-PGPLSKEDGTLLIDTEGRRG

-

>A3SEK5|A3SEK5_9RHOB

-MSVVIHHNPDCGTSRNVLAIIKASGVEPVVIPYLDTGWTRGQLLGLFAAAALTPRSALRVKRSPAEALG

LLDPDVDDEALLAAMLEHPILVNRPLVCTPKGVRLCRPSEAVLDLLDHP-QGPLTKEDGTLLIDAEGNRA

-

>A3TUC9|A3TUC9_9RHOB

--MIVIHHNPDCGTSRNVLAFLRASGVEPVVIEYLKEGWTRPQLLALFAAAGLTPRAALRETKSPARELG

LLEEDVSEDALIDAMLAHPVLVNRPIVCTAKGTRLCRPSEAVFDLLGQP-PGPLFKEDGQMVIDENGAR-

-

>Q0C3H8|Q0C3H8_HYPNA

--MIVIHHNPDCGTSRNVLAIIKASGAEPVVIDYLKEGWTRPQLLGLFAAAGLTPRAALRESKSPAKELG

LLEPGISDEAILAAMLEHPILVNRPLVCSPKGVRLCRPSEAVLDLLESP-PGPLYKEDGEMIIDEKGQRG

-

>Q3DB41|Q3DB41_STRAG

MEKVRIYHNPNCGTSRNVLAIIRHCGIEPEIIYYLKTPPSKMALVELLLEMKLSVRELLRTDVPAYEKFN

LESSSVTDEEMIDAMIQDPVLINRPIVVTSKGAKLCRPCEEILTILPVKMEKDFVKEDGQIIQSL-----

-

>B9CVP9|B9CVP9_9PAST

MCSVTIYHNPKCGTSRNTLALIRHLGIEPTIIHYLEQPPSEAVLRELLTKMALTPRQLLRTNVPPYLEKG

LDDLTLSDDTLIAAMLADPILINRPIVITEKGVRLCRPSEVFLQISP-YLPSDFIKEDGSVITAE-----

-

>C5S0K3|C5S0K3_9PAST

MCSVTIYHNPKCGTSRNTLALIRHLGIEPTIIHYLEQPPSETVLRELLAKMALAPRQLLRTNAPPYLEKG

LDDLTLSDDALIAAMLAEPILINRPIVVTEKGVRLCRPSEVFLQISPRPLQTDFIKEDGNVIAA------

-

>E0EKU5|E0EKU5_ACTPL

MEKITIYHNPKCGTSRNTLALIRHLGIEPTVIYYLETPPDEATLRRLISAMGINVRELLRTNVPAYDKLN

LAQTELSDDELIAAMLAEPILINRPIVITSKGVRLCRPSEKFLEISPVSLDKPFIKEDGGVIEL------

-

>E8KIP4|E8KIP4_9PAST

MEKVTIYHNPKCGTSRNTLALIRHLGIEPTIIHYLETPLDEVTLRNLIAEMGICVRDLLRTNVPPYETLN

LSRMELRDDELIAAMLAEPILINRPIVITSKGVRLCRPSEKFLEISPVSLNKPFIKEDGEIINNL-----

-

>A3N195|A3N195_ACTP2

MEKVTIYHNPKCGTSRNTLALIRHLGIEPTIIHYLETPPDEVTLRNLISEIGIRVRDLLRTNVPPYETLN

LSRMELSDDELIAAMLAEPILINRPIVITSKGVRLCRPSEKFLEISPVSLDKPFIKEDGGVIEL------

-

>A4N4R5|A4N4R5_HAEIF

-MNITIYHNPNCGTSRNVLALIRHAGIEPQVIEYLKNPPSETTLRDLIKRMGITPRQLLRTNVPPYETFD

LQNEKLSDDELISAMLREPILINRPIVVSEKGVKLCRPSETVLAFLP--FATPFVKEDGEIINHK-----

-

>C8PXF2|C8PXF2_9GAMM

MQDITIYHNPNCGTSRNTLALIRHVNGEPNIIYYLETPPSEAKLRELLQQMAMTPRQLLRTNVPPFAERG

LENPDLSDDDIIQHMLAEPILINRPIVVSDKGVKLCRPSEVVLDLLDKPLTEPFVKEDGEVIPP------

-

>C8Q0G8|C8Q0G8_9GAMM

MQNITIYHNPNCGTSRNTLALIKHQGIEPSVIHYLDNPPDETTLRDLITKMGITPRQLLRVNVPPYAERK

LDNLSLTDDDLIQAMLADPLLINRPIVVSDKGVKLCRPSEVVLDLIDTPLTKTFIKEDGEKIEP------

-

>F8DHS8|F8DHS8_STRPA

MEQVTIYHNPNCGTSRNALAIIRAVGIEPTIIEYLKTPPTEQKLRQLLLQMNMSARELLRTNVPEFEEYD

LQKDK-TEQDIINAMMVEPILINRPIVVTDKGTLLCRPSEKVLNILPKSLDKDFIKEDGEIISSS-----

-

>B9DUK2|B9DUK2_STRU0

MEEITIYHNPNCGTSRNVLAMIKHAGIEPTVIEYLINPPSRDQLLDLIDAMGISVRDLLRTNVPEFDKHG

LASLDKSDEDIIDAMMVDPILINRPIVITSKGTKLCRPSEKVLDILPKPLPSPFTKEDGEVVYPK-----

-

>E4L3M8|E4L3M8_9STRE

MEKVCIYHNPNCGTSRNVLAMIKHAGLEPEIVEYLVTPPTREELQELIKAMGVQVRDLLRTTVPEFEKHH

LADPTRSDSQLLDAMMADPILINRPIVVTSKGVKLCRPSEVLLDILPVRLPSPFTKEDGQVVQPK-----

-

>F3LB71|F3LB71_STRPO

MRKVRIYHNPNCGTSRNVLAMIKHAGLDPEVIEYLVTPPTREELQGLIRAMGIQVRDLVRINVPEFEKHH

LADATKSDSQLLDAMMADPILINRPIVVTSKGVKLCRPSEVLLDILPVRLPSPFTKEDGQVVQPK-----

-

>F1Z132|F1Z132_9STRE

MGKVTIYHNPNCGTSRNVLAIIRHAGIEPQVIEYLQNPPSREKLLDLVAAMGISFQELVRKNVPEYNQHG

LDKEAVTEDEILDAMMEDPILINRPIVVTRKGTKLCRPSEALLEILPVPLPSPYTKEDGEVVNPL-----

-

>E7PZ10|E7PZ10_STRDY

TEEITIYHNPNCGTSRNVLAMIRHAGIEATIIEYLQTPPNRETLLELLQSMGISARELLRTNVPEFEAHG

LANQAVAEKDIINAMLADPILINRPIVVTRKGVKLCRPSETLLDILPVPLPSPYIKEDGEIVNPI-----

-

>C5AP15|C5AP15_BURGB

-MSVIIYHNPECGTSRNVLALLGEAGEDLKVVEYLQSPPDRATLERLIADSGMDVRDAMRIKGTPYKELG

LGDPSVTASQLIDAMLDHPILINRPFVVTPRGTRLCRPSDAVLDLVESLPDRDVLKEEGVPFIVAGGSRG

Y

>E1T8E2|E1T8E2_BURSG

-MSVTIYHNPDCGTSRNVLALLREAGENPQVIEYLRNPPDRATLERLIADSGMAVRDVLRKKGTPYEELG

LGNCALSDAQLIDAMLAHPVLINRPIVVTPRGTRLCRPSDAVAGLLANPPQREILKDEGVPFIVAAGSHG

Y

>D7A9W9|D7A9W9_STAND

-MDIIVYHNPDCGTSRNTLALIRHAGIEPHVIEYLKTPPTPAMLLQLASRIGWPLRNLLRERGTPFKELG

LDDSTLDDAALLAAVAEHPILLNRPIVVTPLGVKLCRPSETVLDLLPVP-NVDLDKDDGSPFLRDSGTLG

Y

>G4RCZ2|G4RCZ2_9RHIZ

-MSVVIYHNPECGTSRNTLALIRHYGLAPEVIEYLKAPPTRSELADLIARAGLSVRAALRKKGTPYAELG

LNDPSLNDSVLLDAMMAHPILINRPLVVTPKGVALCRPSDIAADLLPDP-VPNLLKEEGTPFLKDDGATG

Y

>D0SW31|D0SW31_ACILW

MPKVQIYHNPACGTSRNTLALIRNAGIEPEVIEYLLTPPSKDQLVNMIRDAGLTVREVLRKNVDPYTELN

LDDAGWSDEKLLDTMLQHPILINRPLVVTEFGTRLCRPSEVVLDILRLPQLKAFAKEDGEIIVDEQGNRK

-

>C6RNK9|C6RNK9_ACIRA

SQSIKIYHNPECGTSRNTLALIRNTGQEPVIIEYLKTPPLKAELIQLIKNSGLTVREAIRKNVAPYKKLD

LDKLHWTDEQLITLMLEYPILINRPFVVTELGTKLCRPSEIVLDILSAPQRKAFSKEDGELIIDQNGKRR

-

>D0S362|D0S362_ACICA

TELVKIYHNPACGTSRNTLALIRHAGIEPIVIEYLQTPPSKDELTQLISQAGLTVRDAIRKNVDPYKDLD

LEQDSWTDEQLIDFMSQHPILINRPFVVTSKGTRLCRPSEIVLDILDSENLGFFAKEDGEVIIDEQGSRK

-

>D0C0D6|D0C0D6_9GAMM

SDQLKIYHNPACGTSRNTLALIRHAGFEPIVIEYLQTPPSKDELIQLIQDSNLTVREAIRKNVDPYKELE

LEQDHWTDEQLIDFMVQYPILINRPFVVTSKGTRLCRPSEVVLDILDSKSLGYFAKEDGEVIIDEQGHRK

-

>D8JLR4|D8JLR4_ACISD

TELVKIYHNPACGTSRNTLALIRHAGLEPIVIEYLKTPPSKDELIELIKDSNLTVREAIRKNVDPYKDLE

LEQADWTDEQLIDFMVQYPILINRPFVVTPKGTRLCRPSEVVLDILDSKNLGYFAKEDGEVIIDEQGSRK

-

>D6JQ88|D6JQ88_ACIG3

TELVKIYHNPACGTSRNTLALIRHAGFEPIVIEYLQTPPSKDELIQLIKDSGLTVREAIRKNVDPYKELE

LEQDHWTDEQLIDFMVQYPILINRPFVVTPKGTRLCRPSEIVLDILDSQNLSYFTKEDGEVIIDEQGRRK

-

>C6RRD0|C6RRD0_ACIRA

FSTIKIYHNPECGTSRNTLALIQNAGIEPIVIEYLITPPSKAELIELIRSAGLSVREAIRKNVPPYSDLE

IVREDWSDEQLLNFMLQHPILINRPFVVTDLGTRLCRPSEVVLDILPFPQKGAFSKEDGEKIIDENGQRK

-

>D0S8B1|D0S8B1_ACIJO

TPKVKIYHNPECGTSRNTLALIRNANIEPEVIEYLVTPPSKNELIQMIADAGLTVREAIRKNVAPYTELG

IDQEDWTEDQLLNFMLQYPILINRPFVVTELGTRLSRPSELVLEILPLPQKGAFTKEDGEQVLDANGQRK

-

>D0SQ38|D0SQ38_ACIJU

QQQITIYHNPACGTSRNTLGLIRNTGIEPIVIEYLLNPPSKSELIQLISDAGLSVRAAIRTNVDPYRELD

LDRETWTDHELIEFMLEHPILINRPFVVTSQGTRLCRPSELVLDILPVAQQGAFSKEDGEQVIDENGQRN

-

>D6JVY3|D6JVY3_ACIG3

SLDVKIYHNPACGTSRNTLALIRNTGIEPTVIEYLITPPSHDELVKLIQDANLTVREAIRQNVDPYRDLQ

LDRSDLSDEQLLSFMLEHPILINRPFVVTELGTRLSRPSEVVLDILPLPQKGAFTKEDGEQVIDENGQRN

-

>C0VPW1|C0VPW1_9GAMM

SLDVTIYHNPACGTSRNTLALIRNTGIEPTVIEYLVTPPTREKLIQLIADAGLSVRDAIRKNVDPYRDLA

LEREDWTDEQLINFMLEHPILINRPFVVTALGTRLSRPSEVVLDILPLSQKGAFTKEDGEQVIDENGQRN

-

>E6WRJ8|E6WRJ8_PSEUU

MSAITIYHNPRCGTSRNTLAMIRNSGVEPEVIEYLQHPPTLERLRELAAAAGVGVRGLLRAKEPLCAELG

LDDASLDDETLLQAMVANPVLINRPVVVTPLGTRLCRPSEVVLEILPDPQRGAFSKEDGEAVVGADGRR-

-

>B6QZQ7|B6QZQ7_9RHOB

-MNPIIYHNPKCGTSRNTLAMLKQAGTEPEVIEYLKTPPSRETLEKLIKDSGLTVREILRKKGTPYEELG

LDDEKWTDAQLIDFIEEHPILMNRPFVTTDKGTRLCRPSEVLLEILDEEQIGAFTKEDGEIVIPAKG---

-

>A2SIB0|A2SIB0_METPP

MSTVTIYHNPDCGTSRNTLALIRASGIEPTVIEYLKTPPDRETLKALIARMGMGVRDVLRIKGTPYKELG

LDAAHWSDDQLIDQMLAYPILINRPIVVSRSGVRLCRPSDTVIDLLPQP-AGENRKEDGTPLLVDSGERG

Y

>F4G6F4|F4G6F4_ALIDK

MSSVMIYHNPDCGTSRNTLALIRASGIEPTVIEYLKAPPDRETLNALIARMGMRVRDVLRVKGTPHKELG

LDAAHWNDDQLIDQMLAHPILINRPIVVSPLGVRLCRPSDMVVELLPKP-AEEIRKEDGTSLLVDSGERG

F

>A6T1C2|A6T1C2_JANMA

-MHVKIFHNPDCGTSRNTLALIRHLDIEPEIVEYLRTPPARDELSLMIKNAGLTVRDALRKKGTPYAELG

LDDPALGDDALLDAMIAHPILINRPFVITPVGTRLCRPSELVLDLLP--PAKAFIKEDGELVIDEHGQRS

Y

>G0CCQ4|G0CCQ4_XANCA

-MHAVIYHNPACGTSRNTLALIRHVGIEPQIIEYLRDPPTRETLQALIAEAGLSVRDALRQKGTPYLELG

LDDPALDDDALLSVMLAHPILINRPFVRTDRGVRLCRPSEQVLELLP-AATSGFIKEDGERVLDEAGRRS

Q

>F0BJW3|F0BJW3_9XANT

-MHTVIYHNPACGTSRNTLALIRHVGIEPQIVDYLQHPPSRATLQSLIADAGLSVRDAMRQKGTPYLELG

LDNPALDDAALLSAMLVHPILINRPFVRTPLGTRLCRPSEQVLDLLP--ATRAFFKEDGERVLDETGQRG

G

>B2SLS3|B2SLS3_XANOP

TMHAVIYHNPGCGTSRNTLALIRHTGVEPQIVDYLQHPPSRETLQSLIAAAGITVREAIRQKGSPYLELG

LDDPALDDTALLSAMLAHPILINRPFVQTPLGTRLCRPSELVLDLLP--ATRGFVKEDGESLLDEAGQRR

R

>G2LTF8|G2LTF8_9XANT

-MHAVIYHNPGCGTSRNTLALIRHTGIEPQIVDYLQHPPSRQTLQALIAAAGITVRQAMRQKGTPYLELG

LDDPTLDDAALLSAMLAHPILINRPFVQTTRGTRLCRPSELVLDLLP-PATHGFIKEDGERVLDEAGQRG

R

>D4SSL2|D4SSL2_9XANT

-MHAVIYHNPGCGTSRNTLALMRHIGVEPQIVDYLQHPPSRQTLQSLIAAAGITVREAIRQKGTPYLELG

LDDPTLDDATLLSAMLAHPILINRPFVQTARGTRLCRPCERVLDLLP-PATHGFIKEDGERVLDEAGQRG

R

>C8RWG0|C8RWG0_9RHOB

DFPVRIYHNPACGTSRNVLGLIRNAGVEPEIIEYLSAPPTRAELLDLAARMAMPLRDLLRVNGTPYAELG

LADPSLTQEQLLDAMMAHPILINRPIVVTPLGVRLCRPSETVIELLPA-QRGAFTKEDGQPVVNADGWPG

T

>B8EL89|B8EL89_METSB

-MDVIIYHNPKCGTSRNVLGLIRNAGIEPHVIEYLKTPPTRLLISQLAARTGQPLRALLRDKEAIFASLG

LDKPDVSDDALLDAIEKHPVLLNRPIVVSPKGVKLCRPSELVLDLLP-AQQGEFFKEDGERVVDEKGRHA

S

>D9QMN1|D9QMN1_BRESC

-MDVVIYHNPGCGTSRNTLALIRHVGIEPHVIEYLRTPPSRALITELASRASVPLRALLREKEAAFADLG

LGDPGLGDDRLLDAIEAHPVLLNRPIVVSPLGVRLCRPSETVLDLLPAEGLQPFTKEDGEVVVDAAGRRR

S

>F4QV51|F4QV51_BREDI

-MDVVIYHNPACGTSRNALALIRHVGIEPQVIEYLKTPPNRAMIQALVERMGVPLRGLLREKGTPFTELV

LGDPDLTDDQILDAIEAHPILLNRPIVVAPLGVKLCRPSEAVLDLLPSEGLKPFTKEDGEVVIDAGGRRR

-

>F4QXT3|F4QXT3_BREDI

-MDVVIYHNPACGTSRNALALIRQLGIEPHVIEYLKTPPSRAMILGLVARMGVPLHSLLREKGTPFVELG

LSDPALTNDQLLNAIEAHPVLLNRPIVVSPLGVKLCRPSEAVLDLLPPEGLKPFTKEDGEVVIDAEGRRR

-

>B4W8X8|B4W8X8_9CAUL

-MDVIIYHNPACGTSRNTLALIRHVGIEPHVIEYLKTPPSRALIQDLAARTGAPLRDLLREKGTPFAELG

LGDPAISDDQLLDAIEAHPVLLNRPIVVTPRGVRLCRPSEAVLDLLPEDGLKPFVKEDGEVVIDAEGRRR

-

>B4WBH0|B4WBH0_9CAUL

-MDVVIYHNPACGTSRNALELIRHVGMEPHVVEYLKTPPSRAMIAWLAERTSAPLRDLLREKGTPFAELG

LGDPGLTDDQLLDAIEAHPILLNRPIVVSPMGVRLCRPSEAVLDLLPADDLKPFTKEDGEVVIDAAGQRK

R

>A6UJK4|A6UJK4_SINMW

PIDIVIYHNPDCGTSRNTLAMIRNAGIEPHVVEYLKTPPSRPLLEQLLVRMGISVRDLVREKGTPYQELG

LGDPALTDGQLLDAMMQHPILINRPIVVSPLGVKLCRPSEAVLEVLPQEQKGAFIKEDGQVVVDASGRQ-

-

>E9KN46|E9KN46_9RHIZ

PVDIVIYHNPDCGTSRNTLAMIRNAGIEPHVVEYLKTPPSRALLEQLIDRMGISARDLLREKGTPFSELG

LGDTSLSDEQLVDAMMEHPILINRPIVVTPAGVKLCRPSEVVLDILPADQQAAFTKEDGEVVVDQTGRRV

-

>A1B6S2|A1B6S2_PARDP

-MDVVIYHNPDCSTSRNVLAMIRNAGIEPHVVEYLKTPPSRAMLERLIARMGIAPRELLREKGTPYAELG

LDDPALTDAALIEAMLAHPVLINRPIVVSPRGVRLCRPSEQVLDLLP-PQQAAFSKEDGEQVVDAQGNRR

P

>F8HKT8|F8HKT8_PARDS

-MDVVIYHNPDCGTSRNVLAMIRNAGIEPHVVEYLKTPPSRAMLERLIARMGIAPRELLRQNGTPYVELG

LDDPALTDATLIDAMLAHPILMNRPIVVSPKGVRLCRPSEQVLDLLP-PQRAAFSKEDGEQVVDAQGNRR

P

>D5QV15|D5QV15_METTR

APDVIIYHNPDCGTSRNTLGLIRNAGIEPHVIEYLKTPPTRALLAQLIARMGISTRALLREKGTPFQELG

LGDPTRTDEALLDAMMAHPILINRPIVVTSMGVKLCRPSEAVLDILPLPQRGAFYKEDGELIVDSAGRRA

T

>Q21BP6|Q21BP6_RHOPB

-MDVIIYHNPDCGTSRNTLALIRHAGIEPHVVEYLKTPPSRAMLQQLIARIGLGTRALLREKGTPYAELG

LDSPALSDDELLDAMLAHPILINRPIVVSPRGVKLCRPSEEVLEILP-PTAGEFHKEDGELVIDASGRRA

T

>E6VLQ1|E6VLQ1_RHOPX

-MDVVIYHNPDCGTSRNTLGLIRNAGIEPHVIEYLKCPPSRAMLEQLIVRAGLTLRQLLREKGTPYHELK

LDDPALSYEDLMAAVMAHPILINRPLVVTPKGVRLCRPSEQVIDLLP-PQRGQFTKEDGELVTDASGRRA

T

>Q2IYN1|Q2IYN1_RHOP2

KMDVVIYHNPACGTSRNTLGLIRNTGIEPHVIEYLKCPPSRAMLEQLMVRAGLTLRQLIREKGTPYHELK

LDDPELSYDQLMDAVTAHPILINRPLVVTPKGVRLCRPSETVLDLLP-PQRGEFRKENGELVVTATGRRA

T

>Q07KE6|Q07KE6_RHOP5

-MDVVIYHNPGCGTSRNTLGLIRNAGIEPHVIEYLKSPPSRALLQQLIARAGLTPRQLLRQKGTPYQALM

LDDPALTDDDLLGHMMADPILIERPLVISPKGVRLCRPSETVLDLLP-PQQGEFRKEDGELVIDAAGRPA

T

>Q133U7|Q133U7_RHOPS

-MDVVIYHNPACGTSRNTLGLIRNAGIEPHVIEYLKSPPSRALLAQLIARAGLTPRAVLREKGTPYAELK

LDNPALTDDDLLDAMIAHPILINRPLVVTPNGVRLCRPSEHVLDLLP-PQQGEFFKEDGEMVIDASGRRA

T

>B3QFQ5|B3QFQ5_RHOPT

-MDVVIYHNPACGTSRNTLGLIRNAGIEPHVIEYLKCPPTRALLKQLIARAGLTPRQALREKGTPYAELK

LDDLALSDDDLLDAMIAHPILIQRPLVVTPNGVRICRPSEAVLDLLP-PQRGEFVKEDGELVIDASGRRA

T

>D4YY27|D4YY27_SPHJU

AIDIVIYHNPECGTSRNTLAMIRNAGIEPHVIEYLKTPPNRPRLVSLIARMGISARQLLREKGTPFAELG

LADPTLSDDQLIDAMIEHPILINRPIVVSPLGVKLCRPSEEVLDLLPSAQRGAFTKEDGEQVIDAAGKRG

A

>B0UM59|B0UM59_METS4

-MDVVIYHNPACGTSRNALAMIRNAGIEPHVVESLKTPPSRAMVRALARRAGVTVRDLLREKGTPYAELG

LGDPSLSEDQLLDAIAAHPILLNRPIVVSPLGVRLCRPSEAVLDLLP-AQQGEFVKEDGERVVDESGRRA

T

>C5B448|C5B448_METEA

MMDVVIYHNPDCGTSRNTLALIRNAGIEPHVVEYLKTPPNRLLVRQLADRAGVPVRGLLREKGTPYVELN

LGDENLTDDQLLDAIAEHPILLNRPLVVSPKGVALCRPSEAVLDLLP-TQQGEFVKEDGERVVDEHGRRA

T

>C5AY23|C5AY23_METEA

-MDVVIYHNPDCGTSRNTLGLIRNAGIEPHVVKYLKTPPNRILVRQLAERAGITVRDLLREKGTPYADLG

LADPSVTDDQLLDAIEAHPILLNRPLVVTPKGVALCRPSEAVLDLLP-AQQGEFTKEDGERVVDEHGRRA

T

>B1Z6Z1|B1Z6Z1_METPB

PFDVVIYHNPACGTSRNTLAMIRNAGIEPHVIEYLKTPPTRDLVRQLARRAGLAVRDLLREKGTPYAELN

LGDASLSDDQLLDAVAAHPVLLNRPLVVTPRGVRLCRPSEAVLDLLP-PQQGAFVKEDGERVVDAHGRRA

T

>B1M3S7|B1M3S7_METRJ

PFDVVIYHNPACGTSRNTLAMIRNAGIEPHVVEYVKTPPARALLVQLLARAGLSVRDVLREKGTPFAELK

LGDPTLTEEQLLDAIEAHPILINRPLVVTPKGVRLCRPSEAVLDLLP-VQRGDFVKEDGERVVDEHGRRA

T

>B1ZBM2|B1ZBM2_METPB

-MDVVIYHNPACGTSRNTLAMIRNAGIEPHVVEYLKTPPNRALLKQMLARAGLSVRDVLREKGTPYAELG

LADPALTDEQLLDAVEAHPVLLNRPLVVSPMGVRLCRPSEAVLDLLP-DQQGEFVKEDGERVVDERGRRA

T

>C7CFR2|C7CFR2_METED

-MDVVIYHNPACSTSRNALAMIRNAGIELHVVEYLKTPPSRAVVRQMLARAGLSVRDVLREKGTPYAELG

LADPALTDDQLLDAVEAHPVLLNRPLVVSPKGVRLCRPSEVVLDLLP-PQQGEFVKEDGERVVDESGRRA

T

>B7KNK7|B7KNK7_METC4

-MDAVIYHNPACGTSRNALVMIRNAGIEPHVVEYLKTPPSRALVRQMLARAGLSVRDVLREKGTPYAELG

LADPVLTDDQLLDAVEAHPVLLNRPLVVSPKGVRLCRPSEVVLELLP-PQQGEFVKEDGERVVDEHGRRS

A

>C7CI33|C7CI33_METED

AFDVVIYHNPACGTSRNTLAMIRNAGIEPHVVEYLKTPPSRALLRQLLARAGLSVRDVLREKGTPYAELG

LDDPALTDAQLLDAIEAHPALLNRPLVASPKGVRLCRPSEAVLDLLPA-QQGAFVKEDGERVVDE-----

-

>C5B258|C5B258_METEA

AFDVVIYHNPACGTSRNTLAMIRNAGIEPHVVEYLKTPPSRALLRQLLARAGLSVRDVLREKGTPYAELG

LDDPALTDAQLLDAIEAHPALLNRPLVVSPKGVRLCRPSEAVLDLLP--QRGAFVKEDGERVVDEHGRRS

A

>A9W403|A9W403_METEP

-MDVVIYHNPDCGTSRNTLAMIRNAGIEPHVVEYLKTPPARPLLVRMLARAGLSVRDVLREKGTPFAELN

LGDPALTDAQLLDAIEAHPILINRPLVVSPKGVRLCRPSEAVLDLLP-GQQGEFVKEDGERVVDEHGRRA

T

>B8IQK8|B8IQK8_METNO

-MDVIIYHNPACGTSRNTLAMIRNAAIEPHVIEYLKTPPTRALLQQLLARAGLCVRDVLREKGTPYAELG

LGDPTLIDEQLLDAIEAHPILLNRPLVVSPKGVRLCRPSEAVLDLLP-PQQGEFVKEDGERVVDEYGRRA

T

>C7CNC7|C7CNC7_METED

-MDVVIYHNPACGTSRNTLAMIRNAGIEPHVIEYLKTPPTRALLVQLLARAGLAVRDVLREKGTPYAELG

LGDPALTDDQLLDAIEAHPILLNRPLVVTPKGVALCRPSEAVLDLLPA-QQGEFVKEDGERVVDEHGRRA

T

>A6X3V3|A6X3V3_OCHA4

-MDVIVYHNPDCGTSRNTLGLIRNSGVEPHIIEYLKTPPSRALLAQLIERASLTARSILREKGTPYADLG

LGDPELSDDALLDAMMAHPILINRPLVVTPQGVKLCRPSEAVLDLLP-PQRGEFVKEDGERIIDEHGRRA

S

>Q11LJ7|Q11LJ7_MESSB

-MDIIIYHNPGCGTSRNTLAMIRNAGVEPHIVEYLKTPPSRVMLETLIARMGIPVRALLRQKGTPYAELG

LGDASLTDEQLLDAMMAHPVLINRPIVVSPKGVRLCRPSEEVLALLP-PQRGEFVKEDGERVVDEHGRRA

T

>B6JAP4|B6JAP4_OLICO

-MDVIIYHNPECGTSRNTLGLIRNSGVEPHVIEYLKTPPSRTMLEQLIARMEISVRALLREKGTPYAELG

LDDPALSDAQVLDAMMAHPILINRPIVVTPEGVRLCRPSEDVLDLLP-PQRGAFVKEDGERVIDEHGRRA

T

>D6V0A0|D6V0A0_9BRAD

-MDVVIYHNPDCGTSRNTLAMIRNAGIEPHVIEYLKTPPSRTMLKQLIARMGISVRALLREKGTPYAELR

LDDPNLTDKQLLDAMQAHPVLMNRPIVVTPTAVRLCRPSEEVLGLLP-PQRGEFVKEDGERVVDEHGRRA

T

>B9JC58|B9JC58_AGRRK

-MDVIIYHHPDCGTSRNTVALIRNAGIEPHVIEYLKTTPSRAMLIQLIARMGIATRELLREKGTPYAELG

LGDPSLTDDQLLDAMMQHPILINRPIVVSPKGVKLCRPSEEVLDLLP-PQLGEFFKEDGERIVDEHGRRA

S

>Q3SMW9|Q3SMW9_NITWN

-MDVIIYHNPDCGTSRNTLALIRNAGIEPHIIEYLKTPPLRVLLAQLIARMGISARALLRDKGIPYTELG

LADPKLTDDQILDAMMAHPILINRPIVVSPKGVKLCRPSEDVLDLLP-PQQGELTKEDGERVIDEQGRRA

T

>B6J9W0|B6J9W0_OLICO

-MDVIIYHNPDCGTSRNTLAMIRNAGVEPHVIEYLKTPPSRALLTQLIARIGISVRALLREKGTPYAELG

LADSELTDDQLLDAMMAHPILINRPVVVSPKGVKLCRPSEEALDLLP-QQQGEFIKEDGERVIDEQGRRA

T

>A3X0G1|A3X0G1_9BRAD

-MDVIIYHNPDCGTSRNTLAMIRNAGVEPHVIEYLKTPPSRGLLRQLIARMGISARALLREKGTPYAELG

LADPALTDDQLLDAMMAHPILINRPVVVSPKGVKLCRPSEEVLDLLP-PQQGGFIKEDGERVIDEQGRRA

T

>A7IDN4|A7IDN4_XANP2

AMDVIIYHNPACGTSRNTLAMIRNAGIEPHVIEYLKTPPSRTLLAELIARAGLTVRGALREKGTPYAELG

LADEALSDDALLDAMMAHPILINRPLVVSPKGVRVCRPSELVLDLLP-PQKGAFTKEDGERVVDAAGQPA

A

>F1Z741|F1Z741_9SPHN

TVDIVIYHNPECGTSRNALAMIRNAGIEPHVVEYLKTPPSRALLESLIARAGIAPRALLREKGTPYAELG

LDNPDLDDGQILDAMMAHPILINRPLVVSPLGVKLCRPSEEVLDLIPGEQRGAFAKEDGEQVVDAQGQRV

Q

>Q0KJB8|Q0KJB8_9SPHN

TSDIVIYHNPECGTSRNALAMIRNAGLEPHVIEYLKTPPSRALLESLIHRAGMTPRALLREKGTPYGDLG

LADETLSDATLIDAMMAHPILINRPLVVSPLGVKLCRPSEEVLDLLPQRQQAAFAKEDGEPVVDGEGNRA

-

>Q1GQG0|Q1GQG0_SPHAL

MTDIVIYHNPACGTSRNTLAMIRNAGIEPHVVEYLKTPPSRALLEQLIVRAGITARELLREKGTPYAELG

LGDMALGDEALIDAMMAHPALINRPLVVSPLGVKLCRPSETVLDLIPAGQRGAFAKEDGEQVIDAAGNRG

A

>F6ILQ9|F6ILQ9_9SPHN

TTDIVIYHNPECGTSRNTLAMIRNAGIEPHVIEYLKTPPSRAMLESLIERAGLSPRELLRAKGTPYAELG

LGDESLSDTALVDAMMEHPILINRPLVVSPLGVRLCRPSEVVLDILPSPQQGAFTKEDGEQVVDAAGERV

-

>Q1GQ27|Q1GQ27_SPHAL

TSDIIIYHNPECGTSRNTLAMIRNAGIEPHVVEYLKTPPSRALLEQLIERAGLTPRALLREKGTPYAELG

LGDERLTDDALIDAMMAHPVLINRPLVVSPLGVRLCRPSEVVLDILPTPQQGAFTKEDGEQVIDEAGNRS

A

>D4Z1I3|D4Z1I3_SPHJU

MTDIVIYHNPECGTSRNALAMIRNAGMEPHVVEYLKTPPARALLVQLIERAGMSPRDLLREKGTPYAELG

LDDETLTDDALIDAMMAHPILINRPLVVTPLGVKLCRPSEVVLDILPAQQRGAFAKEDGEQVVDADGNRR

P

>F6F1X8|F6F1X8_SPHCR

MTDIVIYHNPECGTSRNSLALIRNAGIEPHVVEYLKTPPARALLVQLIDRAGMSPRDLLREKGTPYAELG

LDDRGLTDDALIDAMMAHPILINRPLVVTPLGVKLCRPSEAVLDILPAPQRGAFAKEDGEQVVDADGNRR

P

>A5V8I6|A5V8I6_SPHWW

AVDIVVYHNPECGTSRNVVGLIRNAGIEPHIVEYLKTPPARALLVQLVERAGIRPRDLLREKGTPYAELG

LDDMSLSDEALLDAMMAHPILINRPLVVSPLGVKLCRPSEAVLDILPEAQRGAFTKEDGERVVDAAGQRP

S

>F3WVD0|F3WVD0_9SPHN

AIDIIVYHNPECGTSRNVLGLIRNAGIEPHVIEYRKTPPSRALLIDLIARAGMTPRALLREKGTPYAELG

LGDPSLSDEALIDAMMAHPVLINRPLVVSPLGVKLCRPSEAVLDLLPTAQCGAFAKEDGEQVVDASGQRA

-

>Q6N7K0|Q6N7K0_RHOPA

AVDIVIYHNPECGTSRNTLAMIRNAGIEPHVIEYLKTPPSRALLVELIDRAGITPRDLLREKGTPYAELG

LGDTSLSDDALVDAMMAHPVLINRPLVVSPLGVKLCRPSEAVLDLLPEAQQGAFAKEDGEKVVDALGQRA

-

>A8I0L3|A8I0L3_AZOC5

MMDVVIYHNPECGTSRNTLAQILNAGVEPHVIEYLKCPPSRVMLRQLVDRAGMTVRALLREKGTPFAALG

LANPALTDDDLLDAMMAHPILINRPLVVSPKGVRLCRPSEEVLDLLP-PQRGAFTKEDGERVVDDAGNRT

N

>A1AYI5|A1AYI5_PARDP

-MDIILYHNPDCGTSRNTLGLIRNAGLEPHVIEYLKCPPSRELLVRLIARMGLTARDLLRQKGTPYADLG

LDDPALSEDQLLEAMMAHPILINRPIVVSPRGVRLCRPSEAVLDLLP-PQRGAFAKEDGEAVVGDDGQPT

P

>F8HGZ2|F8HGZ2_PARDS

-MDVVIYHNPDCGTSRNALGLIRNAGIEPHVIEYLKCPPSREMLVQLVARMGIPVRALLREKGTPFAELG

LGDPALTEDQLLDAMMAHPILINRPIVVSPKGVRLCRPSEAVLDLLP-PQQGAFSKEDGEVVVGADGLRT

P

>F4GS71|F4GS71_PUSST

MDSAIIYHNPKCGTSRNTLALIRHAGIEPQVVEYLQSPPDKETLANMISQAGLTVHDAIRQKEASYAELG

LDNPDLSDDQLLDAMLAHPILINRPFVSTAMGTRLCRPSELVLDILP-PVDQPFAKEDGEVVIDKDGKRA

-

>F1W3U2|F1W3U2_9BURK

-MSITIYHNPDCGTSRNTLALIRNSGVEPTVIDYLTDPPARDVLADMIQAAGLTVRQAIREKGTPYVELG

LDDPAVTDDALLVAMQAHPILINRPFVVTPRGVRLCRPSELVLEILPDAQRAAFSKEDGEAVVDASGKQR

P

>G4FWP9|G4FWP9_9GAMM

PMTATIYHNPDCGTSRNTLALIRHAGIEPTVIEYLRTPPSRKQLTKLIDEAMLKPREALREKGTPFAELG

LDEAGITDDVIIDAMLQHPILINRPFVVTPRGTRLCRPSEVVLNILP-PNLQPFTKEDGEVVVGREPLAF

P

>G0AA84|G0AA84_COLFT

-MNITIYHNPECGTSRNTLALIRNTGVEPEVIEYLKQPPSLAILTKLISDAGLAVREAIRQKGTPYTELG

LDAPGLTDAQLLDAMLAHPILINRPFVVTPNGTRLCRPSELVLDILPLPQKGPFTKEDGEVVINSEGRRT

T

>A4G2H5|A4G2H5_HERAR

--MTTIYHNPACGTSRNTLALIRNTGEEPTVIEYLKTPPSRVILIQMISDAGLSVREAIRQKGTPYAELG

LDNPQLSDDALIEAMLANPILINRPFVVADKGTRLCRPSELVLDVLSKPQQQEFTKEDGEAVINAEGQRT

K

>B2FRK6|B2FRK6_STRMK

-MNAVIYHNPKCGTSRNTLALIRHAGIEPEVIEYLVNPPSRTRLVELIAAAGLDVRGAIRQKGTPYLELG

LHYPALSEDALLDAMLANPILINRPFVQTTMGTRLCRPSEVVLDILP-AVHEPFTKEDGEVVIDQDGKRR

-

>B4SIU2|B4SIU2_STRM5

-MNAVIYHNPKCGTSRNTLALIRFAGIEPDVIDYLANPPSRARLVELITAAGLSVRDAIRQKGTPYDELG

LGDGALSDDALLDAMLAHPILINRPLVQTDRGARLCRPSEVVLEILP-PLPGPFRKEDGELVIGRAESSP

-

>E3HSU1|E3HSU1_ACHXA

MSDVTIYHNPKCGTSRNTLAMIRNAGIEPEVVLYLETPPSRAALKALFKKAGVSVREALREKGTPYQELG

LDDASLSDDALLDAIEQHPILLNRPFVSTPLGARLCRPSELVLDLLPAPQQGAFSKEDGEAVIDAQGKRQ

K

>E5U642|E5U642_ALCXX

MSDVTIYHNPKCGTSRNTLAMIRNAGIEPQVVLYLETPPSRAQLKALFKRAGITVRDALREKGTPYAELG

LDDTTLSDEALLDAIEAHPILLNRPFVSTPLGARLCRPSELVLDILPAPQQGAFTKEDGEAVIDAQGKRA

K

>D4XD76|D4XD76_9BURK

MSNVTIYHNPKCGTSRNTLALIRNAGIEPQVIEYLKTPPDRDTLVDLISRAGLSVREAMRQKEAIYKELG

LDDESLDDAALIDAMLANPILINRPIVVTPAGVRLCRPSELVLDILDAPQRGAFVKEDGEAVIDEHGRRR

-

>A7IME7|A7IME7_XANP2

HSLVTIWHNPACGTSRNTLALIRNAGIEPTIVEYLKTPPAREEVAAAIAAAGLTVRAATRQKGTPYAELG

LDDPALTDDTLLDAMMAHPILINRPFVFTPLGARLCRPSERVLDILPAPQKGPFTKEDGEVVIDAEGRRC

-

>G4M7F5|G4M7F5_9BURK

TMTVTIYHNPKCGTSRNTLALIRNAGIEPTVIEYLTHPPSREVLVALIARAGLLVREALREKGTPYAELG

LDDPTLTDDQLIDAMLTHPILINRPFVDTPKGARLCRPSELVIDILPQPQKGPFTKEDGEVIIDASGKRL

-

>Q2EEV1|Q2EEV1_RHILE

-MTVTIYHNPDCGTSRNTLEMIRNAGIQLTVIEYVKTPPSRDQLVRMIAEAGLTVREAIREKGTPYAELG

LDDPKLTDDQLLDAMLENPILINRPFVITPIGTRLTRPSELVLDILP-AHKGAFTKEDGEAVVSDAIGGS

V

>F0LDH2|F0LDH2_AGRSH

SMDVTIYHNPDCGTSRNTLALIEHAGIQPTVIEYLKTPPSREQLVKMIADAGLTVREAIREKGTPYTVLG

LGYPELTDDQLIDAMLETPILINRPFVITPMGTRLSRPSEVVLDILP-AFKGPFTKEDGEQVLDDEGKRA

-

>F6BP68|F6BP68_SINMB

-MTVTIYHNPACGTSRNTLAMIRNAGIEPTVVEYLKNPPSRAELEAMIAAAGLTVRQAIREKGTPFAELG

LGDPSRSDEELLDAMLEHPILINRPFVVAPLGTRLCRPSEVVLDILP-DHKGPFSKEDGEAVLDAGGKRV

-

>F7U9X5|F7U9X5_RHIRD

-MEITIYHNPSCGTSRNTLALIRAAGIEPKVIEYLREPPTREELATMIADAGLSVRAAIREKGTPYAELG

LDNPDLTDDQLLDAMIGTPILINRPFVVTLLGTRLARPSEVVLDVLP-DFKGPFFKEDGEQVLDQEGKRA

-

>Q7CZ50|Q7CZ50_AGRT5

-MDVTIFHNPSCGTSRNTLALIRAAGIEPTVAEYLQEPPTRERLAKIIADAGLTVREAIREKGTPYAERG

LDNPALTDDQLLDAMMETPILINRPFVITPLGTRLARPSEVVLDILP-EFKGPFFKEDGEQVLDNEGKRA

-

>C6B1U1|C6B1U1_RHILS

TIDVTIYHNPECGTSRNTLAMIQNAGIEPNVIEYLKNSPSRDQLIKVIADAGLSVREAIREKGTPYAELG

LDNPDLTDEQLLDAMLKNPILINRPFVITPLGTRLSRPSELVLEILP-EHQGAFTKEDGEKVLDAGGKRV

-

>B9K2H6|B9K2H6_AGRVS

-MNATIYHNPECGTSRNTLAMIRNAGIEPLVIEYLSTPPTRGELARMIADAGLSVREAIREKGTPYAQLG

LDNPDLTDAQLLDAMLEHPILINRPFVITPLGTRLARPSERVLEILP-NHQGAFTKEDGERVLDSEGKRV

-

>B5ZT41|B5ZT41_RHILW

-MNVTIYHNPACGTSRNTLAMIRNAGIEPTVIEYVKTPPSREELARMIADAGLTVREAIRQKDTPYAELG

LDNPDLTDDQLLQAMLAQPILINRPFVVTPLGTRLSRPSELVLEILP-EHKGAFTKEDGEKVLDAEGKRV

-

>Q2K777|Q2K777_RHIEC

-MNVTIYHNPACGTSRNTLAMIRNAGIEPAIIDYVSTPPSRAELVKMIADAGLTVRQALREKDTPYAELG

LDNPDLTDDQLLDAMLAHPILINRPFVITPLGTRLSRPSELVLEILP-EHKGAFTKEDGEKVLDAEGKRV

-

>B3PRL5|B3PRL5_RHIE6

-MNVIIYHNPACGTSRNTLAMIRNAGIEPTVIEYVNTPPSRTELMKMIADAGLTVRQALREKDTPYVELG

LGNPDLSDDQLLDAMLAQPILINRPFVITPLGTRLSRPSELVLEILP-EHKRAFTKEDGEKVLDAEGKRG

-

>C3KMK9|C3KMK9_RHISN

-MNATIYHNPACGTSRNTLAMIRNAGIEPTIIEYLDQAPSRDELARMIADAGLTVRQAIREKGTPYAELG

LDDPGLADEQLIDAMLKNPILINRPFVVTPLGTRLCRPSEVVLDILP-EHKGEFTKEDGERVLDADGKRV

-

>F7U902|F7U902_RHIRD

-MDATIYHNPACGTSRNTLEMIRAAGIEPTVIEYLQSPPSRDELARMISDAGLSVRQAIREKGTPYGELG

LDDPKLTDDLLLDAMLKDPILINRPFVVTPLGTRLARPSEVVLDILP-GFKGAFFKEDGEQVLDQEGKRV

-

>F5JAF4|F5JAF4_9RHIZ

-MDATIYHNPACGTSRNTLEMIRNAGIEPTVIEYLKTPPSRDQLVKMIADAGLTVRQAIREKGTPYAQLG

LDNPALSDDQLLDAMLKDPILINRPLVVTPLGTRLARPSEVVLDILP-DDKGAFTKEDGEQVLDAEGKRV

-

>B2AJK9|B2AJK9_CUPTR

-MSVTIYHNPACGTSRNALAMIRNAGVEPNVIEYLNTPPDRQTLQQMIRLAGLTVRQAIREKGTPYAELG

LSDPALTDDQLLDAMLAQPILINRPFVITELGVRLCRPSEVVLDILPSPQQAAFTKEDGEVVIDEQGRRP

-

>Q1LRL1|Q1LRL1_RALME

-MTVTIYHNPACGTSRNTLAMIRNAGIEPIVVEYLNMPPDRQTLQSMIRAAGLSVREAIREKGTPYAELG

LGDPALTDDQLLDAMLAHPILINRPFVITELGVRLCRPSEVVLDILPAPQQGAFTKEDGEAVIDTQGRRR

-

>Q471P2|Q471P2_CUPPJ

-MSVTIYHNPACGTSRNTLAMIRNAGIEPTVIEYLDTPPDRQTLLALIRDAGLSVREAIREKGTPYAELG

LADPALTDNQLLDAMLARPVLINRPFVVTELGVRLCRPSEVVLDILPAPQQGAFSKEDGEAVIDAQGRRR

-

>Q2W690|Q2W690_MAGSA

SSEITIYHNPACGTSRNTLGLIRNSGEEPRVVEYLRTPPTRDELVGLIARMGIPVRDLLRKKGTPYADLG

LDNMALTDDQLIDAMMAHPILINRPIVVTPLGVKLCRPSELVLDILPQPQKGAFTKEDGEQVVDGQGKRL

-

>B2TAG2|B2TAG2_BURPP

-MSVTIYHNPDCGTSRNTLAMIRNAGIEPEIIEYLKSPPDRDTLKNLIERAGLTVRDVLREKGTPYDELG

LADLSLSDEQLLDAMLTHPILVNRPIVVTPLGVRLCRPSEIVLDILPAGQQRAFAKEDGEQVIDSAGRRR

-

>D5NL87|D5NL87_9BURK

-MSVTIYHNPECGTSRNTLAMIRNAGIEPEIIEYLKTPPDRETLIGLISRAGLTVRAVLREKGTPYAELG

LNDTSLSDGALLDAMMEHPILINRPFVVTPLGVRLCRPSEVVLDILPTGQKGSFSKEDGEQVVDSKGRRL

-

>Q13JU5|Q13JU5_BURXL

-MSVTIYHNPACGTSRNTLAMIRNAGIEPEIIEYLNTPPDRETLIGLIARAGLTVRAVLREKGTPYAELG

LNDTSLSDDALLDAMMEHPILINRPFVVTPLGVRLCRPSEVVLDILPVGQKSSFRKEDGEQVVDSEGRRP

-

>F3S483|F3S483_9PROT

-MTITIYHNPACGTSRNVLALIRNSGEEPRIIEYLKTPPNRAELVDLIALMGVPVRSVLREKDTPFHELG

LDNPALSDDALIDAIIAHPILMNRPIVVTPLGVALCRPSETVLDILPNPQQGAFVKVDNEKTVDESGKRV

-

>C7JIU6|C7JIU6_ACEP3

-MTIMIYHNPACGTSRNVLALIRNAGIEPTTIEYLKTPPGRAELVDLIARMGVPVRSVLREKGTPFHELG

LDNPALSDDALIDAMMAHPILINRPIVVTPLGAALCRPSETVLDILPNPQRGAFVKEDGEKIVDESGKRI

-

>F3S2S4|F3S2S4_9PROT

-MTITIYHNPACGTSRNVLALIRNSGEEPRIIEYLKTPPSRAELVDLIARMGLPVRSVLREKGTPFHELG

LDNPTLTDDTLIDAMIAHPILMNRPIVITPLGVALCRPSEAALEILPNPQRGAFVKEDGEKIVDESGKRS

-

>G2I843|G2I843_GLUXY

-MTITIYHNPACGTSRNVLALIRNSGEAPCIIEYLKTPPSRAELVSLIARMGVPVRSVLREKGTPFHELG

LDNPALTDDALIDAMIAHPILMNRPIVVTPLGAALCRPSERVLDILPNPQRGAFIKEDGEKIIDKSGKRV

-

>G2I8I9|G2I8I9_GLUXY

-MTITIYHNPACGTSRNVLALIRNSGEEPRIIEYLKTPPSREELVSLIARMGVPVRSVLREKGTPFHELG

LDNPALSDDALIDAMIAHPILMNRPIVVTPLGAALCRPSEAVLDILPNPQRGAFVKEDGEKIVDESGKRS

-

>A5G2C0|A5G2C0_ACICJ

-MTVTIYHNPACGTSRNVLGLIRNAGLEPVVIEYLKTPPSREELRGLIARMGVPVRAVLREKGTPYAELG

LDDETLTDNALLDAMMAHPILINRPIVVTERAVRLCRPSETVLDILPTPQQGAFTKEDGQQVVDDAGRRP

-

>D5RQR7|D5RQR7_9PROT

MSEVTIYHNPACGTSRNTLALIRNSGEDPEVIEYLKTPPTREVLADLIRRMGVPVRDALRQKGTPYEELG

LGDLSLTDDQLLDVMMAHPILINRPIVVTPLGVRLCRPSEAVLDILPNPQRGAYAKEDGEKVVDDQGRRI

-

>D3NYB7|D3NYB7_AZOS1

MTSVTIYHNPDCGTSRNTLALIRNSGVEPTVIEYLKTPPSRDELADLIGRMGVPVRAVLREKGTPYAEPG

LADPSLSDDQLLDAMMAHPILINRPIVVTPLGVRLCRPSEAVLDILPDAQRGAFAKEDGEQVVDAAGHRG

K

>F0J7M6|F0J7M6_ACIMA

-MSVTIYHNLACGTSRNVLALIRNAGIEPTIIEYLKTPPSRETLIDLIARTAMPVRSVLREKDTPYAELG

LADPALPDHALLDAMMAHPILINRPIVVTPLGVKLCRPSETVLDILPAPQRSAFTKEDGEAVVDPAGRRR

-

>F0J369|F0J369_ACIMA

-MSVIIYHNPDCGTSRNVLALIRNAGIEPMIIEYLKTPPSRETLIDLIGRMGVLVRSVLREKDTPYAQLG

LANPALPDAALLDAMMAHPILINRPIVVTPLGVKLCRPSETVLDILPAPQRAGFAKEDGEAVVDSAGRRR

-

>F7SAF8|F7SAF8_9PROT

-MTVTIYHNPDCGTSRNVLALIRNAGIEPTIIEYLKTPPSRETLIDLIARMGVPVRTVLREKDTPYAELG

LADPILPDAVLLDAMMAHPILINRPIVVTPLGVKLCRPSETVLDILPAPQRAAFAKEDGEAVVDSAGQRR

-

>A3JJC5|A3JJC5_9ALTE

-MKSYIYHNPECGTSRNTLAVMKASGEAPEVVEYLKTPPTREQLVTLLSMMQITPRDLLRRKGTPFDELR

LDNPALNDAQVIDTMIAYPILINRPIVVTDKGARLCRPAERVLDMLESP-IAHFTKEDGEVIYQAGAAQ-

-

>Q6WB66|Q6WB66_ALCFA

-MKPVIYHNPECGTSRNTLEMIKASGEEPEVIEYLVNPPSRERLLELIDLMGISPRELLRRKGTPYDELN

LDDPGLTDAQIIDAMMSHPILINRPIVVTEKGVKLCRPSELVLELLEHP-ATTFKKEDGETVNFPAKHM-

-

>F7SLY4|F7SLY4_9GAMM

-MSVTIYHNPNCGTSRNTLAMIKASGEDPEVIHYLATPPSRESLVALLAMMKISPRELLRQKGTPYDELK

LDDPSLDDDQLIDAMIAHPILINRPIVITPKGARLCRPSERVLELLEQP-IALFTKEDGETIYFPAS---

-

>A5WD87|A5WD87_PSYWF

KMTATIFHNPKCSTSRNALAIMQASGETPEVIEYLNTPPSREYLVNLLNQMHMAPRELMRSKEAVFTELG

LDNPEVSDDQLIDAMISHPILINRPIVVTDKGAVLCRPLERVFEVLATP-VETFTKENGEIITYPAL---

-

>B8PYA9|B8PYA9_9BACT

-MKPLIFHNPNCGTSRNTLAIMKASGEQPEVVEYLKNPPSRDELVELLAKMNISPRELLRSKETINDELG

LDNPELSDDQIIDAMIAHPILINRPIVVTDKGAALCRPSERVFELLENP-VSSFTKEDGEVLHHGKD---

-

>Q1QAQ9|Q1QAQ9_PSYCK

-MTSLIFHNPKCGTSRNTLAIMQASGDNPEVIEYLKTPPSREYLVELLAKMALSPRELLRSKESINDELG

LDNPALSDDEIIDAMIDHPILINRPIVVTDKGAALCRPSERVFELLENP-VSSFTKEDGEVIYHGK----

-

>Q4FT52|Q4FT52_PSYA2

-MTSLIFHNPKCGTSRNTLAIMQASGENPEVVEYLKTPPSRDYLVDLLAKIALSPRELLRSKESVNDELG

LDNPALSDDEIIDAMIAHPILINRPIVITDKGAALCRPSERVFALLANP-VSSFTKEDGEIIYNAKDK--

-

>F8QRD6|F8QRD6_9BURK

STDTTIFHNPACGTSRNVLAMIRHAGIEPTVIEYLQTPPSRERLKELIAAMGIPVRALLRQKGTPYDELG

LQEHTLSDEHLLNAMLAHPILINRPIVVTPLGTRLCRPSEAVLDLLP-ALSGPFAKEDGELVIDAQGKRV

S

>F2LF46|F2LF46_BURGS

MSEVTIYHNPDCGTSRNTLAMIRHAGIEPRIVHYLLTPPDRDTLLGLIAAMGIAPRELLRRKDTPHEALG

LDDPAWRDEQLIDFMLAHPILINRPIVVTPRGTRLCRPSETVLELLPAGPRGPFAKEDGDVVIDAEGRRR

P

>F7RNM8|F7RNM8_9GAMM

HSSIKIYHNPACGTSRNTLGLIRNTGVEPTIIFYLETPPNRQTLLQLIADMGISVRSLLRQNVAPFEALG

LGEDQFSADELIDFMLEYPILINRPIVVTPLGTRLCRPSEQVLDILPNPQLGAFTKEDGEVVIDTNGQRK

K

>A0KXQ5|A0KXQ5_SHESA

HSSIRIYHNPACGTSRNTLGLIRNTGVEPTIILYLETPPSRQTLLQLIAEMGISVRSLLRQNVEPFAALG

LSEDKFSDSELIDFMLEYPILINRPIVVTPLGTRLCRPSEQVLDILPNPQQGAFTKEDGEVVIDANGQRA

Q

>A1RM72|A1RM72_SHESW

HNSIKIYHNPACGTSRNTLGLIRNTGIEPIIILYLETPPNRQTLLQLIADMGISVRSLLRQNVEPYTALG

LSEDKFSDSELIDFMLEYPILINRPIVVTPLGTRLCRPSEQVLDILPKPQLGAFTKEDGEVVIDANGQRK

K

>D4F4I3|D4F4I3_EDWTA

MNTITIYHNPACGTSRNTLAMIRNSGVEPTIIHYLETPPSRAELIGLIAALGISVRALLRQNVEPYETLG

LADERLSDAQLIDAMLSHPILINRPIVVTPLGARLCRPSERVLEILPTAQQGAFNKEDGEPVVDETGKR-

-

>D2C295|D2C295_DICD5

MTEITIYHNPACGTSRNTLALIRNSGVEPTVIHYLDTPPSKAELEELIRAMGITPRMLLRQNVEPYTALG

LADDSFSDEQLIAFMLAHPILINRPIVVTPLGTRLCRPSEVVLDILPDAQQGEFIKEDGERVIDASGKRK

S

>E0SL73|E0SL73_DICD3

MTEITIYHNPACGTSRNTLALIRNSGVEPTIIHYLETPPSRAELIGLIIAMGISPRTLLRQNVEPYTELG

LAEDKFSDEQLITFMLAYPILINRPIVVTPLGTRLCRPSEVVLDILPDAQQGAFVKEDGEHIVDKLGKRK

L

>C6CL53|C6CL53_DICZE

MTEITIYHNPACGTSRNTLALIRNSGVEPNVIHYLETPPSRAELEGLISAMGITPRMLLRQNVEPYSELG

LAEDNFSDEQLIAFMLTHPILINRPIVVTPLGTRLCRPSEVVLDILPDAQQGVFIKEDGEQVVDKFGKRK

L

>A7MRG2|A7MRG2_ENTS8

MTPITIYHNPACGTSRNTLGLIRNSGVEPAIILYLETPPPRDELKTLISDMGISVRALLRKNVEPYQALG

LDDEKIGDEALLDAMLAHPILINRPIVVTPLGTRLCRPSEEVLAILPDPQQGPFTKEDGEVLIPQGR---

-

>C9Y5E1|C9Y5E1_CROTZ

MTPITIYHNPACGTSRNTLGLIRNSGVEPTIILYLDTPPTRDELKTLITEMGISVRALLRKNVEPYQALG

LDDEKISDAQLLDAMLAHPILINRPIVVTPLGTRLCRPSEAVLALLPDPQQGPFTKEDGEIVIPPAP---

-

>E6WG50|E6WG50_PANSA

MTDITLYHNPACGTSRNTLALIRNSGVEPVIILYLETPPDRDKLKELIGAMGISVRALLRTNVDPYQVLG

LVDEHFSDDQLLDAMLAHPILINRPIVVTPLGTRLCRPSEVVLDILPDPQQGEFSKEDGEKVIDKDGNRV

-

>F5RY16|F5RY16_9ENTR

MTDITIYHNPACGTSRNTLALIQNSGVEPTVILYLETPPDRDKLKELISAMGISVRALLRKNVEPYEQLG

LTEDRFTDEELIEAMLSHPILINRPIVVTPLGTRLCRPSEVVLDILPNPQKGEFIKEDGEKVVGKDGKRI

-

>C4LAC5|C4LAC5_TOLAT

MSEVTIYHNPKCGTSRNTLEMIRNAGIEPNVILYLETPPTRDTLVKLIADMGISVREVLRQKCDPYVELG

LDDPKWTDEQLLDFMMQHPILINRPIVVTELGTKLCRPSELVLDILPAAQQGAFTKEDGEAVVDAEGKRI

-

>C4SAD1|C4SAD1_YERMO

MSAITIYHNPACGTSRNTLELIRNSGNEPTVILYLETPPSRDELVTLILNMGITARALLRKNVEPYEALG

LSDETFTDDELIDAMLAHPMLINRPIVVTSVGTKLCRPSEAVLDILPDPQQGPFSKEDGDIVIDQQGRRM

K

>C4UXN4|C4UXN4_YERRO

MSNITIYHNPACGTSRNTLALIRNTGTEPEVILYLDTPPSRAVLVTLIADMGIRTRALLRTNVEPYAQLG

LAEERFSDEQLIDFMLQYPILINRPIVITPLGTRLCRPSETVLAILPDAQRGVFTKEDGQQVVEAPGKQE

K

>D4GEF4|D4GEF4_PANAM

MTDITIYHNPACGTSRNTLALIRNGGVEPTVILYLETPPSRDELKKRIADMGIRVRDLLRKNTEPYEHLG

LAEDRFNDDELLDAMLAHPVLINRPIVVTPLGTRLCRPSEAVLGILPEPQKGAFTKEDGEKVIDEYGNRG

H

>E0LTM8|E0LTM8_9ENTR

MTDITIYHNPACGTSRNTLAFIRNSGVEPLVILYLETPPTRDELQKLIADMGISVRELLRKNTEPYEQLG

LAEDKFSDDELLDAMLAHPVLINRPVVVTPLGTKLCRPSEVVLEILPDPQQGPFTKEEGGS--------N

Q

>C6CCN8|C6CCN8_DICDC

MTAITIYHNPACGTSRNTLALIRNSGVEPTVIHYLETPPSRDQLIKLIADMGIPARELLRTNVDPYQQLG

LAENNFTDDELIDAMLEHPILINRPVVVTPLGTKLCRPSEVVLDILPDPQQGSFIKEDGEAVIDEQGQRK

R

>D8MJB0|D8MJB0_ERWBE

MTDITIYHNPACGTSRNTLALIRNSGTEPTVILYLETPPSRDELKKLIADMGISVRTLLRKNTDPYGQLG

LAEDRFTDDELLDAMLAHPILINRPVVVTPLGTKLCRPSEVVLDILPDPQQGAFIKEDGEAIIDEQGNRS

R

>A0KJ12|A0KJ12_AERHH

DAHITIYHNPACGTSRNTLALIRNSGVEPTVIHYLDTPPSRDTLLALIAAMGMPVRNLLRQNVPPYEALG

LAENQFSDDELVDAMLAHPILINRPIVVTPLGTKLCRPSEVVLEILPSPQQGAFSKEDGEQVVDAQGRRI

G

>A4SIL7|A4SIL7_AERS4

NNQITIYHNPACGTSRNTLELIRNSGVEPTVVHYLETPPSRETLVALIGAMGMPVRDLLRKNVPPYEALE

LAENRFSDGDLIDAMLAHPILINRPIVVTPLGTRLCRPSEVVLDILPSPQQGAFTKEDGEQVVDAAGNRI

K

>F4D7A1|F4D7A1_AERVB

MSAITIYHNPECGTSRNTLELIRNSGVEPTVIHYLETPPSRDQLVALIAAMAMPVRDLLRKNVPPYEALG

LAEDRFSDDELIDAMLAHPILINRPIVVTPLGTKLCRPSEVVLDILPDQQKGAFAKEDGEQVVDASGNRL

K

>B5XUH4|B5XUH4_KLEP3

-MSITIYHNPDCGTSRNTLALIRNSGAEPTIIYYLETPPSRDELRQLIAAMAIPVRALLRKNVEPYDALG

LAEDRFTDDQLIDFMLQHPILINRPIVTTPRGTRLCRPSEVVLEILTASQKGAFVKEDGEPVIDAAGQRV

K

>D2ZAA2|D2ZAA2_9ENTR

MSHITLYHNPDCGTSRNTLEMIRNSGNEPEIVLYLDTPPSRETLCKLIADMGISVRDLLRTNVEPYAQLG

LADGQFSDEQLIDFMLQYPILINRPIVVTPLGTRLCRPSERVLDILPDPQRAAFTKEDGEAVVDASGNKR

G

>G0B6L0|G0B6L0_SERSA

MSQITIYHNPACGTSRNTLALIRNSGVEPAVILYLETPPSRAQLVKLIADMGISARELLRKNVEPYQQLG

LAGTQWSDAQLIDFMLQQPILINRPIVVTPLGTRLCRPSEAVLDILPDAQQGPFSKEDGEPVIDALGQRT

G

>D4E4P5|D4E4P5_SEROD

MSNITIYHNPDCGTSRNTLALIRNSGVEPTVILYLETPPSRDELIGLISEMGISVRDLLRKNVEPYQQLD

LAQDSWSDQRLIDCMLQHPILINRPIVVTPLGTRLCRPSEAVLDILPDAQLGPFSKEDGEEVIDAQGRRV

K

>A8GEF0|A8GEF0_SERP5

MSNIVIYHNPACGTSRNTLEMIRNSGNEPTIIYYLDTPPSHDELVHLIADMGISVRALLRKNVEPYEQLG

LDNGVLTDEQLIEFMLQYPILINRPIVVTPMGTRLCRPSEVVLEILPDPQQTAFTNEDGEKVTNQAGNRI

K

>C4S061|C4S061_YERBE

MSNITIYHNPDCGTSRNTLELIRNSGVEPTVIYYLETPPSRDKLVKLIANMGISVRALLRKNVEPYEQLR

LAENPFTDDQLIDFMLQYPILINRPIVVTPQGTRLCRPSEIVLEILPEPQKGVFTKEDGERVIDAFGKQY

K

>E8Y0K7|E8Y0K7_RAHSY

MSNITIYHNPACGTSRNTLELIRNSGAEPTVILYLETPPAREELVKLIADMGIPVQALLRKNVEPYEQLK

LADNHFSDDQLIDFMLQYPILINRPVVVTPLGTRLCRPSEEVLAILPDAQRGAFTKEDGEAVIDENGKRT

K

>E8PS84|E8PS84_YERPE

MSNITIYHNPACGTSRNTLEMIRNSGTEPTIIHYLETPPSHEELVKLIADMGMTVRALLRKNVEPYEQLG

LADDKFTDEQLLGFMLQHPILINRPVVVSPLGTRLCRPSEVVLDILPEPQKSAFTKEDGERVTDESGKRL

R

>G0E3Q8|G0E3Q8_ENTAK

MSNITIYHNPACGTSRNTLEMIRNSGNEPTVIYYLETPPTHDELVKLIADMGIAVRTLLRENVEPYAQLG

LAEDKFTDEQLIEFMLAHPILINRPIVVTERGTRLCRPSEVVLDILPDPQQGAFSKEDGEKVVDERGKRI

P

>D5CDR8|D5CDR8_ENTCC

MSAITIYHNPACGTSRNTLELIRNSGVEPTVILYLETPPARAELVTLIADMGICVRDLLRKNVEPYEQLG

LAEDKWSDDELIDFMLQHPILINRPVVVTPLGTRLCRPSEVVLDILPDAQKGAFSKEDGEQVIDAQGQRV

K

>C4SZ53|C4SZ53_YERIN

MSNVTIYHNPACGTSRNTLELIRNSGTEPTVIQYLETPPSREVLVKLIADMGITVRALLRKNVEPYEQLG

LAEGTFSDDQLIDFMLQHPILINRPIVVTSLGTRLCRPSEVVLEILPDAQRGSFNKEDGEPVIDEQGKRE

K

>A4WGP4|A4WGP4_ENT38

MSNITIYHNPACGTSRNTLEMIRNSGNEPTIIYYLDTPPTRDELIKLISDMGITVRGLLRKNVEPYEQLG

LAEDKFTDGQLLEFMLQHPVLINRPIVVTPAGTRLCRPSEVVLEIIPEPQQGAFTKEDGEKVIDETGKRV

N

>D6DN19|D6DN19_ENTCL

MSNITIYHNPACGTSRNTLEMIRNSGNEPTIIYYLDTPPTHDELIKLISDIGITVRALLRKNVEPYEQLG

LADDRFTDEQLIELMLQHPILINRPIVVTPVGTRLCRPSEVVLEIIPEPQQGAFTKEDGEKVIDEAGKRV

K

>Q6D6S8|Q6D6S8_ERWCT

MSNITLYHNPACGTSRNTLEMIRNSGNEPTIIYYLDTPPTRDELIKLISDMGMTVRALLRKNVEPYEQLG

LDEDIFSDEKLIDFMLKHPILINRPIVVTPLGTRLCRPSEIVLDILPEGQKATFTKEDGEKVIDEDGNRV

K

>B4TP33|B4TP33_SALSV

MSNITIYHNPACGTSRNTLEMIRNSGNEPTIIYYLNTPPTRDELIKLISDMGITVRALLRKNVEPYEHLG

LGKEKFSDEQLIDFMLQHPILINRPVVVTPLGTRLCRPSEIVLDILPEGQKGAFTKEDGEKVIDETGKRV

K

>D4BBB5|D4BBB5_9ENTR

MSNITIYHNPACGTSRNTLEMIRNSGNEPTIIYYLDTPPTRDELIKLISDMGISVRALLRKNVEPYEQLG

LGEDKFSDEQLIDFMLQHPILINRPIVVTPLGTRLCRPSEGVLDILPEGQQGSFTKEDGEKVIDETGKRV

K

>E7BAV4|E7BAV4_YERE1

MSNITIYHNPTCGTSRNTLEMIRNSSNEPTVIYYLETPPTHDELVKLIADMGITVRALLRKNVEPYEELG

LAEGTFSDEQLIGFMLEHPILINRPIVVTPLGTRLCRPSEVVLDILPEPQQGAFTKEDGEKITSQVNDSN

N

>P74984|ARSC_YEREN

MSNITIYHNPTCGTSRNTLEMIRNSGNEPTVIYYLETPPTHDELVKLIADMGITVRALLRKNVEPYEELG

LAEGTFSDEQLIGFMLEHPILINRPIVVTPLGTRLCRPSEVVLDILPEPQQGAFTKEDGEKITDESGKRL

K

>A1JPJ3|A1JPJ3_YERE8

MNNITLYHNPACGTSRNTLEMIRNSGAEPTIIYYLETPPSRDVLMKLIADMGISARALLRKNVEPYETLG

LAKDCFTDNQLIDFMLQYPILINRPIVVTSLGTRLCRPSEVVLEILSDDQKGAFTKEDGEKVIDDAGKRL

K

>F5RTP9|F5RTP9_9ENTR

MSHITIYHNPACGTSRNTLEMIRNSGTEPEIILYLENPPSRDQLTTLIADMGISVCDLLRKNVEPYEQLG

LAQGPFTDDQLIDFMLQYPILINRPIVVTPLGTRLCRPSEVVLDILPDAQKGAFTKEDGEVVVDASGKKS

R

>C4TVU7|C4TVU7_YERKR

MSNITIYHNPACGTSRNTLEMICNSGAEPTVIHYLETPPSRDELVKLIADMGITVRALLRKNVEPYETLE

LAEDRFTDDQMIDFMLQHPILINRPIVVTPLGTRLCRPSEEVLDILPEVQKGAFTKEDGERVVDDQGNRL

K

>C4UJN5|C4UJN5_YERRU

MSNITIYHNPACGTSRNTLDMIRNSGEEPTVIFYLETPPGRDELVKLIADMGITVRALLRKNVEPYEQYG

LGEDKFTDDQLIDFMLQHPILINRPIVVTPLGTRLCRPSEMVLDILP-AQKRAFTKEDGEKVVDDAGKRK

-

>D5CGA6|D5CGA6_ENTCC

MTNITIYHNPACGTSRNTLEMIRNSGTEPEIILYLENPPSRDELIKLIADMGISVRALLRTNVEPYEQLG

LAEETFTDDQLIGFMLQHPILINRPIVVTPLGTRLCRPSEVVLDILPDAQKGAFTKEDGEAVVDASGKKT

Q

>D2U6Y3|D2U6Y3_9BACT

TELGSTSNGRQCAGSRNTLEMIRNNGTEPTVIHYLETPPSRAELVKLIADMGITVRALLRKNVEPFEALG

LAEDRFTDEQLIDFMLQHPVLINRPIVVTPQGTRLCRPSEVVLDILPDAQKSAFTKENGEKV--------

-

>C1IVL4|C1IVL4_ENTCL

MSNITIYHNPACGTSRNTLEMIRNSGTEPTVIHYLETPPSRAELVKLIADMGITVRALLRKNVEPFEALG

LAEDRFTDEQLIDFMLQHPVLTNRPIVVTPLGTRLCRPSEVVLDILPDAQKSAFTKEDGEKVVDEKGNRL

N

>D2U6Y6|D2U6Y6_9BACT

TPSLVPSSDPLVTASRNTLEMIRNSGTEPTVIHYLETPPSRAELVKLIADMGITVRALLRKNVEPFEALG

LAEDRFTDEQLIDFMLQHPVLINRPIVVTPQGTRLCRPSEVVLDILPDAQKSAFTKEDGEKV--------

-

>A9N3V8|A9N3V8_SALPB

MSNITIYHNPACGTSRNTLEMIRNSGIEPTVILYLENPPSRDELVKLIADMDISVRALLRKNVEPYEELG

LAEDKFTDDQLIDFMLQHPILINRPIVVSPLGTRLCRPSEVVLDILPDAQRGEFTKEDGEKVIDAHGQRI

K

>D2NC86|D2NC86_ECOS5

MSNITIYHNPACGTSRNALEMIRNSGTEPTIIYYLETPPTRDELVKLIADMGITVRALLRKNVEPYEELG

LAEDKFTHDQLIDFMLQHPILINRPIVVTPQGTRLCRPSEVVLEILPDAQKGAFTKEDGEKVVDEAGNRL

K

>E2X8I9|E2X8I9_SHIDY

MSNITIYHNPACGTSRNTLEMIRNSGTEPTIIHYLETPPTRDQLVKLIADMRLTVRALLRKNVEPYEELD

LAEDKFTDDRLIDFMLQHPILINRPIVVTPLGTRLCRPSEVVLEILPDAQKGAFSKEDGEKVVDKAGKRL

K

>A1JPZ6|A1JPZ6_YERE8

MSNITIYHNPACGTSRNTLEMIRNSGTEPTVILYLETPPSRDELVKLIADMGIRVRSLLRKNVEPYEELG

LAEDKFTDDQLIYFMLQHPILINRPIVVTPLGTRLCRPSEVVLDILTDAQKGAFAKEDGEKVVDETGKRL

K

>E3G6Q3|E3G6Q3_ENTCS

MSNITIYHNPACGTSRNTLEMIRNSGVEPTVIHYLENPPSRDELVRLIADMGITVRALLRKNVEPYEVLG

LAEDKFTDAQLIDFMLQHPVLINRPIVVTPLGTRLCRPSEVVLDILPDAQKGAFAKEDGEQVVDNAGKRL

K

>C1MF04|C1MF04_9ENTR

MSNITIYHNPACGTSRNTLEMIRNSGNEPTVILYLETPPSRDELVKLIADMGISVRALLRKNVEPYEELG

LAEDKFTDDQLIDFMLQHPILINRPIVVTPLGTKLCRPSEVVLDILPDAQKAAFTKEDGEKVVDDAGKRL

K

>P08692|ARSC1_ECOLX

MSNITIYHNPACGTSRNTLEMIRNSGTEPTIILYLENPPSRDELVKLIADMGISVRALLRKNVEPYEQLG

LAEDKFTDDQLIDFMLQHPILINRPIVVTPLGTRLCRPSEVVLDILQDAQKGAFTKEDGEKVVDEAGKRL

K

>Q9KJI1|Q9KJI1_KLEOX

MSNITIYHNPACGTSRNTLEMIRNSGNEPTVIHYLENPPSRDELVKLIADMGISVRALLRKNVEPYEELG

LAEDKFTDGELIDFMLQYPILINRPIVVTPLGTRLCRPSEVVLDILPDAQKGAFAKEDGEKVVDETR---

-

>C9YA78|C9YA78_9BURK

-MSITIFHNPACGTSRNTLAMIRNSGVEPTVVEYLKTPPTKARLQELLAAMGTGPRELLREKGTPYAELD

LANEKWTDDELLDFMLAHPILINRPVVETPLGTRLCRPSELVLDILPQAQQAAFTKEDGEAVVNDKGQR-

-

>A2W7E8|A2W7E8_9BURK

MTDVTIYHNPDCGTSRNTLAMIRNAGIEPRIVRYLDTPPGRDELVALIAAIGVPVRDVLRRNGTCYDALD

LGNPKWSDAELIDFMLAHPILINRPIVVTPLGTRLCRPSETVLEILPNPQQRPFAKEDGEVVIDTHGRH-

-

>A9AS15|A9AS15_BURM1

MTDITIYHNPNCGTSRNTLAMIRNSGAEPRVVHYLETPPSRGELVDLIAAMGVPVRDVLRRKGTPYDELD

LDNAKWSDADLIGFMLDHPILINRPIVVTPLGTRLCRPSETVLDLLPNPQTGPFTKEDGEVVIDAEGKR-

-

>C5TC21|C5TC21_ACIDE

MSTITIYHNPKCGTSRNTLAMIRNSGVEPDIIEYLKTPPSRETLVALIAAMGTPVIDVVRSKEAVFTELN

LGAPGVTDAQLVDAMLAHPVLINRPIVVTPLGTRLCRPSEAVLDILPQPQQGAFTKEDGEVVIDAKGRR-

-

>A1VQG1|A1VQG1_POLNA

MSFVTIYHNPKCGTSRNVLALIRNTGVEPEVIEYLKTPPSRETLVELIARMAVPVRDVMRAKEALYSELA

LGNPALGDDALIDAMLAHPILINRPIVVTTLGTRLCRPSEAVLDILPLPQRAAFAKEDGEPVVNAQGERA

G

>Q12BE6|Q12BE6_POLSJ

MSSVTIYHNPQCGTSRNVLALIRNTGVEPEVIEYLKTPPTREKLLELIAQMAVPVREVIRQKGTPYGELG

LDDPALPDEALIDAMLAHPILINRPIVVTPLATRLCRPSEAVLDILPLPQRGAFAKEDGEQVVNERGERA

G

>E2MLV6|E2MLV6_PSEUB

MSQITIYHNPECGTSRNTLELIRNSGEEPTVIEYLKNPPDRTTLVRLIEDMGIGVRALLRIKGTPYEELG

LGDASLTNEQLIDAMMAHPILINRPIVVTPLGTRLCRPSEAVLDLLPQEQRGSFVKEDGQVVIDEHGRR-

-

>C5CVF8|C5CVF8_VARPS

RPSITIFHNPACGTSRNTLAMIRNSGEEPHVIEYLKTPPTRETLRELLAAMAVAPRALLRRKGTPYDELG

LDDPKWSDEQLLDFMLAHPILINRPIVVTPIGTRLCRPSEAVLDILPSPQKGPFTKEDGEPVIDAEGRRE

R

>Q7W721|Q7W721_BORPA

MTSITIYHNPACATSRNVLGLIRNSGEEPAIIEYLKTPPDAATLRSLIVAMGMPVRDLLRQKGTPYDELG

LSDPKWTDEQLIDLMLQYPILINRPIVTTPLGTRLCRPSETVLDLLSRPQRGAFDKEDGQPVIDADGNR-

-

>B9BUI7|B9BUI7_9BURK

MSTITIYHNPDCGTSRNVLALIRNSGEEPTVIEYLKTPPAREILVKLLADAGLSVRAVLREKGTPYAQLG

LDDPKWTDEQLLDFIGQHPVLMNRPIVVTPLGTRLCRPSETVLDILPQSQRGAFCKEDGEPVVDVRGQR-

-

>A9IET3|A9IET3_BORPD

MSNITIYHNPACGTSRNTLALIRNSGEDPTVIEYLKTPPTRATLVKLLADAGLSVRDVLREKGTPYAELG

LGDPKWTDEQLLDFIEQHPILMNRPIVVTPMGTRLCRPSEIVLDILPQPQRGAFTKEDGEPVIDAEGRR-

-

>A9XR73|A9XR73_9BACT

MSTITLYHNPACGTSRNTLALIRNSGEEPTIIEYLKTPPDRATLVQLLADAGLSVRDVLREKGTPYAELD

LGNPKWTDAQLLDFIEQHPVLMNRPIVVTPLGTRLCRPSEEVLDILPQPQRGAFNKEDGEPVIDAEGRR-

-

>Q39PK2|Q39PK2_BURS3

MTAITIYHNPDCDTSRNTLALIRNSGEEPAVIEYLETPPPRETLVKLLADAGLTVREVLREKGTPYAELG

LGDPKWTDGQLLDFIEQYPILMNRPVVVTPIGTKLCRPSEAVLDILPNPQKGPFTKEDGEVVIRAEGER-

-

>A9AU87|A9AU87_BURM1

MSNITIYHNPDCGTSRNALALIRNSGEEPVVIEYLRTPPSRETLVKLLADAGLTVRELLREKGTPYAELG

LGDPKWTDEQLLDFIGQHPILMNRPIVVTPIGTKLCRPSEAVLDILPNPQTGPFTKEDGEVVIDAEGKR-

-

>A9AS53|A9AS53_BURM1

MTDITIYHNPNCGTSRNTLALIRNSGEEPIIIEYLKTPPSRETLVKLLADAGLTVREVLREKGTPYAELG

LDDPKWTDEQLLDLIGQHPILMNRPIVVTPIGTKLCRPSEAVLDILPNPQKGSFTKEDGEVVINAEGKR-

-

>C1DN65|C1DN65_AZOVD

MSTITIYHNPDCGTSRNVLGLIRNSGEEPTIIEYLKTPPDRETLVALIRAMGISPRALLREKGTPYAELG

LDDSKWSDGQLVDFMLQHPILIERPIVVTPLGTRLCRPSETVLDILPRLQRGAFDKEDGEPVVDAQGRR-

-

>Q2KXX6|Q2KXX6_BORA1

MSPITIYHNPACGTSRNVLGLIRNSGEDPTIIEYLNTPPAAATLKSLIAAMGVSVREVLRKKGTPYDDLR

LSDPKWTDQELIDFMIQHPILINRPIVVTPLGTRLCRPSEQVLDILPQGQRGAFNKEDGEPLVDAEGNR-

-

>F0QDG4|F0QDG4_ACIAP

MSDITIYHNPDCGTSRNVLALIRNSGVEPTVIEYLKTPPDRATLERLVAAMGTPVRDVLREKGTPYAELG

LAEPQWSDAQLIDCMLQHPILINRPIVVTPLGTRLCRPSEAVLDILPGPQQGAFSKEDGEPVIDAKGRHH

Q

>E3HK99|E3HK99_ACHXA

MSSITIYHNPACGTSRNVLGLIRNSGEEPTIIEYLKTPPNAETLKALIAAMGIPARDVLRKKGTPYDELE

LSDPKWTEEQLIDFMLQYPILINRPIVVTPLGTRLCRPSEAVLDILPQAQRGAFNKEDGEPLIDSEGNR-

-

>E7FJ89|E7FJ89_9BURK

MSTVTIYHNPDCGTSRNVLALIRNSGEEPTVIEYLKTPPDRATLMVLIAAMGVSARAVLREKGTPYAELG

LGDPQWGDDRLIDFMLQHPILINRPIVVTPLGTRLCRPSEAVLDILPQPQRGAFRKEDGEAVVDAEGRR-

-

>A9I9C9|A9I9C9_BORPD

MSNITIYHNPACGTSRNVLALIRNSGEEPTVIEYLKTPPDSETLQHLIADMGVPVRAVLREKGTPYAELG

LDDPKWSDEQLIDFMLQHPILINRPIVVTPLGTRLCRPSETVLDILPQPQRGAFNKEDGKAVVDAQGRR-

-

>B8L2M6|B8L2M6_9GAMM

MSNITIYHNPTCGTSRNVLALIRNSGEEPNVIEYLKTPPDRETLQRLITDMGAPMRAVLREKGTPYAELG

LDDPKWGDEQLIGFMLQHPILINRPIVVTPLGTRLCRPSEAVLDILPQPQRGAFRKEDGEAVVDAEGRR-

-

>A9BR79|A9BR79_DELAS

MSSITIYHNPVCGTSRNVLALIRNSGEEPTVIEYLKTPPDRETLQRLITDMGVPVRAVLREKGTPYAELG

LDDPKWSDAQLIDCMLQHPILINRPIVVTPLGTRLCRPSEAVLDILPQSQRGAFNKEDGEAVVDAEGRR-

-

>B3G202|B3G202_PSEAI

MSQITIYHNPDCGTSRNVLGLIRNSGEEPTIIEYLKTPPDRETLKTLIAAMGVPVRAVLREKGTPYAELG

LDDSKWSDEHLIDFMLQHPILINRPIVVTPLGTLLCRPSEAVLGILPQPQRGAFSKEDGEAVVNAEGRR-

-

>D4X895|D4X895_9BURK

MSTITIYHNPACGTSRNVLGLIRNSGEEPTIIEYLKTPPGRDTLQALIAAMGVPVRDILREKGTPYAELD

LGNPKWSDDDLIGFMLQHPILINRPIVVTPLGVRLCRPSEAVLDLLPQPQRGAFNKEDGEPLVDEKGRR-

-

>A4G6H2|A4G6H2_HERAR

MSHITIYHNPDCGTSRNVLSLIRNSGEEPAVIEYLKTPPDRDMLKALIAAMGIPVRAVLREKGTPYAELG

LGDQKWSDEQLIDFMLQHPILINRPIVVTPLGTRLCRPSETVLDILPQPQRGAFNKEDGEPVVDVEGRR-

-

>C6BDL3|C6BDL3_RALP1

MSHITIYHNPACGTSRNVLGLIRNSGEEPTVIEYLKTPPDRATLKALIAAMGVPVRAVLREKGTPYAELN

LSDPKWSDEQLIDFMLQHPILINRPIVVTPLGTRLCRPSEAVLDLLPQPQRGAFNKEDGEPVVDAEGRR-

-

>E6V7R1|E6V7R1_VARPE

MNDITIYHNPACGTSRNVLALIRNTGDEPKVVEYLKTPPDRATLTRLIADMGVPVREVLRQKGTPYGELG

LGDAKWSDEQLIDFMLQHPILINRPIVVTPLATRLCRPSEAVLDILPRPQQGAFTKEDGEAVIDAKGQRA

N

>A9BTF9|A9BTF9_DELAS

MSTITIYHNPACGTSRNVLALIRNSGEEPTVIEYLKTPPDRATLQQLLVALALPVRDVLRQKGTPFDELG

LGEPSWTDAQLIDFMLQHPILINRPIVVTPLGTRLCRPSETVLDILPGAQQGAFSKEDGQAVIDAKGQRA

N

>D8D799|D8D799_COMTE

MSDITIYHNPSCGTSRNVLALIRNSGVEPMVIEYLKTPPDRATLAGLIQAMGMPVRDVLRQKGTPYDELG

LGAPKWTDEQLIDFMLQHPILINRPIVVTPLGTRLCRPSEAVLDILPKPQQAAFSKEDGEAVIDSKGNRG

K

>B2SJS9|B2SJS9_XANOP

-MALTIYHNPACGTSRNTLEMMRQSGEDPEVIEYLQTPPTREKLIELIAAMGIAPRALLRQKGTPYVELG

LDNPALTDEQLVDAMMAYPILINRPIVVSDRGVALCRPSEKVLALLDSP-VASFTKEDGETVTTGGAST-

-

>F0BIX4|F0BIX4_9XANT

-MTLTIYHNPACGTSRNTLEMMRQSGEDPVVIEYLQAPPTREKLVELLAAMGMSPRELLRQKGTPYAELG

LDNLALTNEQLVDAMMAHPILINRPIVVSGRGAALCRPSEKVLALLDKP-VSSFTKEDGEIVTAAGAST-

-

>G2IM45|G2IM45_9SPHN

-MSVTIYHNPACGTSRNVLGLIRATGEEPRVIAYLETPPTREELVSLIARMGITPRDLLRQKGTPYAALG

LDDPTLGDDALIDAMMAHPVLINRPIVVGPRGVGLCRPSERVLALLDRPLREDFVKEDGEVVKPDAKAP-

-

>Q0G3I4|Q0G3I4_9RHIZ

-MTVTIYHNPKCGTSRNVLAMIREAGEEPEVIEYLKTPPSRERLVELIGEMNMTPRALLRRKGTPYDELG

LDDESLTDDQLIDAMMAHPILINRPIVVSERGTKLCRPSETVLELLPEGAVQSFTKEDGEIVVASGAKA-

-

>G4R8A8|G4R8A8_9RHIZ

-MAITIYHNPDCGTSRNTLAMIRQSGEEPEIIEYLKAPPSRETLVDLIMRSGLSVRGAMRKKEMPYTELG

LDDPSVADETLIDAMLAHPILINRPFVVSEKGVRQCRPSELVLEILPNPDIGPFTKEDGEVIIDGNGKRV

-

>F7Y7V0|F7Y7V0_MESOW

-MTVTIYHNPACGTSRNTLALIEASGETPDVIEYLKNPPSRSRLVGLLKAMDMPARALLREKGTPYTELG

LADAKWSEDELIDQMLAHPILMNRPIVETPRGTRLCRPSELVLDLLDHP-VASFTKEDGEAVTYPAR---

-

>A9X5I6|A9X5I6_9RHIZ

-MSVTIYHNPKCGTSRNTLALIHASGEEPVVIEYVQNPPSRERLVELLRAMQMTPRQLLREKGTPYAELG

LSDLNWTDDELVDFMMAHPILINRPIVETPLGTKLCRPSELVLDILENP-VSSFTKEDGEVITYERKSR-

-

>A6X110|A6X110_OCHA4

-MTVTIFHNPKCGTSRNTLAMIRASGKEPVIIEYVQNPPTRERLLGLLAAMNMTPRELLREKGTPYAELG

LSDPKWTDDELIDFMMAHPILINRPIVETPRGTRLCRPSELVLPLLENP-VASFTKEDGEQITSEGKSR-

-

>C4WHQ3|C4WHQ3_9RHIZ

-MTVTIFHNPKCGTSRNTLAMIRASGEEPVIVEYVQNPPTRERLVGLLAAMNLTPRELLREKGTPYAELG

LSDPKWTDEELIDFMMAHPILINRPIVETPRGTRLCRPSELVLDILENP-VASFTKEDGEQVTY-GERK-

-

>G4F7R3|G4F7R3_9GAMM

-MLPQIYHNPNCGTSRNTLAMMRASGEPPEIIEYLNTPPSRERIVELLAKMGICPRDLLRRKGTPYEALG

LDDPALTDDQLIDAMLAEPILINRPIVVTLKGARLCRPSETVLDLLDHP-VAHFIKEDGEVIHYPGIAP-

-

>G2TBR8|G2TBR8_RHORU

-MGVTIYHNPACGTSRNVLAMIRQSGEEPRVIDYLKTPLSRAELVDLIAVLAIPVRDLLRRKGTPFDALG

LEDPALSDDALIDAMVAHPILINRPIVVTAKGAALCRPSEAVLAILPTPLVGSFTKEDGEVVSAPAPST-

-

>G4JVE9|G4JVE9_9RHIZ

-MTITIYHNPDCGTSRNTLAMIRASGEEPTIVEYLKHPPPRARLEQLIATLEIPVRALLREKGTPYAELG

LADPKWSDGQLLDAMLEHPILINRPIVETPRGTRLCRPSELVLDLLDHP-VSRFTKEDGEVVSHDRGRQ-

-

>Q3SRB8|Q3SRB8_NITWN

TMTVTIYHNPSCSKSRDTLAMIRQGGEEPVIIEYLKHPPDRSRLRALADAMGLPIRGLLRENGSPYEELD

LGNPKWTDDQLLDFILAHPILLQRPIVETPKGVRICRPPERVLDLLT----SPAAGEGGENTQR------

-

>A3WYV6|A3WYV6_9BRAD

TMTVTIYHNPSCSTSRNTLAMIRQSGEEPKIVEYLKHPPDRARLRALANAMSVPIRGLLREKGTPYADLD

LSNLKWTDDQLLDFMVAHPLLIQRPIVETPKGARICRPPETVLELLD---SPVDSKADGEGLRR------

-

>D7DJZ4|D7DJZ4_METS0

-MSVTIYHNPSCGTSRNTLAMIRASGVEPEVIEYIKNPPSRERLVELIAAMGGSVRELIREKGTPYVELG

LDNANVSDDALIGAMLQHPILINRPIVVTNKGVKLCRPSELVLDILENPNIGIFAKEDGEVVNVANKVC-

-

>A4YWB1|A4YWB1_BRASO

MTSVTIFHNPACGTSRNTLAMIRASGEEPDVIEYLKTPPSREQLIALINAMGVRVRDVLREKGTPYADLG

LADPKWTDDQLIEAMLVYPILINRPIVVTGKGARLCRPSELVLDLLDRP-VSSFTKEDGEVVSWTAK---

-

>A5EKJ6|A5EKJ6_BRASB

-MPVTIYHNPACGTSRNTLAMIRASGEEPEVIEYLKTPPSWEQLIALINAMGVKVRDVLREKGTPYAALG

LGDPKWTDDQLIEAMLAHPILINRPIVVTGKGARLCRPSELVLDLLDHP-VTSFTKEDGERVTWPA----

-

>E2CKG9|E2CKG9_9RHOB

-MSITIYHNPKCGTSRNTLAMIRESGVEPTIIEYLKTPPTRETFVGLIEQMGISVRDLLREKGTPFAELG

LGDDKWSDDQLIDFMMEHPILINRPIVVSDKGVRLCRPSETVLDLLPK-TIEKFVKEDGEVVSLGLAKDG

-

>A0NVR3|A0NVR3_9RHOB

-MSMTIYHNPNCGTSRNTLEMIRKSGVEPQVIEYLKTPPSREELVDLIARTGLPVRDILRQKGTPYDELG

LGEDKWTDDQLIDFMMEHPILINRPIVVSEKGVRLCRPSEKVLDLLPQ-DIGSFTKEDGEVVSSGASDA-

-

>B9R1X2|B9R1X2_9RHOB

-MTITIYHNPKCGTSRNTLEMIRKAGVDPVVIEYLKTPPSRDELVNLIARMEISVRDLLRQKGTPFDELG

LGDEKWTDDQLIDFMMEHPILINRPVVVSDKGVRLCRPSERVVDLLPV-DIGAFTKEDGEVVNAGSTNV-

-

>F7QFQ4|F7QFQ4_9BRAD

-MSVIIYHNPDCGTSRNTLAMIRASGEEPTVIEYLKTPPSRARLVELIAAMNITPRDLLRQKGTPYAELG

LGDPALTGEQIVDAMLAHPILINRPIVETPKGTRLCRPSEAVLDLLDNPVV-SFAKEDGEIVRVKTSPNG

-

>D8JRS1|D8JRS1_HYPDA

-MTITIYHNPACGTSRNTLAMIRQSGEEPEIIEYLKTPLAHDTLVDLMTRMGIGPRELLRQKGTPYAELD

LGNPKWADDQLIDVMVEHPILINRPIVVTPLGVKLCRPSEAVLDILPNPEVGMFTKEDGEVVPARKRAP-

-

>F8JAX5|F8JAX5_HYPSM

-MAITIYHNPACGTSRNTLAMIRQSGEEPEIIEYLKTPPSRTTLVDLIRRMGITPRELLRQKGTPFAELD

LGNAKWTDDQLIDFMMEHPILINRPIVVTTIGVKLCRPSETVLDILAEPNIGNFTKEDGEVIAARKKA--

-

>E8L374|E8L374_9RHIZ

-MTITIYHNPACGTSRNTLAMIRQSGEEPVVVEYLKEPPTRERLVELLAAMGISARELLRQKGTPYDALG

LGDPKWTEEELIGFMLEHPILINRPIVVTPKGARLCRPSEAVLDILPNPDIGGFIKEDGEVVGGRASQPP

-

>E8L9V9|E8L9V9_9RHIZ

-MTITIYHNLACGTSRSTLAMIRQSGEEPVVVEYLKDTPTRERLVELIKAMGITARELLRQKGTPYDALG

LADPNWTEDELIGFMLEHPILINRPIVVTPKGVRLCRPSEAVLDILPNPDIGDFTKEDGEVVTRKALNPP

E

>Q1YFL1|Q1YFL1_MOBAS

-MTVTIYHNPDCGTSRNTLAMIRQSGEDPQVIEYLKNPPSRETLIELLDMMQMKPRDLLREKGTPYAELG

LDDPKLSDEEILAAMMQHPILINRPIVVTDKGARLCRPSETVLEILPDPEIGLFTKEDGEVVIEGSEHYD

G

>A9XR76|A9XR76_9BACT

-MTITIYHNPDCGTSRNTLAMIRTSGEEPTVVEYLKDPPSRERLKELVAAMGISVRGLLREKGTPYADLD

LGNPKWSDDDLLDFMMAHPILINRPIVVTPKGVRLCRPSEAVFEVLPNPAIGRFVKEDGEVVDAPIA---

-

>Q2EEV0|Q2EEV0_RHILI

-MTVTIYHNPDCGTSRNTLAMIRQSGVEPTIIEYLKAPPSRERLKELVATMDIPVRALLREKGTPYKELG

LGDPKWTDEQLVDQMLAHPILINRPIVVTPKGVRLCRPSEAVLDLLPNPHIGRFV---GRWRGGKRS---

-

>Q89QP0|Q89QP0_BRAJA

-MSVTIYHNPECGTSRNTLAMIRQSGAEPTVIEYLKTPPSRETLKQLIAAMGISVRALLREKGTPYKELG

LADPKWTDDELLDAMMAHPILINRPIVVTAKGTRLCRPSEAVVDLLDNP-LGRFVKEDGEVIEAR-----

-

>F2J1H5|F2J1H5_POLGS

VVTVTIYHNPACGTSRNTLEMIRRSGEQPVVIEYLKTPPSRAELAGLIARMGIPVRDLLRRKGTPYDDLG

LDDPKWSDDELIDFMMAHPILINRPIVVTDKGVRLCRPSEAVLDILPNPAIGSFTKEDGEVVAERKEPQG

-

>B6JKA1|B6JKA1_OLICO

-MSVTIYHNPDCGTSRNTLAMIRQSGAEPEVIEYLKQPPSREKLVELIARMGICPRDLLRQKGTPYDALG

LADSKWSDDDLIDFMIAHPILINRPIVVTSKGVKLCRPSEEVLSILPNPHIGRFVKEDGEIINAD-----

-

>Q1QFI4|Q1QFI4_NITHX

-MTITIYHNPDCGTSRNTLAMIRQSGIEPEIIEYLKQPPSREKLIELMTRMGISPRDLLRQKGTPYDDLE

LADLRWTDDELIDFMIAHPILINRPVVVTPKGVKLCRPSEEVLSILPNPHIGRFVKEDGEIINAN-----

-

>E3I462|E3I462_RHOVT

-MSIVIYHNPACGTSRNTLAMIRQSGEEPEIIEYLETPPSREKLTELIAAMGISTRALLREKGTPYHDLG

LDDPKWSDDELIDVMLAHPILINRPIVVTPKGVKLCRPSEEVLEILPNPDIGPFTKEDGEVVTPRG----

-

>Q11N24|Q11N24_MESSB

-MKITIYHNPDCGTSRNTLAMIRQSSEEPEVIEYLKTPPSKERLVELIAAMGITPRDLLREKGTPYAELG

LSDPEWTDDELTDFMLAHPILINRPIVVTPLGVTLARPSEAVLEILPNPNIGTFTKEDGEVV--SGPPRA

-

>E8T9P6|E8T9P6_MESCW

-MTITIYHNPDCGTSRNTLAMIRQSGEEPEVIEYLKTPPSRERLVELIAAMGITPRDLLREKGTPYAELG

LADPKWSDQQILDFMLAHPILINRPIVVSPLGVVLARPSEKVLDILPNPEIGAFTKEDGEVVIYASGKRG

-

>Q2EEV3|Q2EEV3_RHIML

-MTVTIYHNPACGTSRNTLAIIRQSGEEPEVIEYLKNPPSREKLVGLIAAMGMTPRALLREKGTPYAELG

LGDPKWSDDEILDFMLAHPILINRPIVVSPLGVVLARPSERVLDILPNPDIGPFTKEDGEAV--------

-

>Q98J03|Q98J03_RHILO

-MTITIYHNPDCGTSRNTLAIIRQSGEEPQVIEYLKTPPSRARLVELLEAMAMTPRQLLREKGTPYAELG

LGDPKWSDDEILDFMLAHPILINRPIVVTPLGVVLARPSEAVLDILPNPDIGPFTKEDGEVVVDASGKRV

-
