# Supplementary material for: Accurate Simulation and Detection of Coevolution Signals in Multiple Sequence Alignments
Source: PLoS One. 2012 Oct 16;7(10):e47108. doi: 10.1371/journal.pone.0047108 (PMC3473043; doi:10.1371/journal.pone.0047108)
Supplement: MSA S4 — MSA of PHBH. (DOCX) [file pone.0047108.s015.docx]

>B6R3G9|B6R3G9_9RHOB

DRTQVAIIGGGPAGLLLSHILSENGIDSVVLEQRTKDYVLSRIRAGLLETGTVQLLRDYDLAERMDKNGK

SKKGSWITRQGFPHFIDTHKWT-GKEMMVYGQTDITEDLYDARERAGGNVINEAADVALHDVTADAPYVT

YVKDGQTHRLDCDYIAGCDGFHGVSRKTIPADILRTYERAYPFGWLGIMAEVPPL-PDLVYAYHERGFAL

ASQRNRLLSRYYIQCPLTDSVMDWSDERFWEELLARFPEEVASEIVTGPSIEKSIAPLRSFVAEPMQYGR

MFLAGDAAHIVPPTGAKGLNLAASDVFYLSRGLIEQIKSNRSTHLERYSELALRRVWSSENFSWRMTQLM

HVFPEMNGFDAKIQQNSYELLLQNETQQRALAEEYVGLPFEELD

>B2JPD4|B2JPD4_BURP8

MRTQVGIIGAGPAGLLLSHLLHLQGIDSVVLESRSRDQIESTIRAGVLEQGTMDLLTETGVGERMKAEGA

LHHGFELAFEGKRRRIDLTDLT-GKAITVYAQHEVIKDLVAARMAAQGELRFGVSEVSLHDIDGAAPAIR

YRHQGEAHELQCDFIIGCDGSQGISRNAIPEALRRDYQRVYPFGWFGILAEAPPSSDELIYARHERGFAL

VSTRSPNVQRMYFQCDPSDNVDNWSDDRIWAEMHARVDSDEGHKVVEGKIFQKNIVGMRSFVSTTMQHGR

LFLAGDAAHIVPPTGAKGLNLAVSDVRILSDALRAFYKEDGSDQLERYSETALKRIWRAEHFSYWMTRMM

HRLDDASPFEQQLQVAELEHVTTSRSAAISMAENYVGAVAV---

>Q65DR2|Q65DR2_BACLD

MRTQVGIIGAGPAGLMLANLLHRRGIEAVVIESRSRKEIEETVRAGVLEQMTVDLLNETGAGERMMKTAM

FHQGIEIRFGGKRHRIDMHKLTGGKYIAIYPQHEVIKDLIAARLQSEGRIVFNVSDVRLSNIDTEAPVIT

YQENGDTEELNCDFIAGCDGFHGPSRQSIPKEIRKEYEKVYPYGWLGILAEAPPSSPELVYAHHEDGFAL

VSTRTPEIQRLYLQVSPADSVGNWPDERIWEKLHLRLSADDGWKLTEGPIIQKNIVSMRSFICDPMQYGR

LYLAGDAAHIVPPTGAKGLNLAMADVQRLAWALEDYYETGRIERLAGYSESCLRRVWKAERFSWYMTSLL

HRHHGYTPFDYQIQLAELDHVTTSTAAAQSLAENYVGLPLEFGE

>C4TP09|C4TP09_9BACL

MRTQVGIIGAGPAGLLLSHLLYLQGIESIIIENRTREEIEGTIRAGVLEQGTVDLMNQMGVGARMMKEGH

FHEGFELRFNGRGHRINVHELTGGKYVTVYAQHEVIKDLVAARLQTGGQIHFNVGDVSLHDVDTSSPKIR

FRKDGELQEIECDFIAGCDGFRGPSRPAIPQSVRKEYQKVYPFSWLGILVEAPPSAHELIYANHERGFAL

VSTRSPQIQRLYLQVDAQDHIDNWSDDRIWSELHARLETRDGFKLLEGPIFQKGIVSMRSFVCDPMQHGR

LFLAGDAAHIVPPTGAKGLNLAAADVQVLARGLEAYYKAGKMEILNRCTEICLRRIWKAERFSWFMTTML

HRDQGHTPFERGIQLAELDYVTSSRAASTSLAENYIGLPMEF--

>D3P5J7|D3P5J7_AZOS1

TRTQVAIVGAGPAGLFLSHLLHRQGIESVILESRSREEIEGTIRAGVLEQWVVDLMNGMGLGERMMREGH

FHSGITLRFDRRSHHIDMAELTGGKRVTVYAQHEVIRDLVSARLEDGGTILFGVSDVQFHDLDGKAPRVR

FHPEEPVEELRCDFVAGCDGFHGPSRQAIPAAGRSEYQIVYPFGWLGILTEAPPSHPELIYANHERGFAL

LSTRSPEVQRLYIQVDPKDDIANWSDDRIWSELHRRLEDESGWTLKEGPIFQKGIIAMRSFVCDPMQHGR

LFIAGDAAHIVPPTGAKGLNLAVADVLVLSRAMTAYYKSGSTDGLERYSATCLRRIWKAERFSWYMTTML

HRNERESPFEQRVHLAELDYVVNSRAAMTALAENYVGLPMD---

>D1A3W7|D1A3W7_THECD

ERTQVAIVGAGPAGLLLSHLLHRRGITSVVLEIRSRDYVEKRVRAGVLEQGTVDTLIEAGVGERLQREGM

PHHGIELRYGGQGHRIAFEKLVPGRCITVYGQQEVVKDLIAARLADAGRIEFEVSDVAVHDVDSPAPSVT

YTSHGRDVRLECDVIAGCDGFHGVTRRSIPEGVLTVYERDYPFAWLGVLARVAPSSQELIYSRTERGFAL

HSMRTPQISRFYLQVAPDETLEAWPDERIWAELKARLETVEGFTLQTGPILEKGITPMRSFVVEPMQYGR

LYLAGDAAHIVPPTGAKGLNLAVADVRVLTNALVSWYRTGSTELLERYSADCLKRVWRAQHFSWWMTTLL

HTFDSDDAFGRRLQLSHLDYVTGSEAAATTLAENYVGLPFESAL

>D1C1C1|D1C1C1_SPHTD

MRTQVAIIGAGPAGLVLSHLLYLEGIDSVVLERRDREYVERRVRAGVLEQGTVDLLRKMGVAERLDREGL

VHRGIILRVEGEDHRIALSDLTGGGVITVYGQQEVVKDLIKARLETGGQILFEVEDVSLHDINTSEPVVR

FRHGGKQHELRCDVIAGCDGSHGVSRPSIPAGVLTTYERVYPFAWLGILAAAPPSTDELIYAYHERGFAL

HSLRSPQISRLYIQVRPDEDIARWPDERIWEELHIRLARE-GWSLTEGPVLEKGITTMRSFLVEPMQYGR

LFLAGDAAHIVPATGAKGLNLAVADVKVLAEALVSWFRTGSTDLLERYSDTCLRRVWRAEYFSWWMTSML

HQNWDADPFERRLQLAQLRYVCSSRAAATSLAENYVGFALV---

>Q1AVQ2|Q1AVQ2_RUBXD

MRTQVGIVGGGPAGLLLSHLLHLRGIESVVLERRSREELESEIRAGVLEQGTMDLLNEIGVGERMMREGA

FHRGVNLQFGGRRHRIDFEDLV-GRGIMLYGQHEVVKDLIAARLASGGKIVFEAQVLAVEGADTDSPSVR

YAPDGREEALSCDFVAGCDGFHGACRPSIPEGARREYERRYPFGWFGILVRAPRSSEELIYCLHERGFAL

ISTRSPEIQRMYLQCDPDDDVANWPDERVWEELHARLAMEDGWRLKEGPILQKGIVAMRSFVCEPMRYGR

LFLAGDAAHIVPPTGAKGMNLAVADVRVLCEALAEFYASGRAELLDAYSERCLRRVWKAQRFSWWMTSML

HRFHGEDEFRYRLQVAELDYVTGSRAASTSLAENYTGLPLE---

>Q02CF2|Q02CF2_SOLUE

MRTQVGIVGAGPAGLLLAHLLHRQGIESVVIETQNLKHVEERVRAGVLEQGTVDLLNSTGVGERMRREGL

VHHGIELRFRRRGHRIDLQDLTGGRAITVYAQHEVVKDLIQARLTYGGPLVFEVEAVRIEGFEGDTPRIH

YRSGGEECEITCDYIAGCDGFHGVCRPSIPAGILRVYEKTYPFGWLGVLAEAKPASGELIYANHERGFAL

LSMRTPHVSRLYLQCRPDEKVAEWPDERIWEELQKRLETVDGFRLNEGPIFQKGVTAMRSFVTEPMQYGR

LFLAGDAAHIVPPTGAKGLNLAATDVRILAHALARRYRDGSAELLEGYSEICLRRVWRAQHFSWWMTSLL

HRFEGGDGFDYRRQLAELDQLTTSRAAATTLAENYTGIPFDFTG

>A4JKC4|A4JKC4_BURVG

MRTQVAIIGAGPSGLLLSHLLRLQGVDSILVEARSREYCENRIRAGVLEQGTVDTLNEAGLGDRMRREGL

EHHGIELLFSGQRHRIDLSGLTGGRAITVYSQHEVVRDLIAAGDTHGHQMHFDVSDVALHDVESDRPFVT

FTADGRAERIDCDYIAGCDGFHGIARQTIPAEKLNTFERVYPFAWLGILADAAPSLDELVYAHHYNGFAL

FSMRSPTVTRLYLQCRPDEDLAEWPDARIWDELHTRFSNDTGWTPTEGPITQKSVTPMRSFVSETMQHGR

LFLAGDAAHIVPPTGAKGMNLAVADVRVLSRALGARYRDGDAAPLERYSATCLERIWRAEHFSYFMTNML

HSSPDDSPFVNRLKFAELKYVTRSRAAAQSLAENYVGLPFDDQL

>B4EGI1|B4EGI1_BURCJ

MRTQVAIIGAGPSGLLLSHLLRLQGVDSILVEARSREYCENRIRAGVLEQGTVDTLNEAGLGDRMRREGL

EHHGIELLFDGQRHRIDLSELTGGRAITVYSQHEVVRDLIAAGVEHGHQMHFEVSDVALHDVESERPFVT

FKADGRADRIDCDYIAGCDGFHGIARQTIPADRLNTFERVYPFAWLGILADAAPSLDELVYAHHDNGFAL

FSMRSPTVTRLYLQCKPDEDLAEWSDARIWDELHTRFSNDTGWTPTEGRITQKSVTPMRSFVSETMQHGR

LFLAGDAAHIVPPTGAKGMNLAVADVRALSRALGARYRTGDATPLERYSATCLERVWRAEHFSYFMTNML

HASPDDSPFVNRLKFAELKYVTRSRAAAQSLAENYVGLPFDDAT

>B1SYI8|B1SYI8_9BURK

MRTQVAIIGAGPSGLLLSHLLRLQGVDSILVEARSREYCENRIRAGVLEQGTVDTLNEAGLGERMRREGL

EHHGIELLFSGQRHRIDLSALTGGRAITVYSQHEVVRDLIAAGDAHGHAMHFEVTDVALHDVESERPFVT

FKADGRADRIDCDYIAGCDGFHGIARQTIPAERLNTFERVYPFAWLGILADAAPSLDELVYAHHANGFAL

FSMRSPTVTRLYLQCKPDENLAEWSDARIWDELHTRFSNDTGWTPTEGRITQKSVTPMRSFVSETMQHGR

LFLAGDAAHIVPPTGAKGMNLAVADVRALSRALGARYRDGDATPLERYSATCLERVWRAEHFSYFMTNML

HASPDDSPFVNRLKFAELKYVTRSRAAAQSLAENYVGLPFDDQT

>A9AQZ3|A9AQZ3_BURM1

MRTQVAIIGAGPAGLLLSHLLRLQGVESILLEARSREYCENRIRAGVLEQGTVDTLNAAGLGDRMRREGL

QHHGIELLFGGQRHRIDLSGLTGGRAITVYSQHEVVRDLIAAGVEHGHPMHFDVTDVALHDVDGDRPFVT

FKADGRADRIDCDYIAGCDGFHGIARQTIPAERLRTFERVYPFAWLGILADAAPSLDELVYAHHERGFAL

FSMRSPTVTRLYLQCRPDEDLGAWSDARIWDELHTRFSNDTGWTPTEGRITQKSVTPMRSFVSETMQYGR

LFLAGDAAHIVPPTGAKGMNLAVADVRALSRALGARYRTGDATALDAYSATCLERVWRAEHFSYFMTNML

HPSADDTPFVNRLKLAELKYVTRSRAAAQSLAENYVGLPFDDEA

>F0G659|F0G659_9BURK

MRTQVAIIGAGPSGLLLSHLLRLQGVDSILVEARSREYCENRIRAGVLEQGTVDTLNEAGLGERMRREGL

EHHGIELLFSGQRHRIDLSGLTGGRAITVYSQHEVVRDLIAAGVEHGHQMHFEVSGVALHDVESAHPFVT

FTADGREDRIDCDYIAGCDGFHGIARQTIPAERLRTFERVYPFAWLGILADAAPSLDELVYAHHERGFAL

FSMRSPTVTRLYLQCRPDENLDEWSDARVWDELHTRFSNDTGWTPTEGRITQKSVTPMRSFVSETMQYGR

LFLAGDAAHIVPPTGAKGMNLAVADVRALSRALGARYRSGDATRLDAYSATCLERVWRAEHFSYFMTNML

HPSADDTPFVNRLKLAELKYVSRSRAAAQSLAENYVGLPFDDDA

>Q46U22|Q46U22_CUPPJ

MRTRVAIIGAGPAGLLLGHLLQRAGIDSVILEDCTQAHVEARIRAGVLEQGTVDLLEEAGVAARLREQGM

VHHGIDLLFDGQRHRIDLSGLTGGRAITVYGQHEVVRDLIAARQACGAPLLFEVSQVALHGLDTQSPSLS

FEHGGQRHVLACDFIAGCDGSHGVSRHAVPAGALQVFERSYPFAWLGILASAAPAAQELIYASHARGFAL

FSMRSPEITRLYLQCRPDEDLAGWTDRRIWDELHARLETGDGWHLNEGPVLQKGITPMRSLVTEPMQYGR

LFLAGDAAHIVPPTGAKGMNLAVADVRNLAHALVTHYRHGRDDLLAGYSSACLARVWRAEHFSWWMTSLL

HRFDDHTPFTARLQHAELAYVCASQAASRMLAENYVGLPFAPLP

>B2U951|B2U951_RALPJ

MRTQIAIIGAGPAGLLLGQLLHRNGIDAVILETKSRAYVEERIRAGVLEQGTVDVLNEAGVGERMRREGL

VHHGIDLLFGGKRHRIDLTAMSGGRSITVYGQHEVVKDLIAARVEQGAPLLFDVSEVSVHDIESATPSVQ

FVHEGVPQTLQCDYIAGCDGFHGICRQAIPARRQAVFERVYPFAWLGILAQAKPAAEELIYANHDRGFAL

FSMRSPKITRLYLQCKPDENLAEWSDARIWDELHTRLENNDGWHLKEGPILQKSVTPMRSFVCETMQYGR

LFLAGDAAHIVPPTGAKGMNLAVADVRVLAQALTARYKQSDTAPLASYSERCLQRVWRAEHFSWWMTSML

HRFDDHTPFMQKLQRAELDYVTGSPAGARVLAENYVGLPFADAP

>C6BKL4|C6BKL4_RALP1

MRTQVAIIGAGPAGLLLGQLLHRNGIDAVILETKSRAYVEERIRAGVLEQGTVDVLNEAGVGERMRREGL

VHHGIDLLFGGKRHRIDLTAMSGGRSITVYGQHEVVKDLIAARVEQGAPLLFDVSEVSVHDIESATPSVQ

FVHDGVPQTLQCDYIAGCDGFHGICRQAIPAQRQAVFERVYPFAWLGILAEAEPAAEELIYANHDRGFAL

FSMRSPKITRLYLQCKPDENLAEWSDARIWDELHTRLETNDGWRLKEGPILQKSVTPMRSFVCETMQYGR

LFLAGDAAHIVPPTGAKGMNLAVADVRVLAQALTARYKQNDTTQLAGYAERCLQRVWRAEHFSWWMTSML

HRFDDHTPFMQKLQRAELDYVTSSAAGARVLAENYVGLPFVDAP

>D8N984|D8N984_RALSL

MRTQVAIIGAGPAGLLLGQLLQRSGIDAVILETRSRQYVEERIRAGVLEQGTVDTLNEAGVGERMRREGL

VHHGIDLLFEGKRHRLDLTALSGGRSITVYGQHEVVKDLIAARIGQGAPLLFEVSEVSLHDIESATPSVR

FVHEGVPQTLHCDYIAGCDGFHGICRPAIPAQRQAVFERVYPFAWLGILAEAAPAADELIYASHARGFAL

FSMRSPTITRLYLQCKPDENLAEWSDARVWDELHTRLENNDGWHLKEGPILQKSVTPMRSFVCETMQHGR

LFLAGDAAHIVPPTGAKGMNLAVADVRVLAQALTARYRQNDTAQLDGYAERCLQRIWRAEHFSWWMTSML

HRFDDHTPFMQRLQRAELEYLTTSPAAARTLAENYVGLPFADAP

>G3A0A6|G3A0A6_9RALS

MRTQVAIIGAGPAGLLLGQLLHRSGIDAVVLETRRRQYVEERIRAGVLEQGTVDTLNEAGVGERMCREGL

VHHGIDLLFGGRRHRLDLTAMSGGRSITVYGQHEVVKDLIAARIGQGAPLLFEVSEVSLHDIESASPSVR

FVHEGVVQTLHCDYIAGCDGFHGICRPAIPAQRQAVFERVYPFAWLGILAEAAPAADELIYASHERGFAL

FSMRSPKITRLYLQCRPDEDLAEWPDARIWDELHTRLETDDGWHLKEGPILQKSVTPMRSFVCETMQHGR

LFLAGDAAHIVPPTGAKGMNLAVADVRVLAQALMARYRQNDATALAGYSECCLQRIWRAEHFSWWMTSML

HRFDDHTPFMQRLQRAELEYLTTSPAAARVLAENYVGLPFADAP

>D8P0G4|D8P0G4_RALSL

MRTQVAIIGAGPAGLLLGQLLHRSGIDAVVLETRSRQYVEERIRAGVLEQGTVDTLNEAGVGERMRREGL

VHHGIDLLYGGRRHRLDLTAMSGGRSITVYGQHEVVKDLIAARIGQGAPLLFEVSEVSLHDIESASPSVR

FVHEGVPQTLHCDYIAGCDGFHGICRPAIPAQRQAVFERVYPFAWLGILAEAAPAADELIYASHERGFAL

FSMRSPKITRLYLQCRPDENLAEWPDARIWDELHTRLETDDGWHLKEGPILQKSVTPMRSFICETMQHGR

LFLAGDAAHIVPPTGAKGMNLAVADVRVLAQALTARYRQNDATALAGYSERCLQRIWRAEHFSWWMTSML

HRFDDHTPFMQRLQRAELEYLTTSPAAARVLAENYVGLPFADAP

>D8NRP0|D8NRP0_RALSL

MRTQVAIIGAGPAGLLLGQLLHRSGIDAVVLETKSRQYVEERIRAGVLEQGTVDTLNEAGVGERMRREGL

VHHGIDLLFGGARHRIDLTAMSGGRSITVYGQHEVVKDLIAARIGQGAPLLFEVSEVSVHDIESATPSVR

FVHEGMPQTLHCDYIVGCDGFHGICRPAIPAQRQAVFERAYPFAWLGILAEAAPAANELIYASHAHGFAL

FSMRSPKITRLYLQCRPDENLADWPDARIWDALHTRLETNDGWHLKEGPILQKSVTPMRSFVCETMQYGR

LFLAGDAAHIVPPTGAKGMNLAVADVCVLAQALTARYRRNDATALAGYSARCLQRIWRAEHFSWWMTSML

HRFDDHTPFMQRLQRAELEYLTTSPAAARVLAENYVGLPFADAL

>F6FZX7|F6FZX7_RALS8

MRTQVAIIGAGPAGLLLGQLLHRSGIDAVILETKSRQYVEERIRAGVLEQGTVDTLNEAGVGERMRREGL

VHHGIDLLFGGTRHRIDLTAMSGGRSITVYGQHEVVKDLIAARIGQGAPLLFEVSEVSVHDIESATPSVR

FVHEGMPQTLHSDYIAGCDGFHGICRPAIPAQRQVVFERAYPFAWLGILAEAAPAANELIYASHAHGFAL

FSMRSPKITRLYLQCRPDENLAEWSDARLWDELHTRLETNDGWHLKEGPILQKSVTPMRSFVCETMQHGR

LFLAGDAAHIVPPTGAKGMNLAVADVCVLAQALTARYRQNDATALAGYSARCLQRIWRAEHFSWWMTSML

HRFDDHTPFMQRLQRAELEYLTTSPAAARVLAENYVGLPFADAP

>A4BGW5|A4BGW5_9GAMM

MKTQVAIVGGGPSGMLLSLLLAKEGIESIVIERSSRDHVLSRIRAGVLEWTSVELLRRAGVGERMDREGH

VHAGTRVAWRGADHMLIDSQKWAGKPFMAYGQTYVTEDLYAAVDSIDGLVLDECDDVVLNELL-DNPYVT

FKRQGEEYRVDAQFIAGCDGYHGVSRQSIPDTVLKTFEKVYPFGWLGVMSETPPL-EDLWYVQHERGFAL

ASQRSPMLSRYYVQCPITDKVEDWSDERFWDELITRFPPDIAAQITTGPSIEKSIAPLRSFVSEPMRYGH

LFLAGDAAHIVPPTGAKGLNLAMSDVYYLWRALTLKLQTGSDELIDRYSATALDRVWKTERFSWWMTSLL

HVFPEHSAFDHRTQAAELDSIEASEHAQAWLAEQYAGFPIEDWG

>D0D2V2|D0D2V2_9RHOB

LTTQVAIIGGGPSGLLLSQLLNRAGIETVILERSTRAHVLSRIRAGILEWGTVELLQVAGVDARMQREGI

PHDGCYMTDDERVVHIDFLELT-GKRVMVYGQTEVTADLYAAQDAMGTTVLHEVSDVEILDLDQPRSAVE

FTHEGTRKRLECLYVAGCDGFHGVSRKTIPEHKRQEFERVYPFGWLGILSETPPVNDELIYSNSRHGFAL

ASMRNSNLVRYYVQVPLTDKVEDWSDQRFWDEFSRRIPSEAAGRLITGPSIEKSIAPLRSFVSEPLRWGN

LFLVGDAAHIVPPTGAKGLNLAASDVYYLHDALIEALSGRGTAGIDAYSEKALSRIWKAMRFSWQMTLML

HRFDDEDAFAAQMRRATLDHLAHSETARRDLAENYIGLPF----

>Q0FH40|Q0FH40_9RHOB

LTTQVAIIGGGPSGLLLSQLLNKSGVETVILERSTRDHVLSRIRAGILEWGTVELLRAAGVDARMDREGI

PHDGCYLTDDELMVHIDFKELT-GKQVMVYGQTEVTADLYNAQDAMGTTVLHGVSDVSIHDLDQSRSRVE

FTHEGERKVLKCLYVAGCDGFHGVSRKTIPEEKRREFERVYPFGWLGILSETPPVHDELIYSNSKHGFAL

ASMRNENLVRYYVQVPLSDKVEDWSDQRFWDEFSRRIPSEAANRLITGPSIEKSIAPLRSFVSEPLRWGN

LFLVGDSAHIVPPTGAKGLNLAASDVFYLHSALTEAVTGKGTGGIDDYSSKALARIWKAMRFSWQMTTML

HRF-DEDAFAEQMRKATLDHLAHSETARRDLAENYIGLPF----

>B7RNE7|B7RNE7_9RHOB

MKAEVVIIGGGPSGLLLSQLLNRAGVDTVILERTSRTRVLDRIRAGVLEDGTVQLLREAGVGHRMDREGI

PHEGCYLTDDDLMVRINFHELT-AKKVMVYGQTEVTADLYAAQDAMGTNIIHNVEDVVINDVKTDAPYVE

YTLEGERKRIDCKFVAGCDGFHGVSRQTIPADKRTEFERVYPFGWLGVLSRTPPANHELIYANSRHGFAL

ASMRNEKLSRYYVQVPLTDKVEDWSDDRFWEALKMRLPQETADAMVTGPAIEKSIAPLRSFVSEPLRWGS

LFLVGDSAHIVPPTGAKGLNLAVSDVYYLHDALIAALKKKDPSGIDSYADRALARIWKAMRFSWQMTTML

HQF-DEDSFAAQMRKATLSHLSQSETARRDLAENYIGLPF----

>A3SG23|A3SG23_9RHOB

MKTEVVIIGGGPSGLLLSQLLNDAGVSTVVLERTTREHVLSRIRAGVLEDGTMNLLRHAGVGARMDQQGL

PHKGCYLTDNDLMVHIDFHKLI-GKKVMVYGQTEVTRDLYDAQDRMGTTIIHEVEDVVIDGADSNAPTVS

FTKDGQRREISCTYVAGCDGFHGVSRATIPAAKRREFERVYPFGWLGILSRTKPVNDELIYASSAQGFAL

ASMRSASLSRYYIQVSSTEKVEDWSDDRFWEALKQRLPRPVSEALVTGPSIEKSIAPLRSFVSEPLRWGN

LFLVGDAAHIVPPTGAKGLNLAVSDVFYLHDALIAALKRGDHSGVEGYSAHALSRIWKAMRFSWQMTTML

HQF-EEDSFAPQMRRASLAHLAQSETARRDLAENYTGLPF----

>A4EGL5|A4EGL5_9RHOB

HKTQVCIIGSGPSGLLLAQFLAQAGIDTIVLDRRDRAYIEGRIRAGVLEQGTVAALEDAGVADRLYAEGL

RHDGFDLAFDGTRHRIDLVGLT-GKSVMVYGQTEVTRDLFSAREKQGQTFFFNVENVVPTDLKSDKPIVR

FRQNGEDHTISCDYIAGCDGFRGVSRAAIPSDVLKTFERIYPFGWLGILVEKPPVSDELIYANHARGFAL

ASMRSKTRSRYYIQCDVADDVALWSDDRFWEEFAARLGPDTAASLQTGTSFEKSIAPLRSFVAEPMRYGN

LFLAGDAAHIVPPTGAKGLNLAVADIRMLSRALITHYNASNDIYLDTYSETMLKRVWKVERFSWQLSTLM

HQFPENSPFEKRMQRAEFDYITSSEAASKVIAENYVGLPLE---

>F4QNZ0|F4QNZ0_9CAUL

SRTQVAIVGGGVAGLMLSQLLHVEGIDCVILERQTRAYTEARIRAGVLEYGTVELLRRVGVNGRMDRNGL

RHEGFALMDDGEAFRIDLHQLA-GQDVMVYGQTEITIDLMDACVERGVPIVFEAADVELHDIEG-APWVG

YTKDGKAYRIDCDFIAGCDGFHGVSRRTMPDAVAHCIEKVYPFGWLGILADVPPMDHELIYSNHERGFAL

ASQRSATRSRYYIQCEASEKIEDWSDDRIWDELAVRLGDD--GRMARGPSIEKSIAPLRSFVCEKMQHGR

LFLAGDAAHIVPPTGAKGLNLAASDVAYLADGLIGFYGRGDEAGLAGYTARALDRIWKAERFSWSMTQLT

HRFPGMSAFDRKMQKADLNYIRGSEAAQTAIAENYIGLPL----

>F3WX59|F3WX59_9SPHN

----MAIVGAGPAGMFLAHLLHAEGIDAVVIERRDRDYVEGRVRAGVLEQGTVALMRRLGLSSRLEREGL

IHGGTNVALDGEVFRIDMAALTGGATVTVYGQQEVMRDLFDAAEDRGLSIAWNARDVVLAGLDGARPSVT

WWQDDVEQHLDCDFVVGCDGYHGVSRTAIPADVLRVFERVYPFGWLGVLADVPPADHELIYANHERGFAL

ASMRSPTRSRYYIQCALDADLAEWSDDRFWDELCLRLGPETASKVTRGPSFEKAIAPLRSFVAEPMRWGR

LFLAGDAAHIVPPTGAKGMNLAVSDVTMLGAALTEHYRERSNAGIDHYSARALSRVWKAERFSWWFTSVT

HRFPTMDGFDRRIQMAELDYLRGSPAAQRTLAENYVGLPLEMA-

>B0UR35|B0UR35_METS4

MRCQVAIIGAGPAGMFLAHLLARAGIDAVVLERRTRDYVEGRVRAGVLEQGTVAVLEALGLDGRLKAEGL

VHTGTNLAYDGEIFRIDMAALTGG-AVTVYGQQEVMRDLFDAAEARGLRIVFEAEDVALRDVAGERPRVE

YRCGGAAHSLSCDFIAGCDGFHGVGRAAIPPDVLRVFERVYPFGWLGILAELPPVNHELIYANHRNGFAL

ASMRSPTRSRYYVQVGLDERIEDWPDARFWDELALRLGPEAASGLVRGPSFEKSIAPLRSVVAEPMRHGR

LFLAGDAAHIVPPTGAKGMNLAVSDVAMLGEALVAHYREGDASGLDGYSARALARVWKAERFSWWFTGLT

HRFPDMDPFARRMQVAELAYIRGSVAAQTVLAENYVGLPMG---

>B8IJ31|B8IJ31_METNO

MRTQVAIIGAGPAGLFLAHLLARAGIDAVVLERRTRDYVEGRVRAGVLEQGTVALLAELGLDARLKAEGL

VHSGTNLAYDGEIFRIDMAALTGGSAVTVYGQQEVMRDLFDAAPARGVRIVFEVEDVVLEDIATSSPSVR

YRVGGEARSLACEFIAGCDGSHGVSRASIPAEVLRVFERVYPFGWLGILAEVPPVTHELIYANHRNGFAL

ASMRSSHRSRYYVQVGLDERIEDWPDSRFWDELALRLGPEVSAGLVRGPSFEKSIAPLRSFVAEPMRHGR

LFLAGDAAHIVPPTGAKGMNLAVSDVAMLAEALVRHYRDRDSAGLDGYSARALARVWKAERFSWWFTGLT

HRFPDMDPFARRMQVAELAYIRESQAAQTVLAENYVGLPLR---

>B8GZG3|B8GZG3_CAUCN

VRTQVAIVGAGPAGLFLGHLLRQAGVDVVILERKDRAYVEGRVRAGVLERITVELMERLGVDERMRREGL

VHAGANLASDGEMFRIDMAELTGGSTVMVYGQQEVMKDLFDAAEQRDLRIVFDADAVRLHDVEGERPHIT

WRKDGAEHRLDCDFIAGCDGYHGVSRATIPDKVLKTFERVYPFGWLGILAEAPPCDHELIYSNHDRGFAL

ASMRSPTRSRYYVQCSLDDRLEDWSDERFWDEVSVRLGPEAAARIVRAPSFEKSIAPLRSFVSEPMRYGR

LFLAGDAAHIVPPTGAKGMNLAVSDVIMLSEALVEHYHERSSAGIDGYSARALARVWKAERFSWWFTSLT

HRFPDQDGFDRKMQVAELAYIKGSRAAQVTLAENYVGLPLV---

>B0SV82|B0SV82_CAUSK

MRTQVAIIGAGPAGLFLGHLLKRAGIEAVILERKDRDYVEGRVRAGVLEQITIDLMEQLDLAERLHREGL

VHGGTNLASDGETFRIDMAELTGGSTVMVYGQQEVMKDLFDAAEARGVQIVFDADEAALHGIDGASPFVT

WRQDGVEKRLDCDFIVGCDGYHGVSRAAIPSDVLKTFERVYPFGWLGILAEVPPCDHELIYSNHERGFAL

ASMRSATRSRYYIQCGLDEKIEDWDDQRFWDELCLRLGPETAAKVTRGPSFEKSIAPLRSFVAEPMRHGR

LFLAGDSAHIVPPTGAKGLNLAASDVVMLSQALVEHYQHGSDAGIDGYSARALARVWKAERFSWWFTSLT

HRFPDRDGFDRKMQVAELAYIKGSRAAQITLAENYVGLPLV---

>D5VGB7|D5VGB7_CAUST

MRTQVAIIGGGPAGLFLAHLLRRTGVDAVVLERRDRVYVEGRVRAGVLEQVTVDLMHRLELGERLRAEGL

VHNGTNIAAEGQLFRIDMAELTGGSTVTVYGQQEVMKDLFDAAESRGVRVIFDAEDVALHDIDSAKPYVT

WRKDGVEQRLDCDVIAGCDGFHGVSRAAIPAGVLKTFERVYPFGWLGVLADVPPCDHELIYCNHERGFAL

ASMRSPTRSRYYLQCGLDEKIEDWSDDRFWDELCLRLGEEAAARVTRGPSFEKSIAPLRSFVGEPMRHGR

LFLAGDAAHIVPPTGAKGMNLAVSDVVMLAEALSEYFLDGSEAGLDGYSARALARVWKAERFSWWFTGLT

HRFPDRDGFDRKMQVAELDYIRGSRAAQVTLAENYVGLPLV---

>D5VDG7|D5VDG7_CAUST

MRTQVAIIGAGPAGLLLAHLLRRAGVEAVVIERRDREYVEGRVRAGVLEQVTVDLMNTLGLGERMRAEGL

VHTGTNIASDGDLFRIDMSELTGGSTVMVYGQQEVMKDLFDAAEPRGVRIIFDAEDVALHDIDGSKPYVT

WRKDGAEHRLDCDFIAGCDGYHGVSRATIPADVLKTFERVYPFGWLGVLADVPPCDHELIYSNHERGFAL

ASMRSPTRSRYYIQCGLDERIEDWSDERFWDELCLRLGPEAGARVTRGASFEKSIAPLRSFVSEPMRHGR

LFLAGDAAHIVPPTGAKGLNLAASDVIMLGEALTEHYLEGSNAGLDGYSARALARVWKAERFSWWFTSLT

HRFPDRDGFDRKMQVAELAYMRGSRAAQVTLAENYVGLPLV---

>D9QKQ9|D9QKQ9_BRESC

SRTQVAIVGAGPAGLLLGHLLRAEGIDVVIVERASADYVLGRIRAGVLEATLTDLLHRLGIDARLNAEGL

PHDGFYLSDGEKLIHIAVGELT-GKRVTVYGQTEVTRDLMAAAPERGLEVVYQADDLALNAIETDAPYLT

YSKDGVAHRLDARFICGCDGFHGPSRKAIPDSAGRTFEKVYPFGWLGILADVPPCDDELIYANHPRGFAL

ASMRSQTRSRYYIQVPSSEDVADWSDDRLWDELTIRLGPEAGSRITRGPAIEKSIAPLRSFVFEPMAYGS

LFLAGDAAHIVPPTGAKGLNLAASDVAYLSEALAGFFRRSDNDGVAGYSARALARVWKSERFSWQLTTLM

HRFPASDAFDRRMQQAELDYIAGSEAARKSIAENYVGLPL----

>F6IEY5|F6IEY5_9SPHN

MRKQVAIIGAGPAGLLLGHLLKAEGVDCVVLERQSGPYVLSRIRAGVLEQVTVGLMERLGLDARMKAEGL

PHDGFNLADGQRLIHIDIARLT-GKQVMVYGQTELTRDLMEASEPRGLEVIYEAADVALHDIDSDAPFVT

YNRDGSEHRIDARFIVGCDGFHGPSRQAIPASAGQSFERVYPFGWLGILADVPPCNHELIYANHERGFAL

ASMRSNTRSRYYIDVPLTEKVEDWSDDRVWDELAIRLGPDAAAHIVRGPSLEKSIAPLRSFVFEPMRHES

LLLCGDAAHIVPPTGAKGLNLAASDVHYAAEALLGFFKSNDNDAISGYSEKALARVWKSERFSWSLTRLM

HRFPEDGPFERRMQVAELDYIASSEAAQRSIAENYVGLPV----

>Q2G5K1|Q2G5K1_NOVAD

MKTQVAIIGAGPAGLLLGHLLKAEGIDCVVLERQTPDYVLGRIRAGVLEQITVGLMERLGLDARLKAEGL

VEEGFNLADGERLIRIDVANLT-GKTVVVYGQTEITKDLMDAAPERGLQVIYGASEVALFDIESDAPYVT

YVHDGAPRRIDARFIVGCDGFHGPSRKAIPASVAREYERVYPFGWLGILADVPPCNHELIYANHERGFAL

ASMRSHTRSRYYVDVPLTEKVEDWSDERIWDELAVRLGPEAAANITRGPSIEKSIAPLRSYVFEPMRHGS

LLLCGDAAHIVPPTGAKGLNLAASDVHYAAEALTGFFKRADNDAVPRYSAKALARVWKSERFSWSLTKLM

HRFPEDGPFERAMQVAELEYIATSKAAQTSIAENYVGLPV----

>G2ISK1|G2ISK1_9SPHN

TKVQVAIIGAGPAGLLLGHLLRAEGIEALVIERASPDYVLGRIRAGVLERTTTDLLDRLGLSDRMNAEGL

PHDGFNLADGERLIRIDIAALT-GKQVMVYGQTELTRDLMDAAPARGLEIVYEAADVALHDIESDRPSVT

FQKDGSVHRIEADFICGCDGFHGPSRQTIPASAGQAFEKIYPFGWLGILADVPPCNHELIYANHENGFAL

ASMRSKTRSRYYIQVPLTDRVEDWPDDRLWDELAVRLGPDAAASMTRGPAIEKSIAPLRSFVFEPMRHGR

LMLAGDAAHIVPPTGAKGLNLAASDVAYLSDALIRYFQQSDSDGIAAYGPRALARVWKAERFSWQLTTLM

HRFPDTDPFARRMQMAELDYIASSTAAQTTIAENYVGLPL----

>F6F1T6|F6F1T6_SPHCR

MKTQIAIVGAGPAGLLLGHLLRAEGLEVVIVERASADYVLGRIRAGVLERTTTDLMDRLGLGGRMHREGL

PHDGFNLADGERLIRIDINALT-GRQVMVYGQTELTRDLMDAADERGLRIIYNAADVALHGLDGDAPYLT

FVEDSAARRIDAQFICGCDGFHGAARAAIPASAACAYQKVYPFGWLGILADVPPCDHELIYANHERGFAL

ASMRSETRSRYYIQVPLDEKVADWPDDRLWDELAVRLGPEAGGRITRGPALEKSIAPLRSFVFETMRHGR

LLLAGDSAHIVPPTGAKGLNLAASDVRYLSEALAGYFRRRDDGAITGYSGRALARIWQAERFSWQLTRLM

HRFPNNDAFDRRMQMADLDHIASSPAAQTAIAENYVGLPL----

>Q9L5B2|Q9L5B2_9RHOB

MKTQICIVAGGPSGLLLGQLCHQAGIDAIVLERRSRKHVLSRIRAGVLEQGFVDLLQEAKVATRMDTEGY

PHDGTVISFEDMEVRVDFRKHT-GTSVMVYGQTEVTRDLYEARDQTGGLTLHDVDEVELGNLDGEGCTVS

FTHDGQRKTIECDYIAGCDGFHGPSRRAIPAEKRKEFERVYPFGWLGILSETPPAHHELIYANSERGFAL

CSMRNERLSRYYLQAPLTDRVEDWSDDAFWAELKRRIPSRIAETLVTGPSIEKSIAPLRGFVCEPMQYGR

LFLAGDAAHIVPPTGAKGLNTAASDIHYLFEALKARYQERDAAAMGGYSERALARVWKTQRFSWWMTKLL

HRFPDTDPYELRIQRAEIEHLATVESAQQTMAINYVGLPY----

>F7Z9Y5|F7Z9Y5_ROSLO

MRTQVVIIGGGPSGLLLAQLLHGRGIDSIVLERRTRAHVLSRIRAGVLETGLLDLLEQADAGTRMRQEGM

PHDGTLITYGDEAFRIDFAELT-GKSVMVYGQTEVTRDLYAARDKTGGQSEFEVDEVQIEGADSDAPAVT

YLQSGVQNRIACDFVAGCDGFHGISRKSIPGATQRSYEKVYPFGWLGILSETPPVSDELIYANSDRGFAL

CSMRNPTLSRYYIQCDAGDHIDNWSDDAFWTELKRRIPAEQAANLITGPSIEKSIAPLRSFVTEPMQWGR

LFLCGDAAHIVPPTGAKGLNTAASDVHYLFSALTRWYQHNDADAVAQYSHRALARVWKAERFSWWFSKLM

HRFPHHSGFDLRMQRAEIEFLRSNEAAQKAMAENYVGLPYEG--

>Q167D1|Q167D1_ROSDO

MRTQVVIIGGGPSGLLLAQLLHTCGIDSIVLERRTRAHVLARIRAGVLETGLIDLLEQAGAGARVRQDGM

PHEGTWISYGDEAFRIDFAKHT-GKSVMVYGQTEVTRDLYAARDRTGGQTEFEVEDVRIEGADSDAPAVS

YLQTGQRKRIACDFIAGCDGFHGVSRTSIPEAKRRYYEKTYPFGWLGILSETPPVNDELIYANSDRGFAL

CSMRTPSLSRYYIQCEAGDHTDNWSDDAFWAELKRRLPAEQAANLITGPSIEKSIAPLRSFVCEPMQWGR

LFLCGDAAHIVPPTGAKGLNTAASDVHYLFSALSRWYQNNDADAVAQYSQHALARVWKAERFSWWFSKLM

HRFPHHSGFDLRMQRAEIEFLRSNQAAQQAMAENYVGLPYDD--

>F7IWD7|F7IWD7_SILPO

MRTQVVIVGGGPSGLLLGQLLHRKGIDTVVLERQSRAHVLGRIRAGVLEVGFVNLMREAGVAERMDREGF

VHHGTILAHGEEQIRISFEELI-GRHVVVYGQTEVTHDLYEARDRVGGKSVFNAEGVTIHDADSDAPYVT

YTANGTEHRIDCDYVAGCDGFHGVSRQTIPTGIRKEFEQVYPFGWLGLLSETPPVSDEVLYSLSDRGFAL

CSMRNATLSRYYIQCALSDKVADWSDDAFWAEFRQRLPRAVAERLVTGPSIEKSIAPLRSFVCEPMRWGR

LFLCGDAAHIVPPTGAKGLNTAASDIHYLYHALVQVFESGETEGIERYSEQALARIWKTQRFSWWMTKLL

HRFPEQSEFDLRIQRAELEFLRDSRDAQTVLAQNYVGLPY----

>C9CZ81|C9CZ81_9RHOB

MRTQVVIVGGGPSGMLLGQLLHLNGIDTIVLERRTKAHVLSRIRAGILEQGMVELMHKAGVGARLERESF

HHEGTQIAHNDRMFGINFEQLI-GKSVILYGQTEVTRDLYEAREKVGAQTIFDVADAAIHDADTDTPYVT

FHKDGREQRIDCDFIAGCDGFHGVSRRTIPASVRTEYEKVYPFGWLGILSETSPVNDELIYANSEDGFAL

CSMRNANLSRYYVQCSLGDDVGDWTDTRFWDTLRRRLPAEVGEALVTGPSIEKSIAPLRSFVSEPMRWGR

LFLCGDAAHIVPPTGAKGLNTAASDVHYLYTGLLRFYQDKDSEGLDRYSEKALARVWKAERFSWWMTSLL

HRFPDQSPFDVKMQAAELAFLRDNEDAQRVLATNYVGLPY----

>Q1GJ72|Q1GJ72_SILST

MRTQVVIVGGGPSGMLLGQLLHLNGIDTIVLERRTKEHVLSRIRAGILEQGLVELMHKAGVGARLERESF

RHHGTLISHNDEMFGINFERLI-GKSVTLYGQTEVTRDLYEARESVGATTFFDVEDATIHDADTESPYVT

FQKDGKETRIDCDFIAGCDGFHGVSRRTIPASVRTEYEKVYPFGWLGILSETPPVNEELIYANSEDGFAL

CSMRNANLSRYYVQCSLGDDVGDWTDTRFWDTLRRRLPSEVAEALVTGPSIEKSIAPLRSFVSEPMRWGR

LFLCGDAAHIVPPTGAKGLNTAASDVHYLYTGLIQYYEDKDSEGIDRYSEKALARVWKAERFSWWMTSLL

HRFPDQGPFDVKMQAAELAFLRDNKDAQRVLATNYVGLPY----

>E2CLA3|E2CLA3_9RHOB

MRTQVVIVGGGPSGLLLGQLLALKGIETVILERKTRDYVLSRIRAGILETGLVNLMRKAGVAERMDRECI

VHDGTYISYENELFSINFKELI-DQSVVVYGQTEVTRDLYDARDQIGAKTVFEAEGVQILDADSNAPLVT

YCVNGVQYTIACDFIAGCDGFRGVSRKTIPETVRQEFEKVFPFGWLGILSETPPVHDELIYANSERGFAL

CSLRNENLSRYYVQCPLTDTVEGWSDDAFWDELRSRLPQSIAEKIVTGPSIEKSIAPLRSFVSEPMRWGR

LFLCGDAAHIVPPTGAKGLNTAASDVHYLSEALIQYYEHSDEQGIDSYSEMALARVWKAERFSWATTNLL

HRFPDQSGFDLKMQRAEIESLHHNKAAQKWFAQNYVGLPY----

>A0P1F9|A0P1F9_9RHOB

MRTQVVIVGGGPSGLLLGQLLHRKGIETIILERKTREYVLGRIRAGILETGLVNMMREAGVAERMDKECF

VHDGTCISYENEMFDINFQKLI-GQNVVVYGQTEVTRDLYDARDAVGGQTIFEVEGVEIRDADTEAPYVT

FTVDGSARRIDCDFVAGCDGFHGVSRKTIPDTVRKDYEKVFPFGWLGVLSETPPVHEELIYANSARGFAL

CSMRNENLSRYYVQCNVNDDIFEWTDEKFWDELKRRVPEAIAEKLVTGPSIEKSIAPLRSFVCEPMRWGR

LFLCGDAAHIVPPTGAKGLNTAASDVHYLYEGLVHYYQDKDSHGIDTYSEKALARIWKAERFSWATTNML

HRFPDQSDFDLKMQRAEIESLHHNETAQKWFAQNYVGLPY----

>A3U2A2|A3U2A2_9RHOB

MRTQVVIIGGGPSGLLLSQLLLKAGIDAVVLERRTQDYVLSRIRAGILEQGFVNLMREAGIAGRLESEGF

VHDGTLIAAGNETFRIDFVERT-GTPVVVYGQTEVTRDLYAAREAAGGRIIYETDGVTIHDARTDRPYVT

FTAGGTEQRIDCDYIAGCDGFHGVSRQTIPDTVRKEYEKVYPFGWLGILSETPPVDDELIYANSARGFAL

CSMRNENLSRYYVQCPLSDRAEDWSDDAFWDELKRRIPEEAADKLVTGPSIEKSIAPLRSFVTEPMQWGR

LFLCGDAAHIVPPTGAKGLNTAASDIHYLYHALKDHYLEGSDAGIEGYSAKALLRIWKAERFSWWMTNLL

HRFPDQTAFDLKMQQAEIAFLRDSDAAQTVMAENYVGLPY----

>A8LPE7|A8LPE7_DINSH

MRTQVAIIGGGPSGLLLSQLLHRRGIDTVVLERQSRAHVLGRIRAGVLERGLVDLMIEAGVGDRVQREGI

PHDGTVISHGDEMFRIDFTALT-GQPVTVYGQTEVTRDLYTAREAAGGILLHGVEGVEIQGADTDTPSVS

YTLDGQRQHLSCDFVAGCDGFHGISRKTIPETARREFEKVYPFGWLGVLSETPPVHEELIYANSERGFAL

CSMRNANLSRYYIQCRLDDRPEDWSDSAFWDELRRRIPKPQAESLITGPSIEKSIAPLRSFVTEPMRWGR

LFLCGDAAHIVPPTGAKGLNTAASDVHYLYNGLRQFYEDGDSEGINRYSEKALARVWKTERFSWWFSKLM

HRFPDQSEFEHKIQLAEIDFLRSNTAAQTAMAQNYVGLPY----

>A3JQM9|A3JQM9_9RHOB

MKTQVVIIGGGPSGLLLAQLLHVQGISSVVLERKTKEYVLGRIRAGVLERGLLDLMREAGVSKRMDAEGF

VHNGTLIAYDNKTFRIDFAQLT-GNSVMVYGQTEVTRDLYAAREAAGGNIEFEVEDVEIHDVKSDAPFVT

FQVGGENRRIECDFVAGCDGFHGVSRQSIPQEKRQEFEKTYPFGWLGILSETPPLDHELIYANSPRGFAL

CSMRNANLSRYYIQCPLSDKPEDWSDDAFWLELKRRIPAEQAAKLVTGPSIEKSIAPLRSFVCEPMQWGR

LFLCGDAAHIVPPTGAKGLNTAASDVQYLYSALRQYYHENDTAGIDGYSEKALVRVWKAERFSWWFSSLL

HSFPEQSEFDQKMQLSELEFLRTNESAQKAMAENYVGLPY----

>A1B7R6|A1B7R6_PARDP

MRTQVVIIGGGPSGLLLGQLLHRKGIEAVVLERKTRDYVLGRIRAGVLETGLVRLMEEAGVSDRLHREGF

VHDGTQIAWDGGMFHIDFKQLT-GTPVVVYGQTEVTRDLYDAREAAGAQTIFEVEDVVIHDADSDRPHVT

YTRAGQGRRIDCDFVAGCDGFHGISRQTIPLDVRREYEKTYPFGWLGILSETPPVHEELIYTSSDRGFAL

CSMRNANLSRYYIQCALSDHTSDWTDAAFWDELRRRLPEDVADRLVTGPSIEKSIAPLRSFVTEPMRWGR

LFLCGDAAHIVPPTGAKGLNTAASDVQYLYNGLVQYYRDKDSEGIDRYSEKALLRVWKAERFSWWFSGLL

HRYPHQSPFDLKMQKADIAFLRDNESQQRAFAENYVGLPY----

>B6AU45|B6AU45_9RHOB

MKTQVCIIGGGPSGLLLSQLLHKRGIDSTVLERQTKDYVLTRIRAGVLERGFVALMREAGVVERLDAEGF

VHDGTLVSFGDTMFRVDFTKHA-GHHVTVYGQTEVTRDLYAAREAANGKIEFNVEDVVINGADTDAPFIT

YTVAGEERRIACDYIAGCDGFHGVSRPTIPADVRKEYEKVYPFGWLGILSKTPPVNHELIYSRSERGFAL

CSMRNENLSRYYIQCSLDDKVDDWSDEAFWDELKRRIPREQADKLITGPSIEKSIAPLRSFVTEPMRWGR

LFLCGDAAHIVPPTGAKGLNTAASDIHYLYNGLRQFYEDGSPEGLDTYSEKALARVWKTERFSWWFSSLM

HSFPDQTEFDVKMQIAEIEFLRTNEAAQKAMAENYVGLPY----

>Q28JN4|Q28JN4_JANSC

MKTQVAIIGGGPSGLLLAQLLHRRGIDSIVLERKTKDYVLGRIRAGVLEQGLVGLLEQAGCADRLHAEGF

THDGTLISYGDQMFRVDFTEHV-GQPVVVYGQTEVTQDLYAAREASGGQIVYNVDDVEIHDAKSDTPFVT

YHVDGHAKRIDCDFIAGCDGFHGISRKTIPEDARREFEKIYPFGWLGILSETPPVNHELIYANHPRGFAL

CSMRNAQLSRYYIQCSLDDHPDNWSDQAFWEELKRRIPPAQADALVTGPSIEKSIAPLRSFVTEPMRWGR

LFLCGDAAHIVPPTGAKGLNTAASDVHYLFEGLKAFYADGSDEGIDAYSEKALARVWKAERFSWWFTTMM

HRFPDQTAFDLKMQVADLEFLRGSASAQKAMAENYVGLPY----

>B6AY33|B6AY33_9RHOB

MKTQVAIIGGGPSGLLLARLLQTRGIESVVLERKTKAYVLGRIRAGVLEQGLVSLMEQAGCADRLHAEGI

AHDGTLISYGDEMFRVNFTEHT-GKPVMVYGQTEVTRDLYGGLEQSSAQIVFNAEDVVIHNAHTDKPHVT

YVVDGKPNRINCEFVAGCDGFHGVSRQTIPIEVRREYEKVYPFGWLGILSETPPVNHELIYANSPRGFAL

CSMRNENLSRYYIQCSLNDKPENWSDDAFWHELKRRIPTDQADKLVTGPSIEKSIAPLRSFVTEPMRWGR

LFLCGDAAHIVPPTGAKGLNTAASDVHYLFNGLRDYYENQSTNGIDKYSEKALARVWKAERFSWWFSSLM

HSYPDQSEFDLKMQIAELEFLRSNRAAQQAMAENYVGLPY----

>A9DEX4|A9DEX4_9RHIZ

MKTRIVIIGGGPSGLLLSQLLHSRGIDSIVLERKTKDYVLSRIRAGVLERGIVKLMEEAGCADRLHAEGI

PHDGTLVSYGDEMFRIDFTELT-GTPVMVYGQTEVTRDLYKAREAAGGRIEFEVDCVEIHGTDGDAPHVT

YTVDGEARRIDCDFIAGCDGFHGVSRQAIPLSVRTEYEKVYPFGWLGILSETPPVSEELIYANSSRGFAL

CSMRNPQLSRYYIQCSLSDSVNDWTDEAFWQELNRRIPANVAEKLVTGPSIEKSIAPLRSFVTEPMRWGK

LFLCGDAAHIVPPTGAKGLNTAASDVHYLYEGLCQFYETGSSDGIDRYSEKALSRVWKAERFSWWFSSLL

HRYPDQSPFDLKMQIAELEFLRSNKAAQKAMAENYVGLPY----

>A3XE68|A3XE68_9RHOB

MKTQVAIIGGGPSGLLLSQLLHTRGIESIVLERKTKDYVLSRIRAGVLEQGLVRLLEEAGCAERLHAEGY

VHDGTLVSYGDEMFRVDFTEHT-GTPVIVYGQTEVTRDLYAAREAAGGIIEYEVEDVVINNADSDAPYIT

YTVGGEPRRVDCDFVAGCDGFHGISRQTIPLDVRREYEKVYPFGWLGILSETPPVNHELIYANSTRGFAL

CSMRNENLSRYYIQCSLSDKPEDWTDEAFWQELKRRIPADQAAKLVTGPSIEKSIAPLRSFVTEPMRYGN

LFLCGDAAHIVPPTGAKGLNTAASDVHYLYNGLRAFYETGSREGIDSYSEKALKRVWKAERFSWWFSSLM

HRYPHQSEFDLKMQIADLEFLRSNKAAQQAMAENYVGLPY----

>A3V8D4|A3V8D4_9RHOB

MKTQVAIIGGGPSGLLLAQLLHRHNIESVVLERKTKDYVLSRIRAGVLEQGLVKLMEEAGCADRLHAEGY

EHDGTLLSYGDEMFRIDFTEHT-GTPVIVYGQTEVTRDLYAAREKAGGLIEFNVDDVVIHGADSDAPYVT

YRVDGEDRRLDCDFIAGCDGFHGVSRQTIPLDVRREYEKVYPFGWLGILSETPPVNHELIYANSPRGFAL

CSMRNENLSRYYIQCSLSDKPEDWTDEAFWQELKRRIPADQAARLVTGPSIEKSIAPLRSFVTEPMRWGR

LFLCGDAAHIVPPTGAKGLNTAASDVHYLYHGLTDYYARGSTEGIDRYSEKALARVWKAERFSWWFSSMM

HRYPDQSEFDLKMQVADLEFLRSNKAAQKAMAENYVGLPY----

>A3V8Q2|A3V8Q2_9RHOB

MKTQVAIIGGGPSGLLLAQLLHTRGIDSVVLERKTKDYVLSRIRAGVLEQGLIKLMQEAGCATRLHAEGI

PHDGTLISYGDEMFRIDFTEHT-GTPVMVYGQTEVTRDLYEAREKTGGKIEFNVEDVVIHGADTDTPHVY

YTVDGTAHRLDCDFVAGCDGFHGVSRQTIPLDVRKEYEKIYPFGWLGILSETPPVNHELIYANSPRGFAL

CSMRNENLSRYYIQCSLSDKPEDWTDEAFWQELKRRIPADQAANLVTGPSIEKSIAPLRSFVTEPMRWGR

LFLCGDAAHIVPPTGAKGLNTAASDVHYLYNGLRDFYENNSTEGIDTYSQKALARVWKAERFSWWFSSLM

HTYPDQSEFDLKMQVAEIEFLRSNKAAQKSMAENYIGLPY----

>B9NWI6|B9NWI6_9RHOB

MKTQVAIIGGGPSGLLLSQLLHRKGIDTIVLERQTKEYVLGRIRAGILETGFVDLMREAGVAARMDAECF

IHEGTIISYGDTRFGINFKEHT-GSHVVVYGQTEVTRDLYEAREALDGKIVFKTENVTIHDAGSDAPSVT

YTLDGVKHRIDCEFVAGCDGFHGVSRQTIPLTVRKEYEKLYPFGWLGILSETPPVHHELIYSGSDRGFAL

CSMRNDNLSRYYIQCSLSDSPDDWTDDAFWQELKRRIPAEFAEQLVTGPSIEKSIAPLRSFVTEPMRWGR

LFLCGDAAHIVPPTGAKGLNTAASDIHYLYNGLAQFYEDGDTEGIDRYSENALARIWKAERFSWATTNLL

HRFPQQNAFDLKMQQADVNFLRDNREAQSVFAQNYVGLPY----

>A3SR79|A3SR79_9RHOB

MRTQVCIIGGGPSGLLLGQLLHRQGIATVILERKTRDYVLGRIRAGILETGTADLLRQAGAGDRMDRESF

AHEGTVIAYGDTQFGVNFHAHT-GRDVIVYGQTELTRDLYDAREAAGAPTLFNVDHVTIHDAKSDAPHVT

YHVAGTEHRIDCDFIAGCDGFHGVSRQTIPLSVRREYEKIYPFGWLGILSETPPVHDELIYSGSERGFAL

CSMRSATLSRYYIQCALSDSPEDWSDANFWEELKRRIPEDAADRLITGPSIEKSIAPLRSFVCEPMQWGR

LFLCGDAAHIVPPTGAKGLNTAASDVHYLYTGLRQHYLDGDSDGIERYSERALARVWKAERFSWSTTNLL

HRYPHQSEFDIKMQQAEVAFLRDNAAAQKVFAQNYVGLPY----

>Q1YFP0|Q1YFP0_MOBAS

TRTRVAIIGGGPAGLLLALILHRAGIESVVLERKSRDYVLSRIRAGVLEQGTTDLLRTVGLGARMDREGL

VHDGFDIAWRGEMLHVDLRELTGGKTVTVYGQTEVTHDLYDALDEACVPVIHEVEDVTPQDITGSAPFVT

YRRDGE-QRLECDFIAGCDGFHGVCRQAIPAASIETFERVYPFAWLGVLTRTPPIQDELIYANHSRGFAL

CSLRNPQLSRYYIQCDADDDVANWPDERFFDELQARLPSAVAGNLVRGPSIEKSLAPLRSFVAEPMRYGR

MFLAGDAAHIVPPTGAKGLNLAVSDIHYLSRALIAHYLDKDDGLLDAYSETALARVWKAERFSWWFTSMI

HRFEEHGAFSQRIQEAEFDYLADSEAAQTSLAENYVGLPF----

>C9P9Z9|C9P9Z9_VIBFU

LKTQVAIIGSGPSGLLLGQLLNKQGIDNIIVERSSAPHVLGRIRAGILEQGFVDLLREADCHQRLDEEGL

VHHGFFISVEGRKQHVDLTRLTQGKVVTCYGQTEVTRDLMQAREQSGQRSFYSSPVTKLHNPEHGLANVE

FEADGETYQIECDFIAGCDGYHGISRPSIPDDIRTEYERIYPFGWLGLLTDTPPVCDELIYCKHSRGFAL

ASQRSETRSRYYLQVPNSEKVEDWSDERFWEELKRRLPTAEAEKLQTGPSIEKSIAPLRSFVCEPMQHRN

LFLVGDAAHIVPPTGAKGLNLAASDVAALYQVLSAYYAGEPRKVVEQYSDVCLRRVWNAERFSWWMTSML

HHFDDQRSFVEKMADCELEFYLHSEAGMKLIAEQYVGLPYEQIQ

>A1K8B0|A1K8B0_AZOSB

MQTQVGIIGGGPSGLCLARLLSQAGIKSIILERQNREYVEARIRAGILEQGMADLMRKAGVGARMDKEGL

LHDGIVLTFDGREERIDMASLTGGKQVMVYGQTELTKDLYDALKDDNITVIFEARDVHPVGYMDGKPKLE

FHKDGKQTIIDCDYIAGCDGYHGASRASVPRDKLKEYERIYPFGWLGLLSETPPVHEELIYANSKRGFAL

CSMRSHTRSRYYLQVGLDEKVEDWSDERFWDEIRRRLPEHIAEKMVTGPSIEKSIAPLRSFVTEPMRFGN

LFLVGDAAHIVPPTGAKGLNLAASDMYYLSTALIAYYKEGRTDLLDRYSETALRRVWAAVRFSWWFTSIM

HKF-NEDPIEHKIQLAELDYLMGSTAGKTTIAENYVGLPFDPTF

>A8TSK3|A8TSK3_9PROT

MRTQVGIVGGGPAGLLLSHLLHRQGIDSVIVERRSRAYVEGRIRAGVLEQGTVDLLAETGLGDRMAREGL

VHGGIEIAIDGQRHRIDLAALT-GKSVTVYGQTEVTKDLIEARLAAGGTIVYEADDVSVDGFDGNAPSVR

WRTADGPQELHCDFIAGCDGFHGVCRPSVPADRIRTYERVYPFGWLGILSQTAPVSDELIYASHERGFAL

ASMRSHALSRYYIQCDLDVKLDDWPDQRIWDELKTRLGPEAAATIETGPSIEKSVAPLRSFVAEPMRFGR

LFLAGDAAHIVPPTGAKGLNLAVADVRVLAEAFAEHFRDGSSAGIDGYSERALKRVWKAQRFSWWFSAQL

HRY-DAEPFARRLQLAELDYLTGSEAASHAFAENYVGLPFG---

>G4RCT0|G4RCT0_9RHIZ

MRTQVAIIGAGPAGLTLGRLLEAAGIDAVVIERRSADYVLGRIRAGVLEQSSVDLLRQMGVNQRMDAEGI

FHDGVELSFNGDMLRINFQELI-GRRVMVYGQTEVTRDLMDARAASGAKSIYEASNVQIYDFDGETPRIT

FDKDGQSHQIECDFIAGCDGFHGVSRASVPEKAISLFERIYPFGWLGVLVDQPPVAEELIYAHHERGFAL

CSMRSHSRSRYYVQVGSSEKVQDWSDDRFWDELRARLNPEIAESLKSGPSIEKSIAPLRSFVAEPVRFGR

LFLTGDAAHIVPPTGAKGLNMAIHDVAELATALSEFYGERSSAGIDTYSARVLENTWRTERFSWWMTQLL

HTFPDGGDFGRRIQRADFDYLGASRIAQQSLAENYTGFRP----

>Q11F68|Q11F68_MESSB

MRVQVAIIGAGPAGLTLGRLLERAGIDAVVIERRSADYVLGRIRAGVLEQSSVDLLETIGVGERMRREGI

PHDGVKLSFDGDMLGIDFQALI-GRKVMIYGQTEVTRDLMEARAASGAKTIYEAEDVSLHDFDGASPKIR

YRKDGVSHEITCDFIAGCDGFHGISRASVPESAIQSFERVYLFGWLGVLVDKPPVDDELIYAHHERGFAL

CSMRSHTRSRYYVQVGSNEKVEDWPDDRFWDELRARLNPDVADRLQTGASIEKSIAPLRSFVAEPLRFGR

LFLAGDAAHIVPPTGAKGLNMALHDVRILAEALSEYYAGHSQAGIDSYSHRVLAHTWKTERFSWWMTSLL

HSFPETGSFGRRIQRAEFDYLASSRIAQQSLAENYTGLLE----

>D2UGK4|D2UGK4_XANAP

MRTQVAIVGAGPAGLLLGQLLHSHGIDNVILERHTQEHVLSRIRAGVLEQGTADLLCAAGAGARMQREGI

IHHGFELALEQRRERIELSAATGGRAVTIYGQTEVTRDLMEARQAVGAISLYEAQDVQLHDIAGPRPWLS

FVQHGAAQRLDCEYVVGCDGFHGVSRQAIPDEVLRVYERVYPFGWLGVLADTPPVCEELIYARHTRGFAL

CSMRSHTRSRYYVQVPSEERVENWSDQRFWDELRRRLPDDVAQTMVIGPSLEKSIAPLRSFVAEPLQYGR

LFLAGDAAHIVPPTGAKGLNLAAGDVGLLAHLFVQAQQENSPAPLQRYSELALQRIWKAERFSWWMTTML

HQFPDEDAFSRHLHDAELDYLLGSAAGRATIAENYAGLPLAVPA

>Q4UZS0|Q4UZS0_XANC8

MRTQVAIIGAGPAGLLLGQLLANVGIDAVLIERQTPEHVLSRIRAGVLEQGSVALLQHAGVDARLQREGL

RHSGFELVVDGRRERIDLQR-AGGRSVTVYGQTEITFDLMQARERAGLRSYYQAQDVALCDISSAQPAVE

FTQHGRRHRVSCDYIVGCDGFHGISRAAIPPERLQLFERVYPFGWLGVLADVPPVHEELIYARHAHGFAL

CSMRSPSRSRYYLQVPAGTQLAQWDDHAFWAELRARLPASLGEQLVTGASIEKSIAPLRSFVAEPMQYGR

LFLAGDAAHIVPPTGAKGLNLALADVGLLAQLFARWHQRGDAAVLQQYSALALQRVWKAERFSWWMTSLL

HTFEGEDAFTARIRDAELAYLLQSDAGRTTVAENYAGLPLVTLP

>F0BFN2|F0BFN2_9XANT

MRTQIAIIGAGPSGLLLGELLLRAGIDTLIVERQTPGHVMARIRAGVLEQGSVELLQRAGVGDRLQREGL

PHHGFELSLDGRRERIDLLRGC-GRGVTVYGQTEVTADLMQARQRSDAPTYYNARDVTLHALDSATPSVE

FIHEGRRVHVACDYIVGCDGFHGVSRASIPAERMRLFERVYPFGWLGVLADTPPVHDELIYARHKHGFAL

CSMRSPTRTRYYVQVPADASVEAWSDAAFWDALRARLPAALAEQLVTGPSIEKSIAPLRSFVAEPMQYGR

LFLAGDAAHIVPPTGAKGLNLALADVGLLAQLFARWKASGDADVVRHYSALALQRVWKAERFSWWMTTLL

HTFDDEDAFTARIRQAELEYVLGSEAGRATIAENYAGLPLVEV-

>F0C327|F0C327_9XANT

MRTQIAIIGAGPSGLLLGELLLRAGIDTVIIERQTPEHVLGRIRAGVLEQGSVELLQRAGVGERLQREGL

LHHGFELSLDGQRERIDLLRGC-GRGVTVYGQTEVTADLMQARARSAAPTYYNAQDVTLCEVQSNAPAVE

FTHDGQRQRIACDYIVGCDGFHGISRASIPPERLRLFERVYPFGWLGVLADTPPVHDELIYARHPRGFAL

CSMRSPTRSRYYVQVPADARVEAWSDQAFWNELRARLPDTLATQLVTGPSIEKSIAPLRSFVAEPMQYGR

LFLAGDAAHIVPPTGAKGLNLALADVGLLAQLFERWNTSGDAGVLQQYSALALQRVWKAERFSWWMTTLL

HTFDDEDAFTARIRAAELDYLLSSEAGRATVAENYAGLPLVELP

>Q3BYR3|Q3BYR3_XANC5

MRTQVAIIGAGPSGLLLGELLRLAGIDCVIVERQTPQHVLARIRAGVLEQGSVELLQRAGVGERLQREGL

LHHGFELSLDGQRERIDLLRGC-GRGVTVYGQTEVTADLMHARQRSGAPTHYNAQDVALCDVEGPTPAVE

FTQDGQRRRLDCDYIVGCDGYHGVSRASIPAERLRLFERVYPFGWLGVLADTPPVHEELVYARHRRGFAL

CSMRSPTRTRYYIQVPASAQVEAWSDQAFWDELRARLPPALAAQLVTGTSIEKSIAPLRSFVAEPMQYGR

LFLAGDAAHIVPPTGAKGLNLALGDVGLLAQLFERWQTSGDAGVLEQYSTLALQRVWKAERFSWWMTTLL

HRFDDEDAFTARLHGAELDYLLGSEAGRATLAENYAGLPLAALS

>Q8PQG9|Q8PQG9_XANAC

MRTQVAIIGAGPSGLLLGELLRLAGIDCVIIERQTPQHVLARIRAGVLEQGSVELLQRAGVGERLQREGL

LHHGFELCLDGQRERIDLLRGC-GRGVTVYGQTEVTADLMHARQRSGAPTHYNAQDVTLCDVEGATPAVE

FTQDGQRKRLHCDYIVGCDGYHGISRASIPAERLRLFERVYPFGWLGVLADTPPVHEELIYARHPRGFAL

CSMRSPTRTRYYIQVPASAQVEAWSDQAFWDELRARLPPALAAQLVTGASIEKSIAPLRSFVAEPMQYGR

LFLAGDAAHIVPPTGAKGLNLALGDVGLLAQLFERWKASGDAGALEQYSTLALQRVWEAERFSWWMTTLL

HRFDDEDAFTARLHGAELDYLLGSEAGRATLAENYAGLPLAALS

>D4SZW0|D4SZW0_9XANT

MRTQVAIIGAGPSGLLLGELLRLAGIDCVIIERQTPQHVLARIRAGVLEQGSVELLQRAGVGERLQREGL

LHHGFELCLDGQRERIDLLRGC-GRGVTVYGQTEVTADLMHARQRSGAPTHYNTQDVTLCDVEGTTPAVE

FTQDGQRKRLDCEYIVGCDGYHGISRASIPAERLRLFERVYPFGWLGVLADTPPVHEELIYARHPRGFAL

CSMRSPARTRYYIQVPASAQVEAWSDQAFWDELRARLPPALAAQLVTGASIEKSIAPLRSFVAEPMQYGR

LFLAGDAAHIVPPTGAKGLNLALGDVGLLAQLFERWKTSGDAGMLEQYSTLALRRVWKAERFSWWMTTLL

HRFDDEDAFTARLHGAELDYLLGSEAGRATLAENYAGLPLAALS

>A3VAU4|A3VAU4_9RHOB

IKTKVGIIGGGPSGLLLSQLLDLKGIDSVVLERKSREYVLSRIRAGVLEQGFTDLMREAGVGERMDREGE

IHDGVVISDNGVEHRIDLKGLTGGDTVMVYGQTEVTRDLYDAREARGGKVIHDADNVQPHDLTTDAPYLT

YEKDGETHRVDCDFIVGADGFHGVSRKSIPADQIREYEKVYPFGWLGILSETKPVNDELIYARSDRGFAL

CSLRSQVLSRYYVQVPLDDKVEDWSDERFWDELKARLPEDVAARLETGPSIEKSIAPLRSFVCEPMRYGN

LFLAGDAAHIVPPTGAKGLNSAASDIYYLYHAMIAYYDEGDASGLDSYSETALRRVWKAQRFSWWMTSML

HQFPDHPEFEQKMQETELDYFLGSEAARITLAENYVGLPF----

>B7QS62|B7QS62_9RHOB

ESTQVAIIGGGPSGLLLSQLLHKAGIDTIVLERQTRDYVLGRIRAGVLETGFVELLRKSGAGARMDREGE

IHHGFHIAHQGRLDRIDLARHAQGQTVMVYGQTEVTRDLYEARDAMGGRVLHEVQDVVLHDLKGAQPHVT

FIHDGAERRIDATFIVGADGFHGVSRKSIPADILREYEKVYPFGWLGILSETAPAAPELIYARHDRGFAL

CSMRNSRLSRYYIQVPLSDKAEDWSDDAFWQELKRRLPKAVAEQMETGPSIEKSIAPLRSFVAEPMRYGA

LFLAGDAAHIVPPTGAKGLNLAASDIHYLYEGFADYFLRGEAGGLDSYSAKALARVWKAERFSWWMTSLL

HRFPDMSPADLRLQQADLDYLFSSEAAQTSLAENYVGLPY----

>A9ESV8|A9ESV8_9RHOB

QTTQVVIVGGGPSGLLLSQLLHRAGIDTIVLERQTRDYVLGRIRAGVLEHGFVDLLRRAGASARMDRDGM

VHHGFHIAHQGRLDRIDLAGSAKGQTVMVYGQTEVTRDLYDARDAMGGQTIHEAANVALHDLTTARPNVT

YEQQGETRRIDTKFIVGADGFHGVSRKSIPSDVLREYEKVYPFGWLGVLSQTSPAADELIYARHDRGFAL

CSMRNAQLSRYYIQVPLTDRVEDWSDAAFWEELKRRLPEDVAKGLQTGVSIEKSIAPLRSFVAEPMRYGA

LFLAGDAAHIVPPTGAKGLNLAASDIHYLYEGLSDHFQRNDDTALDSYSERALARIWKAERFSWWMTNLL

HRFPDMSPADLRLQQADLDYLFSSDAAQASLAENYVGLPY----

>B6BBE4|B6BBE4_9RHOB

HQTQVVIIGGGPSGLLLSQLLHRKGISSIVLEKHTRAHVLGRIRAGVLEHGFVNLMREAGCGVRMDREGE

IHEGFHIAHQGQLDRIDLAKHTGGDTVMVYGQTEVTRDLYEARDAMGGVVIHEAQNVQPHALTEARAFVT

WEQEGEAQRAECDFIVGADGFHGVSRKSIPADVLKEYEKVYPFGWLGVLSETPPVSEELIYARHERGFAL

CSLRSRVLSRYYIQVPLTDRVEDWSDAAFWQELKRRLPDEVAAGLQTGPSIEKSIAPLRSFVAEPMRYGN

LFLAGDAAHIVPPTGAKGLNLAASDIYYLYHGFQDHYLKGDSRGLDAYSEKALARVWKAERFSWWMTNLL

HRAPEGSETDLRLQRADLDYLFSSDAAQASLAENYVGLPY----

>A3XCC6|A3XCC6_9RHOB

STTQVAIIGGGPSGLLLSQLLHRQGIETVVLERQTRDYVLGRIRAGVLERGFVELMREAGCAARMEAEGE

VHHGVYLAHQGRMDRIDLAESADGATVMVYGQTEVTRDLYEAREAMGGIVIHQADKVQPHDLKSQTPYVT

YEKNGVEHRIDCDFIIGADGFHGVSRKSIPADVLREYEKVYPFGWLGVLSETKPASPELIYARHDRGFAL

CSMRSQVLSRYYIQVPLTDKVEDWSDEAFWSELKARLPQEVAEGLQTGTSIEKSIAPLRSFVAEPMRYGQ

LFLAGDAAHIVPPTGAKGLNSAASDIYYLYHGFLDHYKNDDSAGLEGYSEKALARVWKAERFSWWMTNLL

HRFPEMSEVDLRLQCADLDYLFSSEAAQASMAENYVGLPY----

>A4EZG6|A4EZG6_9RHOB

TRTQVAIIGGGPSGLLLSQLLHRQGIDTVVLERQSRDYVLGRIRAGVLEQGFVQLMREAGCATRMEAEGE

QHHGFMIAHQGRMDRVDLQGSANGASVMVYGQTEVTRDLYDARDEMGGEVVHLAENVRLHELTSQHPYVT

YEKDGGTHRVDCDFVVGADGFHGVSRKSIPSDVLCEYEKIYPFGWLGVLSNTPPAASELIYARHERGFAL

CSLRSQVLSRYYIQVPLTDRAEDWSDEAFWSELKKRLPQQVADGLETGPSIEKSIAPLRSFVAEPMRYGN

LFLAGDAAHIVPPTGAKGLNSAASDIHYLYQGFLAHYQKGDSAGLENYSQKALARVWKAERFSWWMTNLL

HRFPEMSKTDLRLQCADLDYLFSSEAAQASLAENYVGLPY----

>A1WL11|A1WL11_VEREI

MKTRVCIIGGGPSGLLLAQLLHLQGIDSVVLERQSREHVLGRIRAGVLEHGFAALMRLARCGTRMDQEGQ

IHHGFLIAHEGRLDRIDLHKYSAGSSVLVYGQTELTRDLYAARERMNGSVIHDVQAVQPQGLTSASPAVT

YRIGQELARVDCDFVIGADGFHGVSRQAIPPGVLKEYEKLYPFGWLGLLSRTPPVSPELVYAKHPRGFAL

CSLRSPQLSRYYIQVPSSERVEDWSDEAFWAELERRLPRSVADKLVTGAAIEKSIAPLRSFVAEPMRYGR

LFLVGDAAHIVPPTGARGLNSAASDVHYLYHALLAHYQNGDDTGLDGYSAKALARVWKAQRFSWWMTNML

HSFPDALAYDQRLQDTELAYLFSSEAALRSLAENYVGLPF----

>Q89SR3|Q89SR3_BRAJA

MKVQVCIIGGGPSGLLLSQLLHLKGIDTVVLEKYSRDHVLARIRAGVLEHGFAKLMREAQCGERMDREGE

IHRGFEIAHDGVLSHIDLHKHSGGNSVLVYGQTELTRDLYEARDRLGGKVVHNAEDVTPHDLASDRPYVT

YRSNDEIVRVDCDYIVGADGFHGVSRKSIPKDVLREYEKVYPFGWLGVLSRTKPVSSELIYVKHERGFAL

CSLRSQVLSRYYIQVPLTDKVKDWSDDAFWAELKRRLPEEVAGRLITGPAIEKSIAPLRSFVAEPMSYGR

LFLAGDAAHIVPPTGARGLNSAASDIYYLYHAMLAHYQSGDDSGLEGYSAKALARIWKAQRFSWWMTMLL

HRFPDRLDYEDRLQQTELEYLFSSETAQRLLAENYVGLPF----

>A4YQK6|A4YQK6_BRASO

MKVQVCIIGGGPSGLMLSQLLHLQGIDTIILEKSSRDYVLSRIRAGVLEHGFAKLMREAQCGERMDREGE

IHHGFYIAHDGKLDRVDLHKYSGGNSVMVYGQTELTRDLYEARDKLGGKVVHNAADVTPHDIATDRPYVT

YRSGEEVIRVDCDYIIGADGFHGVSRKSIPQDKIKEYERVYPFGWLGVLSRTKPVSPELIYAKHERGFAL

CSLRSQVLSRYYVQVPLTDKVEDWSDDAFWAELKRRLPEEVAAKLITGPSIEKSIAPLRSFVCEPMRYGR

LFLAGDAAHIVPPTGARGLNSAGSDIYYLYQGLLDHYKKGDDSGIDGYSQRALARIWKAQRFSWWLTTLL

HRFPDRLPYEDRLQSTEIDYLFSSDAAQRSLAENYVGLPY----

>A5EFA6|A5EFA6_BRASB

MKVQVCIIGGGPSGLMLSQLLHLQGIDTIILEKSSRDYVLSRIRAGVLEHGFAKLMREAQCGERMDREGE

IHHGFYIAHDGKLDRVDLHKYSGGNSVMVYGQTELTRDLYEARDRLGGKVVHNAHDVTPHDIASDAPYVT

YRSGDELIRVDCDYIIGADGFHGVSRKSIPQDKIKEYERVYPFGWLGVLSRTKPVSPELIYAKHERGFAL

CSLRSQVLSRYYIQVPLTDKVEDWSDDAFWEELKLRLPAEIAAKLITGPSIEKSIAPLRSFVAEPMRYGR

MFLAGDAAHIVPPTGARGLNSAASDIYYLYHALVDHYKKGDDRGIDGYSQRALARIWKAQRFSWWMTTLL

HRFPDRLPYEDKLQDTEFAYLFSSEAAQRSLAENYVGLPF----

>G0E394|G0E394_ENTAK

MRTQVAIIGAGPSGLLLGQLLHNAGIHTVILERQSAEYVLGRIRAGILENGTVELLREAGVAKRMDAEGL

VHHGVEFLFEGQRVPVPLSELTGGKSVMVYGQTEVTRDLMAARRDSGAPIIYGVSEVAIEDVKSDRPVVS

YVSGGEKCRLECDFIAGCDGFHGVSRQSIPRDILKEYESVWPFGWLGLLADTPPVNPELIYAHHERGFVL

CSQRSLTRSRYYLQVPLSDNVSAWSDERFWNELKRRLPEDLASKVVTGHSLEKSIAPLRSYVVEPMQYGR

LFLVGDAAHIVPPTGAKGLNLAASDVNYLWRILREYYRHGRADLLASYSRFALDRVWKGERFSWFMTRLL

HDFPEQTDFDKKMQAADRRYYLGSRAGLTTIAENYVGLPMEQVT

>A6THX7|A6THX7_KLEP7

MKTQVAIIGAGPSGLLLGQLLHNAGIHTVILERQTPQYVLGRIRAGILESGTVDLLREAGVAQRMDAEGL

VHHGVEFLFDGQRVPVALSELTDGKSVMVYGQTEVTRDLMAARAASGAPIVYGVSEVAIHDAKSDRPTIT

YLSEGETCRLECDFIAGCDGFHGVSRQSIPAGILQTYESVWPFGWLGLLADTPPVNPELIYAHHQRGFVL

CSQRSLTRSRYYLQVPLSDKVEAWSDERFWQELKSRLPEELASRLVTGHSLEKSIAPLRSFVVEPMQYGR

LFLVGDAAHIVPPTGAKGLNLAASDVNYLWRILREYYHRGRSDLLAAYSQLALDRVWKGERFSWFMTRLL

HDFPDQNAFDAKMQAADRRYYLGSRAGLTTIAENYVGLPMERVA

>D2TRV9|D2TRV9_CITRI

MKTQVAIIGAGPSGLLLGQLLHKAGIRTFIIERQTPEYVLGRIRAGILESGTTDLLREAEAARRMEQEGL

LHHGVEFIFDGERIPVALSELTGGKSVMVYGQTEVTRDLMQARSDCGAPTIYGVQQVEIHDAKSDRPYIT

FEKEGEKCRIDCDFIAGCDGFHGVSRQSIPRDVIREYESLWPFGWLGLLSDTPPVNPELIYAHHERGFVL

CSQRSLTRSRYYLQVPLSEQVEEWSDERFWGELKRRLPEALATRLVTGHSLEKSIAPLRSYVVEPMQYGR

LYLVGDAAHIVPPTGAKGLNLAASDVNYLWRILAQYYRNGRADLLDTYSRLALNRVWKGERFSWFMTHLL

HDFPEKSAFDRKMQAADRQYYLRSRAGLTTIAENYVGLPYEKVG

>E9ZBI0|E9ZBI0_ESCFE

MKTQVIIIGAGPSGLLLGQLLHKAGIRTLIIERQTPEYVLGRIRAGILESGTVDLLREAGVAKRMDREGL

IHHGVEFIFDGKRIPVPLSELTDGKNVMVYGQTEVTRDLMQARNESDAPTIYGVSQVMIHDAKSDRPSVS

FEKDGEHCRVECDFIAGCDGFHGVSRQSIPRDVIREYESVWPFGWLGLLADTPPVNPELIYAHHQRGFVL

CSQRSLTRSRYYLQVPLSEQVEAWSDERFWAELKSRLPHELAEKLVTGHSLEKSIAPLRSYVVEPMQYGK

LFLVGDAAHIVPPTGAKGLNLAASDVNYLWRILTQYYHAGRTDLLARYSQLALNRVWKGERFSCFMTHLL

HDFQDKSEFDRKMQEADRHYYLESVAGLTTIAENYVGLPFENVG

>F8GX30|F8GX30_CUPNE

MKTQVAIIGAGPSGLLLGQLLTRAGIDNIILERQTPEYVLGRIRAGVLEQGTVDLMREAGVSERMDAEGL

VHEGIELVYGGERDRLDLKALTGGSTVLVYGQTEVTRDLMDARLAAGAPTIYQAANVRLHDVKGAQPYVT

FERDGETIRLDCDYIAGCDGFHGISRQAIPAEIQKHYERVYPFGWLGLLSDTPPVSHELIYAHHSRGFVL

CSQRSQTRSRYYLQVPLTERVEDWSDDRFWEELKRRLPAEVADKLVTGPSLEKSIAPLRSYVVEPMQYGK

LFLVGDAAHIVPPTGAKGLNLAASDVNTLYRILRLFYQKGRTDLLDKYSDIALRRVWKAERFSWFMTNLL

HEFPDKEPFDQRMQRTDYDYYTSSEAGLRNIAENYVGLPYDPVE

>C1DQY2|C1DQY2_AZOVD

MKTQVAIIGAGPSGLLLGQLLHKAGIDNVILERHSPDYVLGRIRAGVLEQGVVDLLREAGVAERMDREGL

VHEGIELACSGRRIRLDLKALSGGKTVMVYGQTEVTRDLMDARRASGAPIVYEAQNVRLSGLKDGMPHVT

YEKDGQTHRLDCDYIAGCDGFHGVSRQSIPAEALSHYERVYPFGWLGLLSDTPPVHEELIYAHTDLGFVL

CSQRSPTRSRYYLQVPLSDRVEDWSDERFWNELKRRLPGDVANRLVTGPSLEKSIAPLRSYVVEPMQYGR

LFLVGDAAHIVPPTGAKGLNLAGSDVCYLYRILLKVYREGRTELLEKYSELALRRVWKGERFSWFMTNLL

HDFEGSDAFDRRMQLADRDYYLDSEAGRVTIAENYVGLPYEEIA

>O30873|O30873_9GAMM

MKTQVAIIGAGPAGLLLGQLLHKAGIDNVILERHTPDYVLGRIRAGVLEQGVVDLLREAGVAERMDREGL

VHEGIELVCSGRRIRLDLKALSGGKTVMVYGQTEVTRDLMDARQASGAPIVYEAENVQLYGLKDGTPYVT

YEKDGQPQRIDCDYIAGCDGFHGVSRKTIPAEVLSHYERVYPFGWLGLLSDTPPVHEELIYAHTDLGFVL

CSQRSTTRSRYYLQVPLSEKVEDWSDERFWNELKRRLPGDVANRLVTGPSLEKSIAPLRSYVVEPMQYGR

LFLVGDAAHIVPPTGAKGLNLAGSDVCYLFRILVKVYGEGRTDLLEKYSELALRRVWKGERFSWFMTNLL

HDFAGSDAFDRRMQLADREYFLNSEAGRVTIAENYVGLPYEEVA

>Q4K685|Q4K685_PSEF5

MKTQVAIIGAGPSGLLLGQLLHKAGIDTVIVERQTPDYVLGRIRAGVLEQGTVDMLREAGVARRMDAEGL

VHEGVELLMGGKRVRIDLKALTGGKTVMVYGQTEVTRDLMEARAACAAPIIYSADKVQPHDMKGAQPYIT

YEKDGQLQRIDCDYIAGCDGFHGVARKSIPEDVLTHYEREYPFGWLGLLSDTPPVNHELIYGQHERGFVL

CSQRSLTRSRYYLQVPLSDKVEDWSDERFWNELKARLPEEVAADLVTGPALEKSIAPLRSYVVEPMQYGK

LFLVGDAAHIVPPTGAKGLNLAASDVCYLYRILVKVYREGRTELLEKYSELALRRVWKGERFSWFMTNLL

HDFGDQDAWDHKMQQADREYFLNSHAGLVNIAENYVGLPYEEIC

>Q1I960|Q1I960_PSEE4

MKTQVAIIGAGPSGLLLGQLLHKAGIDTLIVERQAPDYVLGRIRAGVLEQGTVELLREAGVAARMDREGL

VHEGVELVLAGRRQRLDLKALTGGKSVMVYGQTEVTRDLMQAREQSGAPIIYSASRVQPHDIDTTRPYLT

FEKEGRTQRVDCDYIAGCDGFHGVSRQSIPPGVLKVYERVYPFGWLGLLADTPPVSHELIYAHHDRGFVL

CSQRSHTRSRYYLQVPLEERVENWPDARFWEELKARLPQDVAERLVTGPALEKSIAPLRSQVVEPMQHGR

LFLVGDAAHIVPPTGAKGLNLAASDVNYLYRILVKVYREGRTELLAQYSPLALRRVWKGERFSWFMTQLL

HDFGDQDDWDRKMQEADREYFLSSAAGLANIAENYVGLPFEAVE

>B1J6L4|B1J6L4_PSEPW

MKTQVAIIGAGPSGLLLGQLLHKAGIDTVILERQTPDYVLGRIRAGVLEQGTVDLLREAGVAERMEREGL

VHEGVELLVGGRRQRLDLKGLTGGKTVMVYGQTEVTRDLMQAREASGAPIIYSASNVRPHELKGERPYVT

YEKDGQTHRLDCDYIAGCDGFHGVSRQSIPEGVLKHYECVYPFGWLGMLSDTPPVNHELIYAHHERGFSL

CSQRSQTRSRYYLQVPLDERVEDWSDERFWTELKARLPEDVAARLVTGPALEKSIAPLRSLVVEPMQYGH

LFLVGDAAHIVPPTGAKGLNLAASDVNYLYRILVKVYREGRTDLLQQYSPLALRRVWKGERFSWFMTQLL

HDFGEKDAWEQKMQEADREYFLSSPAGLVNIAENYVGLPFEDVV

>F8G0F5|F8G0F5_PSEPU

MKTQVAIIGAGPSGLLLGQLLHKAGIDTVIVERQTPEYVLGRIRAGVLEQGTVDLLREAGVAERMDREGL

VHEGVELLVGGRRQRLDLKALTGGKTVMVYGQTEVTRDLMQAREASGAPIIYSASNVQPHELKGEQPYLT

YEKDGQMHRVVCDYIAGCDGFHGVSRQSIPEGILKQYERVYPFGWLGMLSDTPPVNHELIYAHHERGFVL

CSQRSHTRSRYYLQVPLDEQVEAWPDERFWAELKARLPEDVAARLVTGPALEKSIAPLRSLVVEPMQYGH

LFLVGDAAHIVPPTGAKGLNLAASDVNYLYRILVKVYREGRTDLLQQYSPLALRRVWKGERFSWFMTQLL

HDFGSKDAWDQKMQEADREYFLTSPAGLVNIAENYVGLPFEEVA

>Q9R9T1|Q9R9T1_PSEPU

MKTQVAIIGAGPSGLLLGQLLHNAGIETVIVERQTPEYVLGRIRAGVLEQGTVDLLREAGVSARMDREGL

VHEGVELLVGGRRQRLDLKALTGGKTVMVYGQTEVTRDLMQAREASGAPIIYAANNVQPHELKGERPYLT

FEKDGQAHRLECDYIAGCDGFHGVSRQSIPEGVLKQYERVYPFGWLGLLSDTPPVNHELIYAHHERGFAL

CSQRSQTRSRYYLQVPLDDKVEAWSDERFWDELKARLPAEVAADLVTGPALEKSIAPLRSLVVEPMQYGH

LFLVGDAAHIVPPTGAKGLNLAASDVNYLYRILVKVYGEGRTDLLQQYSPLALRRVWKGERFSWFMTQLL

HDFGSKDAWDQKMQEADREYFLNSPAGLLNIAENYVGLPYEAVV

>F0EDY6|F0EDY6_9PSED

MKTQVAIIGAGPSGLLLGQLLHNAGIDTVIVERQTAEYVLGRIRAGVLEQGTVDLLREAGVAERMDREGL

VHEGVELLVGGRRQRLDLKALTGGKTVMVYGQTEVTRDLMQARAASGAPIFYSASNVQPHELKGEKPYLT

FEKDGQVQRLDCDYIAGCDGFHGVSRQSIPEGVLKQYERVYPFGWLGLLADTPPVNHELIYAHHARGFAL

CSQRSQTRSRYYLQVPLQDRVEEWPDERFWGELKARLPAEVAADLVTGPALEKSIAPLRSLVVEPMQYGH

LFLVGDAAHIVPPTGAKGLNLAASDVNYLYRILLKVYREGRTELLQQYSPLALRRVWKGERFSWFMTQLL

HDFGSKDAWDQKMQEADREYFLTSPAGLLNIAENYVGLPFEEVV

>B0KPT6|B0KPT6_PSEPG

MKTQVAIIGAGPSGLLLGQLLHKAGIDNVILERQTAEYVLGRIRAGVLEQGTVDLLREAGVSERMDREGL

VHEGVELLVGGRRQRLDLKALTGGKTVMVYGQTEVTRDLMQAREASGAPIIYSASNVQPHELKGEKPYLT

FEKDGQVHRVDCDYIAGCDGFHGVSRQSIPEGVLKQYERVYPFGWLGLLSDTPPVNHELIYAHHERGFAL

CSQRSQTRSRYYLQVPLQDRVEAWSDERFWDELKARLPADVAADLVTGPALEKSIAPLRSLVVEPMQYGH

LFLVGDAAHIVPPTGAKGLNLAASDVNYLYRILLKVYREGRTDLLQQYSPLALRRVWKGERFSWFMTQLL

HDFGSKDAWDKKMQEADREYFLTSPAGLVNIAENYVGLPFEEVV

>E4RA37|E4RA37_PSEPB

MKTQVAIIGAGPSGLLLGQLLHKAGIDNIIVERQTAEYVLGRIRAGVLEQGTVDLLREAGVAERMDREGL

VHEGVELLVGGRRQRLDLKALTGGKTVMVYGQTEVTRDLMQAREASGAPIIYSAANVQPHELKGEKPYLT

FEKDGRVQRVDCDYIAGCDGFHGISRQSIPEGVLKQYERVYPFGWLGLLSDTPPVNHELIYAHHARGFAL

CSQRSQTRSRYYLQVPLQDRVEEWSDERFWDELKARLPAEVAADLVTGPALEKSIAPLRSLVVEPMQYGH

LFLVGDAAHIVPPTGAKGLNLAASDVNYLYRILVKVYHAGRVDLLAQYSPLALRRVWKGERFSWFMTQLL

HDFGSKDAWDQKMQEADREYFLTSPAGLANIAENYVGLPFEEVT

>G4F2G8|G4F2G8_9GAMM

MKTQVAIIGAGPSGLLLGQLLHRQGINNVIVERRSGEYVLSRIRAGVLEQGMVDLLREAGVDQRMDKEGL

PHDGFELVFDNRRVRVALDELTGGSKVMVYGQTEVTRDLMEAREFAGATTLYEAENVQPHELESDSPYLT

FEHKGETVRLDCDYIAGCDGYHGVSRQAIPQHRIKEFEKVYPFGWLGVLSDTPPVSDELIYARHERGFSL

CSMRSATRSRYYLQVPSDEKVENWSDERFWEELKRRLPDEVAEKLVTGPSIEKSIAPLRSYVVEPMQYGR

LFLVGDAAHIVPPTGAKGLNLAASDVNTLYRLMVKVYREGRTDLIERYSQTCLKRIWKAERFSWWMTSML

HNFSDEEDFNSRMQLAELDYVTSSTAGLTTIAENYVGLPYESLE

>E1V9E1|E1V9E1_HALED

MKTQVAIIGAGPSGLLLGQLLQRQGIDNVILERRSGEYVLGRIRAGVLEQGMADLLREAGVDERMDAEGL

PHDGVELAFDNRRVRIDLAGLTGGKQVMVYGQTEVTRDLMAARQATGGTTLYEVDDVQPHDLDTERPYVT

FVKHGETQRLDCDYVAGCDGYHGVSRESIPKDRIKEFERVYPFGWLGLLSDTPPVADELIYARHERGFAL

CSMRSETRSRYYLQVPLEEKVEDWSDERFWEELKRRVPEDVAAKLVTGPSLEKSIAPLRSFVVEPMQYGR

LFLVGDAAHIVPPTGAKGLNLAASDVNSLYRLLVKVYHEGRTDLIPNYSRTCLRRIWKAERFSWWMTSML

HKFSDEEDFGSRMQQAELDYVTGSEAGLTTIAENYVGLPYESLE

>A8I4C8|A8I4C8_9GAMM

MKTQVAIIGAGPSGLLLGQLLQRAGIDNVILERRSGEYVLSRIRAGVLEQGMVDLLREAGVEQRMDAEGL

PHDGVELAFDNRRVRIDLAGLTGGKQVMVYGQTEVTRDLMEARAAEGGKTLYEVDNVQPHALETDAPYLT

FEHNGETLRLDCDYIAGCDGYHGVSRQTIPADRLKTFERVYPFGWLGLLSDTPPVSDELIYARHERGFAL

CSMRSQTRSRYYVQVPLEEKVEDWSDARFWEELKRRLPEDVAANLVTGPSLEKSIAPLRSFVAEPMQHGR

LFLVGDAAHIVPPTGAKGLNLAASDVNTLYRLMVKVYHEGRTDLVPRYSQTCLKRVWKAERFSWWMTSIL

HKFSEEEDFGARMQQAELDYVTGSEAGLTTIAENYVGLPYEPLE

>Q1R0P9|Q1R0P9_CHRSD

MKTQVAIIGAGPSGLLLGQLLQRAGINNVILERRSGEYVLSRIRAGVLEQGMVDLLREAGVDRRMDAEGL

PHDGVELAFDNRRVRIDLAALTGGKQVMVYGQTEVTRDLMEARAAEGGQTLYEVDKVQPHDLETDAPYIT

FEHNGETQRLDCDYVAGCDGYHGVSRQSIPADRLKTFERVYPFGWLGLLSDTPPVSDELIYARHERGFAL

CSMRSQTRSRYYVQVPLDEKVEDWSDARFWEELKRRLPEDVAANLVTGPSLEKSIAPLRSFVAEPMQHGR

LFLVGDAAHIVPPTGAKGLNLAASDVNTLYRLMVKVYHEGRTDLVPRYSQTCLKRVWKAERFSWWMTSIL

HKFSEDEDFGARMQQAELDYVTGSEAGLTTIAENYVGLPYEPLE

>A4VJN0|A4VJN0_PSEU5

MKTGVAIIGAGPSGLLLGQLLHNAGIPNVIIERQTPEYVLGRIRAGVLEQGMVNLLREAGVAERLDREGQ

IHEGVELAFGERRVRVDLKALTGGDTVMVYGQTEVTRDLMDARKASAAPIFYGVPDVQLHDLKGEAPWVS

FTLNGETVRLDCDHIAGCDGFHGVSRRSIPAGALTEFERIYPFGWLGLLSDTPPVAEELIYASHERGFAL

CSMRSATRSRYYLQVGLEEKVEDWSDQRFWDELRRRLPEDIAARLITGPSLEKSIAPLRSSVVEPMQYGH

LFLVGDAAHIVPPTGAKGLNLAASDVSTLYRILLKVYREGRVDLLERYSPICLRRIWKAERFSWWMTGLL

HRFPDTDAFARRIQASEQDYFTSTPAALTTIAENYVGLPYEAVQ

>F2MX74|F2MX74_PSEU6

MKTGVAIIGAGPSGLLLGQLLHNASIPNVIIERQTPEYVLGRIRAGVLEQGMVNLLREAGVAERMDREGQ

IHEGVELAFGERRVRVDLKALTGGDTVMVYGQTEVTRDLMDARKASAAPIFYGVPDVQLHDLKGEVPWVS

FTLNGETVRLDCDHIAGCDGFHGVSRRSIPAGALTEFERIYPFGWLGLLSDTPPVAGELIYASHERGFAL

CSMRSATRSRYYLQVGLEEKVEDWSDQRFWDELRRRLPEDVAARLITGPSLEKSIAPLRSSVVEPMQYGH

LFLVGDAAHIVPPTGAKGLNLAASDVSTLYRILLKVYREGRVDLLERYSPICLRRIWKAERFSWWMTSLL

HRFPDTDAFARRIQASKQDYFTSTPAALTTIAENYVGLPYEPVE

>Q06519|Q06519_PSEFL

LKTQVAIIGAGPSGLLLGQLLHNAGIQTLLLERQSADYVQGRIRAGVLEQGMVDLLREAGVSRRMDAEGL

VHDGFELALNGELTHIDLKALTGGQSVMIYGQTEVTRDLMAAREAAGGITLYETQNVQPHGHKTDRPWLT

FEHQGEAFRLECDYIAGCDGFHGVARQSIPAQSLKVFERVYPFGWLGVLADTPPVHDELVYAKHARGFAL

CSMRSPTRSRYYLQVPVEEALDEWSDQRFWDELKTRLPSALAAQLVTGPSIEKSIAPAASFEVEPMQYGR

LFLLGDAAHIVPPTGAKGLNLAASDVSTLFRILLKVYREGRVDLLEQYSAICLRRVWKAERFSWWMTSML

HQFPEADGFSQRIAESELAYFISSEAGRKTIAENYVGLPYEAIE

>C3K4T9|C3K4T9_PSEFS

LKTQVAIIGAGPSGLLLGQLLHNAGIETLILERQSADYVQGRIRAGVLEQGMVDLLRQAGVSQRMDSEGL

VHDGFELALNGQLTHIDLKALTGGQSVMVYGQTEVTRDLMAARADAGAITLYEARDVQPHGLKSDRPWLT

FEHQGEAFRLECDYIAGCDGFHGVARQSIPAESLKVFERVYPFGWLGILADTPPVHAELVYAKHPRGFAL

CSMRSPTRSRYYLQVPVDEPIDEWPDARFWDELKTRLPNHLAEDLVTGPSIEKSIAPLRSFVVEPMQYGR

LFLLGDAAHIVPPTGAKGLNLAASDVSTLYRILLKVYGEGRVDLLERYSAICLRRVWKAERFSWWMTSML

HQFPEADGFSQRIAESELEYFIHSEAGRKTIAENYVGLPYEAIE

>E2XNA5|E2XNA5_PSEFL

LKTQVAIIGAGPSGLLLGQLLHNAGIETLILERQSADYVQSRIRAGVLEQGMVDLLREAGVSQRMDAEGL

VHDGFELALNGRLTHIDLKALTGGQSVMVYGQTEVTRDLMAARAAAGAVTLYEASDVQPHELKSDRPWLT

FEHQGQAYRLDCDYIAGCDGFHGVARQSIPAESLKVFERVYPFGWLGILADTPPVHDELVYAKHPRGFAL

CSMRSPTRSRYYLQVPAEEPLDEWSDARFWAELKTRLPGDLAEQLVTGPSIEKSIAPLRSFVVEPMQYGR

LFLLGDAAHIVPPTGAKGLNLAASDVSTLFRILLKVYGEGRVDLLERYSAICLRRVWKAERFSWWMTSML

HQFPEADGFSQRIAESELEYFIQSEAGRKTIAENYVGLPYEAIE

>Q3K7N9|Q3K7N9_PSEPF

LKTQVAIIGAGPSGLLLGQLLHNAGIDTLILERQTPDYVLGRIRAGVLEQGMVELLREAGVGQRMDAEGL

VHGGFELALDGRRIHIDLQALTGGKTVMVYGQTEVTRDLMAARRETGGQTIYEASHVVPCDVKSDEAYVT

FEKDGETWRVDCDYIAGCDGFHGVARQSIPEDCLKVFERVYPFGWLGILADTPPIHDELVYARHERGFAL

CSMRSATRTRYYLQVPAEENVDDWSDQRFWDELRNRLPEDLAQKLVTGPSIEKSIAPLRSFVVEPMQYGR

MFLVGDAAHIVPPTGAKGLNLAASDVSTLFRILLKVYREGRTELLEKYSQICLRRVWKAERFSWWMTSML

HRFDEHDDFSQRICASELDYFVSSEAGQKTIAENYVGLPYEAIE

>F2K8Q5|F2K8Q5_PSEBN

LKTQVAIIGAGPSGLLLGQLLHNAGIDTVILERQTPEYVLSRIRAGVLEQGMVELLRQAGVGQRMDTEGL

PHDGFELVLNDRRVHIDLKGLTGGKNVMVYGQTEVTRDLMAAREAAGARTLYLASNAQPHDMQTETPFVT

FEHEGETWRLDCDYIAGCDGFHGVARQSIPAEKLKVFERVYPFGWLGVLADTPPVHEELVYARHARGFAL

CSMRSKTRTRYYLQVPAEEQVADWPDERFWDELKNRLPADLAEALVTGPSIEKSIAPLRSFVVEPMQYGR

MFLVGDAAHIVPPTGAKGLNLAASDVSTLFNILLKVYRDGRVDLLEKYSAICLRRVWKAERFSWWMTSML

HRFDD-DAFNQRISEAELEYFVDSEAGRKTIAENYVGLPYEAIE

>Q9X7I6|Q9X7I6_PSESP

MKTQVAIIGAGPAGLLLGQLLHKAGIDTVILERQTPDYVLGRIRAGVLEQGTADLLREAGAGARMDAEGL

LHDGFELVLNGRRERIDLKALTGGKQVMVYGQTEVTRDLMQARAASGAISVYEAADVELHDVTREKPYVT

FTHQGERVRLDCDYIAGCDGFHGVARRSIPAERLKVFERVYPFGWLGLLSDTPPVNHELIYASSERGFSL

CSQRSATRSRYYLQVGLEENVEDWSDERFWEELKRRIPEDAARQLVTGPSLEKSIAPLRSFVVEPLQYGR

LFLIGDAGHIVPPTGAKGLNLAASDVDALHRILVKVYGEGRTDLLEQYSPIALRRIWKAERFSWWMTSML

HRFPDSDAFTRRMLETELDYFVGSEAGRTTIAENYVGLPFEEVA

>A6UY43|A6UY43_PSEA7

MKTQVAIIGAGPSGLLLGQLLHKAGIDNVILERQAPDYVLGRIRAGVLEQGMVDLLREAGVDQRMARDGL

VHEGVEIAFAGQRRRIDLKRLSGGRTVTVYGQTEVTRDLMEARAACGATTIYQAAEVRLHDLEGEHPHVT

FERDGERVRLDCDYIAGCDGFHGVSRQSIPAERLKVFERVYPFGWLGLLADTPPVSHELIYASHPRGFAL

CSQRSATRSRYYVQVPLTEKVEDWSDERFWTELKARLPAEVAQRLVTGPALEKSIAPLRSFVVEPMQHGR

LFLAGDAAHIVPPTGAKGLNLAASDVATLYHLLLKAYREDRADLLERYSAICLRRIWKAERFSWWMTSVL

HRFPDTDAFSQRIQQTELEYYLGSEAGLATIAENYVGLPYEAVE

>Q02UI8|Q02UI8_PSEAB

MKTQVAIIGAGPSGLLLGQLLHKAGIDNVILERQTPDYVLGRIRAGVLEQGMVDLLREAGVDRRMARDGL

VHEGVEIAFAGQRRRINLKRLSGGKTVTVYGQTEVTRDLMEAREACGATTVYQAAEVRLHDLQGERPYVT

FERDGERLRLDCDYIAGCDGFHGISRQSIPAERLKVFERVYPFGWLGLLADTPPVSHELIYANHPRGFAL

CSQRSATRSRYYVQVPLTEKVEDWSDERFWTELKARLPAEVAEKLVTGPSLEKSIAPLRSFVVEPMQHGR

LFLAGDAAHIVPPTGAKGLNLAASDVSTLYRLLLKAYREGRGELLERYSAICLRRIWKAERFSWWMTSVL

HRFPDTDAFSQRIQQTELEYYLGSEAGLATIAENYVGLPYEEIE

>P20586|PHHY_PSEAE

MKTQVAIIGAGPSGLLLGQLLHKAGIDNVILERQTPDYVLGRIRAGVLEQGMVDLLREAGVDRRMARDGL

VHEGVEIAFAGQRRRIDLKRLSGGKTVTVYGQTEVTRDLMEAREACGATTVYQAAEVRLHDLQGERPYVT

FERDGERLRLDCDYIAGCDGFHGISRQSIPAERLKVFERVYPFGWLGLLADTPPVSHELIYANHPRGFAL

CSQRSATRSRYYVQVPLSEKVEDWSDERFWTELKARLPSEVAEKLVTGPSLEKSIAPLRSFVVEPMQHGR

LFLAGDAAHIVPPTGAKGLNLAASDVSTLYRLLLKAYREGRGELLERYSAICLRRIWKAERFSWWMTSVL

HRFPDTDAFSQRIQQTELEYYLGSEAGLATIAENYVGLPYEEIE

>Q4ZQP0|Q4ZQP0_PSEU2

MKTKVAIIGSGPSGLLLGQLLQRAGIDNVIVERKDPDYILSRIRAGVLEQGMTDLLREAGVSERMDAEGM

IHDGFELAFAGRCERIDLKSHADGRTVMVYGQTEVTRDLMAARAATGAMTIYNATDVKTHDLKSDSPYLT

FVKDGETVRLDCDYIAGCDGFHGVSRQSIPADALKVFERVYPFGWLGVLADTPPVNEELVYANHPRGFAL

CSMRSAIRTRYYVQVSADEKVEDWSDERFWDELKSRLPEHLAERLVTGPSIEKSIAPLRSFVVEPMQYGR

LFLLGDAAHIVPPTGAKGLNLAASDVSTLYRILLKVYQEGRTDLLEKYSQICLRRVWKAERFSWWMTSVL

HNFPDTDAFSQRIQQTELDYYVGSEAGRRTIAENYVGLPYEAVE

>F2ZI54|F2ZI54_9PSED

MKTQVAIIGSGPSGLLLGQLLQRAGIDNVIVERKNPDYILSRIRAGVLEQGMTDLLREAGVSERMDAEGL

IHDGFELAFDGRCERIDLKNLAGGKTVTVYGQTEVTRDLMAARASIGAMTVYDAADVNVHDPKTDSPYLT

FVKDGETVRLDCDYIAGCDGFHGVSRQSIPSEALKIFERVYPFGWLGVLADTPPVNEELVYANHPRGFAL

CSMRSAIRTRYYVQVSADEKVEDWSDDRFWGELKARLPAHLAKRLVTGPSIEKSIAPLRSFVVEPMQYGR

LFLLGDAAHIVPPTGAKGLNLAASDVSTLYRILLKVYREGRTDLLEKYSQICLRRVWKAERFSWWMTSVL

HNFPGTDAFSQRIQHTELDYYVGSEAGRRTIAENYVGLPYEAIE

>F3F3G5|F3F3G5_9PSED

MKTQVAIIGSGPSGLLLGQLLQRAGIDNVIVERKDPDYILSRIRAGVLEQGMTDLLREAGVSERMDAEGL

IHGGFELAFDGRCERIDLKTLTGGRTVMVYGQTEVTRDLMAARAAAGAMTVYDACDVNIHDPKTDSPYVT

FVKDGETVRLDCDYIAGCDGFHGVSRQSIPSETLKIFERVYPFGWLGVLADTPPVNEELVYANHPRGFAL

CSMRSAIRTRYYVQVSAEEKVEDWWDERFWTELKARLPEHLADRLVTGPSIEKSIAPLRSFVVEPMQYGR

LFLLGDAAHIVPPTGAKGLNLAASDVSTLYRILLKVYREGRTDLLEKYSQICLRRVWKAERFSWWMTSVL

HNFPDTDAFSQRIQQTELDYYVGSQAGRRTIAENYVGLPYEAIE

>Q885C8|Q885C8_PSESM

MKTQVAIIGSGPSGLLLGQLLQRAGIDNVIVERKNPDYILSRIRAGVLEQGMIDLLREAGVSERMDAEGL

IHDGFELAFDGRCERIDLKSLADGKTVMVYGQTEVTRDLMKARAAIGAMTVYDASDVNIHAPKTDSPYLT

FVKDSETVRLDCDYIAGCDGFHGVSRQSIPSQALKIFERVYPFGWLGVLADTPPVNEELVYANHPRGFAL

CSMRSAIRTRYYVQVSADEKVEDWSDERFWTELKSRLPAHLADRLVTGPSIEKSIAPLRSFVVEPMQYGR

LFLLGDAAHIVPPTGAKGLNLAASDVSTLYRILLKVYREGRTDLLEKYSHICLRRVWKAERFSWWMTSVL

HNFPDTDAFSQRIQQTELDYYVGSEAGRRTIAENYVGLPYEAIE

>F3DVU0|F3DVU0_9PSED

MKTQVAIIGSGPSGLLLGQLLQRAGIDNVIVERKNPDYILSRIRAGVLEQGMVDLLREAGVSERMDAEGL

IHDGFELAFDGRCERIDLKSLADGKTVMVYGQTEVTRDLMAARAATGAMTVYDASDVKIHEPKTDNPYLT

FVKDGETVRLDCDYIAGCDGFHGVSRQSIPAEALKIFERVYPFGWLGVLADTPPVNDELVYANHPRGFAL

CSMRSAIRTRYYVQVSADEKVEDWSDERFWTELKSRLPAHLAGRLVTGPSIEKSIAPLRSFVVEPMQYGR

LFLLGDAAHIVPPTGAKGLNLAASDVSTLYRILLKVYREGRTDLLEQYSHICLRRVWKAERFSWWMTSVL

HNFPDTDAFSQRIQQTELDYYVGSEAGRRTIAENYVGLPYEAIE

>Q9F5W0|Q9F5W0_9PSED

MKTQVAIIGAGPSGLLLGQLLHKAGIDNVIIERQSPDYVLGRIRAGVLEQGMVDLLREAGVGQRMDREGL

VHDGFELAFDGRLERIDLRTLTGGKTVMVYGQTEVTRDLMEARAASGAPCFYEASEVELHELKGENPHVT

FLHQGQRMRLDCQQIAGCDGFHGVSRKSIPAGVLSEFERVYPFGWLGVLADTPPVNDELIYANHERGFAL

CSMRSPTRTRYYVQVGAEEKVEDWSDERFWDELKRRLPEQTAAKLVTGPSIEKSIAPLRSFVVEPMQYGH

LFLVGDAAHIVPPTGAKGLNLAASDVSTLYRILLKVHQEGRNDLLEKYSSICLRRIWKAERFSWWMTSML

HRFPDTDAFSQRMQQTELDYYVGSEAGRRTIAENYVGLPYEAVE

>F6AJ58|F6AJ58_PSEF1

MKTSVAIIGAGPSGLLLGQLLHNAGIDTVILERQSPVYVLGRIRAGVLEQGMTDLLREAGVGARMEREGL

VHDGFELAFDGRRERIDLKGLTGGKTVMIYGQTEVTRDLMQAREASGAVTHYEVSDVQLHDLKDGAPYVT

YMKDGQAQRLDCDYIAGCDGYHGVSRQSIPADTLKVFERVYPFGWLGVLADTPPVAEELIYASHPRGFAL

CSMRSPTRTRYYVQVDADEKVEDWSDERFWAELKSRLPADTAANLVTGPSIEKSIAPLRSFVVEPMQYGR

LFLLGDAAHIVPPTGAKGLNLAASDVSTLYRILLKVYREGRTDLLERYSAICLRRIWKAERFSWWMTSLL

HNFPDTDAFTARMRQTELDYFVGSEAGRTSIAENYVGLPYEPIE

>Q15NZ5|Q15NZ5_PSEA6

VKTKVAIIGAGPSGLLLGQLLAKQGIDNIIVERVSGEYVLGRIRAGILEQGLVDLLREANVNERMDGEGH

VHDGFEISYYGTPYRIDLNKLTDGKTVMCYGQTEVTRDLMQARETKALTTYYSASDVSLHDIESAHPTVT

FSQDGVNYTLECDYIAGCDGFHGVSRKSIPEEKRNEFERVYPFGWLGLLSDTPPVSDELIYCKTERGFAL

ASMRSSSRSRYYLQVPLTDKVEQWSDERFWDELRKRLPQEAASNLITGPSLEKSIAPLRSFVCEPMQLGK

LFLVGDAAHIVPPTGAKGLNLAASDVSTLYRLLTKEYNEGATNASQQYSEIALRRVWHAERFSWWMSNML

HEFTDANNMDERFMASELDFYLSHQEGQQVIATQYVGMPYSDV-

>B8KHQ0|B8KHQ0_9GAMM

LKTQVAIIGAGPAGLLLGQLLAKQGISNLILERVSGDYVLGRIRAGILEQGLANLVREAGVAERMDAEGE

VHEGVEFAVDGVRKHIDLKGLTGGDVVICYGQTELTKDLMDARQAAGLLTCYEAANVQLHDIAESTPSVT

FDYEGEKHSLCCDYIAGCDGFHGTSRQSIPADKRTEYERVYPFGWLGLLSDTPPASEELIYCKSERGFAL

ASRRSATRSRYYLQVPLTDNVEEWSDDDVWAELKRRLPQDVAENMVTGPSLEKSIAPLRSFVCEPMQYGR

LFLVGDAAHIVPPTGAKGLNLAASDVHTLYKVLTRVYQENDTACIARYSEVALKRVWHGERFSWWLSNML

HDYDEGSGVDDRFMDSERDYFLNTEAGRRVLATQYVGLPYEEV-

>A4BGY4|A4BGY4_9GAMM

IKTQVAIIGAGPSGLLLGQLLHKQGIQNIIVERVTGDYVLGRIRAGILEQGFADLVREAGVSENMDKFGD

VHEGFEIAVGDERIRINMAGLTGGKTVVCYGQVDITKDLMDARAEAGLETLYEVESTELHDVDSETPSVT

FIQNGEPVTIKCDYIAGCDGFHGVSRQTIPETLRTEHERVYPFGWLGLLSDTPPVSPELIYCKTPRGFAL

ASMRSETRSRYYLQVPLTDKVEDWSDDKFWTELKKRLPADAASKLVTGPSIEKSIAPLRSFVCEPMQYGN

LFLVGDAAHIVPPTGAKGLNLAASDVATLYKILTKVYETGDKRYLQQYSEIALRRVWHGERFSWWMSNML

HDYNDTKETFTRFMESELSYYTGTEEGRKVIAQQYVGLPYEELS

>F2JUE7|F2JUE7_MARM1

MKTKVAIIGAGPSGLLLGQLLAKQGIDNVIIERVTGEYILGRIRAGVLEQGMVNLLREAGVSERMDKEGE

VHDGFELAFNNKRVRIALDELTGGDTVMVYGQTEVTRDLMEARAKAGYTTVYEASDVKLHDVKSDTPYVT

FEKNGEQVRLDCDYIAGCDGFHGVSRKTIPDDVKTEFERVYPFGWLGLLSDTKPAHDELIYCKTDRGFAL

ASMRSQTRSRYYLQVPLTDKVENWSDEAFWEELKKRLPDDVASKMQTGPSIEKSIAPLRSFVCEPMQYGN

LFLVGDAAHIVPPTGAKGLNLAASDVATLYKIMTRVYKENDKDCINQYSEICLRRVWNGERFSWWMTNMM

HDFADAITFD-RFMSSELNFYTDNEEGRKVVAMQYVGLPYEDLK

>A6VT62|A6VT62_MARMS

MKTQVAIIGAGPSGLLLGQLLAKQGIDNVIIERVSGEYILGRIRAGVLEQGMTDLLREAGVGERMDREGQ

IHHGVELAFNNKRVQIELEKLTGGSTVMVYGQTEVTRDLMEARDGAGLTTYYESSNVALHDVKSDAPYVT

FEHNGETHRLDCDYIAGCDGFHGVSRQTIPESSRKEFERVYPFGWLGVLSDTPPVNPELIYCKTDRGFAM

TSMRSETRSRYYLQVPLTDKVEDWSDDDFWTELKRRLPDDVAEKLVTGPSIEKSIAPLRSFVCEPMQYGN

LFLVGDAAHIVPPTGAKGLNLAASDVATLYKVMTRVYKEGDKECISQYSDICLRRVWHGERFSWWMTNML

HDFGDEHDNDGKFMSSELNFYTDNEEGRRIIAMQYVGLPYEDLV

>F6D1D1|F6D1D1_MARPP

MKTQVAIIGAGPSGLLLGQLLAKQGIDNVIIERVSGDYILGRIRAGVLEQGMADLLREAGVGERMDREGQ

VHKGVELAFNNKRVNIELEELTGGNTVMVYGQTEVTRDLMEARAEKGLTTYYESSNVVLHDVKSASPYVT

FEQDGKEHRLDCDYIAGCDGFHGVSRQTIPESSRKEFERVYPFGWLGILSDTPPVNDELIYCKTDRGFAM

TSMRSATRSRYYIQVPLTDKVEDWSDDDFWTELKHRLPDDVAERLVTGPSIEKSIAPLRSFVCEPMQYGN

LFLVGDAAHIVPPTGAKGLNLAASDVATLNKIMTRVYKEGDKECINQYSDICLRRVWHGERFSWWMTNML

HDFSGEGSKDERFMSSELNFYTDTEEGRRVIAMQYVGLPYEDLK

>F2J586|F2J586_POLGS

MRTQVAIIGAGPSGLLLSQLLHLAGIDCVVLEQRSGDYVLSRIRAGVLEQGMVDMIDRAGVGARMHKEGL

LHDGFTMAVGDTRLRMDLKGLT-GSSVMVYGQTEVTRDLMDAHAARDAKVIYEAEAVTPLDFDTDHPRVT

YVKDGVTHEIACDFICGCDGFHGVCRKSVPEGAITEFEKVYPVGWLGILSHVRPVSHELIYANHDRGFAL

ASMRSHTLSRYYIQVPLDSPVEAWSDEAIWDELKIRLGEEAAASMETGPSIEKSIAPLRSFVVEPLRFGR

MMLAGDAGHIVPPTGAKGLNLAASDIHYLSTGLIEYYADKSAAGIDAYSQKALARIWKAERFSWWMTMLL

HKFPDLGAFNAKMQQADMDYLASSTAAQTSLAENYVGLPF----

>A7IIT2|A7IIT2_XANP2

MRHQVCIIGAGPSGLLLGQLLAKAGVDAVILEARSADYVLSRIRAGVLESGLVDLMRQAGCAERLDREGL

VHDGFHLAFAGRTHRIDLKGLSGGKSVVVYGQTEITRDLMEARAALGVPTIYEAQDVALHDFDGPRPRVT

YHKDGVTHEITCDFIAGCDGFHGIARRSVPETAIAQFERVYAFGWLGLLADVPPASNELIYARHERGFAL

ASMRSHTRSRYYIQVPLSDQVEDWSDERFYDEFRRRLPEEVAARVVPGASMEKSIAPLRSFVAEPLRFGR

LFLAGDAGHIVPPTGAKGLNLAASDVHYLASGLIEHYRDKSDAGIDAYSERALRRIWKAERFSWWMTSML

HLFPEAGDFVARMQLAELDYIAGSEAAATTVAENYVGLPF----

>A8HX00|A8HX00_AZOC5

MRTQVAIIGSGPSGLLLGRLLTLAGIDNVIIDRVGRDYILGRVRAGVLEQGLVDMMREAKAAERLEAEGL

PHAGFDIAVDGALHHIDLKGLTGGKTVTVYGQTELTRDLMDKRAGDGVTTIYDAQNVTPFDFDGDRPWVS

YEKDGVPGRIDADFIIGCDGFHGVSRKSAPSRAIETFERVYPFGWLGVLADVPPAREELVYGRHSRGFTL

CSMRSRTRSRYYIQVPLADQVEDWSDDAFWDELRRRLPEDVADAIVTGPSFEKSIAPLRSFVAEPMRFGT

LFLAGDAAHIVPPTGAKGLNLAASDIRYLFDGLREFYKDGSRAGIDAYSARALARVWKAERFSWWMTTML

HTFTEVDAFGRRIHDAEIAYTLSSHAARAALAENYVGLPY----

>Q986T9|Q986T9_RHILO

MRTQVAIIGSGPSGLLLGQLLAGIGVETIILERSSREHVLGRVRAGVLEQGTVELLEEAGVAARLHAEGM

SHTGISLAFDGRLHRIDLAALTGGKHVTVYGQTEVTHDLMDKREAAGLVTIYEAADVALHDFDGAAPFVT

YAKDGISHRVDCDFIAGCDGYHGVSRKSVPSAALKTFERQYPFGWLGVLAEVPPADHELVYANHERGFAL

CSMRSTHRSRYYVQCPQDDHVEAWSDDRFWDELRRRLPERTAASVTTGPSFEKSIAPLRSFVAEPMRFGR

LFLVGDAAHIVPPTGAKGLNLAASDVRYLFTGLREFYVGKSQAGIEAYSQKALARVWKAVRFSWWMTTML

HRFPDTGDFGQRIQEAELDYLVHSQAASTALAENYVGLPY----

>E8TB39|E8TB39_MESCW

MRTQIVIVGSGPSGLLLGQLLAGIGVETVILERASREHVLGRVRAGVLEQGTVELLEQAGAAARLHAEGL

PHSGISLAFDGRLHRIDLEALTGGRHVTVYGQTEVTHDLMDKRDAEGLTTIYEAANVALHDFDGAATFAT

YDKDGVTHRIDCDFIAGCDGYHGVSRKSVPERALKIFERQYPFGWLGVLAEVPPADHELVYANHERGFAL

CSMRSTHRSRYYVQCPQDDRVEAWPDDRFWDELRRRLPEQTAASVVTGPSFEKSIAPLRSFVAEPMRFGR

LFLVGDAAHIVPPTGAKGLNLAASDVRYLFAGFRDFYIEKSSVGIDAYSGKALARVWKAVRFSWWMTTML

HRFPDTGEFGQRIQEAELDYLVQSRAASTALAENYVGLPY----

>F7YF73|F7YF73_MESOW

MRTQVVIVGSGPSGLLLGQLLAGIGVETVILERSSREHVLGRVRAGVLEQGTVELLEQAGAAARLHTEGL

PHSGISLAFDGRLHRIDLSALTGGKHVTVYGQTEVTHDLMDKREAAGLVTVYEAADVALQDFDGAAPFVT

YDRDGVSHRIDCDFIAGCDGYHGVSRKSVPESALKIFERHYPFGWLGVLAEVPPADHELVYANHERGFAL

CSMRSTHRSRYYVQCPEGDRVGAWSDERFWDELRRRLPERTAASVVTGPSFEKSIAPLRSFVAEPMRFGR

LFLVGDAAHIVPPTGAKGLNLAASDVRYLFAGLRDFYAEKSNAGIDAYSQKALARVWKAVRFSWWMTTIL

HRFPDTGDFGQRIQEAELDYLVHSRAASTALAENYVGLPY----

>D1D157|D1D157_9RHIZ

MRTKVVIIGSGPAGLLLGQLLTRAGIDNVILDRVSENYILGRVRAGVLEEGTVRLMDEAGASDRLHREGL

PHDGFSLAFDGRDHRIDLHKLT-GKRVTVYGQTELTHDLMSERRRSGAHGIYEAGNITPHDFDTASPYVT

YEKDGIMHRIDCDYIAGCDGYHGVSRKSVPQKAIRIFEKVYPFGWLGVLADIPPVDHELIYANHERGFAL

CSMRSLTRSRYYIQCALDEKVEDWSDERFYDELRRRLPAHHAEAMVTGPSFEKTIAPLRSFVAEPMRFGR

LFLAGDAAHIVPPTGAKGLNLAASDVHYLFEGLREFYIENSIAGINAYSARALARIWKAERFSWSMTKML

HRFPDMGPFDQKVQEAELDYFCNSQAASTALAENYVGLPF----

>C0RLF6|C0RLF6_BRUMB

MRTKVVIIGSGPAGLLLGQLLTRAGIDNVILDRVSENYILGRVRAGVLEEGTVRLMDEAGASDRLHREGL

PHNGFSLAFDGRDHRIDLHKLT-GKRVTVYGQTELTHDLMSERHRSGAHGIYEADNITPHDFDTASPYVT

YEKDGITHRIDCDYIAGCDGYHGVSRKSVPQKAIRIFEKVYPFGWLGVLADIPPVDHELIYANHERGFAL

CSMRSLTRSRYYIQYALDEKVEDWSDERFYDELRRRLPAHHAEAMVTGPSFEKTIAPLRSFVAEPMRFGR

LFLAGDAAHIVPPTGAKGLNLAASDVHYLFEGFCANFISKIQPPASMPIRRALARIWKAERFSWSMTKML

HRFPDMGPFDQKVQEAELDYFCNSQAASTALAENYVGLPF----

>A6X5C5|A6X5C5_OCHA4

MRTQVAIIGSGPAGLLLGQLLTKAGIDNVVLDRVSADYILGRVRAGVLEEGTVNLMDEAEASTRLHAEGL

PHDGFSLAFDGRDHRLDLHKLT-GKRVTVYGQTEMTHDLMDERRKSSTTSIYEAANVTPHDFDTTSPYIT

YEKGSKTHRIDCDFIAGCDGYHGISRKSVPDKAIDIFEKVYPFGWLGILADIPPVAHELIYANHERGFAL

CSMRSLTRSRYYIQCSLDEKIEDWSDERFYDELRCRLPVHHAEAMITGPSFEKSIAPLRSFVAEPMRFGQ

LFLVGDAAHIVPPTGAKGLNLAASDVHYLYEGLREFYLDKSKSGIDAYSANALARIWKAERFSWSMTKML

HRFPDMVPFDQKVQEAELDYFCTSEAASTALAENYVGLPF----

>C4WLU0|C4WLU0_9RHIZ

MRTQVAIIGSGPSGLLLGQLLTKAGIDNIVLDRVSADYILGRVRAGVLEEGTVKLMDEAGASARLHAEGL

LHDGFSLAFDGRDHRIDLHEMT-GKRVTVYGQTEMTHDLMDERQKSNAASIYEAADVTPHDFDTSSPYVT

FEKEGRTHRIDCDFIAGCDGFHGVSRKSVPEKAINIFEKIYPFGWLGILADIPPVAHELIYANHKRGFAL

CSMRSLTRSRYYIQCSLDETIEGWSDQRFYDELRRRLPAHHAEAMVTGPSFEKSIAPLRSFVVEPMRFGR

LFLVGDAAHIVPPTGAKGLNLAASDVHYLYEGLRDFYLDRSEAGINAYSGKALARIWKAERFSWSMTKLL

HRFPDMAPFDEKVQEAELDYFCTSHAASTALAENYVGLPF----

>B9K2M7|B9K2M7_AGRVS

MRTQVVIIGSGPSGLLLGQLLHLKGIETVIIDRVGRDYILGRVRAGVLEQGMVGMLEKAGAADRLHREGL

PHDGFSLAFDGRDHRIDLFNLTGGDRVMVYGQTEVTRDLMDQRDAAGALTIYDAANVEPHDFSGESPYVT

YEKDGVSHRIDCDFIAGCDGFHGVSRKSVPQGAIKEFERIYPFGWLGVMAEVPPVAHELIYANHPRGFAL

CSMRSNTRSRYYVQCPLDDKVEDWSDDRFWDELRRRLPAEHAEAMVTGPSFEKSIAPLRSFVTEPMRFGR

LFLAGDAAHIVPPTGAKGLNLAASDIHYLSEGLIEFYGDKSSAGIDDYSVRALARVWKAVRFSWWMTTMM

HRFPDTGDFGQRIQEAELDYLVHSRAASTSLAENYVGLPY----

>F5JJV7|F5JJV7_9RHIZ

MRTQVVIIGSGPSGLLLGQLLARAGVETVILDRVSKDYILGRVRAGVLEEGTVQLMEKVGADKRLRREGL

PHDGFSLTFDGRDHRIDLFDLTGGKRVMVYGQTEVTHDLMDVREAANLVTIYDAANVEPHDFDGASPYVT

YQKDGVNHRIDCDFIAGCDGFHGVSRKSVPDGAIKQFEKVYPFGWLGILADVPPVNHELIYANHPRGFAL

CSMRSNTRSRYYIQCSLDDRPEDWSDERFWDEIRRRLPESHADVMVTGASFEKSIAPLRSFVSEPMRFGR

LFLAGDAAHIVPPTGAKGLNLAASDVHYLSEALIEFYRDRSEAGIDAYSQKALSRVWKAVRFSWWMTTMM

HRFPDTEDFGQRIQEAELDYLVQSRAASTALAENYVGLPY----

>Q7CV80|Q7CV80_AGRT5

MRTQVVIIGSGPSGLLLGQLLAGAGVETVILDRVSKDYILGRVRAGVLEEGTVQLMEKVGADKRLHREGL

PHDGFSLTFDGRDHRIDLFDLTAGKRVMVYGQTEVTHDLMDVREAANLVTIYDAGNVEPHDFDGAGPYVT

YQKDGVNHRIDCDFIAGCDGFHGVSRKSVPDGAIKEFEKVYPFGWLGILADVPPVNHELIYANHPRGFAL

CSMRSHTRSRYYIQCSLDDRPEDWSDERFFDEIRRRLPENHADAMVTGPSFEKSIAPLRSFVSEPMRFGR

LFLAGDAAHIVPPTGAKGLNLAASDVHYLSEALIEFYRDRSEAGIDAYSQKALSRVWKAVRFSWWMTTMM

HRFPDTGDFGQRIQEAELDYLVQSRAASTALAENYVGLPY----

>F0LB18|F0LB18_AGRSH

MRTKVAIIGSGPSGLLLGQLLANEGIDTVILDRAGKDYILGRVRAGVLEEGTVHLLEKVGADKRLHKEGL

PHDGFSLAFDGRDHRIDLFDLTGGKRVMVYGQTEVTHDLMDAREKAGLTTIYEASNVEPHDFEGTSPYVT

YEKDGVSHRIDCDFIAGCDGFHGVSRKSVPERSIKEFEKIYPFGWLGILAEVPPVSHELIYANHPSGFAL

CSMRSHTRSRYYLQCSLEDRPEDWSDERFWDEIRRRLPENHADALVTGPSFEKSIAPLRSFVCEPMRFGR

LFLAGDAAHIVPPTGAKGLNLAASDVHYLSEALIEFYREGSEAGIDSYSQKALSRVWKAVRFSWCMTTMM

HRFPDTGEFGQRIQEAELDYLVHSRAASTALAENYVGLPF----

>F7UBM0|F7UBM0_RHIRD

MRTKVVIIGSGPSGLLLGQLLAKEGIDTVILDRAGKDYILGRVRAGVLEEGTVHLLEKAGADKRLRKEGL

PHDGFSLTFDGRDHRVDLFDLTGGKRVMVYGQTEVTHDLMDVREKAGLLTIYEAANVEPHAFDGASPYVT

YEKDGVTHRIDCDFIAGCDGFHGVSRKSVPEGAIKEFEKVYPFGWLGILAEVPPVSHELIYANHPRGFAL

CSMRSHTRSRYYLQCSLEDRPEDWSDERFWDEIRRRLPENHADALVTGPSFEKSIAPLRSFVCEPMRFGR

LFLAGDAAHIVPPTGAKGLNLAASDVHYLSEALIEFYGEVSEAGIDSYSQKALSRVWKAVRFSWWMTTMM

HRFPDTADFGQRIQEAELDYLVHSRAASTALAENYVGLPY----

>C3KRK3|C3KRK3_RHISN

MRTQVVIIGSGPSGLLLGQLLTLAGIDNLILDRASKEHILGRVRAGVLEEGTVRLMDEAHSGARMHGEGL

PHEGFSLAFDGRDHRIDLSGLTGGKRVLIYGQTELTRDLMDHRQAAGALSIYEAANATPCDFDSHAPYVT

YEKDGVTHRIDCDFIAGCDGFHGVSRRSVPDRAIRAFEKIYPFGWLGILADVPPVDHELVYANHPRGFAL

CSMRSLSRSRYYIQCSLDEKLDGWDDQRFWDELRRRLPVHHADRVVTGPSFEKSIAPLRSFVAEPMRFNR

LFLVGDAAHIVPPTGAKGLNLAASDVHYLFEGLLEHYQDRSNASIDAYSARALARVWKAVRFSWWMTTMM

HRFPDTSDFDQKIQEAELDYLTHSRAAATALAENYVGLPF----

>A6UH77|A6UH77_SINMW

MRTQVVIIGSGPSGLLLGQLLARAGIANVIVDHASKDHILGRVRAGVLEEGTVRLMDEAGSGARMHAEGI

AHDGFSLAFDGRDHRIDLFGLTGGRRVMIYGQMELTRDLMDQRERAGAPSVYEAESVTPRDFEGDAPHVA

YRKNGIAHRIDCDFIAGCDGFHGVSRRSVPQNAFRSFEKVYPFGWLGILADVPPVDDELVYANHPRGFAL

CSMRSHTRSRHYIQCPLGEKIGDWSDQRFWDELRRRLPAHHAERVVTGPSFEKSIAPLRSFVAEPMRFNR

LFLAGDAAHIVPPTGAKGLNLAASDVHYLFEAFVEHYQDRSNAGIDAYSARALARVWKAVRFSWWMTTIL

HRFPETSEFDQKIQEAELAYLTHSSAAATALAENYVGLPF----

>F6E6V9|F6E6V9_SINMK

MRTQVVIIGSGPSGLLLGQLLTGAGIANVILDRATKDHILGRVRAGVLEEGTVRLMEEAGCGARMHAEGL

PHDGFSLAFDGRDHRIDLFGLTGGRRVMIYGQTELTRDLMDHRERVGALSIYEAANVMPRDFDGRTPHVA

YEKDGIAQRIDCDFIAGCDGFHGVSRRSLPEKAIRNFEKIYPFGWLGILADVPPVDHELVYANHPRGFAL

CSMRSHTRSRYYIQCPLEEKIEDWDDQRFWDELRRRLPAHHAERVVTGPSFEKSIAPLRSFVAEPMRFNR

LFLAGDAAHIVPPTGAKGLNLAASDVHYLFEGLLEHYQDRSNAGIDAYSARALARVWKAVRFSWWMTTML

HRFPETSDFDQRIQEAELDYLTHSRAAATALAENYVGLPF----

>B9JKT2|B9JKT2_AGRRK

MRTQVAIIGSGPSGLLLGQLLTEAGIDNVILDRVGKDYILGRVRAGVLEEGTVWLMDKAQSATRLHAEGL

PHDGFSLAFDGRDHRIDLHGLTGGKCVMIYGQTELTRDLMARREQSGGLTIYDAANVCPHEFDGAAPYLT

YEKDGVAHRIDCDFIAGCDGFHGVSRKSVPEKAIRCFEKVYPFGWLGLLADAPPVNHELIYANHPRGFAL

CSMRSMTRSRYYIQCPLEEKIENWSDDRFWDELRRRLPAHHAEALITAPSFEKSIAPLRSFVAEPMRFGR

LFLVGDAAHIVPPTGAKGLNLAASDVHYLFSGLAEHYHDRSNAGLDAYSAHALARVWKAVRFSWWMTTMM

HRFPDTGDFDQKIQEAELDYLTHSRAASTALAENYVGLPF----

>Q2K4N5|Q2K4N5_RHIEC

LRTQVAIIGSGPSGLLLGQLLTEAGVDNVILDRVSKNYILGRVRAGVLEEGTVGLLDEAKAGARLHAEGL

PHDGFSLAFDGRDHRIDLHELT-GRRVTVYGQTEVTRDLMQRREACGSLSIYDAADVAPHDVDGGSPFVT

YTKDGIGHRIDCDFIAGCDGFHGTSRRALPEGAVRSFEKVYPFGWLGLLADVAPVSNELIYANHPRGFAL

CSMRSATRSRYYIQCPLDAKIEDWSDDRFWDELRRRLPAHHAEALTTAPSFEKSIAPLRSFVTEPMRFGR

LFLVGDAAHIVPPTGAKGLNLAASDVHYLFGGLLEHYREGSDSGIDAYSQTALTRVWKAVRFSWWMTTMM

HRFPDTSDFDQKIQEAELDYLTHSRAASMVLAENYVGLPF----

>B3PYV7|B3PYV7_RHIE6

MRTQVAIIGSGPSGLLLGQLLTEAGIDNVILDRVNKDYILGRVRAGVLEEGTVGLLDEAKAGARLHAEGL

PHDGFSLAFDGRDHRIDLHELTGGRRVTVYGQTEVTRDLMERREASGALSVYDAVDVTPHDFDGRSPFVT

YIKDGAAKRIDCDFIAGCDGFHGASRKAVPEGAIRSFEKVYPFGWLGLLADVAPVSHELIYANHPRGFAL

CSMRSATRSRYYLQCPLDERVEDWSDDRFWDELRRRLPAHHAEALKTAPSFEKSIAPLRSFVAEPMRFGR

LFLVGDAAHIVPPTGAKGLNLAASDVHYLFSGLIEHYREGSDHGIHAYSQVALARVWKSVRFSWWMTTMM

HRFPDTSDFDQRIQEAELDYLTHSRAASTVLAENYVGLPF----

>F2A6M0|F2A6M0_RHIET

MRTQVAIIGSGPSGLLLGQLLTEAGIDNVILDRVNKDYILGRVRAGVLEEGTVGLLDEAKAGARLHAEGL

PHDGFSLAFDGRDHRIDLHELTGGRRVTVYGQTEVTRDLMERREASGALSIYDAADVTPHGFDGHSPFVT

YTKDGAARRIDCDFIAGCDGFHGASRKAVPERAIKSFEKVYPFGWLGLLAEVAPVSPELIYANHPRGFAL

CSMRSATRSRYYIQCPLDEKVEDWSDHRFWDELRRRLPPHHAEALKTAPSFEKSIAPLRSFVAEPMRFGR

LFLVGDAAHIVPPTGAKGLNLAASDVHYLFSGLIEHYREGSDHGIDAYSQLALARVWKSVRFSWWMTTMM

HRFPDSGDFDQRIQEAELDYLTHSRAASTVLAENYVGLPF----

>B5ZNK1|B5ZNK1_RHILW

MRTQVAIIGSGPSGLLLGQLLTEAGIDNVILDRVNKDYILGRVRAGVLEEGTVRLLDQAKAGARLHAEGL

PHDGFSLAFDGRDHRIDLHDLTGGRRVTVYGQTEVTRDLMERREASGALSIYDAADVTPHDFDGRLAFVT

YTKGGVAQRIDCDFIAGCDGFHGASRKAVPERAIRHFEKIYPFGWLGLLADVAPVSHELIYANHPRGFAL

CSMRSATRSRYYVQCALDEKIEDWSDDRFWDELRRRLPTHHAEALLTAPSFEKSIAPLRSFVAEPMRFGR

LFLVGDAAHIVPPTGAKGLNLAASDVHYLFSGLIEHYREGSDHGVDAYSQTALARVWKAVRFSWWMTTMM

HRFPDTGDFDQKIQEAELDYLTHSRAASTALAENYVGLPF----

>Q1MCD6|Q1MCD6_RHIL3

MRTQIAIIGSGPSGLLLGQLLTEAGIDNVIIDRVNKDYILGRVRAGVLEEGTVGLLDQAKAGTRLHAEGL

PHDGFSLAFDGRDHRIDLHELTGGRRVTVYGQTEVTRDLMERREASGSLSIYDAVDVAPHDFDGHSPFVT

YVKDGVAKRIDCDFIAGCDGFHGVSRKAVPERAIRSFEKVYPFGWLGVLADVAPVSHELIYANHPRGFAL

CSMRSATRSRYYIQCALDEKIENWSDDRFWDELRRRLPVHHAEALPTAPSFEKSIAPLRSFVAEPMRFGR

LFLVGDAAHIVPPTGAKGLNLAASDVHYLFSGLIEHYREGSDGGIDAYSQKALARVWKAVRFSWWMTTMM

HRFPDTGDFDQKIQEAELDYLTHSRAASTALAENYVGLPF----

>Q59744|Q59744_RHILE

MRTQVAIIGSGPSGLLLGQLLTEAGIDNVILDRVNKDYILGRVRAGVLEEGTVGLLDQARSGARLHAEGL

PHDGFSLAFDGRDHRIDLHELTGGRRVTVYGQTEVTRDLMERREESGSLSIYDAVDVAPHDFDGPSPFVT

YVKDGVAKRIDCDFIAGCDGFHGASRKAVPERAIRSFEKIYPFGWLGILADVAPVSHELIYANHPRGFAL

CSMRSATRSRYYIQCTLDEKIDDWSDDRFWDELRRRLPTHHAEALATAPSFEKSIAPLRSFVAEPMRFGR

LFLVGDAAHIVPPTGAKGLNLAASDVHYLFSGLIEHYREGSNSGIDAYSHKALARVWKAVRFSWWMTTMM

HLFPDTGDFDQKIQEAELDYLTHSRAASMALAENYVGLPF----

>C6AVC3|C6AVC3_RHILS

MRTQVAIIGSGPSGLLLGQLLTEAGVDNVILDRVNKDYILSRVRAGVLEEGTVGLLDQAKSGARLHSEGL

PHDGFSLTFDGRDHRIDLHELTGGRRVTVYGQTEVTRDLMERREESGSPSIYDAVDVAPHDFDGHSPFVT

YVKDGVAKRIDCDFIAGCDGFHGASRKTVPERAIRSFEKVYPFGWLGVLADVAPVSHELIYANHPRGFAL

CSMRSATRSRYYIQCALDEKIGDWSDDRFWDELRRRLPTHHAEALATAPSFEKSIAPLRSFVAEPMRFGR

LFLVGDAAHIVPPTGAKGLNLAASDVHYLFSGLIEHYREGSNSGIDAYSQKALARVWKAVRFSWWMTTMM

HRFPDTGDFDQKIQEAELDYLTHSRAASTALAENYVGLPF----

>D0SH67|D0SH67_ACIJO

LKTKVAIIGSGPAGLLLGQLLYKAGIDHIIVEQRSADYVASRIRAGILEQVSVDLLEKADVDQNLKAHGL

PHSGIEILAQGRKHRVDLASLTGGKQVTVYGQTEVTKDLMQAREQADLLSFYEAQSVRVDNLYG-LPTVG

FEYQNQAYQIECDFIAGCDGYHGVCRASVPEDKIKTFEKVYPFGWLGVLADVPPVADELIYVQSERGFAL

CSMRSETRSRYYLQVPLTDKVENWSDEQFWNELKNRLDPESQEKLVTGPSIEKSIAPLRSFVTEPMRFGK

LFLAGDAAHIVPPTGAKGLNLAASDIAYLSSALIQYYQEGSEQGINDYSEKCLQRVWKAERFSWWMTHLL

HRFETESEFDHKIKQAELNYILGSEAGQTTLAENYVGLPYEFQN

>Q03298|PHHY_ACIAD

MKTKVAIIGSGPAGLLLGQLLYKAGIEHVIVEQRSADYVASRIRAGILEQVSVDLLEQAGVDQNLKEKGL

PHSGIEILTNGQKFRVDLSALTQGKQVTVYGQTEVTKDLMQAREQAGLCSFYESNDVQIHDFYN-APKVT

FESNGTHYQIECDFIAGCDGYHGVCRASVPQDKIKTFEKVYPFGWLGVLADVPPVADELIYVQSERGFAL

CSMRSETRSRYYIQVPLTDHVENWSDDQFWEELKNRLDPESCEKLVTGPSIEKSIAPLRSFVTEPMRFGK

LFLAGDAAHIVPPTGAKGLNLAASDIAYLSSALIEFYTQGSEQGIDQYSEKCLQRVWKAERFSWWMTHLL

HRFETESEFDHKIKQAELSYILGSTAGQTTLAENYVGLPYEIKS

>C0VJC8|C0VJC8_9GAMM

VKTKVAIIGSGPAGLLLGQLLYKAGIDHVIIEQRSAEYVASRIRAGILEQVSVDLLEQAGVDQNLKTKGL

PHSGIEILTNGKKHRVDLSSLTEGKQVTVYGQTEVTKDLMQAREHAELTSFYEAEQVQVDDFYS-EPKVT

FHYHGKDYQIECDFIAGCDGYHGVCRASVPVDKIKTFEKVYPFGWLGVLADVPPVADELIYVQSERGFAL

CSMRSETRSRYYLQVPLTDHVEDWSDERFWEELKRRLDPESREKLVTGISIEKSIAPLRSFVTEPMRFGQ

LFLAGDAAHIVPPTGAKGLNLAASDIAYLSSALVEYYHEGSEQGINEYSEKCLQRVWKAERFSWWMTHLL

HRFETESEFDHKIKQAELSYILTSQAGQTTLAENYVGLPYEIKP

>D0S2U9|D0S2U9_ACICA

LKTQVAIIGSGPAGLLLGQLLYKAGIDHIIVEQRSAEYVASRIRAGILEQVSVDLLKQAGVDQNLKDKGL

PHSGIEILTNGVKHRVDLAALTGGKQVTVYGQTEVTKDLMTAREAEKLTSFYEAQNVQVKDFYT-APKVE

FEYQGKAFQIECDFIAGCDGYHGVCRASVPEDKIKTFEKVYPFGWLGVLADVPPVADELIYVQSERGFAL

CSMRSETRSRYYLQVPLTDHVEDWSDEKFWDELKSRLDPESREKLVTGASIEKSIAPLRSFVTEPMRFGK

LFLAGDAAHIVPPTGAKGLNLAASDIAYLSSALVEYYVEGSEQGIDEYSEKCLQRVWKAERFSWWMTHLL

HRFETESEFDHKIKQAELSYVLGSIAGRTTLAENYVGLPYEIKQ

>B0VBA3|B0VBA3_ACIBY

LKTQVAIIGSGPAGLLLGQLLYKAGIDHIIVEQRSAEYVASRIRAGILEQVSVDLLKQAGVDQNLKEKGL

PHSGIVILTNGEKHRVDLAALTGGKQVTVYGQTEVTKDLMTAREAEQLTSFYEAQNVQVKDFYT-APKVE

FEHQGKTFQIQCDFIAGCDGYHGVCRASVPEDKIKTFEKVYPFGWLGVLADVPPVADELIYVQSERGFAL

CSMRSETRSRYYLQVPLTDHVEDWSDEKFWDELKNRLDPESREKLVTGPSIEKSIAPLRSFVTEPMRFGK

LFLAGDAAHIVPPTGAKGLNLAASDIAYLSSALVEYYAEGSEQGINEYSEKCLQRVWKAERFSWWMTHLL

HRFETESEFDHKIKQAELSYVLGSIAGKTTLAENYVGLPYEIKQ

>A3M4E0|A3M4E0_ACIBT

LKTQVAIIGSGPAGLLLGQLLYKAGIDHIIVEQRSAEYVASRIRAGILEQVSVDLLKQAGVDQNLKEKGL

PHSGIEILTNGELHRVDLAALTGGKQVTVYGQTEVTKDLMAAREAAQLTSFYEAHHVQVKDFYT-APKVE

FEYQGKTFQIQCDFIAGCDGYHGVCRASVPEDKIKTFEKVYPFGWLGVLADVPPVADELIYVQSERGFAL

CSMRSETRSRYYLQVPLTDHVEDWSDEKFWDELKNRLDPESREKLVTGPSIEKSIAPLRSFVTEPMRFGK

LFLAGDAAHIVPPTGAKGLNLAASDIAYLSSALIEYYAEGSEHGINEYSEKCLQRVWKAERFSWWMTHLL

HRFETESEFDHKIKQAELSYVLGSIAGKTTLAENYVGLPYEIKQ

>B1XXU7|B1XXU7_LEPCP

QRTQVAIIGAGPSGLLLGQLLHKAGIANIVIEQRSGDYVLGRIRAGVLEQVCVDLLDEAGVGARMHREGL

VHHGIELAFAGQRHRIDLSGPTGGKAVMVYGQTEVTRDLMDERTANGAPIVYEAEHVEVHDFDGSNPRVT

YLKDGVRHEVLCDFIAGCDGFHGVCRASVPAGAIRNFERVYPFGWLGILADVAPAADELIYSNHERGFAL

CSMRSKTRVRHYVQCSLQDKIEDWSDARFWDELKSRLDPEAAATIPTGPSIEKSIAPLRSFVAEPMRFGR

MFLAGDAAHIVPPTGAKGLNLAAADVRYLSRAFIEFYADKTAAGIDDYSRKALRRIWKAERFSWTMTSLL

HKFPDTGDVGFKLQMAELDYLVNSPAAMASLAENYVGLPFEE--

>Q89CF9|Q89CF9_BRAJA

MRTKVAIIGAGPAGLLLGQLLHNYGIDNFILERQSPDYVLGRIRAGLLEEGTVALLDQVGAGVRAHAEGL

VHEGIELAFSGRRHRIDMKAAT-GKTVMIYGQTEVTLDLMNARKAAGLDTVYEAKDVRPHDFDGSHPRVT

WVRDGVTHTLDCDFIAGCDGFHGVSRASVPASAIEEFERVYPFGWLGILSETPPVSHELIYSNHALGFAL

CTMRSMKRSRYYVQCSLDDHVDQWPDDRFWDELKRRLDQEAADSLVTGPSIEKSIAPLRSFVAEPMRFGK

MFLCGDAAHIVPPTGAKGLNLAASDAHYLSSALREFYDEKSSAGIDAYSAKALARVWKAVRFSWWMTSML

HKFPDTGAIGARIQLAELDYVTQSQAAMSSLSENYVGLPF----

>Q07JZ7|Q07JZ7_RHOP5

MRTQVAIIGAGPAGLLLGQLLHRYGIDNVILERKDPDYVLSRIRAGVLEQGAVDLIDEAGVGARLHREGL

VHDGIELAFAGQRHRIDFKATT-GVSVTVYGQTEITRDMMDARTAEGLVSVYEAEDVSLHDFDGEHPKVR

YVKDGVAHELACDFIAGCDGFHGVARQSPPVGAVTHFERVYPFGWMGLLSDTPPVSPELIYSNHERGFAL

CSMRSPHRSRYYVQCSLAEDVEQWPDERFWDELRRRLDPDTAAKVVTGPSIEKSIAPLRSFVAEPMRFGR

LFLAGDAAHIVPPTGAKGLNLAASDVCYLSRALREFYAEKSSAGLDDYSRKALARIWKAERFSWWMTTML

HRFPDSTGFDGKIQAAELDYLVGSQAATTSLAENYVGLPY----

>Q139X0|Q139X0_RHOPS

MRTQVGIIGAGPSGLLLGQLLHTYGIEAVILERKNPDYVLSRIRAGVLEQGMVDLLDEAGVGRRLHQEAL

VHDGFEIAFSGRRHRIDLKHSTGGRTVTVYGQTEVTRDLMEARKAAGLTTIYEAADITLHDFDGERPRVR

YFKDGVSQELACDFIAGCDGFHGVARQSAPANALQTYERVYPFGWLGVLSDTPPVSSELIYVNHDRGFAL

CSMRSAHRSRYYVQCPLSDDVGEWSDDRFWDELKQRLGPETAGHLVTGASIEKSIAPLRSFVAEPMRFGR

LFLAGDAAHIVPPTGAKGLNLAASDVYYLSRALREFYDEGSKGGIDAYSANALRRVWKAERFSWWMTSIL

HRFPDSDAFTQRIQTAELDYLVSSQAATTSLAENYVGLPY----

>Q2IU32|Q2IU32_RHOP2

MRTQVAIIGAGPSGLLLGQLLHRYGIDAVILERKDPDYVLSRIRAGVLEQGLVGLLDEAGVGARLHQEGL

VHDGFEIAFSGKRHRIDLAGTTGGKHVTVYGQTEVTRDLMEARKAAGLTTVYDAADVSLHDFDGDTPKVR

WVKDGVTHELACDFIAGCDGFHGVSRQSV-AGAVQSFERVYPFGWLGVLSDTPPVSHELIYVNHERGFAL

CSMRSTQRSRYYVQCPLSDDVAQWSDDRFWDELKHRLDPEAADKLVTGASIEKSIAPLRSFVAEPMRFGR

LFLAGDAAHIVPPTGAKGLNLAASDVYYLSRALREFYGEHSKAGIDAYSADALRRVWKAERFSWWMTSML

HRFPDSDAFSQRIQTAELDYLISSQAAITSLAENYVGLPY----

>E6VGR9|E6VGR9_RHOPX

MRTQVAIIGAGPSGLLLGQLLHKYGIDAVILERKDPDYVLSRIRAGVLEQGMVDLLDEAGVSARLHREAL

VHDGFEIAFAGKRHHIDLRGATGGKNVTVYGQTEVTRDLMEARNAAGLTTIYDAADVSLHDFEGAHPKVR

FVKDGTTREISCDFIAGCDGFHGISRQSVPASAVQSFERVYPFGWLGLLSDTPPVSPELIYVNHDRGFAL

CSMRSAHRSRYYVQCPLTDDIADWSDDRFWDELRSRLDPATAGKLVTGPSIEKSIAPLRSFVAEPMRFGR

LFLAGDAAHIVPPTGAKGLNLAASDVYYLSRAMREYYAEKSEAGIDAYSANALRRVWKAERFSWWMTSQL

HRFPDHDAFSQRIQTAELDYLISSQAALTSLAENYVGLPY----

>B3Q9N4|B3Q9N4_RHOPT

MRTQVAIIGAGPSGLLLGQLLHKYGIDAVIVERKDPDYVLSRIRAGVLEQGMVDLLDEAGVSTRLHQEAL

VHGGFEIAFAGQRHHIDLRGATGGKSVTVYGQTEVTRDLMEARSAAGLTTIYDAADVSLHDFEGAHPKVR

YVKDGTTREIVCDFIAGCDGFHGISRQSVPASAVQSFERVYPFGWLGLLSDTPPVSSELIYVNHDRGFAL

CSMRSMHRSRYYVQCPLTDDVADWSDDRFWDELKSRLDPETAGKLVTGPSIEKSIAPLRSFVAEPMRFGR

LFLAGDAAHIVPPTGAKGLNLAASDVYYLSRVMREYYAEKSEAGIDAYSASALRRVWKAERFSWWMTSQL

HRFPDSDAFSQRIQTAELDYLVNSKAALTSLAENYVGLPY----

>F8GTF7|F8GTF7_CUPNE

MRTQVAIIGAGPAGLLLGQLLTRAGIDNIIIEQRSPEYVLGRIRAGILESVTVDALERAGVARRLHAEGL

VHHGIALSYGGQRHHIDLHALI-GRSVTVYGQTEVTRDLMEARRADGTPTIYEAQEVSVQGFDGSTPSVR

YRKDGAMHEVACDYIAGCDGFHGICRESVPVAARQVFERVYPFGWLGLLSETPPVADELVYASHERGFAL

CSMRSRHRSRYYVQVASSERVEDWSDERFWQELRSRLDPQTASALVTGPSIEKSIAPLRSFVCEPMRFGR

LFLAGDASHIVPPTGAKGLNLAASDVLALSEGFEARYRHADASGLDGYSARCLQRVWKAERFSWWMTSLL

HRFPDADAFSLRMQRAELDYLVGSEAASRALAENYVGLPY----

>Q0JYV6|Q0JYV6_CUPNH

MRTQVAIIGAGPAGLLLGQLLTRAGIDNIIIEQRSPEYVLGRIRAGILESVTVDALERAGVAGRLHAEGL

VHHGIALSYGGQRHHIDLHALI-GRSVTVYGQTEVTRDLMDARRADDTPTVYEAQEVSVHGFDGSTPSVR

YRKDGAMHEVACDYIAGCDGFHGICRESVPAAARQVFERVYPFGWLGLLSETPPVADELVYASHERGFAL

CSMRSRHRSRYYVQVAASERVEDWSDERFWQELRSRLDPQTASALVTGPSIEKSIAPLRSFVSEPMRFGR

LFLAGDAAHIVPPTGAKGLNLAASDVLALSEGFEARYRRADASGLDGYSARCLQRVWKAERFSWWMTSLL

HRFPDADAFSLRMQRAELDYVVGSEAASRVLAENYVGLPY----

>Q1LG40|Q1LG40_RALME

RSTRVAIIGAGPAGLLLGQLLTVAGIDNIVIEQRSPEYVLGRIRAGILECVTVEALKRAGVDGRLREEGL

VHHGIELSFGGERHRIDFHALI-GSDVTVYGQTEVTRDLMAARQASGVPTVYEAEDVSLHDFDSNRPRVC

YRKDGVEHEVVCDYIAGCDGFHGVSRASVPEPSKRIFERVYPFGWLGILSETPPVADELIYASHERGFAL

CSMRSKTRSRYYVQVPSTEKPEDWSDARFWDELRHRLDPAAAEALVTGPSIEKSIAPLRSFVCEPMRYGR

LFLAGDAAHIVPPTGAKGLNLAASDVLYLADGLIAHYQHDDSHELDMYSERCLRRVWKAERFSWWMTSLL

HRFPDADAFAHRIQQAELDYLVGSEAACRSLAENYVGLPR----

>Q46R66|Q46R66_CUPPJ

MRTQVAIIGAGPAGLLLGQLLSRAGIDNIIVEQRSPEYVLGRIRAGILESVTVDALERAGVAARLHDEGL

PHAGIELSFNGQRHRIDLQALT-GRTVTVYGQTEVTRDLMAARQAEGTTTIYEAADVSLHGFDGTRPLVR

YRKDGATHEIECDYIAGCDGFHGVSRQSVPATSSQLFERVYPFGWLGILSDTPPVADELIYASHERGFAL

CSMRSKARSRYYVQVPSTERVEDWSDERFWDELRSRLDPQAAESLVTGPSIEKSIAPLRSFVCEPMRFGR

LFLAGDAAHIVPPTGAKGLNLAASDVLYLADALTARYQQDDLRELDSYSEKCLRRIWKAERFSWWMTSLL

HRFPDADAFSARIQQAELDFLVSSEAACKALAENYVGLPY----

>Q08UB0|Q08UB0_STIAD

MRVQVAIIGGGPAGLLLGHLLSQASVDNIVLEQRSREYVLGRIRAGVLEQGTVDLLTSAGLARRLHKQGL

VHGGVELCFDGSRHRIDLHGLT-GKNVTVYGQTEVTHDLMDARDALGASIVYEAANVSVHGFDGASPRVR

YEKNGQTHEVECDFIAGCDGYHGVSRASVPEASLRTYERVYPFGWLGLMADVPPVSHELIYSNHERGFAL

CSMRSPTRSRYYVQCSLSDKVENWSDERFWDELRRRLDTRAAETMITGPSIEKSIAPLRSFVAEPMRFGR

LFLAGDAAHIVPPTGAKGLNLAVSDVRLLSRALIEYFKEGSAVGIDTYSERCLRHVWKAVRFSWWMTSLL

HKFPDTGEFGQKVQHAELEYLVGSVAASTSLAENYVGLPS----

>F5XYH2|F5XYH2_RAMTT

MRTQVAIIGAGPSGLLLGQLLHQAGIANVIVERQSGAYVLGRIRAGILEQVTVDLMAEAGVGARVQREGI

AHHSIELVFQGTRHSIDVHGLTGGKQVTAYGQTELTHDLMEARAAAGLETVYEAADVAVQGFDGERPQVR

FKKDGREQVLQCDFIAGCDGFHGVCRASVPRRALREYEKVYPFGWLGVLADVPPVSHAIVYGNSERGFSL

CSMRSMTRSRYYVQTAMDDKVEAWSDQRFWDELRRRLDPELADRVVTGPSIEKSIAPLRSFVAEPMRFGR

LFLAGDAAHIVPPTGAKGLNLAASDVKYLSSALIEHYREHSDAGLDQYSARALARVWKAERFSWWLTSLM

HRFPDTGDFGQRLQDAELGYLVGSRAAATALAENYVGLPL----

>Q2WAR2|Q2WAR2_MAGSA

MRVQVAIIGAGPSGLILGQLLAKAGIDALILEAQTPDYVLGRIRAGVLEQVCVDLLDEAGVGERLHAEGL

PHQGIELLVDGTRHRIDLAGLTGGKQVTVYGQTELTRDLMEARTRAALKTVYEAKEVMVSDFDTDHPRVS

YLKDGVRHEVECDFIAGCDGYHGVCRASVPKDALRTFERVYPFGWLGLLSDTPPVNHELIYAKSERGFAL

CSQRSLTRSRYYLQVPLSEKVEDWSDERFWGELRQRLDGRANDALVTGPSLEKSIAPLRSFVAEPMRFGR

LFLAGDAAHIVPPTGAKGLNLAASDVRTLGHALIEFY-GGSLEGIDRYSERCLRRIWKAERFSWWMTNLL

HRFPDTPPFEQRALEAELDYVVHSLAGRTTIAENYVGLPFED--

>Q128Y6|Q128Y6_POLSJ

MRVQVAIVGGGPAGHLLGQLLHKAGIEAIVIERHSREYVLGRIRAGVLEQGTTDLLDEAGVGARMHHEGL

LHGGISLDFGGTHHRIDLHHLTGGKQVMVYGQTEVTRDLMDARAAAGLATAYEAEQVSLHDFDGERPRVR

YVQHGVAHEVECDFIAGCDGYHGVSRASVPASALQTFERIYPFGWLGMLTDTPPVSHELVYTNHARGFAL

CSMRSHTRSRYYLQCSLDDKVENWSDQAFWDELRRRLAPDLAEQLQTGPSIEKSIAPLRSFVAEPMRFGR

LFLAGDAAHIVPPTGAKGLNLAASDVGYLGRALTEFYA-GNEGGINAYSDTCLRRVWKAVRFSWWFTSLM

HKFPDSGGFGEKIQAAELDYLIHSQAASTALAENYVGLPL----

>F3KT36|F3KT36_9BURK

TRTQVAIIGAGPSGLLLGALLHKAGIDAVIVERQSGDYVLGRIRAGVLEQVTMDLMDEIGVGARMHKEGL

VHGGFDMLFQGQRHRIDMNRLTGGKNVMVYGQTEVTRDLMDARKQAGLTTIYEAANVAIHDFGAGKPRVT

FEKDGKSHELQCDFIAGCDGFHGVCRDTVKKSAIREYEKVYNFGWLGVLSDTPPVHHELIYVNSERGFAL

CSQRSNTRSRYYLQVPLTDKVEQWSDQAFWDELKLRLDPEAREHLVTGPSIEKSIAPLRSFVTEPLRFGR

LFLSGDAGHIVPPTGAKGLNLAATDVKYLSAALIEFYQDKSEAGIDHYSERCLKRIWRAERFSWWFTTLM

HRFPENGEIGQKLQEAELDYIVHSETGARSVAENYVGLPLDFGT

>Q222G0|Q222G0_RHOFD

IRTQVAIIGAGPSGLLLGQLLHKAGIDAVIVERVSGDYVLGRIRAGVLEQVTIDLLDEAGVGTRMHREGL

VHGGFDMLYGGKRHRIDMNGLTGGKNVMVYGQTEVTHDLMDARAFAGLPTYYEASDVQVADFDTTHPRVT

FTHQGQAVTLSCDFIAGCDGFHGVCRASVPRKSIQEFEKVYPFGWLGLLSDTPPVHDELIYVNSPRGFAL

CSQRSKTRSRYYLQVPLTDRLQDWTDDAFWQELRLRLDDDARAQLVTGPSLEKSIAPLRSFVTEPMRFGR

LFLAGDAAHIVPPTGAKGLNLAATDVKYLCNALVDFYQNRSEEGIDSYSARCLRRIWKAERFSWWFTSLM

HRFPDDGPITARFQEAELDYLIHSHAGSLSIAENYVGLPLDFAE

>A1VNI3|A1VNI3_POLNA

MRTQVAIIGAGPSGLLLGQLLHKAGIDAIILERQTGDYVLGRIRAGILEQVCIDLMDEAGVGERMHKEGL

VHGGFEMLYNGKRHRIDMNKLTGGKNVMVYGQTELTRDLMDARAAAGLPTVYEATHVAVHDFDTAKPRVT

YEKDGQKFEIECDFIAGCDGFHGVCRASAPRSAITEFEKVYPFGWLGLLSDTPPVHDELIYVNSPRGFAL

CSQRSKTRSRYYLQVPLTDRIEEWTDEAFWQELRLRLDDEGREKLITGPSIEKSIAPLRSFITEPLRFGR

MFLAGDAGHIVPPTGAKGLNLAATDVKYLSSAIIEFYQDKTEAGIDNYSERCLKRIWKGERFSWWFTQLM

HRFPDDGAIVAKFQQAELDYLLNSEAGSRSIAENYVGLPLNFGE

>C9YF73|C9YF73_9BURK

MRTQVAIIGAGPSGLLLGQLLHKAGIANVILERQSADYVLSRIRAGVLEQVTTDLLDEAGVGTRMHQEGL

PHTGFDLLFGGERHRIDLHSLTGGQQVMVYGQTEVTRDLMAARQAAGLPTVYQAGDVSVHDFDTQHPQVR

YQQDGAWHTLDCDFIAGCDGFHGVCRASVPEGAVTEYEKVYPFGWLGVLSDTPPVHHELIYANSTRGFAL

CSQRSATRSRYYVQVPLTEKAENWSDEAFWHELRQRLDPEARETLVTGPSLEKSIAPLRSFVAEPMRFGR

MFLAGDAAHIVPPTGAKGLNLAATDVKYLSSAFIEYYAERSNAGIDHYSQRCLQRIWKGERFSWWFTSLM

HRFPDGGAIGQKLQDAELAYLVQSKAASTALAENYVGLPL----

>C5CYQ0|C5CYQ0_VARPS

MRTQVAIIGAGPAGLLLGQLLFKAGIDNVIVERQSGDYVLGRIRAGVLEQVTMDLLARAGVDARAKAEGL

PHEGIELLFKGARHRIDMHGLTNGKQVTVYGQTEVTRDLMEARSAEGLATIYSAANVSLHDFDSQRPRVR

YEKDGQTHEIECDFIAGCDGYHGVSRASVPADAIQTYEKIYPFGWLGVLADVPPVSHELIYANTERGFAL

CSMRSAHRSRYYVQVPADEKVQNWSDEAFWNELRARLDPEARERLVTGPSLEKSIAPLRSFVAEPMRFGA

LFLAGDAAHIVPPTGAKGLNLATADVGYLSRALEIFYGERSASALDRYSDLCLRRVWKAERFSWWFTSLM

HRFPETGAFGQKIQEAELDYLVHSHAASTALAENYVGLPLEDF-

>E6V0R5|E6V0R5_VARPE

MRTQVAIIGAGPAGLLLGQLLFKAGIDNVIVERQSGDYVLGRIRAGVLEQVTMDLLARAGVDARARAEGL

PHEGIELLFKGARHRIDMHGLTGGKQVTVYGQTEVTRDLMEARTAEGLTTIYSAANVSLHDFDSAKPRVR

YEKDGQTHEIECDFIAGCDGYHGVSRASVPADAIQTYEKVYPFGWLGVLADVPPVSHELIYANTERGFAL

CSMRSATRSRYYVQVPTEERVENWSDEAFWNELRARLDPEARERLVTGPSLEKSIAPLRSFVAEPMRFGS

LFLAGDAAHIVPPTGAKGLNLATADVGYLSRAFEIFYGEKSASALDRYSDLCLRRVWKAERFSWWFTSLM

HRFPETGTFGQKIQEAELDYLVHSHAASTALAENYVGLPLEDF-

>A9BX04|A9BX04_DELAS

MRTQVAIIGAGPAGLLLGQLLYRAGIDNVIIEQRSADYVLGRIRAGVLEQVTVDLLEQAGADARMRAEGL

PHDGIELLFGGQRHRIDLHGLTGGKRVMVYGQTEVTRDLMEVRAAEGLTTVYEAGNVQPVGFDGDSPVVR

YEKDGQVHELQCDFIAGCDGFHGICRASVPQDKVRTFEKVYPFGWLGLLSDTPPVSHELIYAQTERGFAL

CSQRSATRSRYYLQVPLTEKVEDWSDEAFWSELRLRLDPEARERLVTGPSLEKSIAPLRSFVTEPMRFGR

LFLAGDAAHIVPPTGAKGLNLAASDVGYLSSAFAQYYRDKAPEGIDGYSEQCLRRVWKAERFSWWMTSLM

HRFPDAGGFDTKVQEAELDYIVNSRAGSTSLAENYVGLPLV---

>D0J822|D0J822_COMT2

MRTQVAIIGAGPSGLLLGQLLYKAGIDNIIIEQRSADYVLGRIRAGVLEQVTVDLLKQAGADKRMNEEGL

PHDGIELLFKGKRHRINLHDLTGGKRVMVYGQTEVTRDLMEVRAQEGLTTVYEASHVQPVDFESDKPKVR

YEKDGQVHEIECDFIAGCDGFHGICRASAPQDKIKTFEKVYPFGWLGLLSDTPPVSHELIYANTERGFAL

CSQRSATRSRYYLQVPLTDKVEDWSDEAFWEELKKRLDPEARANLVTGPSLEKSIAPLRSFVTEPMRFGR

MFLAGDAAHIVPPTGAKGLNLAASDVGYLSQAFVEYYKEQSEAGIDRYSEQCLRRVWKAERFSWWMTSML

HNFPGEGEFNTKVQEAELDYIVHSEAGSTSLAENYVGLPLV---
