# Supplementary material for: Accurate Simulation and Detection of Coevolution Signals in Multiple Sequence Alignments
Source: PLoS One. 2012 Oct 16;7(10):e47108. doi: 10.1371/journal.pone.0047108 (PMC3473043; doi:10.1371/journal.pone.0047108)
Supplement: MSA S5 — MSA of PDR. (DOCX) [file pone.0047108.s016.docx]

>B2TGZ0|B2TGZ0_BURPP

ESKVDADTMHLTVSEVVSLTCNTKLLRLRSTDGRILPRHEAGAHIEVTLPDGAWNAYSLIGNPADRSVFE

IAIKREETGRGGSRYLHDAVSEGGILEARRPVNGFSLDASATSHLLIAGGIGITPIYSLSSQLSRLK-IR

HRVIYCVRDMEDAPLLERLKQSAFA-EVECHADNGQMQRYYDFENLLRDPVDAQRIYVCGPEPFIRAISR

ICEQWNRGTVRSENFGGIR----GAPKLTVHFAKRNKTIVVEQPETIVDAMVRNGLDPLYGCKRGECGIC

AVSVMSGAPLHRDMFLSEEEKQKYMCTCVSWSASEEITLDM

>F0E1K7|F0E1K7_9PSED

KATANHQFW-CTVTSRRSETPDIDSYWLT-PD-EPF-DLEPGMHVSVLTPSRHLRQYSLVNTPEERDVLV

IGVKHEVNSRGGSRSMHADVHVGTKILVSLPRNQFILQPPGRTPLLIAGGIGVTPILAMALHLQYVG-RP

YGFHYLARGEQHIAFPDRIATLASDHPPYLGLD----GTQRTITELL-QDSDKHDVYVCGPQQMIELVAT

VAQGFTPEQIHFEYFGLTPSTG-SGPGYQVAINSTGQEFTVEPGQTLLKACLDHGITIDYSCEQGVCGAC

VTPVISGDINHEDVYLSAKEKGKLIMPCVSGCNSKKLVLNI

>G0CK41|G0CK41_XANCA

SMRKDTHWHHARVVSVADACQGVREIVLD-P-GANTRSFEVGSHLDFRLQHGDVRSYSLVGEPRADGYYQ

VAVRQMPESRGGSRHMWT-LAPGDVVEISPPSNNFALDEAGEEILLIAGGIGVTPIVGMAQRLARRH-AA

FRMLYVGRARDTMAYVDTLEA-LLGARLQVHCDDQA-G-PPDLAAELATLSPNAEVYVCGPLGMLEAVRQ

QWHGRPRARLHFETFGNSG----RVPAFVVKLPGLGMEVQVAENVSMLDALADAGVELIAECRRGECGLC

AVDVLDADIDHRDVFFSDAQRNRKLCACVSRAVGGSISIDT

>A9EYZ0|A9EYZ0_9RHOB

EEGQQMDTRTARLVGKTALSPDTVDFSFELIN-GRFSGLEPGAHVDVHL--GLVRQYSIWNWRQDGRQLN

IAVKLEASGRGGSHAMHA-LEPGDEILLGTPRNNFKLQPDTPYVTLIAGGIGATPLVAMARELANMR-RD

FQVYYLVRSQDLAAMDEPFRALDLGDRYHLHC-DDADG-QLDLAAVLRSMPMGSDVYTCGPEPMLNAVLE

AGSAMRGGTIHFERFAAVA----DAGSFDIEIQSTGAMLRVSAEESILDVLKASGIAVDFGCSEGLCGAC

LVDVLDGEVDHRDGILTPEEQNSYLCTCVSRAKGDKLVLNL

>B8KG84|B8KG84_9GAMM

-PEGPAPVIPVRLVSKTPVSDDIVQFQFEALDGGELPAFEAGAHIDVMADPQFIRQYSLCGDPADSKSYA

IGVLREDEGRGGSLRIHQRLQPGRTLLISRPRNHFPLERNATKSLLLAGGIGVTPMMSMAHELHSRG-ED

FVLYYKAKSRAGAAFVDEIARQPWSANAHFYPSDED---RLDIASVLKDYAPGNHLYTCGPVAFMDAVFE

RAAGWPEEALHREFFAAPEGLEYENHPFKVVLASTGKEIEVAADQKATEALAGAGVAIDTKCSDGICGVC

ATRYVSDDIEHRDFVLSKEQREERVILCSSRAAAPNVIIDL

>A3SEM6|A3SEM6_9RHOB

KPLADSPVLPLTVTRADVLSDGITRYELRDPDGADLPLWQAGAHLDVVIPE-YLRQYSMCGDPSDRSRYV

IAVQRE-QGRGGSALMHRIFTQGRKIFAARPINHFPLTEDAPFSVLMGGGIGITPMIAMAHRLHQLG-RD

FALHYSARTAADLAFAAEIAAAPWAAHAVLHVSDQG-S-RAEFDTILSGHPVGSHAYACGTPAYMEAAMT

AAAGFAEDQCHIEYFAVPEAPPRENHSFTVHLAKTGKDIHVPADRSLSDMLTEAGVPVDVKCADGICGVC

ACGLVEGDADHRDYVLSQAQRETTLITCQSRAAGGHLVLDL

>A9DRJ0|A9DRJ0_9RHOB

KVGSDTPVLDLRITEVTRLNATTALYDIAHPEGHDLPEWTAGAHLDLVVPE-FLRPYSLLGDPSDRKRYR

IAVLREDAGRGGSALLHRVFTKGRRIFVGKPVNHFELVEDAPHSLLMGGGIGITPMIAFAYRLHALG-RP

FDLHYSASTREAAAFAEELAQAPWADRVHLHISSEN-T-RADLSAIMERAAPGTHVYTCGADAYMQAVMA

AAEGIPEDARHLEYF-STP----EVPPFTLKLT-SGREIPVSKDETAADALIAAGVAVDLKCSDGICGVC

KCGLRGGEVEHRDFVLSAKQREGAIILCQSRAAKPDLEVDL

>B7RJ84|B7RJ84_9RHOB

DAGAESPVLDLRITAVQQLNDSVTLYDLAHPDGADLPLWTAGAHLDLVVPE-YLRPYSLLGDPAERSRFR

IAVLREDAGRGGSALLHRIFSKGRRVFVSAPVNHFELVQDALHSLLFGGGIGVTPMIAFAHRLHALG-RP

FELHYSAPTQTAAAFAPLLADVAWADRVHLH--ISADGTRADMDTLMASAPNGTHAYTCGPDAYMQAVMS

AGKGLPDDALHLEYF-APP----AQPPFTLRLP-DGRDIPVSADQSASDALIAADIKLDIKCADGICGVC

KCTLLSGQAEHRDFVLSAKQRESTLILCQSRAASPGLEIEL

>A3JP30|A3JP30_9RHOB

-DQGESPVLQVVLSKVETMTDLVTKYEFSMPDGSPMPVVTAGAHVDVVVPE-FFRQYSLSGDPSNRNNYQ

IAVLREDEGRGGSKLMHRIFQEGRKLFISKPINHFPLVESASFTYLMGGGIGITPMISMAHRLHSIG-AD

FAVHYSCSKRANAGFLNDLAKVPWADKVHYHFSDED-S-RANLHHVL-KPHKDAHVYTCGPDTYMESVMS

AAKGFPEDCRHLEYFSVPEQPEYENFDFTFKLAKSGKVVHVPADTIPSDALLAAGVSIDVKCSDGLCGVC

KCNVVSGDVQHRDFVLSNKERETQMIVCQSRA-AQKVVLDI

>B6B5F6|B6B5F6_9RHOB

-AQGDAPVMRVRLSKAEHMTDAVTKYEFSLPDGSPLPQAAAGAHIDVVVPE-FFRQYSLSGDPADRSKYQ

IAVLREDAGRGGSKLMHRIFEEGRMVFISKPINHFPLVEDAAKSYLMGGGIGVTPMIAMAHRLHALG-RD

FEFRYSCSKMANAGFLEDLKHVPWADKVHLHFSDQG--TRADLSDVL-HNAPGSHVYTCGPAAYMQAVLD

AAAGFPEEARHMEYFSVPEQPDYENHPFTLRLAKSGREILVPAEKTATEALAESGIHVNVKCSDGICGVC

KCGLISGDAEHRDFVLSNKQREGAIILCQSRAAGGVLEIDL

>C7DBT6|C7DBT6_9RHOB

--AADAPVVQVIVDRVTQMTPDLAHYRLVSATDAPLPPWTAGGHLDVVVPD-LLRQYSLCGDPADRSCYE

IAVLREPEGRGGSQMLHRLFTQGRRLFVSKPINHFELVQDAPHSLLMGGGIGITPMIAFAHRLHALG-RP

FDLHYSVRSRSTAGFIFALGQVPWRDNVHLYISDEG-T-RADLDAIMTNLPASAHLYTCGPEAYMAATLD

AGSGFDDDNLHAEYFSAPETPDHVNTPFTLRL-KDGREFEVAPDATAADTLNDNGISVDVKCSDGICGVC

KCALVSGDVEHRDFVLSKAQRETNIILCQSRASSGIIEIDL

>F7ZHJ3|F7ZHJ3_ROSLO

-DTNDSPVVQVQVARVTHLTDKISHYLLKATDGAVLPRWDAGAHLDIVIPD-ALRQYSLMGDPADRTCYE

IAVLRE-TGKGGSRLLHRIFAEGRKVFVSRPINHFPLVRDADHSFLMGGGIGLTPMIAMAHALHAEG-RS

FEVHYSVSHQKTAAFVPLLHSVPWADRVHVHNSQNGT--RAPLDAIFLAPRTGAHVYACGPDRYMAAVME

AAEGVFEEARHQEYFTVPDLPEYQNHDFTLAL-KDGRRIPVPADQSAAQALIAAGVAVDLKCSDGLCGVC

KCGVISGEVEHRDFVLSAKQRSDQMILCQSRADGGELVLDL

>Q160Q3|Q160Q3_ROSDO

-DANDSPVVQVQVARVTHLTDKISHYVLKATDGAMLPRWDAGAHLDIVIPD-TLRQYSLMGDPANRRCYE

IAVLRE-KGKGGSRLLHRIFSEGRKVFVSRPINHFPLVCDADHSFLMGGGIGLTPMIAMAHALHAAG-RS

FEVHYSVSHQKTAAFVPLLYSVPWADRVHIHNSQNGR--RAPLDAIFRAPRTGAHVYACGPDRYMSAVMA

AAEGVSEEARHLEYFTVPDLPEYQNHDFTLVL-KDGRRIPVPADQSAAQALIAAGVAVDLKCSDGLCGVC

KCGVISGEVEHRDFVLSAKQRSDQMILCQSR--GGELVLDL

>E2CBW0|E2CBW0_9RHOB

--DGDAPVIAVEISKAEPMVDGVTKYEFSAIDGASLPEWTAGAHIDIVVPE-FLRQYSLSGNPAERGSYQ

IGVLREDKGRGGSQLLHRIFSKGRRIFISKPINHFPLVENARRSFLMGGGIGITPMIAMAHRLHAIG-AE

FEMHYSCSMRARAGFLEDLETMPWAERVHLHFSDEG-S-RADLDHILGGYHPGDHVYCCGPVPYMDTVMA

AAEGFSEDERHVEYFNVPDMPDYENHPFILKLAKSGLELEVPTSETATDVLAANGIQINVKCADGICGVC

KCGLVSGDVEHRDFVLSKKQRETAVILCQSRAKNGVIEVDL

>B6AU46|B6AU46_9RHOB

--FTDAPVIMARISKAEAMTPDVTKYEFTAWDGSALPDWTAGGHLDVVVPD-YLRQYSMSGDPADTSKYQ

IGVLREDEGRGGSLMMHRIFNEGRRVFISKPINHFDLVEDATKTYLMGGGIGITPMIAFAHRLHALG-KP

FELHYSASKMESAGYVEDLKAMPWAQHVTFHFSDQG-T-RADLDKLLNGYKDGWHVYTCGPDRYMEGVVQ

AAAGFPEEARHLEYFSVPEQPDYVNYPFTVTLAKSGKELSVRADEDLSDVLIANGIPVDVKCSDGICGVC

KCGLQSGDVEHRDFVLSNKQRENEIITCQSRAATPSIVIDL

>Q28K71|Q28K71_JANSC

--EGESPVIQVEITRADVSAEGVSIYEFTPVDGGDLPEWSAGAHLDIVVPE-FLRQYSMSGDPADRSKYQ

IAVLKEDEGRGGSKLLHRIFSTGRRIFISRPINHFPLNAGASNSVLMGGGIGITPMVAMAHELHAQG-RA

FTMHYSGRSRAQMGLLEEIMGFPWADRVQLHVTDEG-S-RADLATILGGYQPGWHVYTCGADRYMSAVME

AAEGFPEDARHLEYFSVPEQPDYVNHDFTLKLVRSGQTLSIPADKTATDVLTENGIHVDVKCSDGICGVC

KCGLISGDVEHRDFVLSKAQREDGIILCQSRAAGGVIEVDL

>E0MQD5|E0MQD5_9RHOB

--DGDAPVIRVEVVKAEPMADGVTKYEFASLDGSPLPAWQAGAHLDIVVPE-FLRQYSMSGDPADRSRYQ

IGVLREDEGRGGSALLHRIFTKGRKVFISRPINHFPLHGEATKTFLMGGGIGITPMIAMAHEAHAKG-MN

FEVHYSMRSRRSAGYLDDLASMPWADRIHLHVSDEG-S-RADFNALFTGAEEGAHVYTCGPDIYMGAVMD

AAEGVSEDNRHLEYFSVPEQPDYENNDFELKLARSGKTLTVPADRVATDVLAEAGIHLDVKCADGICGVC

RCGLVSGEVEHRDFVLSKKQRETSVILCQSRAAGGTIEVDL

>B9NM28|B9NM28_9RHOB

--AGDAPVMRVALTKVDKMTADVTKYEFSTLDGAPLPAWTAGAHLDVLVPE-FLRQYSMSGDPADTSRYQ

IGVLRE-EGRGGSALLHRIFNEGRRVFISKPINHFELDERAAKTFLMGGGIGITPMIAFAHRLHSLG-KV

FELHYSASTRAAAGYLDDLANAPWADRVHYHFSDEA-R-APILIPSCPAIRRLACVY-CGPDRYMDGVMR

AAEGFPEEARHLEYFSVPEQPEYENHPFTLKLARSGRELSVPADKDAAQVLNEAGFHVDVKCADGICGVC

KCGVISGDIEHRDFVLSNRQRRDAIILCQSRAPDGVIEIDL

>D0CYD4|D0CYD4_9RHOB

--AGDAPVMRVEVTKVEKMTPDVSKYEFSAPDGAPLPEWTAGAHLDVLVPE-FLRQYSMSGDPADRSKYQ

IGVLRE-EGRGGSALLHRIFTPGRKVFVSKPINHFELDETATRTLLMGGGIGITPMIAFAHRLHALG-RD

FDLHYSVPSREKAGYLADLAAAPWADRVHLHVSDEG-T-RADLDAFLAGYRDGWHVYTCGPDRYMEAVVQ

AAEGWPEAARHLEYFSVPEQPDYENHPFILRLARSGRDLEVPANKDAAQVLNEAGIHVDVKCADGICGVC

KCGLIAGEVEHRDFVLSRKQRETALILCQSRAAGGVVEIDL

>Q5LVV6|Q5LVV6_SILPO

PDADTAPVIRVEVTRAEKMTGEITKYEFAAPDGAPLPEWTAGAHLDVLVPE-FLRQYSMSGDPTDRTTYQ

IGVLRE-EGRGGSKLMHRIFAPGRRVFISKPINHFELDEGATRSFLMGGGIGITPMIAFAHRLHALG-AD

FELHYSVPSRTSAGYLEDLDAMPWADRVHLHVSDEG-S-RADLDAVLAGFQPGWHVYTCGPERFMDGVMQ

AAEGFPEEARHLEYFSVPEQPAYENHPFTLKLARTGREIAVPADKDAAQVLNENGIHIDVKCSDGICGVC

KCGLIAGAVEHRDFVLSGKQREGAIILCQSRAPGGVVEIDL

>C9CT12|C9CT12_9RHOB

--DDESPVLQVIVSKVEEMAAGVTKYEFRAADGSDLPEWSAGAHLDIVVPE-FLRQYSMSGNPADRSHYQ

IGVLRE-AGRGGSKLLHRIFSEGRRIFISRPINHFPLDESASKTFLMGGGIGVTPMIAMAHRLHALG-AE

FEFHYSIKSREQGGYLDDLARMPWASKVHLHISQEG-T-RAAFDQILSGYQPGWHVYTCGAAPYMEAVMT

AAEGFPEDARHLEYFSVPEQPEYENHPFVLRLARSGRDIHVSAEQTATDALAEQGIHVDVKCADGICGVC

KCGLLAGEAEHRDFVLSKAQRRDAIVLCQSRAPDGVLEIDL

>Q1GLB9|Q1GLB9_SILST

--DAESPVLQVVVSKVEEMAEGVTKYEFRAADGADLPEWTAGAHLDIVVPE-FLRQYSMSGNPADRSHYQ

IGVLRE-AGRGGSKLLHRIFSEGRRIFVSRPINHFPLDETATRTFLMGGGIGVTPMIAMAHRLHALG-AD

FEFHYSIKSRALGGYLRDLADVPWASKVNLHVSDEGS--RANFDQILGQYQSGWHLYTCGAAPYMDAVMQ

AAEGFPEEARHLEYFSVPEQPEYENHPFTLKLARSGRELQVRAEQTATDALAEHGIHVDVKCADGICGVC

KCGLISGEVEHRDFVLSKAQRRESIVLCQSRAPGGCVEIDL

>A4EPC1|A4EPC1_9RHOB

--DADSPVLQVRVSKVEAMSEGVTKYEFQSLDGAPLPEWSAGAHLDIVVPE-FLRQYSMSGDPADCSTYQ

IGVLREDSGRGGSKLLHRIFAEERKVFISRPINHFPLEEGAEKSFLMGGGIGVTPMIAMAHRLHRLG-RD

FELHYSVSRREEAGFLADLDTVPWAAKVHLHISQEG-S-RADLDQLLSGYRQGYHVYTCGPERYMEGVIQ

AAEGYPEEARHLEYFSVPDLPEYENHEFTLRLAHSGREFQVPADKSATDVLQENGISIDVKCSDGICGVC

KCGLVSGEVEHRDFVLSKAQRENTVILCQSRAKDGVIEVEL

>A3X3Z8|A3X3Z8_9RHOB

--DVDSPVLGVRVSRVEAMSPDVTKYEFESLDSSPLPEWTAGAHLDIVVPE-YLRQYSMSGNPADRSKYQ

IGVLREDAGRGGSQLLHRIFAEGRKVFISKPINHFPLEEGAAKSFLMGGGIGVTPMIAMAHRLHALG-QE

FELHYSISNREGAGYLRDLSAMPWSDRVQIHVSAEG-S-RADLDQLLSGYVPGQHVYTCGPDRYMEGVML

AAEGFPEEARHLEYFSVPELPEYENHAFTLKLARSGRELLVPADKVATDVLLENGVQVDVKCSDGICGVC

KCGLLSGEVEHRDFVLSKAQRKDSVILCQSRAKDGVIEVDL

>B7QYT8|B7QYT8_9RHOB

--DGDSPVLRVEVSHVEVMSDGVTKYEFKSLDGSPLPEWTAGAHLDIVVPE-FLRQYSMSGDPADRSKYQ

IGVLRE-LGRGGSKLLHRIFTKGRRVFISKPINHFPLEEGARRSFLMGGGIGVTPMIAMAHRLHALG-QP

FELHYSVSSRKDAGYLDDLAAMPWAEHVHVHVSDEG-S-RADLDQVLADYQDGWHVYTCGPDRYMSGVME

AAEGFPEEARHLEYFSVPEVPEYENHDFTLKLARSGRSLHVPAEKTATDVLAENGIHIDVKCSDGICGVC

KCGLVSGEVEHRDFVLSKAQRKEAVILCQSRAKDGVIEVDL

>B9BP35|B9BP35_9BURK

SSIVQTHSIDVVVTEATSLTGEIRLLRLCADNGVPLPAFEPGAHVMVEAP-GVTRAYSLCSDPADRSHYD

IAVKLEVQGRGGSRALHA-LTAGARLVIGAPRNLFALAPSATHHLLIGGGIGATPLVAMAHQLARDG-AP

FTFVQFSRSDAHLPFARLLREGPWHSQVALHFDDVQ---TIDLARLSAALPPGTHAYCCGPSAFVRWARA

QCANVGDAQWHEERFSADA----SDDAIRLILARSAKQITMQRGQTLLDALRGHGVSVDTACEQGVCGSC

VVEYSDGEPVHGDACLDATERERYVALCCGGCCSESLTLQL

>A6T4C0|A6T4C0_JANMA

VPNKQAGSASIIVLRRWKESDGVMAFELRSI-SGKLPPMQAGAHINLRTPSGLVRQYSIVNAPTERATLT

IGVKLEPDSRGGSASMHASALPGTHLEFVPTRNTFPLLDTPKYPVLIAGGIGITPLVSMAQALHAAG-KD

FRLHYFVRSDEHVSFASRMA--AFQDKLVLHAACTPQETKSILDQVLTQSSQPQIVYACGPGPMLTTIRA

CTQGMPDADVRFEIF-RNDKDFGDGKPFTVKLKRNGQTITVPRGETLLAALKRNGIPVEASCEQGICGSC

LTRVSSGVPEHRDSYLTPQEKNTCMMACVSRAISEELELDL

>D5BUL3|D5BUL3_PUNMI

--MNVNSRQQTLVKSMRQEANDVISVELVSATADSLSPFSAGGHIDVFLKNDLVRQYSLINDPADLDRYM

FAVLLENMGRGGSAYIHKKLRVGDVIEVSEPRNNFALVESAPLQVFVAGGIGITPFMSMADYCQHHN-LP

FKLYYCARHPESTAFTALLAD-RLGDDLHIHFDEGDRSKSLNLAGVF-DAHKGAQFYCCGPKGLMAAMES

MA-GDKGESLITEDFNPASVLK-EDTGFDVVISSQGLTVHVEQGQSILDVLGTVNIDVEKSCEAGVCGTC

VVGYLEGEPVHNDVVLMPDEQTSHVALCVAGCRSSKLVLDI

>C5BRW7|C5BRW7_TERTT

-MNDKPVKLPISVQQLRDLCEGVREYTLAAVDGCVLPAFEAGAHIDITAPNGQTRQYSLCNTAGDCRSYI

IAVRRESDGRGGSIAIHDQLQLNSVVTVSGLRNAFPLAVNAPRSLLFAAGIGITPLLAMARTLQSSG-QP

FHLHYSVPTATQAPYLEELRSDPFAGKLSVYFSREQ-G-RIDAPAVLANADPTAHIYVCGPQQYRDYILQ

SARGWPEAQLHYEAF-ATEINAADNKPFDVVIASTGARVAVRPTQTIVDALDENGVTVPVSCEVGTCGTC

YVGVKEGEVDHRDAFLTEDEKQEHILTCCSRALSDTLVLDL

>A3VMI6|A3VMI6_9RHOB

-----MENIKMRVEKRRDLTPSITEFTLVPVGDDVLPSFDPGAHITLETPSGAMRRYSLINDGSDPKEFV

VAIKKEPNSRGGSASMHEQATVGSELTVEFPENDFPLTD-VQKYLLIAGGIGITPILSMARYLDKKG-KM

LRIIYVSRSPEESAYLDELMR-DFDGRIIVHHDGGAPDAVYDFWDDLVTPRAT-HVFCCGPKPLMDEIKA

VSGHWPEGRIHFEDFKPVDVVRQDDVSFEVELKKSGETVTVPEDRSILEALRDAGFATSSSCESGTCGTC

KTRLLEGEADHRDMVLMEEEKSDHIMICVSRALSGRLVLDL

>D5NP99|D5NP99_9BURK

--------MLLNVTGKTALADGIYQFEFRSPNGEPLPRFTAGAHVLIETPSGIGRRYSLCNAPSELGRYV

VAVKLDPNSRGGSLSMINDVKVGMSMKVSVPENYFALVDEASDYLLIAGGIGITPMRAMMAELDAQG-ER

YRLVYCTRSPETTAFLAELSSLP-SSRVQIHHDHGNPEQSLDFAKLLATREGSTHLYCCGPRPLMQAVRD

GASGWPSSAVHFEDFGTSKSTGDASGGFTVTLRRSGLRVNVAPDQSILEALRASGVETPSSCEAGTCGAC

RTTLCNGEADHRDFVLDDDEHDSAIMICVSRAKSDELVIDL

>A9HXT6|A9HXT6_BORPD

PTSGPETLMPLRIASIADAADGIRSFELRDAGGGELPPFTPGSHVKVRVPNGEMRKYSLCNNPAERDRYV

ITVKRD-HGRGGSVSLVDQAAVGDVLPTSLPDNAFALVDNPDSLIFIAGGIGITPILSMIRSLDEPE-IG

WKLYYLSQSPATTAFLDELACPELQGRVRIHHDHGDPAQSLDLWPVLEKPNRG-HVYCCGPRGLMEAVRD

MSGHWSPGRIHFESFLEGGGKQPDDQPFTVELARSGGEFVVPVGKSILSVLLQAGVRVGHSCESGTCGSC

RTGLLAGTADHRDMVLLPDEHDSQIMVCVSRATCDKLVLDL

>A1WM04|A1WM04_VEREI

-DTRADTPMPLRIARITDVAQGIRSFDLVPADGAPLPPFTPGSHVRVQTPDGALRKYSLCNDAAERHRYV

IAVKREPGGQGGSVSMHDAAHEGRILPTWPPDNAFALMAGARAYLFIAGGIGITPILSMIRCLGAPP-AP

WKLYYLTRSPETTAFADELRSAQWQRKVHIHHDHGDPLQSVDLWPALATPNSA-HLYCCGPRALMESVRD

MTGHWSPRHVHFESFDEGGRAQPDDQPFRIRLARSQRELEVPVGQSILATLRAHGCNAPSSCESGTCGTC

RTTLLAGQAQHRDMVLLPEEMSGQIMICVSRAKSPLLVLDL

>A1VWL7|A1VWL7_POLNA

QTHADTQM-PLRVDRAYDVAQGLRSFELVLPDGVELPAFTPGSHVKVQTPSGLLRKYSLCNAPDERHRYV

ITVKRE-ASRGGSASMHDDVKVGDILPTSVPENAFPLVDNAKSYLFIAGGIGITPMLSMIRSFG-DDPAP

WKLIYLSRFAENTAFLDELGRPELKKNVKIHHSEGSPDQQFDLWPALEKPNTA-HVYCCGPRGLMEAVRD

MTGHWSPKNVHFESFDEGGKTLPDDKPFTVRMARTGQRFEVPVGVSILNAMREHGCNSASSCESGTCGTC

RTGLLEGQADHRDMVLMPEEMDTQIMICVSRAKSAELVLDL

>B7WQT4|B7WQT4_COMTE

HTHSDTQM-PLRVSRIIDLALDIRSFELVHDDGSDLPPFTPGSHVKVQTPNGMLRKYSLCNDPTERKRYV

IAVKRDPEGQGGSLSMHEQLQEGDTLPTSLPSNAFPLVDNAKRYLFIAGGIGITPILSMIRSFGELP-PP

WKLIYLTRFPENTAFREELGAPELKGRVRIHHSHGDAERVFDLWPELEKPNSA-HVYCCGPRPLMEAVRD

MTGHWSPANVHFESFNEGGGVRPDDKPFMVKLANSGAAFEVPVGKSILSVLREHGCNAAASCESGTCGTC

RTNLLSGEADHRDMVLLPEEMDKQIMICVSRAKSDELVIDL

>F6AP70|F6AP70_DELSC

QNHADTPM-PLRIDRIHDVAEGIRSFELVQPDGSALPAFTPGAHIKVQVPNGLLRKYSLCNDPEDRHRYV

ITVKREAQGQGGSLSLCDDAKVGDTLPTSVPDNAFPLVEGAKRYLFIAGGIGITPFMSMIRSGELPP-AP

WKLYYLSRAAETAAFLDELRAPEYKGKVHVHHTHGDPAKAFDLWPLFEQATDT-HIYCCGPRRLMDEVRD

MTGHWASKYVHFESFNEGGEVRPDDQPFAVSLAKTGERFDVPVGKSILAVLREHGCTVPASCESGTCGTC

RTTLVSGEADHRDMVLMPDELDHQIMICVSRAKGAELVIDL

>F8GSL7|F8GSL7_CUPNE

EPHADTPML-LRIARIQDAAQGIRSFEFVQADGTELPPFTPGSHIKVQVPNGMLRKYSLCNDPAERHRYM

ITVKRDEQGQGGSKSLCDDASEGDLIPVGLPDNAFPLLEDAKAFVFIAGGIGITPILSMIRSGELST-AK

WRLYYFTRSPESTAFLDELQAPELKGKVKIHHDFGDPAKAFDLWPVLERPSDT-HIYCCGPRGLMEAVRD

MSGHWPATRIHFESFNEGGGVRADDKPFDVVLSRSGQRYEVPVGASILNVLRQHGCNVPSSCESGTCGTC

RTGLVSGEADHRDMVLMPEEMDHQVMICVSRAKCAELVIDL

>Q12CA0|Q12CA0_POLSJ

GTPAEPDFFNLKVVRKEAVAQGIYLFELGHPEGLDLPAFTAGSHLTVQVPNGARRNYSLCSDPADTGCYQ

IAVKRDAAGRGGSISMADDVQVGQLLAVSAPRNNFELHPRASSFLFIAGGIGITPVLSMMRHLKRQG-RP

FKLIYCTRDADSTAFVQELTGPEFAAHVQLHHTQGDASQALDLWPVFETPGSA-HVYCCGPRGLMDAVAD

MSGHWPSGSIHFESFGVDASTYAADTAFTVRLHKTGINVPVDAGQTILQALRACGHHVSSSCESGTCGSC

RTTLLAGEAEHRDMVLSDDDKTHQIMVCVSRAKSAELVLDL

>A4JLU3|A4JLU3_BURVG

TTPQEDGFLRLKIASKEKIARDIWSFELTDPQGAPLPPFEAGANLTVAVPNGSRRTYSLCNDSQERNRYV

IAVKRD-NGRGGSISFIDDTSEGDAVEVSLPRNEFPLDKRAKSFILVAGGIGITPMLSMARQLRAEGLRS

FRLYYLTRDPEGTAFFDELTSDEWRSDVKIHHDHGDPTKAFDFWSVFEKSKPAQHVYCCGPQALMDTVRD

MTGHWPSGTVHFESFGATNTNARENTPFTVRLSRSGTSFEIPANRSILEVLRDANVRVPSSCESGTCGSC

KTALCSGEADHRDMVLRDDEKGTQIMVCVSRAKSAELVLDL

>2PIA|PDBID_CHAIN

TTPQEDGFLRLKIASKEKIARDIWSFELTDPQGAPLPPFEAGANLTVAVPNGSRRTYSLCNDSQERNRYV

IAVKRDSNGRGGSISFIDDTSEGDAVEVSLPRNEFPLDKRAKSFILVAGGIGITPMLSMARQLRAEGLRS

FRLYYLTRDPEGTAFFDELTSDEWRSDVKIHHDHGDPTKAFDFWSVFEKSKPAQHVYCCGPQALMDTVRD

MTGHWPSGTVHFESFGATNTNARENTPFTVRLSRSGTSFEIPANRSILEVLRDANVRVPSSCESGTCGSC

KTALCSGEADHRDMVLRDDEKGTQIMVCVSRAKSAELVLDL

>B5WEX9|B5WEX9_9BURK

TPPLEDGFLRLKIAGKEKIARDIWRFELTDPQGAPLPLFEAGANLTVVVPNGSRRSYSLCNDSQERHRYV

IAVKRDSNGRGGSMSFIDDTAEGDAVDVSLPRNEFPLDERAKSFVLVAGGIGITPMLSMARQLRAEGLRS

FKLYYLARDPEGTAFFDELTGDEWRSDVKIHHDHGDPSKAFDFWPVFERSKSAQHVYCCGPQALMDTVRD

MTGHWPSGTVHFESFGASNANARESTPFTVRLSRSGTSFDIPANRSILEVLRDANVRVPSSCESGTCGSC

KTALCSGEADHRDLVLRDDEKETQIMVCVSRAKSAELVLDL

>C6BEP5|C6BEP5_RALP1

MTTQDTGFLRLTIAEKEKIARDIWRFELTDPQGAPLPPFEAGSNLTVVVPNGMRRSYSLCNDSQESHRYV

IAVKRDSNGRGGSMSLIDGTSTGDLIDVSLPRNEFPLDERAKSFILVAGGIGITPMLSMARQLKAEGLRD

FKLYYLARDPEGTAFLDELSSDQWRSRVKIHHDLGDPTKSFDFWTLFERPKAA-HIYCCGPQMLMDTVRD

MTGHWPPGTVHFESFGANNANAQVNTPFIVRLKRTGTPLDIPADRSILDVLRAENVHVASSCESGTCGSC

RTALCSGEADHRDMVLRDDEKATQIMVCVSRAKSGELVLDL

>D0J436|D0J436_COMT2

MTTQESGFLRLKIAEKEKIARDIWRFELTHPEGAPLPPFEAGSNLTLVAPNGMRRSYSLCNDSQERNRYV

IAVKRDSNGRGGSMSLVDDTSAGDSIEASLPRNEFPLDERAKSFILVAGGIGITPMLSMARQLKAEGLRD

FKLYYLARDPEGTAFLDELSSDEWRSLVKIHHSFGDPAKAFDFWALFERPKAA-HVYCCGPQSLMDTVQD

MTGHWPSGTVHFESFGAGNANTKENTPFTVQLKRTGTSLEIPSDRSILDVLRASNVRVASSCESGTCGSC

RVGLCSGEADHRDLVLRDDEKDAQIMVCVSRAKSAELVLDL

>B1G6D8|B1G6D8_9BURK

SARNELSFRTLVVMKKDQLAEGIFRFEMRDEAGRELPPSTAGAHITVRVPNGANRNYSLCNDPAETDRYV

IAVKRDAAGRGGSISMADDVAEGDRIDVSEPRNEFGLSERARSFVFVAGGIGITPVLSMMRHLKTAG-PR

FKLYYVSRSPETTAFIDELSSDEWKPHVVIHHDHGDLANAFDFWPVFEKPASGAHVYCCGPRALMDGVRD

MTGHWPAGTVHFESFGADQ----SRAPFSVKLERSGRSFGIPKDRSILEILRDNGIRAPSSCESGTCGSC

RTTLCAGEADHRDMVLSDDEKRDQIMICVSRAKSEELVLDL

>Q05182|PHT2_PSEPU

SEQLDDGFTGLKVIAKTEIAQGIFRFELAHPQGMLLPAFTAGAHLRVRVPNGSIRNYSLSNDPQERERYV

IAVKRDANGRGGSVSMADDIEAGDLLPVATPQNEFELIENARQFIFVAGGIGITPILSMMRHLKASTDLP

FKLYYCTRNPELTAFRDELLGAEFANTVVIHHDFGNRADAYDFWPVFDKPSSGTHVYCCGPRPLMDSVLD

MTGHWPPGSIHFESFGVDQSRFAENRPFSVTLGRSGIDLEIPVDRSILEVLRDNGIRAPSSCESGTCGSC

RTRLIEGDVEHRDMVLREDEQHDQIMICVSRARNDVLVLDL

>B2JRN3|B2JRN3_BURP8

-----MNTIPVRVAVADTIAQDIRLLKLEANDGSSLPGWKPGSHIDLHLGQGITRQYSLCGPQGDTSAYQ

VAVKLEPQSRGGSKYVHESLKVGSELLISPPRNHFEMKADAGHTLLVAGGIGITPIISMARELAEKR-QQ

FELLYFTRSEHHAAFREELEGGPLGESCKLLCGLERDDVEEALEGALDDRYDGSQLYLCGPKPFMDTVRA

VAVGWPDEAVHFEYF-AGAEPVGEQTSFDLKLARSGKTVTIPANKTIVDVLREEGVEVETSCEQGVCGTC

VARVLDGTPEHHDCFLTSQEQGDCMAVCISRSKSRLLVLDL

>Q1N833|Q1N833_9SPHN

FPNSETPWRLARVVATEDIAQDIRLFTLESASPSSLPIYEAGAHIDVRLP-GLVRQYSLIGRPGHAGQWQ

IAVKRDAAGRGGSMAMHSRLQEGTLVEVGSPRNHFPLQVSSGRHVLIAGGIGVTPLISMAYALAA---KD

FALHVFARTPAHVPFDQTLQRFPFAARIQLHCDDGK-G-IEDIASALGQYQADDQVFLCGPAGFMERVKA

VAAGWPADAIATESF-SPPRPSHPDVPFTIELARSGRQLRVEVGQSIVEVLESERLAIDTVCRQGVCGTC

QCRVLSGEIEHRDAVLTTAERGNKILLCVSRGTGSGLVLDL

>C5AL94|C5AL94_BURGB

---MQTFTFKVRVDAIRDEAQDIRSFRIVRLDGLPFDVHEPGAHIDVTAPSGITRQYSLCGDPAERGAQL

FAVKKEAASRGGSRSLHDDVVVGSELIVSPPRNLFRLAEQARTHVLIGAGIGITPLLSMAYRLTARG-TP

FVLHYFARSEAHAAFLPTLS-RPLARHLRLHFGVEPADLEAALHDCLSGAGPGTHVYTCGPGPFMDQVVE

AGEAVGADAVHLERF-AAEPVA-TLDSFEVQVAGSGRVVQVGKGQTIVGALAEIGIDIDTSCGEGVCGTC

IVDVVDGEPDHRDHCLSKAERNRVICCCVSRSRSKRIVLDV

>F2LCE2|F2LCE2_BURGS

--MHSSSSFKVRVDALCDEAQDIRSFRIVRIDGQAFDAHEPGAHIDVTAPSGVTRQYSLCGDPSDRDTQL

FAVKKESESRGGSRSLHDDVKVGSELTVGTPRNLFRLADQARHHVLIGAGIGITPLLSMAYRLAASG-AS

FELHYFARSEAHAAFLPTLRLERLAPQVHYCFGVAPTDLEAALRNCLADAGSGTHIYTCGPGPFMDKVVE

VGEVVGPDAVHLERFAAEP----VAANFEVEVSGSGQVVQVGKGETIVVALARIGIEIDTSCGEGVCGTC

IVDVVDGEPEHRDNCLSKAERNQVICCCVSRSRSKRLVLDV

>Q2T8G9|Q2T8G9_BURTA

---MQASFHLVRVDALQDEAQDIRSFRISRVDGQPFDAYEPGAHIDVTSPCGLIRQYSLCGDPAQRGSYQ

FAVKKEPRSRGGSRSLHDDVRVGTELEVGAPRNLFRIADDARAHLLIAAGIGVTPLLSMAYRLVERR-AP

FRLHYFVRSRAHAAFLDRLS-SPFAPHVALHVGIEPPDLAGALDACVADATPGTHVYTCGPAPFMDQVVA

RAARIDEDAIHLERFPPLP----VARAFEVRLQRSGRSIRVDDSTSIVDALASIGVEVDTSCGEGVCGTC

MVSVVSGEPDHRDHCLSKAERSKVICCCVSRSRSPVLVLDL

>B9BKU3|B9BKU3_9BURK

---MQDNRHQVRIDALIDAAQDIRCFRVSRVDGQPLDAYEPGAHIDVTAPSGVTRQYSLCGRPDERGSYL

FAVKKETQSRGGSRSLHDDVRVGTELSIGTPRNLFRLADDASEHVLIAAGIGITPLLSMAYALAQRG-AR

YRLHYFARSRDHAAFVDELS-APFASHVTFHYGVERDALARELGRCVESVDAQAHVYTCGPAPFMDAVIA

AAARVPDDAIHLERFAAAS----ASE-FEVRLQRSGQSVRVAPDTSIVDALARIGIEVDTSCGEGVCGTC

MVPVVDGEPDHRDHCLSKAERNSVICCCVSRARSPVLVLDL

>Q392L1|Q392L1_BURS3

---MQANRHQVRIDALIDAAQDIRCFRVSRVDGQPFDAYEPGAHIDVTAPSGITRQYSLCGNPDERGSYL

FAVKKEAQSRGGSRSLHDDVQVGAELSIGAPRNLFRLTDDASEHVLIAAGIGITPLLSMAYALHQRG-AS

YRLHYFARSREHAAFVAELS-APFASHVTFHYGIEPAALAAELGRCVESVDARAHVYTCGPGPFMDAVVA

AAARLPEDSIHLERF-AAEPAATATDGFEVRLQRSGQSVRVTPDTSIVDALARIGIEVDTSCGEGVCGTC

MVPVIDGEPDHRDHCLSKAERNTVICCCVSRARSAVLVLDL

>B1FG00|B1FG00_9BURK

---MQANRHQVRIDALIDAAQDIRCFRVSRVDGQPFDAYEPGAHIDVTAPSGVTRQYSLCGRPDERGSYL

FAVKKEARSRGGSRSLHDDVCVGAELAIGAPRNLFRLTDDASEHVLIAAGIGITPLLSMAYALEQRG-AR

YRLHYFARSREHAAFVDELSD-PFAAHVKFHYGIEP--DAAELGRCVESIDAHAHVYTCGPGPFMDAVVA

AASRIPEDAIHLERFAAEP----AAAGFEVRLQRSGQSVRVAPDTSIVDALARIGIEVDTSCGEGVCGTC

MVPVLDGEPDHRDHCLSKAERNTVICCCVSRARSAVLVLDL

>B4EL71|B4EL71_BURCJ

---MQANRHQVRIDALIDAAQDIRCFRVSRVDGQPFDAYEPGAHIDVTAPSGITRQYSLCGNPDERGSYL

FAVKKEARSRGGSRSLHDDVSVGAELSIGTPRNLFRLTDDASEHVLVAAGIGITPLLSMAYALHKRG-AR

YRLHYFARSRDHAAFVDELS-APFAAHVTFHYGVEPDALAAELGRCMESIDPTAHVYTCGPGPFMDAVVA

AAARIPEDAIHLERFAAEP----AEA-FEVRLHRSGQSVRVAPDTSIVDALATIGIEVDTSCGEGVCGTC

MVPVLDGEPDHRDHCLSKAERNTVICCCVSRARSAVLVLDL

>B1K5Y7|B1K5Y7_BURCC

---MQANRHQVRIDALIDAAQDIRCFRVSRVDGQPFDAYEPGAHIDVTAPSGVTRQYSLCGNPDERGSYL

FAVKKEAQSRGGSRSLHDDVSVGAELSIGTPRNLFHLTDDASEHVLIAAGIGITPLLSMAYALHKRG-AR

YRLHYFARSRAHAAFVDALS-APFASHVTFHYGVEPDALAAELGRCIESIDLKAHVYTCGPGPFMDAVVA

AAARIPEDAIHLERF-AAE----PSAGFEVRLQRSGQSVRVAPDTSIVDALATIGIEVDTSCGEGVCGTC

MVPVIDGEPDHRDHCLSKAERNTVICCCVSRARSAVLVLDL

>O86996|O86996_ACILW

APYYQSHLFYNRIDLILDAQPGAASFLLSRSWKEPPSAYASTACVTSPAPPGVTRQYSLCGDPDHRETHL

FAVKRE-ASRGGSRSLHEDVTVGCELTIGAPRNLFQLAPGATEHILIAAGIGVTPLLSMAYRLLKQE-QR

FVLHYFARSAEHAAFLPLLS-RPFNDFVRFHFGVERDQLDAALAGCVANARDGSHLYTCGPAPFMERVVA

IGERVAADAIHLERF-AAEPAA-TLDSFDVRIASSGATVHVDKHTTIVAALASIGIEVDTSCGEGVCGTC

MVDVVSGTPEHRDHCLSKAERGKVICCCVSRAASPLLVLDL

>Q9Z9X8|Q9Z9X8_9GAMM

---MERTTFSVRVDSVRDEQHGVRSFSVSRLDGQPFERYEPGAHIDVTGPSGVTRQYSLCGDPDHRETHL

FAVKRE-ASRGGSRSLHEDVTVGCELTIGAPRNLFQLAPGAIEHILIAAGIGVTPLLSMAYRLLKQE-QR

FVLHYFARSAEHAAFLPLLSRAPFNDFVRFHF-GVAR-DQLDLAGCVANARDGSHVYTCGPAPFMERVVA

IGEHVAADAIHLERF-AAEPAA-TLDSFDVRIASSGATVHVDKHTTIVAALASIGIEVDTSCGEGVCGTC

MVDVVSGTPEHRDHCLSKAERGKVICCCVSRARSPLLVLDL

>D5NG40|D5NG40_9BURK

GKIMERTTFNVRVDSVRDEAHGVRSFSVSRLDGQPFDHYEAGAHIDVTGPSGVTRQYSLCGDPDHREAHV

FAVKREATSRGGSRSLHDDVTVGSELTVGAPRNLFQLAQHAREHILLAAGIGVTPLLSMAYRLLEQR-QR

FALHYFARSAEHAAFLQLLSRAPFSDYVTLHF-GVA---RDQLDAVLANAREGTHLYTCGPAPFMERVVA

IGERVAPEAIHLERF-AADPVS-ALDTFDVQIASSGATVRVDKDTTIVAALASIGIEVDTSCGEGVCGTC

MVDVLSGTPEHRDHCLSKAERGKVICCCISRAASPVLVLDL

>B2T4P0|B2T4P0_BURPP

---MERTTFNVRVDSVCDEAHGVRSFSVSRLDGQPFDRYEPGAHIDVTGPSGVTRQYSLCGDPDQRDAHL

FAVKREDTSRGGSRSLHEDVTVGSELTIGAPRNLFKLAPDAEEHVLIAAGIGVTPLLSMAYRLLKQQ-ER

FVLHYFARSAEHAAFLPLLSRAPFSDYVKLHF-GGA---REQLDAVLANAREGTHVYTCGPAPFMERVVA

IGERLSSDAIHLERF-AAEPVS-ALDTFDVRIASSGATVRVDKDTTIVAALASIGIEVDTSCGEGVCGTC

MVDVVSGTPEHRDHCLSKAERGKVICCCISRATSPVLVLDL

>B1FUC5|B1FUC5_9BURK

ESIMQAQALKVRVDALSEEAHGVRSFKISRLDGMDFERFEPGAHIDVTSPSGITRQYSLCGDPECVSSQV

FAVKKEEQSRGGSRSLHEEVSIGTELQVGAPRNLFQLNDGASEHILIGAGIGITPLLSMAYRLAARG-AR

FTLHYFARSEAHAAFMTLLT-RPFDSHVRLHFGVRRDALAAELDICLSEAGADAHVYTCGPAPFMDLVVD

TAQRLPADAIHLERFAEPA----NETSFQVQLASTGQTVKVDEKTSIVDALATIGIEVDTSCGEGVCGTC

MVDVISGEPEHRDHCLSKAERNSVICCCVSRSRTPVLVLDL

>D5WEA7|D5WEA7_BURSC

---MQASTLKVRVDALREEAHGVRSFSISRLDGLPFERYEPGAHIDVTSPSGVTRQYSLCGDPERLDSQI

FAVKKEEQSRGGSRSLHEEVSVGTELSVGAPRNLFRLEENADEHILIGAGIGITPLLSMAYRLAARQ-AR

FTLHYFARSEAHAAFMTLLT-RPFAQHVKLHFGVESDELKPALDACLRDAGANAHVYTCGPSPFMDLVVA

SAQCLPADAIHLERF-KAEPAP-SLDSFDVQLASSGQTVRVDANTSIVDALASIGIEVDTSCGEGVCGTC

MVDVVSGEPEHRDHCLSKAERNSVICCCVSRSRTPVLVLDL

>E1TJS1|E1TJS1_BURSG

---MEASVLKVRVDAVREEAHGVRSFSVSRLDGLPFERYEPGAHIDVTSPSGVTRQYSLCGDPECLDTQT

FAVKKEQQSRGGSRSLHEDVGVGTELSVGAPRNLFRLVEGADEHVLIGAGIGITPLLSMAYRLAARK-AH

FTLHYFARSESHAAFMTLFT-RPFDAHVKLHFGVERDALPDALDACLRDASDNAHVYTCGPAPFMDLVVE

TAQRLPAESIHLERF-KAEPAP-SLDSFDVQLASTGQTVHVDAKTSIVDALASIGIEVDTSCGEGVCGTC

MVDVVSGEPEHRDHCLSKAERNSVICCCVSRSRSPVLVLDL

>D9WS53|D9WS53_9ACTO

---MRHTMIRATVSEITAETGAVKCFRLRRSDGLPFAPYEAGAHVDVLGPSGITRQYSLCGPPDDGGAYV

IAVKRE-CSRGGSSALHDQVVAGDELVVSEPRNLFRIEPGAARHVLVAAGIGITPLLSMAYQLRRTG-AP

FRLHYFASGREGAAFVPLLE-SEFAADVEFHFGLGRDAQPGLLARALADASPATHVYTCGPKEFMQRVVE

VATSLPETHVHVEHFQPLE----PAAAFDVELD-TGEVFTVPPGRSIVDVLVEGGIEVDTSCREGICGTC

VLPVLDGVPDHRDNCLTRKEKGDQIATCVSRARTARLVLEL

>Q4UZY0|Q4UZY0_XANC8

-MTGEDDGMEVRVAEVVDQGHRQRAIRLE-PIGAELPAFEAGAHVDLHLPDGLIRQYSIASAPHARDHYL

LCVKLA-ASRGGSRHLCEQLAAGDRLQISTPRNLFPLHP-GERHVLLAAGIGITPLLSMVEALEARG-EA

FVLHYYARRQADVAFGQRLQQGFQHGQVQVHLSDGGESPRVHIPTELRKAHARDQLYLCGPAAFMDHFSA

LALGWAPAQLHREHF-AAP----HAAAFEVELAASGRVVQVAAECSIASALMAAGVEVPLSCEQGMCGAC

LTGVLDGVPDHRDSVLSDSEHNTQITLCCSRSRTPRLVLEL

>F0C4C0|F0C4C0_9XANT

-MTAEEHAMEVRVADVFDQGHRQRAIRLE-PVGAELPAFEAGAHVDLHLPDGLIRQYSIASAPHVRDHYL

LCVKLA-ASRGGSRHLCEQLSTGDRLRISTPRNLFPLHP-GERHVLLAAGIGITPLLSMAEALEARG-ET

FVLHYYARRHADVAFGQRLQRGFTHGQVQVHLSDDGESPRVHVPEELGQARARDQLYLCGPAAFMNHFTL

LAKGWAPEQLHREHF-AAAETNAADSAFEVELAASGRVVQVPAECSIASALLDAGIEVPLSCEQGMCGAC

LTGVLAGTPDHRDSVLSDSAHNTQITLCCSRSRSPRLVLDL

>F0B907|F0B907_9XANT

-MTAKERAMEVRVADVVDQGHRQRAIRLE-PVGAQLPAFEAGAHVDLHLPDGLIRQYSIASAPHVRDHYL

LCVKLA-ASRGGSRHLCEQLSTGDRLQISSPRNLFPLHP-GERHVLLAAGIGITPLLSMAEALEARG-EP

FVLHYYARRHADVAFVQRLQQGFTHGQAHVHLSDGGQSPRVHVPDALVQAGAHDQLYLCGPAAFMDHFTQ

LARGWTAAQLHREHF-AAADTGAADSAFEVELAASGRVVQVPADCSIANALLDAGIEVPLSCEQGMCGAC

LTGVVAGTPDHRDSVLSDAERNRQITVCCSRSRSRRLVLDL

>D4SZR7|D4SZR7_9XANT

-----MSLHEVRVADVVDQGHRQRAIRLE-PVGAQLPAFEAGAHVDLHLPDGLIRQYSIASAPHVRDHYL

LCVKLA-TSRGGSRHLCEQLAPGDRLRISTPRNLFPLHP-GERHVLLAAGIGITPLLSMAEALEARG-EP

FVLHYYARRHADVAFSQRLQQGFTHGQVSLHLSDGGQSPRVHVPEELGQARACDQLYLCGPAAFMDHFTV

LAKGWTAAQLHREHF-AAPAPSAADSAFEVELAASGRVVQVPADCSIASALLDAGIEVPLSCEQGMCGAC

LTGVLAGTPDHRDSMLSDAERNRQITVCCSRSRSPRLVLDL

>C6CKL1|C6CKL1_DICZE

-----MNTLNVVVDALSRQGEGNLALRLTAADEQPLPAFEAGAHVDLHLPNGLTRPYSLAGDPSDRSHYL

LCVRHDAASRGGSRYVHTALRVGQRLMISAPRNAFALQP-ACGYLLLAGGIGITPLLAMAHALDQTG-LP

FELHYFVRHRRELAFREQLSRGFRHGRCRIGCADEGGSPRNQLPDSLRDGAEGRRLYLCGPAGFMAHVTR

SALHWPASAIHTEAF-GAPVPVGDQQAFTVELASSGRVFTVPPEKTIAGVLQEHEVAVPLSCEMGMCGAC

LTPVCAGTPDHRDSVQSEAEKHQQIALCCSRSRSAHLVIGL

>E6WJJ7|E6WJJ7_PANSA

----MSTTLQVVVDGMYRQGRHNLAINLVAENGQPLPGWQPGAHIDVHLRDGLVRQYSLTGQSDNSHHYQ

ICIARDKQSRGGSRYVHDMLRPGQVISISPPRNLFPLIP-AGKVILMAAGIGITPLYAMAEALEAAG-VP

FVLHYYVKQRGDEAFLPELSKPFQHGFCEIWHSSEGRSPRHHAPAELLDPAEDTHLYLCGPEGFMSHIHT

AAIGWREANIHTEAF-KPQTT-AGEEGFTVTLASSGQQWPVAPGQTIATVLLENGVDVPLSCEMGMCGAC

LTRVTAGEVDHRDTVQSAAEKEQYIALCCSRSLSPNLVIEL

>G0E7P9|G0E7P9_ENTAK

----MRTLIEVVVDGLWREGEHNLAVRLVTPDGDPLPAWQPGAHIDLHLANGVTRQYSLTGSHRRLERYL

ICVARSAESRGGSRYIHETLRPGQRLMISLPRNQFALQT-ARHVTLMAAGIGITPLYSMARALAAQG-KP

FTLHYYLKRLENGALVAAIARKLAPENVRIYSSAEGESPRENLPGRLSVPHREDRLYLCGPAGFMAAVRQ

AAAGWQAEQIHEEAF-QASMPADQEETFTVTLASSGQCWPVAANQTIVQVLQENGVAVPLSCEMGICGAC

LTRVVEGEVDHRDTVQSAAEKVQHVALCCSRSRSTNLLIDL

>B5XQS0|B5XQS0_KLEP3

----MRDIFPVVVDGLWRQGAKNLAVRLVSAEGQPLPAWTPGAHIDLHLPCGLIRQYSLTGSPAERDHYL

LCIAREAQSRGGSGYVHDTLRPGQPLMISAPRNHFPLHE-GGHVVLLAAGIGITPLLAMAHARAASG-AS

FTLHYYVSRAQEAAFATEIARQLTGGICQIHCSDEGQSPRQRLAQDLGAPDADTRVYFCGPPGFMARVRD

TARGWEEAQLHSEAFQPPA----PTA-FTITLASTGEHWPVPGNKTIAQVLQEHGVAVPLSCEMGICGAC

LTPVREGTVDHRDTVQSEAEKEQHIALCCSRSLSANLVIDL

>D3RCB5|D3RCB5_KLEVT

--MHSHDLISVSVGEIRPNGEGNLSLILQAAAGEILPAYSAGAHIDIIIP-GGPRQYSLCGTPDRSNTYE

ICVRLTDTSTGGSRYLHQQVKSGDRLAISPPRNHFPL-PEAGRYLLFAGGIGITPLLAMAEAIAARK-GA

LELHYYVASSRQTAFSPRLTQLAAGGTVAIHCSEEGASFRQRIPACLTTPDPDTAVVACGPEGFIQRLQS

VMERWSPSQFVFERF-TPAAENNAKNAFYIELASSGQRLQVAADQTIAQVLQHAGVEVMLSCEQGMCGSC

ITGVLDGIPEHRDSVLTAEEKNDQITLCCSRAKSPVLVLDL

>C4X9D8|C4X9D8_KLEPN

--MHSHDLISVSVGEIRPNGQGNLSIILQTAAGEILPAYSAGAHIDIIIP-GGPRQYSLCGTPDRSNTYE

ICVRLTDTSTGGSRYLHQQLKAGDRLAISPPRNHFPL-PQAGRYLLFAGGIGITPLLAMAEAIAARK-GA

LELHYYVANSRQTAFSPRLNQLTANGTVAIHCSEEGASFRQRVPECLTVHHPDTVVIACGPEGFIQRLRS

VMERWSPSQFVFERF-TPAAENNAKNAFYIELASSGQRLQVAADQTIAQVLQHAGVEVMLSCEQGMCGSC

ITGVLDGIPEHRDSVLTAEEKNDQITLCCSRAKSPGLVLDL

>D8MTT4|D8MTT4_ERWBE

--MKDAEIIPVTIKAITENGAGNRSLQLIAQRDYALPGWLPGAHIDLFIPEG-PRQYSLCGEHGDAEYYE

ICVKLA-LSSGGSQHIHHHFKPGDSLTISAPRNHFPLLD-APRYLLMAGGIGITPLLAMAEAIARQG-GE

CELHYYVSNPEQTAFLPRLSAQPLAERVFFHYSDANDSLRHNTPLALTQSDPHTRVMACGPDGFIQRLQD

IMQHWQPDQLHFERF-SNPELARGNTAFHIELSSTGQRYLVSPEQTIAEVLLSAKVDIMLSCEQGICGSC

ITDVIDGIPDHRDCVLTNEEKNSQITVCCSRAKSPVLVLDL

>E8XZ38|E8XZ38_RAHSY

MTMKDAGLIPVTIKTANINGVGNISLQLVAQQGSLLPAYLPGAHIDVFIPEG-PRQYSLCSENSSGEYYE

ICVKLAENSTGGSHFIHHHFRPGDNLTISPPRNHFPL-PQAGRYLLFAGGIGITPLLAMAEHLAEQQ-IE

FELHYSVSDREHIAFLPRLTAPALAKHVFFHDSSANDSLRHNTPLSLLQPDENTRVMACGPDGFIQRLKD

IMQHWQENQLSFERF-SNANLNNDSQAFHIQLNSTGQRYLVGPHQTIAEILLSARVDIMLSCEQGICGSC

ITDVIDGIPDHRDCVLTEEEKNTQITLCCSRSKSPVLVLDL

>D1RTD0|D1RTD0_SEROD

MTMKDAGLIPVTIKAIGENGKGNISLQLIAQQGGLLPAYLPGAHIDVFIPEG-PRQYSLCTENNGGEYYE

ICVKLAENSSGGSHFIHHRFSVGDTLSISMPRNHFPL-PLAGRYLLFAGGIGITPLLAMAEQIAQQE-ID

FELHYYIADLDKAAFTSRLNAPELAKHVFLHDSGANDSLRHNTPLSLRQPDENTRVMACGPDGFIQRLED

IMQHWQKNQLSFERF-SNKNINNDDQEFHIQLNSSGKCYLVGPNQSIAEVLLSAKVDIMLSCEQGICGSC

ITDVIDGIPDHRDCVLTEEEKNTQITLCCSRSKSPVLVLDL

>E0LZA9|E0LZA9_9ENTR

MTMNDAGLIPVTIKAIGENGKGNISLQLIAQDGGLLPAWLPGAHIDVFIPEG-PRQYSLCNENHGGEYYE

ICVKLADNATGGSHFIHHHFSAGDTLNISMPRNHFPL-PAAGRYLLFAGGIGITPLLAMAEQISRQG-IE

FELHYYISGLDKAAYIPRLTAPSLAKNVFLHDSSANDSLRHTTPLSLCQPDANTQVMACGPDGFIKRLEE

IMQHWHKNQLSFERF-SNKNINKANSEFHIELKSSGKRYLVGSDQSIAEVLLSARVDIMLSCEQGICGSC

ITEVIDGIPDHRDCVLTEEEKNTQITLCCSRAKSPVLVLDL

>D8IXA7|D8IXA7_HERSS

FNGSAATTLMLRVRAVRQAAQDIRLLELVSQDGAALPPWTPGAHLRLQLQDGLVRAYSLCNTPQQRDCYL

IAVKREPDSRGGSAFLHEQVQEGTTISASPPVNAFPLIADVRAPLLLAAGIGITPLHAMAAELATQG-RR

HRLHYFARSLAHAAFFHELAAG--SQGMSLHLGLDGAETEHAIAAALAAAPRSSPLYVCGPSPFIDAARR

HAQGWREQDIHFERF-AAPAMPARPVTFELVLQRSGLRCQVLPGQSIVAAAAQVGVVIGTSCGEGFCGSC

ESTVLEGQPWHRDSVLSAAERGRRILPCVSRCAGTRLVLDL

>A2SP35|A2SP35_METPP

TGKYPKTALNLRVRQITYQGIGINAYEFVREDGGELEEFTAGAHVDLYFRDGRVRQYSLCNDPAERRRYL

IAVLRD-NGRGGSIAIHERVHTQRLVAVGHPRNNFPLIEGAPHQILLAGGIGITPLKAMVHRLERIG-AD

YTLHYCAKSSAHAAFQEELAPLAAKGRVIMHFDGGNPAKGLDIAALLRRYEPGWQLYYCGPPGFMEACTR

ACTNWPAEAVHFEYFVGAPVLPAIGSGFQIKIASTGTVLTVPNDKSIAQVLGEHGIEVPTSCQSGLCGTC

KVRYLAGDVEHRDYLLSAEARTQFLTTCVSRSKGATLVLDL

>A4SZ42|A4SZ42_POLSQ

KKVTDNKLLHLKIKQIRQEASGIHSYELVSEDGKALPSFDAGSHIDLHLPSGSIRQYSLSNDPAETHRYV

VGILRD-QGRGGSKEVHQALRVGDFLAVSRPRNHFHLDESAKKVILLAGGIGITPLKSMGHRLKSLG-IP

FELHYCARAQENIAFPQDLQNLSDSGEIQFHLDDGIPGNGLNISEMIEDLESGAHLYYCGPAGFMKACAQ

AATKRSDIHVNCEHFKAPE----KEAELAIQIQSTGQKITLSRSESLIDVLAKLGVEVSTSCQSGLCGTC

KTRYISGDVEHGDCILSDAEHTEYLTPCISHIKSGTLVLDL

>B7WQU2|B7WQU2_COMTE

APYKEGEPIEMVVRAIRMQAEGIHSFELVQSDGLELPAVEAGAHVDVHLPGGVVRSYSLAGDPAHRTHWT

LGVLRELKGRGGSKAMHESLRVGDTVKIGWPRNAFKLATGAAHSILVAGGIGITPLKAMAHTLASRG-ES

FELHYCSRTPKHAAFMDELRSLVSAERLHMHHDGGDRAKALDLIKLLKSAAPGTHVYYCGPSGFMDACTE

AAKHWPSGTVHCEHFKAPE----PKSSFEVKLAKSGETVQVLPDQTIVRALELAGHRVATSCLSGLCGAC

KVEVLEGEVDHQDYILTDEERTHCMTACVSRAKSKCLVLDL

>F0DYG0|F0DYG0_9PSED

LANPNPALLELLIRRVTREAQGINSYELVDPNGGALPPFTAGSHIDIHLPNGMVRQYSLSNSPAEPHRYV

IGVLRD-RGTGGSIAVHQ-LHVGETVRVSVPRNNFMLSKEARKSILLAGGIGVTPMKSMMHALESAK-QE

YELHYCCKGPEFAAFSEEMEELIGEGKVVLHFDGGDPSKSFDLQSFLKDAPEGCHVYYCGPAGFMNACKE

AAAHWPAGTVHFEHF-KAPVKAPQSNSFVARIASTGEEIVVQAHENLAQVLQDAGYPVETSCQSGLCGTC

KIRYLEGEVDHQDYILDDSEKGSCLTACVSRAKSKLLVLDL

>G4MHM6|G4MHM6_9BURK

STQADPDPLELLVRQIRFEGQGIHSYELVDPEGAALPPFTAGAHIDIHLA-GVVRQYSISNAPRERHRYV

IAVLRD-RGRCGSRALHEQLRVQDIVRVSRPRNNFRLVDGARRVLLIAGGIGVTPLKTMAHELEEAG-VE

YEMHYCAKDARCAAFAEEFAPMRASGRLHFHFDGGDPSADLDIRGLLNQPRDGEHVYYCSPGGFMKACAE

AAEHWPAGTVYFEHFKALE----RSPHFIVRIASTGQEIEIAKEQTIADALVQAGVAMETSCCAGLCGTC

KVRYLEGEVEHNDFILSDEEKTEYLTACVSRAASKVFVLDL

>B1FTA3|B1FTA3_9BURK

VEDIEAATLDVRVTQIRYEGKGINSYELTSLNGKALPRFEAGSHIDMHLKNGAIRQYSLCNPPAERNRYV

IAVLKD-NGRGGSRAVHESIAAGDIVTISKPRNHFPLSEDAKKVIMIAGGIGVTPLKAMAHELEERG-ID

FVMHYCARSAEAAAFGPELERMQRSGRLRYHFDDGEVTNRLDLRALLHEPTPGTHVYYCGPGGFMAACAD

AANHWPKGTVHFEHF-KAPEQPNEHQGCDVTIASTGQVVHVGPSQNFSEALNEAGIEVPTSCCAGLCATC

KVRYLEGEVEHNDFILDDADRKEFLTICVSRPVSKTLVLDL

>D5NFE7|D5NFE7_9BURK

IQDIEAGTLDVRVKQVRYEGKGINSYELTSPGGEKLPPFEAGSHIDVHLKNGVIRQYSLCNPPAERHRYV

IAVLKDEAGRGGSRSMHDDVAAGDIVTISRPRNHFSLDGNAKKVILIAGGIGVTPLKAMAHELEAHH-LD

FEMHYCARSREAAAFSEELATMLSTGKLHYHFDDGEKRNQLDLAKLLSQPSSGTHVYYCGPSGFMKACAD

AASHWPKGTVHFEHF-KAPEQPRESAGCDVTIASTGQVIHVGADQSLAEALNAVGVEVPTSCCAGLCATC

KVRYRDGEVEHNDFILSEEERQEYLTACVSRPVSKTLVLDL

>A7IMP6|A7IMP6_XANP2

--MSDEALQSVVVAKKTVEAENMVSFELVPAEGGELAPFTPGAHLDVTIPGGLMRQYSLCNSASERHRYV

IGVWKDANSRGGSKALFQQVNEGDRLQVGEPRNRFAVPRDVKRALLFARGIGATPILSIADHLKAKN-IP

FEFHYLFAGGSPGSFRSIIEASTFAENTSFYFEAAEPR--LNPAALLADRPDDTHLFLCGVDWWLDPVIA

NAQGYGVNRIHVERFAPPP----LLD-FDVKIASSGKVIKIPGDRSVTAALEEAGVKVPTSCEQGACGTC

KVKVLEGEIDHRDKRLKPEEKQGYFLACVSRAKGDLLVLDL

>A4YP96|A4YP96_BRASO

--MSD-SLHTVVVARKAIEAHNMASFELVPADDQPLPGFTPGSHIDVTLPNGLTRQYSLLNSATERDRYC

IGVWKDANSRGGSKALHLDVNEGDRLQVSRPRNRFKIPKDIKRALLVARGIGVTPILSIADTLKAKK-IP

FELHYVFALMSPDSFRGTIEASSFADNTTYYKEATEDNQLLKAADLLADRPDDTELFICGVDWWMDPIIA

LAKGFAEERIHVERF-TAKAAAA-DKVFDVTIKSTGATFKIPGDKTVTAFLEENGVKIATSCEQGMCGTC

KTKVVDGDIDHRDKRLSAAQREGYFLPCVSRAKGDRLVLDL

>A5EDW3|A5EDW3_BRASB

--MSD-TLHTVVVARKAIEAHNMASFELVPADDHPLPGFTPGSHIDVTLPNGLTRQYSLLNSAAERNRYC

IGVWKDANSRGGSKALHLDIKEGDRLQVSRPRNRFKIPKDTKRALLVARGIGVTPILSIADTLKAKA-IP

FELHYVFALMSPDSFRGTIEGSSFAENTRYYTESSEDNQLLKAADLLADRPDDTQLFICGVDWWMDPIIA

QAKGFAEERIHIERF-TAKAAAA-DKVFDVTIKSSGATFKIPGDKTVTAFLEEHGVKIATSCEQGLCGTC

KTRVVEGEIDHRDKRLSAAQREGYFLPCVSRAKGDRLVLDL

>Q7W422|Q7W422_BORPA

-------MLNLCVRSITYEAEGIRSFELVHPLGKDLPPFEAGAHIDVSIPGGFTRQYSLC-DPSQRKHYR

IAVLED-GGRGGSVALHTALRAGEMIEVSEPRNLFPLDSGAGHKVLLAGGIGITPVLAMAEALQRSG-HS

FELHYCTRSPERTAFRERLQTLAAQDKAFFHHDMGDPARGLDIAALLARPEPGSQLYFCGPPGFMKAVQA

ASAHWPQGSVHYEYF-GVNPALTGQVAGEVRLAKSDRILAVRPGQTLLQAIREAGVACESSCESGVCGTC

KVRYFSGQPEHNDYVLSDEERTEFVLVCCAGAGPQALLLDL

>F0GAF4|F0GAF4_9BURK

-MTDRTETVEVLIEPADGGTHDTRVFRMRRRDGLPLPPYRPGSHIDVYLPNGLVRQYSLCGADCHAPEYR

IAVKRSPVSRGGSAWLHDQASTGMALRIGLPRNAFALSDTAACHLLIAGGIGVTPILSMAHALHAAG-RR

YRFDYFARRDEDVVFRDEITAAPLAAHTRLHLGLGPEMARERVVTLLAGAAADTHVYVCGPSAFMQMTVE

LA-ALGDANVHQEAFSA-P----DLSEFELRLEGRADAIAVPPGKSALACLREAGVPVDSSCEVGVCGTC

AMRVVAGELLHRDTYLTDDERGTVFLPCVSRARSAVLVLDL

>B5K3D1|B5K3D1_9RHOB

APAGPKVELRTKVVRKWQVANQVIAYRLESI-GGQLPTAHPGSHIDVSLPNGMSRQYSLTNGPGETDHYT

IGVKRDSASRGGSICLQDDVQEGDVLAISAPHNNFPLRRDAVKTILIAGGIGLTPMLAMAQALKVQD-LP

FELHVFVRLKNDLAFSNLLD--ELGVSVVPYIGLSREVTGGAIADIVGNYTPGQHVYLCGPGSMLEVARG

MAAGWPDSAVHFEYF-KNTNDIDNSTTFEIALARSALTLQVPAGKTILQVLNENGITAPSSCEQGACGTC

LMTVIDGEPDHKDVYLSSIERNQKIVTCVSRSKSQRLVLDI

>Q92XT7|Q92XT7_RHIME

DSRGRKAAIRVQVARKWQTADGIMAFQLRPV-RGLLPTFQPGAHIDVHLPNGLVRQYSLTNGPGETDCFT

IGVKLDPASRGGSQCLHDSVREGDVLAISEPRNNFPLRRDALKTIFVAGGIGVTPLLAMAQTLNNQS-LD

YELHYFAQNEQQLAFSECR--QALGDAVKPHLGLSPGDTVKELRRLLSAYLPDTQLYVCGPGPMLESTRS

LAAGWPEAAVHFEYF-KNTNVIDDSSSFEVALARSCLTIKVAAGQSILEAMREAGVDLPSSCEQGACGTC

LATVIEGEPDHQDVYLSPSERGTKIMTCVSRSKSARLVLDA

>E8TH11|E8TH11_MESCW

TTHGRKAALRVSVARKWQAADGIAGFELRPI-KGILPTFQPGAHIDVHMPNGEIRQYSITNGPGETDSFT

IGVKLERDSKGGSKCMHETVREGDVLAISEPRNNFPLRRDAIKTLFVAGGIGITPLLAMAQALKNQE-LA

HELHYFAQGEEHLAFADRL--KHLGDALKPHLGLSPDATGAELRQLLAGYRNGMHLYICGPGPMLEAARK

IAAGWPDSAVHFEYF-KNTNKIDDSSSFEVALARSCVTLQVPAGKTIMQVMRESGIDVPSSCEQGACGTC

VATLIEGEPDHQDVYLNDAERGTKIMTCVSRAKSARLVLDI

>G4K0C2|G4K0C2_9RHIZ

TTHGRKAALRVTVARKWQTADGIAAFELRPI-KGILPTFQPGAHIDVHMPNGEVRQYSITNGPGETDGFT

IGVKLERDSKGGSKCMHEAVREGDVLAISEPRNNFPLRRDAIKTLFVAGGIGITPLLAMAQALKNQD-LT

HELHYFAQGQEHLAFADRL--KRLGDALKPHLGLSPDQTGAELRQILTGYRDGMHLYICGPGPMLEAARN

IAAGWPDSAVHFEYF-KNTNKIDDSSSFEVALARSCVTLQVPAGKTILQVMRESGIDVPSSCEQGACGTC

IATVIEGEPDHQDVYLNDDERGTKIMTCVSRAKSARLVLDI

>A6ULN4|A6ULN4_SINMW

TAHGRKATIRVTVARKWLAADGIAAFELRPV-QGLLPTFQPGAHIDVHMPNGLVRQYSITNGPGESDSYV

IGVKLERDSMGGSLCMHETVREGDLLAISEPRNNFPLRRDAVRTIFVAGGIGVTPLLAMAQALKNQN-LD

YEFHYFAQNQEQLAFPEKT--ALLGEALKPHLGFLPETTAAKLKEILSGYRPDMHVYLCGPGPMLEAARR

IAAGWPETAVHFEYF-KNTNTIDDSSSFEVALARSCVTLQVTAGKTILETMREAGIDMPSSCEQGACGTC

LATVIEGEPDHQDVYLNDAERGTKIMTCVSRARSARLVLDL

>F6EDH6|F6EDH6_SINMK

TAHGRKATIRVTVARKWMAADGIAAFELRPI-KGLLPTFQPGAHIDVHMPNGLIRQYSITNGPGESDSYV

IGVKLERDSKGGSRCMHETLREGDVLAISEPRNNFPLRRDAEKTIFVAGGIGATPLIAMAQALKNQS-LD

FAFHYFAQNQAQLAFPEKT-A-LLGEALKLGLDPEG-T-AAKLKDILSGYRPGMHVYLCGPGPMLEAARR

IAAGWPETAVHFEYF-KNTNTIDDSSSFEVALARSCVTFKVPAGRTILDVMREVGIDMPSSCEQGACGTC

LATVIEGEPDHQDVYLNDAERGTKIMTCVSRAKSARLVLDL

>E1TJK7|E1TJK7_BURSG

HSMMKDNLIAARVIARERVACDVVSLRLASEANAELPAFEAGAHIDLHLRDGLVRKYSLCSDPSERAFYE

IAVKREPASRGGSVFVHDEIRVGDILSIGKPENYFPLAPDGSGAVLLAAGIGITPLLAMAHTLKRAG-PA

FEFHYFVRSLDDAAYARTLQE-QLAQVFTLHVGLTPELTRETLAELVRRMSSQEHLYFCGPAPFMDAADA

IA-HLPAQQIHCERFAATG----DAAPFEIQLARSQRVLTVPPDKSITDVLYEHGVPIETSCEAGICGAC

RTPVLDGTPDHRDDFLSAADKNDCIMPCVSRCKTERLVLDI

>Q0B329|Q0B329_BURCM

--MIENDLLAARVIARERVACDVISLRLVDAPGLALPAFEAGAHIDLHLRDGLTRKYSLCNDPVERGVYE

IAIKREPASSGGSAHVHDAIRVGDVLRIGAPLNYFPLAPDDSPAVLLAAGIGVTPLLAMAHSLVHAG-RS

LAFHYFVRSADAAAYGATLAS-RLADVATVHTGLTPDATRDAITTIVGAMDSRSHLYFCGPAPFMAAVDA

IA-ALGDARLHHEYFSAPA----AQPAFRIELARSQRALLVPPGQSITDVLYEHGIAVATSCEAGVCGAC

RTTVLEGTPDHRDAFLSAAEKNDCMMPCVSRCRGERLVLDL

>C7JED3|C7JED3_ACEP3

-MTRQSHLLNVIVDA-CQLRGSVLCFDLKASNGADLPVWEAGAHIDLYLEEGLIRQYSLCGNPADRKRYS

LAVLLDPKSRGGSIAVHRLVRDGAQLQISLPRNLFPLDWDAQSSLLVGGGIGVTPMIAMAYELYDKA-QN

FALHYVARDP---VFAPVLQALPFADHVVIHDHSQAERPRFNVQNTLAQNGSGVHVYVCGPEGLMQAVAQ

AGRGLPEEHVHQEAFSAQP----VEGGFEVLAAKSGVRVQVAEDETIAAAFARSGVRVPVSCEQGICGTC

VVSVLEGEPDHRDEYLTDEEKTDQIALCCSRSKTPLLVVDL

>A3V8N6|A3V8N6_9RHOB

RIDAASPWLDVTIATRTAAATDIISLELHSPDGSPLPAYDAGAHVDVYVKSGLIRQYSLTGDPADRMKYR

LGILLDPNSRGGSAAIHAKFQQGAKIRIGRPRNSFPLIADAGHTILFAGGIGITPMLGMAYALERSG-AS

WEMHYCGRTLDRLAFRNELAR--FGGKVHLHTDDGTKDQQLDINAVLSNAAHDHHLYVCGPNGFMDFIVR

SADGWTKDTIHLEHFGAEV----NTDPFTVVAKKSGKTFVVQPGETIAHKLAENSIAVQVSCQSGVCGTC

LTRVLEGMPDHRDMVQTDLEKNAQITVCCSRSKTKTLVLDV

>Q13HX8|Q13HX8_BURXL

DITAAPGELEVVVRVRTEEADGIASFELRTADGSALPSFDAGAHIDVHLP-GMIRQYSLCNPPGERHRYV

IAVLREPSGRGGSARMH-DLKVGDLLIISHPRNAFPLDAENGTKLLVAGGIGITPLKAMAHALTLRG-ID

FELHYFARSVSRAAFRDALATEPFAAAVRFHFDGDG-A-SLPLAETL-APERIAHIYLCGPTGFIDKVRE

QAIGFAAERIHVEHFSA-E----NNASFVVEAVRSGATVTVGENESIARALERAGVQVLTSCEQGLCGAC

LTPVLEGIPEHRDEYQTAAEKNAKITICCSRARTPVLRLDI

>D3P185|D3P185_AZOS1

SGDTQSQSRILTVIERIEEPGDILRVKLAAPDGAALPAFTAGAHIDIRLVDGLWRQYSLCSDPAGSDHYE

IGVLRDPNSRGGSVALHRLAKLGAAFTVEGPRNHFPLTEDAGRSILFGGGIGITPMLAMAQRLHALG-RD

FTLHYCTRRADATAFRALIAETPWRERVVFHHDDQPAAQRLDLGRDLPAPDGGTHLYVCGPQGFMDWVID

TAKGHAPANIHREYFSAQV----DSSSFEVTASRSGVTVSVGGDDTIAKALARAGISIPVKCEEGVCGTC

VTDVIDGTPDHRDQFLTDEERGTMICVCCSRAKSKSLVLDI

>A3K799|A3K799_9RHOB

MSLDMTDTMTLTVTERIDDRGGIARIRLT---GDDLPSVTAGAHLDIYLPD-LWRQYSLCSDPAETGFYE

IGVLKDPASRGGSVEVHRVAQPGATLKVEGPRNHFPLDESATKTVLLGGGIGITPMIAMAKRLHALG-KD

FVLHYCTRSQSVTAFHDALKAAPFSDRVVFHHDDEAAEQKLDLTRDLPAPAPDTHLYVCGPQGFMDWVID

TAEGHATGNVHREYFSADV----DLTSFEVEARASGVTLTIGPEDTIAKKLAEHGIKIDVKCEEGVCGTC

VTDVLSGDIDHRDQFLTDEERGDQMCVCCSRGRGK-LVLDI

>C6RKG9|C6RKG9_ACIRA

----MTALYDVVVKHRYIEGGDIAVMEFESATAQPLPKVEAGAHIDVHLPNGMIRQYSLCQDPAKKGVFR

LGILKDPASRGGSVSAFDDIQNGMQIQVSEPRNLFPLV-QAKHTVLIGGGIGITPLITMAYELLHQG-AL

FELHYCGSSPERCAFVNEIRNGELAAFTRFHFKSEGASHREFFQSAIKDLDRQSHIYTCGPSGFMDWVIN

LAQNFPEQQIHKEYFQVDT----DTSAFEVMAQRSGKIVLVNAEETILQALAREGVEIEMSCEQGVCGTC

MCDVIEGEPDHRDVYFTDEEKNEQILVCCSRSKSARLVLDI

>D0RYE0|D0RYE0_ACICA

----MTTLYDVVVKNRHVEGGNIAVMEFESATSATLPKVEAGAHIDVHLPNGMVRQYSLCQNPNDEGKFR

LGILRDPDSRGGSVSAFDEIKDGMQIQVSEPKNLFPLL-KAKHSVLIGGGIGITPLITMAYQLASEG-TS

FELHYCGASPENCAFVDEIKNGELAKYTTFHFKSEGASHRAFFESAIKDIDLESHIYTCGPVGFMDWVIN

LATNFPEQQIHKEYFQVET----DTSSFEVVAERSGKIIMVEAGETILQALAKEGIEIEMSCEQGVCGTC

MCDVIEGEPDHRDVYFTDEEKNEQILVCCSRSKTPRLVLDI

>F3KUQ9|F3KUQ9_9BURK

--MTSAQTLRVRVAKRQASGQDIALFTLVSADGAQLPPFEAGAHIDVHLP-GIIRQYSLCGNPADRSHYR

IGVLRDANSRGGSVAVHAHLKEGVELSISAPRNHFPLITGAGKAYLFGGGIGVTPMIAMAHTLHKTG-MD

FEFHYSSRSPSHMAFSEELGAAPYAARVHKHFDENAPQQRADAAAILGAAPPGAHVYVCGPKGYMDWVMA

TARGFPAERIHYEFFQVEV----KKGSFTVVAQASGKEVVVEAEDTIANALERIGIRVQVSCEQGICGTC

LTNVLEGTPDHRDEYQTDEEKNEQITICCSRSLTPRLVLDL

>A6VWC4|A6VWC4_MARMS

ESWMSETLLKVVVRKRENHTDGVMALELASLNGEALPVFEAGAHVDVHLSDDLIRQYSLCSDPADTSAYR

LGILKDPNSRGGSIAAHINLQEGTELTIGVPRNHFPLEKSAKRSILIGGGIGITPMIAMAYSLEAEG-KA

FELHYCSRSRETSGFLDELATSSFASHVDLHFDSED--QRLDLETVLKDADADTHLYVCGPNGFMDWVIS

TAKGMADSNVHKEFFNVEV----KTGSFEVVAEQSGVTVQVGENESIADALKAAGVKVKVSCEQGTCGTC

LCDVIEGTPDHRDVYLTEEEKNDQITLCCSRSLSPRLVLDI

>F2JV69|F2JV69_MARM1

---MSDELLKVVVQKREMHTDTVVALELADLEGNALPVFDAGAHIDLHLSDELIRQYSLCGDPANDKVYR

LGVLKDPNSRGGSIAVHENLLEGKELVVSKPRNLFPLDEGAQHSILIGGGIGITPMIAMAYELTHAG-RS

FELWYCSRSPETSGFLDELKSAPFADKVNLHFDSEQGGKPLNLDSVLQDKQSNSHVYVCGPTGFMEWVID

TSLGFADEAIHREFFTVDV----EKGSFEVYAEQSDITVTVKEDESVADALKAAGVKVQVSCEQGTCGTC

LCDVLEGTPEHRDVYLTDDEKNDQMTLCCSRSLSDRLVLDI

>E2XS15|E2XS15_PSEFL

----MNELLNVVVLKRVLQGDDVVVIELARADRGNLPTFEAGAHIDVHLPSSVIRQYSLCGDPADAGIYR

LGVLRDPQSRGGSIAVHQELLEGVKVNISLPRNHFPLDLNAERSILLGGGIGITPMLAMAYALEAKG-KA

FEMHYCARSRSRSAFLEELEGSVFSQAVQLHFDDE-PESKLDLKSVLGGSQETAHIYTCGPGGFMEWVIN

NALGYPDAHIHREYFQADI----DTNGFEVVAARSGKIVNVMTGQTIYQALAEVGIDIEISCEQGVCGTC

LCDVLEGEPDHRDVYLTADEKNDTILVCCSRSKSPRLVLDI

>F3FFB2|F3FFB2_PSESX

----MNELLNVVVLKRVLQGDDVVVIELARADRGNLPLFEAGAHIDVHLPSGVIRQYSLCGDPSDGGSYR

LGVLRDPQSRGGSIAVHKELLEGVTVDISLPRNHFPLDLNAEHSILLGGGIGITPMLAMAYALEAKG-KA

FEMHYCARSRSRSAFLEELERTGFSHAVQLHFDDEPE-SKLDINRVLADSQKTAHIYTCGPGGFMEWVIN

NALGYPDAHIHREYFQADI----DTKGFEVVAARSGKTVNVMNGQTIYQALAEVGIDIEISCEQGVCGTC

LCDVLEGEPDHRDVYLTVDEKNDTILVCCSRSKSPRLVLDI

>F4DZD5|F4DZD5_PSEMN

---MTEQVLNLVVCKRQEQGEGVVILDLADPTGSNLPTFEAGAHVDIHLP-GLVRQYSLCGDPANASVYR

LGVLRDPASRGGSVAVHELLTEGASVSVGTPRNLFPLAAGASRSILIGGGIGITPMIAMAHQLTAQD-SP

FELHYCGRSRQRTAFLDELAGADFAACVRTHFDDEGDAQKLDLPAVLGQPGAGVHVYVCGPSGFMDWVID

GARGYAEEHIHREYFQVEV----DASSFEVVAQRSGKTVQVAEGQTIIDALATVGIKVEISCEQGVCGTC

LCDVLEGEPDHRDVYLTDDEKNDQILVCCSRAKSKKLVLDI

>E2XPD6|E2XPD6_PSEFL

---MTEELLDVVVRKRELQGDGVVVLDLTRRDGAPLPGFDAGAHVDIHIP-GLVRQYSLCSDPADSSLYR

LGVLKDPASRGGSVGVHETLLEGREVRISTPRNLFPLAAQAQRSLLLGGGIGITPMIAMAYALYAAG-KS

FELHYCGRERGRSAFLAELASAPFAPHVFTHFDDEGPEQKLDLLKVLGRGEAGVHMYACGPAGFMDWVIQ

GARGYSESHIHKEYFQVDV----DSSSFEVVAARSGKTVQVAQGQSILAALAQVGIKIEISCEQGVCGTC

LCYVLDGEPDHRDVYLTDDEKNDQILVCCSRAKSKKLVLDI

>B0FXI5|B0FXI5_PSEPU

---MNEELLNVIVRKREIQGADVVVLDLGRADGAALPAFEAGAHVDIHVP-GLVRQYSLCSDPAEVSVYR

LGVLKDPASRGGSVQVHEALHEGREVQISAPRNLFPLAKDGKRSILLGGGIGITPMIAMAHALYRQG-AD

FELHYCGRSRSRSAFLDELAKAPFAAQVVTHFDDEDAAQRLDLPSVLGKADAGVHLYTCGPSGFMDWVIA

GARGFEEAQIHKEYFQVEV----DVSSFEVVAARSNKTVQVAEGQSILDALAQVGIKIDISCEQGVCGTC

MCEVLEGEPDHRDVYLTDEEKNDQILVCCSRAKTNKLVLDI

>B0KNJ0|B0KNJ0_PSEPG

---MNEELLNVVVRKREIQGADVVVLDLGRADGAALPAFEAGAHVDIHVP-GLVRQYSLCSDPADVTVYR

LGVLKDPASRGGSVGVHEALMEGREVQISAPRNLFPLAAGAARSILLGGGIGITPMIAMAHALHQQG-AD

FELHYCGRSRSRSAFLEALADAPFAARVVTHFDDEEATQRLNLADVLGAGMPGTHLYTCGPSGFMDWVIA

GARGYAEEHIHKEYFQVEV----DATGFEVVAARSNKTVQVAEGQTILDALAQVGIRIDISCEQGVCGTC

MCEVLEGEPDHRDVYLTDEEKNDQILVCCSRAKSNKLVLDI

>F8CMB1|F8CMB1_MYXFH

----MNDILRVRVARITREAEDILSYELVAAEGSPLPPFEAGAHLEVHVP-GFKRSYSLCNDPEEAHRYV

IAVARDARGRGGSRAMHERVHEGDVLEVRAPRNDFPLLF-ARGYVLVAGGIGITPILSMARMLQRTG-AE

YTLYYCARAPGRAAFHELLSQPPFAERVRFSFDGGDPSQGLDVKALLATRMPGARLYCCGPSGLMKAVRD

AATRWPWEKVHFESF-TAEGTSAEEQGFEVTIRSTGQVLQVPAGQSVLNVLRRNGVRIPSDCEAGTCGTC

VTRVCDGQPDHRDTFF-QTEPDQRMLVCVSRARSKRLVLDL

>Q1CWA5|Q1CWA5_MYXXD

----MNDILRVRVARVTREAEDILSYELVATEGGPLPPFEAGAHLEVRVP-GFLRAYSLCNDPEETHHYV

IAVARDAKGRGGSNAMHERVHEGDVLEVKPPRNNFPLLF-ARSYVLVAGGIGITPILSMARVLQRTG-AD

YTLHYCARAPGRTAFHELLSQAPFAEHVRFSFDGGDPARGLDVKGLLATRQPGARLYCCGPTGLMKAVRD

AATRWPWEKVHFESF-TAEGTSAEEQGFEVTIRSTGQVLQVPVGQSVLNVLRRNGVRIPSDCEAGTCGTC

VTRVCDGQPDHRDTFF-QAEPDQRMLVCVSRARSKRLVLDL

>A1WML5|A1WML5_VEREI

--MNPQRRLTVRVARIWRQTPHILAFELTHPWGRPLPGYEAGAHIDVHMPGGFSRQYSLAAPPAAAPSYL

IGVKREAASRGGSASMHERVRTGDLLAVSAPRNTFPLCPQAAQHLLLAGGIGITPLLAMAQALAARG-AN

FRLCVFARSREHLAFADALRAPALAPRLRLHLDQGEAAERIDLHALLAERAPGAQLYLCGPGGFMQAVRD

AARHWPEDALHAEYF-AAPADADAGQPFTLRLAQRGISVPVAADQSAVDALRQVGIDIPVSCQQGLCGSC

VVPG-DGAGAHHDFCLTASERQTRLALCCARAKGQELVLQL

>C5CR64|C5CR64_VARPS

--MSLERTLTVRVERISRETPEILAFELAHPWGRTLPGYEAGAHIDVHMPGGFSRQYSLANAPSAAASYV

IGVKRE-QSRGGSASMHERVREGDLLAISVPRNTFALREEAGHHLLMAGGIGMTPLLAMAQALAARG-AS

FTLCVFARSEEHLAFADALHSSALAPHLRLHLDQGGASQRIDLRALLAERAPNTHLYVCGPGGFMRAVRE

AAAHWPEDTLHTEYFAAPT----DANPFTLKLAQRGISVPVAADQTAVDALHEVGIDIPVSCQQGLCGTC

VVEG-DGEGAHRDFCLTGSERRHKVALCCSRARGRELVLQL

>E6UXL9|E6UXL9_VARPE

--MSLERTLTVRVERISRETPEILAFELAHPWGRALPGYEAGAHIDVHMPGGFSRQYSLARAPSNAPSYV

IGVKRELASRGGSASMHERVREGDLIAISTPRNTFPLREEAAHHLLMAGGIGMTPLLAMAQALAARG-AS

FTLCVFARSEEHLAFSDALRDPALAPHLRLHLDQGDASQRIDLQALLADRAPDTHLYVCGPGGFMKAVRD

AAAHWPEDALHTEYF-AAPTDANTGLPFTLKLAQRGITVPVAADQTAVDALHEVGIDIPVSCQQGLCGTC

VVEG-DGEAAHRDFCLTGTERRSKVALCCSRAKGLELVLQL

>Q15XJ1|Q15XJ1_PSEA6

-------MFDVVIKNKTLETPHICSFELARKDGGELPAFSAGSHVDVQLPNSLTRQYSLISHAQSQAFYK

IAVLKDANSRGGSLALHDTLNVGDEIAISEPRNLFPINAKSDKVLLFAGGIGITPILSMAIELDGIG-VD

FELHYRAKSKEQAAFYQLLLNCPFASKVSYYFSDQPENNNTRLNQALSHFTENTHLYTCGPVGYMEHIFD

VARSWKEENLHKENF-KAEPKIAGDKPFKLILKRSGLEIDVAVEQTALEAIEDAGVTVDMSCEMGICGAC

LTPVIDGVPDHRDEFLSADEKNNQFTPCCSRSLTDTLIIDL

>Q1LBV0|Q1LBV0_RALME

NHAGDAPFLELRLSERRKEADGILSFELVHPDGEQLPAFDAGAHILVDIP-GVSRAYSLCNDPAERHRYV

IAVLRT-DSRGGSVGMHQAIEPGQLVRVSAPRNEFELVQSAGRSLLFAGGIGITPLLSMAETLANAD-RE

FELHYCTRDAAKTAFTSRIET-RFSKQARIYHDLASKEQPFDARKVLKQGGSDDHVYACGPSGFIEHILS

TAAGWEKRQLHREFFGSPT----TQNAFDLILASSGKKVHVPCGVSAATALLEAGISLSMSCEQGICGTC

VTTVLNGMPDHRDHYLTDDDRNDCFMPCCSRSLTAELLVDL

>A6SYL6|A6SYL6_JANMA

-----MTTLTVRVHKITQEACDIKSFELRSVSGEALPAFTPGSHIDVHIGEGLIRQYSLCNGPDEPDRYL

IAVKKEVTSRGGSQAMHESIREGDQIKISAPRNNFPLEQSQGPYLLLAGGIGVTPVLSMARHLLAQG-VE

FELQYFTRSIAHTAFHALLSEPEFQGRIKFHYALEPEGLRAYLRKLLWHYPENGSLYLCGPRVFMDLVES

TA-TWPPEAVHLEYF-SADPLSLEQESFEVRLARTGGTYAVPAGMPITEALAAHGIHIDTSCEQGVCGTC

LTGVLEGTPDHRDVYLSDAEKCDKIMPCVSRAKSPLLVLDL

>G0ERV6|G0ERV6_CUPNE

-----MDTIRTVIRRKYPVAEDIIALELVAEHGGALPAFAAGAHIDLHLPGGLIRQYSLCNGPDEAGCYL

LGIKREPGSRGGSLAVHDHLREGDVLTIGMPRNHFALAE-GGPSLLFGAGIGITPLLAMVLQLRAAR-RP

FTLHYFARSPAHVAFHERLVHASQAGQVHYHFGLSPEQTHDALRGAIAQATPATHVYSCGPSAFMDGVMQ

LA-VLPDTQIHYEYF-QAAVAGQADAPFEVVAARRGVHCMVPPGMSIVQALYAQGIEIEVSCEQGVCGTC

MARVLEGTPDHRDVYLTEAEKGDVVMPCCSRALTARLVLDV

>D9WKX2|D9WKX2_9ACTO

---MSTTWTDAVVVARYDATPRIAVFDLARADGGELWEYESGAHIDVQVEAGLVRQYSLCGPPGVREQYR

LAVLNEPASRGGSQAMHT-LAEGDTLRIGAPRNRFGLTP-ARHHLLLAGGIGITPLLSMAQTLDATG-GD

YHLHYCARSRTDAAFVPELEHH---PRATLHFDDQEPEQLLDLGRDLGEPAPDTAAYVCGPGGFMDYALG

RAAGWPASALHTERF-SASTAATGEGGFTVRLSSTGAEYLVPEDQSVLDVLLANGVEAPSSCGQGICGEC

VVRVRVGEPDHRDDVLTDDEREGLFTPCSSRSRSPILELEL

>Q39LN8|Q39LN8_BURS3

----MPATFAVKVTRKWNETPDICVLELHVREDQKLPAFEAGAHVEVHLR-GPSRQYSLCNTPGPTDCYQ

LGIFLEPESRGVSRAIHQTVEVGHTLDISPPKNHFPLAEGATHSLLLAGGIGVTPVLAMAEALASRS-AS

FDFHYCTRSAERMAFQTRIGQSGWRQNAHLYFDDAPDDRRLDLNAVLSSPAAGTHLYVCGPSGFVTWVKQ

AATGWPDTHVHSESFTAVE----VQDAFDVEIAETGQIIHVGKDQTALNALVEAGIDIPSSCEAGNCGTC

QTMVVSGAIDHRDQYLTAAERNKSFIPCCSRAADDMIVIGL

>Q13GB7|Q13GB7_BURXL

-----MQMFKVVVNRVDELAAGIRSYELVAEPHATLPPFEAGSHIDVITPAGHTRQYSLCNDEQERHRYV

IAVKREPQSRGGSASMHDTLTAGTTLTIGYPRNNFELDAHEPAVILLAAGIGITPLLAMAFRLGTQG-RT

FALHYFVRGDEHIAFRDILLSPRFSRWVELHRGLDAAATRATLAKILAAPPAGAGVYLCGPLPFIEAGQQ

CMAGKSGCNLHVEYF-AAPPQAPGDQPFEVILARTGRTLVVPPGKSIVDTLADAGIHADVSCEQGVCGTC

ITPVLGGVPDHRDVYLTDEEKGKCMMICVSRARGARLELDL

>Q88LH5|Q88LH5_PSEPK

----MQNWLELEISRREKATEAISVFELRHPNGEKLPAFTAGAHIDVKVRDGLVRQYSLSNDPVETDRYV

IAVLNEPTSRGGSRAIHEQFLQGMKISVGEPRNHFPLLDADDHFVLVAGGIGVTPILAMARYLNRIG-RS

FEIHYCIRSRSTGAFLDVFQGPEFAGRVTLHVDDDPESTPLELARVLDKERA--RLYVCGPGGFMNWVLS

TA-CLSPERVHKESF-SAEPIAATDDGFEVEVASTGQVFFIPTERSITEVLEEAGVEVLVSCQQGICGSC

ITNVLEGEPDHRDSVLSESERGKVFTPCCSRSKSPRLVLDL

>Q39LC3|Q39LC3_BURS3

HQSGALDRQVLIVRDVADEANGIRSYELATEDGADLPAYEPGAHVAVHLENGITRQYSLYEAAGLRKTYR

IAVLKDPASRGGSRFLHEQVSVGERVRISGPYNHFPMTAGATSSLLIAGGIGITPILSMAARLFSEG-QP

FALHYCARREQDAAFVDLLRRGPFAAHVHLHFDGGDPGKGLDVRSLLSDVTEGRHLYCCGPGGLMNAVEA

AASHWPAGTVHFERF-AAETADAENTAFRIHLCKSGLDLDVPADKSVLQVLKHAGFDISTVCEQGVCGAC

LTDVVDGVPEHRDQILTDEEKNDVMAVCCSRSRSPRLVLDL

>Q13GV4|Q13GV4_BURXL

----MNPLIAVRVARKRIEAADIASLILESADGAPLPPAGPGAHIDVHTPSGCVRQYSLCNLADEHGHYR

IAVLRDSASRGGSISMHDDVLEGGKLSISAPKNHFPLREDARHSILIAGGIGITPVIAMASALHARG-AS

FALHYRTRSAARTAFLRELTHGALAPHCALYHDDAPALRKLELMPLLEAAPPDTHVYVCGPKGFMDAVIS

TARHWSEDRIHFEYF-AGVATPGNANSFEVVLAKSGQVVQVAPSQTIVEVCSAHGVAIPTSCLHGICGTC

ITRVLEGEVEHKDFYLTPQERHTQMLPCCSRAKSHRLVLDL

>O24840|VANB_ACIAD

----MINM-DVIIHKIHQLTPSIRAFELISANGSDLPAFDAGSHIDVHLKNGLTRQYSLSNCCSEQHRYV

IGVLHD-NSRGGSRCIHQDYREGDHLNIGTPRNLFEIHSKTQKAVLFAGGIGITPILSMAYRLKHQQ-IP

FELHYFVRSHEMIAFYGNLTE-HFPEQIHFHIQNQSETQ-CEMSKVLEEVAPDRHLYVCGPAGFMQFVMD

SAQGWSDEQLHQEHF-VAPQIDQQNEAFTIEVLGSDRKIEVSAHQTATQALLEHGFDVPVSCEQGICGTC

ITRVVSGTPDHRDVFMTDEEHNDQFTPCCSRAKSKILVIDL

>C0VH44|C0VH44_9GAMM

-----MNM-DVMIHKIHQLTPSIRAFELVSANGTPLPTFEAGAHIDVHLKNGLTRQYSLSNSCSERHRYV

IGVLHDSNSRGGSRCIHTKYHEGDLLQIGEPRNLFKIHPETKQAILFAGGIGITPILSMAYRLKSAR-IP

FELHYFVRSHDMIAFYGNLTE-HFGSNIHFHIQDQP-GTGCDMSQVLQQNAPDKHLYVCGPTGFMEFVTN

SAVGWQTEHLHQEHF-VAQLADTNNEAFTIEVKGTDRQIKVLAHQTATQALIEHGFDVPISCEQGICGTC

ITRVIEGTPDHRDVFMTDEEHNNQFTPCCSRAKSKKLVIEL

>D0RZS9|D0RZS9_ACICA

-----MNM-DVIIQKIHQLTPTIRAFELVAANGTELPNFEAGAHIDVHLKNGLTRQYSLSNCCSEKHRYV

IGVLHDANSRGGSRCIHTEYREGDHLKISAPRNLFEIHSQTKQAVLFAGGIGITPILSMAYRLKSAN-VP

FELHYFVRSHEMIAFYGNLTE-HFGDQVHFHIQDQPDTE-CNIAEVLGKSSPDRHLYVCGPTGFMQFVMS

SAEGWHNEQLHQEHF-VAQKVDTNDEAFTIEVKGTGRRIEVLPEQTATEALIANGFDIPVSCEQGICGTC

ITRVVEGTPDHRDMFMTEDEHNDQFTPCCSRAKTKNLVIEL

>Q3LFL8|Q3LFL8_9BURK

-----MTTLTVRVLGRREEARDIASFRLARVDGGNLPPFSPDAHIDVHLPGGHVRQYSLCNGPDERDDYR

IAVLRAPDSRGGSVAMRERIPVGDIVTISEPRNTFPLKH-RQRSVLLAGGIGITPLLSMAKHLAAID-LD

FSLHYCPRSPARTAFCEELGASQFAHRVHYHFDDGTPEQKFSLDAAIGVPGPGVVIFVCGPKGFIEHVHR

GAEGFTPDQVHAEHFGAQP----HAAPLTIQIASTGQQIGVAADRTAVQALAAAEIDIMTSCEQGFCGTC

ITRVLAGKCDHRDICLTGEEKNDAFTPCCSRASTPLLVLDL

>Q5GDA2|Q5GDA2_9BURK

-------MLKVKITSKALEARDIVSLELLSLDGAELPAFEPGAHIDLHVP-GVVRQYSLCGSPLLRHGYR

VAVLRDPASRGGSAAVHDVLNEGDIVTIGMPTNLFPLAV-SPHSLLFAGGIGITPILSMAQRLSRDC-AS

FELHYCARSRERMAFLQEIGNSLAPDQLHLHLDDGSPEQRLDVQDILQRSAPGSHLYVCGPAGYISHVTD

TARGWDEARIHFEHF-GNAAAAHGDRPFRICIASSGRIVEVGAQEPATVALARAGLHVPVACEQGICGTC

LTRIVEGVPEHRDMYLTEREKNDQFLPCCSRAAGDLLVVDL

>A7KS55|A7KS55_9BURK

RNGMDQSWMDVRVASLNVEADDIVSLELMPSDGEPLPSFSAGSHIDLKLPNGLIRQYSLCNDSSEKNRYQ

IAVLLDPATRGGSASVHRDIKVNQIINISKPRQNFPLID-ARYSILIAGGIGVTPLLSMAKRLDRTD-AS

FEMHYCTRSSSRTAFTQHIRTASFADRVHFHYDDGDQTQKFDIDSVFVERNSDAHIYVCGPSGFMDFVIN

SAKGWPNEQIHFEYF-SREVIANDHTQFKVEVASSGQRFDIPADRSIVSVLEENGIEIPVSCEQGICGTC

VTRVLEGVPDHRDTFFSDAEKNDQITPCCSRARTPLLILDL

>A5VBE5|A5VBE5_SPHWW

---MSGDWISVRLAGKRLVAEDIAALLLEPADGAALPPFAAGAHVDVELPGGLVRQYSLCNAPGDPGRYE

LGVLLDRQGRGGSRAVHERLAAGQAIRISRPRNLFPLVPAA-HSVLMAGGIGITPILAMAEQLAADR-AS

FELHYFVRSPERAAFVDRLAAPRFADRCRLHVGDRIPP-SFDAPALFDAPRAGHHLYVCGPNAFMDAVLD

GARGWPDDRLHSERFAAAP----LPAPFEIEVEGHGAIVAVAAGQSAAEALAAAGIRIPLSCEQGICGTC

LTTVVGGVPDHRDSYLSDAERNDCFTPCCSRAATPRLVIQL

>B2JSG5|B2JSG5_BURP8

QTTSVHTLLQVKVAAKVWLADGIAGYELSPVSGDALPAFEAGAHIDLHVPGGPVRQYSLYELSGEDGRYR

IGVLRDPNSRGGSVKLLDDVQAGDTLTISAPRNHFALHSANDRSILFAGGIGITPIFCMAQQLAGEQ-RP

FELHYCGRTLSGMAFVDRLQQAQSGSNIHVHVDDGAPDQQLDARSAIGAPSADRHLYVCGPAGFMDHILA

TARGWDEKHLHREYF-AAAPVESDDAPFEIEIASSGKVITVSTAQSAAQALLEAGFDVPLSCEQGVCGTC

MTKVLAGVPEHRDLYLTDDERNDAFMPCCSRSKTSRLTLDL

>D5VDI3|D5VDI3_CAUST

-------MIKVRVASRVPVAEDIIGLDLVHADGEALPPFSAGAHVDLFLGNGMTRQYSLCNDPADQSRYR

IAVLREPSSRGGSAFVHDALLKGAVLTISPPRNLFALDEDGHEHRLFAGGVGVTPILAMAYRLHALG-AR

FILHYCARSRSRAAFLEELAAAPFAASVRLSFDDEPDT-RLDLDAVLAAPSPDRRIYVCGPGGFMAFVTE

GAAGWTSGQIRREHF-AAEVAPTQNRPFDLVIASSGQVVGVAADQTAAQALEAAGVFVPLSCEQGVCGTC

LTPVIEGEVEHRDAFQTDAEKGLAFTPCCSRA-GPRVVAKL

>Q0B7J2|Q0B7J2_BURCM

--MLHGLKMKVLVKSKKLVANGIAAFKLVAPGGEQLPLFSAGAHIDVFL--GLVRQYSLCNAPSDRSHYQ

IGVLLEPNSRGGSRAMHN-LAEGQLIEISEPKNHFPLTAGARRSVLIAGGIGITPILAMAEHLREER-QD

FDLHYCVRDHERAAFRERVAHDDFAPHARLYYDTAPSRERVKFSEVLRSPDRDVHLYVCGPGGFIDAVIK

EATGWGSENVHREYFNAPT----SGEPFQLRLARSGKIVEVRSSQTAAQALAAHGIDIQTSCEQGVCGTC

MTKVLEGVPDHRDVYMTDEEHNDQFTPCCSRAKTP-LLIDL

>F8JFA6|F8JFA6_HYPSM

---MQNATLTVKVKSKKNEAMNIASFELTSLDGSDLPPFTAGAHIDVHLP-GLVRQYSLCNSPSERHRYV

IGVLRAPNSRGGSEFLHDIIRNGSRLSISMPRNNFPLAPTAKGSILFAGGIGITPILCMAEHLSNEG-SP

FELHYSARTPDRLAFSADILASPYADRTIFHFDDAAPEQALNPSEILRMPDAETHIYVCGPSGFIDHIVH

AALGWPNDQIHFEHF-AARGSHANATSFTVTLARSGTIIMVGENETVVQALSRNGVEIPVACEQGLCGTC

MTGILGGVPDHRDHLLTATERNACFLPCCSRALSANLILDL

>Q13J69|Q13J69_BURXL

--------MKVKIVARREVAVGIAAFELMDPRGALMPAFSAGAHIDVHVSNGLVRQYSLCNDSRERSRYM

ICVLHDPETRGGSRAMHE-MREGQTLEISEPRNHFPLASDSGHSVLLAGGIGVTPIVSMAFSLASEG-AS

FEIHYCARGPSHAAFLDRFAEPDLRDRTRLYFDTSPACDRLRMQDVLATPSTEKHLYVCGPKGFIDAAVT

TAMGWKTTNVHREHF-SADPMPSEAGSFTVRLAKNQRTVNVGPNERVIDALGKAGVFVPVSCEEGVCGTC

LTGVLAGEPDHRDSYLTKRERNDRFLPCCSRSKTPLLVLDL

>Q7WE66|Q7WE66_BORBR

----MNARLAVRIAGKHAGARDVCVLELESLDGEPLPAYAPGAHVDVHVGNGISRQYSLCRGPAAR--YQ

IAVLREPASRGGSQAVHDALDVGDVIEIGAPRNLFALADADGPSLLLAGGIGITPILCMAESLAARG-GR

FALHYCGRSRERMAFLDRLQAPGLAARAHIHTDDGPPAQRLDIAQVLARQEPGAHLYVCGPGGFIEAVIA

QARGWDDARIHYERFSNPP----GSDGFEVQLSSSGEVFAVGKDESVVQALARHGVFVETSCEQGICGTC

LLRVLEGEPDHRDMYLSDDEKNDQFLPCCSRARSKRLVLGL

>Q12EU3|Q12EU3_POLSJ

---MSANEIAVKVARKVREAQDINSYELVSVNGQALPAFEAGAHVDVHLPNGLVRQYSLCGMPHQKDRYR

IAVLRDPKSRGGSEAVHDLVHEGDTLRISAPRNLFRLAAGEQTSVLLAGGIGVTPLLAMAYHLHALG-SA

FALHYFARSRDRVAFLAELQNSGFARNVVLHLDDEAVAGARPLRSLLETLQRDAHIYTCGPTGFLNHVLE

TASDWPGGQVHYETFSPPQ----PTASFEVRISSTGQSVVVAADESVVSAVARLGIEIPVSCEQGICGTC

LTRVAEGVPDHHDQYLTNEEHNDQFTPCCSRARSKVLVLDL

>F5RHA9|F5RHA9_9RHOO

---MSAPDLPVVVTRHARVADDIVHIVLESPAGTPLPAWSAGAHIDVQLPNGAVRQYSLLGDAAHATRYE

LGVLKEAGGRGGSRSAHEDVRAGMQLRIGAPRNLFALQP-ARRTLLFAGGIGITPMLAMAQQLAHGQ-AD

FTLHYCSRTPSRTAFIDRLRDAPWADRVRLHFDDGDPRQKLDVAAVLAAPSPDARLYVCGPSGFMAHVID

SARGWADANIHSESF-SGGAAARGDEAFSVRLARSGRTVEVAAGQTVAAALAARGVDVALSCEQGICGSC

AVRVLDGEPDHRDCFFTEAEHGGCFTPCCSRSKSALLVLDL

>F8GVD0|F8GVD0_CUPNE

QDIHVATTLKVLVREKETVADGIVRFELRPTSGELLPSFTAGSHIDVTLPNGAVRQYSLCNAPDDNGRYE

IAVLLEPLGRGGSRSAHVDLHRGSVLQISPPRNLFPLSD-AGHSILIAGGIGITPILSMAHHLACGG-RS

FELHYCTRSVARTAFLERITSSEFADSVTIYHDDYTQQERFSARNTLASPTDDTHIYVCGPTGFMDHVIG

VALGWQTANVHREYF-VAPSADTGDRPFTVEFARTGQAVVVPPSQSVAQALAAHGIDIPVSCEQGICGTC

MMRVLEGEPDHRDTFLTTQEHNDRFTPCCSRSKTARLVIDF

>A2SEH6|A2SEH6_METPP

--------MEVTVAGRETAAEGVASFELRRADGGPLPGFDAGAHIDVELPNGMVRQYSLCNDPAERGHYR

IGVLLEAAGRGGSRSAHAQLLPGSRLRISAPRNHFALVP-ARHTLLLAGGIGVTPILSMAEQLARAD-AS

FELHYCSRSPARTAFVPRLRAAAFAAQVHFHFDDGDAAQRFDARAVLAQAGDTTHLYVCGPKGFMDHVIL

TARGWSEGNIHYEYF-AGAAVDSADRGFEVQIAGSGQVVPVAPGQSVVQALAAHGIDVPVSCEQGVCGTC

VMRVVQGEPEHRDMYFSAAEHNHCFTPCCSRSKSARLVIDF

>E3HNT5|E3HNT5_ACHXA

--MQSQVTSTVRVAHRRQETDDIISLELEALDGGALPPFEAGAHIDVHVP-GIVRQYSLCNASSERHCYV

IGILRDPQSRGGSVAIHERLQPGSVVEVSAPRNHFPLKPQA-KSVLIAGGIGITPMLCMAETLHAAG-TA

FSLHYCARDQGRAAFLDRIAQSPFSENVVFHYDDKADEQKLHPDELFSQVGPEEEIYVCGPAGFIDWVCQ

AADGVPKHRVRYEHF-SAKPVDTQDGAFDVRIASSGQLFHIPADQSVADVLLAAGVDIYTSCGEGSCGSC

VMRLVEGEADHRDVFLTDEEHGNQFTPCCSRAKSPLLVLDL

>F8GU82|F8GU82_CUPNE

--MNPQDTFFVRIAQRNQETDDIISLELRDPNGSVLPPFDAGAHIDVHVP-GVVRQYSLCNAPSERDSYQ

IGVLRDPASRGGSIAIHERLEAGELIEISAPRNHFPLKE-GVRSILIAGGIGVTPMLCMAESLHASG-AG

FTLHYCARSQDKAAFRSRIAQSPYSQQVAFHYDDQADAQKLDPATLFAQAGSDAEIYVCGPAGFIEWICR

AAEGIPKQRVHYEYF-SAKAVDTDDGAFDVKLASTGQVFQIPAERSITSVLLEAGIDIYTSCEEGTCGTC

VTRILEGEPDHRDVFLTDEEHGNQFTPCCSRAKSSLLVLDL

>A2WH31|A2WH31_9BURK

PHRRSVPM-KVTLIRKLPVAADICAFELAPADGTALPPFTAGAHIDVHV-GGFVRQYSLCNSPSETGIYR

LGVLREPASRGGSVALHA-CEPGATLDIGAPRNHFPLDPHAAHSVLLAGGIGITPLLSMALHLAETG-RS

FELHYCAREPARAAFVDRLDAPALKARTQLWFDDDPGGRRIDFAQVLAKPDPRTHLYVCGPAGFIGAVLA

AAAGWDPANVHREYFGAGA----DAGAFQVSLAQSGRVVDVPAGTTIVEALRGCGIEVPVSCEQGVCGTC

LTRVLSGTPDHRDVYLTDDERNDQMLPCCSRAITPMLVLDL

>B9BYI0|B9BYI0_9BURK

-------M-KVTLLHKLPVATDICMFELGPADGAALPPFTAGAHIDVHL-GGLVRQYSLCNSPSEAGVYR

LGVLRERESRGGSVAMHA-CEPGTTLEIGEPRNHFPLDPHAAHSVLLAGGIGITPLLSMALHLAETG-RS

FELHYCAREPARAAFVDRLDTPPLNARTRLWFDDAPAGQRIDFAQVLGKPHAHTHLYVCGPAGFIAAVLG

AAAGWDPANVHREYFGAAGAADEADGPFQVSLAQSGRVVDVPAGMTIVEALRGCRIEVPVSCEQGVCGTC

LTRVLSGTPDHRDVYLTDDERNDQMLPCCSRARTPMLVLDL

>A9IGA3|A9IGA3_BORPD

AFLPDPASLRVRVVRKEALTKDIVLIELASVDGQPLPTFTAGAHIDVQLPGGLTRQYSLCDTAAET--YQ

IAVLKEPQGRGGSVAMHDLVHAGSELVVGTPRNLFELSASAQSSLLLAGGIGITPLLCMAQSLATAR-RP

FSLHYCTRSAEATAFRGALAAAPYAAQVHHHYDDGTPEQKLDLPALLSCPQPGMHLYTCGPSGFMDAVLA

AARGWPESQLHYEFF-KAEPPQSNDGSFEVQVASTGEVIPVAADETVLMALAAHGIELPASCEQGVCGTC

LTRVLGGEPDHRDMYLTPEEQNDQFLPCCSRSKSARLVLDI

>Q13VP3|Q13VP3_BURXL

AFLPDPASLRVRVVRKEALTKDIVLIELASVDGQPLPTFTAGAHIDVQLPGGLTRQYSLC-DTAAET-YQ

IAVLKEPQGRGGSVAMHDLVHAGSELVVGTPRNLFELSASAQSSLLLAGGIGITPLLCMAQSLATAR-RP

FSLHYCTRSAEATAFRGALAAAPYAAQVHHHYDDGTPEQKLDLPALLSCPQPGMHLYTCGPSGFMDAVLA

AARGWPESQLHYEFFKAEPPQS-END-FEVQVASTGEVIPVAADETVLMALAAHGIELPASCEQGVCGTC

LTRVLGGEPDHRDMYLTPEEQNDQFLPCCSRSKSARLVLDI

>B5WQ71|B5WQ71_9BURK

-MNMTDSTLMVRVAARRGEADGIVGFEFVDVDGRDLPPFEAGAHIDVYVPGGPVRQYSLCNAPGERHRYQ

IAVLRDADSRGGSERMHDVVNEGDAIRIGVPRNHFPLAQHHAKPLLLAGGIGVTPILCMAEQLAAMG-AA

FDMHYCARSKSRAAFVERIAASSWVDNVQYHFDDEHGI--LDLNALLAGAGTDRHLYVCGPQGFMNAVLD

TARGWSDDRLHYEYFATQP----SGDSFDVRLARSGRVVSIPADCTVTQALAAAGVDVPVSCEQGICGTC

ITRVLEGEPDHRDLFLSPEEQNDQFLPCCSRAKSRVLVLDL

>D5WGK6|D5WGK6_BURSC

-MNMTDSTLMMRVAARRDEADGIAGFEFVDADGRELPPFEAGAHIDVYVPGGPVRQYSLCNAPHERHRYQ

IAVLRDANSRGGSQRMHDAVNEGDAIHIGVPRNHFPLARHDAKPLLLAGGIGVTPILCMAEQLAAKG-AA

FDMHYCARSKSRAAFVERIAASSWADNVQYHFDDEHGM--LDLNALLTG-GADRHLYVCGPQGFMNAVLD

TARGWSDDRLHYEYFAAQP----SGDSFDVRLARSGRVVSIAADCTVTQALAAAGVDVPVSCEQGICGTC

ITRVLDGEPDHRDLYLSPEEQNDQFLPCCSRAKSRVLVLDL

>C9YAY0|C9YAY0_9BURK

TPVSTPATLQVKVAAKRTEAQDICSLELVAADGGALPAFTAGAHIDVHLPNGLVRQYSLSNAPTETKRYV

IGVLRDAASRGGSTAVHDLVAEGSTLTISTPRNLFPLDSAAPHHLLLAGGIGITPMLAMAEHLAATG-GA

FTLHHCSRSRVRAAFVERLAAAPFAAHTHHHFDDGDAAQKLDIAATLQSAPAGTHLYVCGPQGFMDAVLS

AGRGWPEERLHREYF-GAAPTAKDDGSFELEIASTGKVIKVLPDQTALEALHAGGIDIPMSCEQGVCGTC

LTRVKAGMPDHRDQYLLPEEQNDQFLPCCSRSKSARLVLDL

>B7WT90|B7WT90_COMTE

--MKTETTFEVRIAHKQEQAAGICSLELRALEGASLAPFSAGSHIDVHLPGGLVRQYSLSNDPAELDRYV

IAVLREPASRGGSAAVHEQLQAGQQITISHPRNHFELHAPARKHLLLAGGIGITPILAMARKLAHDG-AE

FALHYCGRSRERMAFAQTIETAQWADKAQIHVDDVNGKSSLGLSELLQQREQGQHLYVCGPKGFMDAVLD

TARGWPAEQLHYEFF-AAEVEHRDDESFEVEVASSGQVVRVTPAQTVVQALESIGVCVQTSCEQGVCGTC

LTRVISGEPDHRDMYLTEDEQNDQFLPCCSRARSGRLVLDL

>A9BSJ5|A9BSJ5_DELAS

--MQANSILQVRVARKCAEAQDICSLELVPLDGQRLPAFTAGSHIDLHLPGGLVRQYSLCNDPQDTGRYV

VGVLREAASRGGSAAVHEQVQEGQQIAIGAPRNHFALHADGARHLLLAGGIGITPVLAMARHLAREG-AD

FELHYCARSRERMAFTALLQSMPWADRVHLHV-DGE-G-ALDMAQLLGAPQAGLHLYVCGPRGFMDAALG

HARGWSEDSLHYEFF-AGEAVQRGDAGFEIEIASTGQVVRVAPEQTVVQALAGIGVEVQTSCEQGVCGTC

LTRVLSGEPEHRDMYLTEDEQCDQFLPCCSRAKSARLVLDL

>E1V9F3|E1V9F3_HALED

-MSNSSLSLIVEVKARHEEAQDIVTFELADPHGRPLPAFSAGAHIDVQVQNTIIRQYSLCNHPEERDRYL

IGVLRDPASRGGSVAMHDEIRVGDLIQISAPKNHFPLEP-AERTLLLAGGIGITPLLCMAERLAHTD-TT

FELHYCTRSPERTAFRDRIAASGFAEKVHFHFDDGPAEQKLDLESLLATPAPDTRVYVCGPTGFIDAVMS

TCQGWPSDQLHTEYF-AGAVANTDDDSFEVKIASSGATLTVPADKTVHQVLTENGIDVMVSCEQGVCGTC

LTRVLEGEPDHRDFFLEDHEHNDQFTPCCSRAKSKVLVLDL

>Q1R0Q3|Q1R0Q3_CHRSD

MNDSTLSLF-VRVTQKRQEAEGIVSYELSDPHGRPLPAFSAGAHIDVRVKEGVIRQYSLCGHPEERDRYF

IAVLREPSSRGGSVAMHDEIEEGDLLQISAPKNHFPLEP-AKRSVLFAGGIGVTPILCMAERLSHTQ-AQ

FEMHYCARSRERMAFVDRLAQSAYADRVHFYYDDAPEAGKLDVAAVAGEPNDETHIYVCGPSGYIDHVMS

TCKGWPSSQLHTEYF-SGVETTSDDESFEVKIASSGATFSVPKDKSVYEVLSENGIDIMVSCEQGVCGTC

LTRVLEGEPDHRDLYLDDDERNDQFTPCCSRAKSKTLVLDL

>B5WFZ8|B5WFZ8_9BURK

---MSHPVLNVVVARRRDEALGIASFELASENGQPLPAFEAGSHVDVHLPNGLVRQYSLCNDPRETHRYL

IAVLRD-AGRGGSKAVHDLVREGDRLQISAPRNHFALADDAAHHLLLAGGIGVTPILCMAERLAASG-AS

FEMHYCTRSKDRTAFVERLAQAGFAKSVQLHHDDHPGGSTFDIEQALRDAPDGTHLYVCGPRGFMDFVLE

TARGWPEHRLHYEFFGAPA----NQASFRVRIASSGATIDVPPECTVVQALAAHGVEVITSCEQGVCGTC

LTRVLSGEPDHLDSYLTDEERNDQFLPCCSRAKSPLLVLDL

>D5WF29|D5WF29_BURSC

---MNQPVLNVVVARRRDEALGIASFELASENGQALPAFDAGSHVDVHLPNGLVRQYSLCNDPRETHRYL

IAVLRD-AGRGGSKAVHDLLREGDRLQISAPRNHFALADDAAHHLLLAGGIGVTPILCMAERLAATG-AS

FDMHYCTRSKQRTAFVERLAQAGFANSVQLHHDDHSGGSTFDIEQTLSDAPAGTHLYVCGPRGFMDLVLE

TARGWAENRLHYEFFGAAA----NQPSFQVRIASSGATIDVPPECTVVQALAAHGVEVITSCEQGVCGTC

LTRILSGEPDHQDSYLTDEERNDQFLPCCSRAKSPLLVLDL

>C5AIT3|C5AIT3_BURGB

--MSEATLI-VEVVRKWDEARGICGFELRRPDRAPLPGFSAGAHIDVHLPGGIVRQYSLCSSPARNDRYE

IAVLRD-RGRGGSMAIHDHVHEGDQIRIGAPRNHFPLELRAARHLLLAGGIGVTPLLSMAEHLHALG-AS

FEMHYCTRSRDRTAFAQRLAGSAYHDHVQIHFDDAGEHQAFELDQVLAAAAADTHLYVCGPRGFMEAALA

AARGWPDERLHYEFFAVAD----SAEAFQVRVASSGAVIDVPAGCSIVDALAKHGVEVLTSCEQGVCGTC

MTRVLEGQPDHRDSYLTDEEKGEYLMPCCSRAKSALLVLDL

>F0GH52|F0GH52_9BURK

---MSDASLTVKVARKWQEARDICGFEFVSDDGSPLPGFTAGAHIDVHLPGGLVRQYSLCNHPAQADRYQ

IAVLRDAEGRGGSRAIHDAVRQGDTVRISAPRNHFPLATGAAHHLLLAGGIGVTPILSMAERLSSSG-EP

FAMHYCVRSTERMAFVERIAASAFRDRVRLHVDDGEPAQRFDLAAVLAAAPAGTHLYVCGPRGFMDAVLN

EARNWAEERLHYEFFVVET----SAAPFQVRIASSGRVIDVPAECTVVAALAANGVDVLTSCEQGVCGTC

LTRVLDGEPEHRDSYLTDDEKGDQFLPCCSRSRTAMLVLDL

>A4JJN4|A4JJN4_BURVG

---MSAPTLTVRVARKWQEARDICGFEFVADDGSPLPRFDAGAHIDVHLPGGLVRQYSLCNDPRHGDRYR

IAVLRDADGRGGSRAIHDEVRQGDTLRIGMPRNQFPLARDASHHLLLAGGIGVTPMLSMAEQLSAAG-ES

FDLHYCARSLERMAFVEQINTAAFRDRTRLHVDDGDAAQRFDLPAALDAAPAGTHLYVCGPRGFMDAVLD

AARGWPDARLHYEFFGGAV----VASAFQVRLASSGKVVDVPADCTVVAALAAHGIDVLTSCEQGVCGTC

VTRVLEGEPDHRDSYLTEAEQGDQFMPCCSRARSDLLVLDL

>Q39AH6|Q39AH6_BURS3

---MSTTTLTVRVARKWQEARDICGFEFVSDDGSPLPHFDAGAHIDVHLPGGFVRQYSLCNHPEHGDRYE

IAVLRDADGRGGSRAIHDDVRQGDTVRIGVPRNQFALVPDAPHHLLLAGGIGVTPILSMAQRLFSSG-TP

FDLHYCARSTDRMAFAERINAAAFHDRARFHVDDGAPAQRFDLAAVLAAAPAGTHLYVCGPRGFMDAVLN

AARNWAEARLHYEFFGGTV----ASSAFQVRIASSGKVIDVPAECTVIAALAANGVDVLTSCEQGVCGTC

ATRVLQGEPDHRDSYLTAEEQGDQFMPCCSRARSDLLVLDL

>B4EIK3|B4EIK3_BURCJ

---MSDATLTVRVARKWHEARDICGFELVSEDGSPLPRFDAGAHIDVHLPGGLVRQYSLCNHPEQRDRYQ

IAVLREADGRGGSRAIHDEVRQGDTVRIGMPRNQFPLASDAPHHLLLAGGIGVTPILSMAERLCSAG-IP

FDMHYCARSADRMAFVERINAAGFRDRVRFHVDDGDPAQRLDLATVLAAAPEGTHLYVCGPRGFMDAVLN

AARGWGETRLHYEFFGGAV----VSSAFQVRIASSGKVIDVPAECTVIAALAAHGVDVLTSCEQGVCGTC

VTRVLEGEPDHRDAYLTDDEKGDQFMPCCSRSRTDLLVLDL

>B1K673|B1K673_BURCC

---MSGATLTVRVARKWHEARDICGFELVSDDGSPLPRFDAGAHIDVHLPGGLVRQYSLCNHPEQRDRYQ

IAVLREAEGRGGSRAIHDEVRQGDTVRIGLPRNQFPLAPDAPHHLLLAGGIGVTPILSMAERLSSSG-TS

FDMHYCARSTDRMAFVERINTAGFHDRVRFHVDDGDPAQRFDLAAVLAGAPDGTHLYVCGPRGFMDAVLN

AARGWSEARLHYEFFGGAV----VPSAFQVQIASSGKVIDVPAECTVIAALAANGVDVLTSCEQGVCGTC

LTRVLEGEPDHRDAYLTDDEKGDQFMPCCSRSRTDLLVLDL

>B1Z0L9|B1Z0L9_BURA4

---MNDATLTVRVARKWQEARDICGFEFVSDDGSPLPRFDAGAHIDVHLPGGLVRQYSLCNHPEHGDRYQ

IAVLRDADGRGGSRAIHDEVRQGDTVRIGLPRNQFPLAPDAPHHLLLAGGIGVTPILCMAERLFSSG-MS

FDMHYCARSADRMAFVERINAAAFRDRVRFHVDDGEPAQRFDVDAVLGSAPDGAHLYVCGPRGFMDAVLN

AARNWADERLHYEFFGGAA----ATSAFQVRIASSGKVIDVPAECTVVAALAANGIDVLTSCEQGVCGTC

MTRVLEGEPDHRDSYLTEAEQGDQFMPCCSRSRTDLLVLDL

>B1T279|B1T279_9BURK

---MSATTLTVRVARKWQEARDICGFEFVSDNGSPLPRFDAGAHIDVHLPGGLVRQYSLCNHPEHGDRYQ

IAVLRDADGRGGSRAIHDEVRQGDTVRIGIPRNQFPLAPDAPHHLLLAGGIGVTPILCMAERLFSSG-IP

FDMHYCARSANRMAFVERINAAGFHDRVRFHVDDGDPAQRFDLAAVLAAAPDGTHLYVCGPRGFMDAVLS

AARDWADERLHYEFFGGAV----ASSAFQVRIASSGKVIDVPAECTVVAALAANGVEVLTSCEQGVCGTC

VTRVLEGEPDHRDSYLTEAEQGDQFMPCCSRSRTDLLVLDL

>F0G0C3|F0G0C3_9BURK

----MIDGLTVRVAARHDEAADICSFELVRADGRPLPPFSAGAHIDVAVPGGPTRQYSLCNAPDESHRYR

IAVLRDPNSRGGSAGMHDRVKIGDTLTISAPKNHFPLAHDAKHSLLLAGGIGVTPILCMAERLARID-AS

FDMHYCTRSRTRTAFLDHIARAPYAGRVQFHFDDEAAEQRFDIAARLAVPEPGTHLYVCGPKGFMDAVLT

TARGWPEAQLHYEFF-AAEARQTHDGRFEVQIASSGRVIVVEKHESVVRALAKAGIEIETSCEQGLCGTC

ATRVLAGEPEHFDHCLTAEERNDQFMPCCSRSKSARLVLDL

>Q39P04|Q39P04_BURS3

-MTSGVGGLTVRVAAKREEAVGICSFELVSTDGQPMPAFSAGSHIDVAVPGGPTRQYSLCNAPEEGHRYQ

ISILRDPNSRGGSAGMHDRVQVGDALAISVPKNNFPLAHDATRSLLMAGGIGVTPILCMAERLAAIG-AP

FEMHYCSRSRARTAFLDRISRATYARHVQFHFDDQPDTPAFDIAARLARPESGTHLYVCGPTGFMDAVLG

AARGWPEAQLHYEFF-AAGERVADSDRFEVEVASSGQVVTVEEDESIVQALARVGIEIETSCEQGVCRTC

ETRVLAGEPDHRDVCLTPAERNDRLMPCCSRSKSARLVLDL

>B7WQU8|B7WQU8_COMTE

---MTAATLRVRVARKTQETPDICSLELVHADGELLPSFSAGSHIDVHLPNGLIRQYSLLNDPAEQHRYM

IAILRDPSSRGGSQAVHDLVQEGQAITIGMPRNLFPLAPVAGHSLLLAGGIGITPIVCMAHHLSSQQ-AD

FTLHYCTRSRERTAFRDSIAQSAFAPQTRFHHDDEHPA---GLPALIGDPTPDRHLYVCGPRGFMDAVLE

TARGWAEQNLHYEFF-SASTVSHTDAAFDIKLAHTGRVIPVLKDQSVTQALAAAGVALSTSCEQGVCGTC

LTRVLEGEPDHRDMFLSPVEQNDSFLPCCSRSRTPMLVLDM

>B1Y697|B1Y697_LEPCP

---MSPGLLTVRVARKAVEALDIATFELVADDGSALPAFSAGAHVDVHLPGGVVRQYSLCNDPGESHRYL

IGVLRDAASRGGSVALHDAVHEGDRLQISAPKNHFPLAHEARRSLLLAGGIGITPLLCMAERLAVIG-AD

FELHHCTRSRERTAFVGRIDASRFADRVQRHFDDGDAAQRLDLDALLGQPEAGVHVYVCGPKGFMDAVLG

RARGWPAAQIHYEYF-GAEVAALGDAAFEVQLASSGRIVIVPPGQSIVHALAEAGVAVMTSCEQGVCGTC

LTRVLEGTPDHRDLYLTPEEQNDQFLPCCSRSKSARLVLDL

>C5CZN4|C5CZN4_VARPS

---MNASELNVRVARKHAEALDICTFELVHAEGQALPAFSAGSHIDVHLPNGLTRQYSLCNDPQESHRYL

IGVLRDPASRGGSQAMHELVQEGSLLRISAPKNHFPLAHAATRSVLIAGGIGVTPILCMAERLALSA-AP

FEMHYCTRSRERTAFAGRIAQSAFASQVQFHFDDGDAAQKLSLPAAIGTPQPGVHLYVCGPKGFMDWVLD

GARGWPAEQLHYEFF-SAEIAASTDAEFQVKLASSGRIVTVPKDCTVVQALAAAGVEVATSCEQGVCGTC

LTRVLEGEPDHKDMYLSPEEQNDQFTPCCSRSRSPMLVLDL

>A1VQU8|A1VQU8_POLNA

--MSMTSTISVRVARKQREAVDICTFELVAEEGGPLPAFSAGSHVDVHLPGGLTRQYSLCNDPTESHRYL

IGVLRDPASRGGSRAMHDQVAEGQVLQISAPNNHFPLAHDAQRHLLLAGGIGVTPILCMAERLANTG-AD

FEMHYCTRSPERTAFHQRIAGSGFAPKVHFHFDDGAAGQKLDIPALLSTPASGVHLYVCGPKGFMDAVLN

TAQGWPEAQLHYEFF-AGTVEKSSDASFEVQLASSGRIVMVPSDKTVVQALADAGVDVQVSCEQGVCGTC

LTRVIEGIPDHKDMYLTPEEQNDQFTPCCSRAKTPRLVLDL

>Q222L5|Q222L5_RHOFD

--MSSTSALSVRVARKATVATDICTLELVAQDGTALPGFSAGSHVDVHLPGGLTRPYSLCNDPKETHRYL

IGVLRDPASRGGSQAVHEQVQEGQVLQISAPKNHFPLAHDAKHSLLLGGGIGITPILCMAERLANTG-AD

FELHYCTRSIERTAFRERIAASSFADQVQFHFDDGDAAQKLNLATLLCAPKAGCHLYVCGPKGFMDAVLK

TARGWPEGRLHYEFF-AAEVLKSADASFEVKLASSGRIITVAKDQTVTKALSDAGVEVQTACEQGVCGTC

LTRVLEGVPDHKDQYLTPEEQNDQFLPCCSRAKTARLVLDL

>D4Z908|D4Z908_SPHJU

---MDQTLLEVRVTKKTLEADDIASFELKNCDGSSLPHFSAGSHVDVNMANGLVRQYSLCNVPGETDRYL

IAVLRDPNTRGGSAFMHDRVQQGDTLTISQPRNLFALARDARRSILFAGGIGVTPILAMAERLAQID-AP

FELHYCGRSRSRTAFLDRISSSHFSKQAHFHFDDGPEEQRLDVNAILASPSADTHLYACGPSGFLDFVLG

AASGWSGSCVHREYF-AAAATTSEDQSFEVELASSGQTFHIPPDKTVLEILAANGIDIPVSCEQGICGTC

VTRVLRGEPDHRDLFMTDQEHNDQFTPCCSRARSLRLVLDL

>C3VA34|C3VA34_9PSED

-------MIEVIISAMRLVAQDIISLEFVRADGGLLPPVEAGAHVDVHLPGGLIRQYSLWNQPGVQSHYC

IGVLKDPASRGGSKAVHENLRVGMRVQISEPRNLFPLEEGVERSLLFAGGIGITPILCMAQELAARE-QD

FELHYCARSADRAAFVEWLKVCDFADHVRFHFDNGPDQQKLNAAALLAAEAEGTHLYVCGPGGFMGHVLD

TAKGWADNRLHREYF-AAAPKVSDDGSFEVRIHSTGQVLQVPADQTVSQVLDEAGIIVPVSCEQGICGTC

ITRVVDGEPDHRDFFLTDAEKNDQFTPCCSRAKSACLVLDL

>F6AJ53|F6AJ53_PSEF1

SLYESATMIDVIITAIEQQAQDILSFDLARADGEPLPAFSAGAHIDVHLPDGLIRQYSLCNHPEERHRYQ

IAVLRSADSRGGSIAMHG-LEQGVRLRISEPRNLFPLQHDAKRHLLMAGGIGITPILCMAERLSHTG-GD

FTLHYFARSAQQAAFVERLRQSPFADRVHLHFDNGEPSKRPDTAALIGPADPHAHLYVCGPGGFMEHVLG

SARGWANDNLHREYFAEPD----DQPGFEIQLASSGEVLQVPEGVSVVEVLRGVGVEIPVSCEQGICGTC

LTRVLDGEPDHRDLFLTEDEQNDQFTPCCSRAKSARLVLDI

>F3HHK6|F3HHK6_PSEYM

-------MLDVTVSSRNDEALDICSYELVQSNGQPLPPFTAGAHIDVHLPEGLIRQYSLCNSPGERHRYL

IGVLNDPASRGGSRSLHQHIHPGAQLRISEPRNLFPLAQDARRSVLFAGGIGITPILCMAEQLALEH-AD

FDLHYCVRSAERGAFIQRLQQSTFAERVTLHLDEQPQTA-LNALNVLAAPQPGTHLYVCGPNGFMQHILD

TARGWPEDTLHREYF-TAEPIDTADGSFSIEIASTGQVIAIPANKTVAQVLESQGIDIPLSCEQGVCGTC

LTRVLKGVPDHRDLFLTEEEQNDQFTPCCSRSKTPLLVIDL

>F3DZR4|F3DZR4_9PSED

-------MIDVTVLSRNDEALGICSFELTRPDGQPLPPFTAGAHIDVHLPDGLIRQYSLCNAPNERHRYL

IGVLNDPASRGGSRALHEQIKPGAQLRISEPRNLFPLAENSRRTLLFGGGIGITPILCMAEQLTLEG-AD

FELHYCIRSAERGAFIKRLRQSPFAERVTLHLDEQPDTA-LDASRVLANPDAETHLYVCGPGGFMQHILD

SARGWAEDNLHREYF-SAEPVDTNDGSFSIEIASTGQVIAVAANKTVAQVLESHGIDIPLSCEQGVCGTC

LTRVLKGVPDHRDLFLTEDEQNDQFTPCCSRSKTPLLVLDL

>F3F0E6|F3F0E6_9PSED

-------MIDVTVLSRNDEALDICSFELVRADGELLPPFTAGAHIDVHLPDGLIRQYSLCNAPNERHRYL

IGVLNDPASRGGSRSLHEQIQSGAQLRISEPRNLFPLAGNSRRTLLFGGGIGITPILCMAEQLVLEG-AD

FELHYCVRSVERGAFIERLKRSSFADRVTLHLDEQPTTA-LDAANVLAQPQPDTHLYVCGPGGFMQHILD

SARGWSEDNLRREYF-SAEPADTNDGSFSIEVASTGQVLAVPANKTVAQVLESHGIDIPLSCEQGVCGTC

LTRVLKGIPDHRDLFMTEDEQNDQFTPCCSRSKTPLLVLDI

>F3FF58|F3FF58_PSESX

-------MIDVTVLSRNDEALDICSFELVRADGALLPPFTAGAHIDVHLPDGLIRQYSLCNAPNERHRYL

IGVLNDPASRGGSRSLHQQIKTGAQLRISEPRNLFPLAENSRRTLLFAGGIGITPILCMAEQLALEA-AD

FELHYCVRSAERGAFIERLKHSAFAERITLHFDEQPDTV-LDAATLLAHPQPDTHLYVCGPGGFMQYILD

SARGWSEDTLHREYF-SAEPVDTNDGSFSIEIASTGQVIAIPANKTVAQVLESHGIDIPLSCEQGVCGTC

LTRVLKGIPDHRDLFLTEDEQNDQFTPCCSRSKTPVLVLDI

>B1J756|B1J756_PSEPW

-------MIDAVVVSRNDEAQGICSFELAAADGSPLPPFSAGAHIDVHLPDGLVRQYSLCNHPAERHRYL

IGVLNDPASRGGSRSLHEQVQAGHRLRISAPRNLFPLAQGARRSLLFAGGIGITPLLCMAEQLACNG-DD

FELHYCARSSDRAAFVERLRAAPFADRLFVHFDEQPETA-LDIAQVLGSPQGDVHLYVCGPGGFMQHVLD

TAKGWQQANLHREYF-AAAPVENDDGSFSVQVGSTGQVFEVPADQSVVQVLERHGIEIAVSCEQGICGTC

LTRVLQGTPEHRDLFLTEQEQNDQFTPCCSRAKTPLLVLDL

>F0EDF8|F0EDF8_9PSED

-------MIDAIVVSRNDEAQGICSFELASADGSLLPAFSAGAHIDVHLPDGLVRQYSLCNHPQERHRYL

IGVLHDPASRGGSRSLHEQVQVGARLQISAPRNLFPLADGARRSLLFAGGIGITPILCMAEQLAHSG-HD

FELHYCARSSERAAFVGRIRNAPFADRLFVHFDEQPETA-LDARQVLGNPQADVHLYVCGPGGFMQHVLG

SARGWQEANLHREYF-AAAPVDANDGSFSVQVNSTGQVFEVPADQTVVQVLEQNGIEIAMSCEQGICGTC

LTRVLQGTPDHRDLFLTEEEQNDQFTPCCSRAKTPLLVLDI

>B0KML4|B0KML4_PSEPG

-------MIDAVVVSRNDEAQGICSFELAAADGSLLPAFSAGAHIDVHLPDGLVRQYSLCNHPEERHRYL

IGVLNDPASRGGSRSLHEQVQAGARLRISAPRNLFPLAEGAQRSLLFAGGIGITPILCMAEQLSHSG-HD

FELHYCARSSERAAFVERIRNAPFADRLFVHFDEQPETA-LDIARVLGNPQEDAHLYVCGPGGFMQHVLD

SAKGWQEANLHREYF-AAAPVDANDGSFSVQVGSTGQVFEVPADQTVVQVLEQHGIEIAMSCEQGICGTC

LTRVLQGTPDHRDMFLTEEEQNDQFTPCCSRSKTPLLVLDI

>F8J732|F8J732_HYPSM

--MSGSPLRRVRVAEVEQVAARIKRFRLVDIDNAPLSEFSGGSHISVVMHSGMRNPYSLMGSPFDTSSYQ

ISVLNC-DSRGGSKFMHENVSVGTELEISEPLNLFAPVKLAKKHVLIAGGIGITPFMSMMNDLDSLK-SN

FELHYGVRSLEEGAYCKYLQS-RYGSRVNVYRQDQG-Q-MIPLESILGNQRLGTHMYACGPKPMIDWALQ

TAEGWPAENVHSEQFSAPP----PGKPFIVKLAKSGREIMVKGHQSILEALEEHGVDAPFLCRGGACGQC

ETGVVDGYIEHNDHFLTEPERGKKIMICMSRIQTGSITLDL

>D4XEG8|D4XEG8_9BURK

MSSMGHPTLTLRVARIERVTPLIKRFTLSALDGGALPPFTGGSHIIVQMRDGVNNAYSLMNSPRAPGCYQ

IGVRRQ-PSKGGSAFLHDKVAEGDTLTVTPPNNLFPLDASANHHVLIAGGIGITPFLSQLHELHDQS-GS

YELHYAFRSATHGAFYDELAA-ACGDRVRFYIDDQG-Q-KLDVPALLRAMAPGSHAYVCGPATLIDAVRS

ATRGLPPSRLHVEQF-AAA----APTAYTVVLAKSGKTVHVASGESILNAIERDAAPVPCLCREGVCGTC

ETRILEGEATHFDQYLSAQEKQKTLLICVSRAKGHTLVLDL

>D6JS63|D6JS63_ACIG3

-MASHYEMFPAVVTHVEQLTPLIKRFTFKRQDGQNFPRFSGGSHIIVKMNEQLSNAYSLMSCTQDLSTYQ

VCVRKD-EGKGGSVFMHDQCNEGCEIQISEPKNLFPLAETGNKHILIAGGIGITPFLPQMDELAARG-AD

FELHYAYRSPEHAALLDELKQ-KHAEHVFSYVDSEG-C-SLKLDELI-SSQPGTHVYVCGPKPMIDAVID

CCNRYRDEYIHWEQFASTV----PEDAFTVVLAKSNQEIEVQSNQTILQAIETLNIDVECLCREGVCGTC

ETAILEGEADHFDQYLSDAEKQKSMMICVSRAKGKKLVLDL

>B9BPC1|B9BPC1_9BURK

-MNARESIF-VDVVAVETLTPLIKRFTLALPDGAPLPPFSGGAHVLVSMQNGHHNAYSLLSSPHDTRQYQ

IAVRRE-ASRGGSAFMHEHVAAGTRLAIGSPANLFELARNARRHVFIAGGIGITPFLAQLAE-HAPGDVE

LELHYAYRSPEHGAFVDELKAGPHAANVHTYVDSLG-Q-RLDLMRLFKTLPADAHVYVCGPQGLNDAVYA

NAAGWPKSQLHAEQF-AAT----DTAAFTVVLARSGIELEVPEDTTILQAIERAGVTIDSLCREGVCGTC

EVAILEGEADHRDQYLDDDEKQKTILLCVSRARTPRLVIDL

>B0KMN8|B0KMN8_PSEPG

-MANTYEMFSVRVTDVEQATPLIKRFTLAREDGAAMPAFTGGSHVIVQMQGGFSNAYSLMSDPRDTRSYQ

IGVRLE-QSKGGSAFMHQQVEVGTRLTISSPNNLFALDPSAGRHVLIAGGIGITPFLAQLHELEGGS-TD

YELHYAFRAPEHGAFQDQLANGPHAANTQFYIDSLD-R-KLDLAALCAGLDAQAHLYVCGPKPLIDAVIA

CAAGIAEQRVHWEQF-AAT----PVTAFTVVLAQSGVELQVEEGMTILQAIEKSAAKVECLCREGVCGTC

ETAILEGEAEHYDQYLSDEEKQQSIMLCVSRARTARLVLDL

>Q1IBK9|Q1IBK9_PSEE4

-MAKQYEMFKVRVTGVEQATPQIKRFTLARADGAALPAFSGGSHVIVQM-QGFSNAYSLMSDPRQLHSYQ

IGVRLE-QSKGGSAFMHQQVEVGSELTISTPNNLFALDPSAGRHVLIAGGIGITPFLAQLHELEGGD-TP

YELHYAFRAPEHGAFQGDLEQGPHAGNTRFYI-DSLE-RKLDLAALCAGLADDAHLYVCGPKPLIDAVIA

TAAGIAEPRVHWEQFAAAA----PAAAFTVVLARSGTELEVEEGMTILQAIEKSAAKVECLCREGVCGTC

ETAILEGEAEHFDQYLSDEEKQQTMMLCVSRARTARLVLDL

>E3GD38|E3GD38_ENTCS

--MSDYQMIEVVVRNIEIITPLVKRFTFARKDNQPLPPFRGGSHIIVQMQNGYSNAYSLMSSPFDTSVYQ

IAVRLE-DSKGGSRFMHHHVQPGDTLTISAPNNLFAISPQAAKHLLIAGGIGITPFLSYLPELERAQ-GN

WALHYCFHHQENNAFQDELIAGPWRDRINFYVSEQG-T-RLDLARLLADVEPGTHIYTCGPAALNDAVKT

AAGGIADEQLHFEQF-AIE----NKGEFTLVLSRSGREFIVPEDKTILQIIENNAAKVECLCREGVCGTC

ETTILEGEADHRDQYFSIEEQQKSMLICCSRAKSKRLVLDL

>A6T775|A6T775_KLEP7

--MSDYQMFEAVVRDVEQITPLVKRFTLVSPTGAPLPAFSGGSHIIVQMQDGYSNAYSLMSSPLDTSSWQ

IAVRLE-PSKGGSRFMHQRVRPGDTLTVSTPNNLFAIEPQARKHLLIAGGIGITPFLSHIPELEQRK-AD

WQLHYCFHDADSNAFVDALSAAPWRDRVNVHVSALG-S-RLDLPRLFADLEPGTHVYTCGPAALNEAVKA

AAEQVPASQLHFEQF-ILE----DKSAFTLVLARSGREFTVPQDMTILQVIENNAAKVECLCREGVCGTC

ETMILEGEADHRDQYYSEEEKQQSMLICCSRAKGGRLVLDL

>C1M5H6|C1M5H6_9ENTR

--MSDYQMQEVRVSRIETITAQVKRFTLTATSGEPLPAFQGGSHIIVQMRDGYSNAYSLMSSPFDTSAWQ

IAVRLE-ASKGGSRYLHQNVKPGDTLTISTPNNLFALAPQAQQHLLIAGGIGITPFLSHIPELEHSK-AQ

WQLHYCSPSQDNCAFYDELLQHPSVDRIHLHLSSAG-T-RLDLSRLLADIEPGTHIYTCGPAVLNDAVRE

VASRLDSNTLHFEQF-ALE----DKSAFTLVLARSGREFVVPEEMTILQVIENNAARVECLCREGVCGTC

ETTILEGEADHRDQYFSDDEKQQSMLICCSRAKGKRLVLDL

>D4B6D8|D4B6D8_9ENTR

--MSDYQMREVRVDRIERITAQVKRFTLTATSGEPLPAFQGGSHIIVQMRDGYSNAYSLMSSPLDTSAYQ

IAVRLE-GSKGGSRFLHQNVKPGDTLTISTPNNLFALAPLAQKHLLIAGGIGITPFLSQLPELEHSQ-AE

WQLHYCAPSQKSCAFHDELLQHRSASRIHLHLSSAG-T-RLDITRLLADVEPGTHIYTCGPAALNDAVRE

AASGLCSEMLHFEQF-AVE----DKSAFTLVLARSGREFVVPEEMTILQVIENNAARVECLCREGVCGTC

ETTILEGEADHRDQYFSEDEKQQSMLICCSRAKGKRLVLDL

>E9XJ43|E9XJ43_ECOLX

--MSNYQIFEVQVSQVEPLTEQVKRFTLVATDGKPLPAFNGGSHIIVQMADGYSNAYSLLSSPLDTSRYQ

IAVRLE-NSRGGSRFLHQKVKAGDRLTISTPNNLFALIPSARKHLFIAGGIGITPFLSHMAELQYSD-VD

WQLHYCSRNPESCAFRDELVQHPQAEKVHLHHSSTG-T-RLELARLLADIEPGTHVYTCGPEALNEAVRS

EAADIATDTLHFEQF-AIE----DKTAFTLVLARSGKEFVVPEEMTILQVIENSAAKVECLCREGVCGTC

ETAILEGEADHRDQYFSDEERQQSMLICCSRAKGKRLVLDL

>B1LD70|B1LD70_ECOSM

--MSDYQMFEVQVNQIEPLTEQVKRFTLVATDGKPLPAFTGGSHVIVQMSDGYSNAYSLLSSPHDTSCYQ

IAVRLE-NSRGGSRFLHQQVKVGDRLTISTPNNLFALIPSARKHLFIAGGIGITPFLSHLAEMQYSD-VD

WQLHYCSRNPESCAFRDELVQHPQAEKVHFHHSSTG-T-RLELARLLTDIEPGTHVYTCGPEALNEAVRS

EAADIAVDTMHFEQF-AIE----DKTAFTLVLARSGKEFVVPEEMTILQVIENNAAKVECLCREGVCGTC

ETAILEGEADHRDQYFSDEERQQSMLICCSRAKGKRLVLDL

>A1WLB5|A1WLB5_VEREI

--MTAAT-LLLRVAQARQLNPLIRMLRLCAEDGGALPGFAAGAHIRVSLPDGDWRHYSLIDTTHNAAQYL

IAVRNEPGGRGGSRFMHQRLKEGDTIAIEAPKNEFPLHTGPGGSVLVAGGIGVTPLATMAARRRAEG-AA

VRMHYAGRSRDLMAFLPELQN-LLGDDLRVHADAEA-GAPLDIDALLDGVPVGDHLYVCGPGAMLDAMLG

RTRGWEPGRVHWEIFAAPA----AAEPFEVELARSGQRFTVPAGQSILDCLIENGCDPMFDCKRGACGVC

AVPVLEGGIDHRDHVLSAREKGSVMQICISRAKGARLVLDI

>Q1LFU7|Q1LFU7_RALME

MAARPIKTVSVVVSAIDEPLPGVKRFVLSDQDHWPLPPFTPGAHIDLHLGGGLVRTYSLCNEPSDRNRYV

IAVKHE-AGRGGSAFVHERLDVGASIGVSLPRGGIRTTDTG-MNVFIAGGIGVTPFISTIRDLELRGQTN

YVLHWS--SAGVPSLVDMLGDAIAAGRVRLYDTRVE-S-APDMDAIL-NACENAKAYCCGPMRMLDAFER

IVEGWPDARKHIERFAPPK-PVEDTDPYTVVLARSGKEAIVEPHVGLVGTLEALGADVSVSCGGGVCGAC

RTTWLEGPPIHRDRVLSPEERAQDVMVCVAGCAGSRLVLDL

>Q89IV7|Q89IV7_BRAJA

MKVRPITTIKTIVTGIEEAGAGTKLFTLADPDRWELPPFKPGAHIDLHLPNGLVRTYSLCNEPADNARYV

VAVKRE-DGRGGSRALHDEVGIGDVIGVSLPRGGLDPG-AGARHVFIAGGIGVTPFLSMARHLVQTG-AG

FVLHLIVR--EEVPLAAHL-A-PDAGRVVVHCTSRA-G-RPDLAALIGEGGADTLVACCGPEGMTEDFEQ

VTAGWPAANVHIERFVAPP-PVIDPDPFTLSLARSGAEIQVRAGQTMLAALQEGGIDIATSCCGGICGAC

KVGWIEGKPVHRDRILSPYERERYLMVCVAGSDAERLVLDL

>F8JG45|F8JG45_HYPSM

AAVSNSMTIPTRLQSVTYATDDVRLFEFA-SN-GPLPLGDAGAHIDVHLPEKRLRQYSLI-TPLSSKSVV

IAVKREPSGRGGSVFLHDTARVGSEISISPPRNNFELDENASETLLLAGGIGITPIYSMFVRLLQLG-RP

VRLHYWCRSEAHALFRDEL-K-HHANTATIH-SAEP-S-RPSVAEIIKSAGPQTEIYCCGPTRMLDASAQ

GVA--DPARLHIERFGGEAIKPSGDAAFTVHLARKGIDVPIAPEQTILEVLLALGVDVPYSCEEGICGAC

ETKVISGSPSHCDRVRSADESRQTMMICCSRCFDGRLVLDI

>Q1QUQ2|Q1QUQ2_CHRSD

--MTATPF-EVVIDSHQRLSSAIYQIELVAADGAALPPAPAGAHLEVRLPNGLIRHYSLLDDA-RHGRYR

IGVLHDPASRGGSAFLAEQASVGTRLCVSAPRDRFPLADDRQHYRLIGGGIGITPLVAMARRLVERG-DT

VEVHYLVRHRDEAAFLDEL-R-APASQLHLHASATQ-G-RLAPRALLGDYTPETGVYACGPEGLLDALTD

AARAWPPGALQMERFRGAS-QAPAARPCRIELARSDKQFTLAEDETLLDGLARAGAAPDSLCCEGACGTC

GIPVLEGEVEHRDVLQSDAEKNDIIYVCVSRPKGERLVLDL

>P94680|P94680_COMTE

----MSADVPVTVAAVRAVARDVLALELRHANGQPLPGASAGAHIDLALPNGLVRQYSLVNATGQADCYQ

VAVGWD-NSRGGSVWIHEKLKVGQALRVTHRATCSEMAPEHRRVLLLAGGIGVTPIYAMAQACAQQG-VD

VELWASARSAPRLAYLEEL-KALLGQRLHLHADDEQ-GGPMNLTERL-ATQRWDAVYACGPAPMLDALTA

ATAHWAPGSVRMERFKGAE--QPASEPFELVLQRAGLSTTVDAHESVLDAMERVGVDFPWSCREGICGTC

EAPVLEGEVQHLDYVLSPEERQRRMMVCVSRCGGGRLVLDI

>A3VA32|A3VA32_9RHOB

SLPGQDMTLPVVVRSMTLLARDTLAVEFT-PDGVTLPPFDAGAHVDLLLPNGIRRSYSLCNAPGDTGRYV

VGVKKA-PSRGASSYIHDTLRVGQAMKISAPKNNFPLDESAAKSVLIAGGIGITPMMSMIDRMEATG-AD

WTLYYSCRTRADAAFLDRL-DLLDKRCVNLNFDGEPGGAMLNLAEII-GAEDGVHFYACGPAPMLDAFEA

ATAGLPEGHAHLERFGGEPLPVSDDATFEVECMQSGLNLTITPETTILDALLDNGIDIPFSCMDGVCGSC

RVGVVEGTPDHRDMVLSDGELNKVMMVCCSGSRSPKLVLDI

>Q1LBR8|Q1LBR8_RALME

-MSMNTSQIELRLKRITFEAERISSFEFVSADANPLPGFQPGAHIDLHLQQGMIRSYSLANAASDPLYYR

VAVQREPNGRGGSRWAHDKLRVGDKVWATPPQNDFPLDECASSTVFFVGGIGITPVLPMLRRLDAIG-RE

WKLMYASRSPGETAFAEELQRAGRGRIVHFH-DSEP-GQRLEIRATVAALPASTHVYCCGPTGMIDDFLA

VTAERDPSTVHYERFGA---AQQAATGFDVVLHRSGAKYRVEPGKTILDVLLDNNVAVPYSCCNGVCGSC

RTEIIDGQADHRDDFLSDEEKNQAIMVCCSGARSKTLVLDL

>Q13QL9|Q13QL9_BURXL

MSQHNADRIDVRLTQIRLEADDVASYEFR-PIGTPLPAFEAGAHIDLYLPENRVRSYSLVNAPHERHRYV

IAVQRE-AGRGGSAWMHRTPRVGDRFTIGAPMNDFPLCEAARQSVFICGGIGITPIVSMIRRLERTG-AS

WQLHYAARERSRAAYADDLAD-EAGSRITMHLESEG-G-RLDLRRVVERAPADAHLYCCGPRGMVDAFIE

ACASRPRAQVHFERFAA---ANESATGFDVVLGRDGRRIPVAPGKTILDTLLDHGVDVQYACSAGVCGTC

RTGVIDGVPDHRDDYLTDEEKNRAVMVCCSGSLSPTLVLDL

>Q143N8|Q143N8_BURXL

MSQHSPERIDVHLTQIRLEADDVASYEFR-PAGGPLPVFEAGAHIDLYLPENRVRSYSLVNDPQERHRYV

VAVQREAAGRGGSAWMHRVPRVGERFTISAPKNDFPLCESARESLFICGGIGITPVVAMIRRLERIG-AS

WRLHYAVRECSRAAYADDLQLDNADSRVEMYF-QNED-RRPDVRGIVR-APAGTHLYCCGPRGMVDDFIA

ACAGRPQDEVHFERFAAAN----DAA-FEVVLHRDGRRIAVAPGKTILDTLLDHGVDVQYACSSGVCGTC

RVGVIDGVPDHRDDFLSADEKNGAIMVCCSGSLSPTLVLDL

>F6F3N9|F6F3N9_SPHCR

-MTNPVSTIDMTVTQITRVAKDINSYELRPP-GVILPEFTAGAHIGVSLPNGIQRSYSLV-NPQERDRYV

ITVNLD-NSRGGSRYLHEQLRVGQRLSIVPPANNFALVETAPHSVLFAGGIGITPIWSMIQRLRELG-ST

WELHYACRGKDFVAYRQEL-EQAAAARFHLHLDEEADGKFLDLAGPV-AQAQDSIFYCCGPEAMLQAYKA

ATADLPSERVRFEHFAALP----ADDVFTVVLARSGQEFTVEPGMTILETLLQNGISRNYSCTQGVCGTC

ETKVLEGEPDHRDWVLSDEKKNSTMLICCSLSKSPRLVLDI

>Q5WPV4|Q5WPV4_9SPHN

-MTSPASTIDMLVTQITRVAKDINSYELRPP-GVILPEFTAGAHIGVSLPNGILRSYSLV-NPQERDRYV

ITVNLDRNSRGGSRYLHEQLRVGQRLSIVPPANNFNLVETARHSVLFAGGIGITPIWSMIQRLRELG-SS

WELHYACRGKEFVAFRQELEQAEAGATFNLHLDEEANGGFLDLSPVA-RAGEDSIFYCCGPEAMLQAYKA

ASADLPSERVRFEHFGAAP----SAPDVFTVLARSGQEFTVEPGMTILETLLQNGISHNYSCTQGVCGTC

ESKVLEGEPDHRDWVLSDEKKNSTMLICCSLSKSPRLVLDI

>B2TEV1|B2TEV1_BURPP

--MKSSETFEVEVRGIRIEAETVRSFELW-PSGEL-PPFTAGAHIDVHLSHGMTRQYSLTNSL-ERHRYV

IAVQRDPGGAGGSMSMHDSVALGSRLIVSAPRNYFPLNEAAVHSVLIAGGIGVTPLLAMVRRLCQLG-LS

WDFYYCARVPARAAFLSEL-KELAARALHCVFDGEAGVTSLNLGDVR-AAHPDSDFYCCGPAPLMDAFST

TFASLPPERVHLEYFKAPVADTLGEAAFTVTLSRQGKTLVVPPNRSILEVLRENGVPMLSSCRQGICGTC

ETRVVEGVPDHRDHVLTPAERNQTMMICVSRCKGEALTLDI

>A6T0Z9|A6T0Z9_JANMA

--MNEQETRQVWVRAIRIEAEQIRSFELC-PAGERLPAAAAGAHIDVHLPNGLVRQYSLL-HPGESKAYM

IGVNRDPVSRGGSSYLHEAVKPGDVLTISVPRNNFPLHEEAAHSVLIAGGIGITPIFSMVQRLNQIG-RA

WTLYYCTRTEERAAFLDEL-KTLAAGNLHTVFDQVPGNQMLNLAELE-AEHGAAHFYCCGPGMLLQGFQQ

ATATVAAERVHVEYFSAPAVAS-NVATFSVTLARSGQTFQIPADQSILEVLLSKGVSVLSSCREGVCGSC

ETAVLAGEPEHRDAVLSAAERNRTMMLCVSRCKGTSLTLDL

>D5WJL8|D5WJL8_BURSC

MSEQLNEVMTLRLTAINYLARNTLAFTFEDPEGNLLPGAEPGAHVGVILPNGLTRQYSLLENSTQRS-YV

VGVKLDPQSRGGSRYMHETMRVGDLIQIEAPRNNFPLNEDAGHTVLVAGGIGITPILCMARRLKELG-QS

PRIYYSCRERVDVAFADELKT--FEGA--LHVDAEA-GTFLNIADVV-KNEPSTHFYCCGPGPMLEAFEA

ACASLPQQQVHVEYFSA---KQEAALNFVIELRKSGKTLTVPQGKTILNVVRDAGIPISYSCEEGVCGAC

EVRVLEGQPDHRDAILSEPEKNNTMIICCSGCKGDRLVLDL

>C4TNS5|C4TNS5_9BURK

------TYLQARVHQMRYEAAGTLSVELRPLVAEEFAPVQAGAHIDLHLADGLIRSYSLI-NPGERHRYV

VAVSLDPASRGGSRFVHEKLRVGQAIQIGGPRNHFPLDETASHSVLVAGGIGITPVLSMLRKLHALG-RT

AHLIYCASSRENAAFVPEIEAAQAGGRVTWHFKDEK-GVRADLYSLL-QAE-GAHFYACGPLVFLASYED

SCQKLGLAHVHLERFAAAPLAAPQTPGYAVELRRTGKTVQVAAGTSLLDTLINAGMNPEYSCREGVCGAC

EVRVISGDVDHRDQILSEQERNKSMMICVSGCRSGNLVLDC

>B7WRK5|B7WRK5_COMTE

------TYLQARVHQMRYEAAGTLSVELRPLVAEEFAPVQAGAHIDLHLADGLIRSYSLI-NPGESHRYV

VAVSLDPASRGGSRFVHQRLRVGDVIQIGGPRNHFPLVETAPHSVLVAGGIGITPVLAMLRRLDALG-RT

AHLIYCASSRASAAFVPEIEAAQVGGRVTWHFKEEK-GVRADLHNLL-QAE-GAHFYACGPLAFLDSYED

SCGKLGLAHVHLERFAAAPLAAPRTPGYAVELRRTGRTVQVAAGTSLLDTLINAGMNPEYSCREGVCGAC

EVRVISGDVDHRDQILSEQERNKSMMICVSGCRSGNLVLDC

>Q5D0Y0|Q5D0Y0_COMTE

------TYLQARVHQMRYEAAGILSVELRPLAAQEFSSVQAGAHIDLHLADGLIRSYSLT-NPGERHRYV

VAVSLDPTSRGGSRFVHQRLRVGDVIQIGGPRNHFPLVETAPHSVLVAGGIGITPVLAMLRRLNALG-RT

AHLIYCASSRASAAFVPEIEAIQAGGRVTWHF-KEEKGVRADLHNLLQEHAEGTHFYACGPLAFLDSYED

SCGGL--AHVHLERFAAAPLAAPRTPGYAVELRRTGRTVQVAAGTSLLDTLINAGMNPEYSCREGVCGAC

EVRVISGDVEHRDQILSEQERNKSMMICVSGCRSGNLVLDC

>Q2KXR3|Q2KXR3_BORA1

LPGSDKGLLELRVQAIRYAARDIHLVELVHPDGAELPAATPGAHIDLYLGQGLVRSYSLV-EPGHPRSYL

LGVKRE-ASRGGSRYVHEQLRVGTTLTVSAPRNHFPLAEHARHSILIAGGIGITPIWCMAQQLEAVG-AS

WELWYSARTRADAAFLDRF-E-PLFAKVHLHFDDEQ-GCFLDLGAVVRGAAEDTHLYCCGPTPMLDAYEA

AAAQRDPRTVHLERFKAAA----SLE-FVVALARSGKEMAVPEGASILKVLLDNGVPVDFSCQEGICGCC

EVAVLEGEVDHRDAVLSESERGKTMMVCCSGAKSARLVLDL

>C5CQT2|C5CQT2_VARPS

---MSSPTLNALVHTMRYEADGIVSVEFRPAPETEFPAFEAGSHIDLHLPNGLVRSYSLC-NPADRQRYV

VGVLND-KSRGGSRYVHQQLRVGMTLPISVPRNNFKLEEGADHSVLVAGGIGVTPIWCMLQRLAALG-RP

VELVYCARTRKEAAFCDAIEAREHAVPVTWHFDEEK-GAPPDLAALLAGKGAASHYYCCGPTPMLDAFEK

SCEGY--AHAHIERFAAVHVEAPSATC-VVECAKSGRSVEVPPGKSILDSLIDAGLNPDHSCKEGVCGAC

ETAVLEGEIDHHDGILTKIERNKTMMICVSRCKGERLVLDI

>E6UYE2|E6UYE2_VARPE

---MSSPTLNALVHTMRYEADGIVSVEFRPAPAVDFPAFEAGSHIDLHLPNGLVRSYSLC-NPADRQRYV

VGVLNDRKSRGGSRYVHQQLRVGMTLPISAPRNNFKLQEDAERSVLVAGGIGVTPIWCMLQRLAAIG-QP

VELLYCARTRKESAFCESIEALEKAIPLTWHFDDEK-GAPPDLAKLL-AGKGGTHYYCCGPTPMLDAFEK

SCEGY--ANAHIERFAAVHVEAPSATC-VVQCAKSGKSVEVPAGKSILDSLIDAGLNPDHSCKEGVCGAC

ETAVLEGEVEHHDGILTKIERNKTMMICVSRCKGERLVLDI

>Q126G1|Q126G1_POLSJ

----MSTTLNAFVHTLRHEADDVISVELRPLPGAEFPAFTAGSHIDLHLPNGMERSYSLSNSCEERNRYV

VGVLRD-ASRGGSRCVHEQLRVGMRLPISAPRNNFPLHEEAGHTVLIAGGIGVTPILSMARRLKAAG-RP

FEVMYFARSRQGAAFVVEL--EALGMPLMLHFDDEK-GGPPDLKALLRAPDAQTHYYACGPTVMLDAFEK

ICGELGHENAHIERFSAVAVAPSANATFTVELRRSGRTFTIKPGKSILDTLLDAGVSVDHSCCEGVCGSC

ETRVLAGEPDHRDSILSAKERNKVMMLCVSGCKSESLALDL

>A9BRP4|A9BRP4_DELAS

----MSTNLQAFVHTLRFEAQDTISVDLRPVAGGELPAFTPGSHIDLHLPNGLVRSYSLS-NPSERHRYV

VGVLRD-ASRGGSRCVHESLRVGMPITISEPRNHFALDESAAHTVLVAGGIGITPLLCMARRLKGQG-RS

FEMLYFARERKGAAFLEEL-Q-ALGMPLHLHFDSEA-GGPPDLRALLAQPDAGTHHYACGPTPMLDAFEK

FCAELGHANAHVERFTPVEVKAADDANYTVELKRSGRFIEITPEKSLLDTLLDAGIQMDHSCCEGVCGSC

ETRVLEGIPDHRDSVLSPKEKNKVMMVCVSGCKSERLVLDI

>B7X3B7|B7X3B7_COMTE

----MSNSLQAFVHTLRFEAQDTISVELRPVDGGEFPAFTAGSHIDLHLPNGLVRSYSLSNDSSERHRYV

VGVLRD-ASRGGSRCVHESLRVGMPITISEPRNHFALDEAATHSVLVAGGIGITPMLCMARRLKSLG-HS

FEMLYFARERKSAAFLAEL--QALGMPLHLHFDAEE-GCPPDLRALLAQPDAGLHHYSCGPTPMLDAFEK

FCAGHVN--AHIERFTPVEVKASSDANYTVELKRSGRFIEITPDKSLLDTLLDAGVDVDHSCCEGVCGSC

ETRVLEGVPDHRDSVLSPKERNKVMMVCVSGCKSERLVLDI

>Q46V97|Q46V97_CUPPJ

--MSAHKTLTMRVQAMRYEARGIVSLELQDPDGATLPEYGPGAHIDLHLGNGLVRSYSLCGAPEVRDRYT

VGVLQD-NSRGGSRYVHEQLRVGATLNVSAPRNNFELDESAAHTVLVAGGIGVTPIVCMARRLAELG-RS

FTLLYCARTRAEAAFVDSL-S-AYGDAVRFHFDDEG-A-PPDMKAMLAGQDAQTHLYCCGPGPMLNAFEA

ACEGYPN--VHIERFAA-DPSTESVQEYVVQLSRTGSLVKVPSGKSLLDALLEAGLDVEHSCREGVCGSC

ETAVLEGCPDHRDSVLSNSERNKTMMVCVSGCKGSRLVLDL

>Q0K388|Q0K388_CUPNH

--MSAHQSLTLRVHAMRYEARGVVSIELQDPEGKTLPEYSPGAHIDLHLGNGLVRSYSLCGAPEARRRYT

VGVLLDRGSRGGSRYVHEQLRVGATLTVGAPRNNFELDESAAHTVLVAGGIGVTPIVCMARRLAELG-RS

FTLIYCARSRAEAAFVEQL-S-AHGDAVHFHFDDEA-GVPPDLNAMLAGQEVQTHFYCCGPGPMLKAFEA

ACEGYPN--VHIERFAA-DPSTEAVQEYTVSLSRTGTTVKVPSGKSLLDALLDAGVQMDYSCREGVCGSC

ETAVLEGCPDHRDSVLSNSERNKTMMVCVSGCKGSKLVLDL

>Q02SR0|Q02SR0_PSEAB

------SRLHLQVTDFRAVARDVVLVELAAVDDTELPAFTPGAHLELHLGNGLIRHYSLLNDCRERQRYQ

LAIGLGDDSRGGARFIHRQLRTGDRLHAAAPRNHFMLREDAARYCFVAGGIGITPILSMVRWCQAQG-KD

WRLLYAVRERQRAAFLAELMT-LGGERTRLHCSDEG-D-PLDSTALIAGLGADEELYCCGPDGLMQAIRD

AG-SGCAERLHFERFDAPVAPASSSGDFRLELRRSALSLRVESHQSILEVLEEQGLEPPYACRAGICRTC

ETRVCAGEPEHFDHVLSDTERGETLLICVSRCRGNYLELDL

>Q39A66|Q39A66_BURS3

IPFPPGDRIAAVVVEAAMLTPEIRGIVLAAADGAALPPAEPGAHVEVRLPDGIVRHYSIVERAADGSTYR

IAVLREPRSRGGSAYLVERLAPGDVLDVSAPRNHFALDEHASEYVLIAGGIGITPILPMARRLDALG-KS

FTLLYLARSRERMALHDAIERGPLREKARLHCSEQH-G-EADLRTLLGVPRAGAQVYVCGPGRLIDSVLE

VARDWPAGTVHFERFAGAAPPREPEAQFEVELARTGRRVAVPAGRSILDVLRDERIAVDSVCGEGVCGTC

AVTLLDGEAEHRDCLQTDAERNNLIYICVSRAKSSRLVLDL

>Q5YU41|Q5YU41_NOCFA

VRRTGYDL-PVVVERIETVAEDVVAVTLRHPAGTQLPAWTPGAHLDVFLPSGTQRQYSINGDPADRRRYR

IAVRRI-ADGGGSREIHDTVRIGDRLRIRGPRNAFDFV-AAPRYLFLAGGIGITPILPMVRAARAAG-RD

ARLVYTGRSLASMPFRAELAAVPGAD-IDIRPDDEY-G-QPDVAAIVRTALPGTAVYVCGPPPMLAAAVR

AVGDDPSISLHTERFSPPP----VAGEFAITLARSGTQVRVGAEETALAAIRRVLPGVAYSCRQGYCGTC

KVRVLAGQVDHRDRALSAAERAESMLTCVSRAADGDLVLDL

>Q0S0Q8|Q0S0Q8_RHOSR

RPKPVRNWGELIVAEVRTEAADVVSLKVVSPDGRDLPSWIPGAHLDLILPSGKQRQYSLCGDPDDLSSYR

IAVRRI-TGLGGSIEVHDSLRAGTPITVRGPRNAFPLV-AADSYLFVAGGIGITPILPMLKRCHDRE-LP

WRLVYLGRSRATMPFLDELAR-YSRGQVEIRPDDEL-G-APDVAGVVATASPGAAVYMCGPTPLMTTARR

VM-IDPTGSLHTERFSPLP----VVDEFEIHLARRGTTVSVGPDETALAAIRREVPGVAYSCQQGFCGTC

RVRVLAGEVDHRDRILTHDEKEDSMLICLSRSAGGSLVVDL

>F8AWK8|F8AWK8_FRADG

PPPAPASTLNLVVADRRYVADGVVRLSLAAPDGAALPAWWPGAHLELHLPSGRRRRYSLCGDPADRGTYQ

IAVRRL-TGGGGSVEVHDALPAGSRLTVGRPRNAFPFAAEP-RVLFIAGGIGITPILPMVREAARRG-LD

WRLVYSGRTRTSLPFTDEL-R-RFHGRVEILSDEEH-G-RPDATSLLRRAPAGAAVYACGPPPVLDGIQA

AFDASPASGLHVERFASAP----IRDPFELRLARSGRVLPVPADRSVLDVLAEAVPTAAYSCRRGFCGIC

RQRVLAGAVEHRDHRLTDTERDGAMLVCVSRARGERLVLDL

>F1YNA8|F1YNA8_9ACTO

DPAADDGVLTLDIIDRAVVAENVIALTFAAPDRSILPEWHPGAHLDLLLPSGRMREYSLCGDPGDRHTYR

IAVRRIPDGGGGSIEVHDGLRIGDSIQIKGPRNAFPLVLPG-FLRFVAAGIGITPILPMVAMADRYG-LD

WSMIYSGRSADSIPFAEELAQ--YGDKVTVRTDDVD-G-LPTMAELVGD-DAGLSVYTCGPVPMLESLRT

FLTDRSDIELHFERFSPPP----ILDEFQVTIASTGRTETVAADESVLTALRRVDPAVPYSCQQGFCGTC

RLKTSAGDVDHRDTLLTQPEREGWFLPCVSRCDSESLTVEI

>D0L5Z7|D0L5Z7_GORB4

DTVRTDGMSTVAIVKREVVAEDVVALTFADPDGAMLHRWHAGAHLDLLLPSGRMREYSLCGDPADRYTYR

IAVRRIPDGGGGSVEVHDDLKVGDLVSIKGPRNAFPLAIPG-HVRFIAAGIGITPILPMLAAAERFG-LD

WSMIYTGRTPDSIPFISEV--GRYGTKVTVRTDDQD-G-LPSMAELLVDPETGLAVYCCGPVPMLESLRR

HLGDRPDIELHYERFSPPP----VENEFTVTLASTGQEIPVAADESALAAIRRVLPSVPYSCQQGFCGTC

KVRTLAGDIDHRDNILTEPERSGTMLTCVSRSHGGNLTLDL

>A1UH05|A1UH05_MYCSK

EPRPREHLITLTVTDRRMVAEDVVALTFTASDGATLPPWHPGAHLDLHLPSGRIRQYSLCGDPSVRDSYR

IAVRRI-DGGGGSVEVHEKLHPGVAVGTLGPRNAFPLTLPG-YLHFVAGGIGITPILPMLALAQRFE-VP

WTMSYAGRSRASLPFLDEV-A-AYGERVRLHTDDVA-G-VPAAGDLLGECPDGTTVYACGPAPMLTAVRS

ALAGRAGVELHFERFAAPP----VVDPFSVSITSTGAVVPVAADETLLSALDRSGVHAAYSCRQGFCGTC

RTRVLAGTVDHRDTLLTEPERGGMMLTCVSRAAGQRLTLDL

>G4I7Q0|G4I7Q0_MYCRH

TPPGRDHTLTLQIIGRHVVARDVVALTLAAPDQRPLPLWHPGAHLDIHLPSGRIRQYSLCGDPDLRSYYR

IAVRRIPDGGGGSIEVHDGLGVGARVTSSGPRNAFPLTVPG-YLRFIAGGIGITPILPMLGLAERLG-VD

WSMIYAGRSEESIPFLDEV-R-RFGSRVHIRTDDVH-G-VPTAAELLGECPDGVGVYTCGPAPMLTAIRA

ALAERDDVELHFERFAAAP----VVDNFTARVASTGATLAVRSDETLLSALRREQIAAPYSCQQGFCGTC

RTRVINGVVQHRDTLLTEPEQAGMMLTCVSRAEGGHLELDL

>B1MEC4|B1MEC4_MYCA9

PENHVDRTLKLVVKDRWIEAQDVASLVLASPDGDRLPPWHPGAHLDLLLPSGRMRQYSLCGDVSDPYHYR

IAVRRIPGGGGGSIEVHDALQIGATVSILGPRNAFPLTQPGRKLHFVAGGIGITPILSMIAFAEQLG-KP

WTMVYTGRHRDSLPFLNELKR--FGDRVIIRTDDQS-G-LPTADDLLPGVGENDAVYCCGPAPMLAVLQR

RL-EMPSVELHFERFAAAP----VVDPFEVQLGPGGPVLDVPADRTALDVIKKRLPHVAYSCQQGFCGTC

KVNVLAGTVDHRDQILTESQREGQILTCVSRGEGR-LVLDL

>Q5YVD5|Q5YVD5_NOCFA

VSTVDDRRLALVVTERRVEAQDVVSLRLEAPDGRDLPAWRAGAHLDLELPSGRIRQYSLCGDPADTRAYR

IAVRRIPDGGGGSVEVHDALPTGTPVVIRGPRNAFPFAVPG-HLHFVAGGIGITPILPMARLAHRLG-VD

WSMVYVGRSRDTIPFLDEL-E-TFGDRVTVRTDDEH-G-LPDAAALLAGVSIGTAVYCCGPVPMTGTVAA

AVRGMRGVELHSERFTPAP----VVDPFEIELAATGEVLRVAADQTALSAVLAARPDHPYSCRQGFCRTC

KVRVLDGEADHRDSVLGPDERAGVLLPCVSRCDGGRLVLDL

>B1MHL3|B1MHL3_MYCA9

PPKWVDRSLHLTVRERSVVAQDVVALSLGAHDGGELPRWRPGAHLDVTLPSGAVRQYSLCGDPRDRFSYR

IAVRRIPNGNGGSIELHDGVSVGDVLGVKGPRNAFPLAHTGRRFHFVAGGIGITPILPMLAVATELG-LP

WTMTYTGRSRESIPFRDELAR--YGDRITIRTDDED-G-LPTPADLLPPLHPDLAVYCCGPAPMLNVVRD

AVAECDGAELHFERFSAPP----IEHPFQVQLGIGGPVLEVGADQSALAAVLTSRPGTPHSCRQGFCGTC

KVKVLAGRPDHRDSLLTETQREGQMLLCVSRADGERLVLDI

>C6RQ75|C6RQ75_ACIRA

IGAPDLKAMTVRVQAVYPEAEDIIRIELAAIHGEELPRWSAGSHIELVLPNGLSRKYSLCGLATDQ-FYT

IAVKREQESRGGSQWIHQYLKAGEQIYIKGPKNFFKLNLQASQYVLIAGGIGITPILSMASSLREQG-RP

YRLIYLSRQRASMALLKEV-A-AHGSAAELYISSEG-K-RIDLQQLLSALPAGTQVCACGPEALLDTLTN

YTEDLSQVQLTVEHFGSGKNLFLYENDFEVELLDSGLTLTVARDQTLLDCLLDKGIDVSFDCTEGLCGSC

QLPVEEGEIDHRDKVLTRAERMKSVISCCSRGKGK-LKLKL

>Q2T7C7|Q2T7C7_BURTA

GESNARGARALRVDSMTPVAERVLRVRLVAPGGEPLPRWSAGAHIDIECGDGLSRQYSLCGDPAERGAYE

IAVLREPASRGGSAWMHERLRPGMRVKIRGPRNHFRLDAHASRAILIAGGIGITPIGAIARQARAGG-ID

YTLHYCGRSRRTMAFVDELAA-LHGERLRLHVSDEG-T-RADFSAL--DVAPGTQVYACGPARMLDALAA

RAAHWPAHALRVEHFAAAAPRVDPAAPFEVELKDSGLTLTVPANRTLLATLRAANVDLQSDCEEGLCGSC

EVRVLAGEIDHRDSVLSAGERNTRMMACCSRAKRGRIVLEL

>D6Y814|D6Y814_THEBD

IG-SARRPLPVTVLAIEPEAEGIVRITLADAHGKPLPAWSPGAHVDLEL-GGLTRQYSLCGDPDDRSTYQ

IAVLKDPDSRGGSRYVHERLAVGDVLLMRGPRNNFPLDPGARRYVFIAGGIGITPIITMADRARRRG-AD

YHIHYCGRSAAAMAFLGRLRR-DHGDRLTLYRSAEG-T-RLDIARLLAEPQEGTQIYVCGPQRMLDAVAE

ATRHWPDDAVRVESFVSALDRFDPAKAFDVRLADSGRVIRVPADQTVLGALRAAGIDVLSDCEEGLCGTC

EVPVAEGEVDHRDMVLTKAERHSSMMTCCSRARGGSITLRL

>C7SFP5|C7SFP5_9NOCA

IGEPSKNTIAMAVSGLESIADDILLITLRDTSGRPLPKWSAGSHIDVDC--GVSRQYSLCGDPHDRTTFQ

VAVLHD-ESRGGSRWIHTELAVGATLRVRGPRNHFKLDPDAKRYVFVAGGIGITPVIAMADQVKAAG-GD

YEIHYAGRSRTSMAFLDRLAR-DHGESVRVYPGDEG-V-RMDLPSLFADPEDGTQVYSCGPERLLSALSE

ATAHWPDDTLHVEHFSSTLEELDPSKGFDVVLKDSGITVPVAADQTVLQALRAANIDAQSDCEEGICGAC

EVPVLDGEVDHRDLVLTKTERGKTMMTCCSRACGDKLTLQL

>F6EFM8|F6EFM8_AMYSD

PVESASVRHTLRVKQKTWEADGVISLSLVDPSGRPLPTWEPGAHVSLRLPNGLVREYSLCSSPHDPFVWT

VAVLRTPDSRGGSSYVHETLSVGELVEVEGPRNAFHL-EEARRHVLIAGGIGITPILAMARRLEESG-AE

WTMLYTGRARSTMAFADEV-KSFGSEKVTIHADDEAGGAYPDIRGML-HALPTSLVYCCGPEPLLQAVSD

AMT--NASLLRVERFKAP--ERTAADSFDILCERSGTRVPVAENESVLDALTGAGVEVESSCTEGICGTC

EVRVLRGDVDHRDFLMSPEEHEGTMFVCVSRCRSKELVLDL

>A1R610|A1R610_ARTAT

PDVPEPKMLTLRVHQKTWEADGVIRMMFVDPAGAALPQWLPGAHLSLHLPNGLTREYSLCSDPEDLTSWT

VAVLRTPDSRGGSRLIHDELPVGTLIKVEGPRNNFHLD-HAGRYALVAGGIGITPIISMVRRLESVG-AD

WSLLYTGRSRSTMAFLPEI-SGLPRQRITIHAADEADGKFADLSEAV-GSLPEALVYACGPESLMQAVAS

AMA--DESQLRIERFKAP--DIVPGPDFDVICQSTGQRIAVGPDVSVLEALNAAGINVPSSCAEGICGTC

ETGVIDGDVEHRDFLLSPAERNKSMFVCVSRCRSRDLILDL

>D7CDM4|D7CDM4_STRBB

MTSPTTERL-L-VKQARWVAHDVVELRLIRPAGGTLPSWEPGAHVELTLPSGLRRAYSLCGDVADTESWT

VAVHRVPDGRGGSREIHDTALVGRELKVRGPVNRFPLVPAEG-YLLLAGGIGITPLLPMARELTTRG-LP

WRLVLGARDRSRLAYTEEL-VALGGDRVLLVPQDEA-G-LPDLAAEL-AASPAHAVYACGPQPMLDAVTA

LCRADPVRTPRLERFAPADSGLPERSQFEVVLRRSGVRTTVPADRSVLDVVREKLPDVPYSCEEGYCGSC

ETSVTAGIPDHRDTVIDPSERATTMMICVSRSASPVLELDL

>Q0S1Y9|Q0S1Y9_RHOSR

-MTQQTNHL-L-VRQQRLESDGVLSLTLERPDGAALGEWEPGAHIDIVLPSGTVRQYSLCGDPTDKTAYR

IAVLREDEGRGGSKEIHDSVRVGDLLAVRGPRNHLALEPAP-HYLLIAGGIGVTPILSMARQLSREN-AS

WSALYGGRSRATMAFVEEFCG--LGE-VNVTPSDES-G-NLDLAAALANVPSGTAVYCCGPEGPLQEVKS

VCGVLGDEVIHFERFGAPVVGADPAAGFDVELRRTGCTLKVPADRTLLEVVLEANPDILYSCEDGFCGSC

ETRVLDGIPEHHDSILSQADRGETMMICVGRSRTPTLVLDA

>A0JVQ0|A0JVQ0_ARTS2

MTASAGATIDAVVDQVDIVADQVVSLVLRRADGQRFQPWQPGAHIDVHVGDGLVRQYSLCSSPDELDHLR

IGVLHVPDSRGGSKAVHA-LLAGTPLTISEPRNNFPMR-ESRRYLFIAGGIGITPLIPMLEAAQASG-KE

WTLIYGGRSRNTMAFAQQL-EDRYGDRIRIIAEDEV-G-RLDLDQIL-GMPAHMLVYACGPGGLLGAVEE

RCMGWPPGALHTERFVAST---LGAANFEVELARTGTTVTVPNDKTILEAVEEVGVRVLSSCRGGLCGTC

ETQIISGEPEHRDAVLSEGDRGEVMLVCVSRAAGCRLVLDL

>F1YK66|F1YK66_9ACTO

MMNLETGTVELVVREMRWEADGVIAVRLEDQGGAALPDWQPGAHIDIHLGKGIVRQYSLCGDADDKTAYR

IAVLRDPSTRGGSKYVHESLRPGHVVSVSEPRNNFELL-SSPKYVFIAGGIGITPMLPMIAQAERSG-AE

WELHYGGRSRDTMAFVDAL-R-AYGDRVSVVSEDRE-G-VLDLDALLGESRPDTLVYTCGPEGLLAAVEE

RGGGWADETIRLERFKPKTRAENEEEEISVHCESSGTTVEVAPGEAILDALETAGVDVPNSCREGMCGSC

ETRVLRGVPDHRDYVLSSAEQGETMMICVSRAKTDELVLDL

>Q0S9K5|Q0S9K5_RHOSR

-MTTDADTVSMMVRQMRVESSGVVSLRLERQDGGAVDRWTPGAHLDLHLNRGTIRQYSLCGDPDDKTGYR

VAILHE-TGRGGSRHVHETLRPGDSLSVSAPRNNFELLPSPR-YVFVAGGIGITPILAMIGEAERMG-AE

WELHYGGRARASMAFLDEL-S-AYGDRVHVVTEETD-G-VLDLAGILGTARVETLVYACGPEGLLGAVEG

YTDSWPDGAIRLERFKAKE-IPADTLDVRVICERSGISATVAPDRTILDTLEESGVDVPNSCREGICGTC

ETRVLAGTPDHRDSLLSSSEQGTTIMICVSRARSDELVLDL

>B2JRQ4|B2JRQ4_BURP8

--MSERDF-ELVIARKEAVATGVVRLTFARKDGLQLPTWEPGAHIDVHFSNGYVRQYSLCGKTADRRHWQ

VAVRRAVDGRGGSAHIHEVLAEGDTIRVSAPRNNFPLV-RAKRYLFVAGGIGITPILPMIAEVSAAG-AD

WRLVYCGRIAESMAFIDEVRA-LGGDKVSLHESELK-G-QLDLAALINEADSETVLYCCGPEPMLSSIDD

ICASWSAERVHYERFSPRD---DGSINFEVEFARSKLVLTVPPDRSILELAEEHGVDIDSSCQEGVCGSC

ETRVLEGIPDHRDSVLSQKERGQKIMVCVSRSCSSRLVLDA

>F6ER21|F6ER21_AMYSD

PSLHEFTT-ELTVAIRRQVADAVVALELVHSEGDDLPAWEPGAHVDLLLAEGLVRQYSLCGDPRSSDRWH

IGVLRDPNSRGGSQHVHEQLCEGATVTVRGPRNHFELV-DSPRYQFIAGGIGITPMMPMIAAADRAG-AD

WSLLYLGRSRTTMAFSDDL-LQMYGDKVTVWADDER-GAMFDLQGSL-QTPTETEVYCCGPGPLMDAVEK

LCSHWPEGSLHLERFAARKQTGEEAALYQVVCQRSGITVAVEPGISMLEALEDADVPILSSCLDGVCGTC

QTRVLEGTPDHRDSLLNAAERGSVILPCVSRSRTEKLVLDL

>B4VCE4|B4VCE4_9ACTO

DVLSPPPALRLRVQDTVREADGVLGLLLADPWGAPLPAWEPGAHLEVSLPSGRIRHYSLSGDPGDLRTYR

LGVLRDPGGRGGSEEIHTSIPAGSLLDVRGPVNRFPLVPAE-RYAFIAGGIGITPLLPMVREAAAGG-AA

WSLLYGGRTLTSMAYRDELAA-LGGGNLVLP-EDTH-G-RLDIDSLLRGLPDTAAVYCCGPAGLIAAVES

RCALLPARPPHVERFTAAP----DTAGFEVELRRSGAVLHVPPGRSLLDVVREAVPSVPSSCEQGICGTC

ETKVLGGAPDHRDSVLSEAEKGNTMMICVGRSRGGRLVLDL

>Q0S051|Q0S051_RHOSR

-MNGDNVICPLRVDRVVPESDGVVSLRLVHPDGQRLPDWTPGAHLDVLLPSGLLRQYSLCGDPQDDTGYR

IAVLREARGRGGSREIHDSALLGCTLGIRGPRNHFALS-TAPRYLFLAGGIGITPILAMAREVSRRG-VP

WRLVYGGRTRSTMAFVDELQNLPSG-QVELVPQDER-G-HIPLDDILAATPPDTHIYCCGPEPMIRAVEA

ACACSALDRLHVERFTAPSLAEQPPVVFEVELHRSGVVLRVPANRTLLEVVRDVVPGVMSSCEEGFCGAC

ETKVLEGVPEHHDSILSDAERCNTMMICVGRARSGLLVLDL

>D6AXZ5|D6AXZ5_9ACTO

SGRPRSRATTLRVVRRDTPADGVVRLRLEGT-GEL-PAWEPGAHLDLVLPSGTVRQYSLCGDPADRSAYT

VATRLIEGGRGGSLELHRELREGTEVTVRGPRNRFPLT-AAPAYVFVAGGIGITPLLPMVRAAQAAG-AD

WKLLYGGRTRASMPFADELEKLGGPERVTLA-EDES-G-RPDLAAALAGTPEGTTVYCCGPEGLMAAVEA

ALP--AGCLLRTERFAGGPTGRPSAADFEVELRRTGRTVTVPADRTALEAVREELPDAPYSCTQGFCGTC

RQRVVSGEVDHRDELLADSERDEAMLLCVSRSRGGRIVLDL

>Q13I77|Q13I77_BURXL

--MDKAQEINLVVRAITQEADGVIGLTLAAADGQDLPVWTPGAHIGLLVDGGLERQYSLCSEPEDRRQWR

IAVLREPESRGGSAWLHSRLQVGDILRATGPGNQFELV-DADRYVFVAGGIGITPLLPMIRAVEARG-AQ

WSLLYGGRHRSSMAFAAALEA--YGERVRIQPEDQC-G-LLDLKAALGAPQAGVAIYSCGPERLLEAVEA

TCVGWPEGALHVERFRPRP---GALESFEVVLAKSNLSCQVREGQTIIDALDTAGFHVPRSCGEGTCGTC

VAKVLEGVPDHRDSFLMGKKRNQTICVCCSRSLTPRLVLDL

>G2G5S7|G2G5S7_9ACTO

VHRDPPGSRPVVVTDTRRAADGVVTLDLAAPDGTPLPAWQPGAHVDLVLPSGRIRQYSLCGDPADRTTYR

IGVLHQPDGRGGSAEAHA-LEEDQPLAIKGPRNRFPLV-LADHYLFIAGGIGITPILPMIRAVAAAG-RE

WHLLYGGRSRATMAFTDEL-TALGGDRVRLVPQDTD-G-LPDLTTAL-AGTPGAAVYACGPEPLISAAEK

TVAGFPDRHLHIERFAAPGASSQATGAFHVELRRTGRTLPVPAERTLLDVIRQAVPDAPSSCEEGFCGSC

ELRVLAGTPDHRDTVLPPGERRDVVYPCVSRSRSPRLVVDL

>F0V3X5|F0V3X5_9ACTO

RVASEEQRLRLRVEALVREADGVLSAHLRRPDADDLPPWRPGAHLDLHLPGGPVRPYSLSGSPADRSTWR

ISVLKEPASRGGSRAVHESLRPGDVVDVTGPRNAFPLVPES-EYLFIAGGIGITPIMPMIEEIAASG-AR

WSLLYGGRSRSSMAFLPDL-A-RHGGRVTVRPQDEY-G-LLDLDTFLGPPRPDTLVYSCGPEPLLSAVEA

RCAAWPPGTLHLERFAARP---APSATFELVLHRTGRSITVAPGSTVLEALEENGVEPPNSCREGICGTC

ETKVVEGVPDHRDSLLTQEERNDTMMICVGRARCR-LVLDL

>F6EEN9|F6EEN9_AMYSD

GTYREVEL-DLQVRKRERVAEGVVVLTLADPAGADLPEWTPGAHIDLMMPT-LIRQYSLCGDTSNSAEWK

VGVLLDPASRGGSQFVHDKLHEGATVRVRGPRNHFPMVQAA-RYKFIAGGIGITPIYTMIAAAAATN-AD

WQLLYGGRTRSSMAFLDVLED--HGDRVSVCPQDET-G-MLDLAAELSAPDEDTVVYCCGPEGLLSAVEQ

ACTSWPTGSLHIERFAAKA--FEPSVDFEVECQRSGVSVTVPPDKTIYEAVEEAGVDVLGSCMEGVCGTC

ECDVIEGEPDPRDSVLSEAERGATIMICVSRSRSERLVLDL

>G4HS51|G4HS51_MYCRH

ASVHEFTA-DLTVLHRYVAANDVVVLTLAAPDGRELPRWQPGAHIDLVLGDGLIRQYSLCGDPLDSALWR

VGVLLEPHSRGGSRYVHDNLHEGSVVRVRGPRNHFPLA-DARRYRFIAGGIGITPILAMLETSHRVG-KD

WTLLYGGRSRDSMAFAADLVQ-RYPDRVTIWPQDEC-G-LLDLDSLLGDPEDGTAVYCCGPEALLTAVEQ

RCARWPGGSLHVERFAARTAATTDDTFFEVVCQRSGITLEVTPDQSILELIEEAGIPITTSCYEGVCGTC

EARVLDGVPDHRDSVLNDEQKGEVMLVCVSRSRSQRLVLDL

>Q0SJT1|Q0SJT1_RHOSR

NGSTNTDQRKMRVIGKDVLAHGVMAVRLADPDGGAVPAWEPGAHIDVIIDRSTVRQYSLCGDASDTTSLA

IAVLRE-DGRGGSRWVHDSLQVGDLIAIAGPRNNFTLEPAQ-SYLFVAGGIGITPLLPMLRSAEAAS-AD

WRLLYGGRIRTSMAFAEELVA-RYPGRIELRPQDEY-G-ILDLSAALDAVPDGTAVYCCGPEPLLQAIEN

EC-AHPNLSLYVERFAAKE---RPANDFAVELRRAGRTITVDANESLLDAVLRSDVDAPFSCREGTCGTC

ETRVLEGVPEHRDSILTEDEQNDCMMICVSRSCTAKLVLDL

>C1AZ91|C1AZ91_RHOOB

TSNANQQRRQMQVAGKDTLADGVVRLTLVDHDGATAPAWTPGSHIDLILDDTTTRQYSLCGDPGDTTSLT

VAVLRE-DGRGGSIRVHDKLESGDLVDVGGPRNHFALV-DADSYLFVAGGIGITPLLPMTREVDAKG-AP

WRLLYGGRTRASMAFADELAA-AHPDQVRIRPQDEHG--HLDLATALAEVEPGTVVYCCGPEPLLQAIES

ACRERPHVTLHVERFAPKE---PPEHDFEVELARSGRTVQVAADETILDAVLREGVDADFSCREGTCGTC

EVAVLRGTPDHRDSVLSEDEQNDAMMICVSRSCTPKLTLDL

>A1UD45|A1UD45_MYCSK

LVMREYEA-DVVVAGKEVIADGIAALTLKEVGGHPLPEWTPGSHIDLIMSSGLVRQYSLCGDPADRGTWR

IAVLREPDSRGGSVYVHDSLEHGAEIRIRGPRNHFQLAPSP-NYLFIAGGIGITPIRAMLAEAEAAG-AN

WKLFYGGRSTESMAFLDEL-A-PYGDRVEFWPQDQR-G-LLDLKYILGAPQADTLVYCCGPQGLLTAVEE

RCTSWPAGALRLERFSSST---VETENIEVELAQQGVTLTVPADQSVLEAIVAHGVDVMSSCQSGMCGTC

ETPVLSGVPEHRDDVLTYEERNDCMMICVSRSRGDKLVLDI

>E4NJK7|E4NJK7_KITSK

-MTAPEPTLALVVTGRRTEAEGVVSLTLARPDGGPLPGWTPGAHLDLLLGDGLERQYSLCGDPAETAAWT

VAVLREAGGRGGSVRVHDTLHPGAAVRARGPRNHFALRPAP-RYLFVAGGIGITPLVPMLAAAEAAG-AD

WRLLYTGRARAAMAFAGQLAD-RYGPRVLLHPRDET-G-RPDLAAHLDALDEAAEVYCCGPRPLLAAVER

HREGRRP--ARVERFQPRE---PAGADFTVLLARSGRTLTVPADASVLDTVRAAGVEVLYSCERGTCGTC

ETDVLDGRPDHRDALLTEAERSASMMVCVSRCLGERLVLDL

>D9WJE0|D9WJE0_9ACTO

QSTPGHAA-TLVVAARERVAEDVVSLTLARPDGGRLPNWTPGSHIDLVLPEGATRQYSLCGDRWDAHTYR

IAVLREPAGRGGSAYVHDRLRPGDRVGVGGPRNHFPLVPSE-KYFFIAGGIGITPLLPMVHQAELLG-AD

WQLLYGGRTRASMAFRQELTD-AYGERVHVVPQDEL-G-LPDLAALLGTPRPGTKVYCCGPAPLLAAVEA

ACAAWPPYALRFERFTAA-----AQTPFEVELRRTGHTVTVTPEVSVLEAVRRAGADVLSSCEQGTCGTC

LTPVLEGQPDHRDSVLTDQQRNDCMFVCVSRSCGDRLVLDL

>C1B9I6|C1B9I6_RHOOB

MPTAPEPVLELELTARTTGADGVVVLEFRDPSGAALPGWTPGAHIDLLLPE-LTRQYSLCGDPADTGTWR

VAVLRE-MGRGGSQFVHDKLSEGDTVTVRGPRNHFALEPSP-RYVFVAGGIGITPILTMAHAAENAG-AD

WTLVYGGRSRSSMAFADTLAE-RFDDRVVIHPQDEH-G-LIDLPGLLGNVRENTLVYCCGPAPLLDAVTA

HCDDWPSGTLHLERFAAKD----LEERFEVELTLSGKTVTVAPEQSILQAVTAAGVQVLSSCEEGTCGTC

ETPVLGGTVDHRDSLLTADEQNDTMMICVSRASCPRLTLEL

>Q0S930|Q0S930_RHOSR

MPIPPEPVRELELTSKTTGADGVVVLEFRDPSGAALPAWTPGAHIDLVLPE-LTRQYSLCGDPADTGTWR

VAVLRE-MGRGGSQFVHDKLSEGDHVTVRGPRNHFELEPSP-RYVFVAGGIGITPILTMAHAAENAG-AD

WTLVYGGRSRSSMAFADALAE-RFGDRVVVHPEDEH-G-LIDLPGLLGDVRENTLVYCCGPAPLLDAVTG

HCDSWPSGTLHIERFAAKD----LEERFEVELRLSGKTVTVAPEQSILQAVTAAGVQVLSSCEEGTCGTC

ETAVLGGTVDHRDSLLTADEQNDTMMICVSRASCPRLTLEL

>A4FDH3|A4FDH3_SACEN

MPSEPDHPLALVVEKKESLADCVVQLTLRGADGAELPDWTPGAHVDLVL--GLTRQYSLCGDPAERSVWQ

VAVLRE-AGRGGSAHVHDTLTEGDTVHARGPRNNFQLV-DASRYLFIAGGIGITPIVPMIARADAAG-AD

WRLVYGGRTRRSMAFLDRLEQ-RYRDRVGIRP-QDETG-LLDLPTLLAEPDDETAVYCCGPEPLLDAVAE

QCARWPQGCLHVERFTPKT---GATERFEIELARTGTTLTVPEDRSVLEVVEESGVPVLSSCQEGTCGTC

ETTVLAGVPDHRDSVLTAEEQNDTMMICVSRSCSARLVLDL

>D7CFA8|D7CFA8_STRBB

PSTPDGER-SLVVAERVESADDVASLILRHPGGELLPEWQPGAHIDLLLDDGLVRHYSLCGDPDDRSSWR

IGVLRVPDSRGGSAYVHGKLHPGTPVRVRGPRNNFALQ-RADRYLFVAGGIGITPLLPMIAAAETAG-AE

WRLLYGGRTRSSMAFLDEL-A-AYGEAVTVHPQDQH-G-LLDLDGYLGEPDTGTLVYCCGPEPLLAAAEE

RCGAWPEGALRVERFQAKE----QEEAFEVVLSRSGHTLHVPPGKTVLEAVQEAGVSVPYSCAEGICGTC

ETEVLTGEPDHRDSVLSAEERGETMMICVSRSRGPQLVLDL

>D7BU10|D7BU10_STRBB

GPEGEFTD-TLVVTGRADAASDVVVLTLRHPTGRELPAWQPGAHIDLLLDESLTRQYSLCGDPADRARWQ

IAVHRAPDGRGGSAYVHDALTEGAAVQVRGPRNRFPLRPAA-RYLFIAGGIGITPLVPMTAAAEAAG-AD

WTLLYGGRTRASMAFADQLAD-RYGPKVRLVPQDES-G-LLDLASFLAAPTPDALVYCCGPEPLLRAAEE

RCRAWPSGSLRTERFQPR-TDIPGPADFELVLARSGLTLTVPPERSVLRAVEAAGISVLYSCGEGTCGTC

ETDVVEGEVDHRDSVLTDEERGETMMICVSRCRGRRLVLDL

>D9WN85|D9WN85_9ACTO

HEVTEGHPYDL-VAARADATPDVVTLTLRHPAGHELPGWEPGAHVDLVLDDGLVRQYSLCGDPADAASWR

IAVLRVPDGRGGSARVHETLTEGARVRVRGPRNHFALRPAA-RYLFIAGGIGITPILPMTAAAEAAG-AD

WTLLYGGRTRASMAFADQL-ADAYGEKARLVPQDEA-G-LLDLAAYL-GEPPGTLVYCCGPEPLLRVAEE

RCRAWPAGALRTERFQPRPADESDETNFEVILDRSGRSLTVKPGQSVLRTLEEAGVPVLYSCEEGTCGTC

ETDVIEGEVDHRDSVLTDEERGETMMICVSRCRGPKLVLDL

>G4HXS6|G4HXS6_MYCRH

APPAWPGFRPLKVTAIHDESRYVRSVLLADPAGTGLPKWLAGQSIVVRVPAGQVRNYSLSNEPGSA-QYR

IGVKRE---PFGSGYLHDHVNVGDLIEVAAPRGSFFLDDNPCPVVLISAGVGVTPVLSMLHSVAASA-PV

WWL-HSARNGSEHTFAPEVGLLPSQSRIFYSPDRE--GYRGRLSESLLGLPVEADVYVCGPQPFMDSVTD

GLVGINARSIHAERFGAHAATTVDGPGPIVQFARSALSAPWRPGISLLEFAEACDVPTRWSCRTGVCHTC

ETALLSGRVRYDPEPLEPPAE-GNVLLCVATPSEG-IVVDL

>C0SMV8|C0SMV8_9ACTO

PPPAWPGFRRLEVARIEPESASIFSLTLA-ADGEPLPAALPGQFLTVRLPDPVIRNYSLSGRPGAET-YR

ISVKQE--PHGVSTYLRQHVKVGDVLDVAAPRGTFLLADADTPVLLLSAGVGATPVLAMLHALAARSRAV

WWL-HAARDGESHPFAGEARALSPAGRSFVSSRPSP-GVDYTFSGRFREPPPDADAYICGPSAFMGDMTG

ALEGLPSSRIHTEVFGATSGSAQAAPGPSVAFARSGLIVPWHPDGTLLGLAEACDVPVRWSCRTGICHTC

ETALLSGHVEYDPQPL---EPGGSALICCSTPPAD-VVLDM

>Q1M5R5|Q1M5R5_RHIL3

PPPAWRGFRPLRVSRKVRESGSVVSLVLE-PDGQPVAAALPGQFVVLRLGPSLMRSYSLSGEPSAT-RYR

VSVKRE--ADGASGYIDDELQVGDIVQASAARGSFTLRPGDTPVVLMSAGIGVTPVLAMLHVLAAEA-SV

WWL-YGTRNGREHPFAEEARGLALAHHHSCYSPDRPDDGHLDAQALL-NLPRDCDVYVCGPSSFMSDLSV

GLAGIAPDRIHTEMFGAGPSSRLAGPGPLVSFARTGLNVRWDQTQSLLELAEACDVPVRWSCRTGVCHTC

ETGLVEGSVGYRPDPV---DADGNVLICCAKPEGD-IVIDL
