# Supplementary material for: Accurate Simulation and Detection of Coevolution Signals in Multiple Sequence Alignments
Source: PLoS One. 2012 Oct 16;7(10):e47108. doi: 10.1371/journal.pone.0047108 (PMC3473043; doi:10.1371/journal.pone.0047108)
Supplement: MSA S6 — MSA of MDH. (DOCX) [file pone.0047108.s017.docx]

>B2W690|B2W690_PYRTR

YSTKPAQWEAAARAAVPLPNFLYVSGSASSESTYTANLSAFSRYRLRPWMLVQATRRDMSVTLFGQKYPS

PLLVAPIGVQEILHPDAEEATARACAAVKVPMILSTAATRSIEQVAAANGPDRWYQLYWPAEELTASLLS

RAKTSGYKVLVVTLDTFMIGWRPSDLDEAY-IP-FLWGQGCQLKRPGSLAG-AAKKAMFF-TSVLASGRD

WGDLQVLRKYWDGPIVLKGIQTLEDAQRAVECGMDGIVVSNHGGRQLDGAIASLDALAEIGDCIKSSILF

DSGIRTGSDVLKALALGAKAVLVGRPYAYGLAMGGEEGVKHVLNCMLADTDNSLANLGKRNLGEITREDL

RVM

>E3RSQ8|E3RSQ8_PYRTT

YSTKPAQWEAAARAAVPLPNFLYVNGSASSESTYTANISAFSRYRLRPWMLVQATRRDMSVTLFGQKYPS

PLLVAPIGVQEILHPDAEEATARACASLKIPMILSTAATRSIEQVAAANGPDRWYQLYWPAEELTASLLS

RAKQSGYKVLVVTLDTFLIGWRPSDLDEAY-IP-FFWGQGCQLKRPGSLAA-AKMKKAMFFTSVLASGYD

WNDLKVLRKYWDGPIVLKGIQTVEDAQRAVDYGMDGIVVSNHGGRQLDGAIASLDALAEIGDRIKSTILF

DSGIRTGSDVLKALALGAKAVLVGRPYVYGLAMGGEEGVKHVLNCMLADTDNSLANLGKKNVGEISREDL

RVM

>A1ZDK4|A1ZDK4_9BACT

PV-TPQLLEEKAARRMTKEATGYIIGGAGLGKTMQNNRSAFDQYQIVPRMLKDVSKRDTSITLFGQKFPS

PLLTAPVGVLEMVHKRADLAVAEATSSLGVPMIFSNQASYPMEACAS-LMGPRWFQLYWSSNELVLSFLK

RAEASGCSAIVVTLDTSVLGWRTQDLDLAF-LP-FLQGKGIAIKNALRMKKNYPKAVKRF-VNIYSRDST

WENLQFLRKHTQLPLLLKGILHPDDAQKAIDYGMDGIVVSNHGGRQVDGAIGSFAALPDIVQKVKDPVLL

DSGVRSGADMLKALAIGAKAVCVGRPYVYGLALAGAAGVQEVLANLMADFELNMALAGCKSVGELGRELL

KKP

>A3TPN2|A3TPN2_9MICO

VPTDPDELEARARKEMSAQAWAYVSGGAGAGATVRANRAAFDRWQIVPRMLHGNTTRDLRTEILGTPLAA

PVMLAPVGAAELVAPDSDLDIARGAAAAGTPYIFSTQGCSPMEETAKAMGDPWWYQLYWSDEGLVDSLIG

RAESAGARALVVTLDTTMLGWRTQDLDLGSLFSIQTSDPRFM--TLLNQSRRHPAAVETFLDIYSNPGLS

WAHIETLRERTRIPVLLKGILHPDDAQRAVDLGIDGIIVSNHGGRQVDRSIASLDALVGIRERIGRVVLL

DSGVRTGADVMIALALGADAALIGRPHIYGLALDGADGVRDVIDNLIAELDLTMGLTGAATIADITREAF

TTT

>D0RUC5|D0RUC5_9STRE

FI-NTYDLEPMAQQVIPKAAFGYIASGAEDTFTLRENIRAFNHKLIVPHTLCDVENPSTDIEFAGEKLSS

PIIMAPVAAHKLANEQGEVATARGVHEFGSLYTTSSYSTVDLPEITEALQGPHWFQFYFSDDGINRHIMD

RVKAEGYKAIVLTADATVGGNREVDKRNGFVFP-V-------MPIVEEYL--PEFVYKSA-KQRLSPR--

--DVEFIAAYSGLPVYVKGPQCREDVERSLAAGASGIWVTNHGGRQIDGGPAAFDSLQEVAEAVDKPIVF

DSGVRRGQHVFKALASGADLVAIGRPVIYGLALGGSVGVRQVFEHLNTELKTVMQLSGTQTIEDVKHFKL

RHN

>A9QH69|A9QH69_STRIN

FV-NVFDLEKMAQKVIPKGAFGYIASGAGDTFTLHENIRSFNHKLIVPHGLKGVENPSTEITFDGDKLAS

PIILAPVAAHKLANEQGEIASAKGVKEFGTIYTTSSYSTTDLPEISQTLGDPHWFQFYYSDDGINRHIMD

RLKAEGVKSIVLTVDATVGGNREVDKRNGFVFP-V-------MPIVQEYL--PNYVYKAT-KQALSPK--

--DVEYIAQYSGLPVYVKGPQCAEDAFRALEAGASGIWVTNHGGRQLDGGPAAFDSLQEVAEAVDRPIVF

DSGVRRGQHVFKALASGADLVALGRPVIYGLAMGGSVGTRQVFEKINDELKMVMQLAGTQTIDDVKHFKL

RHN

>C5WF04|C5WF04_STRDG

FI-NVFDLEKMAQQVIPKGAFGYIASGAGDTFTLHENIRSFNHKLIVPHGLKGVDNPSTEITFDGDRLSS

PIIMAPVAAHKLANEQGEVASAKGVSEFGTIYTTSSYSTTDLPEITTALNGPHWFQFYYSDDGINRHIMD

RLKEQGVKSIVLTVDATVGGNREVDKRNGFVFP-V-------MPIVQEYL--PDYVYKSA-KQALSPK--

--DVEYIAQYSGLPVYVKGPQCAEDTLRALDAGASGIWVTNHGGRQLDGGPAAFDSLQEVAETVDKPIVF

DSGVRRGQHIFKAIASGADLVALGRPVIYGLAMGGSIGTRQVFEKLNDELKMVMQLAGTQTVEDIRNFTL

RHN

>A2RG61|A2RG61_STRPG

FI-NVFDLEKMAQQVIPKGAFGYIASGAGDTFTLHENIRSFNHKLIVPHSLKGVENPSIEITFDGDHLTS

PLILAPVAAHKLANEQGEVASAKGLKEFGSIYTTSSYSTTDLPEISAALGGPHWFQFYYSDDGINRNIMD

RVKAQGCKAIVLTADATVGGNREVDRRNGFVFP--VGMPIVQ--YLPDGA----GKTMDYVYKSAKQALT

SKDIEYIATYSGLPVYVKGPQCAEDTLRALDAGASGIWVTNHGGRQLDGGPAAFDSLQEVAEAVDQPIVF

DSGIRRGQHIFKALASGADLVALGRPAIYGLAMGGSIGTRQVFEKLNDELKMVMQLAGTQTIQDVKAFNL

RHN

>B5XK09|B5XK09_STRPZ

FI-NVFDLEKMAQQVIPKGAFGYIASGAGDTFTLHENIRSFNHKLIVPHSLKGVENPSTEITFDGDHLTS

PLILAPVAAHKLANEQGEVASAKGLKEFGSIYTTSSYSTTDLPEISAALGGPHWFQFYYSDDGINRNIMD

RVKAQGCKAIVLTADATVGGNREVDRRNGFVFP-V-------MPIVQEYLP-DGGKTMDYVYKSAKQALT

SKDIEYIATYSGLPVYVKGPQCAEDTLRALDAGASGIWVTNHGGRQLDGGPAAFDSLQEVAEAVDQPIVF

DSGIRRGQHIFKALASGADLVALGRPAIYGLAMGGSTGTRQVFEKLNDELKMVMQLAGTQTIQDVKAFNL

RHN

>C2E451|C2E451_LACJH

MV-NLDELEHEAKYVMPEAAYYYVASGAENEWTWRNNTQAFNHFQIVPRALTGMQNPELNTEFLGKS---

PVMICPIACHGIANAEAEIDTAKGAKAAGALFAMSTYANKSVQEVQSAVGDPRFMQLYLSNWDFNKMVIE

ESVKAGFTGFFLTVDALVSGYREANLRTNFTYP--VPLAFFNGKGEGQSV----QMYASS-AQNIGPD--

--DIHKIKKIADVPVIVKGVECAEDAMLAVGAGADGIVVSNHGGREVDGAPATIDVLPEIAKAVNHPVIL

DGGVRRGSHVFKALALGADLVGIGRPFLYGLTLGGAQGVQSVIEQLNKELLIDMQLTVCKTIEDIKIDHI

SST

>D0R6B9|D0R6B9_LACJF

MV-NLDELENEAKYVIPEAAYYYIASGAENEWTWRNNTQAFNHFQIVPRALTGMQDPELNTEFLGMKLKT

PVMICPIACHGIANAEAEIDTAKGAKAAGALFGMSTYANKSVQDVQSAVGDPRFMQLYLSNWDFNKMVIE

ESVKAGFTGFFLTVDALVSGYREANLRTNFTYP--VPLAFFNGKGEGQSV----QMYASS-AQNIGPD--

--DIRKIKEIADVPVIVKGVECAEDAVLAIGAGADGIVVSNHGGREVDGAPATIDVLPEIAKAVDHPIIL

DGGVRRGSHVFKALALGADLVGIGRPFLYGLALGGAQGVQSVIDQLNKELLIDMQLTGCKTIEDIKIDHL

SST

>C4VMW0|C4VMW0_9LACO

MV-NVDELEDRVRKVMPEAAYYYIASGSENEWTWRNNTAAFNHFQIVPRSLTNMDNPSTETQFMGMDLKT

PIMICPIACHGIAHKDAEVATAQGAKAAGALFSSSTYANRSVEDIATATGDPKFFQLYLSDWDFNKMVFD

AVKSAGYKGIMLTVDALVSGYREANLRTNFTFP-V-------LDFFTRYVG-AEQMYANS-AQKIGPA--

--DVAKIKEMSGLPVFVKGVMNAEDAYMAIGAGADGIVVSNHGGREIDTAPATIDMLPEIAAAVNGPIIL

DSGVRRGSHVFKALALGADLVGIGRPFLYGLALGGAKGVESVINQINNEFKILMQLTGCKTVEDVKHADI

RQI

>C2EPE3|C2EPE3_9LACO

MI-NVDELEERAKKVMPEGAYYYIASGAENEWTWRANTSAFNHYQIVPRALTDMQDPQTDTQFMGMKLKT

PIMISPIACHGIAHKDAEVATQKGVAAAGALFSSSTYANKSVEDIAAVAPEPRFFQLYLSDWDFNKMVFD

AIKKADYKGIFLTVDALVSGYREANLRTKFTYP-V-------LDFFTRYLG-AKQMYAAS-AQKIGPE--

--DVARIKKESGLSVFVKGVMCAEDAYKAIGAGADGIYVTNHGGREIDGSPATIDVLPEIAKAVNHPIVF

DSGVRRGSHVFKALALGADLVGIGRPYLYGLALGGPKGVESVIDQLNTELKIDMQLTGCKTIEDIKISRI

SNT

>C2KG80|C2KG80_9LACO

MV-NVDELEERVKKVMPEGAYYYIASGAENEWTWRNNTAAFNHFQVVPRALTDMADPQTNTDFMGMHLKT

PIMIAPIACHGIAHKDAEVATQKGAAMAGALFSSSTYANKSVEEIAAAAPEPRFFQLYLSDWNFNQMVFD

AIKKAGYQGIFLTVDALVSGYREANLRTNFTYP-V-------LDFFKRYLG-AKQMYASS-AQKIGPE--

--DVERIKKESGLPVFVKGVMCAEDAYKAIGAGADGIYVTNHGGREVDGAPATIDVLPEIAQAVNHPIIF

DSGVRRGSHIFKALALGADIVGIGRPYLYGLALGGAHGVASVIEQLNAELKIDMQLTGCKTIDDVKLTHI

SNT

>B2IIZ7|B2IIZ7_BEII9

KVPRIEDLALITRRRVPKITLGYLESGTGEEVALRRNREALDRVLLVPHYLKSVGARSTQTRLFGRTYDL

PIGISPVGLANAIWPGIDKMLAEAARNANVPYGLSTVGTTRLETISEIAPEHLWFQLYVAKDEVTFDLLQ

RAHQAGVDVLLVTVDVPISSKRVRDIRNGFQLPLRPSPTMALMPRFETLEK-YGPSLAAYITDQLGPDL-

---MRRIRDAWPGKLVIKGIMSVGAAEEAAEIGADGIVVSNHGGRQFDAAPAAIEVLPEIAAAVGASVMM

DSGVRSGEDVLRAVSLGAEFVFSGRSFVYGAAAAGPAGAAHALQIFKDDILRGMAQLGITDLTQMRPSRI

EDA

>B5J7V5|B5J7V5_9RHOB

HP-SIEDLRQAARRRIPKFAFEYLDSATGRELGLKTNRDALDAIGFMPSVLCGRTKADLQTTLLGQKYDL

PFGIAPIGMSGMMWAGAERMLAQAAVAHNIPFSLSSVAVASPEDVAPHIGNNGWFQHYPVSAELRRTMLP

RIKAAGFHTLIITVDVPEESRRERQRRANLTVPPKADLRTIVMRFFDDYVP-QRRESFTH-AGALIRGID

WQYLQELRQEWDGHLVVKGVLRPEDAARMAAEGVDCIWVSNHSGRQFEAGPAVIEQLPKIREAVGPPLIY

DSGVAWGMDIMRALAKGADFVMVGRAFQFSVAAFGARGIDHLIHILKADIEANMSQLGVENINRLAEYLL

LND

>A3JUQ7|A3JUQ7_9RHOB

HP-SIEDLRHAARKRIPKFAFEYLDSATGRELGLKVNREALDAIGFMPSVLCGRTRANLQTTLMGQTYDL

PFGIAPVGMSGLMWAGAERMLAQAAVAHNIPFSLSSVAVASPEDVAPHIGNNGWFQHYPVSADLRRKMLP

RIKAAGFHTLIITVDVPEESRRERQRRANLTVPPKTDLRTLTMRFFDDYVP-TKRESFTH-AGALIRGID

WQYLRDLRQEWDGKLIVKGVLRPTDAKRIASEGVDCIWVSNHSGRQFEAGPAVIEQLPKIREAVGSPLIY

DSGVVWGLDVMRALAKGADYVMVGRAFQYAVAAFGARGIDHLVHILKSDITANMSQLGVEDINQLSDYLL

KSD

>A4EQ71|A4EQ71_9RHOB

HP-SIEDLRRAAQKRIPKFAFEYLDSATGRELGLKVNRDALDAVGFMPSVLCGRTKANLQTNLLGQCYDL

PFGIAPVGMSGLMWAGAERMLAQAAVAHNIPFSLSSVAVASPEDVSPYIGQNGWFQHYPVSADLRRKMLP

RIKAAGFHTLIITVDVPEESRRERQRRANLTVPPKTDLRTLTMRFFDDYVP-QRRESFTH-AGALIRGID

WRYLQELRGEWDGHLIVKGVLRPEDAQRMVDLGADCIWVSNHSGRQFEAGPAVIDQLPKIREAVGPPLIY

DSGIAWGLDIMRALAKGANFVMVGRAFQYAVAAFGAKGIDHLVHVLKADVAANMSQLGVEQLGQLSQYLL

-KD

>A8LSL9|A8LSL9_DINSH

HP-AISDLRRAARRRIPGFVFEYLDSATGDEIGVQTTRAALDAIHLLPGILHGQITPELETPLLGQTYAR

PFGIAPVGMSGLIWPDAERLLAAEAATARIPYGLSTVATQTPERVGP-VAGEMWFQLYPPDPGIRDDIMA

RARASGFGTLVLTVDVPADSRRERQRRANLTIPPKITPRMIFTPSLKLAES-YVASYMAHAGKAIRGAPD

WAYLDAVRAGWDGPLVVKGVLRPEDAVRLRAAGVDAIWVSDHSARQFEGGPGAITQLPAIRRAVGPPVIY

DSGIEGGLDILRAVGLGADFVMLGRAWHFALAGLGPAGVRHLIHILTQDLVTNMQICGIAKLADFRDQLA

THS

>A3JT03|A3JT03_9RHOB

FP-ALSDVMIRAKRRVPHFVWEFLDSATGAETTKTRNTDALDRILFAPAVLKGDLDPTLKTTFLDHEYDL

PFGIAPVGMSGLICPGAEQMLARHGADANIPYCLSTVASQLPEDVGP-LAGSNWFQLYPPDGDMRREILT

RVRAAGFHTLVLTLDIPAPSRRERQLRADLTQPLKFTPRLIRIPRLKLVQE-YELPSNNHIGYRLRCNPD

LAYLKQLQTEWDGPIIAKGVMDPDAAVVLASAGVDAIWVSNHGGRQFDAAPASISVLPDIRTAVGPPIIF

DGGIRTGLDVLRAFAHGANFAMLGRAHHYGLAAFGEKGAAHVSHILSEDMKSAMAQMGINSPSDAVNSLV

QKG

>A3JZN5|A3JZN5_9RHOB

YM-TVADLRERARRRVPRFAWAYLDTATGTGATPRRNRAALDRIGFVPSILDGEVTPDLATELLGVPHPL

PFGVAPVGQSGLLWPDAEAMLARGAAQAGLPYTLSTVATATPEEVGPLTGGQGWFQLYPPAVDVRLDMLA

RAQDSGFRVLVLTVDVPVASRREAQVKGGLVQPPRLTPRIAAMPRMKTLDR-YTRDPTAHVGYLLRTAPD

WDYVTWIRDHWDGPLVVKGVMRASDAARLEAAGVDAIWVSNHGGRQFDAAPAVAEVLPEVRAAT--PVIA

DSGFDSGLDMLRGIALGADFIMMGRAWHYAVCALGEDGPAHLTEMLRRDLVAAIGQLGVARPTDLRGRTE

TLS

>B5IXG2|B5IXG2_9RHOB

YP-ALADLKSHARRRIPHFVWEYLDSATGSESVTRRNRDALDRVLFRPAALRGEIVPRLETTLLGRTYPV

PYGIAPLGMSGLVWPDAERHLARHGARAGIPYALSTVATKTPEDMRG-AHGDQWFQMYPPDADVRRDMLK

RAKDVGFHTLILTVDVPAASRRERQTRGGLVQPPRLTPRLAAMPRMRLMDS-YALPSTAHVGYLLRAAPD

WEYVRHLRDEWDGPIVLKGVCEPEVAAKAQNEGVDAVWVSNHAGRQFDATPASIDLLPDIRAAT--PVIF

DSGIEGGLDILRALALGADFVFMGRAWHYALGALGANGPAHLHDILAKDMMSNMAQIGATSIEDLSARLV

HKH

>B6B421|B6B421_9RHOB

YP-ALADLHRAAKRRIPHFVWEYLDSATGLEASKARNRTKLDEVLMMPSVLHGEITPDLSVDFLGHTLPL

PFGIAPLGMSGLIWPNAETILASKAATLDIPYTLSTVATRTPEDIAPSLGQHGWFQLYPPDEGIRRDMLE

RVKANGFHTLVLTVDVPVASRRERQIRGGLRQPPKITPRLLAMPRMRTLDK-YIRSSTAHIGYLLRTSPD

WEYVHWLRDNWDGKLILKGVLDVRDVTKSEATGIDALWLSNHAGRQFDAAPAPIEVLPKIRAAT--PLIM

DSGIEGGLDILRAYALGADFVMLGRAWHYALGALGEQGPAHLADMLASDLRANMGQLGLTQLHDAPQTIL

PQL

>D0CW50|D0CW50_9RHOB

YP-GIEDLKRRARRRLPRFVWDYVDSGTGAETALKRNRAALDRIGFLPAILKGPLEFDTSTRFLGRDHPL

PFGMAPIGMCGLVWPGAEALMASAAAQAGIPYVLSTVASQSPEDMAPHIGPDAWFQLYPPDPEIRADLLN

RARSAGFGTLVLTVDVPVASRRERQTRSGLTHPPRLTPRLLAKPRMRTLDK-YITAHIGY-LLRTSPGMD

Y--VKWLRDHWQGPFIIKGVLRPEDAERLKQAGVDALWVSNHGGRQFDGAPASVDMLPAIRAAT--PLIF

DSGVSGGLDILRARALGADLIMLGRAFLYGVAALGARGPAHVIDILRQDMLANMGQLGAATLKDLPDVNA

NID

>C7D8R2|C7D8R2_9RHOB

HP-AIADLKARARRRIPHFVWEYLDSATGDERTLARNRSRLDQVRFLPSILHGEFEPDLRTTLLGRDYTV

PFGIAPVGMSGLIWPGAEQMLARTAARHGMPYTLSTVASQLPEDVGGHAGDNGWFQIYPPDRDIRLDILR

RAREAGFHTLVLTVDVPVASRRERQVRGGLTQPPRLTPRLAMMPRLRLMES-YAPSNQHI-GYLLRTSPD

WSYLRALRDEWDGPLIVKGVMNPDDCERLAKDGADAIWISNHAGRQFDAAPATIEQLPAIRAAT--PVIM

DSGVETGLDVLRALALGADFVMLGRAWHYGLGALGEAGAAHVMDILAKDMAANMGQIGARDLSDLSGKIV

-EK

>A3V6N5|A3V6N5_9RHOB

YP-AISDLKTRARGRIPHFVWEYLDSATGVEATQRRNRTALDQVLLNPSILHGEFAPDLSTTLLGRDHPL

PIGIAPLGMSGLIWPGAEQMLARMAARENIPYTLSTVASQLPEDVGPHAGDQGWFQLYPPDPVIRDDILN

RAKSAGFHTLVLTVDVPVASRRERQTRGGLTQPPKLTPRLALMPRLRLMES-YSLPSNQHIGYLLRTSPD

WDYFKALRDAWDGPLVVKGVGRADDAARLTDEGADAIWVSTHAGRQFDGGPASIETLPAIRAAT-PPVIF

DSGIEGGLDVLRALALGADFVMLGRAFHYGLAAMGEAGAAHVLDILRQDMISNMGQLGARSLKDL-PACL

RQT

>A4EJL5|A4EJL5_9RHOB

YP-AIADLKARARRRIPHFVWEYLDSATGVEATQRRNRDQLDQVLLNPSILHGEFDPDLSTTLLGQTHPL

PIGIAPVGMSGLIWPGAEQMLARTAARENIPFTLSTVASQLPEDVGPHAGAHAWFQLYPPDPGIRDDILK

RAKDSGFHTLVLTVDVPVASRRERQTRGGLTQPPRLTPRLAMMPRLRLMES-YSNEHVGY---LLRTSPD

WDYFKSLRDVWDGPLIVKGIGNADDAARLTDEGADAIWVSNHAGRQFDGGPATIETLPLVRAAT--PVIF

DSGVEGGLDVLRAIALGADFVMLGRAFHYGLAALGEPGAAHVLDILRKDMISNMGQIGARKLADL-PGCL

RR-

>A3VXP9|A3VXP9_9RHOB

YP-ALSDLRARAARRIPHFVWEYLDSATGTEATKARNRLMLDQVRLSPSILHGEFTPDITAPLLGQIHAL

PVGIAPVGMSGLIWPDAERLLARAATQVGIPYGLSTVASQKPEDIGPHLNGNGWFQMYPPDTGIRTDMLN

RARNAGFSVLVLTVDVPVGSRRERQVRSGLTQPPKLTPRLLAMPRMRLIDD-YAPSNKHA-GYLLRTSPD

WDYLRWLRDAWDGPLVVKGVLRGDDAAALEKAGVDAIWISNHAGRQFDAAPATIEALPEVRAAT--PVIM

DGGIEGGLDVLRAIALGADFVMLGRGWHYALGALGEIGPAHLADILAEDLRANMGQLGTRTLWDVRSRLI

PNG

>A6FQL7|A6FQL7_9RHOB

YP-ALSDLRARARGRIPRFVWEYLDSGTGAEATKARNRTKLDEVILMPSILHGEVKPDLSVELMGRTLPL

PFGIAPVGMSGMIWPGAEPMLARAAARAGIPYCLSTVATQTPADLSRDLGEDAWFQMYPPDPDIRTDMLQ

KARDAGFGTLILTVDVPVPSRRERQVRSGLTTPPKLTPRLMAMPRMKLIDE-YAPSNKHA-GYLLRTAPD

WDYLRWLRDAWSGPMIVKGVLDADDAGALEAAGVDAVWISNHAGRQFDGAPATIERLPAIRAAT-GPVII

DGGFEGGLDILRAIALGADHVMFGRAWHYALGALGADGPAHLVEILKLDLEANMGQLGLTTLTEVRNRVI

-SH

>A3SQ25|A3SQ25_9RHOB

YP-AIADLKARARRRLPRFVWEYLDAGTGTEATCRRNEEVFAGLRLMPSLLHGEQTPDLTTRLLGQAYQM

PVGIAPVGMSGLIWPDAEGHLARAATAAGLPYTLSTVASQTPEAVAPHLAGNGWFQLYPPDPEIRQDMLR

RARAAGFTTLVLTVDVPVASRRERQLRSGLTQPPRLSPRLLAMPRMALIDD-YSSSTAHA-GYLLRTSPD

WDYLSWLRDAWQGPLVVKGVLDPDTVPRLMAAGVDALWLSNHGGRQFDAAPAPLEVLPAIRAAT--PLIV

DSGISGGLDILRALALGADFTMLGRAWHFALAALGAQGPAHLARILRLDLESNMGQLGLIRPSEAPDRQM

KIT

>A4EY31|A4EY31_9RHOB

FP-ALSDLRKQARRRLPPFVWEYLDSGTGSEATKGRNRAVLASIGFLPSILHGPQECDLSVDLLGKPCAL

PFGFAPIGMSGLVWPNAEARLAKCAAQEQIPYCLSTVASQSPEDLAPHLGENAWFQLYPPDEGIRRDMLE

RARDAGFGTLILTVDVPVASRRERQTRSGLTQPPRLTPRLLAMPHMRTLDK-YVRSSTAHIGYLLRTSPN

WNYVKWLRENWQGKLVIKGVMRPEDASRLQELGVDALWVSNHAGRQFDASPASIEMLPAIRKAC-APLIF

DSGAETGLDILRALALGADFVMLGRAPHFALAALGDQGLTHLCDILRKDLEANMGQLGLIKPTEI--RKV

EMR

>C9CW05|C9CW05_9RHOB

FP-GLDDLRAKARARLPHFVWEYLDSATGREATQARNRRCLDRIGLMPAILGGPQEVDLSTTLFGTRLPR

PFGIAPVGMSGLIWPDAEGHLARHAAAAQIPYGLSTVASQSPEDLAPHLGEQGWFQMYPPDEGIRKDMLA

RARAAGFKVLVLTVDVPVASRRERQVRSGLTQPPRLTPRLLAMPHMRTLDK-YILSSTAHIGYLLRTAPD

WDYLQWLRDHWEGPLVVKGVLDARDAPRLEAAGVDAIWISNHAGRQFDAAPAPIEVLEEMRAAT--PLIL

DSGIEGGLDIVRAMALGADFVMLGRAWHYALAALGAAGPAHLHDILSKDLSANMGQLGISTLAEL--RDL

KRL

>A3X8V0|A3X8V0_9RHOB

YP-SLADLRARAQRRLPKFVWEYLDSATGNEATKRRNRSALDQIGFLPSILHGPQQVDLSTSFLGRDLPL

PFGIAPLGMSGLIWPDAEGRLARFGARSGIPYSLSTVASQSPEDLAPHLGAEAWFQLYPPDEDIRRDMLE

RARKAGFKTLVLTVDVPVASRRERQVRSGLTQPPRLTPRLLAMPHMRTLDK-YISSTAHI-GYLLRTSPD

WDYAKWLRDNWQGSFVIKGVMRAEDAAPLEQIGVDALWVSNHAGRQFDAAPASTEVLPELRAAT-SPLVF

DSGIEGGLDILRALALGADFIMLGRAFHFALAALGDRGPNHLVEILSKDLEANMGQLGLRSLSEVRTLTL

HSQ

>B7QW22|B7QW22_9RHOB

YP-ALSDLRQRARRRLPRFVWEYLDSGTGTEATKARNRMALDQVGFLPSILHGPQKPDLSRRFLGVDRPL

PFGIAPVGMSGLVWPDAEGHLARAAAAHGLPYCLSTVASQSPEDVAPHLGASPWFQLYPPDPGIRRDMLA

RAKKAGFTGLVLTVDVPVASRRERQTRSGLTQPPRLTPRLLAMPHMRTLDK-YVLSSTAHVGYLLRTSPD

WDYVRWLRDHWQGSLIIKGVMRAEDAAPLETIGVDALWISNHAGRQFDAAPAAIEVLPDLRAAT--PLIF

DSGIEGGLDILRALALGADFVMLGRAFHFALAALGPKGVDHLIDILRKDMSANMGQLGAATLDALP-IPR

SLA

>A9EPG4|A9EPG4_9RHOB

YP-ALIDLRQRARRRLPRFVWEYLDSGTGTEATKARNRAALDQLGFAPSILHGPQTPDLSRRFLGIDRPL

PFGVAPVGMSGLIWPDAERLLARCAAAQGLPYCLSTVASQSPEDLAGDLGAP-WFQLYPPDPDMRRDLLA

RAKAAGFAGLVLTVDVPVASRRERQTRSGLTQPPRLTPRLLALPHMRTLDK-YVLSSTAHVGYLLRTSPD

WDYVKWLRDHWDGPLIIKGVMRAEDAAPLEAIGADALWVSNHAGRQFDAAPSTIEALSGIRAAT--PLIF

DSGIESGLDILRALALGADYVMLGRAFHFALAALGSRGPDHLVDILRKDLDANMGQLGLETLSALPRSFA

RSD

>B7RPA6|B7RPA6_9RHOB

YP-ALSDLRQRAKQRLPKFVWDYLDSATGGAATKRLNRAALDRIGFMPSILHGEFVPDLSTELFGHRFPL

PFGIAPVGMSGLIWPDAEAHLARTAARVGLPYSLSTVASQNPEDLSPHLGENAWFQLYPPDPEIRTDMLN

RARGAGFKGLILTVDVPVASRRERLTRSGLTNPPRLTPRLMAMPNMRMLDK-YKSSTAHV-GYLLRTSPD

WDYVKWLRDAWDGPFLIKGVLRPDDATQLEQIGADAIWVSNHAGRQFDAAPATIDMLPGIRAAT-GPVIF

DSGIENGIDILRALALGADFVMLGQAFHLALAALGPKGIDHLIDILAKDLTANMGQLGARTLRDL-PPTL

PLN

>A9E2V1|A9E2V1_9RHOB

YP-ALSDLRSRAQSRLPKFVWEYLDSATGTEATKHRNRAALDRVGLMPSVLHGEFSPDLGVELMGQKLPL

PFGMSPLGMSGLIWPDAEAHLARAADRAGIPFGLSTVAAASPEDVAPHLGKHGWFQLYPPDPEIRTDMLA

RAKAAGFTTLVLTVDVPVASRRERQTRSGLTSPPKLTPRLMALPHMKMLDK-YTLPPTAHVGYLLRTAPD

WDYLHWLRDHWDGPLVIKGVLRPEDATALEQAGADAIWVSNHAGRQFDAAPASAEALPAIRAAT--PVIF

DSGVETGLDILRAFALGADFVMLGRAFHIALAALGPRGVDHLIDLLARDLTANMGQLGAHNLRELTPFDL

SPL

>B2T8Q3|B2T8Q3_BURPP

GKATIRDFVEPARKHLPAFAFNTLAGGAGNDAGVAENEAAFGRRFFVSRRFAPAST-DQTATVFGHSYAS

PFGVAPMGLANLFYPGADLLLAQAAQAGNFPFVLSTAASTSIERITKVAPDVSWYQLYLLDDRLNAELLS

RVAGCGVAVLVLTVDVPVAGRRNSAIRDGVTLPLRWTSALLAAPKLENYAP-HAGAASRHIASVMKMGLE

WDDLKKVRAMWPGKLVIKGILHPDDAARSVALGADGIWVSNHGGRQLEGAIASLDALSEIRRAVGRAVFL

DGGVRTGEDILKACALGAGLCFSGRSFAFPVAAYGERGVRAAVEILKEEIRVGLAQLGVQSLSALTSDSL

SNV

>A4AG23|A4AG23_9ACTN

PFSNLAEAEKLAKKRLPKAVWLAFKAGNEQGWTLDENIRAYNDLGFSPTIFDRPSRGDTKTNILGVDIDF

PVIVSPVGAQAI-HPDAEVAAARAAKRMGTAIGLSSYASSSIKDVTAANDKSF-FQLYWVTRDRIEARVE

EARRSGAKALIVTLDWSFTPRRDWGVPP---APPKMDLKTMMELKVPNLFI-TPTAWNEF---VHTPTPT

WEDVKWLRELWGGPFMIKGISTIKDAKLAVDMGADAISVSNHGGNNIDGTPSPIRFLPSIVDAVGSDVMV

DGGIRRGSDVVKAMALGAKAVFIGRAYLYGLAVSGEDGVHKVLEIMRDGIDETMFGIGRDSIHDLSMDDL

---

>B9L4Y0|B9L4Y0_THERP

WFESVAEAQRRARKRLPRSVYDAIIAGSERGVTLADNVAAFSELGLRPRVAAAPAQRELATTVLGFPIPF

PVLISPTGVQAV-HPDAEVAVARAAASAGTIMILSSFASKPLEEVVAA---PLFFQIYWLTRDRLRALLE

RAKAAGVKGIVVTLDWSFATRRDWESPF---IPDRLTLRNMINLTVPNLAL-PGEAYFEW---MHTPPPT

WTDLAWLRKQWDGPFLLKGILHPEDARRAVALGADAISVSNHGGNNLDGAPASIRVLPVIVEAVGGEILL

DGGIRRGSDVVKALALGARAVLIGRAYLWGLAANGEAGVRNVLELLRSGIDETLLGIGKASVHDLGPDDL

PPN

>D1C8X6|D1C8X6_SPHTD

WFESVAEAQRRAKKRLPRSVYYALIAGSEQGITLNDNVTAFAELGLRPQLADRPNTRDLTTTVLGEEISF

PVIISPTGVQAV-HPDAEVAVARASAAAGTIMGLSSFASKPIEEVVAANPRTF-FQIYWLSRDDMLHYLD

RAKRAGAKGLIVTLDWSFDTRRDWGSPW---IPERLNLEALLDLTVPNLAL-PGGAYATW---MQTPLPT

WEDIAWLRKQWDGPFIIKGVMLPEDARRAVEIGADAISVSNHGGNTLDGTPASIRALPAIVEAVGDEVLL

DGGIRRGSDVVKALALGARAVMIGRAYLWGLAANGEAGVRNVLDILRNGIDTTLIGIGRASVRDLDPDVL

PPD

>D0L326|D0L326_GORB4

WFETVVEAQRRARKRLPRSVYSSLVAGTQAGITVNDNMNAYGELGWAPHVVGAQPDRELSTSVMGQEISF

PVMISPTGVQAVDP-DGEVAVARAAAARGTAMGLSSFASHPVEEVTE-VNDKVFFQIYWLSRDDILARAM

RAKEAGAKGLIVTTDWVFNVGRDWGSPE---IPEKVDMRALLDLTAPNLPA-KGGAYGEW---MNTAPPT

WEDLQWLREQWGGPFMVKGITRVDDAKRAVDIGATALSVSNHGGNNLDGTPAAIRLLPAIADAVGNEVLL

DGGIRRGSDVAKALALGARAVMIGRAYLWGLAANGQTGVENVLDLLRMGLDGVVMGLGHKSVHELSRDDL

PAD

>A8LEE1|A8LEE1_FRASN

WFESVAEAQRRARRRLPKSVYSALLAGSEAGVSYRDNTAAFDELGFAPHVAGLSPKRDQQTTVLGQPISL

PVIISPTGVQAV-HPDGEVAVARAAAARGTAMGLSSFASKPIEQVIAANPQTF-YQTYWMTRDWMIRRLE

HAHRAGAVGLIVTLDWSFSHSRDWGSPW---IPERLDLKTMVDLSVPNLAE-PGGAYGEW---MQTPPPS

WEDIAWLRQQWDGPFLLKGVSRVDDARRARDAGVSAISVSNHGGNNLDSTPAPIRALAAVVDAVGTEVLM

DGGIRRGGDVVKALALGARAVMIGRAYLWGLAAGGQAGVENVLDVLRNGIDSALLSLGHSSIHELTPDDV

PDG

>D3D1Y4|D3D1Y4_9ACTO

WFESVAEAQRRAQRRLPKSVYSALLAGSEAGVTYRDNTGAFDELGFAPHVAGLKEKRELATTVLGQEISL

PVIISPTGVQAV-HPDGEVAVARAAAARGTAMGLSSFASKPLADVVAANPRTF-FQIYWMTRESMVRRLE

RAREGGAVGLIVTLDWSFSHSRDWGSPW---IPERLDLLTMVDLSVPNLAE-PGGAYGEW---MQTPPPS

WDDIAWLRQQWDGPFMLKGVSRVDDALRARDAGVSAISVSNHGGNNLDSTPAPIRALRAVVEAVGGEVVM

DSGIRRGGDVVKALALGARAVMIGRAYLWALAANGQAGVENVLDVLRNGIDSALLALGHSSIHDLTPDDV

PSG

>D1A2X8|D1A2X8_THECD

WFETVAEAQRRAKKRLPYMVYGALLAGSERGRTVQDNTDAFGELGFAPRVVGHHAQRDLSTTVMGVPTSM

PVVISPTGVQAV-HPDGEVAVARAAANRGVIMGLSSFASKPIEQVVEANPNVF-FQMYWSDRDAILQRME

RARNAGAKGLIVTLDWSFSMGRDWGSPT---IPEKLDVKTMVDLTVPNLQP-PGGAYGEW---MQTPPPT

WDDVKWLREEWGGPFMLKGVTRVDDAKRAVDIGVTALSVSNHGGNNLDTTPATIRLLPAIAEAVGDEVLL

DGGVRRGGDVAKALALGARAVLIGRAYLWGLAANGQAGVENVLDILRSGLDSAVLGLGRSSVHELAPDDL

PPG

>B1MGG3|B1MGG3_MYCA9

WFETVAIAQQRAKKRLPKSVYSSLISASEKGLTVSDNVEAFGELGFEPHVVGIQPDRELSTTVLGQDISL

PVMISPTGVQAVDP-DGEVAVARAAAARGTAMGLSSFASKPIEDVVAANPKTH-FQIYWLGRDDVAQRIQ

RAKDAGAVGLIATLDWSFSHGRDWGSPA---IPEKMDLRSMIDLRVPNQAA-RGDAYGQW---MGTPAPT

WDDVRWMREQWDGPFMLKGVMRIDDAKRAVDCGVSAISVSNHGGNNLDGTPASIRALPGIADAVGHEVLL

DGGIRRGSDVVKALALGARAVMIGRAYLWGLAASGQAGVENVLDIMRGGIDSALMGLGKKSVHELSPDDL

PEG

>A0QL24|A0QL24_MYCA1

WFETVAIAQQRAKRRLPKSVYAALIAASEKGITVSDNVEAFGELGFAPHVVGAPAKRELATTVMGQEISM

PVLISPTGVQAVDP-DGEVAVARAAAARGTAMGLSSFASKPIEEVIAVNPKVF-FQVYWLGRDAIAERVE

RARQAGAVGLIVTTDWSFSHGRDWGSPK---IPEEMNLRTILELRVPNQGR-RGAAYGEW---MGTPPPT

WEDIAWLREVWGGPFMLKGVMRVDDAKRAVDAGVSAISVSNHGGNNLDGTPASIRALPAIAEAVGDEVLL

DGGVRRGSDVVKAVALGARAVMIGRAYLWGLAAAGQAGVENVLDILRGGIDSALMGLGHSSVHDLGPSDI

PPG

>A0PM50|A0PM50_MYCUA

WFETVAIAQQRAKRRLPKSVYSSLISASEKGITVADNVAAFSELGFAPHVIGAAEKRDMSTTVMGQDISM

PVLISPTGVQAV-HPDGEVAVARAAAARGTAMGLSSFASKTIEDVIAANPKIF-FQIYWLGRDAIAERVE

RARQAGAVGLIVTTDWTFSHGRDWGSPK---IPEQMNLRTILDLRVPNQGR-RGAAYGEW---MGTPPPT

WDDIAWLRELWGGPFMLKGVMRVDDAKRAVDAGVSAISVSNHGGNNLDGTPASIRALPAVAAAVGDEVLL

DGGIRRGSDVVKAVALGARAVLVGRAYLWGLAANGQAGVENVLDILRGGIDSALMGLGHSSIHDLRSDDI

PAD

>A1KGH4|A1KGH4_MYCBP

WFETVAIAQQRAKRRLPKSVYSSLIAASEKGITVADNVAAFSELGFAPHVIGATDKRDLSTTVMGQEVSL

PVIISPTGVQAVDP-GGEVAVARAAAARGTVMGLSSFASKPIEEVIAANPKTF-FQVYWQGRDALAERVE

RARQAGAVGLVVTTDWTFSHGRDWGSPK---IPEEMNLKTILDLRVPNQGR-RGAAYGEW---MATPPPT

WEDIGWLRELWGGPFMLKGVMRVDDAKRAVDAGVSAISVSNHGGNNLDGTPASIRALPAVSAAVGDEVLL

DGGIRRGSDVVKAVALGARAVMIGRAYLWGLAANGQAGVENVLDILRGGIDSALMGLGHASVHDLSPADI

PTG

>A1UBM5|A1UBM5_MYCSK

WFETVAIAQQRAKKRLPKSVYSSLISASEKGVTVSDNVESFSELGFAPHVVGAPEKRDLSTTVMGQEISM

PVMISPTGVQAVDP-DGEVAVARAAAARGTAMGLSSFASKPIEEVIAANPKLF-FQIYWLGREAILERAQ

RAKEAGAVGLIATTDWSFSHGRDWGSPK---IPEQMNLKTMLDLRVPNQAR-RGQAYGEW---MGTPPPT

WEDIAWLRERWDGPFMLKGIVRVDDAKRAVDAGVSAISVSNHGGNNLDGTPAAIRCLPAIADAVGQEVLL

DGGIRRGSDVVKALALGARAVMIGRAYLWGLAANGQAGVENVLDILRGGIDSALMGLGRASIHDLGPDDI

PDG

>A0QSB9|A0QSB9_MYCS2

WFETVAIAQQRARKRLPKSVYSSLISASEKGVTVTDNVESFAELGFAPHVVGAPEKRDMATTVMGQQISL

PVIISPTGVQAV-HPDGEVAVARAAAARGTAMGLSSFASKPIEEVVA-VNDKIFFQIYWLDRDAILARAE

RAKAAGAVGLIVTTDWSFSHGRDWGSPK---IPEKMDLKTMVNLRVPNQGA-RGQAYGEW---MGTPPPT

WEDIAWLREQWDGPFMLKGVIRVDDAKRAVDAGVSAISVSNHGGNNLDGTPAAIRALPVIAEAVGDEVLL

DGGIRRGSDVVKAVALGARAVMIGRAYLWGLAAEGQVGVENVLDILRGGIDSALMGLGRSSIHDLVPEDI

PEG

>A1T4N1|A1T4N1_MYCVP

WFETVAIAQQRAKKRLPKSAYSSLISASEKGVTVTDNVESFAELGFAPHVIGATEKRDMATSVLGQDISL

PVIISPTGVQAIDP-DGEVAVARAAAARGTAMGLSSFASKPMEDVTA-VNDKIFFQIYWLSRDDILARME

RARAAGAKGLILTTDWSFAHGRDWGSPK---IPERMDLKTMIDLRVPNQGR-RGEAYGQW---MGTPPPT

WEDVAWLREQWGGPFLLKGTVRVDDAKRAVDAGVSAITVSNHGGNNLDGTPAAIRCLPAIADAVGDEVLL

DGGIRRGSDVVKAVALGARAVMIGRAYLWGLAANGQAGVENVLDILRGGIDSALMGLGKSSIHELTREDI

PDG

>A4T1S5|A4T1S5_MYCGI

WFETVAIAQQRAKKRLPKSAYSSLISASEKGVSVSDNVEAFAELGFAPHVVGATDKRDMATTVMGQDIPL

PVIISPTGVQAVHP-DGEVAVARAAAARGTAMGLSSFASKPMEEVTAVNDKIF-FQIYWLSRDAIAARME

RARAAGAKGLILTTDWSFSHGRDWGSPK---IPEQMDLKTILDLRVPNQGR-PGEAYGEW---MGTPPPT

WEDVAWLREQWGGPFLLKGLVRVDDAKRAVDAGVSAITVSNHGGNNLDGTPAAIRCLPAIADAVGDEVLL

DGGIRRGSDVVKAVALGARAVMIGRAYLWGLAANGQAGVENVLDILSGGIDSALRGLGKSSIQELTPEDI

PEG

>C8X9C2|C8X9C2_NAKMY

WFETVAEAQRRAKKRLPKSVYLALVAGSEKGLTVADNQAAFGELGFAPHVAGLSAKRDLATTVMGQEISL

PVIISPTGVQAVDP-DGEVAVARAAAARGTAMGLSSYASKPIEEVIAANPQTF-FQVYWSSRDQIRQRVE

RARAAGAKGLILTLDWSFSMGRDWGSPK---IPEKVNLKAMWDLTVPNLTD-PGGVYYEW---MQTPPPT

WDDVAWLVELWGGPLMLKGICRVDDARNAVAAGVSAISVSNHGGNNLDSTPASIRALPAIVDAVGDEIVL

DGGIRRGSDVVKAVALGARAVMIGRAYLWGLGANGQAGVENVLDILRGGIDSAVLGLGHSTIHDLSPADL

PPG

>C0ZVX3|C0ZVX3_RHOE4

FFETVAEAQRRAKKRLPKSVYAALVAGSEKGLTVDDNVAAFSELGFAPHAAGLSDKREMSTTIMGQDISL

PVMISPTGVQAV-HPDGEVAVARAAAARGTAIGLSSFASKSIEEVAAANPQVF-FQMYWVSRDVLLQRME

RARAAGAKGLIITTDWSFSYGRDWGSPS---IPEKMDLKAMFDLTTPNLAA-PGGAYGEW---MQTPLPT

WEDIAWLREQWGGPFMLKGIMRIDDAKRAVDAGVSAISVSNHGGNNLDGTPAPIRVLPGIAEAVGDEVVL

DGGIRRGGDVVKALALGAKAVMLGRAYLWGLSANGQAGVENVLDLMRMGIDSGLMGLGHSSITELSPADL

PEG

>C1AZV7|C1AZV7_RHOOB

FFETVAEAQRRAKKRLPKSVYAALIAGSERGVTVDDNIAAFGELGFAPHVVGLSDKRELSTTVMGQSISL

PVVISPTGVQAV-HPDGEVAVARAAAARGTAIGLSSFASKSIEEVTAANPQTF-FQMYWVSREVLIQRME

RARAAGATGLIITTDWSFSYGRDWGSPA---IPEKMDLKAMFDLTTPNLAQ-PGGAYGEW---MQTPLPT

WDDIAWLREQWGGPFMLKGVMRVDDAKRAVDAGVTAISVSNHGGNNLDGTPAPIRALPAIAEAVGDEVLL

DGGIRRGSDVVKAVALGARAVMIGRAYLWGLSANGQAGVENVLDVLRGGIDSALLGLGHSNIHDLTPSDV

PPG

>A2C543|A2C543_PROM1

GVLNIDDLRSRAKNRLPAMVFNYIDSGADREQTLSQNCNAYNEILFRPRCAVSVPSCELGISVLDQQFQL

PFLLGPVGSSRMFYPQGEVVAAREAGKAGTGYTLSILSGCLLEDVKAATNGPAWYQLYLLGKEVALKTIA

RAKEAGFSAIVVTIDTPVSGLRERDMRSGTQQLPLEMLPYIPLMSFPNVQL-DDMGYTAI-GPALEQSVT

WDDLQWIREAWGGKIIVKGIHIGDDAKKAVELGADAIVISNHGARQLDSVAPTIRVLPEILAAVDGDVLL

DGGIRRGSDVVKALCLGAKGVLIGRAYAYGLAAAGGKGVARAIEILQTDIVRTMKLLGCGSVADLNKSYI

PES

>A9BCT8|A9BCT8_PROM4

NVVNISDLRLLAKKRLPQMVFDYIDSGADREQTLSQNCTAFKEIYFRPRCAVATPSCDLNISVLDQEFKL

PFILAPVGSSRMFYPKGEVVAAREAGIAGTGYTLSTLSGCRLEEVKQATNCPAWYQLYLLGRDVAMQTIE

RAKSAGFSAIVVTIDTPISGLRERDVRNGTKQLPIQMLPYIPLMSFPNVEL-ESMGYTEI-GPALEESVT

WEDLNWIREAWGGKIIVKGIHIGEDARKAIDLGVDAVVVSNHGARQLDSVAPTIQVLPEVVKAVNGDVLI

DGGIRRGGDVVKALCLGAKGVLISRAYAYGLAAGGGPGVAKAIEIIKTDILRTMKLLGCDSVKKLDRSFV

PPS

>C1MXG2|C1MXG2_MICPS

SAVNIADLRLAARQRAHRMVFDYLDAGADDEITLRRNKDAYSSLELHPRVLAGLKPLDLSARFMRSECAL

PFFVSPTAGSKMFHADGEQGVARAAAKHGVMYSLSTMGTSAPAEVAAAIPPPKLFQLYVWDRALVRDMLR

QAMDNGFDALALTVDLTWYGNRERDVRNGFTVPPAYTLRQIAEYAYAAVK--LAYTLVAFIRDAFDPSFD

WDDAEWLVNEWGGPVALKGVLTPEDARLAVERGFDAVWVSNHGGRQLETSPAAIDVLPNIRDALGGELVV

DGGVQRGTDVLKGLALGADAVALGKPYLYGLGAGGEAGVDRAFTILRDELERAFGLLGVGTTAELRREGL

RRR

>B7FUG8|B7FUG8_PHATC

RAVNIADLRLIAKSRAHKMVFDYLDAGADDEISLRRGKDAYSEFEMHYKVLAGIKPLDLSTKIFGQDVTL

PFFGCPTAGNRMFHWEGETAAAKAAEHHGTMYGLSSLATTGITEIGELFNGPKVFQLYVWDRELVKDVLA

KAKEGGFNALALTVDFTWYGNRERDIRNDFSIPPKYNITQTIPYTYACINT-DV-SLAAFVNSQLSPEFS

WSDAEWLLGEWNGPAAPKGVVRPEDAKKAIEIGFSSIWVSNHGARQLETSPATIDVLPSIRAAVGPEIIM

DGGVQRGTDICKALALGADAVGVGKPYLWGLAAGGTAGVIKAYDILKVELDRAMGLLGTPTVAALKKPSL

IKR

>B8BXJ5|B8BXJ5_THAPS

KAVNIADLRLCAKQRAHKMVFDYLDAGADDEISLRRGKDAYSELEMHFHILSGLKPLDLSTKIFGQDVKL

PFFGCPTAGNRMFHWEGETAAAKAAQHHGTLYGLSSLATTGITEIGKLTDGPKVFQLYVWDRELVKEVLA

KAKEGGFNAMALTVDFTWYGNRERDIRNDFSIPPKYSMAQIVPYTYACINT-DV-SLAAFVNSQLCPEFD

WRDAEWLLGEWNMPSAVKGVCRPDDAIKAVETGFTTMWVSNHGARQLETSPATIDVLPSIREAVGPEIIL

DGGVQRGTDICKALALGADSVGVGKPYLYGLAAGGTEGVIKAYDILKVELDRAMGLLGAGTVDELKKPGL

IKR

>B6BRU7|B6BRU7_9RICK

DCHNFSDFRKLAKKKLPSPIFHYIDGGADDESTLRRNTDSFNDCDLVPNILASVGKPDLSTTLFGRKIDM

PIFLSPAAMQRLYHPDGDQASARAAEKFNTFYSMSSMGNNTIEEVSNISSGPKLFQLYVHDRSISDDLID

RSRRSGFDAMCLTVDTLVAGNREKDHRTGFTTPPKLTLQSLMKFELSNVKK-KTKSVIEYINEQYDPAMG

WKDAEYCAKKWNGPFALKGVMSVEDAKKAIDIGCTAIMISNHGGRQLDGSRSPFDQVKAISDAVGDEIIL

DGGVRRGTHVLKALAAGATACSFGKMFLFSLAAGGQQGVEHLLQNMHDEINRNMVLMGCKNLKELNSSKL

YRK

>D0RQU8|D0RQU8_9RICK

DCHNVDDFRKLAKKKLPSPIFHYIDGGADDESTLKRNTDSFNKCDLVPNVLTDVSNVDTSTTVLGQKIDF

PLFLSPTAMHQMYHHEGEQATARAAEKFGTFFSLSTMGTKSIEEVSNISGGPKMFQLYIHDQGLTDNLIE

RCQRSGFKAMCLTVDTIVAGNRERDHRTGFTTPPKLTLESLFKFKLANISH-LTISIMDYINSQFDTTMN

WKHAEYAAKKWNGPFALKGVMSVEDAKRAIDIGASAIMISNHGGRQLDGSRAPFDQLETLVDAVGDEIIL

DGGIRRGTHVLKALALGAKACSMGKAYLYALGAGGQPGVERVLQKMKDEITRGMTLMGTRNVNELTKDKI

AYR

>B3TCR8|B3TCR8_9BACT

DCHNAEDFRKLAKKKLPAPIFHYIDGGSDDEVTLKRNTDSFNKCDLIPDVLTGASNIDLSTTVLGQKIDF

PLFLAATAMHRLYHHHGERATARAAEKMGTMFGISTMATTSLEEIGKLTSGPKLFQLYIHDKGLTDNLIE

RSRKAGFNSMCLTVDAAVAGNRERDRRTGFTTPPRLTFESLLKFILANIIH-MTKSVIDYINEQFDPAMN

WKDAEYCVKKWNGPFALKGVMSVEDAKKAIDIGCTAIMISNHGGRQLDGSRAPFDQLAEIVDAVGDEVIL

DGGVRRGTHVLKALALGAKACSFGKAYLFALGAAGQQGIEALLQKMKAEINRDMILMGCKSVKDLNRSKV

FRK

>A3WCM9|A3WCM9_9SPHN

DCHNIDDFRKLAKARLPFPVFDYIDGAADDELTKARNTASYDAVDLVPDVLAGVAEIDTSCTILGRKSAL

PLMLSPTAVQRAFHWQGETAVAKAAEKHSLWFGISSLATRSIEEIAALTSGPKMFQLYVHDKGLNTHMIE

RCQAAKFDAMALTVDTIVSGKRERCLRSGFTTPPRFTPASVWKFRLPNLDT-HVVSIAEYFNTMLDTSMD

WSTAAAIREQWGGTFVLKGVMSAGDARRAVEIGADAIMISNHGGRQLDGSRAPFDQLPEIVDAVGGEIIC

DGGVRRGTHVLKTMCSGATAASGGRLYLYALAAAGEEGVMRALDILKDEIERGMRLMGVTSVDQLTQERL

RAR

>A5P8D0|A5P8D0_9SPHN

DCHNIDDFRRLAKRRLPWPVFDYIDGAADDELTKARNTSAFDEVDLVPDVLAGVERIDTSCTIMGCRSEL

PLMLSPTALQRAFHRDGERAVAKAAEKFGVWFGISSLATHSIEEIAALTSAPKLFQLYVHDKGLNASMIE

RCKAADFDALALTVDTIVSGKRERCLRSGFTTPPRFSASSLWKFRLPNLDG-YVVSIQDYFNTMLDTGMD

WDTAARIRQDWGGTFALKGIMSVADARRAVEIGADAIWISNHGGRQLDGSRAPFDQLKEIVDAVGGEIIL

DGGVRRGTHVMKSLAMGATAASGGRLYLYALAAAGQEGVERALTILKEEIERAMRLMGVASVQQLNRERL

RFR

>B6BRU6|B6BRU6_9RICK

DCHNFSDFRKLAKKKLPSPIFHYIDGAADDEITYARNTSAFDDVDLVPNVLRGVENVDLSTTIFGKKLDL

PFYLAPTALQRLFHYDGERAVGKAAKKFNTMFGVSALATVSVEEISSMIDTPKMFQFYFHDRGLNDSCLE

RAKAAKFDVMALTVDTITGGNRERDLRTGFTSPPKLTLSSLFKFELPHLQD-YVTSIGNYFSTMLDQSMN

WKDAEKLCSQWGGHFALKGVMSVEDAKRAVDIGCTGIMVSNHGGRQLDGSRSPFDQLAEIVDAVGDDVIC

EGGIHRGTHMLKALSLGAKACSGGRLYLYALAAGGQAGVERAIEKYKTELVRDMKLMGCTKISDLNRNNL

RFR

>A0Z5I0|A0Z5I0_9GAMM

DCHNFSDFRRLAKKRLPGPIFHYIDGAADDELTYRRNTAAYEDVDLVPNVLRGVADIDTSVEVMGQKLDM

PLFCAPTALQRLFHHDGERAVAKAATEYGTMFGVSSLATVTVEEIAELAPGPKLFQFYFHDRGLNNALLE

RARAANFNVMALTVDTITGGNRERDLRTGFTSPPKLNLSSMWKFDMPHLSG-HIVSVGDYFSTMLDPTMS

WDDAEKLCAQWNGQFALKGIMSVEDAERAVDIGCTGIMVSNHGGRQLDGSRAPFDQLEEICDAVGDDVIC

EGGIQRGTHVLKALSAGAKAVSGGRLYLYALAAAGQAGVERALGNFKTEIERDMRLMGVQRIDELGRGNL

RRR

>B9R3Q8|B9R3Q8_9RHOB

DCHNFQDFRKLAKKRLPGPIFNYIDGAADDEVTYRRNTEAYDSCDLVPNVLAGVENVDMSVEVMGQKLDM

PIYCAPTALQRLFHHDGERAVARAATNYGTMFGVSSLATVTVEEIAQIANTPKMFQFYFHDRGLNDALLE

RAREANFEVMALTVDTITGGNRERDLRTGFTSPPKLTLGSLMKFDMPHLSG-YVVSVGNYFSTMLDQSMN

WKDAEELCAKWNGQFALKGIMSVEDAKRAVDIGCTGIMVSNHGGRQLDGSRSPFDQLAEIVDAVGDDVIC

EGGIQRGTHVLKALSVGAKACSGGRLYLYALAAAGQPGVERALGNLRTEIERDMKLMGITSLDQLSRENL

RET

>A3VEF1|A3VEF1_9RHOB

HCHNFHDFRRLAKRRLPSPIFNYIDGGADDEVTMRRNSAAFDAVDLVPHVLRGTKDVELSVEVMGQKLAL

PFYLSPTALQRLFHHDGERAVAAAAAKYGTMFGVSSLGTTSLEELRRTHDTPQVYQFYFHDRGLNRAMMQ

RAKEAGVDVMMLTVDSMTGGNRERDKRTGFSIPFKLTLAGMAKFSLPQLDD-HVMSIGRYFTEMLDPTMN

WEDLAEMIELWDGKFCLKGVIHPDDAARAADLGCDAVVLSNHAGRQLDGSLAPFDALGGIVDQVGDDVMM

DSGIQRGTHIIKALAMGAKAVGIGRGYLFPLAAAGQAGVERMVGLLKDEVERDMRLMGAAKISDLTRDML

RPR

>A3VM43|A3VM43_9RHOB

DCQNFHDFRTLAKRRLPGPIFNYIDGAADDEVTYRRNSDSFDQVDLLPKVLRGTDEIDLSVEIMGQKLAL

PFYLSPTALQRLFHHRGERAVAAAAEKYGTMFGVSSLGTTSLEELRRKHKTPQVYQFYFHDRGLNRAMMQ

RAKEAGVDVMMLTVDSITGGNRERDKRTGFSIPFRLTLGGMVKFSLPQLDE-HVLSIGRYFTEMLDPTMN

WDDLAEMVEEWGGKFCLKGIIHPEDAVRAAEVGCDAVILSNHGGRQLDGSRAPFDGLAEVVDAVGDDVIM

DSGVQRGTHIVKALSMGAKAVGIGRGYLFPLAAAGQAGVERMVGLLRDEVERDMRLMGAAKVADLSRDNL

RWR

>A6W1N5|A6W1N5_MARMS

DCHNFQDFRTLAKKRLPGPIFNYIDGGADDETTYRRNTAAFEACDLVPSVLTGVKDVDLSVTVMGQKLAL

PIYCSPTALQRLFHHDGERAVANSAEKYGTMFGVSSLGTVSMEEIAKQTTTPQVYQFYFHDRELNRAMMQ

RAKDAGVQVMMLTVDSITGGNRERDLRTGFAIPFKLNLKGIMKFRLPQLEE-HITSIGDYFTNMLDPSMN

WDDVAEMVKFWDGQFCLKGIMSREDARKAVEIGCTGVIISNHGGRQLDGSRSSFDQLAEIVDEVGDDVIF

DSGVQRGTHVLKALSLGAKAVGIGRMYLYPLAAAGQPGVERALGLMKAELERDMKLMGKTSIDQLTRENL

RFR

>B9JFR7|B9JFR7_AGRRK

DCYNFHDFRALAKSRLPGPIFNYIDGAADDEVTLRRNTTSFENCDLVPNVLRGVSSVDMSVTVMGQKLAT

PFYCSPTALQRLFHHQGENAVAAAASSMGTMFGVSSLGTVSLEEVRKKHQGPQVYQFYFHDRGLNRAMMQ

RAKDAGVNVMMLTVDSITGGNRERDLRTGFSIPFKLNLAGLAKFRLPQLDE-HVMSIGKYFTEMLDPSMN

WDDVAEMVRHWGGQFCLKGVMSVEDAKRAVEIGCTGIVLSNHGGRQLDGSRSAFDQLAEIVDAVGDDVMM

DGGIQRGTHVLKALSLGAKAVGVGRFYLYPLAAAGQPGVERALGMLRTEIERGMKLMGCTTVDQLSRANL

RFR

>A0B292|A0B292_BURCH

DCHNFHDFRELAKRRLPGPIFNYIDGAADDETTYRRNTSAFESCDLVPNVLRGVRDVDLSVTVMGQKLGM

PVYCSPTALQRLFHHDGERAVAAAAAKFDTMFGVSSLGTVSLEEARAISPGPQVYQFYFHDRGLNREMMN

RSREAGVNVMMLTVDSITGGNRERDKRTGFSIPFRLTLAGLTRFRLPQLDR-HVMSISRYFTDMLDPSMS

WDDVAAMVREWNGQFCLKGVMSVDDARRAADIGCTGIVLSNHGGRQLDGSRAAFDQLAEVVDAVGDDVMM

DGGVQRGSHVLKALALGAKAVGLGRYYLFPLAAAGQPGVERALQLMRTEIERDMRLMGCASVAQLGRNQL

RFR

>B1M052|B1M052_METRJ

SCHSFHDFRRMAKARLPGPIFDYIDGAADDEVTYRRNTSAFDRCDLVPNVLRGVGDIDLSVTVMGQRLAL

PVYCSPTALQRLFHHQGERAVAAAAGKYGTMFGVSSLGTVSLEEARRISGGPQVYQFYFHDRGLNREMMA

RAKQAGIEVMMLTVDSITGGNRERDKRTGFSIPFRLTLAGMISFKLPQLDG-HVLSISRYFTEMLDPSLS

WDDVAAMVREWGGQFCLKGVMSVEDAKRAVDIGCTGIILSNHGGRQLDGSRTAFDQLAEIVDAVGDDVIM

DGGVQRGTHVLKALSVGAKAVGLGRYYLFPLAAAGQAGVERALDLMRSEIERDMRLMGCASVDQLTRSNL

RFS

>A6UFA2|A6UFA2_SINMW

DCHNFHDFRRLARRRLPGPIFNYIDGAADDEVTLRRNAAAFEACDLVPNVLCGVAEVDMSVTVMGQKLAM

PVYCSPTALQRLFHHQGERAVAAAASKTGTMFGVSSLGTVSLEEARRIGQGPQVYQFYFHDRGLNRAMMQ

RAKEAGVEAMMLTVDSITGGNRERDKRTGFSIPFRLNLAGITRFALPQLDG-HVMSISRYFTEMLDPALH

WDDVAEMVGEWGGPFCLKGVMSVADAKRAVDIGCAGLVLSNHGGRQLDGSRTAFDQLAEIVDAVGDDVMM

DGGVQRGTHVIKALSLGAKAVGLGRYYLFPLAAAGQAGVERALDLMRLEIERSMKLMGCKCVDDLTRSNL

RFR

>A9CFS2|A9CFS2_AGRT5

DCYNFHDFRRMAKQRLPGPIFNYIDGAADDEVTYRRNTAAFENCDLVPDVLQGVADVDMSVTVMGQKLAM

PVYCSPTALQRLFHHQGEKAVAAAAGKFGTMFGVSSLGTTSLEEARRISGGPQVYQFYFHDRGLNRDMMA

RAKSAGVETMMLTVDSITGGNRERDKRTGFAIPFKLNLSGIARFSLPQLDD-HILSISRYFTEMLDPSMN

WDDVALMVREWGGPFCLKGIMSVDDARRAVEIGCSGIVLSNHGGRQLDGSRSAFDQLAEIVDAVGDDVMM

DGGVQRGTHVLKALSLGAKAVGLGRYYLFPLAAAGQPGVERALETMRIEIERGMKLMGCTTVDQLTRRNL

RFH

>C6AZ70|C6AZ70_RHILS

DCYNFHDFRRMAKRRLPGPIFDYIDGGADDEVTYRRNTAAFEACDLVPDVLRGVADVDMSVTVMGQKLAM

PVYCSPTALQRLFHHQGERAVAAAAAKHGTMFGVSSLGTISLEEARQISNGPQVYQFYFHDRGLNREMMA

RAKNAGVQAMMLTVDSITGGNRERDKRTGFAIPFKLNLAGMTRFRLPQLEN-HVLSISRYFTEMLDPSMS

WDDVAEMVREWGGPFCLKGIMSVEDAKRAAEIGCSGIVLSNHGGRQLDGSRSAFDQLAEIVDAVGDDVMM

DGGVQRGTHVLKALSLGAKAVGLGRYYLFPLAAAGQPGVERALETMRTEIERGMKLMGCTSVSQLSRRNL

RFR

>A0YAQ7|A0YAQ7_9GAMM

SCYNIADLRVRAKRKLPAPMFHYIDGGADDEWTLLRNSQAFSDYQIIPNHLRNIESIDLRTDILGTTLDL

PFFLAPTGMSRLFHHHKEPAACRAANEAGTLYSLSTLATSSLEEVAACAVGPKMFQIYILDRGLTREFVQ

RCKESRYQALCLTVDTTIAGNRERDLRNGMTMPPKITMKNFFDFTLANVAH-RVMGLIDYVNSQFDRTIT

WDDAAWLAEQWDGPFVIKGLQSVADVKKARDIGATAVMVSNHGGRQLDGAPAPVDCISVLRDAIGAELIC

DGGIRRGTDIIKAIGLGADACSIGRPYLYGLAAGGQPGVARAIHLLKTEVERSLGLMGCCSIDEVSADQI

VRL

>A0Z3K9|A0Z3K9_9GAMM

RYFNIAELRKAAQKNLPASIFHYLDGGADDEWSLRRNTNAFDQWEIIPSALTGITKPTLNTRLFDRDIAL

PFFLSPTGMSRLFHHDKELAAARAAGKAGTFYSLSSMGSSTIEEVASAVRGPKLFQIYVFDRALTQSFLE

RCKSARYDAICLTVDTTVAGNRERDIRTGMTIPPSLALKSLLDFTLANLSK-NILNIFDYVNQQFDPSIS

WEDVTWLRDRWEGPLIIKGLLSAEDAKQAQRIGCTGVIVSNHGGRQLDSAAAPIDCISAMRDAVGDDLIL

DGGIRRGSHICKALALGASACSIGRPYLYGLAAGGEPGVNQAIEILASETRRCMQLAGFHSVAALQSSGA

VRK

>E9FAV5|E9FAV5_METAR

PI-TVAEVEAIAAKTLSKQIYEFYASGSDEQKLLKRNMSGYDRLYIVPRVLRDVSDVDTRVEMFGSKLNM

PIGIAPSAMQRLAGRGGEIDVARAAVHERVNFTLSSQSTTSLENVMAVDSTPDWFQIYLTDLDKSVDLIK

RAEVAGYKALVVTVDTPVLGNRVNERKNVLALPRGMRLANLERLLMDARTK-HDLVVELGGGEMHASNLS

WAKLSFLRGVTTMKIVLKGVMTPQDARLAILYGADAIVVSNHGGRQLDDAPSTIEVLADIAHAVRGPIIL

DGGIRRGADVFKAIALGADLVWIGRPVLWGLAYDGDKGVGAVLNILERELSRTMALAGVREISEISSAYL

AVA

>C1H9Z6|C1H9Z6_PARBA

PI-TIAELATLAQKKLPKQVWDYYASGADEENALRRNRGAFDRLILRPRVLRDVSRVDTSTTLLGKKYSI

PIGISPSAMQRLAGGNGEIDMARAAASRGTTMILSSHTTCALEDVIRAGGSDFWFQLYISNRERCAQVIG

RAEAAGYKALVLTVDTPILGNRINERKTALILPPHLSLANLHKPTMNRIL--LEIARGNH-DTLNDSSLT

WSNISWLRSKSSLKIILKGIMTAEDALLAIDYGADAVIVSNHGGRQLDSVSSTIEALPEIVSAVRGPVII

DSGITRGSDVFKALALGADFTLVGRSALWGLSFGGQEGVIRVLDILERELSRTMALAGAGTVGEIRRSML

KKD

>E3QWR4|E3QWR4_COLGM

PV-NIADVYEIAREKLAKPAWDYYRTGADDELTLERNHAAYNDILLRPQMLRNVSSIDTTTTIFGKRYDI

PIAIAPTAYQKLAGGEGELDVARAVSNLGTNLTLSSNATTSLEDVEKARGAPRWFQLYFLNRDLTAQLIR

RADNAGYEALVLTVDTVILGNRLQERRTPLELPPGIAMANAE---FGAIS--TEAEYNRIRDRLVNSSLE

WNEIPWLRSQTKMKIILKGILTAEDTQRSIDAGVDAIIVSNHGGRQLDGVPSTIEALPEITEVVRGPVII

DGGITRGTDVFKALALGADLCLIGRTALWGLAWDGQRGVEGVLNILERELARAMALMGVAKLKDISRGLL

RAK

>F9FEE1|F9FEE1_FUSOF

HI-TVDEIKALAQKRLPAYIWRYYADGADDQLTTWQNGEVYKTLVIRPRILRNVSTIDTSTQIFGKHYDV

PIAIAPSAYQRLAGYNGEIDVARAAFARGTNICLSSNATTSLEDVAQARDAPKWFQLYFVSRLITKELIK

RAERAGFEALVLTVDTTTMGNRLHERTNPLKLPADLSMANMTRLILNAETA-EEKIEREHSDLLIDSALT

WTEIPWLRSQTSMKIILKGVLTAEDALLAVDAGVDAIIVSNHGGRQLDSVPATLEALPEVSEAVKGPVLF

DGGITKGTDVFKALALGADLCLLGRSALWGLAVNGQQGVETVLNILERELWRTMVLSGAAAITDISRSMV

-GV

>C1N8W0|C1N8W0_MICPS

RVVCLDDLERAAGRVMDRQDFDYFAGGAETESTLRANASAFADVTLWPRVLVDVRDVDTSTSALGRKLRT

PLLVAPVAMQSMAHPDGERAAVRACASRGVPYCAAQQATTSVERIARAGG-ARWFQLYVRAWAATAALAR

RAALAGATAIVVTVDAPTLGRRERDVRNGFKMRDGLKLANVDGNDSDSDSS-DAKRIAKRVGDR-DASLT

WSSIPWLKSIVALPIILKGVMTREDAALAVAHGVDGVWVSNHGGRQLDGAPSTLRALAEVVAGVNGPVVF

DGGVRRGSDVLKALALGADVVAIGRPVAWGLACGGEAGVRKAIDVLTEELESSMRLAGVTSARDAREKGI

ARA

>B8BZV0|B8BZV0_THAPS

IC-NAGDYQRVARSILPTPLYEYLASGTDDEQTLSENESAFKAWYLRPRVMRPVGSISTVTTLFGQRLSM

PVFVSPAGVHALCDVHGECAAARACGKVGTIFGLSQHATRSIEQVAEATQGNLWYQSYILDREMTLRLAR

RAAKAGYRGIFLTVDSVRFGFREADARNNFSLPPRVNYDDEVPEASVDKSKIYSGQEEAWDEQLFEQNPS

WEDVRWLKREVCLPLIVKGIMTAEDAIEAKKAGADGVMVSNHGGRGLDSALPTIDVLPEIVAAVGDPVLL

DSGIRRGTDVLKALALGATAVGIGKPLFFALSVGGEDAVLNLLQMFQRETEAAMAICGCKSVSDVTRQLV

PSG

>D8LSB4|D8LSB4_ECTSI

RCISLDDFQRQAKPILGKALYEYVASGTDDEQTLSENRQAFKRMFLLPRMMRVVSDIDLRLDVFGQRLSM

PVFVSPAGVHKLMHPEGECATARACAEAGTLMGVSQHATVSLEDVAAAAPRARWFQLYILDRELTAGILR

RSEKAGYTAICLTVDSVRFGSREADWRNNFNLPPGVTLANYPQD------G-YNRVKDAWDEKLFDERAT

WSDIAWLKSLTSLPILVKGILTAQDAVSAVEAGASGVIVSNHGGRALDGSLSSIESLAPVVKAVRGPIFL

DSGVRRGTDVLKALALGATAVLLGRPMFFSLAVGGQEGVQRMLSIIRDELEAAMALCGCQRLQDITKDLV

TDF

>G3MPZ0|G3MPZ0_9ACAR

--MCISDIHRLANEKLETAVRLYYDSGAGEEQTLRENREAFNRLRFRPKLLMDVSRVNTETTLLGSAVSM

PVGFAPSVMQQLAHPDGETGTAQAAEAAGTVMILSALSTVSLEEVRHSAPNCTWLQTFLFDRALTESLVK

RAADAGFSAIVLTVDSPLFGHEMKPSKCRFSLP-NFRLSNLE--SLPKTN----TAFDLFVDDLISQSGV

WSDIAWLRSVSGLPVVVKGVLTPEAAVNSLRSGAAAIIVSNHGGRQLDGTPASIEALPVILAAVGEEVYL

DSGVRTGADVAKALALGTRAVFIGRPVLWGLAYNGKEGVSTVLHIIKNELERTLKLLGCSDISALSEDYV

NKD

>G3MJZ1|G3MJZ1_9ACAR

LVATIADIQRLGEANLENATRRYIASGVDQEQTXXXXTEAFTRFRLRCQVRVDLSRVNTATTVLGRRISI

PIGLSPSATHMMAHPVGELGTVKAARDAGTAMIVSAMSTATLEDIRASAPDTVWQQTYLFNRSLTESLVR

RAAIQDFGAIVVTVDSPISGQASILTKTNFRLS-KLSFANLEASMPGRSLT-YDASADSI-GNLHSPSAT

WEDIRWLRHVSGLPIVVKGVLTAESALTALKYGAAAVLVSNHGGRILDGVPATIQALPEIVAAVGDEIYL

DGGVRSGADVTKALSLGARAVFLGRPVLWGLAYNGKEGVDKVLHIFKNELKRTLQDLGCRDSDDLCTEYI

AAE

>G3MPH2|G3MPH2_9ACAR

LVVTIEDIQRLGEENMDNATRSYVASGAEKEQTLRENAEAFTRFRFRPRALVDVSKINTATTVLGKKISF

PIGFSPTAAHMIANPVGEFGTAKAARDAGTVMIVSSMSTATLEDIRACVPDLVWQQTYIFNRSITESLVR

RAAAQNFGAIVVTVDSPVAGQTISLSKNMLRLP-E---------RFANLEASSPPARENFVGNLLSPTTT

WEDIRWLRQISHLPIVAKGVLTAEAALRALEYGASAVLVSNHGGRQLDSVPATIEALPEVVAAVGDEVYV

DGGVRSGADAAKALSLGARAVFVGRPALWGLAYNGKEGVDKVLNIFRSELKRTIQLLGCPDSNDLCTDYV

VRE

>B7RR92|B7RR92_9RHOB

PV-TLSDFEIDAAGRLSADLLAYLEGGAEAGQSVTENRAAFGRIGLLPKLLSPCAGGHTRTTILGKQAPH

PIMVAPMAFQNLFHPQGESATAMAAAAQDATMVLSCQTSTPPEDIAT-IPGRRWFQLYMQDHEATMALVT

RAVDCGADALVVTLDAPINGLRDREVAAGFTLP-DDVRPVMLRPHLRDGQVVFD-------MMVFAPTA-

-DDLARLIADSPVPVIVKGCLRPADATRLIDLGAQGIIVSNHGGRVLDTVPAPITQLAAVVDAVAGPVYV

DGGIRRGSDVFKALALGAQAVLVGRPVMHGLIVDGPRGASQVLRRLRDELEVTMALCGCATVADITPDLL

FSG

>A3SFF5|A3SFF5_9RHOB

PV-TLSDFEASAHATLAADVFAYLEGGAGGELSLRQNRASFGRIGVLPRLLADCAGGHTRTTISGQPAAH

PIMVAPMAFQRLFHPEGETATALAAAAQEAVMVVSCQTSTAPEDIAT-IPGRRWFQLYMQDHDSTMTLVN

RAVDCGAEALVVTLDAPINGLRDREVAAGFVLP-DGVRPVMLPQDGQSVV--FD-------MMVFAPT--

WADLTRLIADSPVPVIIKGCLRATDARRFVDAGVAGIIVSNHGGRVLDTVPAPVTQLAAVVQAVGQPVYL

DGGIRRGSDVFKALALGAEAVLVGRPVMHGLIVDGARGASQVLRRLRDELEVTMALCGCATVEDITPDMI

TAF

>A3K4B4|A3K4B4_9RHOB

LPGGLADYAAQATALLPPGPAAYFLRGAGAEDTCRANLRDLEAIRIWPRALAPIAGGHTRLTLLGQSLDA

PMLVAPMAYLRVLDAGGEAGVAAAATAQGLGMCLSAQAGQPMEAVRDVGPACRWMQLYWQGRAPTMALAE

RAARAGFTALVLTVDAPVNGIRDAEIASGFALP-DLRAVNL-LQDRESLL----FDRVAHV-------LD

WEDVAWFCANAPLPVLLKGILHPDDATQAVKTGAAGIIVSNHGGRVLDGAPSAIAALPGVVAQVGGPVLM

DGGIRRGVDVFRALALGATAVLIGRPVCHGLAVAGALGVSHVLRLLRDELEVTMALAGCRTLDDITADCI

PTL

>A8HYI4|A8HYI4_AZOC5

EAYDLSDYEALARERVPAASWAYLAAAAGDGLTNAANRAAYDRLRLLPRVLSDLSKATTRINLMGFALEH

PILLAPVAYHRLFHPDGELATAQGAAIAQAPLVVSTQASTSLEEVRAASRGQLWFQLYIQDWGFTVNLLR

RAEAAGYSAVVLTVDAPV-SLRTQERRAGFSLP--PGVEAVNPLHSGGIG----SPLFGT-ALPHTPL--

WGDVARLRSLTRLPILLKGVLAPDDASRALAEGVDGIIVSNHGGRVLDSLPASIEALPRIVETLEGPVLV

DGGIRRGTDILKAMALGANAVMIGRPYIHALAVAGAAGVAHAMHVLRAELEVAMALTGRPTLDTVNRSIL

PPA

>A1WMF9|A1WMF9_VEREI

IV-NLADHESHARAHLDPRAWAYFSGGAADEITLAANRSAWERIRLLPRVLRPLAGGHTRVQLLGRTWAH

PILLAPVAYQRMAHPDGELGSACAAAALGAGIVLSTQASTRLEVVAEADPGPLWFQLYLQDRGFTRALLE

RAEQAGYEALVLTVDAPCHGARDRERRAGFHLP-CVSAVNLHLRPAPRVT--LQDQSALF-DGLLRHAPT

WADVQWLQANTRLPVLLKGLMHPDDARQAAALGVAGLIVSNHGGRTLDTSPGTASVLPRVADAVAGALLV

DGGIRRGTDVLKAMALGASAVLIGRPALYGLANAGAAGVAHVLRLLRDELEIAMALTGCATLAEATPALL

DAA

>A7HQ18|A7HQ18_PARL1

EIACAADYEAFARERVEEGAWAYLDGAAADALTHAENLKAFARIRLVNRVLADLAGGHTRLELFGCAFDH

PVMVAPVAFQKLAHPDGELATVTAAGVLKAGMVVSAQASMDMEELARQAAGPLWFQLYIQDRDFTLQLVR

RAEKAGYRALVLTVDAPVHGARNSEQRAGFSLPPDVEAVNLK--AMRPLPP-YMAVFA---SPLLAAAPG

WKDLNWLAAHTNLPILLKGILHPADAARAVEAGASGIVVSNHGGRTLDTLPAAIEALPGIVEAVAGPVLM

DGGVRRGTDIVKALALGAKAVLVGRPVIDGLAAAGAPGVAHVLHMLRAELEVAMVLTGCRTLADIDASIL

WQD

>A4XQF6|A4XQF6_PSEMY

TIAAVADYEPYARERMSEQAWAYLAGGAADELSLADNRAAFERLRLRGRVLQDLSGGNTRLRLFGQDFAH

PVFLAPVAYQKLAHPDGELASVLAASALGAGMVVSTQASVELEAIAAQAQAPLWFQLYIQDREFTAALIR

RAESAGYQALVLTVDAPVNGVRNREQRAGFALP-AVEAVNLRMRPLQAQAEPHNSLLLG--GPLLAAAPT

WADLTWLREQTRLPILLKGIMSGADAEQALTAGMDGLIVSNHGGRTLDGLPATIDVLPEVAAAVQGPLLL

DGGIRRGSDILKALALGADAVLVGRPYVFALATAGAIGVAHVLQLLRAELEVAMALTGCADLASIGPQVI

WRS

>C1DQ10|C1DQ10_AZOVD

SIAALADYEPFARERMSEQAWAYMAGGAADELTLRDNCAAFQRLRLRSRALPDLTDGHTRLELFGQRFEQ

PILLAPVAYQKLVHPDGELATVLAASAARAGMVVSTQASVALEDIAR--AQPLWFQLYVQDRAFTRELVQ

RAEAAGYQALVVTVDAPVSGLRNREQRAGFALPEGVEAVNL-MRALPPTIA-RISPL--FGGPLLAAAPT

WRELAWLRSLTRLPLLVKGVMHPEDARRALAEGIDGIIVSNHGGRTLDTQPATIEVLEEIAGVVEGPLLL

DGGIRRGTDVLKALALGASAVLVGRSYVFALAAAGAPGVCHALQLLRAELEVAMALTGCRTLADIGPELL

PGR

>C6WFW1|C6WFW1_ACTMD

APGVLAELHERARAALSPEFYDYVAGGAGRERVVAGNERAFDRLALLPRVLRGRAVRDTAVDLPGARTAF

PVLVAPTAFHRLAHPDGELATARATAGAGTALITGMAATTAVAEVVAAAG-AVWFQLYLQDPAVTACLVR

RAEDAGCSALVVTADSPVFGRRERDLRHGFTLPPGYAAENMR--DLPGAPP---GMSAGS---------S

WDDLAALVASTPLPVLVKGVLHPADADLAVRAGAAGVLVSNHGGRQSDVTPPAVTALPAVVDAVAGPVLV

DGGVRRGSDVAVALALGASAVGVGRPVVWGLAADGEAGVRRVLEVLRDEYDHALALCGGRSNADLTRDLV

VDV

>F1L8K8|F1L8K8_ASCSU

HLTTIEEIERAALERLPLDIRQYYAGGSGTESSLRRNKFAFDRLLIRPHVLRNISTIDTSVKIFSKIFDF

PIGIAATAFHKLADPLGEIATVKAAGEMNSLMICSILSNTKLEDIASNAPLTLWHQLYVFDHDVTKQLLQ

RIADAGFDAIVLTVDTPVLGRRPADKRNAFNLPAHLSLANI--ANAHMKQT-EISAFGSYVQQLFDDSLT

FDDLEWLIRESKLPIIVKGVMRAEDADIAVRCGVKGIIVSNHGGRQLDFTPATIECLPEIVRVVARPVFI

DGGVRNGGDIFKAIALGADSVFVGRPILWGLAFQGKDGVRHVLQILRDEFLNIMQLAGCRTIDEICKDIV

VHE

>B1GRK5|B1GRK5_CAEEL

LL-TLDDYRKFSEKNLVKLARDYYESGAEQEESLRRNISAFNNLLIRPRCLRSVENIDTSIDWLGKKSVF

PVGIAPTAFQKMATLDGELSTVRGAAASNSIMICSSWSTTSVEDIGKEVG-TIWFQLYVYDRAITESLIH

RAEAAGVEALVLTVDTPVLGRRLKDTYNKFSLPKHLKFANF-NTQAEMPKG-HVSGFMQYVSSQIDPSLD

WNTLKWIRTKTNLPVIVKGVMRGDDALLALEAGVDGIIVSNHGGRQMDCTVATIESLPEVLRAVDNPVWM

DGGVRNGRDILKAVALGARGVFVGRPVLWGLATSGSAGVSAVLGLLQSEFYHALQLSGFRSIKELKHAIV

SKL

>G0NNG2|G0NNG2_CAEBE

TLLTLEDYRKYSERNLVKLARDYYESGAEQEETLRRNVSAFDRLLIRPRCLRSVAKIDTSIEWLGKKVPF

PVGIAPTAFQKMATKDGELSTVRGAAASKSIMICSSWSTTSIEDIGKEVG-VLWFQLYVYDRKVTEELIH

RAERAGVEALVLTVDTPVLGRRLKDTYNKFSLPSHLKFANF-NTQEKMPKG-GKSGFMQYVSSQIDPSLD

WNTLKWIRTKTKLPVIVKGVMRGDDALLALNAGVDGIIVSNHGGRQMDSCIATIEALPDVLRAVDKPVWM

DGGVRNGRDILKAVALGARGVFVGRPVLWGLATAGSSGVSSVMEILQNEFRHAMQLSGYRSIEELQKVHV

SKL

>A8WQL3|A8WQL3_CAEBR

SLLTLDDYRKYSERNLIKLARDYYESGAEQETTLRRNVSAFDNLLIRPRCLRSVESIDTSVTWLGKRAAY

PLGIAPTAFQKMATKDGELSTVRGAAASKSIMICSSWSTTSIEEIGKEVG-ALWFQLYVYDRNVTESLIH

RAEAAGVEALVLTVDTPVLGRRLKDTYNKFSLPHHLKFANF-NTQAEMPKG-HTSGFMQYVSLQIDPSLD

WNTLEWIKTKTKLPVIVKGVMRGDDALLALGAGADGIIVSNHGGRQMDSSIATIEALPEVLAAVDKPVWM

DGGVRNGRDIFKAVALGARGVFVGRPVLWGLATSGSSGVVAVLGILQKEFLHSMQLSGYRSIEEL-----

KDD

>E3LK37|E3LK37_CAERE

VLLTLEDYRKYSERNLVKLARDYYESGAEQEETLRRNVSAFDRLLIRPRCLRSVESIDTSVEWLGKKVDF

PVGIAPTAFQKMATKDGELSTVRGAAASKSIMICSSWSTTSIEDIGKEVG-TLWFQLYVYDRKVTEKLIH

RAEAAGVEALVLTVDTPVLGRRLKDTYNKFSLPKHLKFANF-NTQAEMPKG-HTSGFMQYVSSQIDPSLD

WKTLEWIRTKTILPVIVKGVMRGDDALLALGAGVDGIIVSNHGGRQMDSSIATIEALPGVLRAVDKPVWM

DGGVRNGRDIFKAVALGARGVFVGRPVLWGLATSGSSGVAAVLGILQSEFRHSMQLSGFRSIAEL-----

KDD

>A9B6H8|A9B6H8_HERA2

PI-NLHEYEQQAMTLLDGPTCDYYAGGCEDEVTLRANLLSFEQVRLRPRFLVDVREVSTATTLLGKPLDS

PILVAPSAYHGLAHAEGECETARGVAQAGSIFTVSTLATRSLEEVAAAAECPLWFQLYVYDRSVSERLIA

RAEAAGYQALMLTIDRPWLGRRERELRSGFGVPAHLSMANF-DVPAAQNYR-RANALPDPKADMFDAGLT

WESIAWLRSVTSLPIIVKGILTAEDALLAAEAGAAAIVVSNHGGRQIDGTVTTLEALPEVVAALAQEIYI

DGGIRRGSDALKALALGAQAIMLGRPVLWGLAVAGSAGVADVLTTMQRELQRSMALCGRPNLASIDRSLV

SGM

>B7T1A3|B7T1A3_9BACT

YV-CLADLEHAARDVLRGEIWDFLAGGSGAEASLGANRTALERIFVIPRMLRDLTDCTTEVEILGRRAAL

PMAVAPVAYQRLFHPEGELAAARAARDAGVPYTICTLSSVPLEEIAAVGGRP-WFQLYWLDEKRSLELVG

RAEDAGCEAIVFTVDVPWMGRRLRDMRNGFALP-HVTAANFD---AGVAA--HRSAVADHTAREFAAA-T

WESVEAVRAHTDLPVVLKGILAVEDARRAVDAGVGGIVVSNHGGRQLDGAVPGIEMLGEIAAAVSGEVLL

DGGIRDGGDVLKATALGASAVLVGRPVMWGLAAAGQDGARQVLDLLATELRDAMGLAGCESVSAARRLST

VVG

>C6WLN8|C6WLN8_ACTMD

DPEDLAEVERAAAARLPGDVRDFIAGGSGDEVTLAANRAALDDVALLPRVLAGVQAADTSTSLVGTAATL

PVAVAPMGYQCLVHPDGEVAAAAAAGAAGVPFTVGTLSSRSVEEIAE-TGASLWFQLYWLDRGLVAELVA

RAEAAGCRALVITVDVPVMGRRLRDVRNGFTLP-RVRAVHLAPSSAHEPR--QVSGVAQHTSAVFDPAFG

WRDLEWLRARTRLPLVVKGVLDPRDATRCVELGASAVVVSNHGGRQLDGAAPSAVALPRVVDAVAGEVLF

DSGVRSGVDVLRALALGATGVLLGRPILWGLAVGGERGAARVLELLRTEFAQALLLAGCADVDAARGLAT

---

>A8IEL8|A8IEL8_CHLRE

FL-NLEEVEEEAKKVMPKMAFDYYSTGSDTCYTVGENRSCFSRYLLLPRMLRNVSRVDTSHELFGIRSSM

PVWVAPMAMHGLAHPGREVATCRAAAAAGVPFTFSTVATSSLQEIQE-TGHRI-FQLYVINREVVRRWVT

EAESRGFKALMVTVDAQRLGNREADARNKFTLPPGLALRNLEYLSSASTADSQDSGLMKLFTSEVDDSLT

WEFIPWLRGVTKLPIIVKGLLSPADAELAVQYGVDGIVVSNHGGRQLDYAPSGLHMLPAVVAAVGSPVLV

DGGVRRGTDVIKALALGASGVLLGRPVLYGLAVGGQAGVERVLQLLRSEIELSMALAGCSSVQQIGPQLL

AGP

>A8M0A4|A8M0A4_SALAI

IA-SVDDLRRLARARLPGPVWDYVTGGAGEERTVRANRDAFRRLTLLPRVLVDVAARDPRTTVLGTGVAA

PVGIAPTSYQSLAHPDGELATARAAGSRGLLDVVSVFSSVSLEDVAEVATGPLWFQLYCLDRGVTRELVQ

RAAAAGYRALVLGVDLPVIGYRDRDIRNRFQLPPSVAPVNL------PTRV-APSVLVELNRALVDPALT

WRDVEWIREISPLPVVVKGIVAADDADRAARIGADAVLVSNHGGRQLDGAPASITALPDVVSVVADEVYL

DSGVRRGTDVLAAVARGARMAFVGRPVMWGLAAGGADGVRAALDLYLTELDLAMAVCGCPDVPSIGPHLL

GPI

>B2J901|B2J901_NOSP7

PI-NLFEYEKLAKEHLSQMTLDYYSSGAWDEITLRDNRAAFERVKLRPRILVDVSDRNLTTSILGQPLQL

PLLIAPMAFQCLAHPDGEVATALAAASAGVGMVLSTMATKSIEEVATAFPESLWFQLYIHDKGLTRALVE

KAYKAGYKALCLTVDAPVLGQRERDRRNEFALPTDLHLANLAL----DISH-EKSGLFTYFAQQLNPAVT

WDDLEWLQSLSPLPLVIKGVLRGDDAVRAVEYGAKAIVVSNHGGRQLDGAIASLDALVEIVAAVDGEVLL

DGGIRRGTDILKALALGAKAVLIGRPILWGLAVAGQVGVSHVISLLQGELNVGMALSGCAKLQDINLSLL

PRF

>A3IHB5|A3IHB5_9CHRO

PI-NLFEYESLAQQQLSSMTWGYYSSGALDEITLKNNRKSFETYQLYPKVLVDVSEINLSTTLLGQTLSI

PIGVAPMAFQCLAHPQGEKATAKVLSDLKTLLILSTLSTTSLEEVAACQEHNLWFQLYIHDKGLTKALVE

RAEKAGYTAICVTVDAPMLGKREIDIRNQFTLP-ELKLANLV--SLEDLA--IPSGLFAYFQQQIDPSLT

WKDLEWLQSITKLPIVLKGILRADDARLAVENGSKGIIVSNHGGRQLDGAITTLEALPKIVETVGNDIII

DGGIRRGTDVFKALALGAKAVLIGRPILWGLTVNGEAGVNHVLELLKDELLLAMALSGCPSIADINDSFL

LKD

>B1WYQ0|B1WYQ0_CYAA5

PI-NLFECESLAKQQLSSMTWGYYSSGALDEITLKNNRKSFNNYQLYPKVLVDVSQINLSTKLLGQTLSM

PIGVAPMAFQCLAHPHGEKATAKVLSDLKSLLILSTLSTTSLEEVAACQENNLWFQLYIHDKGLTKALVE

RAEKAGYTAICVTVDAPMLGKREIDIKNQFTLP-ELKLANLV--TLKDLD--IPSGLFAYFQQQIDPSLT

WKDLEWLQSITKLPIVLKGILRADDARLAVENGVKSIIVSNHGGRQLDGAITTLEALPKIVEAVGNDIIM

DGGIRRGTDVFKALALGAKAVLIGRPILWGLTVNGEAGVNHVLELLKDELLLAMALSGCPSVTEINDSFL

IKS

>A9F5V5|A9F5V5_SORC5

LL-TVDDFERAARARLSKMAYDYYRSGADEGRTLRENRRAFRRLEIHYRVLVDVAERDMSTTVLGTRVPF

PILVAPTAYQRLAHPDGEIASSRAASELGTIFTLSTLSTTSLEAVAGASPGPKWFQLYVHDRGLTRALVE

RAESSGYRALMLTVDTPVLGRRIADVRNGFALP-ELVMANLA----------ERSLLASYVATRHDASLT

WRDVGWLASLTRLPLLLKGIVRPDDALRALEAGAAGVVVSNHGARQLDGAPATIEALPAIADAVAGLVLM

DGGIRWGTDVLKAIALGARAVLIGRPVLWGLAALGGEGVARVLAGLRDELSIAMALAGCPTLASIDRDLI

AHA

>B9XKJ6|B9XKJ6_9BACT

AL-NIFDLEKLAKENLPPTAYDYYSSGAWDEVTLRENCNAFNRIQVHYKVMVDVSKRDLTTTVLGQKVSM

PILLAPTAFHKLAHPDGEVATVRAAGASNTIMTLSSLSTTKVEEVTAAAKSPVWFQLYINDRGFTRDLVA

RVKAAGCKALMLTVDTPEWGRRERDVRNCFHLPPGLSAINLISNERGEFIGQHGGQAFTW---MLDPSLT

WKDVEWLRSITDLPIIVKGVCRPDDAELAIQHGVSAVLVSNHGARQMDTAPATIEVLPAIAEQVAGPVLL

DGGIRRGLDVFKALALGATAVQIGRPVLWGLANGGQQGVQTALELLRKELDLAMALAGCPDIASIKRDFV

PNR

>D0LGI4|D0LGI4_HALO1

PI-HVADFERLARARLAGSAWDYYASGANDELTLRENQAAFARLALHYRVLVDVSERSTRTQLQGHPLSM

PVILAPSAFHRLAHRDGELATARAAGEAGTVMVLSTLSTTRVEEVTAAATGPVWFQLYVYDRAVTRALIE

RVEAAGCEALVLTVDAPLLGRRDRDVRNRFQLPADLHLENLQPA---GLEDLPRAAYFAT---LLDPALS

WDDIEWLRSITRLPLYVKGIVRADDAARAMAAGVDGIWVSNHGGRQLDTSPATIDVLPDIAEAVAVAIIL

DGGVRRGTDVIKAVALGASAVALGRPVLWGLAYDGQAGLSKLLGLLRDEIDLAMALCGCPSVGDLAGELV

RPM

>E9GL31|E9GL31_DAPPU

FV-CVEDYENHAKKALPSYALEYYRSGADEEQTLRENRESFKRWRLMPRMLRGVQNRSMNTTALGCRVSA

PFGIAPTAMQRMAHPDGECATAKAAAAHGIIYILSTIATSSIEEIAEAAPNGIWFQLYIYDRQATIDLIR

RAERANFKALVVTVDTAVLGRRLVNERHGFDLPPHLKLGNFNKSDFHTVQK-EESRLAAYASVMFDSSLT

WKDIDWLKSITKLPIVLKGILRPDDAELAVQHGVSAIGVSNHGGRQLDGVQATIDALPAIVKQVNGEVFL

DGGVTRGTDVLKALALGAKMTFFGRPTLWGLAHSGEQGVKNIIQLLKTEIDVAMALSGCSSVDEIDSSLV

QEL

>B3S6M3|B3S6M3_TRIAD

EVICVRDVEKYAIAHLNKNALGYYDSGADDEETLNDNINACKKLRLRPRMLVDVTKVDCSTTILGQKISF

PVGIAPSAMQRMAHPDGEIATVKAADSLKTCMTLSTLSTTSMESVAEASPNTLWFQLYVVDREITRQFVK

RAEMSGYKALVLTVDAPVLGNRRIDVRNRFHLPPHLSLGNFETLHIEKNK--KSSELSRYFVSEMDASLT

WKDITWLKSITSLPVIVKGILTAEDAEMAVRVGVEGIWVSNHGGRQLDGVPTAIEALPEIVKAVNNEIYA

DGGFRTGTDVFKAIALGARAVFVGRPILWGLVYNGQKGVEKVLQLLQQEFHRTMQLSGCVSIKDIKSSLI

ASS

>F0ZDN7|F0ZDN7_DICPU

FV-TVAEMKEEARKKLPKMVYDYYASGSNDQSTLAENENAFTRIKLVPRSLVNVSKVSTKTKIYGQDLST

PIMIAPWAMQRMAHPNGELDTLEAAKEFGTIMTLSSLSTTSVEDVSKHSNGPGWFQLYVFDRKVSEDLVK

RVEKLGYKALVVTVDTPFLGKRDADYKNQFKLPNGLFLKNF-HLLLSNLE----GGLNQYMATMIDPGLT

WKDLEWLRSITTLPVLVKGVMCPQDAAEALKHGADGIIVSNHGGRQLDTSPSTIEVLPAISKVVQGPLIL

DGGIRRGTDILKALAFGANAVLIGRPVIWGLSCGGKDGVLRVLNLLNSELQLSMAFTGMNSIHEITENII

QNK

>D3BRV9|D3BRV9_POLPA

FV-NIDEFKYAAEKKLPRMVYDYYASGSFDQITLAENQNYFSRIKLLPRCLIDVSNVDMRTNVLGIDLSF

PLMIAPTAMQKMAHPVGETATWSAANELGTSMTLSSLSTTSIEELSKHANGPGWFQLYVFDRAITKNLVQ

RAEQIGYKAIVLTVDTPYLGRREADYRNGFRLPHGLKLQNF-DLPLADVE----GGLNAYVATMIDSSLT

WKDLDWLKSITKLPIIVKGVMSPRDAEIAVTHGVDAIIVSNHGARQLDTAPSTIEVLPYIVKAVNGPVIL

DGGVRRGTDILKALACGAKAVMIGRPVLWGLAVGGKDGVKRVLSLLHDELKLSMALAGVKSISQINKSLI

PSE

>F4QDC7|F4QDC7_DICFS

SYLNIEEFRIVAERKLPRMVYDYYASGSDNQITLGENVNFYSRIKLTPRCLVDVSNINTKTSVFGIPLSF

PVMIAPTAMQKMAHPNGEIDTCLAARDMGTLMTLSSLATTSVEDLGKA-SGPGWFQLYVFDRSISEKLVK

RAEMAGFKAILLTIDTPFLGRRESDYRNEFSLPTGLQLRNF-DLPLADIQ----GGLNKYMATMIDSSLT

WNDLAWLKSITKLPVIVKGVMCPQDALLAVKYGADGIIVSNHGARQLDTSPSTIEVLPYVVRAVGGPVIV

DGGVRRGTDILKALAYGACAVMIGRPVLWGLAADGYDGVLKVLQLLRDELVLSMALAGVNSISKIDESLI

WSS

>C3Z3V2|C3Z3V2_BRAFL

FV-CLADFENFARESLDTNASNYYNSGANNEQTLRDNVDAFRSYRLRPRFLRDVSRRDTTTTVLGELLDF

PVALAPTAMQRMAHPDGEVASAKAAASMNTGMILSSWATSTIEEVAEAAPRGLWFQLYVYDRQVTRNLVE

RAEKAGYKAIFLTIDTPILGKRLEDTRNKFKLPAHLRLANFSDVR-SSRVQ-SDSGLAAYVASLIDPSLS

WEHVDWLRSVTKLPIILKGVLTAEVAREAVEHGVDGILVSNHGARQLDGVPATIDALREVASAVNGEVYL

DGGVRTGTDVLKALALGARCVFVGRPVLWGLAYKGQEGVQEMLQMLKEEFSLSMALSGCSRVSAITPALV

VHE

>G3NMJ1|G3NMJ1_GASAC

RV-CVADFEEQAKKILPKAVYDYYCSGADEQNTLADNVAAFNRWRLVPRVLRDVSTVDLSVSLLGHRLNM

PICVAATAMQRMAHPEGETATARACRAVGTGMMLSSWATSTIEEVMSALGGVMWLQLYIYDRELTLSLVR

RAEEAGYKAIFVTVDTPYLGRRRDDMRNRFKLPPHLSMSNFS--NYGNDS------LAVYVANAIDPSLS

WDDITWIKQHTCLPVIVKGVVNGEDATQAVNYGVDGIVVSNHGARQLDGVPAKLDVLEEVVQAVQGDVYM

DGGVRRGTDVLKALALGAKAVFIGRPVLWGLACQGEQGVIEMLELLQEELRLAMARTSCRSVSEVSRSLT

WHD

>A6H8K0|A6H8K0_XENLA

PI-TVSDYEECARGSLGKSVFDYYGSGADDQQTLADNVDAFSRYRLYPRVLRDVSVTDLSTTVLGQRIRM

PICVGATAMQRMAHPDGETATARACGALGTGMMLSSWATSSIEEVASASPDSLWMQLYIYDRRLTQSLVQ

RAERSGYRAIFLTVDTPRLGRRLADVRNKFQLPPHLRMKNFDELAFSSKQG-FGSGLAVYVAQAIDASIN

WNDIDWLRGITSLPIIVKGIVRADDAKEAVKRGASGILVSNHGARQLDGVPATIDVLQEIIEAVDGEVYL

DGGIRKGTDVLKALALGARAVFVGRPVLWGLAYQGEEGVKDVLNILMEELRLAMSLAGCSSVNEIDKSLV

RKT

>F7C196|F7C196_XENTR

PI-TVDDYEAYARRSLRKSVYDYYRSGAEDQQTLADNVAAFSRYRLYPRVLRDVSATDLSTTILGQKISM

PICVGSTAMQRMAHPDGETATARACRAVGTGMMLSSWATSSIEEVAEAAPDSLWMQLYIYDRNLTKSLVQ

RAERSGYKAIFLTVDTPYLGRRLADVRNKFQLPPHLRMKNFDELAFSSKQG-YGSGLAVYVAQAIDPSIN

WNDIEWLRGITSLPIIVKGIVRADDAKEAVKRGVSAILVSNHGARQLDGVPATIDVLQEITEAVDGEVYL

DGGIRKGTDVLKALALGARAVFVGRPALWGLAYQGEEGVKDVLNILMEEFRLAMSLAGEHMTYHRQGDLI

KPP

>G1N447|G1N447_MELGA

PV-CVADFEHYAKTFLPKSVYDYYRSGADDQETLADNVAAFSRWKLYPRVLRDVSVMDLSTSVLGQKISM

PVCVAATAMQRMAHPDGETATAKACQAMGTGMMLSSWATSSIEEVAEAAPGGLWLQLYVYDREVTKSLVK

RAERAGYKGIFVTVDTPFLGRRIDDVRNKFQLPPHLRLKNFS--DFGENS------LAVYVANAIDASIS

WKDIKWLRELTSLPIVAKGILRADDAKEAVKLLVSFLIFVNRNCVHVVGTAAMIDILPEIVEAVEGEVFL

DGGVRKGTDILKALALGAKAVFIGRPLIWGLVYQGEEGAKEVLQMLKEEFRLAMALTGCRTVKEIGRTLI

RRH

>G1KBH8|G1KBH8_ANOCA

PI-CIADFEHYAKAFLGKSVYDYYKSGADEQQTLAENVAAFSRLKLYPRMLKDVSSLDLSTSVLGQKVSM

PICVAATAMQCMAHADGEIATVRACRSMGTGMMLSSWATSSIEEVAQAAPEAVWLQLYIYDREVTKSLVR

RAEKTGYKGIFVTVDTPFLGKRLDDVRNKFQLPPHLRMKNFEDLAFSSEKG-YGSGLSVYVAEAIDPSIN

WEDMKWLRGLTSLPIVAKGIIRADDAREAVKHGVNGILVSNHGARQLDGVPATIEILPEIIEAVEGEVFL

DGGIRKGTDVLKALALGARAVFLGRPIIWGLAYQGEQGVKEVLQILKEEFHLAMALSGCQSVEAIDRTLV

RRE

>F6VW26|F6VW26_ORNAN

LV-CIDDYEKHAKMVLQKSVYDYYRSGANDEETLADNIDAFSRWKLYPRVLRDVSALDLSTSVLGQRVSM

PICVAATALQRMAHADGEIATVRACRAMGTGMMLSSWATSSIEEVAQAAPDGIWLQLYIYDRELTKQLVE

RAEKMGYKAIFLTMDTPYLGNRLDDTRNQFHLPPHLRMKNFEDLAFSSKKG-YGSGLAGYVAQAIDPSIN

WQDIKWLKGLTSLPIVAKGILRADDAREAVKYGVSGILVSNHGARQLDGVPATIDVLSEVVEAVEGEVFL

DGGVRKGTDVLKAIALGARAVFIGRPIIWGLAYQGEEGAKNVLKMLKEEFQLAMALTGCRNVKGIDKTLV

RKN

>B0BNF9|B0BNF9_RAT

LV-CISDYEQHARTVLQKSVYDYYKSGANDQETLADNIRAFSRWKLYPRMLRNVADIDLSTSVLGQRVSM

PICVGATAMQCMAHVDGELATVRACQTMGTGMMLSSWATSSIEEVAEAGPEALWMQLYIYDREVSSQLVK

RAEQMGYKAIFVTVDTPYLGNRFDDVRNRFKLPPQLRMKNFE--NFGDNS------LAEYVAQAIDPSLS

WDDIKWLRRLTSLPIVVKGILRGDDAQEAVKHGVDGILVSNHGARQLDGVPATIDALPEIVEAVEGEVFL

DGGVRKGTDVLKALALGARAVFVGRPIIWGLAFQGEKGVQDVLEILKEEFRLAMALSGCQNVKVIDKTLV

RKN

>G5BAS2|G5BAS2_HETGA

LV-CINDYEQHAKSVLQKSVYDYYRSGANDQETLADNIAAFSRWKLFPRILQNVAEVDLSTSVLGQRVSM

PICAGATAMQCMAHVDGERATVRACQTLGTGMMLSSWATSSIEEVAEACPDALWMQLYIYDREVTKQLVK

RAEKMGYKAIFVTIDTPYLGNRFDDVRNRFKLPPQLRMKNFE--NFGDNN------LAEYVAKAIDPSIS

WEDITWLRGLTSLPIVAKGILRGDDAREAVKRGMDGILVSNHGARQLDGVPATIDALPEIVEAVEGEVFL

DGGVRKGTDVLKALALGAKAVFLGRPIIWGLAFQGEKGVQNVLEILKEEFHLAMALSGCQNVKVIDKTLV

RKN

>G1T1H2|G1T1H2_RABIT

LV-CINDYEQQAKLILQKSVYDYYRSGANDQETLADNVAAFSRWKLYPRMLRNAAEIDLSTSVLGQRISM

PICAGATAMQCMAHEDGELATVRACQSLGTGMMLSSWATSSIEEVAEAGPDALWMQLYIYDREVTKQLVR

RAEQMDYKAIFVTVDTPYLGNRFDDVRNRFKLPPQLRLKNFE--NFGDTN------LAAYVAKAIDPSIS

WEDIKWLRGLTSLPIVAKGILRGDDAKEAVKHGLDGILVSNHGARQLDGVPATIDVLPEIVEAVEGEVFL

DGGVRKGTDVLKALALGAKAVFVGRPIIWGLAFQGEQGVQDVLEILREEFRLAMALSGCQNVQVIDKTLV

RKN

>G3TGL6|G3TGL6_LOXAF

LL-CINDYEQHAKSVLPKSVYDYYRSGANDQETLADNVTAFSRWKLYPRMLRNVAEMDLSTSVLGQRVSM

PICVGATAMQRMAHVDGELATVRACLSLETGMMLSSWATSSIEEVAEAGPNTLWLQLYIYDREVTKQLVR

RAEQMGYKAIFLTVDTPYLGNRFDDVHNRFKLPPQLRMKNFE--NFGDNS------LAAYVAKAIDPSIS

WEDLKWLRGLTSLPIVAKGILRGDDAREAVKQGVDGILVSNHGARQLDGVPATIDALPEIVEAVEGEVFL

DGGVRKGTDVLKALALGAKAVFLGRPIIWGLASQGEKGVQNVLEILKEELRLAMALSGCQNVKAIDKTLV

RKN

>F7FSQ5|F7FSQ5_CALJA

LV-CINDYEQHAKSVLPKSIYDYYRSGANDEETLADNIAAFSRWKLYPRMLRNVAETDLSTSVLGQRVTM

PICVGATAMQRMAHVDGELATVRACHSLGTGMMLSSWATSSIEEVAEAGPEALWLQLYIYDREVTKRLVR

QAEKTGYKAIFVTVDTPYLGNRLDDVRNRFKLPPQLRMKNFE--SFGDDS------LAAYVVKAIDPSIN

WEDIKWLRRLTSLPIVAKGILRGDDAREAVKHGLNGILVSNHGARQLDGVPATIDALPEIVEAVEGEVFL

DGGVRKGTDVLKALALGAKAVFVGRPVIWGLAFQGEKGVRDVLEILKEEFRLATALSGCQNVKVIDKTLV

RKN

>E1BC79|E1BC79_BOVIN

LV-CISDYEQHAKSVLQKSIYDYYKSGANDQETLADNIAAFSRWKLYPRMLRNIAEIDLSTSVLGQKVSM

PICVGATAMQCMAHVDGELATVRACRSLGTGMMLSSWATSSIEEVAEAGPEAIWLQLYIYDREVTKQLVR

RAERMGYKAIFVTVDTPYLGNRFDDVRNRFKMPPQLRMKNFE--NFGDKS------LAAYVAKAIDPSIS

WEDIKWLRRLTSLPIVAKGILRGDDAKAAVKHGLDGILVSNHGARQLDGVPATIDVLPEIVEAVEGEVFL

DGGVRKGTDVLKALALGAKAVFVGRPIIWGLASQGEKGVQDVLEILKEEFWLAMALSGCQNVKVIDKTLV

RKN

>G1MC49|G1MC49_AILME

LV-CISDYEQHAKSVLQKSIYDYYRSGANDEETLADNSAAFSRWKLYPRMLRNVAEVDLSTSVLGQRVSM

PICAGATAMQCMAHVDGELATVRACRSLGTGMMLSSWSTSSIEEVAEASPELRWLQLYIYDRDVTKQLVQ

RAERKGYKAIFLTVDTPYLGNRFDDVRNSFKLPPHLRMKNFE--NFGDKS------LASYVTKSIDPSIS

WEDIKWLRGLTSLPIVAKGILRGDDAREAVKHGLNGILVSNHGARQLDGVPATIDALPEIVEAVEGEVFL

DGGVRKGTDVLKALALGAKAVFVGRPIIWGLASQGEKGVQDVLEILKEEFRLAMALSGCQNVKVIDKTLV

RKN

>F7D6F2|F7D6F2_MONDO

PV-CIDDFEKYAKTILQKSVYDYYRSGANDQETLADNIAAFSRWKLYPRILRNVAKVDLTTSVLGQKISM

PICVASTAMQRLAHVDGELATVRACHSMGTGMMLSTWATSSIEEVAQAAPDTRWLQLYIYDREISEQLVK

RAERNGYKGIFLTVDTPYLGNRFDDVRNRFQLPPHLRMKNFQDLAFSSKEG-YGSGLAQYVANMIDSSIN

WEDITWLKKLTTLPVVAKGILRADDARTAVKYGVDGILVSNHGARQLDGVPATIDVLPEIVEAVEGEVFL

DGGIRKGTDVLKALALGAKAVFLGRPIIWGLAYQGEKGVKQVLEMMKEEFQLAMALTGCRNVKDIDKTLV

RTN

>G3VF29|G3VF29_SARHA

PV-CIDDFEKYAQSVLQKSIYDYYRSGANDQETLADNIAAFSRLKLYPRMLRNVVKVDLTTSVLGQRISM

PICVASTAMQRMAHVDGEIATVRACHSVGTGMMLSTWATSSIEEVAQAAPDGTWMQLYIYDREVTEQLVK

RAERNGYKGIFLTVDTPYLGNRFDDVRNRFQLPPHLRMKNFQDLAFSSEEG-YGSGLAEYTANAIDASIN

WKDITWLKKLTTLPIVAKGILRADDAREAVKYGVNGILVSNHGARQLDGVPATIDVLPEIIEAVEGEVFL

DGGVRKGTDVLKALALGAKAVFVGRPIIWGLAYQGEKGVKEVLEMLKEEFRLAMALTGCRNVKDIDKTLV

RTN

>F0WZW4|F0WZW4_9STRA

PI-NALDYEEFAREYLPKNAYDYYATGADDKVTLKENQNAFQRIKLRPRVLRNVSTMHMRTSLLGSEVDT

PVCIAPTAMHCMAHYEGEVATARAAARMNTCMILSTLSTKSIEDVANASGNGLWFQLYVFDRDLTLSLVK

RAEQAGYKAIVLTVDTPVFGQREADVRNRFALP-RLKLANFTAHSVQSTE----SGVAEYVSTFFDPTLD

WDDVKWLKRNTTLPLVIKGILTAEDAVLVAEIGCDAIIVSNHGARQLDGVLATIEALPEVVKAVKGEVYV

DGGFRRGTDIFKALALGARAVFLGRPILWGLSHDGETGAYKVLRMLTDELQTTMVFSGTRRLCDISLEYV

QDQ

>D0N9T9|D0N9T9_PHYIT

PL-NVLEYEEYAKEYLPKNAYDYYASGADDMVTLKENREAFKRLVLHPRVLRDVSNMDTNTTLLGHRISS

PVCVAPSAMHRMAHPDGEIASTSATAKADTCYILSTISTTSLEDVAKANPHALWYQLYVFDREITRGLVR

RAEKAGYKAIVLTVDTPMLGHREPDVRNRFSLPNHLTMANFADHENGVS---SLSGLAHYVSELFDLTLN

WSDVKWLKSITKLPVVVKGVLSPEDAKIAVDMGCEGVLVSNHGARQLDGVAATIDALPAIAEAVGGEVYL

DGGVRRGTDVFKALALGARAVFLGRPVLFGLAHSGEAGVSNVLRILNDELKHAMLFSGTAKLADIGPAYV

RRG

>G5AB06|G5AB06_PHYSP

PL-NVLEYEEYAKEYLPKNAFDYYASGADDMVTLQENREAFKRLVLHPRVLRDVSNMDTSTTLLGHRVSS

PVCVAPSAMHRMAHPDGEIASSSATAKADACYILSTISTTSLEDVAVANPNALWYQLYVFDREITRGLVK

RAEKAGYKAIVLTVDTPMLGHREPDVRNRFSLPSHLTMANFAEHEHGVN---SLSGLAHYVSELFDLTLN

WNDVKWLKSITKLPVVVKGVLSPEDAKIAVDMGCEGILVSNHGARQLDGVAATIDALPAIVQAVDGEVYL

DGGVRRGTDVFKALALGARAVFLGRPVLFGLAHSGEAGVSNVLRILNDELRHAMLFSGTAKLADIGPAYV

RRG

>D7FMI7|D7FMI7_ECTSI

HV-NVADYERRAKVVLPKGEFDYFAGGANDMVTLRENRAAYRRLRLRPRVLRDVSSVDTTRTVLGERMAH

PIGISPTAEHRAAHDDGELATARAAAGTCSMMVVSSSATTALEDVATAGGPQRWFQLSLSNRTVLAGLVR

RAIAAGYTALVVTVDRPVLGRREADLRNCYEAP-RLAEGRVV--ATGARIGRRPGQASDA-RPEAGKSLN

WDDVHWLRTICDMKIVVKSVMTREAAEEALAHGVDAVWVSNHGGRQLDTVPATIEILPEVVQAVRGEIFV

DGGIRRGTDVLKALALGASAVFIGRPVIWGLAHSGEHGVTDVINLLNEELVQAMRLMGCKKLGDIERSMV

AHQ

>D8QQ12|D8QQ12_SELML

VV-NVDEYEDLARVKMPKMYYDFYAGGAEDKWTLRENRSAFSRIRIRPQVLVDVSHTDLTTSVLGLKIAC

PIMVAPTALHKLAHPEGELATARATAAANTVMVVSTSSSHTIEEIADTGPGIRFFQLYIFNKVRAMELVA

RAEKAGYKAIVLTVDTPILGRREDDLRNRLVLPPDVSMKLIDQHSQPTEP----SSLAAVASEYKDKSIT

WKDVQAFMKLTKLPFLLKGILTKEDALKAIDICVDGIIVSNHGGRQLDHVPATISVLEEVVAAAAGPVFV

DGGIRRGTDVFKALALGASGVFVGRPVLFGLAIDGEQGVKKVLDMLKDELRTTMVIAGCPTLAHINRSSV

QTP

>D8LDI6|D8LDI6_ECTSI

PV-NVREFERHAQLMLSKNAFDYYASGANDMVTLRENRAAFNRLRLRPRILRDVSMVDTSTSVLGQKISS

PICIAPTAMQRMAHDSGECATAGAAAKAGALMTLSSWSTTSLEDVAKAGGPARWFQLYVYDRKITEQLVK

RALAAGYTALAVTVDTPVLGRREADMRNRFKLPEHLTMGNFVAHASGTKD--GGSGLAAYVASLIDRTLD

WNDIKWLRTICSMKIVVKGVMTAEDAAESVRQGVDGIWVSNHGARQLDTTPATIEVLPEVVAAVSGEIYL

DGGICRGTDVFKALALGAKAVFIGRPVLWGLAHSGEEGVSKVLKLLHDELVMALQLTGCTRVSSASRSMV

THQ

>B8B8K5|GLO4_ORYSI

PV-NVREYQELAKKALPKMAYDYINGGAEDEHTLRENIAAYTRIILRPRVLVDVSKIDMSTTLLGYTMRS

PIIVAPTGGHKLAHPEGEKATARAAASCNAIMVLSFSSSCKIEDVASSCNAIRFYQLYVYNRNVSATLVR

RAESCGFKALLLTVDTPMLGRREADIRNKMVFP-RGNLEGLM--TIDDHDT-TNSQLERFARATLDPSLS

WKDIEWLKSITSMPIFLKGIVTAEDARRAVEAGVAGVIVSNHGARQLDYAPATIAALEEVVRAVAGPVLV

DGGIRRGTDVFKALALGARAVMXXXPVFFGLAARGEAGARHVIEMLNGELEVAMALCGCRSVGEITRSHV

MTE

>C5XE15|C5XE15_SORBI

PV-NVREYQELAKKALPKMHYDYINGGAEDEYTLRENIAAYGRILLRPRVLIDVSKIDMSTSLLGYNMPS

PIIVAPTGSHKFANPEGEVATARAAAACNTIMVLSFSSNCRIEEVASSCDAIRFYQLYVYRRDVSATLVR

RAESLGFRAIVLTVDTPVLGRREADIRNKMIAP---QLSNL-LMSLDDFDG-GESKLERFSRETLDPSLS

WKDVEWLKSITSLPILLKGIVTAEDARKAVEVGVAGVIVSNHGARQLDYAPPTISALEEVVKAVAGPVLV

DGGVRRGTDVLKALALGAKAVMVGRPVFYGLAARGEAGARHVIEMLNKELELAMALCGCRSVAEVTRAHV

QTE

>F4MKM3|F4MKM3_PINPS

PV-NVNEFSTLARNVLPKMIYDFYAGGAEDEWTLRENVAAFQRTRLRPRVLVDVSNVDLSTTILGFKISA

PIMIAPTAMHKLAHPEGVTATARAAAAAGTIMVLSFSATSTVEEVAATCDAVRFFQLYVYNRSISAVLAQ

RAERAGYKAIVLTADTPKLGRREADIRNKLVVP---TLKNL-LLSI-NMD--TESGLASYASQTLDSSFS

WKDIKWLQSLTSLPILIKGILTAEDAELAIQAGFAGIIVSNHGARQLILCHQRLWLIEEVTKAVRGPVLF

DGGIRRGTDVFKALAIGAQAVLVGRPIIYGLAVKGESGVKKVLEMLQDELELAMSLSGCCRVEEITRSHV

QTE

>A9SQ21|A9SQ21_PHYPA

VV-NVDEYELLAKAKMSKMAFDYFARGSEDQVSLRENREAFSRIRLRPRILVDVSNIDVATSVMGFKISM

PIMVAPTAHHKLAHPEGELATARAASAADTLMILSSSANCSMEEVAATGPGVRFFQLYVYDRNITITLVR

RAEQFGFKAIVLTVDTPRLGRREADIKNRFKLPSHLVYKNL-LMNLEQMD--KSSELASWADSHFDRSLN

WKDVEWLQSITHLPVLVKGILTAEDASLALQAGVKGIIVSNHGARQLDHVPATISVLEEVVYAVRGPVFL

DGGIRRGSDVFKALALGASGVFVGRPVPYALAVDGEAGATKVLQMLRDEFELTMALIGVRSVKEIRRQHV

LTE

>D8QNN2|D8QNN2_SELML

EITNVTEYEELAKQRLPKMAFDYYASGAEDQWTLKENRTAFERIRFRPRILVDVTNVDMTTTVLGFKISM

PIMVAPTAFQRMAHPEGELATARAVSSHGTIMTLSSWATSSVEEVASTGPGIRFFQLYVYDRNVVAQLVR

RAEKAGFKAIALTVDTPRLGRRESDIKNRFVLP-GLTLKNFDGLDLGKMDK-SQSGLATYVAGQIDRSLS

WKDVKWLKTITSLPILVKGVITAEDAHIAVEAGAAGIIVSNHGARQLDYVPATISALEEVVQAAAGPVFL

DGGVRRGTDALKALALGAAGVFIGRPVVFSLAVHGETGVRKVLQMLRDEFEIAMALAGCTKVSEINRSHV

ETD

>A9RJ44|A9RJ44_PHYPA

EVTNVTEYEELARQKLPKMVFDYYASGAEDQWTLRENRNAFERIRFRPRILIDVTKVDLTTNVLGFNISM

PIMVAPTAMQRMAHPDGELATARAVSKAGTIMTLSSWATSSVEEVASVGPGIRFFQLYVYDRNVVAQLVR

RAERAGFKAIALTVDTPRLGRRESDIKNRFALP-SLTLANFEGLDLGKMDK-TQSGLASYVAGQIDRSLS

WKDVKWLQTITKLPILVKGVITAEDTQLAIQSGAAGIIVSNHGARQLDYVSATISALEEVVLAARGPVFL

DGGVRRGTDVLKALALGASGVFVGRPVVFGLATDGQKGVEKVLQMLRDEFELAMALAGCTKVSDIKRSHI

QTE

>A9SRU3|A9SRU3_PHYPA

EVTNVTEYEELARQKLPKMVYDYYASGAEDQWTLKENRSAFERIRFRPRILIDVTKVDLSTNVLGFNISM

PIMVAPTAMQRMAHPEGELATARAVAKAGTIMTLSSWATSSVEEVASVGPGIRFFQLYVYDRNVVAQLVR

RAERAGFKAIALTVDTPRLGRREADIKNKFVLP-SLTLANFE--DLGKMDK-TASGLASYVAGQIDRSLT

WKDVKWLQTITSLPILVKGVITAEDTELAVQHGAAGIIVSNHGARQLDYVSATISALEEVVQAARGPVFL

DGGVRRGTDVLKALALGASGVFIGRPVVFGLATDGQKGVENVLQMLRSEFELAMALAGCTKVSDIKRCHI

QTE

>A9RWX7|A9RWX7_PHYPA

EIVNVSEYEELARQKLPKMVYDYYASGAEDQWTLKENRSAFERIRFRPRILIDVTKVDLSTNVLGFNISM

PIMVAPTAMQRMAHPDGELATARATAKAGTIMTLSSWSTSSVEEVASVGPGIRFFQLYVYDRNVVAQLVR

RAERAGFNAIALTVDTPRLGRRESDIKNRFALP-KLTLANFE--DLGQMDK-TQSGLASYVAGQIDRSLS

WKDVKWLQSITELPILVKGVITAEDTKLAIQNGAAGIIVSNHGARQLDHVSATISALEEVVQAAAGPVFL

DGGVRRGTDVLKALALGASGVFIGRPVVFGLACDGQQGVEKVLQMLRDEFELAMALAGCTKVSDISRAHV

QTE

>F2DHZ5|F2DHZ5_HORVD

MITNVSEYERLAKEKLPKMVYDYYASGAEDQWTLNENREAFSRILFRPRVLIDVSHINMATSILGFDVSM

PIMIAPTAMQKMAHPEGELATARAAASAGTIMTLSSWATSSVERVNSVGPGIRFFQLYVYDRNIVRQLVK

RAEMAGFKAIALTVDTPRLGRREADIKNRFILPPHLVLENFAALDLGKMDK-TDSGLASYVASQVDQSLC

WEDVKWLQTITSLPILVKGVMTAEDTRIAIEYGAAGIIVSNHGARQLDYVPATISCLEEVVREAKGPVFL

DGGVRRGTDVFKALALGAAGVFIGRPVLYSLAVDGEAGVRKVLQMLRDELELAMALSGCASLRDITRAHV

DGD

>D7MCT8|D7MCT8_ARALL

EITNVMEYEKIAKEKLPKMVYDYYASGAEDQWTLQENRNAFSRILFRPRILIDVSKIDVSTRVLGFNISM

PIMIAPTAMQKMAHPDGELATARATSAAGTIMTLSSWATCSVEEVASTGPGIRFFQLYVYDRNVVIQLVK

RAEEAGFKAIALTVDTPRLGRRESDIKNRFALP-RLTLKNFEGLDLGKIDK-TNSGLASYVAGQVDQSLS

WKDIKWLQSITSLPILVKGVITAEDARIAVEYGAAGIIVSNHGARQLDYVPATIVALEEVVKAVEGPVFL

DGGVRRGTDVFKALALGASGVFVGRPSLFSLAADGEAGVRKMLQMLRDEFELTMALSGCRSLREISRNHI

KTD

>C6TM54|C6TM54_SOYBN

MITNVTEYEAIAKEKLPKMVYDYYASGAEDQWTLNENRNAFSRILFRPRILVDVSKIDLTTTVLGFKISM

PIMIAPTAMQKLAHPEGELATARAASAAGTIMTLSSCASSSVEEVAS-TGSDIFFQLYVLDRNVVAQLVR

RAERAGFKAIALTVDTPILGHREADIKNRLTLPLNLALKNFEGLDLGKLDK-TSSGLASYVAGQIDPSLN

WKDIKWLQSITSLPILVKGVLTVEDTRIAIQAGAAGIIVSNHGARQLDYVPATIMALEEVVKAAQGPVFL

DSGIRRGTDVFKALALGAAGVFIGRPVVFSLAADGEAGVRKVLQMLRDELELTMALSGCRSLKEITRDHV

VTE

>B9H2B3|B9H2B3_POPTR

QITNVMEYQEIARQKLPKMVYDYYASGAEDQWTLKENRNAFSRILFRPRILIDVSKIDMSTTVLGFKISM

PIMIAPTAMQKMAHPEGEYATARAASAADTIMTLSSWATSSVEEVASTGPGVRFFQLYVHDRNVVAQLVR

RAERAGFKAIALTVDTPRLGRREADIKNRFTMPPYLTLKNFEGLDLGKMDK-TDSGLASYVAEQIDRSLS

WKDVKWLQTITSLPILLKGVLTAEDARLAVQNGAAGIIVSNHGARQLDYVPSTIIALEEVVKAVQGPVFL

DGGVRRGTDVFKAMALGASGIFIGRPVVFSLAADGEAGVRKVLQMLRDEFELTMALNGCRSLKEISRNHI

VAD

>D7EZN6|D7EZN6_9ASTR

GVTNVTEYEAIAKEKLPKMVYDYYASGAEDQWTLEESRNAFSRILFRPRILIDVSKIVMTTTILGFKISM

PIMVAPTAMQKMAHPEGEYATARAASSAGTIMTLSSWATSSVEEAASTGPGIRFFQLYVYDRNVVAQLVR

RAERAGFKAIALTVDTPRLGRREADIKNRFTLPPFLTLKNFEGLDLGKMDE-ANSGLASYVAGQIDRTLS

WKDVQWLQTITKMPILVKGVITAEDTRLAIQAGAAGIIVSNHGARQLDYVPATISALEEVVKAAQGPVFL

DGGVRRGTDVFKALALGAAGIFIGRPVVLSLAAEGEAGVRKVLQMLRDEFELTMALSGCTSLKEITRDHI

VTE

>B9S0Y9|B9S0Y9_RICCO

EITNVMEYEEIARQKLPKMVYDYYASGAEDQWTLKENRNAFSRILFRPRILIDVSKIDMTTSVLGFKISM

PIMIAPTAMQKMAHPEGEYATARAASAAGTIMTLSSWATSSVEEVASTGPGIRFFQLYVYDRNVVAQLVR

RAERAGFKAIALTVDTPRLGRREADIKNRFTLPPFLTLKNFEGLDLGKMDK-SDSGLSSYVAGQIDRTLS

WKDIKWLQTITSLPILVKGVLTAEDTRLAIQNGAAGIIVSNHGARQLDYVPATIMALEEVVKAAQGPVFL

DGGVRRGTDVFKALALGASGIFIGRPVVFSLAAEGEAGIRKVLQMLRDEFELTMALSGCRSLREITRDHI

VTD

>B0M1B1|B0M1B1_SOYBN

EITNVSEYEAIAKQKLPKMVFDYYASGAEDQWTLQENRNAFSRILFRPRILIDVSKIDITTTVLGFKISM

PIMLAPTAMQKMAHPEGEYATARAASAAGTIMTLSSWATSSVEEVASTGPGIRFFQLYVYDRNVVAQLVR

RAERAGFKAIALTVDTPRLGRREADIKNRFTLPPFLTLKNFEGLDLGKMDK-ADSGLASYVAGQIDRTLS

WKDVKWLQTITKLPILVKGVLTAEDTRIAVQSGAAGIIVSNHGARQLDYVPATISALEEVVKAAEGPVFL

DGGVRRGTDVFKALALGASGIFIGRPVVFSLAAEGEAGVRNVLRMLREEFELTMALSGCTSLKDITRDHI

DWD

>D7KVA4|D7KVA4_ARALL

EITNVNEYEAIAKEKLPKMVFDYYASGAEDQWTLQENRNAFSRILFRPRILIDVSKIDMTTTVLGFKISM

PIMVAPTAMQKMAHPEGEYATARAASAAGTIMTLSSWATSSVEEVASTGPGIRFFQLYVYDRNVVAQLVR

RAERAGFKAIALTVDTPRLGRRESDIKNRFTLPPYLTLKNFEGLDLGKMDE-ANSGLASYVAGQIDRTLS

WKDVQWLQTITKLPILVKGVLTAEDARMAVQAGAAGIIVSNHGARQLDYVPATIIALEEVVKAAQGPVFL

DGGVRRGTDVFKALALGASGIFIGRPVVFSLAAEGEAGVRKVLQMMREEFELTMALSGCTSLKEITRNHI

ITD

>E1AXT8|E1AXT8_NICBE

EVTNVMEYEAIAKKKLPKMVFDYYASGAEDQWTLAENRNAFSRILFRPRILIDVSKIDMSTTVLGFKISM

PIMIAPTAMQKMAHPEGEYATARAASAAGTIMTLSSWATSSVEEVASTGPGIRFFQLYVYDRNVVAQLVR

RAERAGFKAIALTVDTPRLGRREADIKNRFVLPPFLTLKNFEGLDLGKMDQ-ASSGLASYVAGQIDRTLS

WKDVQWLQTITSLPILVKGVLTAEDARLAVQAGAAGIIVSNHGARQLDYVPSTIMALEEVVKAAQGPVFL

DGGVRRGTDVFKALALGASGIFIGRPVVFSLAAEGEAGIKKVLQMLRDEFELTMALSGCRSLNEITRNHI

VTE

>A9PJK1|A9PJK1_9ROSI

EITNVMEYEAIAKQKLPKMVFDYYASGAEDQWTLAENRNAFSRILFRPRILIDVSKIDMATTVLGFKISM

PIMIAPTAMQKMAHPEGEYATARAASAAGTIMTLSSWATSSVEEVASTGPGIRFFQLYVYDRNVVAQLVR

RAERAGFKAIALTVDTPRLGRRESDIKNRFSLPPFLTLKNFEGLDLGKMDK-ADSGLASYVAGQIDRTLS

WKDVEWLQTITRLPILVKGVLTAEDARLSVQAGAAGIIVSNHGARQLDYVPSTIMALEEVVKAAQGPVFL

DGGVRRGTDVFKALALGASGIFIGRPVVFSLASEGEAGVRKVLQMLREEFELTMALSGCRSLKEITRDHI

VAD

>A5B9Z0|A5B9Z0_VITVI

EITNVTEYEAIAKAKLPKMAFDYYASGAEDQWTLRENRNAFSRILFRPRILIDVSKIDMTTTVLGFKISM

PIMIAPTAFQKMAHPEGEYATARAASAAGTIMTLSSWATSSVEEVASTGPGIRFFQLYVYDRHVVAQLVR

RAERAGFKAIALTVDTPRLGRREDDIKNRFTLPPFLTLKNFEGLDLGKMDK-ADSGLASYVAGQIDRSLS

WKDVKWLQTITKLPILVKGVLTAEDARIAVNVGAAGIIVSNHGARQLDYVPATIMALEEVVKATQGPVFL

DGGVRRGTDVFKALALGASGIFIGRPVVYSLAADGEAGVRKALQMLRDEFELTMALSGCRSLKEIXRNHI

MTD

>F1CWA9|F1CWA9_MANIN

EITNVMEYEAIAKQKLPKMVFDYYASGAEDQWTLRENRFAFSRILFRPRILIDVSKIDMTTTVLGFKISM

PIMIAPTAMQKMAHPEGEYATARAASAAGTIMTLSSWATSSVEEVASTGPGIRFFQLYVYDRNVVAQLVR

RAERAGFKAIALTVDTPRLGRREADIKNRFTLPPFLTLRNFEGLDLGKMDQ-ANSGLASYVAGQIDRSLS

WKDVKWLQTITKLPILVKGVLTAEDARLAIQAGAAGIIVSNHGARQLDYVPATIMALEEVVKASQGPVFL

DGGVRRGTDVFKALALGASGIFIGRPVVFSLAADGEAGIRKALQMLRDEFELTMALSGCRSLKEITRDHI

VTD

>A5B1R1|A5B1R1_VITVI

EITNVTEYEAIAKQKLPKMVFDYYASGAEDQWTLYQNRHAFSQILFRPRILIDVSKIDMTTTVLGFKISM

PIMIAPTAMQKMAHPEGEYATARAASATGTIMTLSSWATSSVEEVASTGPGIRFFQLYVYDRHVVAQLVR

RAERAGFKAIALTVDTPRLGRREADIKNRFTLPPFLTLKNFEGLDLGKMDK-ADSGLASYVAGQIDRTLS

WKDVKWLQTITNLPILVKGVLTAEDTRLAIQAGAAGIIVSNHGARQLDYVPATIMALEEVVKAAQGPVFL

DGGVRRGTDVFKALALGASGIFIGRPVVFSLAAEGEAGVRKVLQMLREEFELTMALSGCRSLKEITRDHI

EWE

>B8B7C5|GLO5_ORYSI

EITNVTEYQAIAKQKLPKMIYDYYASGAEDEWTLQENREAFARILFRPRILIDVSKIDMATTVLGFKISM

PIMIAPSAMQKMAHPDGEYATARAASAAGTIMTLSSWATSSVEEVASTGPGIRFFQLYVYDRRVVEQLVR

RAERAGFKAIALTVDTPRLGRREADIKNRFVLPPFLTLKNFEGLELGKMDQ-ASSGLASYVAGQIDRTLS

WKDVKWLQTITTLPILVKGVITAEDTRLAVENGAAGIIVSNHGARQLDYVPATISALEEVVKAARGPVFL

DGGVRRGTDVFKALALGAAGVFIGRPVVFSLAAAGEAGVRNVLQMLRDEFELTMALSGCTSLADITRNHV

ITE

>C0P702|C0P702_MAIZE

EITNVMEYQAIAKQKLPKMAYDYYASGAEDEWTLQENREAFSRILFRPRILIDVSKIDMTTTVLGFKISM

PIMVAPTAMQKMAHPDGENATARAAAAAGTIMTLSSWATSSVEEVASTGPGIRFFQLYVYDRKVVEQLVR

RAERAGFKAIALTVDTPRLGRREADIKNRFVLPPHLTLKNFE--DLGKMDQ-AASGLASYVAGQVDRTLS

WKDVKWLQTITTLPILVKGVLTAEDTRLAVANGAAGIIVSNHGARQLDYVPATISALEEVVKAARGPVFV

DGGVRRGTDVFKALALGAAGVFVGRPVVFSLAAAGEAGVSNVLRMLRDEFELTMALSGCTSLAEITRKHI

ITE

>F2EB29|F2EB29_HORVD

TITNVSEYQAIAKQKLPKMAYDYYASGAEDEWTLKENREAFSRILFRPRILIDVSTIDMTTSVLGMKMSM

PIMISPTAFQKMAHPEGEYATARAASAAGTVMTLSSWATSSVEEVASTGPGIRFFQLYVYNRKVVAQLVK

RAEKAGFKAIALTVDTPRLGRREADIKNRFVLPPGLTLKNFEGLDLGTMDQ-ANSGLASYVAGQIDRTLS

WKDVKWLQSITTMPILVKGVITAEDARLAVHSGAAGIIVSNHGARQLDYVPATISALEEVVTAAQGPVYL

DGGVRRGTDVFKALALGASGVFIGRPVVFALAAEGEAGVRNVLRMMREEFELTMALGGCTKLSDITREHI

FTE

>B8AKX6|GLO1_ORYSI

EITNVMEYQAIAKQKLPKMIYDYYASGAEDEWTLKENREAFSRILFRPRILIDVSKIDMSATVLGFKISM

PIMIAPSAMQKMAHPDGEYATARAASAAGTIMTLSSWATSSVEEVASTGPGIRFFQLYVYDRNVVEQLVR

RAERAGFKAIALTVDTPRLGRREADIKNRFVLPPYLTLKNFEGLDLAEMDK-SNSGLASYVAGQIDRTLS

WKDVKWLQSITSLPILVKGVITAEDARLAVHSGAAGIIVSNHGARQLDYVPATISALEEVVTAAAGPVYL

DGGVRRGTDVFKALALGAAGVFIGRPVVFALAAEGEAGVRNVLRMMREEFELTMALSGCTSLADITRAHI

YTD

>C5WY71|C5WY71_SORBI

EITNVMEYQAIAKQKLPKMAYDYYASGAEDEWTLKENREAFSRILFRPRILIDVSKIDMTTSVLGFKISM

PIMVAPTAMQKMAHPDGEYATARAASAAGTIMTLSSWATSSVEEVASTGPGIRFFQLYVHDRKVVEQLVR

RAERAGFKAIALTVDTPRLGRREADIKNRFVLPPHLTLKNFE--DLGKMDQ-ANSGLASYVAGQIDRTLS

WKDVKWLQSITSMPILVKGVVTAEDARLAVHSGAAGIIVSNHGARQLDYVPATISALEEVVKAAQGPVYL

DGGVRRGTDVFKALALGAAGIFVGRPVVFALAAEGEAGVRNVLRMLRDEFELTMALSGCTTLADINRSHV

EGD

>A3K9S1|A3K9S1_9RHOB

RAASIDDLRAMARRRIPRFAFDLVDGGAESERNMRRNCTAFEEVELVPRYMVDVSSIDTRTELFGQTYDA

PFGMAPIGMLNAFWPGADLSLARLCKRQNLPYVASSAASTTLEALAEAADGNGWFQLYVSDDTVTEGLVA

RAEAAGYDVMIVTADVPAAGKRDRDIRNRLAVPFRITPEVALKPNIANYAD-LLTSYADVQKTLITPAFN

WEALKRLRDRWGGKLLVKGILHPDDAARCTEAGCDGIVVSNHGGRQVAFGPATADVLPAIAEAVAGKVIV

DSGIRRGADMMRAKALGADFTLTGRALAFGVGAGGAPGAARAVEILELELVRALGQLGVPRFADVGPEHL

AVS

>B7RQK5|B7RQK5_9RHOB

RAASIDDLRTRAKRRIPRFAFDLVDGGAESERNLRRNIEAFEEVELTPRYMVDVSDIDTRATLFGQTYNL

PFGMAPIGMHNAFWPDADLILARLCARENIPYTASSASSTTLERLAEAAAGNGWFQLYVSDPSVTEGLIA

RAEAAEYKVMMVTADVPAAGKRDRDIRNQLAVPFKITPEVVARPNIANYAD-LLTSYADVQKTLITPGFT

WDDLKRLRDRWKGTLLVKGILHPSDAAKCAELGCDGIIVSNHGGRQVAFGPPTIEALPPIADVLGGKIIL

DSGIRRGADILRAKAHGADFALTGRAMAYGVGAGGAAGAQRAKEILHLELVRALGQLGITSFETVDAAGL

TAV

>C4U4W7|C4U4W7_YERAL

NAWNIDDLRKITKSNVPSIFYDYLIGGASSESTLKSNVDDFALWELKQRVLSGITDVDLSVHLLGNTHKL

PVMLGPVGFAGMYYKDGEIEVSYAADKMGIPQCLSTFSICSMEDVASVRKGPLYSQLYIFHRELTLDMLE

RCKKIGIDTIFITIDTPYTPVRERDERNGFRSP-VPSAKMILAHGVPKVH----EKLGSWSGREIDPTLT

WDDIRWFREQWKGKLVVKGILSAQDAQLAADAGADAIVVSNHGGRQLDPASSTIRRLPEIKNALGDEIIF

DGGIRRGSDIIKAIALGANCVSLGRAYIYGLGAGGEKGVLRSIEILKNEMEPALKMMGFKSINELGPEAL

HFL

>A3NMK2|A3NMK2_BURP6

KAWNIDDLRKMARKRVPKYFFDYLDGGANSETTMRANENDFARWRLRQKVLTGAQSRGLGATYLGAEHRL

PILLGPVGFAGMYWSRGEIAAGRAADDAGIGQCLSTFSICSLEDVAAARSGPLYFQLYMFDRDLTEDILA

RCRLANVDAVFLTVDTCYIPIRERDARNGFRADTRLSARGVWPR-IGNVLR-YPEQSAAV-GRMIETRLS

WADVKWLRARWPGRIVIKGILDPDDARRAVDEGVDGILISNHGGRQLDPAPSVMDVLPEIAEAVGKEILM

DGGVRRGADVIKALALGASAVSIGRAYVYGLGAAGEKGVSRCLELLKGEMLPALNMMGFESIAELGKSAL

TAA

>B0TZG8|B0TZG8_FRAP2

NIYDITDMHKAAKKRLPRVFLDYIDSGSYQQQTVYENEQAFRKIRINQSAFKDCSERNQAIEIFGFKSSV

PFAIAPIGMAGMFWPKGEIALAQAAEKLDIAYTMSTMAICSLETVRDEVNNPFWFQLYLMDRGFIKSLLE

RAKVSGCKTIFVNADLPVSGIRYSDMRNGLSIPPKFGIRDLIYKQFGNLSG-HIKSVTDFMDSQFDQSVT

WKDVEWLRSIWDGNLIIKGLLNTQGAENAVKVGADGIVVSNHGGRQLDGVLPTIEALPAIAEKVKGKIIL

DSGIRSGQDVIKALALGADFTLVGRPFLYGLSAFGQKGVEKVYNILKKEIDNTMALAGITDLNNISTDIV

---

>B2SH40|B2SH40_FRATM

NIYDITDMHKAAKKRLPRVFLDYIDSGLYQQQTVYENEQAFRKIRINQSAFKDCSRRNQTIEIFGFKSSV

PFAIAPTGLAGMFWPKGEIALALAAEKLDIAYTMSTMAICSLETVAKEANNHFWFQFYLMDRGFTKSLLE

RAKACGCQTIFVNADLPVSGIRYSDMRNGLSIPPKFGIRDIIYKQFGNLSG-HIKSVTDFMDSQFDQSVT

WKDIEWLRNIWDGNLIIKGLLNTQGAENAVKVGVDGIVVSNHGGRQLDGVLPTIEALPAIADKVKGKIIL

DSGIRSGQDIIKALALGADFTLVGRPFLYGLSAFGQKGVEKVYDILKKEIDNTMALAGISDLNNISTDVV

---

>B0U110|B0U110_FRAP2

KITSLDDMRKIYHRRVPKMFVDYCESGSWQQKTLEHNQKDFDKYFFRQKVLTDIQHRSLKTKILGQEYSM

PLAFAPVGLLGMQHADGEIHAAKAAEEFGIPFTLSTMSICSTEEVAKHTTKPFWFQLYMMDRKFMANLIA

SAKHAGCSALVLTADLQMLGNRHADIKNGLTVPPKPTLKNLINRTFGNIAN-HAASLGKWTNEQFDLSLN

WHDVEWVQKQWNGPMIIKGIMDTQDAIMAQNTGADAIVVSNHGGRQLDGAPSSISMLEEIVDAVDPEVLI

DSGIRSGQDLLKAKALGAKAGLIGRPMVYGLGAYGEQGAYRVLEIFHQEMDKTMAFCGFTDINNVDKSIL

---

>A7YRM3|A7YRM3_FRATU

KITSLDDMRKVYHRRVPKMFVDYCEAGSWQQQTLKYNQQDFGNYLFRQKVLTDIQNRSLKTKILGQEYKM

PLVFAPIGLLGMQHADGEIHAARAAEKFGIPFTLSTMSICSTEEVAKHTTKPFWFQLYMMDRKFMANLIA

SAKHAGCSALVLTADLQMLGDRHADIKNGLTVPPKPTLKNLINRTFGNIVN-HAASLGKWTNEQFDLSLN

WHDVEWVQKQWNGRMIIKGIMDTQDAIMAQNTGADAIVVSNHGGRQLNGAPSSISVLEEIIDAVDREVLI

DSGIRTGQDLLKAKALGATAGLIGRPMVYGLGAYGEQGAYRVLEIFYQEMDKTMAFCGHTNINNVDKSIL

KRN

>B6JIM0|B6JIM0_OLICO

NITCIDDLRDLHMRRVPKAFFDYCDRGSYTESTLRANREDLDRIKFRQRILVDVASRSLNTTILGEPAAM

PMILAPVGLTGMQHGDGEIYACRAAHEAGIPYTLSTMSICSIEDVAANVKKPFWFQLYMMDRGFMKSLIE

RAIAAKCSALVLTVDLQVIGQRHADIKNGMTVPPQLKLRTLYRKTFGNLAG-QMTSLSEWISTQFDPSLS

WKDIEWIRNIWPGKMVIKGILDIVDAREAVRTGAEALVVSNHGGRQLDGAPSSISVLPEIVQELGSEIMF

DGGIRTGQDILRALAFGAKSCMIGRAYVHGLGAGGQAGVAKAIDILAKELSTTMGLCGINRVEDIDRRIL

RNN

>B3Q6Z1|B3Q6Z1_RHOPT

EITCIEDLRQIHKRRVPKMFFDYVDHGSYAEETLRANVDDLKRIKFRQRILVDISKRDLATTILGDTYAM

PLILAPVGSTGMQHADGEIHACRAAQAAGIPYTLSTMSICSIEDVAANVEKPFWFQLYVMDRGFAKALIE

RAIAAKCSALVLTVDLQVIGQRHQDIKNGMTVPPQFKLKNVIRRNFGNIAG-HLESVSAWVASQFDASLN

WRDIDWIRSIWPGKLIIKGILDVEDAREAVKVGAEALVVSNHGGRQLDGAPSSIEVLPEIVHTVGSEVMF

DGGIRSGQDVMRALALGAKSCMIGRAYIYGLGAYGGPGVAKAIDIIGKELSTTMGLCGVNSIHEIDEKVL

-AD

>A4Z0D9|A4Z0D9_BRASO

HVTCIEDLRLLHKRRVPKAFFDYADRGSYAEETLRANRDDLQKIKFRQRILVDVSKRDLSTTILGEPSSM

PLVLAPVGLLGMQHGDGEIHACRAAQAAGIPFTQSTMSICSIEDIASSVEKPFWFQLYVMDRGFIKALIE

RAIAAKCTALCLTVDLQVIGQRHQDIKNGMSVPPEWSLSKLFRRTFGNIAG-HVTKLSAWTASQFDTSLN

WKDVDWIRSIWPGKLIIKGIHDIEDAKLAAATGAQAMVVSNHGGRQLDGAPSSIHVLPGIAEAVGDEIMF

DGGIRSGQDVMRALALGAKSCMIGRAYAYGLGAGGQVGVAKAIDIIRNELLTTMGLCGVNTVAEIDRKVL

-AD

>A8I5E1|A8I5E1_AZOC5

PVTNIADLRDIARRRIPRAMFEYAQRGSYDERTLAANYAELDALRLRQRVMVDVSKRNVATTFLGQDVTI

PVGIAPTGLTGLFHADGEILGARAAQAFGVPFTLSTMSICSIEDVAGAVDKPFWFQLYVMDRAFTQSLVE

RARAAGCPVLVLTLDLAAHGQRHRDIKNGLSVPPRLTLANALRRSFGNLQG-WMNAMAQWVAQQFDPSLS

WKDVAWIRSLWPGKLVLKGILDPEDARIAADHGADAIVVSNHGGRQLDSAPASISVLPEIASAVGSEILL

DGGIRTGQDVLKALALGARGCLIGRSWLYGLAAGGQGGVTQVLEILRKELDTSMALAGLTDVRSVTPAAL

PRR

>A1BC78|A1BC78_PARDP

VITEIEDLRRLHRRRVPRMFYDYVDVGAWTGGTYRANRADFERILFRQRVARNIEARTLATTMLGQPVSM

PLALAPVGLLGMQHPDGEIYAARAAQAAGVPFTLSTMSMCSLEDIAQATGAPFWFQLYTLDEEFLEDILD

RARRAGVTALVLTLDLTIQGQRHKDLKNRMTAPPRLTLPNLIRRSFGNIVG-HAGDLMDWTARQFDQQLD

WGRVEQIIRKWGGPVILKGINDPEDAQRALDTGCDAILVSNHGGRQLDGAPSTIRALPAIRRAVGPPLYL

DSGIQSGQEALKAIASGANGVFVGRAFTYGLGAMGQRGVEAALAILRREMDITMALCGVNDIKDFGPGCL

PDD

>A3TV76|A3TV76_9RHOB

VITCIDDLRELHRRRTPKMFFDYCESGSWTEQTFRENTADFQDIRLRQRVAVNMEGRTLATRMIGQEVAM

PVALAPVGLTGMQRADGEIKAARAAEKAGVPFTLSTMSICSIEDVAEHTTKPFWFQLYTMDQDYLRRLIQ

RAKDAKCSALVITLDLQILGQRHKDLKNGLSAPPRLTPATIARRKFGNIVG-HVSRLGEWTAQQFDQKLD

WKKIEEIKKLWGGPVILKGILDPEDAILARKVGCDAIVVSNHGGRQQDGAISSIRALDPILQAVGDEVHI

DSGIRSGQDVLRAIAMGARGTYIGRPWVYGLGAMGEAGVTRALEVIRNELDIAMAFTGKRDIENVDRSCI

PAG

>A3JW19|A3JW19_9RHOB

TITNIYDLKKIYKRRAPKMFYDYTESGSWTEQTFRENVTDFDHIRLRQRVAVDMSGRSTASTMIGEDVAM

PVALAPIGITGMQCADGEIKSARAAEAFGVPYTLTTMSVNSIEQVAEATEKPFWFQLYVMDENFVDTMIE

RAKAAKCSALVLTLDLQILGQRHMDIKNGLTTPPKPTLKNIISWTFGNIVG-HASSLSSWAAEQFDPTLD

WDKIKEIKKKWGGELILKGILDAEDAKKAINVGADAILVSNHGGRQLDGALSSIRSLAPILDAVNGEVFL

DSGIRSGQDVLKAMAMGADGVFIGRSYIYGLGAMGQKGVTTALEVIHKELDTTMALCGRRDVKTLDRSDL

PKN

>A1B9E7|A1B9E7_PARDP

VITCIDDLKAIYRRRVPRMFYDYAESGSYTEGTFRENCTDFQRIKLRQRVAVDMTGRTTESTMIGQKVAM

PVALAPVGMTGMQCADGEIKAARAAKAFGVPFTLSTMSICSIEDVAEAVQAPFWFQLYVMDQEFLEAIIE

RARRANCSALVLTLDLQILGQRHKDLKNGLSAPPRLTLPVLLRRFFGNIVG-HASSLIAWTAEQFDPQLD

WGKIARIRDLWGGKLILKGINDPEDARMAADFGADAIIVSNHGGRQLDGAVSSIRMLPEIVKAVGDEIHL

DSGIRSGQDVLKALAMGAHATHIGRAFIYGLGAMGEAGVTRALEVIQKELDITMALCGERDVKALGRHNL

PPD

>C8S2X0|C8S2X0_9RHOB

VITCIEDLKRIYKRRVPKMFYDYCESGSYTEQTFRDNVSDFSQLRLRQKVAVDMSDRSTASTMIGQPVAM

PVALSPVGLTGMQAADGEIKAARAAARFGVPFTLSTMSICSIEDVAAHSPAPFWFQLYVMDEDFVDAILE

RAKAAKCSALVLTLDLQILGQRHKDLKNGLSAPPKLTLPVLARRSFGNIVG-HASSLTSWTAEQFDTQLD

WTKIARIRDQWGGKLILKGILDADDARLAADFGADAIIVSNHGGRQLDGALSAIRMLPSIVAAVGEEVHM

DGGIRSGQDVLKALALGAKGTWIGRSYIYGLGAMGEAGVSKALEVIQKELDVSMALCGERDVKSLRRENL

PRD

>A4WPE2|A4WPE2_RHOS5

VITCIEDLKRMHRRRTPRMFFDYCESGSYSEQTFRDNCSDFQQIRLRQKVAVDMLGRSTRSTMIGQEVAM

PVALAPVGLTGMQCADGEIKAARAAEAFGVPYTLSTMSICSVEDVAAATTKPFWFQLYVMDEEFVDSMLE

RAKKAGCSALVLTLDLQILGQRHKDLKNGLSAPPKMTLPVMMRRTFGNIVG-HATSLASWTAEQFDPQLD

WGKIARLRDKWGGKLILKGILDEEDARRAADFGADAIIVSNHGGRQLDGALSSIRMLPPIVRAVGDEIHM

DGGIRSGQDVLKALAMGAKGTYIGRSYIYGLGAMGEAGVRRALEVIWKELDVSMALCGEKDVKALGPHNL

PQD

>A3V9J4|A3V9J4_9RHOB

VITNIDDLKRLYRRRAPKMFYDYCESGSWTEQTFRENVSDFDAIRLRQRVAVDMSNRTTKTQMIGQDVAM

PVALAPVGMTGMQSADGEIKAARAAEKFGVPFTLSTMSICSIEDVAEHTTAPFWFQLYVMDEDYLSRLIQ

RTKDAGCSALVITLDLQIMGQRHKDIRNGLSAPPKPTVRNLVRRSFGNVVG-HVSKLMSWTNDQFDPSLD

WSKIEKIKEMWGGPLILKGVMEAEDAVMAAKVGADAIIVSNHGGRQLDGALSSIRALDPILQAVGDEVHL

DSGIRSGQDVLKAMAMGAKGTYIGRAFVYGLGAMGEAGVTRALEVIHKELDTSMAFCGHRDINQVDRDIL

PRD

>B5IYX1|B5IYX1_9RHOB

VITTIDDLKRLHKRRTPKMFYDYCESGSWTEQTFRENTSDFDKIRLRQRVAVDMANRTTASQMIGQDVAM

PVALAPVGLTGMQCADGEIKAAKAAEKFGVPFTLSTMSICSIEDVADNTTKPFWFQVYTLDDDFMQRLFD

RAKAAKCSAIVITLDLQILGQRHKDLKNGLTAPPKFTIPTLLRRFFGNIVG-HASSLATWSAEAFDPSLN

WDRVAQLMKMWGGPVILKGILDVEDAKKAADLGADAIIVSNHGGRQLDGALSSIRMLEQIVDAVGDEVHF

DSGIRSGQDVLKAIALGAKGTYIGRAFVNGLGAMGEAGVTKALDVIHTELDLTMALCGHRDIKGVNKDIL

PDD

>B5K599|B5K599_9RHOB

VITTIADLKRLHKRRTPKMFYDYCESGSWSEQTFRENTSDFDKIRFRQRIAVDMTNRTTASQMIGQDVAM

PVALAPVGLTGMQCADGEIKAAKAAEKFGVPFTLSTMSICSIEDVAENTTKPFWFQVYTLDDDFMQRLFD

RAKAAKCSAIVITLDLQILGQRHKDLKNGLTAPPKFTIPTMLRRFFGNIVG-HASSLASWSAEAFDHSLN

WDRVAQLMKMWGGPVILKGILDVDDAKKAAELGADAIIVSNHGGRQLDGALSSIRMLEQIVDAVGDEVHF

DSGIRSGQDVLKALALGAKGTYIGRAFVNGLGAMGEAGVTKALDVIHSELDLTMAFCGHRDIKSVDKNIL

PED

>A3V3C5|A3V3C5_9RHOB

VITTIADLKRLHKRRTPKMFYDYAESGSWTEQTFRENTSDFDLIRLRQRIAVDMTNRTTQSQMIGQDVAM

PVALAPVGLTGMQSADGEIKAAKAAEKFGVPFTLSTMSICSIEDVAENTTKPFWFQVYTLDDDFMQRLFD

RARAAGCSAIVITLDLQILGQRHKDLKNGLSAPPKFTLASMARRFFGNIVG-HASSLSSWTAEAFDHALD

WDRVAQLMKMWGGKVILKGILDADDARKAAALGADAIIVSNHGGRQLDGAVSSIRALPAILDAVGDEVHF

DSGIRSGQDVLKALALGAKGTYIGRAYINGLGAMGEAGVTRALEVIHKELDTTMALCGRRDIRTVDRDIL

PKD

>A4EIP4|A4EIP4_9RHOB

VITTIADLKRLHKRRTPKMFYDYAESGSWTEQTFRENTSDFDLIRLRQRIAVDMTNRTTASQMIGQDVSM

PVALAPVGLTGMQCADGEIKAARAAEKFGVPFTLSTMSICSIEDVAAKTNKPFWFQVYTLDNDFMQRLFD

RARDAWCSAMVITLDLQIMGQRHKDLKNGLSAPPKFTLASMARRFFGNIVG-HASSLASWTAEAFDPALD

WNRVAELMKMWGGPVILKGILDVEDAKKAAELGADAIIVSNHGGRQLDGALSSIRALPAIMDAVGDEVHL

DSGIRSGQDVLKALAMGAKGTYIGRAFVNGLGAMGEKGVKTALEVIHKELDTTMALCGRRDVKELDRDIL

PEN

>A8LRY6|A8LRY6_DINSH

VITEIEDLKRIYKRRVPKMFYDYAESGSWTEQTFRENSSDFDLLKLRQRIAMDMDNRSTKTTMVGQEVAM

PVALAPVGLTGMQHADGEIKAARAAEKFGVPFTLSTMSICSIEDVAAHTETPFWFQVYTLDDDFMKRLFD

RAKEAKCSALVITVDLQLLGQRHRDLKNGLSAPPKLTPASIARRFFGNIVG-HASSLSSWTAEAFDQSLD

WERIKQFRSWWDGPVILKGILDPEDAKMALNVGADAIVCSNHGGRQLDGALSSIRMLPQIMDAVGDEVHL

DSGIRSGQDVLKAVALGARGTMIGRAWTYGLGAMGEAGVTRALEVIHKELDLSMGLCGRRSVEDLDASNL

PED

>A3K8A7|A3K8A7_9RHOB

VITNIEDLKRIYRRRVPKMFYDYCESGSWSEQTFRENVSDFDDIYLRQRVAIDMANRSTKTQMIGQDVAM

PVALAPVGLTGMQNADGEIKAARAAEAFGVPYCLSTMSICSIEDVASHTSKPFWLQVYTLDNDFMQGLFD

RAKEAKCSAAVITVDLQLLGQRHKDLKNGLSAPPKLTPKSVARRFFGNIVG-HASSLTTWTAESFDQSLN

WDRIREFRRMWDGPLIIKGIIDPRDALEACNVGADAIVVSNHGGRQLDGALSSIRALEPIVDAVGDEVHL

DSGIRSGQDVLKAVAMGAKGCWIGRAYVYGLGAMGQAGVTKALEVIHKELDTSMALCGHRNVNTVDRDIL

PKG

>D0D3E6|D0D3E6_9RHOB

VITEIEDLKRIYKRRVPRMFYDYCESGSWTEQTFRENSSDFDDLYLRQRVAIDMTGRSTATQLIGQDVAM

PVALAPVGLTGMQHADGEILAAKAAETFGVPYTLSTMSICSIEDVAEHTSKPFWLQVYTLDDDFMQRLFD

RAKDAKCSAAVITVDLQMLGQRHKDIKNGLSAPPKLTARSILRRFFGNIVG-HASSLSTWTAESFDQALN

WDRIREFRKMWDGPLIIKGILDPRDALEALNVGADAIVVSNHGGRQLDGALSSIRALGPIMDAVGDEVHL

DSGVRSGQDVLKAVAMGAKGCWIGRAYIYGLGAMGEKGVSEALRVIHKELDTSMGLCGRTDINAVNRDIL

PKG

>A3SLV7|A3SLV7_9RHOB

VITNIDDLKRIYRRRVPKMFYDYAESGSWTEQTFRANTTDFSELLLRQRIAVDMGNRTTRTQMIGQDVSM

PVALAPVGVTGMQCADGEIKAARAAEAFGVPFTLSTMSICSIEDVAEHTEKSFWFQVYTLDDDFMQRLFD

RAKAAQCSAAMITVDLQVLGQRHKDIKNGLSAPPKLTPKTVARRFFGNIVG-HASSLSSWTAEAFDVSLD

WDRIRTFRKMWDGPLIIKGIIDERDALEALNVGADAIIVSNHGGRQLDGALSAIRALPRIMDAVGDEVHL

DSGIRSGQDVLKAVAMGAKGTYIGRAFVYGLGAMGEAGVTRALEVIHKELDVSMAFCGHRDIKTVDRDIL

PRD

>A6FVD2|A6FVD2_9RHOB

VITSIADLKRIYHRRVPKMFFDYAESGSWTEQTFRENTSDFSDIYLRQRVAVDMDGRSTASQMIGQDVAM

PVGLAPVGLTGMQHADGEIKAARAAGKFGVPYTLSTMSICSIEDVAENTDKPFWMQVYTLDDDFMQRLFD

RAKAANCSAAMITVDLQVLGQRHKDLKNGLSAPPKLTPASVARRFFGNIVG-HASSLSTWTSEAFDPSLN

WDRIREFRKMWDGPLIIKGIMDPRDAREALNVGADAIIVSNHGGRQLDGALSAIRALPAILDAVGDEVHI

DSGIRTGQDVLKALAMGAKGTYIGRAYVYGLGAMGEAGVTRALEVIHKELDVSMALCGRRDVRTLDRDIL

PEG

>A3W851|A3W851_9RHOB

VITNIADLKQIYRRRVPKMFYDYAESGSWTEQTFRENTTDFDKIYLRQRVAIDMTGRSTASQMIGQDVAM

PVGLAPVGLTGMQHADGEIKAARAAEKFGVPFTLSTMSICSIEDVAENTTKPFWMQVYTLDDDFMQRLFD

RARDAKCSAAMITVDLQLLGQRHKDLKNGLSAPPKLTPKSVARRFFGNIVG-HASSLSTWTSEAFDQALD

WDRIRQFRKMWDGPLIIKGIMDPRDAREALNVGADAIIVSNHGGRQLDGALSAIRALPAIMDAVGDEVHL

DSGIRSGQDVLKALSLGAKGTYIGRAFVYGLGSMGEAGVTRALEVIHKELDSSMGLCGRRAVKDLDRDIL

PED

>C7DAF4|C7DAF4_9RHOB

VITSIDDLKRIYKRRAPKMFYDYAESGSWTEQTFRENTSDFDQIRLRQRVAVDMQDRSTATQMIGEDVAM

PVALAPVGLTGMQSADGEIKAARAAEKFGVPFTLSTMSICSLEDIAEHTTKPFWFQLYVMDADFVADMIA

RAKDVGCSALVLTLDLQILGQRHKDLKNGLSAPPKLTPKTIARRTFGNIVG-HASSLSSWTEEQFDPRLD

WDKVARLKEQWGGKLILKGILDAEDAKMAAKIGADAIIVSNHGGRQLDGALSSIRMLPSIIDAVGPEVHL

DSGIRSGQDVLKAMAMGAKGTYIGRAYIYGLGAMGEHGVSEALRVIHTELDTTMALCGHKNINQVDRDIL

PKD

>B6B3Z4|B6B3Z4_9RHOB

VITEINDLKRIYERRVPRMFFDYTESGSWTEQTFRENSSDFDQLRLRQRVAVDMTGRSTASQMIGQDVAM

PVALAPVGLTGMQHADGEIKAAKAAEAFGVPFTLSTMSICSIEAVAERTSKPFWFQLYAMDEDYVRRLVE

RAKAAKCSALVITLDLQILGQRHKDLKNGLSAPPKLTPKTIANREFGNIVG-HVSQLSSWTAEQFDPALD

WDKIAKIKEMWGGKVILKGILDAEDAKMALKVGADAIVVSNHGGRQLDGAMSSIKMLQSILDAVGDEVHM

DGGIRSGQDVLKALAMGAKGTYIGRAFIYGLGAMGQAGVTKALEVIHKELDTSMALCGKRNVGELTNDAL

PKD

>A9DQX7|A9DQX7_9RHOB

VITSIDDLKRIYKRRVPQMFYDYAESGSWTEQTFRENSSDFDQIRLRQRVAVDMSGRSTATQMIGEDVAM

PVALAPVGLTGMQCADGEIKAARAAEAFGVPFTLSTMSINSIEDVAEATTKPFWFQLYTMDQDYVSRLIQ

RAKDAKCSALVITLDLQILGQRHKDLKNGLSAPPKLTAKTIARRHFGNIVG-HVADLGAWTAEQFDPSLD

WDKIAKIKEQWGGKVILKGILDAEDARMALKVGADAIIVSNHGGRQLDGALSSIRMLPEILDAVGDEVHL

DGGIRSGQDVLKAMAMGAKGTYIGRAFIYGLGAMGQQGVTRALEVIHRELDLTMALCGETQVANLGRHNL

PRN

>B7RJA2|B7RJA2_9RHOB

VITNIQDLKRLHERRVPRMFYDYAESGSWTEQTFRENTSDFDKIRLRQRVAVDMSGRTTKTQMIGQDVAM

PVALAPVGLTGMQHADGEIKAARAAEAFGVPYTLSTMSINSIEDVAEATTKPFWFQLYTMDEDYVSRLIQ

RAKDAKCSALVITLDLQILGQRHKDLKNGLSAPPKLTAKTIARRTFGNIVG-HVANLGAWTAEQFDPSLD

WGKIAKLKEQWGGKVILKGILDAEDAKMALQVGADAIIVSNHGGRQLDGAISSISALPSILDAVGDEVHL

DSGIRSGQDVLKAMAMGASGTFIGRAYIYGLGAMGQKGVTTALEVIHKELDLTMALCGETSVKDLGKHNL

PKD

>A3SEZ0|A3SEZ0_9RHOB

VITTIDDLKRLHERRVPRMFYDYAESGSWTEQTFRENTTDFEQIRLRQRVAVDMSGRSTKTQMIGQDVAM

PVALAPVGLTGMQHADGEIKAARAAEAFGVPFTLSTMSINSIEDVAEATTKPFWFQLYTMDEDYVARLIQ

RAKDAKCSALVITLDLQILGQRHKDLKNGLSAPPKLTAKTLARRSFGNIVG-HVANLGAWTAEQFDPTLD

WGKIAKLKEQWGGKVILKGILDADDARMALKVGADAIIVSNHGGRQLDGAISSIRALPSILEAVGDEVHL

DSGIRSGQDVLKAIAMGAKGTYIGRAFIYGLGAMGQAGVTSALEVIHKELDLSMALCGETSVAGLGKHNL

PKG

>A3X3D1|A3X3D1_9RHOB

VITNIQDLKRIYERRVPRMFFDYAESGSWTEQTFRENTSDFEQIRLRQRVAVDMSGRSTAAKMIGEDVSM

PVALAPVGLTGMQHADGEIKAAKAAEAFGVPFTLSTMSINSIEDVAEATSKPFWFQLYTMDEDYVRRLIQ

RAKDANCSALVITLDLQILGQRHKDLKNGLSAPPKLTPKTIARREFGNIVG-HVSSLGAWTSEQFDLSLD

WGKIAKLKEMWGGKVILKGILDAEDAKMAVKVGADAIVVSNHGGRQLDGALSSIRMLPQILDAVGGEVIL

DSGIRSGQDVLKSLAMGADGTMIGRAFVYGLGAMGQKGVTTALEVIQKELDTTMALCGERSVENLGRHNL

PKD

>B9NQA7|B9NQA7_9RHOB

VITNIQDLKRIYERRVPRMFFDYAESGSWTEQTFRENSSDFEDIRLRQRVAVDMTGRSTASQMIGQDVSM

PVALAPVGLTGMQHADGEMKAAKAAEEFGVPFTLSTMSINSIEDVAEYTNAPFWFQLYTMDEDYIRRLIQ

RAKDAKCSALVITLDLQILGQRHKDLKNGLSAPPKLTPKTIARREFGNIVG-HVSSLGTWTAEQFDPSLD

WKKVEKLMEQWGGKVILKGILDADDAKMAAKLGADAIVVSNHGGRQLDGALSSIRVLPEIMDAVGGEVHL

DSGIRSGQDVLKALALGAKGTYIGRAFVYGLGAMGQKGVTTALEIIQRELDTTMALCGERNVTKLGRHNL

PEN

>D0CXJ9|D0CXJ9_9RHOB

VITNIDDLKRIYERRVPRMFYDYAESGSWTEQTFRDNSADFHDIRLRQKVAVDMSGRSTASQMIGQDVAM

PVALAPVGLTGMQHADGEMKAAKAALDFGVPFTLSTMSINSIEDVAEHTGAPFWFQLYTMDEDYVRRLIQ

RAKDANCSALVITLDLQILGQRHKDLKNGLSAPPKLTAKTIARREFGNIVG-HVSSLGAWTAEQFDPSLD

WKKIEKLMEQWGGKVILKGILDPEDAKMAAKLGADAIVVSNHGGRQLDGALSSIRMLPRIMDAVGGEVHL

DSGIRSGQDVLKALALGAKGTYIGRAFVYGLGAMGQKGVTTALEVIQKELDTTMALCGERNVADLGPHNL

PQD

>A4EWQ6|A4EWQ6_9RHOB

VITNIEDLKRIYQRRVPRMFYDYAESGSWTEQTFRENTSDFEKIRLRQRVAVDMSGRSTQSQMIGQDVAM

PVALAPVGLTGMQHADGEIKAAKAAEEFGVPFTLSTMSINSIEEVAEATSKPFWFQLYTMDEDYIRRLMQ

RAKDAKCSALVITLDLQILGQRHKDLKNGLSAPPKLTPSTVARRNFGNIVG-HVSQLGAWTAEQFDPSLD

WGKVEKLMEMWGGKVILKGILDAEDAKMAAKLGADAIVVSNHGGRQLDGALSSIRMLPQILDAVGDEVHL

DSGIRSGQDVLKALAMGAKGTMIGRAFVYGLGAMGQQGVTEALNVIHKELDTTMALCGERELGNLGRHNL

PDD

>B6B5H1|B6B5H1_9RHOB

VITNINDLKRIYERRVPRMFYDYAESGSWTEQTFRENTSDFEKIRLRQRVAVDMSGRTTASRMIGQDVAM

PVALAPVGLTGMQHADGEIKAARAAEDFGVPFTLSTMSINSIEEVAEATSKPFWFQLYTMDEDYIRRLIQ

RAKDAKCSALVITLDLQILGQRHKDLKNGLSAPPKLTPKTIARRNFGNIVG-HVSQLGAWTAEQFDPALD

WGKVEKLMEMWGGKVILKGILDAEDARMAAKLGADAIVVSNHGGRQLDGALSSIRMLPEIVDAVGNEVHL

DSGIRSGQDVLKALALGAKGTMIGRAFVYGLGAMGQKGVTAALEVIRKELDTTMALCGERSVEGLGRHNL

PED

>C9CU18|C9CU18_9RHOB

VITNIEDLKRIYARRVPKMFYDYAESGSWTEQTFRENTTDFDQIRLRQRVAVDMAGRSTAAQMIGQNVAM

PVALAPVGLTGMQCADGEIKAARAAEAFGVPFTLSTMSINSIEEVAEATSKPFWFQLYTMDEDYVRRLIE

RAKAANCSALVITLDLQILGQRHKDLKNGLSAPPKLTPKTIARRNFGNIVG-HVSSLGAWTAEQFDPSLD

WGKIEKLKEMWGGKVILKGILDEEDAKMAAKVGADAITVSNHGGRQLDGALSSIRMLPRIMDAVGDEVHL

DSGIRSGQDVLKALALGATGTMIGRAFVYGLGAMGQKGVTRALEVIHKELDTSMALCGEKNVANLGRHNL

PED

>B7QQZ5|B7QQZ5_9RHOB

VITNIDDLKRIYERRVPRMFYDYAESGSWTEQTFRDNTNDFEKIRLRQRVAVDMAGRSTATQMIGQDVTM

PVALAPVGLTGMQHADGEIKAARAAEAFGVPFTLSTMSINSIEDVAEATTKPFWFQLYTMDEDYVRRLIQ

RAKDARCSALVITLDLQILGQRHKDLKNGLSAPPKLTPKTIARRNFGNIVG-HVSSLGAWTAEQFDPSLD

WGKIEKLMEMWGGKVILKGILDVEDAKMAAKLGADAIVVSNHGGRQLDGALSSIQMLPAIMDAVGDEVHL

DSGIRSGQDILKAIALGAKGTMIGRAFVYGLGAMGQAGVTKALEVLHKELDTTMALCGEKTVHGLGRHNL

PED

>A9ERC2|A9ERC2_9RHOB

VITNINDLKRIYERRVPRMFYDYAESGSWTEQTFRDNTNDFEKIRLRQRVAVDMAGRSTASQMIGQDVSM

PVALAPVGLTGMQHADGEIKAARAAETFGVPFTLSTMSINSIEEVAEATTKPFWFQLYTMDDDYVRRLIQ

RAKDARCSALVITLDLQILGQRHKDLKNGLSAPPKLTPKTIARRNFGNIVG-HVSSLGAWTAEQFDPSLD

WSKIAKLIELWDGKVILKGILDVEDAKMAAKLGADAIVVSNHGGRQLDGALSSIQMLPAIIDAVGDEVHL

DSGIRSGQDVLKALALGAKGTMIGRAFVYGLGAMGQHGVTRALEVLHKELDTTMALCGEKSVADLGRHNL

PED

>A7GXF4|A7GXF4_CAMC5

LMTNIEDLRRVCYRNVPKMFYEYVDTGSWTQSTYRENHTDFEPIKFKQKILVDMANRSLETKLLGKTAKF

PAMTAPVGFMGMMWADGEIHMARAAQKFGIPFTLSTMSICSIEDLVEAGVEPFWFQLYVMDREFMKDLIR

RAKAANCSALVVTVDLQVLGNRHRDIKNGLSTPPKFTIPNLIRWTFRNIAG-HASSLSSWTKEQFDPSLQ

WSDIEEIKNLWGDKIILKGIMLPEDAQLAVKHGADAIIVSNHGGRQMDGTLSAIKALPDIVSAVGDEVWI

DSGFYSGQDMLKAWAMGARGIMLGRAPVYGLGAYGEDGVTRALQIMYDEMDTTMAFAGHRDIQNVTSDIL

PGT

>C2M7F3|C2M7F3_CAPGI

KMTNIEDLRVVCKRNVPKMFYEYVDTGSWTQSTYRENVSDFNPIKFKQRILVDMDNRTLETTLLGQKVKF

PAMTAPVGFMGMMWADGEIHMAKAAQKFGIPFTLSTMSICSIEDLVEAGVEPFWFQLYVMDREFMKDLIR

RAKEAKCSALMVTVDLQVLGNRHRDIKNGLSTPPKFTIPNILRWTFRNIAG-HASSLSSWTKEQFDPSLS

WKDIAEIKELWGGPIILKGIMTPEDAQEAVKYGADAIIVSNHGGRQMDDTISSIKALPDIVSAVGSEVWI

DSGFYTGQNMLKAWALGAKGIMLGRAPVYGLGAYGEEGVTRALQILYDEMDTTMAFSGHRNIQDVDKSIL

EGT

>A4BLC2|A4BLC2_9GAMM

IITCIDDLQRLAKRRVPRMFYDYADSGSWTESTYRANQDDFSALKLRQRVMVDISQRSLRSTLLGRSYRM

PVALAPIGLAGMQYPDGEIHAARAAETFGVPFTLSTMSICSIEDVAANTTQPFWFQLYMMDRDYIARLIK

RAEAARCSALVLTADLQILGQRHKDVRNGLTVPPRLTLENLIRRTFGNIAG-HVDSLSAWTAAQFDPSLS

WDDVAWIKARWGGKLIIKGIMEPEDAGAAIDAGADAIIVSNHGGRQLDGAPSSIRALPAIVAAVGHEVYM

DGGIRSGQDVLKAIALGAKAVFIGRAFLYGLGAMGEKGVTTCLDLIHRELDITLALCGLRNIRQVSEKVL

PGD

>A0NUY8|A0NUY8_9RHOB

VVTDVADMQALARRRVPKMFYDYADTGSWTQSTYRENEAALKRQKLRQRVACNIDNRSVKTTMVGQDVAM

PVALAPVGLTGMQHADGEILAAQAAEEFGVPFTLSTMSVCSIEDVAENTKNPFWFQLYVMDRGFSENLMQ

RATDAGCSALVLTLDLQVLGQRHKDLKNGLSTPPKPKPHVLLRRQFGNIHG-HVTSLAEWTNSQFDPTLD

WSSVEWVKSHWKRKLILKGINDVEDAKIAADVGADAIVVSNHGGRQLDGALASYEVLQDIVDAVGDEVHF

DSGIRSGQDVFKAVAMGAKSTYIGRAFIYGLGAMGKEGVSKVLQIMHKELDVTMGLCGETDINKVGRHNL

-VL

>B9R2J7|B9R2J7_9RHOB

IVTDVADMQALAKRRVPKMFYDYADTGSWTQSTYWDNEAAFQRQKLRQRVARNIDNRSVKTTMIGQDVAM

PVALAPVGLTGMQHADGEILAAQAAEEFGVPFTLSTMSVCSIEDVAEHTKNPFWFQLYVMDRGFSENLMK

RAHTAGCSALVLTLDLQVLGQRHRDIKNGLSTPPKPKPHVLVRREFGNIVG-HVTSLAEWTASQFDPTLD

WSSVEWVKKHWDRKLILKGINDVEDARIAADLGADAIVVSNHGGRQLDGALASYDILRDIVDAVGDEVHV

DGGIRSGQDVFKAVAMGAHSTYIGRAFIYGLGAMGKPGVRQVLEIIHKELDVTMGLCGETDIKKVGRHNL

-VL

>B2HAH2|B2HAH2_BURPS

PITCIEDLRVLAKKRIPRMFYDYVDTGSYTESTYRANEADFRKIRLRQRVGVDISNRNLRTTMAEQDVAM

PVALAPTGLVGMMRADGEILAARAARHFGVPFTLSTMSICSIEDIVAHVGGPFWFQLYMMDRAFIERLIE

RASAAGCPALVLTMDLQIAGQRHKDVKNGLSAPPRITLPNLLRRHFGNIVG-HVWSLDSWTREQFDPTIG

WRDAEWVRRRWNGKLIVKGVLDADDALRAADAGADAIVVSNHGGRQLDGAMSSVEALPAIVEAAGKEVWL

DGGVRTGQDVLKAVALGARGTMIGRAFLYGVAALGEQGARRALELIARELDTTMALCGCTDIRSVNADVL

ARR

>A8EMN0|A8EMN0_BURPS

PITCIEDLRVLAKKRIPRMFYDYVDTGSYTESTYRANEADFRKIRLRQRVGVDISNRNLRTTMAGQDVAM

PVALAPTGLVGMMRADGEILAARAARHFGVPFTLSTMSICSIEDIVAHVGGPFWFQLYMMDRAFIERLIE

RASAAGCPALVLTMDLQIAGQRHKDVKNGLSAPPRITLPNLLRRHFGNIVG-HVWSLDSWTREQFDPTIG

WRDAEWVRRRWNGKLIVKGVLDADDALRAADAGADAIVVSNHGGRQLDGAMSSIEALPAIVEAAGKEVWL

DGGVRTGQDVLKAVALGARGTMIGRAFLYGVAALGEQGARRALELIARELDTTMALCGCTDIRSVNADVL

ARR

>C0DX06|C0DX06_EIKCO

KITCIADLQRIARRRVPKMFYDYADTGSWTESTYRANEADFQSILFRQRVLVDMENRSLESKMIGQTVKM

PLALAPVGLTGMQHADGEILAARAAAKFGVPYILSTMSICSIEDVAANSPDPFWFQLYVMDREFMRDLIR

RAKAAQCSALVLTADLQVLGQRHKDIKNGLSTPPKPTLMNLLRRGFGNIEG-HVSSLSAWTAEQFDPGLS

WDDVARIKDEWGGKLIIKGIMDPEDAEAAVKSGADAIVVSNHGGRQLDGAPSSIRALPRIVSAVGNEVWM

DGGIRSGQDILRAWALGARGVLIGRTYIYGLGAYGEAGVTRALEILYNEMDITMAFTGHRNIQTVTRDIL

EGT

>C4GG06|C4GG06_9NEIS

QITTIEDLRQIARRKLPKMFYEYADTGSWTQTTYHANAADFAPIQFRQRVLVDMENRSLKTQMLGQEVKM

PLAIAPTGLTGMFHADGEILAARACEKFGIPYTLSTMSICSIEDVAENTTAPFWFQLYVMDREFMADLIR

RAKAAQCSALVLTADLQIVGQRHRDIKNGLTVPPRPTLANLIRRTFRNIAG-HASELMPWVAKQFDPKLS

WDDIAHIKDLWGGKLIIKGILDPEDAEKAVQHGADAIIVSNHGGRQLDGAPSSIRALPAIIQAVGSEVWL

DGGIRTGQDILKAWALGARGTFIGRPYLYGLAAYGEAGVTRALEILYNEMDLSMAFTGHRDIQNVTREIL

KGS

>A5WG31|A5WG31_PSYWF

KMTEIEDLRRVAERKVPRMFYDYVDSGSWTQTTYRNNETDFDRIKLRQRVLVDMDNRSLATQMIGEDVKM

PIAIAPTGFTGMMWANGEMHAAKAAKDFGVPFSLSTMSINSIEDVAEYTNHPFWFQLYVMDKDFMANLIR

RAKEANCSALILTADLQVLGQRHKDIKNGLSAPPKPTLANILRRTFGNIVG-HASSLSSWTAEQFDPSLS

WDDVARIKDMWGGKLIIKGIMEPEDAILAARSGADAMVISNHGGRQLDGAPSSIACLSEVVQAVKSEVWL

DSGIRSGQDVLKAIALGAKGTMVGRAFLYGLGAYGEDGVRRALEILYKECDITMAFCGRTNISDVTDDIL

KGT

>C8N9B7|C8N9B7_9GAMM

KMTNIEDLRRVARRKVPKMFFDYVRSGSWTESTLHHNTRDFDPIKFQQRVLVDMTNRTLETTMIGETVKM

PVAIAPTGFTGMMYADGEILAAKAAEKFGVPFSLSTMSICSIEDVAANTSKPFWFQLYVMDREFMEDLIK

RAKAANCSALILTADLQVLGQRHRDIKNGLSAPPKPTLLNMMRRTFGNIVG-HASSLSSWTSEQFDPRLS

WDDVARIKDLWGGKLIIKGIMTTDDAEKAAKSGADALIVSNHGGRQLDGALSTIKVLPDIVSAVGSEVWL

DSGIVSGQDILKCIALGAKGTMIGKSFLYGLGAYGEDGVRRCLEILYTEMDTTMAFCGHTDIKKVGKEIL

PGT

>D4DNN4|D4DNN4_NEIEG

KMTCIEDLRRVAKFKMPKMFYDYIDSGSWTQTTYRANTADFIPIQFRQKVLVDMEGRSLAAKMIGQDVKM

PLAIAPTGFTGMAWADGEIHAARAAEKFGVPFSLSTMSICSIEDVAENTSAPFWFQLYVMDREFMENLIK

RAQAAKCSALILTADLQVLGQRHKDIKNGLSAPPKPTLLNCIRRTFRNIVG-HASSLSSWTSEQFDPRLS

WDDVARIKDLWGGKLIIKGIMEPEDAELAVKHGADAIVVSNHGGRQLDGAPSSIHALPDIVQAVGSEVWL

DSGIRSGQDMLKAWAMGARGFMTGRAFLYGLGAYGEDGVRRALEIMYNEMDITMAFTGHRNLHDVDKNIL

EGT

>D0W7K6|D0W7K6_NEILA

KMTCIEDLRRVAKRKMPRMFYDYIDSGSWTETTYRENTSDFKDIRFRQKVLVNMEGRSLETKMIGQDVKM

PVAIAPTGFTGMAHADGEILAARAAEKFGIPFTLSTMSICSIEDVAENTSAPFWFQLYVMDREFMENLIK

RAKDAKCSALVLTADLQVLGQRHKDIKNGLSAPPKPTIANLIRRTFRNIVG-HASSLSSWTAEQFDPRLS

WDDVARIKDLWGGKLIIKGIMEPEDAEKAAKSGADALVVSNHGGRQLDDTVSAIKALPDIVSAVGSEVWM

DSGIRSGQDILKAWALGAKGTMIGRAFLYGLGAYGEEGVTRALEILYKEMDVSMAFTGHRDIQDVDASIL

RGK

>A4BES7|A4BES7_9GAMM

TICTIEDLQKLARRRVPKMFYDYADSGSWTESTYRANESDFQSIKLRQRVAVDMTNRSTAMPMVGQPTSM

PVALAPTGLTGMQCADGEIKAARAAEKAGVPFTLSTMSICSIEDVAEHTQAPFWFQLYVMDKEFAQNLID

RARNAGCSALVLTLDLQILGQRHKDIRNGLSTNPLKSLKGWSRHSFRNIVG-HADSLFSWTAEQFDPQLS

WDDVQWIKERWGGKLILKGILDVEDAKLAVASGADAIIVSNHGGRQLDGAPSSISQLKAIVDAVGDEVHM

DGGIRSGQDVLKAIALGAKGTYIGRPFLYGLGAQGETGVSKALEIIHKELDLTMAFCGERELTRINRNHL

PGT

>B6R6R6|B6R6R6_9RHOB

PILEISDLKERARRRVPKMFFDYADSGSWTESTYQANESDFAKIKLRQRIAVDMTNRTLATKMIGQDVSM

PVALAPTGLTGMQHADGEILAAQAAEEFGVPFTLSTMSICSIEAVAAKTTKPFWFQLYVMDRDFINSLID

RAKNAGCSALVLTFDLQILGQRHKDLRNGLSAPPKFTPKHVWNRTFGNIVG-HASSLSSWTAEQFDPRLS

WDDIEWIKKQWGGPLILKGILDKEDARHAVDSGCDAIIVSNHGGRQLDGAPSSIEILPEIVDEVGDEIHI

DGGIRSGQDVLKAICLGAKGTYIGRPFLYGLGAGGKQGVTQSLEILQKELDTTMALCGRRDLNTLNRDNL

SIN

>B9K1T0|B9K1T0_AGRVS

IALTIDDLKRQAKRRVPKMFFDYADSGSLSESTYHANESDFSRIKLRQRVLVDMTERSLASTMIGEPVTM

PVALAPTGMTGMQHADGEILAAEAAEAYGVPFTLSTMSICSIEDVALHTRRPFWFQLYVMDRDFVNSLID

RAKAAHCSALVLTLDLQILGQRHKDLRNGLSAPPKFTPKHLWRRHFGNIVG-HASSLSAWTAEQFDPKLS

WSDVAWIKERWGGKLILKGILDVEDARAAADSGADAIIVSNHGGRQLDGAPSSISMLASIVEAVGDEVHI

DGGIRSGQDVLKALALGAKGTYIGRPFLYGLGADGRAGVQRALEIIARELDISMALCGKRLISEVNASIL

MGN

>B9J7Y6|B9J7Y6_AGRRK

PL-TIADLKQLAKRRVPKMFFQYADSGSWTESTYEANEADFRKIKLRQRVLVDMTNRTLESTMIGQKVSM

PVALAPTGMTGMQHADGEMLAARAAEEFGIPFTLSTMSICSIEDVASATTKPFWFQLYVMDRDFVMSLID

RAKAAKCSALVLTADLQILGQRHNDVRNGLSAPPKFAPKHVWRHSFGNIIG-HAKSLSNWTTGQFDQRLS

WSDVAWIKEYWGGPLIIKGILDVEDAKAAVDTGADAIIISNHGGRQLDGAPSSISVLPGIVDAVGDEVHI

DGGIRSGQDVLRAVALGAKGTYIGRPFLYGLGAMGKDGVTLALEIIRKEMDLSMAFCGKRDIKTVDRGII

AGA

>C6ATQ6|C6ATQ6_RHILS

PL-TIADLKKLARRRVPKMFFDYADSGAWTESTYAANESDFSQIKLRQRVMVDMTNRTLATTMIGQKVSM

PVALAPTGLTGMQHADGEMLAARAAEEFGVPFTLSTMSICSIEDVASATTRPFWFQLYVMDKDFVVNLIN

RAKAAGCSALVLTADLQILGQRHKDLRNGLSAPPKFTPKHVWRRNFGNIVG-HAASLSAWTHEQFDPRLS

WADVAWIKEQWGGPLIIKGILDPEDAKAAADTGADAIVVSNHGGRQLDGAPSSISMLPKIVDAVGDEIHL

DGGIRSGQDVLKAVALGAKGTYIGRPFLYGLGAMGKEGVSLALGIIRKEMDITMALCGKRDINDVNSSII

DGR

>B3PVM2|B3PVM2_RHIE6

PL-TIADLKQLAQRRVPKMFFDYADSGAWTESTYRANESDFSRIKLRQRVLVDMSDRTLETTMVGQKVSM

PVGLAPTGLTGMQHADGEMLAARAAEEFGVPFTLSTMSICSIEDVASVTTRPFWFQLYVMDKDFVLGLIN

RAKAAKCSALVLTADLQILGQRHKDLRNGLSAPPRFTPKHLWRRTFGNIIG-HATSLAAWTHEQFDPRLS

WADVAWIKEQWGGPLIIKGVLDPEDARAAADTGADAIVVSNHGGRQLDGAPSSISMLPAIVDAVGDEIHL

DGGIRSGQDVLKAVALGAKGTYIGRPFLYGLGAMGKEGVTLALGIIRKEMDITMALCGKRDINDVNASII

SGQ

>A6UI13|A6UI13_SINMW

QILEIRDLKALARRRVPKLFFDYADSGAWTEGTYRANEEDFARIKLRQRVLVDMSDRSLETTMIGQKVSM

PVALAPTGLTGMQHADGEMLAAQAAEAFGIPFTLSTMSICSIEDVASATTKPFWFQLYVMEREFVLNLID

RAKAAKCSALVLTLDLQILGQRHKDLRNGLSAPPRLTPKHLWRRTFGNIVG-HASSLQVWTNEQFDPQLS

WKDVEWIKERWGGPLILKGILDPEDAKMAAKSGADAIIVSNHGGRQLDGAHSSISMLPRIVDAVGDEVHL

DGGIRSGQDVLKAVALGAKGTYIGRPFLYGLGALGKEGVRIALDIIRKEMDTTMALCGKRRITDVGLDVI

-AD

>C3MG80|C3MG80_RHISN

QILDISDLKALAKRRVPKLFFDYADSGAWTEGTYRANEEDFAKIKLRQRVLVDMTNRSLETSMIGQKVSM

PVALAPTGLTGMQHADGEMLAAQAAEAHGVPFTLSTMSICSIEDVASVTTKPFWFQLYVMEREFVLNLID

RAKAAKCSALVLTLDLQILGQRHKDLRNGLSAPPRMTPKHLWRRTFRNIVG-HASSLHAWTAEQFDPQLS

WKDVEWIKERWGGPLILKGILDPEDAKMAAKTGADAIIVSNHGGRQLDGAPSSISMLPKIIDAVGDEVHV

DGGIRSGQDVLKAVALGAKGTFIGRPFLYGLGAMGKDGVTLALDIIRKEMDITMALCGKRSITDVGRDVI

EYA

>A9CI08|A9CI08_AGRT5

KILTIADLKQQAQRRVPKMFFDYADSGAWTESTYRANEDDFAKIKLRQRVLVDMTDRSLATEMVGEKVSM

PVALSPTGLTGMQHADGEMLAAKAAEEFGVPFTLSTMSICSIEDVASVTSKPFWFQLYVMDRDFVNNLID

RAKAAGCSALVLTLDLQILGQRHKDLRNGLSAPPKFTPKHIWRRSFGNIVG-HASSLSTWTAEQFDPRLS

WQDVEWIKQRWGGKLILKGILDEEDARAAIDTGADAIIVSNHGGRQLDGAHSSIAMLPKIVDAVGDEVHM

DGGIRSGQDVLKAVALGARGTYIGRPFLYGLGAGGKQGVTTALEIIRKELDISMALCGKRLITDVDRSIL

---

>C0RM64|C0RM64_BRUMB

NIVEIADLKRLARRRVPKMFFDYADSGAWTESTYRANEDDFKKIKLRQRVLVDMTNRSLETTMIGEKVAM

PVALAPTGLTGMQHANGEMLAAQAAEAFGVPFTLSTMSICSIEDVASVTKKPFWFQLYVMDRDFVKNLIG

RAKAAGCSALVLTLDLQILGQRHKDIRNGLSAPPKFTPKHIWRRTFRNIAG-YASSLSSWTAEQFDPQLN

WSDVEWIKEQWGGKLILKGILDVEDAKMAAKSGADAIIVSNHGGRQLDGAPSSISMLQPIVEAVGDEVHV

DGGIRSGQDVLKARALGAQGVYIGRPFLYGLGAMGNEGVTLALEIIRKEMDITMALCGKRDINEIDKSII

SID

>B1M8X2|B1M8X2_METRJ

IITTVEDLRRVAKRRVPRMFYDYCDSGSWTESTYRANEADFAGIKLRQRVAVDMTDRSLATTMAGQAVAM

PVALAPTGLTGMQHADGEILAARAAEAAGVPFTLSTMSICSIEDVAETVSKPFWFQLYVMDRSFNDGLID

RAKAAGCSALVLTLDLQILGQRHKDIRNGLSAPPRLTPGTALRRTFRNIVG-HVRSISAWTADQFDPRLD

WDDVRRIRDRWQGPLILKGILDVEDAEKAAATGADALIVSNHGGRQLDGAPSSIAALPGIADAVGPEVLM

DGGIRSGQDVLKAVALGAKGVFIGRAFLYGLGAYGQAGVARSLEIIRTELDTTMALCGHRDIRAVDRSIL

PAA

>D3NZM3|D3NZM3_AZOS1

PVTCIEDLRRLAEWRVPRMFYDYADSGSYTESTYRANESDFGRIKLRQRVAVDMTNRTLASSMVGQPVAM

PVALAPTGLTGMQHADGEILAARAAAKAGVPFTLSTMSICSIEDVAENTDRPFWFQLYVMDRAFIDKLID

RAKAAGCSALVLTLDLQILGQRHKDIRNGLSTPPKLTIGNILRRTFRNIVG-HASSLSSWTAEQFDPTLN

WDDVRRIRDRWGGKLILKGILDPEDAVMAADTGADALIVSNHGGRQLDGAISSISALPAIVEAVGDEVLM

DGGIRSGQDVVKALALGAKGTFIGRAFLYGLGAGGEAGVSQCLEIIRKEMDVTMAMCGLRDIRTVTANIL

AGK

>B0UE51|B0UE51_METS4

PATCIEDLRVLAERRVPRMFYDYADSGSYTEGTYRANEADFAAIKLRQRVAVDMTNRTLASTMVGQPVSM

PVALAPTGLTGMQHADGEILAARAAAKAGVPFTLSTMSICSIEDVAENTDRPFWFQLYVMDRDFINRLID

RAKAAGCSALVLTLDLQILGQRHKDIKNGLSTPPRMTLPNILRRTFRNIVG-HASSLSSWTAEQFDPTLN

WDDVKRIQDRWGGPLILKGILDPEDAELAARSGAQALIVSNHGGRQLDGAPSSITALPAIAEAVGSEVLM

DGGIRSGQDVIKALALGAKGVFIGRAFLYGLGAGGEAGVTQCLDIIRKELDTTMAMCGLRDVKAVTSDIL

ATR

>D0J1E2|D0J1E2_COMT2

TITCVEDLRVLAHRRVPRMFYDYVDVGSWTESTYRANAADFQSILLRQRVALDLSRRSVRSTMAGQDVAM

PVAIAPTGLTGMQHADGEILAARAAKAFGVPFTLSTVSICSIEDVAEGTGGPFWFQLYVMDRKFVQRLIQ

RAEAAQCSALVVTLDLQISGQRHKDLKNGLSAPPKLSLLNLLRHSFGNIIG-HVTSMAEWSSQQYDPALS

WRDIAWIRQLWKGKLILKGIQDVEDARLAVASGADALIVSNHGGRQLDGAPSSIRALPAIAEAVGQEVHM

DGGVRSGQDVLKAIALGAKGVYIGRAMLYGLGAMGEQGVARTLEIIHKELDLTMAFCGRTDIRDVDASIL

LSS

>B3R288|B3R288_CUPTR

DINEIEDLRRLARQRVPRMFYEYADSGSWTESTYRANQREFGNILLRQRVAVNIGERRLATRMLGQDVAM

PVAIAPTGLAGMQHADGEILAARAARDFGVPFTLSTVSICSIEDVAEATGGPFWFQLYVMDRAFVERLMD

RARAAGCPALVLTLDLPVSAQRHKDLRNGLSAPPRLTPWNLLRRTFGNIIG-HVSSLADWSSRQYDPTLD

WDDVAWIRRRWPGKLVLKGIQDVEDARLACQSGADALIVSNHGGRQLDGAPASIRALPAIAQAVGEEVHM

DGGIRSGQDVLKAVALGARGVYIGRPMLYGLGAMGQAGVTRALEIIRKELDLTMAFCGHTDIRAVGTDIL

PPH

>C3JXI3|C3JXI3_PSEFS

LITTIEDLRKLAQKRVPRMFYDYADSGSWTESTYRANESDFARIKFRQRVARNIDERSIRATMIGQDVAM

PVALAPTGLAGMQHADGEILTARAAAAFGLRYTLSTMSICSLEDIAEQVGQPFWFQLYVMDRAFVEQLIE

RAKAAGVDALVLTLDLQILGQRHKDLINGLSAPPKLTLPNILRRGFGNIVG-HVSSLSSWTAQQFDPRLS

WDDVAWIKQCWGGKLIIKGILDVEDARLAANSGADALVVSNHGGRQLDGAPSSISQLPAIVEAVGEEVWL

DGGIRSGQDVLKAMALGAKGTMIGRAHLYGLGAMGEAGVTKALQIIARELDVSMALCGYNDIRDVNREIL

PGT

>A3KUE8|A3KUE8_PSEAI

VITDIEDLRRLARKRVPRMFYDYADSGSWSEGTYRANQDDFAAIKLRQRVARNIENRSLRTRMLGQEMAM

PVAIAPTGLAGMQHADGEILAARAAAEFGVRYTLSTMSICSLEDIATEVGQPFWFQLYVMDRDFIERLID

RAKAAGCDALVLTLDLQIIGQRHKDLKNGLSAPPRPTLANLLRRGFGNIVG-HVGSLSEWTARQFDPRLN

WGDVEWIKRRWGGKLVLKGILDAEDARLAADSGADALVVSNHGGRQLDGAPSTISALPAIVEAVGEEVWL

DSGIRSGQDVLKAIALGARGTMIGRPYLYGLGALGQAGVTRALEIIARELDLTMAFCGHTDIREVGRDIL

PGS

>A1WAZ5|A1WAZ5_ACISJ

KITCIEDLRVVAERRVPRMFYDYADSGAWTEGTYRANEDDFHPIKLRQRVAVNMEGRTTATTLVGQQAKM

PVCIAPVGLTGMQHADGEIHAARAAEKFGIPFTLSTMSICSIEDIAENTSAPFWFQLYMMDRDAMARMIQ

RAKDAKCSALVLTLDLQVIGQRHKDIKNGLTAPPKPTLANIIRRTFRNLVG-HVSSLAAWTNEQFDPRLS

WADVAWVKEQWGGKLILKGIMVEEDARLAVQHGADAIVVSNHGGRQLDGAPSAIHALPAIVDAVGTEVWM

DGGIRSGQDVLKAWALGARGTMIGRAMVYGLGAFGEAGVTKALQILHKELDVTMAFCGHTNIQNVDRSIL

PGT

>A1VS24|A1VS24_POLNA

VITTIEDLRVMAQKRVPRMFYDYADSGSWTESTYRANEADFQTIKLRQRVAVNMENRSTATKMVGVDVKM

PVAIAPVGLTGMQHADGEIKAARAAEKFGIPFILSTMSICSIEDIAASTQRPFWFQLYMMDREAMAAMIG

RARKAGCDALVLTLDLQVIGQRHKDLKNGLTAPPKPTLANIIRHTFGNLVG-HVNSLSAWTNEQFDPRLS

WADVAWVKEQWGGKLILKGIQDVEDARLAVQSGADALVVSNHGGRQLDGAQSSITALPAIVEAVGSEVWM

DGGIRSGQDVLKAWALGARGTLIGRAMVYGLGAMGEAGVTKALQIIHKELDITMAFCGRTQIGAVDKSIL

PGT

>C9Y9E7|C9Y9E7_9BURK

VITNIEDLRVLAEKRVPRMFYDYADSGSWTEGTYRANEEDFQKIKLRQRVAVNMENRTTATTMVGTVAKM

PVAIAPVGLTGMQHADGEIHAARAAEKFGIPFTLSTMSICSIEDIAENTSAPFWFQLYMMDRNAMANMIE

RARAARCSALVLTLDLQVIGQRHKDLKNGLSAPPRPTLANIIRHTFRNLVG-HVKSLAAWTNEQFDPRLS

WDDVKWVKEKWGGKLILKGIQDVEDAVLAAQSGADAIVVSNHGGRQLDGAPSSISALPAIVAAVGDEVWM

DGGIRSGQDVLKAWALGAKGTMIGRAMVYGLGAMGEAGVTKALQIIHKELDVTMAFCGHTNIQNVDRNIL

PGT

>B1Y7G5|B1Y7G5_LEPCP

VITTIEDLRVLAEKRVPRMFYDYADSGSWTESTYRANESEFQKIKLRQRVAVNMENRSTAVKMIGIDARM

PVAIAPVGLTGMQHADGEIHAARAAEKFGIPFTLSTMSICSIEDIAQNTTAPFWFQLYMMDRDAMARMIE

RCRAAKCSALVLTLDLQVIGQRHKDLKNGLTAPPRPTLKNIIRHTFRNLVG-HVRSLSAWTNEQFDPTLS

WADVAWVKAQWGGKLILKGIQDVEDARLAVASGADAIVVSNHGGRQLDGALSSIEALPAIVEAVGDEVWM

DGGIRSGQDVLKAWALGARGTMIGRAMVYGLGAMGEAGVTKALEILHKELDVTMAFCGHTKLTNVDRRIL

PGS

>A4G5T0|A4G5T0_HERAR

VITNIEDLRVLAQKRVPRMFYDYADSGSWTESTYRANSEDFARMKFRQRVAVNMENRTLKTTMVGQEVHM

PVAIAPTGLTGMQHADGEILAARAAEKFGIPFTLSTMSICSIEDIAAHTSKPFWFQLYVMDRPFIERLIE

RAKAAKCSALVLTLDLQILGQRHKDLKNGLSAPPKLTIPNILRRSFGNIVG-HASSLSAWTSQQFDLALS

WKDVEWIKKCWGGKLIIKGIMDAEDARLAVASGADAIIVSNHGGRQLDGALSSIAALPSIVEAVGDEVHM

DGGIRSGQDVIKALALGAKGTYIGRSFLYGLGAMGEEGVSKCLEIIERELDLTMAFCGLTDVKKVDRKIL

PGT

>A2SJP4|A2SJP4_METPP

VITCIEDLRVLARRRVPRMFYDYADSGSWTEGTYRANETDFARILLRQRVAVNMEGRSLRTTLAGQDCAM

PVVIAPTGLTGMQHADGEILGARAAEAFGVPFTLSTMSICSIEDIAAHTKAPFWFQLYWMDRDFMERLIE

RAKAARCSALVLTLDLQVLGQRHKDLKNGMTAPPKPTLANLIRHSFGNLVG-HASSLGTWTKEQFDPRLS

WDDVAWIKQRWGGRLILKGIMEVADAKLAADSGADAIVVSNHGGRQLDGAPSSIAALPAIAEAVGDEVWM

DGGIRSGQDVLKAVALGARGTMIGRAFLYGLGAMGQAGVTRALEIIRNELDITMAFTGHTDIRRVGREIL

PGS

>A4SYM1|A4SYM1_POLSQ

IITNIEDLRVLHKKRTPKMFYDYADSGSWTESTYRANESDFQKIKLRQRVAVNMTNRTTKTTMVGQEVAM

PVALAPTGLTGMQHADGEILAAKAAEKFGVPFCLSTMSICSIEDVAEQTTKPFWFQLYVMDRGFIERLIE

RAKAAKCSALVLTLDLQILGQRHKDLKNGLSAPPKLTIANMIRRTFRNIVG-HASSLSSWTAEQFDPGLN

WGDVEWIKKLWGGKLIIKGILDEDDARLAANSGADALIVSNHGGRQLDGAVSSIQALPGIVNAVGNEVWM

DGGIRSGQDVLKAWALGARGTMIGRPFLYGLGAMGEAGVTKCLELIHNELDITMAFTGHRDIQNVTKDIL

PGT

>A9HVG8|A9HVG8_BORPD

KITCIEDLRILAQKRVPRMFYDYADSGAWTEGTYRANETDFQAIKLRQRVAVDMEGRSLRTTMAGADAVM

PVAIAPTGLTGMQHADGEMVAAQAAAEFGVPFTLSTMSICSIEDVARATGKPFWFQLYVMDREFVANLID

RAKAAGCSALVLTLDLQILGQRHKDIRNGLSAPPKPTLANLIRRTFGNIVG-HASSLSSWTAEQFDPRLS

WADVEWIKQRWGGKLILKGILDVEDARLAADSGADALIVSNHGGRQLDGAMSSIAALPAIADAVGSEVWM

DGGIRSGQDVLKAVALGARGTMIGRAFLYGLGAYGKAGVTRALEILYKEMDVTMALCGRKSLTPGDRSVL

PGT

>A1K478|A1K478_AZOSB

PITCIDDLRRLALKRVPRMFYDYADSGSWTESTYRANEADFQSIKLRQRVAVNMDGRTLRTTMAGQEVAM

PVAIAPTGLTGMQHADGEILAARAAEKFGVPFTLSTMSICSIEDVAAHTTAPFWFQVYVMDRDFVERLID

RAKAARCSALMLTLDLQILGQRHKDLKNGLSAPPKPTLANLIRRSFGNIVG-HASSLASWTAEQFDPGLS

WADVEWIKKRWGGKLILKGIMDAEDARLAADSGADALVVSNHGGRQLDGAPSSIHALPGIVDAVGKEVWM

DGGIRSGQDVFKAVAMGARGTLIGRAFLYGLGAMGEAGVAKSLELIRKELDLTMAFCGHTDIRKVDRRVL

ERA

>A9ASD6|A9ASD6_BURM1

IITTVEDLRVLAERRVPRMFYDYADSGSWTESTYRANESDFQKIKLRQRVAVNMEGRTTRTAMIEQDVAM

PVAIAPTGFTGMQHADGEILAARAAEKFGIPFSLSTMSICSIEDVAAHTQAPFWFQLYVMDKDFVERLIE

RAKAAECSALILTLDLQVLGQRHKDLKNGLSAPPKPTIANLIRRTFGNIVG-HASSLSSWTAEQFDPALS

WADVEWIKKLWGGKLILKGIMDAEDARLAAASGADALIVSNHGGRQLDGAPSTIEALPPIVEAVGTEVWL

DSGIRSGQDVLKAIALGARGTMIGRAFLYGLGAMGEAGVTKTLEIIHKELDITMAFCGHRDIRTVDRSIL

PGT

>A1TNK5|A1TNK5_ACIAC

RITCIEDLRTVARRRVPRMFYDYADSGSYTESTYRANSEDFQKIKLRQRVAVNMENRTTRTRMVGQDVAM

PVAIAPTGLTGMQHADGEILGARAARAFGVPFTLSTMSICSIEDVAQHAGPGFWFQVYVMDRDFVERLID

RAKAAGVSALQVTLDLQILGQRHKDIKNGLSTPPRPTLANLLRRSFGNIVG-HASSLASWTAEQFDPRLN

WRDIEWIKKRWGGKLILKGIMDADDARLAVETGADAIVVSNHGGRQLDGAPSSIHALPAIVEAVGKEVWM

DGGIRGGQDVLKAWALGARGTLIGRSFLYGLGAFGEAGVTRALQIIQKELDITMAFCGHTDIHQVDRSIL

PST

>C5CRQ0|C5CRQ0_VARPS

KITCIEDLRVIAKRRVPKMFYDYADSGAWTESTYRANESDFQKIKLRQRVAVNMEGRSTRSTMIGQDVAM

PVAIAPTGLTGMQHADGEILGARAAKAFGIPFTLSTMSICSLEDIAEHTGRPFWFQLYVMDRDFIERLIE

RARAANVSALQLTLDLQILGQRHKDIKNGLSTPPKPTIANMIRRTFGNIAG-HASSLSSWTAEQFDPALS

WADVEWIKKRWGGKLILKGIMDVEDARLAAASGADALIVSNHGGRQLDGAPSSIAALPAIVDAVGREVWM

DGGIRSGQDVLKARALGARGTLIGRSFLYGLGAHGQAGVTRALQIIHKELDITMAFCGRTDIEKVDSSIL

PGS

>A1WMQ1|A1WMQ1_VEREI

KITCIEDLRLVAARRVPRMFYDYADSGSYTEGTYRANEADFQAIKLRQRVAVNMEGRSTRTTMVGQDVAM

PVAIAPTGLTGMQHADGEILGAKAAKAFGIPFTLSTMSICSIEDIAEHTGRPFWFQVYVMDRDFIERLID

RAKAANCSALQLTLDLQILGQRHKDIKNGLSAPPRPSLANLIRRSFGNIVG-HASSLSAWTAEQFDPRLH

WGDVEWIKKRWGGKLILKGIMDAEDARLAVNSGADALIVSNHGGRQLDGAPSSIAALPGIAAAAGKEVWM

DGGIRSGQDVLKARALGAQGTLIGRSFLYGLGAFGQAGVTRALEIIHKELDITMAFCGLTDINRVDASIL

PGT

>C5T904|C5T904_ACIDE

KITCIEDLRVVAQRRVPRMFYDYADSGSYTEGTYRSNTADFQGIKLRQRVAVNMEGRSTRTTMIGQDVAM

PVAIAPTGLTGMQHADGEILGARAAKAFGIPFTLSTMSICSIEDVAEHTGRPFWFQLYVMDRDFIERLID

RAKAAGCSALQLTLDLQILGQRHKDIKNGLSTPPKPTIANLIRRTFGNIVG-HASSLSSWTAEQFDPQLN

WSDVEWIKKRWGGKLILKGIMDAEDARLAANSGADALIVSNHGGRQLDGAPSSIAALPAIAQAVGKEVWM

DGGIRSGQDVLKARALGARGTLIGRSFLYGLGAYGEAGVTRALEIIQKELDITMAFCGHTDINAVDRGIL

LGT

>A9BNM8|A9BNM8_DELAS

KITCIEDLRVVAQRRVPRMFYDYADSGSYTQGTYRANEDDFQKIKLRQRVAVNMEGRSTRTTMIGQQVAM

PVAIAPTGLTGMQHADGEILGARAAQAFGIPFTLSTMSICSIEDIADHTARPFWFQLYVMDRRFMERLIE

RARAANCSALVLTLDLQILGQRHKDIKNGLSTPPKPTLRNLARRTFGNIVG-HVSSLSSWTASQFDPSLN

WGDVERIKKLWGGKLILKGIMDAEDARLAADSGADALIVSNHGGRQLDGAPSSIEALPGIAEAAGKEVWM

DGGIRSGQDVLKARALGAQGTMIGRSFLYGLGAYGQDGVTRALQIIQKELETTMAFCGHTQIDTVDRSIL

PGT

>B7WSV3|B7WSV3_COMTE

KITCIEDLRVVAKRRVPRMFYDYADSGSYTQGTYRANEDEFQTIKLRQRVAVNMEGRSTRTTMIGEEVAM

PVAIAPTGLTGMQHADGEILGAKAAKAFGVPFTLSTMSICSLEDIAEHTDHPFWFQLYVMDKAFMERLIN

RAKAANCSALVVTLDLQILGQRHKDIKNGLSTPPKPTLANLLRRSFGNIVG-HVSSLSSWTADQFDPSLN

WNDVEWIKKLWGGKIILKGVMDAEDARLAAQSGADALVVSNHGGRQLDGAPSSIAALPSIAEAAGKEVWM

DGGIRSGQDVLKARALGAQGTMIGRSFLYGLGAYGQAGVSKALQIIHKELDTTMAFCGHTHIDQVGKEIL

PGT

>A7HR52|A7HR52_PARL1

PV-SVSDYRELARRRLPTQLFDYIDGGSYAEATLDDNVAAFRRLKLRQRVLRDVSTIDTSAEIFGTQWKI

PAALAPVGFAGMYARRGEVQAAKAAEKFGVPFTLSTVGICAIEEVAKATSVPFWFQLYVIDRGYARALMQ

RAHEAGCPVLVFTVDLAVLGARYRDTRNGMNTALGKKLKVAMPLDFGNLRE-AVGEFGAWVAQNLDPAMT

WKDLEWVRANWPGKIIIKGVMDREDARLAMEVAPEGIVVSNHGGRQLDGTPATLDALPAIREEVGDVLLL

DGGIRSGLDIVKARARGADACLLGRAWAFALAAQGEAGVKAMLGTMRQEMHVAQALTGFTRARDIDSSAI

---

>B4R822|B4R822_PHEZH

RAASVSDFRELARRRLPKIFFEYIDGGSYAEVTLKRNVEDLEAIALRQRVMKDMTELSMTVETLGQTLAM

PVGLAPVGMAGMYGRRGETQAARAAAAAGVPFCLSTVGVCSVEEVAR-TGTPPWFQLYMLDRGYMRELLA

RAHELGSPVLVFTVDLPIPGARYRDVRSGFTGALEAVLNQAWPHTLGSVAG-AVTDFLVWIARNFDRSVT

WKDLDWVRENWDRPIVVKGVLDVEDARDAVRAGAQGVVVSNHGGRQLDGVKSSIASLPRIADAVGGEVFM

DGGVRSGLDVLKALALGAKACFVGRPWAYALGAGGEAAIGKMLGLMRSELAVAMILTGCNDVRRAGRELL

-DI

>B3PBR7|B3PBR7_CELJU

ILASIEDYRTLARKRLPHFLFEYIDGGAFSETTLRNNQRDLQHIALRQRVLRDVSNITTTTRLFGQEFKL

PLGLSPVGIAGLNARRGEVQAAQAAEAAGVPFCLSTVSACSIDEVRAGVNHPVWFQLYMIDRGFLHEMLN

RAKAAGTSTLLFTVDMPVPATRYRDMRSGLSSGWQRKLTRVGPHSLGNIAP-ILDEFWAWLGNNFDPRVT

WADIDRIRSEWDGHFVIKGILDAEDARQAKSIGCDGLIVSNHGGRQLDGALSSIKALPAIADAVGNSLIL

DSGIRSGLDIVRALALGARMVMIGRPWVYALAARQKKGVEEILDIFARELRVAMALSGCTRLEDITPAIL

-AS

>A6TAF0|A6TAF0_KLEP7

IVSAPSDYREAARRRLPRFLFDYIDGGAVAENTMNANAAELASVALRQRVLCGAGEPTLATTILDAPWAM

PVALGPVGATGMYARRGEVQAARAASRAGIPYTLSTVSVCSIEEVASHASGALWSQLYVLDRGYMRNALE

RAWAAGMKTLVFTVDMPIPGSRYRDNRSGMSGP-HATLRQYLPLSFGNIEA-YTDDYMGFISNNFDPSIA

WHDLEWIRDSWQGKLIIKGILDADDARNAVRLGADGIVVSNHGGRQLDGAIPTARALPRVVDAVGDTVLA

DSGVRSGVDVIRLLALGAKGVLLGRAYIYALAAAGEAGVAHLLRLFAEDMKVTMTLTGATSPSAISLDCL

RLE

>C6XQD4|C6XQD4_HIRBI

KIASTHDFRRKAKAKLPRFLFEYVDGGSYDEVTLHQNVEALQKIALRQRVLCDVSDIDLSTTLFGQKMAL

PAILAPVGLAGMYARRGEVQAAQAAEEFGIPFTLSTVSACPLKEVASKTKRPFWFQLYMIDRAFMKDLLQ

QAMEAECSALVFTTDMPVPGARYRDYHSGLAGSVLGDMRRIFPHQLGNVAP-VLEDFFAWMRNNFDPSVT

WEDIDFIRSIWKGPLIIKGILDKDDAIRAADFGADGLIVSNHGGRQLDGVPASCHALPAIAEAVGSTILA

DGGVRNGLDIVRLMALGANGVLLGRSWIYALAAEGKHGVSKMLDLFAAEMKVAMTLTGVTRPEQINQSIL

NNN

>C6XPT8|C6XPT8_HIRBI

RIASTADFREAARRRVPRFLFDYADGGSGTEDTMLSNSADLRKVALRQRVLKDVASIDLSTEILGQKQDL

PVALGPVGISGMFARRGEVQAASSASKAGVPACLSTVSICSIEEVVAATER-FWFQLYVIDRSVMLDIIE

RAKVAGAKALVFTVDMPVPGSRARDVHSGMSGP-NAGIRRIMPHTLGNLVA-ALNDYMGWLGKNFDPSIQ

WKDLEWIRSAWDGPLIIKGILDPEDAREAVALGADGIVVSNHGGRQLNGALSTAHALPAIAEAVGDTVLA

DGGVRSGLDVVRMLALGADGVLLGRLWLYALAAGGSAGVTQMFDFLKQDMKVTMTLAGVNSISKIDRSIL

-VD

>A5VFB0|A5VFB0_SPHWW

IAASAIDYREAARRRLPHFLFEYIDGGAYAEVTLRRNIADLEAIALRQRVLRDVSAIDLSTELFGQKLAM

PVALAPVGLAGLTARRGEVQAVRAAEAAGIPFTLSTVSACPLAEVARGASKPFWFQLYMIDRAFMRDLLA

QAVEANCSALVFTIDMPVPGTRYRDRRSGLSGALGGQLRRIGPHHLGNVAP-VLEDFFAWVGGNFDPGIH

WRDLDFIRSEWKGPLILKGILDPEDAREAVASGADGIVVSNHGGRQLDGVLSTARALPPIADAVGGPILV

DGGVRSGLDVVRLLALGADTVMLGRAWAYALAGGGQRGVAHLLELIEAEMRVAMALTGATSIAAIDRDSL

ELP

>B0T7X2|LLDD_CAUSK

IISSTTDFREAARRQLPRFLFDYIDGGAYAERTLARNVSDLADISLRQRVLKDVSRVSTRTTLFGVEQTL

PVALAPVGLTGMYARRGEVQAARAAAAKGVPFCLSTVSVCDLAEVSRASSAPIWFQLYMLDRGFMRDLLA

RAADAGATALVFTVDMPVPGARYRDAHSGMTGP-NAAMRRLVPHTLGNVAP-VLEDFMGWLGANFDPSIQ

WKDLDWIRDQWKGPLILKGVLDPEDAKAAADIGADGIVVSNHGGRQLDGVLSSARALPDIAEAVGDTVLA

DGGVRSGLDVVRMLALGAKGVLLGRAFVYALAARGGPGVSQLLDLIEKEMRVAMALTGVNTLDQIDRSIL

KTD

>B8H3Q5|LLDD_CAUCN

IVSSTTDFREAARRRLPRFLFDYIDGGAYAERTMARNIDDLADIALRQRVLMDVSVVDPSTTLFGVRQAL

PVALAPVGLTGMYARRGECQAARAAAAKGVPFCLSTVSVCDVDEVRAASATPFWFQLYVLDRGFMRDLLA

RASAAGATTLVFTVDMPVPGARYRDAHSGMSGP-NAAARRLVPHRLGNVAP-ALQDFMGWLAANFDPSIQ

WSDLKWIRDAWKGPLVIKGVLDPEDAKAAADIGADGVVVSNHGGRQLDGVLSSARALPAIADAVGDTVLA

DGGVRSGLDVVRMLALGARGVLIGRAYAYALAARGEAGVTQLLDLIDKEMRVAMALTGVRDVASINETIL

PRA

>A9IN89|LLDD_BART1

IIASTFDYRKAAKRRLPPFLFHYIDGGAYAEETLRRNCSDLQALALRQRILRQVGGVDLSIKLFEQRLDL

PIVLAPVGLTGMYARRGEVQAAHAATAKGIPFTLSSVSVCPIAEVQEAVGGGFWFQLYVLDRGFMRDALE

RAWASGVRTLVFTVDMPIPGARYRDAHSGMSGP-YAGLRRFLPHDLGNVST-YLDDYVGWLGANFDPSIG

WHDLQWIRDFWKGKMILKGILDPEDAREAVQFGADGIVVSNHGGRQLDGVLSTARALPAIAEAVKNVILA

DSGVRSGLDVVRMIAQGADAVMIGRAFVYALAAAGEKGVAHLLDLFANEMRVAMTLTGAQTLKEITCESL

NTD

>C6ABS8|C6ABS8_BARGA

IISSTLDYRKAAKRRLPPFLFHYIDGGAYAEETMRRNCTDLQALALRQRILRQVGEVDLSIKLFEQTLDL

PIVLAPVGLTGMYTRRGEVEAARAAAAKRIPFTLSSVSVCPIAEVQEAVGGAFWFQLYVLDRGFMRDALE

RAWLAGVRTLVFTVDMPVPGARYRDAHSGMSGP-YAGLRRFLPHDLGNVST-YLDDYVGWLGENFDPSIG

WHDLQWIRDFWKGKMILKGILDPEDAREAVKFGADGIVVSNHGGRQLDGVLSTTRALPAIADAVKDTILA

DSGVRSGLDVVRMIAQGADAVMIGRAFVYALAAAGKKGVMHLLDLFANEMRVAMTLTGTQTIKDITRKSL

NID

>C5ALC1|C5ALC1_BURGB

IISSTLDYREAARRRLPRFLFDYIDGGAYAEDTLRRNSEDLRALALRQKVLKEVGDVDLSTRIFGQQLAL

PVALGPVGLTGMYARRGEVQAARAASAKGVPFTLSTVGVCSIEEVQSQVARPIWFQLYVLDRGFMKNVLE

RAWAVGIRTLVFTVDMPVPGARYRDKHSGMSGP-HAAIRRYWPHDLGNVSA-YLDDYVGWLGANFDPTIG

WRDLQWIRDFWKGSMILKGILDPLDARDAVKFGADGIVVSNHGGRQLDGVLSTARALPTIADAVKKTVLV

DSGVRSGLDVVRMLALGADTVLLGRAYIYALASAGERGVAHLLDLIRNEMRVAMTLTGARSIADISRSNL

SPG

>C3K053|LLDD_PSEFS

IISSASDYRAAAQRKLPRFLFDYIDGGAYAEHTMRANSSDLAEISLRQRILRNVDNLSLKTTVFGQELDM

PVILSPVGLTGMYARRGEVQAAKAAANKGVPFCLSTVSVCPIEEVASQSARAIWFQLYVLDRGFMRNALE

RAQAAGVTTLVFTVDMPTPGARYRDAHSGMSGP-FAAQRRMLPHDLGNISK-YLEDYIGWLANNFDPSIS

WKDLEWIREFWKGPMIIKGILDPQDAKDAVSFGADGIVVSNHGGRQLDGVLSTAKALPPIADAVGDTVLV

DSGIRSGLDVVRMLALGAKACLLGRASAYALAADGQNGVENLLDIFAKEMRVAMTLTGVTSIEQIDHTTL

GQR

>C6AK34|C6AK34_AGGAN

IISSANDYRAAARRRVPPFMFHYADGGSYTEYTLARNVSDLSEIALRQRVLNDMSQLNTEIELFGEKLSM

PVILAPVGACGMYASRGEVQAAKAAAAKGLPFTLSTVSICPIEEVAPAINRPMWFQLYVLDRGFMKNALE

RAKAAGCSTLVFTVDMPTPGARYRDMHSGMSGD-YKWLRRVIPFTLGNVSK-YMDDYIGWLTENFDPSIS

WKDLEWIRDFWDGPMVIKGILDPEDAKDAVHFGADGIVVSNHGGRQLDGVLSSARALPPIADAVKGKILV

DGGIRNGLDVVRMMALGADATMIGRPFVYALGADGQRGVENLLDIFKKEMRVALTLTSTKDISNITADAL

SSN

>A3N3E5|LLDD_ACTP2

IISSANDYREAARRRVPPFMFHYADGGSFSERTLERNVTDLADLALRQRVLKDMSQLDTEIELFGEKLAM

PAVLAPVGACGMYARRGEVQAAQAAENKGIPFTLSTVSICPIEEVTAAIKRPMWFQLYVLDRGFMKHVLE

RAKAAGCSTLVFTVDMPTPGARYRDRHSGMSGD-YKEIRRALPHTLGNVSA-YTDDYVVWLGENFDPSIS

WKDLEWIRDFWDGPMVIKGILDPEDAKDAVRFGADGIVVSNHGGRQLDGALSSARALPSIADAVKGKILA

DSGIRNGLDIVRMLALGADATMLGRAFVYALGAAGKAGVENMLDIFKKEMHVAMTLTSNQKISDITRDAL

DLS

>A0FKM3|A0FKM3_PASHA

IISSANDYRRAAKRRVPPFMFHYADGGSYAEQTLRRNVSDLEDIALRQRVLKDMSQLDTGIELFGEKLSM

PVTLAPVGALGMYARRGEVQAAKAADNKGIPFTLSTVSICPIEEVAPAIKRPMWFQLYVLDRGFMKNALE

RAKAAGCSTLVFTVDMPTPGARYRDMHSGMSGP-YKDIRRVLPHTLGNVSN-YMNNYIGWLTDNFDPSIS

WKDLEWIREFWDGPMVIKGILDPEDAKDAVRFGADGIIVSNHGGRQLDGVLSSAKALPSIADAVKGKILA

DSGIRNGLDVVRMLALGADCTMIGRSFVYALSAAGQAGVENLLDIFLKEMKVAMTLTSNAKISDIGRDAL

DLS

>C9R5R3|C9R5R3_AGGAD

IISSANDYREAARRKVPPFMFHYADGGSYAEQTLKRNVNDLENIALRQRVLKDMSQLDTQIELFGEKLSI

PAILAPVGALGMYARRGEVQAAKAAASRNIPFTLSTVSICSIEEVAPKIDRPMWFQLYVLDRGFMRNALE

RAKAAGCSTLVFTVDMPTPGARYRDMHSGMSGP-YKEIRRIIPHTLGNVSH-YMDDYIGWLTENFDPSIS

WKDLEWIREFWDGPMIIKGILDPKDAKDAVLFGADGIVVSNHGGRQLDGVLSSARALPPIAEAVKGKILA

DSGIRNGLDIVRMIALGADACMIGRSFVYALGAAGQLGVENMLDIFKKEMHVAMTLTSNQKISDITKDAL

LSK

>A4NKD6|A4NKD6_HAEIF

IISSASDYREAARRRVPPFMFHYADGGSYAEQTLARNVSDLENIALRQRVLKDMSELDTSIELFGEKLSM

PTILAPVGACGMYARRGEVQAAQAADNKGVPFTLSTVSICPIEEVAPAIKRPMWFQLYVLDRGFMKNALE

RAKAAGCSTLVFTVDMPTPGARYRDMHSGMSGP-YKEIRRVLPHTLGNVST-YMDDYIGWLTENFDPSIS

WKDLEWIREFWEGPMVIKGILDPEDAKDAVRFGADGIVVSNHGGRQLDGVLSSARALPPIADAVKGKIIA

DSGIRNGLDIVRMLALGADATMLGRAFVYALGAAGRQGVENMLDIFKKEMCVAMTLTSNRTISDIKPEAL

LSK

>A7IMB0|LLDD_XANP2

IISSSSDYREAARRRLPPFLFHYIDGGAYAEATLRRNVEDLSDLALRQRVLKSVGEVDLSTTLLKQQLSM

PVGLAPVGLTGMYARRGEVQAAQAATQKGIPFTLSTVSVCSIEEVQSQVGKPIWFQLYVLDRGFMKNALE

RAWAAGIRTLVFTVDMPVPGARYRDAHSGMSGP-NAAFRRMVPHDLGNVSA-YREDYVGWLGNNFDPSIG

WKDLEWIREFWKGPMVIKGILDPEDARDAVRFGADGIIVSNHGGRQLDGVLSSARAMPAIADAVKGTLLA

DSGIRSGLDVVRMLAQGADGVLLGRAFVYALAAAGRAGVENLLDIIAKEMRVAMTLTGARAISDISRDSL

REI

>B9K115|LLDD_AGRVS

IISSSTDYREAARRRLPPFLFHYIDGGAYSEHTMRRNIDDLADLALRQRVLKSVGTVDISTTLFDEELAM

PVVLAPVGLTGMYARRGEVQAARAAEKKGIPLTLSTVSVCPIEEVQAASNRPIWFQLYVLDRGFMKNALE

RAWAAGIRKLVFTVDMPVPGARYRDAHSGMSGP-NASLRRIIPHDLGNVSA-YRADYVGWLGENFDPSIG

WKDLEWIRDFWKGPMIIKGILDPEDAKDAVRFGADGIIVSNHGGRQLDGVLSSARALPAIAAAVKGTILA

DSGIRSGLDVVRMIAQGADGVLIGRAFVYALAAAGQAGVENLLDLFAKEMRVAMTLTGARSIAEISPDSL

RGL

>A8HTC9|LLDD_AZOC5

IISSPNDYRAAAKSRLPPFLFHYVDGGAYAEYTLRRNVEDLSHIALRQQVLRNVADLSLETELFGQKLTM

PVALAPVGLTGMLARRGEVQAAKAAQAKGVPFTLSTVSVCPIEEVQSQCAKPIWFQLYVLDRGFMRNALE

RAQAAGINTLIFTVDMPVPGARYRDAHSGMSGR-SGPTRRVLPHDLGNIST-YRADYIGWLAANFDPSIS

WKDLEWIRSFWKGPMIIKGILDPVDARDAVAFGADGIVVSNHGGRQLDGVLSSARALPAIADAVGDTVLA

DSGIRTGLDVVRMLALGAKGVLLGRAFAYALATHGQAGVANLLDLIEKEMRVAMALTGARSIAEITRDSL

GLP

>B7WSV7|B7WSV7_COMTE

IISSTTDYRAAAQKRLPPFLFHYLDGGAYAEKTLARNVDDLADVALRQRVLKNMSQLDTSIELFGEKFSI

PVALAPVGLTGMFARRGEVQAAMAADKKGIPFTMSSVSVCPIEEVAPRLGRPMWFQLYVLDRGFMKNALE

RAQAAGVSTLVFTVDMPVPGARYRDAHSGMSGP-NAAMRRYLPHTLGNIST-YKEDYMGYLGANFDPSIS

WSDLEWIRDFWKGPMLIKGILDPEDARDAVRFGADGIIVSNHGGRQLDGVLSSARALPAIADAVKGKILA

DSGVRNGLDIVRLLALGADCTMIGRAFVYALAAEGEAGVTNLLNLLEKEMRVAMTLTSVKNVSEITGDLL

VRD

>A1TSY6|A1TSY6_ACIAC

IISSTADYREAARKRLPPFLFHYIDGGAYAEQTLRRNVEDLAAVALRQRVLKDMSRLDTRVELFGEQLSI

PVALAPVGLTGMFARRGEVQAARAADRHGVPFTLSSVSVCPIEEVAPELGRPMWFQLYVLDRGFMKNALE

RAQAAGCTALVFTVDMPVPGARYRDAHSGMSGP-NAALRRYWPHDLGNISA-YLADYMGYLGANFDPSIS

WKDLEWIRAFWKGPMLIKGILDPEDAKDAVRFGADGIIVSNHGGRQLDGVLSSAHALPPIADAVKGKILA

DSGIRNGLDVVRTIALGADAAMIGRAFIYALAAAGEAGVKHVLELLEKEMRVAMTLTSVAKVSDITGDLL

VKA

>A9BZS6|A9BZS6_DELAS

IISSTSDYRAAAQKRLPPFLFHYIDGGAYAEQTLRRNVEDLAAVALRQRVLKDMSRLDTSIELFGEKLSI

PVALSPVGLTGMYRRRGEVQAARAADAHGIPFTMSTVSVCPIEEVAPKIKRPMWFQLYVLDRGFMQNALE

RAQAAGCSTLVFTVDMPVPGARYRDAHSGMSGP-NAAMRRYWPHDLGNISA-YRADYIGWLGANFDPSIS

WKDLEWIRAFWKGPMVIKGILDPEDAKDAVRFGADGIIVSNHGGRQLDGVLSSARALPAIADAVKGKILA

DSGIRNGLDVVRAIALGADCAMIGRAFIYALATSGEAGVKHLLELLEKEMRVAMTLTSVSKVSDITGDLL

VRQ

>C5T829|C5T829_ACIDE

IISSSADYRAAAQKFLPPFLFHYIDGGAYAEQTLRRNVDDLAAVALRQRVLKDMSRLDTSIELFGEKLSI

PVALSPVGLTGMYRRRGEVQAARAADQHGIPFTMSSVSVCPIEEVAPKLQRPMWFQLYVLDRGFMQNALE

RAQAAGCTTLVFTVDMPVPGARYRDAHSGMSGP-NAALRRYWPHDLGNISA-YREDYMGYLSANFDPSIS

WKDLEWIRAFWKGPMVIKGILDPEDARDAVRFGADGIIVSNHGGRQLDGVLSSARALPAIADAVKGKILA

DSGIRNGLDVVRAIALGADCAMIGRAYIYALAAAGEAGVKHLLELLEKEMRVAMTLTSVAKVGDITGDLL

VRE

>B4WC00|B4WC00_9CAUL

IISSPGDYREAARRKLPPFLFHYIDGGAYAEQTLRRNVEDWQAIALRQRVLQDMTSLSLETRLFDETLRL

PIILAPVGLTGMYARRGEVQAAKAAASRGVPFTLSTVSVCSIEEVAPAIDRPMWFQLYVLDRGFMKNALE

RARAAGVKTLVFTVDMPTPGARYRDAHSGMSGP-HAEIRRMIPHDLGNVSA-YLADYIGWLGQNFDPSIS

WKDLQWIRDFWDGPMIIKGVLDAQDARDAVSFGADGIVVSNHGGRQLDGVLSSARALPAIAEAVKGRILI

DSGVRNGLDVVRAIALGADAVMLGRAFVYALAAGGEAGVANLLDLFEKEMRVAMTLTGAKSVAGISQDML

-AL

>A3Y7B1|A3Y7B1_9GAMM

IIASSTDFRKAAKAKLPPFLFHYIDGGSYGEHTLRKNTQDLAEIALRQRVLNDMSQMDLSTELFGEKLSL

PISLAPVGLTGMYARRGEVQAAKAADKKGIPFTMSTVSVCPIEEVAPSIERPMWFQLYVLDRGFMKNALE

RAKAAGVTTLVFTVDMPVPGARYRDMHSGMSGE-NAPIRRVLPHDLGNIST-YRADYIGWLGDNFDPSIS

WKDLEWIRDYWDGPMVIKGILDADDAKDAVKFGADGIIVSNHGGRQLDGVLSSAKALPYIADAVKGKILV

DSGIRNGLDVVRMLALGADSTLLGRSFIYALAAKGQAGVESLLDLYEKEMRVAMTLCGANKLSDLTRDSL

SFK

>A5F0Z6|A5F0Z6_VIBC3

IISASTDYRAAAKAKLPPFLFHYIDGGSYGEHTLRRNTDDLADIALRQRVLSDMSELSLETELFGEKMAL

PIALSPVGLTGMYARRGEVQAAQAAEAKGIPFTLSTVSVCPIEEVAPSIHRPIWFQLYVLDRGFMKNVLE

RAKAAGVKNLVFTVDMPVPGARYRDMHSGMSGP-NAAMRRVLPHDLGNISK-YREDYIGWLGANFDPSIS

WKDLEWIRDFWDGPMIIKGILDTEDAKDAVRFGADGIVVSNHGGRQLDGVLSTVQALPAIADAVKGKILV

DSGIRTGLDVVRMLALGADCTMLGRSFIYALAAQGRTGVENLLELYEKEMRVAMTLTGAKSIAELSRDSL

VKR

>C9NV94|C9NV94_9VIBR

IISASTDYRKAAKSKLPPFLFHYIDGGSYGEHTLRRNTEDLADIALKQRVLKNMSDLNLETEIFGEKFAL

PIALSPVGLTGMYARRGEVQAAIAAENKGIPFTMSTVSVCPIEEVTPELARPMWFQLYVLDRGFMKNVLE

RAKAAGVTTLVFTVDMPVPGARYRDMHSGMSGP-NAAMRRVFPHDLGNIST-YREDYIGWLGENFDPSIS

WEDLEWIRDFWDGPMVIKGILDEQDAKDAVKFGADGIVVSNHGGRQLDGVMSSAKALPSIADAVKGKIFV

DSGIRTGLDVVRMLALGADCAMLGRSYIYALAAQGQAGVENLLDLYEKEMRVAMTLTGAKNIQELTRESL

NFK

>A5KVR7|A5KVR7_9GAMM

IISASTDYRAAAKSKLPPFLFHYIDGGSYGEHTLHRNTADLAEIALKQRVLNDMSDLNLETELFGEKLAM

PIALAPVGLTGMYARRGEVQAAKAADNKGIPFTMSTVSVCPIEEVAPKIERPMWFQLYVLDRGFMKNVLE

RAKAAGVTTLVFTVDMPVPGARYRDMHSGMSGP-NAAVRRVFPHDLGNIST-YREDYIGWLGDNFDPSIS

WKDLEWIRDFWDGPMVIKGILDEEDAKDAVRFGADGIVVSNHGGRQLDGVLSSAKALPSIADAVKGKILV

DSGIRTGLDVVRMMALGADCTLLGRSFVYALAAQGQAGVENLLDLYDKEMRVAMTLTGAKTIKDLTRESL

VGL

>C9QHF5|C9QHF5_VIBOR

IISASTDYRAAAKAKLPPFLFHYIDGGSYGEHTLRKNTEDLADIALKQRVLNNMEDLSLETEVFGEKLSM

PIALAPVGLTGMYARRGEVQAAKAAENKGIPFTMSTVSVCPIEEVAPAIERPMWFQLYVLDRGFMKNVLE

RAKAAGVTTLVFTVDMPVPGARYRDMHSGMSGP-NAAARRVFPHDLGNIST-YREDYIGWLGDNFDPSIC

WKDLEWIRDFWDGPMVIKGILDEQDAKDAVSFGADGIVVSNHGGRQLDGVLSTAKALPSIADAVKGKIFV

DSGIRTGLDVVRMLALGADCTLLGRSFIYALAAQGQTGVENLLDLYEKEMRVAMTLTGAKSIKDLNSDSL

KIK

>A8SZG8|A8SZG8_9VIBR

IISASTDYRAAAKAKLPPFLFHYIDGGSYDERTLKRNTEDLGDVALRQRVLRDMSELSLETEIFGEKLAM

PIALAPVGLTGMYARRGEVQAAKAAEKKGIPFTMSTVSVCPIEEVAPAIERPMWFQLYVLDRGFMRNVLE

RAKAAGVTTLVFTVDMPVPGARYRDMHSGMSGP-NAAMRRVFPHDLGNIST-YREDYIGWLGANFDPSIS

WKDLEWIRDFWDGPMVIKGILDEEDAKDAVRFGADGIVVSNHGGRQLDGALSTAKALPSIADAVKGKIFA

DSGIRTGLDVVRMLALGADCTLLGRSFVYALAAKGGEGVENLLDLYDKEMRVAMTLTGAKTIADLSQGSL

VKM

>C9P866|C9P866_VIBME

IISASTDFRAAAQAKLPPFLFHYIDGGSYREDTLRRNTTDLADIALRQRVLNDMSELSLETELFGESLAM

PIALAPVGLTGMYARRGEVQAAHAAANKGIPFTLSTVSVCPIEEVTATLTRPMWFQLYVLDRGFMKNVLE

RAKAAGVTTLVFTVDMPVPGARYRDMHSGMSGP-NAASRRILPHDLGNIST-YREDYIGWLGNNFDPSIS

WQDLEWIRDFWDGPMVIKGILDVEDAKDAVRFGADGIVVSNHGGRQLDGVLSTAQALPSIADAVKGKILV

DSGIRSGLDVVRMLALGADCTLLGRAFIYALAAQGQAGVEHLLDLFDKEMRVAMTLTGAKRVQDLSRDSL

VNR

>C9PF18|C9PF18_VIBFU

IISASTDYRAAAKAKLPPFLFHYIDGGSYSEHTLRRNTDDLSDIALRQRVLNDMSQLDLSTELFGESLAM

PIALAPVGLTGMYARRGEVQAATAASNKGIPFTLSTVSVCPIEEVAPAIDRPMWFQLYVLDRGFMKNVLE

RAKAAGVTTLVFTVDMPVPGARYRDMHSGMSGP-NAAARRVMPHDLGNIST-YREDYIGWLGTNFDPSIS

WKDLEWIRDFWDGPMVIKGILDTEDAKDAVRFGADGIVVSNHGGRQLDGVLSTARALPDIADAVKGKILV

DSGIRTGLDVVRMLALGADCTLLGRAFVYALAAQGQAGVENLLDLFEKEMRVAMTLTGAKTIQDLSRESL

-VN

>A3J7V8|A3J7V8_9ALTE

IISASTDYRVAAKRRLPPFLFHYIDGGSYNEHTLKRNVEDLSDIALRQRVLNDMTQLDLTTELFDETLSM

PVALSPVGLTGMFARRGEVQAARAAANLGVPFTMSTVSVCPIEEVAPAISRPMWFQLYVLDRGFMRNALE

RAKAAGVTTLVFTVDMPVPGARYRDAHSGMSGP-YAAQRRILPHDLGNISA-YRGDYIGWLGDNFDPSIC

WKDLEWIREFWDGPMVIKGILDPDDARDARSFGADGIIVSNHGGRQLDGVPSTCRALPAIADAVKGKILV

DSGIRTGLDVLRMLALGADCTMIGRAYIYALAADGEAGVTNLLKLIESEMRVAMVLTGARTIADISPELL

REL

>C8PYV6|C8PYV6_9GAMM

IISSANDYREAARRRLPPFLFHYIDGGAYAEYTLKRNVEDLSSIALRQRVLKDMTQLDLSTEIFGEQLSL

PVALSPVGLTGMYARRGEVQAAMAADKKGIPFTMSTVSVCPIEEVTPKINRPMWFQLYVLDRKFMQNVLE

RAKAAGCSTLVFTVDMPVPGARYRDAHSGMSGP-NAAMRRYLPHDLGNVSK-YLEDYIGWLGNNFDPSIS

WKDLEWIRDFWDGAMVIKGILDPQDAKDAVRFGADGIVVSNHGGRQLDGVMSTATALPKIVDAVKGKILV

DSGIRNGLDVVRMLALGADLCMLGRAFVYALAADGEAGVTNLLNLIDKEMRVAMTLTSANRIQDINRDCL

DFS

>C6RQU9|C6RQU9_ACIRA

IISSSNDYREAARRRLPPFLFHYIDGGAYAEYTLQRNVEDLSKIALRQRVLNDMSELSLETKLFNETLSM

PVALAPVGLTGMYARRGEVQAAVAAEKNGIPFTLSTVSVCPIEEVAPAIQRPMWFQLYVLDRGFMKNVLE

RAKAAGCSTLVFTVDMPVPGARYRDVHSGMSGP-NAAMRRYMPHDLGNISK-YLEDYIGWLGANFDPSIS

WKDLEWIREYWDGPMVIKGILDPEDAKDAVRFGADGIVVSNHGGRQLDGVLSTTRALPAIADAVKGKIMV

DSGVRNGLDVVRMIALGADLCLLGRAFVYALGAAGGEGVNHLLELINKEMRVAMTLTGAKTIQDISSECL

KRI

>A3M0X0|LLDD_ACIBT

IISSGNDYRAAAQRRLPPFLFHYIDGGAYAEYTLKRNVQDLSEIALRQRVLNDMSALSLETKLFNETLSM

PVALAPVGLTGMYARRGEVQAAMAADKKGIPFTLSTVSVCPIEEVAPAINRPMWFQLYVLDRGFMRNALE

RAKAAGCSTLVFTVDMPVPGARYRDAHSGMSGP-NAAMRRYMPHDLGNISK-YLEDYIGWLGSNFDPSIS

WKDLEWIREFWDGPMVIKGILDPEDAKDAVRFGADGIVVSNHGGRQLDGVMSSARALPAIADAVKGAILA

DSGIRNGLDVVRMLALGADTVLLGRAFVYALAAAGGQGVSNLLDLIDKEMRVAMTLTGAKSISDINADCL

QAI

>D0SAD2|D0SAD2_ACIJO

IISSANDYREAARRRLPPFLFHYIDGGAYAEYTLKRNVEDLSKIALRQRVLNDMSQLSLETKLFDETLSM

PVALSPVGLTGMYARRGEVQAAVAADKKGIPFTLSTVSVCPIEEVAPAIQRPMWFQLYVLDRGFMKNALE

RAKAAGCSTLVFTVDMPVPGARYRDAHSGMSGP-NAAMRRYMPHDLGNISK-YLEDYIGWLGSNFDPSIS

WKDLEWIREFWDGPMVIKGILDPEDAKDAVRFGADGIVVSNHGGRQLDGVLSSARALPPIADAVKGKILA

DSGIRNGLDVVRMLALGADTCMLGRAFVYALGAAGGEGVSNLLDLIDKEMRVAMTLTGAKTIADITSDCL

KLE

>D0SHZ4|D0SHZ4_ACIJU

IISSANDYREAAKRRLPPFLFEYIDGGAYAEYTLKRNVEDLSKIALRQRVLNDMSELSLETQLFGENLAL

PVALSPVGLTGMYARRGEVQAAVAADKKGIPFTLSTVSVCPIEEVAPAIQRPMWFQLYVLDRGFMKNALE

RAKAAGCSTLVFTVDMPVPGARYRDAHSGMSGK-NAAMRRYMPHDLGNISK-YLEDYIGWLGNNFDPSIS

WKDLEWIRDYWEGPMVIKGILDPEDAKDAVRFGADGIVVSNHGGRQLDGVLSSARALPSIASAVKGKILA

DSGIRNGLDVVRMLAMGADICMLGRAFVYALGAAGGAGVSNLLDLIEKEMRVAMTLTGARTIADITSDCL

LEK

>D3UZD7|D3UZD7_XENBS

IISASTDYRTAAQAKLPPFLFHYIDGGAYAEHTLKRNTEDLSNIELRQRVLKNMSELNLETRLFGEKMAM

PVALAPVGLSGMYARRGEVQAARAAAKKGIPFTLSTVSVCPIEEVASAIDRPIWFQLYVLDRGFMHNVLE

RAQAAGVKNLVFTVDMPIPGARYRDAHSGMSGP-NASMRQILPHDLGNISA-YRKDYIGWLGNNFDPSIS

WKDLEWIRDFWKGPMILKGILDPEDAKDAVRFGADGIVVSNHGGRQLDGVLSTARALPAIADAVKNTILT

DSGIRTGLDVVRMLALGADSVLLGRAFVYALAAAGEAGVSNLLDLIDKEMRVAMTLTGAKSIAEINSNLL

GKQ

>D3VC53|D3VC53_XENNA

IISASTDYRAAAQAKLPPFLFHYIDGGAYAEHTLQRNTADLSDIELRQRVLKDMSELSLETSLFGEKMSM

PVALAPVGLSGMYVRRGEVQAARAAAKKGIAFTLSTVSVCPIEEVAAAIDRPIWFQLYVLDRGFMRNVLE

RAQAAGVKNLVFTVDMPVPGARYRDAHSGMSGP-NASMKRIFPHDLGNISA-YRGNYMEWLGNNFDPSIA

WKDLEWIRDLWKGPMILKGILDPEDAKDAVRFGADGIVVSNHGGRQLDGVLSTARALPAIADAVKSTILT

DSGIRTGLDVVRMLALGADSVLLGRAFVYALAAAGEAGVSNLLDLIDKEIRVAMTLTGARSISEINSELL

QHQ

>D4GL46|D4GL46_PANAM

IISAASDYRAAAQRILPPFLFHYLDGGAYAEHTLQRNVADLSEVALRQRILRNMSELSLETTLFNETLSM

PVALAPVGLCGMYARRGEVQAARAAAGKGIPFTLSTVSVCPIEEVAPQINRPMWFQLYVLDRGFMRNALE

RAKAAGCSTLVFTVDMPTPGARYRDAHSGMSGE-NAALRRYWPHDLGNIST-YLEDYIGWLAKNFDPSIS

WQDLEWIRDFWDGPMVIKGILDPEDARDAVRFGADGIVVSNHGGRQLDGVLSSARALPAIADAVKGTILA

DSGIRNGLDVVRMIALGADSVLLGRAFIYALATQGQRGVEHLLTLIEKEMKVAMTLTGAKRIADITQASL

VQA

>A7MNF6|LLDD_CROS8

IISAASDYRAAAQRILPPFLFHYIDGGAYAEYTLKRNVEDLSQVALRQRVLKNMSDLSLETTLFNETLSM

PVALGPVGLCGMYARRGEVQAARAASAKGIPFTLSTVSVCPIEEVAPVMNRPMWFQLYVLDRGFMRNALE

RAKAAGCSTLVFTVDMPTPGARYRDAHSGMSGT-NAAMRRYWPHDLGNISA-YLEDYIGWLANNFDPSIS

WKDLEWIREFWDGPMVIKGILDPEDARDAVRFGADGIVVSNHGGRQLDGVLSSARALPAIADAVKGTILA

DSGIRNGLDVVRMIALGADSVLLGRAYLYALATHGEKGVANLLNLIEKEMRVAMTLTGAKSIKEITRESL

PSP

>B5XMV0|LLDD_KLEP3

IISAASDYRAAAQRILPPFLFHYIDGGAYAEHTLRRNVEDLSDVALRQRILRNMSDLSLETTLFNEKLAM

PTALAPVGLCGMYARRGEVQAAGAADDKGIPFTLSTVSVCPIEEVAPTIKRPMWFQLYVLDRGFMRNALE

RAKAAGCSTLVFTVDMPTPGARYRDAHSGMSGP-NAALRRYWPHDLGNISA-YLEDYIGWLANNFDPSIS

WKDLEWIRDFWDGPMVIKGILDPEDARDAVRFGADGIVVSNHGGRQLDGVLSSARALPAIADAVKGTILA

DSGIRNGLDVVRMIALGADSVLLGRAYLYALATHGKQGVANLLNLIEKEMKVAMTLTGAKTIREISRDSL

VQN

>B1EHX1|B1EHX1_9ESCH

IISAASDYRAAAQRILPPFLFHYMDGGAYSEYTLRRNVEDLSDVALRQRILKNMSNLSLETTLFNEKLSM

PVALAPVGLCGMYARRGEVQAAKAADAHGIPFTLSTVSVCPIEEVAPAIKRPMWFQLYVLDRGFMRNALE

RAKAAGCSTLVFTVDMPTPGARYRDAHSGMSGP-NAAMRRYWPHDLGNISA-YLEDYIGWLGNNFDPSIS

WKDLEWIRDFWDGPMVIKGILDPEDARDAVRFGADGIVVSNHGGRQLDGVLSSARALPAIADAVKGAILA

DSGIRNGLDVVRMIALGADTVLLGRAFLYALATAGQAGVANLLSLIEKEMKVAMTLTGAKSIKEITGDSL

QEL

>D2U840|D2U840_XANAP

IISAATDYRAAAQRRLPPFLFHYIDGGAYAEHTLRRNVADLADIALRQRVLRDMSALDLHTELFGERLAL

PVALAPVGLTGMYARRGEVQAARAAAAKGVPFTLSTVSVCPIEEVAPAIDRPMWFQLYVLDRGFMRNALE

RAKAVGVTTLVFTVDMPTPGARYRDAHSGMSGP-NAALRRMLPHDLGNISA-YRADYIGWLGANFDPSIS

WKDLEWIREFWTGPMVIKGILDPDDARDAVRFGADGIIVSNHGGRQLDGVLSSTRALPAIADAVKGKILA

DSGIRSGLDVVRMLALGADAVLLGRAFVYALAAGGQAGVENLLSLIEKEMRVAMTLTGAKSIDAITRDSL

AQV

>B0RLM2|LLDD_XANCB

IISAASDYRAAAEARLPPFLFHYMDGGAYAEHTLRRNVSDLADIALRQRVLRNMSDLSLSTELFGETLAM

PVALAPVGLTGMYARRGEVQAARAAAARGIPFTLSTVSVCPIEEVAPAIDRPMWFQLYVLDRGFMRNALE

RAKAAGVTTLVFTVDMPTPGARYRDAHSGMSGP-NAPLRRMLPHDLGNIST-YRQDYIGWLAANFDPSIS

WKDLEWIREFWTGPMVIKGILDPEDARDAVRFGADGIVVSNHGGRQLDGVLSSARALPAIADAVKGKILA

DSGIRSGLDVVRMLALGADAVLLGRAFVYALAAGGQAGVENLLTLIEREMRVAMILTGTHSVAEISGDAL

SRV

>B8L5A3|B8L5A3_9GAMM

IISASTDYRAAAQRRLPPFLFHYIDGGAYAEHTLKRNVSDLSDIALRQRILRNMSDLSLETELFGEKLAM

PVALAPVGLTGMYARRGEVQAARAADSRGIPFTLSTVSVCPIEEVAPAIQRPMWFQLYVLDRGFMRNALE

RAQAAGVTTLVFTVDMPVPGARYRDAHSGMSGP-NASLRRIGPHDLGNIST-YREDYIGWLGSNFDPSIS

WKDLEWIREFWKGPMLIKGILDPDDARDAVKFGADGIVVSNHGGRQLDGVLSTARALPAIADAVQGKILA

DSGIRTGLDVVRMLALGADTVLLGRAFVYALAAQGEAGVANLLDLIAKEMRVAMTLTGARRIADIGRDSL

VSL

>A4XYG7|LLDD_PSEMY

IISASTDYRAAAERKLPPFLFHYADGGAYAEHTLRRNVADLSNIELRQRVLKNMSELDLSTELFGEKMSM

PVGLAPVGLTGMYARRGEVQAAKAAAAKGIPFTLSTVSVCPIEEVAPAIDRPMWFQLYVLDRGFMRNALE

RAKAAGCSTLVFTVDMPVPGARYRDAHSGMSGP-NGPLRRVLPHDLGNISA-YRADYIGWLGANFDPSIS

WKDLEWIRDFWDGPMVIKGILDPEDARDAVTFGADGIIVSNHGGRQLDGVLSSARALPAIADAVKGKILA

DSGIRTGLDVVRMLALGADTVLLGRAFIYALAVAGQAGVSNLLDLIEKEMRVAMVLTGAKSIAEITSDLL

KER

>A3L3A6|A3L3A6_PSEAI

IISASTDYRAAAQRKLPPFLFHYIDGGAYAEYTLRRNVEDLSAIALRQRVLKNMSELSLETRLFDETLAM

PVALAPVGLTGMYARRGEVQAARAAAAKGVPFTLSTVSVCPIEEVAPAIDRPMWFQLYVLDRGFMRNALE

RAKAAGVTTLVFTVDMPVPGARYRDAHSGMSGP-YAAPRRILPHDLGNISA-YREDYIGWLGANFDPSIS

WKDLEWIREFWDGPMVIKGILDPEDARDAVKFGADGIVVSNHGGRQLDGVLSSARALPAIADAVKGAILA

DSGIRTGLDVVRMIALGADSVLLGRAFVYALAAAGEAGVRNLLELIEKEMRVAMVLTGAKSIGEISADSL

REL

>B1J244|LLDD_PSEPW

IISASTDYRAAAQRKLPPFLFHYADGGAYAEHTLRHNVSDLAGIALRQRVLKNMSELSLETRLFDETLSM

PVALAPVGLTGMYARRGEVQAARAAAAHGIPFTMSTVSVCPIEEVAPAIDRPMWFQLYVLDRGFMRNALE

RAKAAGVKTLVFTVDMPVPGARYRDAHSGMSGA-NGPMRRVLPHDLGNISK-YRADYIGWLGSNFDPSIS

WKDLEWIREFWDGPMIIKGILDADDARDAVKFGADGIVVSNHGGRQLDGVLSSARALPAIADAVKGKILA

DSGIRSGLDVVRMIALGADTVLIGRAFLWALAVHGQAGVKNLLELFEKEMRVAMVLTGAKAISEISRDSL

REL

>A4TKI4|LLDD_YERPP

IISASTDYRAAAQRKLPPFLFHYIDGGAYNEQTLRRNTADLADIALRQRVLKNMSELSLETQLFGETQAM

PVVLGPVGLSGMYARRGEVQAARAADKKGIPFTLSTLSVCPIEEVAPAIARPMWFQLYVLDRGFMRNALT

RAQAAGVKTLVFTVDMPVPGARYRDAHSGMSGP-NAAARRLLPHDLGNISA-YLEDYMGWIATNFDPSIS

WKDLEWVREFWQGPMIIKGILDPEDAKDAVKFGADGIVVSNHGGRQLDGVLSTARALPAIADAVKGTILA

DSGIRTGLDVVRMIALGADSVLLGRAFVYALATAGEAGVINLLTLIEQEMRVAMTLTGAKRIADINRDSL

VSE

>C4ULK3|C4ULK3_YERRU

IISASTDYRAAAQAKLPPFLFHYIDGGANAEHTLRRNTEDLSGIALRQRVLKNMSELSLETKLFDEILSM

PVVLAPVGLTGMYARRGEVQAARAAAKKGIPFTLSTVSVCPIEEVAPAIDRPMWFQLYVLDRGFMRNALE

RAKAAGVKTLVFTVDMPVPGSRYRDAHSGMSGP-NAAIRRVLPHDLGNVSA-YREDYIGWLGNNFDPSIS

WKDLEWIREFWQGPMIIKGILDPEDAKDAVRFGADGIVVSNHGGRQLDGVPSTAHALPAIADAVKGKIFA

DSGIRSGLDVVRMIALGADSVLLGRAFIYALATAGEAGVANLLELFDKEMRVAMTLTGAKSISEIGAGSL

KNG

>A8GIL1|LLDD_SERP5

IISASTDYRAAAQAKLPPFLFHYIDGGAYAEHTLRRNTEDLAGIALRQRILRNMSDLSLETSLFGEKLAM

PVILGPVGLTGMYARRGEVQAAKAAAQKGIPFTLSTVSVCPIEEVAPAIDRPMWFQLYVLDRGFMRNALE

RAKAAGVKTLVFTVDMPVPGARYRDAHSGMSGP-NAAVRRMLPHDLGNVSA-YREDYIGWLGTNFDPSIS

WKDLDWIREFWQGPMIIKGILDPEDAKDAVRFGADGIVVSNHGGRQLDGVLSTAHALPAIAEAVKGTLLA

DSGIRSGLDVVRMIALGADGVLLGRAFAYALAAAGQAGVANLLELIDKEMRVAMTLIGAKTIADISADSL

QGL

>D1RZN1|D1RZN1_SEROD

IISASTDYRAAAQAKLPPFLFHYIDGGAYAEHTLRRNTEDLANVALRQRVLRNMSDLSLETSLFGEKLAM

PVILGPVGLTGMYARRGEVQAAQAAAQKGIPFTLSTVSVCPIEEVAPAIDRPMWFQLYVLDRGFMRNALE

RAKAAGVKTLVFTVDMPVPGARYRDAHSGMSGP-NAALRRMLPHDLGNVSA-YREDYIGWLGTNFDPSIS

WKDLEWIREFWEGPMIIKGILDPEDAKDAVRFGADGIIVSNHGGRQLDGVLSTARALPAIADAVKGTLLA

DSGIRNGLDVVRMIALGADSVLLGRAFVYALAAAGGAGVSNLLELIDKEMRVAMTLTGAKTIAEIGAGSL

AGH

>D4E265|D4E265_SEROD

IISASTDYRAAAQAKLPPFLFHYIDGGAYAEHTLRRNTADLADIALRQRILKNMSELSLETTLFGEKLAM

PVILAPVGLTGMYARRGEVQAARAAAAKGIPFTLSTVSVCPIEEVAPAIDRPMWFQLYVLDRGFMRNALE

RAQAAGVKTLVFTVDMPVPGARYRDAHSGMSGP-NAAMRRVWPHDLGNVSA-YREDYIGWLGANFDPSIS

WQDLEWIREFWQGPMIIKGILDPEDARDAVRFGADGIVVSNHGGRQLDGVLSTARALPAIADAVKGAILT

DSGIRNGLDVVRMIALGADSVMLGRAFVYALAAAGEAGVANLLSLIDKEMRVAMTLTGAKSIGDITPELL

KSA

>C6DIF2|C6DIF2_PECCP

IISASTDYRAAAQRKLPPFLFHYVDGGAYGEHTLRRNTADLADIALRQRILKNVSDLSLETQLFGEKLAM

PVVLAPVGLTGMYARRGEVQAARAAAQKGIPFTLSTVSVCPIEEVAPTIERPLWFQLYVLDRGFMRNVLE

RAQAAGVKTLVFTVDMPTPGARYRDAHSGMSGP-NAAIRRVLPHDLGNVSA-YRENYIGWLAENFDSSIS

WQDLAWIREMWKGPMIIKGILDPEDAKEAVRFGADGIVVSNHGGRQLDGVLSTAHALPAIADAVKGTILA

DSGIRTGLDVVRMIALGADSVMLGRAFVYALAAAGEAGVVNLLNLIEKEMRVAMTLTGTKSIADITTDSL

QAT

>A4A9T3|A4A9T3_9GAMM

----MSDYERRARRCLPRFLYDYAAGGANNEETLSANCADFSKIRLRQRVMYDVSRGSTDTTLLGQPASM

PLALAPVGMAGMYARRGEVQAAKASETVGIPFTGSTMGVCSINEINAATNTAAWFQLYMLDRDFVQEMLQ

NAWDSGTRTLIFTVDLAVPGLRLRDFRNGMIGGWMGKASQMLPHFLGNLSG-KVNSYKSFVESQFDPSVT

WEDIRWLRDQWKGQLLIKGVLEADDARAARDCGAEGVVVSNHGGRQLDAVASSISKLPAVVDAVGSEVFI

DGGIRSGLDVVRAVALGARGVLMGRPWIYALAVNGEAGVRNLLEIFQREIAIALALTGVNSVQELNRELI

DSE

>A5V6U0|A5V6U0_SPHWW

TLASHADFRRIARSRIPPLIFDYFEGGAGDEMTIHENETAFAAVRLPQRVMVDMSAVETAGDLLGEACSM

PLALAPIGFAGLMCRRGETQAARAAEAAGVPFCLSANAICSVEEVAQAARRPFWFQLYMMDRDVVVDLLR

RAWDGGCRTLVFTVDLAVPGIRRRDIRNDLFGG--TTPAKLLPHTFGNLAP-YVAGFGGWLMRQFDAGVT

WKDIAWLRNQWKGRLVIKGILDRRDATMALDAGADALVVSNHGGRQLDGVAPTAVALPAIARAVGGPLLV

DGGVRSGQDVLKALLLGADGVLIGRAWAYAAAAGGEAAIAALLARFQVELRTAMTLAGFADIDTIRDRRI

PAS

>A1U351|A1U351_MARAV

YPATAADYQQLARRKLPRFLADYLDGGATEEHTLRANVRGWQDIALRQRVLIDVDNVDTRTELAGQSCSM

PVALAPLGLAGMMAQRGEAQAVKAANSAEVPFTLSTVGICPLAEVKAAATAPFWFQLYMIDRGYVENLLK

KAWDSGCQTLIFTIDLPLPGPRHRDTRNGLNSAARSVALKAQPLTFGNLSD-AVDSFKQWVDTQFDASVT

WQAIEWLRERWPGKLILKGILEVDDAKAAVNVGADGIVVSNHGGRQLDGVAATARKLPDIVAAAGNEILV

DGGIRNGVDVFRALALGANGVMIGRPWAWALAAEGQAGLTRLLNTWQQELKLAMTLTGVTRIADINETHL

DRT

>A1WBH5|A1WBH5_ACISJ

PA-SVADWRRLAAQRLPRFLFDYIDGGASDERTLAANVQDFAALRLRQRVLVDVAQVDTRATLAGQPCAL

PLALAPIGLAGMAARRGEAQAARAAHAAGVPFTLSTVGICPISEVAAAAGAPPWFQLYMLDRGAVRALLD

GAWAAGCRTLVFTVDLPLPGMRLRDIRHGMAASARPALIRAAPLRFGNLTA-QVGAFKAWVDAQFDPSVT

WQDIDWLRGQWKGRLLLKGILDVQDAQAAVQVGADGIVVSNHGGRQLDSVASTAAKLPAIAQAVGAEVLV

DGGVRSGVDVFKALALGARGVLIGRPWVWALAAQGEAGVRTLLAQWQRELLLAMTLAGVTRVADIGPQHL

DTD

>A9H4V1|A9H4V1_GLUDA

FL-NLHDYAPAARRLLPRALFAYINGSAGDGRALAANRAAFDRWCLVPHVLRGVSRRSARTHLMGHHYAM

PVGISAFGAAAVIGFDADRAMARAAYAAGVPYQLSANSITPMEDVIRDNPD-AWFAAYLPNPSLIDRMLT

RIGRAGFRTLVITVDVPVAARREDETRAHYAMPLRPSARLAFIPHIQNITG---ARSVGAVGGAAD--FN

WDHIRAIRRSWPGRLVLKGILSAQDAVTAQQIGADGIIVSNHGARLCDCVISPLEALPAIRQAC-PTVLL

DSGVRRAGDVITAIALGADGVMIGRPFFFATILGGQPGLAHAIGLIAGELDRDMAFLGLLDLRESRENRL

HRR

>B1FTY1|B1FTY1_9BURK

NHLSIADLERSASRRLPASIFGYVSGGAEDHRAVVGNRTAFNRWVLVPRMLTGVAERSQEVEIFGQRYTS

PVGIAPMGLAGLCAYEGDLQLAAAARDAKVPFVLSAASTVPLEKVAMAAPG-SWYQGYLSDRSTITPLLA

RIERAGFGVLVITVDVPLAAQRENELRNGFSVPLRLSRRLVYVPHFENFTA-NRATGDHR---SGRAALC

WNDIHWIRSQWKGTLVVKGILHPDDALRAKQAGADGIIVSNHGGRQLDGALATLDALPAIT-AVAGPVIL

DSGVRRGTDVIKALSLGARMVLVGRPAMYGLAVGGHAGVRHALQLLRREIDVDLALLGCPRIEKLNRDFV

QVS

>B1LST6|B1LST6_METRJ

DLLALDDFERHARKLLPPMIFQYVSGGVETGSALAQSRGAYADYALVPRLMRDTSARDTATTLFGQTYAA

PFGVGPLGGAAFIAYRGDLVLAEAARRMNLPMCLSASSLIRLEDVHAQNPQ-AWFQGYLPDQNRIDRLLD

RVEATGYRTFVVTADTPTLGNREHNIRSGFSMPIKVTPKVAWAPHFENTEA-ERGAVRNT---IARDQLS

WKNLEAIRKRWSGNLLVKGLLAPEDVDIARACGADGVILSTHGGRQLDYAVAPLDVLPEIAARKGGKIIV

DSGVRRGTDVMKALALGADFVLLGRPFMFAAALGGVPGVEHAMRILKEELNRDMALIGVNRLSELNPDFL

RRA

>A3K480|A3K480_9RHOB

RIFALDDLEAAAARRLPRPLFRFIAGGSETSGSLNANRQSYGEIFFKPRTLRDVGKRQQAVELFGRRHAA

PFGIAPMGAAALMGFDADVAMARAAQAAGVPFILTSAALTPLERVREATGT-GWFAGYLPDRERMGALVD

RVANAGYEVLVVTADVPVPANREQNLRSGFSVPLRLTPSLLLVPHFENFSA-SRPQAPD----TSRARLT

WDDLAWVRQRWSGPLIVKGILAPDDAVAARQAGADGVIVSNHGGRQLDGAVAPLQALPSIV-SVAGTVMI

DGGLRRGTDVLKALALGADFVFLGRPFLYAAALAGEAGVAHAIDLLSQEIDRDLALLGCPDIATLDARFL

GTF

>A3JY84|A3JY84_9RHOB

FL-AMDDFEPAARRRLPRPLFGYISGAAETNAARQDNRDALDAWRLVPNVLRGVEGRSTASTLLGERYTA

PFGVAPMGLSALMARDGDVALARAASAAGLPFVLSGSSLTRMEDVVTANPK-AWFQAYLPEEDRIRALIA

RVKAAGFGTLMLTADTAVLANRENNLRAGFSTPLKVSPRLIWMPHFENSDA-VRQAARDF---GRKDHLN

WDHLALMRDLWPGKLLLKGVIAPADVAHARALGCDAVVLSNHGGRQLDHAISPLRLLPEAR-AQAGGLLI

DGGIRRGTDVIKALALGADMVLVGRPFLYAATLGGQPMVERAADILKAEVHRNLGLLGLRDLSEIGPGIL

-HP

>D0DAW6|D0DAW6_9RHOB

FL-TLDDFEPAARRRLPRPLFGYVSGAAETNAARQDNRDALDAWRLVPNVLRGVEGRSTACTLLGESYAA

PFGVAPMGLSALMARDGDVALARAAAVAGLPFVLSGSSLTRMEDVVTANPN-AWFQAYLPEEDRIRALIA

RARMAGFGTLVLTADTAVVANRENNTRAGFSTPLRVRPRLIWMPHFENSDA-VRQAARDF---GRKDHLN

WDHLTLMRDLWPGKLVLKGVIAPADVAQARALGCDAVVMSNHGGRQLDHAISPLRIMPEAR-AQAGALLI

DGGIRRGTDVIKALALGADMVLVGRPFLYAATLGGQPMVERAAEILKAEVHRNLGLLGLRGLSEISPEIL

HPV

>A3JK74|A3JK74_9ALTE

SILNLHDFEKAARGHLPRPIFGYISSAAEDGKTLHANRSAFDNYCFLPRALVDVSKVSLQTELFGKQYAA

PFGIAPMGISALSAYRGDKVLAEGAAKANIPMIMSGSSLIPMEDVSGPNGT-DWFQAYLPDEEGIEALLA

RVEKSGFKNLVITVDYPVPPNSENHVRSGFSSPLRPSVRLLIMPHFENNYA-TRNVNRDF---SGRSHLN

WESLALVRRLWPGNLIVKGILHPQDALKAEAAGADGIIVSNHGGRQLDGTIAPMNVLSAIVKAV-SPVMI

DSGFRRGSDVLKALGLGAKFVFVGRSFNYAAAYAGEEGVSHAAKLLSAEIQRNMALLGINRVEEMNSERM

LKD

>B9K4L3|B9K4L3_AGRVS

DVLSLDDFEVLAKRHLPGPLFGYIAGASETNASLRMNAEAFRDISLTPRVLRNVSTRTTKTILFGEEWSA

PFGIAPMGISALMAYRGDLVLAKAAQDAGIAMIMSGSSLIRLEEIIEAAPR-SWFQAYLPEPDRIDGLID

RVASAGYKTLVLTVDTAVLANRENNIRAGFSTPLRPSLRLTWIPHFENSYA-TRNVMRDF---GKKDHLS

WGHFSRIRQRWTGNLVVKGILHPEDAAMAAERGADGIIVSNHGGRQLDGAIAPMKALPAIVDRVGSVVMI

DGGIRRGTDMVKALALGAKFVFVGRPFVYAVAVGSKAGVAKATSILSDELHRDMGLLGVTEIIEL-PSIL

TRI

>B6A2Q2|B6A2Q2_RHILW

AMLCLDDFEPKAKRHLPKPLYGYISGGSETNASLRNNLDAFQAYAFRPRILQDVSKRSTETTLFGQTFTA

PFGIAPMGISALMAYRGDIVLAAGAAQVGIPMIMSGSSLIRLEEVVAAAPA-TWFQAYLPEPERIDALVD

RVAAAGFGTLLLTVDTATLPNRENNIRAGFSTPLRPSLALAWIPHFENSYA-TRNVTRDF---GRRDHLN

WSHLERIRNRWNGKLIVKGILHPDDAARAAETGADGVIVSNHGGRQLDGAISPLAALPEIVERLGDPIMI

DGGFRRGTDIIKALALGARFVFVGRPFLYAAAVAGLPGVLRAADILKSELHSNMALLGVTTIEQISRGHL

VSA

>B3PXW4|B3PXW4_RHIE6

DVLCLDDFEIKARRHLPKPLFGYIAGATETNASLRHNAEAFQAYAFRPRVLRDVSTRSTATSLFGKTHAV

PFGIAPMGISALMAYRGDIVLAQGADQSGIPMIISGSSLIPLEEIAAASPQ-AWFQAYLPEPDRIDALID

RVAAAGIDTLLLTVDTATLPNRENNVRAGFSTPLRPGLRLAWIPHFENSYA-TRNVTRDF---GKRDHLN

WSHLERIRKRWSGKLVVKGIMHPEDAARAADTGADGVIVSNHGGRQLDGTASPLQVLPEIAARVGDAVMV

DGGIRRGTDIMKALALGACFVFVGRPFLYAAAVAGLPGVLRAADILKTELYSNMALLGVTRVGDISADYI

TRA

>C6AWF6|C6AWF6_RHILS

EVLCLDDFEIKARRHLPKPLFGYIAGATETNASLRHNAEAFQAYAFRPRVLRDVSKRSTETSLFGKTHAA

PFGIAPMGISALMAYRGDIVLAQGADQSGIPMIISGSSLIPLEEIAAVSPQ-AWFQAYLPEPDRIDALID

RVGAAGLRTLLLTVDTATLPNRENNVRAGFSTPLRPGLRLAWIPHFENSYA-TRNVTRDF---GRRDHLN

WNHLERIRNRWSGKLVVKGIMHPDDAALAVDTGADGVIVSNHGGRQLDGTASPLQVLPEIASRVGDAVMV

DGGFRRGTDIMKALALGACFVFVGRPFLYAAAVAGLPGVLKAADILKTELHSNMALLGVTKVGDISADYI

THA

>A9BSM3|A9BSM3_DELAS

RILSLDDFERAARHHLPAPIFAYVSGGCETDKSLRANREAFDAHRWVTRVLTDTSRRTLATPLLGQTWAA

PFGIAPMGISALSAYRGDLVQTRAAAAARIPAILSGTSLIRMEEVVQANPQ-AWFQAYVPETERIVQLLE

RVEAAGFATLVVTVDTPVSGNRENNIRAGFSTPLRPGLRLAWMPHFENSQA-TRTVTRDF---GARDHLS

WEHLALIRRRWKGRLVVKGILSPQDALAARDAGADAIILSNHGGRQLDGAVSPLHMLPLVVGALGPPVMI

DSGFRRGNDVLVALALGAHFVFVGRPFNYAGAVGGEAGVLHAIAILAAEMRRNMALIGVQGLQELGPQHL

RPR

>B7WXK6|B7WXK6_COMTE

NILSLQDFEAAARKHLPRPLYGYVSGAAEDCVSLQANRDSFQQYGFSSRVMVDVSQRHQKVELFGQTWDS

PFGVAPVGISAISAYRGDLVLAQTAAANRIPAIMSGTSLIPMEEVAKAAPS-TWFQAYLPDTTRIDGLIE

RIEAAGFGTLVITVDIPVWANRENNVRTGFSMPLRPSLRLAYMPHFENSFA-TRTAIRDT---TGRDHLN

WKHIERIRQRWQGNLIIKGILNEDDAVMAADIGAQGIVVSNHGGRQLDGVVAPLQMLPYVVDRVGHAVMM

DSGIRRGSDVLKAVALGARMVFLGRPFMYAAAVGGAQGVDHAITLLRDEVDRNMAMLGATSMAEITRDCL

ATR

>A9C0W3|A9C0W3_DELAS

QVLSLDDFERLARRHLPRPIHAYISGAVEDNASLADNRRAFAELAFVPRALVGVAQRDPSFELFGRRYGA

PFGVAPMGIAALSAYRGDLVLAQAAQQAGVPAIMSGSSLIRLEEVMAAAPH-TWFQAYLPDQGQIDALLD

RVAAAGVQTLVITVDTPVAANRENNVRAGFSTPLRPGPSLAWMPHFENNYA-HRSVMRDF---SDRSHLA

WPHLAAIRQRWQGQLVVKGILSAADAVLARDHGADGLIVSNHGGRQLDGAVAPLRVLPGIVRAVPGPVML

DSGVRRGTDVLKALALGARCVFVGRPFNYAASVAGPAGVTHAMALLREEVLRDMAMLGATRLDQVTPACV

RHA

>A9HXW7|A9HXW7_BORPD

RFLSLHDFEAAARRRLPRPIFEYVAGSVEDRQAERDNRQAFSRYGFRTRVLVDVSSRRQDVELFGQRYAS

PVGIAPMGIAALTAYRGDIVLARAAQAANVPCIMSGSSLIRLEEVMDAAPG-TWFQAYLPDDSHIGALID

RVAAAGVQTLVLTVDTPVQANRENNVRAGFSTPLKPGLGLAYMPHFENNYA-TRRVLRDF---SDRGYLN

WTHAAQIRRRWQGPMVIKGILGTDDARRAREQGMDGIIVSNHGGRQLDGAVSPLRVLPEILEQAGGTVML

DSGVRRGTDAMKALALGAHAVFVGRPFNYAASVAGEDGVRHALQLMRDEIARNMGMLGITRLQELDRSFL

ALD

>B9Z1X3|B9Z1X3_9NEIS

GVLALDDFEALARQRLPRPIFGYVAGAAEDNASLADNRAAFGDYGFQTRVLVDVSQRNQSVELFGRRYAA

PFGIAPMGISALSAYRGDIVLARAAQAANIPAILSGTSLIPLEEVIEQAPG-TWFQAYLPDPARIDALVQ

RVARAGVETLVLTVDIPVSANRENNVRTGFSTPLRPSLALAYMPHFENSFA-TRSVLRDF---SARDHLS

WPHFDRIRRQWKGPLIIKGILSAEDARQARLHGADGIIVSNHGGRQLDGAVSPLRVLPDIVDVAQ-TVMM

DSGVRRGSDVLKALALGARCVFVGRPFNYAAAVAGEAGVAHACRLLYDEVDRNMAMLGVNSCAELHAGLL

HRK

>A9BS25|A9BS25_DELAS

RMLSLHDFEEAARRRLPRPIFGYIAGAAEDNASLRDNREVFGEWGLTTRVLADVSQRSQAVELFGERYAS

PFGIAPMGINALSTYRGDLVLARAAQRAGIVSVMSGTSLIPMEEVARESPA-TWFQAYIPDQARIDALID

RVERAGFRTLVVTVDIPISANRENNIRTGFSTPLKPSLRLAWMPHFENSFA-TRSVMRDF---SARDHLS

WRHIEAIRRRWKGPLVIKGVLSVEDALQARRVGADGIVLSNHGGRQLDGAVSAMRILEDVVAALGPPVLI

DGGFRRGSDVLKAVALGARMVLVGRPFNYAAAVGGEAGVLHAIGLLRDEVDRNLAMLGASSCGALDRSHI

RRA

>B9B909|B9B909_9BURK

NMLSLHDFEARARRVLPRPIFGYVSGAAEDNRTRDDNRSVFDEFGFTTRVLRNVSARTQAVDLFGQRFAA

PFGIAPMGINALSAYRGDIVLARAAQAAGIASIMSGSSLIPLEAVAAAAPS-TWFQAYLPDPERIAALLE

RVARAGYRTLVVTVDIPVAANRENNVRTGFSTPLRPSMRLAWMPHFENSFA-TRHVLRDF---SARDHLD

WTHLAQIRAQWKGSLVVKGILSVEDALAARDVGADGIILSNHGGRQLDGAVSPMRILRDVVTALEPPVML

DGGFRRGADVLKAIALGARMVFVGRPFNYAMAVAGEAGVAHAIRLLQEEVDRDMAMLGARTCRELHPGLI

RKR

>B4E9V0|B4E9V0_BURCJ

NMLSLHDFEAAARRVLPRPIFGYVSGAAEDNRTRDDNRAVFDEYGFATRVLRNVSQRQQTVELFGRRYAS

PFGIAPMGIHALSTYRGDIVLARAAQRAGIASIMSGSSLIPLEEVAAAAPG-TWFQAYLPDPDRIAALLE

RVARAGYRTLVITVDIPVSANRENNVRTGFTTPLRPGPRLFWMPHFENSFA-TRTVLRDF---SARDHLD

WGHLKRIRQEWKGELVIKGILSVDDAVIARDIGADGIILSNHGGRQLDGAVSPMRILPDVVRALGAPVMI

DSGFRRGSDVLKAVAMGARMVFVGRPFNYAAAVAGQAGVLHAIGLLRDEVDRNMAMLGVGQCSALTPDVL

RKS

>B2HCM4|B2HCM4_BURPS

GVLSLHDFEARARGALPRPIFGYVSGAAEDNRTRDDNRAAFDEYGFVTRVLHDVSQRQQGVELFGRRYAS

PFGIAPMGINALSTYRGDIVLARAAQHAGIASIMSGSSLIPLEDVAAAAPD-TWFQAYLPDAGRIRALVE

RVARAGYRTLVVTVDIPVSANRENNVRSGFSTPLRPSPRLFWMPHFENSFA-TRNVLRDF---SARDHLS

WAHVRQIREQWAGELVIKGVLSVEDARIAREAGADGIILSNHGGRQLDGAVSPMRILRDVVQAVGNPVMI

DSGFRRGSDVLKAVALGARMVFVGRPFNYAAAVAGEAGVAHAIRLLREEVDRNLAMLGANGCDALTPDML

RKR

>A5VE54|A5VE54_SPHWW

AAHSIDDLRDAARRTLPRMVFDYVDGGAQSESTMRQNRAAFEHHRLLSAAPVDVSRRSTATALFGQSLAM

PLIIGPTGYASAFWPKGDLALARGAAAAGVPFVVSNGANARLRDIAEASGGQAWLQLYIADKQATLGLIG

EARALGFETLEVTVDTALPGRRLRDIRNGFTVP-YWTPAKVV--MLRALP--HGEQGRSWSRSQINPAIG

WDDLKWLRDQWPGTLIVKGLLDPGQVEPAIAAGYDGIVISNHGGRQLDGAVSTLDVLPDFAAAAKRPLLI

DSGVRTGTDILKAVALGASAVQVGRATLYGLSTAGEAGVGHALGIFRTELDMAMALVGLNRVADATPAIV

RAI

>C7Q1I4|C7Q1I4_CATAD

RCATIEDVARVARRRLPLGARAYLENGGEGQHTLHRNRAAFGSYTFRPRQPRDVSGVDTGTTVLGQRIPL

PFALSPVGAPRMFHHDGELAVARAARDAGIPYGISTLANTSVEDVAEQTDSPLWFQLYIWDRSKSKEAVA

RAKAAGYQALLVNIDTSVRSERIPEKHSGLVLPSQLPLKTLFTVSFPNIGP-PDRSLEVM-SDMFDGTVC

WDDLDWIRRIWDGPIVLKGVTTVEQAREAVDHGLDAVIVSNHGGRQLDRLPATIDVLPEIADAVGDEVLV

DSGFRSGGDIATALALGAKAVLVGRAHLYGLAAAGEAGVRHCVDILARELRMTMQLNGARNIAELDRGLI

ASA

>A8LEH5|A8LEH5_FRASN

RAASVHDLRRLARRRLPGGVFDYIDGAAEDERTLAANERAYAATTFRPRVLRGLPEVSVGTEILGAPAAY

PLVLAPTGFTRIADPQGELAVARAAARAGLPYTLSTLSTRSIEEVRAVSDGRLWFQVYAWDRGLVKEMID

RAAAARYEAIVLTVDTAVFGRRERDVRRGFSLPPTIGPGTILPIRFSNVAGRDVVTLSDYINTQFDPGLS

WADLTWLRSVWAGRVVVKGIQTVADAKLAAEAGVDAIVLSNHGGRQLDGAPATLPLVAPVADAVGGEIIC

DGGVRRGSDIVKAVAAGATAAMAGRAYLYALGAAGERGVDRLLAWFAADIHRTLALLGAAGVADLGRDHL

LPA

>A4FHQ5|A4FHQ5_SACEN

NALTIEHLRRAARRTTPRSVFDYVDGAAEEEITAARNIAAYRRVTLRPDALHPVAEPELGVDLFGKRIAM

PLVFAPTGYTRMMHHHGEAAVARVAEHFGVPYSLSTVGTTSIEDVRAAAPGDLWFQLYRTDPATNELLVS

RAEAAGYSTMLLTVDTSVAGKRLKDVVNGLTIPPTLTARTILGIGFASLS----SSEDVA-RTLFDPGLD

FAALEWLRERWPGKLLVKGITTPESAREVVRRGADGVVVSNHGGRQLDRSAATLDVLPAVRAAVGATVII

DGGIRHGQDIVAARALGADAAMVGRAYLYGIMAGGQDGAVRAYEILADEYQRCMQLLGVRRSEDLGERHV

LPR

>D2S4B7|D2S4B7_GEOOG

NALSVRDLREAARRTTPRAVFDYVDGAAEEEITAARNSAAYRRVTFRPDALRSVAEPDTSVELLGRRIAM

PLVFAPTGYTRMMHHHGEAAVATVAQHVGVPYALSTVGSTSIEDVRAASPDDLWFQLYYTDPEVNEDLLA

RAEAAGYSTILLTVDTTVSGMRLRDVVNGLTIPPTLTARTVLGMTFASLS----TPAEVA-SMMFDPGLD

LGSLDRLRKRWRGDLLVKGITTPASAREVMEHGADGVVVSNHGGRQLDRSAATLDVLPAIRSAVGQPVLI

DGGVLHGQDVVAARALGADAVMIGRAYLYGLMAGGQDGVLRAYEILAEEYQRSIQLLGVRRSEDLSDRHV

FTA

>C7LZ21|C7LZ21_ACIFD

RAATIDDLRRLAKLRTPRPVFDYVDGAAEAERSMLRNEGSFADVVFRPHVLRDVSSVDPTWTVLGSPSAL

PFGFAPTGFTRMMHTDGELAVGRVAASLGIPYGLSTVGTTTPEELAAELPHRRWFQLYVWDRGPTRAFVE

RAREAGFEALILTVDVPVAGARMRDVRNGLTLPPTPSLRTFLPVRFASLET-GF-TAGSFIDRMFDPTVT

FDDIEWVRSLWSGKIVVKGVQRIDDAERLAAIGVDAIVVSNHGGRQLDRTLAPLALLPIVRERLDGEVWV

DGGVRAGSDVVAAIGLGAQFVLVGRAYLYGLMAGGERGVAVAGRILADGVTRTMALLGIRSFDELESDMV

RAR

>B2GHA4|B2GHA4_KOCRD

KAADVEALRRIARRRTPKPAFDYVDGAAGQELTYRRSREAFESVELLPRILHGTDTADLSTEITGFRSAL

PFGIAPTGFTRFMHSEGEIGGVRAAERAGIPFSLSTMGTRSIEEVRDAAPDERWFQLYLWEHDASLDLIR

RAKAAGTTTLLVTVDTPVPGQRLRDTRNGMVIPPRLTPKTVLSLKFASLS--DTGALADLISTMFDPGLN

LADLEWIREQWDGTLYVKGVLTREDARRAMSVGADGLVVSNHGGRQLDRAPVSLTALPELRDEVGPPLIL

DSGVLSGADVVTALCAGADFVLIGRAYLYGLMAGGEQGVSRVIELLEAQIRTTMMLMGAASTADLGPHGM

RAP

>C2CM78|C2CM78_CORST

EAADVWDLRKIAKRRIPKAAFDYVDGAARDEVTYDESRETFRSIRLLPNVLTGATELDLSVDIAGGRSAL

PFGIAPTGFTRFMHAEGEDAGASAATAAGIPFTLSTMGTRSVEEVERASGRRRWFQLYLWDRAASAELLR

RAAASGYDTLVVTVDTPVAGQRLRDTRNGMRIPPRLTAGTVLPVTFASLT--STGTLGELVNTMFDPGLN

FDDLEWIREQWDGKMLVKGIVNPADARTVIELGADGVVVSSHGGRQLDRVVNTLRALEAIRAELGPEIVY

DSGIMSGTDIAIALALGANFVLIGRAYLYGLMAGGREGVDRIIELLTSELETACTLLGVSSVRDLKREHV

TPW

>C3PEY8|C3PEY8_CORA7

EAADVWDLRKIAKRRTPKAAFDYVDGAARDEVSYRESRDFFRDVRLMPNVLNGANDISLSTEIAGEPAAL

PFGIAPTGFTRFMHAEGEDAGSQAARDAGIPFTLSTMGTRSVEEVAASQGNRRWFQLYLWDHSACQELIE

RAAANGYDTLVVTVDTPVAGQRLRDTRNGMRIPPRLTAGTVFPVTFASLT--STGTLGELVNTMFDPGLN

FEDLAWIRKQWTGKLFVKGIVNPEDARKVIDLGADGIVVSSHGGRQLDRVVNTLQALEAVRAEVGPEIIY

DSGIMSGVDIAIALSLGADFVLIGRAYLYGLMAGGKEGVDRVIELLAEEFKNTLQLLGVKKIEDLSRQHV

TPW

>C7NJZ2|C7NJZ2_KYTSD

KAADIADVRRIARRVTPTGPFDYVDGAANSEESMRRNTEAYRNLELRPTVLRDVGEVDLSTEVFGQRSEL

PVGLAPTGFTRMMHAAGEPAVARAAQSAGVPYTLSTMGTTAIEDLAAQVPDRRWFQLYSWDRDRARGLVE

RAQENGYDTLMVTVDTATGGLRYRDHRNGMTIPPQLTARTLVPLRFATLSS-SADSMDVI-MKTFDPTLS

WADIEWIREVWAGPLLVKGIQTPSDAQRALDAGCDGVYLSNHGGRQLDRAPVPLAELPGIREVLGPPIIV

DSGITSGVDVLGALALGADFTMIGRAYLYGLMAGGQRGVERVLDILRAELQVGMQLLGVRSVDELGPQHV

RLD

>A4FLZ5|A4FLZ5_SACEN

RAHTIGDLRAIARRRTPRAVFDYTDGAAEGETSLRRARQAFRDVEFRPSVLRDVSGVDTTTSVLGKPSAM

PFSLAPTGFTRMMNHEGETAVVRVAQRAGIPYGLSTMGTTSIEDTATAGPARKWFQLYVWDRAASRDLVQ

RAREAGYEALILTVDTPVAGARLRDMRNGLTIPPALTLKTIAPLSFASFSR-WE------INEMFDPSLN

FTDVEWLREAWDGPLIVKGLQNVPDARRVVELGADAVILSNHGGRQLDRAPTMLELLPQVREAIGDEIML

DTGILSGADIVAALALGADSCLVGRAYLYGLMAGGEQGVQRAVDILRTEVVRTMQLLGVSKVDDLDGSYA

LRR

>C5C1C6|C5C1C6_BEUC1

KALTIPELRRLALRRTPRSVFDYTDGAAEAEISLRRARAAFRSVEFQPSILHDVSDLSTATPMLGVDSAL

PFAFAPTGFTRMMQTQGESAVVRVAGRRGIPYALSTMGTTSIEDVAAASPERKWFQLYVWDRSAGEDLMA

RARAAGYEALVLTVDVPVAGARLRDARNGFSIPPALTLKTIAPLQFASLES-WDGTIADLLDALFDPTMT

MADLEWIRSQWDGPLVIKGIQTLDDARRVADAGADAIILSNHGGRQLDRAPVPLRLVPDTREAVGDEVWV

DTGILSGADVVAAIALGAHATLVGRAYLYGLMAGGERGVERAVDILEAEVRRTMKLLGVNDIASLGPQHV

RLP

>D2PV50|D2PV50_KRIFD

KALTIADLRAIAKRRTPRSVFDYTDGAAESEISLQRSRRLFAEMELQPSILRNVSEIDLGTNILGKRSEL

PFAFAPTGFTRMMNHEGESAVVKVAQQAGIPYALSTMGTTSIEDVAAAGPDRKWFQLYVWDRDAGEDLVK

RSAAAGYEALMLTVDVPVAGARLRDVRNGFTIPPSLTAKTVLPLTFASLSS-WDGTVAELLDQLFDPTMT

IDDFNWLRSIWDGPLIVKGIQTVEDARRVVDAGADAIVLSNHGGRQLDRAPTPLRILPDVREAVGTEVYL

DTGIMTGADIVAALALGADACLVGRAYLYGLMAGGQRGVERATDILTKEIRRTMALLGVPSVDALNPSHV

-RL

>A9WTC6|A9WTC6_RENSM

KASTIWDLRAMAKRRTPAAPFDYTDGAADREITMNRSRQAFDHLEFNPEVLHDVSKVDLNTKIFGKTSSM

PIGIGPTGFTRLMQSEGEYAGSAAAAAAGIPYTLSTLGTASIDDVAAHAPTRRWFQLYATDVESNLALIQ

RAAAAGNDTIILTVDTAVGGMRLRDVRNGMTIPPQLTLKTIVPLEFSSLSS-FDGTVSELLSNTFNTGLN

YQDLDWLRANWHGNLLVKGVQSVTDAQKAIDHGADGVVLSNHGGRQLDRAPLPFHLIPKVRATVGTTIMM

DTGIMCGGDIIAAIASGADFTLIGRAYLYGLMAGGQRGVARSLEILRTEMVRTMTLLGVTKISDLNPDHV

QRA

>A1R9V8|A1R9V8_ARTAT

RASTIWELRDMAKRRTPRAPFDYTDGAAEEEITLRRARQAFQDIEFRPGILRDVSTIDLRTDILGQESRL

PFGIAPTGFTRMMQSEGEYAGSQAAEAAGIPYTLSTMGTASIEDVATAAPNRNWFQLYLWDRDRSLELIE

RAAKAGNDTLMVTVDTAVAGARLRDVRNGMTIPPALTIKTVLPLTFASLSR-YTGTVADLINSMFDPTLT

YEDLDWLRETWKGKLVVKGIQTVEDARKVVDHGADGIILSNHGGRQLDRAPIPFHLLPEVTAALNSAVML

DTGIMSGADIVAALALGADFALIGRAYLYGLMAGGREGVDRTIQILEKDMTRTMALLGVSKISDLNPDHV

LLQ

>A0K098|A0K098_ARTS2

RASTIWELRDMAKRRTPQAPFDYTDGAAEAEITLRRAREAFLDIEFRPGVLRNVSSIDLSTDILGKPSRL

PVGIAPTGFTRMMQSEGEYAGSQAAEAAGIPYTLSTMGTASIEDVAEAAPNRNWFQLYLWDRDRSLELIE

RAAKAGNDTLMVTVDTAVAGARLRDVRNGMTIPPALTIKTVLPLTFASLSR-YTGTVADLINSMFDPTLT

FEDLDWLRETWKGKLVVKGIQTVDDARKVVDHGADGVVLSNHGGRQLDRAPIPFHLLPGVKEAFNSAIIL

DTGIMSGADIIAALAQGADFTLIGRAYLYGLMAGGRAGVDRAIQILEKDMTRTMALLGVSKLSELTPDHV

ILG

>B8HFD4|B8HFD4_ARTCA

RASTVWELRDIAKRRTPQAPFDYTDGAAEGEITLRRARQAFLNIEFRPGILRNVSSIDLSTDILGKPSRL

PVGIAPTGFTRMMQSEGEYAGSQAAEAAGIPYTLSTMGTASIEDVAAAAPNRNWFQLYLWDRDRSLELIE

RAAKAGNDTLMVTVDTAVAGARLRDVRNGMTIPPALTLTTVLPLTFASLSR-YT-TVADLINSMFDPTLT

FEDLDWLRETWKGKLVVKGIQTVEDARRVVDHGADGVVLSNHGGRQLDRAPIPFHLLPDVRQAFNRAIML

DTGIMSGADIVAALALGADFTLIGRAYLYGLMAGGRAGVDRTLQILEKDMARTMALLGVSRIADLTPEHV

LLA

>C0E1Z9|C0E1Z9_9CORY

KLATIDDLRKIAKRRTPAAAFDYTDGAADDEISMNRARQAFKDVEFHPSILNDVSNVDTSCEVFGGPSAL

PFGIAPTGFTRLMQTEGELAGASAAGKAGIPFCLSTLGTTSIEDVKAANPHRNFFQLYVMQREISYGLVK

RAAAAGFDTLFFTVDTPIAGARLRDKRNGFSIPPQISLGTVATLSFASLS--STGTVGELLNSAMDPSIQ

FSDLEEIRSMWPGKLVVKGVQNVEDSKKLADLGVDGIILSNHGGRQLDRAPVPFWLLPEVVREVGKDVTM

DTGIMHGADIVAAMAMGAKFTFIGRAYLYGLMAGGEAGVTRAIEILAEQVRRTMQLLQVETIDELSPKHV

QLT

>C8RUB9|C8RUB9_CORJE

DAQTIWDLRAIAKRRTPAAAFDYTDGAADEEISMNRARQAFRDVEFHPSILNDVSNVDTTAEIFGGKSSL

PFGIAPTGFTRLMQTEGELAGASAAGSAGIPFCLSTLGTTSIEDVQKANPNRNWFQLYVMEREISYGLVE

RAAKAGFDTLLFTVDTPVAGNRLRDARNGFSIPPEISLGTVVPLEFASLT--STGTVGELLDSAMDPSIK

FEDLKTIREMWPGKLVVKGVQNLPDSKKLADLGVDGIILSNHGGRQLDRAPVPFQLLPEVVREVGNDVAM

DTGIMNGADIVAAIAKGAKFTLIGRAYLYGLMAGGEAGVNRAIEILASEVRRTMRLLQVSSLDELTPEHV

QLN

>C7MBT7|C7MBT7_BRAFD

SALTIEDLRTIAKRRTPAAAFDYTDGAAEGEISMDRSVEAFEDIEFHPSILHDVSTVDTTAQILGGSSAQ

PFGIAPTGFTRLMQTEGEIAGASAAGAAGIPFTLSTLGTTSIEEVHAANPLRNWFQLYVMQREISYGLVE

RAAQAGYDTLYFTVDTPVAGARLRDSRNGFSIPPQLSLGTVIKLEFASLS--QTGTVGELLDSAMDPSID

VEDLAEIRRMWPGKFAVKGVQTLEDAKKLADLGVDAIVLSNHGGRQLDRAPVPFHLLPQVAREVGDEIIL

DTGIRSGADIVAAIALGADFTLIGRAYLYGLMAGGRQGVDRAIAILSDQVERTMKLLQVPTLQDLGPEHV

QLS

>D0WMV1|D0WMV1_9ACTO

NSLTVWDLRKIAKRRTPKAAFDYTDGAAEGELSLRRARRAFQDIEFHPDILHPAEDVDTSCEILGGPSSM

PFGIAPTGFTRLMQTEGEIAGAGAAGAAGIPFTLSTLGTTSIEDVKAANPRRNWFQLYVMKREISYALVE

RAKKAGFDTLMFTVDTPVAGARLRDKRNGFSIPPQITLKTVLKLEFASLK--STGTVGELLDSAMDPTIS

HDDLAVIRSMWDGKIVVKGVQTVADAKRLADAGVDGVLLSNHGGRQLDRAPVPFHLLPHVVREVGKAVMV

DTGIMNGADIVASVALGADFALIGRAYLYGLMAGGRAGVDRTIAILRDELVRTMKLLGVSSIAELEPRHI

QLT

>C4LGA5|C4LGA5_CORK4

NALTIYDLRKIAKRRTPASAFDYTDGAAEGEISIARARKAFEDVEFHPSILKDASEIDMSTSILGGPSSL

PFGIAPTGFTRLMQTEGEVAGAGAAGAAGIPFCLSTLGTTSIEDVKATNPTRNWFQLYVMKREISYGLVE

RAAQAGFDTLFFTVDTPVAGNRMRDVRHGFSIPPQLTVKTVVPLEFASLS--STGTVGELLNNAMDPTIS

FDDLKTIREMWPGKLAVKGVQNLEDSKKLADLGVDSIVLSNHGGRQLDRAPVPFLLLPEVAREVGKEIMV

DTGIMNGADIVAALALGADFTLIGRAYLYGLMAGGRAGVDRTIEILRSQIERTMKLLQVTSIEELGPQHV

QLT

>A6W7T3|A6W7T3_KINRD

SALTIEDLRAIAKRRTPKAAFDYTDGSAEGEISLARARQAFADVEFHPSILRDVSKVDTSTTIFGGPSSL

PFGIAPTGFTRLMQTEGETAGAGAAGAAGIPFTLSTLGTTSIEHVKAANPTRNWFQLYVMQRDISYGLVE

RAAAAGFDTLMFTVDTPIAGARLRDKRNGFSIPPQLTASTVLKLEFASLT--ETGTVGELLDYAMDPSID

YDDLAEIRALWPGKLVVKGVQNVADSRRLADLGVDGIVLSNHGGRQLDRAPIPFHLLPEVVREVGREIAI

DTGIMNGADVVASIAMGARFTLVGRAYLYGLMAGGRQGVDRAIAILADQVVRTMKLLEVASLEELTPAHV

QLE

>A4AF96|A4AF96_9ACTN

SALTIYDLKAIAKRRTPKAAFDYTDGSAEGELSLSRARQAFEDIEFHPSILRDASNVDTTTQILGGTSAM

PFGIAPTGFTRLMQTEGEIAGAGAAAAAGIPFTLSTLGTSSIEDVKAANPERNWFQLYVMDRDISYGLVE

RAAAAGFDTLMFTVDTPVAGARLRDKRNGFSIPPQLTVGTIMPLEFASLA--STGTVGELLDSAMDPSID

YHDLTIIRDMWPGKIVIKGVQNLEDSKRLADLGVDSILLSNHGGRQLDRAPIPFHLLPNVVREVGNEVMV

DTGIMNGADIVASMALGAKFTLIGRAYLYGLMAGGREGVDRTIEILSEQVIRTMKLLEVTSIEELEPKHV

QLQ

>C8XD73|C8XD73_NAKMY

KALTIYDLRKIAKRRTPAAAFDYTDGAAEAELSLTRARQAFEDVEFHPDILRPAPDVNTSTTILGDTSAL

PFGIAPTGFTRLMHTEGEIAGAGGAGAAGIPFTLSTLGTSSIEDVKAANPHRNWFQLYVMQREISYGLVE

RAARAGFDTLMFTVDTPVAGYRMRDKRNGFSIPPQLTPGTIITLEFASLS--STGTVGDLLNSAMDPTIS

YDDLKVIREMWPGKLLVKGVQNVPDAVRLIDQGVDGIILSNHGGRQLDRAPIPFHLLPQVVREVGRTVMV

DTGIMNGADIVASIALGAKFTLVGRAYLYGLMAGGREGVDKTIAILRSEIERTMALLGVSTLDELEPRHV

QLA

>A4QHX2|A4QHX2_CORGB

SALTIYDLRKIAKRRTPAAAFDYTDGAAEAELSIKRAREAFENIEFHPDILKPAEHVDTTTQILGGTSSM

PFGIAPTGFTRLMQTEGEIAGAGAAGAAGIPFTLSTLGTTSIEDVKATNPNRNWFQLYVMDREISYGLVE

RAAKAGFDTLMFTVDTPIAGYRIRDSRNGFSIPPQLTPSTVLTLEFASLS--STGTVGDLLNSAMDPTIS

YEDLKVIREMWPGKLVVKGVQNVADSVKLLDQGVDGLILSNHGGRQLDRAPVPFHLLPQVRKEVGSTIMI

DTGIMNGADIVAAVAMGADFTLIGRAYLYGLMAGGREGVDRTIAILRSEINRTMALLGVSSLEELEPRHV

QLA

>C8NJC6|C8NJC6_COREF

SALTIYDLRKIAKRRTPAAAFDYTDGAAEAELSIKRAREAFENIEFHPDILKPAENVDPSTQILGGHSAL

PFGIAPTGFTRLMQTEGEIAGAGAAGAAGIPFTLSTLGTTSIEDVKATNPQRNWFQLYVMDREISYGLVE

RAAAAGFDTLMFTVDTPIAGYRIRDTRNGFSIPPQLTPGTVLTLEFASLS--STGTVGDLLNSAMDPTIS

YEDLKVIREMWPGKLLVKGVQNVPDAVKLLEGGVDGLILSNHGGRQLDRAPVPFHLLPQVRREVGSTIMI

DTGIMNGADIVAAIALGADFTLIGRAYLYGLMAGGRQGVDRTIEILRTEITRTMALLGVSTLDELEPRHV

QLT

>A0QGJ2|A0QGJ2_MYCA1

SAHTIEDLRRIAKRRTPKAAFDYTDGAAEDELSIQRARQAFRDIEFHPTILRDVSTVTAGWDVLGGPVAL

PFGIAPTGFTRLMHTEGEIAGVRAAARAGIPFSLSTLGTCAIEDLAAAVPQRKWFQLYMWDRERSMALVR

RAADAGFDTLLATVDVPVSGARLRDNRNGMTIPPTLTLRTVLPLAFASLDR-WPGTVAEYLSTMFDPSLT

FDDLEWIKARWPGKLVVKGIQTLDDARAVVDRGADGIVLSNHGGRQLDRAPVPFHLLPTVARELGKEILL

DTGIMSGADIVAAIALGARCTLVGRAYLYGLMAGGEAGVTRAIEILAEGVIRTMRLLGVTCLEELSPRHV

QLR

>B5AEM8|B5AEM8_9MYCO

AALTIQDLRRIAKQRTPKAAFDYTDGAAEDELSIKRAQQAFRDIEFHPAILRDVTNVCAGWDVLGHPVML

PFGIAPTGFTRLMHTEGEIAGAQAAAAAGIPFSLSTLATSAIEDVVAAVPQRKWFQLYMWDRERSMALVE

RAANAGYDALLVTVDVPVSGARLRDTRNGMSIPPALTLQTVFPLAFASLDR-WSGTVSEYLNTMFDPSVT

FDDLAWIKAQWPGKFVVKGIQTLDDARAVVERGADGIVLSNHGGRQLDRAPVPFHLLPTVARELGKEILL

DTGIMSGADIVAAIALGARCTLVGRAYLYGLMAGGEAGVRRAIEILDNGVIRTMRLLGVTCLEELLPRHV

TQL

>B8ZSM2|B8ZSM2_MYCLB

AALTIQDLRCIAKQRTPKAAFDYTDGAAEDELSIKRAQQAFRDIEFHPAILRDVTNVCAGWDVLGHSVLL

PFGIAPTGFTRLMHTEGEIAGARAAAAAGIPFSLSTLATSAIEDVVAAVPQRKWFQLYMWDRDRSMALVE

RAADAGYDALLVTVDVPVAGARLRDTRNGMSIPPALTLRTVFPLAFASLDR-WS-TVSDYLNTMFDPSVT

FDDLAWIKTQWPGKFVVKGIQTLDDARAVVERGIDGVVLSNHGGRQLDRAPVPFHLLPTVAREFGKEILL

DTGIMSGADIVAAIALGARCTLVGRAYLYGLMAGGEAGVRRAIEILESGVIRTMQLLGVTCLEELSPRHV

QLQ

>A2VIY7|A2VIY7_MYCTU

AALTIQDLRRIAKRRTPRAAFDYADGGAEDELSIARARQGFRDIEFHPTILRDVTTVCAGWNVLGQPTVL

PFGIAPTGFTRLMHTEGEIAGARAAAAAGIPFSLSTLATCAIEDLVIAVPQRKWFQLYMWDRDRSMALVR

RVAAAGFDTMLVTVDVPVAGARLRDVRNGMSIPPALTLRTVLPLAFASLDR-WPGTVGEYLNTVFDPSLT

FDDLAWIKSQWPGKLVVKGIQTLDDARAVVDRGVDGIVLSNHGGRQLDRAPVPFHLLPHVARELGKEILV

DTGIMSGADIVAAIALGARCTLIGRAYLYGLMAGGEAGVNRAIEILQTGVIRTMRLLGVTCLEELSPRHV

QLR

>A0PSD6|A0PSD6_MYCUA

TALTIEDLRRVAKRRTPRAAFDYTDGAAEDELSIERARQAFRDIEFHPTILRDVTSVRTGWDVFGKPVAL

PFGIAPTGFTRLMHTEGEIAGARAAAEAGIPFSMSTLATCAIEDLQAAVPQRKWFQLYMWDRDRSMALVK

RAADAGFDTMLVTVDVPVAGARLRDVRNGMSIPPALTLRTVLPLAFASLDR-WSGTVGEYLNTMFDPSVT

FDDLEWMKAQWPGKLVVKGIQTLDDARAVVERGADGIVLSNHGGRQLDRAPAPFHLLPLVARELGKEIVV

DTGIMSGADIVAAIALGARCTLIGRAYLYGLMAGGEAGVKRAIEILSAGVSRTMRLLGVTCLEELSAKHV

QLQ

>A1SDE0|A1SDE0_NOCSJ

DALTIEDLRRAARRRTPRAAFDYTDGAADDEISLARARQAFRDVQFNPGVLRDVSSVDTSREVLGARASL

PFGIAPTGFTRLMHTEGEVAGATAAAAAGIPFALSTMGTTSIEDVAAAAPSRHWFQLYMWDRDRSMALVE

RAARAGFDALLVTVDVPVAGARLRDVRNGMTIPPTLTPRTVLPLAFATLDS-WSGTVAELLDTMFDPTVT

FEDLAWIKEQWPGRLVVKGIQTVDDARRVADLGADAVLLSNHGGRQLDRAPIPFRLLPEVVAAVGQEVHL

DTGIMSGQDIVAALAHGARFTLVGRAYLYGLMAGGRDGVDRAVEILRSQVERTMRLLGVRSLGDLEPGHV

QLD

>D3PXN3|D3PXN3_STANL

SAVTIYDLREAARRRTPKAAFDYTDGAAEAELSLARARQAFEDIEFNPTILRDVSSVDTGWEVLGERVAL

PFGIAPTGFTRLMQTEGEIAGATAAEAVGIPFALSTLATTSIEDVKAASPNRHWFQLYMWDRDRSMALVE

RAAAAGYDTLMVTVDTPVAGARLRDKRNGFSIPPQLTLKTMLPLSFASLDR-WPGTVAELLDTMFDPTVD

FDDLAWIKQQWPGKIVVKGVQNLADAKRLADLGVDGVVLSNHGGRQLDRAPVPFHLLPTVVREVGAEVHV

DTGIMSGADIVASVALGARFTLVGRAYLYGLMAGGRRGVDKTIEILSEQVVRTMRLLGVSSLEELEPAHV

QLR

>A3VA28|A3VA28_9RHOB

TVSNIDDLRSRARCALPRALFDFVDGAAGDETTARHNRADFDRYGFRPRVGRDVSTIDLSATMAGRPAAL

PIALSPIGFAGLCWPEGEVLAARAAARAGIPACLSTNSIASIEDVARAVPEENWFQLYFLDRDWMMGLVR

RAKDAGYRVLVLTLDLPVAGRRERDVRNAFTVP-IPRLATLARFRFGNFEG-NPVSIAKHVSSLFDPSAT

WEDVARVRAAWDGPMIVKGLLHPDDVEAARRIGVQGISVSNHGGRQLDGSLSAVAALPDMVATAGDEVLL

DSGVRRGTDILKARALGASGVLIGRAWAYGLAAAGEAGVDKAIELLRDEMTNAMMLLGEREIAALTPSHL

EIT

>A1BC61|A1BC61_PARDP

AV-NIDEIERAARRYLPGFLFDFIAGGVDGEAGLMRNRQAFARHRLVPRYLRDVSRRSLSTRILGRDYAM

PLGISPTGPAQLFRRDADRMLARAAREADIPFVLSGAAGASLEEITRIAPEHGWFQLYPADRAITRDQIR

RAADAGTPALVLTVDTPVTPKRERHLRNRISVPPALWPRLAKMEAMGNWAP-YAEIAAFFFAPGESGTLC

WQDLEACRALWPGPLIVKGILHPEDARRAASLGADAIMVSNHGGKALDAAPAALDMLPAIRHAVGPPLFL

DSGVRRGSDIVIALCLGADFVFAGRPTLYGTAAGAEAGARKALSILRQETDLVMAGIGCTSPAELGPDYL

MGD

>B2JRL0|B2JRL0_BURP8

GALSIGDLRKLAQRALPRVLFDYVEGGPDDEHGIVHNREVFNRWALVPRYMQDVSDRSTATSILETRHSA

PFGVSPTGFAGLLRPRADLMLARAANEAGLPFVLSGVSNATLESVAAEIGEALWFQLYPSDRQISDDMVR

RAGSAGVTHLVVTVDLPVTSNRERDARNGFGFPPALKPSGYLAPLFANWTE-YASDVARL-IKSNSPAMT

WSDVRRLRDSWPHKLIVKGILHPDDALNAQRHGVDAVIISNHGGRQLDRAIASINALPLIRREVGDPLMI

DGGVRRGSDIAIALCLGANFVFVGRPTLYGVAAAGEAGASRAIQILRTEFDRVMGQLGATRPEILDTSFL

ADS

>A6UKQ5|A6UKQ5_SINMW

QLLNARDFREAARRYLPRALFEYIDRGSEDECALARLRESLGAIELVPSILTAHQARDLRTEVLGKEHSL

PIIIAPTALAGLVSHDGEVKIARVAARQGIPVCISTQSVTTVEDVASGAPGDIWFQLYMWDRSRSKALLE

RVAAAGVTTLVITADTPASPKREYNDRNGFSIPIKYSLRAGLMPTYGHYPE-EFSVAEAV---RLENLLN

WEDIRQIRQWWKGKILIKGILSVTDALKAKAAGAEGIVVSSHGARNLDVAPPPARVLPQIADAVGREVLA

DSGVMRGSDVLKYVALGARSVMIGRLPLWGLAAGGEDGADLLLSMLRNEIDLTLCMLGLQKPADCALAVH

PAQ

>A1BBP3|A1BBP3_PARDP

PL-NTDDFRATARRRLPRGLFDYIDRGTEDEVSLARIRASLDGVRLRPRILNGDCPASLETTLLGRRHPT

PLVIAPTALAGMVADKGETKLARAASRFGIPFTVSTQSVEPVEDIRRGAPDELWFQLYVWDRARTAELLR

RVAACDCDTLVLTVDTQMPPKREYNQRNGFGVPFRPTPGNVAGLRRGMPS--YGAGLLSPVELRLDPALT

WQDFRALRDGWQGRIILKGVLGTEDAMRAKAEGADAIVVSTHGGRNFDALPTTAEALPRIAANPAAELLA

DSGVRRGSDVLKYLALGASAVQLGRAPLWGLAAGGETGAATLLEIILAEMRTGMGFLGARTLADLCP---

FDN

>A8I3U6|A8I3U6_AZOC5

LL-NVEDARRLARRRLPRGLFEYLDRGTEDEVSIAGNRAGLDAIRLAPFALEDVSQRAQDTVLFGTPQPC

PLVIAPTAVAGLMSYDGEVAMARAAKAHDIPFCVSTQSMTSIETIARDSGARLWFQLYVWNRARTFALLD

RAAGAGADTLVLTVDTAVSPKREYNQRNGFGIPLKPSVRAGILRTTGMPT--YATALGRAVEISLATDVS

WKDVAALRAHWKGRLILKGILRASDATRAIAHGVDGIVVSNHGARNLDCAPHPAHVLPAIVAAAGGTVLA

DSGVRRGSDIAKYLALGADGVLVGRAPLYGLAAAGTPGASRVIELLRAELDTTMALLGVTRLDQLPRTLQ

-ME

>A6VWC6|A6VWC6_MARMS

RVQSVAEMAALARYRLPDFAWEYLAGGAENEQTLSCNESDFAKIRLTSHTLVANYPPELTRSLVGSASAL

PMMIGPTGFNGMLWPQADVALAKAANVKKIPFCLSTVSNASMEQVREAAQEDFWFQLYGLNAQLNDDLLA

RAKAVGVSTLVITSDAFVVGNREWDRRN-FARPRQLTWHNKALPTMGNLNP-YLLGAMKFIQEQLDTLFN

WESVARIRDQWHGKLILKGVLHPDDAKQAVKLGFDGIVVSNHGGRQLDGALSSIDALPAIVKAVGGDILL

DSGIRRGSDVVKAAALGVQGVMLGRATLFGVAAGGQIGVSRVLDILQEELSRSLNLMGVQRLDELSPAHL

YLS

>B7XGW4|B7XGW4_PSEPU

RVHSVAELADMARRRLPYFAWEYLSGGAQDELTLTDNLDGFSAYGLHARAMVPCHPPATARAVLGRVLPV

PLLIGPTGYNGLLHRDADIHLARAATARGLPFCLSTAANTSLEALVAAVPENLWFQLYAMDPRVQNDLLR

RAAAVGSRTLLLTCDAMVLGNREWDRRN-FAKPRQLAWRNTLLPGMGNLEP-YLLGSMAFIGRQMDSLLD

WDKLARLRDQWGERLLLKGVLHPADVERAIALGLDGVVVSNHGGRQLDGAPSSLAALAAVAPQARGSLLL

DGGIRRGSDIVKALALGADAVLLGRATLYGVAVAGEAGAGRALDLLTQELVQTLNLMGCTHLSHLGRDNL

ERR

>C4ZN28|C4ZN28_THASP

RAHSIAELRAMAARRLPDFCLEYLDGGADDELALARNRRVLDEILLLPRTLVDVSQRELAVPLFGTEIAL

PAVIAPTGFNGLLTHAGDRVLAEAAHAAGIPFCQSMVSTVALEDI-AA-TGRHWMQIYPFDRDNLAAVVK

RAEHAGCEAIVLTTDASVFGNREWDRRN-YRAPMKLAWRKLLMPRFRNLGD-FLKNAAAFLAAQMDTSLT

WEDLRRLRDLWPRRLLVKGVLLPEDALRAQEAGADGVVVSNHGGRQLDCAPAPIETLAAVRQAVGATVIV

DSGFRRGSDFVKARALGADAAMSGRATLYGLAAAGAAGAARALEILRGEMERTLGLIGCARMDEVDARYV

GRG

>B2JMA7|B2JMA7_BURP8

RAHSIDDLRAMARRRLPNFCFEYIEGGAEDEATLRRNRDVFDEIAFLPRTLVNVEHRNQSRTLFGQRTAS

PFMIGPTGYSGLMFREGDVQLASAAAAAGIPFVLSNASTVALEEVVQRAGGRVWMQVYMYTREFVAKLAQ

RSLAAGIEALVVTTDSAVFGKREWDLRN-YIKPLMLDWRNKFMPHFANLGD-LLKGATITLGQQLDPSLS

WDDILWLRDLWPKRLVVKGVLGAPDAVRAIEAGVDGIVLSNHGGRQLDGAVSAMDVLPEVVDQVRGAVML

DGGFRRGSDILKAVALGADAVLLGRATTYGLSAGGRPGAARAIQILQTEVDRGLGLLGCSDIAALDRSYL

WQL

>B5WIU5|B5WIU5_9BURK

RAHSIDDLRAMARKRLPNFCFEYVEGGAEDEATLRRNRDVFGEIAFLPRTLVNVEHRNQSVTLFGQRSAA

PFMIGPTGYSGLMFREGDVKLASAAAAAGIPFVLSNVSTVALEDVVRRAGGRVWMQVYMYTREFLAKLAQ

RAKAAGIEALVVTTDSAVFGKREWDLRN-YIEPLKLDWRNKFMPRFANLGD-LLKGATITLGRELDPSLS

WDDIRWLRDLWPNRLIVKGVLGAPDALRALETGVDGIVLSNHGGRQLDSAVSAMDVLPEVVEQVGGCVML

DGGFRRGSEILKAVALGADAVLLGRATTYGLSAGGQPGAERAIEILKTEIDRTLGLLGCSDIAGLDRSYL

WLR

>D1RQ13|D1RQ13_SEROD

AILNVADLQRAARYYLPRFAYRYLAGGAEDEHTLRGNRVAFGQWQFVPPVLRDASRRTLNIRLWQQELAA

PLLIAPTGYNGMLRYQADLMLARSARAFGIPYIQSTVSTASLEEIAADGQGQHWFQLYVLDRQVTAGLLQ

RALAAGCNTLVLSVDAVHFGNRERD-RRSYRRPMKLSLASLCMPGFGNLQP-YLLSGAAYFAREMDAALN

WQTLDWVRQCWPGKLLVKGILHPQDARQALDAGADGIVLSNHGGRQLDGSVAPISLLPAVRAACGPTILI

DSGFRRGTDVVKALALGANAVLLGRPLLYGVALAGQAGATQALRIFSEEIDRTLAQLGCSSVQELGPHLL

PVN

>A1K8D2|A1K8D2_AZOSB

--MDIDDLRRQARRFLPRFVFDFLEGGAGDETCLQENLAALRNIRLWPSVLRDTSGIDTSIEVFGERWRL

PFAVAPTGFNGLFRPDGDILIARAAARAGVPFSLSTASNTRLEEVARQADGLRWLQLYVMDRSIAEQIMR

RGWDAGYRVLVLTVDVPVNGYRKRDIRNGFRLPFRPGLMTALFPNFANLSE-HPQVQAALLNRTMDRTLA

WESLSWVRAHWKGPVVVKGVLHPDDAARAVAEGADGIVVSNHGGRQLKSAPATIEALPLVVERVDGPVFV

DGGFRSGEDVAKALGRGAKAVFLGRPVLYGLAAAGEAGVERVFDWLREDLERTMILMGRRRIDELAT---

GWR

>C5CVM3|C5CVM3_VARPS

LL-NVEDHRRRARRMLPRLVFDYVDGGAEDERCLQRNRDALEQLPLIPECLRDTSTVDIGIELFGRRWRA

PFAVAPIGLAGLVRPGADALLARAAQGAGVPFVLSTASNTRIEDVRAAAPDALWMQLYVMERAIAERIVR

RARAAGFEALVLTVDVPVSGLRERDLRHGFRLPMRLTPATVLMPQFANLLP-DDQAQAALLSRTMDRRLT

WESLAWLRKLWDGPLLVKGLLGAEDARRAVRHGADGIVVSNHGGRQLDAAPASIAVLPAMVDAAGGPVLM

DGGIRRGSDVVKALALGARGVLAGRAPLYGLACGGEQGALSVLQLLAQEIERTMTLLGATRAAELGLRHV

RPE

>A1VL09|A1VL09_POLNA

LL-NAEDFRCQARRALPRFVFDYIDGAADDGACRQRNQADFAALTLLPRVLRDTSQIDTTVTVFGSPWSV

PFGVAPTGLNGLIRPGGDALLAAAAARAGIPFALSTASNMPLEAVRAAAPELQWMQLYVMHREMAERIVQ

RARRAGYQALVLTVDVPVSGNRELDLRNGFRMPFKPTPQIAWTPDFANLTV-AGSLQAALMARAMDRSLV

WETLGWLRKSWPGPLLLKGLLHPEDARLAVEHGIDGLIVSNHGGRQLDAAPSAIHALPAVVASVQGPVFM

DSGVRRGSDIAKAIALGAKAVFLGRPLLYGLAAQGAAGIDAVMKQFSDELVRTMILLGASRIADL--SAA

---

>A3VMI7|A3VMI7_9RHOB

AI-NIEDLREQARRRLPRVVFDYLDGGAEAEVTLRRNRSSFTDIVLTPRILKGGS-VDLTLELFGETYSK

PFFIGPTGLNGLYWPQGDLHLAAAAERSGVGFTVSTASNTTLEEIAGKSKGPLWFQLYPWQGAFAEALID

RAQASGYSALVLTVDSLVGGKRERDLRHGFAEI-RIGPRTVLRPRLENLLD-FVSELAEFTRSQRNPEFS

WDDVRRIREKWKGPLLIKGIMCPEDAIDAQRAGVDGVIVSNHGGRQLDGAPATIDVLADIIAALDRPVLL

DGGIRRGSDIVKALTLGAKGVLLGRAPLYGLAAQGEAGVSRALSILEDEMTRTMTFVGARSVSAVSGVNV

RRS

>C5A8L6|C5A8L6_BURGB

PI-NVDDFRMLARRRLPRRVFDYLDGGAEDERGLRRNRAAFERLAFVPRRLADVGTRELSTTLLGTRLAA

PFVIAPTGLNGLIHPDGDLALARAARRAGIPFAMSTASNVSLERLAGEAGGELWFQLYVMHRELADSLVQ

RAARAGYRTLVVTVDVPLNGKRERDLRNGFALPLRCTPGVLLLPTLANLGA---EAKTALLRRQMDASFG

WDDLRRLRERWPHRLLVKGILHTGDAVACLEAGADGLILSNHGARQLDDAVAPLDVLSAARQACGAALLV

DSGVRRGSDVVKALALGANAVMLGRATLYGLAAAGEAGVTRVLEILRDEVDRTLAMLGCRGLAELSASHL

PAA

>B9JQ29|B9JQ29_AGRRK

SI-KVEDYRHLARRRLPKMVFDYLDGGAEDEYGLRHNRDVFLDWHFKPSRLIDVSRRDLTVELFGQRYPL

PFMIGPTGLNGIFRPNGDLLLAQAAARLGIPFVLSTASNLTIEEVASNCDGELWFQLYVVHRDLANSLTD

RALAAGYKTLVLTTDVTVNGYRERDMRSGFGLPLRYTPKVILVPQLANFR----QAQAALMSRQMDASFS

WEDLARLRDRWPHRLLVKGILRSEDAQKCVELGADGVILSNHGGRQVDSCLSPMEVLSQTARLVTKPILI

DSGFRRGGEIVKALALGAKIVLLGRATLYGLAARGEPGIDDVLSILRTEIDRTLALIGCNSVAQLSSDFL

---

>1HUV|PDBID_CHAIN

NLFNVEDYRKLAQKRLPKMVYDYLEGGAEDEYGVKHNRDVFQQWRFKPKRLVDVSRRSLQAEVLGKRQSM

PLLIGPTGLNGALWPKGDLALARAATKAGIPFVLSTASNMSIEDLARQCDGDLWFQLYVIHREIAQGMVL

KALHTGYTTLVLTTDVAVNGYRERDLHNRFKIPPFLTLKNFEGIDLGKMDKANLEMQAALMSRQMDASFN

WEALRWLRDLWPHKLLVKGLLSAEDADRCIAEGADGVILSNHGGRQLDCAISPMEVLAQSVAKTGKPVLI

DSGFRRGSDIVKALALGAEAVLLGRATLYGLAARGETGVDEVLTLLKADIDRTLAQIGCPDITSLSPDYL

QNE
