# Supplementary material for: Accurate Simulation and Detection of Coevolution Signals in Multiple Sequence Alignments
Source: PLoS One. 2012 Oct 16;7(10):e47108. doi: 10.1371/journal.pone.0047108 (PMC3473043; doi:10.1371/journal.pone.0047108)
Supplement: MSA S7 — MSA of ATP11. (DOCX) [file pone.0047108.s018.docx]

>A4HML6|A4HML6_LEIBR

KRPGRKSLDEICKLSLFEVSTPQQVATTWNNHHMQFLQYWGRTIS-SEAYYALEPRLRMSPYFVVPVFR-

-----------DKGLFNVVTNF-----KDDLVGVAPLGEWQKKQDNAQIHMTIQFFTELARSKRLVLVRC

EI-KDEVFKRLDCIFVTQMLLKYYT--------FPRLYEWVETFNKRPGQFDYHAFLRAMKDIKI

>E9B673|E9B673_LEIMU

KRPGRKSLDEVCKQSLFEMSTPQQVATIWNHHHMQFLQYWGRTIS-SEAYYALEPRLKASPYFVVPVFR-

-----------DKGLFNVVTNF-----KDDLVGVTPLAEWQKKQDDAQIHMTIQFFTELARSKQLVLVRC

EI-KDEVFKRLDCIFITQMLLKYYT--------FPQLYEWVETFNKRPGQFDYHAFLRAMKDIRI

>G0U3S1|G0U3S1_TRYVI

KIPGRKCLDEICKLNLLEATQAARVAVIWNEHHKQFMQYWGRAIS-AEAYNALQPRLRQCPYFVIPVFR-

-----------TKGLFNVVTNW-----DRDLIATAPLAEWQQKQDHAQPHMTIQFFTELARTKNLVLVRC

EI-KDNVFVRQDCVFITQMLLRYYS--------IPRLYEWVEVFNKHPNRFDYHMFLKNMKDIRI

>Q4CTG5|Q4CTG5_TRYCC

KLPGRKSLDEICKLSLLEATPTAQVATIWNEHHKQFVQYWGRVIS-ADAYDALRPRLQNCSYFVIPVFR-

-----------TKGLFNVVTNW-----DKDLVGVAPLAEWQKKQDHAQIDMTIQFFTEFSRTKRIVLVRC

EI-KDKIFVRQDCVFITQMLLKYYT--------LPHLYEWVEVFNKRPHMFDYHMYLRHMKEIHI

>C9ZZL8|C9ZZL8_TRYB9

KVPGRKSLGEICKLSLLEATPEPQLATIWNEHHKQFVQYWGRTIS-ADAYYALQPRLKQSPYFVIPVFR-

-----------TKGLFNVVTNW-----DRDLVGVAPLAEWQEKQDSAQVHMTIQFFTELARTKRMVMVRC

EI-KDKVFVRQDCVFVTQMLLKYYS--------DPQLYEWVEVFNKRPTQFDYHMYLRNMKDIRI

>G0UV13|G0UV13_TRYCI

KTPGRKSLDEICKLSLLEATPALRVAEVWNEHHKQFVQYWGRTIS-AEAYYALQPRLKQSPYFVVPVFR-

-----------SKGLFNVVTNW-----DRDLIGVAPLAEWQQQQGNTQVHMTIQFFTELARTKRVVLVRC

EI-KDEVFVRQDCVFVTQILLRYYT--------IPQLYEWVEAFNKRPNQFDYHMYLRNMKDIQI

>E9CJ89|E9CJ89_CAPO3

LPSYMQPLSSLIPADVIANGSRSDIIAAWNAAHLGKPDHFASVMP-AEAYRRLTSRSRRNSRFVLPVPR-

-----------GEGFEWMVAEF-----TNASLLFTSLLDYQTHQEHASSLLSVTFFGVL-EKHGIALQRA

YA-DHKKLPRELAESLLRNIAHVYLSNIALARRGQGAYGFVETFNQQPKQFDFNILLDELKARGL

>E6ZMM3|E6ZMM3_SPORE

QEGPIKPLSKILDVEKLQNQDAETITKLWAGYHMI-KNKLSAVIP-TEKYLEMLANARKYPQFVLPLPRQ

VIDEESDAAGTKDAFEMQFLEWAVV--PSATTIFTPLAEYKLKQDFSQPVLILTFYTDLCHSNGIVLMRG

EVTGGR-IDQAQAQLLALTLQRFYLSGSAAAQDGDDDCTLLHDFHKRPTEFDVEHLVNAFRL---

>Q4P7P6|Q4P7P6_USTMA

QESPIKPLSKILDVEKLMTQDVETITKLWAGYHTI-KNKLSAVIP-TERYLVMLANARRYPQFVLPLPRQ

VIDEDSEVSGAKEAFEMQFLEWAVIPSPSSTTLFTPLAEYKLKQDFSQPVLILTFYTDLCQSNGLVLMRG

EVTGGR-IDQAQAQLLALTLQRFYLSSSTPPTDAEGDCALLHDFHKKPEEFDVEELVNAFRL---

>B6K823|B6K823_SCHJY

STTNLKKLHDFVDLDKVETLTTEQATELWKEFCLRRPCTVGAVLP-ASMYKRMLETAKRYPVFVLPIPRG

-----------QQGLETHILQWLFP--NTAHLVLTTLLEYKLRREYAVPHTTVLHFPELSETKKIVLTLS

EFDPNKSISALDVQILLHGVQKFFT--STDTSITGRRRLMLRSFNDG-CQYDLKQVAEDMDMLE-

>G1X7F3|G1X7F3_ARTOA

KSSNQKSLDSIIDTSKILLHDAKEIEYIWRARHISDENSLCAIVP-LETYRRIEKAAKRHPMFVLPLPRP

-----------DQGIELHFLQWQFS--TTVNVMFTSLIEYKLRGEFASPHTTVTHHLDISEEKGIVLLQG

SVVENKGVSVEEAKWLLMALQKFYG-AEEGVPGAGEERRLLRLFSEGKQEFDVQMLIKEVETIN-

>D5GMY5|D5GMY5_TUBMM

PPPGVKTLSSFVDVDRLTAHSPKEIEFIWRARFLDDKNSLCAVIP-KDTYASLEESACQYPMFILPLPR-

-----------DNGAEMHFLQWTFPTPRTSTIIFASLAEYKLRGEFAVPHTTFSHHLELAEDKGIVLAQG

AVTPDRGVTANDARWLVMALQRFYG--ALDSEESGVRRKLLEMFSKGDDGFKVESLIEEVEKWG-

>F0XH61|F0XH61_GROCL

SSRGARPLSPFLDLEKSRSLGADELTAVWRLRLAADPSSLCAVIP-AATYAAMEAAARARPQFVLPVPHP

-----------EQGAEVHFLQWTFDEQRSSTVLFTQLAEYKLRGEFAQPHTTVTHHTDLATDCGVVLMQG

QVMTDRGASVDSARWLVMCLQRFYGTETAAATLDREGRRLLQWFAAGDPQFSIERLMEEVEKLG-

>G4MS73|G4MS73_MAGO7

SGSQVKPLSAIVDLPKVRQLPVPELTEIWRLLHTTDPKSLCAVVP-APTYKAMEALARQRPQFVLPVPHP

-----------EQGAEMHFLQWTFD--GTSTVLFTQLAEYKNRGEFAQPHTTVTHHPDLADDKGVVLMQG

SMVDNRGVKIADAQWLVMCLQRFYGSAGGSRLQSEERRRLVEWFGTGNENFSIEKLLAEAERLG-

>C9SM31|C9SM31_VERA1

SASGVKPLGDILDLEKAAALPEKELTAIWRLRHAHTPNTLCAVIP-SPTYTAIEALARRAPQFVLPVPRP

-----------DQGAEMHFLQWVFDAARTATVMFTQLAEFKARGEFAQPHTTVTHYTDL-REAGLVLMQG

QVVDGRGASVEDAPLAASVPAALLR---AAGTSRPRAIRGSRGLSRGGCSSGSPRRIRSASRRGW

>G2WYM3|G2WYM3_VERDV

SASGVKPLSDMLDLEKAAALPAKELTAIWRLRHAHTPNTLCAVIP-SPTYAAIEALARRAPQFVLPVPRP

-----------GQGAEMHFLQWVFDAARTATVLFTQLAEFKARGEFAQPHTTVTHYTDL-REAGLVLMQG

QVVDGRGADVEDARWLLLCLQRFYGKGGEPDPQRLERRRLLEWFAAADPRFSVEKLLEEAERMG-

>F7W1V0|F7W1V0_SORMK

SGSGIKPLSEILDMTKARDLPIKELSAIWRLRYATNPHSLCAVIP-SETYSAMDTLARSRPQFVLPVPHP

-----------DQGAEIHFLQWTWD--ATSTVLFTQLAEYKNRGEFAQPHTTVTHYMDFSADKGVVLMQG

QVMEDRGVTPENAQWLLMCLQRFYGWDGTGGEQGQERRRLLDWFAMGDQRFSVEKLLEEAERMG-

>G4U673|G4U673_NEUT9

SGSGIKPLSEILDMTKARDLPIKELSAIWRLRYATNPHSLCAVIP-SETYSAMDTLARSRPQFVLPVPHP

-----------EQGAEIHFLQWTWD--ATSTVLFTQLAEYKNRGEFAQPHTTVTHYMDFSVDKGVVLMQG

QVMGDRGVKPEDAQWLLMCLQRFYGWDGTGGEQGQQRRRLLDWFASGDQRFSVEKLMEEAERMG-

>G0SBM6|G0SBM6_CHATD

SPTGVKPLSAILDLPKASALPTEELSTIWRVHHQAKPNSLCAVIP-ARTFEAMETLAKSYPQFVLPVPHP

D----------QGGAEMHFLQWTWDAARSATVLFTQLAEYKARGEFAVPHTTVTHYKDLAQEKGVVLMQG

MVMDDRGVKVDDARWLVMCLQRFYGDGVQMSKEARERRRLLEWFGKGDTRFSVEKLMEEAEKMG-

>Q2HDY8|Q2HDY8_CHAGB

PNTGIKPLSSILDLPKASLLPATELTAIWRLRHASQPNSLCAAIP-TPTFTAMNTLARTYPQFVLPVPHP

-----------SQGAELHFLQWTWD--RSVTVLFTQLAEYKARGEFAAPHTTVTHYLDLAEGEGVVLMQG

TVMEDRGVKVDDARWLVMCLQRFYGWDGAGGEAGKVRRRLLEWFGKGDARFSVEALLEEAERMG-

>G2Q2Q9|G2Q2Q9_THIHA

SPTGLKPLSEILDLPKASALPQAELTAIWRLRHAARPNSLCAVIP-AATFAAMEAMARTHPQFVLPVPHP

-----------EQGAEMHFLQWTWD--RSATVLFTQLAEYKARGEFAAPHTTVTHYKDLAAQPGVVLMQG

TVLEDRGVKVADAQWLVMCLQRFYGWDGSGGEAGKERRRLLEWFGKGDTRFSVEKLMEEAERMG-

>G2RF67|G2RF67_THITE

SPTGIKPLSEILDLQKAAALPLEELTAIWRLRHAANPNSLCAVIP-AATFATMNALARAHPQFVLPVPHP

Q----------QGGAEMHFLQWTWDAPRSATVLFTRLAEYKARGEFAAPHTTATHYDELAGKGGVVLMQG

AVVADRGVKVEEARWLVMCLQRFYG--GWDGVESRERRRLLEWFGKGDARFSVEKLMEEAERMG-

>E3QEQ9|E3QEQ9_COLGM

SGNGIKPLDEILDLPKVRDLPDKELTAIWRLRHASDPTSLCAVIP-TPAYRVMEALAKQNPQFILPVPHE

-----------EQGAEIHFLQWVFD--RTATVMFTQLAEFKTRGEFAQPHTTITHHTDLADDRGVVLMHG

KCLEDRGVKTAHAQWLVMCLQRFYG-----DAEGKERRQLLEWFRTGDERFSVDKLMEEAERIG-

>G3JMG6|G3JMG6_CORMM

DRPAVKTLDEILDLDKVRALPEKEITALWRLRHAASPAQLCAVIP-AATYAAMEDVARAAPQFVLPVPHP

-----------TQGAEMHFLQWVFD--RTSTVLFTQLAEYKTRGEFAQPHTTVTHHLDLAADNGLVLMQG

QLVEDRGVRPEHAKWLAMCLQRFYGAGVELDGERKERRKLLEWFAAGDARFTVEKLLEEAERMG-

>C7YYJ5|C7YYJ5_NECH7

GKPAVKTLNNIIDLPKARELPDKELTAIWRLRHASNENAICAVIP-TSTYKIMEEAAQNAPQFVLPVPHP

-----------TQGAEIHFLQWTFD--KTSTVLFTQLAEYKNRGEFAQPHTTITHHLDLADDRGLVLMQG

QVVEDRGIKPENARWLVMCLQRFYGENAELNGERKERRKLLDWFAAGDSRFSVEKLMEEAERIG-

>F9G523|F9G523_FUSOF

DKPAIKTLNDIIDLPKARELPEKELTAIWRLRHASSEQNLCAVIP-MSTYKAMEDAARTAPQFVLPVPHP

-----------AQGAEIHFLQWTFD--KTSTVLFTQLAEYKNRGEFAQPHTTITHHLDLADERGLVLMQG

QVLPDRGVTPENAKWLLMSLQRFYGEEVELTGERKDRRKLLNWFAAGDSRFSVDKLLEEAERMG-

>E9EHH2|E9EHH2_METAQ

GKAAIQSLDEIVDVEKIRELPEKELTAVWRLRHANSPQKLCAVIP-TASYKAMEEMARKSPQFVLPVPHE

-----------SQGAEIHFLQWTFD--NTSTVLFTQLAEFKTRGEFAQPHTTVTHHLDLADDKGLVLMQG

QTMEDRGVQPEHAKWLVMCLQRFYGTEAELDGSRKERRKLVEWFGSGDSRFSVEKLLEEAERIG-

>E9ETP3|E9ETP3_METAR

GKAAVQSLDEIVDVEKIRELPEKELTAVWRLRHANSPQKLCAVIP-TSSYKAMEEMARKSPQFVLPVPHE

-----------SQGAEIHFLQWTFDAANTSTVLFTQLAEFKTRGEFAQPHTTVTHHLDLAEDKGLVLMQG

QTMEDRGVQPEHAKWLVMCLQRFYGTEAELDGSRKERRKLVEWFGSGDSRFSVEKLLEEAERIG-

>G9NNF0|G9NNF0_TRIAT

EKPAIKPLDDILDLEKVAELPEKELTAIWRLRHASSPQTLCAVIP-APAYQAMDSLARSNPYFVLPVPHE

-----------SQGAEMHFMQWTFD--KTSTVLFTQLAEYKARGEFAQPHTTVTHHLDLIKDKGLVLMQG

QVMEGRNVQPDHAKWLVMCLQRFYGKGDELDGQRKERRQLLEWFTNGDPRFSIEKLLEEAERMG-

>G0RQC9|G0RQC9_HYPJQ

TPAAIKPLGDILDLEKVAELPEKELTAIWRLRHASSPQTLCAVIP-AATYKAMEDLARSSPFFVLPVPHE

-----------SQGAEMHFLQWTWD--KTSTVLFTQLVEYKTRGEFAQPHTTVTHHLDLIEDKGLVLMQG

QVMEGRGVQPDHAKWLVMCLQRFYGKGDEIDGQRKERRKLLQWFTNGDSRFSVEKLLEEAERMG-

>G9N7T8|G9N7T8_HYPVG

EKPAIKPLGDILDLEKVADLPEKELTAIWRLRHASSPQTLCAVIP-APTYEAMEDLARSSPFFILPVPHE

-----------DQGAEIHLLQWTFDSAKTSTVLFTQLVEYKTRGEFAQPHTTVTHHLDLIKDKGLVLMQG

QVMEGRGVQPDHARWLVMCLQRFYGKGDELDGHRKERRKLLEWFTKGDPRFSVEKLLEEAERLG-

>F9XFS7|F9XFS7_MYCGM

SAPPVRTLSSFIDVEKSSSLPQKELETIWRLRHVRDPQSLAAVLP-SSTYNRIATTAKQHPQFVLPIPHP

-----------DRGAEIHFLQWTFP--TTATVLFTHLAEFKLRGEFAQPHTTVTYHVDFAEEKGLVLMEG

RVQEGKGVSVEEGRFLVMGLQKFYG---WEGKGEGKRRRLMEQFSGGDEGFRVEELLEETEKVYK

>A8U3R3|A8U3R3_ERYGR

SKTGIRALSSYIDIEKARKLPLKELESIWRLRHAHNAQSLCGVIP-LSLYNTIQNTAKKNPQFILPVPRE

-----------GKGAEIHFMQWTFP--NISTVIFTQLCEYKLKGEFSQPHTTITHHLEMSEEKGVVLLRG

EIVSGRGVSVDEAKWLLLCLQRFYG--GLGGAEVATKRKLVEMFGRGDGEFRVEDLVEEAEKLI-

>E4ZFS6|E4ZFS6_LEPMJ

PPPGIKTLDSFLNLSKIRPLPAQEIQTLWRLRHASNKQSVHFAVP-AQTFASMMRTAKQHPTFVLPMPRE

IPKEEEGGGEGQQAAELHYMQFAHP--DTTTLMFTTLAEFKLRGEFATPHTTVTFHQELAESHALVLGQG

IVMEGRGVSVDDARWLIMCMQKFYV----QSEEGKARQELLDLFTRGDSAFRVETLIDEAEKIL-

>Q0UY07|Q0UY07_PHANO

PPPGVKTLNSFLDLEKVRDLPLKEVQALWRLRHAGNPQSIHFAVP-ANVFRSLLQTAKQHPAFVLPVPRE

IPVEGAEASPEQQAAELHYLQFAHP--DTTTLLFTSLAEFKLRGEFASPHTTVTFHQELADSHDVVLGQG

LVIENRGVSLDEARWLVMCMQKFYV----QSEEGKGRGELLNMFTRGDSGFQVERLIDEAEKIL-

>B2VZ95|B2VZ95_PYRTR

PPPGVKTLNSFLDLDKVRTLPEKEVQALWRLRHAGNPQSIHFAVP-ANTFSQLLRSAKQHPSFVLPVPRQ

IPVDSPSADGQQQAAELHYLQFSHP--DTTTLMFTTLAEFKLRGEFASPHTTITFHQELTESHNLVLGQG

LVIENRGVSLDEARWLVMCMQKFYV----QTAEGKGRSELLDMFTRGDSGFQVDRLIDEAEKIL-

>E3RU01|E3RU01_PYRTT

PPPGVKTLNSFLDLDKVQTLPEKEVQALWRLRHAGNPQGIHFAVP-ANTFSQLLRSAKQHPSFVLPVPRQ

IPVEDSAAGGQQQAAELHYLQFSHP--DTTTLMFTTLAEFKLRGEFASPHTTITFHQELAESHNLVLGQG

LVIENRGVSVDEARWLIMCMQKFYV----QTAEGKGRSELLDMFTRGDSAFQVDRLIEEAEKVL-

>A7F5C5|A7F5C5_SCLS1

SNPGLKTLSSYLDLEIVRTLPQKEIEALWRLRHVEDAQSLCAAIP-LHTYKTIEATAKKFPTFVLPLAKE

-----------GQGAEIHFLQWTFP--NTVVVLFTHLAEYKLRGEYSQPHTTITHHLELEKDKELVLLHG

QVVEGRGVSVDDAKWLIMCLQKFYG----PVGEQSDRRRLLEMFGKGDPAFKVEDLVEEAEKII-

>A6RX21|A6RX21_BOTFB

STPGLKTLSSYLDLEKVRTLSQEKIEALWRFRHASEARSLCAVIP-LDTYKTIEKTAKAFPHFILPLPKE

-----------GQGAEIHFLQWTFPAENTVAVLFTHLAEYKLRGEYSQPHTTITHHLELEKDKGLVLLQG

QVVEGRGVSVDEAKWLVMCLQKFYG----PVGEKSDRRRLLEMFGRGDPAFKLEDLVEEAEKII-

>B6QEY9|B6QEY9_PENMQ

NTPGIKPLSSYLNVEKILTLPPKEIESLWRLRHANNARSICAVIP-LETYQRMAAAARSNPQFILPLPRQ

GDNAQSEDSAAEGGADIHFLQWGFH--HTSTIVFTHLAAYKLHGSYAQPHTTITHHLDLADEKGIVLMHG

QVMPDSGVSVSEASWLVSCVQRFYD---FDGEGSGRKGELLRMFTRGDEGFKVEDLVSEAETLS-

>B8MBC4|B8MBC4_TALSN

NTPGIKPLSSYLNLEKILTLPPKEIETLWRLRHANNARSICAVIP-LETYQRMATAARSNPQFILPLPRQ

SGSTQSEESAAEGGADIHFLQWGFHPPHTSTVVFTHLAAYKLHGSYAQPHTTITHHLDLADEKGLVLMHG

QVMPDSGVSVSEASWLVSCVQRFYD---FDGEGSGRKGELLRMFTRGDEGFKIEDLVSETETLS-

>B6HMT0|B6HMT0_PENCW

ESSPVKPLSSYLDVEKIRELPHKEIEALWRLRFAENPHAITAAIP-LDTYKRIMQAARENPQFILPLPRP

QTAEEAQQAPEGTVADIHFLQWAFH--HTSTVIFTNLGVYKMHGAYAQPHTTITHHLDLADDKGLVLMHG

QIIPDGGVSAMEATWLVSCVQRFYD---FEGQAAGRKSELVRMFTRGDENFKVEELVDEAERLQ-

>B8MW54|B8MW54_ASPFN

SSTGIKPLSSFIDVEKVLDLPPKEIEAIWRLRHASNPHSVCAVIP-VETYQRIASAARQNPQFVLPLPRT

QPEADQPEQQGGAGAEIHFLQWAFH--HTSTVIFTQLAAYQLHGSYAQPHTTITHHLDLADEKGLVLMHG

QIMPNSGITSTDATWLVSCVQRFYD---FGGQASGRKGELLRSFTKGDNVFKLEALLEEAEKL--

>G7XUE8|G7XUE8_ASPKW

TSTGIRPLDSFIDVEKVRSLPPKEIEAIWRLRFANNSHSVCAVIP-VETYQRIVSAARQNPQFVLPLPRT

QSEAQTAEENAKGGADIHFLQWGFH--HTSTIIFTALASYQLHGSYAQPHTTVTHYLDLADEKGLVLMNG

QVMPDSGVSATDATWLVSCVQRFYD---FGGQANGRKGELLQAFTRGDQNFKVEDLMAEAEKL--

>G3XXV6|G3XXV6_ASPNA

TSTGIRPLDSFIDVEKVRSLPPKEIEAIWRLRFANNSHSVCAAIP-VETYQRIVSAARKNPQFVLPLPPA

-----------KGGADIHFLQWGFHPPHTSTIIFTALASYQLHGSYAQPHTTITHYLDLADEKGLVLMNG

QVMPDSGVSATDATWLVSCVQRFYD----FGGQANGRKGLLQAFTRGDQNFKVEDLMAEAEKL--

>Q5B6S2|Q5B6S2_EMENI

SSSGIKPLGSYLDVEKVRALPPKEIEAIWRLRHASNSNSICAVIP-LETYQRIASAARQNPQFILPLPRS

ASESQSSEAETKGGADIHFLQWAFH--HTSTVIFTPLAAYKLHGAYAQPHTTITHHLDLADDKGLVLMHG

QVMPDSGVSTAEATWLVSCVQRFYD---FGGQASGRKGELLRMFTQGDQNFRIEELMAEAEKL--

>A1CDF1|A1CDF1_ASPCL

SSPGIKPLDSYLDVAKVRALPPKEIEALWRLRHANNPNSICASIP-LDIYQRIAAAARQNPQFILPLPRA

HTEQAPAADDAATGADIHFLQWAFH--HTSTVIFTQLAAYKLHGAFAQPHTTITHHLDLADEKGLVLMHG

QVMPDSGISTTEASWLVSCLQRFYD---FGVQASGRKGELVRMFTRGDEGFKMEELMEEAEKL--

>A1DCC9|A1DCC9_NEOFI

SSTGIKPLSSYLDVEKVLALPQKEIEALWRLRHANNPNSICACIP-LETYQRIASAARHNPQFILPLPRS

STEQAPAEDGSAVGADIHFLQWAFH--HTSTVIFTQLAAYKLHGAFAQPHTTITHHLDLADEKGLVLMHG

QVMPDSGISTTEASWLVSCLQRFYD---FEGQASGRKGELVRMFTRGDEGFKMEELMEEAERL--

>C5FYJ2|C5FYJ2_ARTOC

SAPGIKPLSSYLDLEKTSALPADTIGKLWRARHVTNPNSICASIP-IETYNRMVKVARQHPQFILPLPRE

LEIPADPKAEQSMGAEMHFLQWGFHPPHTSTVIFTSLAEYKLHAGFAPPHTVITHHLDFADDKGIVLMNG

TVVTDKGMTVDDAQLLVLWLQKFYDWEAEGAGSQGGRKGMLRMFTSGDEGFKVQELIDEVQRV--

>E4UVK7|E4UVK7_ARTGP

SAPGIKPLSSYLDLEKTAALPPDMIGKLWRARHVTNPNSICASIP-IETYNRMASVARQNPQFILPLPRE

LETPSDPNAESAIGAEMHFLQWGFH--HTSTVIFTSLAEYKLHAGFAPPHTVITHHLDFADDKGIVLMNG

TVVTDRGMTVDDAQLLVLWLQKFYDEAEGAGSQGGRKGEMLRMFTSGDEGFKVQELIDEVQRV--

>F2SG95|F2SG95_TRIRC

SPPGIKPLSSYLDLEKTAALPPDIIGKLWRARHVTNPNSICASIP-IETYNRMVKVARQHPQFILPLPRE

LETPQDPKESAAMGAEMHFLQWGFHPPHTSTVIFTSLAEYKLHAGFAPPHTVITHHLDFADDKGIVLMNG

TVVTDRGMTVDDAQLLVLWLQKFYDWEAEGAGSQGGRKGMLRMFTSGDEGFKVQELIDEVQRV--

>D4B1T0|D4B1T0_ARTBC

SPPGIKPLSSYLDLEKTAALPPDTIGKLWRARHVTNPNSICASIP-IDTYNRMVKVARQHPQFVLPLPRE

LETPQDPKENAAIAAEMHFLQWGFHPPHTSTVIFTSLAEYKLHAGFAPPHTVITHHLDFADDKGIVLMNG

TVVTDRGVTVDDAQLLVLWLQKFYDWEAEGAGSQGGRKGMLRMFTSGDEGFKVQELIDEVQRV--

>C4JZW6|C4JZW6_UNCRE

SAPGIKPLSAYLDIPKILTLPQKEIETLWRLRHAPNPNSVCATIP-LETYNRLVSTARQNPQFILPLPRE

IETPASDGERSSIAAEIHFLQWGFH--HTSTVIFTHLGAYKLHGAYAQPHTIITHHLDLADEKGLVLMNG

TVVPDKGVSLDEAKLLIMWLQRFYD-WGVDGSEGGKKGEMLRMFSRGDAGFKVEDLVEEVERI--

>C5PBE0|C5PBE0_COCP7

ATPGIKPLSAYLDIQKILTLPQKEIETLWRLRHAPNPNSVCATIP-LETYHRIIKTAKQNPQFILPLPRE

IETPAEDANSSATAAEMHFLQWGFH--HTSTVIFTHLASYKLHGAYSQPHTIITHHLDLADEKGLVLMNG

TVVPDRGVSLDEAKLLIMWLQRFYD-WGVDGSQGGRKGEMLRMFSRGDEGFKVEDLVEEVERV--

>C1GW48|C1GW48_PARBA

QMPGIKPLSSYINIPKILTLPAKEIETVWRLRHAANPQSVCATIP-LDTYMRMVGIARQNPQFVLPLPRK

AAGSGPDDVEGGTGADMHFLQWAFH--HTSTVLFTHLASYKLHGAYSQPHTIITHYLDLAVTKGLVLMSG

AVVSDRGVTMDEAKLLIMWLQRFYD-WGVDGAQAGKRAELVKMFNQGDNGFKLEDLMDEIERV--

>C0SJL8|C0SJL8_PARBP

QMPGIKPLSSYIDIPKILTLPAKEIETVWRLRHAANPQSVCATIS-LDTYMRMVGIARQNPQFVLPLPRK

AAGSWPDDVKGGTGADIHFLQWAFH--HTSTVLFTHLASYKLHGAYSQPHTIITHYLDLAETKGLVLMSG

AVVSDRGVAMDEAKLLVMWLQRFYD-WGVDGAQAGKRAELVRMFNQGDNGFKLEDLMDEVERV--

>C1FZG0|C1FZG0_PARBD

QMPGIKPLSSYIDIPKILTLPAKEIETVWRLRHAANPQSVCATIS-LDTYKRMVGIARQNPQFVLPLPRK

AAGSWPDDVKGGTGADIHFLQWAFHPPHTSTVLFTHLASYKLHGAYSQPHTIITHYLDLAETKGLVLMSG

AVVSDRGVAMDEAKLLVMWLQWFYD-WGVDGAQAGKRAELGENVQPG------------------

>C5GR50|C5GR50_AJEDR

QTPGIKPLSSYLDIPKILTLPAKEIETLWRLRHAANPRSICAAIP-IDTYLRMVDTARENPQFVLPLPRT

ADSGQPEDMNDSTGADIHFLQWAFH--HTSTVLFTHLASYKLHGAYSQPHTIITHHLDLADNTGLILMNG

SVVQDRGVSVDEAKLLVLWLQRFYD-WGVDGKQAGKKADLVRMFTQGDNGFKLEDLMEEAEKI--

>A6R7P9|A6R7P9_AJECN

QTPGIKALSSYFDIPKILTLPAKEIATLWRLRHASNPQSICATIP-IDTYLRMVATARQNPQFVLPLPRT

EESGQPEGVKACTRADIHFLQWAFH--HTATVLFTHLASYKLHGAYSQPHTIITHHLDLADKSGLVLMNG

SVVPDRGVSVDEAKLLVMWLQRFYD-CGVGGNQAGKKAELVRMFTQGDNGFNLEDLMEEAERI--

>C6HBD9|C6HBD9_AJECH

QTPRIKALSSYFDIPKILTLPAKEIATLWRLRHASNPQSICATIP-IDTYLRMVATARQNPQFVLPLPRT

EESSQPEAAAACTRADIHFLQWAFHTPHIATVLFTHLASYKLHGAYSQPHTIITHHLDLADKSGLVLMNG

SVVPDRGVSVDEAKLLVMWLQRFYD-WGVGGDQAGKKAELVRMFTQGDDGFNLEDLMEEAERI--

>F0UCC7|F0UCC7_AJEC8

QTPRIKALSSYFDIPKILTLPAKEIATLWRLRHASNPQSICATIP-IDTYLRMVATARQNPQFVLPLPRT

EESSQP-----CTRADIHFLQWAFHPPHIATVLFTHLASYKLHGAYSQPHTIITHHLDLADKSGLVLMNG

SVVPDRGVSVDEAKLLVMWLQRFYD-WGVGGDQAGKKAELVRMFTQGDDGFNLEDLMEEAERI--

>P87127|ATP11_SCHPO

KKSDLSGLNRYIDVEKIKELPTSTIEKLWRARNIG-DDILSACIP-KEIYEKMLSRARMYPYFVLPLPRG

-----------DKGIESHFLQWNFPNKNEAHLLVTSLLEYKLKGSYAAPHTIMLHFADLLNLKGITLMRC

QF-EPKKLSANDVQLLVLAIQKFYN-ASENTPLGKERLALLAAFSKG-ADFDLHKVATHMDMLE-

>Q6CEA1|Q6CEA1_YARLI

PKNELKNLDSFVKLDKFKELGKQEIEFLWRARHINNERALCAVVD-PQLFYKMFLNGRKHPMFVLPLPKG

-----------DEGCEMHIVQWNFV--HLTHVIFTTLAEFKLHQDFARPHTTLMFHTDLAADKDCVLLNG

QVEKDSAMSLQDAQFLILALQEFYG-ATITDPETTKRRQLLQSFTEGSDNFPIDMVIEEIEAFTK

>G8B8W3|G8B8W3_CANPA

EKLPFKVLNDFIDVDKARGLPTEDIKLIWKHRFLEKERALHASLD-NRQFADIYANAYRYPNFVLPLPKP

H----------NDGYELEFVQWAFA--NTIHCMFTTLAEYKLNKEFARPHTTLTFHQEFSQDKDLVLMNG

VSEKEGGLTMDEAQLLAVNLQRFYS------GKFPQMTKLLKEFNEGSADFSVDELIKEATSV--

>C5MFL6|C5MFL6_CANTT

PKLPYKTLDDFVNVGKTRELPRDDIIKIWNARFASNDRALHAVLS-HLQFAELYVNAFKYQQFILPVPKP

S----------QDGYELQYVQWQFV--ETINCMFTTLAEYKLHGEYASPHTTLTFHLELAEDKDLVLMNG

YVDKESGITMDEAHLLVVNLQRFYS------GKYPEKTKLLKEFNEGDENFNIDELIQQSTTV--

>G1UAF9|G1UAF9_CANAX

PKLPYKVLDNYINLSKAKELPRDDIIKIWTARFINNDRALHAILT-HLQFAQLYTNAFKYPQFVLPLPKP

Q----------QDGYELEFVQWQFVGPNTINCMFTTLAEYKLHGEYASPHTTLTFHLELAQDKDLVLMNG

FNNKESGISMDEAHLLVVMLQRFYS------GKNPQMTQLLHEFNKGNAEFDIDALIKQATSV--

>B9WK21|B9WK21_CANDC

SKLPYKVLDDYINVSKVQKLPREDIIKIWTARFINNDRSLHATLT-HLQFSQLYINAFKYPQFILPLPKP

Q----------QDGYELEFIQWQFI--NTINCMFTTLAEYKLHGEYSSPHTTLTFHLELANDKDLVLMNG

FNNRESGISMDEAHLLVVMLQRFYS------GKNPQMNQLLYEFNKGNSEFDIDSLIKQATSV--

>A5E784|A5E784_LODEL

EKAPYKKLDDYIDVEKVKPLPRDQIELIWKTRFQNDPRSLHAVIN-NQQFEEITQNASEYPFFLLPLPKT

NEKDE------HKGYQLEFVQWSFVGANTIHCLFTSLAEYKLHGEFAKPHVVLSFHQDLKNDKDIVLMNG

LNEKDGGLTTQEAQLLVLNLQRFYN------GKDQKRLALVKDFNTGSENFSVEKLISEATTV--

>G3BBJ3|G3BBJ3_CANTC

PIAPYKTLDSFVDSAKLKDLNKQEIEYIWRARFQKDDRSMVAILG-ATQFSNMYALGFKNRTFILPLAKE

-----------SGGYEMHFVQWSFV--ETTHCMLTTLAEYKLHNEYAKPHSTIMFHQELAHEKDVVLMNC

HVEKESNVSLADAQFLILNIQRFYG---AMKSISKAKLELLEAFNRGEEMFDMEKLIEEATSFE-

>E7R405|E7R405_PICAD

PKDPYKTLNSYLDLQKIKGLEANEIEFLWRLRFQKGENTLSAVVP-TSTFERLFNNAIKNPTFVLPLPRE

DAQVEGEGK--GDPVEIHFVQWNFV--NTTHVLITSLAEYKLHKEYARPHTTVMFHQELKEDKGIVMMNG

QVEKDASVTLQEAQLLLLNVQRFYGLAETTSESNQKRLELLRRFNSGDPAFSMEELIELAQRMEN

>G8YCA6|G8YCA6_MILFA

SKKPFKTLDSYIAVDKTKELPRKELEFIWRARFNNKENNSHAVLD-SKQFASMYSNAFKNPNFILPVPRG

-----------NEGYEMHFVQWSFAGPQTTHCMITTVVEYKLHKEFAKPHTTLSFHQELMESHDAVLMNG

QTDPDANVSMEDAQLLVLNIQRFYG--GVGSDDNVMKKVLLRDFNSGKEDFPIDALISEATKFD-

>G8YEQ7|G8YEQ7_MILFA

SKKPFKTLDSYIAVDKTKELPRKELEFIWRARFSNKENNVHAILD-SKQFASMYSNAFKNPNFILPVPRG

-----------NEGYEMHFVQWSFA--QTTHCMITTVVEYKLHKEFAKPHTTLTFHQELMESHDAVLMNG

QTDPDANVSMDDAQLLVLNIQRFYG--GVGSDDSVMKKVLLRDFNSGKEDFPIDALIAEATKFD-

>G3AV38|G3AV38_SPAPN

PKLPYKVLNDYVDLDKLKELPRREIEYIWKARFQDKQKSVHAVID-AVPFAAMYANAFKNPNFILPLPR-

-----------DNGYEMHFVQWAFV--ATVHCMLTTVAEYKLHGEYAKPHTTLSFHQEVS-DKGVILMNG

VI-ENDTIPMDEAQLLVLNVQRFYG----MGEQNEKKLKLLKQFTTGDDGFSTEDLIKEATTF--

>C4YBZ6|C4YBZ6_CLAL4

PKAPYKTLSSFLDVDKVKELPEKEVDFLWRARFQQKERALHATLN-ATQFANIFANAFKNPNFILPLPKD

-----------GEGYEMHFVQWAFV--HTTHCMLTSLAEYKLHKEYAKPHTTLMFHQELVDEVGKVLMNG

QVEEDVPLSMDEAQLLVLNVQRFYG-GLTESEGSKRKLELLKEFTAGNPDFNMERLIEEAASFD-

>A5DLP1|A5DLP1_PICGU

EKKPYKTLDSYLASDKLKDLGQKEIELIWRARFENKERTLNAILT-DIQFATMYANAFKNPSFILPLPRN

-----------EDGYEMHFVQWSFA--QTTHCMLTTLAEYKLHKEYAKPHTTLMFHQELVNSKNLVLMNG

QVEEEAALSMDDAQLLVLNVQRFYG-AVGDEASIKRKHELLRAFTTS-ENFDVDTLIEEATSV--

>A3LWI5|A3LWI5_PICST

PKLPYKDLNAYVDVEKVKELPRKELEFIWKARFHSKERTLHAVLE-DLQFASMYVNAFKNPSFILPLPKN

-----------SDGYEMHFVQWSFV--QTTHCMLTTVAEYKLHKEYAKPHTTLMFHQDLAQEKGVVLMNG

HCETESSLSMDEAQLLVLNVQRFYG-----GLGKHESALLLRDFTSGSEKFDMDKLIAEATSFQ-

>Q6BL75|Q6BL75_DEBHA

PQAPYKVLDSFVDVEKLKDLPLKELEFIWRARFQNIERTMVAVLN-NLQFASMYANAFKNPSFILPLAKN

-----------EDGYEMHFVQWSFI--KTTHCMLTTVAEYKLHKEYAKPHTTLMFHQELSNSKDVVLMNG

QVEEESSLTMDEAQLLVLNVQRFYG-GIASAKGNEKKLALLRDFTSGNADFNMEELIAEAASID-

>C4QVC4|C4QVC4_PICPG

PIKPYKVLDDFLKLEKIKDLSPDQVEFLWRARFQEKENVLVAVIP-KDVYQKLYTNARENPSFILPLPKE

-----------DEGVEIHFIQWAFP--NTTHCMFTSLAEYKLHKEYAKPHTTLMFHHDISEDKNIVLMNG

TVEQDANITLPETQLLVMNLQRFYG-ALAESPASNRRLQLLKDFTSGNENFSLDLVIQEAQSLEN

>G8C2C6|G8C2C6_TETPH

KNAPFKTLASYLELEKVKDLSPQEIEYLWRAKWGQTDGAMNAVVP-LETFDKMLVKVKEHPVFVLPLPRA

NTSGEKDEA--SEGMELHYIQWQFPNSQTSHCLMTSLAEYKLHKEFARPHTTLEFHSELGKDKNIVLMNG

NVEPSMNISIQEAQLLLLNVQRFYGAMGYDTPSSKQRLQLLKDFTNGSSDFNLDKLIILSQSMEN

>2P4F|PDBID_CHAIN

KDKPYKTLDDYLKLDKIKDLSKQEVEFLWRAKWSNRDDSLVAVVPYVKTFQGMYKYAVKNPLFVLPLPRE

NAADGNKADKDSVPVELQYVQWQFAGPNTVHCLITSLAEYKLHQDFAKPHTTIQFHLDLANDKDMVLMNG

QVESDSNVSLQDAQLLLLNVQRFYGAMGSETSIAKERIQLLEDFNKGSQNFDINKLIQLAQSMEN

>C5DSU2|C5DSU2_ZYGRC

ERVPYKTLDSFIKVDKFSELSKQEVEFLWRAKWMGKDDSLCAVAP-ADVFEKMTKTAKENPVFVLPLPRV

MEGETKDGEP-NEGSELHYIQWQFV--NTTHCMITSLAEYKLHKDFARPHTVVQFHSDLAKDKGLVLMNG

HVEKDTNVTLQDSQLLLLNIQRFYGAVNDQTPIEQQRLRMLKAFTKGSPDFNIELLISLAQAMET

>C5DJU4|C5DJU4_LACTC

EPAPFKTLDSFLKLEKIKDLSKQEVEFLWRARWANKPDALSAVVP-LEIFEKMDRHIRACPTFVLPLPRE

VPAEASSSGTQEQGMELHYIQWQMA--NTVHCIMTSLAEYKLHSEFAKPHTTFQFHKELASDKKIILMNG

HVEKDVNITLPDAQLLLLNIQRFYGAMGEETVSSKQRTKLLHDFAKGSPEFSVEKLINLAQSMET

>Q75AU9|Q75AU9_ASHGO

PRAPFKTLDSYLDVEKIRQLSKQEVEFLWRARWMNKDGVLNAVVP-VDVFERMSGYARANPAFVLPLQRD

ATGAGEEQG--GQPVEMHYVQWQFV--KTVHCIITSLAEFKLHKEFARPHSTFQFHLELADQKKVVFMNG

QIESDSNVTLQDAQLLLLNVQRFYGAMGDATQAAKQRIKILQDFTAGSVDFKVDDLIALAQSLET

>Q6CP66|Q6CP66_KLULA

PKQPFKTLDSFLDVEKVKNLSKQECEFLWRARWANKDNVLNAVVP-IDIWEKISALAKENPFFVIPLPRE

RAVEETAAESAEPAMEMHYIQWQFVGPKTVHCIMTTLAEYKLHKEFARPHTTLQFHLDLAKEKNIVLMNG

QVESDSNVSLPESQLLLLNVQRFYGAMGQDSGFAQKRVQLLKDFTNGSPNFNVDLLVALSQVME-

>G8JNZ0|G8JNZ0_9SACH

PQLPFKTLNSFLNVEKMKQLSKQEVEFLWRARWMEKDNMLNAVVP-VDVFNKMSSNAKSNPILVLPLPRD

IQAANTEGQG-EQGIEMHYIQWQFV--KTVHCIMTSLAEYKLHKGFARPHTTFQFHLDLAEEKKIVLMNG

QVETDANISLQEAQLLLLNVQRFYGAMGDHTTMAKARLKILKDFNTGSSDFNVDLLISLSQSMET

>G0WIM7|G0WIM7_NAUDC

LKVPFKTLGSYLQLEKITELSKQEIEFLWRAKWAATKDSLCAVVP-VDIFNKMLGNAKANPVFVLPLPRV

VGEGKE-----PEGMELHYIQWQFLGSNTIHCIITSLAEYKLHTEFARPHTTFQFHLDLESEKKIVLMNG

HVQTDMNVSVQDAQLLLLNIQRFYGAMGEETPMAKERLKILRDFTKGSTAFNVDTLIECAQSMEN

>G0VFE1|G0VFE1_NAUCC

PKAPFKTLDSYLATEKIKDLSKQEVEFLWRARWAEVKDSLCAVVP-VDVFNKMLVNVRGNPIFVLPLPRV

LDPSKTANE--PQGMELHYIQWQFVGKDTIYCIVTSLAEYKLHTEYARPHTTFEFHLEMEPTKKIVLMNG

HVETDMNVSLADAQLLLLNIQRFYGAMGEETPAAKQRLALLRSFTAGSSEFNVDLLIASAQSMEN

>G8ZL87|G8ZL87_TORDC

PKIPFKTLDSFLDVEKIKELSKQEVEFLWRAKWMSKDDALCAVVP-VDIFEKMIVNAKSNPIFVLPLPRQ

SQDDGKDAKE-NQSVELHYIQWQFV--NTVHCIMTSLAEFKLHNEYSKPHTIFQFHSEMAADKKIILMNG

QVEPDTNISLQDAQLLLLNVQRFWGAMGEETPVAQQRVKLLRDFTKGSPDFSVDALISLAQSMEN

>A7TNE4|A7TNE4_VANPO

PKVPFKTLDSYLNVEKISQLSRQEVEFLWRAKWANKDNVLNAVVP-IDVYEKMLANSKENPAFVLPLPRE

AEGQ-------DSGMELHYIQWQFV--HTVHCIMTSLAEYKLHKEYARPHTTLQFHSELAKDKNCVFMNG

QVETDMNVSLQDAQLLLLNVQRFYGAMGEETPSSKQRLQLLRDFTKGSASFNVDTLITLSQSMEN

>H0GZU6|H0GZU6_9SACH

PKVPFKTLNSFLDVAKLNDLSKQEVEFLWRARWAQNDHALCAVVP-VAIYDKIMANARNNPIFVLPLPRQ

AQSEDAKPSE-HQGMELHYIQWQFVGPQTTHCMMTSLAEYKLHQEFARPHTTFQFHSDLVKDKGIVCMNG

QVEPDTNISVQDAQLLLLNVQRFYGAMGEETPAAKQRVQLLRDFSKGSPQFSVEKLISLSQSMEN

>G2WLF4|G2WLF4_YEASK

PKVPFKTLDSFLDVGKLKDLSKQEVEFLWRARWAQKDNTLCAVIP-VSVYDKMMANARNNPIFVLPLPRQ

VQSEDAKPNE-EQGMELHYIQWQFVGPQTTHCMMTSLAEYKLHQEFARPHTTLQFHSDLVKDKGIVFMNG

HVEPDTNVNVQDAQLLLLNVQRFYGAMGEETPVAKQRVQLLRDFSKASPGFTVEKLISLSQSMEN

>G7DZQ0|G7DZQ0_MIXOS

SASPVRPLSDIIKLDKAFEETPKKISDLWNAYHMT-KGKLSAVIP-TEIYRAMAKEARQYSSFVVPLPRG

PSAEASDR---PPAAEMFFLQWAHL--DVTTVLFTPLAEYQLHQTYAQPYLILTHYTDLAHSHGIVLMRG

EITPDVNLTSADAQLLIWKLQQFYNTANMKADKGQAVRRLLRTFHEKPSDFDLARLIELADQI--

>E6R8P4|E6R8P4_CRYGW

MKEKREPLSSIINLPLIHLHDTNAISQIWNAYHTS-HPTLSASLP-ASTYASMIALAKQNPFFVIPLPRL

SEAPVEAANHPTDEYEMFYLQWLFH--LTTSAIFTPLEEFKKSGEWAQPYLVLTHYPDLAQTHSLILMRG

EISSASLLSQQQAQLLALALQRFYCMPGESAKQQEERRDALRGFRERPEEWDWMGLVEAYGGLV-

>Q55QB6|Q55QB6_CRYNB

DKSGIKPLSSIINLPLIHLTPTNAISQIWNAYHTSHPTLLSASLP-ASTYASMLALAKQNPFFVIPLPRL

SEAPVEAANHPTDEYEMFYLQWLFHPTLTTSAIFTPLEEFKKSGEWAQPYLVLTHYPDLAQTHSLVLMRG

EISSASLLSQQQAQLLALALQRFYCVPGESVKQKDERQDALRGFRERPEEWNWTGLVEAYGGLV-

>F4S7U3|F4S7U3_MELLP

YRSPIKPLDDILDLSKVINQSSDTIKQLWTAYHLKPSSPLGAVIP-LEIYQTMLKVAQKYSSFVIPIPTS

STDEIKPD---QTPLQMHFLQWSFL--SPTTVLFTPLIEYQLKQEFAQPGLILTHYTELAESHGIVLMRG

DLTSGR-IGTTEAQLLILRLQQFYYNESLMNDSQNRLRQLLRCFHEKPNEFDIEELIKSIKDI--

>E3K0V8|E3K0V8_PUCGT

YSSPIKPLDSIIDLNKIATQSPSEIRKLWTAYHLS-KSDLGAVIP-CATYQEMLQAARKYPSFVVPLAKS

SAVDSSEQAKPDVPYEMQYLQWDFV--PATIVMYTPLGEYKLRQTFAQPTLILTHYTDLAESHGIVLMRG

DITPHSKITAIEAQMLVLRLQQFYH-ATPSANQDQSVRRLLESFHEQPDRFQLSELLQLVNDL--

>A8PYI9|A8PYI9_MALGO

SPSPVQPLSSFMKLEKVVHETPDNIGKLWAGFHIM-KNKVSAVVP-AATYVQMIVTAKKFPQFVLPLPRA

VKNEAGNA---ENGYEIYYLQWVSLPKPPMAVLFTPLAEYKLRQEYAQPTLVLTHYTELLESKGIALMRG

DITESEHVQQQDAQLLALGLQRFYNDWALEGLDNDEERRLLRSFYERPEAFKLEDLIEAWSV---

>G4T8S5|G4T8S5_PIRID

DSSPVTPLSDIMDLGRILQHTSEQIGALWTTYHTAKATQLSAVIP-RETYESFLPAAKKYPSFILPVPRP

EAQVELKEGEERTPYEFFYMEWGHH--PCTTVIFTPLQEYKLRQAYALPHLVLTHYTDLAQSHGLVLMRG

ELTPGRLMTPSTAQLLAFGLQQYYL------PKSDNRHALLTTFHENPEQFSWEELISASQL---

>D8QLW5|D8QLW5_SCHCM

DSSPVKPLASILNVEKMLDHTAEQVSQIWTTYHDLQSGGVSASIP-LPTYEKMLSIARRYPTFVIPLPRL

VEKDDAPSSTEETAYEFYLMQWDIH--PTATIIFTPLQEYKLRQQFATPYMVLTQYTDLAASHGVVLARG

EITPSTMLGQEDARSLVMAIQKFYL----WDQGKKEN--LLQVFHERPEEFKWEDLIQQVKVVA-

>A8N2H4|A8N2H4_COPC7

DSSPVKPLSTIFNVEKLVSHTPEQISTLWTAYHASRSGGVCASIP-SDMYGKLEKVGQKYPAFVLPVPRP

RPDDTGPLKEGDTAYEFYFMEWAFH--PLATVLFTPLQEYKSRQTFATPYLIVTFYTDLVTTHQRVLMRG

EITPAALLSQEDAQVLIMGLQKFYL-WTSEGEGESEAAKLLRVFHEKPEDFKWEDLVKIGNLTG-

>B0CQX7|B0CQX7_LACBS

DSSPVKPLDTILNLPRIMATPAAQVSSLWTAYHLSRSGGLCASIP-LPLYLKMEAVGQKYPSFVVALPRP

KNPSDDQLAENDTAYEFYFLQWSFHDRQISTVLFTPLQEYKLRGAYATPYLVLTHYTDLASTHGISLLRG

EITTDRLLGQDDAQLLSMAIQKFYLWGENKECGDSER--LLRTFHDNPGEFKWKELLK-FGSLSL

>F8NKT0|F8NKT0_SERL9

TAPPVKPLSSLLNIPLILSSPPAQLSALWTAYHASRSGGVCASVP-VGLYENMIGTARKYGSFIVPVPRL

GGEGGK-----EEAYEFYFLQWAFHTPPTSTVLFTPLQEYKLRASFATPFLALTFYPDLARSHGNVLLRG

EITGAAMLTQNDAQLLVMELQKFYLWESGKSTSTAER--LLKTFHERPEEFKWEELLEHAKLTA-

>F4P0T9|F4P0T9_BATDJ

KLPSYEKLDQIVKLELLMKETPEMVSIIWNKYHSD-KNCLSASID-SATYKKLHQRGRKYPLFILPLPR-

-----------NDGYELYFIQF-----SGHQTYYTPLLEYKTHGALSRPSFVVTHYDDLAASKSIVLMVG

ELGQSSNLTLTEAQNLVYQTQLFYI----TGNENQQK--LVETFHEQPESFQYEELIKAVETLT-

>F0ZCX5|F0ZCX5_DICPU

GPPG--KLNDVVKLELLEKEDASTVKQIWLQYHLQ-KECLCAVIP-SDIYKKLISRSKECPLFIIPLPG-

-----------DKGFISILYQN-----QGDHLVFTYLEQFKKHSVNAVPWLIASHYTDFIDSKGIVLMRA

EP-NLEVLNNTQAQYLYNQIQSYLL--------DDTKYKIMETFTKRPHEFDFNLVIKDMEKMSL

>Q54VM9|Q54VM9_DICDI

GPPG--KLNEVVKIELMEKEDVATIKDIWLQYHLK-KDCLCAVIP-KDIYKKLIERSKACPIFIFPLPG-

-----------DKGFISMLYQN-----QGDHFVFTYLEQYKKHTVNAVPWMVASHYIDFIDSKEIVLMRA

EP-NLEVLNNIQAQYLYNQLQTYLL--------DDNKYKIMQTFTNRPNEFDFNDVIKNMDSMSL

>E4XS24|E4XS24_OIKDI

SYVAPKTLDTVMDLQRLKNKDGKEISDIWRKFHSH-RSAVFASIS-KKYWDYFATIKTHFPSFIYPLPRD

-----------DNKWLFYIGFW-----GGNELNFTTLEHYKLKGADAPVLLSMCHYPDLVDTKGICLMVA

DV-DESLIEKHDAQLLAYYAQYFHT------EPNGMS--LVQIFNTRPQDFKYDDVIKAVETIGQ

>C4WY42|C4WY42_ACYPI

LLVKPKTLEQVMNIELLRDKSWRDVSEIWLAYHRTRDETLSAVIP-LNQFEEFYNQSIKYPMFLLPLPRN

-----------TSGYEFIFAQFQHYQESNCTVHLTPLISYQTFNENAPECMTIKYYTDLSDDESCVLMRS

TY-DGKLLSAAEAACLVNQLRIFYA--VDSGVAEKEK--LLKSFNEGDVTFKHSDVIALVETLSI

>B7QP22|B7QP22_IXOSC

TKPKPLDPDTLLELRG---KTSSELTEAWRKLHSD-RDAVCAVLP-SAVYERIHERALEFPVFLFPLPR-

-----------QNGYEFVLSQF-----LGDQCHMTPLAAYQRYASEAPPCLSLTFRTELGPERGVTLMSG

EY-DPGLLGPAQAQCLVNQLQLYYG----GSELKKKL--LLWNFNREPLSFKHEELVREFERSLA

>G3MI71|G3MI71_9ACAR

DADAIRELRSRC-------KTTAELTDAWKALHSA-RDAVCAIIP-SSVYETIRERARDYPVFLYPLPRD

-----------GSGYEFVLSQF-----VGDQCHMTPLASYQRHGSEAPACLALAYWTQLAEDGGVVLMSG

EY-DAKVIGPTEAQCLVNQLQLYYG-------GNELKKKLLWNFNREPRSFKHEMLISEFEASAT

>F2U4W5|F2U4W5_SALS5

LPPFVPRLDSILHVHKLRDVAPEDIARTWTQHIAT-SDSLSAVIP-AETYDLLHARGKESPLFLYPLPQG

-----------GSGYDFYVGQF-----AGNVCAFTSLLEYQTHAENARPVLTLFHYTELKRSKGHVLMMG

ELDIDR-MNMAAAQLLVHQLQLFYA---NQTEDAKDAYDLVRQFNTRPGSFDYNDLLRRFQEAGV

>B3S1T1|B3S1T1_TRIAD

VVGQRKNLNDIMYVSKIQEKSGTEIAQIWTEYHKQ-RDCIGAVIP-NAIYERMYKRSFECPVFVYPLPR-

-----------NEGVEFIFAQF-----DGNDCHFTPLLSFKTFGENAPPILTISHYKEFSDNKGIVLMSG

NW-DPKQLNTTEAQFLANQLQLFYA------GEDESRYSVVKSFNHFPENFNYEDILQSLKK---

>F6VHJ1|F6VHJ1_XENTR

GFSKNKTLDSILNIELIKDKDADEIREIWKQYFSL-RNSVYAVIP-GESFELIWRRAKTCPSFLYALPR-

-----------KEGYEFFVGQW-----SGSELHFTALINIQTAGDAAPSQLILYHYPEFQKDKGIVLMTS

EI-DTKFLNVQDAQCLANQVQLFYG------SDGAETFGLVEKFNHKSDEFKYMAVVSFLEQSGL

>F6PSH6|F6PSH6_ORNAN

RNK---TLDSILNLDLVKDKTAEEIGQIWQRYFSA-KDTVFAVIPPAKTFDLIWTRARSCRSFLCALPR-

-----------REGYEFFVGQW-----SGTELHFTALINIQTRGDAAASQLVLYHFPELKAEKGIVLVTA

EL-EPTFLTVPEAQCLASQVQLFYA------TDCQQTFALVETFNHRPGEFKHMAVITALQQSGL

>F6W119|F6W119_MONDO

KD---KTLSSIFNIEMVKDKTAAEIQQIWQQYFAT-KDTVYAVIP-AETFDLIWSRAQKCPSFLCALPR-

-----------REGYEFFVGQW-----SASELHFTALINIQTRGEAAASQLILYHFSELKEDKGIVLMTA

EM-DSTFLKVPEAQCIANQVQLFYA------TDRKETFGLVETFNLRPNEFKYMSVIAELEQSGI

>H0V0N6|H0V0N6_CAVPO

KD---KTLSSIFNLEMVKDKTAEEIKQIWQQYFAA-KDTVYAVIP-KEKFDLIWSRAQSCPTFLCALPR-

-----------REGYEFFVGQW-----TRTELHFTALINIQTRGDAAASQLILYHYPELKEEKGIVLMTA

EM-DSTFLNVTEAQCIANQVQLFYA------TDRKETYGLVETFNFRPNEFKYMSVIAELEQSGL

>D3ZY50|D3ZY50_RAT

KD---KTLSSVFNVEMVKDKTGEEIKQIWQQYFSA-KDTVYAVIP-KEKFDVIWNRAQSCPTFLCALPR-

-----------RDGYEFFVGQW-----TGTELHFTALINIQTRGDAAASQLILYHYPELKEEKGIVLMTA

EM-DSTFLNVAEAQCIANQVQLFYA------TDRKEIYGLVETFNFRPNEFKYMSVIAELEQSGL

>G3GYU5|G3GYU5_CRIGR

KD---KTLSSIFNIEMVKDKTAEEIQQIWQQYFSA-KDTVYAVIP-KEKFDLIWNRAQSCPTFLCALPR-

-----------RDGYEFFVGQW-----TGTELHFTALINIQTRGDAAASQLILYHYPELKEEKGIVLMTA

EM-DSTFLNVAEAQCIANQVQLFYA------TDRKEIFGLVETFNFRPNEFKYMSVIAELEQSGL

>Q811I0|ATPF1_MOUSE

KDK---TLSSVFNVEMVKDKTAEEIKQIWQQYFSA-KDTVYAVIP-KEKFDLIWNRAQSCPTFLCALPR-

-----------RDGYEFFVGQW-----TGTELHFTALINIQTRGDAAASQLILYHYPELKEEKGIVLMTA

EM-DSTFLNVVEAQCIANQVQLFYA------TDRKEIYGLVETFNFRPNEFKYMSVIAELEQSGL

>D2HC60|D2HC60_AILME

KD---KTLSSIFNIEMVKDKTAEEIRQIWQQYFAA-KDTVYAVIP-KEKFDLIWTRAQSCPTFLCALPR-

-----------REGYEFFVGQW-----TGTELHFTALINIQTRGDAAASQLVLYHYPELKEEKGIVLMTA

EM-DPTFLNVAEAQCIASQVQLFYA------TDRKETYGLVETFNFRPNEFKYMSVIAELEQSGL

>G1PA73|G1PA73_MYOLU

KD---KTLSSIFNIEMVKDKTAEEIKQIWQQYFAA-KDTVYAVIP-EKKFDLIWNRAQSCPTFLCALPR-

-----------REGYEFFVGQW-----SGTELHFTALINIQTRGEAAASQLILYHYPELKEEKGIVLMTA

EM-DSTFLNVAEAQCIANQVQLFYA------TDRKETYGLVETFNFRPNDFKYMSVIAELEQSGL

>G1SWT7|G1SWT7_RABIT

KD---KTLSSIFNIEMVKEKTAEEIKQIWQQYFAA-KDTVFAVIP-KEKFDLIWNRAQSCPTFLCALPR-

-----------REGYEFFVGQW-----TGTELHFTALINIQTRGDAAASQLILYHYPELKEEKGIVLMTA

EM-DSTFLNVAEAQCIANQVQLFYA------TDRKETFGLVETFNFRPNEFKYMSVIAELEQSGL

>H0XB80|H0XB80_OTOGA

KD---KTLSSIFNIEMVKEKTAEEIKQIWQQYFAA-KDTVYAVIP-EEKFDLIWSRAQSCPTFLCALPR-

-----------REGYEFFVGQW-----TGTELHFTALINIQTRGETAASQLVLYHYPELKEEKGIVLMTA

EM-DSTFLNVAEAQCIANQVQLFYA------TDQKETYGLVETFNLRPNEFKYMSVIAALEQSGL

>C0KHL4|C0KHL4_HORSE

KD---KTLSSIFNIEMVKDKTAEEIKQIWQQYFAA-KDTVYAVIP-EEKFDLIWNRAQSCPTFLCALPR-

-----------REGYEFFVGQW-----TGAELHFTALINIQTRGEAAASQLILYHYPELKEEKGIVLMTA

EM-DSTFLNVAEAQCIANQVQLFYA------TDRKETYALVETFNFRPNEFKYMSVIAE------

>B5FZC4|B5FZC4_TAEGU

KD---KTLDSILNVEMVKEKSAEEITQIWNQYFSA-KDTVYAVIP-ADKFDLMWKRAQKCPSFLYALPR-

-----------KEGYEFFVGQW-----SRTELHFTSLINVQTQGEAAPSQLVLYHYPDLQKEKGIVLMTA

EM-DSKFLGVHDAQCLASQVQLFYA------TDRSETYGLVETFNHRSSEFKYMSVVAELEQSGL

>H0ZDJ2|H0ZDJ2_TAEGU

GFTKDKTLDSILNVEMVKEKSAEEITQIWNQYFSA-KDTVYAVIP-ADKFDLMWKRAQKCPSFLYALPR-

-----------KEGYEFFVGQW-----SGTELHFTSLINVQTQGEAAPSQLVLYHYPDLQKEKGIVLMTA

EM-DSKFLGVHDAQCLASQVQLFYA------TDRSETYGLVETFNHRSSEFKYMSVVAELEQSGL

>F1N9I9|F1N9I9_CHICK

RD---KTLDSILNVEMVKEKSAEEIEQIWNRYFSA-KDTVYAVIP-AEKFDLIWKRAQKCPSFLYALPR-

-----------KEGYEFFVGQW-----SGTELHFTSLINIQTQGEAAPSQLVLYHYPELQEEKGIVLMTA

EM-DSKFLVVHEAQCLANQVQLFYA------TERSETYELVETFNHRSSEFKYMSVIAELEHSGL

>G1NE40|G1NE40_MELGA

GFTRYKTLDSILNVEMVKEKSAEEIKQIWNQYFSA-KDTVYAVIP-AEKFDLIWKRAQKCPSFLYALPR-

-----------KEGYEFFVGQW-----SGTELHFTSLINIQTQGEAAPSQLVLYHYPELQEEKGIVLMTA

EM-DSKFLVVPEAQCLANQVQLFYA------TDCSETYELVETFNHRSSEFKYMSVIAKLEHSGL

>E3TEE9|E3TEE9_ICTPU

GFTKNKTLDSILNLDLVKDKSGLEIGELWMNFYST-KDTISAVIP-GSTFEKIINRAKLCPTFVYALPR-

-----------KEGYEFFLGQW-----AGQELHFTSLINVQTLGENAPSQLILHHYSDLKQDKDLVLMTA

EM-DRKFMTVHEAQCLANQCSYFYA------AQRHETFQLVETFNHRPAEFRHMSVIAELEQSGI

>F1Q4Z5|F1Q4Z5_DANRE

GFTKDKTLGSILNLEMVQEKSGAEITELWMQYFSK-KDTISAVIP-SSTFDVIFGRAKSCPTFLYALPQ-

-----------NEGYEFFVGQW-----AGNELHFTSLINVQTMGENAPSQLILYHYTDLQKDKDIVLMTA

EM-DSKFVTVHQAQCLANQVQLFYG------SQRLETFRLVETFNHKPEEFKHMAVIAELEQSGI

>G3NNZ4|G3NNZ4_GASAC

GFTKNKTLDSILDLDMIRDKTGEEIAELWMNYYST-KDTISAVIP-TPMYELILSRSKSCQMFLYALPQ-

-----------KEGYEFFLGQW-----SGHELHFTSLINVQMHGENAPSQLILYHYADLKDQKGVVLMTA

EL-DPKNITVHQAQCLANQVQLFYG------TQRKETYQLVETFNQHPSDFKHMLVIAELEQSGL

>F7BEP5|F7BEP5_CIOIN

VTKKTKTLDDILKLEMVMKLKAEDIQKLWVDRYAN-KDAVCAVIS-SQSYCIIRTILEKYPVFVFPLPR-

-----------KDGYEIFVGQF-----SNNDIYFTSLINYQRHKENAPSQLTLNHFTELESEKGIVLMSG

PVFDDA-LSVTDAQLLAYQVQHFCT--------EHPK--LIRGFNVSPQDFDINSVISSLDTSSL

>A7RVY8|A7RVY8_NEMVE

------TLDSVLRMELISELSGEEIGKLWREFHKD-KDCISAVIP-ANVYKTIEERSTKYPLFIYPLPR-

-----------ESGYEFIYSEF-----SGKHCYLTSLINFQTMAENAPWFLAVTHFTELQDTKGVVLMVG

EV-DTNHLSVVDAQWLAYQIQMYYA------SDSAERESLLHTFNIEPNKFDHMSVVEQLNNITA

>C3YMH8|C3YMH8_BRAFL

SMAGAKKLDAVLRTDLIQDKTAEEIAKIWTDYHAQ-KDALCAVIP-KDTYAVLNARTNMCPTFLLPMPR-

-----------EQGYEFFLLQF-----SGQECHFTPLINFQTHKENAPSCIQLVHYPDLAEEKGIVLMKA

EV-DTKVLTTLEAKFLVDQMTMYYT------ARSDQRFSIVRNFTVKPTEFDHMVLIKELEALGG

>E9FXZ9|E9FXZ9_DAPPU

MHSKTKELNSIMKTELLEDKNFEEIKQIWHEYFKD-KISVSGVLT-KPIYDQLNNRAMQYPTFLFPLPR-

-----------DEGYEFLLCQF-----AGNEAHFTSLINFQTYGENAPECLTVVFFPDLAEQKNIVLFRG

DY-DKNILNAIEAQCLVNQIVLYFA------QPSERKLDLLERFNKQPDTFQHMELVQELENIKL

>Q7Q7R6|Q7Q7R6_ANOGA

RDITHRKLSDVMRLELIEDKSADDIKHLWLEYHRD-KDVITAAIP-VDQFNQMMECAKQYPVFILPIPR-

-----------SEGYEFIMLQF-----AANTIHFTPLLNYQVHKENAPECLNITMFTECS-DKGIILMRG

EY-DTKVINAQEAQCLANQVQLYYC------QKNVSKLHLLETFTRHPDKFKHMDIIEELNNLKI

>B0XE94|B0XE94_CULQU

PPQPMDKLGDVMKMELIQDKTAEEIRAIWLQYHIG-KEVISAAIP-GEQYDLMMERARKYPVFILPVPR-

-----------SQGYEFFLLQF-----FANTVHFTPLINYQVHKENAPECLNVTLYTELRESKALVLMRG

EY-DPKVINGQEAQCLANQLQMYYS-----QRQNPTKLAQLERFTHQPDQFKHMDVIEELNNLKI

>Q16U88|Q16U88_AEDAE

DDTAQKKLGDLMKLDLIEQRSPEEIEQIWIQYHQG-KEVICATVP-LEKYDLMMERAKKYPMFILPIPR-

-----------SQGYEFIMLQF-----FANTIHFTPLINYQVHKENAPECLNIILYTEL-REKGLVLMRG

EY-DTKVINGQEAQCLANQLQMYYS------QQSESKLGLLETFTNKPEQFKHMDVIEELNNLKI

>G6DMQ4|G6DMQ4_DANPL

ASVEDKKLNDILKLELVLDKSAAEVQNIWEEYHKE-KEVISATIP-KDSYVSIAQNMKEFPTFLFPLPR-

-----------SQGYEFIMCQS-----FGNAVHFTPLLAFQVHKENAPECLTMVHYLEL-KEKGIVLMRG

DY-DKNVLDGKEAQCLANQFQMYYS------GKDPKKVTLLELFNRSPDTFKHMDLIEELESIQL

>D3TM47|D3TM47_GLOMM

AEIPYKKLSDVMKTELIQDKNADEISKIWLEYHKT-KDVLAATLT-LEHHDALMETAKKHPIFLLPLPR-

-----------SEGFEFIMLQF-----ASNAVHFTPLLAYQVHKENAPECLTLLHYTEF-REKGIILMRG

EY-DSKVLNGQEAQCLANQLQMFYC----KADKGKQR--LLEIFTNKPREFKHKDLIKEVENIQL

>B4GZ27|B4GZ27_DROPE

AELPHKKLSDIMKLELLADKSAEELSQIWIEYHKT-KDVLAATLT-KAQYETLMGRAKEHPIFLLPLPR-

-----------SEGFEFFLLQF-----AANTVHFTPLLAYQVHHENAPECLTVVHYTEV-QDKGVVLMRG

EY-DTKVLTAQEAQCLANELQMFYY------KTDESKMKLLETFTKRPDEFKHTDLIKEVENIQL

>B4JRD4|B4JRD4_DROGR

AELPHKKLSDIMKLELIADKDAEEISQIWIEYHKT-KDVLAATLT-TAQYETLMARAKEHPIFLLPLPR-

-----------SEGFEFIMLQF-----SANTVHFTPLLAYQVHKENAPECLTVVHYTEK-QDKGLVLMRG

EY-DTKVLTAQEAQCLANELQMFYY------KTDEAKLKLLETFTKRPDEFKHMDLIREVENIQL

>B4K5G2|B4K5G2_DROMO

AEVPHKKLTDIMKMELIADKNADEISQIWIEYHKT-KEVLAATLT-TSQYQTLTERAKEHPIFLLPLPR-

-----------SEGFEFIMLQF-----AANSVHFTPLLAYQVHQENAPECLTLVHYTEM-QDKGIILMRG

EY-DTKVLTAQEAQCLANELQMFYY------KTDESKLKLLETFTKRPDEFKHMELIKEVENIQL

>B4LXR2|B4LXR2_DROVI

AELPHKKLTDIMKMELIADKNAEEISQIWIEYHKT-KDVLAATLT-KAQYETLIGRAKEHPIFLLPLPR-

-----------SEGFEFILMQF-----AANSVHFTPLLAYQVHHENAPECLTLVHYTEM-QDKGIILMRG

EY-DTKVLTAQEAQCLANELQMFYY------KTDENKLKLLETFTKRPDEFKHMDLIKEVENIQL

>B4NF42|B4NF42_DROWI

AELPHKKLTDIMKLELLADKNCEEISQIWLEYHKT-KDVLAATLT-PAQYNTLMERAKEHPIFLLPLPR-

-----------SEGFEFIMLQF-----AANTVHFTPLLAYQVHQENAPECLTLVHYTEM-QDKGIVLLRG

EY-DNNVLTAQEAQCLANELQMFYY------KTDENKLKLLDTFTKRPDEFKHMDLIKEVENIQL

>B3M2L5|B3M2L5_DROAN

AELPHKKLTDIMKLELIADKSAEEISQIWLEYHKT-KEVLAATLT-TAQYETLMARAKEYPVFLLPLPR-

-----------SEGFEFIMLQF-----AANTVHFTPLLAYQVHHENAPECLTLVHYTEE-QDKGVVLMRG

EY-DSKVLTAQEAQCLANELQMFYL------KADENKLRLLETFTRKPDEFKHMDLIKEVENIQL

>B4PMY3|B4PMY3_DROYA

AELPHKKLTDIMKLELIEDKTAEEVSRIWLEYHKT-KEVLAATLT-TAQYESLMARAKEHPVFLLPLPR-

-----------SEGFEFVMLQF-----AANTVHFTPLLAYQVHHENAPECLTLVHYTEV-QDKGVVLMRG

EY-DTKVLTAQEAQCLANELQMFYL------KPDEGKLRLLNTFTRKPDEFKHMDLITEVENIQL

>O16050|O16050_DROME

AELPHKKLTDIMKLELIEDKTAEEVSQIWLEYHKT-KEVLAATLS-TSQYENLMARAKEHPVFLLPLPR-

-----------SEGFEFVMLQF-----AANTVHFTPLLAYQVHHENAPECLTVVHYTEV-QDKGVVLMRG

EY-DTKVLTAQEAQCLANELQMFYL------KPDEGRLRLLNTFTRKPDEFKHMDLIKEVENIQL

>E0VNJ2|E0VNJ2_PEDHC

PFTKTKSLNDIMKVDLLMDKTPEEIKHIWIDYHRS-KDVVTSVVP-KDTYLVMHERGKEFSTFLLPLPR-

-----------SQGYEFIVCQF-----LAHEVHFTPLIAYQTHKENAPECLTLTFYPDLVNEKGIVLMKG

EF-NTDILNCQEAQCLVNELQLYYA------QNNEKRLNLLKKFTYSQDEFDHMDLIKELETLSL

>D6WXK4|D6WXK4_TRICA

SEA---PLDKVMKLDLIKDKTPEEIKEIWHQYHIQ-KNYIAAVVP-AEDYNELEARGRQYPTFLFALPR-

-----------KEGYEFIMSQF-----ERNCVHFTPLLYYQVHKENAPECLTMTHYTELKDEKKIVLMRG

EY-DKNVIDLKEAQCLANQLQLYYV------RPTEAHLELMERFTKRPDEFKHMDLIKQIETLSL

>F4X4A6|F4X4A6_ACREC

------CLDSIMKVDMIKNKSKEEIIKIWNEYHKQ-KDCICGTMT-PEQYDKMFARGKQYSTFLLPLPR-

-----------EQGYEFIMCQF-----YGSEVHMTPLLWYQTHKENAPECLTMIHYTELRENKGIILMRG

EF-DTKLLQVQEAQCLANELQLYYC------NDHEHRLQLLETFTNRPDEFKHMDLIAQLETISL

>E2A2Y8|E2A2Y8_CAMFO

QAQEAR-LDNIMKVDMIKDKTKEEIIEIWKEYHKQ-KDCICGTMT-PEQYDMMFTRGKEYSTFLLPLPR-

-----------EQGYEFIMCQF-----YGSEVHMTPLLWYQTHKENAPECLTMIHYTELRDDKGIILMRG

EF-DTKLLQVQEAQCLANELQLYYC------NDNKHRLQLLETFTQKPNEFKHMDLIAQLETISL

>E2BH24|E2BH24_HARSA

RTKGAQ-LSNIMKVDMIRDKSTEEIMEIWKEYHKQ-KDCICGTMT-SEQYDKMFARGKQYSTFLLPLPR-

-----------EHGYEFIMCQF-----YGSEVHMTPLLWYQTHKENALECLTMIHYTELRDDKGIVLMRG

EF-DTKLLSVQEAQCLANELQLYYC------TDNEHRLQLLDMFTHKPNEFKHMDLIAQLETISL

>C5KUH7|C5KUH7_PERM5

TMPLPKKLNEIVRLPLFEREDPVKIRSMWLEHINE-KSRIGAVMA-KPEWDIFHKNALACPMFIIPVQK-

-----------PEGYFNMVSQIQ----DGKYCLMTFLDHFRSNPTDAHPFMVMNFYDDLLKSKGLTLLRA

DLVAPD-LSKSEGEAVVRMLREFYG--------QPTLFKWVETFNLRPREFDFGEFTR-ANDFGK

>D2VLC9|D2VLC9_NAEGR

VTPGPKSLDQILKVPLVERESPEAITRIWREHHEMNPHILAEVVP-YSQYQVLKHRSKECPMFVLPVFK-

-----------NIGFQTVVLQF-----QTEHVLFTAMRDFKQKGEWASPQMSLSHYTEFAQNKGIVLMRG

ET-NDTHINKAEGRDLARFMYKFYL--------DDNLYHFVEVFNKEPHRFNFEELINAVKAIKP

>C1MPB1|C1MPB1_MICPS

LIPPGKTLGEVTHVDLLEKENADVVVKIWREYHEGKSGKTGAIVE-PRVYDALSDRSKVCPMFVMPLHKS

-----------MSQYLTLVVQS-----KMPYVSFTAIEDFRKLQDAATPMLVASHYPELAASKGLALVQA

VHVDKTHLTTQEAVRLVRLCHTFYA--------EDDLYEFVKPFNHNQKDFDFDAMVLKVRDLTW

>B6A928|B6A928_CRYMR

LTPIPKYLKDIAKIPLLEREDSTCIKAIWKERFQYSRNIIVDTIN-SETYTNIYLAAKVNPMFIIPPSEK

-----------HNSFHWMIFQCQEY--PLKHILITYIKEFNN--GNFTPFIIFNWFNELQKSKSLVLLKL

DIIHP-GISKLQGSFIFNYILRCYQ--------DPKIFQFVKIFNQSPREFNINQFVEVSRNISN

>Q4UFK8|Q4UFK8_THEAN

------KLNDIMKLQLLKKLSSKDIIEIWKSKNNE---KIYSTVMNSEKYMKIKTNSKICKNFIIPIKF-

-----------NENYYNIFLQF-----NEKIILFTSLDYIHKFGKNTNPIIYMTFYDELVKTHDLILLRT

NILNNH-LNKLQVINLIENTLKFYL--------DFNLFQWVKLFNLNPKQFNYKQYLNQNKHIFN

>F0VD12|F0VD12_NEOCL

SYPSPKKLSDVVKLPLLRLKSREEVAKIWAEHFRTKTLSVAASTG-HDAFERLASNAQVAPHFVVPLPRG

EG---------GASFETFFIQFQ----GSRTCLITSLHEYTQNPDRPSPFLVVTFFDELGEEKNLTLVQG

DILRGECLSKEEAGHVLSLLLLFYS------DPNLSR--WVLDFNLKPREFSFELFQDEQRRLQV

>A8IGB8|A8IGB8_CHLRE

SAPSPTQLGQVVKLDELIKKDRDEVADIWLGYHADEKGGVGSVLS-ADDYKTFLSRAKESPMFVFAMPKP

HKG--------KGAYEAMLIQC-----QMPYVLITGLEEFKRHGEGAPPYLTLTHYPELLDSHGLALVRG

DIIHEKGITRDEARTALELTRAFYC--------GDEDYALVHTFNKKPASFDFGAVLRKLNLA--

>E1ZMF8|E1ZMF8_CHLVA

SLPSSKNLWDVVNRQRMEPHGAEAVRDIWMEFHADAKNRIATGLT-APRYLKFQENAAQSPIFVLPVFKG

-----------PNAFENFMVQC-----QLPLVLFTTLEEYKQRGPSAQPHMVLTHYTELMSDKNIVLVRG

DILQPTICSRGEAEQLTRLLHDFYT--------QDQKYSFVHAFNHRQADFDYKRMLDSMGHRD-

>Q8LCY7|Q8LCY7_ARATH

TPLQQKPLDSIMDLARAKTKSPEELTSIWDDYHLG-RGHIGLTMK-AQLYRLLEQRASECRYFVIPSWR-

-----------GNGYITMFAQV-----EAPHMIFTGLEDYKARGTQAAPYLTTTFYTELSETKDLVFIRG

DVVFTSKLTDEEAKWIMETAQSFYL--------NDSRYKLLERFNKHTHDFEFKDVLQALDMPLL

>F2DAN7|F2DAN7_HORVD

PLEPKK-LGSILDVERAKGLSPEHLVAAWDDYHLG-RGHIGVSMK-AKLYRLLEQRSATCPYFVIPLWR-

-----------GSGYTTMFMQV-----QLPHMIFTGLEDYKARGTQASPYCTVTHFTEFAETKDTVLVRG

DVVFTSKLTDAEAKCLLETAHSFYL--------NDVRYKLVERFNKETRDFEFKDVLQALEMPSM

>B8AG84|B8AG84_ORYSI

TPLQPKPLASILDVERASGLSPDHLVAAWDDYHLG-RGHIGASMS-AKLYHLMEQRSATCRHFVIPLWK-

-----------GTGYTTMFMQV-----QMPHMIFTGLEDYKARGTQASPYYTITHYTEFAETKDTVLIRG

DVVFTSKLTDSEAKCLLESAHSFYL--------NDVRYRLVERFNKEPHEFEFKDVLQVLEMPTM

>C5XZN6|C5XZN6_SORBI

PLKP-KPLGSILDIERAKGLSPEHLVAAWDDYHLG-RGHIGASMK-AKLYHLLEQRSDSCRHFVIPLWK-

-----------GSGYTTMFMQV-----QMPYIIFTGLEDYKARGTQASPYYTVTHYTEFSETKDTVLIRG

DVVFTSKLTDSEAKTLLETAHSFYL--------NDVRYRLVERFNKETHDFEFRDVLQVLDMPTM

>B6TQ44|B6TQ44_MAIZE

P-LKPKPLGSILDIERAKGLSPDHLVAAWDDYHLG-RGHIGASMK-AKLYHLLEQRSASCRHFVIPLWK-

-----------GSGYTTMFMQV-----QMPYMVFTGLEDYKARGTQASPYYTVTHYTEFAETKDTVLIRG

DVVFTSKLTDSEAKTLLETAHSFYL--------NDVRYRLVERFNKETHDFEFGDVLKVLDMPTM

>B9H2M6|B9H2M6_POPTR

TPLEPKPLDSIMDIERAKTKSPEDLASIWDDYHLG-RGHIGASMK-AKLYQLLVQRAADCKYFVIPLWR-

-----------GSGYTTMFAQV-----QMPHMIFTGLEDYKSRGTQASPYLTVKFYTEFAESKDLVLIRG

DIVFTSKLTDEEAEWILETAQSFYL--------NDVRFKLVEQFNKQTRDFEFKDVLRSLNMPIM

>D7TJH1|D7TJH1_VITVI

TPLQPKPLGSILDIERVKDRSSEDIASIWDDYHLG-RGHIAASMK-PKLYHLLEHRAANCRHFVIPLWR-

-----------GSGYATMFAQV-----QMQHMIFTGLEDYKARGTQAAPYFTVTFYTDFAESKDLVLIRG

DIVFTSKLSDSEAKWLLETAQSFYL--------NDVRYKLVERFNKETREFEFKDVLQALDMPVL

>A9RXB1|A9RXB1_PHYPA

TALAPKKLEQIMKIESVIFSPPEEITQIWNDYHIG-RGHISAVMG-SELYKIFEQRANECPIFVLPLRK-

-----------GNGFISVVVQA-----QMPYLLFTALEDYRVRGSEAAPYFTVTHFTELVPTKSLVLVRG

DIVFTRKLSDDEADTLLKTAHSFYI--------NDERYRKVRKFNKDSREFDFKEVLQELNIP--

>D8SCE2|D8SCE2_SELML

TLITPKSLNSILKVESVKNKSADEVTFIWNQYHIG-RGHVSAVMS-TDFFKRFQERARECPNFVLPLRKP

------------KGYISFFLQA-----QMPFLLLTSLEDYKLKGSNAAPYLTIAHYTDFADSKGIVLVRG

DIVFPSQLSDDEGNSLLKYAHSFYI--------RDDRYLLVKAFTHNSEDFNFKDVVRELDYDYV

>A0DDW3|A0DDW3_PARTE

TYPCPRKLREIVKMSLFERESKDQVVSLWMEYHKDKQNNVAYVVS-KDEHDILKRNTKESPLFLLPIKR-

-----------KGGHFQLIGQA-----QTNSILFTFLEEYKKSGSFSSPYFILTIFEELLAQKQVSLIRG

DIMDYK-IDKDEATFLTNQFLKFYM--------TPELYEYIYTLNHKQQEFNYDDFKNHFQI---

>A0E8S3|A0E8S3_PARTE

TYPCPRKLREIVKMSLFERESKDQVISLWMEYHKEKQNNVAYVVS-KDEYEILKRNTKESPLFLLPIKR-

-----------KGGHFQLIGQS-----QTNSILFTFLEEYKKSGSFSSPYFILTIFEELLAQKQVALIRG

DIMDYK-IDKDEATFLTNQFLKFYM--------TPELYEYIYTLNHKQQEFNYDDFKNHFQI---

>B8BUG9|B8BUG9_THAPS

SYAGPRKLQDILKTELIEGKTKSEIQDLWLTYHEGKEKIHGLSLD-GAKAKTVLSRAAQCPFFIHPVFR-

-----------DEGHFMIVSQFQ----APNHFLLAFLEDYQMDPSRAQPLLTVSVFDDLADSKDLALVRC

DI-INQGIEEDEGYKLCQCLLGDYS------DEDDFR--LVHLFNKKPDAFNVDEYLKEREKSWK

>F0WA42|F0WA42_9STRA

SYPAPRALNEIVKIDLLANENADKIAQIWAEYHRDKIDSLAKVIP-DKEMQLITDRASSARLSVIPVYR-

-----------EEGFLNMLCQF-----QDTCFLVTSLEAYQKAPANASPCVTFSIYNDLASEKSITLVRG

DIV---TVSKKESKSLLSSILHIYS--------VPELYEIVQ-LNQASEKFDFEAYREALDKEKD

>D0N4T2|D0N4T2_PHYIT

SYPGARSLEQIVKMELLESEQAPKIRNIWEEFHADKDDAVATTLD-VSEFQSLVKRAEAAPYFIFPVYR-

-----------QEGFFNMLCQF-----QQSCFLITYLEAFKENPSAAPPCVAISLYDDLLTKKELALVRA

DVI---MLDKKESQLLLKQLLASYQ--------DDQLYDHVDKFNNKPDHFDFEAYRVMLQEATT

>G5A2K1|G5A2K1_PHYSP

SYPGARSLEQIVKLELLENEQAPQIRSIWEEFHADKDDAVATTLG-ADEFQALVKRAEAAPYFIFPVYR-

-----------QEGFFNMLCQF-----QQSCFLVTYLEAFKENPSAAPPCVAVSLYEDLLAKKELTLVRA

DVI---MLDKKESQLLLQQLLTSYQ--------DDKLYEHVDKFNNKPEQFDFETYRSMLKDVIE

>B3LB85|B3LB85_PLAKH

SLPSSRALKNIVKLPLLEREDKQKIIHIWKEKYQNDKYVVADHIS-ISKYEQIKNNCKNNSHFIIPQRN-

-----------QNGYINFYSQFI----DNKLLFITTLGDYNKFREKSTPYVTLHFFDEL-KNREIILTKL

NI-VNNVITKNQAIKFYNYILAFYS--------DANYFTYVCKFNNDSRNFHYDAFIEKFKHMF-

>Q8I5V1|Q8I5V1_PLAF7

SLPCSRDLKNIVKLPLLEREDKYKIINIWKEKYKDNKYVISDYMD-INKYEVIKNNCKNNSHFIIPFKN-

-----------NNGYITYYTQFI----DSKLIFVTSLEYYNKHKSNSTPFITLHFFDEF-KNKEIILSKI

HIINPA-ISKYQAIKIYNNILSFYY--------DTNYFQYVKKFNNDSRNFNYDKFFGKFKEIF-

>D8LNM0|D8LNM0_ECTSI

SFPGPRKLEDITNLPLLAKEEKESIADIWTAYHDEREDSLGTVIP-GDSLDGLQAKAKKCPMFVLPVWR-

-----------DGGHFMMLSQY-----QDKCFLLTYLEDYKVNPGGAQPYATISMYNDLVDSKGLGLIRA

DITP--NLTKKEVDRLVRLLIRFYS-------PHAHH--HVEAFNLRPQEFDLDKLLQTPRD---
