# Supplementary material for: Accurate Simulation and Detection of Coevolution Signals in Multiple Sequence Alignments
Source: PLoS One. 2012 Oct 16;7(10):e47108. doi: 10.1371/journal.pone.0047108 (PMC3473043; doi:10.1371/journal.pone.0047108)
Supplement: MSA S8 — MSA of ATP12. (DOCX) [file pone.0047108.s019.docx]

>A0NWE0|A0NWE0_9RHOB

SRRELPKRFYKDVTHVPADGGFAIHLDGRPVKTPGKAPLLLPSEALAKAVAAEWQAQEKEINPASMPLTR

ISNSALDAVSVRFDEVADDITRFAGNDALCYRADTPQSLVDTQNRLWDPVVDWAGGLLGGRFVLIEGVMH

AAQPEPLLVAYRARL-SNTPMRLAAFHTITSLTGSALLALGLMEDYLDADAAWTAAHVEEDFNIERWGED

AEAALVRAYKRKEFDAAVLILM--EG

>A1B060|A1B060_PARDP

MSEWKARRFWASVGIHKEEGGWAVLLDERPLRTPGKQPLRLPTEALALAIAEEWQAVQEVIDPNAMPLTR

SANSAIEKVAPQFDAVAAMLGDYGGTDLLSYRADAPEALVRAQAEGWDPLIDWAATELRAPLRITHGVIP

VPQDPVVLLKLRAEVASLDPFGLTALHDLVTLPGSLILGLAVIRGRIDAPTAHALSRIDEEFQAERWGRD

EEAEAQAASRLAAMRDSERFWHLTRG

>A1USS7|A1USS7_BARBK

SHQLLMKRFYKDVSIVREERGVSILLDGRPITTPAKRHIFVPTEALAALVAQEFKIQEKVIDPAKMPITR

LINTVIDGIADNMQVIFEDLLRFVACDMIFYRAQTPKELAKRQCEHWDFLLDWAEEKIGARFNIAEGVMH

IEQPWESIHAVSNYLRKISPYVLAALHTMTTLTGSALIAFAVAEKKIDLDHAWSIAHLDEDWTIEQWKVD

EEAMIRRAYKKVEFDAAVTMMTAAHS

>A3JN54|A3JN54_9RHOB

MSEWAAKRFWKTVEISENDQGFGITLDGRSVKTPLKTALTVPSKSLAERVAREWEAVEEKIDPREMPFTR

SVNAALDKVSTQHSEVANLIADYADSDLLCYRADTPAELVQRQDDAWEPLLDWIKSKHGVEFTVNTGIMH

RAQPAKTVAYLRDWTHSLNNFQLTGFHDLVSLSGSFILGMSAAERAFEPAKIWAFSRVDENWQSEQWGND

DEADSVAALKQRSFLHACEFFQLIEQ

>A3JXI9|A3JXI9_9RHOB

MSEWALKRFWKDATVEPSGPGFAVKLDGRGVKTPAKTPLVVPTEGLADAVAAEWRAQEEHVNPMTMPFTR

MSNSALDKVATQRAEVADMLAAYGDSDLLCYRADRPAGLVERQNESWDPLLDWASETYGARLEPRIGVMH

HPQPSEALARLAQEVHGQDAFQLAAFHDLVSMSGSLVLALAVIRGHREAEDAWRLSRIDETWQEEQWGID

EEAAEIAALKRNEFMHAARFHMLSSV

>A3PGP2|A3PGP2_RHOS1

MAGWGAKRFWKEASVAEEPGGFAVLLDGRGVRTPAKRPLILPTCALAEAVASEWQAQEGEVRPETMPVTR

SANSALDKVAPQFDEVTEMLAAYGGTDLLCYRATAPEALVARQARAWDPVLAWAAERFEAPLETTAGVMH

QAQPEASLTRLAEHVRGFSPFQVAGFHDLVAISGSLILGLGVTEGHLLPEEAWELSRLDESWQIEQWGAD

EEAAEIEAFRRTAFLQAARFYALC-G

>A3S821|A3S821_9RHOB

MSDWKAKRFWKEAAVVEAEGGYTVELDGRGIKTPAKRALVVPTRAMAQKIAAEWQAQDGVINPDTMPATK

TANAAIDKVALQHAEVADMLAAYGDCDLLCYRADSPAELVARQADQWDPMLEWAREALGVSLQTRVGIMH

EPQSPADVARLSARTHALSNFQLAAFHDLVSLSGSLILGFAAALDAKEAESIWEISRLDEIWQAEQWGND

DEADALAAVKKTSFLHAKLMFDLSPE

>A3SK08|A3SK08_9RHOB

MSEWKARRFWKDSSVESAEDGFTVHLDGRPVRTPAKRSLILPTEALAQEVAAEWQAQDEEINPLSMPFTR

SANAAIDKVAVQKDEVAEMIAAYGDSDLLCYRADSPTELVERQSAAWDPYLDWAESDLSARLVPVAGVVH

QPQNPAAVAALRAEVMAQDIYALTALHDLVSLSGSLVLGLAAQRGIAPPETLWTLSRLDEDWQIEQWGED

EEASELAATKQRAFLHAARLYQLSRR

>A3TU37|A3TU37_9RHOB

MSEWAPKRFWKTVAVTESEGGFGVALDGRPVRTPAKRPLVVPTRALAEAVAAEWEAQEDVLDPRTMPATR

GANAAIDKVALQHAEVAGMLAAYGDSDLLCYRADSPAGLVARQAEGWDPLLDWAEEALGARLMPRAGVMH

EPQNPEALNRLSAAVHALGPFQLAAFHDLVSLTGSLVLGFAAAREVRPVEEIWDLSRIDEDWQAEQWGID

DEAAAAAAAKRASFLQAKRFFDSVAA

>A3UD61|A3UD61_9RHOB

ENRELPKRFYKEVSTQPGEGGWSILLDGRPVKTPAKRALHVPSETLATALAAEWAAQETVIDPFTMPITR

ILHVALDRMEAVREGAAEEVANFGRTDLLSHRAEESQ-LAARQAELWDPYLEWAKTALDAPLNAAATVLA

LEQPESSIAALKARA-LADDLRLTALVSVTPILGSAILAFALLEGEADAEAVWKAARVDDDYQIERWGED

AEAALAAANRKRDLIASETVMRALGL

>A3V4J6|A3V4J6_9RHOB

MSDWKPKRFWKAATPQACDGGFTVTLDGRPVKTPAKAALIVPTLPLAEAVAQEWDAQTGLVDPRTMPVTR

SANAAIDKVRTQRAEVIGLLSEYGGSDLLCYRAPAPDGLVMRQRQVWDPLLDWAARDLGVTLTIGEGVVP

VPQPAESLDILQRELDYVDDFGLAAAHDLISLSGSLILAFAVMRRHLDAARAWDVSRVDEDWQTSQWGVD

EDAAAAAAVKRAAFLHAERFYHLSWH

>A3VF97|A3VF97_9RHOB

MSDWAPKRFWKGTRADACDGGYTVYLDTRTVRTPAKAAFVVPSRALAEAIAVEWDAQTDKIDPSTMPMTR

TANSAIDKVRLQHAEVADLLAAYGDSDLTCYRADTPAELVARQAEAWDPLLDWADKTFGARLEPRIGIMH

APQDPGALTRLGALVHDLDPFRLAAFHDLVSLSGSLIIALAVMHGRIDPETGWAISRIDENWQAELWGED

EEATEHAALKRQAFLDAALFFRLANS

>A3VN51|A3VN51_9PROT

--EAGPKRFYETVTVAA-DGVYVIKLDGRPAKTPGRHLLGAESQILAEALAAEWSQQQDIIDLTTMPLTR

LTGFARDGGEDARGPWRQTILAYAGSDLLCYRAPE-TGLAARQAEIWDPFLARFAERVGAPLTVTEGIIA

VEQDPKSLAAIEAYLDPYSLERLYAAKLLTEMTGSAVLALAALDEDAPAHEIFAASRLDETFQAERWGID

AEAAIREASLRRDFEDTLRFASLT-P

>A3VYV4|A3VYV4_9RHOB

MSEWKAKRFWKEAGVVEDDAGFGVRLDGRVVRTPAKATLIVPTRALAEAIAAEWDAQDGKIDPNTMPCTR

SANAALDKVAPQRDEVVEMLAAYGDSDLICYRAASPEELIKKQAEAWDPLLDWTESVLSVKLLPVVGVVH

VPQDAQAISLLKAHVEALDTWALTAFHDLVSMSGSLVIGFAALDDLYPADALWDLSRVDETWQAEQWGKD

EEAEEMAARKQSDFLHAKRFYDLSAI

>A3WAF3|A3WAF3_9SPHN

-----MKRFYKEVDIQPALGGWQVTLDGRAIRTQKGAGQIVPTEALARALAAEWDAQGEKIDPATLPLRD

MADYALDVIAPDPAAVADKVLTYGDTDTLLYRADPDEPLYVRQQELWEPITKRFEERHGIELVRVSGIVH

RPQNEATLAKLRDALGKQTPFALAGMEAMTSLAASLMVALEASEPGADALALWQAASLEEEWQAELWGRD

EEAEERRAKRESDFLRAREFVRLASD

>A3WVV2|A3WVV2_9BRAD

ARSGQRQRFYACASVKETPEGFAILLDDKPVRTPSRNVLAAPARAIAEAIAVEWDAQRDVINPMTMPLTR

LANSVIDGVAGRVDVVVEDIAKYLETDLLFYRAGHPDGLVARESVHWDPVLLWAAEALEARFILSEGIVH

VCQPDQAVAAARKAL-P-DPWMVGALHVVTTLTGSALLAIALMRGRLDAGEVWAAAHVDEDWNSEQWGVD

EEAAVRRASRLVDFQAAATVLRARTG

>A3X423|A3X423_9RHOB

MSEWKQKRFWKEAKAVEVEGGFSVHLDGRGLKTPAKTSLILPSRAMAEAIAKEWDAQVEGINPQTMPYTR

SANAAIDKVANQHGEVADMLADYGDSDLLCYRAESPQELVKRQADHWDPALSWAEEVLGARLETRHGLLH

SGQDPAALKVLRQAVHALDNFRLAAFQDLVSMSGSLVLGFATAQNWRTADDIWQISRLDETWQEEQWGVD

EEAQEAAGLKRVAFLHAKSFYDI--C

>A4EN88|A4EN88_9RHOB

MSDWQPKRFWKQAQAEVCEGGFTVKLDGRLVRTPAKAALTVPTLAMADAIATEWDAQEELIDPRTMPVTR

GANAAIDKVRTQRSEVIALLAEYGDSDLLCYRAAGPEGLIQRQAEGWDPMLDWAADTLGARLFVGEGVMH

VTQKPDVLSKLTTEVATFDDFALAGVHDLISLSGSLILALAITKDAIAVEDAWLLSRIDEHWQIEQWGAD

EEAAAAELTKKTAFQDAARFYQLSLT

>A4EPD7|A4EPD7_9RHOB

MSEWKQKRFWKEVSVVEEGDGFAITLDGRKVKTPAKAALVVPSRAMAEAIAAEWDAQEEGINPETMPCTK

SANAAIDKVANQHEEVANMLADYGDSDLLCYRAEAPQELVMRQNETWDPILDWAENALNARLEPRSGLMH

QPQKPEALAKLRASVHAMTPFQLAAFHDLVSMSGSLVLGFAAAKDCRSPEEIWRISRLDEAWQEEQWGVD

EEATEVAAIKEQAFLHAKRFFDF--S

>A4U0K1|A4U0K1_9PROT

-QGKTMKRFHKQAGIVA-DGGFAVQLDGRGVKTPVGRKLEVPSERLALAIAGEWDAQGEVIKPYTMPLTQ

LATTALDRVGPERAVITDQMIAYAATDLLCYRAESPSDLVAVQTKTWQPLLDWCRTQLDAGLIVTTGVIA

IDQPAASLAALRARLDGYDLWRLTAAQAACSASGSLVLALALTEGRLTGAECFTASNLDEAYQVAQWGDD

YEAADRRAELQRDIQAAADLLALL-P

>A4WVH8|A4WVH8_RHOS5

MAGWVAKRFWKGVSVAEDAGGFAVLLDGRALRTPAKRPLILPTGPLAEAVADEWRAQEGEVRPGTMPFTR

SANSALDKVEPQFDEVAGMLAAYGGTDLLCYRAPAPAALTARQAEAWDPILAWAAEAFGAPLEPTVGVMH

RPQPEESLRRLAERVGALSPFQVAAFHDLVAISNSLVLAFGVTEGRLLAEEAWELSRIDETWQVEQWGED

EEAAEIEAARRAAFLQAARFYALC-G

>A4YWT6|A4YWT6_BRASO

MRAPQRKRFYKEAGTAEAEGGHYVTLDGRPIRTPSGRVVVVPVRELAEAVAAEWGAQGETIDPASMPLTR

FANSVVQSVVDRVEDVRADMARYLQSDLLFYRAGHPEGLVEREAAHWDPVLDWARDSLGAHFILSEGIMH

VTQPDAAVRAAREVL-P-GPWAVAAAHVVTTVTGSALLALALTHGVRDADQVWAAANVDEDWNIEQWGAD

EEAVHRRAAKQVDYQAAVRILRAVAI

>A5ELI9|A5ELI9_BRASB

MRAPQRKRFYKDVGTAAVDGGYHVTLDGRPIRTPSGRVVVVPVQALAEAVAAEWQAQGEIIDPATMPLTR

FANSVVQSVIDRTDDVREDMAKYLQSDLLFYRAGHPEGLVAREATHWDPVLDWARDTLGAHFILSEGIMH

VTQPDAAVQAARNAL-P-GPWAVAAAHVVTTITGSALLALALAQRVRDADQVWAAANVDEDCNVEQWGAD

EEAMRRRAAKEIDYQAAVRILQDVAS

>A5G1Z0|A5G1Z0_ACICJ

-----MKRIWSAVAVQA-ETGFGITLDGRPVRLPGGAPLAVPAEALARAIAAEWDAPDKDIRPDDLPLTR

LAGTAVERIAPDTAPVRAGLLAYGRSDLLCYRATGPDGLVAEQARLWQPWLDWAGATLGARLAVTAGITA

IDQPPEAIAGLDAALATHDAWSLAGLGVTVPALGSLVLGLAVAAGAIAPAAAHDCATLDERWQERLWGED

AEAAARRARIGGEIASAARFVNLARS

>A5PEL8|A5PEL8_9SPHN

-QPRAMKRFYKDVSTAKTDLGWTVSLDGRPIKTQGGQPQVVPSEALAEKLAAEWSGQGETIDPAAFRFRD

MTDYALDVVARDRDSLVEKLLGYAETDTLCFRADPEEALYRRQQEVWEPILEGMEAREGVKLHRISGILH

RPQIEGTRARLAARLEGLDPFTLTALEQVTLLAASLCIGLEALQPGADGEALWDAANLEEDWQADLWGRD

EEAEERRAKRKGDFLAAMEFHRAAAG

>A5V9V2|A5V9V2_SPHWW

-----MKRFYKQVSVEPVAGGHAIRLDGRPVKTPARADLTLPTSALAHAVAAEWDAQAEEIDPRRMPLTG

LANAAIDRIAPDPAAFARGLAAYAETDLLCYRADSPAKLVDRQAASWDPLLDWARDRYDVHFETVAGIIH

RPQPDETVQRLAAAVAAHDAFHLAALQPLVTITGSLVIALALAGGRIDAEQAFAAAHLDELWQAEQWGED

ALATEARENRRADFKAAARLLGLL-V

>A6E223|A6E223_9RHOB

MSEWKAKRFWTEAGVVEEGDGFGVRLDGRSVRTPAKAVLSVPTRALAEAIAAEWDAQEGKIDPGTMPFTR

SANAAIDKVAHQKSEVAEMLAAYGDSDLICYRAVSPSELVEKQAAAWNPLLDWTESVLSAKLLAVEGVVH

VPQDVQAMARLRAHVDALDIWALTAFHDLVSLSGSLVIGFAALDGFYPVKTLWDLSRVDETWQAEQWGSD

EEAEEMAARKQSDFIHAKRFYDLSSV

>A6FLR2|A6FLR2_9RHOB

MSEWKAKRFWKTAEVDARDDGFAVRLDGRPVKTPAKAPLVVPTQDMARAIAAEWDAQEDVINPHSMPVTR

AANAAIDKVRHQHSEVAQMLADYGDADLLCYRAASPVELIRRQSEAWDPLLDWAEATFGVRLRTVTGVMH

APQDEAALAALTGPVHKMDAFTLTAFHDLVSLSGSLVIGFAALTGHRPPEALWQLSRVDEQWQAELWGVD

EQAAEQAALKESDFLAAKRFHDLSRD

>A6U890|A6U890_SINMW

MQKPLPKRFYKQASAAPADGGYAVLLDGRPVRTPAKRPFAVPSEKLAQLLAAEWDAQADVIDPSAMPLTR

LVNTAIDGVALEERAVFDDILRFAGSDLLCYRADSPKELVARQNDQWNPVLDWAARTLGARFILVEGVIH

QEQPAEAISAFAGGLNAFTPLGLACLHTVTSLTGSALLSLALAMGRLSAEEVWTAAHVDEDWQIEHWGTD

EEAFRRREIRWQEMLAAAVVLDAL-K

>A6X0P8|A6X0P8_OCHA4

MLAQLPKRFYEKAEVAE-EGGFTVHLDGRPVKTPARNLLLLPTKAAAQIVADEFTAQEKVIDPGKMPATR

LVNTAIDGIAQDPQAVFEDILRFAGTDMLCYRADSPQELVSRQTEQWDPLIDWMEG-LGARFSLAEGVMH

IEQPREAIAAFGVHLAGFDPIALASLHTMTTLTGSAIIALAIAKDEITAEQGWNLAHIDEDWTIEHWGSD

AEAIERRKNREIEMMMAARVLKA--L

>A7HWU5|A7HWU5_PARL1

EKRRVLKRFYKKAEAGP-EKGHAILLDGRAVKTPAKEPLAVPVLALARAIADEWEAQAEEIDPRAMKLTK

LANTAIDLVAPRREAVIAELVNFAATDLLCYRADAPAALAARQAAAWEPLLAWAAG-QGIRLRVTTGLMH

VPQDEAALDAYGASVAALDPFRIAGLHNAVTLTGSAIIGLAVALGHIGPEAAFETAHIDETWQMEICGED

DEELARLAARRAELLETARFLALMDA

>A7IPN4|A7IPN4_XANP2

QRTPLPKRFYTDVSVGEADGGFTILLDGRPVRTPARGLLLAPTRPLAEAMAAEWAEQEKEINPFFMPLTR

LVNVALDRVGPEAEAVREEVVRYAGSDMLFYRADSPQTLVKRQAEQWDPVLDWLSGAHDARFFLSEGIRH

VTQPDTSLERVRALV--PAPLKLAAVHSITTLTGSALLALAVAEGALDADAAWAAAHVDEDFNREQWGED

EIATARRAARRTEMDAAARLLTLI-T

>A8IAK5|A8IAK5_AZOC5

GRRTLPKRFYKEVTVAEGEGGHEVRLDGRTLRTPAKAKLAVPSAALAAALAEEWAAQGVEIDPFTMPLTR

LVNVALDGVARNPQEVKEEIVRYMGSDLLLYRAEGPEGLVARQATHWDPVLAWLDRAHDARFFLAEGIRH

VEQPADMVARVAALV-GTDPLSLAALSTLTALTGSAFLAVALANGVLDADAAWRAAHVDEDWTIERWGED

AEATARRAAREKEMRAAVRLLDLVKG

>A8LM89|A8LM89_DINSH

MSGWAAKRFWKETDIVEAGTGFEVRLDGRSVRTPLKTLLVVPSRGFAERIAAEWDAQDETVNPQSMPFTR

AANAALDKVTPQHAEVAEMLSAYGGTDLLCYRATGPDTLCARQAESWDPLLDWAAERYGARLRVTAGVLP

VDQDPDSLARLSQAVAAFTPFQLTGFHDLVAISGSLVLGLAVAEGRMSAEAGFAASRIDEEWQISQWGED

EEEAERIAVKRADYLRAKEIFDL--S

>A8TZH0|A8TZH0_9PROT

----SMKRIYKTVAVAT-DAGFTVLLDSRPVGSPGQRPIILPGRVLADAIAAEWDGQGETIDVYSMPMMG

FAATVIDRVAPQRDYVVGEVAGYGGSDLLCYLADDPPVLTARQETAWSPLRGWAEATFGARLLPTVGVMP

VAQSPDSLAALRRTVEAVNDWELAALHTLTAITGSLVLGLAVLHDRLDAEAAYTVSEIDEAYQVERWGTD

REAEQRRRIRRAEVAEAAKFVELLAG

>A9CJ14|A9CJ14_AGRT5

MQKPLPKRFYKDVTVADVEGGFTILLDGKPLRTPAKKPLVAPSRALADLLRDEWDAQKEVVNPVVMPVSR

HVNTAIDGIASDTQAVFEDILRFSSSDLLCYRAGDPEALVARQTDYWDPVLDWATNVLGARFILVEGVMH

RDQPREAIAAFAVTLKKYTPIALAALHTMTSLTGSAILALALAEGELTLEEAWALAHLDEDWTAEQWGED

EEALERRAVRLIDMRAALNVLESLAS

>A9DCN5|A9DCN5_9RHIZ

LRKQLPKRFFTEAGVKPGGGGFVVALDGKPVRTPSRRELEVPFEALAEAVAAEWSAQKEHIDPAVMPLTR

MVNTAIDGVSTAREAVFEEILRYGGTDMLCYRAEGPDTLIAREAEHWDPYLDWAAQ-QGARLVLAEGIMH

VEQPAESIRALATLMRRHSDLQLTALHTITTLTGSLVLALALAEGHAEADEIWLAAHVDEDFNISQWGED

HEAAARKAKRLLEFEAAALILSAHWT

>A9DU60|A9DU60_9RHOB

MSDWKAKRFWKDADVVEVDGGFTVELDGRRVKTPAKRPLTLPTRAMAEAVAAEWQAQEKQIDPRTMPVTK

TANAAIDKVAVQQDEVVAMLAAYGDSDLLCYRADNPEELMARQAAEWDPMLNWAAAFLGVKLETRQGIMH

RPQSPEALAKMHRRTAELDAFELAAFHDLVSLTGSLVLGLAAAEQAFDPQEIWALSRLDENWQAEQWGRD

DVAEAEAEIKRQAFLHACAMIVLSAA

>A9GKH7|A9GKH7_9RHOB

MSEWKQKRFWKAVSVAETEDGFAVELDGRRVKTPAKAALAVPGREMAEAIAAEWDAQTESVNPNTMPVTR

SANAAINKVTHQHAAVADMLAEYGDSDLLCYRAEMPVELVQRQAEIWDPALDWAAETLGARLEPRSGILH

APQNPEALAHLRRLVHEMTPFQLAAFHDLVAMSGSLVLGFAATRSWRPADQIWEMSRLDELWQEQQWGQD

EDAQATADLKRAAFLHAKRFYDFS-C

>A9HEF6|A9HEF6_9RHOB

MSEWKAKRFWKAAAVDETAEGFGISLDGRPVKTPAKRSLIAPTRQFAERIANEWNAQGEVIDPGAMPFTR

SANAALDKVAVQHDEVADMLAAYGDSDLLCYRAEYPERLVARQSAQWDPILDWAAGVFGTRLQTRAGVMH

VPQEPEALARLTEKTRKMSAFELAGFHDLVSLSGSLILGFAATHDFLSPKAIWDISRLDEIWQAEEWGSD

EEAEAAAAVKEAAFVHAKQVFELSRD

>A9HLU9|A9HLU9_GLUDA

ARDDARKRFWDQATVET-PDGFAVLLDGRTVRLPGGTALCVRSAALATALAAEWQAAGGVFTPADLPLTR

IAGTMLERIAPDRSATVAVLAGYAGGDLLCYRVRTPALLVERQRQEWDPWLDWLRVRYGISLTVTEGVMP

IAQSEEALGAVRAVLDALSDGALAALGVAVPALGSLVLGLALAEGRLTAEQATACATLDERTQMELWGHD

AQQAERLSRLERDVRDAETFLRLVES

>A9IVU5|A9IVU5_BART1

SCQSHPKRFYKQVKIACEEGDFTILLDERPVKTPAKRPFLVPTEVFAEFIAQEFESQKHVVDPTKMPMTR

LVNTVIDGIADDMQVVFEDLLRFVACDMIFYRAQTPKELVQKQSEQWDPLLDWAEEKLGARFYLTEGLMH

VEQSPEALQAVSHYLRSVSPYMLAALHMMTTLTGSALIALAVAAGKIDADHAWSIAHLDEDWMMEQWGKD

KETMAHRAHKKAEFKAAATIVIAC-L

>B0T3E7|B0T3E7_CAUSK

DLLQRPRRFYKAATVGP-DGGFAVLLDGRTPKSPAKAPLVLPSQALADLVAGEWEAQDQVIDSTVMPATR

LAFTAIDRIRETRAEVAAEVAAYAGSDLLCYWADHPTPLVERQKRDWGGMLDWARAELDLHLQPVSGVIH

TAQSPAALASVEALALTMDDFTLAGVAYGAGLLGSTVLALALRAGKVTGRKALDLSRLEEVFQAETWGQD

AEAIARAETLAIEAQVLERWFAALSL

>B0U940|B0U940_METS4

SRPALPKRFYAEAGTAE-EDGHRLVLDGRPARTPGRRVVAVPQPAVARALAEEWGAQAEVIDPARMPLTR

LVNTALDGVAERREAVVQDIAAYAGSDLLAYRAGDPARLVASQAAAWDPVLDWARDELGALIVLSEGVMH

VAQPAGSLEAVRRAVEAVSPLALTGLHVMTTLTGSVLLALAVLRGRLTAEEAWEAAHVDETFQASVWGRD

EEAEARRALRKQDFLTAARLVALT-A

>B1LYN6|B1LYN6_METRJ

AKPALPRRFYDEAAFAE-QGGYRLTLDGRPANTPARNPLRLPSRILAERVAAEWGAQDTAIDPARMPLTR

LANTAIDGVTPRLAEVAADLCAYAGTDLLAYRAGDPERLVAAQAEAWDPILAWARDAFGARVILSEGVMH

VAQPADTIRALSEAVNAVDPFQMAGLHTLTTLTGSLLIALAVLKGRLTPAEAWAAAHVDETYQAAVWGRD

AEAEARLEARRTEFEAAAAFVGAG-D

>B1ZI49|B1ZI49_METPB

TAPTLPKRFYAKAGLAE-EGGYRLVLDGRGANTPGRRPLVVPDIGLGEALAAEWAAQETVIDPRTMPLTR

LVNTTIDGVVERRAAVAEDLGAFAGTDLVAYRAGTPERLVAEQAAAWDPLVAWAAETFGARLFLAEGVMH

VEQPEGSVAALRAAIDAVDPFRLAALHALTTLTGSLVIALAVLHRRLGADEAWAAAHVDETYQASVWGRD

AEAEARLAHRRAEFDAAATVAQRR-S

>B2IK22|B2IK22_BEII9

LKKALPKRFYREARAEP-EGGFALTLDGKPAKTPARQDLVLPTLAAAEALAAEWNAQEDIIDPSAMPLTR

LANSAIDGVRAALMETVEEIARYGGSDLVCYRAEGPDSLVAAQAAAWDPVLAFAREKLGARFLCAEGVVF

VTQPEEAEAALLKAVQFAAPFAVAALHVMTSLMGSALMALGVAHGLLRPEEAWASAHVDEDFQMRVWGRD

EEALERRTRRWRDMEAAARLFQS-QS

>B3PP11|B3PP11_RHIE6

MKKPLPKRFYAEVAVAEHEGDFAITLDGKMVRTPARQVLAVPTEALAQLVAAEWRAQGEEIDPVTMPATR

LVNTALDGVATNAQAIFEDILRFSSSDLLCYRADGPELLVERQRQRWDPVIDWAANDLGARFILIEGVMH

HEQPREATAAFAVTLARHSPMALAALHTITTLTGSAILALAFAEGRLTVEEVWSLAHLDEDWTIEHWGRD

EEAEERRAKRFVEFKAAADVFFALSD

>B3QAZ9|B3QAZ9_RHOPT

SRAQLPKRFYKQASVAEDSGGFAVRLDDRPIKTPSRHALAAPDRTLAETIAAEWQAQGETIDPSTMPLTR

LANSVIDGVAGRTEAVTDDIAKYFGSDLLFYRAEHPEELIAREAAHWDPVLFWAKDALGAHFILSQGIVH

VGQPETALAATRAAL-P-HPWLLGALHVVTTITGSALLALALAHGRLDADQVWAAAHVDEDWNIEQWGLD

EEAAARRAAKQAEFAAAAQVIAALRS

>B4R912|B4R912_PHEZH

EPAEKPRRFYKDVSVGE-AAGFAVLLDGRALRTPKGQPFRAPSREIAEQVAEEWAAQGETLELATMHATR

LANTALESISQNREAVAGQVAQYAASDLVCYFAEGPEALVSRQVAAWGPVLERAQAEEGLSFVRAAGIVH

REQPSETLEKVRALALELDDFALSGLAFGVALFGSAVLGIAVLRGWLGGEAAFELSRVDEAFQEEKWGVD

AEAAERADKLRDEARMLERWFRGLAA

>B4WEA3|B4WEA3_9CAUL

ETEETVRRFWTAVSVGPSGGGWAVLLDGRTPQTPAKAPLVLPTEAAAQLVAEEWAAQGEIVEPSTMPATR

LASTAIDRIGQAREPVAEEIAAYAGSDVVCYLAEHPTPLVERQRREWGPWRDWAAREMGVALEPVAGIVH

RPQSPDAIARVKAHAMAMDDFRLTGLATAVPLLGSAVLALAVEQGALAGGAAFELSRIDELFQEEQWGVD

AEAAERTQARRAEAELLDRWFRTL-D

>B5J3B8|B5J3B8_9RHOB

MSNWVAKRFWTDVTVIETDGGFAVQLDSRLVKTPAKAALVLPTRAMADAVAGEWRLVVEKIDPNVMPVTR

SANAAIDKVAIQFSEVAAMLAAYGGSDLLCYRAENPEGLVQRQSDGWDPLLDWAHDTFGARLIQTAGIMP

IDQNKTDLNVLTAPIFAATPFELAAFHDLIAMSGSLVIALGVTRGRLAPKEAWALSRIDETWQAERWGAD

DDAARVAEIKRTAFVHAADFYQMAHR

>B5KDF4|B5KDF4_9RHOB

MSNWVAKRFWTDVNVIEIDGGFAVHLDARPVKTPAKAALVVPTRSMADAVAGEWRLVVEKIDPNLMPVTR

SANASIDKVATQFGEVATMLAAYGGSDLLCYRAENPEGLVQRQTEGWDPLLDWAHATFGARLVSTVGIMP

IEQSEVDLAALAAPLFEASNFELAAFHDLIAMSGSLVIALGVAQGHLTPAEAWTLSRIDETWQAEQWGAD

DDAAQVAEIKRAAFVHAADFYQMARG

>B5ZQN9|B5ZQN9_RHILW

MKKPLPKRFYAEVAVAEHEGGFAITLDGKMVRTPARQVLAVPTQALARLVAAEWQAQGEEIDPVTMPVTR

LVNTALDGVDANRQAIFEDILRFSSSDLICYRADGPELLVERQTERWDPVVDWAANDLGARFILVEGVMP

REQPREATAAFAVTLARYNPMALAALHTITTLTGSAILALALAEGHLTMEEAWSLAHLDEDWTIEHWGSD

EEAEERRAKRFAEFKAATDVFFAVSA

>B6AVG1|B6AVG1_9RHOB

MSDWKMKRFWEVAAAVEVEGGFTVHLDGRAIKTPGKAHLIVPTLVMAEAMALEWDAQVEGVNPETMPVTK

SANSAVDKVTPQFDVVADMLGEYAGTDLLCYRASQPAELVARQEAAWDPLLAWCTRVFAAPLTPVSGVMF

APQSESSLHILRAALSKLSAFELTAMHDLITLPGSFVIGLAAMKGYATPAELWELSRLDERYQQEQWGLD

EEAEDMARIKGEAFEHAYDFLQKSRN

>B6BD95|B6BD95_9RHOB

MSGWAQKRFWKAVSVAQTGDGFAVELDGRRVKTPAKALLAVPTREMAEAIAAEWDAQAESVDPSAMPCTR

SANAAIDKVTHQHREVAAMLAEYGDSDLLCYRADAPVELMSRQAQEWDPALDWAAETLGVRLQPRTGVLH

QPQDAAALAVLAENVREMNPFQLAAFHDLVGISGSLILGFAAAHDWRSAEDIWLLSRLDERWQEEQWGVD

EEAAAAAEVKRQEFLHAKRFFDF--S

>B6IQX2|B6IQX2_RHOCS

------KRVYREVGTAA-EGGWEVRLDGRSLKSPARAPLVLPTRALAEAVAAEWDAQTETVEPHSMPMMQ

LASTTVDRIVPQRAAIVDGVAAYAGTDLLCYRADHPRSLVARQEEAWQPLLDWVASRYDAMLLPTTGIVH

RPQSDLTLAALRRAVEAQDDWRLSGLQNAVALSGSLVVALALLEGRIDAEEAFAVSQLDESWQSETWGVD

AEAAARRAGIRADLGATERFLRLLAG

>B6JEZ6|B6JEZ6_OLICO

SRTPLRKRFYQAASVDEMPEGFAVTLDGKPVRTPGKRLLAAPVRTLADTMAAEWGAQKEQIDPMSMPMTR

LANSVIDGVTENAVAVRDDAARYLGTDLLFYRASFPEQLIANQTEHWDPVLRWAADNLGAHFILGEGVMH

VAQPARAVQAAQQAL-P-TPWPVGAFHIVTSITGSALLALALQHKALTADAVWQAAHVDEDWNSRQWGED

DEVTQRRAMRRRDFDAAAAVLAAIPR

>B6R0Y7|B6R0Y7_9RHOB

TKRNLPKRFYERVSVEQKDDVYAVLLDGRSIKTPGRNALVFKDEVIAEGVAAEWELQEEEIDPGTMPLTR

IAHSAIDAVEEKFADVADEITRYAGNDHLCYRADSPAELVGRQTAQWDPVLGWAEKLLDGRFKLVEGIMH

VAQDEAVTAAYRAEL-DKDALQLAAIHTVTSICGSALLALALAEKAFDADAVWSAAHVDEDWNIEQWGQD

EEAARIRRFKNDEFNAAALILVGAES

>B7QX43|B7QX43_9RHOB

MSEWKQKRFWKEVTVADTEGGFAVELDGRRIKTPAKAALVVPSRAMADAIAAEWDAQTESVDPSTMPTTR

SANAAIDKVSHQHGEVSDMLADYGDSDLLCYRADSPAELVARQAESWDPALAWAEEVLGAKLAPRSGILH

VAQDAAAVARLRGLVHEMSAFQLAAFHDLVSMSGSLVLGFAAARDWRPAEEIWQISRLDELWQEEQWGKD

DEASATAELKRLAFMHAKRFYDFS-S

>B7RL97|B7RL97_9RHOB

MSDWKAKRFWTTAQVVAAEGGYAVELDGRPLKTPAKRSLIVPTEQMAQAVAAEWQAQEGTIDPRTMPCTK

TANAALDKVAIQHAEVADMLAAYGDCDLLCYRADSPQELVERQNALWDPMLDWAEEALEVRLAPRVGIMH

VPQDAAVVARLTDRTHALDKFQLAAFHDLVSLSGSLVLGFAATLNARDTDTLWDLSRLDEIWQAELWGAD

DDAEALAAVKKASFEHAKLMFDLSAP

>B8ERI8|B8ERI8_METSB

LKKSLPRRFYSHAAAAPCEGAFALLLDGRPARTPARNPLALPTLEAAEAIAAEWERQQELIDPGQMPLTR

IANSAIDGVSNEMDATIADIAQFGGSDLICYRAGEPEALALAEAAAWDPMLNFAREKLGARLICAQGVNY

VEQPEPARRAVLQAVREAGAFALAALHVMTTLTGSALLALAVAHGALTAEEAWAAAHVDEDYQMQLWGAD

EAALARRARRWSEMEAAARLLRAAPA

>B8IK76|B8IK76_METNO

SRPALPKRFYAEAGVAA-EDGHRLVLDGRPARTPGRRMLAVPQAAVAQALAEEWGAQGEEIDPARMPLTR

LVNTALDGVADRRAAVAEDIAAYAGSDLLAYRAGDPARLVASQSAAWDPVLDWARSELGARFMLAEGVMH

VPQPEESLQAVRRAVEAVSPLALTGLHVMTTLTGSVLLALAVLHGRLTAEEAWAAAHVDEIFQASVWGRD

EEAEARRALRWEEFAAAARLVALT-V

>B9JFK1|B9JFK1_AGRRK

MKKPLPKRFYKDVGIREGEDGHVIELDGKVVKTPARRSLAVPTEALAKLVAAEWARQVEIIDPTVMPVTR

LVNTALDGVATDPQAVFEDILRFSSSDLLCYRADQPERLVERQSERWDPLIDWAANELGARFILAEGVMH

QEQPREAIAAFAVTLRKYTPLELASLHTITTLTGSAILALAFAEGQLSLAEVWSLAHLDEDWTIEQWGHD

EEADHRRAQRFEEFQAATDVFSALRS

>B9JWJ0|B9JWJ0_AGRVS

MKKPLPKRFYKDVTIAAGQDGHAVLLDGKTVKTPARNALVLPTEPLAALVAGEWQGQGEFIDPATMPVTR

LVNTALDAVSANTQEVLDDIVRFCGNDMLCYRADAPQELVERQSAKWDPVLGWLADTHGARVLQTSGIIY

QPQPSDAIEAFERALQRYDGVALASLHVMTSLTGSAILALALADGALTLFDAWDLAHLDENWTDEHWGSD

MEAEARRAARFVDMQSAYDVLRAARA

>B9NL88|B9NL88_9RHOB

MSDWKPKRFWTSSAVVETDGGHTVELDGRRVKTPAKAALVLPTRAMAEAVAAEWEAQEKEVDPTTMPFTR

SANAAIDKVRHQHREVANMLADYGDSDLLCYRATHPEALQARQAEAWDPALDWADEVLGARLIPLAGVVH

QPQNTEALRVLRRQVQALTAFQLAAFHDLVSLSGSLILGFAAAKGWRNPDEIWRISRLDELWQIEQWGDD

DEALIVAEAKESAFLHAKRFYDIS-S

>B9QXU9|B9QXU9_9RHOB

SRRELPKRFYKSAEHIETEDGFAIHLDGRPVKTPAKSTLLLPTEVLGAAVAAEWDAQEKEINPARMPLTR

IANSAQDAVANRFGEVADDITGFAANDALCYRADDPESLCDTQRRVWDPVVEWAGDQLSGRFVLIEGIMH

TPQDQALLTAFRTRI-GESPLRLAGLHTVTTLTGSALLALALRDGFLDADSVWTAAHVEEDFNIERWGED

AEAKQIRAYKRSEFDAAALVLA--NA

>C3MB10|C3MB10_RHISN

MQKPLAKRFYKTVGVAPAGGGHAVLLDGRSVRTPAKRPLAVPTRKLAELLAAEWDAQAEIIDPSAMPLTR

LVNTALDGVALDQRAVFDDILRFTGSDLLCYRADSPAGLVERQNAIWNPVIDWAAQSLGARFILVEGVIH

QEQPREAISAYAEGLRAFTPLGLACLHTITTLTGSALLALAFGMGRLSAEDAWSAAHVDEDWQIDHWGTD

EEAFQRREKRWQEMQAATAVLDAL-R

>C4WJL2|C4WJL2_9RHIZ

MLAQLPKRFYEKAEVAE-EGGFAVHLDGRPVKTPARSLLRLPTKAAAVIVADEFAAQEKVIDPGKMPATR

LVNTAIDGIAQDPQAVFEDILRFAGTDMLCYRADSPQELVSRQTQQWDPLIDWMEG-LGARFSLAEGVMH

IEQPREAIAAFGVHLSAFDPIALASLHTMTTLTGSAIIALAIAKGEVTAEQGWQLAHIDEDWTIEHWGAD

AEAVERRRNREIEMMMAARVLAA--L

>C5B3B1|C5B3B1_METEA

SGPTLPKRFYAQAGLAE-EGGFRLVLDGRGANTPGRRPLVVPDRVLGEALAAEWEAQADVIDPRTMPLTR

LVNTTIDGVVERRAAVAEDLAAFAGTDLVAYRAGAPERLVAVQSEAWDPLVAWVAETLGARLFLAEGVMH

VEQPEGSVAALRAAIEAVDPFRLAALHTLTTLTGSLVIALAVLHGRLSADDAWAAAHVDETYQASVWGRD

AEAEARLAHRRTEFETAAMVAQRP-N

>C5SPD2|C5SPD2_9CAUL

QIGAKPKRFWKTVEIKA-GPGYGVTLDGRAVKTPKGATLVLPNFALAALVGREWEAVEETVDFTAMPLTR

LGFAALDHMDSGLEAALAEAARFAETDLVCYPSDYPQALIAREQAAWGPVIDWLRRELSLEFVPQTSIMA

RGQPVATIEGVKTLLTTASVYVRAGLMAAIPLLGSVALALALYKGRLSAEEAFAASRVGETFQKETWGED

AEALKREAAMRHDLTHLEAWFRG--V

>C6AE77|C6AE77_BARGA

SCQPLAKRFYKQVKIACEEGRFTILLDERPVKTPARRHFHVPTEVFAEYVAQEFESQKHVVDPAKMPMTR

LVNTVIDGIADDMQVVFEDLLRFVACDMIFYRAQTSKELVQKQSEQWDPLLDWAEKKLGSRFYLTEGLMH

VEQSPEALQAVSHYLRSVSPYMLAALHMMTTLTGSALIALAVAAGSIDADHAWSIAHLDEDWMMEQWGID

KETMVRRAHKKVEFKAAATIVTTC-L

>C6AYR0|C6AYR0_RHILS

MKKPLPKRFYTDVSVAEHEGGFAITLDGKLVRTPARQVLAVPTEALARLVAAEWQAQGEEINPVSMPVTR

LVNTALDGVAANAQAIFEDILRFSASDLICYRAEEPELLVERQAEHWDPVIDWAANDLGARFILVEGVMP

QEQPREATAAFAVTLARYSPMALAALHTVTTLTGSAILALAFAEGRVTVEEAWSLAHLDEDWTIEHWGSD

EEAEQRRAKRFAEFKAAADVFFALSA

>C6QGS2|C6QGS2_9RHIZ

LAKPLARRFYKEASTGD--APFQILLDGRAVKTPKKRALAVPTKALALAIADEWQAQVDTIDPSRMPLTR

FANTAIDAVSETLDAVAADIVAYAGSDLVCYRAETPDELVALQSRDWNPIVAWADATLDARFRVVPGVVH

VEQSSEALAAVGHALTPHDPFRLTGLHVLTTLTGSALLALALESKAVTVDAAWNAAHVDEDYQISLWGED

AEATARRRGRRAEFDAAWRWLALLRT

>C6XLD4|C6XLD4_HIRBI

NAGQRMKKFYKQAAIEKLDGNWTISLDGRQLKTPAKKPLSLPTEELAEAVAGEWADQVEFIDVATMHITR

LVNVAIDRTPLARPEMADEVARYAETDLVSHLAEGPTVLRERQQEGWAPIRDWAAQELNVFLLPVEGVMA

SPQPTTSLEAARQHAAN-DDMRLTGLNFGLGLFGSAVLSLAVEQGRLLAEEAFDLSRIDEIYQAEQWGED

EEAMHRVAYNRHQALGLGVFFAALKR

>C7D6Y6|C7D6Y6_9RHOB

MSEWKQKRFWDTAVAKQVEGGWGVSLDSRALKTPVKSALVVPTAQVAEAIAAEWNAQGENIDPSTMPFTK

TSNSAIDKVTPQQAEVADMLAEYGGTDLLCYRADAPQGLVDRQAEGWDPMLAWASSRLDATLVSVTGVMF

SAQDETALANLRAHVHALDAFELAAFHDLVGLSGSLVLGLAAAHAHGEADQIWGLSRIDETWQEDQWGRD

DTAHAEAEIKREAFEHAYRFFQMVWR

>C7JP74|C7JP74_ACEPA

NAPAGRKRFWKQADVVP-GQGFVVQLDGRSIRLPRKTPLCVTSRALADALAAEWQAAGQRFSPADLPLTG

IAGSMIERIPAEREGVLRSLLAYAGSDLLCYREQGEGKLAQAQRKEWDPWLEWLRKQYGAILHTSCGVMP

IVQPEESLQKLYDAMEKLNPAELAVLAVSVPALGSLVLGLALVNGAGSVDDLVASATLDECMQMAVWGED

TEITDRIAGIQREVADAARFLELAAA

>C8S4Y3|C8S4Y3_9RHOB

MSGWVAKRFWKQATAEPVGAAFTVRLDGRAVKTPAKTLLEVPTLALAQEIAAEWDAQQGVIKPDAMPFTR

MANSALDKVAPQFAEVAGLIAAYGASDLICYRANGPEKLLARQAAAWDPLLAWSASLLEAPLITTAGVMH

VEQPHASIARLAAEVQACTPFQLAALHDLVMISGSLVGGLAVSRGWMDAQTLWDISRVDERWQAELWGED

EEAAQSEGLRHAALIHAGRFYGLC-G

>C8SWB9|C8SWB9_9RHIZ

MKTPLPKRFYKTVSVAPVEDGFAVHLDGRPVRTPGKALLSLPTEAAAALVADEFAAQGETINPVTMPVMR

LVNTAIDGVASDPQAVLEDILRFASSDLLCYRADAPQGLVERQNEHWDPVIDWARGSLGARFNLAEGIIH

VEQPRETIAVLGAHLAQRDPLRLAAIHVMTSLTGSALLALAVDFGELDVEAAWAAGHVDEDWQIEQWGQD

AEAVARRSARKRDMMAAVQLLEALKA

>C9CUC4|C9CUC4_9RHOB

MSEWKQKRFWKEVSVEEDGAGFAVALDGRRVKTPAKTALVVPTRALAEAIAAEWDAQEEQIAPLSMPNTR

SANAAIDKVAVQFAEVADMLAEYGDSDLLCYRAESPEGLVARQSESWDAALEWAEAALGARLLPRAGILH

APQDGAALEVLRKRVHQMTPFQLAAFHDLVGLTGSLILGFATAANWRSLDEIWEISRIDERWQEELWGPD

DEAQAIEAQKKQAFEHAAGFYAR--V

>D0CUP3|D0CUP3_9RHOB

MSDWKPKRFWTESAVVAVDDGYTVELDGRRVKTPAKAALVVPTRAMAEAVGREWDAQEKEVDPATMPFTR

SANAAIDKVRHQHAEVADMLADYGDSELLCYRATYPQELAARQSEQWDPALDWAAETLGARLQVVAGVVH

QPQPVDAVNRLRDMVHALDPFRLAAFHDLVSLSGSLILGFAAARNWRTAEEIWSISQLDEIWQAEQWGDD

EDAAQVAATKKAAFLHAKNFYDLS-V

>D0D5C7|D0D5C7_9RHOB

MSEWAPRRFYTEATVTETEGGYGIALDGRRVMTPGKSPLVVPTRALAEAIAAEWAAQGEKIAPETMPFTR

TANSAIEKVAPQRAAVADMLAEYGDSDLLCYRAVEPEPLVALQSERWDPMLAWAAEHLGAALEPRAGLMH

APQPAEALERLSVRTHTLDHFRLAAFHDLVALTGSLVLGFAATDPAQDPEALWALSRLDETWQEETWGID

EEAREMSENKRRAFLHAHRFFAHCTD

>D0XS69|D0XS69_9CAUL

ESEERLKRFWKDASVAP-GEGHVVLLDGRAPKTPAHARMVLPTEAAARLVADEWAAQGEFIEPGTMPATR

LAATAIDRVSQTREPVADEIASYVGSDLLCYLAEHPTNLVAEQARDWAPWRHWAGAELGVHVEATQGIIH

RPQPPETLARVKTLALELDDFALTGLATAVPLFGSAILGLAVQRGALSGAAAFEISRLDEAFQERQWGVD

ADNAERTEARRAEAALLDRWFRA--L

>D0XVR7|D0XVR7_9CAUL

ELLLKPKRFYKAAAAVP-DNGFAVQLDGRTPKSPARKPLVLPTLALAELIAAEWDAQVEFIDNSLMPASR

LAFTAIDRVSETRADVAREITAYAASDHLCYRADHPTALVERQSREWGAMLDWVKAEHDLSFTPVAGIIH

QPQPDTTLATVEALALTLDDFTLTGTAFAAGLFGSTILALAVRAGRLTGQRALDLSRLDEMFQAEQWGED

AEAKARAEALAVEAVMIDRWFAALRR

>D1CXW9|D1CXW9_9RHIZ

MQAQLPKRFYEKAEVAE-EGGFAVHLDGRPVKTPARNLLLLPTRAAAQIVADEFAAQEKVIDPGKMPATR

LVNTAIDGIAQDPQAVFEDILRFAGTDMLCYRADSPQELVSRQTENWDPLIDWMES-LGARFALAEGVMH

VEQPREAIAAFSVHMAGFDPLALAALHTMTTLMGSAIIALAVAKGEISAEKGWAIAHIDEDWTIEHWGSD

AEAIERRKNREIEMMVAARLLEV--L

>D2LAQ4|D2LAQ4_RHOVA

SEQKTLKRFYKDATVAE-DGAFQIHLDGRTIKTPAGRALAVPTRGLADAIAGEWNEQGETIAPHSLFFTR

LANSAADAVAPREAEVVDEIVSFAASDLLCYRAPFPAALATRQAEAWNPVLAFIREKYGATFEVAEGVGH

VAQPPASLEAIRNAVAAYGPFRLAALHMMTTLTGSALLAIAHVDGFLDLAATWAAAHVDEAWQAAQWGQD

FDAAERLKRRSDDFDKASRFFAL--A

>D2M6P2|D2M6P2_RHOPA

SRAQLPKRFYTEAGVAEENGGFAVRLDGRSVKTPSRNALAAPDRALAEAIAAEWQAQGETIDPSTMPLTR

LANSVIDGVAGRINAVTDDIAKYFGSDLLFYRAEHPEELIAREAAHWDPVLYWAAEAFGAHFILAQGIIH

AAQPETAIAAARAAL-P-DPWSIGALHVVTTITGSALLALALAHGRLDADQVWAAAHVDEDWNIEQWGLD

EEAAARRAAKQAEFAAAAQVIAALRS

>Q07KQ9|Q07KQ9_RHOP5

TRGPRPKRFYKDAGFIETADGFAITLDGKNVRTPSKQLLVAPNRVLAQAIAAEWQAQPELIDPSSMPLTR

LANSVIDGVADRVIAVTDDIANYFQSDLTFYRASFPEELVALEAQHWDPVLFWAAETLNAHFILAEGIVH

VRQPEAAIAAARAAL-P-DAWRVAALHIVTSITGSALLALALYARQLDADQVWAAAHVDEDWNISHWGVD

EEVAARRAARLVDFKAAVMVLETLTG

>Q0ANT6|Q0ANT6_MARMM

HESSLPKRFYKAVDVVA-DESFAVHLDGRPVKSPAKRTLALPTRALAELVASEWEAQGERINAPTMPATR

LCFVALDLIPDARSATVAEVTKYASTDLVCFRAPEPPELTASQAAAWDPVLAWAEDSLGAHFVAATGLMP

IHQDPVALQRVMQRAGELDDWRLTTLAHVTAVCGSALLALQLLDGEIDGEQAFALSTLDEHFQISQWGED

HEAADRLARLRTELVTMGEVLRALAA

>Q0BUM0|Q0BUM0_GRABC

-----MKRFWKEAAVVQ-AAGLAVTLDGRPMRLPGGVSLRFANRALAEAIAVEWGQAGAPFSFDMVPMTR

LAGTAQERVAVDPAASVEALVSYGGSDLLCYRADGPEELTTRQERLWQPLLDWAEQRYGARLHVTTGIIH

VSQPEASLMALRAALTALAPAALAALGIVVPALGSLVIGLALAEGRLDPPEALEIALLDDLYQEEKWGAD

AEAAKRRTHLAADVETAIRFLRLSAV

>Q0C3G5|Q0C3G5_HYPNA

SAFEHPKRFYTDVTAEPAGGGWQILLDGRSVKTPGRALLRLPTEALAQEVAEEWAEQQTYIHLIGMHLTR

LANVAIDRTPEIREEMAEEVARYCETDLLCHIAEEPFELAALEEARWRPVLDWAGEKLGVILMTTEGIIA

APQPGASLQAARDYALG-DDFRLTALVFACGVFGSAVLAMALVEGALTAESAFHLSRVDEDWQAQKWGQD

EEAKAAADAKEIEADAIGRWLDGLQG

>Q0FAA9|Q0FAA9_9RHOB

MTPSFKKRFWKDARVVDLNDGYVVELDGRVIRTPSKALLKVDFRKIADQIAFEWMAQEEIVNPATMPNTR

MANSVIDKIMVNKEAVIEMLSDYSGSDLLCYRAVSPQGLISQQNEIWNPILEWSKDVFLAPMVTTSGVMH

IKQNNNSMNIYRNELKKMNLYQLAGMHDLITISGSFVIAMALISNHLNISDAWHAATVDERWQEMQWGID

DEASEALEKKRKDFEFAYKFWENATN

>Q0FIH2|Q0FIH2_9RHOB

MSEWAPRRFYETATATQVEGGHGIALDGRRVMTPGKSPLVVPTRGLAEAIAEEWQAQGEKIDPNTMPFTR

TANSAIEKVTPQRAAVADMLADYGDSDLLCYRADQPDELVQRQSERWDPLLDWAAARFGARLEPRAGVIH

APQDPDALAALARQAHALDAFELAAFHDLVSLTGSLVLGLAATDPDHDPEALWALSRLDESWQEELWGVD

EEASDVAERKRAAFLHAHRFYRLCAD

>Q0G6Y4|Q0G6Y4_9RHIZ

MRRDLPKRFYEEAALGSAETGYQVLLDGRPVKTPAKKALVLPNDEISNAIRDEWAAQGERIDPGTMPATR

LANTVVDAVALDPKPVLAEVPRYAETDLLFYRAGHPDSLVERQRERWDPIVVWASELLEVRFVLTEGVMH

VEQSAESLKAFAKRVSPIDPWVIAGLQQATSISGSGLIALALFERRLGVDEAWALSRLDEDWNAERWGED

EEAQLVSRRRKADFETAALF-MG--R

>Q11HT3|Q11HT3_MESSB

MRQPMPKRFYERVEVSGEGNAWKVLLDGRAVRTPAGVELVLPNEAAASLIAGEFAAQGEQIDPMSMPVTR

LVNTAIDGVAADVQAVMEDILRFASTDLLYYRADAPERLIQLQAEAWDPVLDWAEAQLSTRFILAEGVMH

VEQPRSAIAAVGAHLRPRDPLRLAALHVMTTLTGSAILALAVEAHAIEAEAAWEAAHVDEDWNISQWGED

AEASARRAARKRDMMGAVALLAAL-S

>Q134Y6|Q134Y6_RHOPS

TRTALPKRFYTEAGITEAADGFAITLDGRGVKTPSRNALTAPTRELAEAIAAEWQAQQELIDPTTMPLTR

LANSVIDGVVGRVEAVRDDVAKYFGSDLLFYRASHPEELIALEAQHWDPVLFWAADEFGAHFILAEGIVH

VSQPEPAIAAARGAM-P-DPWSVGAFHVVTTITGSALLALALVRGLRDPDQVWTAAHVDEDWNIARWGLD

EEVAARRAAREIEFKAAAQVLAALGS

>Q16AB2|Q16AB2_ROSDO

MSEWKAKRFWKEAVIDETAEGFGIALDGRAVKTPAKRALIAPTRPFAEKIAAEWNAQGEQIDPATMPFTR

SANAALDKVAVQKQEVADMLAAYGDSDLLCYRAEYPEGLVARQAAQWDPLLDWAADALGARLDARAGVMH

VPQAPEALAVLTEKTRALSSFELAGFHDLVSLSGSLILGFAATHDFLPAKTIWDISRLDEIWQAEEWGRD

AEAEAAAAIKEAAFIHAKQVFDLSFV

>Q1GJZ0|Q1GJZ0_SILST

MSEWKQKRFWKAVSVEEDDSGFAVALDGRRVKTPAKTALLVPTRALADAIAAEWDAQEEQIDPLSMPYTR

SANAAIDKVAVQFAEVADMLAEYGDSDLLCYRAESPEGLVARQSESWDAALDWAEGALGARLLPRAGILH

APQDSAALETLRTRVHQMTPFQLAAFHDLVGLTGSLILGFATAEGWRALDEIWEISRIDERWQEELWGPD

DEAQAIEAQKKQAFEHAAAFYAR--A

>Q1GRA9|Q1GRA9_SPHAL

-----MKRFWKEVTVVA-GVGWSIALDGRPVRTPQRATLMVASAALAEAIAAEWDDVGETIDPAAMPMTG

LANAAIDLAAPDLTAFAAPIAAYATSDLLCYRDARDATLQAEQAAAWNPLLAWAEERYGVEFALTQGVIP

VDQPAATVAALRDAAFAQDQWRITALTPLVTIGGSLVAGLALIENAFDADALWQAVSLDELYQERRWGAD

GEAQKARAAKQRDWDNAARFLGL--L

>Q1MG70|Q1MG70_RHIL3

MQKPLPKRFYAEVSIAQHEGGFAITLDGKMVRTPARQVLAVPTEALARLVAAEWQAQGEEINPMSMPVTR

LVNTALDGVTANAQAIFEDILRFSSSDLICYRAEEPERLVERQAERWDPVIDWAANDLGARFILVEGVMH

HEQPREAIAAFAVTLARHSPIALATLHTVTTLTGSAILALAFAEGRVTVEEAWSIAHLDEDWTIERWGRD

EEAEERRAKRFAEFKAAADVFFALSA

>Q1N7U2|Q1N7U2_9SPHN

-----MKRFYKDVAIVAGSEGFAIELDGRAVRTPARALLALPTRNLAEAVAAEWRAQEGEINPAAMIFTG

LSNAAIDHIAPNPGAFAAGIARYAQSDLLCYRADGPDALVARQAAAWDPLLDWAASRYDAPMIVTQGVIP

VSQPEDSLARLEAAVRTHDPFMLAGLSTLVTLSGSLICGLAIAEGGYDPEELWQAVQVDEQWQSEQWGED

SEAAARNLLRAREFSTAGAFCTMSRV

>Q1QN00|Q1QN00_NITHX

GRTGQPKRFYTSAGVREVPEGFAVLLDDKPVRTPSRNLLAAPAREIAASIAAEWQAQQDVINPMTMPMTR

LANSVIDGVAGRVDAVVADIVKYFGSDLLFYRAGHPDALVTREAAHWDPVLFWAAETLGAHFILTEGVVH

VRQPDQAIAVARAAL-P-DPWAVGALHVVTTLTGSALLALALMRERLNADEVWAAAHVDEDWNGEQWGVD

EEVTVRRAARLADFRAAAAVLAAQFG

>Q1YNH4|Q1YNH4_MOBAS

AKAELPKRFYSEVTLAPTDDGFAVLLDGRPVKTPGRRPLAVPMQRAAEVVAAEWGAQRERIDPATMPMTR

LVNTVVEAIADDPIPVRDDLARYIETDLLFYRAGTPERLVARQQELWDPVLDWARDSFGARYLLTEGVMH

VAQPPAAIAAFKERLAGIDPFKVAAMHQATTLTGSALLALALAEGQLSAEDVWLRAHVDEDWNIEQWGAD

DEASARRELRWQEMQVAAVLLGN--E

>Q211J5|Q211J5_RHOPB

TRGPRPKRFYQNAGLVEAEGGFAITLDDKAVRTPSRGHLIAPNRALAEAIVGEWQAQQQFIEPTTMPLTR

LANSVIDAVTTRVEAVTDDVAKYFETDLLCYRASFPEGLVARESEHWDPVLFWAAETYSAHFILAEGIVP

VRQPDAAVAAVRAAL-P-DPWAIAALHMVTSITGSALLALALHDRFRDADQVWAAAHVDEDWNIAQWGID

EEVAARRAARQVDFKAAAVVLAALPG

>Q28UR9|Q28UR9_JANSC

MTEWKAKRFWTEATVAEADGGFKVLLDGRGVNTPGKLPLVMPTRAMALAVAAEWNAQEGEIAPLTMPHTR

SANSAIERVTPQLADVSDMLLGYAETDLLCYRAEGPDALTQRQAAEWDPMLDWGAEALDARLEPRTGVMW

VSQEPTAIKALDTDLRQIGPFPMTALHDLVTLTGSLILGLAVARGRISAKEAWRLSRIDETWQMEQWGAD

EEAEDAAAIKEAQLRHAEAFWDLCDK

>Q2CBL7|Q2CBL7_9RHOB

MSEWKAKRFWTAAHVAERGDGFAVTLDDRPVRTPAKAPLTLPTRALAEGVAAEWDAQQEQIDPLSMPLTR

AANAALDKVAPQFDEVAAMVADYGGTDLLCYRADAPEALVARQAEAWDPLLDWAADTLGARLRPAVGVMH

VEQDPGALLRLEGQVRALDPFRLTAFADLVALSGSLVIAFAVLEGARTPEDGWALSRIDEEWQIEQWGED

DEASAFAARKRADFLAAARFLRLARR

>Q2G4E6|Q2G4E6_NOVAD

-----MKRFYKQVTVEAAEGGFAVKLDGRAIRTVGKRTQVVPTHALAEAMAAEWAGQGEEIDPQAFLFRD

MADYAIDVVAQDPASVIGELLPYAETDTLCYRAEPDEAFAARQRLMWEPLLADAETRLGVRFVRVSGIMH

KPQPPETLARLKVELEGLGAFQLAALRNTASLAASLVLGLATLRPDADIDALWDAANLEEDWQAELWGKD

AEAMERREKRAAAFAAAARFAAL-AR

>Q2IXL8|Q2IXL8_RHOP2

TRTSLPKRFYTTAAISETPDGFAITLDGRPIKTPTRNALAAPSRDLAEAIAAEWQAQQELIDPITMPLTR

LANSVIDGVAGRIDEVTDDIAKYFGSDLLFYRAGHPEELIAREAQHWDPVLFWAAEEFGAHFILAEGIIH

VDQPETAIAAARAAL-P-HPWSVGALHVVTTITGSALLALALAHGRLDPEQVWAAAHVDEDWNIARWGLD

DEVAARRAARQVEFQAAARVLAALGS

>Q2K822|Q2K822_RHIEC

MKKPLPKRFYAEVAVADHEGGFAITLDGKMVRTPARQVLAVPTEALAQLVAAEWQAQGQEIDPVTMPVTR

LVNTALDGVATNAQAIFEDILRFSSSDLLCYRADGPELLVERQRERWDPVIDWAANDLGARFILIEGVMH

HEQPREATAAFGVTLARHGPMALAALHTITTLTGSAILALAFAEGRLTVEEVWSLAHLDEDWTIEHWGRD

EEAEERRAKRYAEFKAAADVFFALST

>Q2ND83|Q2ND83_ERYLH

-----MKKFWKEVSVEQVDGGYQVALDGRGIRTQGKRPQIVQTAALAELLADEWRAQGKDIDPASFPHRD

MADYAIDRIATAADDIPAKLIGFMETDTLCYRADPDEPLYKRQLDMWEPLVTAFEMREDIRVERASGIIH

KPQPPESLAKLRARIDALDPFTLAALFATTSLSASLIIGLGTLEADADSETLWDAANCEEDWQVELWGDD

FEAADRRARRKRDFLRAVEFARASTP

>Q2RQZ2|Q2RQZ2_RHORT

LSAQLRRRFYTTATVGA-EGGFTLLLDGKAVHTPGKRPLVVPTKGLAEAMAGEWAGQGETIEPDSMPLTQ

LANTAIDRMAAARPALEEDLLRYGGSDLLCYRAERPRELVERQHRAWQPALDWLAAHAGADLVVTSGLMP

IDQPETALEALGRLVRAYDDWTLTAVQAATAACGSLVLALALIEGRISAEEAFTLAFLDDSYQMEQWGED

AEAVARRDHLRDEILAVGRFLVLLRG

>Q2W2L6|Q2W2L6_MAGSA

-SSKSIKRFYKESAAEL-DGGFAILLDGKPIKTPGGRPLSVPAKPLAEAIAGEWRSQGEQVLPSSMPMTQ

LASTAIDRVGPERPHITGQLMNYAGTDLLCYRAETPGDLVARQTAAWQPLLDWAAQALDAPLLVTTALAA

IAQPDASLAALRRHVDAYDDWRLTALQSSTAAMGSLILGLALMEGRLDPEAAFQASQLDETYQIELWGED

WEAADRRAELRGDIDAAARFRDLIPS

>Q3SST2|Q3SST2_NITWN

APAGPRKRFYARVGVNETPEGFAILLDDRPVRTPSRNVLAAPVREIAETIAAEWDAQHDLINPMTMPLTR

LANSIIDGVTVRVDAVVEDIAKYLETDLLFYRASHPDALVAREATQWDPVLQWAAEALGARFIPAEGIVH

VRQPDQAVTAVRKAL-P-DPWVVGALHVVTTLTGSALLAIALMRGRLDADEVWAAACVDEDWNSEQWGVD

EEAAVQRAVRLVDFRAAATVLRARAG

>Q5LW25|Q5LW25_SILPO

MSDWKPKRFWKQAAVAETAEGYAVELDGRPVRTPAKAPLLLPTRALAEAIAAEWDAQESEVDPINMPFTR

TANAAIDKVRIQQAEVADMLAAYGDSDLLCYRADSPAELVELQAQTWDPALDWAAETLGVRLRPVQGVMH

QPQPASAIETLTRKVHALNPFQLAAFHDLVSLSGSLVLGFAAALNWRKADEIWQISRLDETWQEEQWGPD

DEARALAAVKRAAFLHAKAAYDIS-T

>Q5NQE0|Q5NQE0_ZYMMO

M---KRKRFYKKATVDKAEIGFAVKLDDRQIMTPARHPLILPTRALAEAVAEEWNNQPKEIDPASMPITG

YANAAVDLVPDRYDDFVAGIRQFAESDVTCYRADSPQALVDREIELWEPLLEWAEKRFDIHFHRVVGIIH

KKQPEVTLQRIGAAVTDFNHFEIVALTQLATISGSLVIPLAILADEITPEKAFDAAHIDEIWQAEQWGQD

EVAANALKRRRRDFLAAARFFSLVTN

>Q6FZF4|Q6FZF4_BARQU

SCQPLQKRFYKEVKISCEEGGFSVFLDGCSVKTPAKRHFLVPTEVFAAFVAQEFKSQKEVIDPTKMPMTR

LVNTVIDGIADDMQVVFEDLLRFVACDMIFYRAQTPKELVQRQCEQWDPLLDWAEEKLGSRFHLAEGLMH

IEQPWEAIQAVSNYLRKVSPYMLAALHTMTTLTGSALIALAVAGGKVDSDHAWNIAHLDENWMMEQWGAD

EETMARRAYKKAEFNAAATIIKTC-L

>Q6G2Z7|Q6G2Z7_BARHE

SCQTHTKRFYREVKISCEEGGFSVLLDGCPVKTPAKRHFCLPTEVFAAFVAEEFENQKQVIDPAKMPMTR

LVNTVIDGIADNMQAVFEDLLRFVACDMIFYRAQTPKELVQRQCEQWDPLLDWAEEKLGARFHVTEGLMH

IEQSRESIQAVSNYLRKVSPYMLAALHTITTLTGSALIALAFSEGKIDSEHAWKIAHLDENWMMEQWGID

EETLARSSHKKSEFDAAGTIITTC-L

>Q6N4X6|Q6N4X6_RHOPA

SRAQLPKRFYTEAGVAEENGGFAVRLDGRAVKTPSRNALAAPDRALAEAIAAEWQAQGETIDPSTMPLTR

LANSVIDGVAGRVEAVTDDIAKYFGSDLLFYRAEHPEELIAREGAHWDPVLFWAKDALGAHFILAQGIVH

VGQPETALAAARAAL-P-DPWLLGALHVVTTITGSALLALALAHGRLEADQVWAAAHVDEDWNIEQWGLD

EEAAARRAAKQAEFAAAAQVIAALRS

>Q89JB9|Q89JB9_BRAJA

ARTPQRKRFYKEAGVAEAEGGFAITLDGRPIRTPSGRQVVIPSRALADAVAAEWAAQGETIDPVTMPLTR

IANSVVEGVVDRVELVSDDLAKYFESDLLFYRAGHPEALVAREATHWDPVLFWAAETLGAHFILSEGIMH

VKQPDEAVEAARAAL-P-DAWSVAALHVVTTLTGSALLALALAHGVRGADQVWAAAHVDEDWNADQWGVD

EEAAARRAARARDFEAAVAVLDAVGP

>Q92KE8|Q92KE8_RHIME

MQKPLPKRFYKQASAAPADGGYAVLLDGRSVRTPAKRAFTVPTEKLAGLLAAEWDAQTEVIDPSAMPLTR

IVNTAIDGVALDDRAVFDDILRFVGSDLLCYRADSPKELVARQNVHWNPIIDWAARTLGARFILVEGVIH

QEQPAEAVSAFAEGLRGFTPLGLACLHTVTSLTGSALLALALAMGRLSAEQAWAAAHVDEDWQIEQWGTD

EEAFRRRENRWREMLAAAVVLDAL-K

>Q9A6V6|Q9A6V6_CAUCR

ELLLKPKRFYKSAAAAP-EQGFAIQLDGRTPKSPARKPLVAPTQALGAMIAAEWEAQVEYIDNSLMPATR

LAFTAIDRIAETRAEVAREITAYAASDHLCYRAESPRVLVERQEREWGAVLDWARAEHGLVFTPVAGIIH

TPQPPETLASVEALVLTLDDFALAGVAFAAGLFGSTVLGLAVRAGQLAGQQALDLSRLDEIYQAEQWGED

AEAKARAAALAVEAAMIDRWFAALRH

>A0BMW6|A0BMW6_PARTE

QQPIQLKRFYKEATIEM--HQWLVKLDGKTVKTPSKNTLAVPSPQLASFIAHEFNMQTEYIRPTTMPLLT

LARNAIDIE-RIRQFMEQSIISYLERDTVLFRENPETKLYKIQKEKLDPQLKIFNEKFGLHLKTNFGLNI

EPLKQYDQIRIETIVS--NNWQLVSLDAKVENLKSCILALLIWNNHLQVEEAVKLSRLEEDFQIAQFGKV

EGHHDYDETIMMNVSASKLFAQ-LAY

>A0CQB9|A0CQB9_PARTE

QSQTYLKRSYKEATVEM--HQWFIKLDGKSAKTQQRNILAVPSPQLAACIASEFNRQKEYLSFKQMPLLM

LARNAIDLD-TNREYIEKAIVNHLENDVILHRKNQKSQLLQIQQQQLDPQLRFFNSKFGMDIQSNDGVQI

GSLSQQNIVKIESLIR--NNWQLVSLSSQADNLKSCILAIQLSYGQVDLEKALSLCDIENQFNKKVIENE

NPQEDSEDIISMNVKAAQLFSS-LLY

>A0CSS1|A0CSS1_PARTE

SQTLQIKRFYKDVKIEM--RQWLVKLDGKTVKTPSKNELSIPTPQLAQRIADEFSAQAEFINPATMPLMT

LARNAVDIE-NMREFMEHSIISYLERDTVLFREQSHSELYQIQMQKLDPQLKLFNQKFGMHLKANFGLDV

EPLKQYDQIRIETILK--NSWQLVCLDSKVENLKSCILAFQIWNNQIDVQEAVKLSRIEEDYQISLNGKI

EGHHDFDETILANVKAAKLFSQ-LTY

>A0CWS4|A0CWS4_PARTE

QSQISIKRCYKEASIEM--HQWIIKLDGKSIKTQKRNILAVPSPQLASYIASEFNNQNKNMSLLSMQLFL

LASHAVDLD-SSRDIMEMSFIGNFENDVILKRHQSQDKLLQKESQTFEPLIAQLNRKFNVEISSKDNRNQ

EFLNQLSKIKLESFIR--NNWQLVSLNSKIENLESCILGLNLQLGSIDITKALALRKIEQKNSM---NKS

FEKSYLKVLEAAQLFSQSITTQ-SMY

>A0DSW6|A0DSW6_PARTE

QSQTHLKRTYKEATVEM--HQWFIKLDGKSIKTQQRNILAVPSPQLAACIAQEFNRQKEFLSFKQMPLMM

LARNAIDLD-TNREYTEKTIISHLENDIILHRRNQNSQLLKIQQQQLDPQLRLFNSRFGMDIQANDSIQI

ASLSQQNKVKIESLIR--NNWQLVSLSSKADNLKSCILAIQLSYGLVDLNKALSLYDIENQFNKKLTETE

NPQEDEEDKISMNVQAAQLFSS-LLY

>A1CFZ3|A1CFZ3_ASPCL

PSSVLKKRFWKNVDVKR-DGDYEVMLDTRPIRTPAKDILSIPSTKLANAIALEWDVMTSALKNHLIPLTS

LASRAADIATTSRDQIVNVAMRYLSTDTLLCWVPEAESLREAQVRVAKDIIAFLSTKVGIDIVPINSILP

VSQPQATKDIIKQWVASLHAYDLAALERGIVASKSLLVAVRLVFERFGIEEAAEASSLEVKWQTDIWGEV

EDTHDVNKDLKRQLGSVIVVVS-GTR

>A2QY61|A2QY61_ASPNC

KSSVLKKRFWKNVDVKR-DGDYQVLLDTRPIRTPSKDVLSIPSTKLAHAIALEWDVMTTALKNHLIPLTS

LTARAADIATTTRDQIVKTAMRYLETDTLLCWVPEPESLREAQIRVAKDTIAFLSTKVGVDIQPIDSILP

ASQPQATKDIILQWISGLQAYDLAGLERGVLAAKSLLIAVRLVAERFGIEEAAEASSLEVRWQTDMWGEV

EDTHDVDKDLKRQLGSVIVLVS-GTR

>A3KP55|A3KP55_DANRE

SATAERKKFYETVSITQ-GGGFEINLDKRKLKTPSGKLFTVPN--LAIAVANEWDVQKDTLKFYTMHLTT

LCNTALDNP-TKE-QMISAALKFLETDTICYRVEE----VELQTNEWDPVMNWIEQRYNVVIGSSSNIMG

PQIPEETKETFHQHL-N-NFWSLTGLEFVINQLKSLVLSFALIDRHLNVEEAVLLSRLEEEYQIRSWGNV

EWVHDYDMELRARTAAGALFVH-LQD

>A3LR77|A3LR77_PICST

RLAKTLTKFWEKVDTHY-KDLYEVQLDGKTLKTPLGFPLALPSSKLAYLIANEWANLPDKIKTNMMPLTS

IAARSIDLI-GDLDDIKFNMLRYLDTDTCLIFTTRRGTLRKRQDELYLPLIAEFEDFFEIKLEYLDGIRG

NKQSITTQNVVLQWLHLLPIYDLIALEKAILTSKSFLCGVTLLRSKETVEEIVELGNMETIFQTREWGEV

EDTHDVDKDWLRNLTSAALLC----H

>A4H9D9|A4H9D9_LEIBR

RSSGAVRVFWKDVDVVELPGWFAVVVDGRKVKAMSSQVLAVPSEAMACCCAQEYAEQTGYINQLLMPMSD

ICSGALHIAPQMLAPRIDYLLSFFQSDNLYFRAA---PIAAKQDVMIAPIISWFERVYEMDVPRVVGIGD

PHITPHASAKMRDALIAMNPYQVLAMCVTAQFTSSLLLPLALFSGVVDLPTALAINRAEEHHSISEAGLV

EGYHDIREDVVTKICACTMTWR-LAV

>A4S7W8|A4S7W8_OSTLU

-ADGTAPRFYERVDARERDGTWRVELDARALRTPKRNEYAFATEGLARAVAAEWDAQGERIAPFTMPLTS

LSATAIDHMAETRRVHVETLLKYFGTDATRVRSP-DEATAARQAKAHDPIVAWAEREFG-PVETSDSIFG

PGTSEKTVEVLRRRLHAMCPWELTCAFALSAATKSLLISLKTLRGGLTVDEAIAAARVEEEAQIEEWGLV

EGGHDLDQDIRVKVSAPVVLMKL-KN

>A5DD02|A5DD02_PICGU

RLEKTLSRFWEKVDAKY-TNSYEVQLDGKTLKTPLGYKLAVPATKLAHLIAHEWANVPEKIKHGSLPLTS

LASRAIDLQ-GRLEDIRLNLLKYLDTDTCLIFTTLEGKLRARQDELYLPLIKEYEDFFKVELNSLDGLRG

NEQSITTQNVVSHWLESLPIYDLVALEKAILASKSFLCGASVLRAIKSIDEIVELGNLETIFQTEQWGEV

EDTHDVDKEWLRSLTSAALVV----H

>A5DWA1|A5DWA1_LODEL

KLAKTGRKFWDKCDVYL-TNLYEIQLDGKTLRTPLGFPLTVPKNKLAYLISHEWTNLPNSVKSSALPLTG

LAARAIDLL-IAIKDLKESLLRYLNTDTCLIFAGKDGKLRKRQEELYRPLIKEFQEFLKIVLQTLDGLRG

NYQTQSTQDVVIEWMDQLDIYELIALEKSILTTKSFLCGASILRSYKNVEEIIEMGNLETILQTGEWGEV

EDTHDVDQEWLRSLACAALVS----H

>A5KE38|A5KE38_PLAVI

SDKWTVNKFANGIHLRKNCGKVEIFIDDSILLTNGGKVLSFES--LCFLIKQELLRNRE-MDLQKMPLTL

MANNLVDFLQEQRSIMENKIFENFQNDLILYQNGQPNMMREEENIVYSKFISIFENIHGVKLRRANHFE-

PVQDLHVEEKIKNLI-K-NSSGIFLFYKCTQILSSFVFSYLFLHGHMGYKDVYRCCNLESIHQFVKWGYV

YDVHGARDGALLALSSLRLGA--LAE

>A6SAD6|A6SAD6_BOTFB

GGTAKLKRFWKDVNVQD--EGLQIFLDKRALRRPSKDILTVPHHKLASAIALEWDLLVSALKTHLIPMTS

LVNRALDIIDTIRNHIVTTVMRYLDTDSLLCWAPEVESLRSIQRKSAEPIIQFLSEKVGVELIPVDSIVP

KSQPQMTKDIIRGWVSGLPPFELAGLERGVLAGKSLLGAARLVVEKFGVEEAAKAASLEVDWQTGMWGAV

EDTHDVERDIRRQLGSVVLLVS-GRN

>A7EJN1|A7EJN1_SCLS1

GGTAKMKRFWKDVNIQH--EGLQIFLDKRALRRPSKEILTIPHHKLASAIALEWDLLVSALKTHLIPMTS

LVNRALDIINTIRDNIVTTVMRYLDTDSLLCWAPEVESLRSIQRKSAEPIIQFLSEKVGVELIPVDSIVP

KSQPQMTKDIIRGWVSGLPPFELAGLERGVLAGKGLLGAARLVVETFGVEEAAKLASLEVDWQIGMWGAV

EDTHDVERDIRRQLGSVVLLVS-GRK

>A7SDZ9|A7SDZ9_NEMVE

----ERKRFYKSAGVEE-TGGFQITVDNRKVKTPARNWLVVPN--LAVAMASEWNMQTGTIKPASMHLTS

LANTVIDKP-SKD-QRIEDILEYLYTDTVRFPASD----VELQKTEWGPLISWFSSRFGVSVPSCEGLLA

TPLEPGDVNKLKYEL-T-NHWAMTGLEYAVDTSKSFITSMALLDNHVTVEKAAYLTRLELEFQIGRWGSV

EWAHDVDLELRCRLAAAALFYH-LNS

>A7TGS3|A7TGS3_VANPO

RLSKTLTKFWDKVSYHY-SGKYLIQLDSKTINTPMGNLLAVDKNKLALMLSNEWKNLPNSIKKYSLPLTS

LTSRCIDLENGDRNKIINDLLRYLDTDTLLVFSPAEGALRKEQNKLYLPVIDSIENFLPIKLNILHGLRG

NQQDLNTKEAAKKYLESLSYWDLAIFEKIVLTTKSFICGILLLQGEYNVEDIVRYATLETIHQVDRWGEV

EDTHDVDKDIRRNVNAAAVVAY-N-A

>A8J6P7|A8J6P7_CHLRE

NTTSKISRFYKAAHVVPARSGFQVMLDRKPVRTPGKKLAVLPSHPLALAVAAEWEWQEKKPQLHTMPMMS

LVAHALDQP-P-RDKVIAHIMNYVHTDAACCLYER-GTLPHVGHRGQPP--DGRAR--GGR----EGLAG

PRLAPSGYGAAHRHHGGHTRGTAEGSHHSGPGAGCGACGGGLPGRGMGPCGGGPRRGRPAQSRVRAVAVR

ADAVKLKREGRSHRFGAGAERNG-YC

>A8J6P8|A8J6P8_CHLRE

NTTSKISRFYKAAHVVPARSGFQVMLDRKPVRTPGKKLAVLPSHPLALAVAAEWEWQEKKPQLHTMPMMS

LVAHALDQP-P-RDKVIAHIMNYVHTDAACCLYER-GTL-RRQQEVFGPILEALRQDAGWRFLMSDNIAG

SHQTDELVEGVRAWLAGLDDWHLAAMEQLTGTTKSVVIPAALLRGHITPAQALAAARVEEDFQAEEWGRV

EAGHDLDEDLRSRVFGPSLFVRL-MR

>A8NH50|A8NH50_COPC7

KAEITMKRFWKEVGIGK-GDDFTVTLDKRALKTPSGNTLLLPSNKVAALIAAEWDHQE-LLKPHALPMTS

IVSRAIDSL-STRQEVEKTLLNYLETDTVCFFHDDPEPLHRLQTKYWVPLIDWARSALGLEIAVSNSVLS

PNQPRKTIEEASKLIRALDRWELAALERATIATKSLIIGLALVK-HLTVEGAALAASVEVDSQIERWGEV

EDTHDVDYDVRRQLGSAALLLS-KQS

>A8P7S8|A8P7S8_BRUMA

GISSTKNRFYEEARVVF-VEVYNIYLDKHCLVTPKRNPVKIYS--LALAVAQEWNMQTNELRVNLMRLTG

LIFTATDNP-QKS-DLLSQVLQFLDKDTVLYRLEE----LHLEETNWNPVVEWVNWEYGLSVKPKA----

-VIDNNSRVRLANQL-S-NFLQLVGLQYATEALKSVFLTLATVSSRLDIDEAVELALLEQKYQSDVWGKV

EWAHDIERELISRLSAGVLLVH-LVV

>A8Q3G8|A8Q3G8_MALGO

RAEKTMERFWKDVFLGY-DEHFVVQLDRRNLRTPQGAKLAIPADRLACLIAQEWDEQT-VVKPHSLPLTS

LMSRAIDAL-QGHKDVQDYLMRYFDTDAVCFHDSEPARLVRLQEERWTPLLDWMRRSFDIEVNVAKDSLV

CQQSPETREKVARIVAGLQPLDLASLERAVMTSKSMIIGLALIH-HIEAEQAALAAEVETASQVAAWGAI

DDTHDVDHELRRQLASVACAQVVLRS

>A8WN22|A8WN22_CAEBR

SALTKPKKFYKEVTLASEGQIHKVLLDHHVLKTQGGQVLKLDS--LALAIAQEWSSQDEFLQLGQMRLTG

LAFTSQDNP-QSADTISQKILDYVDGDTVLFFNTE----HRYQEENWAPLIKNLNSDLGIQVRPSESILD

CDASETDKDKIDRWV-R-NFPALVGLQYATESVKSFIIAYNAIRHHIDPETAIDAATLEQRTQAETWGSV

EWAHGLERELLSRLSAACLFVY-FTV

>A9PI48|A9PI48_POPTR

TGSIVGKRFYKQVTTREAGNGWNVMLDYRTLKTPSKRPLKLPTLALAKAIAAEWDYQQTGIRPFTMPLMK

LACTALDRVPLTRPKIIEHLMKKFSQDLVFCRAPEDNVLYERQVEKFDPLIGWIKSEFGFKPVVHSCLFG

GKQEEGLVKAIENLLKQTDDCQLAVIDAIASAAHSLIIAVGIVKGKLDIEEAIELIRLEEDFQVDTWGLV

EGGHDIDIDLRVQISSAAVFLGL-RK

>A9TW86|A9TW86_PHYPA

SSGTMGRRFYKKTHTKPAGSGYIVMLDGRELKTPARKPLKVPNAALALAIAAEWEWQQSGIRPYTMPMMK

LAATSIDQIPRDRERVIHTLLKYFHTDSLCLRAEDTDPVAEKQSAVWDPLIDWAEQEIGERPAVTSSIFG

TTQPSHVLEAMEKVLMQSSDWQLAAIDWLAGTARSLIVALAIARGRLGIEEAMEVIRLEENHQVEEWGYV

EGGHDIDEDMRVKIAACSVFMRL--L

>B0D3L6|B0D3L6_LACBS

RAEATKKRFWSTVGVST-GDTLAITLDGRALKTPSGNTLLLPANKLASVIAAEWDNQE-LLKPHALPMTS

IASRAVDEL-STRQEVRKALVAYLDTDTICFFNNYPEPLEKLQTQHWEPLLSWARETFGIQLNISGSILS

VPQPEETKKIVERVLESLDKWEIAALERATYTTKSLIIALALVK-HLSVEKAALAAQVEVNSQIERWGEV

EDTHDVDYDVRRQLASAAILLS-N-S

>B0WBZ8|B0WBZ8_CULQU

--AAPVKRFYRKTGIIS-SGRYEITLDQRKLKTPKGAPFYVES--LAVAVATEWDAQKETIDRSSMHLTA

LSSTVLDNP-KKM-DIVNYLVNYISTDAILYHSSH----KELQLAEWSPIVDWFNKRYDVELKATDGLEV

PSFPPGTAMNISRYL-S-NEAALNGFMFAVDTIKSVVLTCACMDRFISVEKAVLLARLEEEYQLGHWGRV

EWAHDMQQESQARLSAAVMFVY-FEI

>B0YC60|B0YC60_ASPFC

PSSTLEKRFWKNVDVRK-DGEYQVLLDTRPIRTPTKDILSIPSTKLAHAIALEWDVMTSALKNHLIPLTS

LTARAGDIATTTRDQIVKLAMRYLDTDTLLCWVPEPESLREAQMRVAKDIIAFLGTKVGVDIVPIDSILP

VSQPQATKDIIKQWVSSLQPHDLAALERGIVASKSLLVAVRLVVEKFGIEEAAEASSLEVRWQTDMWGEV

EDTHDVDKDLRRQLGSVIVVVS-GRE

>B2ALW4|B2ALW4_PODAN

NAPVLKRRFWKDVSIKE-VGAYQIHLDSRPLRHPTKSIIRIPLSKLAHALAVEWDQLLSATKQHLIPLTS

LVCRAVDIGGPIRESIVTGMMRYLDTDSLLCWAPPGKSLRDLQEEAAGGVVGWLTSKVGVNIVPVGSILP

RKQEPGVREVVQGWVLGLSCWELAGIERATLAGKSLLTAARLVCEKFGVEEAARVVSVEVEWQTRRWGEV

EDTHDVEKDLRRQLGSVILLVG-GGR

>B2G4I5|B2G4I5_ZYGRO

RLSQIAKKFWDQVSVGE-NGKLVIQLDKKPVRTPLGNHLAVDKDRLARLLQKEWSNISHSVKTHALPLTS

LIARCIDLEGGNRNELSNSLLRYLDTDTILVFSPREGKLRAAQDDMYLPIIGSVEKFLPISLQILHGLRG

NAQKPETLAAARKFLDSLSAWDLAIFEKTVLTTKSFICGILLLQNQYTMEDIARAATLETIHQTERWGEV

EDTHDVDKDIRRNVHAAAIAAY-K-A

>B2WKK8|B2WKK8_PYRTR

PKNMLAKRFWKDVSVQE--GGLQVFLDHRPVRMPNKQTLTVPTSKLATAIALEWDLLMSALKNDYIPMTS

LAARAIDIEDNVRNDILAYFMRVLSTDTLLCWAPEGKTLRQVQEEVATGIIAYLQTNVGVEIKPTETIIP

VEQPELTQQVIRGWCARLPAYELAGLERAVLASKSLLVSVRLIHERFSIEDATRASSLEVSWQTSQWGEV

EDTHDVQKDLRRQLGSAIVLVG-GSS

>B3L213|B3L213_PLAKH

NDKRTLNKFANNIHLKKNCGKVEIFIDDLILLTNGKNVLSFES--LCFLIKYELLRNRE-MDLQKMPLTL

TANNFVDFLQEQRMIMENKIFENFQNDLILYQNGQPNMMMEEENNVYTKFISIFENIHGVKLRRANHFE-

PVQDLCVQEKIKNLL-K-NSSDIFLFYKCTQILNSFVFSYLFLHGYIGYKDVYRCCNLEYIYQFFKWGYV

YDVHGTKDGALLALSSLSVRVL-LKR

>B3MMC5|B3MMC5_DROAN

--TTPPKRFYKKTSVLS-SGGYEVVLDHRKLKTPKGTPFTVRS--LAIAVATEFDAQKEHIERSRMHLSA

LCFTAIDNP-NKL-DMVNYLLNFIPTDTVLFQYDD----QELQQNEWDPVIDWFNQRFEVNLQKTMNITP

PQVSEEDTIKIAKHF-Q-SLETLHGFVFAVDTLKSIILACAVIEQMLPVEKAVALARLEEEYQLKFWGRV

EWAHDLSQELQARLAAAVLYVH-LIL

>B3NLY0|B3NLY0_DROER

--ASAPKRFYKKTSVLS-GSGYEVVLDHRKLKTPKGAAFVVRS--LAIAVATEFDAQKQNIERSRMHLSA

LCFTAIDNP-SKP-DMVNYLLNFIGTDTVLFQYDD----QDLQVNEWDPVIAWFNQRYDTNLQKTMNITP

PQVSEEDKMNVAKHF-Q-SLETLHGFIFAVDTLKSIVLACAVIEQMLTVEKAVALARLEEEYQLKFWGRV

EWAHDLSQELQARLAAAVLFVH-LIL

>B3RMW4|B3RMW4_TRIAD

---TDIKRFYKNVTIET-IGGFLIKLDSKPLKTRNGQQLIVPN--LAVAVATEWAVQGKKIAPHNMPLTV

MCNGALDRP-NLR-VETAQIMEYLATDTICIRATE----VAVQNHYWNPLLDWMNDRFQVRLSCSTSFTG

ADHSPAVKNAIQDEV-S-DAWSLTGLTVLVESLKSLVIALAVTNRHITIDEAVDLARLEVNYQTAKWGNV

EWAHDLEVELKSKTAAAAIFYQ-CID

>B4GT38|B4GT38_DROPE

--AAPPKRFYKKTSVLY-TGGYEVVLDHRKLKTPKGAPFTVKS--LAIAVATEFDSQRDHIERSRMHLSA

LCFTAIDNP-NKL-DMVNYLLNYMPTDTVLFQYDD----KELQKNEWDPVIEWFNQRFETNLQKTMNITP

PTITDEDRVKVAKHF-Q-SLDTLHGFVYAVDTLKSIILACAVIEQMLPVEKAVALSRLEEEYQIKFWGRV

EWSHDLSQELQARLAASVLFIQ-LIL

>B4IFN0|B4IFN0_DROSE

--ASPPKRFYKKTSVLS-GSGYEVVLDHRKLKTPKGTPFIVRS--LAIAVATEFDAQKENIERSRMHLSA

LCFTAIDNP-SKL-DMVNYLLNFIGTDTVLFQYDD----QDLQVNEWDPVIAWFNQRYDTNLQKTMNITP

PQVSERDKMNIAKHF-Q-SLETLHGFIFAVDTLKSIVLACAVIEQMLTVEKAVALARLEEEYQLKFWGRV

EWAHDLNQELQARLAAAVLFIH-LIL

>B4JAY2|B4JAY2_DROGR

--ATPPKRFYKTTNVLC-TAGYEVTLDHRKLKTPNGTLFTVKS--LAIAVATEFDSQKDHIERSRMHISA

LCFTAIDNP-TKT-DMVNYLLNFAATDTVLFQYDD----QELQCNEWDPVIDWFNQRFETNLQKTNNITP

PQVTGDDRMKIAKHF-Q-NLETLHGFVFAVDTLKSIVLACAVIEQMLSVERAVALSRLEEEYQLKFWGRV

EWAHDFSQELQARLAAAVLFVH-LLL

>B4KLA4|B4KLA4_DROMO

--AAPPKRFYKTTNVLS-TSGYEVTLDHRKLKTPNGTLFTVKS--LAIAVATEFDAQKEHIERSRMHISA

LCFTAIDNP-TKP-DMVNYLLNFIATDTVLFQYDN----QELQQNEWDPVIDWFNQRFETNLKKTMDITP

PQVSDEDKMKIAKHF-H-NLETLHGYIFAVDTLKSIVLACAVIEQKITVERAVALSRLEEEYQLKFWGRV

EWAHDFSQELQARLAAAVLFVH-LII

>B4LRW5|B4LRW5_DROVI

--ATPPKRFYKTTNVLC-TAGYEVTLDHRKLKTPNGAPFMVKS--LAIAVATEFDGQKDHIERSRMHISA

LCFTAIDNP-TKL-DMVNYLLNFVATDTVLFQYDD----QELQRNEWDPLIEWFNQRFETNLQKTMNITP

PQVSEVDKMKIAKHL-Q-SLETLHGFVFAVDTLKSIVLACAVIDQQITVERAVALSRLEEEYQLKFWGRV

EWAHDFSQELQARLAAAVLFVH-FIL

>B4N7P6|B4N7P6_DROWI

--ASPPKRFYKKTSVLC-TNGYEITLDHRKLKTPKGTLFTVKS--LAIAVATEFDSQKEHIERSRMHLSA

LCFTALDNP-SKL-DMVNYLLNFIATDTVLFQYDD----QDLQHNEWDPLIAWFNQRFETNLQKTMNITP

PLVTDEDKIKIAKHF-Q-NLETLHGFIFAVDTLKSIVLACAVIEQQIPVEKAVALARLEEEYQLKFWGRV

EWAHDLSQELQARLAAAVLFVH-FIL

>B5X6N9|B5X6N9_SALSA

SIVTERKKFYEDVSISH-GGGFEINLDRRKLKTPGGKLFTAPN--LAIAVANEWDTQKDMLKFYSMHMNT

LCNTALDNP-SKD-QMITAALKYLETDTVCYRVEE----VELQNNEWDPVLNWIEDRYNVVIGSSTSILG

PEIPQATMDTFRQHL-G-NFWSLTGLEYVITQLKSVVLAFALIDKHITVEQAVLLSRLEEEFQIGHWGNV

EWAHDVDLELRSRTAAGALFVH-FQD

>B6DTF4|B6DTF4_9EUGL

RASGAVRVFWTDVAVVPFPGWFAVAVDGRKVKAFSQRVLALPNEELALACAREYAAQKGHINKLLMPITD

LCSGAMQVAPQAIQPRIDYLMTFYQNDNCYFRAA---AIAAKQDEMITPITNWFSRVFDVDVPRVLGIGH

PGITPQSVEKVREALIALNPYQVVAMCVVAQFTSSIMLPLALFNRVVDLPTAFAINRAEEGHNIATAGMI

EGYHDIREDSVVKICASATAWQ-LPY

>B6HKH8|B6HKH8_PENCW

LTAALKKRFWKDVHVHG-LDGYQVLLDKRPVRTPMKEVLSIPSTKLAHAVALEWDVMTSALKSHSIPMTS

LAARATDIASTTRKQIITTAMRYLDTDTLLCWEPEIETLRQIQTRIAGNVMSFLSTKVGLEIVPINSILP

LSQPKGTKDSICTWVSELSAFDLAGLERAILASKSLLIAVRLVVEKFGIEEAAEASSLEVTWQTDMWGEV

EDTHDVGKDLKRQLGSVVVLVS-GTR

>B6QV24|B6QV24_PENMQ

KTSPLKKRFWKDVDVKE-ADGYQILLDTRPVRSPTKTILTVPSNKLAEAISLEWDLLTSALKQHLIPLTS

LTTRAADIVNRIRQEIARTAMRYLETDTLLCWVPEEETLREKQVRVAREIINFLTRTIGVEIKPVNSIIP

MSQDEKTLETIRSWISALPPYELAGLERGILASKSLLVAVRLVVERFGVDEAACASSLEVTHQTDQWGEV

EDTHDVDRDLRRQLGSVILLVS-GRR

>B6T754|B6T754_MAIZE

TGSVVGKRFYRDATVRRAGNGWTVMLDYRTLKSPAKRVLKLPSRALAMAIAAEWEYQESGIRPFTMPLMK

LACTALERVPLTRRKVIDNLMKKFHQDLVFCRSPADSELHQKQKEKIDPILEWVDTEFGFKPVVYTSFFG

GKQDEGLAKAVETVLKKATDCELASIDAMAAAAHSLVIPLAIFRERLGIEEAIELIRLEEDHQVDRWGLV

EGGHDVDIDLKVQMSSAVVFLGL-GL

>B7GEH3|B7GEH3_PHATR

TRLAGRRRFYKIVGTTPSFDWHGVTLDGRVLRTPLGQPLSVPSVTLATMIAAEWNAQTPYIVPTQMPLMT

LACTALDQTSRQMRTYQETSLNFVGTDTICYWEDPMED-YQAQERLWGPIHELVKQQ-GHALAQTLGASK

LFHPPALYDYAREFVAQLDAWQLTTLHACAAEAKSFWLAWSLLMHSLMVDLAIQAARVEEEYQIANWGLV

EGGHDYDRNSSIQIRSARAMLDC-KV

>B8B545|B8B545_ORYSI

TGSVVGKRFYREATVRRAGNGWSVMLDYRTLKSPAKRPLKLQSRTLAMAIAAEWEYQEAGIRPFTMPLMK

LACTALERVPLTRKKIIDNLMKKFHQDLVFCRSPDDNELYQRQKEKIDPILDWVNTEFGFKPVVYTSFFG

GKQDEGLANAVETVLKNTTDFELASIDAMAAAAHSLVIPLAIFRGKLGIEQAIELIRLEEDHQVDRWGLV

EGGHDVDIDLKVQMSSAVVFLLL-QL

>B8BZ98|B8BZ98_THAPS

RRLAGRQRFYKNVGIAPSSGWHTVTLDGRALRTPLGLPLTLPSAHLALAVASEWDAQEKVLRPAQMPLMT

LCCTAIDQVASDPSAHQQDVMRYLRNDTVCYWACPTED-HRRQTQAWEGLHKSLSMELGLS--DDLGMSG

LPHPPVLVEKAQQWVDSLDAWRLASLYSACAESKSFFIGAALVFEKVQTNAAVRAARVEEELNIETWGLV

EGGHDYDRNCSIQMHSASFLAQT-TA

>B8MTY2|B8MTY2_TALSN

KTSPLKSRFWKDVNVKE-SDGYQILLDTRPVRSPTKAILTVPNTKLAEAIALEWDFLTSALKQHLIPLTS

LTTRATDIVQRIRQEIARTAMRYLETDTLLCWVPEEETLRDKQVRVAKEIINFLTRTIGVEIKPVNSILP

TPQDETTLETIRNWVSGLPAYELAGLERAILASKSLLVAVRLVVERFGVEEAARASSLEVTHQTEMWGEV

EDTHDVDRDLRRQLGSVILLVS-GRK

>B9FUH3|B9FUH3_ORYSJ

TGSVVGKRFYREATVRRAGNGWSVMLDYRTLKSPAKRPLKLQSRTLAMAIAAEWEYQEAGIRPFTMPLMK

LACTALERVPLTRKKIIDNLMKKFHQDLVFCRSPDDNELYQRQKEKIDPILDWVNTEFGFKPVVYTSFFG

GKQDEGLANAVETVLKNTTDFELASIDAMAAAAHSLVIPLAIFRGKLGIEQAIELIRLEEDHQVDRWGLV

EGGHDVDIDLQSESTDRLVFFCLCRI

>B9SLS3|B9SLS3_RICCO

TGSIVGKRFYKKVTTREAGIGYTVMLDYRTLKTPSKKPLKLPTLSLAKAIAAEWDCQQTGIRPFTMPLMK

LACTALERVPLTRLKIIENLMKKFNQDLVFCRAPEDNDLYERQVEKIDPLLDWVKSEFGFKPVVYSSFFG

GKQEEGLVKAIEDLLKKTDNCELAAIDAIAASAHSLVIAIGIVRGKLDIEEAIQLIRLEEDLQVDRWGLV

EGGHDIDIDLRVQISSAAVFLGL-RT

>B9W716|B9W716_CANDC

KLSKIGKRFWKNGQVKF-TEKYEIQLDGKTLRTPLGYPLELPKNKLAYLIAHEWTHLPDKVKSSTLPLTS

LVTRAIDLS-LELQDIKLQMLKYLDTDTCLIFATNDGKLRKRQEEIYRPLIKEFNEFFSIELKYLDGLKG

NKQSKIIQEIVLNWLNQLPIYDLISLEKTILITKSFLCGIILLRSYKSLEELIELGNLETIYQTKQWGEV

EDTHDVDKDWLRNLASAALVCH-D-N

>C0ILU2|C0ILU2_HYDSY

YLKANKIRFYKNVDIHP-DNGYLIQLDNRTIKTPLLNQLCVPT--LAVAVANEWFMQTEVIDQHNMPLTA

ICNTAIDNP-TQE-ELVDEILNFFHTDTICALTEE----MWLQKEKWTPIHEWFSKKFNVEVNASSDLFG

LYQPDHTISTMKNFL-M-NRWQLCGVQAAVDSIKSFILPLCVIKKHISIEEALYLSRLELEFQIEKWGNV

EYAHDIDRNQQSLLTTAVLVFH-LLP

>C0S0S7|C0S0S7_PARBP

FTPRSPLRLWVDSHTRH--KGHPISVRGPPPKPPPSSEFLERIEQMQQENVSEVQQGTKLLKRRDVHVKE

VAARAQDVADTIRNEILKTTMRYLDTDTLLTWAPEQESLRDIQIKTAMPIINFLTEKVGIEIKPADSIIP

TPQTQITKDVIRGWIYGLPAYELAGLERAVLASKSLLVAARFIVEEFGIEKAAEASTLEVTWQTGKWGEV

EDTHDVDRDVRRQLGSVVLLGS--SK

>C1ECJ0|C1ECJ0_9CHLO

RGDGTAPRFYKKVEVVRVGGGWGVALDGRALKTPKRAALAVPSKSLAMAIAAEWEWQSGSIRPFTMPLMA

LVATSIDQMEEVRDFHVRKLLEFFPTDVVLIKHE-PGKLADRQAEIHAPILKWARSELGPGVEPTESLYG

AQIPEEAMAAAEKRLRAMDPFELTATFNAAASAKSLLTGMALIRGAIDVEQAEMSARVEEDFQIDEWGLV

EGGHDIDKDIAVRLAAPRALMSL-TL

>C1GAJ0|C1GAJ0_PARBD

TISLLKRRFWKDVHVKE--DGHQVLLDSFPVRTPEKKIITIPPSKLAHAIALEWDLLVSALKHHLIPLTA

LTARAQDVADTIRNEILKTTMRYLDTDTLLTWAPEQESLRDIQIKTAMPIINFLTEKVGIEIKPADSIIP

TSQTQITKDVIRGWIYGLPAYELAGLERAVLASKSLLVAARFIVEEFGIEKAAEASTLEVTWQTGKWGEV

EDTHDVDRDVRRQLGSVVLLVS-GRR

>C1HC34|C1HC34_PARBA

TLRLLKRRFWKDVHVKE--DGHQVLLDSRPIRTPEKKIITIPPSKLAHAIALEWDLLVSALKHHLIPLTA

LTARAQDIADTIRNEILKTTMRYLDTDTLLIWAPEQESLRDIQIKTAMPIINFLTEKVGIEIKPSDSIMP

TPQPQITKNVIRGWIYGLPAYELAGLERAVLASKSLLVAARFIVEEFGIEKAAEASTLEVTWQTGKWGEV

EDTHDVDRDVRRQLGSVVLLAA-W-L

>C1N6I5|C1N6I5_9CHLO

RGDGTAPRFYKSVSVVPQPGLWGIALDGKTLKTPSKAPLAVPSKALALAVAAEWEWQSGSIRPFTMPLMA

LVATAMDQMPAIRAQHIHTLLEFFPTDVVLCRHE-PSPLADYQAIAHEPVLRWARKELG-DVTPTESIFG

AEIPEEVTRAASKRLDAMDAFELTATFNACASAKSLLIGLALVRGAITVEEATRAARAEEDFQTEEWGLV

EGGHDVDADVSVRLRAPRAMMSL-VG

>C3XYY2|C3XYY2_BRAFL

QMAAARKRFYKNVSISQ-SGMYEINLDRRKLKTPMSRLFSVPN--IAIAVATEWEAQHKEIKMSQMHMLP

FMVC-----------------CF--FPTISYREES----AELEEREWEPLLDWVRQKYDIQLQSSTSIHG

PTIPKATKSTLKKHL-L-NHWSLVGVTSLVETLKSVVLSLALMDKHLTVEKAVALARLETEFQIMRWGNV

EWAHDMELDLRARAAASALFIH-YSA

>C4JZG7|C4JZG7_UNCRE

KTSPLRKRFWKDVHVKE--EGYQVLLDSRPIRTPAKTILTIPRSKLAHAIALEWDQLVSALKHHLIPLTS

LAARAEDIITTIRNEILRTLMRYLDTDTLLSWAPEAESLRDVQKRTAQPIIGFLTTAVGVEIKPVDSILP

ISQPQITKEVIRGWISGLPAYELAGLERAVLASKSLLIGVRLIVETFGIEKAAEVSSLEVRWQTEQWGEV

EDTHDVEKDLRRQLGSAILLVS-GKQ

>C4R2T3|C4R2T3_PICPG

RLEKTLQTFWEKVDVAK-KDGFHVRLDGKSLKTPLGNQLVIPSAKLAYMVCNEWKNLSNKVKPHSVPLTS

LSTRAIDLG-GNIEDIKTDLLRYLDTDTVLIFSPKDGKLRRAQEEMYRPLIASMESYLKITLNFLDGFRS

NYQNEATKRAALNWMNKLNVWELVALEKATLTAKSFLCGVAIVRAPMDLESIARAASLEVIYQTERWGEV

EDTHDVDKDLRRNLAAAALVAY-EEN

>C4WTB3|C4WTB3_ACYPI

RRSYAINKFYKTTSIIE-NDSYGVLLDSSKLKTPLGKELIINN--LALAVAEEWEMQKEHIKTDPMHLTK

LCFLAVDNP-SEH-DVVQQILSYLETDTVFFISDA----EKLLMEKWMPLIQKFNGYFDTNLKPSKGIYV

ESLDAQTKALVEKYF-L-GFPALHGVLHAVETLKSLVLTTCCLHQDISVKDAVLLFKMEEEYQCTKWGRV

DWIKDYIDDSVIRLSAAMLFIY-YKQ

>C4Y0I0|C4Y0I0_CLAL4

RLEKTLTKFWDKVETSF-TQSFETHLDGKALKTPLGNILSVPANKLAYLVSHEWENLIDKIKPNSLPLTS

MVARATDLG-GDLQDIKYNILRYLDTDTCLIFTTLEGRLRNKQEELYRPLIEEFESYFKVELTYLDGIRG

NSQTITTQNIVLSWLDSLPIYDLVALEKAVLSSKSFLCGAAIVRSKKSVEEIVEMGNLETIFQTQEWGEV

EDTHDVDNDWLRSLSSAALVC----R

>C4YF43|C4YF43_CANAL

RLAKTGTRFWKKGEVKF-TQKYEIQLDGKTLRTPLGFPLELPINKLAYLIAHEWTHLPDKVKSSTLPLTA

LATRAIDLS-LALEDIKLQMLRYLDTDTCLIFATNDGKLRKRQEEIYRPLINEFNEFFSIELKYLDGLRG

NKQDETTQLVVLDWLNQLPIYDLIALEKTILTTKSFLCGITLLRSHKTLEELVELGNLETIYQTEEWGEV

EDTHDVDKDWLRNLASAALVCH-H-N

>C5DKF4|C5DKF4_LACTC

RASKTLQKFWNDVSVKE-DTHLTVMLDNKPLRTPLGNALSVSKDRLALLLQNEWSSMSSAIKPHSLPITS

IVSRCIDLEGGDRSVISESLLRYLDTDTLMCLSPREGALRAAQDELYLPVISQVEKFLPVKLQILHGLKG

NLQDKNTKEAALAYLNSLSMWDFAVFEKAVLTTKSFLCGLLLLHNKVTMEKIAQFATLETIYQVERWGEV

EDTHDVDKDVRRNINAASIVAF-K-E

>C5FTL5|C5FTL5_NANOT

KPSPLRKRFWKDVHVKE--GGHQIYLDSRPVRTPEKKILTVPASKVAHAIALEWDLLKTATKYHLIPMTS

LTGRAEDIATTIRDEITRVMLRYLETDTLLSWAPERETLREKQIKTAQPIITSLVSTVGVELKPTNSIMP

LPQSQETLDVIRGWLSTLSPYDLAGVERAGIATKSLLVGARVVIETFGIEEAAHASSLEVRWQTENWGEV

EDTHDVEKDLRRQLGSVILLVS-GRR

>C5GH26|C5GH26_AJEDR

ESKPLRRRFWKDVHVKE--DGYQIFLDSRPVRTPEKKILIIPASKLAHAIALEWDLLVSALKQHLIPLTS

LTARVQDLASTIREQIVASMMRYLETDTLLSWAPEEESLRDIQIRTARPIVDFLTTKVGIEIRPANSILP

SPQLPLTMEVIRGWIYGLPAYELAGLERGVLASKSLLVAARFVIETFGIEKAAEASTLEVTWQTDKWGEV

EDTHDVDKDVRRQLGSVVLLVS-GRR

>C5K9F3|C5K9F3_9ALVE

NTARAHKRFYDVVKVARNGGGWTVLLDGKRLSTPAKHRLALPSEGLAFAVAEEWAEQDKFIRPHFMPLMA

LAATTIDLTAKDMSTVVERNLHYLNTDLTCYGEY---P-------EWVEYRSFVSKEFDCKIASCRGISL

PKHSEGADAALRAYL--STPWELTAFDEMSRTAKSVVIALNYYLGNTSLEEACRASVLEELDNRGKWGTV

EGDHDVSDTLKMAMGAAKFF-AEEAD

>C5MEZ7|C5MEZ7_CANTT

KLAKTGARFWKKGDVYF-TKKYEIQLDGKTLRTPLGFPLELPESKLAYLIAHEWTHLPDKVKSSTLPLTA

LATRAIDLS-LALEDIKLQMLRYLDTDTCLIFATNDGKLRKRQEEIYRPLIAEFDEFFSIELKFLDGLRG

NKQGEKTQEVVLDWLDQLPIYDLIALEKTILTSKSFLCGITLLRSHKSLEDVVELANLETIFQTGEWGEV

EDTHDVDKDWLRNLASAALVC----H

>C5PB14|C5PB14_COCP7

AASPLRKRFWKDVHVKE-LKGYQVLLDSRPIRTPAKTILNIPRSKLAHAIALEWDQLVSALRHHLIPLTS

LTARAEDIVSTIRNEIMRTLMRYLDTDTLLSWAPQPRSLRDLQIRTAQPIIGFLTTVVGIEIKPVDSILP

VSQPQMTKEVIRGWITGLPAYELAGLERAVLASKSLLIGVRLVVETFGIEKAAEASSLEVKWQTEQWGEV

EDTHDVEKDLRRQLGSAVLLVS-GRR

>C6H3T2|C6H3T2_AJECH

SSKPLKRRFWKDVHVKE--DGYQIFLDSRAVRTPAKKILTIPASKLAHAIALEWDLLVSALKQHLIPLTS

LTARAQDIANTIRNQITKATMRYLDTDTLLSWAPNEKSLRDLQIRTARPIIDFLTAKVGIEIRPANSIMP

TPQLPLTKEVIRGWIYGLPAYELAGLERGVLASRSLLVAARFVIEEFGIEQAAAASTLEVTWQTDKWGEV

EDTHDVDRDVRRQLGSVVLLVG-GQR

>C7GS06|C7GS06_YEAS2

RLSKTSQKFWEKVSLNR-KGKIALQLDGRTIKTPLGNGIIVDNAKLAYLLKLEWSSLSSSIKTHSLPLTS

LVARCIDLQGGNSDVIKNQLLRYLDTDTLLVFSPMEGRLRNAQNELYIPIIKGMEEFLNIRLQILHGLRG

NQQSDIVKSAAKKYMSSLSPWDLAILEKTVLTTKSFICGVLLLENKTDMDNIVRAATLETIFQVEKWGEV

EDTHDVDKDIRRKIHTAAIAAF-K-Q

>C7YMZ6|C7YMZ6_NECH7

SSGPLRKRFWKDVAVKE-DGSLQVFLDTRPLRHPSKEIVCLPMSKLASALALEWDLLTSATRQHLIPLTS

LTCRALDIASEVRNAISTTLLRYLDTDSILCWAP-GESLRDVQKRIAEDIVSFLTTHVGITITPVHSILP

QAQSPGVREVVQGWIAGLDAFEIAGLERVALAGKSLIAAARFIVEKFGVEEASVATSLEVDWQTGQWGEV

EDTHDVNKDVRRQMGSVVLLVS-GKA

>C8VH85|C8VH85_EMENI

RPTVLKKRFWKDVDVKQ-GGDYQVLLDKRPVRTPSKSVLSIPSTKLAQAIALEWDVMNAALKNHTIPLTS

LTARAADIARVIRTQIVKTAMRYLETDTLLCWVPEQETLREAQMRVAKDVIAFLSTKVGIDIVPVNSIFP

ASQSQATKDIIRQWVEGLEAYDLAGLERGILASKSLLVAVRLVTERFGIEEAAEASSLEVRWQTDMWGEV

EDTHDVDKDLKRQLGSVIVLVA-GVQ

>C9SB68|C9SB68_VERA1

VKGGLKRRFWKEVRVEE-DGALQVMLDNRPLRHPTTNIIRLPTSKLASAVAIEWDLLTSATKQHLIPLTS

LICRAIDIE----EEVPSASAAAAAADGEAAPVE---PFSPDTERPAADVLAYLRAHVGVSIAPVNAIVP

RPQADGVRDIVKAWVSGLSAWELAGLERAVLAGKSVVAASRLVVEKFGVEQAAVATSLEVAWQTGHWGEV

EDTHDVEKDLRRQFGSVVLLVS-GRR

>D0A042|D0A042_TRYBG

SSRAAVRVFWKDVDVRKLEGWYTVLVDGRKVKAFSRGVLAIPSEAMAYACAREFSEQKDYLNKLLMPLSD

MCSGALTVAPQMITPRIDYLMSFYQNDNMYFRSP---PIVEEQDRIINPVTEWFSHAFEVSVPRIVGIGH

PLIPPRATFKVRDALLAMNPYQVVALCVAAQFTSSLILPLAVFNSIVDLPTALSINRAEERHNTRTEGII

EGYHDIRDDVVTKLCAAAVTWK-LVM

>D1H954|D1H954_VITVI

TGSIVGKRFYKQASTREAGNGWTVMLDYRTLKTPSKRPLKLPTLSLAKAIAAEWEYQQTGIRPFTMPLMK

LACTALERVPLTRMKIIEYLMKKFHQDLVFCRAPGDSDLLERQVEKIDPLLQWVESQFGFKPIVYSSFFG

GKQEDGLVNAIENLLKKTDDCELAAIDAIASAAHSLTIAIGIFRGKLQIEEAIELIRLEEDLQVDKWGLV

EGGHDVDVDLKVQISSAAAFLGL-RY

>D1Z4T4|D1Z4T4_SORMA

GGIGLKRRFWKSVTVVT-DDMNEIHLDSRALRRPTKSIIRLPLTKLASALAIEWDQLVSATKQHMIPLTS

LICRALDIATAIRDSIAKVLLRYLDTDSLLCWAPVGYTLRELQEEAYSTVVSFLTTRVGVTIVPVTSIMP

RQQEPGTREVVQGWMLGLSAWELAALERAALAGKSLLMAARLVVERWGIEEAATAVSLEVDWQTNQWGEV

EDTHDVEKDLRRQLGSAVLLCA-GKL

>D2HLB8|D2HLB8_AILME

---TERKRFYQNVSITQ-GGGFEINLDHRKLKTPQAKLFTVPS--LAIAVATEWDSQQDTIKFYTMHLTT

LCNTSLDNP-NKD-QLIRAAVKFLDTDTICYRVEE----VELQKNEWDPIIEWAEKRYDVEIGSSTSIMG

PSIPARTREVLVSHL-A-NMWALQGIEFVVTQLKSLVLTLGLMDLRLTVEQAVLLSRLEEEYQIQKWGNI

EWAHDYELELRARTAAGTLFVH-LQQ

>D2VDZ9|D2VDZ9_NAEGR

SY---NKKFYRKVYIEEQNPEFRVRIGQQLLRTTTNKVVTVPS--IAAVAAGEWELQEEYVRPATLPFTE

LLCRIEDVK-SVSYNMKKTIHGFFDGEFVCLRQGM----TNSIKEHWDPLVQWFNKEFNTELVVLEDFA-

DPQEDARIKFIERYTTD-SPSGLVLLNAMVEVTGSVVIASALYAGRINARQAALATQLPVREQTNTFGLV

MGEHDLQFEQFCKLSALELLLE-IVL

>D3AZW0|D3AZW0_POLPA

TSDGTSLRWYKHVGYT--ENGYLPLLDNRPMKTVNRKLFIVPT--IAMAIATEWMVQGKYIMPHRLPLTT

VAATCIDMN---RQKCIDELIGHLATDQVCNRDSD----KKLQNEAFDDLLHWSSDYYGKPFYLS--LD-

SRHPPSLLKTIREHL-E-NNWQLLCMQTLTTSTKSFLISLNLYYNRVRLDKLYKIVALEEEFQSETWGKI

PFGHDLAEETLNEIAPALFVLR-SKN

>Q00UT4|Q00UT4_OSTTA

RADGTAPKFYEEVSVRRDSGTWRVTLDERLLRTPRRNEYTFGTKALAVAIAMEWDAQTDHVAPFTMPLTQ

LSATALDHMRETRELHVETLLKHFRTDVVRVRSM-DEAVAKRQVETHAPILKWAEKEFG-EVEVSDSIFG

PETSDKTLEVLRKRLNAMCPWELTCAFALSAATKSLLIGLKTLRGELSVDEAIAAARVEEETQIEEWGLV

EGGHDLDQDIRVKVAAPVMLMKL-GE

>Q16TS9|Q16TS9_AEDAE

--PAPPKRFYRQTGIIT-SGKFEITLDQRKLKTPKGAPFFVES--LAIAIATEWDAQKDVIDRSRMHLTA

LSSTVIDNP-QKA-DIVNYLVNYASTDAILFQSNE----KELQVVEWNPVIEWFNKRYDVQLEATDALEV

PSFAPGTAMNISRYL-S-NEAALHGIMYAVDTLKSVILTCACVDRFIAVEKAVLLSRLEEEFQLGHWGRV

EWAHDVNMDLQARLSAAILFVY-FSI

>Q1LZ96|ATPF2_BOVIN

VPPAERKRFYQNVSISQ-GGGFEINLDHRKLRTPQGKLFTVPS--LAIAVATEWDSQQDTIKMYTMHLTT

LCNTSLDNP-DKD-QLIRAAVKFLDTDTVCYRVEE----VELQRNEWDPVISWAEKRYGVEIGSSTSITG

PSIPARTREVLVSHL-A-NMWALQGIEFVVTQLKSLVLTLGLTDLRLTVEQAVLLSRLEEEYQIQKWGNI

EWAHDYELELRARTAAGTLFVH-LQG

>Q22WQ9|Q22WQ9_TETTH

QGGQFMKKFYKKATVKK----YGVFLDGKLIRTPLKNKLALPTYELAFAIAHEFNMQNEYLKPATMPITS

ISRTTVDMD-NIRQHIEDSVNQFVRNDTILFREEG--KLGVIQNEKLNPVIEYVNKLMNIKLQPTDSLFS

RELEDIEIQNIQKYIA--DNWTLMAIEQATVNTKSTCLGVSLINGFLSIEQALEYSRLEENFQIEQFGMV

EGSHDLEETTLLNISTAKLFSY-LNL

>Q2KG00|Q2KG00_MAGGR

RASGLKRRFWRDCHVRE-DGAYEVHLDTRGLRHPTKEIVRIPLSKLAYALAVEWDQLESATKQHLIPLTS

LVCRAIDLAGSIRTSIIDTVLRYLDTDTLLCWAPAGRTLRQAQEEAARPIIAHLQTKVGVTIEPAGGLLP

KPQAPGVRDVIRGWLMGLSSWDLAGVERATLAGKGLLAATRLVCEAFGVEEAAQAVSVEVNWQTKVWGEV

EDTHDVEKDVRRQFGSVVLLVS---G

>Q4E1X8|Q4E1X8_TRYCR

RSRAAVRVFWKDVNVGPLEGWYTVLVDGRKVKAFSTHVLAIPNEAMAYCCAQEYAEQSGYLNKLLMPMTD

MCSGALTVSPQMIALRVDYLMSFYQNDNIYFRAA---PIAEEQDRVIGPIVDWFSRAFNVEVPRIVGIGH

PHIPIGSKLKVRDALLAMNPYQIVALCVAAQFTSSLLLPLAMFNSVVDLPTALSINSLEERHNTSTEGAI

QGYHDIRDDVVTKLCACAVTWQVAAL

>Q4QE07|Q4QE07_LEIMA

RSSCAVRVFWKDVDVVELPGWFAVTVDGRKVKAMSSQVLAVPSEAMACCCAQEYAEQTGHINQLLMPMSD

ICSGALHIAPQMLTPRIDYLLSFFQNDNLYFRAA---PIAAKQDAMIAPIIAWFERVYEMDVPRVVGIGN

PHITPHATAKMRDALIAMNPYQVLAMCVTAQFTSSLLLPLALFSGVVDLPTALAINRAEEQHSISEAGLV

AGYHDIREDAVTKICACALTWK-LAV

>Q4S4H0|Q4S4H0_TETNG

TAASERKRFYQDVTISQ-GGGFEINLDRRKLKTPGGKLFTVPN--LAIAVATEWDAQKDTLKFYTMHLTT

LCNTALDNP-NKD-QMITAALKFLETDTVCYRVDE----VELQKNEWDPVLQWTENRYNVTIGSSSSILG

PDIPEATKDTLRQHL-N-NFWSLTGFEYVITQLKSVVLSLGIIDRHLSVEQAVLLSRLEEEYQIRCWGNV

EWAHDYDVELRARTSAGALFVH-LQD

>Q4UFR8|Q4UFR8_THEAN

--NNPKFPFLTKTPTII-NSGYNILLDNRILLTPLGNTLYTSN--VANLIIEEFKTNLNLTNFNDLPYTM

LLSKSIDLS--DKINHLKTLKESINSDSVLFFEKVPDELEMVQSFFFPGVLRSFSKLLNVPSLVTSSVSK

PRQHPDTVSVFESYI-D-NTFRLISTLKVLCNLKSIILSLLYLNGLITTTRCLRLSRIEETVQCSHWGLT

DSFGLEERIISELEKCSNFYKL-N-P

>Q4V7H3|Q4V7H3_XENLA

AVAVERKKFYENVSISH-GGGFEINLDRRKLKTPQGKLFAVPS--LALAVATEWDCQRDVIKFYTMQLTT

LCNTALDNP-NKE-QLIKPALKFLETDTICYRVED----VELQKNEWDPVIEWAEKRYNVVIGSSTSIHG

PIIPTETKDVFSRHL-A-NSWGLLGIEFIISQLKSLILTMGLIDRHLPVEKAVLLSRLEEEYQIQRWGNV

EWAHDYDLELRSRTAAGTLFVH-LQD

>Q4XAC3|Q4XAC3_PLACH

NILKMPLTL--NNLID-----FINPKEGIGVKTKSMNTIQTENNNQINNLEKEEKSSTHHVDKECRNGTE

EFSSNFSNCDIQRALIEKNIYDHFKTDLIFYRSDEINSFREEENNIYNKFMNMFENIHKIKLNSAKNFE-

PEQDEHVHKTIQNLI-K-NNSEIFIFYKCTQILNSFIFSYLFLKGYINYKDVYRYCNLEYIYQFSKWGYV

YDINSVKDSSLLTLSSLMIMRA-INS

>Q54CV4|Q54CV4_DICDI

--DGSSLRWYKTAGMCREPQGYYPLIDERKIRTPSNHVIITPS--IAYAVAAEWRAQEKYIKPSRLPITQ

TIISCLDVR---RFKIIGEFINHLATDPICNREKN----KKLQSELYEPILQFANEYYGIPFSIS--LS-

SKHPKELLDKIERHL-H-NNWELVCLQLISQSSKSFLVALSLYYGKLRLDNLYQTIALEEEYQSETWGRI

PFGHDLAEETHNEIAPPLFMLR-NPK

>Q5KKG8|Q5KKG8_CRYNE

RAEQTLRRFWKTVNISA-SDGYLITLDHRALKTPFGAKLEIPKERLAALIANEWENQD-VLKQHALPVTS

LASRAIDGL-PTRPAVIEALLQYLETDTILYPDDAPPPLVRLQKKHWDPLYEWLKEDFGVELQLAQGFDA

VKQSDDNMEKLKRVVETMDGWELAAFERAVYATKSFVIALALCR-RLTAHEAAQASHVEVSSQIERWGEV

EDTHDVDYDIRRALGSAACLLI--KS

>Q6BVJ6|Q6BVJ6_DEBHA

RLSKTLTKFWEKVDAVH-LNQYEVQLDGKTLKTPLGFPLSLPAEKLAHLVAHEWANLPDKVKINALPLTS

LSSRAIDLY-GNVDDIKVNLLRYLDTDTCLIFTILEGQLRSKQDELYFPLIKEFEDYFRVTLNFLDGLSG

NRQSITTQNIVMSWMNDLSMYELVALERAILTAKSFLCGASLLRSHKTVEELVELGNLEIIFQTEEWGEV

EDTHDVDKDWLRHLSSSALLT----Y

>Q6CBE8|Q6CBE8_YARLI

IQVKGLKRFWEELAVKD-NGNITVTLGGKSLRTMGQHDLILPATKLAHLLMHEWQVLPSKLKNHSVPLTS

LVSRAIDIS--VRDAAIKDIMPYIDTDALLIFEPSQGRLRAAQENDFRPVIADAEKFWGVTLKSMKGLLG

NRQTAEDKEAVKQWAYTLSPWQLAALERATLTSKSFICGAFLIS-KLTPTQVAELVGLETKFQVERWGEV

EDTHDVDFDIRRHLASCSLLAR---M

>Q6CXR0|Q6CXR0_KLULA

RLSKTLTKFWEQVSLEE-PENVTIKLDSKPLRTPLGNPLTLPQSRLSVMVLNEWSNLPSSVKPYVLPLTS

LVSRCIDLEGGNREKLTEGLLRYLDTDTLLVFSPAEGALRKAQDELYLPIISGVEKFLEVKLQILHGLRG

NAQSGETREVAKQYLDSLSVWDLAIFEKTVLTTKSFICGLLLLLNQVSMEDIARAATLETIYQVERWGEV

EDTHDVDKDVRRNIHAAAIVAF-GSK

>Q6FSV6|Q6FSV6_CANGA

KLSKTGQKFWDQVGLDF-GDKITVQLDSKPLRTPLGNNLAIDHDRLGLMLKKEWSNLQEASKKFSLPLTS

LVSRCIDLEGGDTTVIKNQLLRYMDTDTLLVFSPAEGALREEQDKLYLPIIKKIEEFLQLTLQILHGLRG

NVQSQEVKDAAMNYMDSLSPWDLAVFEKTVLTTKSFICGILLMESVKSLDEIIRLATLETIFQVERWGEV

EDTHDVDKDIHRKISSAAIVAF-K-N

>Q6P847|Q6P847_XENTR

AAATERKKFYENVSISH-GGGFEINLDRRKLRTPQGKIFTAPS--LAVAVATEWDCQRDVIKFYTMHLTT

LCNTALDNP-NKE-QLITAALKFLETDTVCYRVEE----VELQRNEWDPVIEWAEKRYNVVIGSSTSIQG

PIIPTETKDVFSRHL-A-NSWGLLGIEFIISQLKSLVLTMGLIDRHLPVEKAVLLSRLEEEYQIQRWGNV

EWAHDYDLELRSRTAAGTLFVH-LQD

>Q758A0|Q758A0_ASHGO

RLAKTFTKFWDKVDLAR-GGHVTVHIDGKPVRTPLGSPLRVDERRLAHMLREEWAGLTSAVKPYSLPLTS

LVSRCIDLEGGDRSAISQGLLRYLDTDTLLCFSPREGALRKAQDEMYKPIIAGVETLLPVSLRCLHGLRG

NVQSEDTRAAAGKYMDNLSVWDFAVFEKVVLTTKSFICGILLLQNKLSTEEIARAATLETIYQVERWGEV

EDTHDVNHDIRRNINAAAVVAY-R-E

>Q7PWR4|Q7PWR4_ANOGA

--AAPPKRFYRNTGVIS-SGRFEITLDSRKLKTPRGLPFYVES--LAVAIAMEWDAQKDVIDRSSMHLTA

LSSTVIDNP-QKH-DMVNYLVNYINTDTVLFHSSE----KKLQSQEWTPIVDWCNKRYEINLASTDSLVV

PTFEPGMAMNLSRYF-S-NTAALHGFVFAVDTIKSIILTMACADRYISIEKAVQLARLEEEFQQGHWGKV

EWAHDIQRDSQARLSAAVLYIY-FSL

>Q7SHX7|Q7SHX7_NEUCR

GIGLLKRRFWKSVHVAT-NDMNEIHLDSRPLRRPTKSIIRLPLTKLASALAIEWDQLVSATKQHLIPLTS

LVCRALDIATDIRNAIATVLLRYLDTDSLLCWAPAGYTLREVQEEAYSSVVSFLTTRVGVTIVPVTSIMP

RQQEPGTREVVQGWMLGLSAWELAALERATLAGKSLLVAARLVVERWGVEEAAKAVSLEVDWQTTQWGEV

EDTHDVEKDLRRQLGSAVLLCA-GKL

>Q8N5M1|ATPF2_HUMAN

APPTERKRFYQNVSITQ-GGGFEINLDHRKLKTPQAKLFTVPS--LAIAVATEWDSQQDTIKYYTMHLTT

LCNTSLDNP-NKD-QLIRAAVKFLDTDTICYRVEE----VELQRNEWDPIIEWAEKRYGVEISSSTSIMG

PSIPAKTREVLVSHL-A-NTWALQGIEFVAAQLKSMVLTLGLIDLRLTVEQAVLLSRLEEEYQIQKWGNI

EWAHDYELELRARTAAGTLFIH-LKE

>Q91YY4|ATPF2_MOUSE

VPPTERKRFYQNVSISQ-GGGFEINLDHRKLKTPQAKLFTVPS--LAIAVATEWDSQQDTIKFYTMHLTT

LCNTSLDNP-SKD-QLIRAAVKFLDTDTICYRVEE----VELQKNEWDPVIEWAEKRYGMEIGSSTSIMG

PSIPTQTREVLTSHL-S-NMWALQGIEFVVAQLKSMLLTLGLIDLRLTVEQAVLLSRLEEEYQIQKWGNI

EWAHDYELELRARTAAGTLFVH-LQE

>Q9FM31|Q9FM31_ARATH

TGSIVGKRFYKKVTTREAGNGWTVMLDYRTLKTPSKRPLKLRSLALAKAIAAEWEYQLTGIRPFTMPLMR

LACTALERVPLTRSKIIEHLSRKIHQDLVFFRAPEDNDLHDIQVESIDPLLEWIESEFRVKPKVYSSIFG

GKQDDKLVKAVEELLKKTNDGELASIDALQASAHSIVIALGIFCGKLQIDDAIKLIRLEEDLQVDKWGLV

EGGHDIDVDLKVQISSATVFLAL-EN

>Q9U2U3|Q9U2U3_CAEEL

SALTKPKKFYKEVSVINTGQIHKVLLDHRVLKTQGGQVLKLDS--LALAIAEEWSSQDEFLQLGQMRLTG

LAFTAQDNP-QTADTISQKILDYVEGDTVLFFNTE----HRYQEEKWAPLIKNLNNDLGIKVRPSENILD

CDASENDKEKIDRWI-R-NFPALVGLQYATESVKSFVIAYNAIRHHIDPDTAIDAATLEQRTQAETWGNV

EWAHGIERELMTRLSAACLFVY-FTV

>Q9UT16|ATP12_SCHPO

LPQPSFRRFWKNTATKI-QGEVLIQLDGRNLKSPSGKIVKVPKEMLAHLIALEWDRLPSSVRQHNLPITS

LVSRAIDIS---KELLSTQLIRFLDTDTILIYSPE----LEEQKENWWPLKETFENKLGVQLSYLAGII-

HKQTQETHERIRNWL-S-NSWQLAAFERSVSCCKSFIVSFMILKGYLNSEKAAALTNLELQYQTNRWGSL

EDDNEDLKNKLASSAILSCIED-MAH
