# Supplementary material for: Accurate Simulation and Detection of Coevolution Signals in Multiple Sequence Alignments
Source: PLoS One. 2012 Oct 16;7(10):e47108. doi: 10.1371/journal.pone.0047108 (PMC3473043; doi:10.1371/journal.pone.0047108)
Supplement: Text S1 — Flowchart/pseudocode of the db, dgb, and nbZPX2 algorithms. (PDF) [file pone.0047108.s020.pdf]

Flowchart/pseudocode of the db, dgb, and nbZPX2 algorithms.

The first step in the application of these algorithms using MSAvolve is the conversion of a MSA from ascii format to MATLAB integers representation (1-20 for all aa's, 25 for gaps) , called here NMSA. This can be accomplished with two MSAvolve functions:

aln_to_nmsa (if the alignment is in Clustal format)

faln_to_nmsa (if the alignment is in Fasta format)

For example, at the Matlab prompt type:

```
[~,nmsa] = faln_to_nmsa(msa.fasta);
```

ZPX2

1. Calculate the MI matrix for an MSA (function NMSA_to_MI in MSAvolve)

   $MI_{ij} = H_i + H_j - H_{ij}$

2. Calculate the MIP values from the MI values (function MI_to_MIP in MSAvolve):

   $MIP_{ij} = MI_{ij} - (\overline{MI_i} \times \overline{MI_j} \div \overline{MI})$

3. Calculate the ZPX2 values from the MIP values (function MIP_to_ZPX in MSAvolve):

   $$ZPX2_{ij} = \frac{MIP_{ij} - \overline{MIP_i}}{\sigma MIP_i} \times \frac{MIP_{ij} - \overline{MIP_j}}{\sigma MIP_j}$$

If using MSAvolve, at the Matlab prompt type in sequence:

```
[MI] = NMSA_to_MI(nmsa);
[MIP] = MI_to_MIP(MI);
[~,ZPX2] = MIP_to_ZPX(MIP);
```

dbZPX2 (function NMSA_to_dbZPX2 in MSAvolve)

1. Calculate a distance matrix for the sequences of the msa.
2. Reorder the msa by placing as the 1st and 2nd row the two sequences most similar to each other.
3. Place in the 3rd row the sequence most similar to the sequence in the 2nd row.
4. Loop by placing in each consecutive row the sequence most similar to the sequence in the previous row, until the entire msa is reordered.
5. Convert the msa to 'binary differential': the 1st row is assigned all O's. In each consecutive row a 0 is placed at every position at which the aa is the same as in the previous row, and a 1 at every position in which the aa is different.
6. Calculate the MI matrix for the 'binary differential' msa.
7. Calculate the positional covariance (COV) matrix for the 'binary differential' msa.
8. Scale MI and COV matrices by linear regression.
9. Sum scaled MI and COV matrices.
10. Calculate a ZPX2 matrix from the summed matrix.

If using MSAvolve, at the Matlab prompt type:

```
[dbZPX2] = NMSA_to_dbZPX2(nmsa);
```

dgbZPX2 (function NMSA_to_dgbZPX2 in MSAvolve)

1. Calculate a distance matrix for the sequences of the msa.
2. Reorder the msa by placing as the 1st and 2nd row the two sequences most similar to each other.
3. Place in the 3rd row the sequence most similar to the sequence in the 2nd row.
4. Loop by placing in each consecutive row the sequence most similar to the sequence in the previous row, until the entire msa is reordered.
5. Convert the msa to 'binary differential': the 1st row is assigned all O's. In each consecutive row a 0 is placed at every position at which the aa is the same as in the previous row, and a 1 at every position in which the aa is different.
6. Calculate the MI matrix for the 'binary differential' msa.
7. Convert each row of the reordered msa to 'long binary' format in which every symbol (including gaps) is represented by a vector of twenty 0's and a single 1.
8. Convert the 'long binary' format msa to 'binary differential as in step 5.
9. Calculate the positional covariance matrix (largeCOV) of the msa in 'long binary format'.
10. Calculate the Frobenius norm of every submatrix of largeCOV that corresponds to the covariance between two columns of the reordered msa. This generates a positional covariance matrix (COV) of the reordered msa.
11. Scale MI and COV matrices by linear regression.
12. Sum scaled MI and COV matrices.
13. Calculate a ZPX2 matrix from the summed matrix.

If using MSAvolve, at the Matlab prompt type:

```
[dgbZPX2] = NMSA_to_dgbZPX2(nmsa);
```

nbZPX2 (function NMSA_to_nbZPX2 in MSAvolve)

1. Calculate a distance matrix for the sequences of the msa.
2. Reorder the msa by placing as the 1st and 2nd row the two sequences most similar to each other.
3. Place in the 3rd row the sequence most similar to the sequence in the 2nd row.
4. Loop by placing in each consecutive row the sequence most similar to the sequence in the previous row, until the entire msa is reordered.
5. Convert the msa to ‘binary differential’: the 1st row is assigned all O’s. In each consecutive row a 0 is placed at every position at which the aa is the same as in the previous row, and a 1 at every position in which the aa is different.
6. Go back to the reordered msa in standard format and change to 0 every position that is a 0 in the ‘binary differential’ msa: the result is the ‘normal binary’ msa or nb_msa.
7. Calculate the MI matrix for the nb_msa.
8. Calculate a ZPX2 matrix from the MI matrix.

If using MSAvolve, at the Matlab prompt type:

```
[nbZPX2] = NMSA_to_nbZPX2(nmsa);
```
