# Supplementary material for: Accurate Simulation and Detection of Coevolution Signals in Multiple Sequence Alignments
Source: PLoS One. 2012 Oct 16;7(10):e47108. doi: 10.1371/journal.pone.0047108 (PMC3473043; doi:10.1371/journal.pone.0047108)
Supplement: Text S2 — Descriptions and simulation conditions for the 8 protein families (KDO8PS, ArsA, ArsC, PHBH, PDR, MDH, ATP11, ATP12) with experimental MSAs under 500 sequences. (DOCX) [file pone.0047108.s021.docx]

**Descriptions and simulation conditions for the 8 protein families with experimental MSAs under 500 sequences that were analyzed in this study.**

*KDO8P synthase*

KDO8P synthase is a bacterial protein involved in the 1^st^ step of lipopolysaccharide biosynthesis. Six different sets of MSAs, each one comprising 100 MSAs of 300 sequences, were developed under the following set of rules:

1. The reference experimental MSA consisted of 348 sequences (MSA S1, Supporting Information): the sequence numbering of *Neisseria meningitidis* (*Nm.*) KDO8PS (PDB 2QKF) was used as reference for the entire family, and only positions in the MSA with a corresponding residue in *Nm.* KDO8PS were retained, giving a total of 280 positions in each sequence. Although some gaps are present in this MSA, no gaps were allowed in the sequences of the simulated MSAs.

2. The ancestral sequence was based on a) the background probability of aa’s at the positions of the experimental MSA of KDO8PS; or b) the emission probability of aa’s at each position in a Hidden Markov Model (HMM) of the experimental MSA.

3. Covarying positions (~10% of all positions) were selected among the positions in the experimental MSA with a) low relative entropy, or b) intermediate levels of relative entropy.

4. Nine recombination zones were implemented, as defined by crossover points at positions [1] 20 61 89 167 193 216 235 259 [280]. The zones were identified by means of the SCHEMA algorithm [[1-4](#_ENREF_1)]. SCHEMA uses the structure of one or a few proteins in the family to find crossovers that partition the structure into a set of fragments that maximize the number of conserved amino acids at their interfaces, and minimize the number of broken interactions when a fragment is swapped between two sequences. In the current version of MSAvolve, recombination is implemented as the spread of a fragment from a sequence to one or more other sequences. No reciprocal swaps are allowed.

5. The relative amount of point mutations (which decrease the similarity between sequences in the MSA) and recombination (which increases the similarity between sequences) was fine tuned in order to reproduce the overall level of similarity among the sequences of the experimental MSA.

*Arsenic transporter ArsA*

ArsA is the catalytic subunit of the bacterial arsenic transporter (ArsAB ATPase, [[5](#_ENREF_5),[6](#_ENREF_6)]). The representative X-ray structure for the protein family (*Escherichia coli* ArsA, PDB 1IHU) was determined by Zhou *et al.* [[7](#_ENREF_7)].

1. The reference experimental MSA consisted of 202 sequences (MSA S2): the sequence numbering of *Escherichia coli* ArsA (UniProt P08690; PDB 1IHU) was used as reference for the entire family, and only positions in the MSA with a corresponding residue in this sequence were retained, giving a total of 583 positions in each sequence.

2. Gaps were allowed in the simulated MSAs reflecting the presence of gaps in the middle of the experimental sequences, but not in the flanking regions (C or N terminal).

2. Covarying positions (~15% of all positions, 87 pairs) were selected among the positions in the experimental MSA with medium levels of relative entropy, including positions that may contain gaps.

3. Eighteen recombination zones were defined by crossover points at positions [1] 19 45 86 114 148 206 228 280 302 337 361 388 423 453 503 519 567 [583].

*Arsenate reductase ArsC*

ArsC is a small (14 kDa) reductase that converts arsenate to arsenite, the substrate of the ArsAB pump (see above). This function of ArsC is particularly beneficial to cells, which become resistant also to the pentavalent state of arsenic. ArsC type proteins are present both in Gram negative and Gram positive bacteria, although their amino acid sequence is only marginally related (< 20% similarity). Arsenate reduction by ArsC requires glutathione (GSH) and glutaredoxin [[8](#_ENREF_8)], a small protein with redox active sulfhydryls that participate in the transfer of electrons from GSH. High resolution structures of ArsC in complex with sulfate, arsenite and arsenate were obtained [[9](#_ENREF_9)].

1. The reference experimental MSA consisted of 294 sequences (MSA S3): the sequence numbering of *Escherichia coli* ArsC (UniProt P08692; PDB 1JZW) was used as reference for the entire family, and only positions in the MSA with a corresponding residue in this sequence were retained, giving a total of 141 positions in each sequence.

2. Gaps were allowed in the simulated MSAs reflecting the presence of gaps in the middle of the experimental sequences, but not in the flanking regions (C or N terminal).

2. Covarying positions (~15% of all positions, 21 pairs) were selected among the positions in the experimental MSA with medium levels of relative entropy, including positions that might contain gaps.

3. Nine recombination zones were defined by crossover points at positions [1] 9 12 34 65 93 107 127 137 [141].

*p-Hydroxybenzoate hydroxylase (PHBH)*

PHBH is a flavoprotein hydroxylase that catalyzes the monooxygenation of *p*-hydroxybenzoate to 3,4-dihydroxybenzoate [[10](#_ENREF_10)]. It has been studied extensively both kinetically and crystallographically due to the unique dynamic properties of both the protein itself and its ligands [[11-13](#_ENREF_11)].

1. The reference experimental MSA consisted of 183 sequences (MSA S4): the sequence numbering of *Pseudomonas aeruginosa* PHBH (UniProt P20586; PDB 1DOB) was used as reference for the entire family, and only positions in the MSA with a corresponding residue in this sequence were retained, giving a total of 394 positions in each sequence.

2. Gaps were allowed in the simulated MSAs reflecting the presence of gaps in the middle of the experimental sequences, but not in the flanking regions (C or N terminal).

2. Covarying positions (~15% of all positions, 59 pairs) were selected among the positions in the experimental MSA with low and medium levels of relative entropy, including positions that might contain gaps.

3. Thirteen recombination zones were defined by crossover points at positions [1] 12 45 69 102 159 184 210 237 269 293 343 385 [394].

*Phthalate dioxygenase reductase (PDR)*

PDR, a soluble iron-sulfur flavoprotein, is a member of the FNR family of flavoprotein reductases [[14](#_ENREF_14)]. It is characterized by a modular structure with an N-terminal FMN binding domain, a central NAD binding domain, and a C-terminal [2Fe-2S] domain similar to plant ferredoxins [[15](#_ENREF_15),[16](#_ENREF_16)]. PDR is responsible for the transfer of reducing equivalents from NADH to a dioxygenase that converts phthalate to its cis-dihydrodiol, with incorporation of molecular oxygen.

1. The reference experimental MSA consisted of 271 sequences (MSA S5): the sequence numbering of *Pseudomonas (burkholderia) cepacia* PDR (UniProt P33164; PDB 2PIA) was used as reference for the entire family, and only positions in the MSA with a corresponding residue in this sequence were retained, giving a total of 321 positions in each sequence.

2. Gaps were allowed in the simulated MSAs reflecting the presence of gaps in the middle of the experimental sequences, but not in the flanking regions (C or N terminal).

2. Covarying positions (~15% of all positions, 48 pairs) were selected among the positions in the experimental MSA with low and medium and high levels of relative entropy, including positions that might contain gaps.

3. Fifteen recombination zones were defined by crossover points at positions [1] 13 43 57 81 103 123 145 171 199 223 245 277 291 309 [321].

*(S)-mandelate dehydrogenase (MDH)*

MDH is an enzyme in the mandelate pathway of several strains of *Pseudomonas*, which converts (*S*)-mandelate to phenylglyoxalate. It is a member of a widespread family of homologous FMN-dependent α-hydroxyacid oxidizing enzymes, which includes also glycolate oxidase (GOX). While MDH is tightly bound to the bacterial membrane, GOX is soluble and we were able to determine the X-ray structure of a soluble chimeric protein MDH-GOX, in which 19 residues from GOX were grafted into MDH, without the use of detergents [[17](#_ENREF_17)].

1. The reference experimental MSA consisted of 391 sequences (MSA S6): the sequence numbering of *Pseudomonas putida* MDH-GOX chimera (PDB 1HUV) was used as reference for the entire family, and only positions in the MSA with a corresponding residue in this sequence were retained, giving a total of 353 positions in each sequence.

2. Gaps were allowed in the simulated MSAs reflecting the presence of gaps in the middle of the experimental sequences, but not in the flanking regions (C or N terminal).

2. Covarying positions (~15% of all positions, 53 pairs) were selected among the positions in the experimental MSA with low and medium levels of relative entropy, including positions that might contain gaps.

3. Thirteen recombination zones were defined by crossover points at positions [1] 22 48 76 106 126 153 213 228 252 286 305 335 [353].

*F_1_ assembly factors ATP11p and Atp12p*

We have determined the X-ray structure of two chaperones, Atp11p and Atp12p [[18](#_ENREF_18)], required for the assembly of the F_1_ ATPase, which is the catalytic component of the F_1_F_O_ H^+^-ATPase. While Atp11p appears so far to be involved only in the biogenesis of mitochondria, Atp12p is required for the assembly of both bacterial and mitochondrial F_1_ ATPase.

1a. The reference experimental MSA of Atp11p consisted of 178 sequences (MSA S7): the sequence numbering of *Candida glabrata* Atp11p (UniProt Q6FJS2, PDB 2P4F) was used as reference for the entire family, and only positions in the MSA with a corresponding residue in the X-ray structure were retained, giving a total of 205 positions in each sequence.

2a. Covarying positions (~15% of all positions, 31 pairs) were selected among the positions in the experimental MSA with low relative entropy.

3a. Ten recombination zones were defined by crossover points at positions [1] 10 29 43 64 91 107 124 137 162 184 [205].

1b. The reference experimental MSA of Atp12p consisted of 230 sequences (MSA S8): the sequence numbering of *Paracoccus denitrificans* (*Pden.*) ATP12p (UniProt A1B060, PDB 2R31) was used as reference for the entire family, and only positions in the MSA with a corresponding residue in *Pden.* Atp12p were retained, giving a total of 236 positions in each sequence.

2b. Covarying positions (~10% of all positions, 24 pairs) were selected among the positions in the experimental MSA with low relative entropy.

3b. Ten recombination zones were defined by crossover points at positions [1] 12 37 57 70 103 122 142 178 211 [236].

REFERENCES

1. Meyer MM, Hiraga K, Arnold FH (2006) Combinatorial recombination of gene fragments to construct a library of chimeras. Curr Protoc Protein Sci Chapter 26: Unit 26 22.

2. Meyer MM, Hochrein L, Arnold FH (2006) Structure-guided SCHEMA recombination of distantly related beta-lactamases. Protein Eng Des Sel 19: 563-570.

3. Li Y, Drummond DA, Sawayama AM, Snow CD, Bloom JD, et al. (2007) A diverse family of thermostable cytochrome P450s created by recombination of stabilizing fragments. Nat Biotechnol 25: 1051-1056.

4. Heinzelman P, Snow CD, Wu I, Nguyen C, Villalobos A, et al. (2009) A family of thermostable fungal cellulases created by structure-guided recombination. Proc Natl Acad Sci U S A 106: 5610-5615.

5. Gatti D, Mitra B, Rosen BP (2000) Escherichia coli soft metal ion-translocating ATPases. J Biol Chem 275: 34009-34012.

6. Rosen BP, Liu Z (2009) Transport pathways for arsenic and selenium: a minireview. Environ Int 35: 512-515.

7. Zhou T, Radaev S, Rosen BP, Gatti DL (2000) Structure of the ArsA ATPase: the catalytic subunit of a heavy metal resistance pump. Embo J 19: 4838-4845.

8. Shi J, Vlamis-Gardikas A, Aslund F, Holmgren A, Rosen BP (1999) Reactivity of glutaredoxins 1, 2, and 3 from Escherichia coli shows that glutaredoxin 2 is the primary hydrogen donor to ArsC-catalyzed arsenate reduction. J Biol Chem 274: 36039-36042.

9. Martin P, DeMel S, Shi J, Gladysheva T, Gatti DL, et al. (2001) Insights into the structure, solvation, and mechanism of ArsC arsenate reductase, a novel arsenic detoxification enzyme. Structure 9: 1071-1081.

10. Entsch B, Ballou DP (1989) Purification, properties, and oxygen reactivity of p-hydroxybenzoate hydroxylase from Pseudomonas aeruginosa. Biochim Biophys Acta 999: 313-322.

11. Gatti DL, Palfey BA, Lah MS, Entsch B, Massey V, et al. (1994) The mobile flavin of 4-OH benzoate hydroxylase. Science 266: 110-114.

12. Gatti DL, Entsch B, Ballou DP, Ludwig ML (1996) pH-dependent structural changes in the active site of p-hydroxybenzoate hydroxylase point to the importance of proton and water movements during catalysis. Biochemistry 35: 567-578.

13. Wang J, Ortiz-Maldonado M, Entsch B, Massey V, Ballou D, et al. (2002) Protein and ligand dynamics in 4-hydroxybenzoate hydroxylase. Proc Natl Acad Sci U S A 99: 608-613.

14. Karplus PA, Daniels MJ, Herriott JR (1991) Atomic structure of ferredoxin-NADP+ reductase: prototype for a structurally novel flavoenzyme family. Science 251: 60-66.

15. Correll CC, Batie CJ, Ballou DP, Ludwig ML (1992) Phthalate dioxygenase reductase: a modular structure for electron transfer from pyridine nucleotides to [2Fe-2S]. Science 258: 1604-1610.

16. Gassner GT, Ludwig ML, Gatti DL, Correll CC, Ballou DP (1995) Structure and mechanism of the iron-sulfur flavoprotein phthalate dioxygenase reductase. FASEB J 9: 1411-1418.

17. Sukumar N, Xu Y, Gatti DL, Mitra B, Mathews FS (2001) Structure of an active soluble mutant of the membrane-associated (S)-mandelate dehydrogenase. Biochemistry 40: 9870-9878.

18. Ludlam A, Brunzelle J, Pribyl T, Xu X, Gatti DL, et al. (2009) Chaperones of F1-ATPase. J Biol Chem 284: 17138-17146.
