# Supplementary material for: Accurate Simulation and Detection of Coevolution Signals in Multiple Sequence Alignments
Source: PLoS One. 2012 Oct 16;7(10):e47108. doi: 10.1371/journal.pone.0047108 (PMC3473043; doi:10.1371/journal.pone.0047108)
Supplement: Text S3 — In silico evolution of a protein family and simulation of multiple sequence alignments with MSAvolve. (PDF) [file pone.0047108.s022.pdf]

***In silico* evolution of a protein family and simulation of multiple sequence alignments with MSAvolve.**

MSAvolve v.1.0 is implemented as a Matlab Toolbox under BSD licensing. In order to run smoothly it requires the Bioinformatics, Statistics, and Curve Fitting Toolboxes for Matlab. Additional Toolboxes that are required for specialized functions are included with their own license: these include DCA, PSICOV, OMES, GLASSO, QUIC, DWINNEL_MI.

Given a real MSA of a protein family, MSAvolve generates one or more artificial MSAs that mimic the statistical properties of the real MSA (**Figure S9**), although each MSA is distinctly different from every other one (**Figure S10**).

Key features of MSAvolve are the capacity to simulate MSAs with uneven branch sizes (**Figure S9**), and to analyze mutation, coevolution, and recombination events in only some parts – a branch or a specific group of sequences – of the evolutionary tree. In particular, the automatic branch assignment of MSAvolve can satisfy a user's request to identify clusters in the experimental MSA using more than 2 principal components (PCs) of the covariance or other type of distance matrix (**Figure S9**).

MSAvolve generates MSAs that match very well the overall similarity score (OSS, see below) of the experimental MSA, regardless of whether the ancestor is created using the background frequencies of amino acids in the family or the HMM emission probabilities. In addition, MSAvolve builds coevolution matrices of each simulated MSA by counting the mutations that segregate at specific times during the evolution of a protein. This function can be equated to that of an external observer that witnesses the historical process of evolution. There are two main observers in MSAvolve: one is a 'low power' observer, who counts all the co-segregating mutations regardless of whether they originate from chance, recombination, or functional/structural demands (the latter are the mutations under constraint of coevolution). There is also a

‘high power’ observer, who counts selectively the true co-segregating mutations of functional/structural significance.

Although it is possible to build ancestor sequences of arbitrary length, in most cases, the MSA simulation starts with reading the experimental MSA of a real protein family and converting it to a numeric format (each aa is represented by a number between 1 and 20, gaps are represented as number 21). The total length (including gaps) of each aligned sequence in the real MSA defines the length of each sequence in the simulated MSA. First, a Hidden Markov Model (HMM) of the real MSA is built: the HMM provides the emission probabilities and background frequencies of the 20 amino acids at each position in the MSA. A non-parametric kernel smoothing distribution with a smoothing bandwidth of 0.01 is fitted to the vector of emission probabilities for each position of the MSA. An object representing the fitted distribution is stored for each MSA position, and an ancestor sequence is built by random drawing (rounded to the nearest integer) from the probability distribution of each position. Alternatively, MSAvolve can be prompted to produce an ancestor also from the equilibrium frequencies.

Next, a phylogenetic tree built from the matrix of pairwise distances is used to determine the number of main branches in the real MSA, which will be replicated in the simulated MSA (an arbitrary number of branches can however be set manually). Several options are available to calculate pairwise distances and trees, as offered in the Matlab Bioinformatics Toolbox (which is required for MSAvolve to function). Alternatively, branches can be determined from a spectral analysis of the covariance matrix of the sequences in the MSA, as originally described by Halabi *et al.* [1]. The covariance matrix provides also a convenient way to define an overall similarity score (OSS) between all the sequences in the MSA using the definition of multidimensional mutual information, *I*, developed by Wang and Shen [2] to express the similarity between the images in a set:

$$I\left(x_1,x_2..x_n\right)=1+\frac{\sum_{i=1}^{n}\frac{\lambda_i}{\sum_{i=1}^{n}\lambda_i}\log_2\left(\frac{\lambda_i}{\sum_{i=1}^{n}\lambda_i}\right)}{\log_2(n)} \qquad (i = 1,2,\ldots,n)$$

where λ_i(i = 1,2. . .n) are the eigenvalues of the MI matrix. This definition guarantees that n-dimensional MI is positive, and $0 \le I(x_1,x_2 \ldots x_n) \le 1$. When $\lambda_1 = \lambda_2 = \ldots = \lambda_n$, i.e. when $I_{ij} = 0$ ($i \neq j$) and $I_{11} = I_{22} = \ldots I_{nn}$, which means the images are not correlated to each other, the n-dimensional MI reaches its minimum. When the n images are identical, $I(x_1,x_2 \ldots x_n) = 1$. Since the MI matrix is self-adjoint positive definite, it is itself a covariance matrix and thus the definition of multidimensional MI can be extended also to the MSA covariance matrix to express the similarity between sequences.

Once the number and size of the main branches are decided, identical copies of the ancestral sequence become the starting point for populating the nodes of the evolutionary tree. Next a certain percentage of the position in the sequence is selected as covarying pairs. These pairs represent the positions that in a natural evolution would be under structural and/or functional constraints. Therefore, correlated evolution of these positions is the source of the most important signal that we seek to extract in coevolution analyses. Pairs can be selected in a strictly random fashion or based on the entropy (or relative entropy) of positions in the real MSA. For example, if we want 10% of all positions in a sequence of 300 aa's to be covarying with another 10%, we can select two vectors of 30 indices (the covarying matrix) corresponding to the columns in the MSA with the largest (or smallest or intermediate levels of) entropy. A non-parametric kernel smoothing distribution with a smoothing bandwidth of 0.01 is fitted to the vector of the joint probabilities of any two aa's at the covarying positions in the real MSA. An object representing the fitted distribution is stored for each covarying pair (a row in the covarying matrix). If the evolution tree is split into branches (reflecting the branches of

the experimental MSA), different objects representing the fitted distribution of the same selected covarying pairs are built for each branch.

MSAvolve adopts a fixed 3-LEVELS architecture with each level containing alternating steps (epochs or periods) of point mutations and steps of recombination to generate the final MSA. Each level represents an expansion of the evolutionary tree. While the program could have been designed with an arbitrary number of levels, in early testing we found that additional levels were only slowing down the execution time without changing the final outcome. Thus, while the general architecture is fixed (only 3 levels), inside each level all the steps can be repeated an arbitrary number of times with arbitrary parameters, giving rise to a very high degree of flexibility in balancing the amount of point mutations against the amount of recombination in and between branches. As an example we describe here the generation of a MSA corresponding to an evolutionary tree with 3 main branches and 45 sequences each of 300 residues.

LEVEL 1: the simulation starts with 3 identical copies of the ancestor (**Figure S11**; only the first 8 residues of the ancestor are shown). Each copy is subjected to *n* cycles of point mutations. Each cycle represents an arbitrary amount of time at the end of which a certain amount of changes in the sequence of the ancestor has become fixed. In each cycle and for each copy of the ancestor the mutational rate is chosen randomly from within a chosen range of values (e.g. 2-5%). The range of target rates used in the default setting of MSAvolve do not reflect known mutational rates of one or more particular organisms, but have been optimized by trial and errors in order to produce simulated MSAs that in most cases mimic well the experimental MSAs. However, the user can adjust the range of the target rates at will.

In practice, the protein is evolved inside a single matrix containing 3 identical rows corresponding to the ancestral protein. For example, if for a certain row we want to achieve a target mutational rate of 5% we will generate a random number from a

Poisson distribution assuming that 5% of all the sequence positions may have changed during the mutation cycle. Thus the target number of mutations (λ of the Poisson distribution) is 300 x 0.05 = 15. We draw a number from this distribution once for each sequence in the MSA. Then, for each sequence we draw the positions that must change from a uniform distribution of 300 positions: these positions are stored in a "random mutation vector". If any of these positions corresponds to a column in the MSA that is listed in the "global (or branch) covarying matrix", that position is pulled out of the random mutation vector and added to the first column of a "cycle covarying matrix". The second column of this matrix is completed retrieving the other element of each pair from the global covarying matrix.

At the beginning of the evolution process, the 3 sequences in the matrix are identical to the ancestral sequence (**Figure S11**), but they will start diverging as mutations accumulate. For each of the three sequences in the MSA, the positions defined by the random mutation vector are evolved first by random drawing from the aa probability distribution of each of these positions in the real MSA, as determined previously (see above). The covarying pairs are evolved next by random drawing from the joint aa probability distribution of these pairs in the real MSA, as determined previously (see above). It can be seen here how by always drawing each aa change from the observed probability distribution of aa's at each position or from the observed joint probability distribution at each pair of positions, the profile of the evolved MSA becomes progressively more similar to the profile of the real MSA, although individual sequences may be significantly different. Once the changes in all the sequences are completed (corresponding to the fixing of all mutations during the arbitrary amount of time represented by a mutation cycle) they are simply counted and added to a coevolution matrix; for example if there was a change at position 20 and a change at position 121, the value of the coevolution matrix at the position with indices [20,121] is

increased by 1. A separate coevolution matrix is maintained for the random mutations and for the mutations in coevolving pairs. With regard to the latter it should be mentioned that if in a mutation cycle more than one covarying pair becomes fixed, the coevolution matrix will reflect additional cross-changes between pairs: for example if pairs [10,15] and [34,70] both change, the coevolution matrix will record an increase by 1 at indices [10,15], [34,70], [10,34], [10,70], [15,34], [15,70]. We have decided to keep the coevolution matrices for the random mutations and for the true coevolving pairs separate, but one could argue that the correct count of the true correlation between positions at the end of each mutational cycles should include also the cross-correlation between random mutations and covarying mutations. Doing so dramatically increases $C_{stochastic}$ with respect to $C_{structure/function}$, and represents one of the possible user choices that affect the interpretation of the simulation. At any rate, MSAvolve also calculates a "global coevolution matrix" (this is the 'glob_COV' variable in the simulation output) that incorporates the cross-correlation between random mutations and co-varying positions.

An important parameter for the simulation is the ratio between mutational rate in each cycle and number of consecutive cycles. The same overall number of mutational changes can be obtained with different combinations of these numbers as long as their product remains approximately constant. However, since the counting of co-evolution takes place at the end of each mutational cycle (which we equate to the segregation of all mutations after a certain amount of time), having fewer cycles with more mutations increases $C_{stochastic}$ with respect to $C_{structure/function}$. On the other end, cycles cannot be made arbitrarily small, because it would imply that no mutations are ever deleterious (any mutation is fixed), and in the limit, from the practical standpoint of how the programs works, all mutations would appear to be the product of functional or structural coevolution. This effect can be exploited by the user to test the 'strength' of a coevolution detection method. Weaker methods are expected to detect progressively

fewer true covarions than stronger methods as the mutational rates increases, and the number of cycles decreases. Furthermore, since the mean entropy of the columns in the MSA grows monotonically with the overall mutation rate, to simulate data that are analogous to a given real MSA, it is useful to find a value of the product "mutation_rate x mutation_cycles" that gives an entropy similar to that of the real data. However, as previously discussed, while the final MSAs obtained maintaining this product constant will have the same column entropy, the corresponding coevolution matrices will have significantly different levels of $C_{stochastic}$.

MSAvolve alternates rounds of mutations (consisting of multiple mutation cycles) with rounds of recombination. In the beginning of our example recombination occurs between the three branches of the MSA (**Figure S11**; at this point each branch is represented by just one sequence). The recombination process implemented in MSAvolve is the unidirectional transfer of a contiguous block of aa's from one sequence to one or more other sequences. It is meant to simulate the spread of a successful block of residues among the members of the family. It is critical to define how the recombination blocks are chosen. We have explored two strategies: in one strategy the sequence is split in a finite number of blocks whose boundaries are selected by randomly drawing from a uniform distribution of the indices of all positions. This strategy is very convenient if a simulated MSA is generated multiple times (e.g. 100 times) for the purpose of collecting coevolution statistics, in which case having different recombination fragments in different simulations spreads the effects of recombination evenly throughout a sequence. A second strategy requires knowledge of the three-dimensional structure of at least one member of the real MSA. In this second case the SCHEMA algorithm [3-6] can be used to predict which fragments of homologous proteins can be recombined without disturbing the integrity of the structure [7].

Once the cross-over point are defined, MSAvolve carries out recombination in two steps:

1. It selects at random the number and identity of the sequences that will receive the donated fragment.

2. It selects at random which fragment will be donated and which sequence will donate the fragment to the other sequences.

Usually, within one round of recombination, multiple cycles of recombination are carried out, each with a different random selection of the donor, the recipients, and the donated fragment. Each recombination cycle represents an arbitrary amount of time after which the effects of the recombination process are fixed in the MSA. At the end of each recombination cycle the changes in all the sequences are simply counted and added to a recombination coevolution matrix; for example a fragment encompassing positions [5,6,7,8,9,10] may have spread from sequence 3 to sequence 1 and 2. If this spread produces a change at positions [5,6] in sequence 1 and positions [5,6,9] in sequence 2, the recombinant coevolution matrix will report an increase by 2 at indices [5,6] and an increase by 1 at indices [5,9] and [6,9].

The 1st level of the MSA simulation is completed by another round of point mutations such that altogether the flowchart of this level is: point mutations → recombination → point mutations (**Figure S11**, orange section).

LEVEL 2: this level starts expanding the tree to bring it to the final expected size of 45 sequences. For example, to achieve the desired number of sequences we can multiply the original 3 sequences by 3 at level 2 and by 5 at level 3. In practice, at level 2 two copies of each of the 3 sequences of level 1 are added to the MSA matrix, which now contains 9 rows and 3 different sequences (the original 3 branches of the tree)

derived from a single ancestral protein. The corresponding coevolution matrices are expanded accordingly by multiplication of the original counts.

The flowchart of level 2 is: mutations in each sequence → recombination between all the sequences regardless of the branch they belong to → mutations in each sequence → recombination between sequences of each branch → mutations in each sequence (**Figure S11**, pink section).

LEVEL 3: 4 copies of each of the 9 sequences of level 2 are added to the MSA matrix, which now contains 45 rows and 9 different sequences (3 for each of the original 3 branches of the tree) derived from a single ancestral protein. The corresponding coevolution matrices are expanded accordingly by multiplication of the original counts.

The flowchart of level 3 is: mutations in each sequence → recombination between all the sequences inside each of the 3 main branches → mutations in each sequence → recombination between sequences inside each of the 9 sub-branches → mutations in each sequence (**Figure S11**, green section).

The 3-level architecture of the program reflects the hypothesis that as the protein family expands and the members diverge recombination between members of distant branches of the tree becomes more difficult. Once all levels of MSAvolve are completed, the global and/or individual coevolution matrices can be analyzed and compared to those derived from the final fully evolved MSA by an external program. It is also possible to select randomly or (not randomly) only certain sequences of the evolved MSA, in which case the corresponding coevolution matrices are also made available. This feature allows the effect of $C_{phylogenetic}$ to be readily evaluated.

REFERENCES

1. Halabi N, Rivoire O, Leibler S, Ranganathan R (2009) Protein sectors: evolutionary units of three-dimensional structure. Cell 138: 774-786.

2. Wang B, Shen Y (2006) A method on calculating high-dimensional mutual information and its application to registration of multiple ultrasound images. Ultrasonics 44: e79-e83.

3. Meyer MM, Hiraga K, Arnold FH (2006) Combinatorial recombination of gene fragments to construct a library of chimeras. Curr Protoc Protein Sci Chapter 26: Unit 26 22.

4. Meyer MM, Hochrein L, Arnold FH (2006) Structure-guided SCHEMA recombination of distantly related beta-lactamases. Protein Eng Des Sel 19: 563-570.

5. Li Y, Drummond DA, Sawayama AM, Snow CD, Bloom JD, et al. (2007) A diverse family of thermostable cytochrome P450s created by recombination of stabilizing fragments. Nat Biotechnol 25: 1051-1056.

6. Heinzelman P, Snow CD, Wu I, Nguyen C, Villalobos A, et al. (2009) A family of thermostable fungal cellulases created by structure-guided recombination. Proc Natl Acad Sci U S A 106: 5610-5615.

7. Voigt CA, Martinez C, Wang ZG, Mayo SL, Arnold FH (2002) Protein building blocks preserved by recombination. Nat Struct Biol 9: 553-558.
