# Supplementary material for: Accurate Simulation and Detection of Coevolution Signals in Multiple Sequence Alignments
Source: PLoS One. 2012 Oct 16;7(10):e47108. doi: 10.1371/journal.pone.0047108 (PMC3473043; doi:10.1371/journal.pone.0047108)
Supplement: Text S4 — List of MSAs and corresponding PDB files for the set of 150 protein families analyzed in this study. (DOCX) [file pone.0047108.s023.docx]

**List of MSAs and corresponding PDB files for the set of 150 protein families analyzed in this study.**

The set was originally described in [[1](#_ENREF_1)] and can be downloaded from http://bioinf.cs.ucl.ac.uk/downloads/PSICOV/suppdata.

MSA:

1a3aA.aln, 1dixA.aln, 1hdoA.aln, 1k7jA.aln, 1rybA.aln, 1a6mA.aln, 1dlwA.aln, 1hfcA.aln, 1kidA.aln, 1smxA.aln, 1a70A.aln, 1dmgA.aln, 1hh8A.aln, 1kq6A.aln, 1svyA.aln, 1aapA.aln, 1dqgA.aln, 1htwA.aln, 1kqrA.aln, 1t8kA.aln, 1abaA.aln, 1dsxA.aln, 1hxnA.aln, 1ktgA.aln, 1tifA.aln, 1ag6A.aln, 1eazA.aln, 1i1jA.aln, 1ku3A.aln, 1tqgA.aln, 1aoeA.aln, 1ej0A.aln, 1i1nA.aln, 1kw4A.aln, 1tqhA.aln, 1atlA.aln, 1ej8A.aln, 1i4jA.aln, 1lm4A.aln, 1tzvA.aln, 1atzA.aln, 1ek0A.aln, 1i58A.aln, 1lo7A.aln, 1vfyA.aln, 1avsA.aln, 1f6bA.aln, 1i5gA.aln, 1lpyA.aln, 1vhuA.aln, 1bdoA.aln, 1fcyA.aln, 1i71A.aln, 1m4jA.aln, 1vjkA.aln, 1bebA.aln, 1fk5A.aln, 1ihzA.aln, 1m8aA.aln, 1vmbA.aln, 1behA.aln, 1fl0A.aln, 1iibA.aln, 1mk0A.aln, 1vp6A.aln, 1bkrA.aln, 1fnaA.aln, 1im5A.aln, 1mugA.aln, 1w0hA.aln, 1brfA.aln, 1fqtA.aln, 1iwdA.aln, 1nb9A.aln, 1whiA.aln, 1bsgA.aln, 1fvgA.aln, 1j3aA.aln, 1ne2A.aln, 1wjxA.aln, 1c44A.aln, 1fvkA.aln, 1jbeA.aln, 1npsA.aln, 1wkcA.aln, 1c52A.aln, 1fx2A.aln, 1jbkA.aln, 1nrvA.aln, 1xdzA.aln, 1c9oA.aln, 1g2rA.aln, 1jfuA.aln, 1ny1A.aln, 1xffA.aln, 1cc8A.aln, 1g9oA.aln, 1jfxA.aln, 1o1zA.aln, 1xkrA.aln, 1chdA.aln, 1gbsA.aln, 1jkxA.aln, 1p90A.aln, 2arcA.aln, 1cjwA.aln, 1gmiA.aln, 1jl1A.aln, 1pchA.aln, 2cuaA.aln, 1ckeA.aln, 1gmxA.aln, 1jo0A.aln, 1pkoA.aln, 2hs1A.aln, 1ctfA.aln, 1guuA.aln, 1jo8A.aln, 1qf9A.aln, 2mhrA.aln, 1cxyA.aln, 1gz2A.aln, 1josA.aln, 1qjpA.aln, 2phyA.aln, 1cznA.aln, 1gzcA.aln, 1jvwA.aln, 1ql0A.aln, 2tpsA.aln, 1d0qA.aln, 1h0pA.aln, 1jwqA.aln, 1r26A.aln, 2vxnA.aln, 1d1qA.aln, 1h2eA.aln, 1jyhA.aln, 1roaA.aln, 3borA.aln, 1d4oA.aln, 1h4xA.aln, 1k6kA.aln, 1rw1A.aln, 3dqgA.aln, 1dbxA.aln, 1h98A.aln, 1k7cA.aln, 1rw7A.aln, 5ptpA.aln.

PDB:

1BRF, 1M8A, 1MK0, 2ARC, 1ROA, 1FK5, 1G2R, 1DQG, 2MHR, 1I1J, 1JO0, 1IIB, 1TIF, 1XKR, 1SMX, 1CTF, 1AG6, 1I71, 1AAP, 1VFY, 1DLW, 1NPS, 1BKR, 1P90, 1KW4, 1M4J, 1VJK,,1D4O, 1JOS, 1SVY, 1LPY, 1VMB, 1WJX, 1CXY, 1PCH, 1BEH, 1HXN, 1TQG, 1ATZ, 1BEB, 1HFC, 1MUG, 1D0Q, 1H4X, 1EJ8, 1WHI, 1I4J, 1C9O, 1JYH, 1J3A, 1GZC, 1NB9, 1HTW, 1ABA, 1QL0, 1RW1, 1T8K, 1KU3, 1DMG, 1NRV, 1IHZ, 1EJ0, 1DIX, 1ATL, 1CHD, 1JFX, 1WKC, 1RYB, 1VHU, 1CKE, 1GUU, 1KQ6, 1TZV, 1A70, 1AOE, 1A6M, 1FX2, 1GZ2, 1JO8, 1D1Q, 1XDZ, 1JWQ, 1FVG, 1LM4, 1CC8, 1FVK, 1DBX, 1KID, 1BDO, 1QJP, 1FQT, 1FL0, 1K6K, 1EAZ, 1JVW, 1IWD, 3DQG, 1K7C, 1CZN, 1LO7, 1A3A, 2TPS, 1K7J, 1GBS, 1O1Z, 2VXN, 1NY1, 1GMI, 1FCY, 1RW7, 1QF9, 1I1N, 1H98, 1AVS, 1G9O, 1IM5, 1H0P, 1GMX, 1BSG, 1W0H, 1R26, 1C52, 1JKX, 1VP6, 1F6B, 1NE2, 1JL1, 1H2E, 1EK0, 1JBK, 2PHY, 1I5G, 1FNA, 3BOR, 2CUA, 1KTG, 1XFF, 5PTP, 1PKO, 1JFU, 1CJW, 1TQH, 1HDO, 2HS1, 1HH8, 1I58, 1JBE, 1KQR, 1C44, 1DSX.

REFERENCES

1. Jones DT, Buchan DW, Cozzetto D, Pontil M (2012) PSICOV: precise structural contact prediction using sparse inverse covariance estimation on large multiple sequence alignments. Bioinformatics 28: 184-190.
