# Supplementary material for: Accurate Simulation and Detection of Coevolution Signals in Multiple Sequence Alignments
Source: PLoS One. 2012 Oct 16;7(10):e47108. doi: 10.1371/journal.pone.0047108 (PMC3473043; doi:10.1371/journal.pone.0047108)
Supplement: Toolbox S1 — MSAVOLVE_v1.0a Toolbox for Matlab. (ZIP) [file pone.0047108.s024.zip › MSAVOLVE_v1.0a/COEVOLUTION_METHODS/FUNCTIONS/OMES/Bioinformatics-2004-Dekker-1565-72.pdf]

BIOINFORMATICS
Vol. 20 no. 10 2004, pages 1565–1572
doi:10.1093/bioinformatics/bth128

# A perturbation-based method for calculating explicit likelihood of evolutionary co-variance in multiple sequence alignments

John P. Dekker[1,†], Anthony Fodor[2,†], Richard W. Aldrich[2] and Gary Yellen[1,*]

[1]Department of Neurobiology, Harvard Medical School, 220 Longwood Avenue, Boston, MA 02115, USA and [2]Department of Molecular and Cellular Physiology, Howard Hughes Medical Institute, Stanford University School of Medicine, Stanford, CA 94305-5345, USA

Received on September 22, 2003; accepted and revised on December 19, 2003
Advance Access publication February 12, 2004

## ABSTRACT

**Motivation:** The constituent amino acids of a protein work together to define its structure and to facilitate its function. Their interdependence should be apparent in the evolutionary record of each protein family: positions in the sequence of a protein family that are intimately associated in space or in function should co-vary in evolution. A recent approach by Ranganathan and colleagues proposes to look at subsets of a protein family, selected for their sequence at one position, to see how this affects variation at other positions.

**Results:** We present a quantitative algorithm for assessing covariation with this approach, based on explicit likelihood calculations. By applying our algorithm to 138 Pfam families with at least one member of known structure, we demonstrate that our method has improved power in finding physically close residues in crystal structures, compared to that of Ranganathan and colleagues.

**Contact:** gary_yellen@hms.harvard.edu

**Supplementary information:** www.afodor.net/bioinfosup.html

## INTRODUCTION

It has long been appreciated that evolutionary correlation could provide information about protein structure (Neher, 1994; Atchley *et al.*, 1999; Larson *et al.*, 2000; Kass and Horovitz, 2002). Most previous approaches have used correlation functions to assess co-variation between positions in a protein, with the main goal of predicting contacts between amino acids that are not adjacent in the linear sequence (Gobel *et al.*, 1994; Thomas *et al.*, 1996; Olmea *et al.*, 1999). The basic assumption underlying these studies is that such contact residues in a protein should demonstrate mutually constrained patterns of amino acid substitution. When these co-variation methods predict a contact that is not borne out by structural analysis, the ‘spurious’ predictions may be evolutionary noise, or they may correspond to bona fide co-variation at a distance. For instance, many types of allosteric conformational changes involve a physical perturbation that must communicate energetically through the structure of the protein with the active site conformation. Lockless and Ranganathan (1999) and Suel *et al.* (2003) have proposed to search for networks of co-varying residues that might reveal such conformational fault lines.

Their analytic approach begins with the application of some constraint on the distribution of residues at a chosen position in a multiple sequence alignment (MSA). The subset of sequences conforming to this constraint is selected, and the degree of bias present in the distribution of residues at each other position in this subset is assessed. The overall strategy of this approach is novel and potentially powerful because its central subsetting operation allows the hypothesis-driven exploration of co-variation occurring in response to freely chosen *in silico* evolutionary ‘perturbations’. For instance, one may choose to subset the MSA according to physicochemical classes of perturbations that are found experimentally to alter ligand binding, allosteric coupling or conformational equilibria. This approach therefore differs fundamentally from previous methods developed to calculate global pairwise co-variation statistics (Gobel *et al.*, 1994; Neher, 1994; Thomas *et al.*, 1996; Olmea *et al.*, 1999; Atchley *et al.*, 2000; Larson *et al.*, 2000; Kass and Horovitz, 2002).

However, the co-variation algorithm proposed by Lockless and Ranganathan (1999) for use in this method, which is based loosely on an analogy to Boltzmann’s statistical mechanics, lacks a rigorous connection to the actual statistics of co-variation. We present here an alternative algorithm for quantifying evolutionary co-variation based on explicit

*To whom correspondence should be addressed.

†The authors wish it to be known that, in their opinion, these two authors should be regarded as joint First Authors.

Downloaded from bioinformatics.oxfordjournals.org by guest on April 9, 2011

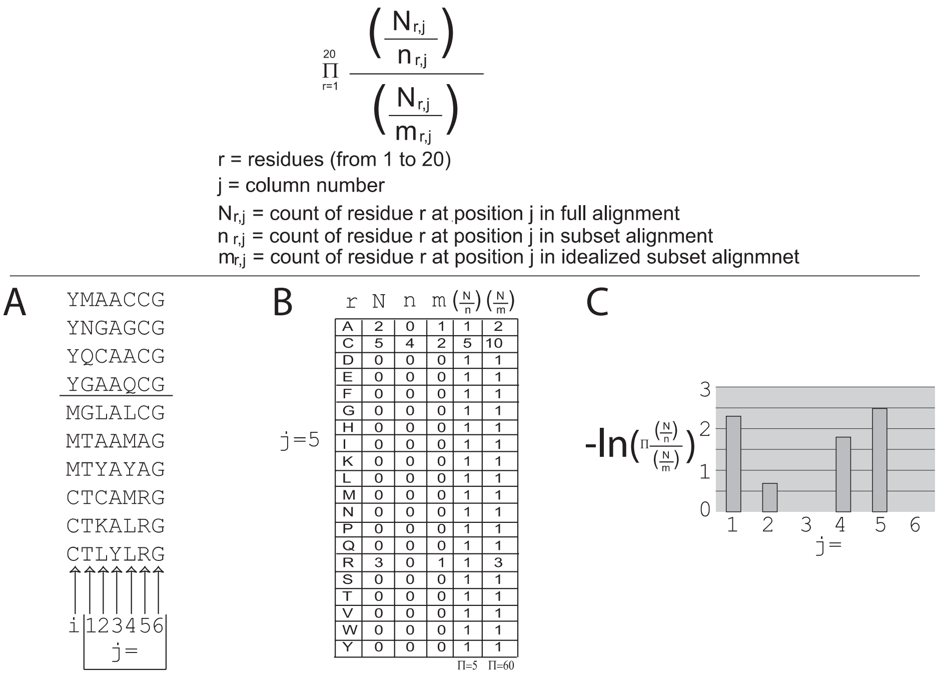


**Fig. 1.** Application of the ELSC algorithm to a simple, hypothetical alignment. (**A**) A hypothetical alignment for which the perturbation is taken to be the presence of tyrosine in column $i$, a constraint that yields a sub-alignment of the four sequences above the horizontal line. (**B**) The details of the ELSC calculation for the given sub-alignment for column $j = 5$. The count of residues in the idealized subalignment ($m$) represents the number of each kind of residue that would be in column $j$ in the sub-alignment if the residue composition in the sub-alignment were identical to the residue composition in column $j$ of the full alignment. (**C**) The ELSC calculation for the given sub-alignment for columns $j = 1$–6.

likelihood calculations that, when applied to a set of 138 Pfam families, appears to have greater power than the Lockless and Ranganathan method in predicting residue proximity in crystal structures.

## SYSTEM, ALGORITHMS AND METHODS

### Perturbation-based co-variance

Perturbation-based co-variance algorithms (Lockless and Ranganathan, 1999; Suel *et al*., 2003) work by choosing a subset of sequences in an MSA and comparing the characteristics of the subset with the characteristics of the full alignment. Consider two columns $i$ and $j$ in an MSA. We begin by choosing a subset of sequences from the full set by placing a constraint on the identity of the residue occupying position $i$ in the alignment. For instance (Fig. 1A), one may choose the subset of sequences containing tyrosine at a particular position, $i$. Next, we quantify the degree of bias present in the distribution of residues at a second position, $j$, in this subset. In the case that substitutions at positions $i$ and $j$ occur independently throughout the sequences sampled by the MSA, the distribution of AAs at position $j$ in the subset should be similar to the distribution at that position in the full MSA. However, if the two positions co-vary, the composition at position $j$ in the subset may be biased by the constraint placed on position $i$.

### Statistical coupling analysis

In this paper, we compare the performance of two perturbation-based co-variance algorithms. The first of these, statistical coupling analysis (SCA), has been described previously (Lockless and Ranganathan, 1999; Suel *et al.*, 2003), although the description of the algorithm that follows has significant differences from the published description (see Detailed methods section) . The SCA method is described in terms of metaphorical 'energies' ($\Delta G$'s), which correspond roughly to log (probabilities). The description of the SCA algorithm (Lockless and Ranganathan, 1999) begins with an 'overall empirical evolutionary conservation parameter' for each column $i$ in the alignment:

$$\Delta G_i = \sqrt{\sum_x \left(\ln P_i^x\right)^2},$$

where $x$ spans from 1 to 20 for all amino acid residues and is related to the probability of finding the observed number of $x$ residues in column $i$. (Here, we ignore the authors' metaphorical prefactor, $kT^*$, as it represents an undefined scalar that does not impact the present analysis.) The parameter $P_i^x$ is calculated using a function in binomial form that compares



Downloaded from bioinformatics.oxfordjournals.org by guest on April 9, 2011

the frequency in the given column with the 'mean frequency in all proteins':

$$P^x = \frac{N!}{n_x!(N-n_x)!} p_x^{n_x}(1-p_x)^{N-n_x},$$

where $N$ is set arbitrarily to equal 100, $n_x$ is the numerical percentage of sequences with residue $x$ at the given position and $p_x$ is the 'mean frequency of amino acid $x$ in all positions' as determined from all Swiss-Prot entries. We note that as a consequence of the authors' chosen definitions of $N$ and $n_x$, which seem difficult to rationalize, the calculated parameter, $P^x$, is related to, but is not equivalent to, the 'probability of any amino acid $x$ at site $i$', as the authors claim.

We have found that the $\Delta G_i$ measurement is well correlated to a common measure of conservation known as sequence entropy (Shenkin *et al.*, 1991), $H_i$:

$$H_i = -\sum_x [p_x(i) \ln p_x(i)],$$

where $i$ is the column of interest, $x$ spans the 20 possible amino acid residues and $p_x(i)$ is the frequency of residue $x$ at position $i$. The median Spearman rank-order correlation coefficient (Press *et al.*, 1995) between the sequence entropy, $H_i$, and $\Delta G_i$ is $\sim$−0.92 for 138 alignments where columns with > 50% gaps have been removed (see Supplementary Figure 1 online). This high degree of correlation between the two measures suggests that despite the comparison of amino acid frequencies with background frequencies from all Swiss-Prot entries and the modified binomial calculations, the SCA $\Delta G_i$ parameter is essentially a measure of column conservation.

The SCA algorithm uses as its co-variance measure for a pair of columns $(i, j)$ the parameter $\Delta\Delta G_{i,j}$, which the authors refer to as the 'statistical coupling energy'. $\Delta\Delta G_{i,j}$ is related to the difference between the $\Delta G_i$ values for the full alignment and the sub-alignment summed over all 20 amino acids as follows:

$$\Delta\Delta G_{i,j} = \sqrt{\sum_x \left(\ln P^x_{i|\delta j} - \ln P^x_i\right)^2}.$$

Here, the $P^x_{i|\delta j}$ conditional term represents the modified binomial probability, $P^x_i$, as defined for column $j$ for the sequences in the sub-alignment created by the perturbation at column $i$. That is, $P^x_i$ in the equation is calculated for the full alignment, while $P^x_{i|\delta j}$ is calculated for the sub-alignment. The SCA algorithm works, therefore, by looking for differences in the degree of conservation between the sub-alignment and the full alignment in column $j$.

## Explicit likelihood of subset co-variation

The formulation of the SCA algorithm does not lend itself to a straightforward statistical interpretation of the resulting correlation values. We reasoned that an approach with a more direct connection to the statistics of co-variation would yield a different form of perturbation-based co-variance algorithm, which might have significantly different properties. As above, we seek a co-variance score for a pair of columns ($i$ and $j$), where we select a subset of the MSA by constraining the identity of the AA in column $i$ and then examine the effect on each other position $j$. (In our notation, we use capital $N$s to describe properties of the full MSA and small $n$s to describe properties of the subset; for instance, $N_{\text{total}}$ is the number of sequences in the full MSA and $n_{\text{total}}$ the number in the subset.) After selecting a subset, we calculate the observed AA composition of the subset at position $j$. We then ask, given the AA composition at position $j$ in the full MSA, how many possible subsets of size $n_{\text{total}}$ would have at position $j$ exactly the observed composition of $n_{\text{ala},j}$ alanines, $n_{\text{asn},j}$ asparagines, $n_{\text{asp},j}$ aspartic acids, etc. The number of such combinations is given exactly by $\Omega_j^{<i>}$:

$$\Omega_j^{<i>} = \binom{N_{\text{ala},j}}{n_{\text{ala},j}} \cdot \binom{N_{\text{asn},j}}{n_{\text{asn},j}} \cdot \binom{N_{\text{asp},j}}{n_{\text{asp},j}} \cdots = \prod_r \binom{N_{r,j}}{n_{r,j}}.$$

Here, $N_{r,j}$ denotes number of residues of type $r$ at position $j$ in the full MSA, and $n_{r,j}$ denotes number of residue type $r$ at position $j$ in the constrained subset. The combinatorial factor

$$\binom{N_{\text{ala},j}}{n_{\text{ala},j}} = \frac{N_{\text{ala},j}!}{n_{\text{ala},j}!\left(N_{\text{ala},j} - n_{\text{ala},j}\right)!}$$

gives the number of different ways of choosing the exact number of alanine-containing sequences actually found in the subset $(n_{\text{ala},j})$ from the total number of alanine-containing sequences in the full MSA $(N_{\text{ala},j})$. Because this choice for each amino acid is independent of the others, the total number of possible subsets with the given composition is given simply by the product of these combinations, $\Omega_j^{<i>}$.

If we then divide $\Omega_j^{<i>}$ by the total number of possible subsets of size $n_{\text{total}}$, we arrive at the exact probability that a random selection of a subset of size $n_{\text{total}}$ from the MSA will give the observed amino-acid composition at position $j$ in the constrained subset. This probability is given by $L_j^{<i>}$:

$$L_j^{<i>} = \frac{\prod_r \binom{N_{r,j}}{n_{r,j}}}{\binom{N_{\text{total}}}{n_{\text{total}}}}.$$

Given that different MSAs and subsets will differ in size and combinatorial complexity, we also calculate a normalized statistic that gives the probability of drawing the observed composition at random relative to the probability of drawing the most likely composition. For this normalization, we first construct an ideally representative subset, whose values we denote $m_{r,j}$. To do this, we compute the set of integral values where $m_{r,j} \approx \left(N_{r,j}/N_{\text{total}}\right) \cdot n_{\text{total}}$. This is implemented



Downloaded from bioinformatics.oxfordjournals.org by guest on April 9, 2011

by calculating the decimal values of $m_{r,j}$ and then rounding each of these decimals to integer values with the constraint that $\sum_r m_{r,j} = \sum_r n_{r,j}$. Once this ideal subset of $m_{r,j}$ has been constructed, the probability of drawing this subset from MSA at random is given by $L_{j,\max}^{<i>}$:

$$L_{j,\max}^{<i>} = \frac{\prod_r \binom{N_{r,j}}{m_{r,j}}}{\binom{N_{\text{total}}}{n_{\text{total}}}}.$$

We then compute the normalized ratio, $L_j^{<i>}/L_{j,\max}^{<i>}$, which we denote $\Lambda_j^{<i>}$ :

$$\Lambda_j^{<i>} \equiv L_j^{<i>}/L_{j,\max}^{<i>} = \prod_r \frac{\binom{N_{r,j}}{n_{r,j}}}{\binom{N_{r,j}}{m_{r,j}}}.$$

In order to compare the results of this algorithm with the results of SCA, the scores of which are related to the logarithms of probabilities, we take $-\ln \Lambda_j^{<i>}$ as our score for a pair of columns $(i, j)$. Figure 1 shows this algorithm, which we call Explicit Likelihood of Subset Co-variation (ELSC), applied to a simple, hypothetical alignment.

## DETAILED METHODS

The Pfam 7.7 (October 2002) text archive was downloaded. All alignments that did not have a >95% match to a crystal structure in the Protein Data Bank (PDB) database were removed from the analysis set. In an attempt to correct for apparent co-variation due to a common phylogenetic origin of closely related sequences, duplicate sequences with greater than 90% identity were removed from the alignments. All alignments that did not have at least 50 of these non-duplicate sequences were removed from our analysis. All columns that did not have at least 50% non-gapped residues were removed from the alignments. For the purposes of this paper, we constructed our 'perturbation' subsets by constraining each position $i$ to contain only the most conserved residue. For example, if at position $i$ in an alignment, glycine is the most conserved residue, accounting for 24% of the residues at this position, then our sub-alignment would consist of the 24% of the sequences that have a glycine at position $i$. It should be noted that while we and the original SCA papers (Lockless and Ranganathan, 1999; Suel *et al*., 2003) use conserved residues as the perturbation constraint, this choice is arbitrary and any constraint yielding appropriately large sub-alignments could in principle be used. The effect of perturbation constraint choice on the calculated co-variation scores was not addressed in this study. CLUSTALW was used to map the sequences in the alignment to residues in PDB files for which C$\beta$–C$\beta$ (C$\alpha$ for glycine) distances were measured. In order to avoid trivial residue contacts, all residue pairs within eight residues in the primary sequence were removed from the data set. The methods used for calculation of pair distance were identical to those described previously (Fodor and Aldrich, 2004). All Java code used to make the figures is available on request.

For all SCA calculations in our paper, we used Windows binaries, which were generously distributed by the Ranganathan laboratory. Our definitions of $\Delta\Delta G$, $\Delta G$, and $P^x$ in the results section are based on our reading of the C source code that we received as part of this distribution. These definitions differ from those described by the authors in their original paper (Lockless and Ranganathan, 1999), which for $P^x$ state that '$N$ is the total number of sequences', and '$n_x$ is the number of sequences with amino acid $x$'. One can tell by inspection that these original descriptions of $N$ and $n_x$ must be incorrect as they would lead to any conserved column $j$ generating a high $\Delta\Delta G_{i,j}$ score since binomial probabilities $P_i^x$ scale exponentially with the number of identical residues and in a highly conserved column $j$ there have to be fewer identical residues in the sub-alignment than the full alignment. Indeed, we have found that with the published definition of SCA the average $\Delta G_i$ score for a pair of columns $(i, j)$ was highly correlated with the $\Delta\Delta G_{i,j}$ score (data not shown). That is, as originally described, SCA conservation was essentially the same thing as SCA co-variance. In addition, the original paper stated that each $P_i^x$ term for each residue type should be normalized by a value related to 'a hypothetical site where all amino acids are found in their mean frequencies in the MSA'. In the distributed binaries, no such normalization takes place. We checked the output of the Windows SCA binaries by creating a Java implementation of the SCA algorithm as defined in our paper and found a perfect correlation (data not shown, Java code available upon request). We are therefore reasonably confident that our description of the SCA algorithm is correct, despite significant differences with the published description.

The SCA algorithm treat gaps in alignments in a somewhat different way from the ELSC algorithm. Consider the following column in an alignment:

```
A
A
–
C
C
```

When counting the number of sequences in the alignment, the ELSC algorithm discards gaps. The ELSC algorithm, therefore, would consider the frequency of A and C to be 2/4 and the total number of residues in the column to be four. The SCA algorithm as implemented in the Windows binaries, however, counts the gaps and would therefore view the frequencies of A and C to be 2/5 and the total number of residues in the column to be five. We implemented a version of the SCA algorithm that does not count gaps and found that this version was generally well correlated with the version of the SCA algorithm that



Downloaded from bioinformatics.oxfordjournals.org by guest on April 9, 2011

does count gaps. For the 138 alignments used in this study, the median Pearson correlation coefficient between the two SCA implementations was $\sim 0.9$, although the version of the SCA algorithm that did not count gaps performed slightly worse in finding pairs of physically close residues (data not shown). We are therefore confident that the differences between the ELSC and SCA algorithms described in our paper are not the result of the two algorithms treating gaps differently.

The SCA algorithm requires columns to meet a certain threshold of 'statistical equilibrium' (Suel *et al.*, 2003). In order to meet this requirement, we ran each of the Pfam alignments through the 'RandomElim' program in the SCA package with the default parameters. Following the guidelines posted on the author's Web site (http://www.hhmi.swmed.edu/Labs/rr/world/sca/sup_figure2.pdf), we chose as our cutoff value the smallest number of sequences for which the 'RandomElim' program in the SCA package gave a value of over 0.07. For example, for the Pfam alignment 2-Hacid_DH_C, we got a cutoff value of 31%. This is interpreted by the authors of the SCA package to mean that any sub-alignment with fewer than 31% of the sequences is not in 'statistical equilibrium' and will not generate meaningful results when analyzed by the SCA package. Since the 'perturbation' that created the sub-alignment is based on conserved residues, this requires that each column $i$ that does not have a single residue in at least 31% of the sequences should be excluded from the analysis. So, for example, if a three column alignment was highly conserved in column 1 but poorly conserved in columns 2 and 3, we would generate scores under SCA only for columns $(1, 2)$ and $(1, 3)$. Removing poorly conserved columns in this way does lead to marginal improvements in the SCA algorithm when compared with a data set in which poorly conserved columns were included (Fodor and Aldrich, 2004). We removed from our data set any alignment that did not have at least 100 $i$ columns that met the criteria for inclusion by the SCA package. Applying these criteria to our original set of alignments yielded a set of 138 alignments (listed in the supplementary materials), which were used in this paper. In order to perform a fair comparison, we only generated $(i, j)$ scores for the ELSC algorithm that were also generated for the SCA algorithm.

One final consideration concerns the assignment of columns $i$ and $j$ for the co-variation calculation. Since it need not be the case that $\text{SCA}(i, j) = \text{SCA}(j, i)$ or that $\text{ELSC}(i, j) = \text{ELSC}(j, i)$, we constrained the relationship between $i$ and $j$ such that it was always true that $j > i$ and then performed the co-variation calculation for this pair only. Thus, for columns 1 and 20 in an alignment, we used the most conserved residue in column 1 to form the sub-alignment and reported the SCA and ELSC scores for the pair $(1, 20)$ but not for the pair $(20, 1)$. Although one might imagine a number of strategies for combining the $(i, j)$ and $(j, i)$ scores, these were not explored in the original SCA papers or in this study.

## EXPERIMENTAL RESULTS

### Algorithm performance in predicting physically close residues

The underlying hypothesis guiding the design of algorithms that detect correlated mutations is that if two columns in an alignment show a high degree of correlation, the corresponding residue positions in a protein should be linked either functionally or energetically or by virtue of being physically close in some important conformation of the protein. Algorithms that measure correlated mutations in multiple sequence alignments have long been used to predict inter-residue contacts in proteins (Altschuh *et al.*, 1987), and it is reasonable to expect that even if a correlated mutation algorithm finds networks of energetically coupled residues (Lockless and Ranganathan, 1999; Suel *et al.*, 2003), the residues of the network should, on average, be closer to each other than residues drawn at random from the protein. In order to compare the performance of the SCA and ELSC algorithms, therefore, we took alignments from the Pfam database that met certain criteria for sequence diversity (see Detailed methods section) and asked how well the algorithms were able to use the information in these alignments to make predictions about physically close residues in crystal structures that correspond to one of the sequences in the alignment. Figure 2A shows the results of this analysis for 38 858 $(i, j)$ ELSC and SCA comparisons for the Pfam family Cys_Met_Meta. The $y$-axis shows $C\beta$–$C\beta$ distances for each calculated $(i, j)$ pair of columns for the 1qgn crystal structure that corresponds to the Q9ZPL5 sequence in the Cys_Met_Meta alignment. Clearly, both algorithms are able to successfully generate some information about the structure as represented by the fact that the highest scoring pairs of co-varying residues (to the right on the $x$-axis) tended to be physically close to each other (to the bottom of the $y$-axis).

We can begin to quantify the power of the two algorithms by forming a null hypothesis that an algorithm that simply chooses pairs of residues at random could match the performance of the ELSC or SCA algorithm. To assess the probability that this null hypothesis is true, we make an arbitrary choice to examine only the top 75 pairs of residues for each algorithm. This division is indicated in Figure 2 by the vertical line. We then arbitrarily choose the 50th percentile of pair distance (represented by the dark gray horizontal line) and ask what the probability is that a random pairing algorithm could find as many residues within the bottom 50th percentile of pair distance as the ELSC and SCA algorithms. We would expect a random pairing algorithm to choose 37.5 pairs of residues below the 50th percentile of pair distance. In fact, the ELSC algorithm chooses 62 out of 75 below the 50th percentile, while the SCA algorithm chooses 56 out of 75. One can easily show (Fodor and Aldrich, 2004) that the probability that a random pairing algorithm could match this performance is $p < 10^{-8}$ for ELSC and $p < 10^{-4}$ for SCA. By this measure, while both algorithms are statistically significant, the ELSC



Downloaded from bioinformatics.oxfordjournals.org by guest on April 9, 2011

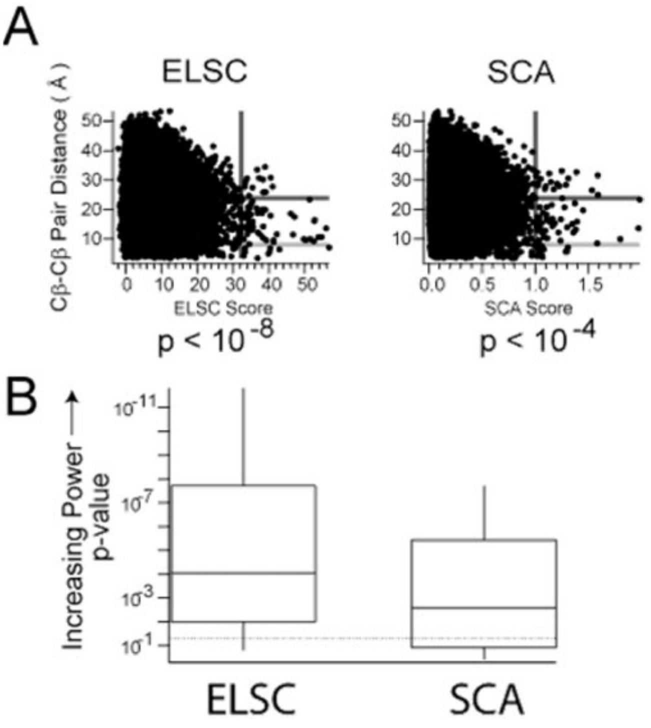


**Fig. 2.** Comparison of the ELSC and SCA algorithms for a single protein family. (**A**) C$\beta$–C$\beta$ pair distance from the 1qgn crystal structure is shown as a function of ELSC and SCA scores for the Cys_Met_Meta family. The vertical lines mark the top 75 pairs of predictions, and the horizontal dark gray lines mark the 50th percentile of all pair distances. The light gray horizontal lines mark the 8 Å C$\beta$–C$\beta$ pair distance cutoff for the CASP definition of an inter-residue contact. The probabilities below each graph are the probability that a random pairing algorithm could do as well in finding 75 pairs of residues below the 50th percentile of all pair distances (see text). (**B**) The probability of the null hypothesis that a random pairing algorithm could match the performance of ELSC and SCA for all 138 Pfam families in our study for the top scoring 75 pairs of residues. The dashed line shows the $p = 0.05$ level. The line in the middle of the box is the median value. The edges of the box are the 25th and 75th percentiles. The whiskers are the 10th and 90th percentiles.

algorithm has greater power. Of course, our choice of the top 75 pairs of residues and the 50th percentile is arbitrary. We have found, however, that the relative power of the ELSC and SCA algorithms is maintained even while choosing different parameters for our test (e.g. the first 50 residues below the 25th percentile). We argue, therefore, that our test is a reasonable metric of the power of the algorithms, despite the presence of these two free parameters.

We see in Figure 2A the results for a single protein family. Supplementary Figures 2 and 3 online show C$\beta$–C$\beta$ distance plotted against ELCS and SCA score for all 138 protein families in our study. As in Figure 2A, the vertical lines mark the top scoring 75 pairs of residues for each protein family

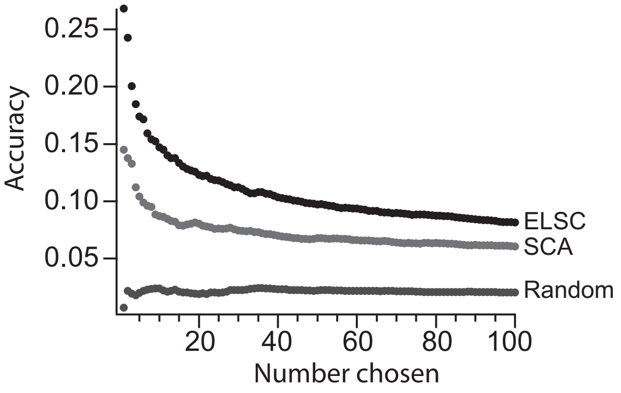


**Fig. 3.** Accuracy as a function of the number of predictions made by each algorithm. Shown are the average values for all 138 Pfam families in our study. Accuracy is defined as the number of correctly chosen residue contacts with C$\beta$–C$\beta$ distances of $\leq$8 Å divided by the number of predictions made. Random indicates an algorithm that simply chooses residue pairs at random.

and the horizontal dark gray lines mark the 50th percentile of residue pair distance. Figure 2B shows the range for the 138 families of the probability of the null hypothesis that a random pairing algorithm could match the performance of each algorithm in finding residues below the 50th percentile of pair distance for the highest scoring 75 pairs of residues. By this metric, although there is a good deal of variability in the power of both algorithms, ELSC on average outperforms SCA.

The most common use of correlated mutation algorithms in the literature has been as predictors of inter-residue contacts (Gobel *et al.*, 1994; Olmea and Valencia, 1997; Larson *et al.*, 2000). The CASP contest (http://predictioncenter.llnl.gov/casp5/) defines two residues as forming an inter-residue contact if the C$\beta$–C$\beta$ distance is less than or equal to 8 Å. The light gray horizontal lines in Figure 2A and Supplementary Figures 2 and 3 show this 8 Å cutoff position. A CASP long range contact is defined as any inter-residue contact where the two positions are separated by more than eight amino acids in the primary sequence. We have removed all residue pairs from our data set that were within eight amino acids of each other in the PDB sequence. Our results for ELSC and SCA, therefore, can be used to predict long range residue contacts in accordance with the CASP guidelines. CASP defines accuracy as the number of correctly predicted residue contacts divided by the number of residue pairs submitted. Supplementary Figure 4 online shows for all 138 Pfam families the accuracy for ELSC, SCA and a random pairing algorithm as a function of the number of predictions that each algorithm was asked to make. Figure 3 shows the average values of these 138 traces. Again, we see that ELSC on average outperforms SCA, although for both algorithms the performance is modest and the accuracy decreases rapidly as a function of the number of predictions made.

Downloaded from bioinformatics.oxfordjournals.org by guest on April 9, 2011

Inspection of Figure 2A and Supplementary Figures 2 and 3 suggests that focusing only on residue contacts does not take full advantage of the power of either ELSC or SCA. In Figure 2A, for example, there are a significant number of high scoring residue pairs that are well below the 50th percentile of distance (horizontal dark gray line) but are not below the light gray 8 Å cutoff line. It is clear that true co-variance can happen outside of the residue contact cutoff. In order to compare the algorithms in a way that takes into account co-variation that happens at distances greater than 8 Å, we looked for a way to average pair distance and algorithm score across all 138 Pfam families. To account for the fact that different alignments can produce very different ranges of co-variance scores, we converted scores for both algorithms to percentiles and plotted this on the $x$-axis. Because different proteins have different average volumes, we likewise converted the pair distance scores to percentiles and plotted this percentile on the $y$-axis. We then asked for both algorithms what the relationship is between score percentile and pair distance percentile. Supplementary Figures 5 and 6 online show the results of this analysis for each of the 138 protein families in our study, and supplementary Figure 7 shows the average of these 138 results for both algorithms. By this metric, the ELSC algorithm has, on average, more power than the SCA algorithm as the most highly co-varying pairs of residues tend to be closer to each other for ELSC than for SCA.

## CONCLUSION

In this study, we have introduced a new perturbation-based correlated mutation algorithm. A major motivation for this work is the intriguing possibility that analysis of evolutionary co-variation may reveal functional couplings in a protein that are not immediately apparent from an analysis of residue proximity. Although it was not our goal in this work to predict residue contacts *per se*, we have used contact prediction as a quantifiable surrogate measurement with which to compare the performance of two co-variation algorithms. By the metric of either prediction of residue contacts (Fig. 3) or the probability that a random algorithm could match the performance of the top scoring residue pairs (Fig. 2B), our ELSC algorithm outperformed the previously described SCA algorithm on a set of 138 Pfam families. It has been argued elsewhere (Fodor and Aldrich, 2004) that a small part of the performance differences between correlated mutation algorithms in finding physically close residue pairs can be explained by the preference of some correlated mutation algorithms to choose more conserved residues, which tend to be more clustered. However, the average sequence entropy, $H_i$ (see Detailed methods section), of the top 75 scoring pairs of residues for all 138 Pfam families was $1.61 \pm 0.35$ (mean $\pm$ SD) for ELSC and $1.60 \pm 0.42$ for SCA. The fact that ELSC and SCA give high scores to co-varying pairs with similar levels of background conservation rules out differences in background conservation as an explanation for the difference in power between the two algorithms.

A previous study (Fodor and Aldrich, 2004) evaluated a number of other correlated mutation algorithms, two of which (Gobel *et al.*, 1994; Kass and Horovitz, 2002) are based on an analysis of global MSA statistics rather than an analysis of perturbation subsets. The ELSC method is most appropriate to use when there is an experimental reason to create a sub-alignment, such as a mutation at a given column position that has been found to alter ligand binding, allosteric coupling or conformational equilibria. In this case, the ELSC algorithm might be used to predict residues that are energetically coupled to the altered residue. If, on the other hand, one is approaching an alignment with no a priori knowledge of experimental perturbations, the use of a non-perturbation based algorithm may be more appropriate. By choosing the most appropriate algorithm for the problem at hand, one can maximize the odds that a correlated mutation algorithm can be used to gain useful insight and guide experimental analysis.

## REFERENCES


Altschuh,D., Lesk,A.M., Bloomer,A.C. and Klug,A. (1987) Correlation of co-ordinated amino acid substitutions with function in viruses related to tobacco mosaic virus. *J. Mol. Biol.*, **193**, 693–707.

Atchley,W.R., Terhalle,W. and Dress,A. (1999) Positional dependence, cliques, and predictive motifs in the bHLH protein domain. *J. Mol. Evol.*, **48**, 501–516.

Atchley,W.R., Wollenberg,K.R., Fitch,W.M., Terhalle,W. and Dress,A.W. (2000) Correlations among amino acid sites in bHLH protein domains: an information theoretic analysis. *Mol. Biol. Evol.*, **17**, 164–178.

Fodor,A. and Aldrich,R. (2004) *Correlated Mutations as a Function of Conservation in Protein Multiple Sequence Alignments*. *Bioinformatics*, In press.

Gobel,U., Sander,C., Schneider,R. and Valencia,A. (1994) Correlated mutations and residue contacts in proteins. *Proteins*, **18**, 309–317.

Kass,I. and Horovitz,A. (2002) Mapping pathways of allosteric communication in GroEL by analysis of correlated mutations. *Proteins*, **48**, 611–617.

Larson,S.M., Di Nardo,A.A. and Davidson,A.R. (2000) Analysis of covariation in an SH3 domain sequence alignment: applications in tertiary contact prediction and the design of compensating hydrophobic core substitutions. *J. Mol. Biol.*, **303**, 433–446.

Lockless,S.W. and Ranganathan,R. (1999) Evolutionarily conserved pathways of energetic connectivity in protein families. *Science*, **286**, 295–299.

Neher,E. (1994) How frequent are correlated changes in families of protein sequences? *Proc. Natl Acad. Sci., USA*, **91**, 98–102.

Olmea,O. and Valencia,A. (1997) Improving contact predictions by the combination of correlated mutations and other sources of sequence information. *Fold Des.*, **2**, S25–S32.

Olmea,O., Rost,B. and Valencia,A. (1999) Effective use of sequence correlation and conservation in fold recognition. *J. Mol. Biol.*, **293**, 1221–1239.


Downloaded from bioinformatics.oxfordjournals.org by guest on April 9, 2011

Press,W.H., Teukolsky,S.A., Vetterling,W.T. and Flannery,B.P. (1995) *Numerical Recipes in C*, 3rd edn. Cambridge University Press, Cambridge.

Shenkin,P.S., Erman,B. and Mastrandrea,L.D. (1991) Information-theoretical entropy as a measure of sequence variability. *Proteins*, **11**, 297–313.

Suel,G.M., Lockless,S.W., Wall,M.A. and Ranganathan,R. (2003) Evolutionarily conserved networks of residues mediate allosteric communication in proteins. *Nat. Struct. Biol.*, **10**, 59–69.

Thomas,D.J., Casari,G. and Sander,C. (1996) The prediction of protein contacts from multiple sequence alignments. *Protein Eng.*, **9**, 941–948.

Downloaded from bioinformatics.oxfordjournals.org by guest on April 9, 2011
