# Supplementary material for: Accurate Simulation and Detection of Coevolution Signals in Multiple Sequence Alignments
Source: PLoS One. 2012 Oct 16;7(10):e47108. doi: 10.1371/journal.pone.0047108 (PMC3473043; doi:10.1371/journal.pone.0047108)
Supplement: Toolbox S1 — MSAVOLVE_v1.0a Toolbox for Matlab. (ZIP) [file pone.0047108.s024.zip › MSAVOLVE_v1.0a/COEVOLUTION_METHODS/FUNCTIONS/OMES/Cross_talk_conservation_correlation.pdf]

Influence of Conservation on Calculations of Amino Acid Covariance in Multiple Sequence Alignments

Anthony A. Fodor and Richard W. Aldrich
Department of Molecular and Cellular Physiology, and Howard Hughes Medical Institute, Stanford University School of Medicine, Stanford, California

ABSTRACT **It has long been argued that algorithms that find correlated mutations in multiple sequence alignments can be used to find structurally or functionally important residues in proteins. We examined the properties of four different methods for detecting these correlated mutations. On both simple, artificial alignments and real alignments from the Pfam database, we found a surprising lack of agreement between the four correlated mutation methods. We argue that these differences are caused in part by differing sensitivities to background conservation. Correlated mutation algorithms can be envisioned as "filters" of background conservation with each algorithm searching for correlated mutations that occur at a different background conservation frequency. Proteins 2004;56:211–221.** © **2004 Wiley-Liss, Inc.**

Key words: structure–function; protein engineering; genomics; evolution; Pfam; Protein Data Bank; covariance

INTRODUCTION

It has long been realized that correlated mutation information in multiple sequence alignments can be used to predict residue contacts in proteins.[1–8] More recently, it has been suggested that a member of this class of algorithms can be used to find energetically linked chains of functionally important residues.[9–11] The idea behind correlated mutations is simple: if every time a given residue in a column of an alignment changes there is a corresponding change in another column of the alignment, then these residue positions may be linked either functionally, energetically or by virtue of being in physical proximity in some important conformation of the protein.

By their nature, algorithms that measure correlated mutations have to favor an intermediate level of conservation. If, on the one hand, one or both columns in a protein alignment are not at all conserved, that is, are completely random, then there can be only spurious correlations between the two columns and application of any correlated mutation algorithm should yield a low score. A perfectly conserved column, on the other hand, presents a conceptual challenge to covariance; a correlated mutation algorithm is supposed to detect how the changes in column i effect column j. If there are no changes in a column, a correlated mutation algorithm must choose to report no score, a perfectly high score, or a perfectly low score.

This article examines four correlated mutation algorithms that were chosen from among the many algorithms described in the literature. We construct simple, artificial alignments and show that these four algorithms make very different choices in their description of the intermediate level of conservation that defines covariance, with some algorithms favoring more conserved conditions than others. We suggest that this choice of how to "filter" conservation defines much of the functionality of correlated mutation algorithms as seen in a collection of 224 Pfam alignments.

METHODS

Generation of Predictions of Distance from Pfam Database

The Pfam 7.7 (October 2002) text archive was downloaded from the Pfam[17] database (http://pfam.wustl.edu/). We removed from our data set any Pfam alignment that did not have an associated PDB file as indicated by the GF DR comment line. For performance reasons, we removed any Pfam alignment from our data set that had more than 1000 sequences. Using the CLUSTALW program[23] with the default parameters, each sequence with the gaps removed in each remaining protein family was aligned against the sequence of each chain in each PDB file[18] as specified by the Pfam alignment's GF DR comment line. Any structure that gave duplicate coordinates for any atom was excluded. For each Pfam alignment, the PDB file with the longest >95% identity match to a sequence in that Pfam alignment was selected. We refer to the row in a Pfam alignment that had the best match to a chain in the chosen PDB file as the reference sequence. Using CLUSTALW, we created for each protein family a reference alignment between the reference sequence and the sequences of all of the chains in the chosen PDB file that were specified by the GF DR comment. We excluded from our study any columns in a Pfam alignment for which the reference sequence had a gap. Also excluded from each Pfam alignment were any columns in which the residue in the reference sequence did not perfectly match the residues in all of the PDB chains in the reference alignment.

Grant sponsor: Mathers Foundation.

*Correspondence to: R.W. Aldrich, Department of Molecular and Cellular Physiology, and, Investigator, Howard Hughes Medical Institute, Stanford University School of Medicine, Stanford, CA 94305-5345. E-mail: raldrich@stanford.edu.

Received 24 July 2003; Revised 4 December 2003; Accepted 11 December 2003

Published online 14 May 2004 in Wiley InterScience (www.interscience.wiley.com). DOI: 10.1002/prot.20098

© 2004 WILEY-LISS, INC.

This guaranteed that every pair of Pfam columns for which we generated a conservation or covariance score could be mapped with high confidence to a pair of residue positions in the chosen PDB file.

For each of the chosen PDB files, the distances between pairs of residues were measured as Cβ–Cβ distances (Cα for glycine). In the case of multichain proteins, the smallest distance was used. For example, the distance between positions 3 and 7 in our reference alignment of a crystal structure with chains A and B would be the smallest distance between A3-A7, A3-B7, B3-A7, and B3-B7. Each Pfam alignment for which a PDB file had been chosen was filtered so that any redundant sequences (sequences with >90% identity to another sequence in the alignment) were removed. This was accomplished by creating a new alignment and adding sequences one at a time from the old alignment where sequences were added only if they had <90% identity to all sequences already in the new alignment. All columns with >50% gapped residues were then removed from the alignments. A gap was considered as any character that was not a valid, upper case symbol for an amino acid. Alignments that had fewer than 100 such columns with >50% nongapped residues were removed from the analysis set. Any alignment that had fewer than 100 protein sequences in the nonredundant alignment was also removed from the analysis set. There were a total of 224 protein families that met all of our criteria and were retained in the analysis set. To correct for any possible artifacts resulting from residues close to each other in the protein sequence, any pair of residue positions that were within eight residues of each other in the sequence of the PDB file were discarded from all analyses.

Note that the SCA algorithm has a free parameter that has the effect of excluding poorly conserved columns from the alignment (http://www.hhmi.swmed.edu/Labs/rr/world/sca/sup_figure2.pdf). Because a goal of our study was to examine the effect of conservation on correlated mutations, we did not use this parameter and therefore included poorly conserved columns in our study. This decision negatively impacted the power of the SCA algorithm, which has a tendency to assign an excessively high score to poorly conserved columns [Fig. 1(B)] and therefore benefits to a modest degree from the removal of these columns from the alignment. Even with these poorly conserved columns removed, however, SCA still underperforms OMES and McBASC and outperforms MI (data not shown). In another study we have described the behavior of the SCA algorithm with the most poorly conserved residues removed and made some suggestions for improving SCA's performance.[22]

Unlike the other correlated mutation algorithms, the SCA algorithm is not symmetrical in the scores it generates. That is, SCA(i, j) does not equal SCA(j, i) because the first column is used to define the "perturbation" that creates the subalignment. In the original SCA article,[10] SCA scores were reported based on the perturbation of the first column without consideration of the SCA(j, i) score. In subsequent articles[9,11] the SCA(i, j) and SCA(j, i) scores might appear in separate columns in a matrix of SCA scores, although the presence of SCA(i, j) guarantees neither the presence nor absence of SCA(j, i) because poorly conserved columns in the alignment are removed from consideration as columns, but not rows, in the SCA score matrix. In our study, in order to make a single prediction of pair distance, we need to generate a single score for each pair of columns in the alignment. To do this, we constrained the relationship between i and j so that it was always true that $j > i$. So, for example, for columns 1 and 5 in an alignment, we used the most conserved residue in column 1 to form the subalignment and reported the SCA score for SCA(1, 5) but not SCA(5, 1). One can imagine a number of strategies for combining SCA(i, j) and SCA(j, i) scores that might effect the power of the SCA algorithm. For example, one could average the two scores, take the higher of the scores, and so forth. Because these sorts of strategies were not part of the original SCA description, however, we have not evaluated the effects of these strategies in this study.

Our list of the 224 Pfam families included in this study is available in the Supplementary Materials online. The Java code used in this article is available at http://www.afodor.net.

Calculation of *p* Values

For both covariance and conservation calculations, the predictions of pair distance for the highest 75 scoring pairs of residues was chosen. We then counted the number of these pairs that was less than or equal to the 50th percentile of all pair distances as calculated for that Pfam family. We then asked what was the probability that an algorithm that chooses pairs of columns at random could choose as many residue pairs less than or equal to the 50th percentile. Because our analysis was limited to alignments with at least 100 columns, there is a minimum of ~5000 ($100^2/2$) possible pairs of residues for each alignment. Because this number is so large, choosing the first 75 pairs of residues can be very closely approximated as choosing with replacement and the probability is therefore almost exactly described by the binomial distribution. Therefore, the probability that we are seeking is given by

$$\sum_{n}^{75}\left(\frac{75!}{n!(75-n)!}\right) 5^{n} \times 5^{75-n},$$

where n is the number that the algorithm chose within the first 75 paris that were less than or equal to the 50th percentile.

Note that our choice of 75 pairs of residues and the 50th percentile in this equation is arbitrary. For example, we could have instead examined the first 25 or 100 pairs of residues instead of 75 or required that pair distances be within the 10th or 25th percentile instead of the 50th percentile. We have found, however, that the relative power of the conservation and four covariance algorithms is not substantially different even when, for example, examining only the first 25 residues and requiring that pair distances be within the first 10th percentile (data not shown). We argue, therefore, that whereas the choice of 75

residues and the 50th percentile is arbitrary, using these parameters gives a reasonable estimate of the power of each of the algorithms.

RESULTS

Observed Minus Expected Squared (OMES) Covariance Algorithm

Our first covariance analysis (which we call OMES) is derived from the covariance method of Kass and Horovitz.[12] For every possible pair of columns (column i vs. column j), generate a list L of all distinct pairs of amino acids. Discard any pairs that have a gap at either i or j. The score for each column pair i, j is given by

$$\sum_{1}^{L} \frac{(N_{\text{obs}} - N_{\text{ex}})^2}{N_{\text{valid}}},$$

where N_{valid} is the number of sequences in the alignment that have nongapped residues at both positions i and j, N_{obs} is the number of times that each distinct pair of residues was observed, and N_{ex} is the number of times that each distinct pair of residues would be expected based only on the frequency of each residue in each column. The value of N_{ex} for a given pair with residue x at position i and residue y at position j can be calculated by

$$N_{\text{ex}} = \frac{C_{xi}C_{yj}}{N_{\text{valid}}},$$

where c_{xi} is the number of times residue x occurs at position i and c_{yj} is the number of times y occurs at position j. Table SI in the Supplemental Materials online shows how two columns in a short hypothetical alignment would be scored under the OMES covariance scheme.

Note that for any two perfectly conserved columns, the covariance algorithm must give a score of zero. If column x has only residue i and column y has only residue j, then $N_{\text{ex}} = N_{\text{valid}} = N_{\text{obs}}$ and the OMES score reduces to zero.

Mutual Information (MI) Covariance Algorithm

Our implementation of the MI algorithm follows the description given by Atchley et al.[13] According to this algorithm, "the extent of 'correlation' or association between residues at amino acid sites X and Y that might arise from evolutionary, functional, or structural constraints" is defined as

$$\sum_{j=1}^{n}\sum_{k=1}^{m} p_{jk} \log \frac{p_{jk}}{p_j q_k},$$

where one column has n different kinds of residues, the other column has m different kinds of residues, p_j is the probability of residue type j being in the first column, q_k is the probability of residue type k being in the second column, and p_{jk} is the number of sequences with both j in the first column and k in the second column divided by the total number of sequences.[14] Note that in the case of either i or j being perfectly conserved, $p_j \times q_k = p_{jk}$ and the assigned score reduces to zero. In calculating p_j, q_k, *and* p_{jk} frequencies for each pair of columns, we only included sequences that were ungapped at both residue positions.

Statistical Coupling Analysis (SCA) Covariance Algorithm

We used the SCA software package Windows binaries that were generously provided by Rama Ranganathan (University of Texas, Southwestern Medical School). These algorithms have been previously described.[10] Briefly, this method of detecting correlated mutations works by creating subalignments and asking if a subalignment has a changed residue composition compared to the parent alignment from which it was drawn. Following the original articles,[9–11] we chose the most conserved residue in each column as our "perturbation," meaning that each subalignment generated for each column consisted of all the sequences that had the most conserved residue at that position.

McLachlan Based Substitution Correlation (McBASC)

Our implementation followed the description provided by Olmea et al.,[2] which in turn was based on an earlier article by Gobel et al.[3] If N is the number of sequences in the alignment, for each column i in the alignment construct a two-dimensional $N \times N$ matrix running from $k = 1$ to N in one dimension and from $l = 1$ to N in the other dimension. Each entry in the matrix is the value from a substitution matrix that assigns a high score if there is an identity or conservative substitution for the pair of residues in sequences k, l at column i and a low score if there is a nonconservative substitution. Following Olmea et al.,[2] we used the McLachlan substitution matrix.[15] $\langle s_i \rangle$ is the average and σ_i is the standard deviation of all the entries in the $N \times N$ matrix. The correlation score between two columns i and j is defined as

$$r_{i,j} = \frac{1}{N^2} \frac{\sum_{kl} (s_{ikl} - \langle s_i \rangle)(s_{jkl} - \langle s_j \rangle)}{\sigma_i \sigma_j}.$$

This value ranges from $-1 \leq r \leq +1$ with a score of $+1$ indicating highly covarying columns. Note that $r_{i,j}$ is undefined if either column i or j is perfectly conserved as for all entries $s - \langle s \rangle = 0$ and hence $\sigma = 0$. We therefore follow the previous implementations and remove perfectly conserved columns from our analysis of the McBASC algorithm. It can be shown, however, that the limit of $r_{i,j}$ approaches $+1$ as columns i and j approach perfect conservation. This is a consequence of $r_{i,j}$ being equal to $+1$ for any two columns that are identical to each other. If columns i and j are identical, then the $N \times N$ matrices for the two columns are identical and hence

$$\langle s_i \rangle = \langle s_j \rangle,$$

$$\sigma_i = \sigma_j,$$

$$s - \langle s_i \rangle = s - \langle s_j \rangle \quad \forall\, k,l.$$

For this special case $r_{i,j}$ reduces to

$$r_{i,j} = \frac{1}{N^2}\frac{\sum_{kl}(s-\langle s\rangle)(s-\langle s\rangle)}{\frac{\sum_{kl}(s-\langle s\rangle)(s-\langle s\rangle)}{N^2-1}} = \frac{N^2-1}{N^2}.$$

This reduces to ~1 for any alignment with a nontrivial number of sequences. The McLachlan matrix gives very similar scores for all 20 residues when a residue is substituted by itself. As a consequence of this the McBASC algorithm will tend toward a high covariance score for highly conserved pairs of columns in an alignment. This is a conceptually legitimate choice, but it is a different choice than that made by the SCA, OMES, and MI algorithms which give a score of zero (or approaching zero for SCA) if column i or j or both are perfectly conserved columns.

For performance reasons, the average and standard deviation of the matrix for each column i in the alignment was cached. In calculating these cached values, N^2 was considered the number of entries in the matrix that were ungapped for all pairs of sequences k and l in column i. When calculating the final McBASC score for the (i,j) column pair, a score of zero was assigned if there was a gap in either sequence k or l at either column i or j. N^2 was then simply considered the square of the number of sequences in the alignment. In addition, in order to fairly compare McBASC with algorithms such as OMES in which negative correlations could receive high scores, we reported the absolute value of the McBASC score. Java code for McBASC, and all other algorithms, is available at http://www.afodor.net

Conservation Algorithm

We take as our conservation measure the absolute sequence entropy[16]:

$$-\sum_{x}(p_x(i)\ln p_x(i))$$

where i is the column of interest, x spans the 20 possible amino acid residues, and $p_x(i)$ is the frequency of residue x at position i. In order to compare the results of the conservation algorithm to the covariance algorithm, the conservation score of pairs of positions were averaged.

Performance of Correlated Mutation Algorithms on Simple, Artificial Alignments

All metrics of correlated mutations must give higher scores to intermediate levels of conservation. If two columns of an alignment are not at all conserved, that is, both are completely random, there can be few correlated mutations and all covariance algorithms should give these random columns a low score. On the other hand, perfectly conserved columns present a problem for correlated mutation algorithms. Correlated mutation algorithms are meant to score how changes in one column effect changes in another. In a perfectly conserved column, there are no changes.

To illustrate how the different correlated mutation algorithms handle this issue of background conservation, we constructed simple two-column artificial alignments. Each point in Figure 1(A) shows the results of running the algorithms over a different 1000-sequence two-column alignment starting with an alignment consisting of 1000 MM sequences on the left of the x axis and ending with 1000 YY sequences on the right of the x axis. Moving from left to right on the x axis, each point represents an alignment in which one MM row in the alignment is replaced with YY. The green curve represents the average pair conservation score. As expected, we see that the perfectly conserved sequences at $x = 0$ and 1000 receive the highest conservation scores. The most highly covarying alignment in the set of alignments in Figure 1(A) occurs at $x = 500$, which has 500 MM sequences and 500 YY sequences. As expected, all four correlated mutation algorithms give a high score to this highly covarying alignment. The four correlated mutation algorithms, however, show dramatic differences in how they evaluate the other alignments. The SCA and OMES algorithms have relatively narrow "peaks" around the highly covarying alignment at $x = 500$. That is, as the conservation level moves away from perfectly intermediate, the scores of the SCA and OMES algorithms rapidly drop off, however. The MI algorithm has a wider "peak" around the highly covarying alignment whereas the McBASC algorithm gives an equally high score to conserved or covarying alignments. This reflects a fundamental difference between McBASC and the other correlated mutation algorithms. SCA, MI, and OMES choose to give a zero score to perfectly conserved columns whereas McBASC gives a high score to columns that approach being perfectly conserved. That is, McBASC allows, without a reduction in score, substitution of conserved pairs of residues for covarying ones whereas the other algorithms approach zero as perfect conservation is approached.

We can further elucidate the differences between these algorithms by constructing another set of 1000 artificial, two-column alignments each with 1000 sequences. Figure 1(B) shows the performance of the algorithms on a set of alignments starting with 1000 random two residue sequences (at $x = 0$) and ending with 1000 perfectly conserved YY sequences (at $x = 1000$). The value on the x axis is therefore the number of YY sequences in the alignment and $(1000 - x)$ is the number of random sequences in the alignment. As expected, the average conservation score (green curve) increases as the number of random sequences in the alignment decreases. We see that in the background of random sequences the four correlated mutation algorithms yield dramatically different results with each algorithm giving high scores to different alignments. By this view, correlated mutation algorithms are "filters" of background conservation with each algorithm having a different sensitivity to a different background conservation "frequency."

Several characteristics of Figure 1(B) are worth pointing out in more detail. One is that the MI algorithm is the only one of the correlated mutation algorithms that gives a nonzero score to the completely random sequences. This reflects the fact that, even with 1000 random sequences, p_j

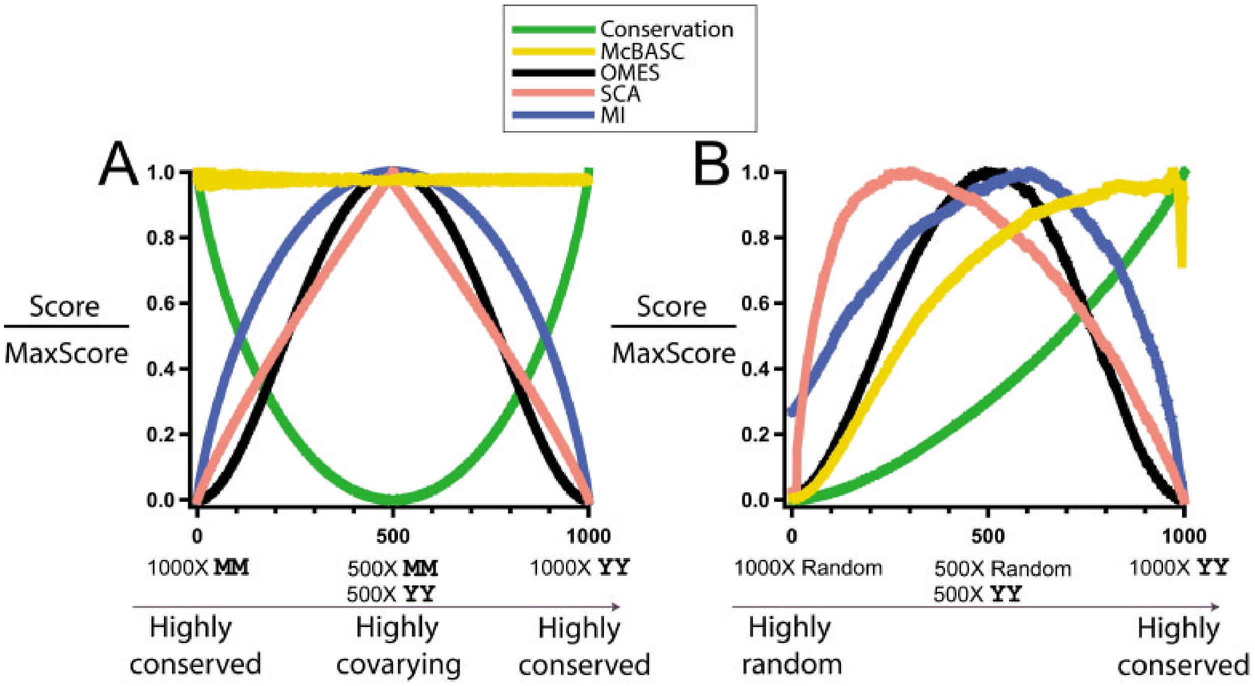

Fig. 1. The performance of the conservation, McBASC, OMES, SCA, and MI algorithms on simple, artificial two-column alignments. Each panel shows the results of running the algorithms over 1000 two-column alignments each with 1000 sequences. (**A**) The leftmost alignment ($x = 0$) consists of 1000 sequences with residues MM. For each point on the x axis an MM row is removed from the alignment and is replaced with a YY. At $x = 500$, therefore, there are 500 MM sequences and 500 YY sequences. (**B**) The leftmost alignment ($x = 0$) consists of 1000 two-column sequences with a residue inserted randomly at each position. For each point on the x axis, one random sequence is replaced with a YY. At $x = 500$, therefore, the alignment consists of 500 YY sequences and 500 random sequences. Because low sequence entropy scores indicate a highly conserved column (see Methods section), conservation scores in both panels (green) are plotted as $1 - (\text{score}/\text{MaxScore})$, so that toward the top on the y axis indicates a highly conserved column pair.

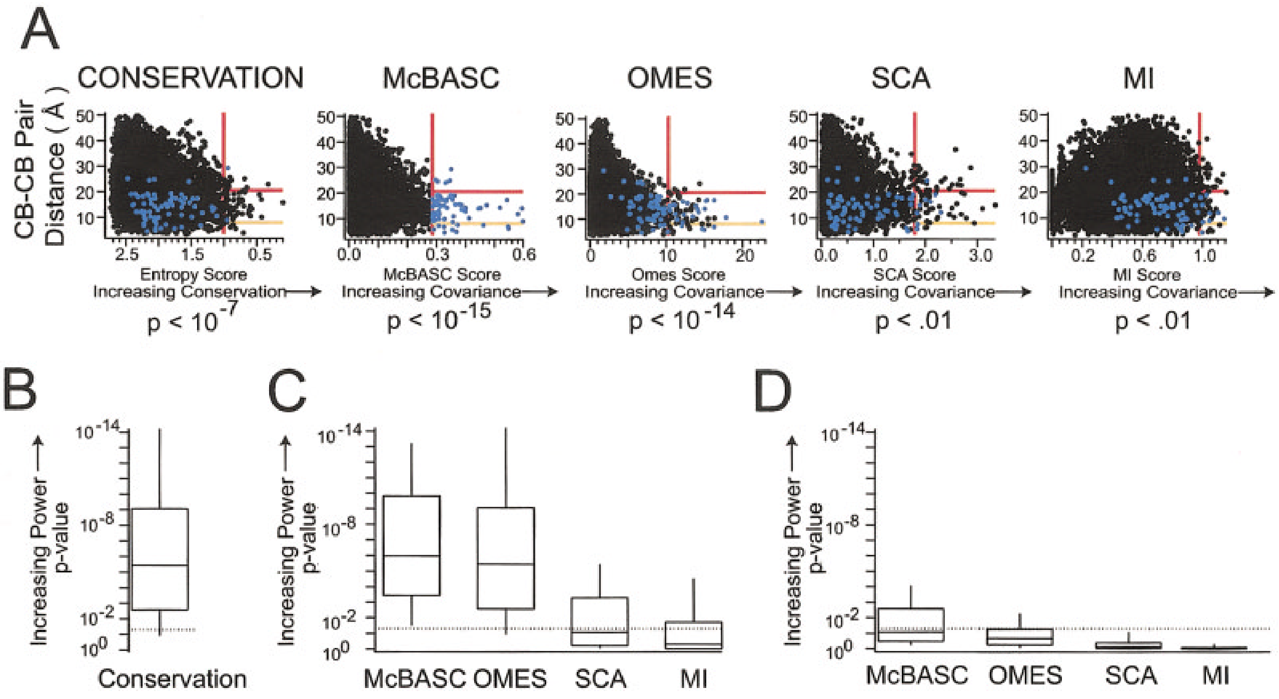

Fig. 3. The performance of the conservation, McBASC, OMES, SCA, and MI algorithms in predicting pair distance. (**A**) The Cβ–Cβ pair distance for 10,395 residue pairs from the PDB file 1cli, which corresponds to the PUR5_ECOLI line in the AIRS_C Pfam alignment. (vertical red lines) The 75 highest scoring pairs of residues for each algorithm; (horizontal red lines) the 50th percentile of all pair distances; and (horizontal yellow lines) the 8 Å cutoff, which is the CASP cutoff for residue contacts.[24] The probabilities shown below each graph are the odds that random pairing could do as well in finding physically close residues for the highest scoring 75 pairs of residues (see Methods section). (Blue symbols) The 75 highest scoring pairs of residues for the McBASC algorithm. (**B**, **C**) The probability for the 224 protein families in our study that a random pairing algorithm could match the performance of the (**B**) conservation and (**C**) correlated mutation algorithms for the top 75 scoring residue pairs. (- - -) The $p < 0.05$ level. (**D**) The power of the algorithms for all 224 protein families over sets of 75 residue pairs that have the same background conservation as (**C**) but for which the covariance has been ignored (see text).

$\times\ q_k$ is not always exactly p_{jk} but MI's division of these terms by one another can exaggerate these random differences. Likewise, SCA tends to favor highly random alignments. As we shall see in the real alignments below, MI and SCA tend to give high scores to poorly conserved pairs of columns. This may be explained in part by their tendency to be "tricked" by random or near random columns.

The "noise" characteristics of the McBASC algorithm in Figure 1(B) are also interesting. One can see that as the McBASC algorithm approaches the perfectly conserved alignment at $x = 1000$, the noise, or the difference in score between each immediately adjacent alignment, is substantially increased. This makes intuitive sense as we are approaching a division by zero error.

Covariation as a Function of Conservation in Real Protein Alignments

We have seen for very simple, artificial alignments that the four correlated mutation algorithms have very different sensitivities to background conservation. Real protein alignments are, of course, much more complicated than the simple alignments used in Figure 1. To begin to understand the properties of the correlated mutation algorithms on real alignments, we turned to the Pfam database, a collection of 4832 protein families.[17] For each of these protein families, we selected alignments that met certain criteria for size and diversity and that had at least one protein sequence with a >95% pairwise identity to a sequence of a crystal structure from the Protein Data Bank (PDB[18]; see Methods section). There were 224 Pfam families that met all of our criteria and were included in our study. Figure 2(A) shows the results for a single Pfam family, AMINO_OXIDASE. Each panel shows all 65,137 residue pairs except for the McBASC panel in which the 358 residue pairs in which either or both columns are perfectly conserved have been removed (as required for that algorithm). For each algorithm, the normalized score is plotted against the pair conservation score. We see that, as is the case for the artificial alignments in Figure 1, the covariance algorithms appear to be sensitive to different levels of background conservation. In particular, SCA and MI appear to favor poorly conserved residue pairs when compared to McBASC and OMES. As we expect from Figure 1, McBASC does give high scores to some of the more conserved column pairs whereas the other three algorithms give reliably low scores to highly conserved columns. This comparison is complicated, though, by the fact that the residue pairs in which either column is perfectly conserved have been removed from the McBASC panel.

The data in Figure 1(A) from a single Pfam family support the idea that each of the covariance algorithms has a favored level of background conservation, but does this trend hold in general for all Pfam families? To address this question, we made an arbitrary choice of the 75 pairs of residues with the highest covariance score under each algorithm. Figure 1(B) shows for each of the 224 Pfam families in our study the average pair conservation of the highest scoring 75 pairs of residues under each algorithm. The sensitivity to conservation shows a clear trend with algorithms favoring increasingly poorly conserved residue pairs in the order McBASC > OMES > SCA > MI.

Algorithm Performance in Finding Clustered Residue Pairs

We have, so far, examined the influence of conservation in assigning correlated mutation scores. We now examine the power of these algorithms in predicting protein structure and function. Figure 3 shows the ability of each algorithm to find physically close pairs of residues. Figure 3(A) shows all 10,395 residue pairs for the AIRS_C Pfam family. For the McBASC algorithm, the 135 residue pairs involving one or two perfectly conserved columns have been removed. For all panels, Cβ–Cβ pair distances were generated from the PUR5_ECOLI sequence within the AIR_C alignment corresponding to the PDB structure 1cli. Supplementary Figures S1–S5 online show similar score versus pair distance plots for all 224 Pfam families in our study.

We have seen that the different correlated mutation algorithms are sensitive to different levels of background conservation in assigning scores to the top 75 pairs of residues (Fig. 2). The blue dots in all panels of Figure 3(A) are the top scoring 75 pairs of residues for the McBASC algorithm. We see that the four correlated mutation algorithms make different predictions for the top 75 scoring residue pairs. For example, both the MI and McBASC algorithms and the SCA and McBASC algorithms share only 9 of their top 75 scoring pairs of residues. McBASC has more in common with OMES, but, even so, only 24 out of the top 75 pairs of residues are shared. As we would expect from their differing sensitivities to background conservation, the covariance algorithms on average assign high scores to distinct residue pairs.

One can see by visual inspection that the pairs of residues with the highest scores under the OMES, McBASC and conservation algorithms tend to be physically close to each other. But are these trends statistically significant? We constrain this question as follows: consider the 75 most highly conserved and covarying pairs of residues [those to the right of the vertical red lines, Fig. 3(A)]. What are the odds that by choosing pairs of columns at random, one could do as well as the conservation and covariance algorithms in choosing pairs of residues below the 50th percentile of all Cβ–Cβ distances [horizontal red lines, Fig. 3(A)]? In choosing 75 pairs at random, we would expect to choose 37.5 out of 75 at or below the 50th percentile. That is, one would expect by chance to have as many pairs of residues below the horizontal red line as above it. We see that for AIRS_C, the conservation algorithm in fact predicts 61 out of 75 pairs of residues falling below the 50th percentile. The probability that a random pairing algorithm could have done as well is $p < 10^{-7}$ (see Methods section). The same calculation is carried out for each of the correlated mutation algorithms and the resulting p value is shown under each panel in Figure 3(A). We

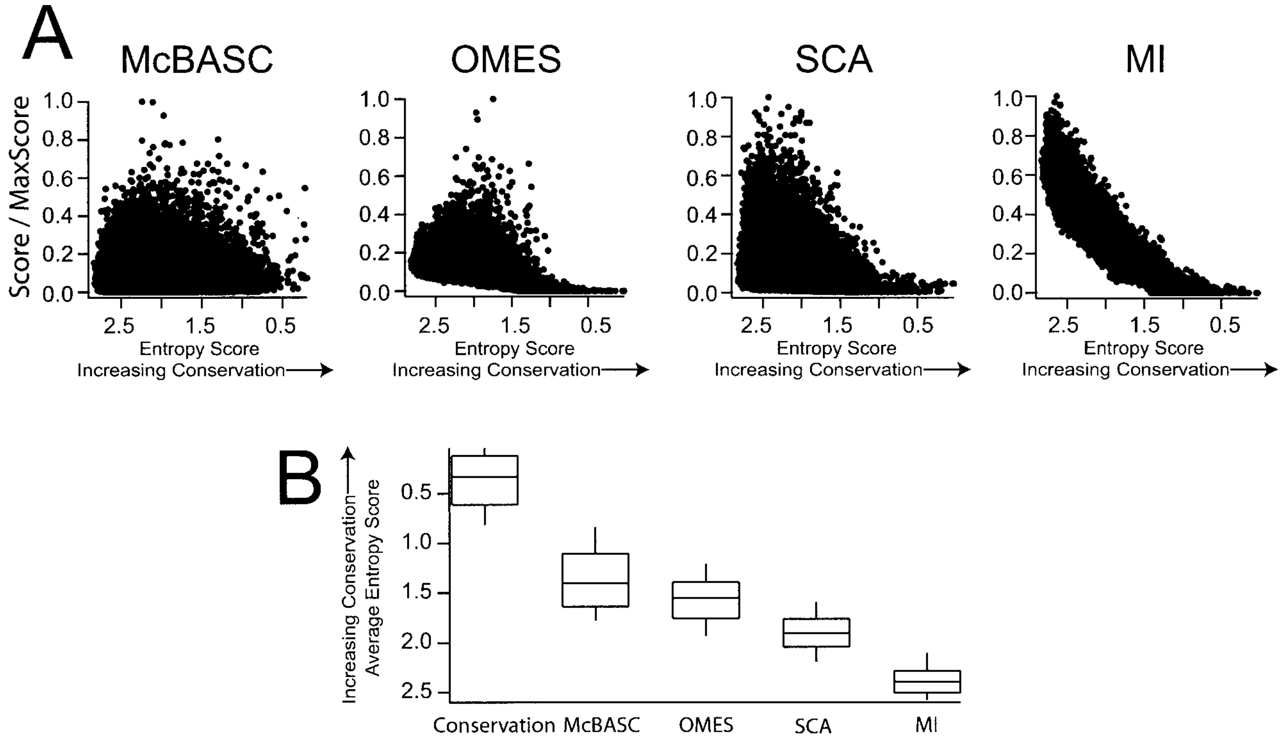

Fig. 2. Covariance as a function of conservation in Pfam alignments. (**A**) The four correlated mutation algorithms as a function of conservation for the Pfam family AMINO_OXIDASE. The covariance score for each algorithm is plotted against the pair conservation score. All panels show all 65,137 residue pairs except for the McBASC algorithm in which the 358 residue pairs in which either or both columns were perfectly conserved have been removed. (**B**) The average pair conservation score of the top scoring 75 pairs of residues for each algorithm for the 224 Pfam families included in our study. The box plot edges show the 25th and 75th percentiles, the line in the middle of the box plot indicates the 50th percentile, and the whiskers (vertical lines) indicate the 10th and 90th percentiles.

see that the SCA and MI correlated mutation algorithms have lower power for the AIRS_C alignment than do the McBASC and OMES algorithms.

In Figure 3(A) we have chosen a single Pfam family to demonstrate the properties of the conservation and correlated mutation algorithms. Figure 3(B) shows the probability of the null hypothesis that a random pairing algorithm could perform as well as the conservation algorithm in finding physically close pairs of residues for the top scoring 75 residue pairs in all 224 Pfam families. The dashed line indicates the $p = 0.05$ level. Clearly, the most conserved 75 residue pairs tend to be clustered in space. Figure 3(C) shows the results of this calculation for the correlated mutation algorithms. We see a wide difference in the power of these algorithms with McBASC and OMES displaying far more power than SCA and MI.

The Performance Differences of Correlated Mutation Algorithms Cannot be Explained by the Tendency of Conserved Residues to Cluster

As we have just seen [Fig. 3(C)], the different correlated mutation algorithms have different levels of performance with decreasing power in the order McBASC > OMES > SCA > MI. As we have seen in figure 2(B), the algorithms have decreasing sensitivity to background conservation in the same order McBASC > OMES > SCA > MI. The fact that conserved residues are clustered in space [Fig. 3(B)] raises the possibility that the performance differences between the algorithms are due to the fact that they are sensitive to different levels of conservation. That is, it seems possible that the performance differences between the covariance algorithms are not attributable to the ability to detect covariance but only to differences in the ability to find conserved, and hence clustered, residue pairs.

To address this possibility, we took for each algorithm for each protein family the set of 75 residue pairs with the highest covariance scores. For each family, we wanted to create an alternative set of 75 residue pairs that would have the same average background conservation as the original set of 75 but for which the covariance would be ignored. To do this, we calculated the average conservation of the 75 residue pairs in the original set. We then found the 75 residue pairs in each protein family with the average conservation closest to this calculated conservation. The data in Figure 3(D) shows the results generated for all 224 Pfam families from this new set of 75 residue pairs that have been constrained to have the same average conservation as in Figure 3(C) but in which the covariance was ignored. By visually subtracting Figure 3(D) from 3(C), therefore, one can get a sense of how much of the power of each algorithm is due to the ability to detect correlated mutations independent of the tendency for conserved residues to cluster together. We see that only a

small part of the performance of McBASC and OMES can be explained by the tendency of conserved residues to cluster. Therefore, we can conclude that, at least for Pfam alignments, McBASC and OMES are more successful in finding clustered covarying pairs than are SCA and MI. In other words, McBASC and OMES appear to look for covariance at a background conservation frequency where it is much more common in Pfam alignments than in the lower background conservation frequency examined by SCA and MI. SCA and MI do have some power, though, and generally find different pairs of residues than McBASC and OMES. It therefore seems likely that there are relatively rare correlated mutations that occur at poorly conserved frequencies that SCA and MI detect and McBASC and OMES do not.

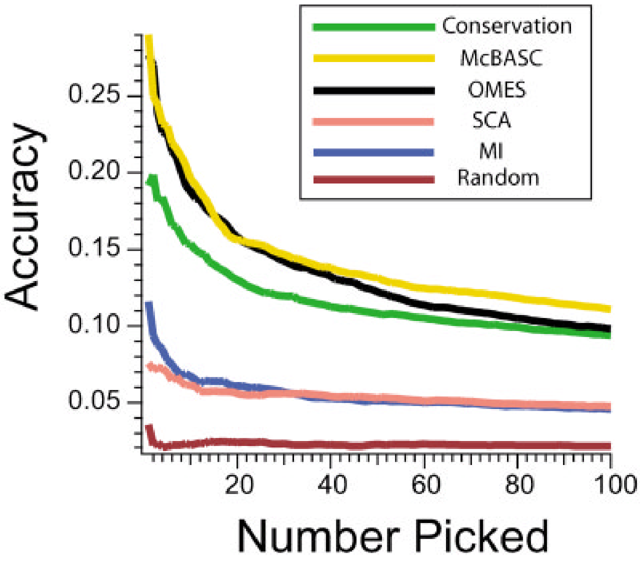

Fig. 4. The accuracy of the algorithms in predicting residue contacts. Accuracy is defined by CASP as the number of residue pairs correctly predicted within 8 Å divided by the number of residue pairs submitted for evaluation. Shown is the average accuracy for the 224 Pfam families in our study as a function of the number of residue pairs chosen.

Using Correlations to Predict Residue Contacts

The predominant use of covariance algorithms in the literature has been as predictors of interresidue contacts. According to CASP guidelines (http:/www.predictioncenter.llnl-.gov/casp5), a "long-range" interresidue contact is defined as two residues that are separated by at least nine residue positions in the linear sequence in which the Cβ–Cβ distance is ≤8 Å. Accordingly, in all of our analyses we removed any residue pairs that were within eight positions in the linear sequence. CASP defines accuracy as the number of correctly predicted residue contacts divided by the number of total predictions submitted. Supplementary Figures S6–S11 online show the accuracy for all 224 protein families as a function of the number of residues that each algorithm was asked to predict. For all of the algorithms, the accuracy goes down dramatically as the number of "submitted" predictions goes up. Figure 4 shows the average accuracy across all 224 protein families for each algorithm. The relative power of each algorithms is

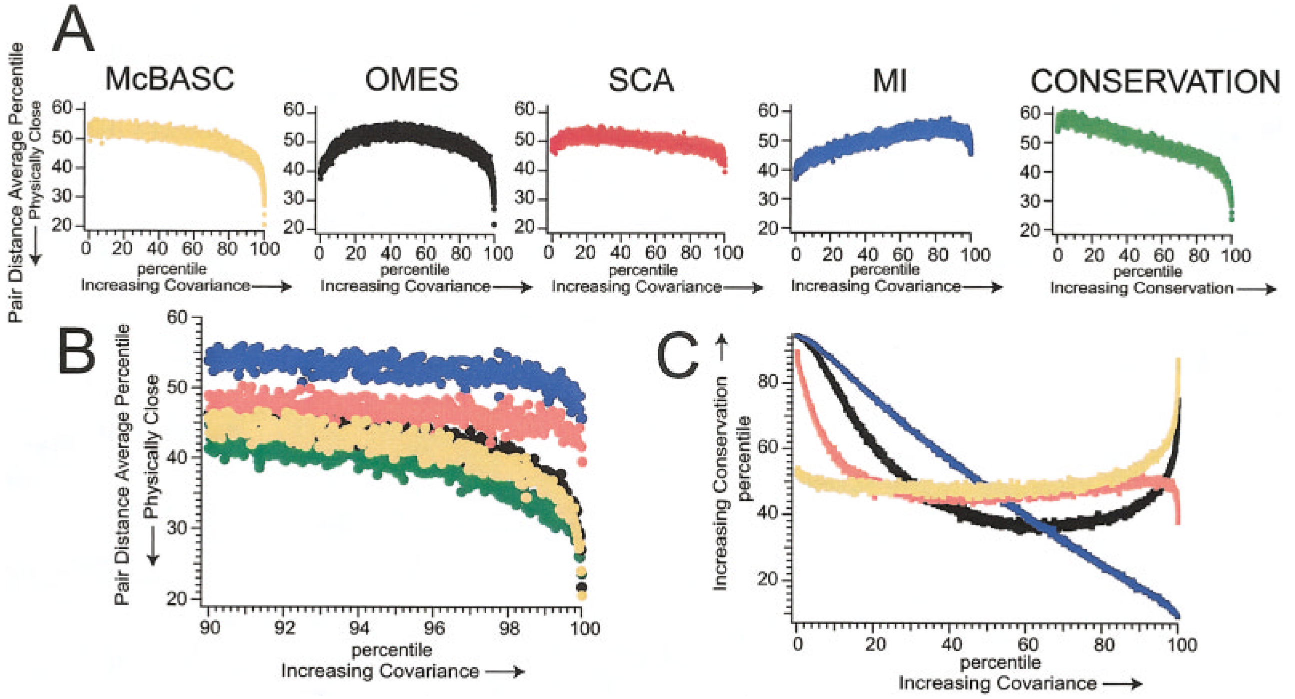

Fig. 5. Algorithm performance for multiple Pfam families. Performance of (yellow) McBASC, (black) OMES, (red) SCA, (blue) MI, and (green) conservation for all 224 Pfam families that met our criteria. Each panel is a histogram with each point on the *x* axis representing 0.025 percentile. So, for example, each of the rightmost points in (**A**) and (**B**) is the average pair distance percentile across all 224 Pfam families for conservation or covariance scores between the 99.975th and 100th percentile scores for each algorithm. (**A**) The performance of the algorithms in predicting pair distance. (**B**) The same data as in (**A**) with the *x* axis expanded to show only the 90th to 100th percentile. (**C**) The average conservation as a function of the covariance scores.

the same as we would expect from Figure 3 with McBASC and OMES outperforming SCA and MI. As we would anticipate from the previous literature, the overall accuracy is quite low, consistent with previous observations that covariance algorithms have only modest power in predicting interresidue contacts.[1–4] Because the algorithms generally choose distinct residues, it may be possible to combine them to produce an algorithm with higher overall power. This has already been achieved with the combination of McBASC and conservation for fold recognition.[1,2]

Using Correlations to Predict Energetic Connectivity

Not all of the suggested uses for correlated mutation algorithms have been for solving protein structures. Recently, more attention has been paid to using covariance algorithms to find functionally important residues.[19] In particular, it has been suggested that the SCA covariance algorithm can be used to successfully find "evolutionarily conserved pathways of energetic connectivity" in proteins.[9–11] In the laboratory, this argument contends, the energetic connectivity between two residues can be measured by mutating both residues independently and comparing the results to a double mutant. If the double mutant causes emergent free energy properties compared to the sum of the two single mutants, the two residue positions are considered energetically coupled.[20] We call the hypothesis that the results of correlated mutation algorithms can be correlated with the nonadditivity of double mutant cycle experiments the SCA hypothesis, after the article in which it was originally argued that the SCA algorithm is a "good indicator of thermodynamic coupling in proteins."[10]

In our study we do not directly consider energetic data as generated by double mutant cycles. We argue below, however, that our PDB distance data do reveal some inherent limitations of the SCA hypothesis. Unlike predicting residue contacts, which makes use of only the most highly covarying pairs of residues, the SCA hypothesis argues that information can be gleaned from the entire range of covariance scores. Figure 3(C) from Lockless and Ranganathan, for example, suggests that pairs of residue positions with intermediate scores under the SCA algorithm will have intermediate nonadditivity under double mutant cycle experiments. In order to evaluate the SCA hypothesis, we therefore needed to evaluate correlated mutation algorithms across the entire range of their scores. Correlated mutation algorithms can generate very different ranges of scores for different protein families. Proteins in the PDB can likewise have very different average Cβ–Cβ distances. To compare covariance scores and pair distances across protein families, we therefore transformed the covariance and pair distance scores for each protein family to percentiles. Supplemental Figures S12–S17 online show the relationship between covariance score percentiles and pair distance percentiles for each of the 224 Pfam families for each of the algorithms. Figure 5(A) shows the average values for the 224 Pfam families. The tendency for poorly covarying pairs to be physically clustered for OMES and MI can be explained by the fact that these algorithms give low scores to highly conserved columns and, as we saw in Figure 3(B), conserved residues also tend to be clustered.

Figure 5(B) shows the same data as Figure 5(A) except that the *x* axis has been expanded to show only the 90th to the 100th score percentiles. We see that for all four covariance algorithms the ability to predict pair distance occurs only for the highest scoring covarying pairs. As scores decrease toward the 90th percentile, the covariance algorithms rapidly approach the 50th percentile for pair distance, which is the pair distance percentile that one would generate by choosing pairs of residue positions at random (see Supplementary Fig. S17). In particular, the performance of the SCA algorithm approaches random for SCA scores below the 99th percentile. This suggests one problem with the SCA hypothesis: nearly all of the information that the SCA covariance algorithm provides is in the top 1% of covariance scores. SCA covariance seems unlikely to be a "good indicator of thermodynamic coupling in proteins"[10] if covariance scores below the 99th percentile all mean essentially the same thing.

Of course, one could at this point save the SCA hypothesis by arguing that the reason that 99% of all SCA scores appear to have no meaning is that thermodynamic coupling between residues is rare. Indeed, the fact that a modest subset of residue positions participate in the generation of high scores under the SCA algorithm has led the authors of the SCA algorithm to conclude as "a central result" that there exists "simplicity in the pattern of coupling between amino acids in proteins."[11] They argue, "Although, in principle, the pattern of all inter-residue interactions could be complex, reality seems to be much simpler."[11] We suggest as an alternative explanation that the observed "simplicity" is a result of the low power of the SCA covariance algorithm. The fact that a covariance algorithm does not successfully detect complex patterns of "coupling between amino acids" does not mean that these couplings do not in fact exist. Rather, viewing the world through a low power algorithm will inevitably yield a simple view of the world. It may turn out to be true that the vast majority of neighboring residues in proteins do not thermodynamically interact with one another. It would certainly make the job of understanding how proteins work easier if this were the case. However, the fact that a covariance algorithm has low sensitivity does not, in itself, provide any evidence for or against the hypothesis that protein energetics are reliably simple.

A defender of the SCA hypothesis might argue that energetic connectivity, which is the topic of the SCA hypothesis, and physical distance, which is what we measured in this study, do not necessarily have to be linked. The presence of covariation at a distance was in fact one of the results of the original SCA study.[10] Indeed, we see plenty of covariation at a distance as well. There are a significant number of pairs of residues in Figure 3(A), for example, that appear to be highly covarying but are above the yellow 8 Å residue cutoff line. However, the highly

covarying residues are still, to a statistically significant extent, closer to each other than one would expect at random. We argue that even if evolutionary covariance works at a distance on "chains" of energetically linked residues, the residues that are within a linked chain should, on average, be closer to each other than residues chosen at random. It would be extraordinary if SCA scores were reliably sensitive to energy below the 99th percentile when they are reliably indifferent to distance. A more likely explanation is that the SCA hypothesis is simply incorrect, at least for all but the most highly covarying pairs of residues.

Another problem with the SCA hypothesis is the complex relationship between conservation and covariance. Figure 5(C) shows conservation scores as a function of covariance for the four correlated mutation algorithms. As we would expect from our results on artificial alignments seen in Figure 1, the SCA algorithm yields low scores for highly conserved columns. This means that if the SCA hypothesis were true, performing a double mutant cycle experiment should yield a nearly perfectly additive double mutation under the SCA algorithm if either or both residue positions were perfectly conserved. That is, if this hypothesis were valid, then under the SCA algorithm no highly conserved residue position in a protein could ever cooperate with any other residue position to produce a large emergent, nonadditive effect on free energy. This prediction simply cannot be true and is clearly incompatible with existing experimental data. For example, it has been shown through double mutant cycle analysis that two highly conserved residues in the C-terminal of the human BK potassium channel do not have independent effects on the function of the channel.[21] It seems unlikely that the SCA algorithm could be a "good indicator of thermodynamic coupling in proteins"[10] if there is a whole class of thermodynamically linked conserved residues to which it is insensitive. This suggests that McBASC, which does not necessarily give a low score to highly conserved columns, might be a more appropriate algorithm for use in examining the SCA hypothesis, although some modification would be required to take into account perfectly conserved columns. But, even the power of McBASC in outperforming random pairing algorithms is mostly exhausted below the 90th percentile of McBASC scores [Fig. 5(B)]. We argue, therefore, that the SCA hypothesis is unlikely to be generally true, no matter which correlated mutation algorithm is used.

Although searching for energetically linked residues in all but the very highly covarying residue pairs is likely to be fruitless with any of the correlated mutation algorithms, it remains a possibility that these covarying residues will generally display a high degree of energetic coupling. The overall low power of the SCA algorithm, however, appears to make it a particularly poor choice for exploring this sort of SCA-type hypothesis. As we have seen in our artificial alignments in Figure 1(B), the SCA algorithm tends to give high scores to columns that are highly random. Not surprisingly, therefore, the power of the SCA algorithm can be modestly improved by eliminating poorly conserved columns from the alignments (see Methods section). However, even with the poorly conserved columns removed,[22] the SCA algorithm still substantially underperforms OMES and McBASC. The built-in ability of the OMES and McBASC algorithms to filter out highly random columns, together with their higher overall power, suggests that a research program based on either of these algorithms is more likely than SCA to meet with success in exploring the link between the evolutionary record and energetic connectivity.

CONCLUSION

In the view of correlated mutation algorithms presented in this article, these algorithms act as a filter of conservation. A correlated mutation algorithm has a preferred level of background conservation and within that level of conservation chooses the residue pairs that truly covary. Figure 2(A) suggests that this view is accurate for OMES, SCA, and MI, which have very constrained regions of background conservation to which they give high covariance scores. The situation is more complicated for McBASC, which gives high scores to a wider range of conservation than the other algorithms. This is to be expected from Figure 1 where we see that McBASC acts as a "covarying or highly conserved" filter whereas the other algorithms act as "covarying but not highly conserved" filters. Despite the more complex relationship between McBASC and conservation, it appears from Figures 2(B) and 5(C) that the very highest McBASC scores are given to a background conservation level similar to the conservation level seen in the highest OMES scores. This similarity in sensitivity to background conservation between OMES and McBASC for high covariance scores is matched by a similar level of performance in our set of 224 Pfam families [Fig. 3(C)]. This similar level of performance suggests that in Pfam alignments, at least, covariance more often occurs at the background conservation frequency expected by OMES and McBASC than by SCA or MI. It is significant, though, that SCA and MI do have some power [Fig. 3(C)] and in a number of alignments find a statistically significant number of physically close residues, despite favoring columns that are much less conserved than OMES and McBASC [Figs. 2(C) and 5(C)]. This suggests that some real covariance does occur in conservation "frequencies" to which OMES and McBASC are insensitive. Moreover, because the covariance algorithms generally choose distinct residue pairs, it may be possible to combine them in some way to produce an algorithm sensitive to a wide spectrum of background conservation frequencies.

We have only evaluated these algorithms for Pfam alignments. Of course, methods of generating alignments other than Pfam might yield very different results because the average background conservation generated by other alignment methods will inevitably be different than that generated by the Pfam procedure. It may very well be that there are alignment methods that produce a lower overall average conservation, and for these methods SCA or MI might show higher power than they do for the Pfam alignments. Careful consideration of the effects of conser-

vation in relation to a given set of alignments may help investigators elicit the most information out of these notoriously finicky, low powered algorithms.

NOTE ADDED IN PROOF

Supplementary materials are hosted at http:www.afodor.net.

While this article was in press, we published another paper that explicitly considered the use of correlated mutation algorithms to discover evolutionarily conserved energetic pathways. We refer interested readers to Fodor A. and Aldrich R., On Evolutionary Conservation of Thermodynamic Coupling in Proteins, JBC, 2004, in press.

ACKNOWLEDGMENTS

We thank J. Dekker, S. Larson, M. Levitt, M. Maduke, T. Middendorf, S. Pyott, C. Rohl, M. van Tuinen, W. Weis, and G. Yellen for critically reading an earlier version of the manuscript. Rama Ranganathan generously provided the Windows binaries for the SCA package. This work was supported by the Mathers Foundation.

REFERENCES

1. Olmea O, Valencia A. Improving contact predictions by the combination of correlated mutations and other sources of sequence information. Fold Des 1997;2:S25–32.
2. Olmea O, Rost B, Valencia A. Effective use of sequence correlation and conservation in fold recognition. J Mol Biol 1999;293:1221–1239.
3. Gobel U, Sander C, Schneider R, Valencia A. Correlated mutations and residue contacts in proteins. Proteins 1994;18:309–317.
4. Larson SM, Di Nardo AA, Davidson AR. Analysis of covariation in an SH3 domain sequence alignment: applications in tertiary contact prediction and the design of compensating hydrophobic core substitutions. J Mol Biol 2000;303:433–446.
5. Altschuh D, Lesk AM, Bloomer AC, Klug A. Correlation of co-ordinated amino acid substitutions with function in viruses related to tobacco mosaic virus. J Mol Biol 1987;193:693–707.
6. Fariselli P, Olmea O, Valencia A, Casadio R. Progress in predicting inter-residue contacts of proteins with neural networks and correlated mutations. Proteins 2001;45(Suppl 5):157–162.
7. Shindyalov IN, Kolchanov NA, Sander C. Can three dimensional contacts in protein structures be predicted by analysis of correlated mutations? Protein Eng 1994;7:349–358.
8. Thomas DJ, Casari G, Sander C. The prediction of protein contacts from multiple sequence alignments. Protein Eng 1996;9:941–948.
9. Hatley ME, Lockless SW, Gibson SK, Gilman AG, Ranganathan R. Allosteric determinants in guanine nucleotide-binding proteins. Proc Natl Acad Sci USA 2003;100;14445–14450.
10. Lockless SW, Ranganathan R. Evolutionarily conserved pathways of energetic connectivity in protein families. Science 1999;286:295–299.
11. Suel GM, Lockless SW, Wall MA, Ranganathan R. Evolutionarily conserved networks of residues mediate allosteric communication in proteins. Nat Struct Biol 2003;10:59–69.
12. Kass I, Horovitz A. Mapping pathways of allosteric communication in GroEL by analysis of correlated mutations. Proteins 2002;48:611–617.
13. Atchley WR, Wollenberg KR, Fitch WM, Terhalle W, Dress AW. Correlations among amino acid sites in bHLH protein domains: an information theoretic analysis. Mol Biol Evol 2000;17:164–178.
14. Atchley WR, Terhalle W, Dress A. Positional dependence, cliques, and predictive motifs in the bHLH protein domain. J Mol Evol 1999;48:501–516.
15. McLachlan AD. Tests for comparing related amino-acid sequences. Cytochrome *c* and cytochrome *c* 551. J Mol Biol 1971;61: 409–424.
16. Shenkin PS, Erman B, Mastrandrea LD. Information theoretical entropy as a measure of sequence variability. Proteins 1991;11: 297–313.
17. Bateman A, Birney E, Cerruti L, Durbin R, Etwiller L, Eddy SR, Griffiths-Jones S, Howe KL, Marshall M, Sonnhammer EL. The Pfam protein families database. Nucleic Acids Res 2002;30:276–280.
18. Berman HM, Westbrook J, Feng Z, Gilliland G, Bhat TN, Weissig H, Shindyalov IN, Bourne PE. The Protein Data Bank. Nucleic Acids Res 2000;28:235–242.
19. Bickel PJ, Kechris KJ, Spector PC, Wedemayer GJ, Glazer AN. Inaugural article: finding important sites in protein sequences. Proc Natl Acad Sci USA 2002;99:14764–14771.
20. Carter PJ, Winter G, Wilkinson AJ, Fersht AR. The use of double mutants to detect structural changes in the active site of the tyrosyl-tRNA synthetase (*Bacillus stearothermophilus*). Cell 1984; 38:835–840.
21. Jiang Y, Pico A, Cadene M, Chait BT, MacKinnon R. Structure of the RCK domain from the *E. coli* K+ channel and demonstration of its presence in the human BK channel. Neuron 2001;29:593–601.
22. Dekker J, Fodor A, Aldrich R, Yellen G. A perturbation based method for calculating explicit likelihood of evolutionary covariance in multiple sequence alignments. Bioinformatics, In press.
23. Thompson JD, Higgins DG, Gibson TJ. CLUSTAL W: improving the sensitivity of progressive multiple sequence alignment through sequence weighting, position-specific gap penalties and weight matrix choice. Nucleic Acids Res 1994;22:4673–4680.
24. Aloy P, Stark A, Hadley C, Russell RB. Predictions without templates: new folds, secondary structure, and contacts in CASP5. Proteins 2003;53(Suppl 6):436–456.
