# Supplementary material for: Accurate Simulation and Detection of Coevolution Signals in Multiple Sequence Alignments
Source: PLoS One. 2012 Oct 16;7(10):e47108. doi: 10.1371/journal.pone.0047108 (PMC3473043; doi:10.1371/journal.pone.0047108)
Supplement: Toolbox S1 — MSAVOLVE_v1.0a Toolbox for Matlab. (ZIP) [file pone.0047108.s024.zip › MSAVOLVE_v1.0a/COEVOLUTION_METHODS/FUNCTIONS/PSICOV/glasso.pdf]

Package ‘glasso’

February 14, 2012

Title Graphical lasso- estimation of Gaussian graphical models

Version 1.7

Author Jerome Friedman, Trevor Hastie and Rob Tibshirani

Description Graphical lasso

Maintainer Rob Tibshirani <tibs@stat.stanford.edu>

License GPL-2

URL http://www-stat.stanford.edu/~tibs/glasso

Repository CRAN

Date/Publication 2011-09-14 06:21:04

R topics documented:

glasso 1
glassopath 3

Index 6

---

glasso *Graphical lasso*

Description

Estimates a sparse inverse covariance matrix using a lasso (L1) penalty

Usage

```
glasso(s, rho, zero=NULL, thr=1.0e-4, maxit=1e4,  approx=FALSE, penalize.diagonal=TRUE, start=c("cold"
```

Arguments

- `s` — Covariance matrix:p by p matrix (symmetric)
- `rho` — (Non-negative) regularization parameter for lasso. rho=0 means no regularization. Can be a scalar (usual) or a symmetric p by p matrix, or a vector of length p. In the latter case, the penalty matrix has jkth element sqrt(rho[j]*rho[k]).
- `zero` — (Optional) indices of entries of inverse covariance to be constrained to be zero. The input should be a matrix with two columns, each row indicating the indices of elements to be constrained to be zero. The solution must be symmetric, so you need only specify one of (j,k) and (k,j). An entry in the zero matrix overrides any entry in the rho matrix for a given element.
- `thr` — Threshold for convergence. Default value is 1e-4. Iterations stop when average absolute parameter change is less than thr * ave(abs(offdiag(s)))
- `maxit` — Maximum number of iterations of outer loop. Default 10,000
- `approx` — Approximation flag: if true, computes Meinhausen-Buhlmann(2006) approximation
- `penalize.diagonal` — Should diagonal of inverse covariance be penalized? Dafault TRUE.
- `start` — Type of start. Cold start is default. Using Warm start, can provide starting values for w and wi
- `w.init` — Optional starting values for estimated covariance matrix (p by p). Only needed when start="warm" is specified
- `wi.init` — Optional starting values for estimated inverse covariance matrix (p by p) Only needed when start="warm" is specified
- `trace` — Flag for printing out information as iterations proceed. Default FALSE

Details

Estimates a sparse inverse covariance matrix using a lasso (L1) penalty, using the approach of Friedman, Hastie and Tibshirani (2007). The Meinhausen-Buhlmann (2006) approximation is also implemented. The algorithm can also be used to estimate a graph with missing edges, by specifying which edges to omit in the zero argument, and setting rho=0. Or both fixed zeroes for some elements and regularization on the other elements can be specified.

This version 1.7 uses a block diagonal screening rule to speed up computations considerably. Details are given in the paper "New insights and fast computations for the graphical lasso" by Daniela Witten, Jerry Friedman, and Noah Simon, to appear in "Journal of Computational and Graphical Statistics". The idea is as follows: it is possible to quickly check whether the solution to the graphical lasso problem will be block diagonal, for a given value of the tuning parameter. If so, then one can simply apply the graphical lasso algorithm to each block separately, leading to massive speed improvements.

Value

A list with components

- `w` — Estimated covariance matrix

wi	Estimated inverse covariance matrix
loglik	Value of maximized log-likelihodo+penalty
errflag	Memory allocation error flag: 0 means no error; !=0 means memory allocation error - no output returned
approx	Value of input argument approx
del	Change in parameter value at convergence
niter	Number of iterations of outer loop used by algorithm

References

Jerome Friedman, Trevor Hastie and Robert Tibshirani (2007). Sparse inverse covariance estimation with the lasso. Biostatistics 2007. http://www-stat.stanford.edu/~tibs/ftp/graph.pdf

Meinshausen, N. and Buhlmann, P.(2006) High dimensional graphs and variable selection with the lasso. Annals of Statistics,34, p1436-1462.

Daniela Witten, Jerome Friedman, and Noah Simon (2011). New insights and faster computations for the graphical lasso. To appear in Journal of Computational and Graphical Statistics.

Examples

```
set.seed(100)

x<-matrix(rnorm(50*20),ncol=20)
s<- var(x)
a<-glasso(s, rho=.01)
aa<-glasso(s,rho=.02, w.init=a$w, wi.init=a$wi)

# example with structural zeros and no regularization,
# from Whittaker's Graphical models book  page xxx.

s=c(10,1,5,4,10,2,6,10,3,10)
S=matrix(0,nrow=4,ncol=4)
S[row(S)>=col(S)]=s
S=(S+t(S))
diag(S)<-10
zero<-matrix(c(1,3,2,4),ncol=2,byrow=TRUE)
a<-glasso(S,0,zero=zero)
```

---

glassopath *Compute the Graphical lasso along a path*

Description

Estimates a sparse inverse covariance matrix using a lasso (L1) penalty, along a path of values for the regularization parameter

Usage

```
glassopath(s, rholist=NULL, thr=1.0e-4, maxit=1e4,  approx=FALSE, penalize.diagonal=TRUE, w.init=NULL,
```

Arguments

`s`	Covariance matrix:p by p matrix (symmetric)
`rholist`	Vector of non-negative regularization parameters for the lasso. Should be increasing from smallest to largest; actual path is computed from largest to smallest value of rho). If NULL, 10 values in a (hopefully reasonable) range are used. Note that the same parameter rholist[j] is used for all entries of the inverse covariance matrix; different penalties for different entries are not allowed.
`thr`	Threshold for convergence. Default value is 1e-4. Iterations stop when average absolute parameter change is less than thr * ave(abs(offdiag(s)))
`maxit`	Maximum number of iterations of outer loop. Default 10,000
`approx`	Approximation flag: if true, computes Meinhausen-Buhlmann(2006) approximation
`penalize.diagonal`	Should diagonal of inverse covariance be penalized? Dafault TRUE.
`w.init`	Optional starting values for estimated covariance matrix (p by p). Only needed when start="warm" is specified
`wi.init`	Optional starting values for estimated inverse covariance matrix (p by p) Only needed when start="warm" is specified
`trace`	Flag for printing out information as iterations proceed. trace=0 means no printing; trace=1 means outer level printing; trace=2 means full printing Default FALSE

Details

Estimates a sparse inverse covariance matrix using a lasso (L1) penalty, along a path of regularization paramaters, using the approach of Friedman, Hastie and Tibshirani (2007). The Meinhausen-Buhlmann (2006) approximation is also implemented. The algorithm can also be used to estimate a graph with missing edges, by specifying which edges to omit in the zero argument, and setting rho=0. Or both fixed zeroes for some elements and regularization on the other elements can be specified.

This version 1.7 uses a block diagonal screening rule to speed up computations considerably. Details are given in the paper "New insights and fast computations for the graphical lasso" by Daniela Witten, Jerry Friedman, and Noah Simon, to appear in "Journal of Computational and Graphical Statistics". The idea is as follows: it is possible to quickly check whether the solution to the graphical lasso problem will be block diagonal, for a given value of the tuning parameter. If so, then one can simply apply the graphical lasso algorithm to each block separately, leading to massive speed improvements.

Value

A list with components

`w`	Estimated covariance matrices, an array of dimension (nrow(s),ncol(n), length(rholist))

`wi`	Estimated inverse covariance matrix, an array of dimension (nrow(s),ncol(n), length(rholist))
`approx`	Value of input argument approx
`rholist`	Values of regularization parameter used
`errflag`	values of error flag (0 means no memory allocation error)

References

Jerome Friedman, Trevor Hastie and Robert Tibshirani (2007). Sparse inverse covariance estimation with the lasso. Biostatistics 2007. http://www-stat.stanford.edu/~tibs/ftp/graph.pdf

Meinshausen, N. and Buhlmann, P.(2006) High dimensional graphs and variable selection with the lasso. Annals of Statistics,34, p1436-1462.

Daniela Witten, Jerome Friedman, Noah Simon (2011). New insights and fast computation for the graphical lasso. To appear in Journal of Computational and Graphical Statistics.

Examples

```
set.seed(100)

x<-matrix(rnorm(50*20),ncol=20)
s<- var(x)
a<-glassopath(s)
```

Index

∗Topic **graphs**
glasso, 1
glassopath, 3
∗Topic **models**
glasso, 1
glassopath, 3
∗Topic **multivariate**
glasso, 1
glassopath, 3

glasso, 1
glassopath, 3
