# Supplementary material for: Accurate Simulation and Detection of Coevolution Signals in Multiple Sequence Alignments
Source: PLoS One. 2012 Oct 16;7(10):e47108. doi: 10.1371/journal.pone.0047108 (PMC3473043; doi:10.1371/journal.pone.0047108)
Supplement: Toolbox S1 — MSAVOLVE_v1.0a Toolbox for Matlab. (ZIP) [file pone.0047108.s024.zip › MSAVOLVE_v1.0a/COEVOLUTION_METHODS/FUNCTIONS/OMES/covariance1_1.zip!/ReadMe.doc]

# Last updated Sept. 2, 2005

This document provides directions for using the Java code to go along with these two papers:

(1) Fodor A. and Aldrich R., Influence of Conservation on Calculations of Amino Acid Covariance in Multiple Sequence Alignments, Proteins: Structure, Function and Genetics, Proteins. 2004 Aug 1;56(2):211-21.

(2) Dekker J., Fodor A., Aldrich R. and Yellen G. A perturbation-based method for calculating explicit likelihood of evolutionary covariance in multiple sequence alignments. 2004 Jul 10;20(10):1565-72.

**Disclaimer**:

This software is distributed as is. The authors take no responsibility for any use or misuse. We ask that any work benefiting from the use of this package cite one or both references above.

If you have question, concerns, complaints or find bugs you should contact Anthony Fodor. My e-mail is anthony.fodor@gmail.com. New versions of this software (and news as to what I may or may not be up to) may from time to time be released at [http://www.afodor.net](http://www.afodor.net/).

**Using the code to generate scores under McBASC, ELSC, OMES, SCA and MI.**

When you unzip the distribution, make sure you unzip with the option on to preserve directory structure.

You will need:

**1.** An alignment to run the code over. (Alternatively, you can use the one supplied called pnase.txt in the zip file). The alignment should be in the format:

NAME1	VLGGPG.... ESIKKR

NAME2	VLGGPG.... ESIKKR

NAME3	VLGGPG.... ESIKKR

That is, each sequence should in one line. The first word of the line should be the name of the sequence. The second word of the line should be the amino acid sequences all as one word. Gaps can be ‘.’ or ‘-‘ but should not be spaces.

**2.** A java runtime environment ( at least 1.5). If you are not sure what that is, the best thing to do is go to [http://java.sun.com](http://java.sun.com/) and grab a recent copy of the Java 2 Platform, Standard Edition (J2SE).

**3.** There is a file in the distribution called Energetics.properties. This file needs to be in a directory in your classpath and needs to be edited so that it points to the correct place on your hard drive. See the descriptions below for what needs to be set in this file. To use all the code, you need to set these 3 lines to point to right place on your hard drive:

HOME_DIRECTORY=C:\YourDirectoryHere

CLUSTAL_EXECUTABLE=c:\cygwin\clustalw\clustalw.exe

CLUSTAL_DIRECTORY=c:\cygwin\clustalw

Obviously, the CLUSTAL lines need to point to a working installation of CLUSTAL ( you only need to do this if you want to use the algorithms to predict pair distances in a crystal structure). Google can probably lead you to a version of CLUSTAL that will work on your operating system. I found that CLUSTAL for Windows didn’t work under XP, but does work under cygwin.

Note that any of these programs that use CLUSTALW are not remotely thread safe even if called from separate VMs.

In perusing the code, you’ll notice that the different algorithms can handle gaps in the alignments in different ways. As is often the case with filling in missing data, it’s hard to know the best thing to do when faced with gaps in the sequences. For each algorithm, I’ve tried to make the choice that makes that algorithm work the best. But I haven’t studied the effects of these choices in any systematic way. So if your alignments have lots of gaps in them, you might consider making different choices about how to treat gaps to see if you can increase the power of the algorithm you are using.

We’ve included our implementation of the Java SCA algorithm. In our papers, we used a windows binary of the SCA algorithm which was given to us by the Ranganathan lab ( see references in our papers). You’ll have to ask them for a copy of those binaries if you want to use them. We have looked at several alignments and our SCAcode and the binary SCA code give perfectly correlated results ( differing only by a constant), so you’re probably ok just using our Java implementation.

Since this is pure Java it should run anywhere Java can run. For example, to use the SCA algorithm, simply change to the directory where you’ve installed our code and type:

java covariance.algorithms.JavaSCA pnase.txt yourOutFile.txt

Likewise, for OMES, ELSC, MI, and the conservation sum algorithms:

java covariance.algorithms.ELSCCovariance pnase.txt elscOut.txt

java covariance.algorithms.MICovariance pnase.txt miOut.txt

java covariance.algorithms.OmesCovariance pnase.txt OmesOut.txt

java covariance.algorithms.ConservationSum pnase.txt cSum.txt

java covariance.algorithms.RandomScore pnase.txt randomOut.txt

The random pairing algorithm simply assigns a score between 0 and 1 for each pair of columns. Note that the random pairing code doesn’t keep track of the old scores it’s assigned, so calling getScore( i, j ) twice will not result in the same score returned.

These commands produce a text file with three columns of output which are the alignment i, the alignment j and the covariance score.

**Important:** The McBasc algorithm has a requirement for the file Maxhom_McLachlan.metric. This file should be in the subdirectory “data” where you unzipped the distribution zip file. You’ll need to edit the Energetics.properties file so that the line

HOME_DIRECTORY=C:\MyCovarianceInstall

Points to the directory where you unzipped the zip file.

Once that line has been property set in the Energetics.properties file you can run:

java covariance.algorithms.McBASCCovariance pnase.txt mcBASCOut.txt

For all of these algorithms, if you have a large alignment, you might need to allocate more than the default amount of memory to Java. For example, to allocate 256 Megs, you can invoke Java as follows:

java –mx256m covariance.algorithms.ELSCCovariance pnase.txt elscOut.txt

Note that the main methods in these algorithms will skip over any column that has no valid upper-case residues

**A test suite.**

This test suite tests some of the basic functionality of the code. To run the tests you will need a copy of junit, which is available at <http://www.junit.org/index.htm>. If you’ve followed the directions up to this point, you should be able to run all of the tests that are in TestSuite1. See the Junit documentation for information on how to run the test.

There are additional tests ( which require a good deal more set up ) for other parts of the application. If you are interested in this test code, please contact me.

**Predicting Cb-Cb distance**

The main() method in the class covariance.utils.RunOne runs all of the algorithms and shows predictions of Cb-Cb distance for a corresponding crystal structure. If you’ve set these three lines in the Energetics.properties file, you should be able to run this method from your HOME_DIRECTORY with this command line:

java covariance.utils.RunOne

You can view the output in the file called PNASE_results.txt

By modifying the first few lines in this method, you should be able to drop your own structures and alignments in.

Note that RunOne makes some choices about what it does to the alignment. For example, it removes any columns in the alignment that have >50% gaps and it removes any sequences in the alignment that are >90% redundant to another sequence. Also, Cb-Cb predictions are only made if all the chains in the crystal structure have the same residue as the alignment line that Clustal has aligned to the PDB sequence. By putting your own values in the appropriate places, you can change these choices. However, new values may cause the program to throw exceptions. For example, the SCA algorithm will complain if you try and run it on a column in which there are no valid residues.

# Changes between 1.0 and 0.91

Changes only to this document and to some of the comments in the code. No functional changes to the code.

**Changes between 0.9 and 0.91**

A bug in the ELSC implementation was quashed. This bug affected the way that m, the count of residues in the idealized subset, was rounded so that the sum of m across all 20 residues equals the number of sequences in the sub-alignment. The bug caused this rounding to occur in an incorrect way. The bug occasionally caused ELSC scores to be negative. (We thank Masha Niv for noticing this). The following graph shows the relationship between the published ELSC implementation and the corrected implementation for the pnase.txt alignment included in the code distribution:

As you can see, the bug has very small effects. Note that even with the corrected code, the ELSC value is occasionally negative. This is due to rounding errors due to the fact that we must round m to an integer to calculate the "N choose m" values. You can see in the graph, however, that in the corrected rounding code the negative values are much closer to zero. The new file in version 0.91 is

covariance.algorithms.ELSCCovarianceSubAlignment.java.

The published version is in the code distribution as

covariance.algorithms.ELSCCovarianceSubAlignment.java.published.

# Documentation for version 1.1. Added Sept. 2, 2005

This release of the code allows for automatic predictions of Cb-Cb distance to be generated from the covariance algorithms for the entire PFAM database. **This code is extremely slow.** It’s not an exaggeration to say that with a little work it could be made to run 1,000 times faster. If anyone is interested in working on addressing the performance (and other) problems with this code as an open-source project, let me know (anthony.fodor@gmail.com) and I will get all this stuff checked into SourceForge.

In order to generate predictions against multiple PFAM families you will need all of the requirements listed above plus:

(1) A copy of the PFAM alignments. This can be downloaded from the PFAM FTP site <ftp://ftp.sanger.ac.uk/pub/databases/Pfam/current_release/> The file

Pfam-A.full.gz

contains the alignments. You will need to uncompress this file with:

(2) A working copy of gzip. If you are using any UNIX platform (including OS X), you already have this. If you are using Windows, you can get this as part of the cygwin distribution (<http://www.cygwin.com/>).
(3) a working copy of CLUSTALW
(4) PDB files. You can either download them all in one lump sum from the PDB web site, or the program will download them as it needs them if it is connected to the internet (this is slow though) or some combination of the two.
(5) A fair amount of disk space (on the order of a few gigs ) and at least half a gig or RAM.

**Finding the best match between PDB structures and PFAM alignments.**

The program scripts.ScanForBestAlignment will examine each PFAM alignment and download the PDB structures that the comments of the PFAM alignment indicate are matched to the alignment. It will then perform an incredibly slow and somewhat pointless CLUSTAL alignment between each sequence in the alignment and each PDB file. Again, if anyone wants to work on the code to make it faster, let me know. The program will record the longest match between the PDB sequence and the line in the PFAM alignment in a file called “AlignmentScanResults.txt” in your HOME_DIRECTORY.

This program can be invoked from the command line in your HOME_DIRECTORY with:

java -mx512m scripts.ScanForBestAlignment

You can restrict the program to only perform the match for a set of PFAM families by creating a file called “includedFamilies.txt” in your HOME_DIRECTORY. The name of the PFAM families you want included should be the first word of each line in the file. There is a sample “includedFamilies.txt” file in your HOME_DIRECTORY as part of this distribution. Its contents, covering only two PFAM families are:

14-3-3

ALAD

You can obviously change the contents of this file to include the PFAM families that you are interested in. **If you want to run the ScanForBestAlignment program over all of PFAM you must delete or rename the includedFamilies.txt program** (or change the Java code in scripts.ScanForBestAlignment so that it ignores the file). If you don’t delete or modify includedFamilies.txt the only PFAM families that will get analyzed are the two listed above.

Before running the program, make sure to have the following in place in the Energetics.properties file in your HOME_DIRECTORY:

```
# the directory where you unzipped the files in the covariance package
HOME_DIRECTORY=C:\CovariancePackage

# these should point to a working version of CLUSTALW
CLUSTAL_EXECUTABLE=c:\cygwin\clustalw\clustalw.exe
CLUSTAL_DIRECTORY=c:\cygwin\clustalw

# Get this file from the PFAM database
FULL_PFAM_PATH=C:\pfam\Pfam-A_ver17.full

# A working copy of GZIP.  Will be included in any UNIX distribution
# if you are working in Windows, download cygwin
GZIP_FULL_PATH=C:\cygwin\bin\gzip

# where to put the output data.  This directory must exist or the Java
# program will throw
OUT_DATA_DIR=C:\MyData

# this directory must exist.  If the program is looking for a PDB and can't
# find it in this directory, it will connect to the internet and download it
# (using the GZIP program to uncompress it)
LOCAL_PDB_DIRECTORY=e:\pdbs
```

**Running covariance algorithms to make predictions of PDB structures from PFAM alignments.**

Before performing this step, you must perform the previous step to generate a AlignmentScanResults.txt file in your HOME_DIRECTORY. (I’ve include a sample AlignmentScanResults.txt file in this distribution). The program covariance.utils.RunMany will run all of the covariance programs over each PFAM family listed in AlignmentScanResults.txt to generate predictions for the associated PDB file listed in AlignmentScanResults.txt. You will probably want to modify the RunMany program to exclude certain PFAM families (such as those with > 5000 sequences) and certain columns (such as those that have more than 50% gaps). You can examine the comments and code in the scripts.RunOne program (and the documentation above) for more information on how to do this. The Alignment objects that the PfamParser generated will have many useful methods. For example, you can call

a.getNumSequencesInAlignment()

a.getNumColumnsInAlignment()

to get some basic statistics about the alignment.

With the Energetics.properties set up as above and the PFAM families that you are interested in represented in the AlignmentScanResults.txt file in your HOME_DIRECTORY, invoke from the command line in your HOME_DIRECTORY:

java -mx512m covariance.utils.RunMany

The predictions of the covariance algorithms will be in your OUT_DATA_DIR with the predictions for each PFAM family in a separate file. This make take a long time depending on how many PFAM families you are examining. The McBASC algorithm, as implemented, is particularly slow. Again, it could be made to run a good deal faster with a little work.
